# Gender-specific pathway differences in the human serum metabolome

**DOI:** 10.1007/s11306-015-0829-0

**Published:** 2015-08-04

**Authors:** Jan Krumsiek, Kirstin Mittelstrass, Kieu Trinh Do, Ferdinand Stückler, Janina Ried, Jerzy Adamski, Annette Peters, Thomas Illig, Florian Kronenberg, Nele Friedrich, Matthias Nauck, Maik Pietzner, Dennis O. Mook-Kanamori, Karsten Suhre, Christian Gieger, Harald Grallert, Fabian J. Theis, Gabi Kastenmüller

**Affiliations:** 1 Institute of Computational Biology, Helmholtz Zentrum München, Neuherberg, Germany; 2 German Center for Diabetes Research (DZD e.V.), Neuherberg, Germany; 3 Research Unit of Molecular Epidemiology, Helmholtz Zentrum München, Neuherberg, Germany; 4 Institute of Epidemiology II, Helmholtz Zentrum München, Neuherberg, Germany; 5 Institute of Genetic Epidemiology, Helmholtz Zentrum München, Neuherberg, Germany; 6 Institute of Experimental Genetics, Genome Analysis Center, Helmholtz Zentrum München, Neuherberg, Germany; 7 Lehrstuhl für Experimentelle Genetik, Technische Universität München, Freising-Weihenstephan, Germany; 8 German Center for Cardiovascular Disease Research (DZHK e.V.), Munich, Germany; 9 Hannover Unified Biobank, Hannover Medical School, Hannover, Germany; 10 Division of Genetic Epidemiology, Department of Medical Genetics, Molecular and Clinical Pharmacology, Innsbruck Medical University, Innsbruck, Austria; 11 Institute of Clinical Chemistry and Laboratory Medicine, University Medicine Greifswald, Greifswald, Germany; 12 DZHK (German Center for Cardiovascular Research), Greifswald, Greifswald, Germany; 13 Department of Clinical Epidemiology, Leiden University Medical Center, Leiden, Netherlands; 14 Department of Endocrinology, Leiden University Medical Center, Leiden, Netherlands; 15 Department of Physiology and Biophysics, Weill Cornell Medical College in Qatar, Qatar Foundation, Doha, Qatar; 16 Institute of Bioinformatics and Systems Biology, Helmholtz Zentrum München, Neuherberg, Germany; 17 Department of Mathematics, Technische Universität München, Garching, Germany

**Keywords:** Epidemiology, Metabolic networks, Metabolomics, Gender differences, Systems biology

## Abstract

The susceptibility for various diseases as well as the response to treatments differ considerably between men and women. As a basis for a gender-specific personalized healthcare, an extensive characterization of the molecular differences between the two genders is required. In the present study, we conducted a large-scale metabolomics analysis of 507 metabolic markers measured in serum of 1756 participants from the German KORA F4 study (903 females and 853 males). One-third of the metabolites show significant differences between males and females. A pathway analysis revealed strong differences in steroid metabolism, fatty acids and further lipids, a large fraction of amino acids, oxidative phosphorylation, purine metabolism and gamma-glutamyl dipeptides. We then extended this analysis by a network-based clustering approach. Metabolite interactions were estimated using Gaussian graphical models to get an unbiased, fully data-driven metabolic network representation. This approach is not limited to possibly arbitrary pathway boundaries and can even include poorly or uncharacterized metabolites. The network analysis revealed several strongly gender-regulated submodules across different pathways. Finally, a gender-stratified genome-wide association study was performed to determine whether the observed gender differences are caused by dimorphisms in the effects of genetic polymorphisms on the metabolome. With only a single genome-wide significant hit, our results suggest that this scenario is not the case. In summary, we report an extensive characterization and interpretation of gender-specific differences of the human serum metabolome, providing a broad basis for future analyses.

**Electronic supplementary material:**

The online version of this article (doi:10.1007/s11306-015-0829-0) contains supplementary material, which is available to authorized users.

## Introduction

As pointed out in recent studies, gender bias represents a considerable issue in biomedical research (Kim et al. 2010; Liu et al. 2012; Regitz-Zagrosek 2012). For example, besides gender-specific disease susceptibility, sexual dimorphisms play a substantial role for pharmacokinetics and—dynamics (Gandhi et al. 2004). Thus, differential treatment of females and males would constitute one of the simplest forms of personalized medicine (Redekop and Mladsi 2013). However, this requires an extensive understanding of the intrinsic molecular differences between the two gender.

Several studies investigated molecular differences between the two genders in blood or urine on small sets of healthy individuals (Kochhar et al. 2006; Slupsky et al. 2007). Only few high-powered studies reported gender-differentiated results. For example, Dunn et al. (2014) investigated gender-specific changes of the metabolome (n = 1200), and Jansen et al. (2014) found gender-biased gene expression profiles in a Caucasian population (n = 5241). In (Mittelstrass et al. 2011) we showed significant gender-specific differences in amino acid, lipid and sugar serum concentrations in around 3000 subjects. Furthermore, there are several reports of sexual dimorphisms in the genetic effects of common polymorphisms on metabolism (Kolz et al. 2009; Mittelstrass et al. 2011).

**Fig. 1:**
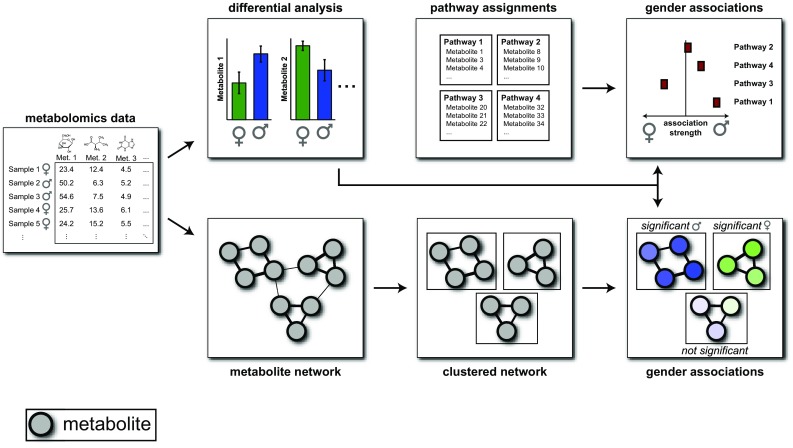
Study approach. Gender differences for each measured metabolite are calculated and evaluated at a pathway level. In parallel, a data-driven, unbiased metabolite interaction network is computed based on Gaussian graphical models. Differential changes are then mapped to this network and analyzed as clusters

Aiming at a more comprehensive picture of metabolic differences between the two genders on a broad range of different metabolic pathways, we extended our previous study by applying a non-targeted metabolomics approach covering metabolites from all major parts of human metabolism. To identify systematic gender differences at the pathway level, we pursued two routes of bioinformatics analyses. First, we performed a classical pathway analysis (Fig. 1, top) using both a coarse level of metabolic pathway assignment (e.g. lipid, amino acid) and a more fine-grained level (e.g. branched-chain amino acids (BCAAs), short-chain fatty acids).

Second, we introduce a network-based clustering algorithm to identify affected pathway regions in a fully data-driven fashion (Fig. 1, bottom). Predefined metabolic pathway annotations are usually biased and rather strictly defined (Gagneur et al. 2003). For example, the often-used pathway classification into “glycolysis” and “citric acid cycle” is only reasonable to a certain extent, since obviously those two processes are strongly intertwined. Moreover, untargeted metabolomics measurements contain a substantial fraction of poorly characterized metabolites with little or no functional annotations. Such metabolites necessarily have to be ignored by a classical pathway analysis, neglecting a large portion of the measured data. As an alternative, we generated a data-based metabolic network using Gaussian graphical models (Krumsiek et al. 2011, 2012; Shin et al. 2014). Those networks represent an intrinsic footprint of metabolic associations between metabolites, without arbitrary borders, and are able to include even uncharacterized metabolites.

Metabolic changes between genders are then mapped onto this metabolic network. This approach has previously been used in a series of papers with different phenotypes, for example with asthma (Ried et al. 2013), dietary and physical parameters (Floegel et al. 2014), and our previous gender study (Mittelstrass et al. 2011). While in those studies the network was merely used for visualization purposes, we extend the approach here by adding an additional layer of clustering and cluster enrichment. The goal is to computationally identify network regions affected by gender. Intuitively, we first identify strongly connected regions in the network without taking into account gender effects, in order to get intrinsic, data-defined metabolic modules. For each cluster, we then evaluate whether it carries a statistically significant gender effect, i.e. coordinated up- or down-regulation of all metabolites in the cluster. The general approach of evaluating phenotypic effects on interaction networks has recently been described as the new field of “differential network biology” (Ideker and Krogan 2012).

To test whether the observed gender differences in metabolite levels are caused by dimorphisms in genotype–metabolite interactions, we performed a stratified genome-wide association study (GWAS) with metabolites as phenotypic traits. That is, we searched for cases where the effect of a SNP on serum concentrations of a metabolite is different between males and females. In our previous study (Mittelstrass et al. 2011), only a single metabolite-gene association (glycine to CPS1) reached genome-wide significance. With the larger and broader set of metabolites and the extended SNP panel, this analysis can be revised to identify possible further dimorphisms in the genetic architecture of the metabolome.

## Results

### Gender-specific metabolite differences

We investigated 1756 fasting serum samples of the KORA F4 discovery cohort, subdivided into 903 females and 853 males. The dataset contains untargeted measurements of 507 metabolites, thereof 318 with known identity and 189 unidentified metabolites (*unknowns*). Replication samples originate from the German SHIP population cohort measured on the same metabolomics platform, containing 1000 fasting plasma samples, thereof 561 females and 439 males (see Sect. 4). The set of measured metabolites was similar, but not identical. Metabolite concentrations were logarithmized, since a test of normality showed that log-transformed concentrations were substantially closer to a normal distribution than untransformed values (see Sect. 4).Gender differences were assessed using standard linear regression, corresponding to a pairwise *t* test due to the dichotomic outcome variable, corrected against age and BMI as cofactors. An overview of the metabolite panels, the replication cohort and overall statistics can be found in Supplementary material 1. Full association result tables including replication data are available in Supplementary material 2.

**Fig. 2:**
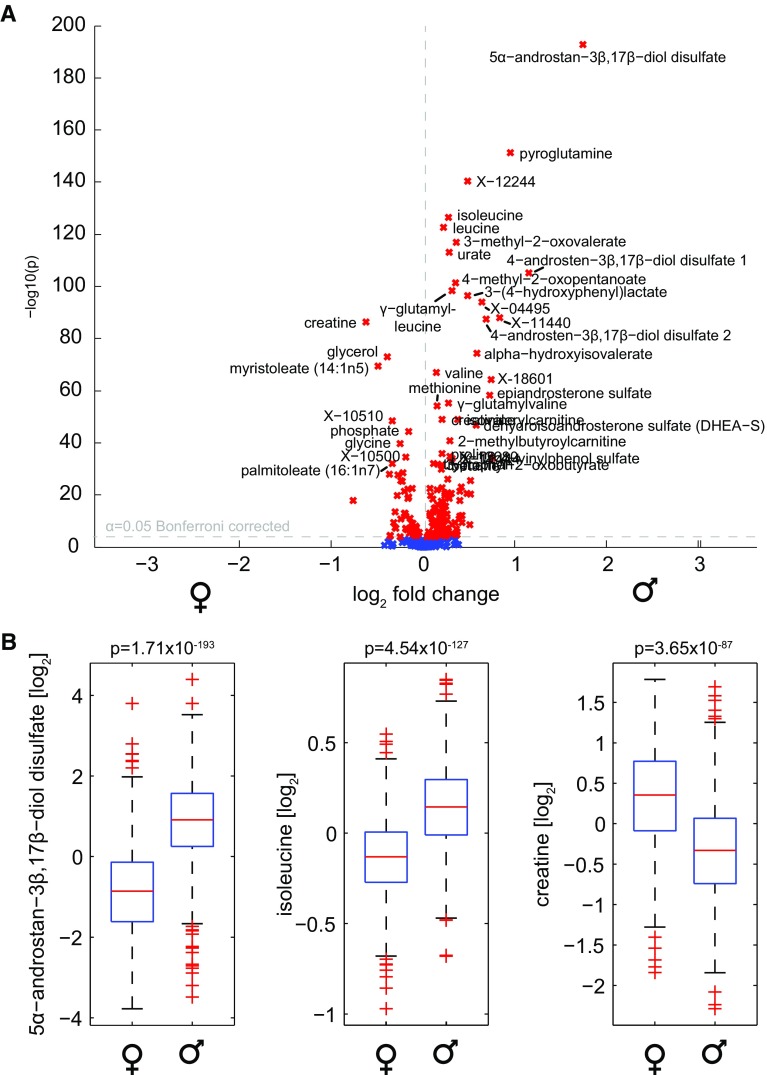
Overview of gender differences at a single metabolite level. **a** Volcano plot visualizing p values of pairwise *t* tests and the log_2_ fold changes. We observe remarkably low p values down to 10^−190^. 180 of 507 metabolites in the discovery cohort (35.5 %) were significantly different after Bonferroni correction, with 50 metabolites showing higher concentrations in females and 130 metabolites with higher concentrations in males. **b** Three exemplary boxplots of strongly differential metabolites. The steroid derivative 5α-androstan-3β,17β-diol disulfate and the BCAA isoleucine showed elevated levels in males, whereas creatine was higher in females

The results of the primary analysis sorted by general pathways are displayed in Table 1 and Fig. 2. Out of the 507 measured metabolites, 180 (35.0 %) showed significant gender differences after Bonferroni correction. Notably, we find extremely low p values in our analysis, down to values around 10^−193^. These values should not be interpreted in its original meaning, i.e. the probability of the effect occurring by chance, but rather as a variance-normalized measure of the effect size. A total of 114 of these 180 were also measured in the validation cohort, where 88 out of these 114 metabolite differences (77.2 %) could be replicated.

**Table 1:** Gender-specific concentration differences on single-metabolite level

Metabolite	β	r^2^	N	p value	Repl. p value	Repl. direction	Replicated?
Amino acids (72 total, 42 significant)							
Pyroglutamine	−1.127 ± 0.039	0.349	1746	6.21E−152	1.92E−95	Same	Yes
Isoleucine	−1.005 ± 0.038	0.358	1746	4.54E−127	2.40E−61	Same	Yes
Leucine	−1.004 ± 0.039	0.337	1744	2.79E−123	1.90E−60	Same	Yes
3-Methyl-2-oxovalerate	−1.000 ± 0.040	0.303	1747	1.04E−117	2.94E−62	Same	Yes
4-Methyl-2-oxopentanoate	−0.946 ± 0.041	0.264	1748	3.21E−102	8.96E−58	Same	Yes
Carbohydrates (14 total, 6 significant)							
1,5-Anhydroglucitol	−0.397 ± 0.047	0.049	1731	6.72E−17	1.87E−20	Same	Yes
Mannose	−0.352 ± 0.043	0.190	1737	8.57E−16	–	–	–
Glucose	−0.319 ± 0.045	0.143	1737	1.25E−12	–	–	–
Arabitol	−0.308 ± 0.047	0.047	1727	8.33E−11	–	–	–
Lactate	−0.287 ± 0.046	0.097	1743	4.03E−10	–	–	–
Cofactors and vitamins (15 total, 7 significant)							
Threonate	0.440 ± 0.047	0.057	1741	1.52E−20	–	–	–
Pyridoxate	−0.341 ± 0.047	0.065	1697	7.09E−13	4.57E−01	Same	No
Biliverdin	−0.337 ± 0.059	0.032	1162	1.08E-08	8.38E−17	Same	Yes
Ascorbate	0.280 ± 0.050	0.021	1550	3.39E−08	–	–	–
Bilirubin (Z; Z)	−0.249 ± 0.048	0.024	1702	2.61E−07	6.40E−01	Same	No
Bilirubin (E; E)	−0.230 ± 0.047	0.023	1750	1.31E−06	3.04E−02	Same	No
Energy (6 total, 4 significant)							
Phosphate	0.654 ± 0.045	0.119	1746	4.46E−45	1.83E−19	Same	Yes
Acetylphosphate	0.470 ± 0.047	0.063	1744	2.38E−23	–	–	–
Succinylcarnitine	−0.231 ± 0.048	0.120	1518	1.92E−06	9.23E−05	Same	Yes
Malate	−0.207 ± 0.050	0.048	1561	3.29E−05	5.72E−05	Different	No
Lipids (128 total, 49 significant)							
5α-Androstan-3β, 17β-diol disulfate	−1.257 ± 0.037	0.411	1727	1.71E−193	8.54E−72	Same	Yes
4-Androsten-3β, 17β-diol disulfate 1	−0.955 ± 0.041	0.281	1745	8.38E−106	6.45E−43	Same	Yes
4-Androsten-3β, 17β-diol disulfate 2	−0.883 ± 0.042	0.244	1741	2.92E−88	6.99E−48	Same	Yes
Glycerol	0.798 ± 0.042	0.244	1739	1.01E−73	–	–	–
Myristoleate	0.811 ± 0.044	0.168	1750	3.64E−70	7.30E−46	Same	Yes
Nucleotides (13 total, 5 significant)							
Urate	−0.954 ± 0.039	0.340	1749	7.04E−114	1.08E−52	Same	Yes
Allantoin	−0.395 ± 0.072	0.068	728	6.84E−08	–	–	–
Inosine	0.257 ± 0.048	0.036	1715	7.47E−08	–	–	–
Hypoxanthine	0.198 ± 0.048	0.018	1727	3.54E−05	9.98E−01	Same	No
Pseudouridine	−0.175 ± 0.044	0.150	1741	8.47E−05	2.61E−01	Same	No
Peptides (25 total, 10 significant)							
γ-glutamylleucine	−0.925 ± 0.041	0.273	1745	3.92E−99	5.78E−86	Same	Yes
γ-glutamylvaline	−0.681 ± 0.042	0.253	1728	7.56E−56	3.22E−50	Same	Yes
γ-glutamylphenylalanine	−0.449 ± 0.045	0.123	1731	1.20E−22	8.00E−28	Same	Yes
γ-glutamylisoleucine	−0.553 ± 0.060	0.106	1008	2.44E−19	1.60E−48	Same	Yes
γ-glutamyltyrosine	−0.353 ± 0.046	0.131	1650	2.93E−14	3.97E−26	Same	Yes
Xenobiotics (39 total, 3 significant)							
4-Vinylphenol sulfate	−0.584 ± 0.046	0.091	1708	6.43E−35	1.00E−04	Same	Yes
Piperine	−0.319 ± 0.047	0.062	1720	1.46E−11	2.14E−02	Same	No
2-Hydroxyisobutyrate	−0.204 ± 0.048	0.068	1604	2.56E−05	–	–	–
Unknowns (189 total, 54 significant)							
X-12244	−1.096 ± 0.039	0.327	1747	3.44E−141	–	–	–
X-04495	−0.954 ± 0.043	0.231	1653	1.15E−94	–	–	–
X-11440	−0.900 ± 0.042	0.220	1743	7.46E−89	1.21E−44	Same	Yes
X-10510	0.677 ± 0.044	0.146	1742	3.29E−49	–	–	–
X-12680	−0.657 ± 0.051	0.112	1365	1.68E−35	–	–	–

Top metabolite associations and replication information for each metabolic class. Negative β values indicate higher concentrations in males, positive values represent higher concentrations in females. r^2^ = explained variance, N = number of valid measurements of analysis. p values originate from a linear regression model further corrected for BMI and age

We performed a comparison of the overlapping metabolites between this study and our previous study (Mittelstrass et al. 2011), which was conducted on a larger subset of the cohort but with a targeted lipid-focused metabolomics panel of only 131 metabolites. Out of the 41 metabolites which overlap between the two metabolomics platforms, 22 metabolites showed significant gender differences in the same direction in both studies and 5 metabolites were consistently not significant in both analyses. 14 significantly different metabolites from the previous study could not be replicated in the present study. We assume that this effect is due to the more accurate measurements obtained from a targeted metabolomics platform in contrast to the possibly higher experimental variations for some metabolites on an untargeted platform. This is corroborated by the fact that the non-replicating lipids have substantially higher measurement coefficients of variation (CV) than the replicating ones (Supplementary material 2).

Moreover, we compared our findings to a metabolic profiling study in a UK population recently published in Metabolomics by (Dunn et al. 2014). Out of the metabolites reported to be significant in that study, 34 were also detectable with our platform. In general, we observe a substantial agreement between the two cohorts. For 21 metabolites, we find significant effects in the same direction, for 10 metabolites we could not observe a significant signal in our data. Since the power was slightly higher in the present study (1756 vs. 1200 individuals), we believe the non-significant findings either to be due to measurement platform differences, or true differences between the two cohorts. Three metabolites showed opposite directions between the two studies. Dunn et al. reported higher concentrations in females for tyrosine and creatinine, whereas previous studies (Kochhar et al. 2006; Marescau et al. 1997) and our analysis found higher concentrations in males for both metabolites. Moreover, there is a disagreement for myo-inositol, which we attribute to the fact that their study measured a mixture of myo-inositol and scyllo-inositol. In our data, we detect significant differences in opposite directions for those two metabolites. Detailed replication and comparison results can be found in Supplementary material 2.

**Fig. 3:**
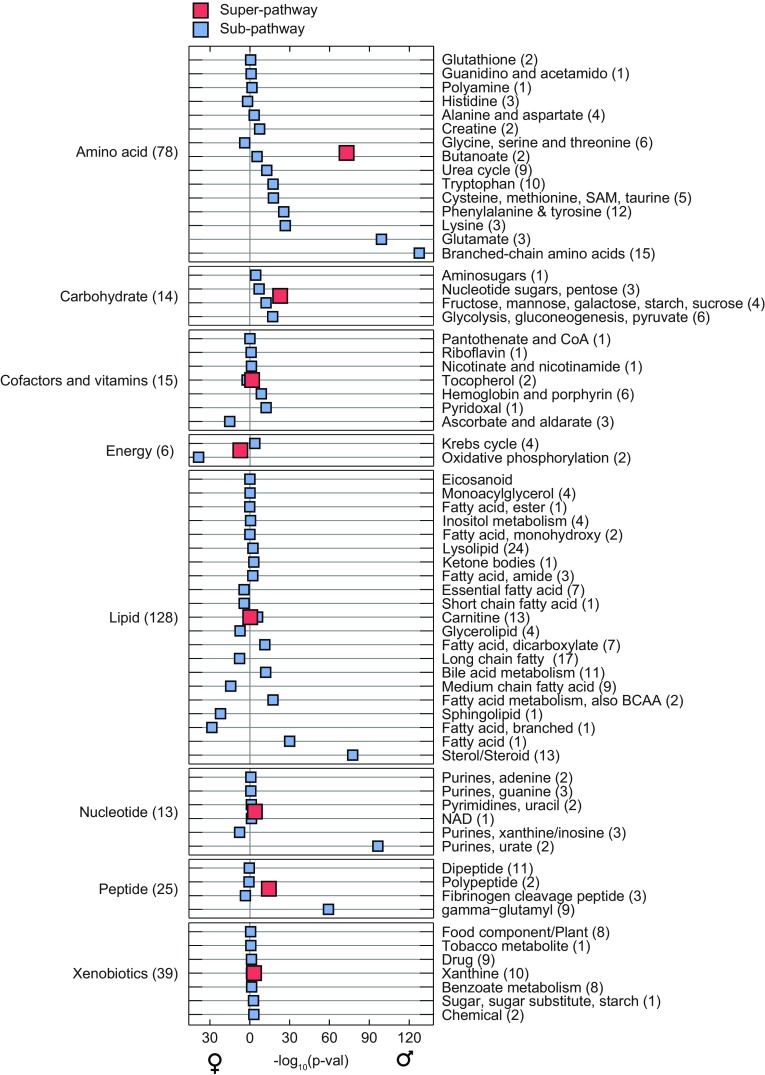
Pathway enrichment. The *left-hand panel* shows association −log_10_ p values for eight super-pathways, *the right-hand panel* contains results for 66 sub-pathways. p values are plotted directionally, i.e. pathways which are higher in females are *left of the zero line*, and pathways up-regulated in males point to the right. The log_10_ p values can be interpreted as a variance-normalized measure of effect size. A detailed discussion of the enrichment results can be found in the main text

In addition to the analysis of concentration differences of single metabolites, we performed a pathway-based statistical analysis in order to provide more interpretable, systematic insights. We decided to follow a simple statistical approach based on mean pathway activity. Specifically, for each group of metabolites (i.e. a “pathway”) we compute the mean z-score over all samples as a measure of average expression (see Sect. 4). The averaged z-scores are then subjected to the same linear regression analysis used for single metabolites. A similar approach has been used in a study by Lee et al. (2008), but in a permutation-based framework. In contrast to classical set enrichment methods (like e.g. GSEA (Subramanian et al. 2005) and MSEA (Persicke et al. 2011), the approach allows to specifically detect up- or down-regulation of concentrations with respect to the given grouping. We performed analyses for two layers of pathway annotations. First, we have “super-pathway” annotations including ‘Lipid’, ‘Carbohydrate’, ‘Amino acid’, ‘Xenobiotics’, ‘Nucleotide’, ‘Energy’, ‘Peptide’, ‘Cofactors and vitamins’. Each super-pathway is further subdivided into two or more “sub-pathways” like ‘Oxidative phosphorylation’, ‘Carnitine metabolism’ or ‘Valine, leucine and isoleucine metabolism’. All pathway annotations are listed in Supplementary material 2. The overall gender effects for both super- and sub-pathways are visualized in Fig. 3. Detailed boxplots of metabolite differences and pathway associations can be found in Supplementary material 3.

Interestingly, we observed substantially more metabolites being higher in males than in females (130 significantly higher in males compared to 50 metabolites higher in females). At this point, we believe that this is not necessarily a general feature of the human metabolome, but rather a bias towards classes of measured metabolites that tend to be higher in males, such as amino acids. A discussion of single metabolite and pathway associations ordered by the eight super-pathways is given in the following. Note that also a plethora of unknown metabolites (names starting with “X−“) showed high concentration differences between males and females. Due to the obvious lack of functional annotations, however, we initially conducted an analysis of known metabolites. Unknowns were then added to the network analysis and are described later in the manuscript.

#### Amino acids

In the amino acid class, the strongest differences were observed for pyroglutamine (p = 6.21 × 10^−152^) and several BCAA metabolites including isoleucine (p = 4.54^−127^), leucine (p = 2.79 × 10^−123^), 3-methyl-2-oxovalerate (p = 1.04 × 10^−117^) and 4-methyl-2-oxopentanoate (p = 3.21 × 10^−102^). All metabolites show higher concentrations in males and could be replicated in the validation cohort. Note that valine (p = 7.28 × 10^−65^) and 3-methyl-2-oxobutyrate (p = 1.41 × 10^−32^) are significantly higher in males as well, completing the list of BCAAs and their first-step degradation products. Previous studies have already described differences in BCCA catabolism of rats (Kobayashi et al. 1997) and humans during exercise (Lamont et al. 2001). To the best of our knowledge, gender differences of pyroglutamine, a cyclic derivative of glutamine, have not been described in literature before. We also confirmed the well-known (Perrone et al. 1992) dimorphisms for creatine (p = 3.65 × 10^−87^, higher in females) and creatinine (p = 4.97 × 10^−47^, higher in males).

At the pathway level, we detect strong differences for the entire class of amino acids (p = 3.26 × 10^−73^), with higher concentrations in males. This is an extension of the findings from our previous study (Mittelstrass et al. 2011). At the more fine-grained sub-pathway level, the strongest differences are constituted by BCAAs (Valine, leucine and isoleucine metabolism, p = 3.16 × 10^−128^), glutamate metabolism (1.02 × 10^−99^) and lysine metabolism (p = 2.77 × 10^−27^). All pathway results replicated in the validation cohort. Enrichment of the BCAA class was obviously expected due to the strong associations of single metabolites. Further amino acid pathways occur among the remaining top hits at the sub-pathway level (e.g. the lysine, phenylalanine, cysteine and tryptophan pathways, all higher in males).

#### Carbohydrates

Several carbohydrates showed differences between males and females, albeit not as profound as for the amino acids and lipids, for example. The strongest difference was detected for 1,5-anhydroglucitol (p = 6.72 × 10^−17^), a well-known marker for short-term glycemic control (Dungan 2008). We detect higher levels in males, which is in accordance with a previous report of non-diabetic individuals (Li et al. 2008). Moreover, the two hexoses mannose (p = 8.57 × 10^−16^) and glucose (p = 1.25 × 10^−12^) showed higher levels in males. While higher glucose (=blood sugar) concentrations in males are well described and have been attributed to physiological differences (Faerch et al. 2010), the actual source of blood mannose is yet to be fully clarified (Sharma and Freeze 2011). Two further affected carbohydrates are arabitol (p = 8.33 × 10^−11^, higher in males) for which gender differences have not been described yet, and lactate (p = 4.03 × 10^−10^, higher in males), which has been reported to show a gender effect in Chinese type 2 diabetes patients (Shen et al. 2012). The 1,5-anhydroglucitol hit replicated, whereas the other four sugars were not measured in the replication cohort (which can be attributed to the missing GC–MS run, see Sect. 4).

The entire super-pathway of carbohydrates was significantly higher in males (p = 1.43 × 10^−23^). At the sub-pathway level, ‘Glycolysis, gluconeogenesis, pyruvate metabolism’ and ‘Fructose, mannose, galactose, starch, and sucrose metabolism’ showed the strongest differences (p = 5.96 × 10^−18^ and p = 6.31 × 10^−13^, respectively), both with higher activity in males. All pathway results replicate, however only with a modest p value for the fructose pathway (p = 0.002).

#### Cofactors and vitamins

Among the top hits in the class of cofactors and vitamins, we observed several substances related to vitamin C, vitamin B6 and heme metabolism. First, we detected higher levels of vitamin C (p = 3.38 × 10^−8^) and, showing an even stronger difference, its degradation product threonate (p = 1.52 × 10^−20^) in females. This sexual dimorphism has already been described three decades ago (Garry et al. 1982). Both metabolites were not measured in the replication cohort. Vitamin B6 (pyridoxine), in contrast, is known to be higher in males (Driskell et al. 2000). While pyridoxine is not contained in our panel of measured metabolites, we could detect elevated levels of its degradation product pyridoxate in males in the discovery cohort (p = 3.67 × 10^−13^), but not in the replication cohort. In addition to the vitamins, we detected higher concentrations of heme (p = 3.32 × 10^−05^) and its degradation products biliverdin (p = 1.08 × 10^−08^) and bilirubin (p = 2.61 × 10^−07^ and p = 1.31 × 10^−06^ for two different isoforms) in males. The bilirubin and biliverdin hits did not replicate, but the heme association was considerably stronger in the replication cohort despite the smaller sample size (p = 4.60 × 10^−22^). Gender-specific concentration differences in the heme degradation substance bilirubin have already been described in previous studies (Rosenthal et al. 1984).

The entire super-pathway of cofactors and vitamins was not significantly different between genders (p = 2.41 × 10^−2^). The three sub-pathways ‘Ascorbate and aldarate metabolism’ (p = 6.02 × 10^−16^, higher in females), ‘Pyridoxal metabolism’ (p = 7.09 × 10^−13^, higher in males) and ‘Hemoglobin and porphyrin metabolism’ (p = 2.56 × 10^−9^, higher in males), however, showed strong gender differences. This is an interesting scenario where the super-pathway grouping is too coarse to detect changes, but still a smaller group of specific molecules in a sub-pathway is coordinately changed between males and females.

#### Energy metabolism

Four metabolites associated to energy metabolism showed significant differences between genders. Phosphate was detected to be considerably higher in females (p = 4.46 × 10^−45^) which is in accordance with previous results from a coronary heart disease (CHD), study (Tonelli et al. 2005). Similarly, acetylphosphate also appeared higher in women (p = 2.38 × 10^−23^). This association has, to the best of our knowledge, not been described in literature yet. Two merely significant associations are furthermore conferred by succinylcarnitine (p = 1.92 × 10^−6^) and malate (p = 3.29 × 10^−5^), both with slightly higher concentrations in males. The phosphate, succinylcarnitine and malate signals replicated in the validation cohort, while acetylphosphate was not measured in that study. The entire class of energy-related metabolites was significantly higher in females (p = 4.12 × 10^−8^). This signal was mostly dominated by the ‘Oxidative phosphorylation’ sub-pathway (p = 1.69 × 10^−39^), which consists of the above-mentioned two metabolites, phosphate and acetylphosphate. The pathway results were confirmed in the validation cohort.

#### Lipids

The strongest associations in the ‘Lipid’ class were constituted by three androsterone sulfate derivatives, with elevated levels in males. 5alpha-androstan-3beta, 17beta-diol disulfate represented the highest gender difference in the entire analysis (p = 1.71 × 10^−193^). These metabolites are degradation variants of androsterone, a steroid sex hormone related to testosterone. Different levels of sex hormones between males and females are amongst the most obvious and expected findings, and thus can be seen as a ‘proof of principle’ for our metabolomics dataset. All steroid signals replicated in the validation cohort. Interestingly, we detected these differences despite the fact that most women in this study are considered to be post-menopausal (age range 60.51 ± 8.77 for females).

In addition to steroid hormones, we observed elevated levels of glycerol (p = 1.01 × 10^−73^) and myristoleate (p = 3.64 × 10^−70^) in females amongst the top list of lipids. Plasma glycerol levels have previously been described to show sexual dimorphisms during fasting (Mittendorfer et al. 2001) and exercise (Hellström et al. 1996). Monounsaturated fatty acids (like myristoleate) also have been reported to be higher in females (Rogiers 1981). More generally, all significantly affected fatty acids in the dataset show higher concentrations in females, which is confirmed in the validation cohort (glycerol was not measured in that study).

In accordance with the single metabolite findings, the pathway group of steroids was systematically higher in males (p = 4.34 × 10^−78^). Other lipid classes, especially around fatty acid metabolism, also display strong differences. For example, “fatty acid metabolism” showed elevated activity (p = 1.25 × 10^−30^) in males and “fatty acid, branched” (p = 2.27 × 10^−29^), “Sphingolipid” (p = 6.76 × 10^−23^) and “Medium chain fatty acid” (p = 3.01 × 10^−15^) were higher in females. Similar to the nucleotide case, the entire class of lipids was not coordinately affected in one or the other direction (p = 0.18). The results for fatty acids and sphingolipids are in general accordance with our previous study on the same cohort (Mittelstrass et al. 2011).

Interestingly, the phospholipid pathways signals were rather weak (p = 3.26 × 10^−8^), which is in contrast to our previous findings for lysophosphatidylcholines (lyso-PCs). The majority of discrepancies in single metabolite associations between the present and the previous study are for lyso-PC metabolites. As outlined at the beginning of this section, we expect differences in the precision of measurements between the targeted and non-targeted measurements. Moreover, the non-targeted metabolomics platform applied in this study distinguishes between sn-1 and sn-2 lysophosphatidylcholines (i.e. the binding position of the fatty acid side chain at the glycerol backbone), whereas the platform from the previous study always measures total sums of side chains.

#### Nucleotides

For the class of nucleotides, urate showed a highly significant difference with higher concentrations in males (p = 7.04 × 10^−114^). This association is well known and manifests in a substantially higher prevalence of gout in males (So and Thorens 2010). The association was investigated in detail in a recent study published by our group, which was based on the same dataset as in the present study (Albrecht et al. 2013). A similar, but considerably weaker association was detected for the urate metabolite allantoin (p = 6.84 × 10^−8^). The sources of blood allantoin are not fully understood. The metabolite is believed to originate either from non-enzymatic oxidation of urate (Sautin and Johnson 2008) or external sources such as vegetables (Todd et al. 2006). Interestingly, a previous study with 134 individuals also reported higher concentrations of allantoin in men (Pavitt et al. 2002). Finally, we observed weak but significant differences for inosine (p = 7.47 × 10^−8^, higher in females), hypoxanthine (p = 3.54x10^−5^, higher in females) and pseudouridine (p = 8.47x10^−5^, higher in males), all of which do not appear to have been described in literature before. The urate association could be replicated, while the hypoxanthine and pseudouridine signals did not replicate, probably due to lack of power. Allantoin and inosine were not measured in the validation cohort.

At pathway level, we observed strong gender differences for the sub-pathway purine/urate metabolism (p = 5.85x10^−97^), also with higher concentrations in males, but virtually no differences for the entire group of nucleotides (p = 2.16 × 10^−4^). This is another example where the super-pathway is too coarse to identify gender-specific signals, but single sub-pathways are affected. The major contribution to the purine/urate pathway was by urate itself, which we already described among the most strongly changed metabolites above.

#### Peptides

A series of peptides showed profound differences between males and females. Interestingly, the top 5 peptide hits are gamma-glutamyl dipeptides (p = 3.92 × 10^−99^ to p = 2.93 × 10^−14^, all higher in males). All five associations could be replicated. GGT1, gamma-glutamyl transferase 1, is an enzyme that generates gamma-glutamyl dipeptides by transferring the glutamyl moiety from glutathione to amino acids. Sexual dimorphisms for this enzyme have been previously reported in rats (Fujiwara et al. 1982) and humans (Skurtveit and Tverdal 2002). Moreover, genetic variation in the GGT1 enzyme induces concentration changes in the gamma-glutamyl dipeptides (Krumsiek et al. 2012). In this study, we could show for the first time that also the substrates of the enzyme GGT1 showed substantial gender differences, but could not detect a dimorphism for the genetic GGT1-dipeptide effects (see Sect. 5.2 below).

Analogously to these single-metabolite findings, the class of gamma-glutamyl peptides was elevated in males (p = 9.34 × 10^−60^). We observed a modest elevation in males for the entire class of peptides (p = 6.47 × 10^−15^) and no differences for other dipeptides (p = 0.25).

#### Xenobiotics

Three metabolites classified as xenobiotics, i.e. substances that are neither produced nor naturally occur in the human body, showed significant differences. These include 4-vinylphenol sulfate (p = 6.43 × 10^−35^), piperine (p = 1.46 × 10^−11^) and 2-hydroxyisobutyrate (p = 2.56 × 10^−5^), all of which were higher in males. There might be different explanations for these observed gender-specific levels of xenobiotics. For example, piperine is a component of black pepper (Srinivasan 2007). The association might be either due to different dietary habits between the genders or might represent differential metabolization efficiencies caused by known dimorphisms in cytochrome C expression (Nicolson et al. 2010). As another example, derivatives of the plant metabolite 4-vinylphenol occur, among others, in wine, beer and tobacco (Etievant 1981; Rodgman and Perfetti 2013). Again, the observed differences might be caused by life-style factors or intrinsic differences in degradation of xenobiotics. Only the 4-vinylphenol sulfate hit replicated, 2-hydroxybutyrate was not measured in the validation cohort.

At the pathway level, gender-specific signals were rather weak. The entire group of xenobiotics was detected to be slightly higher in males (p = 6.47 × 10^−4^), but none of the sub-pathways was significantly different.

### A network view of gender differences

In the next step, we generated a network view of interactions between the measured metabolites to get an unbiased description of the underlying metabolic pathways. To this end, we generated a Gaussian graphical model (GGM), a correlation-based model which statistically extracts pathway relationships from large-scale metabolomics data and thereby groups biochemically related molecules (Krumsiek et al. 2011, 2012; Shin et al. 2014) (see Sect. 4). The advantage of such a data-driven network approach is the lack of necessity for precise annotations of each metabolite. We can even incorporate unknown metabolites, which had to be excluded for the analyses above, into these networks. In a previous study we demonstrated that GGMs place unknown metabolites into the correct biochemical context (Krumsiek et al. 2012).

**Fig. 4:**
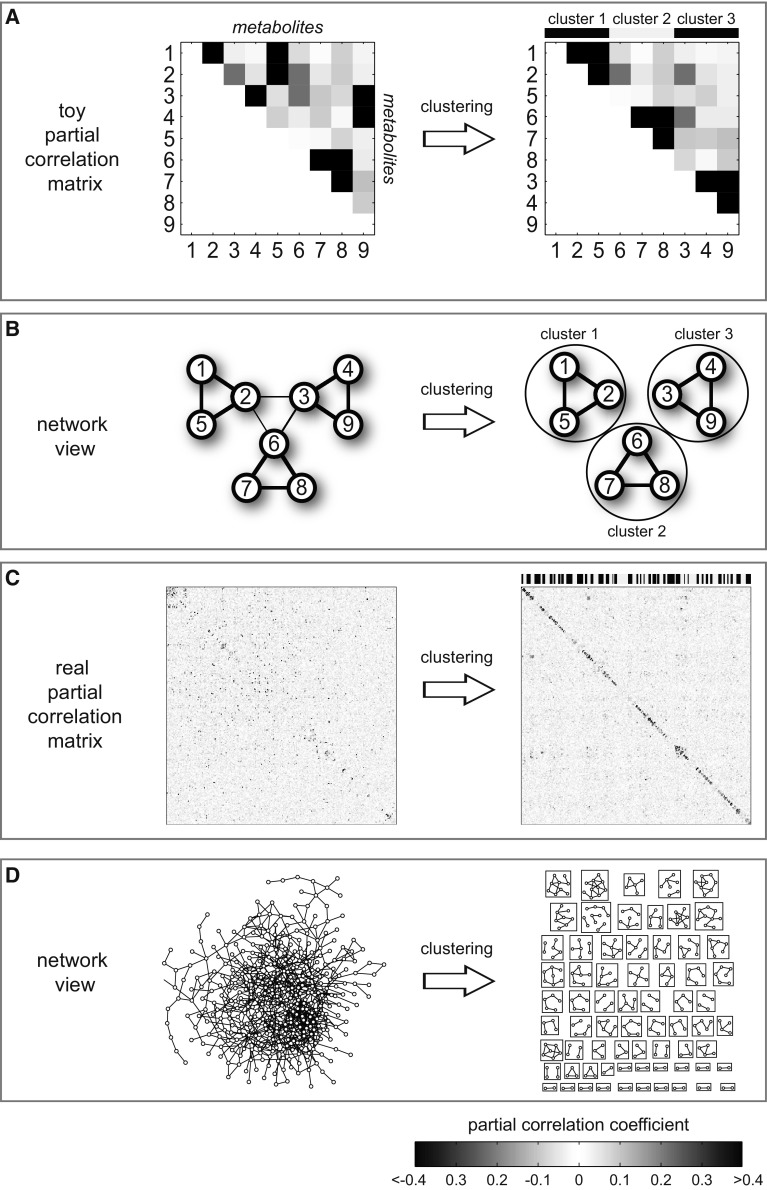
Network clustering approach. **a** Clustering of an artificially constructed partial correlation matrix. Metabolite sets with strong partial correlations are collected in clusters. **b** Network representation of the same clustering process. Strong links lead to clustering of the respective nodes. **c** Clustering of the real partial correlation matrix into 75 clusters. **d** Network representation of the metabolite network clustering process. A detailed description of the clustering algorithm can be found in Supplementary material 6

We mapped the −log_10_ p values from the single metabolite statistical analysis to this network (full network in Supplementary material 4), followed by a systematic clustering and enrichment approach (Figs. 1, 4). A detailed description of the procedure can be found in the Methods section and Supplementary material 5. Briefly, we first used the partial correlation matrix underlying the GGM to cluster groups of highly connected metabolites, and then determined whether this group is coordinately affected by gender differences. For this dataset, we chose an ad-hoc number of *k* = 75 clusters. In a separate analysis we verified that the clustering does not depend on the choice of *k* (Methods, Supplementary material 6). Furthermore, we assigned a quality score to each cluster, measuring the strength of network connections of the metabolites within the cluster.

**Fig. 5:**
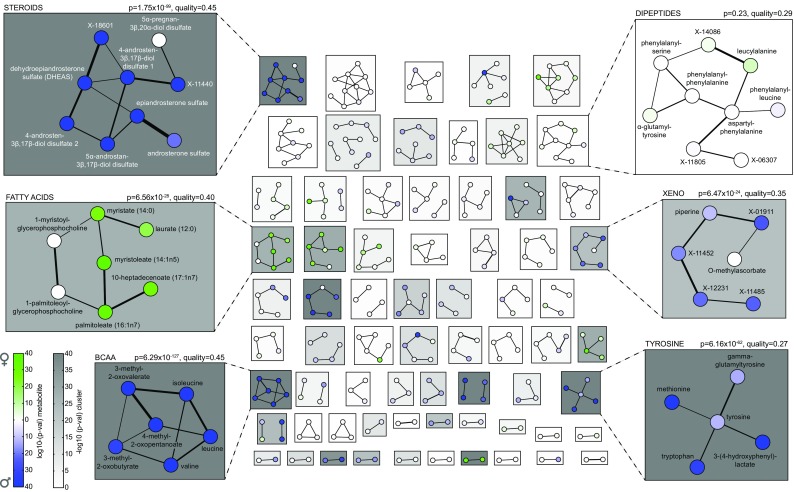
Network cluster enrichment. Each *box* represents a cluster of metabolites. *Node colors* indicate gender effects. *Box background colors* represent the enrichment p value of the respective cluster. Six clusters are highlighted, which are specifically described in the main text. Note that clusters might contain disconnected nodes due to low quality of the respective cluster

The resulting clusters along with their significance regarding gender are shown in Fig. 5. Replication was performed by analyzing phenotypic effects of the same clusters in the replication data (not by repeating the entire clustering process). A full list of results can be found in Supplementary material 7. In the following, we present six exemplarily highlighted clusters from Fig. 5 and specifically stress the additional benefit gained from the network method.

The first cluster TYROSINE contains metabolites from four different sub-pathways and two different super-pathways according to the predefined pathways used in the previous section. Specifically, tyrosine and 3-(4-hydroxyphenyl)lactate are from the “Phenylalanine & tyrosine” pathway, tryptophan is from the “Tryptophan” pathway, methionine is from the “Cysteine, methionine, SAM, taurine” pathway, and gamma-glutamyltyrosine belongs to the “gamma-glutamyl” peptide pathway. This pathway is significantly higher in males (p = 6.16 × 10^−62^) and also replicates in the validation cohort (p = 1.16 × 10^−25^). Importantly, the cluster contains connections across the borders of predefined pathway annotations. Thus the dimorphism in this biochemical module could not have been detected without the network approach.

Cluster XENO contains the xenobiotic substance piperine, the vitamin metabolite O-methylascorbate and four unknown metabolites. The cluster had a high quality score (q = 0.36), was up-regulated in males (p = 6.47 × 10^−24^), and also replicated in the validation cohort (p = 7.25 × 10^−5^). While the annotation of the unknown metabolites is still missing, the data-driven network is able to put all compounds into a metabolic context. Similar to the TYROSINE cluster, this module is affected by sexual dimorphisms and could not have been found with common methods since they completely omit unknown metabolites.

In contrast, cluster BCAA is an example of how the network method also recovered signals that were detected by the common pathway analysis above. It represents a cluster of three BCAAs and their direct degradation products, and showed higher concentrations in males. Similarly, the sexual dimorphisms in clusters STEROIDS and FATTY ACIDS were also already detected in the pathway analysis.

However, for several clusters we get additional information on the biochemical associations between metabolites using the network approach. For example, while several androsterone-based and one pregnan-based metabolites are strongly connected in the STEROID cluster (which is biochemically plausible), they show substantially different associations to gender. The entire cluster is up-regulated in males (p = 1.75 × 10^−99^), but 5α-pregnan-3β, 20α-diol disulfate appears almost unaffected (p = 0.04). Pregnanes are precursors for both androgens and estrogens and thus expected to be similar for both genders. Similar scenarios of highly connected metabolites with differential effects can be found in the FATTY ACIDS and XENO clusters.

Irrespective of sexual dimorphisms, cluster DIPEPTIDES demonstrates how the quality measure helps to detect stable network clusters in the metabolome. The cluster had a reasonably high quality (q = 0.29) but negligible gender differences (p = 0.23, p = 0.13 in the replication cohort). Thus, this is an example of a highly connected pathway region that appears to be stably regulated between the two genders.

### A gender-specific genome-wide association study

Up to this point, the analysis included metabolomics measurements only. Previous studies have established strong links between serum metabolite concentrations and common genetic polymorphisms (Kettunen et al. 2012; Nicholson et al. 2011; Rhee et al. 2013; Shin et al. 2014). To check whether the observed gender differences are due to differential influences of these polymorphisms in males and females, we performed a gender-stratified GWAS with metabolites as phenotypic traits. To this end, we determined for each SNP-metabolite combination whether the effect of the SNP onto the metabolite is statistically different between the genders. We analyzed a subset of 1703 individuals from our study cohort for which both metabolites and genome-wide SNP data were available. This subset included 875 females and 828 males. The analysis was limited to 277 known and 188 unknown metabolites that had at least 250 valid measurements for females and males. Genotyping information for a total of 9,277,001 SNPs was available after quality control and filtering (see Sect. 4). This analysis represents an extension of our previous study (Mittelstrass et al. 2011) with the broader set of metabolites used in this study and a more fine-grained set of SNPs. The sample size is comparable to the previous study (1703 here vs. 1809 previously).

Only two metabolite-SNP associations reached the genome-wide significance level of 1.08 × 10^−10^: Glycine associated with rs1047891 (p = 1.8 × 10^−45^) and rs715 (p = 1.6 × 10^−43^), both of which lie in the autosomal CPS1 gene. This represents the same genetic association described in our previous paper (Mittelstrass et al. 2011). CPS1 encodes a mitochondrial enzyme which plays a role in protein and nitrogen metabolism of the hepatic urea cycle. Interestingly, even with the much broader set of SNPs and metabolites analyzed here, the glycine-CPS1 association appears to be the only gender-specific genetic effect on blood metabolites. Note that unknown metabolites were included in this analysis, but did not yield any further genome-wide significant results. A detailed list of GWAS results including plots can be found in Supplementary material 8.

## Discussion

In this study, we systematically analyzed gender-specific differences in the metabolism of 1756 participants from a German cross-sectional cohort. Out of 507 blood metabolites measured on a non-targeted metabolomics platform, more than one-third (180) showed statistically significant differences between males and females. 114 of the 180 hits could be checked in an independent population cohort comprising 1000 subjects, where 88 hits replicated (77.2 %). Thus, a large fraction of the identified gender-specific differences can be considered stable across populations. We found well-known metabolic dimorphisms, for example for steroid hormones, BCAAs and creatine, but also a series of previously unreported differences.

Pathway analysis based on eight major super-pathways (“Amino acids”, “Carbohydrates”, “Cofactors and vitamins”, “Energy”, “Lipids”, “Nucleotides”, “Peptides”, “Xenobiotics”) and their sub-pathways (e.g. “glycolysis”, “BCAAs”, “steroids”) provided interesting insights into gender-specific pathway activity on different levels. For example, the entire set of amino acids was systematically elevated in males, with different sub-pathways around BCAAs, glutamate and lysine constituting the strongest contributions. On the other hand, the class of lipids was not changed in its entirety, while specific sub-pathways (e.g. steroids or fatty acids) carry strong gender-specific effects (Fig. 3).

In addition to the pathway analysis, which is limited to predefined pathways and metabolites with known chemical structure, we implemented a novel network clustering approach (Fig. 4). This provides an unbiased, purely data-driven view on the parts of metabolism with major gender-specific differences. This approach has two major advantages over classical pathway analysis. First, the method can find biochemical modules across the borders of predefined pathways. For example, we discovered a module containing tyrosine-related metabolites from two different predefined pathways. All five metabolites of the identified biochemical module were coordinately higher in males. With most common definitions, however, these metabolites would belong to four different pathways, and thus the module could not have been found without the network approach. Second, the method readily incorporates unknown metabolites into the network. In our dataset, 37 % of the measured metabolites are unknown, which have to be completely omitted in pathway analysis otherwise.

Both pathway approaches revealed gender-specific differences in all major parts of metabolism. Besides more obvious metabolic differences between men and women, such as higher levels of the steroid androsterone and its derivatives in males, we also observed systematically higher blood concentrations of amino acids in males. In particular, branched chain amino acids (BCAAs) and their degradation products exhibited very clear gender differences (p = 6.29 × 10^−127^) with higher levels in males. BCAAs play an important role in muscle metabolism and have been shown to differ between genders both in their basal levels as well as their oxidation kinetic during exercise (Fujita et al. 2007; Lamont et al. 2001). In many studies, BCAAs and their degradation metabolites have been reported as early markers of insulin resistance and diabetes (Lu et al. 2013; Newgard et al. 2009). Gender differences in BCAA metabolism may thus have implications for the care of diabetes patients.

We also find significant effects for metabolites and pathways related to CHD, which has a substantially higher prevalence in men (Go et al. 2013). For example, there are strong gender differences in lipid metabolism, especially for medium- and long-chain free fatty acids (Fig. 5, FATTY ACID cluster). The fatty acid myristate has previously been described to affect LDL and HDL lipoprotein levels (Zock et al. 1994), which are strongly different between genders (Wang et al. 2011) and represent well-known risk markers for CHD (Arsenault et al. 2011). As another example, we observed marked differences in gamma-glutamyl dipeptides, which are products of the GGT (gamma-glutamyl transferase) enzyme. Serum GGT level in turn is a marker for alcohol consumption and obesity (Puukka et al. 2006) and represents another risk factor for CHD and overall mortality (Jiang et al. 2013). Such pathway associations might represent starting points to elucidate the molecular basis of sexual dimorphisms in a complex disease such as CHD.

In order to test whether the gender differences are conferred by gender-specific effects of genetic variants on the metabolite levels, we performed a gender-stratified GWAS. Although metabolite levels are strongly influenced by genetic variance in general (Shin et al. 2014), we only found a single gene-metabolite association (CPS1-glycine) that was significantly different between genders. Our observation that sexual dimorphisms in genetic influences have no major impact on gender-specific metabolite levels is similar to findings from gene expression studies. Jansen et al. (2014) also report strong differences in gene expression, which cannot be explained by major dimorphisms in genetic influences. A gender-specific association between urate and nine loci, detected in a substantially larger cohort of almost 30,000 individuals (Kolz et al. 2009), could not be replicated in our dataset. An explanation might be the power in the present study, which might not be sufficient to detect all such dimorphisms. Moreover, the statistically and genetically complicated analysis of dimorphisms in X chromosomal effects has been omitted in our study.

In addition to higher sample sizes and analysis of the X chromosome, our study and methodology could be extended in several directions. (1) The menstrual cycle should be considered in a gender-specific analysis. In our present cohort, this was not an issue since the dataset mainly included women after menopause. Future studies spanning a larger age range, however, should investigate this important factor. (2) We only used after-night fasting serum samples from Caucasian individuals for our analysis. It is to be expected that under challenge conditions (e.g. diet, drugs or exercise), further interesting pathways will emerge that are not detectable under steady state conditions. Cohorts including different ethnicities might reveal further metabolites showing sexual dimorphisms, and would allow for a comparison of ethnically stable or differentially regulated parts of the metabolism. Furthermore, systematic confounders such as the composition of the gut micriobiome should be taken into account wherever available. (3) An important aspect in all gender-related research is the differentiation between biologically originating sex differences and socially influenced gender effects. There is a plethora of differences in behavior, disease prevalence and treatment. Since a randomized-controlled study design to separate these factors is not realistically possible, other routes have to be taken. For example, an extensive collection and analysis of life-style and medical parameters could allow dissecting biological and behavioral differences. (4) While various previous studies have established links between blood metabolites and intracellular and physiological processes, e.g. (Krug et al. 2012; Newgard et al. 2009; Shin et al. 2014), this body fluid is certainly not fully representing the entire human metabolism. To complete the picture of sexual dimorphisms in the metabolome, it will be interesting to investigate other body fluids and, if available, tissue samples in future studies. (5) When interpreting epidemiological study results, such as the ones presented here, one must be aware that statistical significance does not necessarily imply biological relevance. Therefore, our study should be mainly considered as hypothesis-generating. Dedicated follow-up studies would be needed to establish the functional role of specific metabolic dimorphisms we observed. (6) The pathway analyses proposed in this study are generally applicable and can directly be transferred to the analysis of other phenotypes. (7) On the methodological side, the pathway activity and clustering methods could be extended. For example, our z-score approach only detects one-sided effects, i.e. groups of metabolites that are either high in males or females. For the present study, this was intentional, but might be revised for the investigation of other phenotypes.

Taken together, we report a wide range of gender-specific differences in the human serum metabolome, covering a variety of different pathways and processes. This provides insights into the basic molecular differences between the two genders. Moreover, many of the affected pathways are known to be relevant in different diseases with gender-specific susceptibility, such as CHD and gout. Extensive knowledge of the underlying metabolic differences might lead to concrete starting points for further research, in order to develop gender-specific personalized health care.

## Materials and Methods

### Study cohorts and metabolomics measurements

Data from 1756 fasting serum samples of the German KORA F4 population cohort (Holle et al. 2005) were used, comprising 903 females and 853 males. Age distribution was 60.51 ± 8.77 for females (mean ± standard deviation) and 61.17 ± 8.79 for males. BMI distribution was 27.86 ± 5.25 for females and 28.47 ± 4.29 for males. Metabolomics measurements were performed using ultrahigh-performance liquid-phase chromatography and gas-chromatography separation, coupled with tandem mass spectrometry by Metabolon, Inc. Metabolites were identified following the Metabolomics Standardization Initiatives guidelines (Sansone et al. 2007). A detailed description of the experimental procedures and metabolite identification steps can be found in Supplementary material 9. Levels of identification for each metabolite are provided in Supplementary material 2. A total of 515 metabolites were quantified, with 324 metabolites of known identity and 191 signals which are not annotated with a chemical structure yet (“unknowns”). Eight metabolites with less than 10 valid measurements or zero variance were excluded, leaving 507 total metabolites, including 318 knowns and 189 unknowns. Each known metabolite is classified into one of the following eight major metabolic groups (“super-pathways”): “Lipid”, “Carbohydrate”, “Amino acid”, “Xenobiotics”, “Nucleotide”, “Energy”, “Peptide”, “Cofactors and vitamins”. Each super-pathway is subdivided into two or more “sub-pathways” like “Oxidative phosphorylation”, “Carnitine metabolism”, and “Valine, leucine and isoleucine metabolism”.

Replication samples were contributed by the Study of Health in Pomerania (SHIP-TREND) (Völzke et al. 2011), providing 1000 fasting plasma samples, thereof 561 females and 439 males. Metabolomics measurements were performed on a similar platform as the discovery study, however without the GC–MS runs, leading to a total of 662 measured metabolites in the replication cohort. Age distribution was 50.14 ± 13.17 for females (mean ± standard deviation) and 50.08 ± 14.24 for males. BMI distribution was 26.99 ± 5.12 for females and 27.85 ± 3.75 for males.

A detailed list of measured metabolites including pathway annotations and coefficients of variation can be found in Supplementary material 2.

## Statistical analyses

Raw metabolite quantifications were median-scaled to one for each run day to correct for day-to-day variance due to instrument drift during runs. Subsequently, concentrations were log_2_-transformed, since the log-transformed concentrations were closer to a normal distribution than the untransformed values. To this end, the non-normality for all log_2_-transformed and non-transformed metabolite was assessed by Kolmogorov–Smirnov tests (Massey 1951) and then systematically compared in the discovery cohort (Supplementary material 10). Metabolite differences between males and females were investigated by standard linear regression analysis. Metabolite concentration was used as the dependent variable, gender as explanatory variable and age and BMI as covariates. In order to adjust for multiple hypotheses testing, stringent Bonferroni correction was applied, leading to an adjusted significance level of 0.05/507 = 9.86 × 10^−5^ in the KORA cohort. For each regression analysis, missing values for the respective metabolite were ignored, leading to varying sample sizes for each metabolite association.

Pathway enrichment was calculated using aggregated z-scores. First, metabolite concentrations over all samples for each metabolite were transformed into z-scores. Positive values indicate higher metabolite concentration than the population mean, whereas negative values represent lower concentrations than the mean. For each super-pathway and sub-pathway, average z-score over all metabolites contained in that pathway were computed. This provides a measure of average expression or activity in that pathway for each sample. The aggregated pathway values were then subjected to the standard linear regression analysis for gender effects described above.

### Network generation, clustering & cluster enrichment

A GGM was calculated as described previously (Krumsiek et al. 2011). Briefly, for each pair of metabolites, partial correlations conditioned against all other metabolites as well as age, gender and BMI were computed. The calculation requires a full data matrix without missing values, thus a series of preprocessing steps was necessary. First, metabolites with more than 90 % missing values were removed from the dataset, since no reasonable correlation calculation is possible for those measurements. This step reduced the number of metabolites for the network analysis from 507 to 494, leaving 311 knowns and 183 unknowns. Remaining missing values were then imputed by drawing from a normal distribution with the same mean and standard deviation as the non-missing values from the respective metabolite. This approach lowers correlation between metabolites rather than artificially increasing it, favoring false negative edges over false positive edges.

The resulting GGM was then clustered with a k-means clustering approach on a transformed correlation matrix. Intuitively, nodes that are few steps apart in the network should get a higher similarity value for the clustering process than distant nodes. Since network distance is strongly dependent on the cutoff chosen, a cutoff-independent approach that utilizes the full partial correlation matrix was developed. For each pair of metabolites, the *multiplicative strongest path* was calculated. This represents the path from one metabolite to another metabolite over zero or more other metabolites which maximizes the product of edge weights. A visual description of the approach can be found in Supplementary material 5. The resulting strongest path matrix was then subjected to standard k-means clustering. For the present paper, an ad-hoc number of *k* = 75 clusters was chosen. We performed three stability analysis approaches to evaluate the clustering as such as well as the choice of *k*: (1) We calculated Silhouette coefficients (Rousseeuw 1987) to objectively evaluate the number of obtained clusters. (2) Sample bootstrapping (Efron 1979) was used to verify the stability of clustering. (3) We assessed whether high-quality clusters are preserved independent of the choice of *k*. Detailed descriptions and results can be found in Supplementary material 6.

To assess the quality of each cluster, a maximum spanning tree-based approach was implemented. This measure identifies the strongest possible connection of all metabolites in a cluster, while visiting each metabolite just once. The rationale for this approach is that simple measures, like the mean partial correlation over all pairs of metabolites in a cluster, do not account for the inherent sparsity of GGM. Again, a visual explanation of the approach can be found in Supplementary material 5.

Gender enrichments for each cluster were calculated analogously to the pathway enrichment approach (see above). For each cluster, the mean z-score was calculated as a measure of mean activity. The resulting activity scores were then subjected to standard regression analysis with gender as explanatory variable and BMI and age as cofactors.

### Genome-wide association studies

Genotyping was performed with the Affymetrix Axiom Genome-Wide Population-Optimized Human Array in all available samples of the KORA F4 cohort. Samples with less than 97 % call rate or differing genetic and phenotypic gender were excluded. All samples that passed quality control showed genetic European ancestry (PCA on genotypes in comparison with HapMap3 samples, results not shown). Before imputation SNPs with less than 98 % call rate, minor allele frequency <0.01 or a p value for deviation from Hardy–Weinberg equilibrium <5 × 10^−6^ were excluded. Pre-phasing of the genotypes was performed with SHAPEIT v2 (Delaneau et al. 2013) and imputation with IMPUTEv2.3.0 (Howie et al. 2009). The 1000 g phase1 all ethnicities (release v3, macGT1, August 2012) was used as reference panel. In total, 523,260 genotyped SNPs passed pre-imputation filtering and were imputed to 30,067,091 SNPs. In this study, only SNPs with minor allele frequency >0.01 and imputation quality (info) >0.4 were analyzed, resulting in a final number of 9,277,001 SNPs.

Gender-specific genome-wide association studies (GWAS) were then calculated from 1703 individuals for which both metabolites and valid genome-wide SNP data were available. Metabolites were filtered to 277 known and 188 unknown metabolites that had at least 250 valid measurements (leaving 465 metabolites). The GWAS were performed on residuals from a regression on age calculated for the log transformed metabolites for males and females separately without any further adjustment. Standard linear regression was used to analyze additive SNP effects using dosages with the package MixABEL (function GWFGLS) of the GenABEL (Aulchenko et al. 2007) suite. Beta estimates per SNP were then checked for gender-specific differences using the following statistic:

$$\frac{{beta_{females} \, - \,beta_{males} }}{{\sqrt {se_{females}^{2} \, - \,se_{males}^{2} } }}$$

which is approximately normally distributed (Paternoster et al. 1998). The Bonferroni-corrected p value for genome-wide analyses 5 × 10^−8^ (Pe’er et al. 2008) had to be further corrected for the number of metabolites. This led to a significance threshold of 5 × 10^−8^/465 = 1.08 × 10^−10^ for genome-wide significance.

## Data availability

Raw data from the KORA F4 study are available upon request from KORA-gen (http://epi.helmholtz-muenchen.de/kora-gen). Requests should be sent to kora-gen@helmholtzmuenchen.de and are subject to approval by the KORA board to ensure that appropriate conditions are met to preserve patient privacy. Formal collaboration and co‐authorship with members of the KORA study is not an automatic condition to obtain access to the data published in the present paper. More general information about KORA, including F4 study design and clinical variables, can be found at http://epi.helmholtz-muenchen.de/kora‐gen/seiten/variablen_e.php and http://helmholtz‐muenchen.de/en/kora-en/information-for-scientists/current-kora-studies.

## Electronic supplementary material

Below is the link to the electronic supplementary material.

Supplementary material 1 (XLS 25 kb)

Supplementary material 2 (XLSX 128 kb)

Supplementary material 3 (PDF 1957 kb)

Supplementary material 4 (ZIP 72 kb)

Supplementary material 5 (PDF 401 kb)

Supplementary material 6 (PDF 1147 kb)

Supplementary material 7 (XLS 60 kb)

Supplementary material 8 (PDF 201 kb)

Supplementary material 9 (PDF 69 kb)

Supplementary material 10 (PDF 123 kb)
