# Supplementary material for: Gender-specific pathway differences in the human serum metabolome
Source: Metabolomics. 2015 Aug 4;11(6):1815–33. doi: 10.1007/s11306-015-0829-0 (PMC4605991; doi:10.1007/s11306-015-0829-0)
Supplement: Supplementary file 3 — Supplementary material 3 (PDF 1957 kb) [file 11306_2015_829_MOESM3_ESM.pdf]

Boxplots of metabolite and pathway differences for KORA and the SHIP replication cohort.

Contents:

- KORA metabolite boxplots
- KORA pathway boxplots
- SHIP metabolite boxplots
- SHIP pathway boxplots

KORA metabolite boxplots

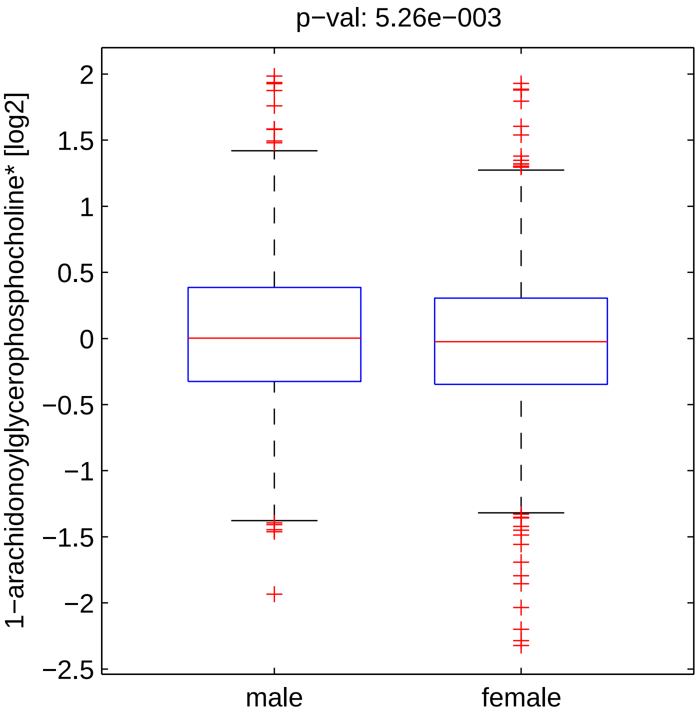
p−val: 5.26e−003
1−arachidonoylglycerophosphocholine* [log2]
2
1.5
1
0.5
0
−0.5
−1
−1.5
−2
−2.5
male
female

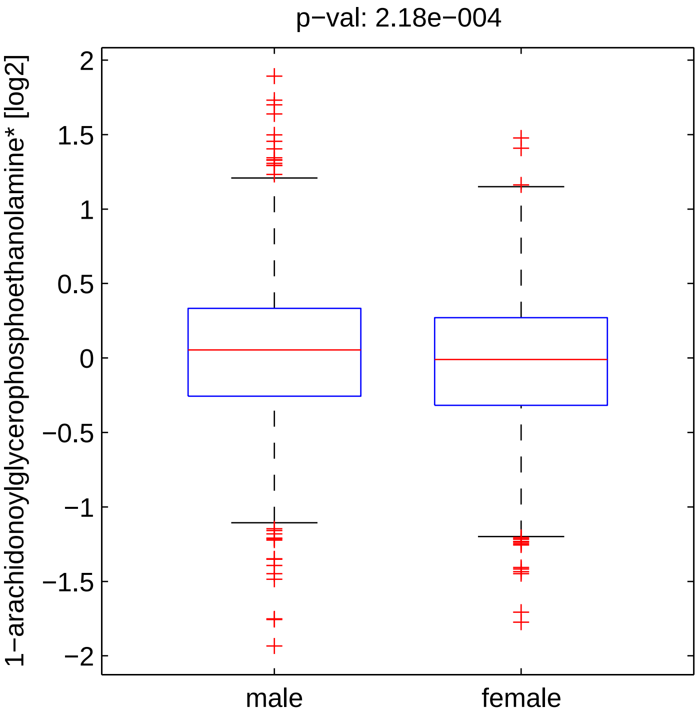

p−val: 2.18e−004
1−arachidonoylglycerophosphoethanolamine* [log2]
2
1.5
1
0.5
0
−0.5
−1
−1.5
−2
male
female

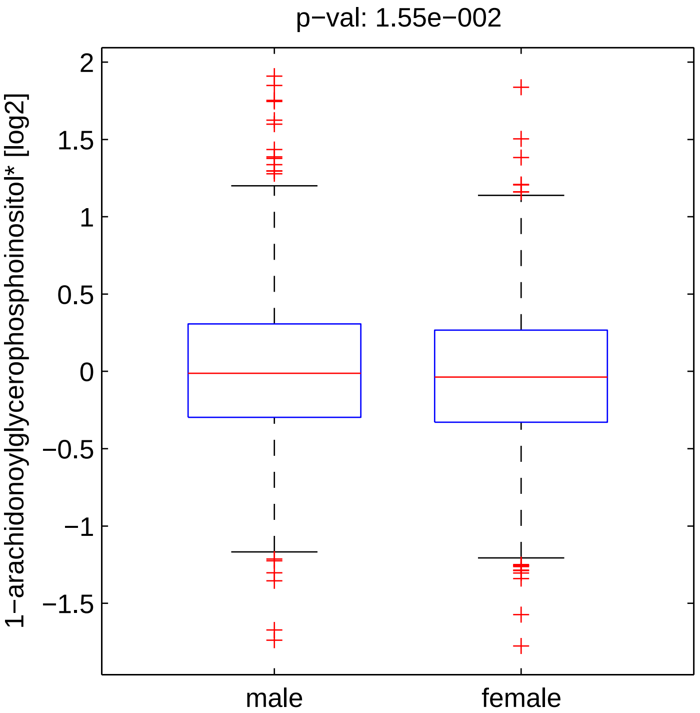

p−val: 1.55e−002
1−arachidonoylglycerophosphoinositol* [log2]
2
1.5
1
0.5
0
−0.5
−1
−1.5
male
female

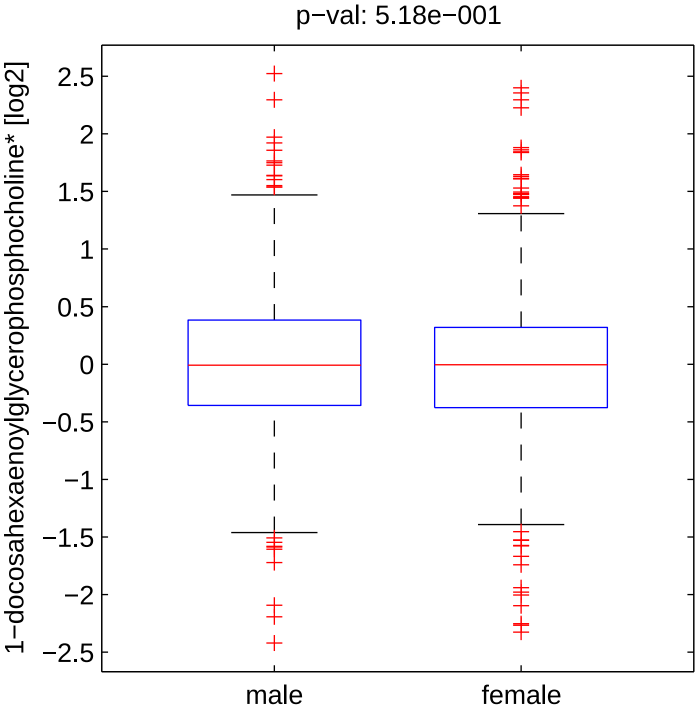
p−val: 5.18e−001
1−docosahexaenoylglycerophosphocholine* [log2]
2.5
2
1.5
1
0.5
0
−0.5
−1
−1.5
−2
−2.5
male
female

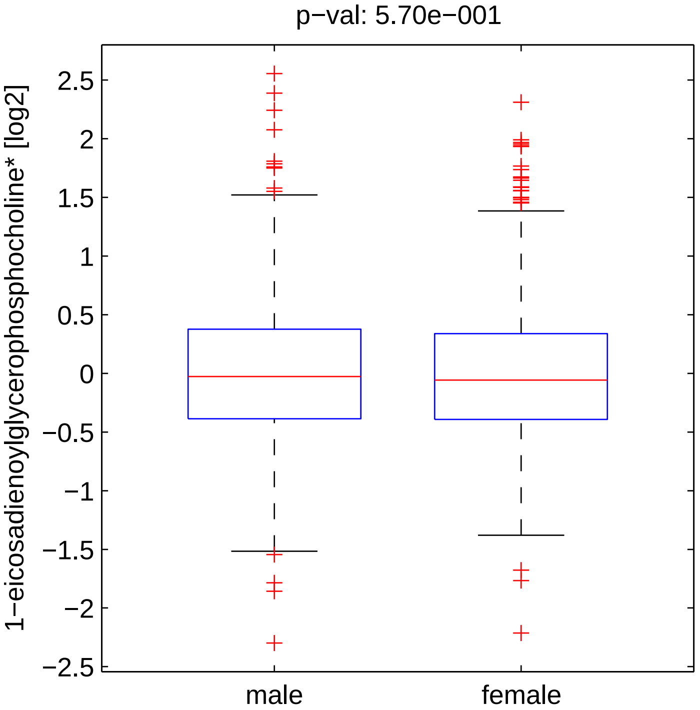
p−val: 5.70e−001
1−eicosadienoylglycerophosphocholine* [log2]
2.5
2
1.5
1
0.5
0
−0.5
−1
−1.5
−2
−2.5
male
female

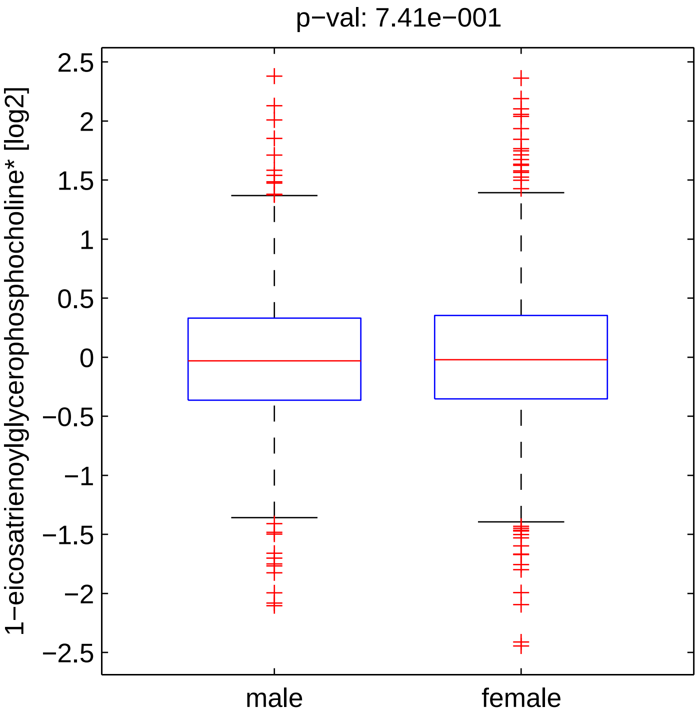
p−val: 7.41e−001
1−eicosatrienoylglycerophosphocholine* [log2]
2.5
2
1.5
1
0.5
0
−0.5
−1
−1.5
−2
−2.5
male
female

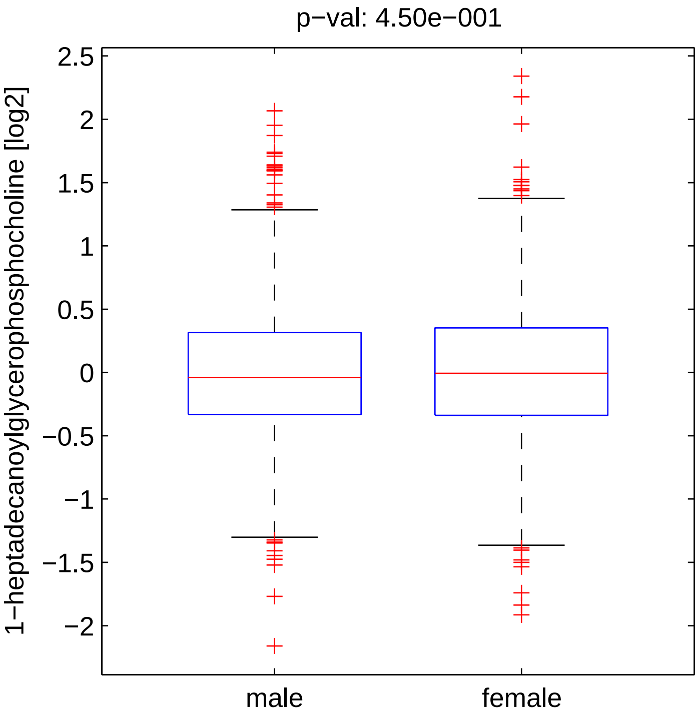

p−val: 4.50e−001
1−heptadecanoylglycerophosphocholine [log2]
2.5
2
1.5
1
0.5
0
−0.5
−1
−1.5
−2
male
female

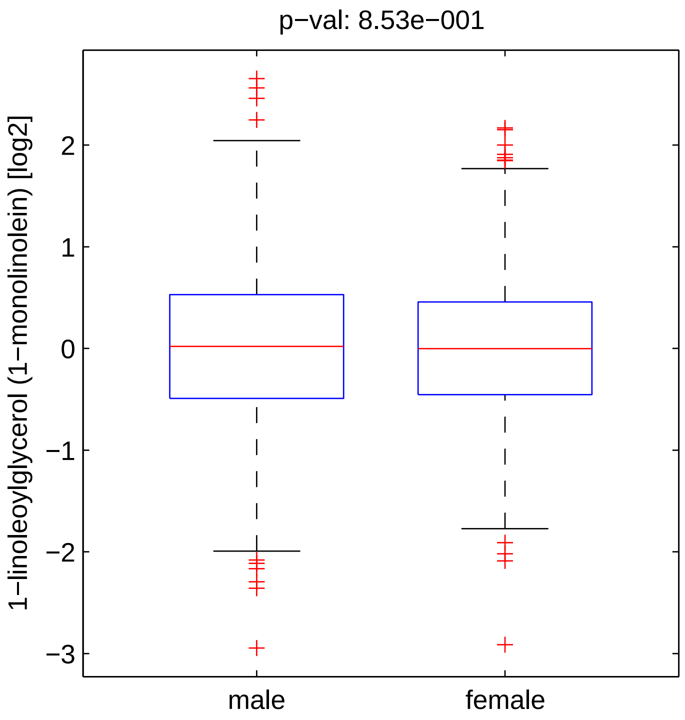
p−val: 8.53e−001
1−linoleoylglycerol (1−monolinolein) [log2]
2
1
0
−1
−2
−3
male
female

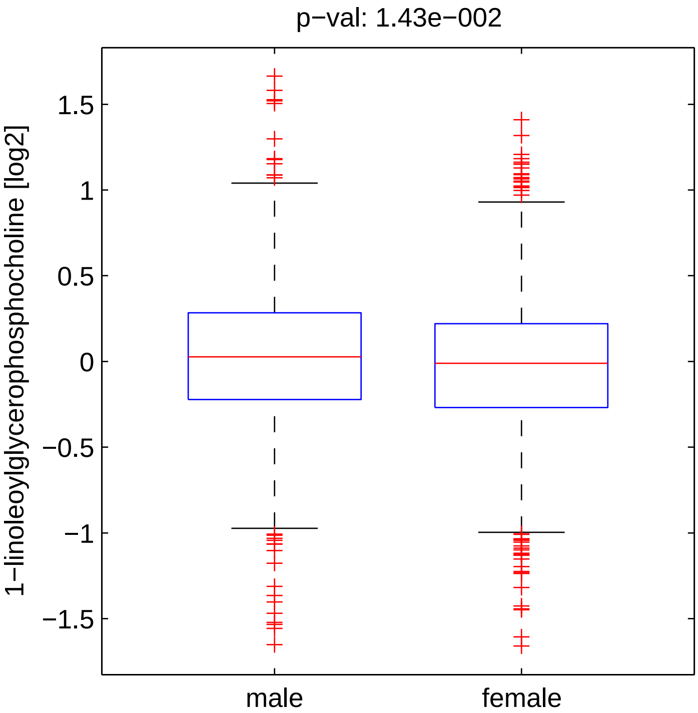

p−val: 1.43e−002
1−linoleoylglycerophosphocholine [log2]
1.5
1
0.5
0
−0.5
−1
−1.5
male
female

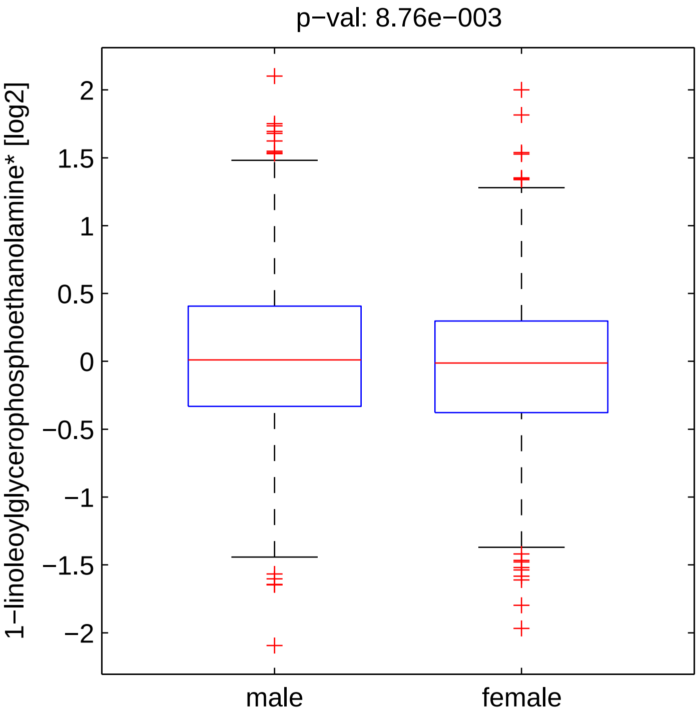

p−val: 8.76e−003
1−linoleoylglycerophosphoethanolamine* [log2]
2
1.5
1
0.5
0
−0.5
−1
−1.5
−2
male
female

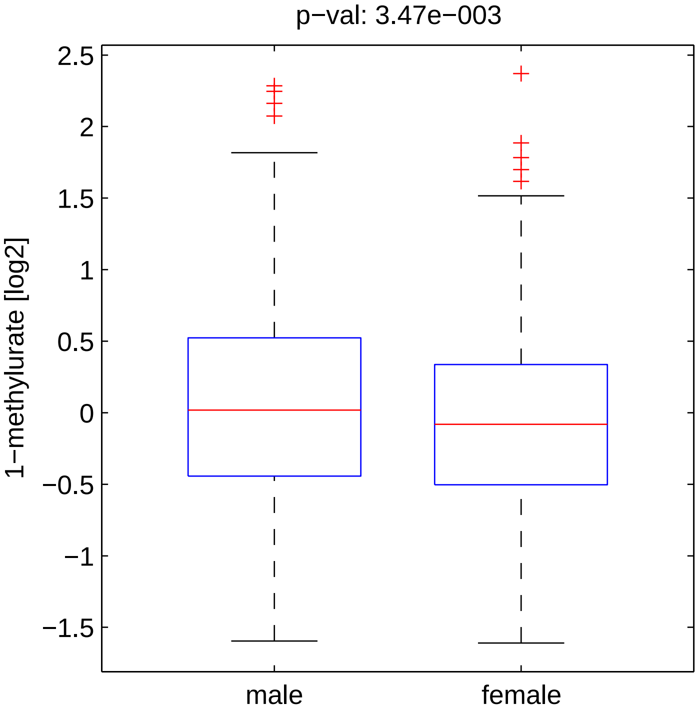
p−val: 3.47e−003
2.5
2
1.5
1
0.5
0
−0.5
−1
−1.5
1−methylurate [log2]
male
female

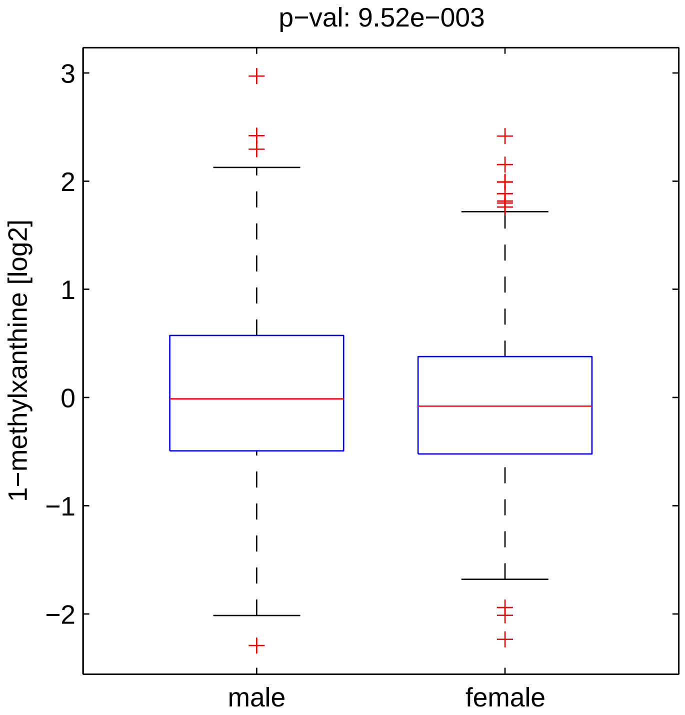
p−val: 9.52e−003
1−methylxanthine [log2]
3
2
1
0
−1
−2
male
female

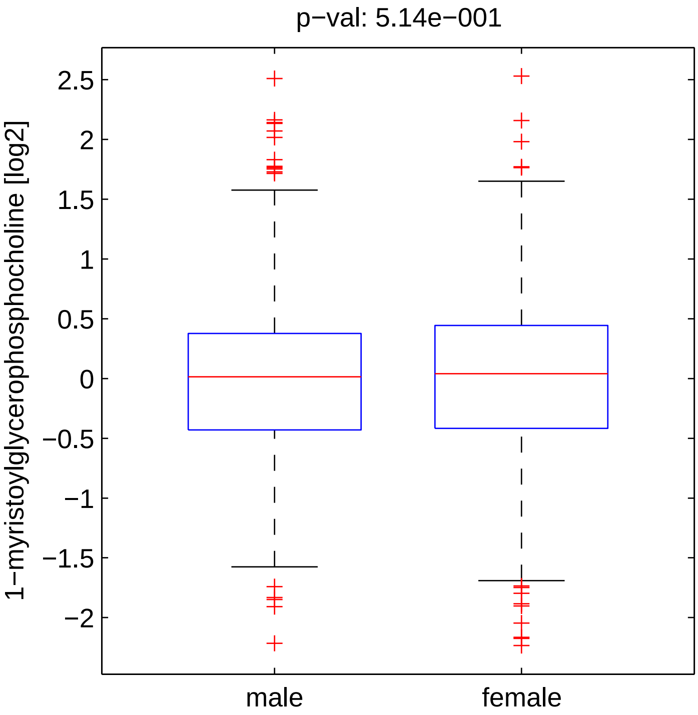

p−val: 5.14e−001
1−myristoylglycerophosphocholine [log2]
2.5
2
1.5
1
0.5
0
−0.5
−1
−1.5
−2
male
female

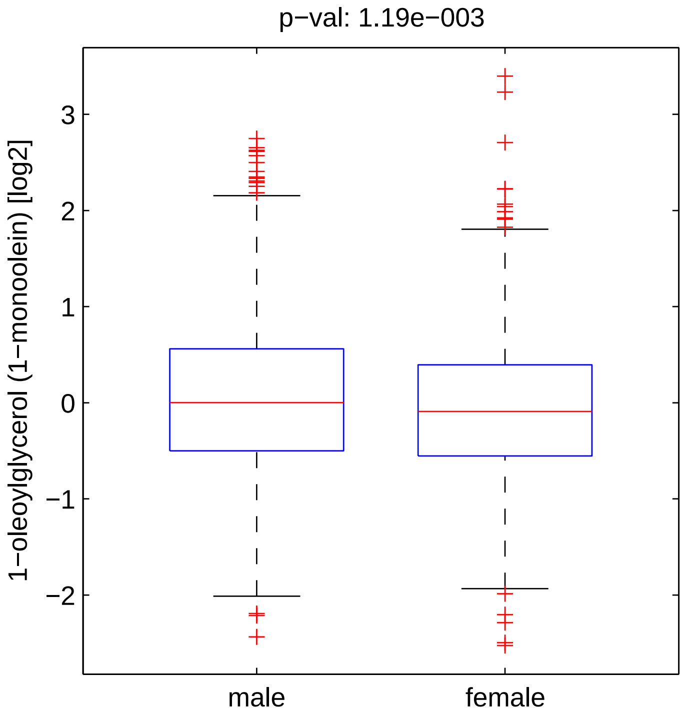
p−val: 1.19e−003
1−oleoylglycerol (1−monoolein) [log2]
3
2
1
0
−1
−2
male
female

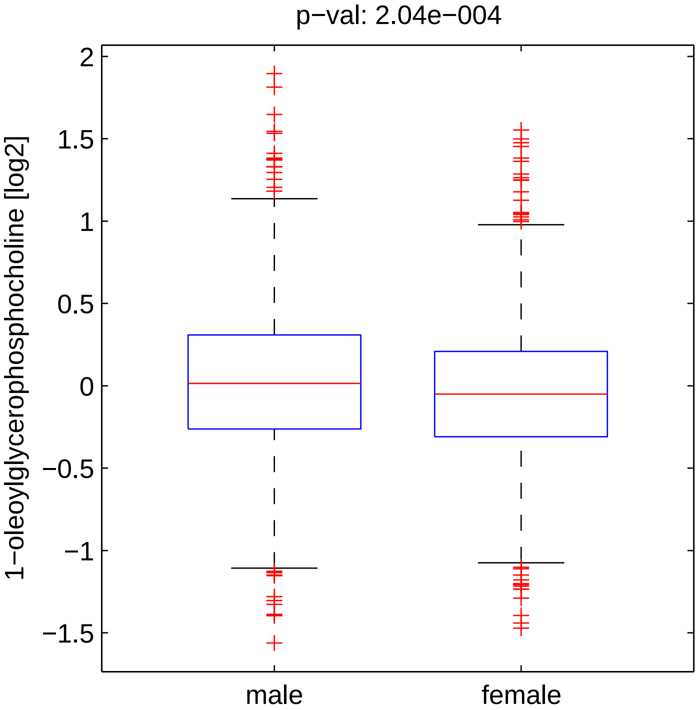

p−val: 2.04e−004
1−oleoylglycerophosphocholine [log2]
2
1.5
1
0.5
0
−0.5
−1
−1.5
male
female

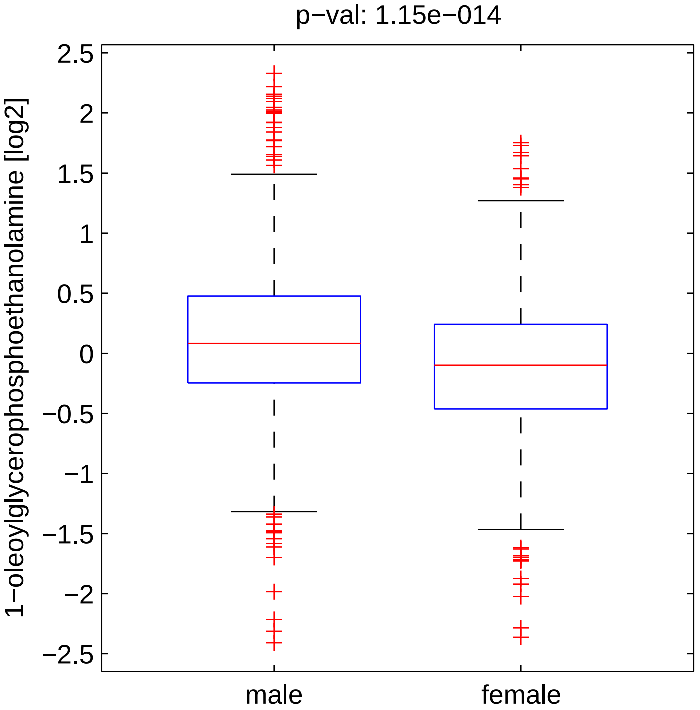

p−val: 1.15e−014
1−oleoylglycerophosphoethanolamine [log2]
2.5
2
1.5
1
0.5
0
−0.5
−1
−1.5
−2
−2.5
male
female

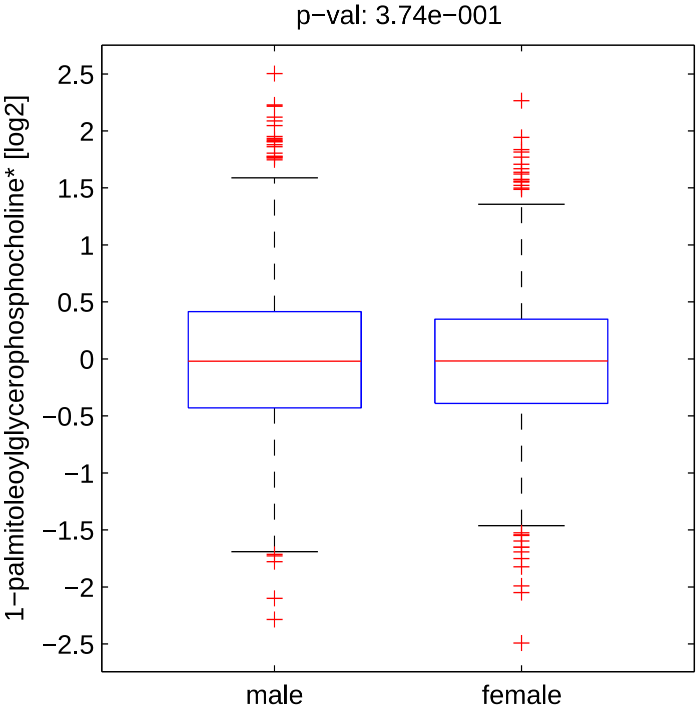

p−val: 3.74e−001
1−palmitoleoylglycerophosphocholine* [log2]
2.5
2
1.5
1
0.5
0
−0.5
−1
−1.5
−2
−2.5
male
female

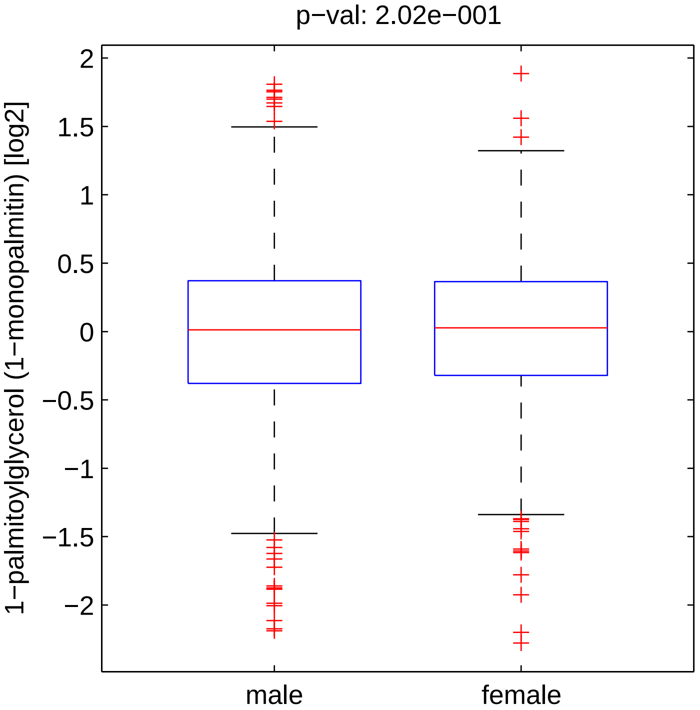

p−val: 2.02e−001
1−palmitoylglycerol (1−monopalmitin) [log2]
2
1.5
1
0.5
0
−0.5
−1
−1.5
−2
male
female

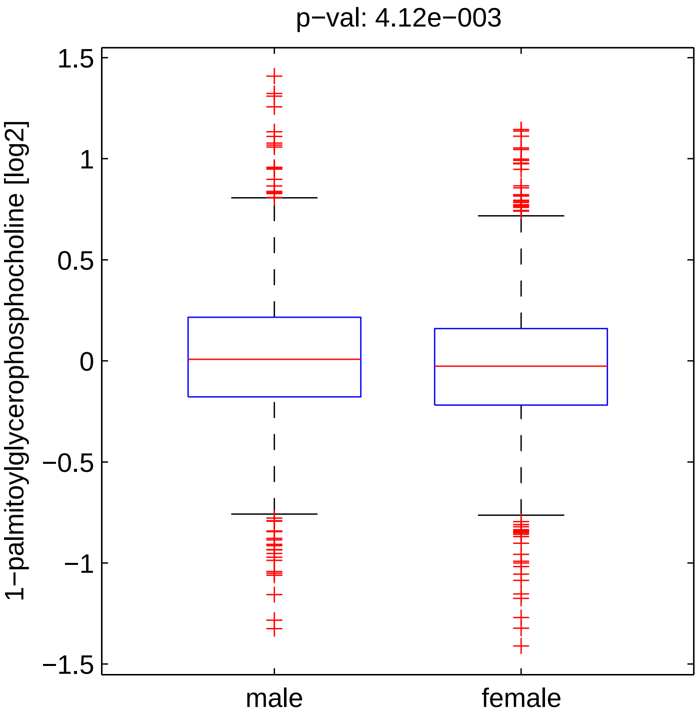

p−val: 4.12e−003
1−palmitoylglycerophosphocholine [log2]
1.5
1
0.5
0
−0.5
−1
−1.5
male
female

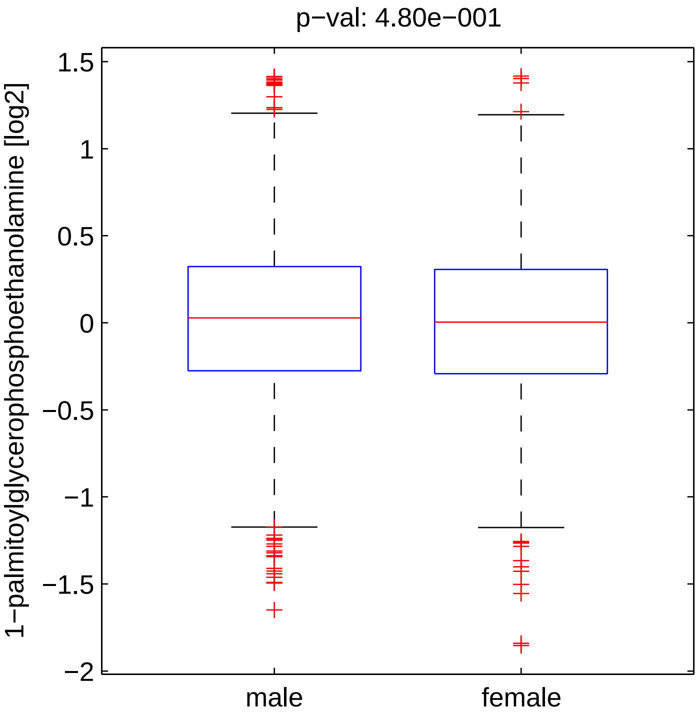
p−val: 4.80e−001
1−palmitoylglycerophosphoethanolamine [log2]
1.5
1
0.5
0
−0.5
−1
−1.5
−2
male
female

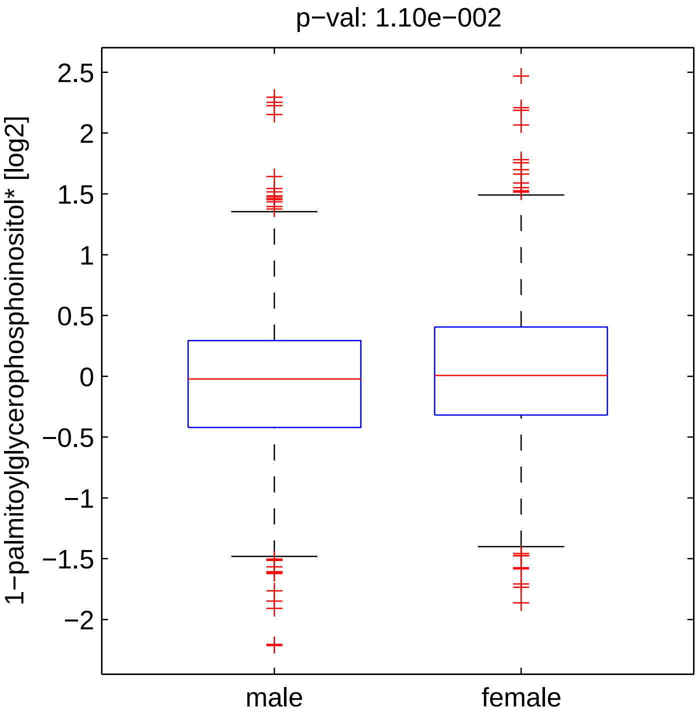

p−val: 1.10e−002
1−palmitoylglycerophosphoinositol* [log2]
2.5
2
1.5
1
0.5
0
−0.5
−1
−1.5
−2
male
female

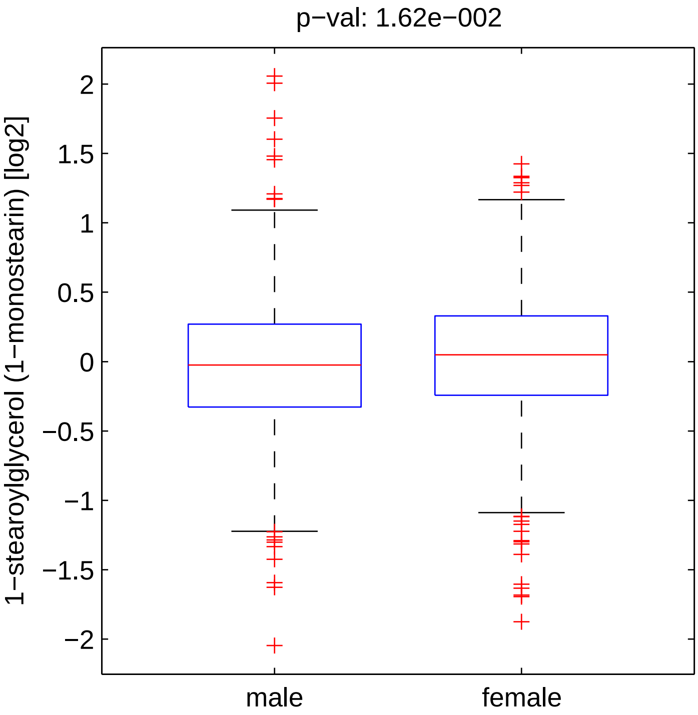

p−val: 1.62e−002
1−stearoylglycerol (1−monostearin) [log2]
2
1.5
1
0.5
0
−0.5
−1
−1.5
−2
male
female

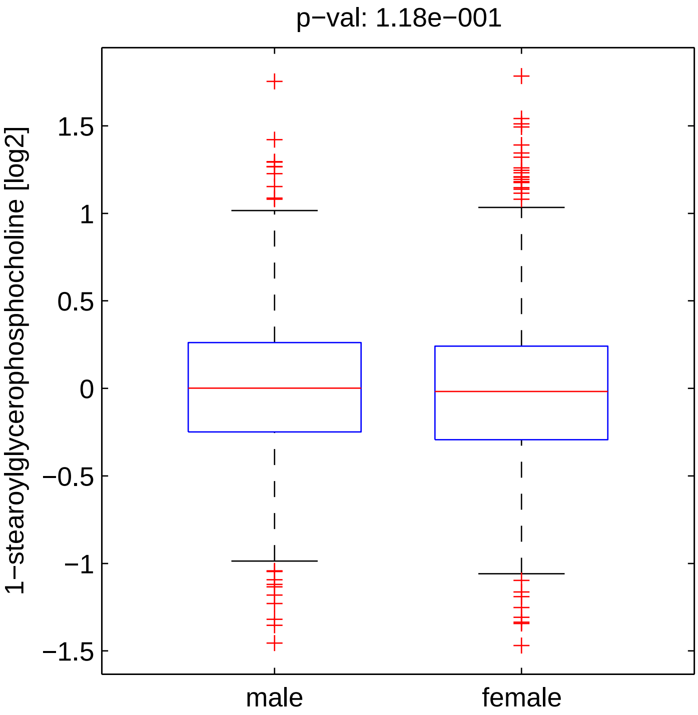
p−val: 1.18e−001
1−stearoylglycerophosphocholine [log2]
1.5
1
0.5
0
−0.5
−1
−1.5
male
female

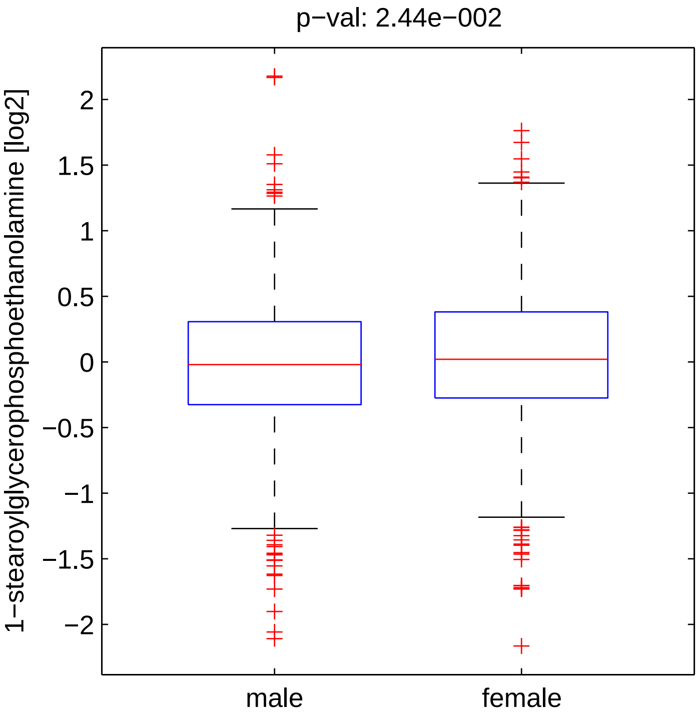
p−val: 2.44e−002
1−stearoylglycerophosphoethanolamine [log2]
2
1.5
1
0.5
0
−0.5
−1
−1.5
−2
male
female

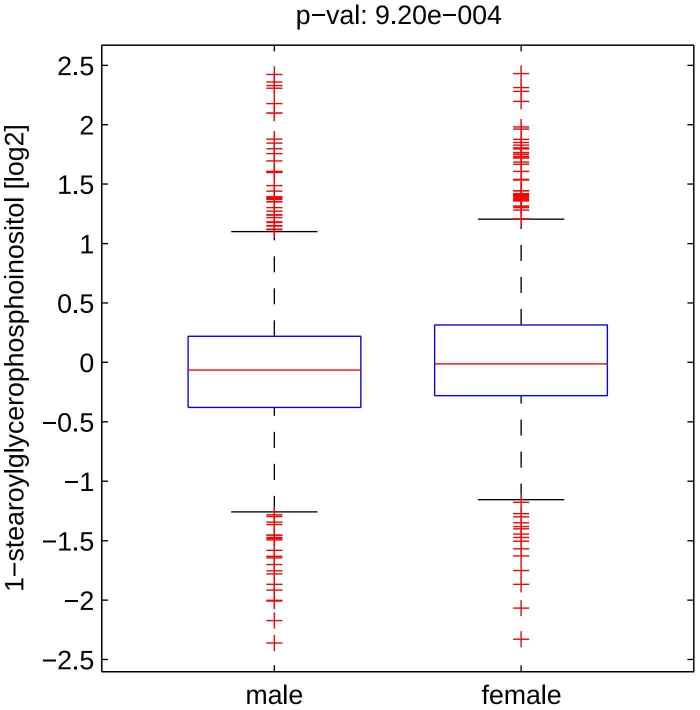

p−val: 9.20e−004
1−stearoylglycerophosphoinositol [log2]
2.5
2
1.5
1
0.5
0
−0.5
−1
−1.5
−2
−2.5
male
female

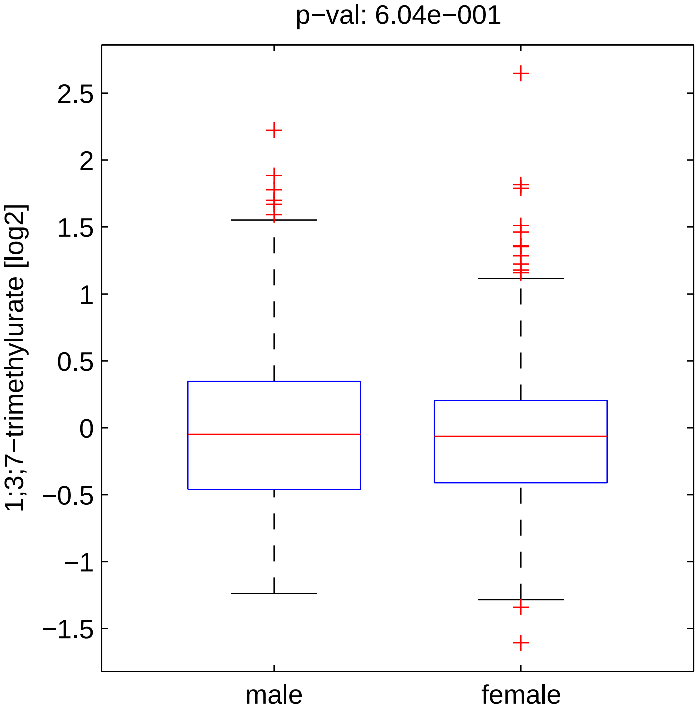

p−val: 6.04e−001
1;3;7−trimethylurate [log2]
2.5
2
1.5
1
0.5
0
−0.5
−1
−1.5
male
female

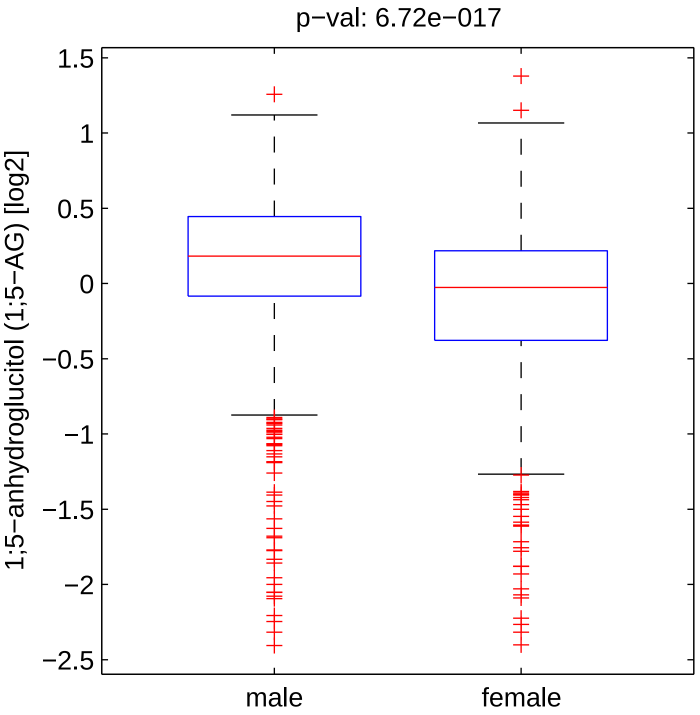
p−val: 6.72e−017
1:5−anhydroglucitol (1;5−AG) [log2]
1.5
1
0.5
0
−0.5
−1
−1.5
−2
−2.5
male
female

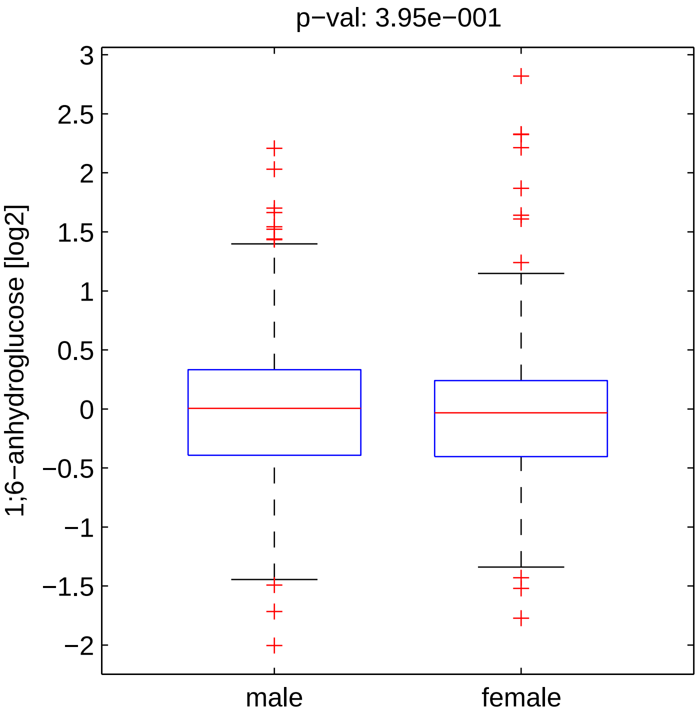

p−val: 3.95e−001
1;6−anhydroglucose [log2]
3
2.5
2
1.5
1
0.5
0
−0.5
−1
−1.5
−2
male
female

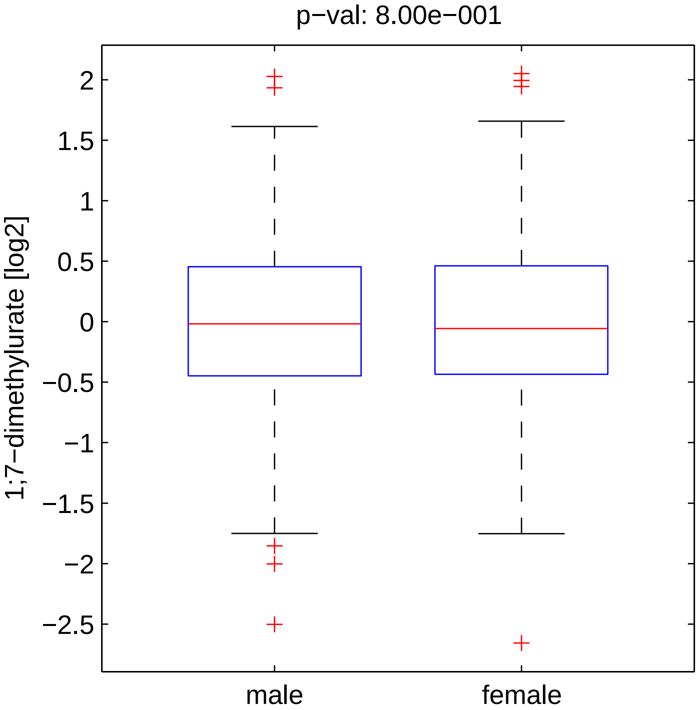

p−val: 8.00e−001
1;7−dimethylurate [log2]
2
1.5
1
0.5
0
−0.5
−1
−1.5
−2
−2.5
male
female

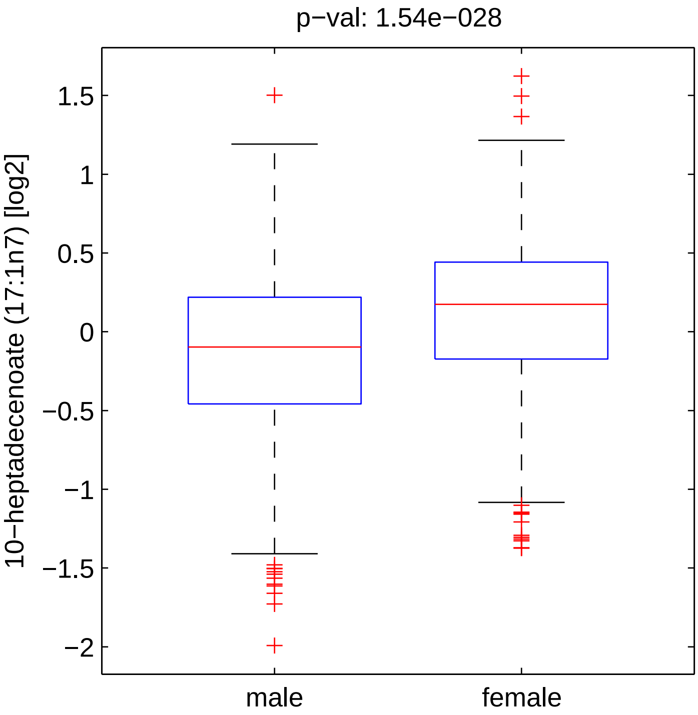

p−val: 1.54e−028
10−heptadecenoate (17:1n7) [log2]
1.5
1
0.5
0
−0.5
−1
−1.5
−2
male
female

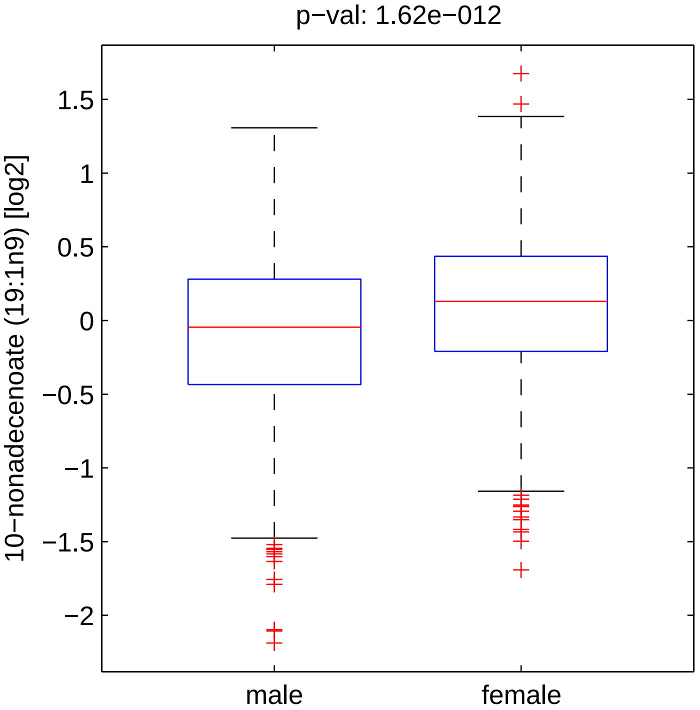
p−val: 1.62e−012
10−nonadecenoate (19:1n9) [log2]
1.5
1
0.5
0
−0.5
−1
−1.5
−2
male
female

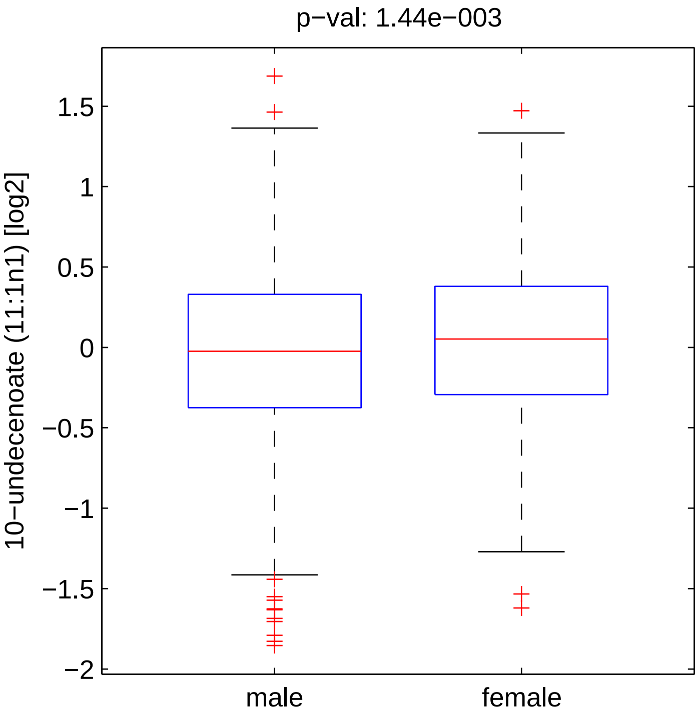
p−val: 1.44e−003
10−undecenoate (11:1n1) [log2]
1.5
1
0.5
0
−0.5
−1
−1.5
−2
male
female

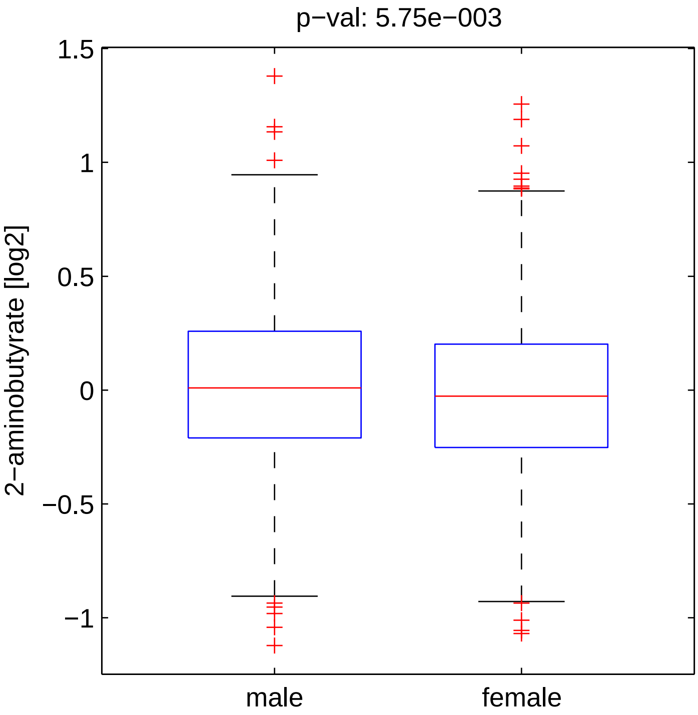
p−val: 5.75e−003
1.5
1
0.5
0
−0.5
−1
2−aminobutyrate [log2]
male
female

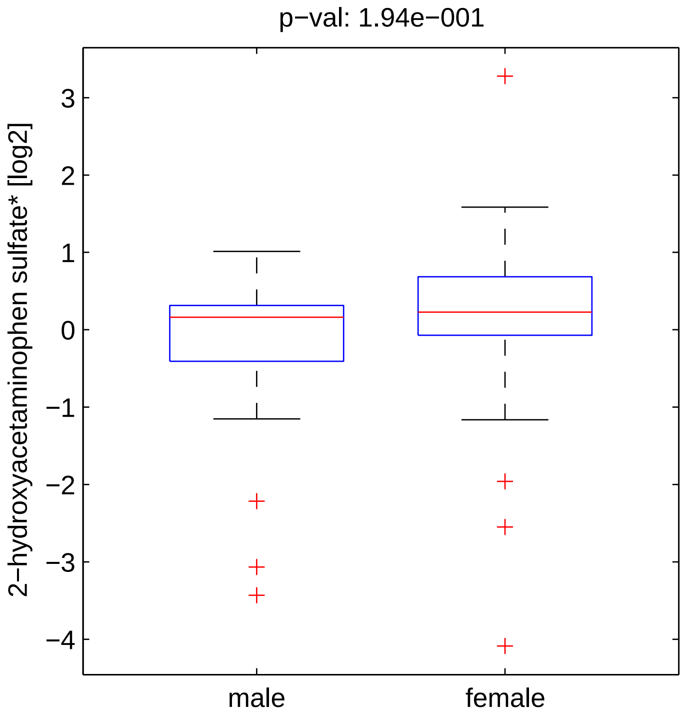

p−val: 1.94e−001
2−hydroxyacetaminophen sulfate* [log2]
3
2
1
0
−1
−2
−3
−4
male
female

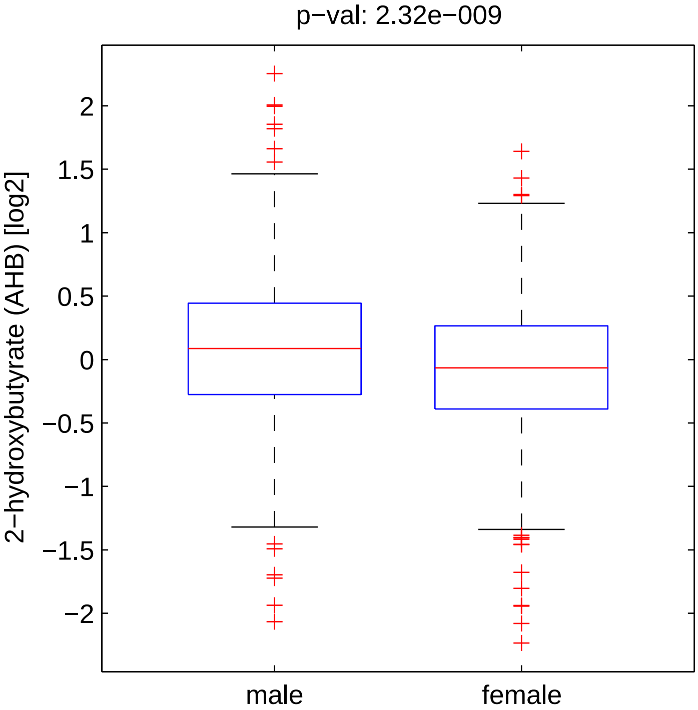
p−val: 2.32e−009
2−hydroxybutyrate (AHB) [log2]
2
1.5
1
0.5
0
−0.5
−1
−1.5
−2
male
female

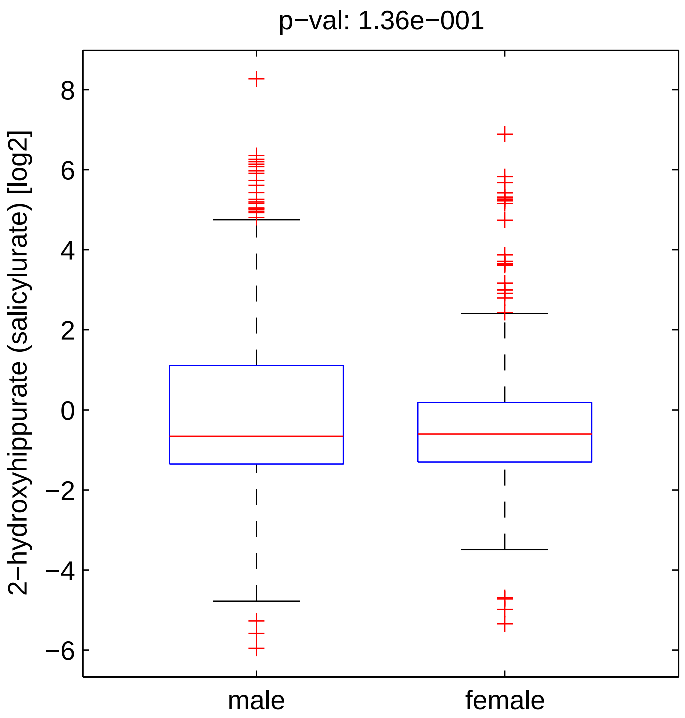
p−val: 1.36e−001
2−hydroxyhippurate (salicylurate) [log2]
8
6
4
2
0
−2
−4
−6
male
female

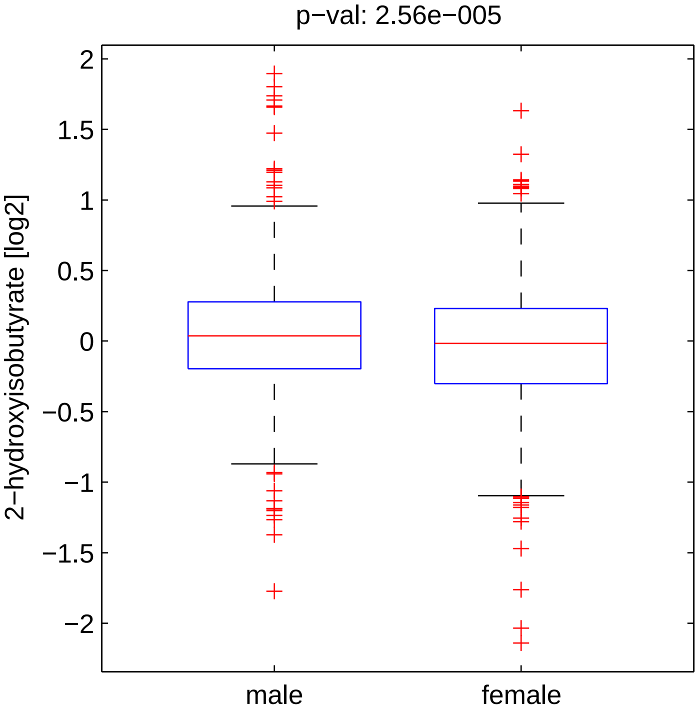

p−val: 2.56e−005
2−hydroxyisobutyrate [log2]
2
1.5
1
0.5
0
−0.5
−1
−1.5
−2
male
female

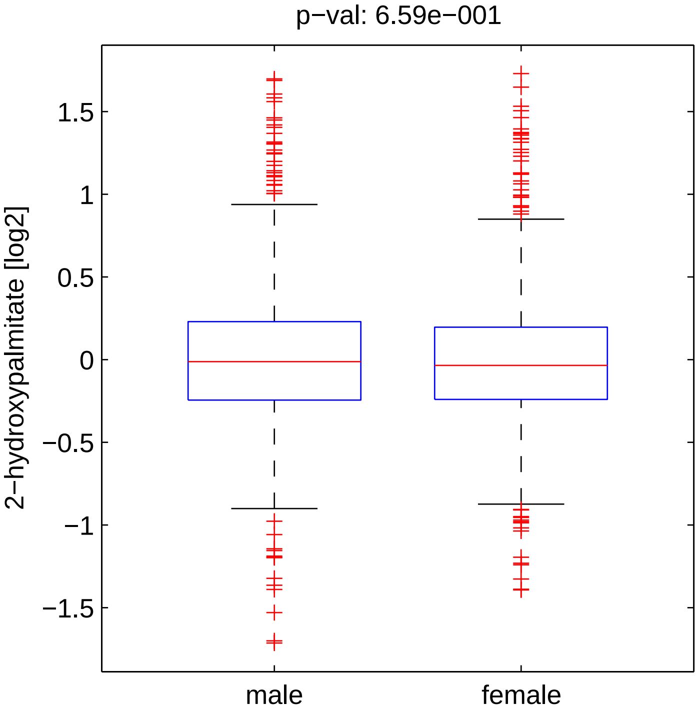
p−val: 6.59e−001
2−hydroxypalmitate [log2]
1.5
1
0.5
0
−0.5
−1
−1.5
male
female

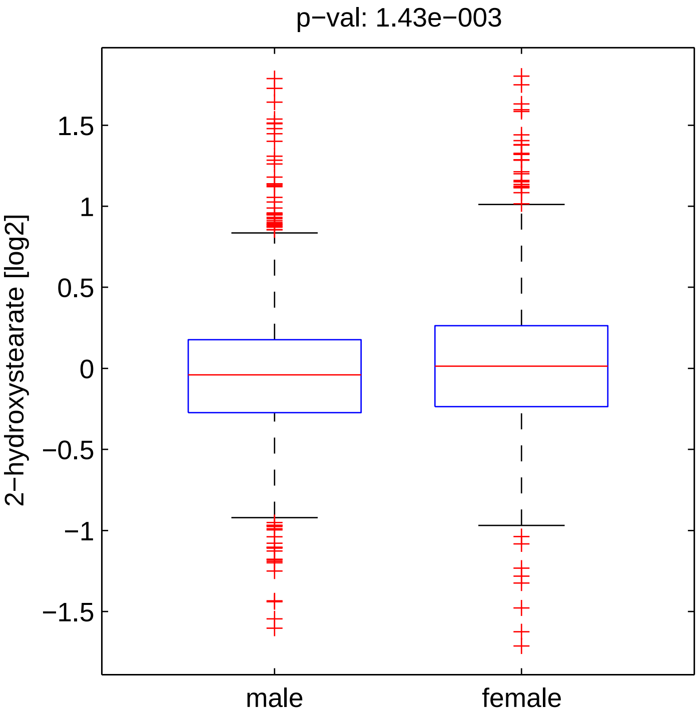

p−val: 1.43e−003
2−hydroxystearate [log2]
1.5
1
0.5
0
−0.5
−1
−1.5
male
female

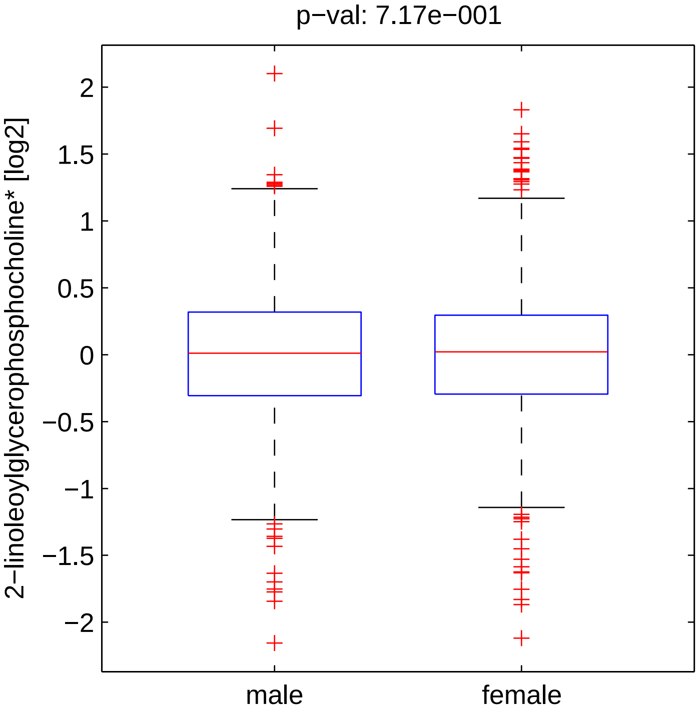
p−val: 7.17e−001
2−linoleoylglycerophosphocholine* [log2]
2
1.5
1
0.5
0
−0.5
−1
−1.5
−2
male
female

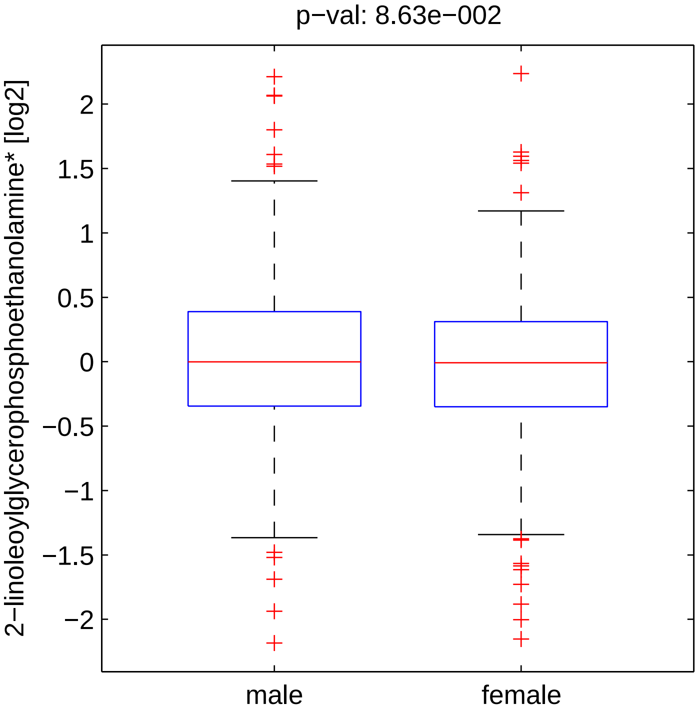
p−val: 8.63e−002
2−linoleoylglycerophosphoethanolamine* [log2]
2
1.5
1
0.5
0
−0.5
−1
−1.5
−2
male
female

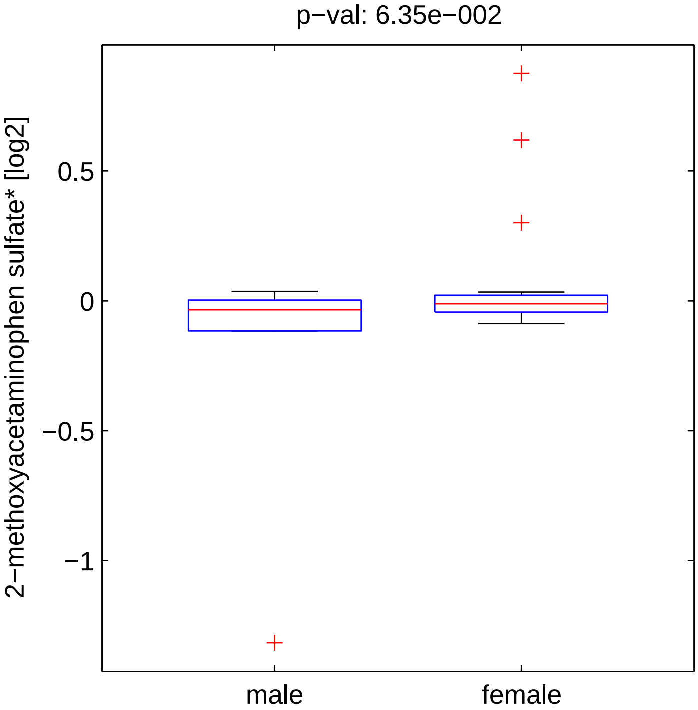
p−val: 6.35e−002
2−methoxyacetaminophen sulfate* [log2]
0.5
0
−0.5
−1
male
female

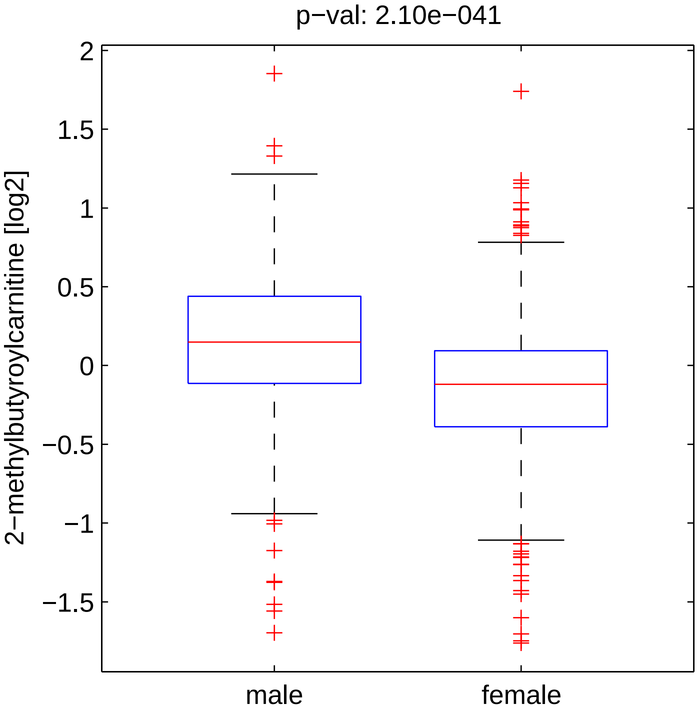

p−val: 2.10e−041
2
1.5
1
0.5
0
−0.5
−1
−1.5
2−methylbutyroylcarnitine [log2]
male
female

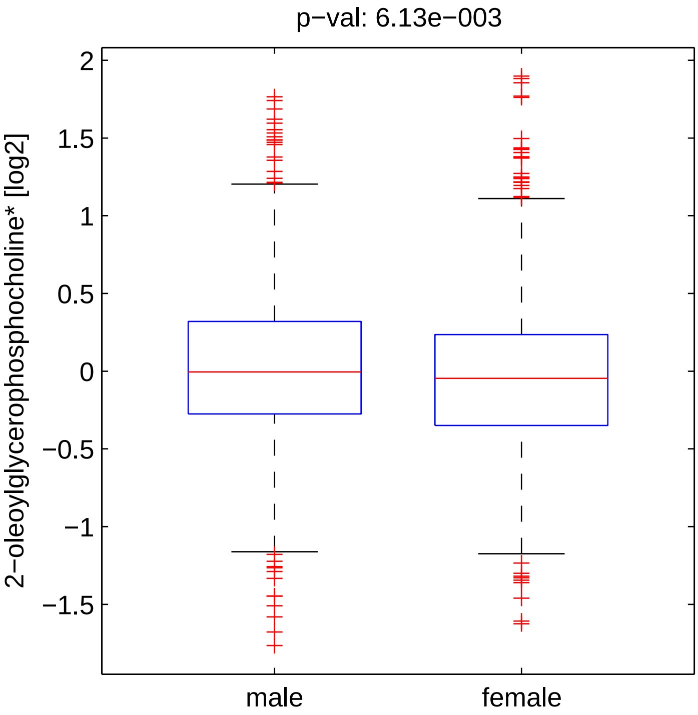

p−val: 6.13e−003
2−oleoylglycerophosphocholine* [log2]
2
1.5
1
0.5
0
−0.5
−1
−1.5
male
female

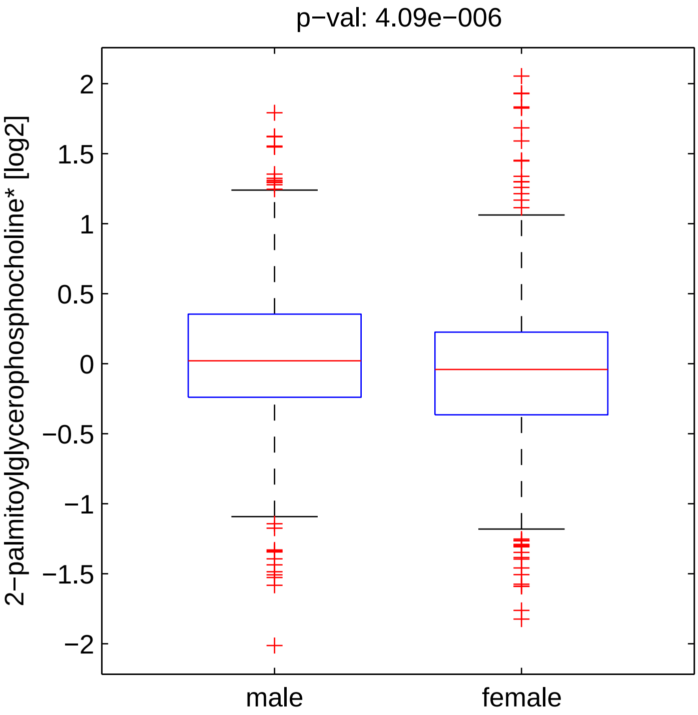
p−val: 4.09e−006
2−palmitoylglycerophosphocholine* [log2]
2
1.5
1
0.5
0
−0.5
−1
−1.5
−2
male
female

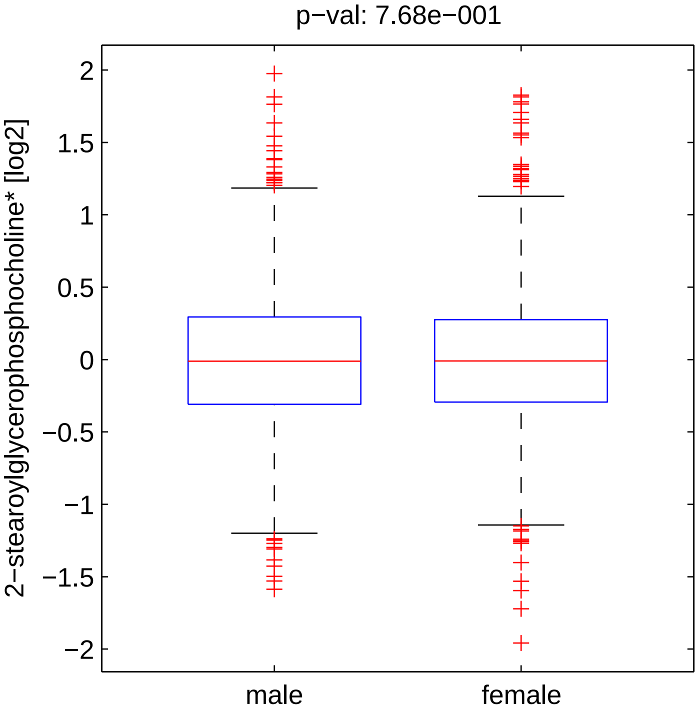
p−val: 7.68e−001
2−stearoylglycerophosphocholine* [log2]
2
1.5
1
0.5
0
−0.5
−1
−1.5
−2
male
female

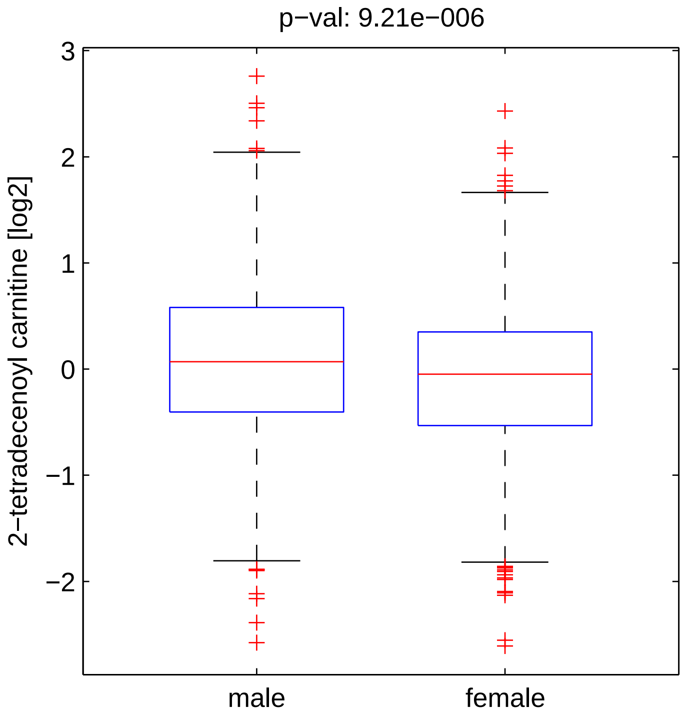
p−val: 9.21e−006
2−tetradecenoyl carnitine [log2]
3
2
1
0
−1
−2
male
female

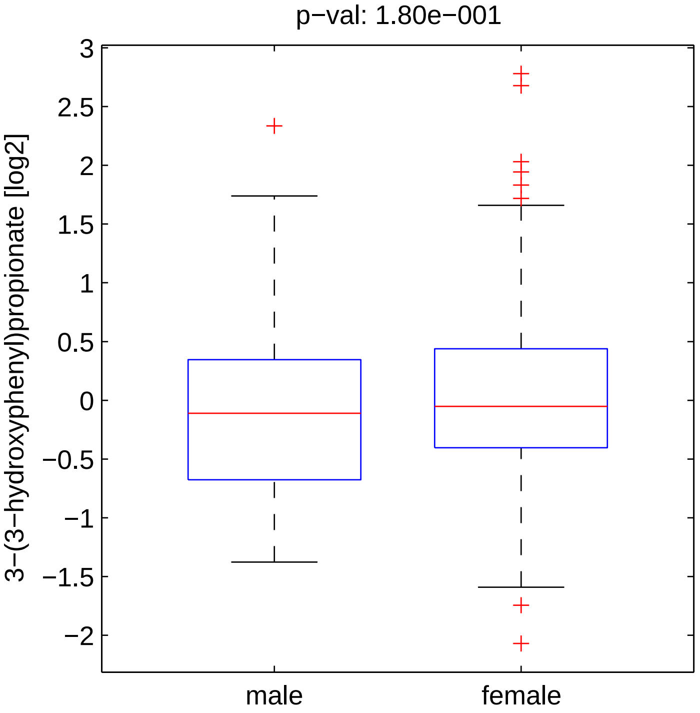
p−val: 1.80e−001
3−(3−hydroxyphenyl)propionate [log2]
3
2.5
2
1.5
1
0.5
0
−0.5
−1
−1.5
−2
male
female

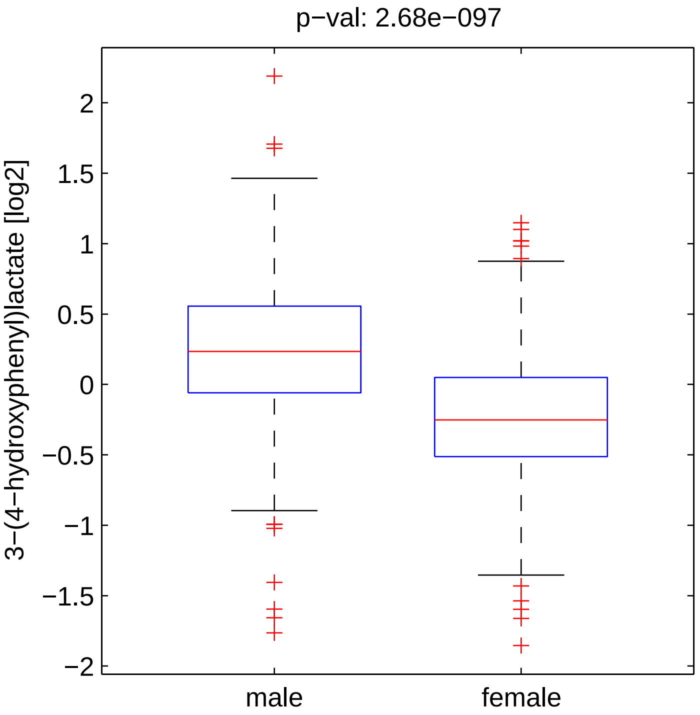
p−val: 2.68e−097
3−(4−hydroxyphenyl)lactate [log2]
2
1.5
1
0.5
0
−0.5
−1
−1.5
−2
male
female

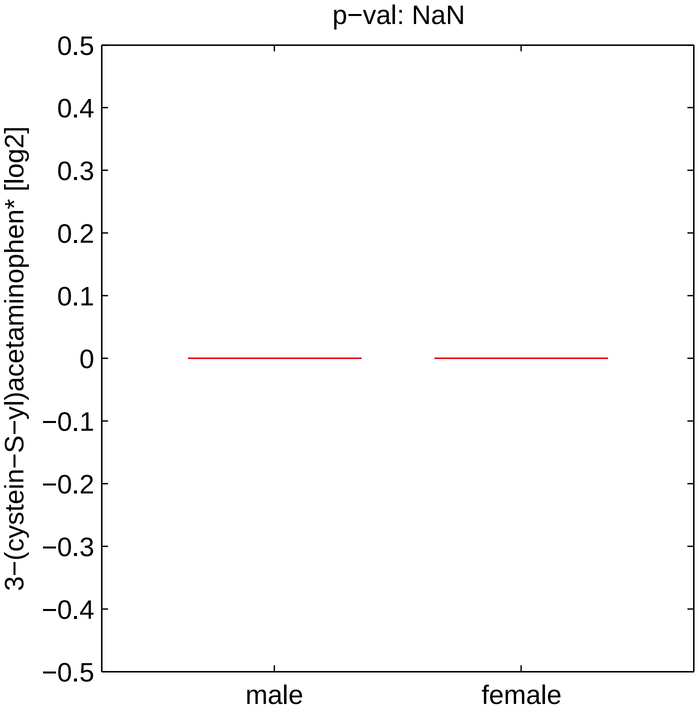
p−val: NaN
3−(cystein−S−yl)acetaminophen* [log2]
0.5
0.4
0.3
0.2
0.1
0
−0.1
−0.2
−0.3
−0.4
−0.5
male
female

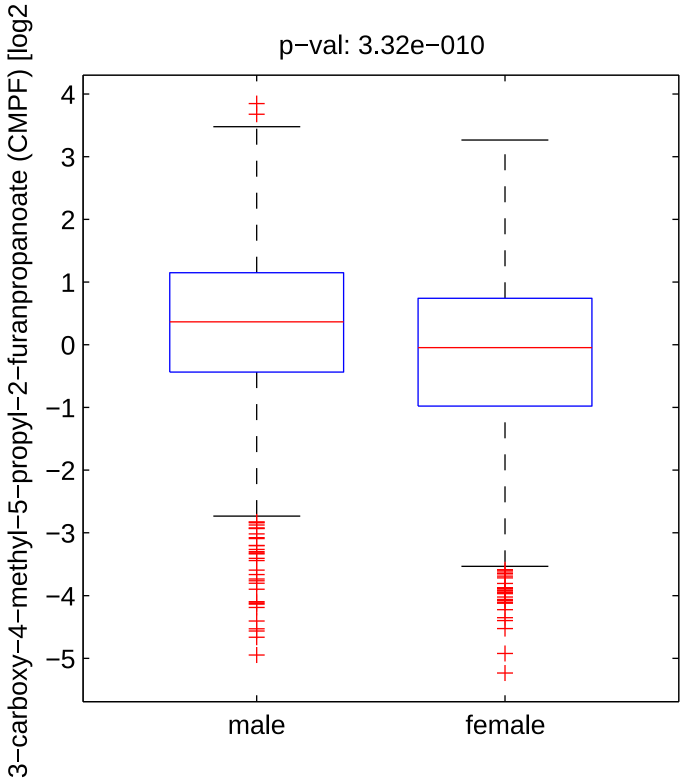
p−val: 3.32e−010
3−carboxy−4−methyl−5−propyl−2−furanpropanoate (CMPF) [log2
4
3
2
1
0
−1
−2
−3
−4
−5
male
female

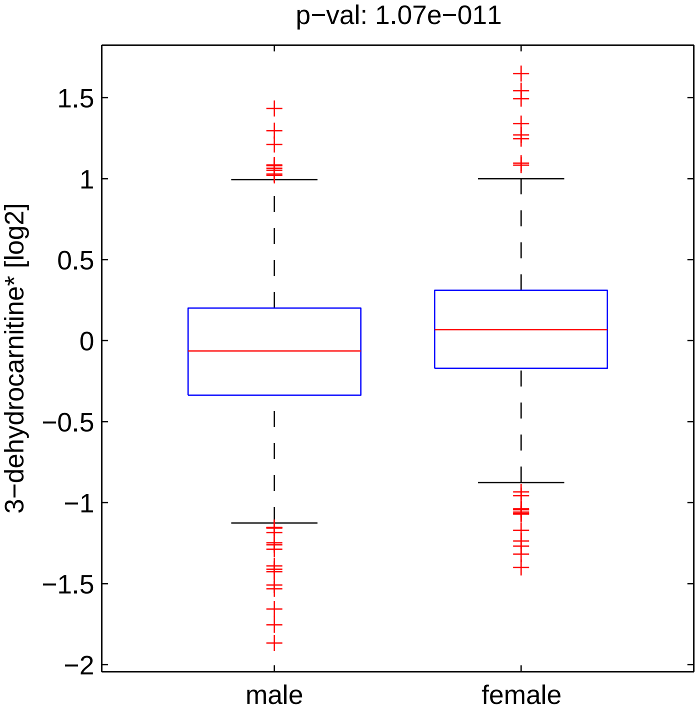
p−val: 1.07e−011
3−dehydrocarnitine* [log2]
1.5
1
0.5
0
−0.5
−1
−1.5
−2
male
female

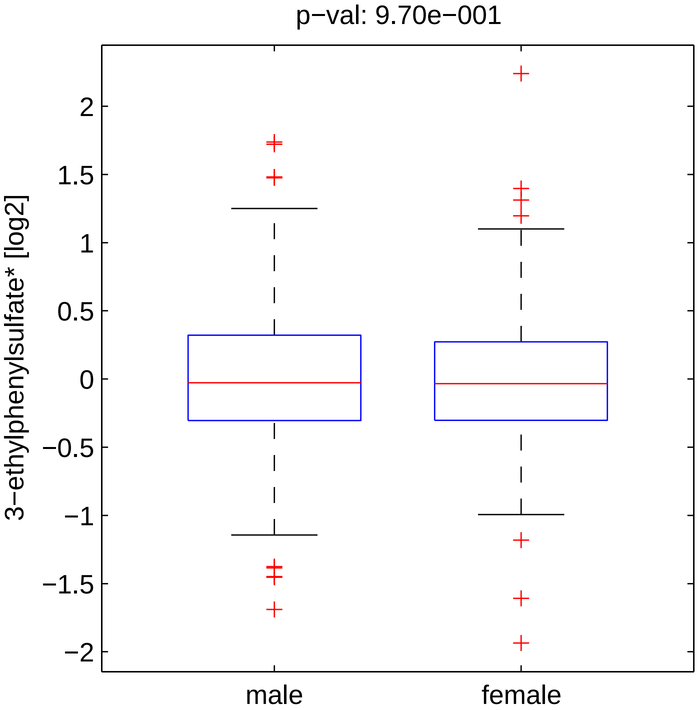
p−val: 9.70e−001
3−ethylphenylsulfate* [log2]
2
1.5
1
0.5
0
−0.5
−1
−1.5
−2
male
female

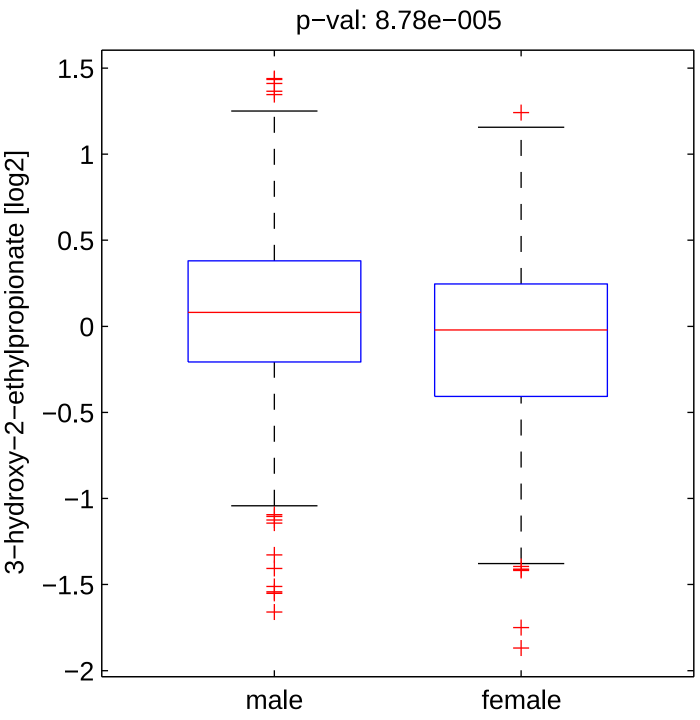
p−val: 8.78e−005
3−hydroxy−2−ethylpropionate [log2]
1.5
1
0.5
0
−0.5
−1
−1.5
−2
male
female

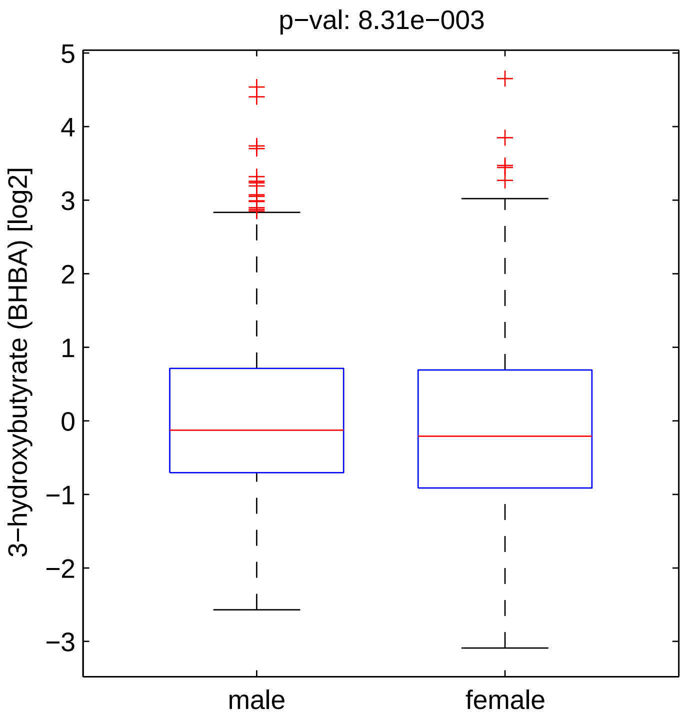
p−val: 8.31e−003
3−hydroxybutyrate (BHBA) [log2]
5
4
3
2
1
0
−1
−2
−3
male
female

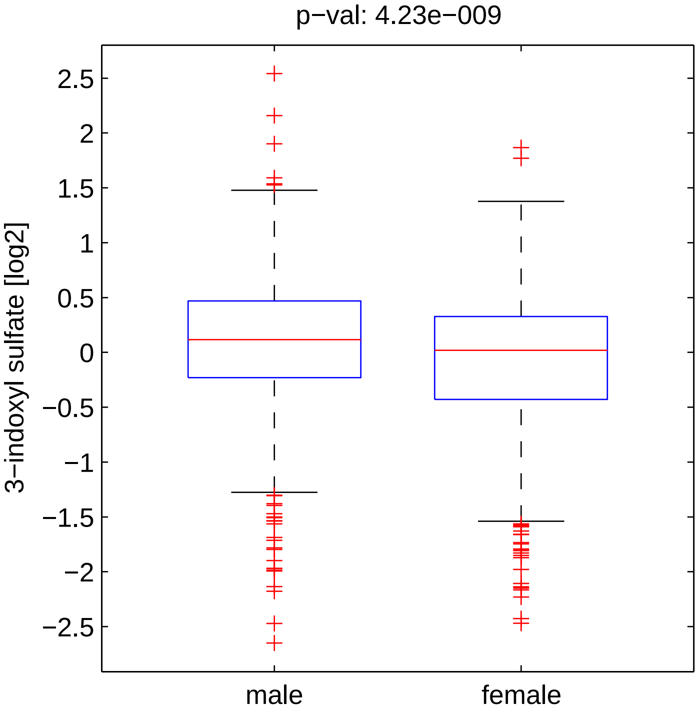
p−val: 4.23e−009
3−indoxyl sulfate [log2]
2.5
2
1.5
1
0.5
0
−0.5
−1
−1.5
−2
−2.5
male
female

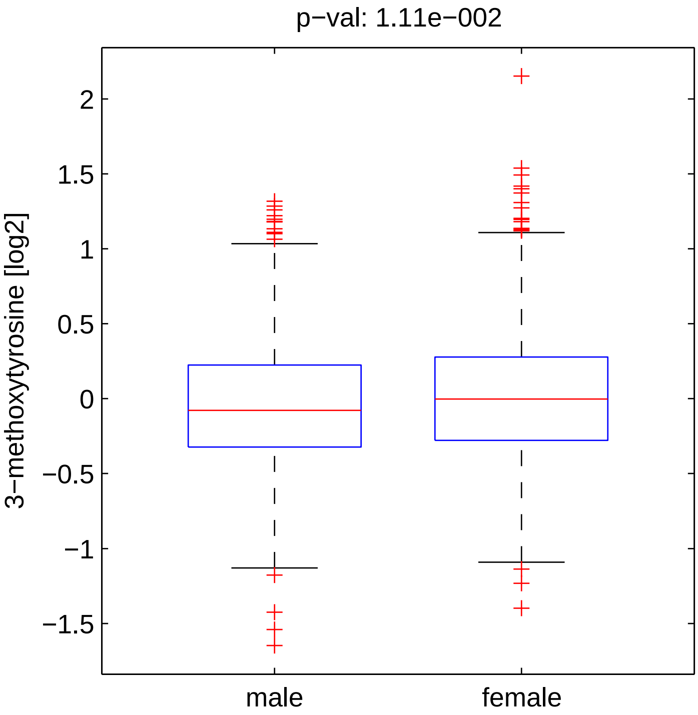

p−val: 1.11e−002
3−methoxytyrosine [log2]
2
1.5
1
0.5
0
−0.5
−1
−1.5
male
female

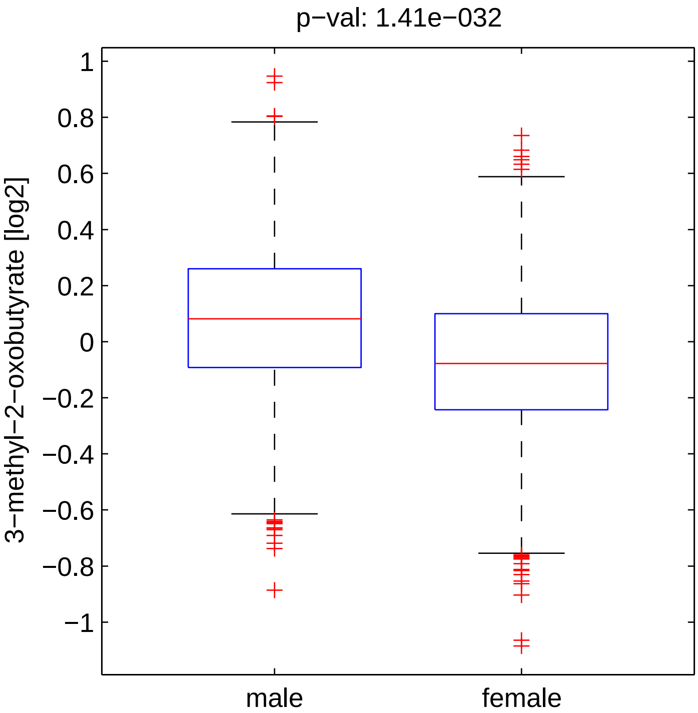
p−val: 1.41e−032
3−methyl−2−oxobutyrate [log2]
1
0.8
0.6
0.4
0.2
0
−0.2
−0.4
−0.6
−0.8
−1
male
female

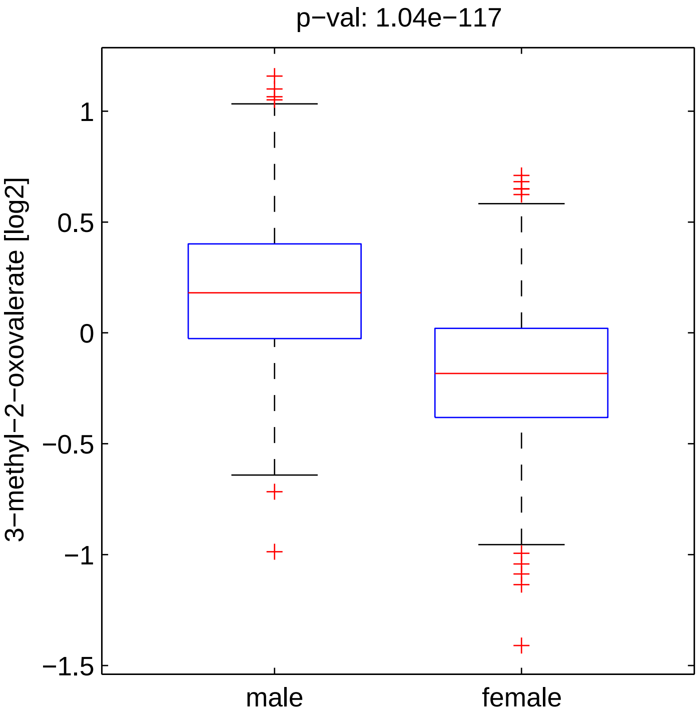
p−val: 1.04e−117
3−methyl−2−oxovalerate [log2]
1
0.5
0
−0.5
−1
−1.5
male
female

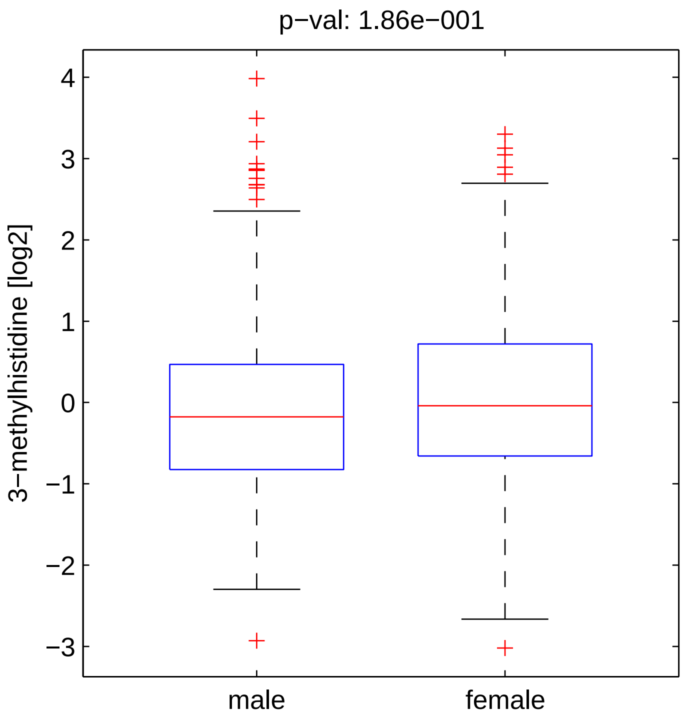
p−val: 1.86e−001
3−methylhistidine [log2]
4
3
2
1
0
−1
−2
−3
male
female

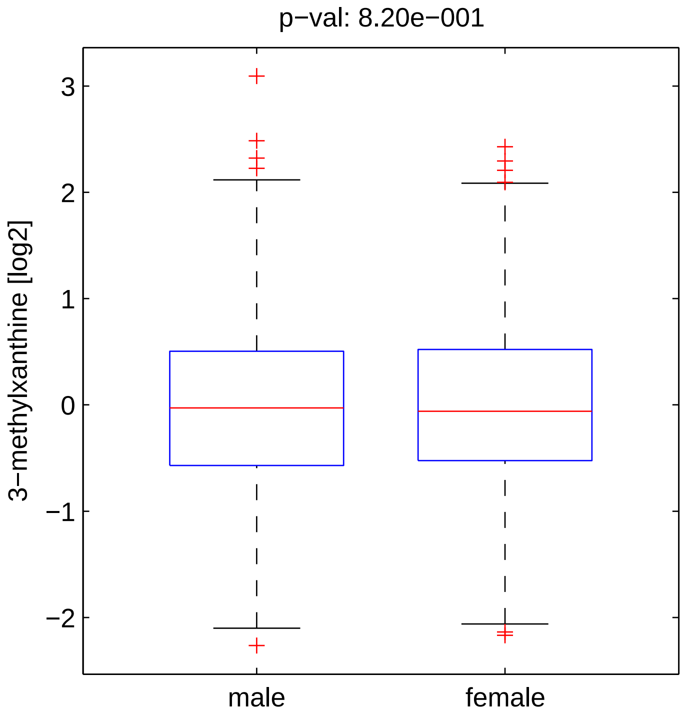
p−val: 8.20e−001
3−methylxanthine [log2]
3
2
1
0
−1
−2
male
female

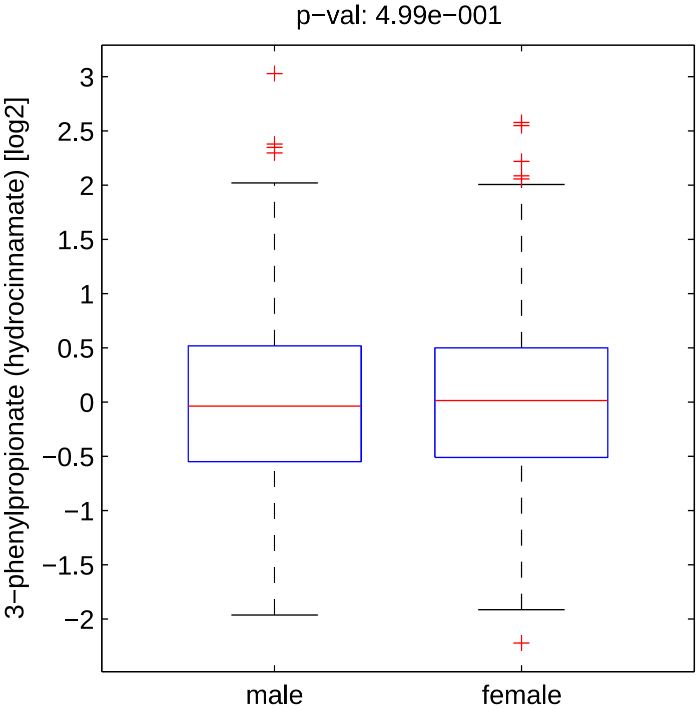
p−val: 4.99e−001
3−phenylpropionate (hydrocinnamate) [log2]
3
2.5
2
1.5
1
0.5
0
−0.5
−1
−1.5
−2
male
female

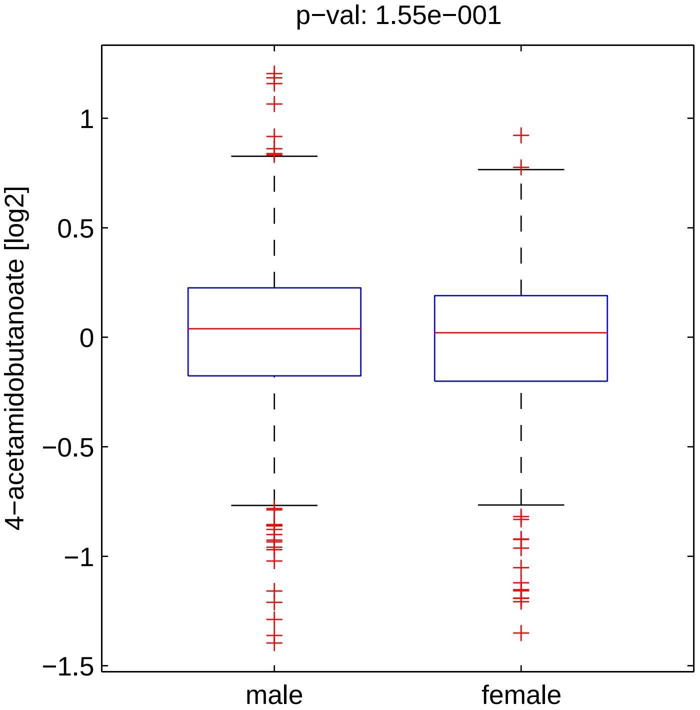
p−val: 1.55e−001
4−acetamidobutanoate [log2]
1
0.5
0
−0.5
−1
−1.5
male
female

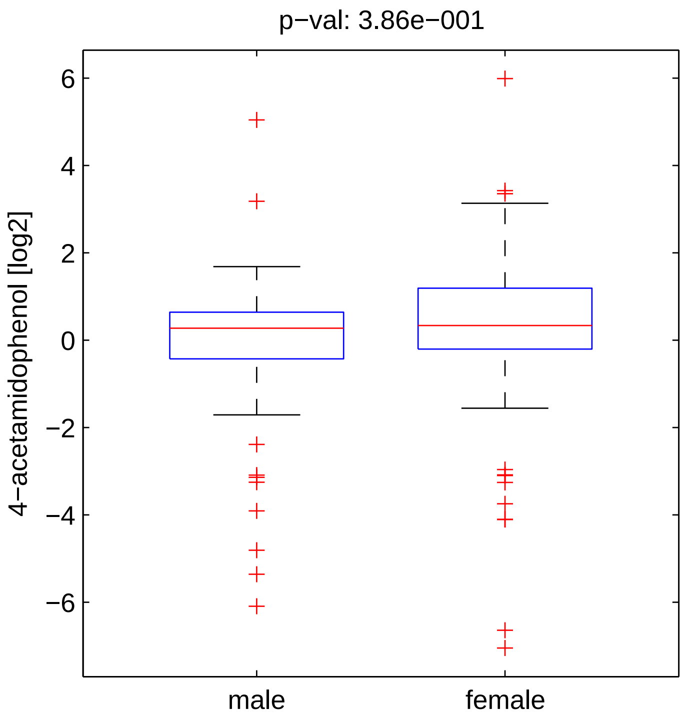
p−val: 3.86e−001
4−acetamidophenol [log2]
6
4
2
0
−2
−4
−6
male
female

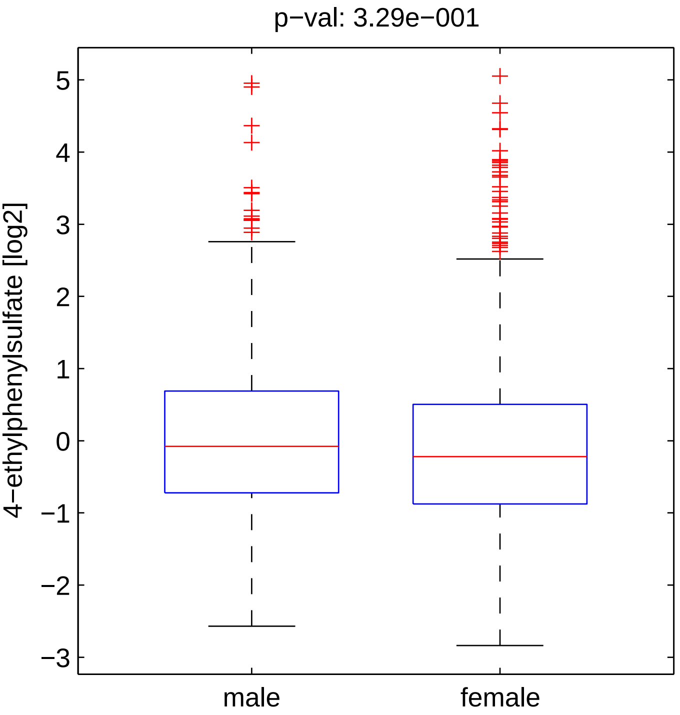

p−val: 3.29e−001
4−ethylphenylsulfate [log2]
5
4
3
2
1
0
−1
−2
−3
male
female

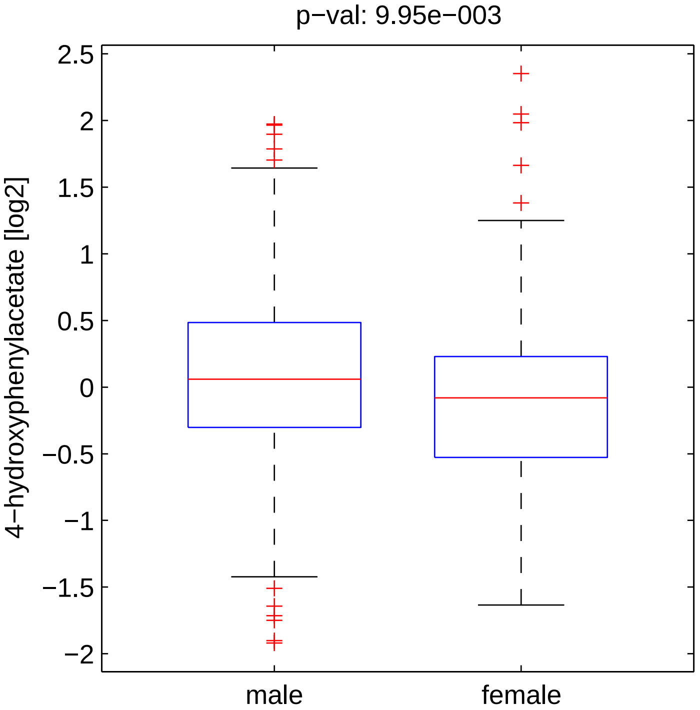
p−val: 9.95e−003
4−hydroxyphenylacetate [log2]
2.5
2
1.5
1
0.5
0
−0.5
−1
−1.5
−2
male
female

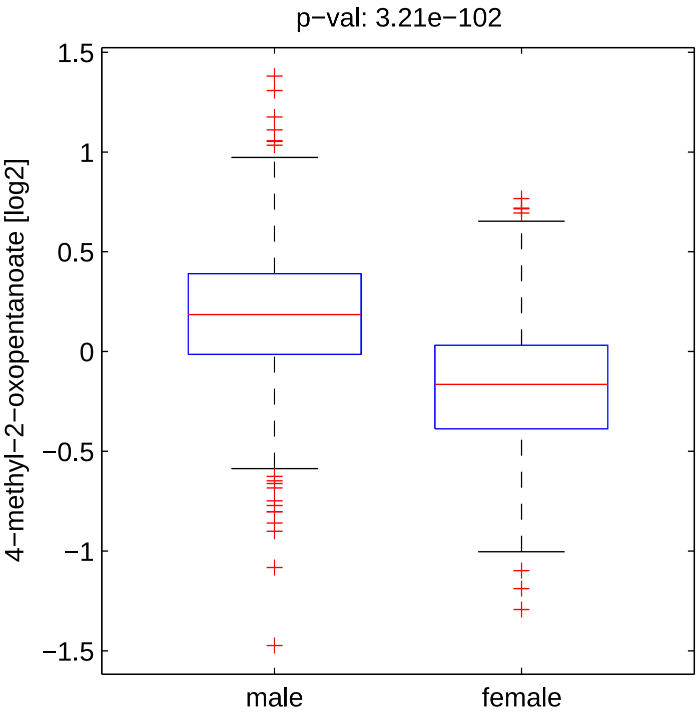
p−val: 3.21e−102
4−methyl−2−oxopentanoate [log2]
1.5
1
0.5
0
−0.5
−1
−1.5
male
female

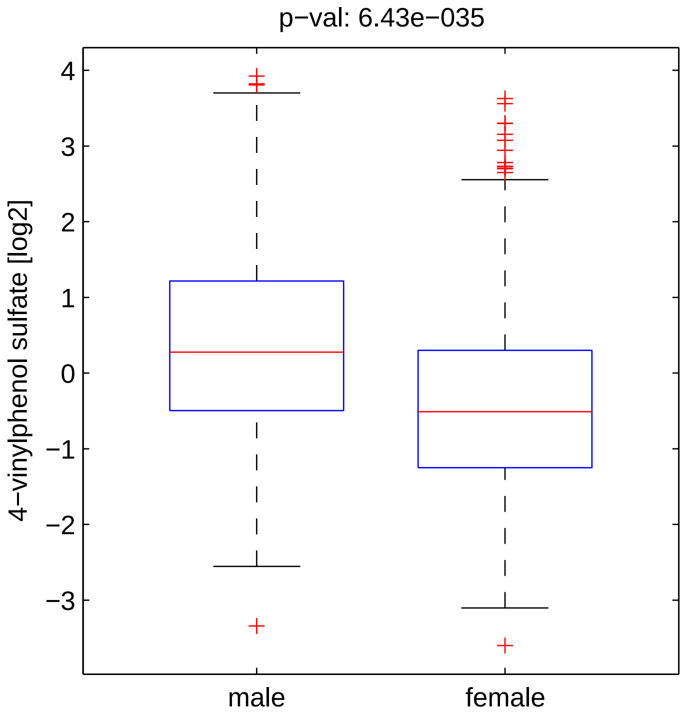
p−val: 6.43e−035
4−vinylphenol sulfate [log2]
4
3
2
1
0
−1
−2
−3
male
female

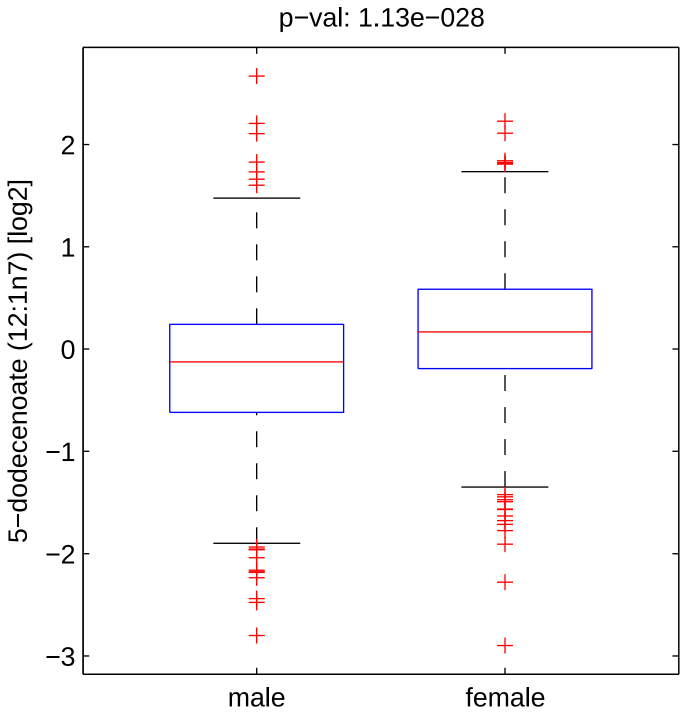
p−val: 1.13e−028
5−dodecenoate (12:1n7) [log2]
2
1
0
−1
−2
−3
male
female

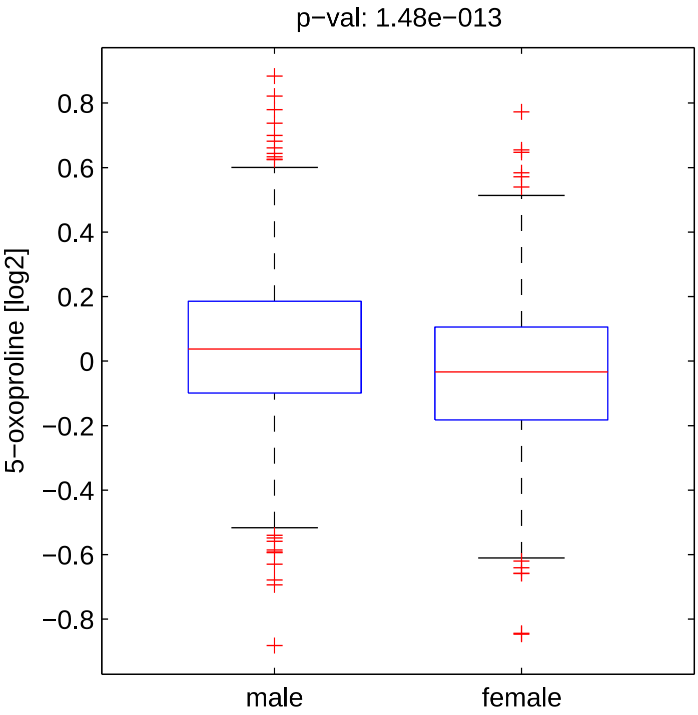
p−val: 1.48e−013
5−oxoproline [log2]
0.8
0.6
0.4
0.2
0
−0.2
−0.4
−0.6
−0.8
male
female

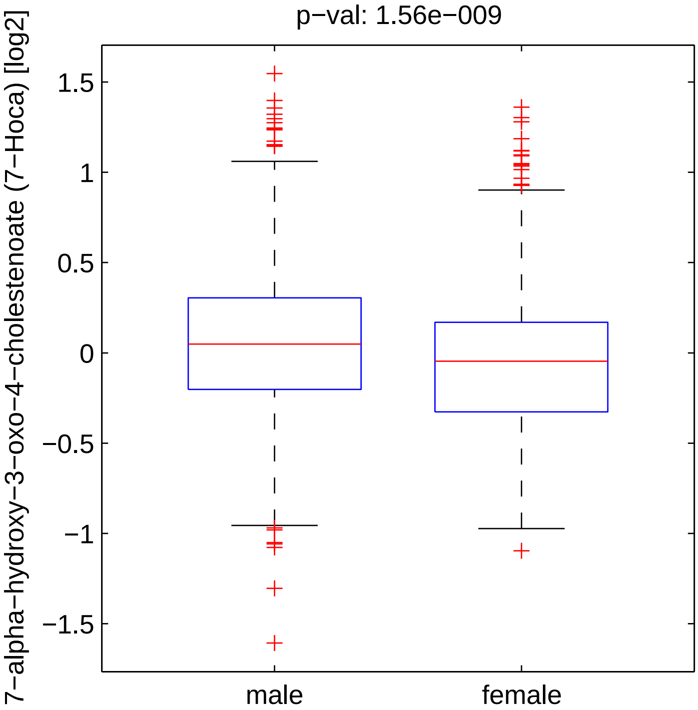
p−val: 1.56e−009
7−alpha−hydroxy−3−oxo−4−cholestenoate (7−Hoca) [log2]
1.5
1
0.5
0
−0.5
−1
−1.5
male
female

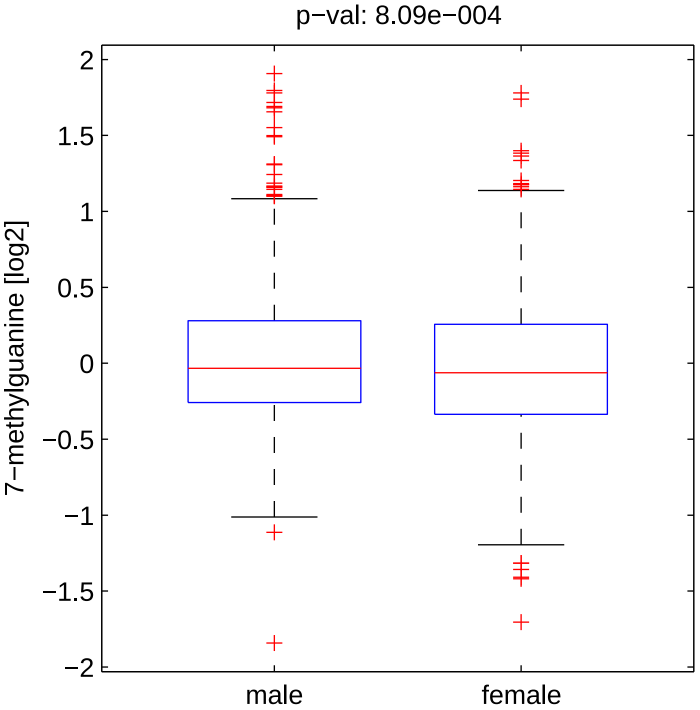
p−val: 8.09e−004
7−methylguanine [log2]
2
1.5
1
0.5
0
−0.5
−1
−1.5
−2
male
female

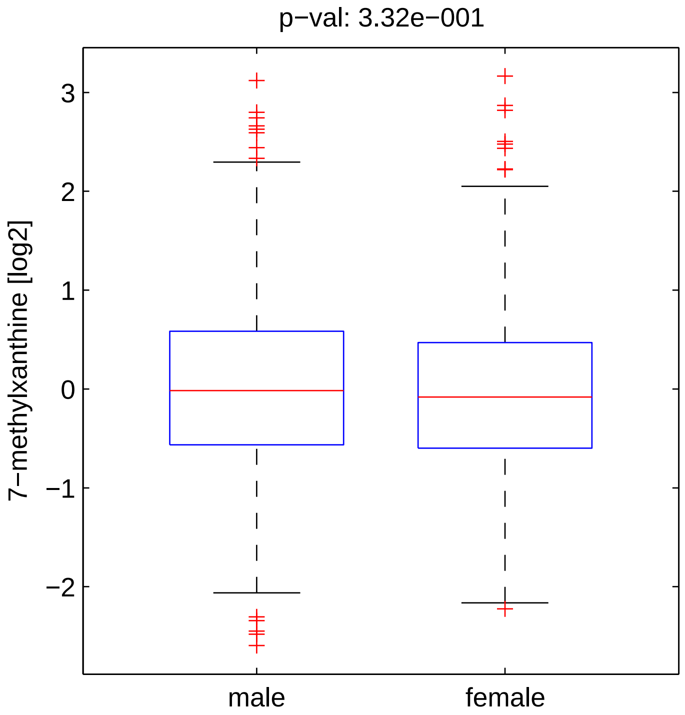
p−val: 3.32e−001
7−methylxanthine [log2]
3
2
1
0
−1
−2
male
female

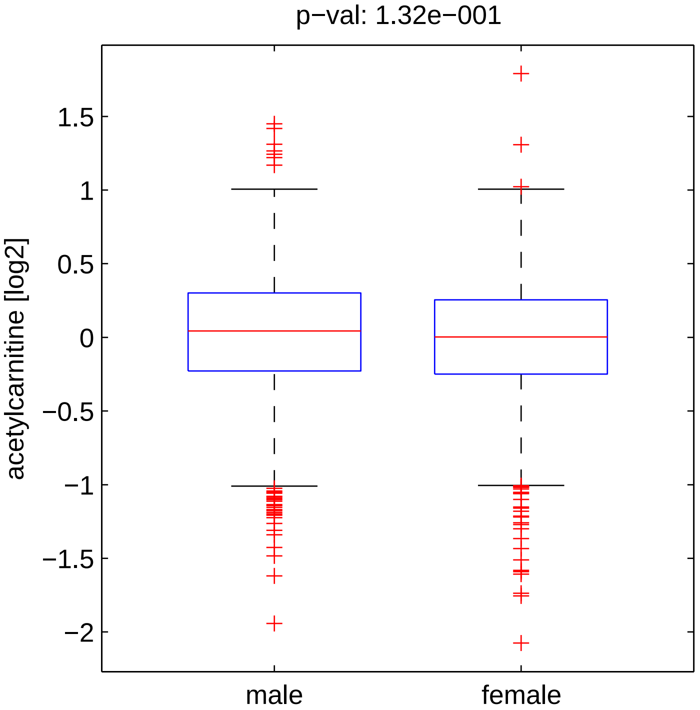
p−val: 1.32e−001
acetylcarnitine [log2]
1.5
1
0.5
0
−0.5
−1
−1.5
−2
male
female

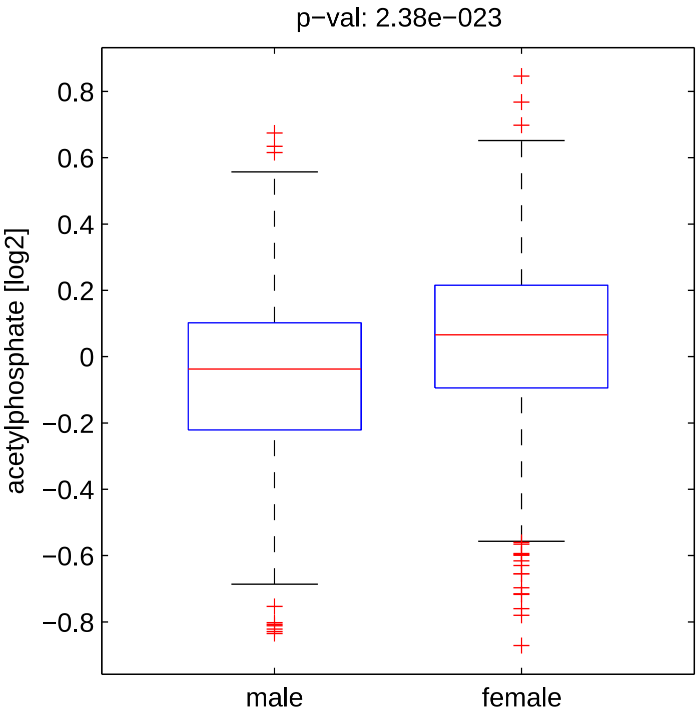
p−val: 2.38e−023
acetylphosphate [log2]
0.8
0.6
0.4
0.2
0
−0.2
−0.4
−0.6
−0.8
male
female

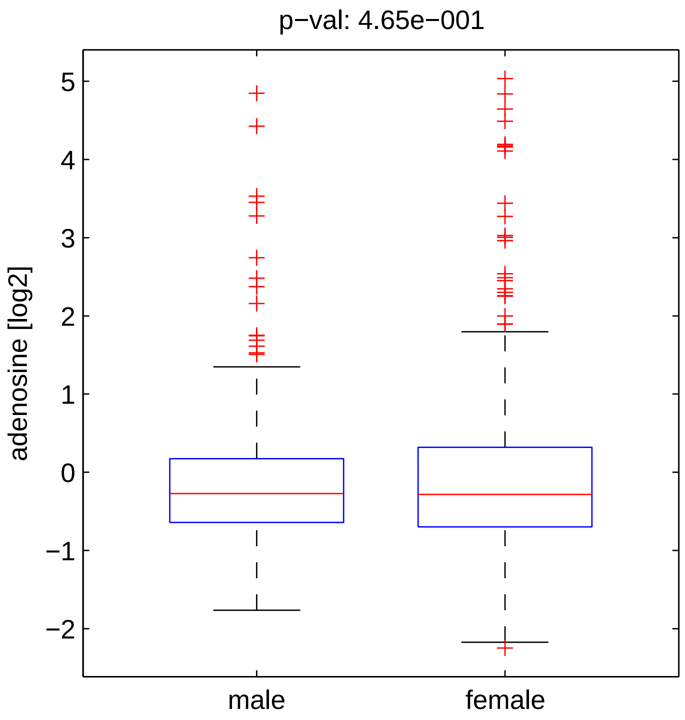
p−val: 4.65e−001
adenosine [log2]
5
4
3
2
1
0
−1
−2
male
female

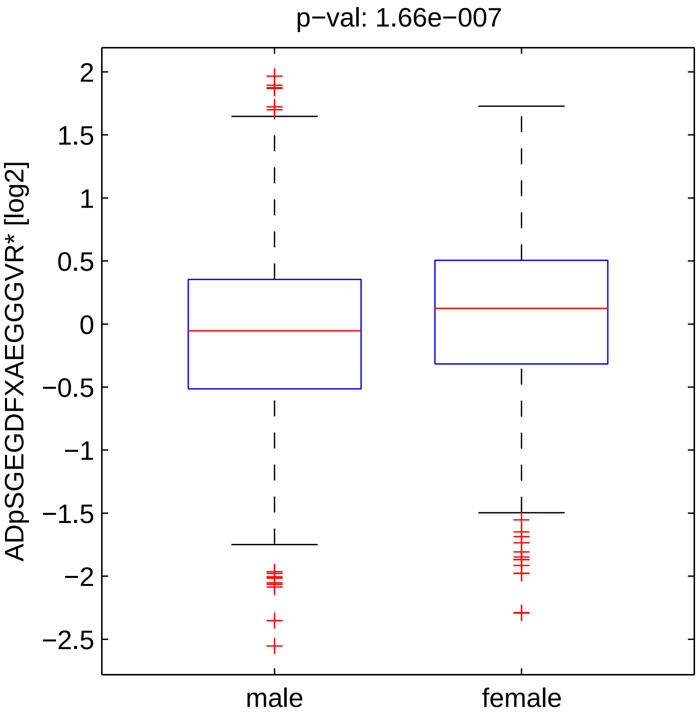
p−val: 1.66e−007
ADpSGEGDFXAEGGGVR* [log2]
2
1.5
1
0.5
0
−0.5
−1
−1.5
−2
−2.5
male
female

p−val: 8.74e−001

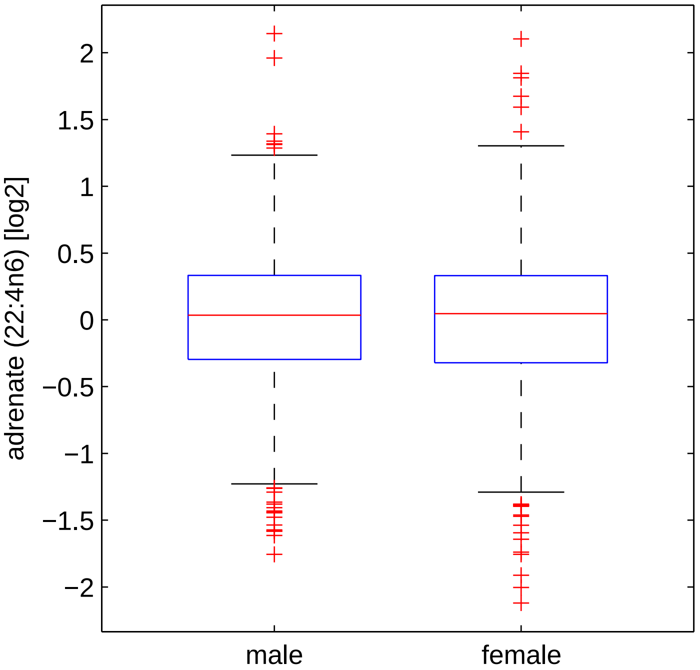

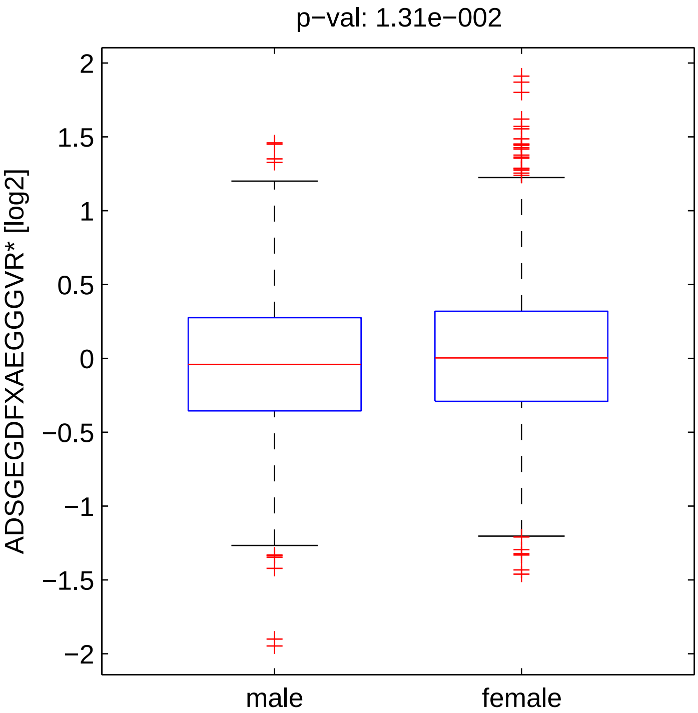

p−val: 1.31e−002
ADSGEGDFXAEGGGVR* [log2]
2
1.5
1
0.5
0
−0.5
−1
−1.5
−2
male
female

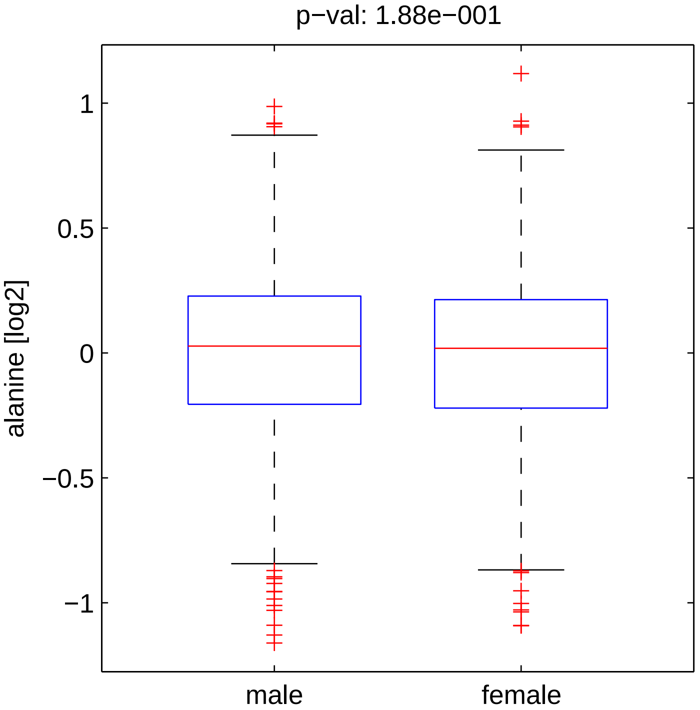
p−val: 1.88e−001
alanine [log2]
1
0.5
0
−0.5
−1
male
female

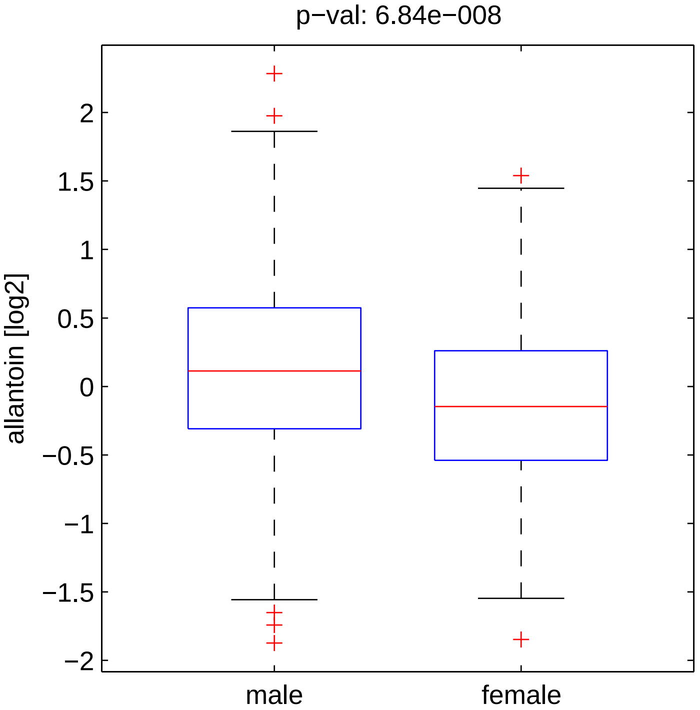
p−val: 6.84e−008
allantoin [log2]
2
1.5
1
0.5
0
−0.5
−1
−1.5
−2
male
female

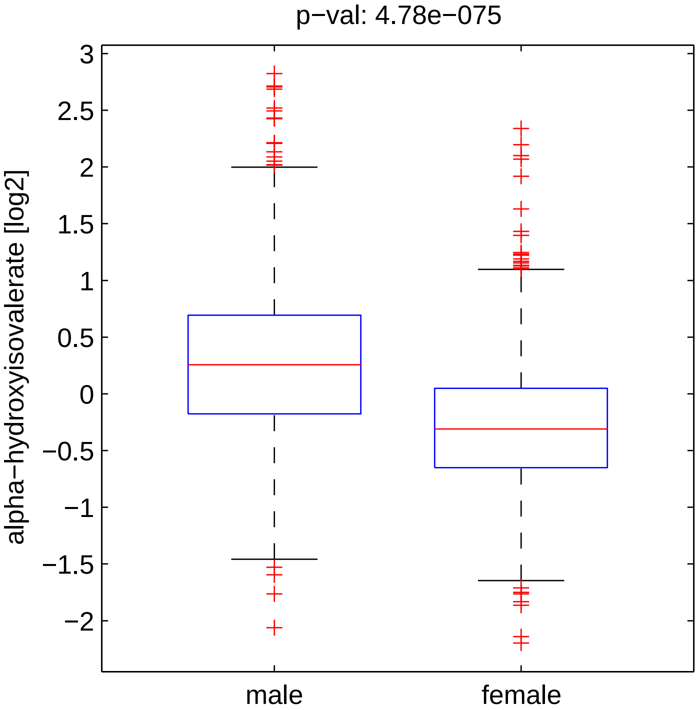

p−val: 4.78e−075
alpha−hydroxyisovalerate [log2]
3
2.5
2
1.5
1
0.5
0
−0.5
−1
−1.5
−2
male
female

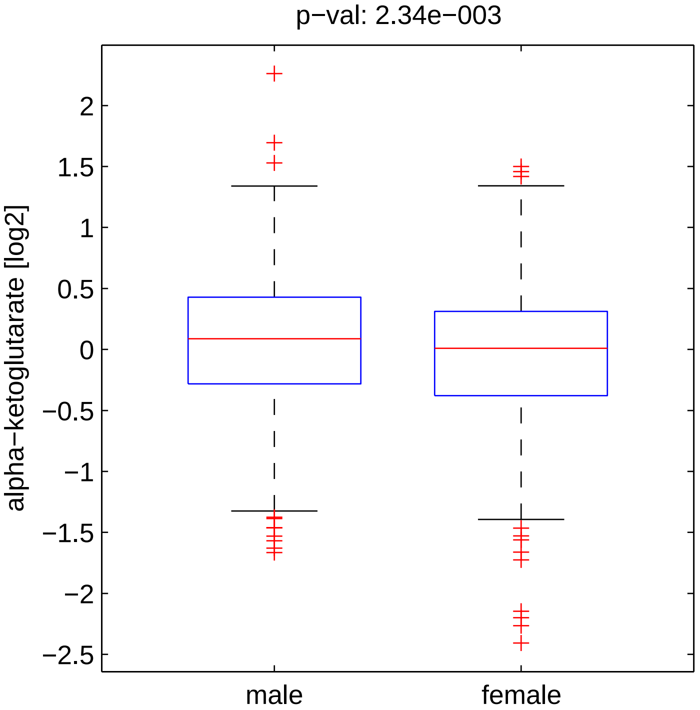
p−val: 2.34e−003
alpha−ketoglutarate [log2]
2
1.5
1
0.5
0
−0.5
−1
−1.5
−2
−2.5
male
female

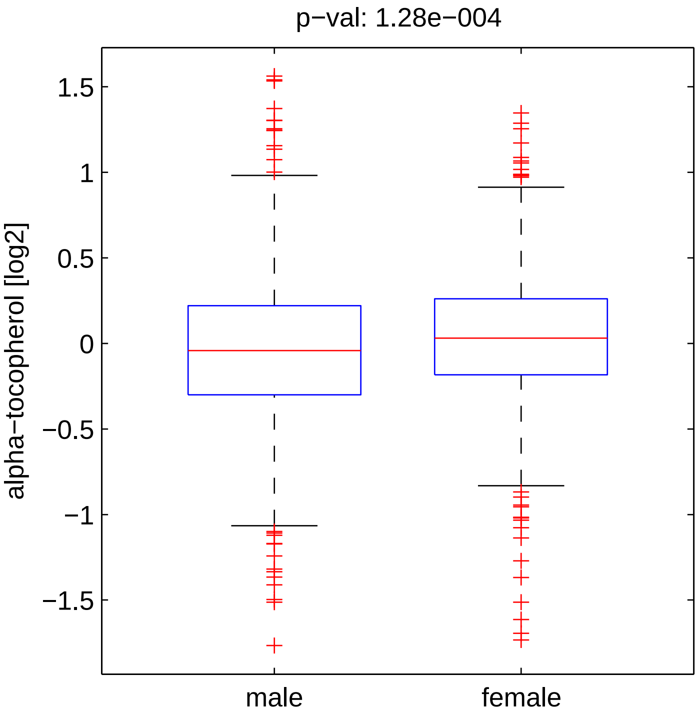

p−val: 1.28e−004
alpha−tocopherol [log2]
1.5
1
0.5
0
−0.5
−1
−1.5
male
female

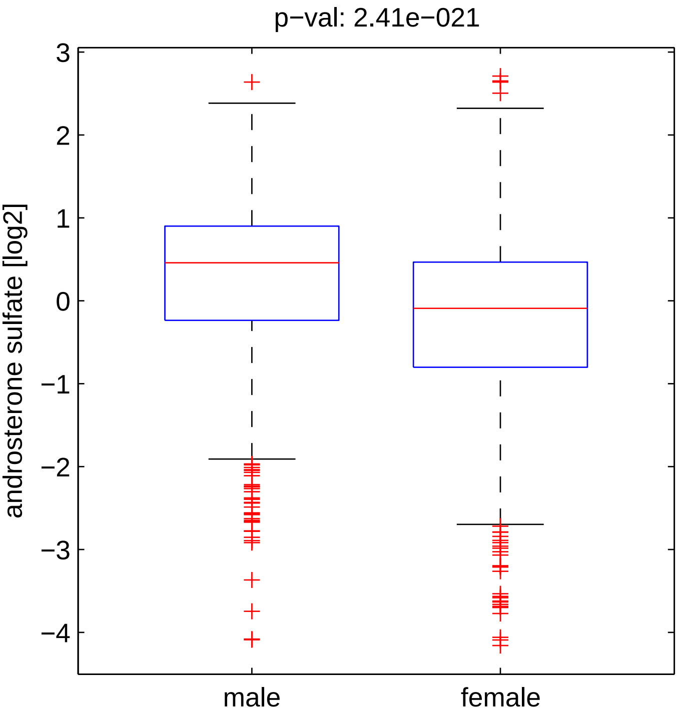

p−val: 2.41e−021
androsterone sulfate [log2]
3
2
1
0
−1
−2
−3
−4
male
female

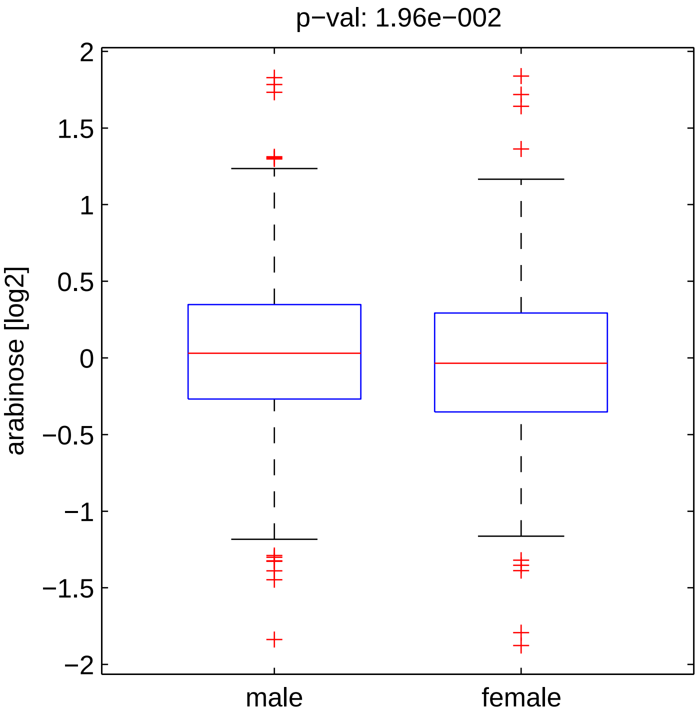

p−val: 1.96e−002
arabinose [log2]
2
1.5
1
0.5
0
−0.5
−1
−1.5
−2
male
female

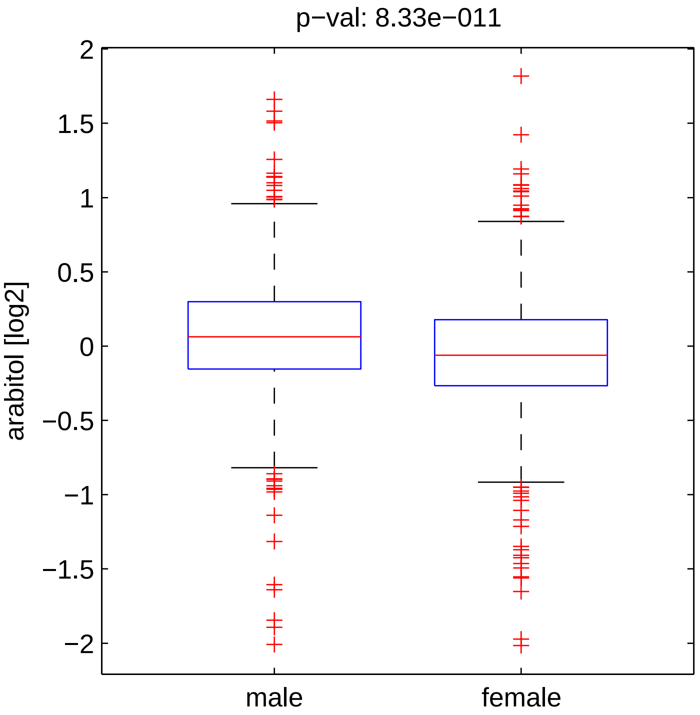
p−val: 8.33e−011
arabitol [log2]
2
1.5
1
0.5
0
−0.5
−1
−1.5
−2
male
female

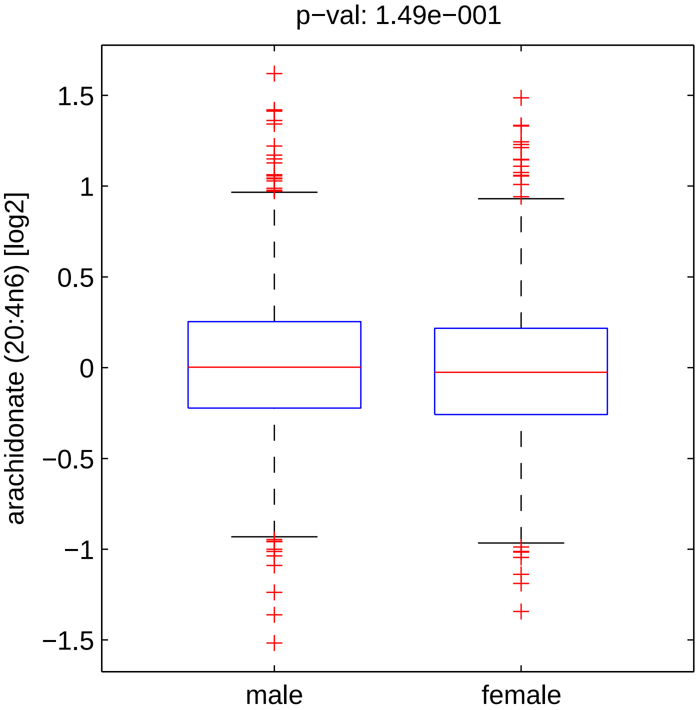
p−val: 1.49e−001
arachidonate (20:4n6) [log2]
1.5
1
0.5
0
−0.5
−1
−1.5
male
female

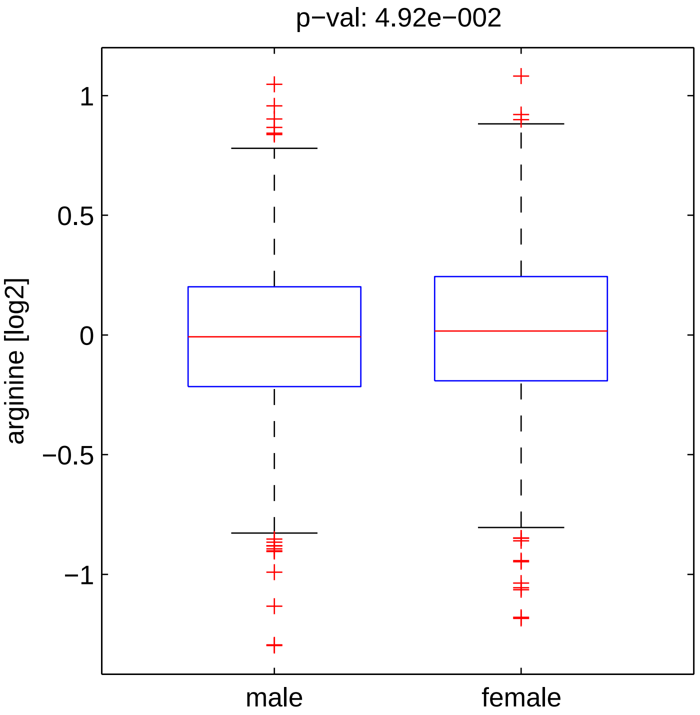

p−val: 4.92e−002
1
0.5
0
−0.5
−1
arginine [log2]
male
female

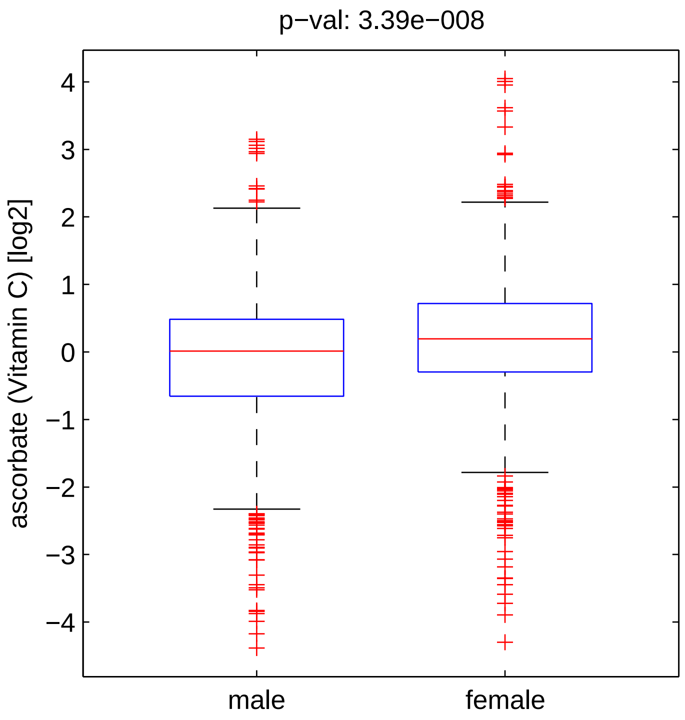
p−val: 3.39e−008
ascorbate (Vitamin C) [log2]
4
3
2
1
0
−1
−2
−3
−4
male
female

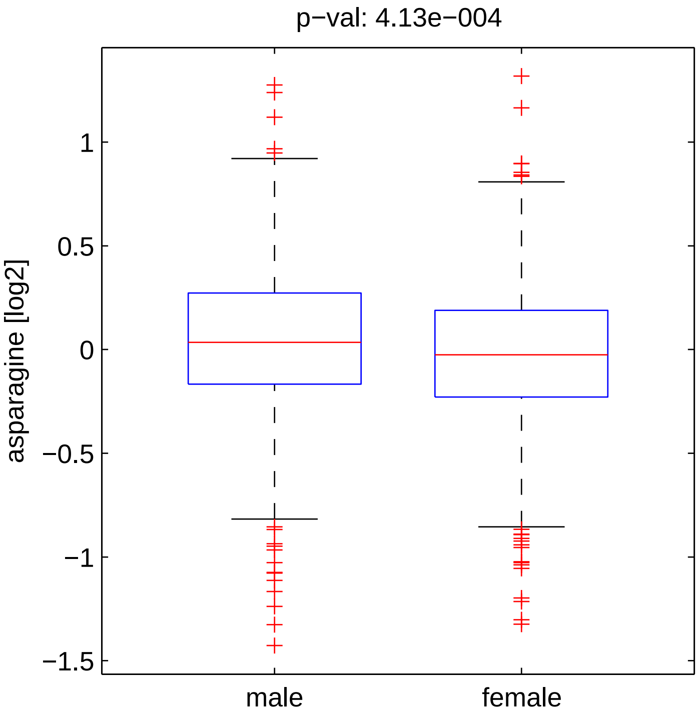

p−val: 4.13e−004
asparagine [log2]
1
0.5
0
−0.5
−1
−1.5
male
female

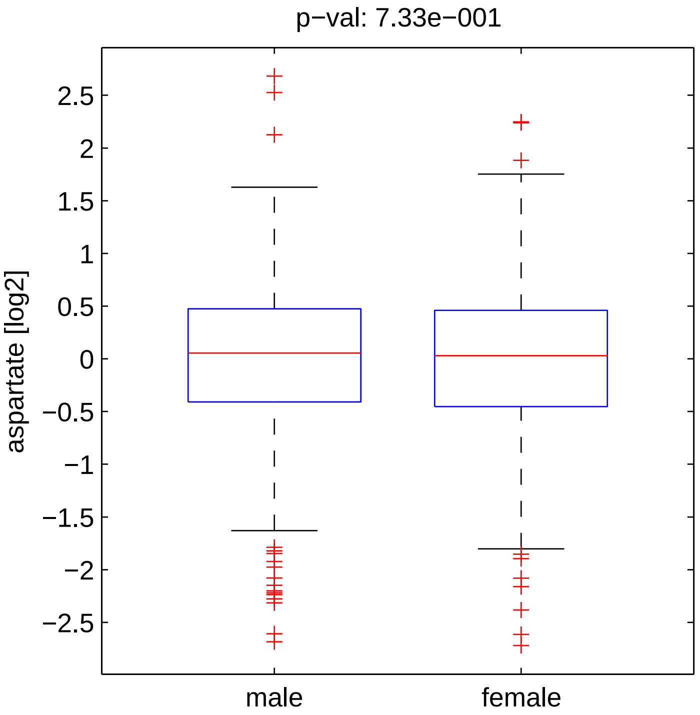
p−val: 7.33e−001
aspartate [log2]
2.5
2
1.5
1
0.5
0
−0.5
−1
−1.5
−2
−2.5
male
female

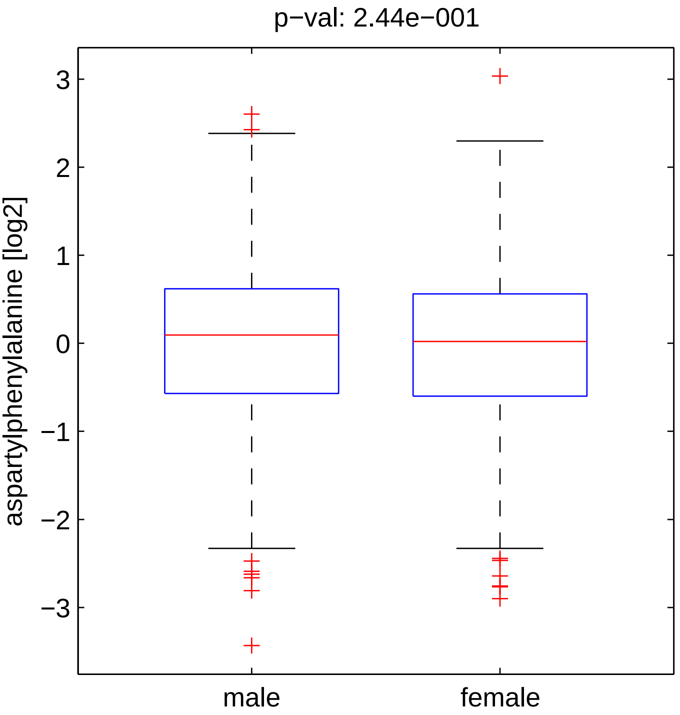
p−val: 2.44e−001
aspartylphenylalanine [log2]
3
2
1
0
−1
−2
−3
male
female

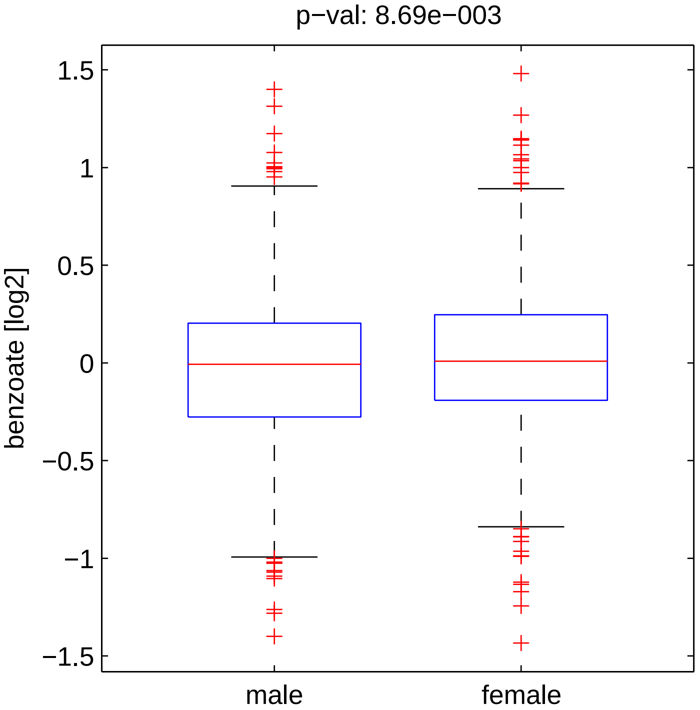
p−val: 8.69e−003
benzoate [log2]
1.5
1
0.5
0
−0.5
−1
−1.5
male
female

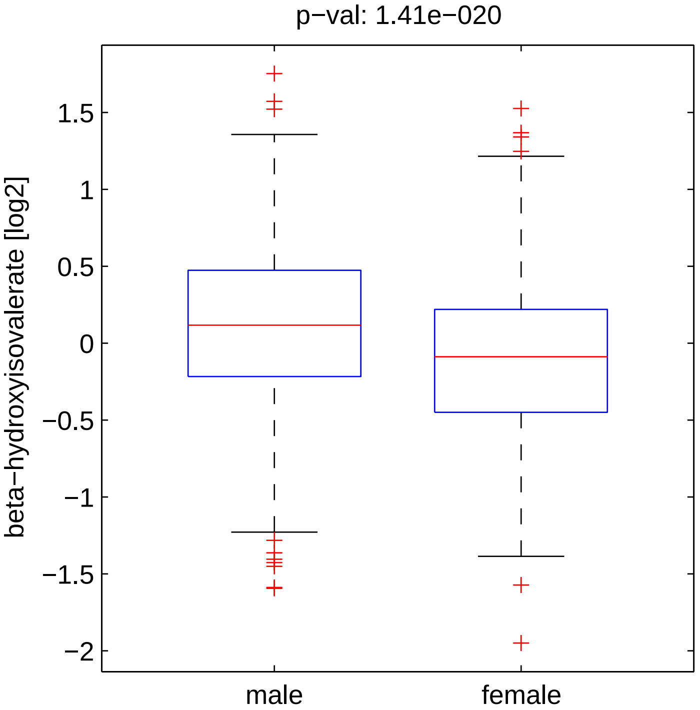
p−val: 1.41e−020
beta−hydroxyisovalerate [log2]
1.5
1
0.5
0
−0.5
−1
−1.5
−2
male
female

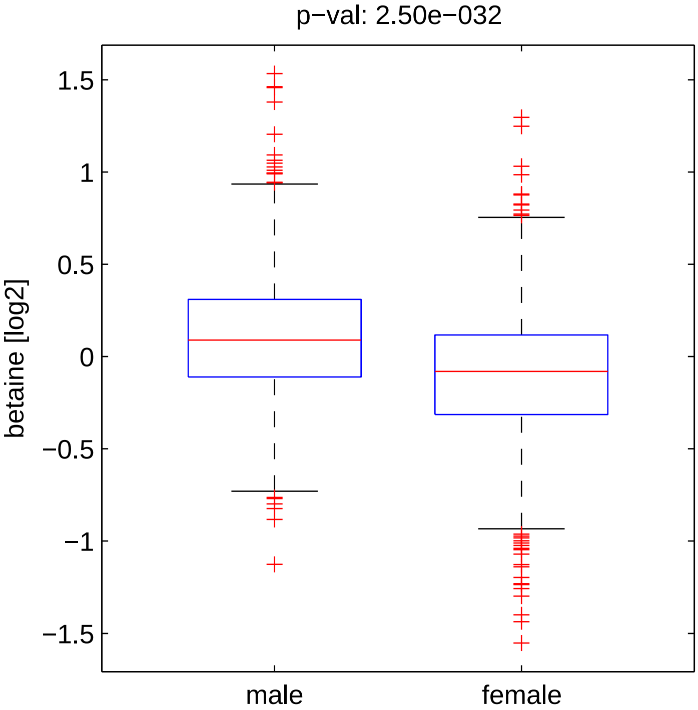
p−val: 2.50e−032
betaine [log2]
1.5
1
0.5
0
−0.5
−1
−1.5
male
female

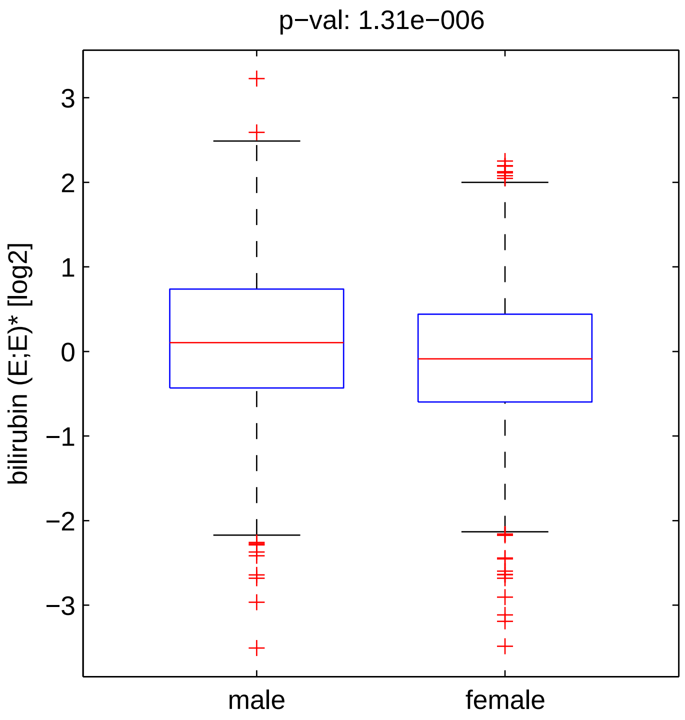
p−val: 1.31e−006
3
2
1
0
−1
−2
−3
bilirubin (E;E)* [log2]
male
female

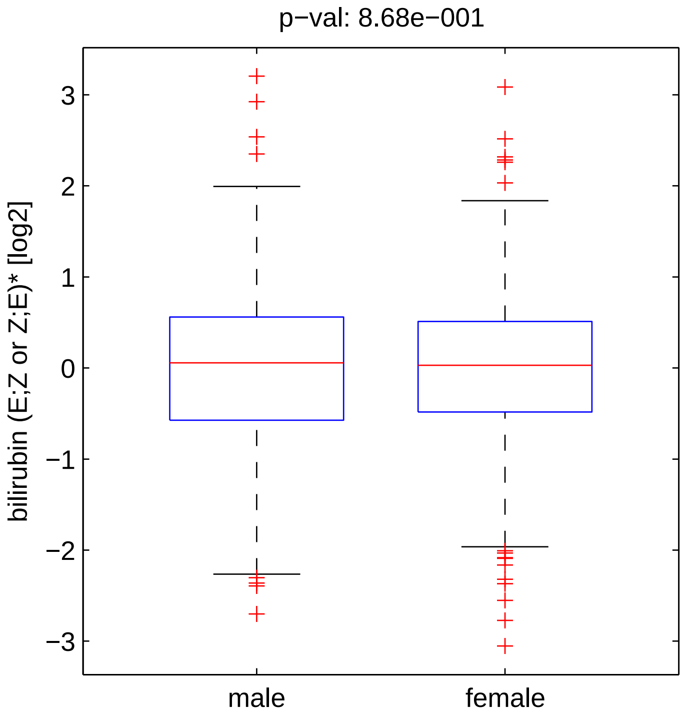
p−val: 8.68e−001
bilirubin (E;Z or Z;E)* [log2]
3
2
1
0
−1
−2
−3
male
female

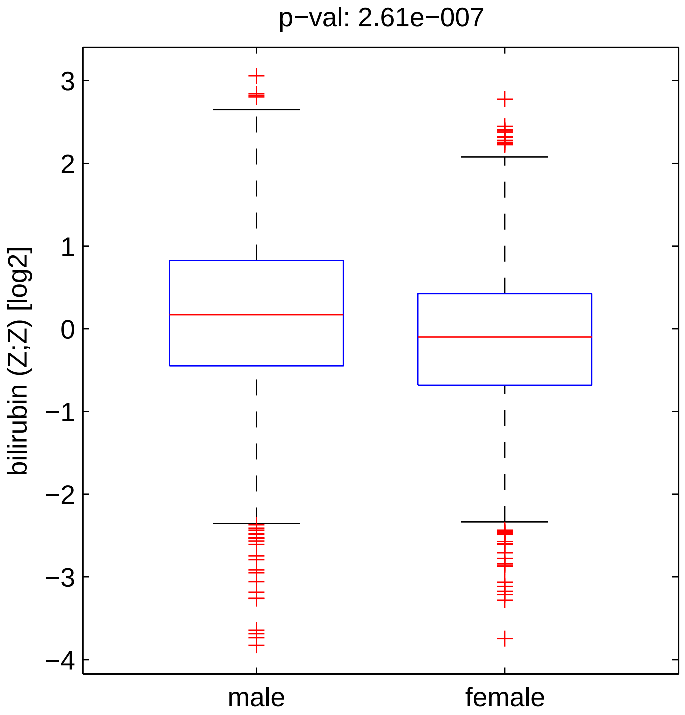
p−val: 2.61e−007
bilirubin (Z;Z) [log2]
3
2
1
0
−1
−2
−3
−4
male
female

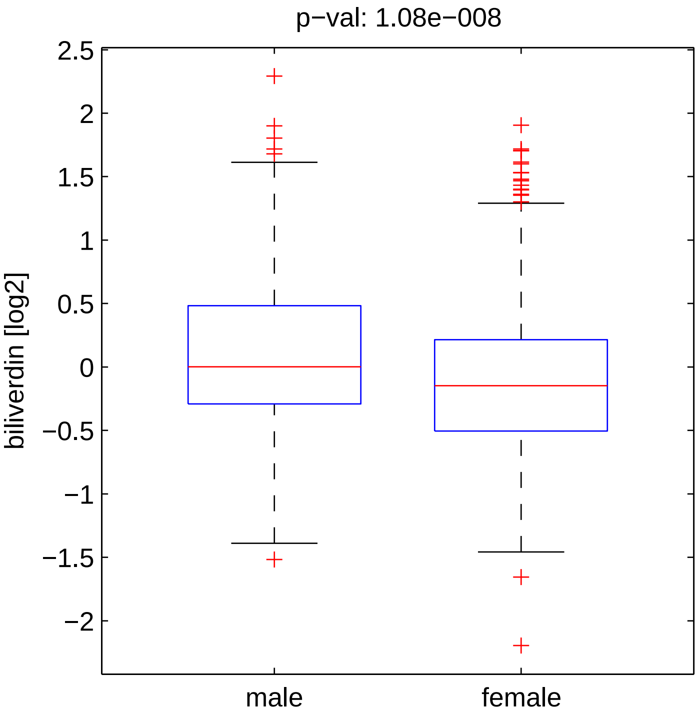
p−val: 1.08e−008
2.5
2
1.5
1
0.5
0
−0.5
−1
−1.5
−2
biliverdin [log2]
male
female

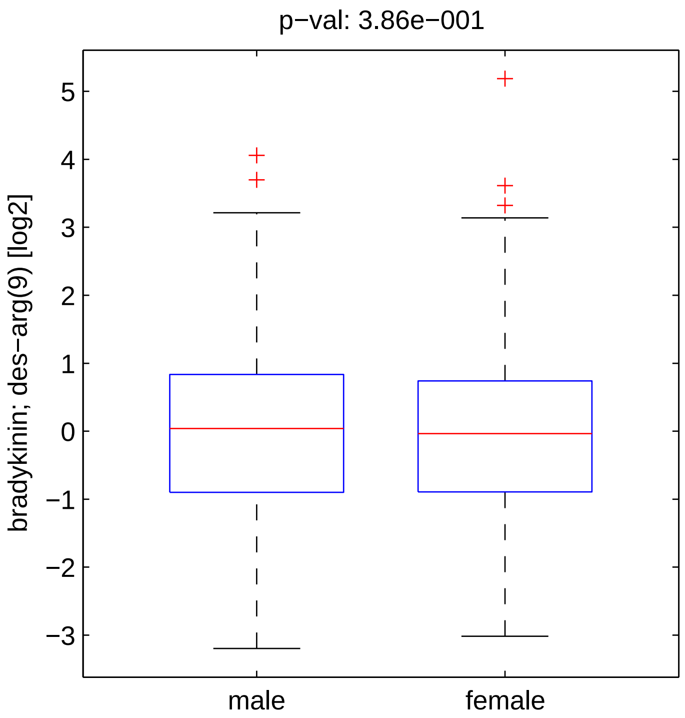
p−val: 3.86e−001
bradykinin; des−arg(9) [log2]
5
4
3
2
1
0
−1
−2
−3
male
female

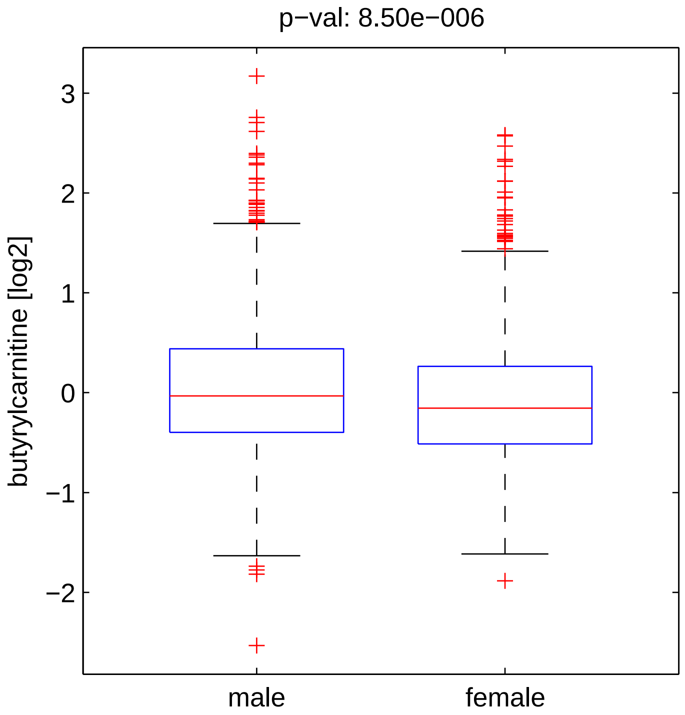
p−val: 8.50e−006
butyrylcarnitine [log2]
3
2
1
0
−1
−2
male
female

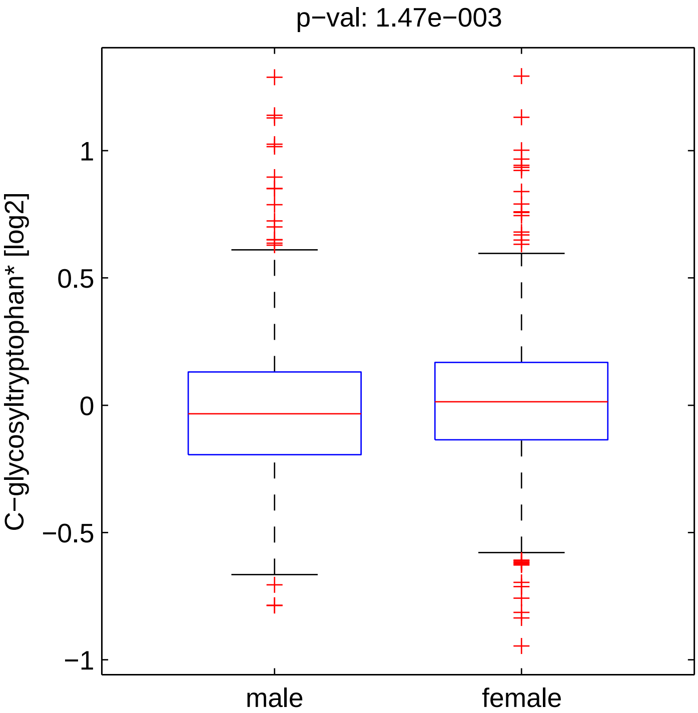

p−val: 1.47e−003
C−glycosyltryptophan* [log2]
1
0.5
0
−0.5
−1
male
female

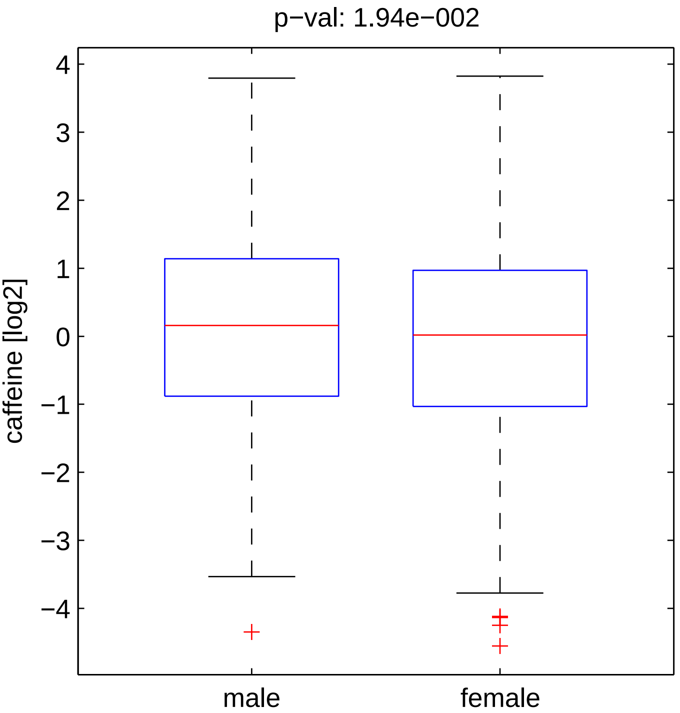
p−val: 1.94e−002
caffeine [log2]
4
3
2
1
0
−1
−2
−3
−4
male
female

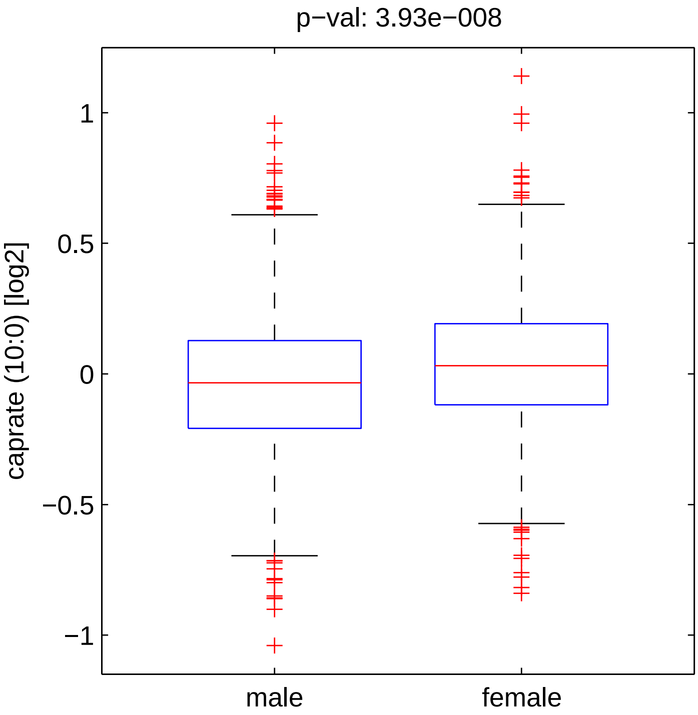
p−val: 3.93e−008
caprate (10:0) [log2]
1
0.5
0
−0.5
−1
male
female

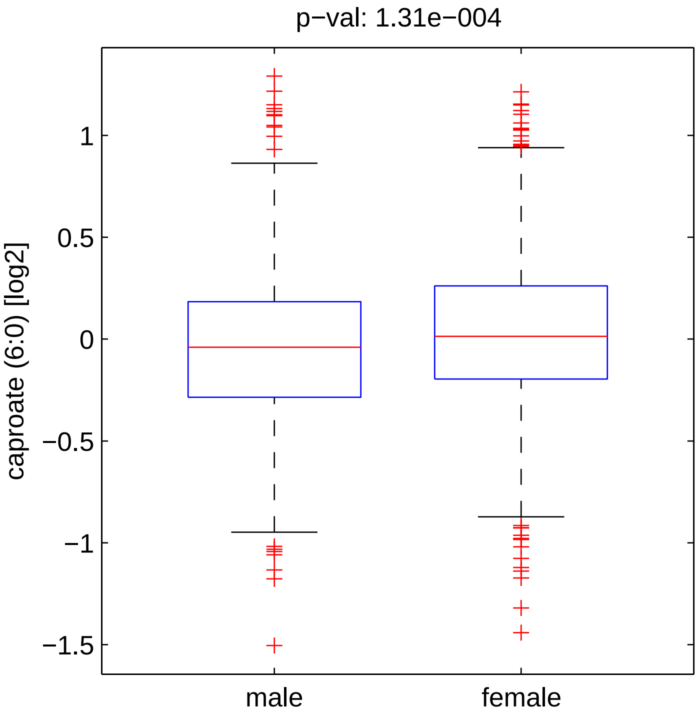
p−val: 1.31e−004
caproate (6:0) [log2]
1
0.5
0
−0.5
−1
−1.5
male
female

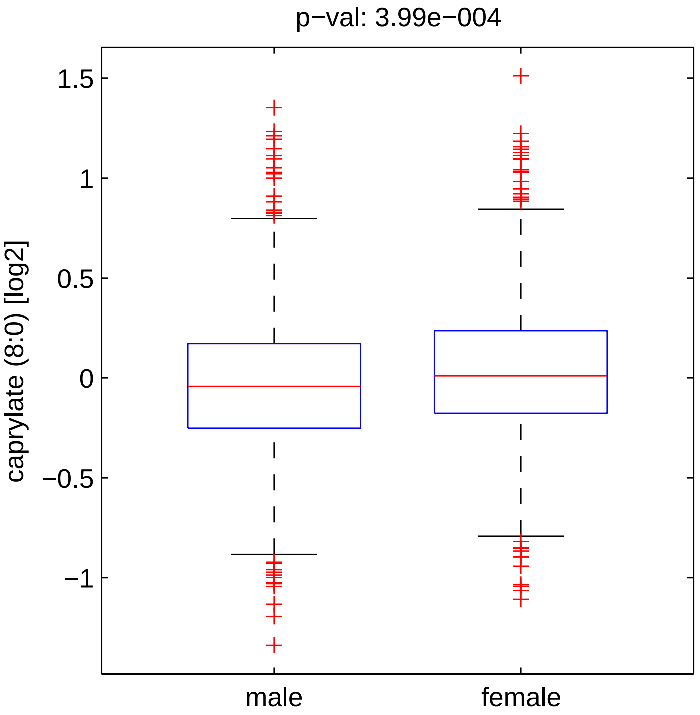
p−val: 3.99e−004
caprylate (8:0) [log2]
1.5
1
0.5
0
−0.5
−1
male
female

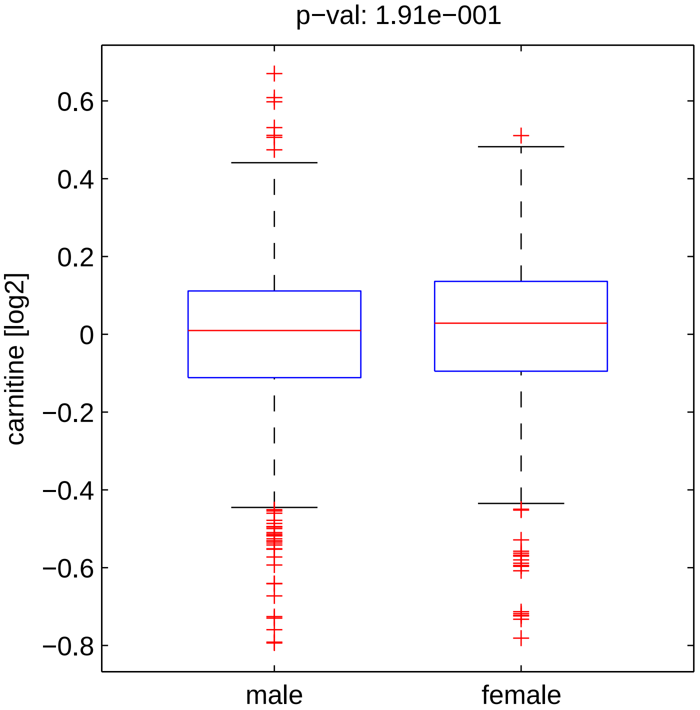
p−val: 1.91e−001
carnitine [log2]
0.6
0.4
0.2
0
−0.2
−0.4
−0.6
−0.8
male
female

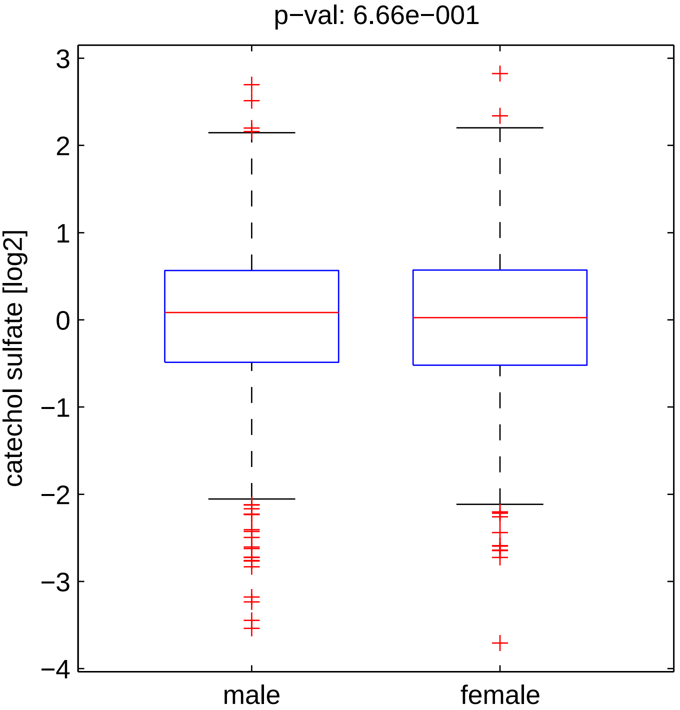
p−val: 6.66e−001
catechol sulfate [log2]
3
2
1
0
−1
−2
−3
−4
male
female

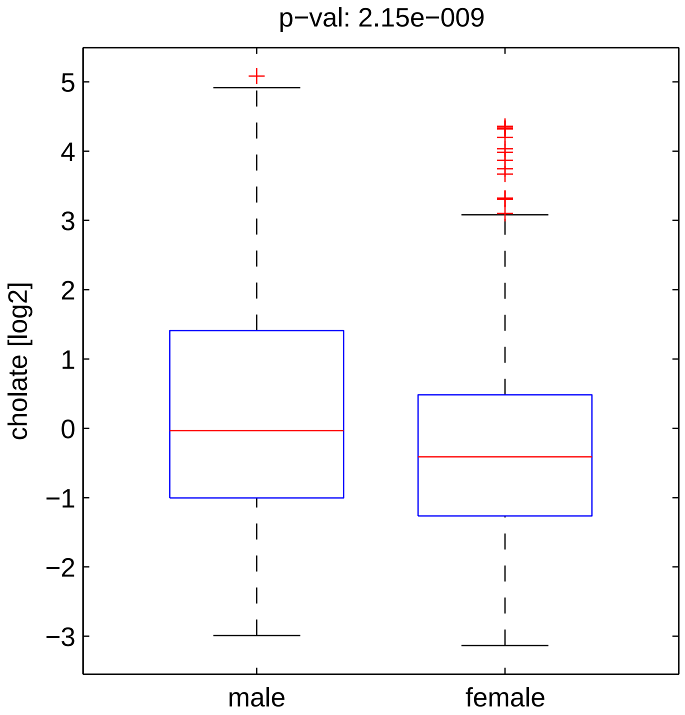
p−val: 2.15e−009
cholate [log2]
5
4
3
2
1
0
−1
−2
−3
male
female

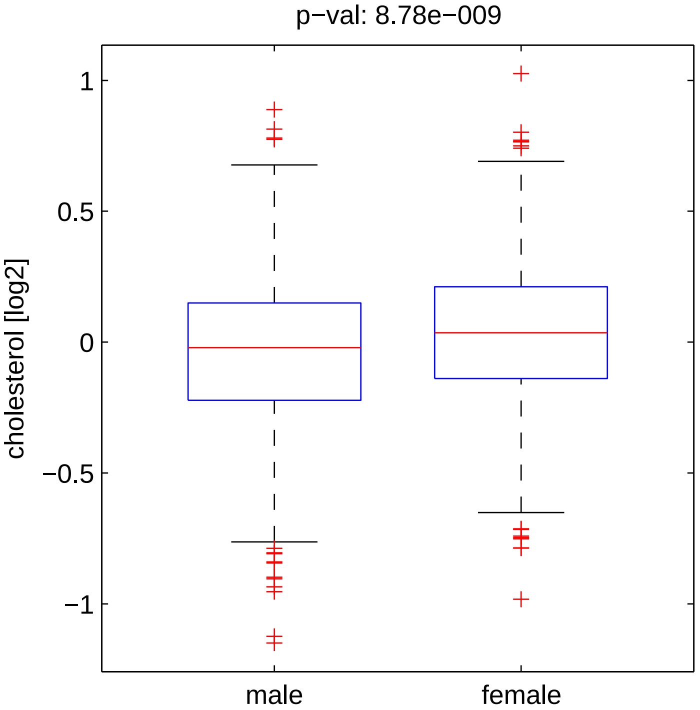

p−val: 8.78e−009
cholesterol [log2]
1
0.5
0
−0.5
−1
male
female

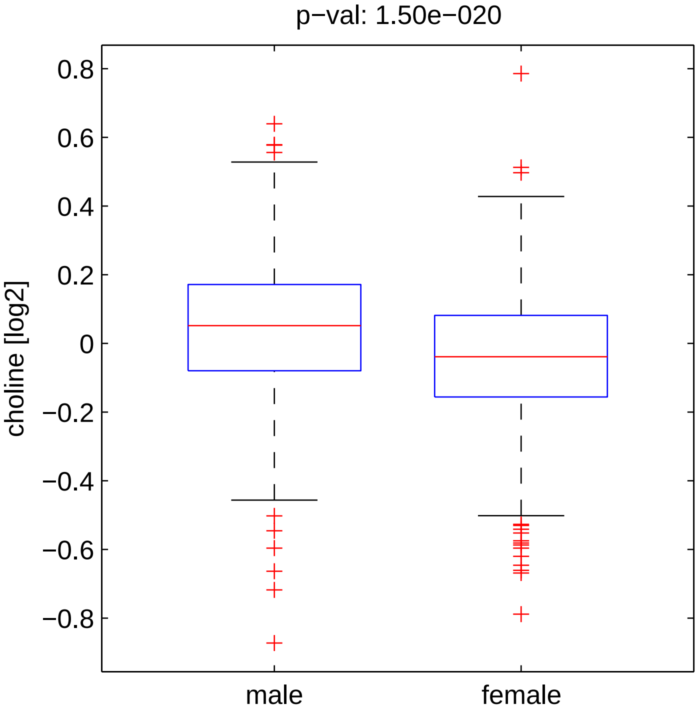
p−val: 1.50e−020
0.8
0.6
0.4
0.2
0
−0.2
−0.4
−0.6
−0.8
choline [log2]
male
female

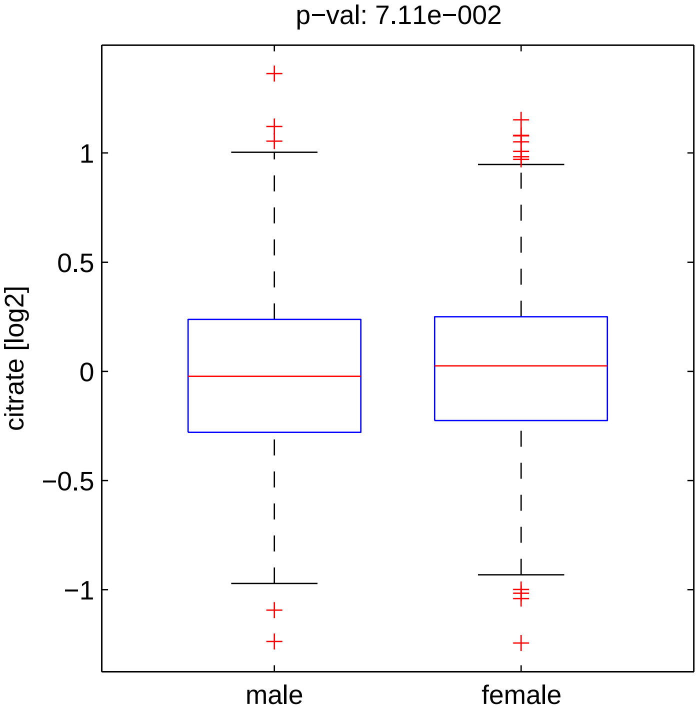

p−val: 7.11e−002
citrate [log2]
1
0.5
0
−0.5
−1
male
female

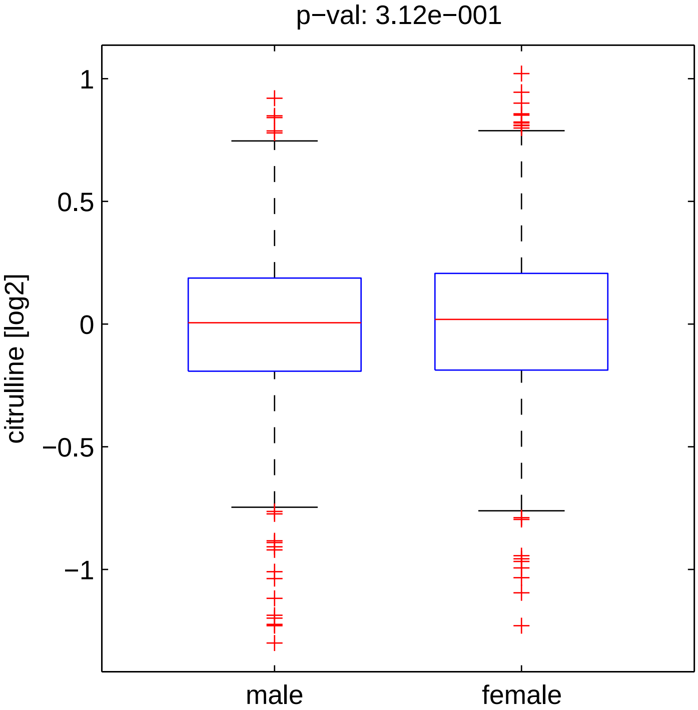
p−val: 3.12e−001
1
0.5
0
−0.5
−1
citrulline [log2]
male
female

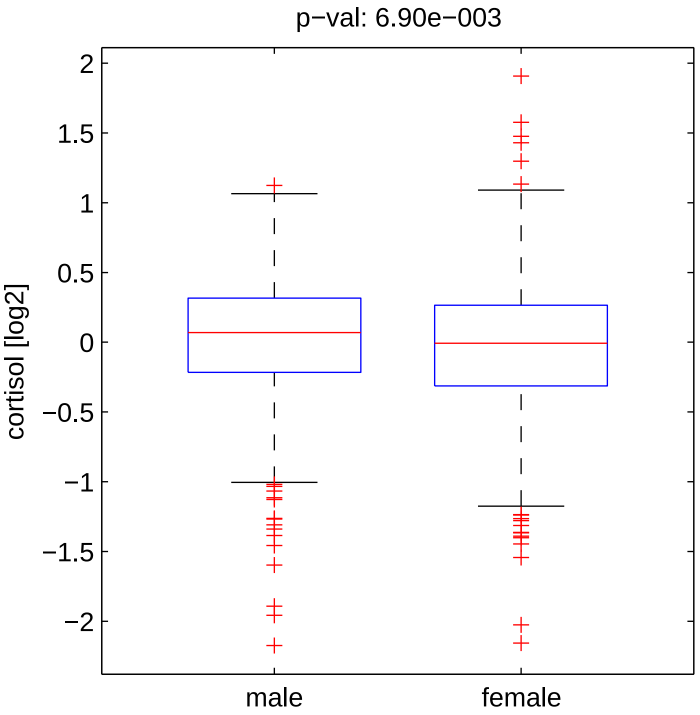

p−val: 6.90e−003
cortisol [log2]
2
1.5
1
0.5
0
−0.5
−1
−1.5
−2
male
female

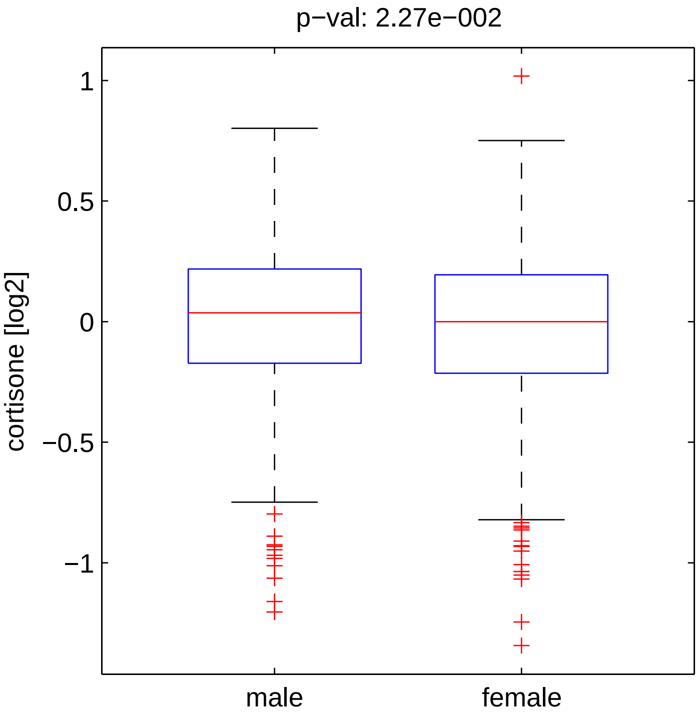

p−val: 2.27e−002
1
0.5
0
−0.5
−1
cortisone [log2]
male
female

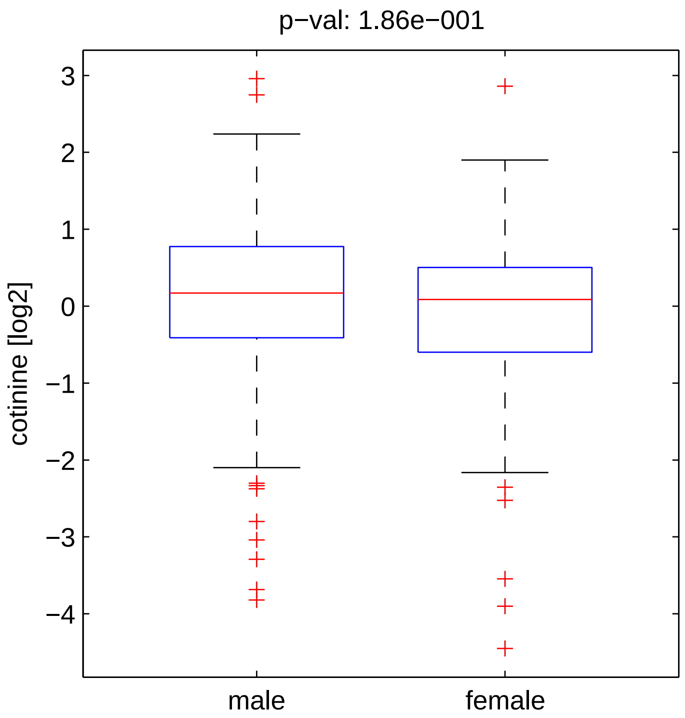
p−val: 1.86e−001
cotinine [log2]
3
2
1
0
−1
−2
−3
−4
male
female

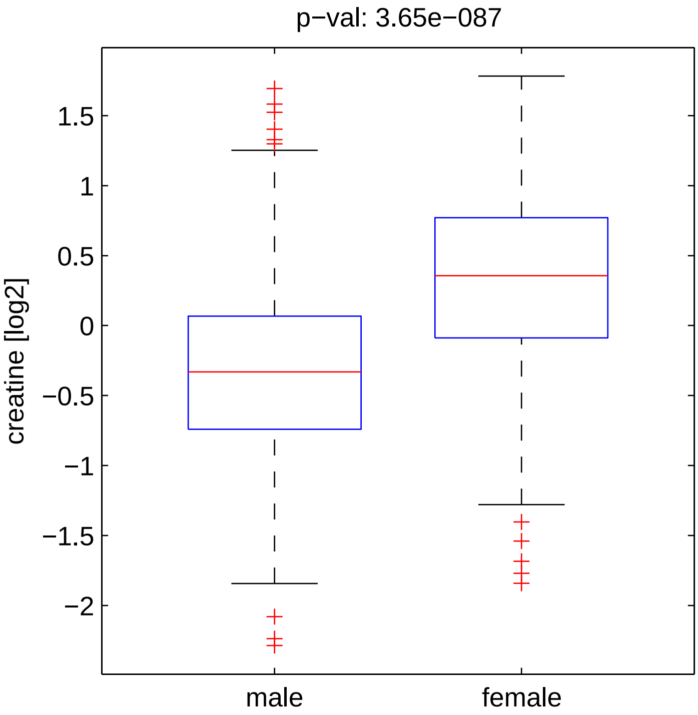

p−val: 3.65e−087
creatine [log2]
1.5
1
0.5
0
−0.5
−1
−1.5
−2
male
female

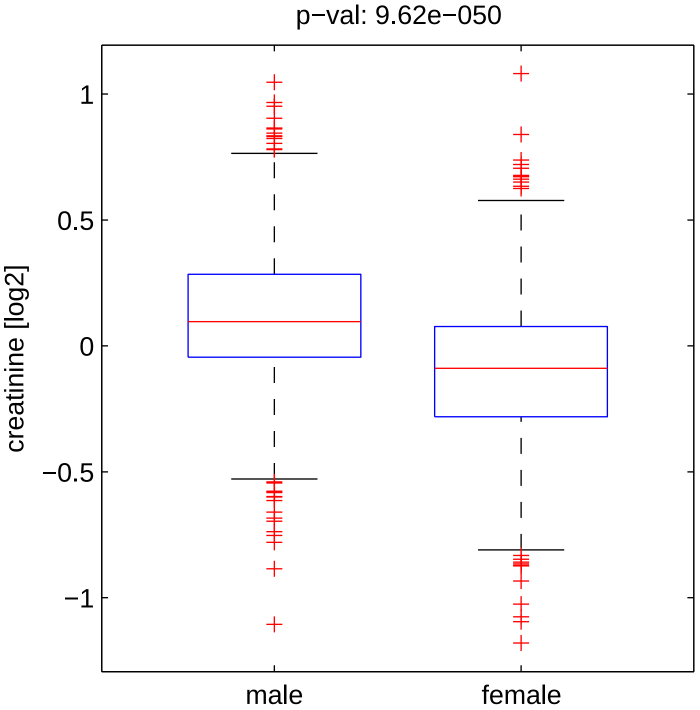
p−val: 9.62e−050
creatinine [log2]
1
0.5
0
−0.5
−1
male
female

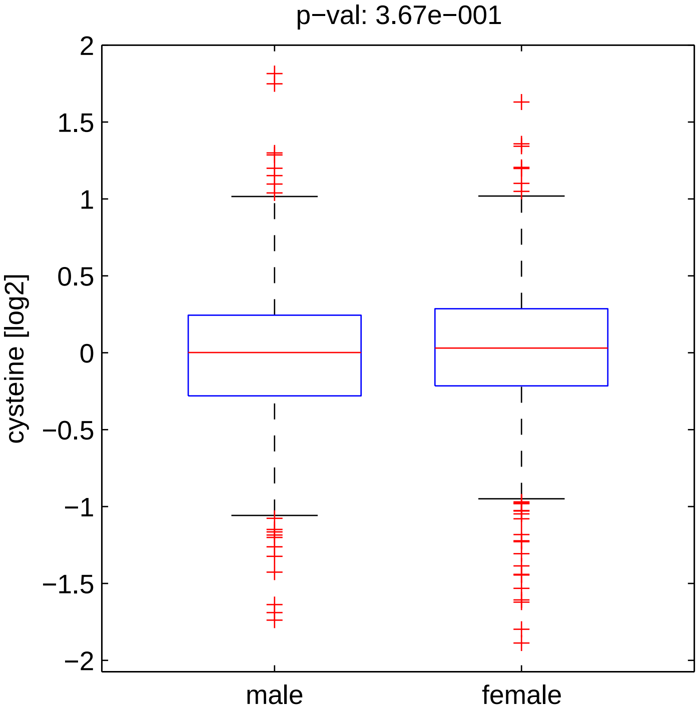
p−val: 3.67e−001
cysteine [log2]
2
1.5
1
0.5
0
−0.5
−1
−1.5
−2
male
female

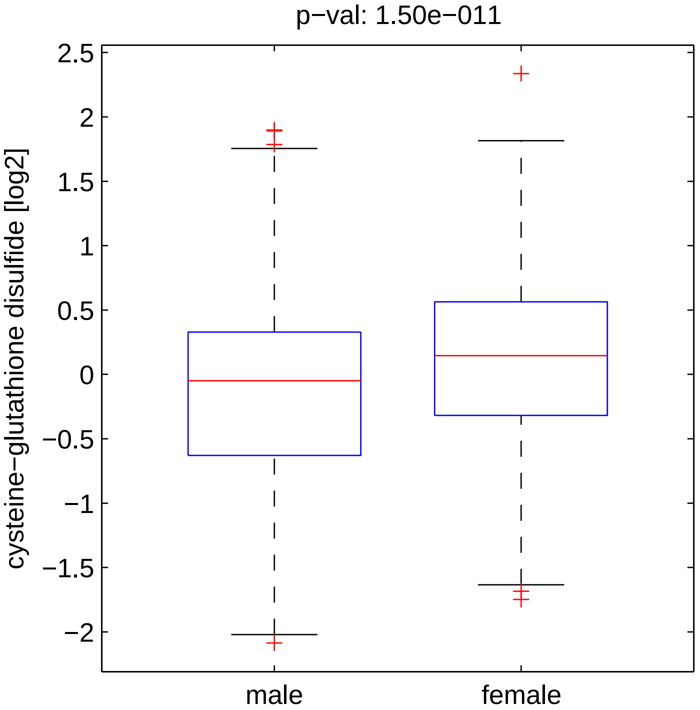

p−val: 1.50e−011
cysteine−glutathione disulfide [log2]
2.5
2
1.5
1
0.5
0
−0.5
−1
−1.5
−2
male
female

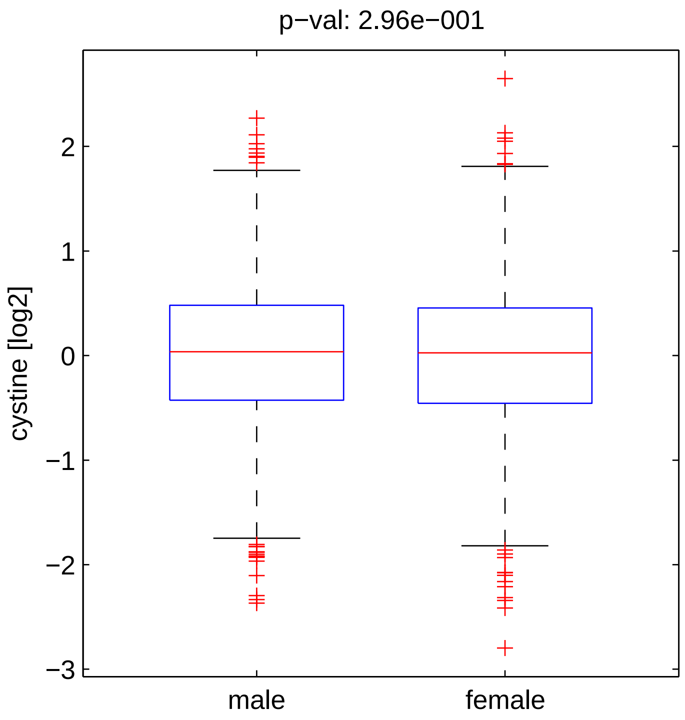
p−val: 2.96e−001
cystine [log2]
2
1
0
−1
−2
−3
male
female

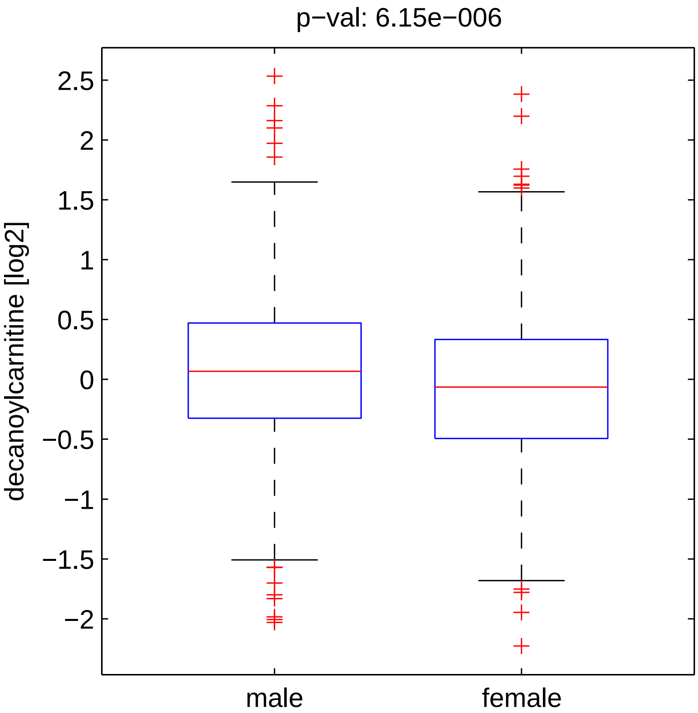

p−val: 6.15e−006
decanoylcarnitine [log2]
2.5
2
1.5
1
0.5
0
−0.5
−1
−1.5
−2
male
female

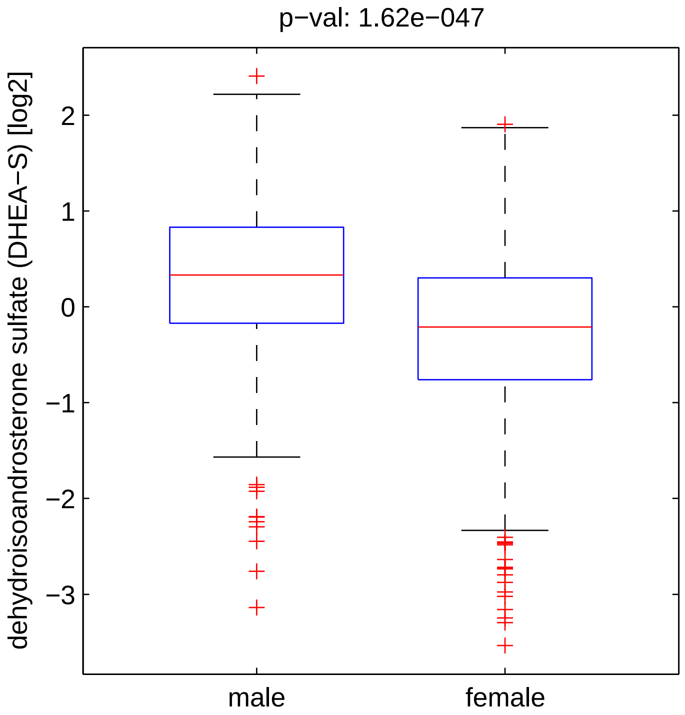

p−val: 1.62e−047
dehydroisoandrosterone sulfate (DHEA−S) [log2]
2
1
0
−1
−2
−3
male
female

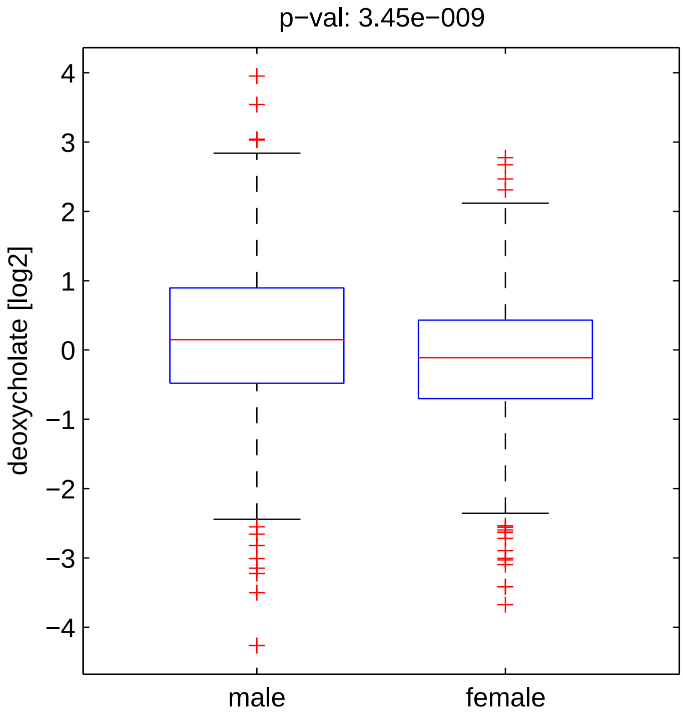

p−val: 3.45e−009
deoxycholate [log2]
4
3
2
1
0
−1
−2
−3
−4
male
female

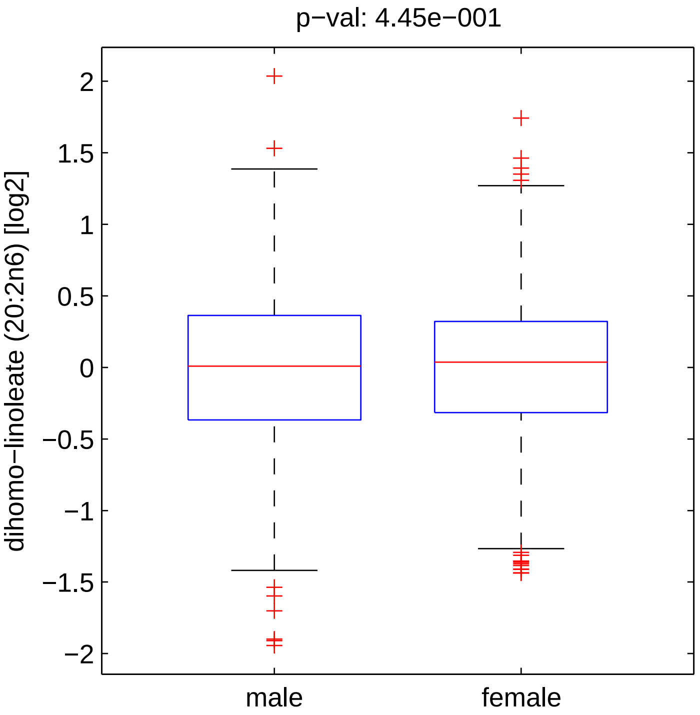
p−val: 4.45e−001
dihomo−linoleate (20:2n6) [log2]
2
1.5
1
0.5
0
−0.5
−1
−1.5
−2
male
female

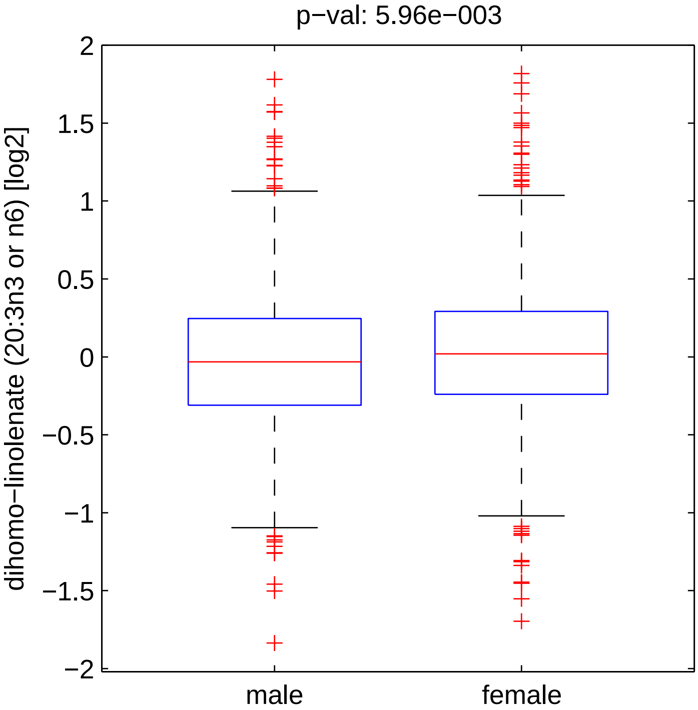

p−val: 5.96e−003
dihomo−linolenate (20:3n3 or n6) [log2]
2
1.5
1
0.5
0
−0.5
−1
−1.5
−2
male
female

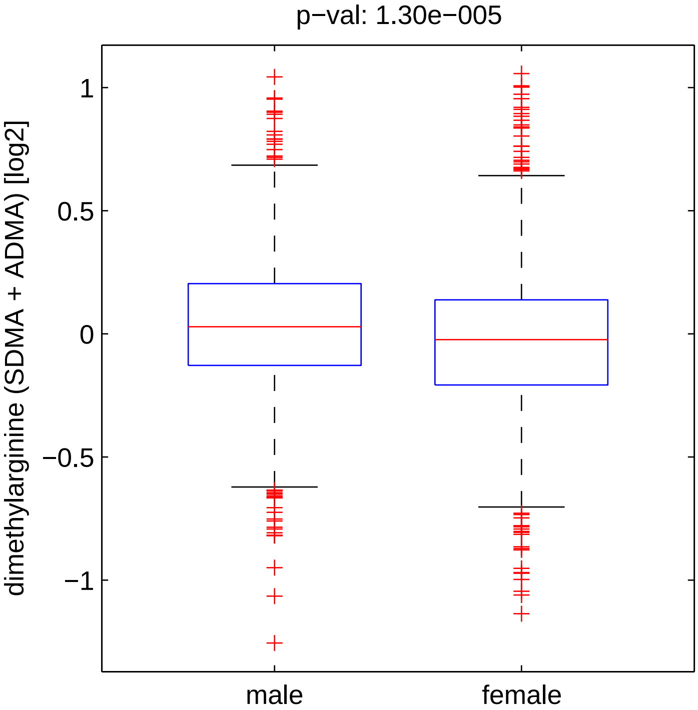

p−val: 1.30e−005
1
0.5
0
−0.5
−1
dimethylarginine (SDMA + ADMA) [log2]
male
female

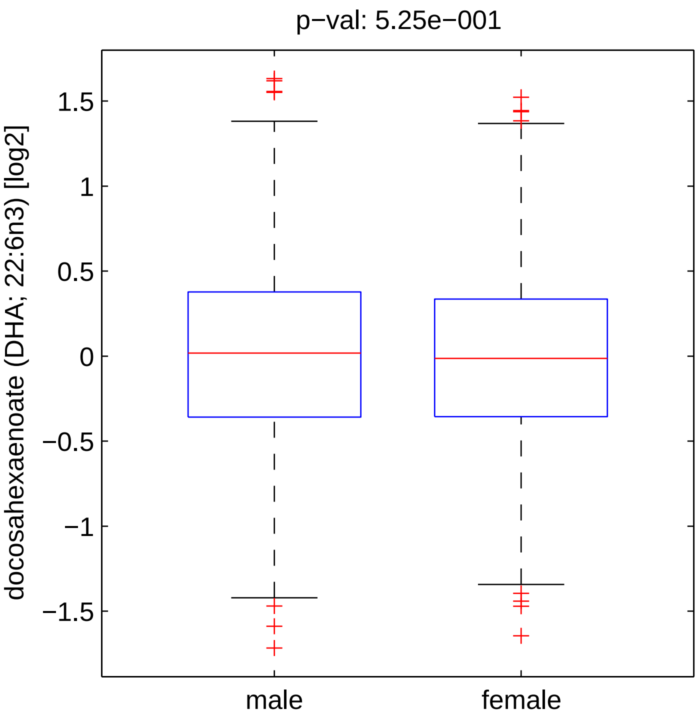
p−val: 5.25e−001
docosahexaenoate (DHA; 22:6n3) [log2]
1.5
1
0.5
0
−0.5
−1
−1.5
male
female

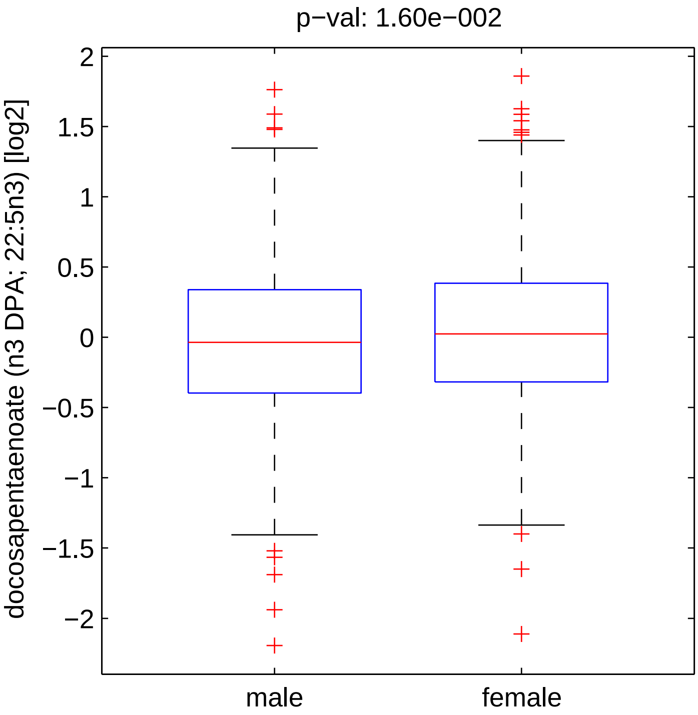
p−val: 1.60e−002
docosapentaenoate (n3 DPA; 22:5n3) [log2]
2
1.5
1
0.5
0
−0.5
−1
−1.5
−2
male
female

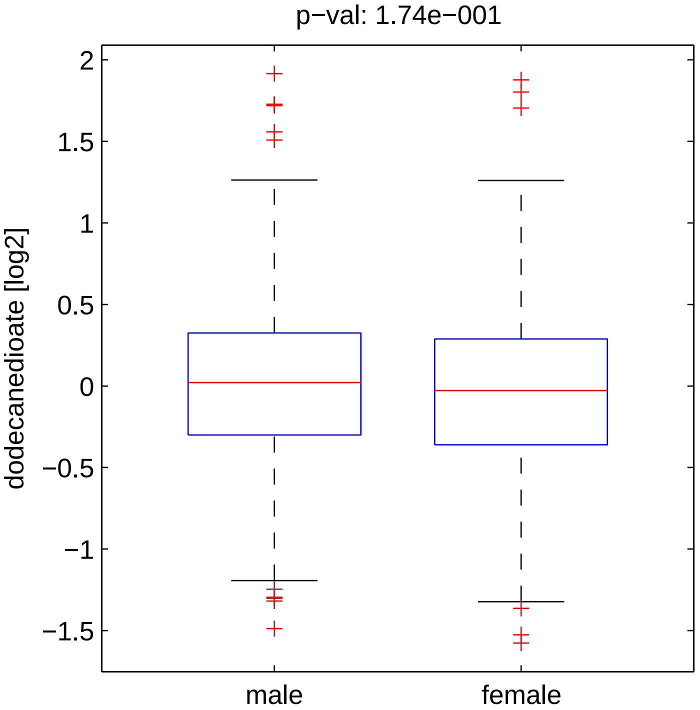
p−val: 1.74e−001
dodecanediodate [log2]
2
1.5
1
0.5
0
−0.5
−1
−1.5
male
female

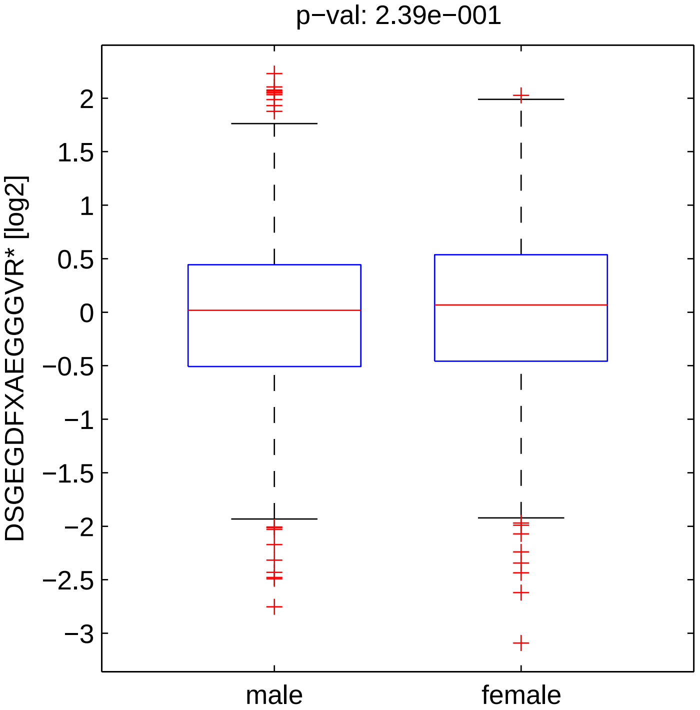
p−val: 2.39e−001
DSGEGDFXAEGGGVR* [log2]
2
1.5
1
0.5
0
−0.5
−1
−1.5
−2
−2.5
−3
male
female

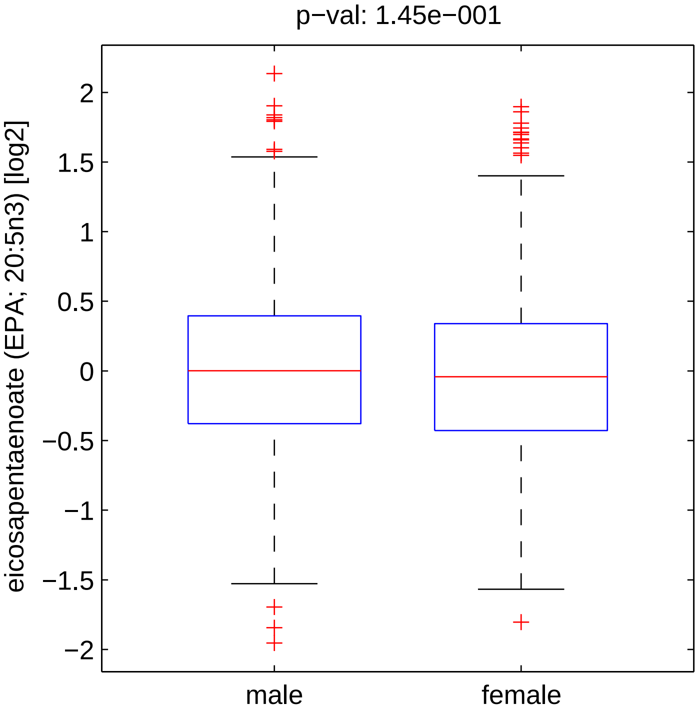

p−val: 1.45e−001
eicosapentaenoate (EPA; 20:5n3) [log2]
2
1.5
1
0.5
0
−0.5
−1
−1.5
−2
male
female

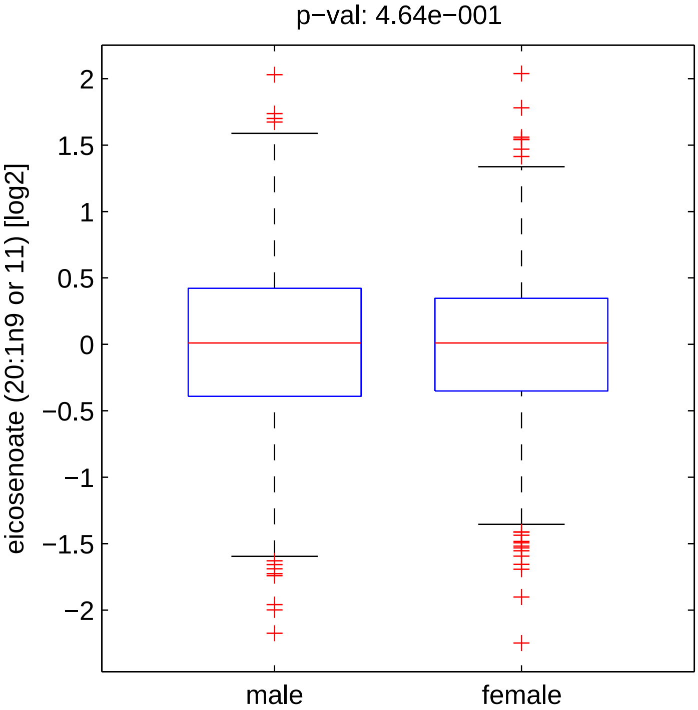

p−val: 4.64e−001
eicosenoate (20:1n9 or 11) [log2]
2
1.5
1
0.5
0
−0.5
−1
−1.5
−2
male
female

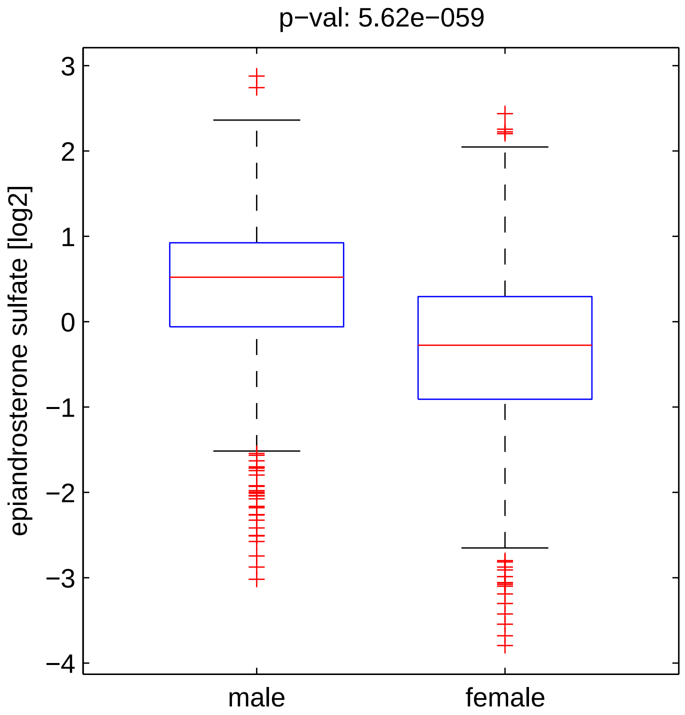
p−val: 5.62e−059
epiandrosterone sulfate [log2]
3
2
1
0
−1
−2
−3
−4
male
female

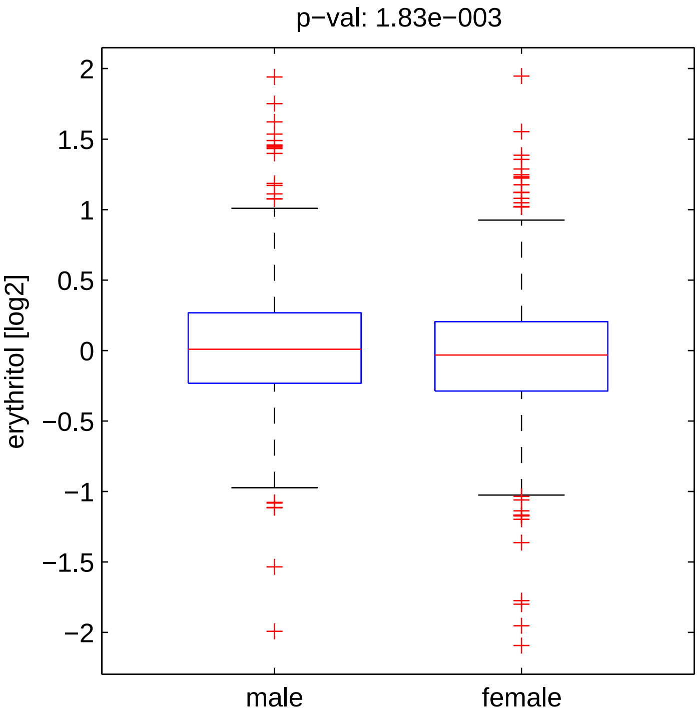
p−val: 1.83e−003
erythritol [log2]
2
1.5
1
0.5
0
−0.5
−1
−1.5
−2
male
female

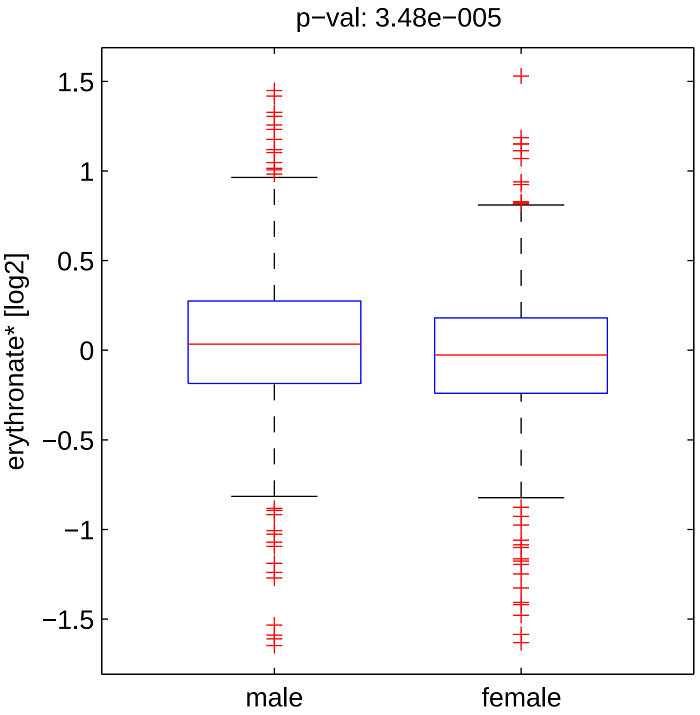
p−val: 3.48e−005
erythronate* [log2]
1.5
1
0.5
0
−0.5
−1
−1.5
male
female

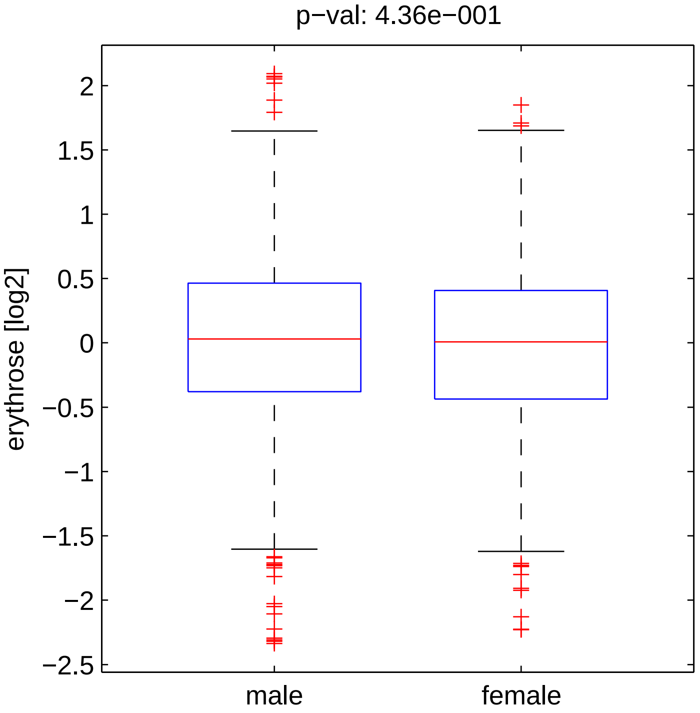
p−val: 4.36e−001
erythrose [log2]
2
1.5
1
0.5
0
−0.5
−1
−1.5
−2
−2.5
male
female

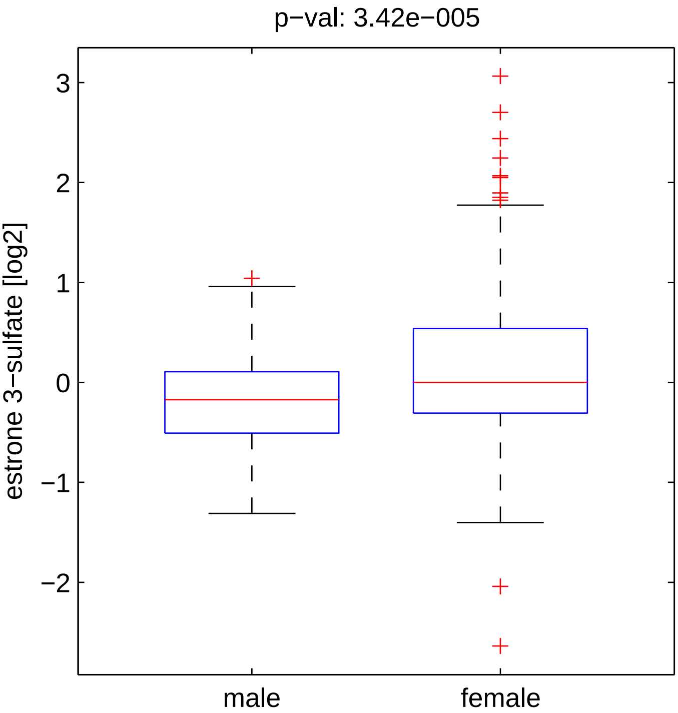

p−val: 3.42e−005
estrone 3−sulfate [log2]
3
2
1
0
−1
−2
male
female

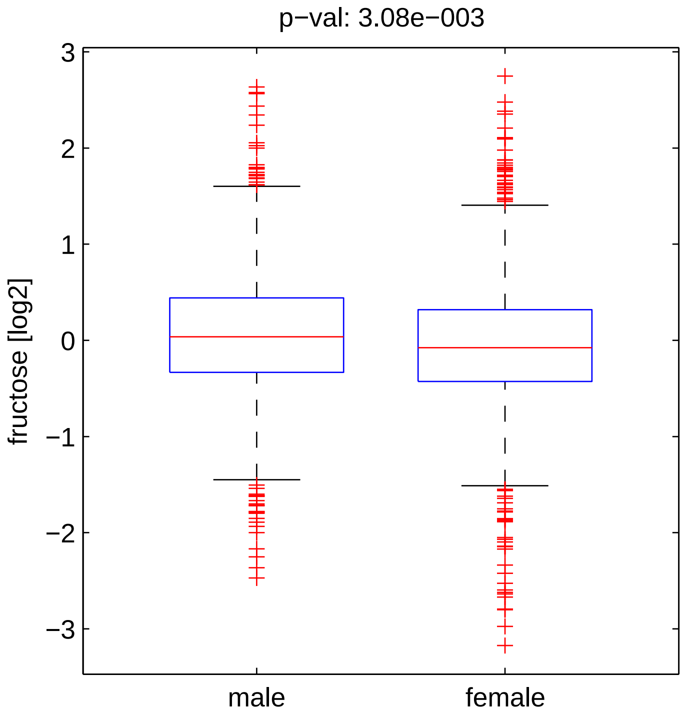
p−val: 3.08e−003
fructose [log2]
3
2
1
0
−1
−2
−3
male
female

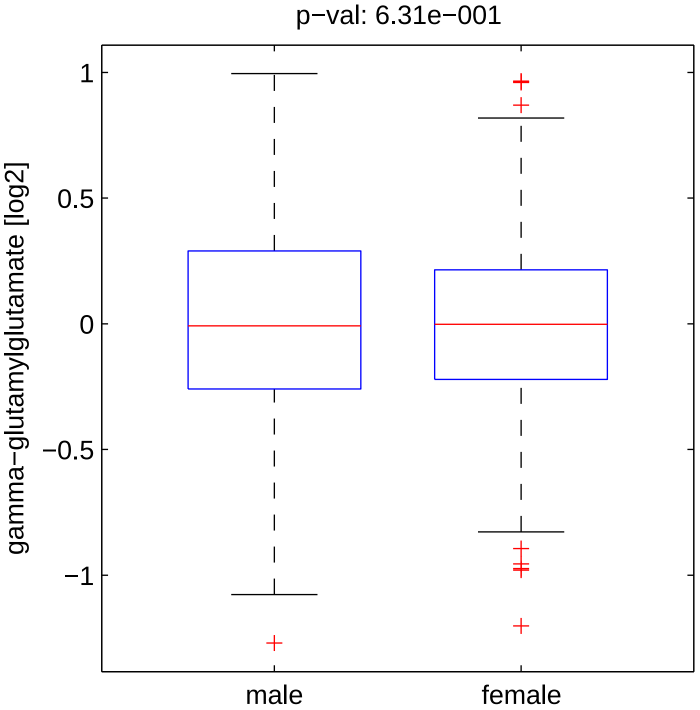
p−val: 6.31e−001
gamma−glutamylglutamate [log2]
1
0.5
0
−0.5
−1
male
female

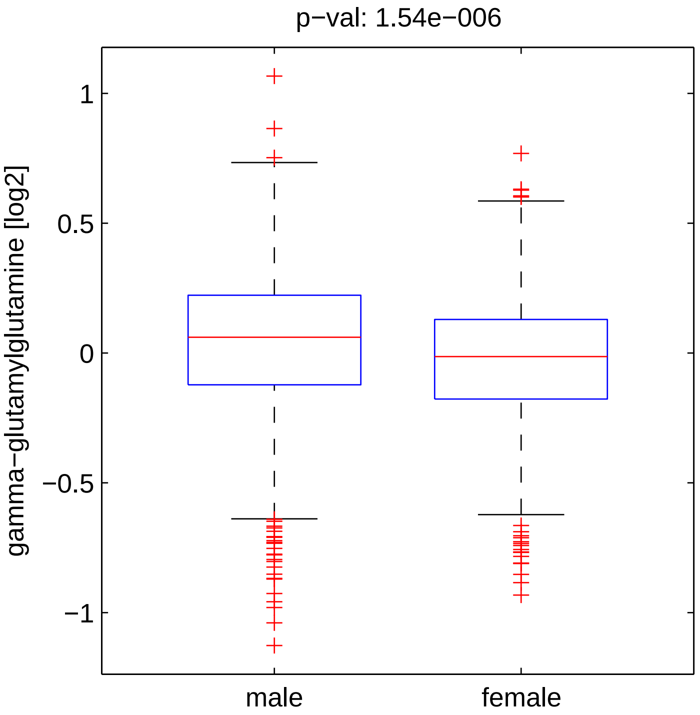

p−val: 1.54e−006
gamma−glutamylglutamine [log2]
1
0.5
0
−0.5
−1
male
female

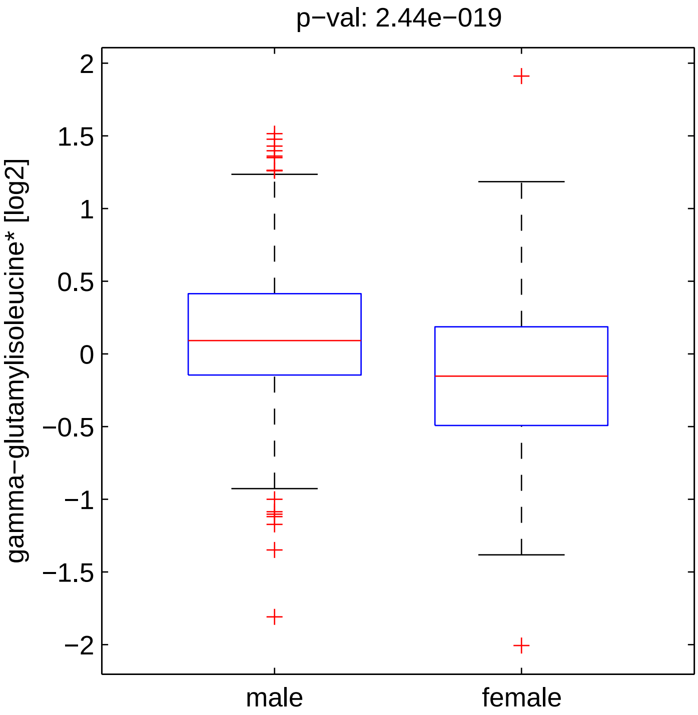
p−val: 2.44e−019
gamma−glutamylisoleucine* [log2]
2
1.5
1
0.5
0
−0.5
−1
−1.5
−2
male
female

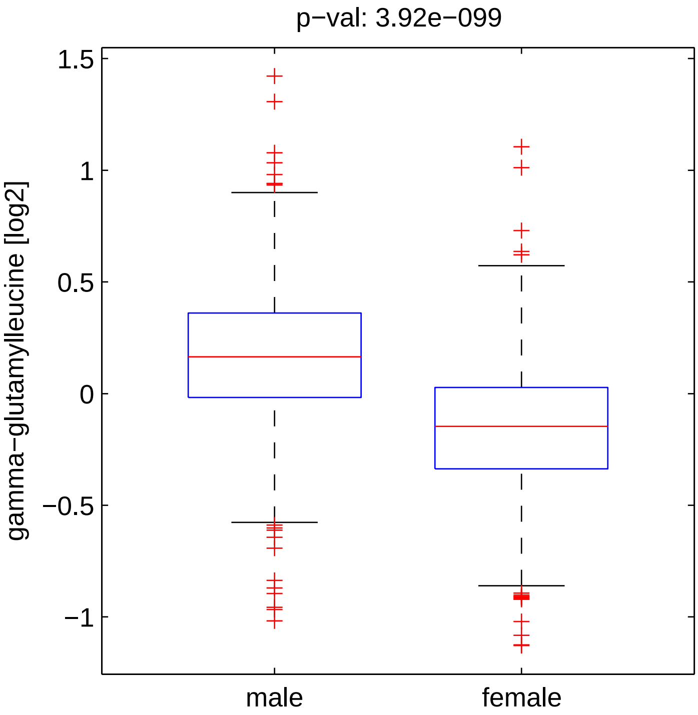
p−val: 3.92e−099
gamma−glutamylleucine [log2]
1.5
1
0.5
0
−0.5
−1
male
female

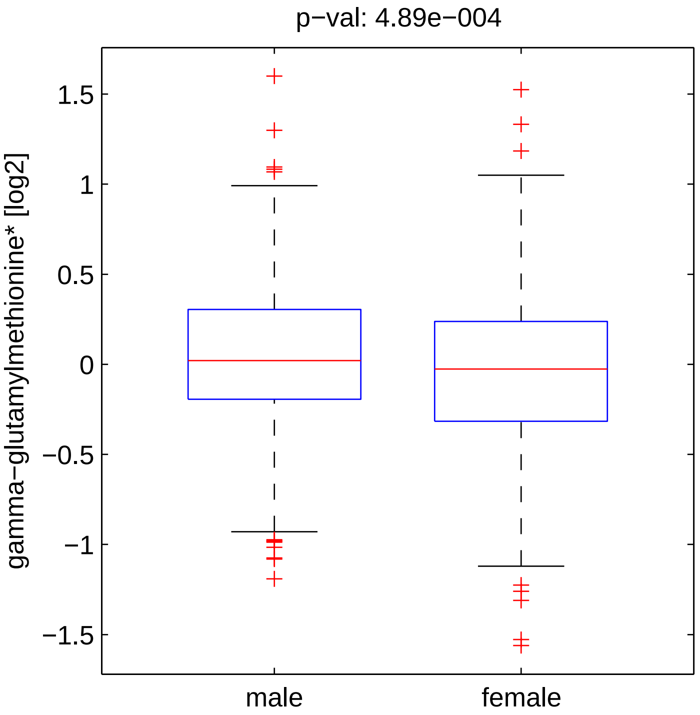
p−val: 4.89e−004
gamma−glutamylmethionine* [log2]
1.5
1
0.5
0
−0.5
−1
−1.5
male
female

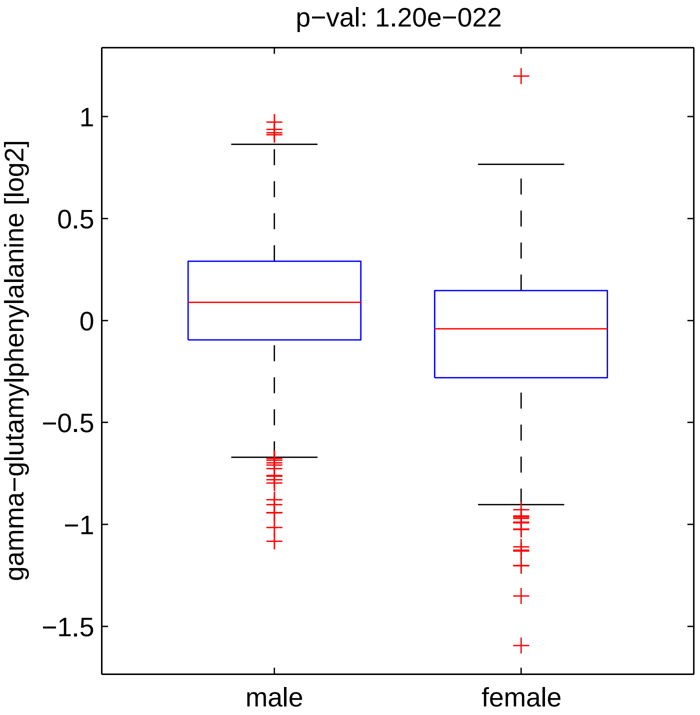

p−val: 1.20e−022
gamma−glutamylphenylalanine [log2]
1
0.5
0
−0.5
−1
−1.5
male
female

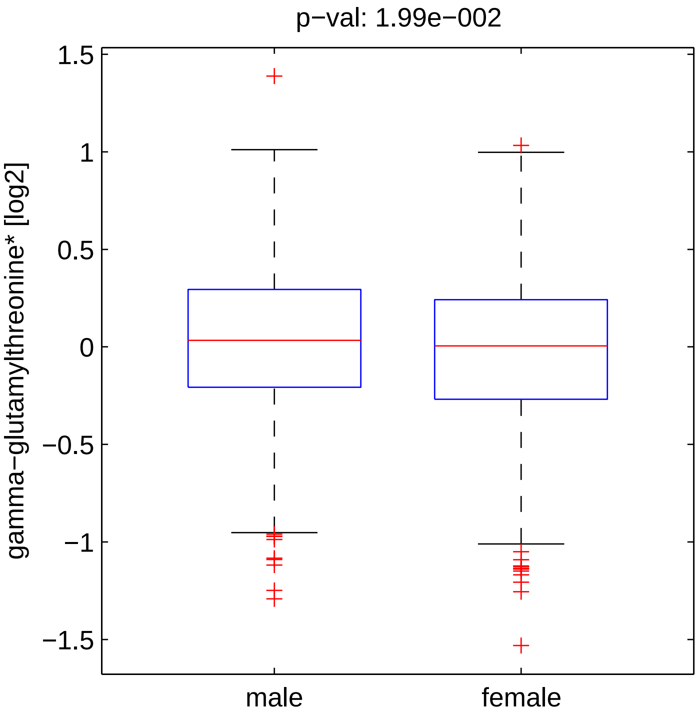
p−val: 1.99e−002
gamma−glutamylthreonine* [log2]
1.5
1
0.5
0
−0.5
−1
−1.5
male
female

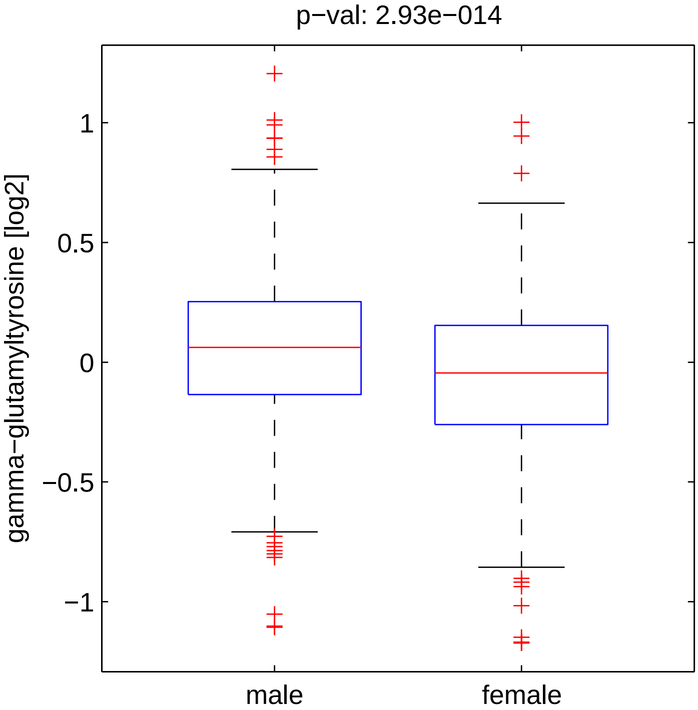
p−val: 2.93e−014
gamma−glutamyltyrosine [log2]
1
0.5
0
−0.5
−1
male
female

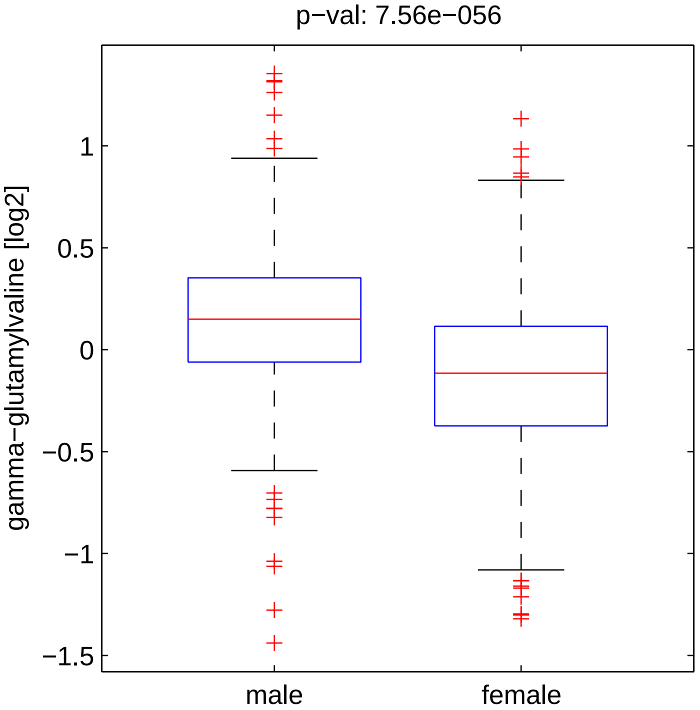
p−val: 7.56e−056
gamma−glutamylvaline [log2]
1
0.5
0
−0.5
−1
−1.5
male
female

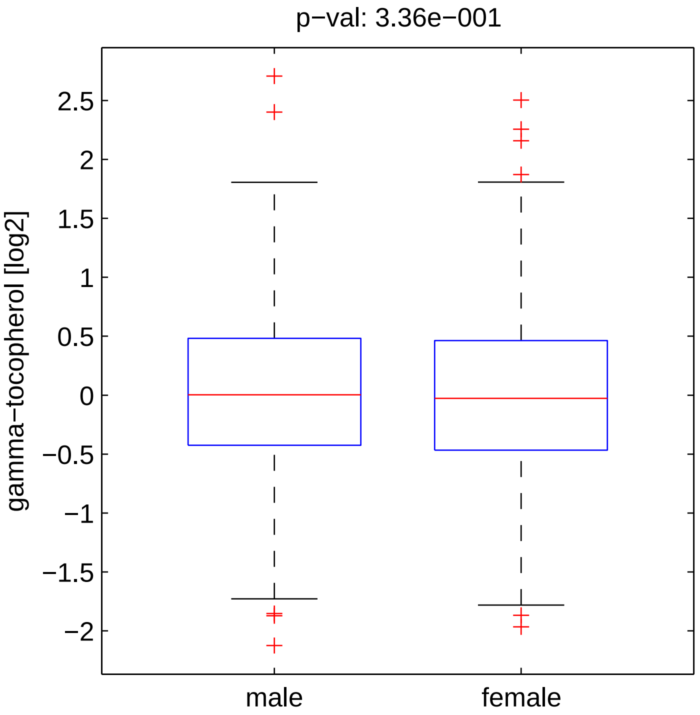
p−val: 3.36e−001
gamma−tocopherol [log2]
2.5
2
1.5
1
0.5
0
−0.5
−1
−1.5
−2
male
female

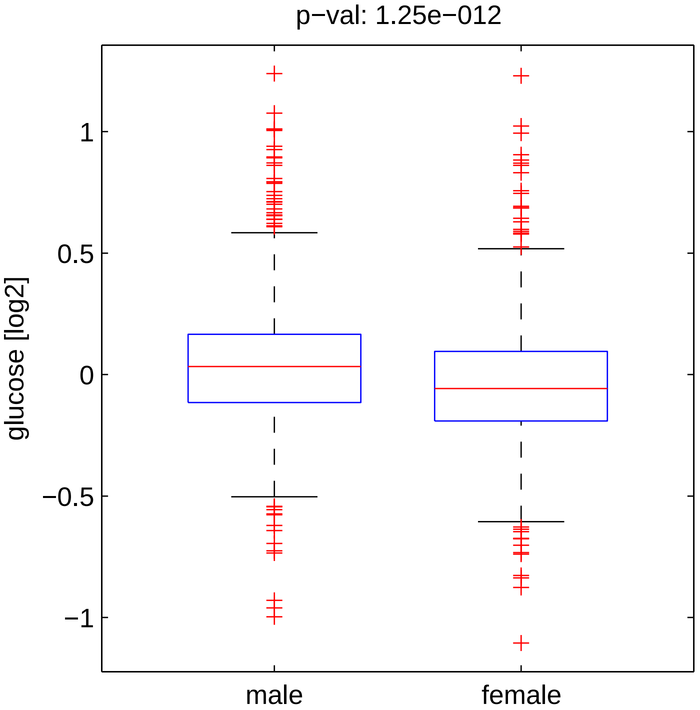

p−val: 1.25e−012
glucose [log2]
1
0.5
0
−0.5
−1
male
female

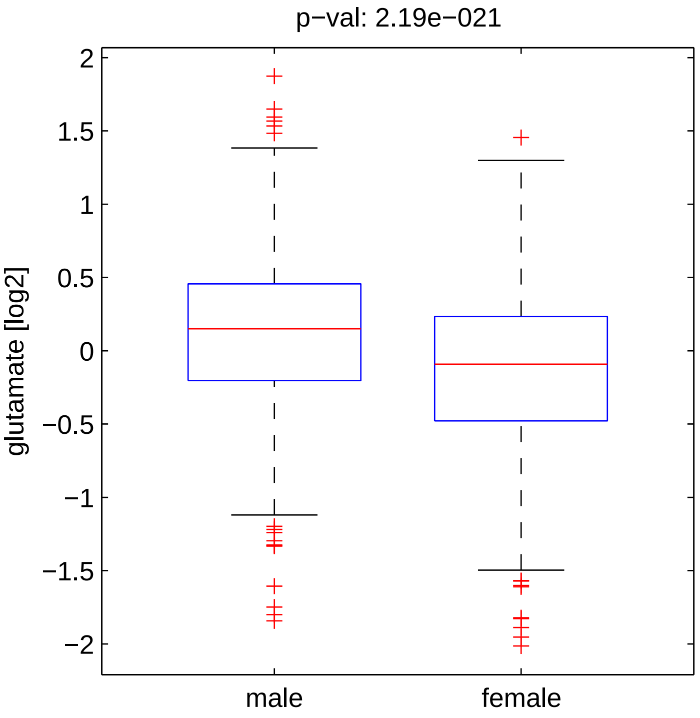
p−val: 2.19e−021
glutamate [log2]
2
1.5
1
0.5
0
−0.5
−1
−1.5
−2
male
female

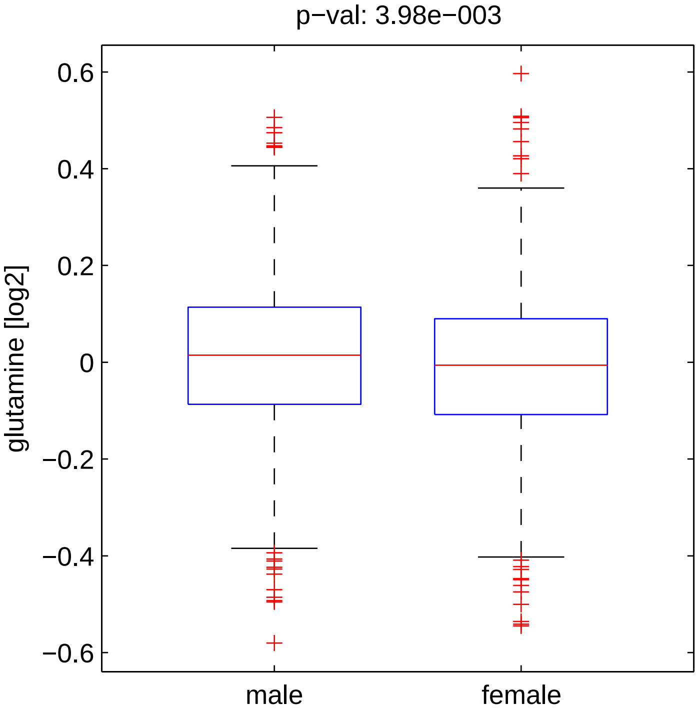

p−val: 3.98e−003
0.6
0.4
0.2
0
−0.2
−0.4
−0.6
glutamine [log2]
male
female

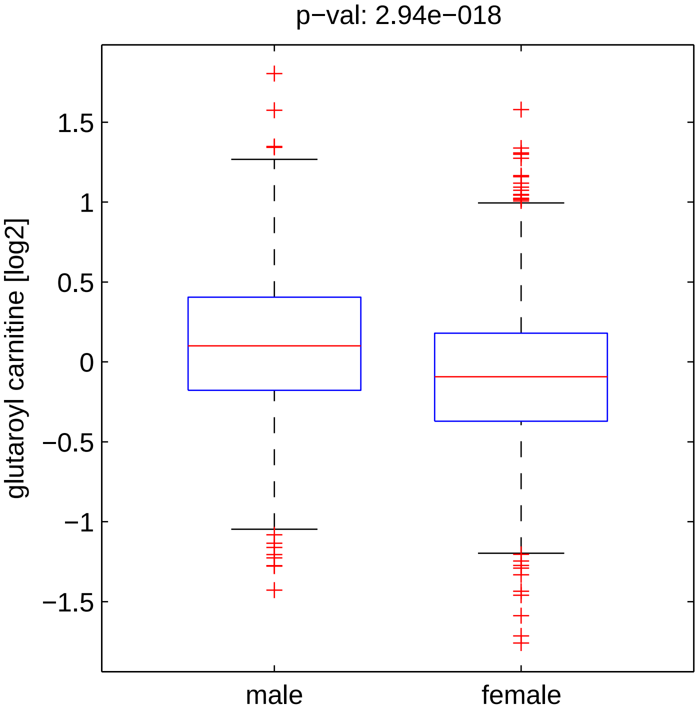
p−val: 2.94e−018
glutaroyl carnitine [log2]
1.5
1
0.5
0
−0.5
−1
−1.5
male
female

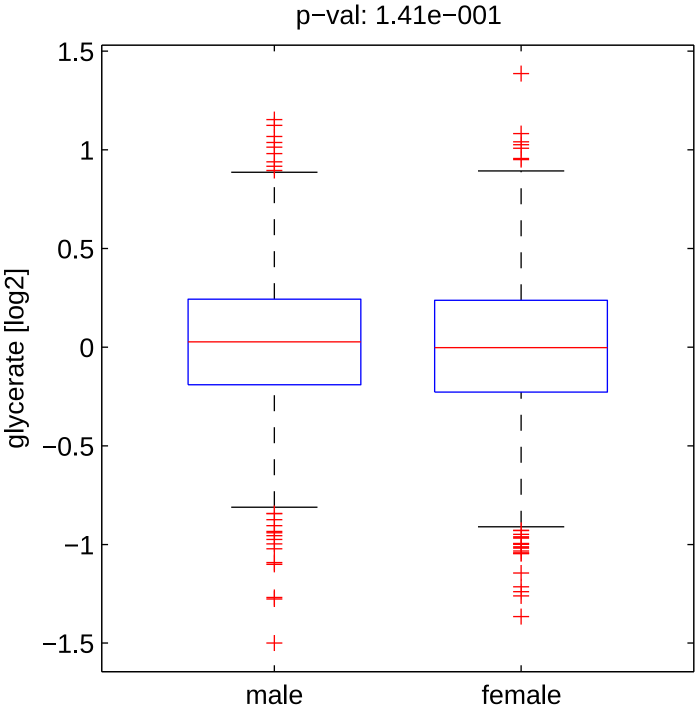

p−val: 1.41e−001
1.5
1
0.5
0
−0.5
−1
−1.5
glycerate [log2]
male
female

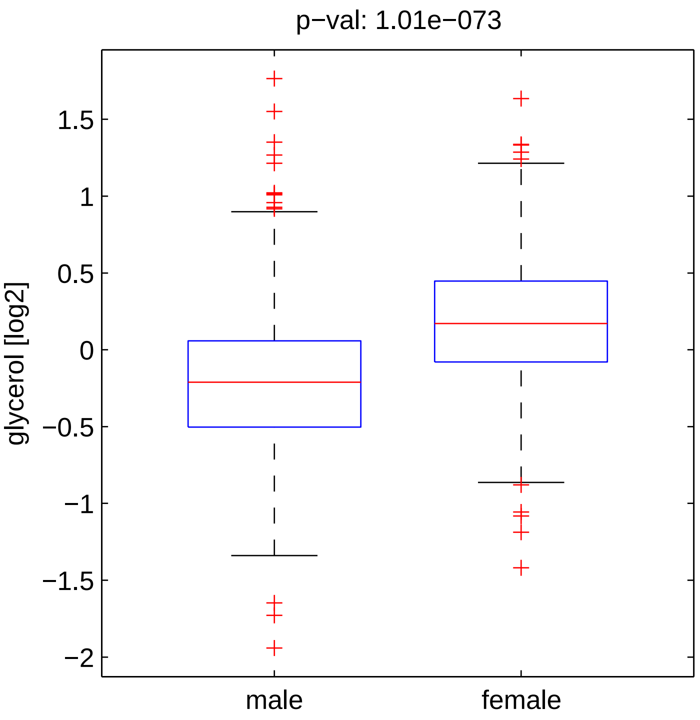
p−val: 1.01e−073
glycerol [log2]
1.5
1
0.5
0
−0.5
−1
−1.5
−2
male
female

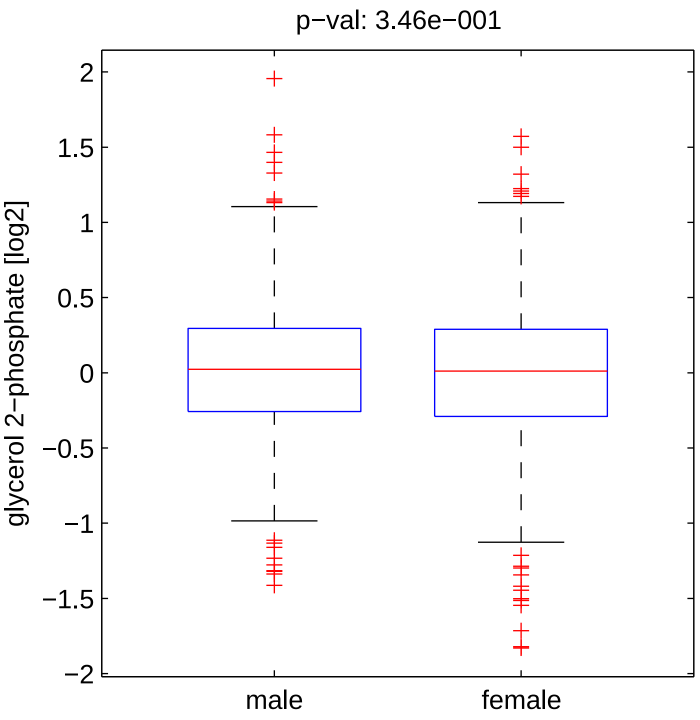
p−val: 3.46e−001
glycerol 2−phosphate [log2]
2
1.5
1
0.5
0
−0.5
−1
−1.5
−2
male
female

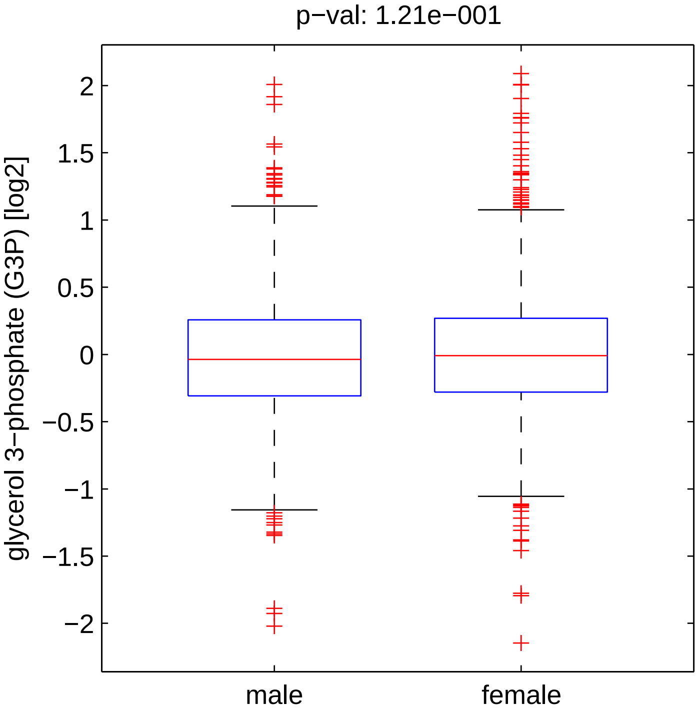
p−val: 1.21e−001
glycerol 3−phosphate (G3P) [log2]
2
1.5
1
0.5
0
−0.5
−1
−1.5
−2
male
female

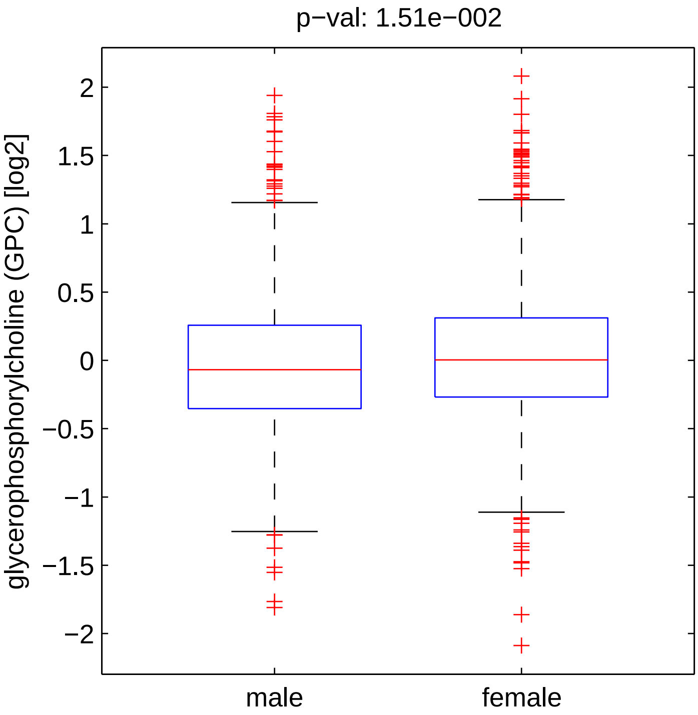
p−val: 1.51e−002
glycerophosphorylcholine (GPC) [log2]
2
1.5
1
0.5
0
−0.5
−1
−1.5
−2
male
female

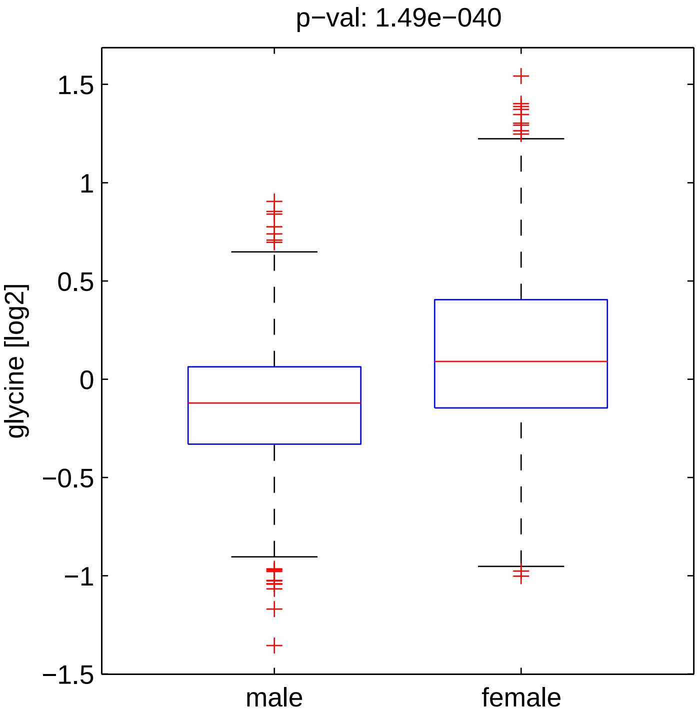
p−val: 1.49e−040
glycine [log2]
1.5
1
0.5
0
−0.5
−1
−1.5
male
female

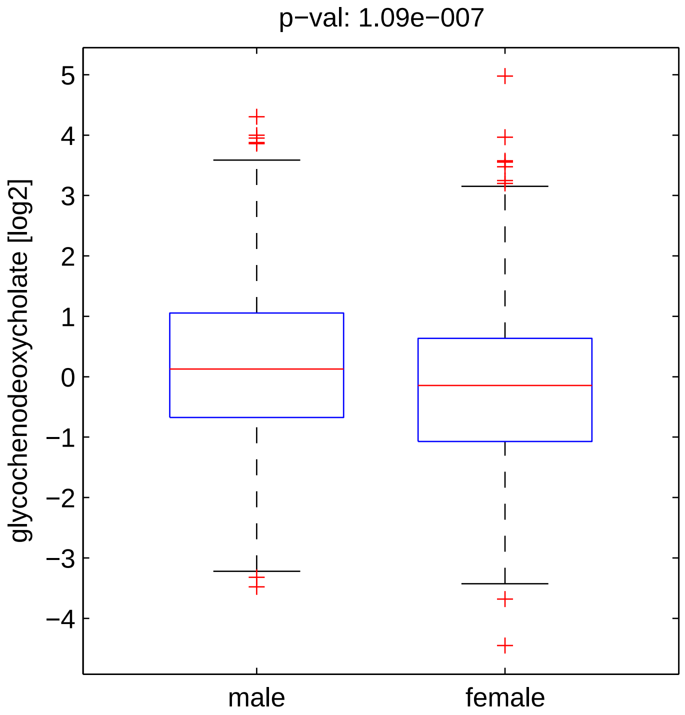

p−val: 1.09e−007
glycochenodeoxycholate [log2]
5
4
3
2
1
0
−1
−2
−3
−4
male
female

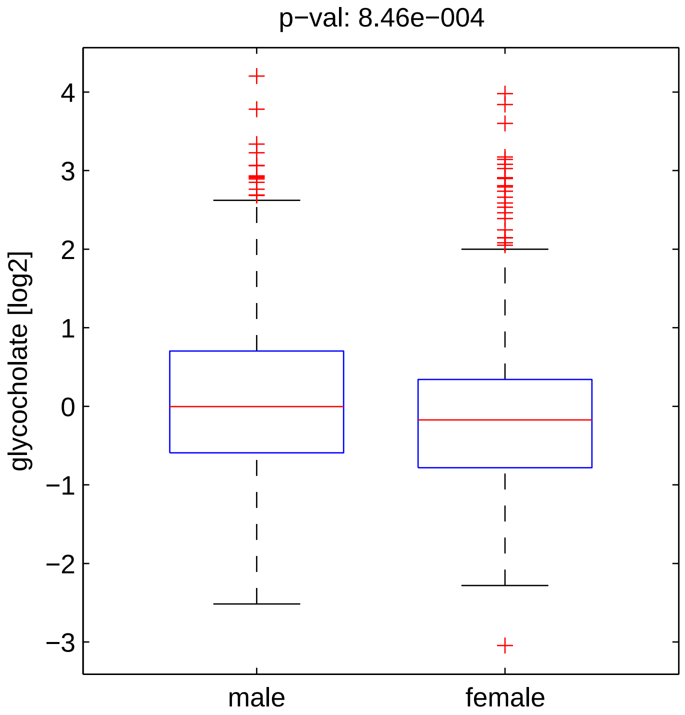

p−val: 8.46e−004
glycocholate [log2]
4
3
2
1
0
−1
−2
−3
male
female

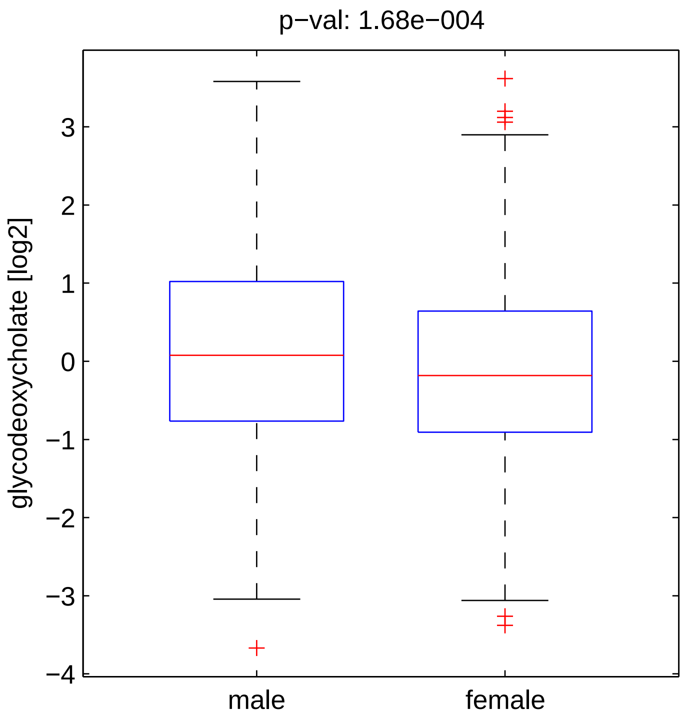
p−val: 1.68e−004
glycodeoxycholate [log2]
3
2
1
0
−1
−2
−3
−4
male
female

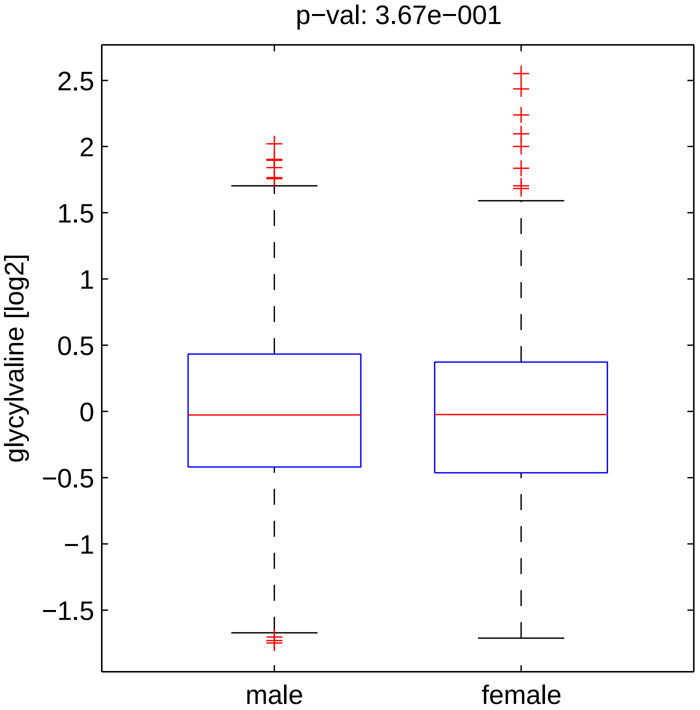
p−val: 3.67e−001
glycylvaline [log2]
2.5
2
1.5
1
0.5
0
−0.5
−1
−1.5
male
female

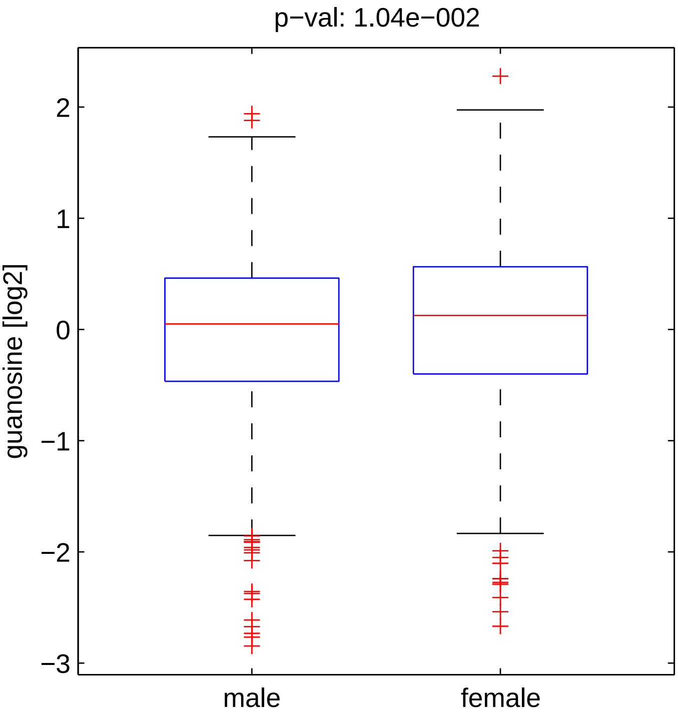
p−val: 1.04e−002
2
1
0
−1
−2
−3
guanosine [log2]
male
female

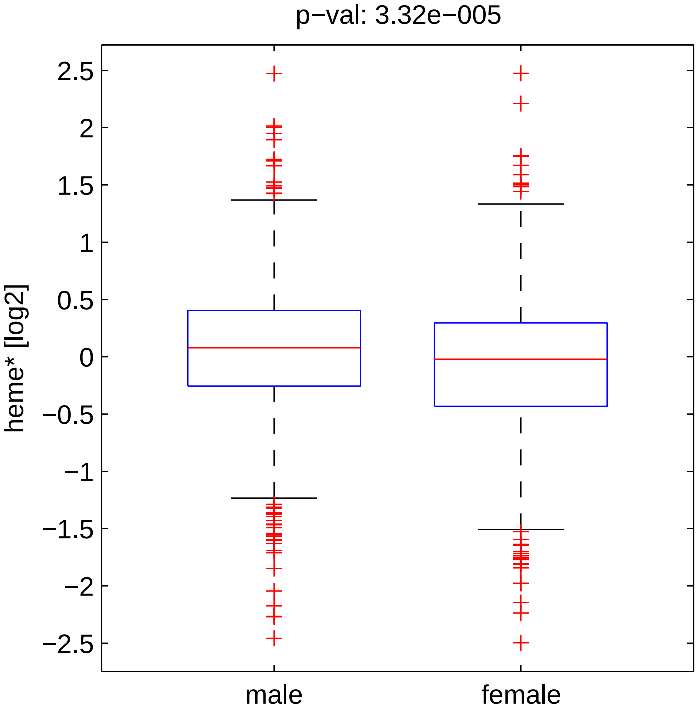

p−val: 3.32e−005
heme* [log2]
2.5
2
1.5
1
0.5
0
−0.5
−1
−1.5
−2
−2.5
male
female

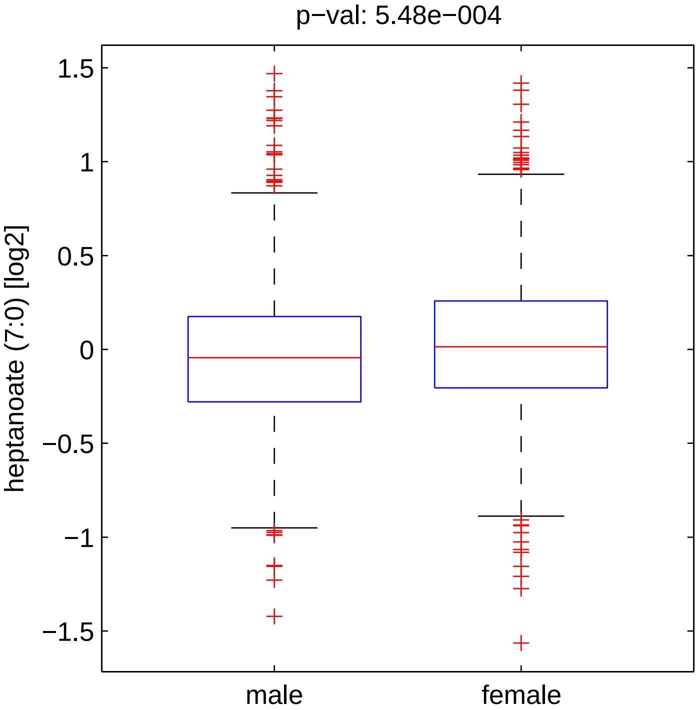
p−val: 5.48e−004
heptanoate (7:0) [log2]
1.5
1
0.5
0
−0.5
−1
−1.5
male
female

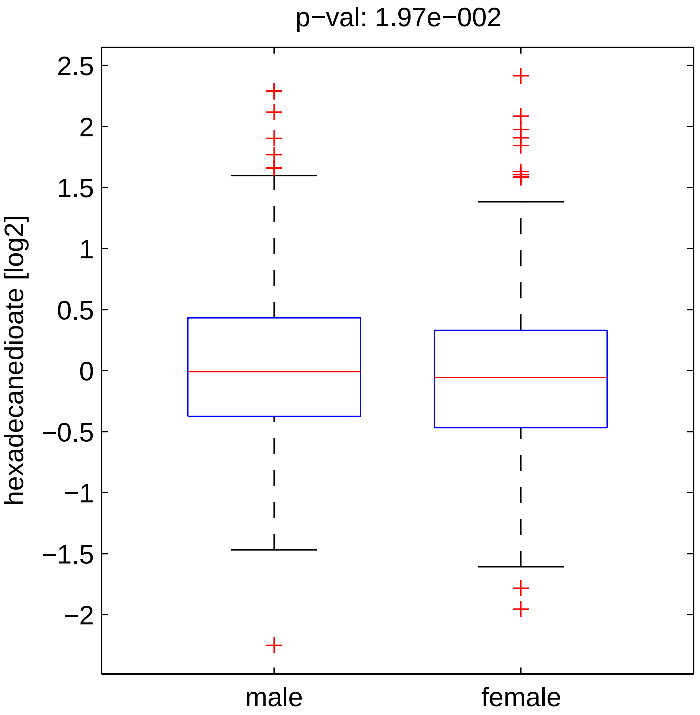

p−val: 1.97e−002
hexadecanedioate [log2]
2.5
2
1.5
1
0.5
0
−0.5
−1
−1.5
−2
male
female

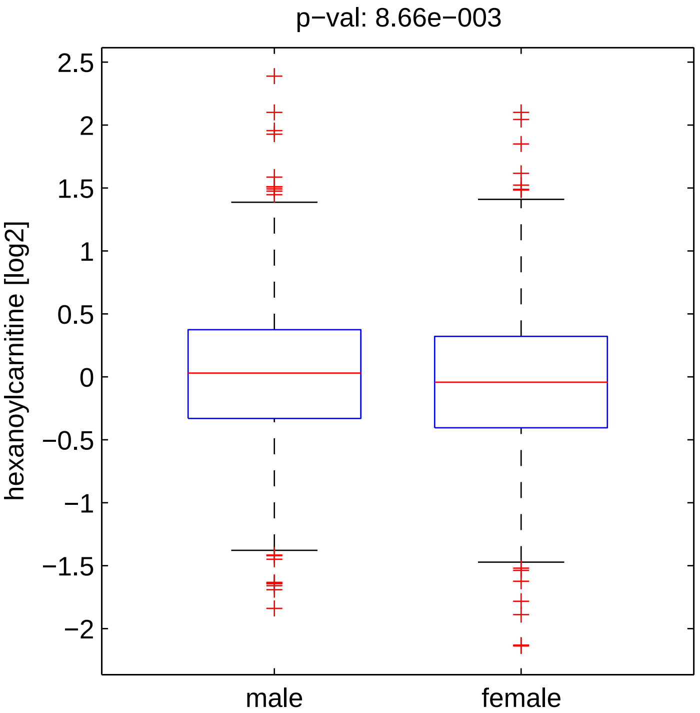

p−val: 8.66e−003
2.5
2
1.5
1
0.5
0
−0.5
−1
−1.5
−2
hexanoylcarnitine [log2]
male
female

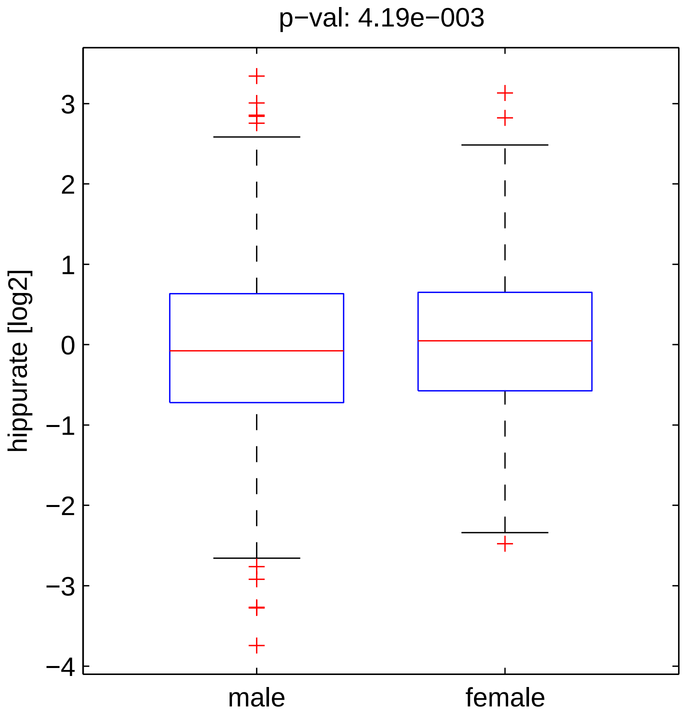

p−val: 4.19e−003
hippurate [log2]
3
2
1
0
−1
−2
−3
−4
male
female

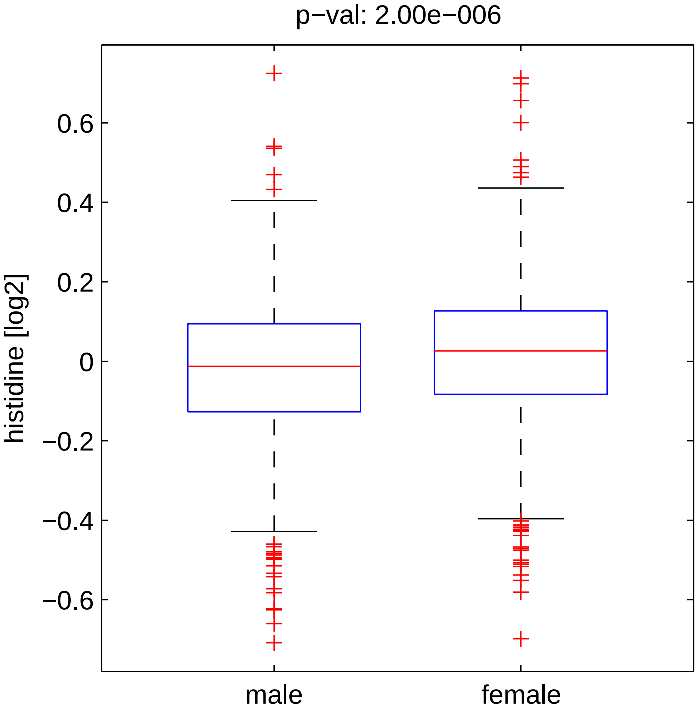

p−val: 2.00e−006
histidine [log2]
0.6
0.4
0.2
0
−0.2
−0.4
−0.6
male
female

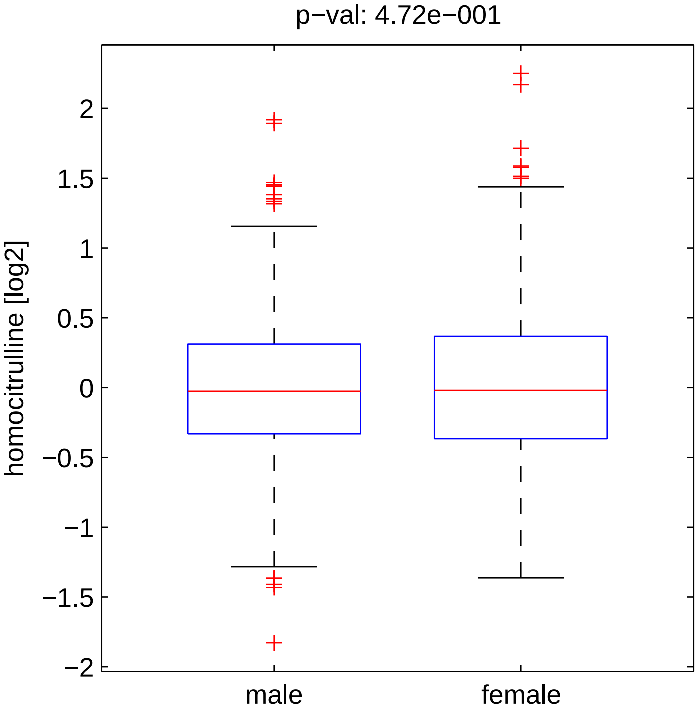
p−val: 4.72e−001
homocitrulline [log2]
2
1.5
1
0.5
0
−0.5
−1
−1.5
−2
male
female

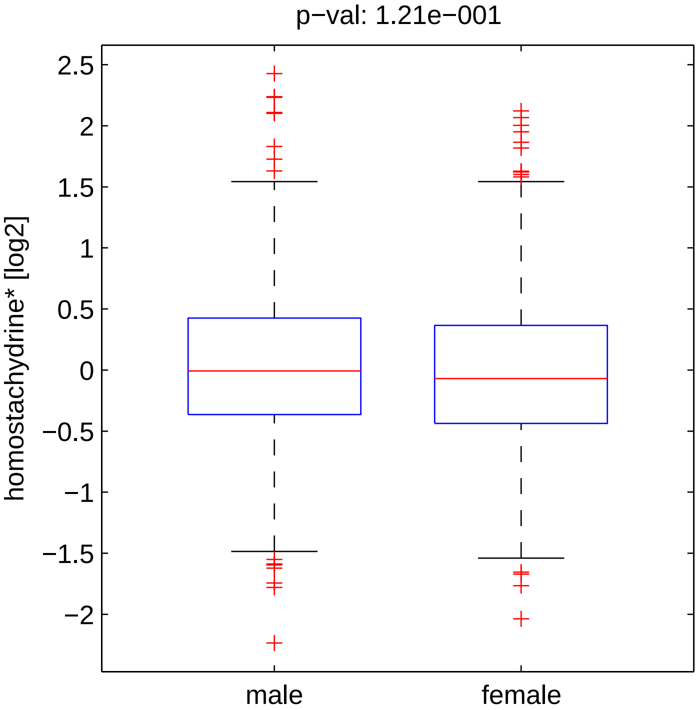

p−val: 1.21e−001
homostachydrine* [log2]
2.5
2
1.5
1
0.5
0
−0.5
−1
−1.5
−2
male
female

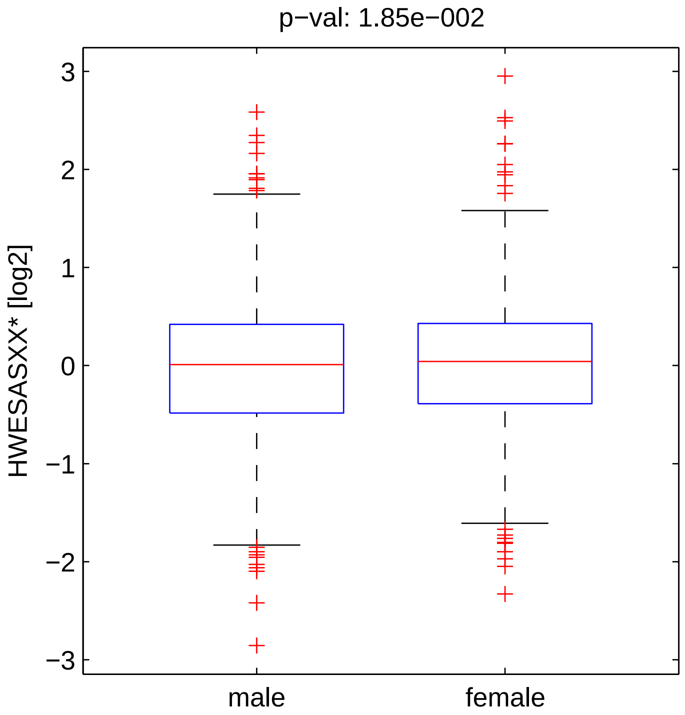
p−val: 1.85e−002
HWESASXX* [log2]
3
2
1
0
−1
−2
−3
male
female

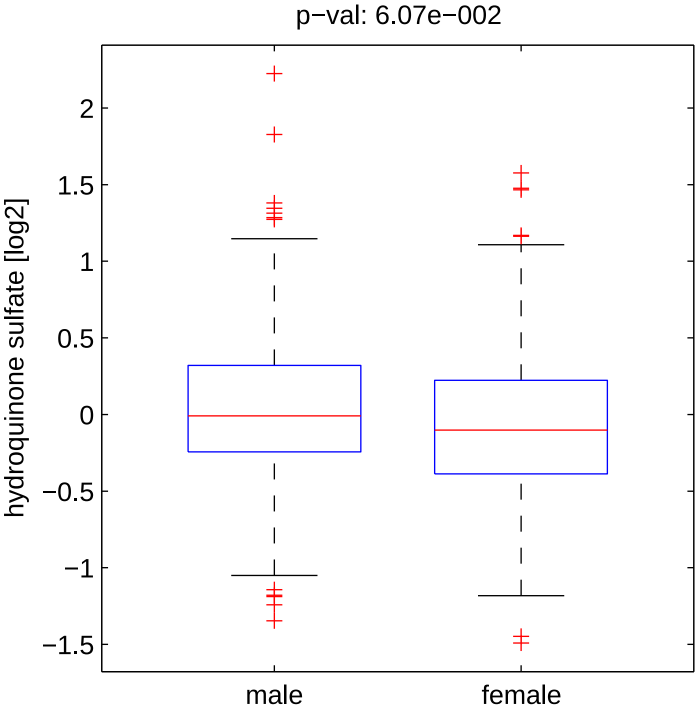
p−val: 6.07e−002
hydroquinone sulfate [log2]
2
1.5
1
0.5
0
−0.5
−1
−1.5
male
female

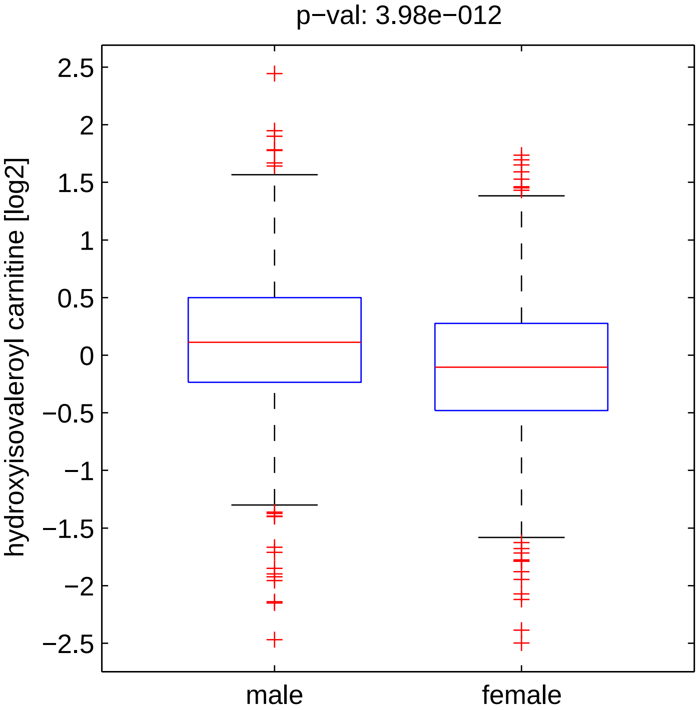

p−val: 3.98e−012
hydroxyisovaleroyl carnitine [log2]
2.5
2
1.5
1
0.5
0
−0.5
−1
−1.5
−2
−2.5
male
female

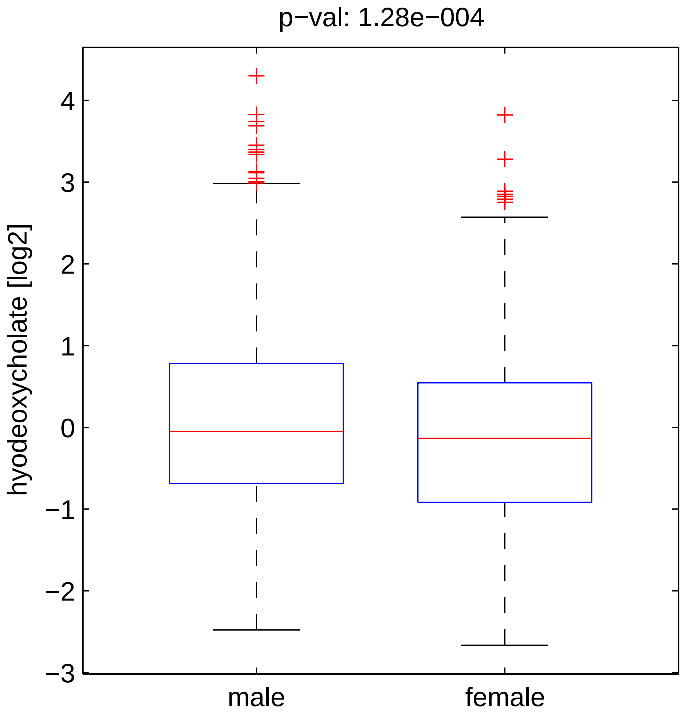
p−val: 1.28e−004
hyodeoxycholate [log2]
4
3
2
1
0
−1
−2
−3
male
female

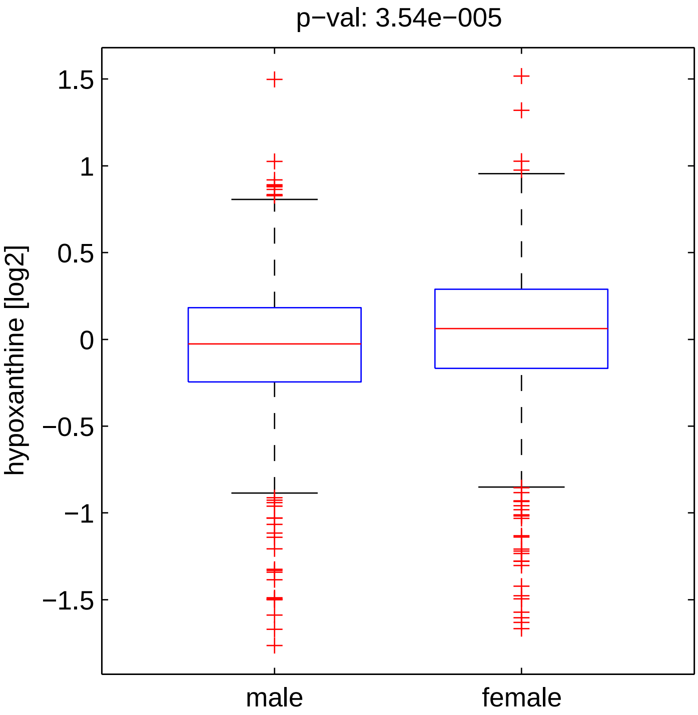
p−val: 3.54e−005
hypoxanthine [log2]
1.5
1
0.5
0
−0.5
−1
−1.5
male
female

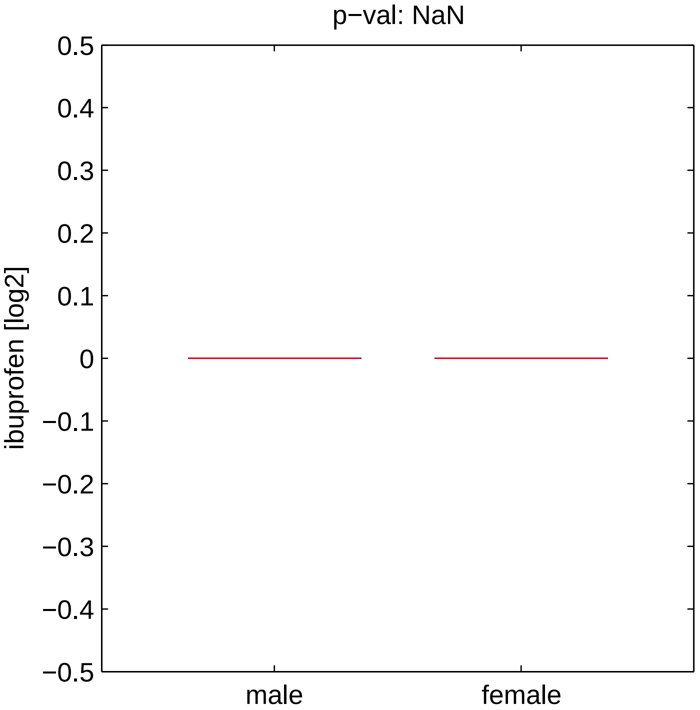
p−val: NaN
ibuprofen [log2]
0.5
0.4
0.3
0.2
0.1
0
−0.1
−0.2
−0.3
−0.4
−0.5
male
female

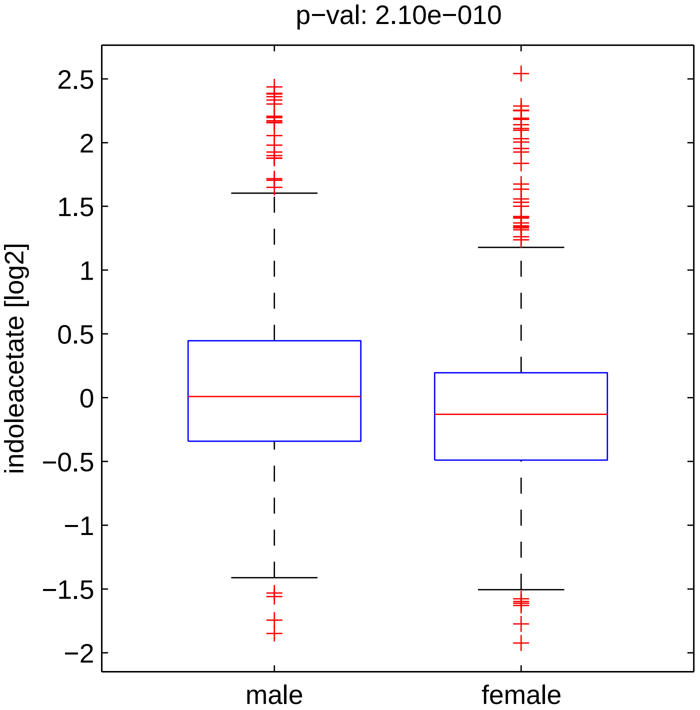
p−val: 2.10e−010
indoleacetate [log2]
2.5
2
1.5
1
0.5
0
−0.5
−1
−1.5
−2
male
female

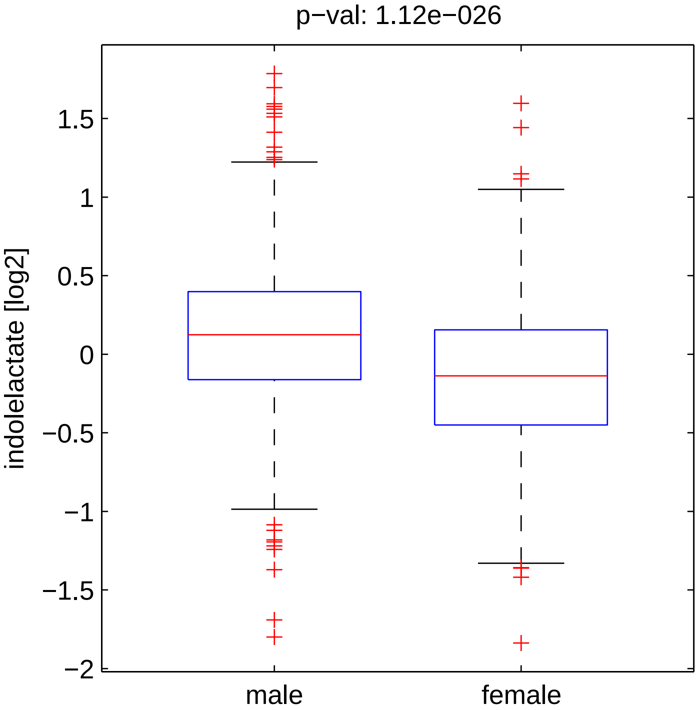

p−val: 1.12e−026
indolelactate [log2]
1.5
1
0.5
0
−0.5
−1
−1.5
−2
male
female

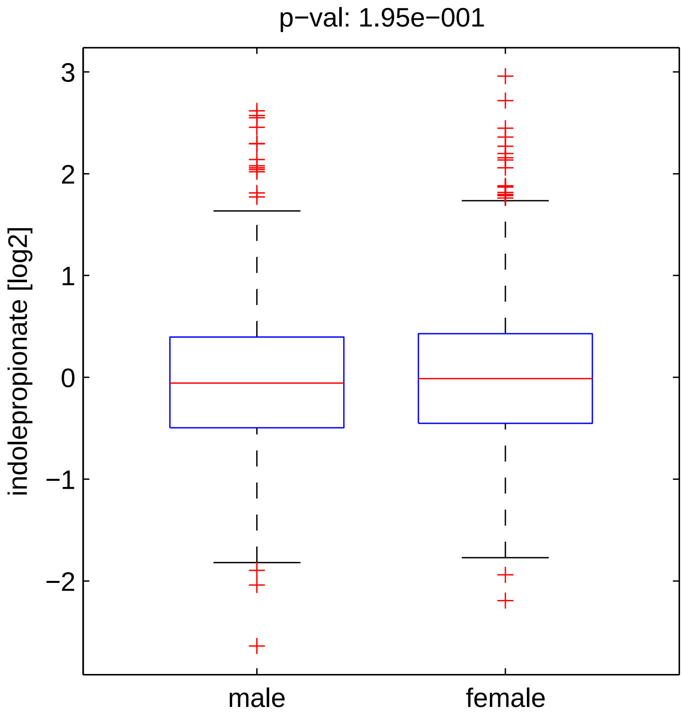
p−val: 1.95e−001
indolepropionate [log2]
3
2
1
0
−1
−2
male
female

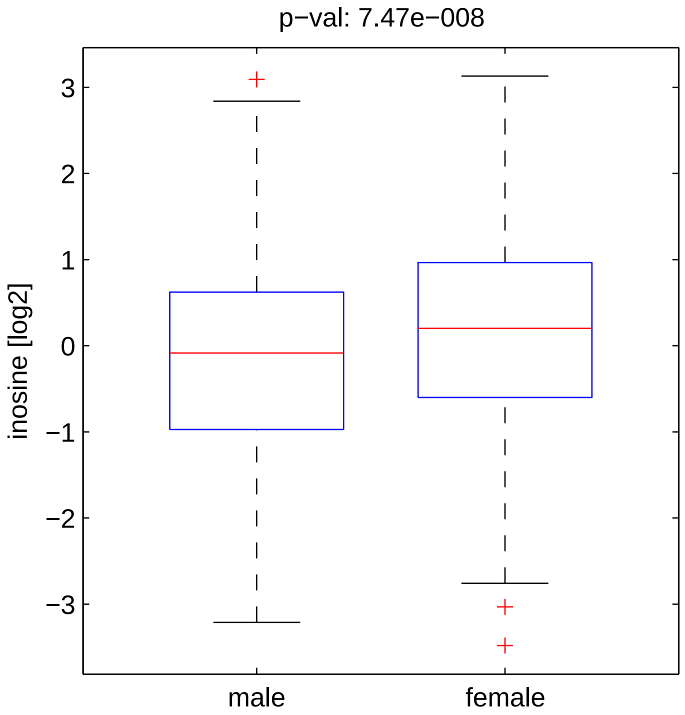

p−val: 7.47e−008
inosine [log2]
3
2
1
0
−1
−2
−3
male
female

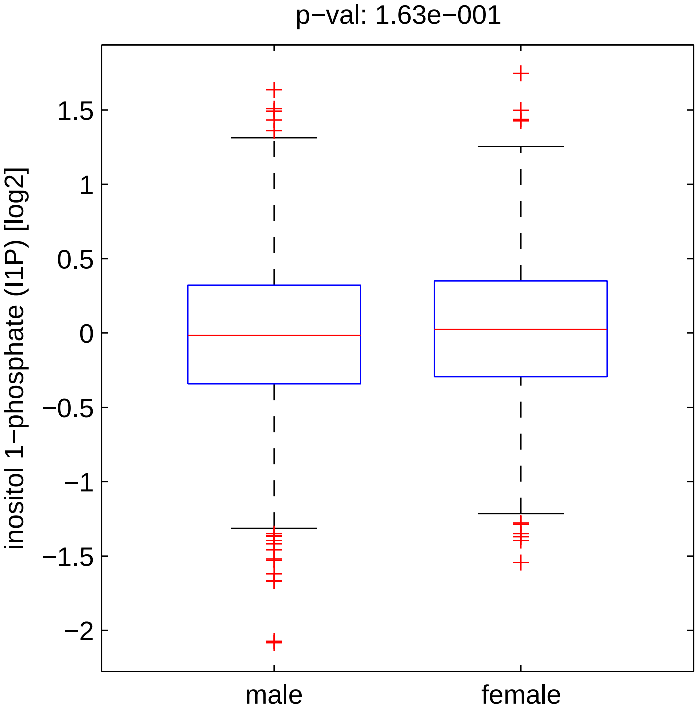

p−val: 1.63e−001
inositol 1−phosphate (I1P) [log2]
1.5
1
0.5
0
−0.5
−1
−1.5
−2
male
female

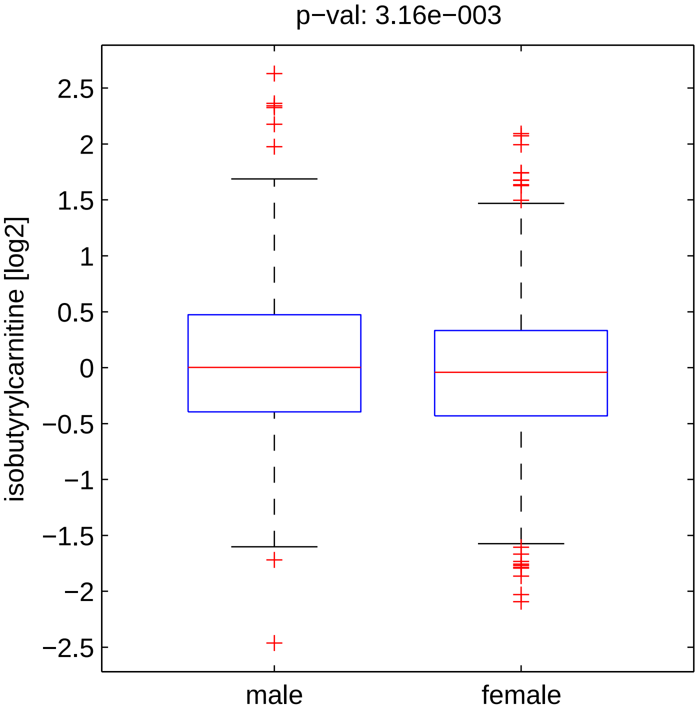
p−val: 3.16e−003
isobutyrylcarnitine [log2]
2.5
2
1.5
1
0.5
0
−0.5
−1
−1.5
−2
−2.5
male
female

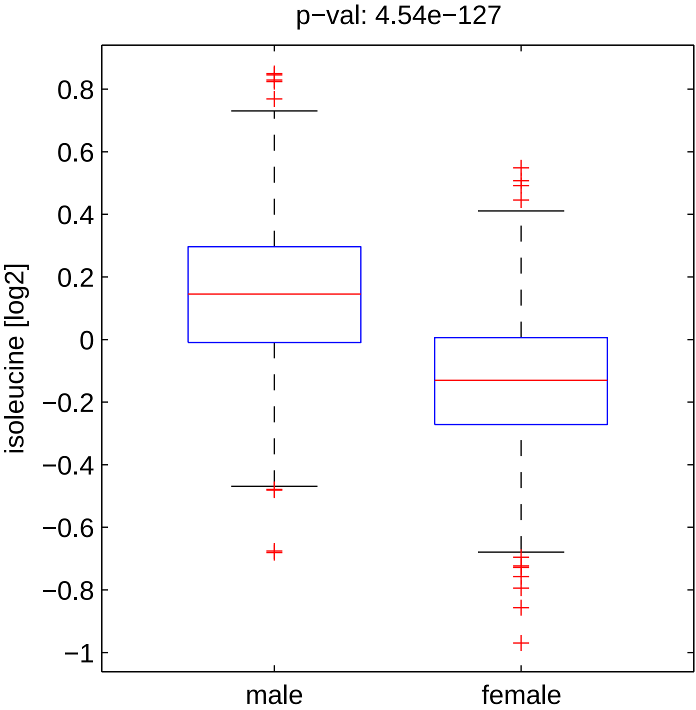
p−val: 4.54e−127
0.8
0.6
0.4
0.2
0
−0.2
−0.4
−0.6
−0.8
−1
isoleucine [log2]
male
female

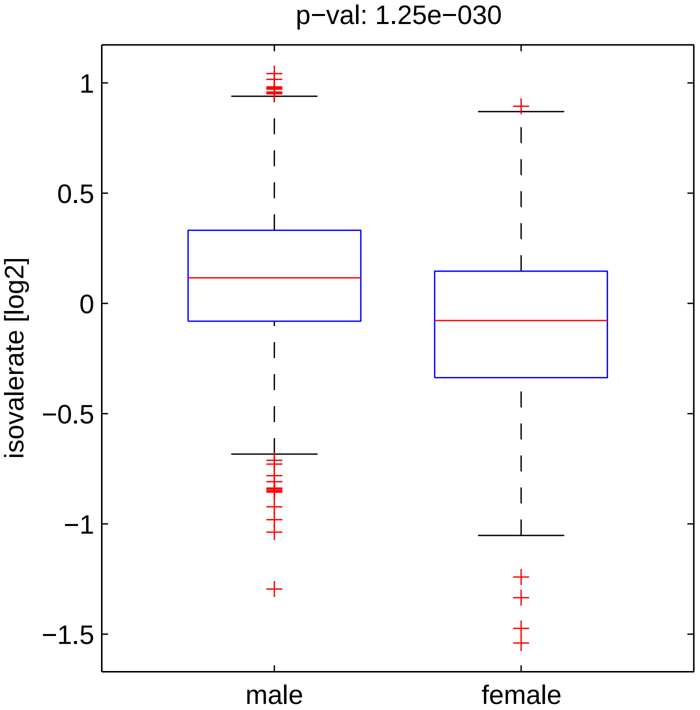

p−val: 1.25e−030
isovalerate [log2]
1
0.5
0
−0.5
−1
−1.5
male
female

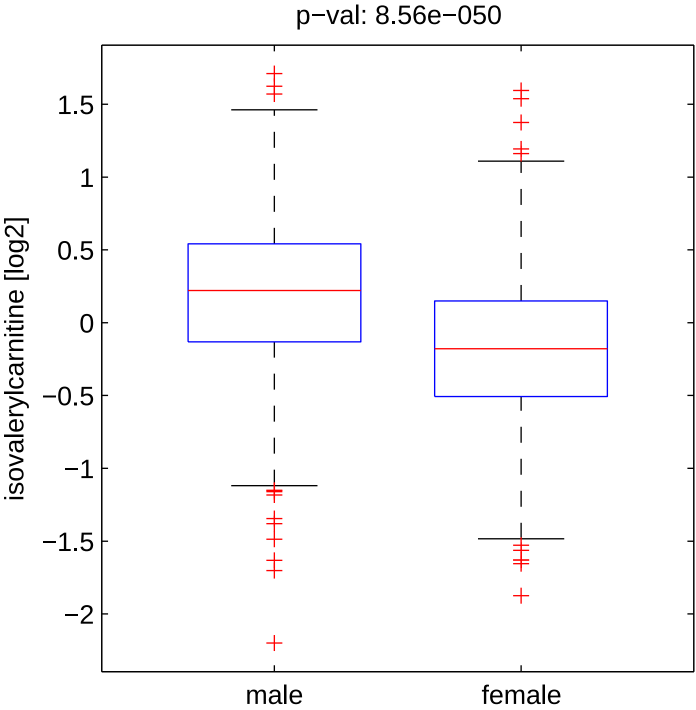

p−val: 8.56e−050
isovalerylcarnitine [log2]
1.5
1
0.5
0
−0.5
−1
−1.5
−2
male
female

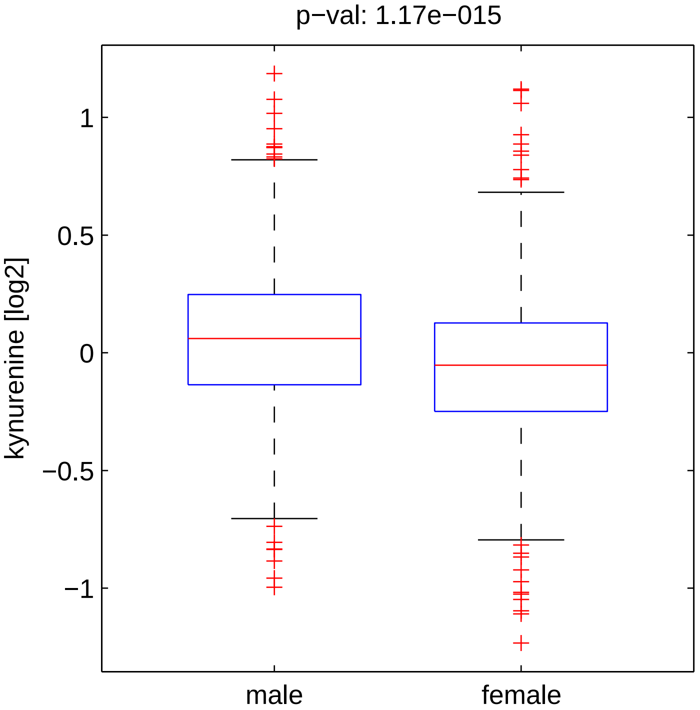
p−val: 1.17e−015
kynurenine [log2]
1
0.5
0
−0.5
−1
male
female

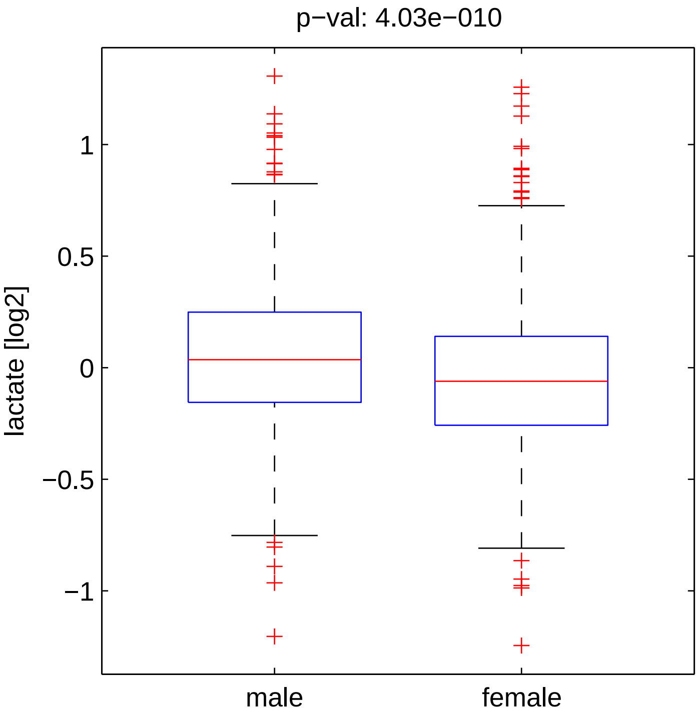
p−val: 4.03e−010
1
0.5
0
−0.5
−1
lactate [log2]
male
female

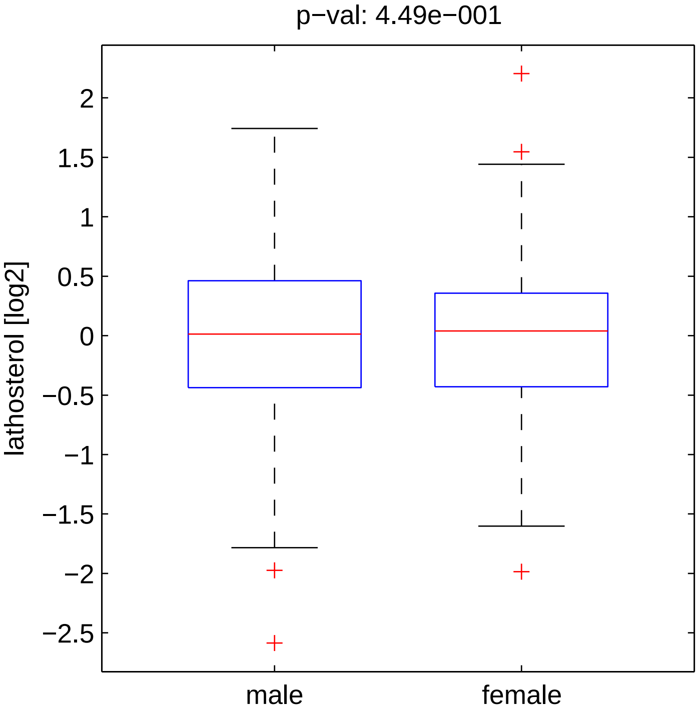

p−val: 4.49e−001
lathosterol [log2]
2
1.5
1
0.5
0
−0.5
−1
−1.5
−2
−2.5
male
female

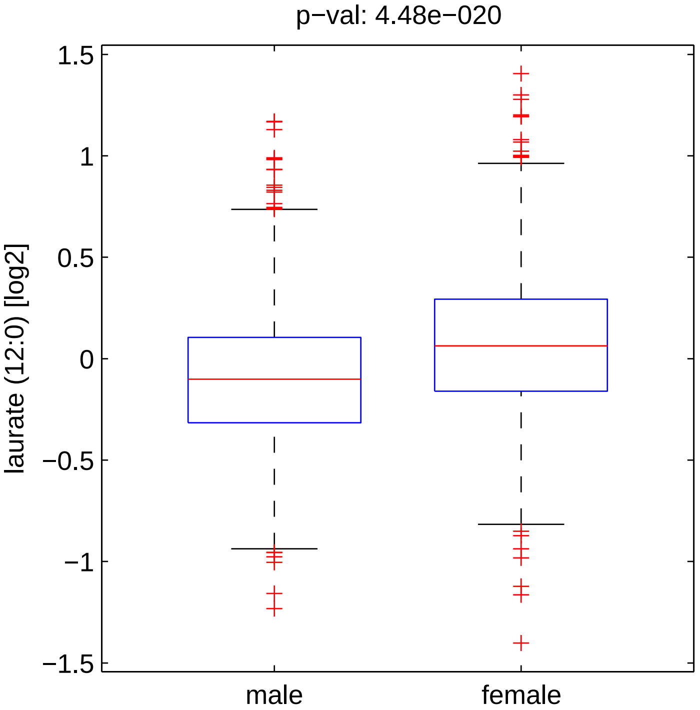

p−val: 4.48e−020
laurate (12:0) [log2]
1.5
1
0.5
0
−0.5
−1
−1.5
male
female

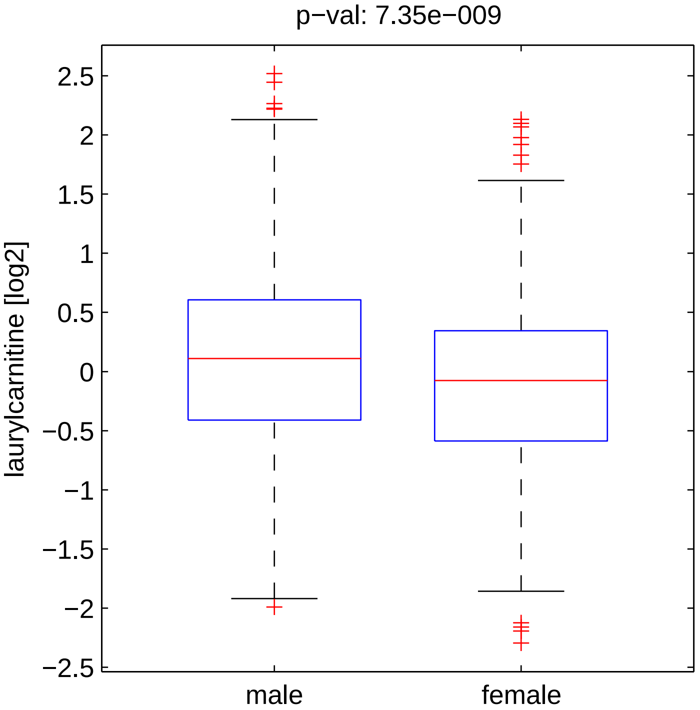

p−val: 7.35e−009
laurylcarnitine [log2]
2.5
2
1.5
1
0.5
0
−0.5
−1
−1.5
−2
−2.5
male
female

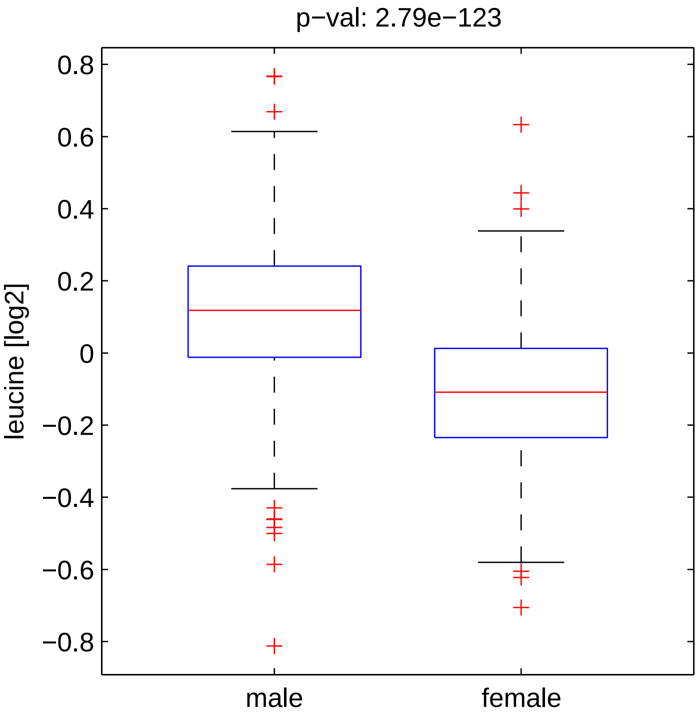
p−val: 2.79e−123
leucine [log2]
0.8
0.6
0.4
0.2
0
−0.2
−0.4
−0.6
−0.8
male
female

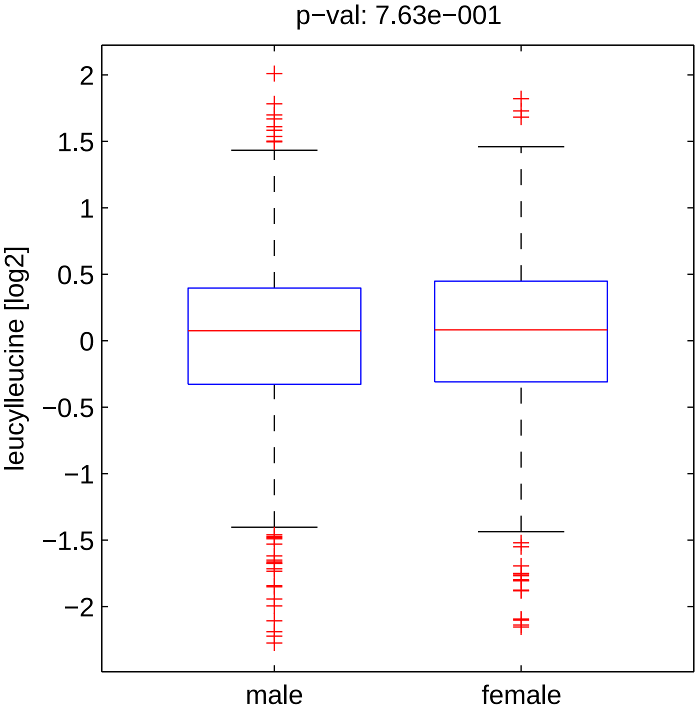
p−val: 7.63e−001
leucylleucine [log2]
2
1.5
1
0.5
0
−0.5
−1
−1.5
−2
male
female

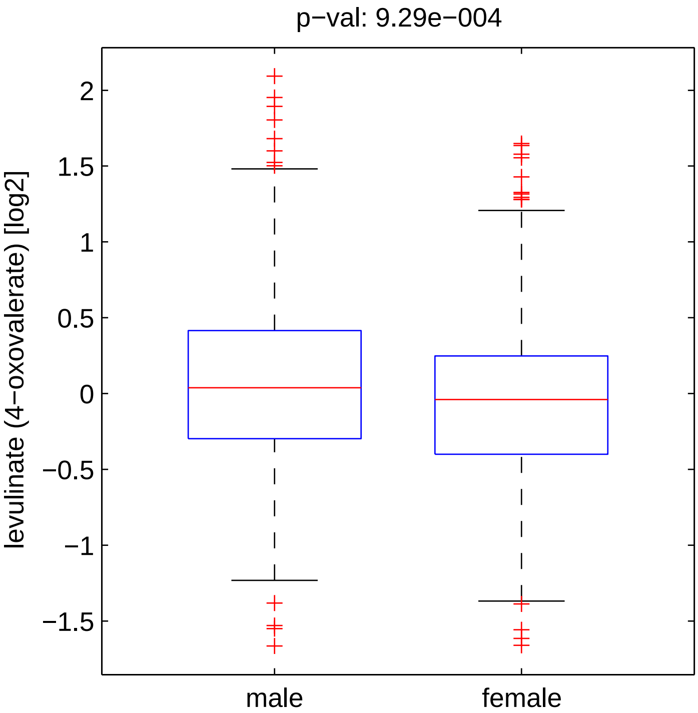

p−val: 9.29e−004
levulinate (4−oxovalerate) [log2]
2
1.5
1
0.5
0
−0.5
−1
−1.5
male
female

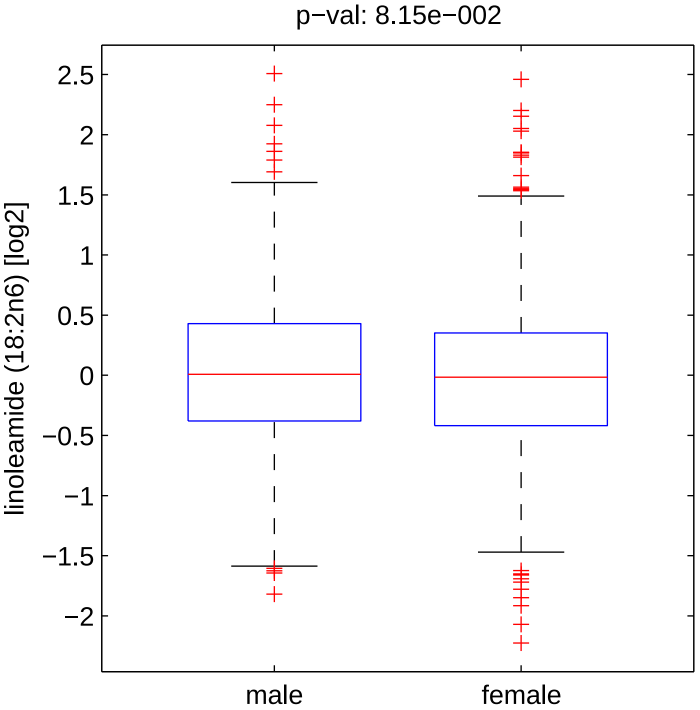
p−val: 8.15e−002
linoleamide (18:2n6) [log2]
2.5
2
1.5
1
0.5
0
−0.5
−1
−1.5
−2
male
female

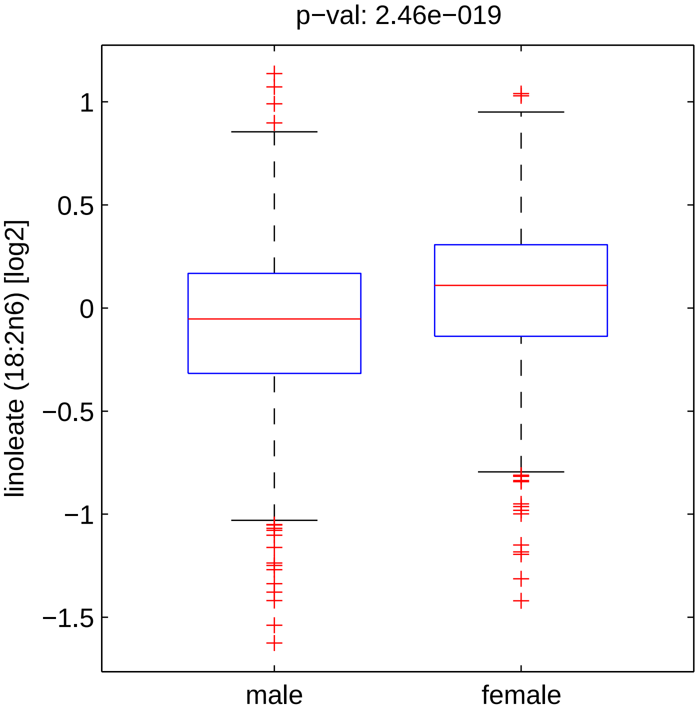
p−val: 2.46e−019
linoleate (18:2n6) [log2]
1
0.5
0
−0.5
−1
−1.5
male
female

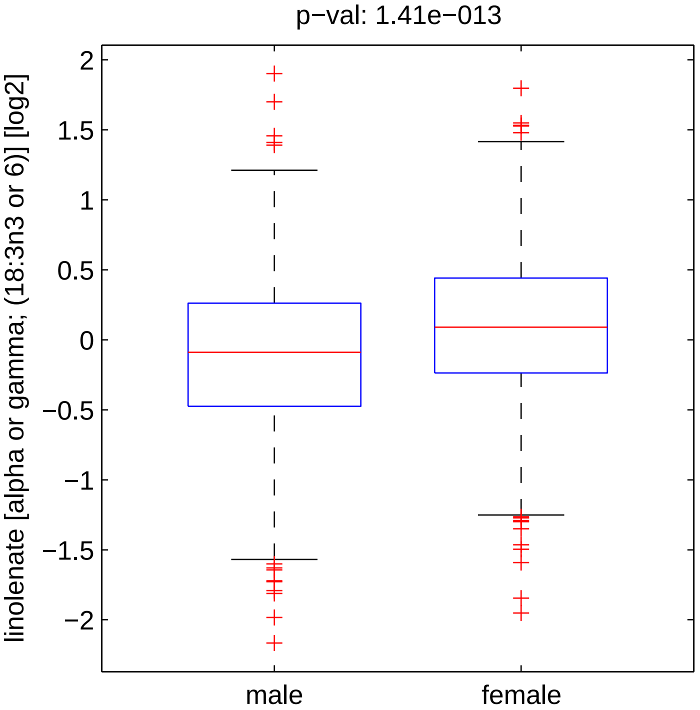
p−val: 1.41e−013
linolenate [alpha or gamma; (18:3n3 or 6)] [log2]
2
1.5
1
0.5
0
−0.5
−1
−1.5
−2
male
female

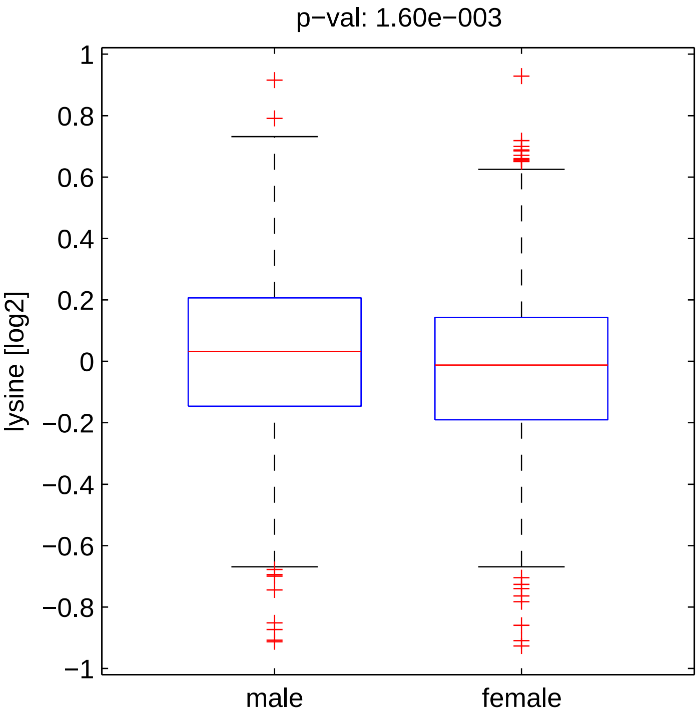

p−val: 1.60e−003
lysine [log2]
1
0.8
0.6
0.4
0.2
0
−0.2
−0.4
−0.6
−0.8
−1
male
female

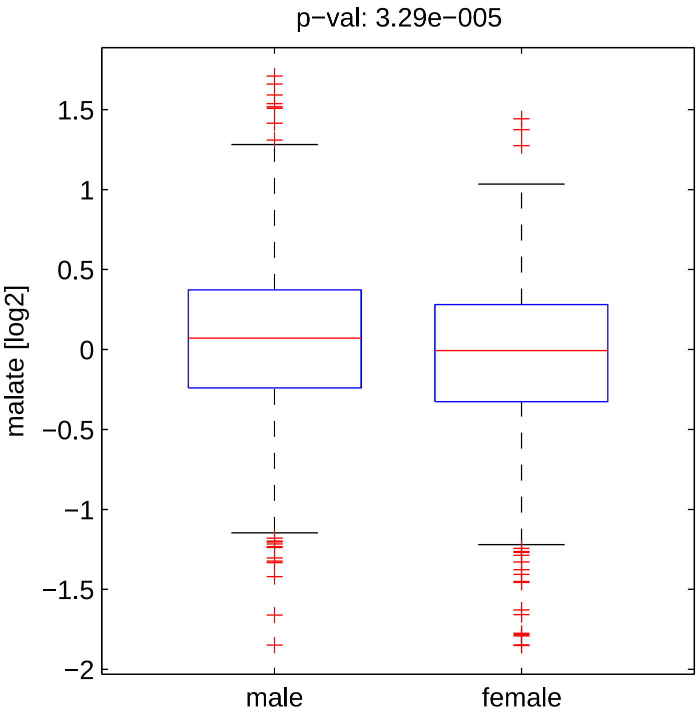

p−val: 3.29e−005
malate [log2]
1.5
1
0.5
0
−0.5
−1
−1.5
−2
male
female

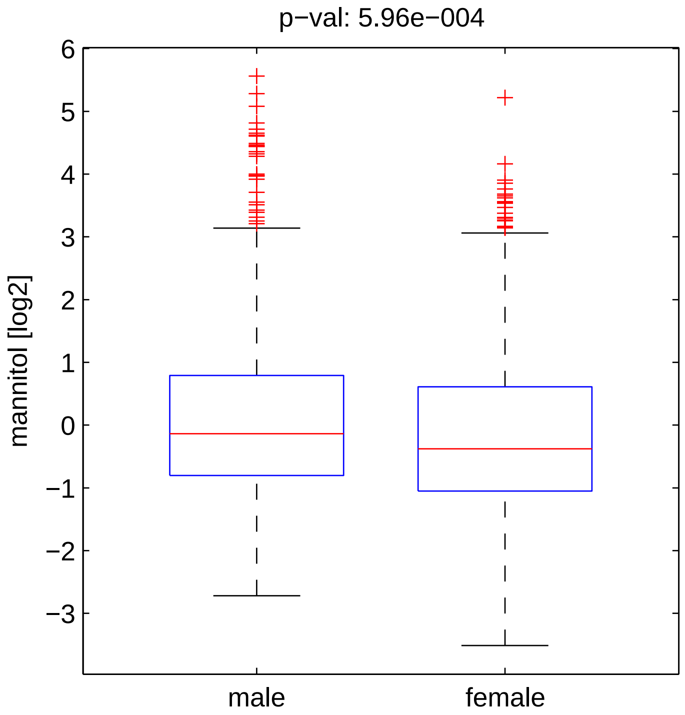

p−val: 5.96e−004
6
5
4
3
2
1
0
−1
−2
−3
mannitol [log2]
male
female

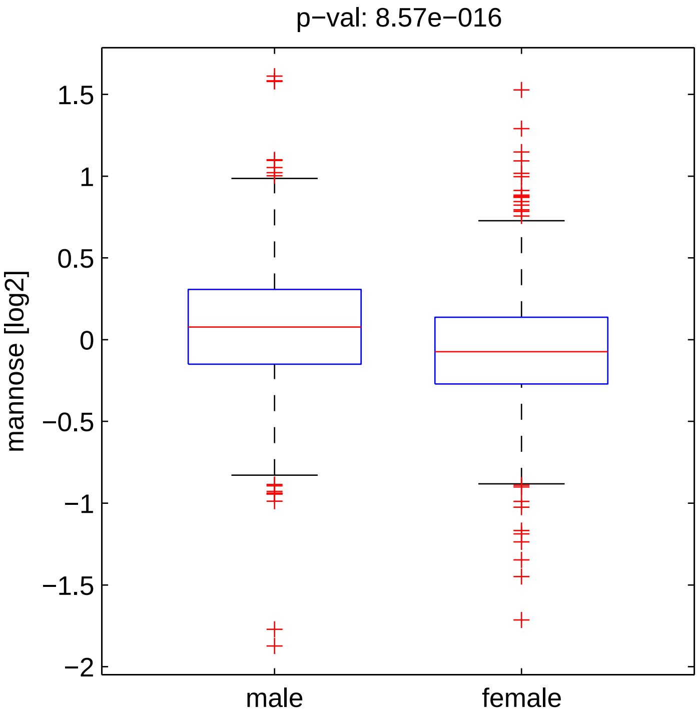
p−val: 8.57e−016
mannose [log2]
1.5
1
0.5
0
−0.5
−1
−1.5
−2
male
female

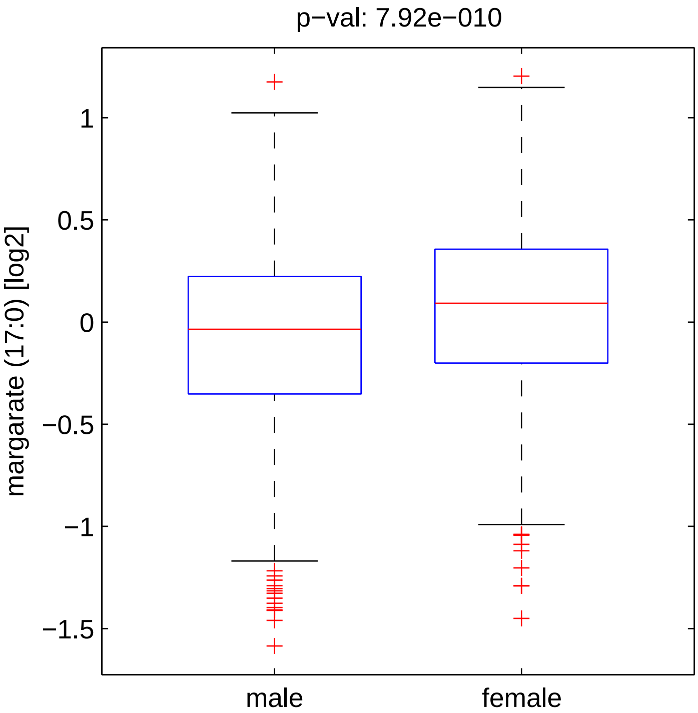
p−val: 7.92e−010
margarate (17:0) [log2]
1
0.5
0
−0.5
−1
−1.5
male
female

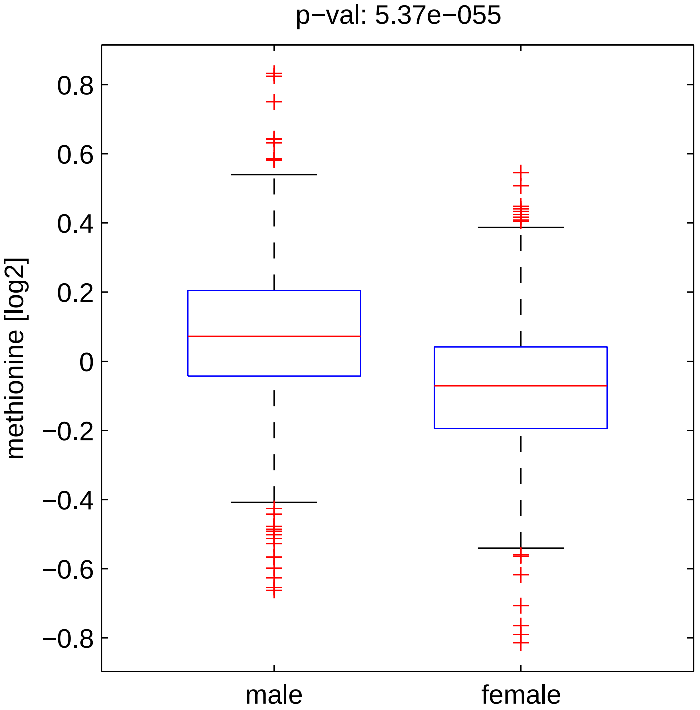
p−val: 5.37e−055
methionine [log2]
0.8
0.6
0.4
0.2
0
−0.2
−0.4
−0.6
−0.8
male
female

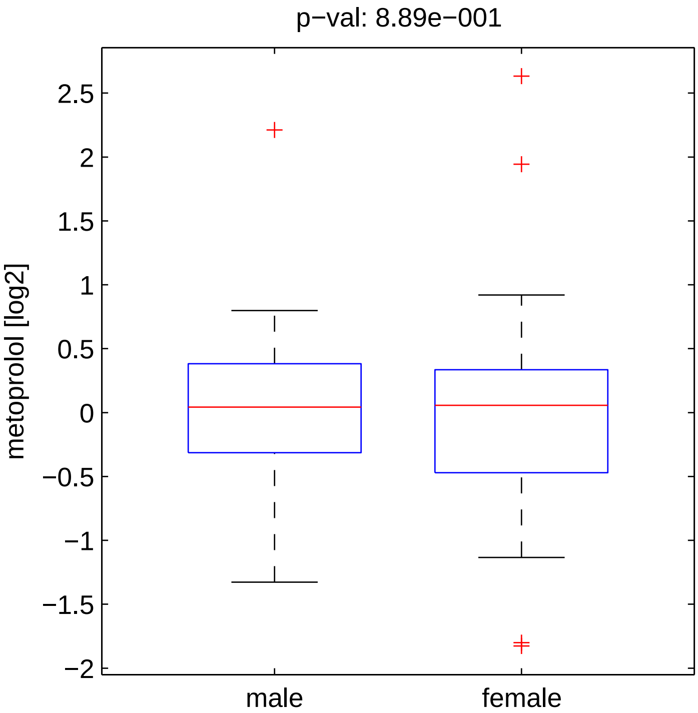
p−val: 8.89e−001
metoprolol [log2]
2.5
2
1.5
1
0.5
0
−0.5
−1
−1.5
−2
male
female

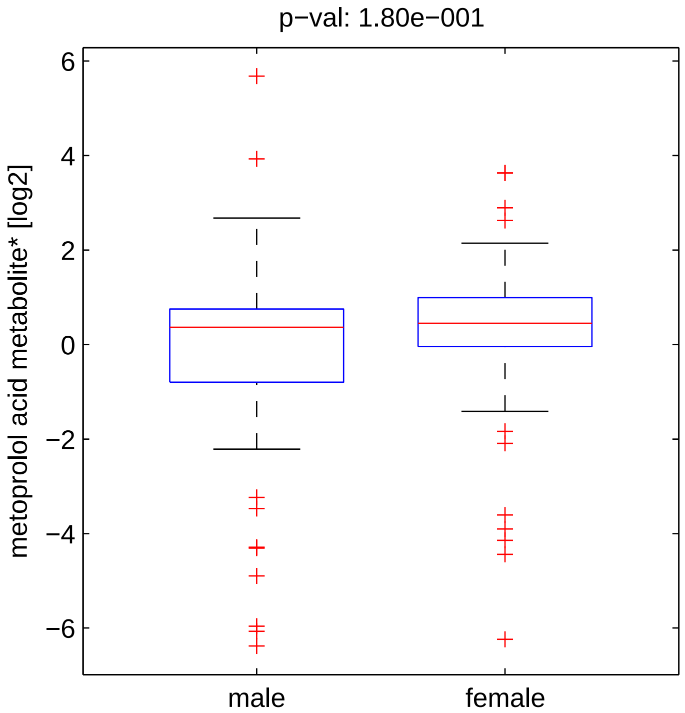
p−val: 1.80e−001
metoprolol acid metabolite* [log2]
6
4
2
0
−2
−4
−6
male
female

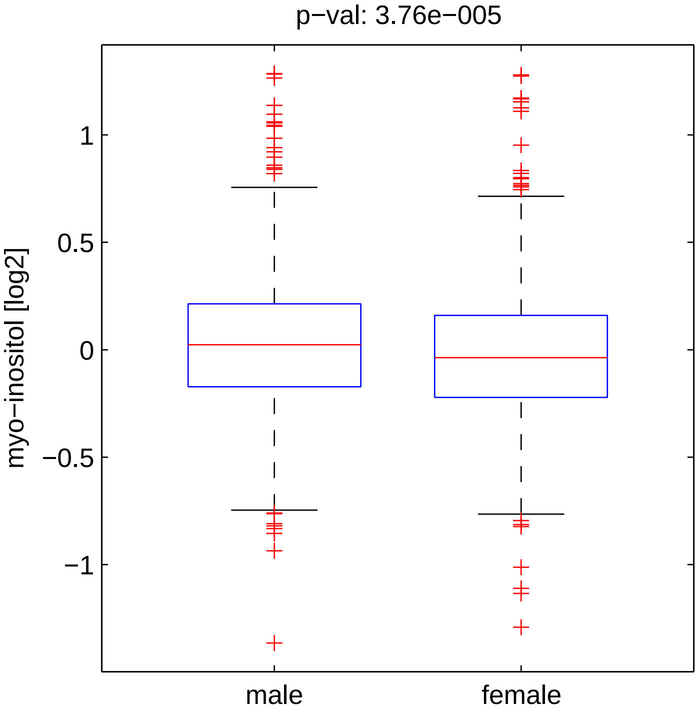

p−val: 3.76e−005
myo−inositol [log2]
1
0.5
0
−0.5
−1
male
female

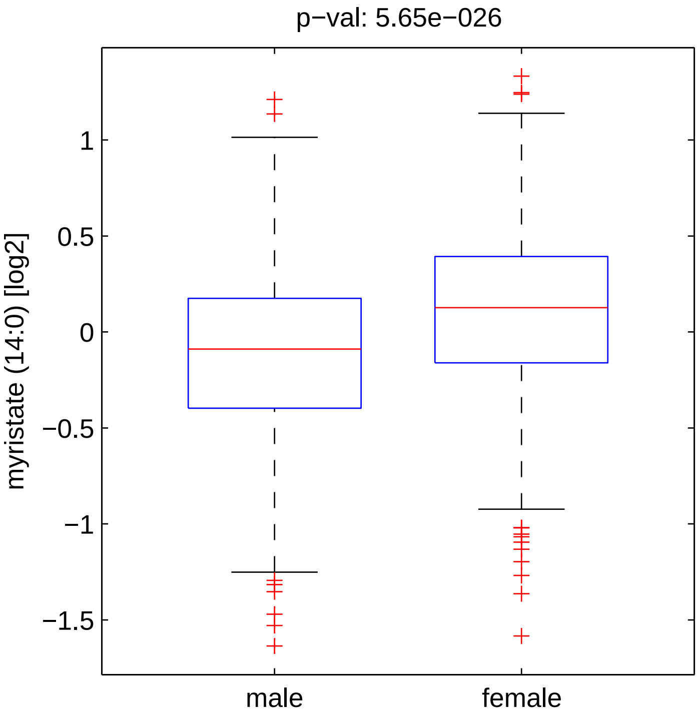
p−val: 5.65e−026
myristate (14:0) [log2]
1
0.5
0
−0.5
−1
−1.5
male
female

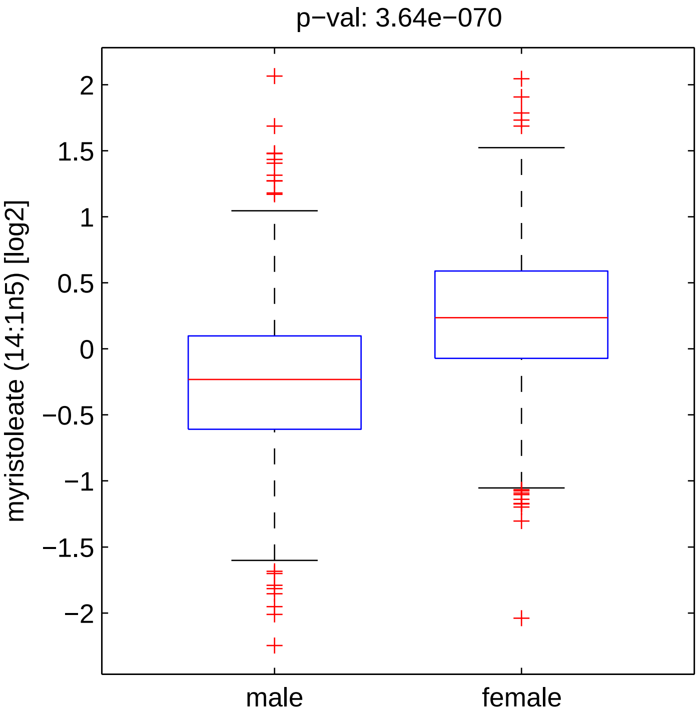
p−val: 3.64e−070
myristoleate (14:1n5) [log2]
2
1.5
1
0.5
0
−0.5
−1
−1.5
−2
male
female

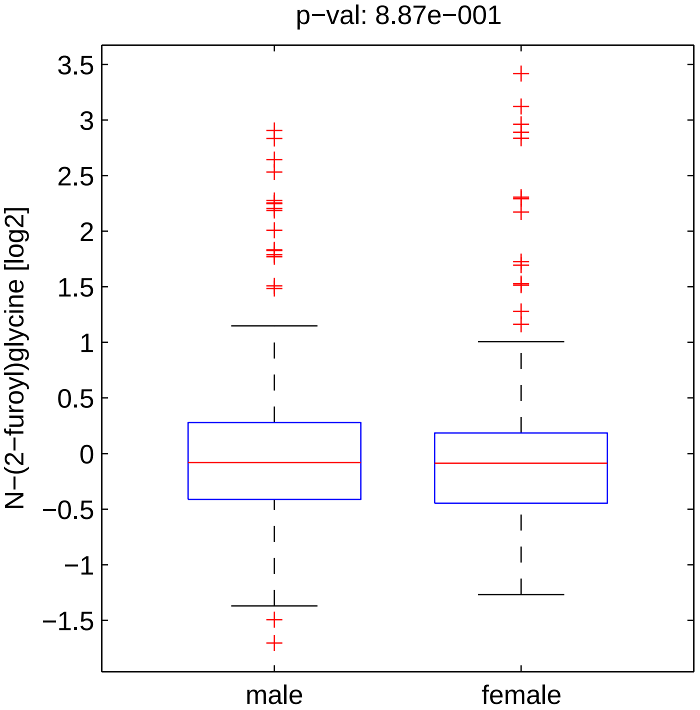
p−val: 8.87e−001
N−(2−furoyl)glycine [log2]
3.5
3
2.5
2
1.5
1
0.5
0
−0.5
−1
−1.5
male
female

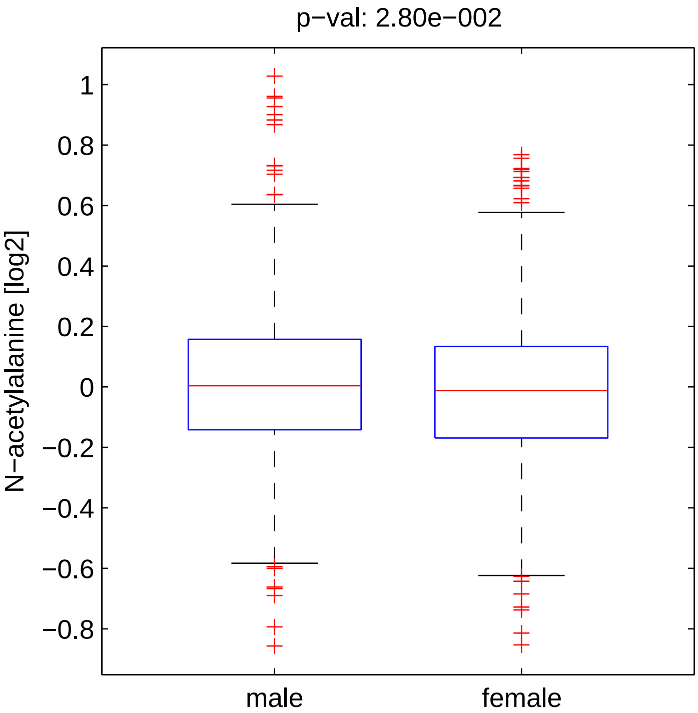
p−val: 2.80e−002
N−acetylalanine [log2]
1
0.8
0.6
0.4
0.2
0
−0.2
−0.4
−0.6
−0.8
male
female

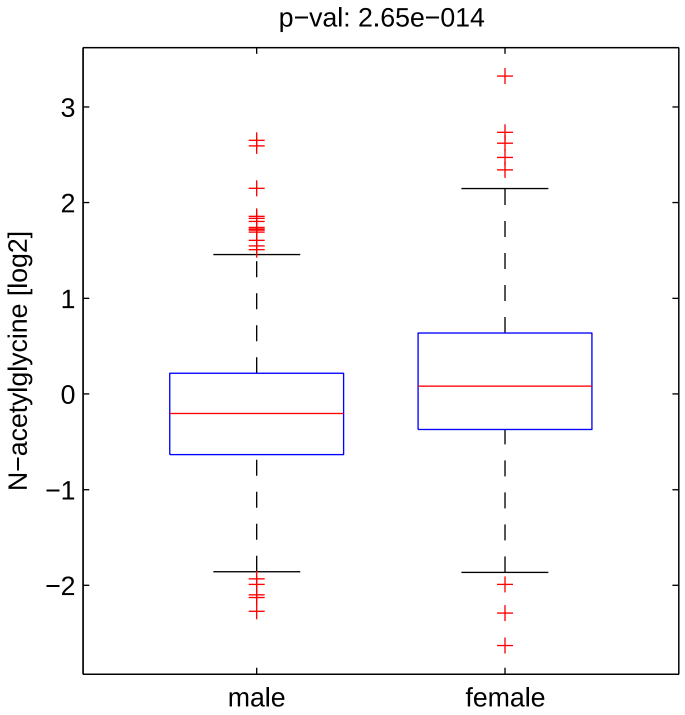
p−val: 2.65e−014
N−acetylglycine [log2]
3
2
1
0
−1
−2
male
female

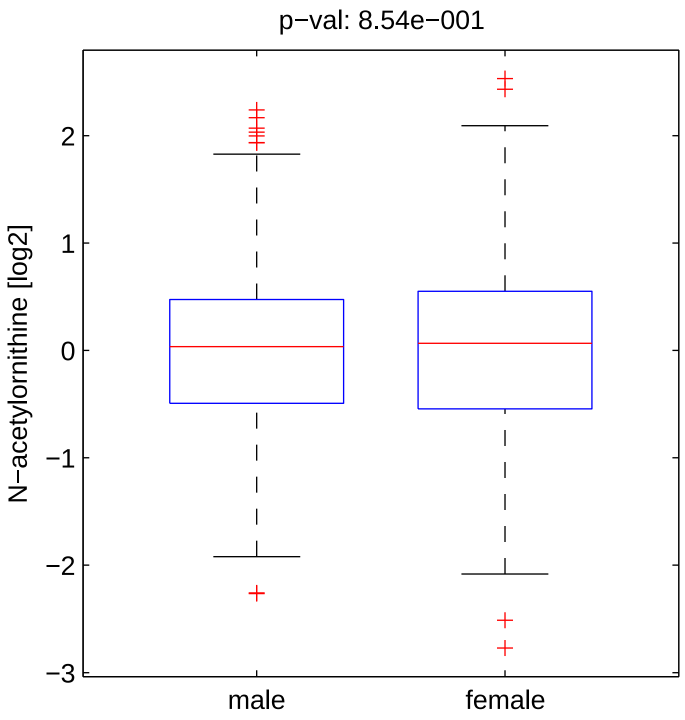
p−val: 8.54e−001
N−acetylornithine [log2]
2
1
0
−1
−2
−3
male
female

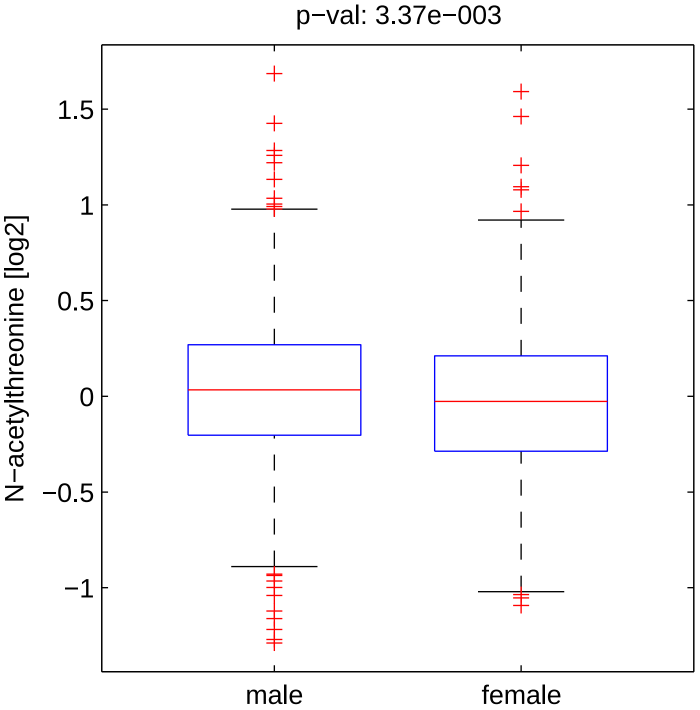
p−val: 3.37e−003
N−acetylthreonine [log2]
1.5
1
0.5
0
−0.5
−1
male
female

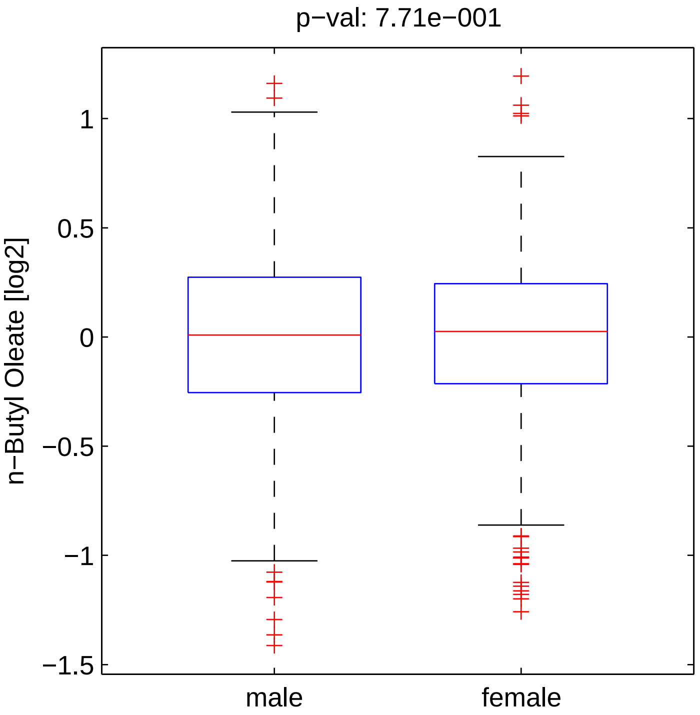

p−val: 7.71e−001
n−Butyl Oleate [log2]
1
0.5
0
−0.5
−1
−1.5
male
female

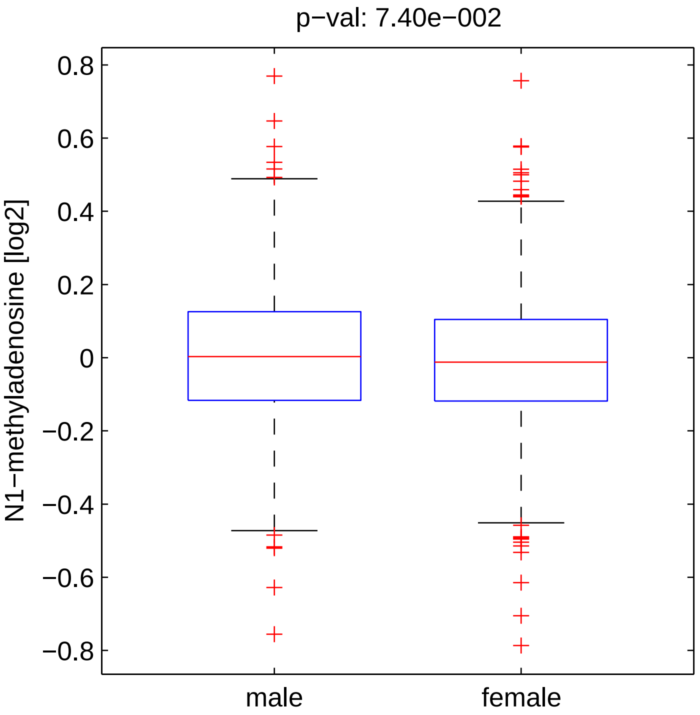

p−val: 7.40e−002
N1−methyladenosine [log2]
0.8
0.6
0.4
0.2
0
−0.2
−0.4
−0.6
−0.8
male
female

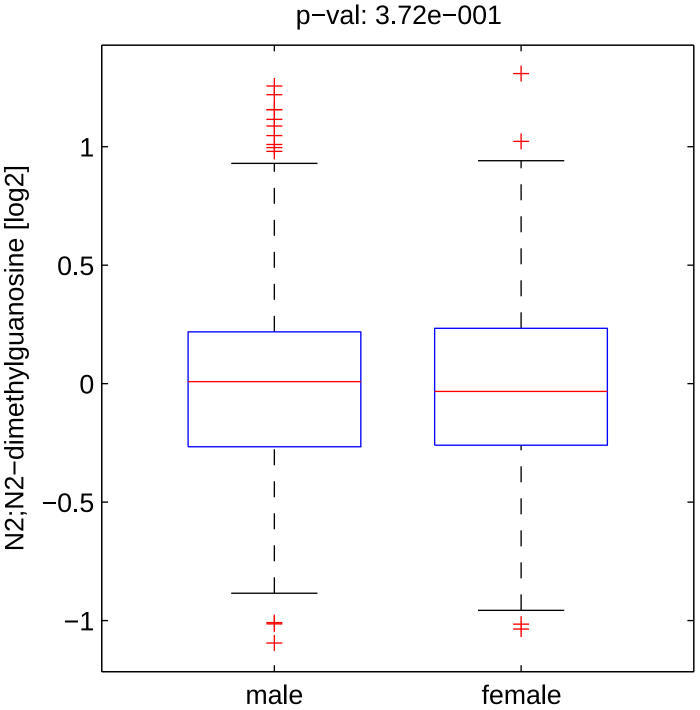

p−val: 3.72e−001
N2;N2−dimethylguanosine [log2]
1
0.5
0
−0.5
−1
male
female

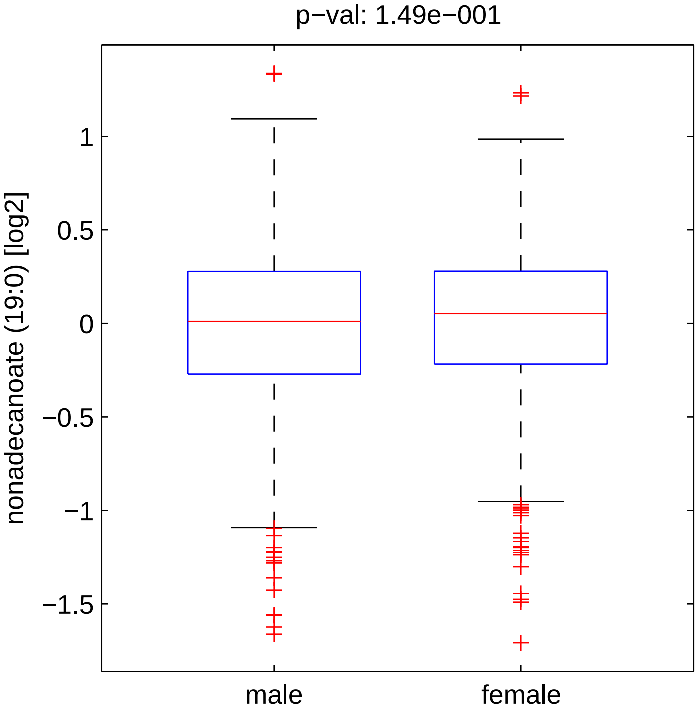
p−val: 1.49e−001
nonadecanoate (19:0) [log2]
1
0.5
0
−0.5
−1
−1.5
male
female

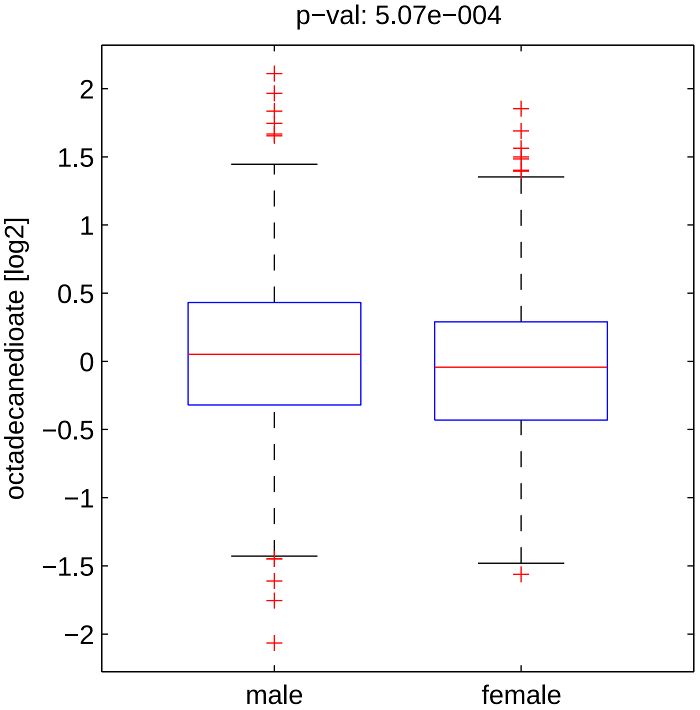
p−val: 5.07e−004
octadecanedioate [log2]
2
1.5
1
0.5
0
−0.5
−1
−1.5
−2
male
female

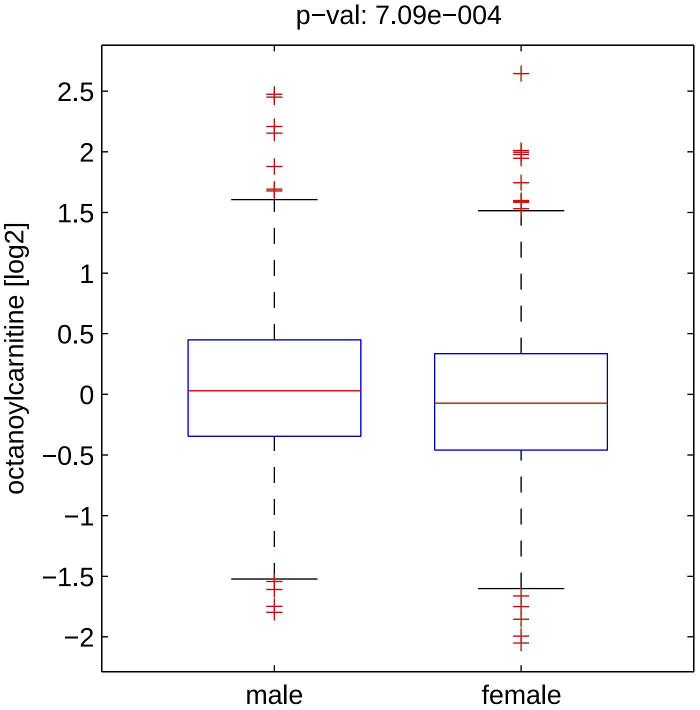
p−val: 7.09e−004
octanoylcarnitine [log2]
2.5
2
1.5
1
0.5
0
−0.5
−1
−1.5
−2
male
female

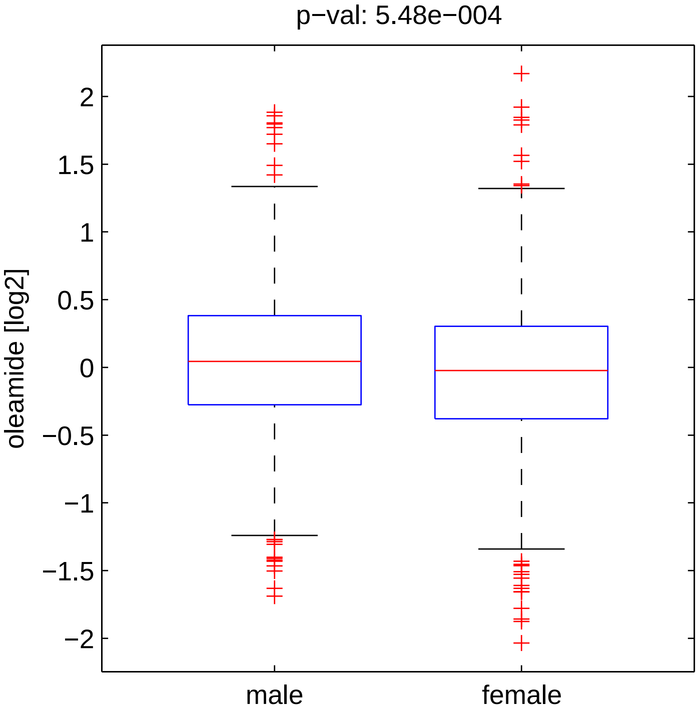
p−val: 5.48e−004
oleamide [log2]
2
1.5
1
0.5
0
−0.5
−1
−1.5
−2
male
female

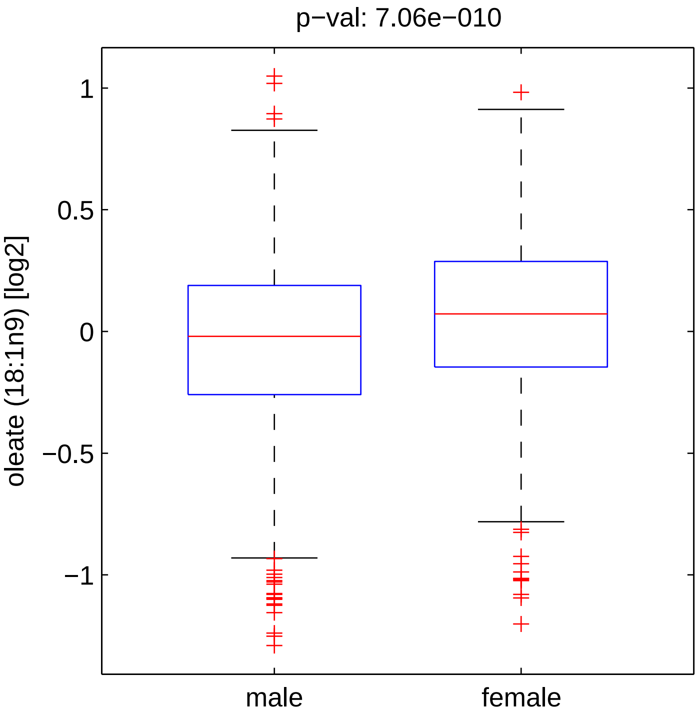
p−val: 7.06e−010
1
0.5
0
−0.5
−1
oleate (18:1n9) [log2]
male
female

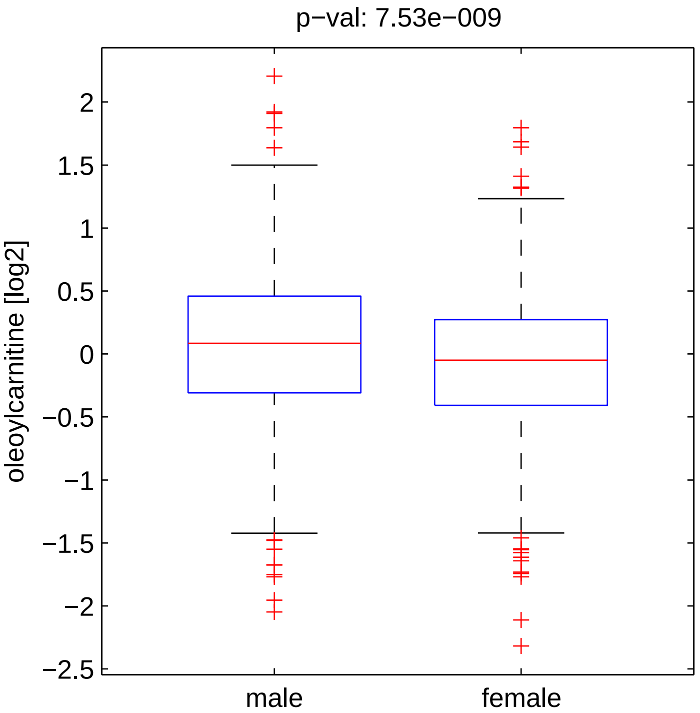
p−val: 7.53e−009
oleoylcarnitine [log2]
2
1.5
1
0.5
0
−0.5
−1
−1.5
−2
−2.5
male
female

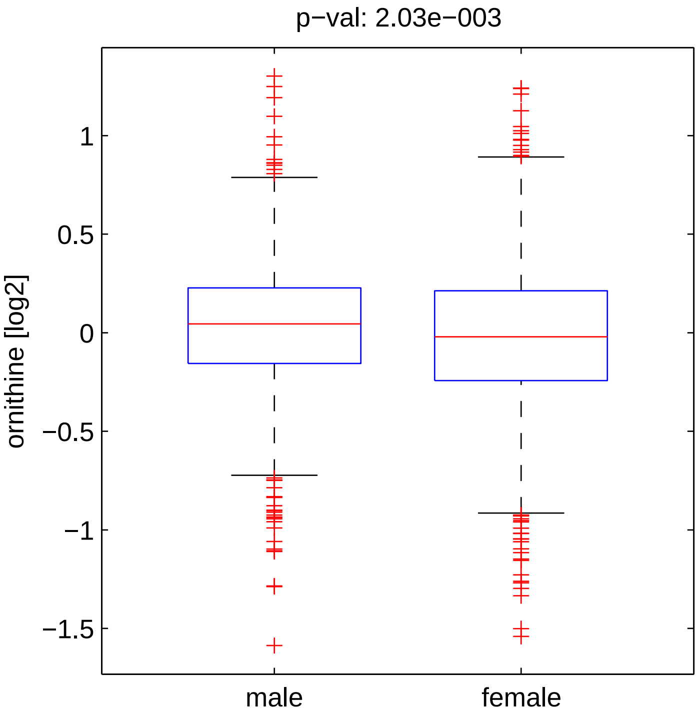
p−val: 2.03e−003
ornithine [log2]
1
0.5
0
−0.5
−1
−1.5
male
female

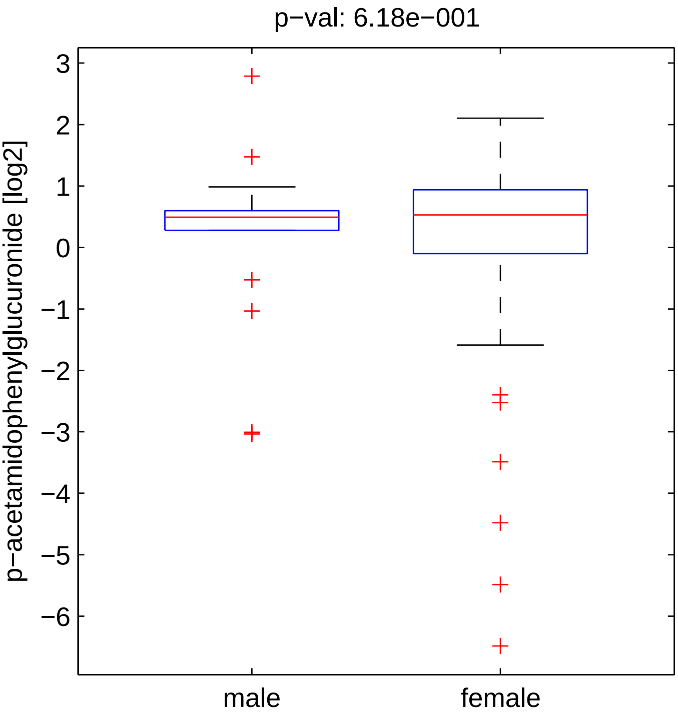
p−val: 6.18e−001
p−acetamidophenylglucuronide [log2]
3
2
1
0
−1
−2
−3
−4
−5
−6
male
female

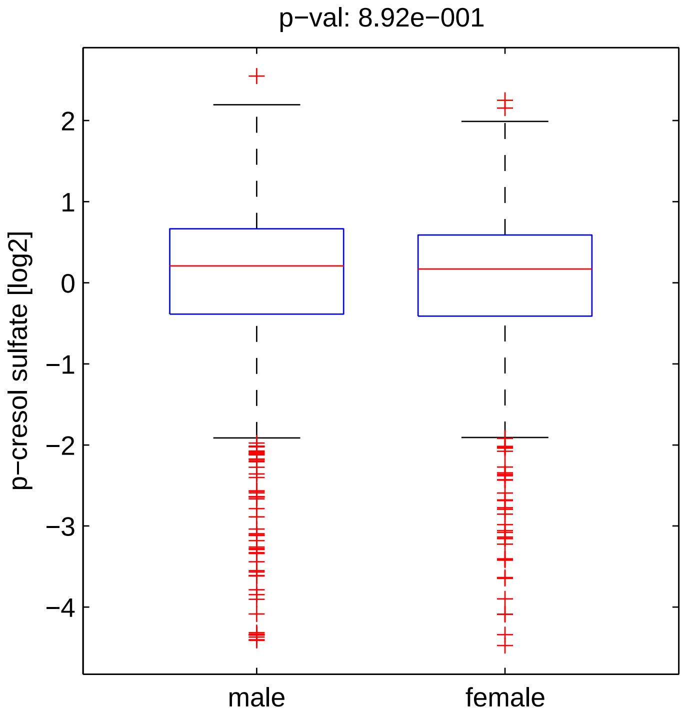
p−val: 8.92e−001
p−cresol sulfate [log2]
2
1
0
−1
−2
−3
−4
male
female

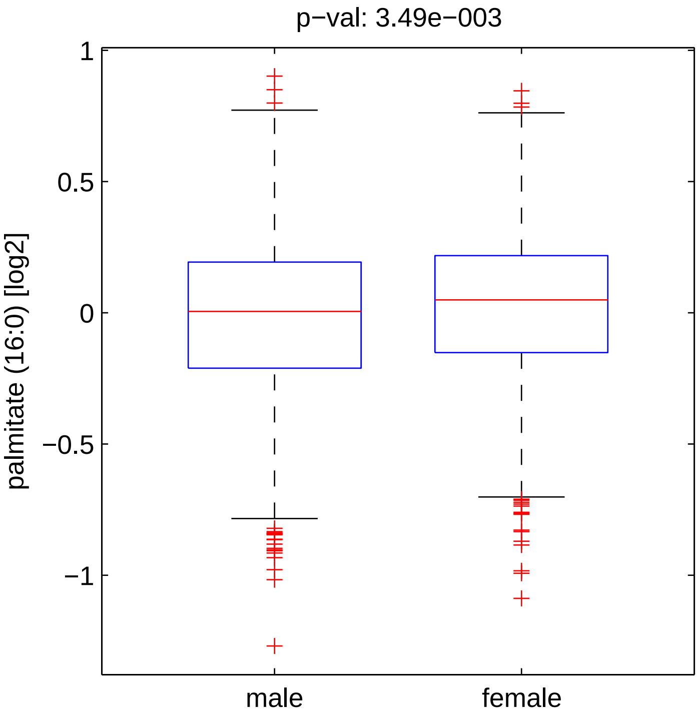

p−val: 3.49e−003
1
0.5
0
−0.5
−1
palmitate (16:0) [log2]
male
female

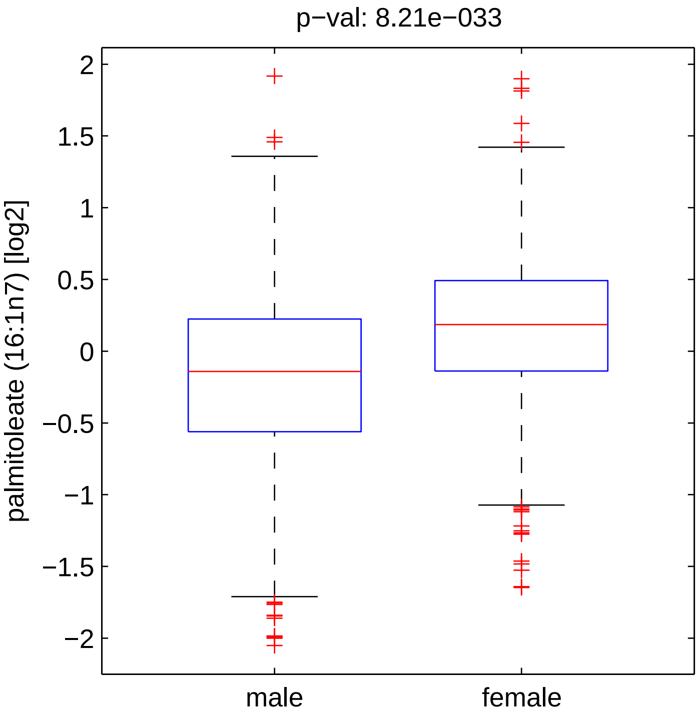
p−val: 8.21e−033
palmitoleate (16:1n7) [log2]
2
1.5
1
0.5
0
−0.5
−1
−1.5
−2
male
female

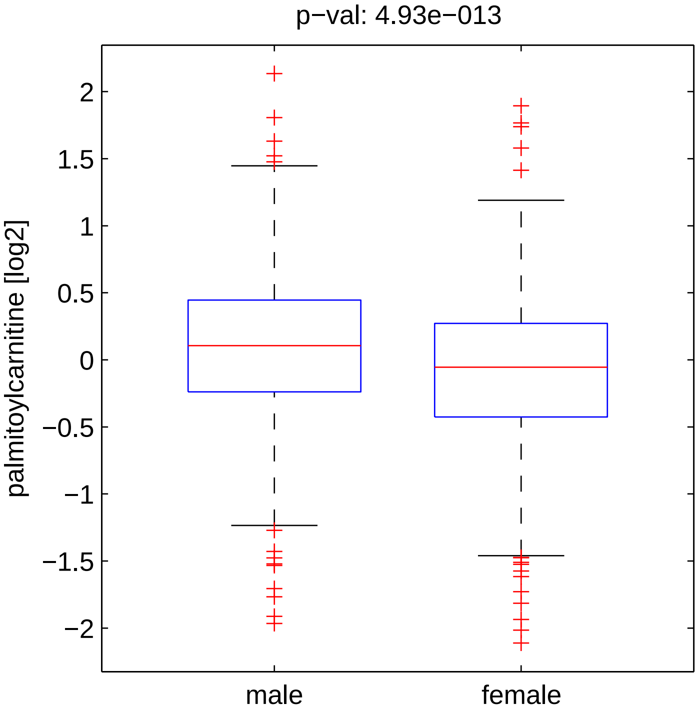

p−val: 4.93e−013
palmitoylcarnitine [log2]
2
1.5
1
0.5
0
−0.5
−1
−1.5
−2
male
female

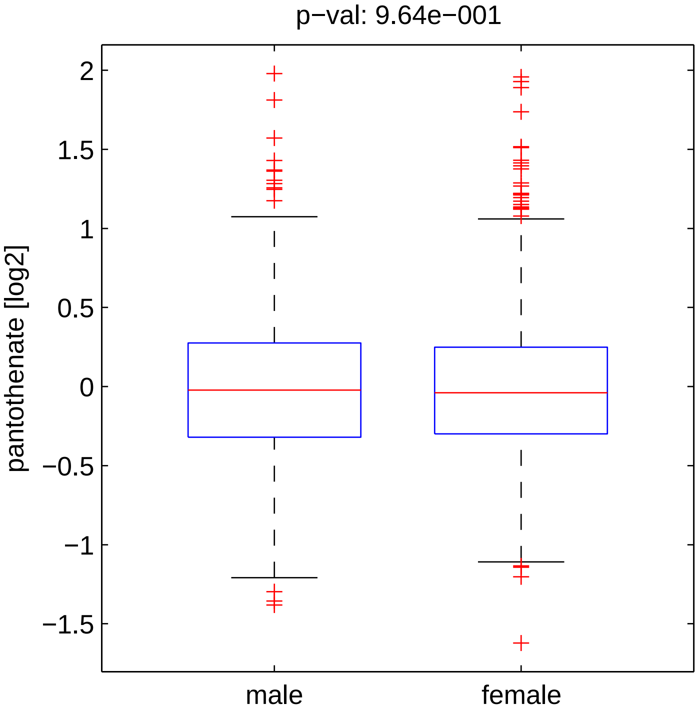
p−val: 9.64e−001
pantothenate [log2]
2
1.5
1
0.5
0
−0.5
−1
−1.5
male
female

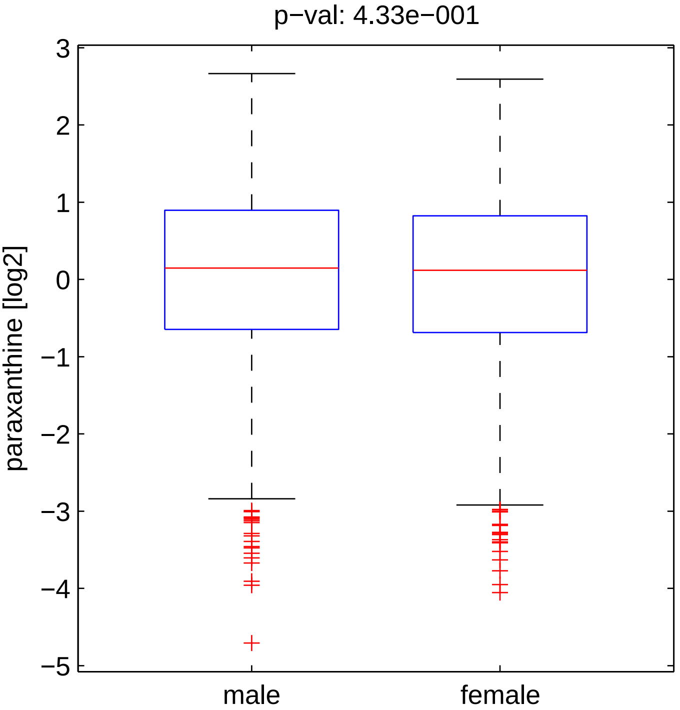

p−val: 4.33e−001
3
2
1
0
−1
−2
−3
−4
−5
paraxanthine [log2]
male
female

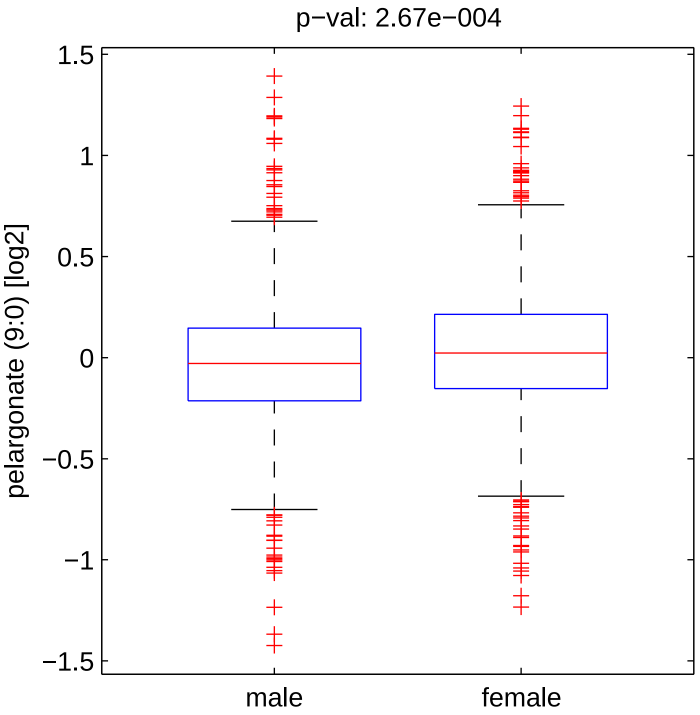

p−val: 2.67e−004
1.5
1
0.5
0
−0.5
−1
−1.5
pelargonate (9:0) [log2]
male
female

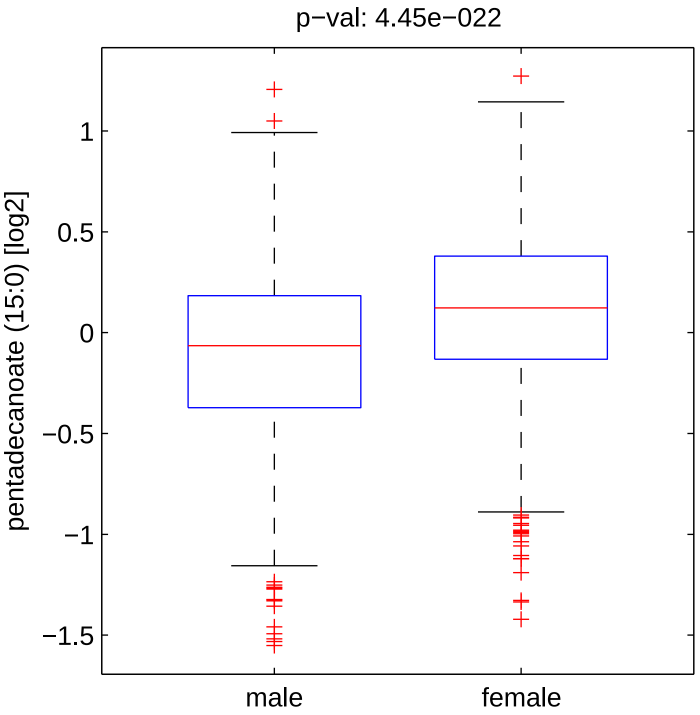
p−val: 4.45e−022
pentadecanoate (15:0) [log2]
1
0.5
0
−0.5
−1
−1.5
male
female

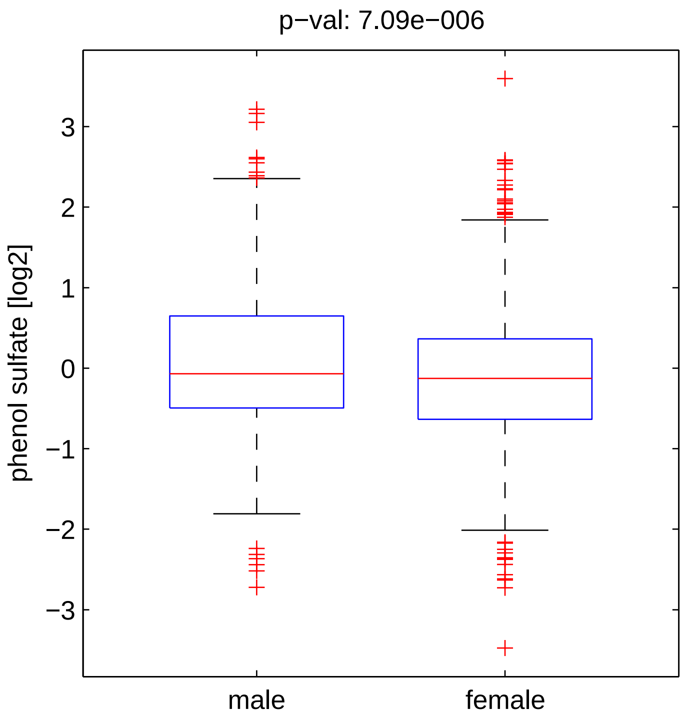

p−val: 7.09e−006
phenol sulfate [log2]
3
2
1
0
−1
−2
−3
male
female

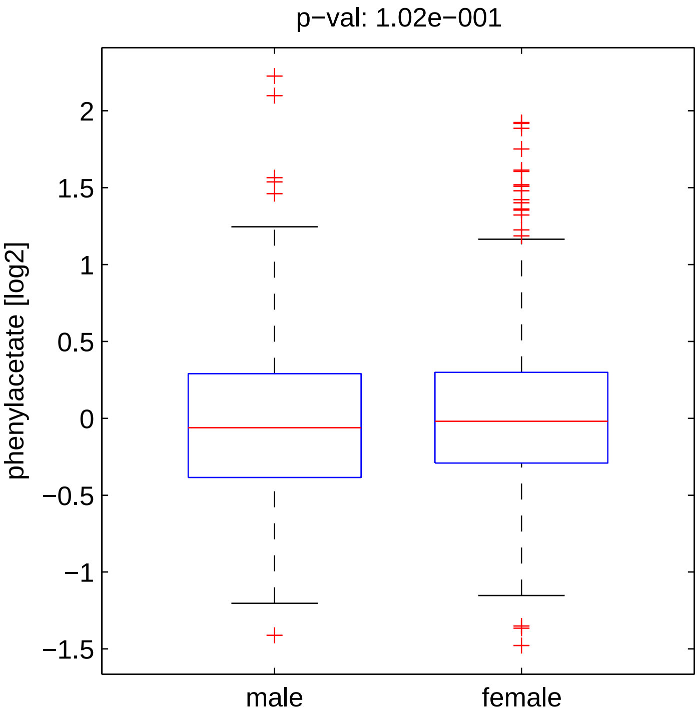

p−val: 1.02e−001
phenylacetate [log2]
2
1.5
1
0.5
0
−0.5
−1
−1.5
male
female

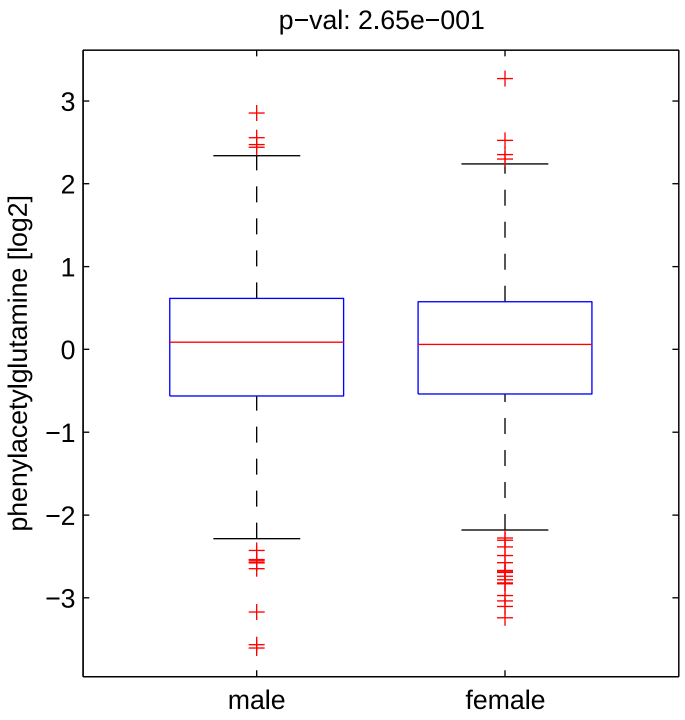
p−val: 2.65e−001
3
2
1
0
−1
−2
−3
phenylacetylglutamine [log2]
male
female

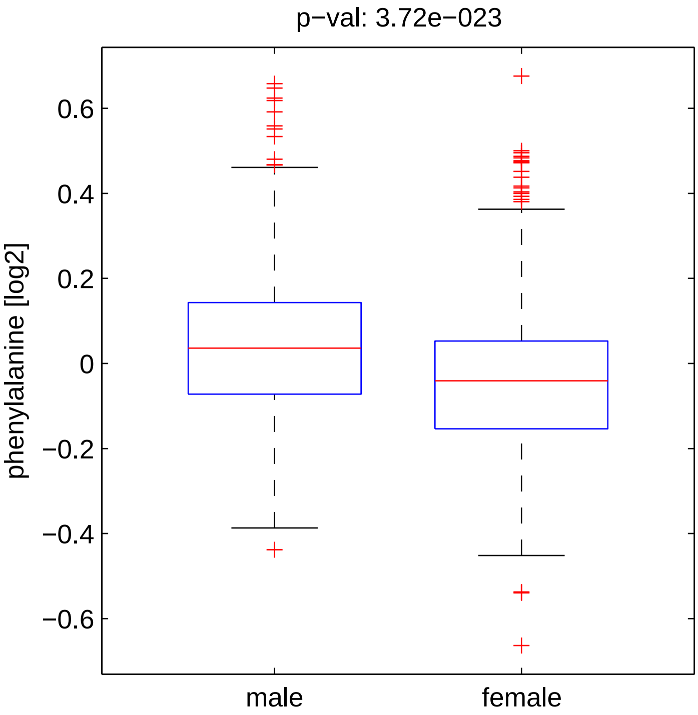

p−val: 3.72e−023
0.6
0.4
0.2
0
−0.2
−0.4
−0.6
phenylalanine [log2]
male
female

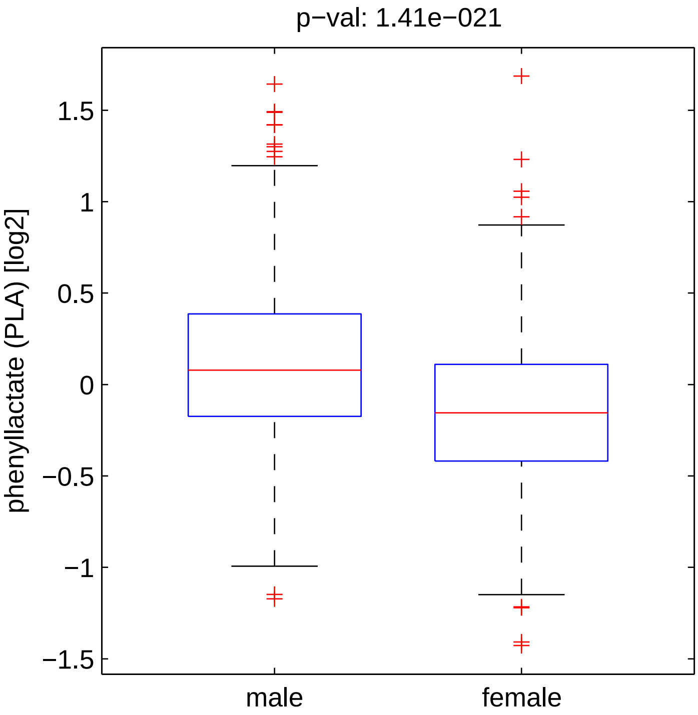

p−val: 1.41e−021
phenyllactate (PLA) [log2]
1.5
1
0.5
0
−0.5
−1
−1.5
male
female

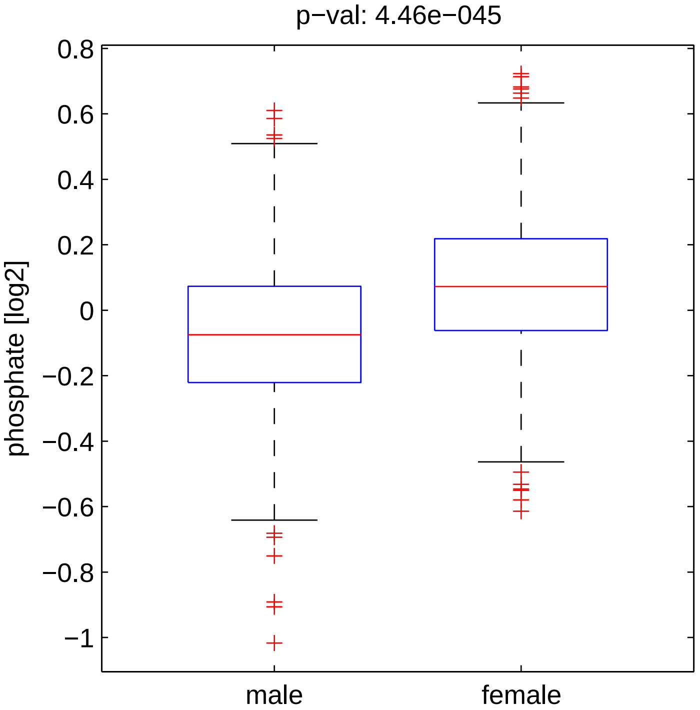

p−val: 4.46e−045
0.8
0.6
0.4
0.2
0
−0.2
−0.4
−0.6
−0.8
−1
phosphate [log2]
male
female

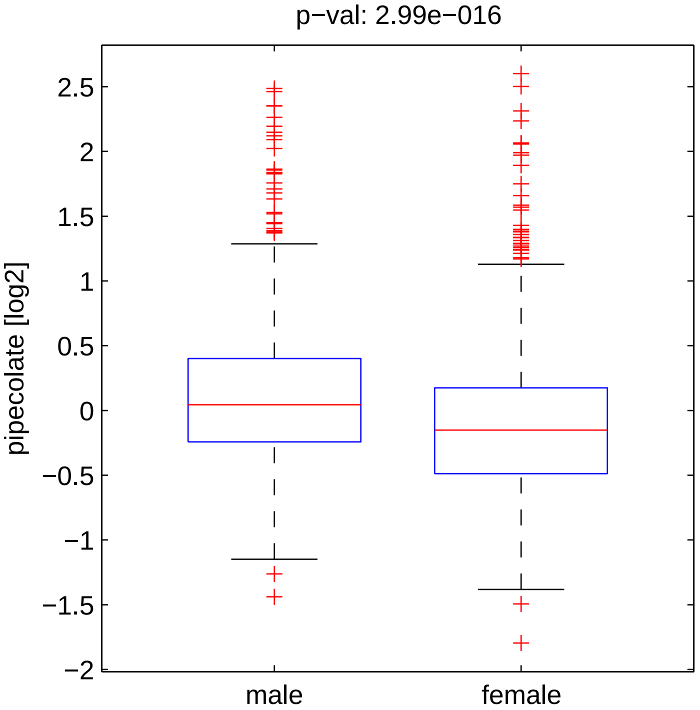
p−val: 2.99e−016
pipecolate [log2]
2.5
2
1.5
1
0.5
0
−0.5
−1
−1.5
−2
male
female

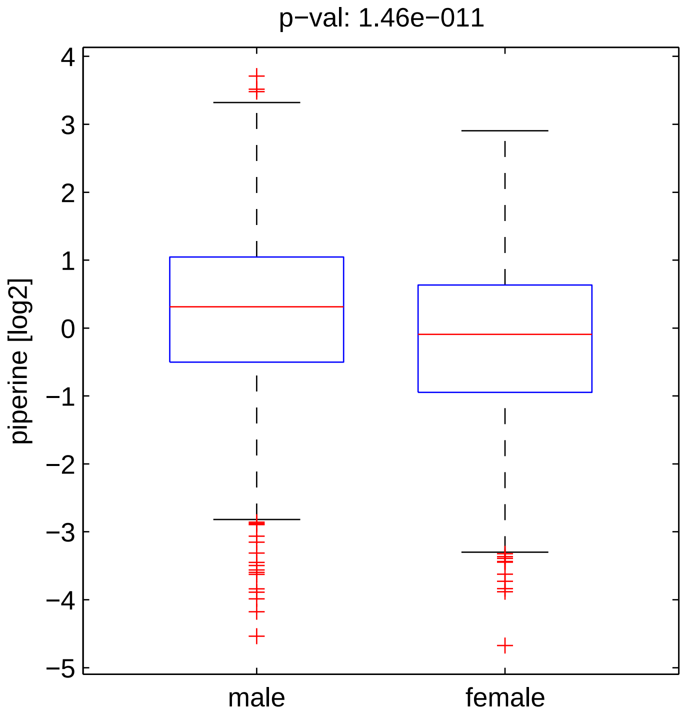
p−val: 1.46e−011
4
3
2
1
0
−1
−2
−3
−4
−5
piperine [log2]
male
female

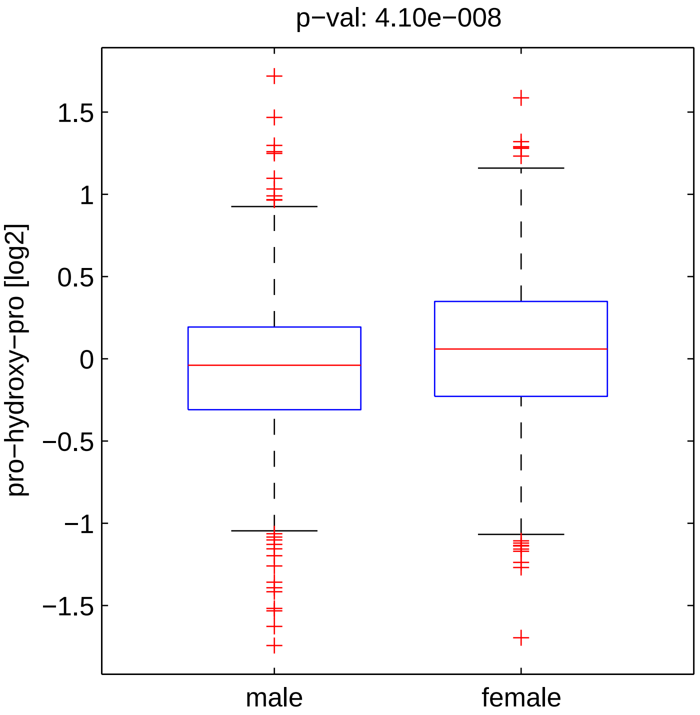
p−val: 4.10e−008
pro−hydroxy−pro [log2]
1.5
1
0.5
0
−0.5
−1
−1.5
male
female

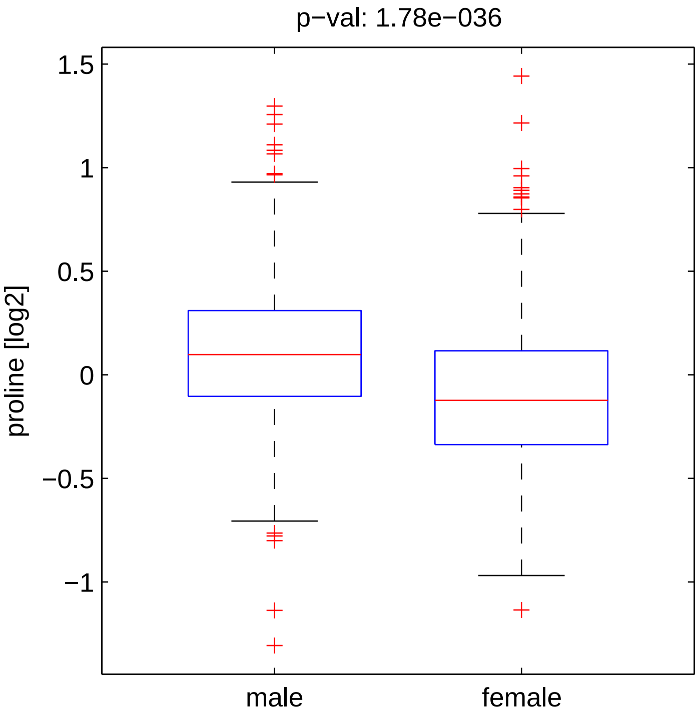
p−val: 1.78e−036
1.5
1
0.5
0
−0.5
−1
proline [log2]
male
female

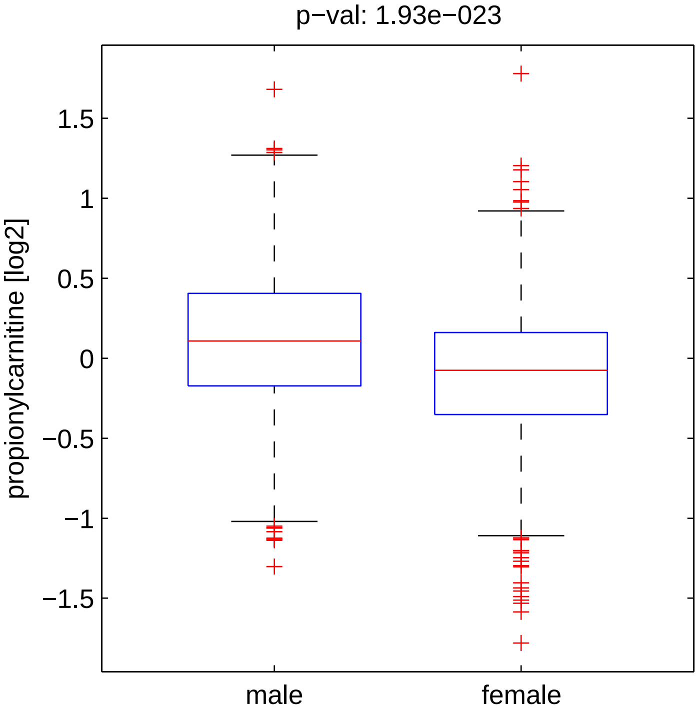
p−val: 1.93e−023
propionylcarnitine [log2]
1.5
1
0.5
0
−0.5
−1
−1.5
male
female

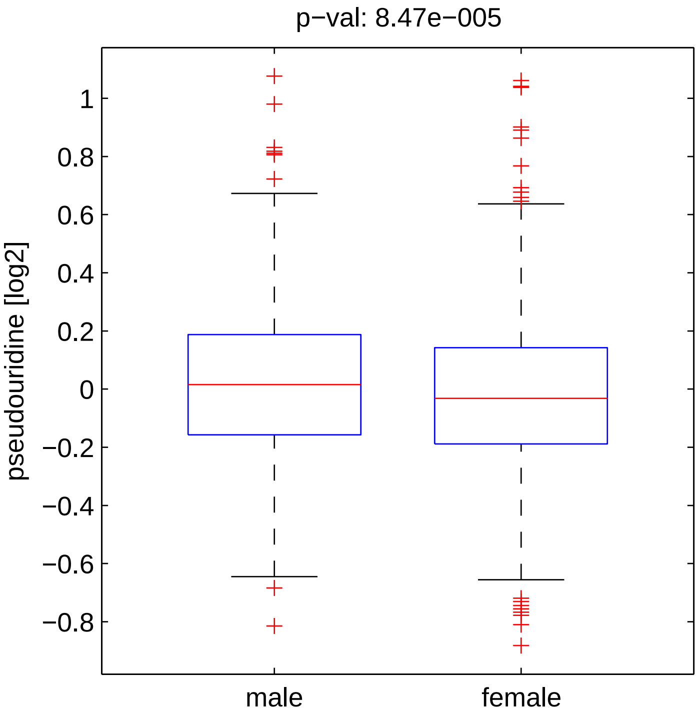
p−val: 8.47e−005
pseudouridine [log2]
1
0.8
0.6
0.4
0.2
0
−0.2
−0.4
−0.6
−0.8
male
female

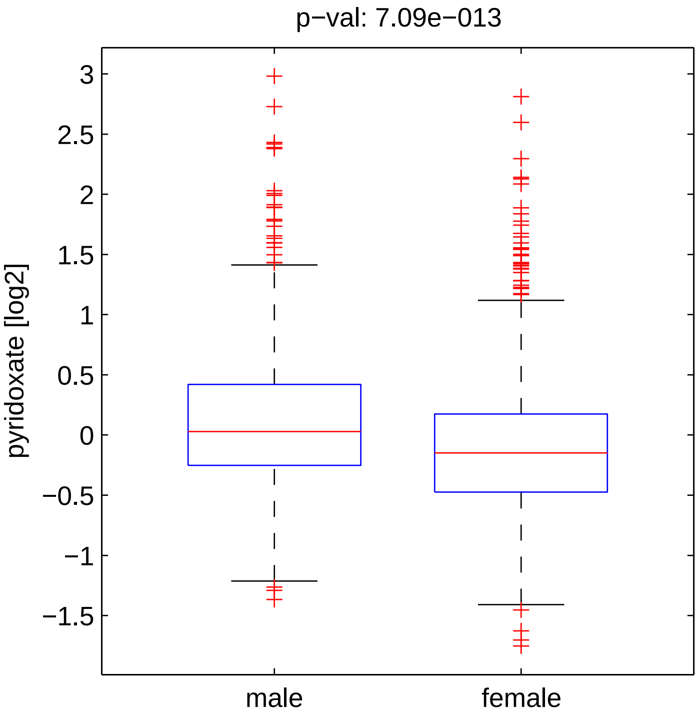
p−val: 7.09e−013
pyridoxate [log2]
3
2.5
2
1.5
1
0.5
0
−0.5
−1
−1.5
male
female

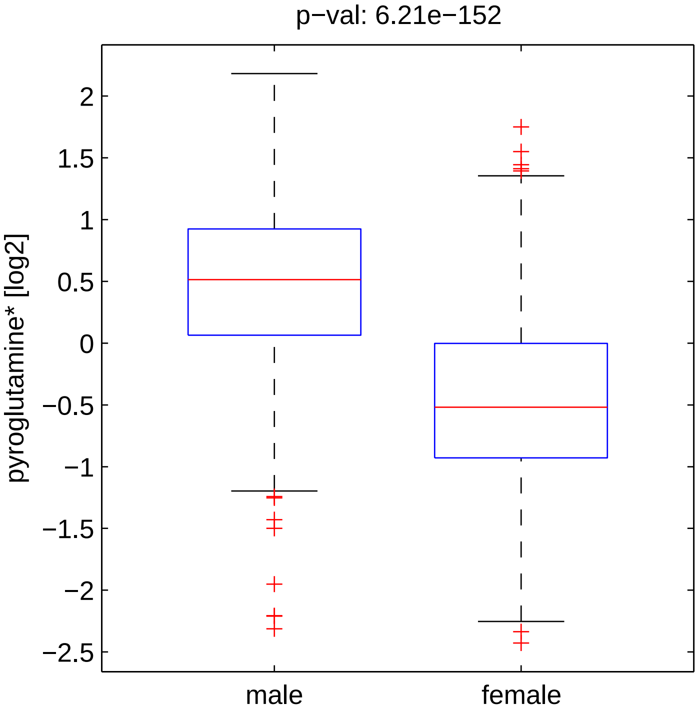
p−val: 6.21e−152
pyroglutamine* [log2]
2
1.5
1
0.5
0
−0.5
−1
−1.5
−2
−2.5
male
female

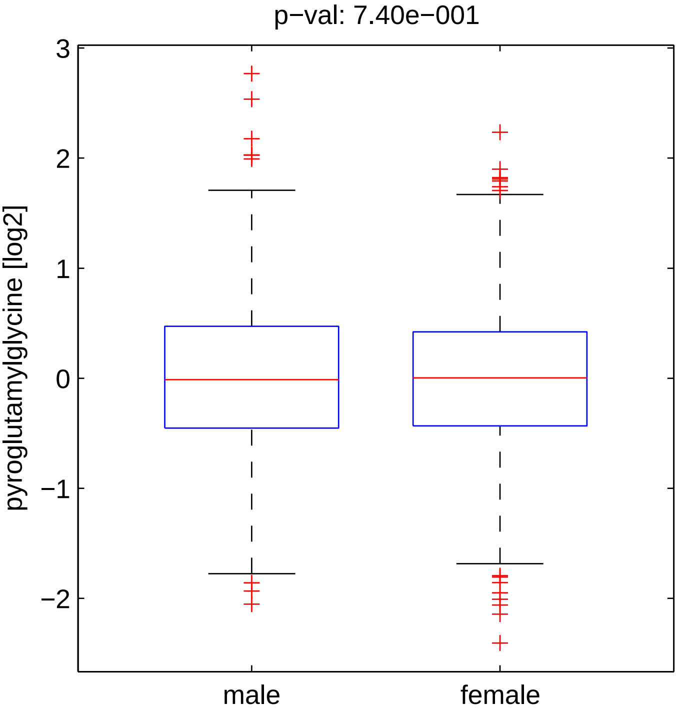
p−val: 7.40e−001
pyroglutamylglycine [log2]
3
2
1
0
−1
−2
male
female

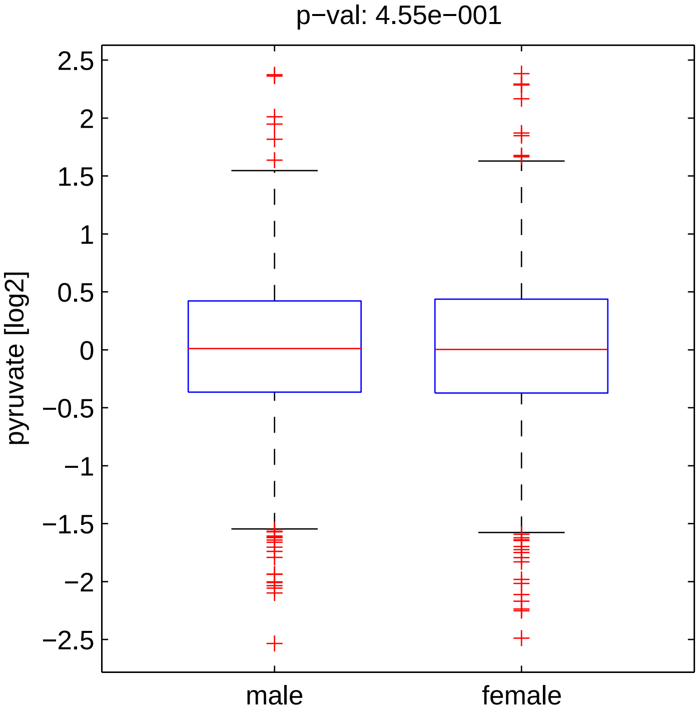
p−val: 4.55e−001
pyruvate [log2]
2.5
2
1.5
1
0.5
0
−0.5
−1
−1.5
−2
−2.5
male
female

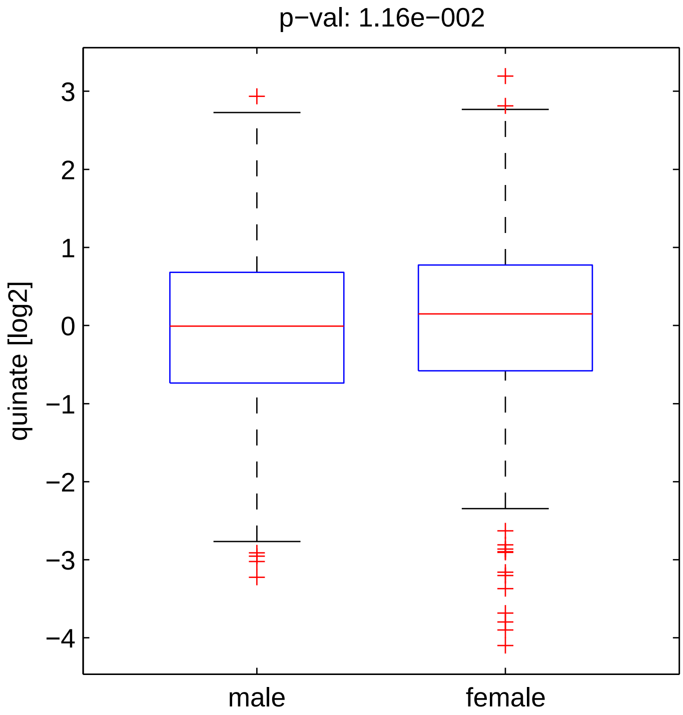
p−val: 1.16e−002
quinate [log2]
3
2
1
0
−1
−2
−3
−4
male
female

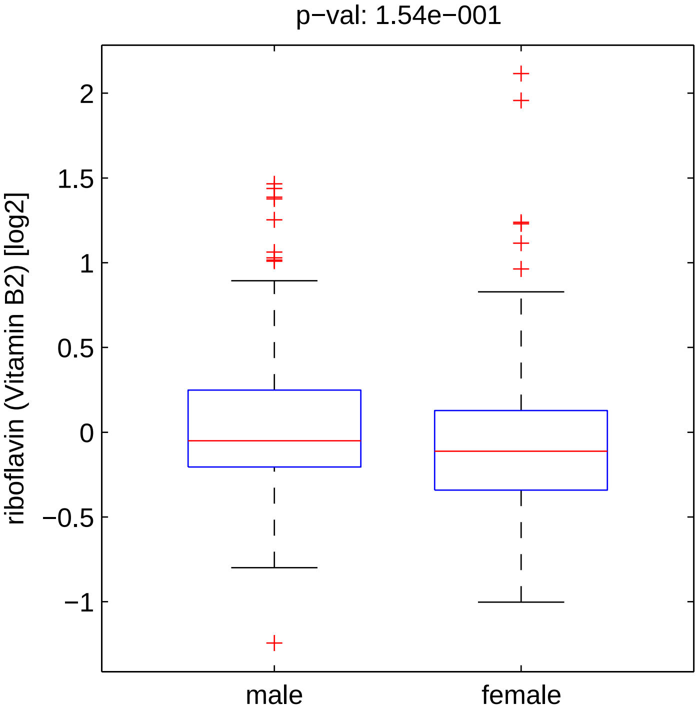

p−val: 1.54e−001
riboflavin (Vitamin B2) [log2]
2
1.5
1
0.5
0
−0.5
−1
male
female

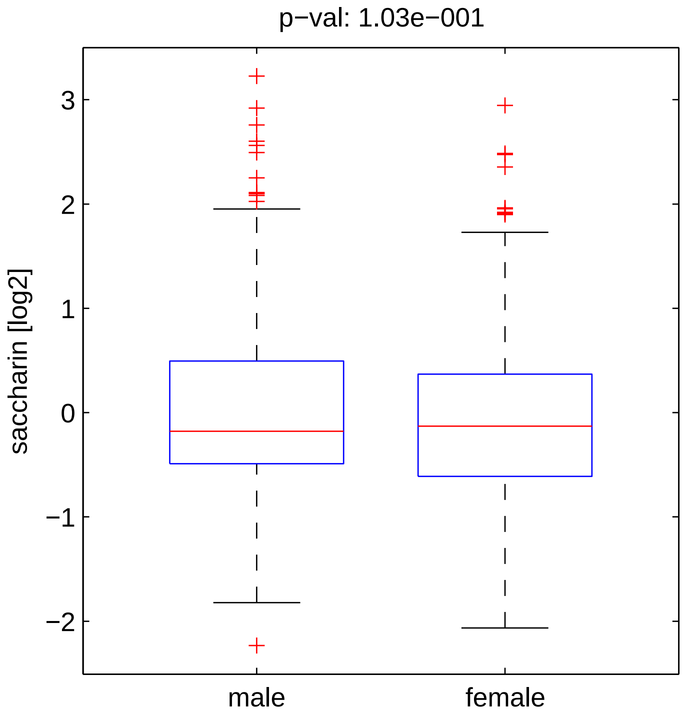
p−val: 1.03e−001
saccharin [log2]
3
2
1
0
−1
−2
male
female

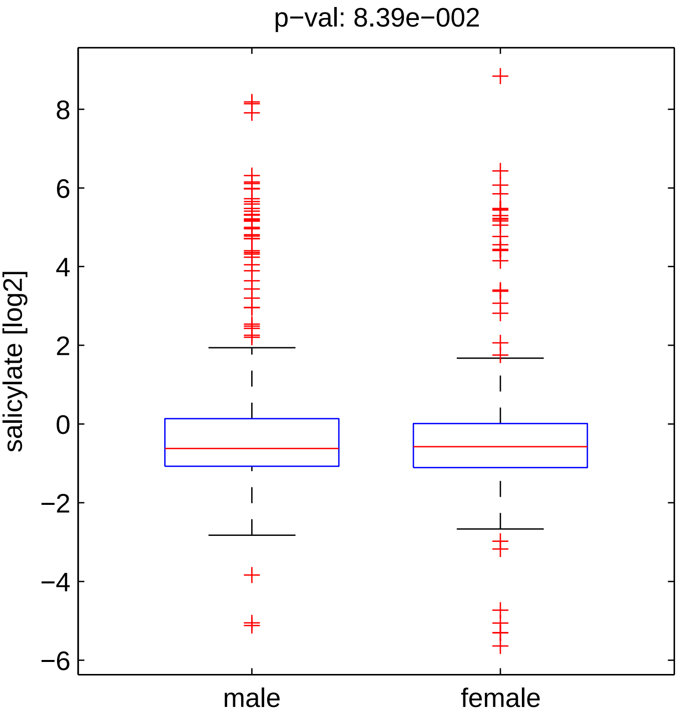
p−val: 8.39e−002
salicylate [log2]
8
6
4
2
0
−2
−4
−6
male
female

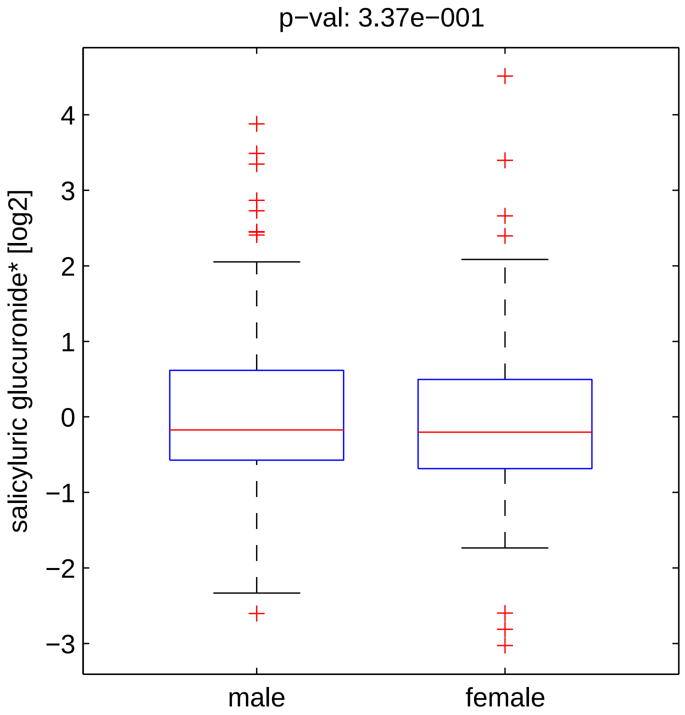

p−val: 3.37e−001
salicyluric glucuronide* [log2]
4
3
2
1
0
−1
−2
−3
male
female

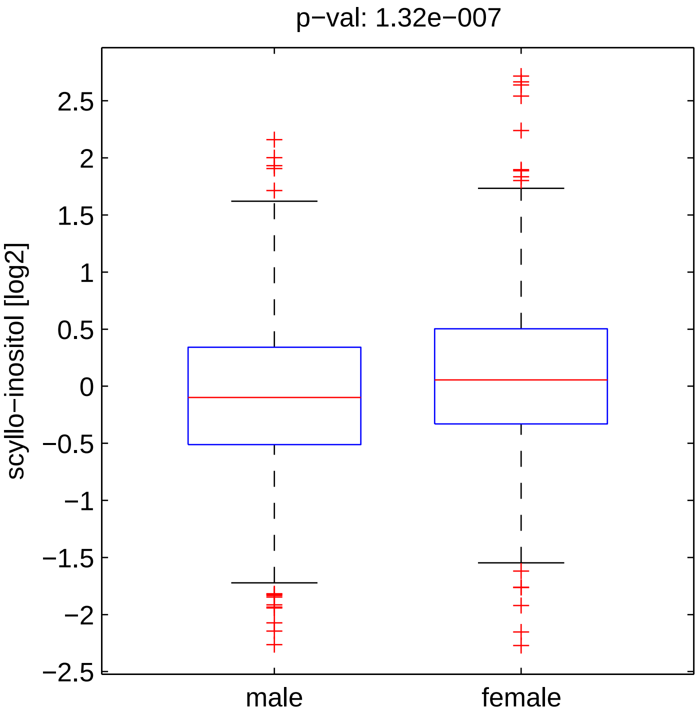
p−val: 1.32e−007
scyllo−inositol [log2]
2.5
2
1.5
1
0.5
0
−0.5
−1
−1.5
−2
−2.5
male
female

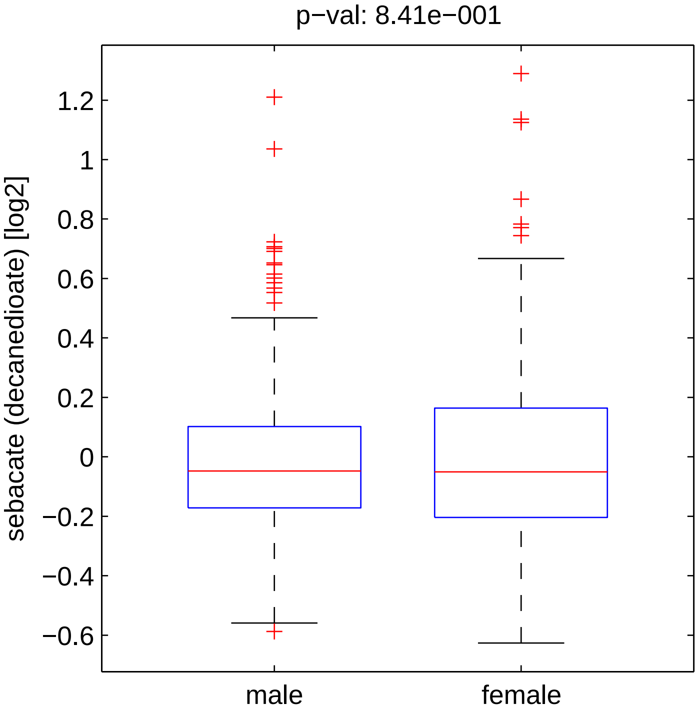
p−val: 8.41e−001
sebacate (decanedioate) [log2]
1.2
1
0.8
0.6
0.4
0.2
0
−0.2
−0.4
−0.6
male
female

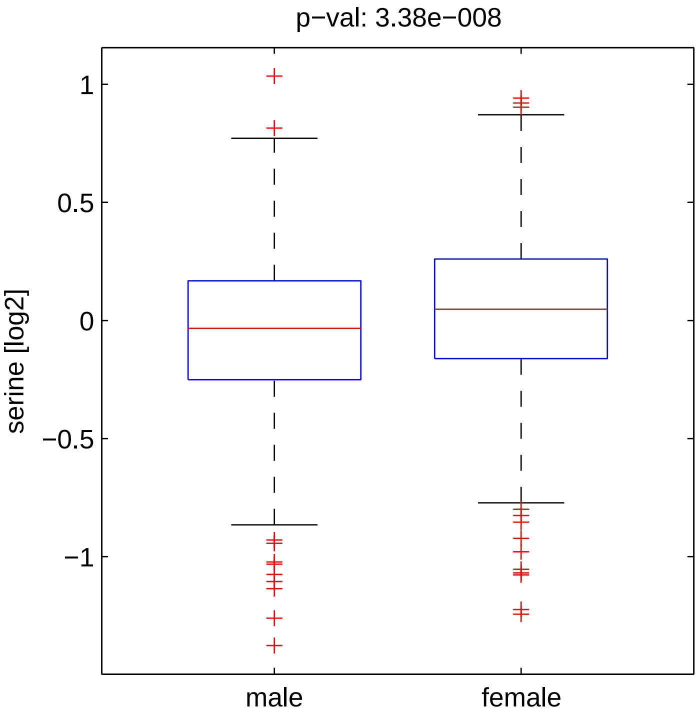
p−val: 3.38e−008
1
0.5
0
−0.5
−1
serine [log2]
male
female

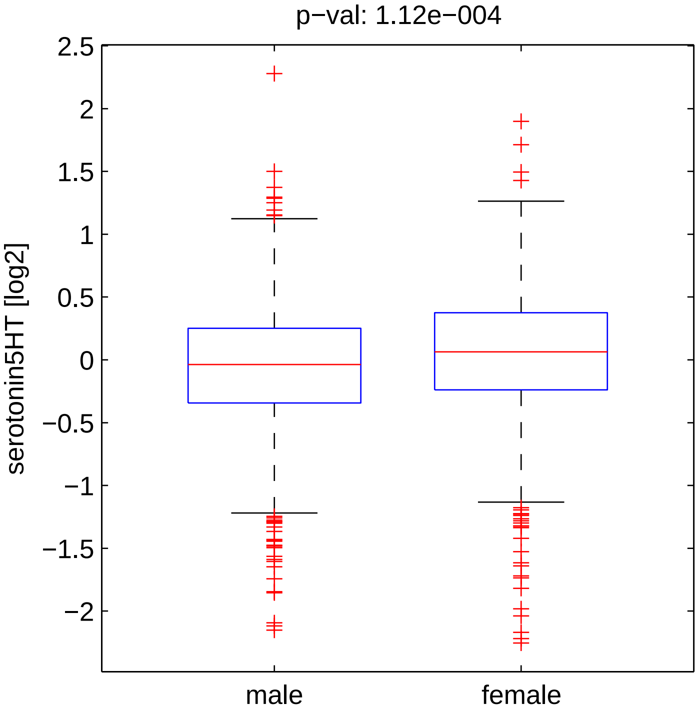
p−val: 1.12e−004
serotonin5HT [log2]
2.5
2
1.5
1
0.5
0
−0.5
−1
−1.5
−2
male
female

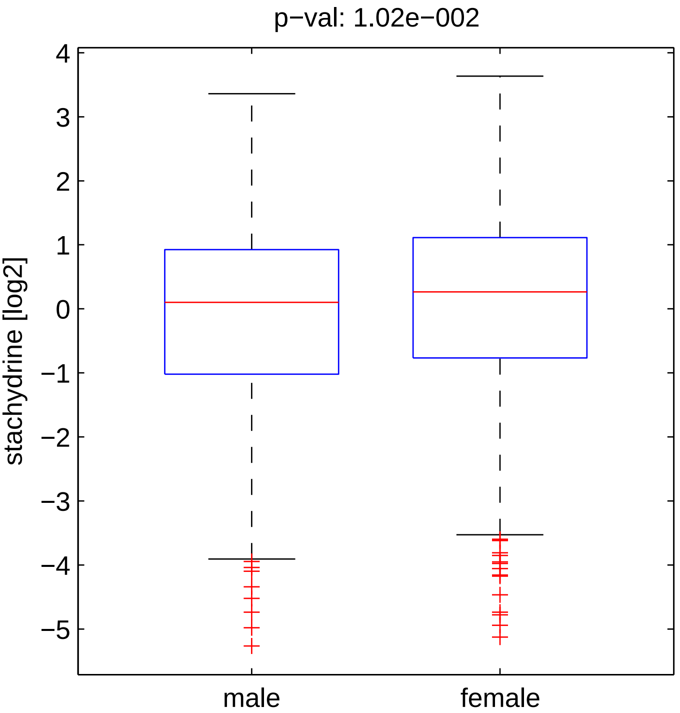

p−val: 1.02e−002
4
3
2
1
0
−1
−2
−3
−4
−5
stachydrine [log2]
male
female

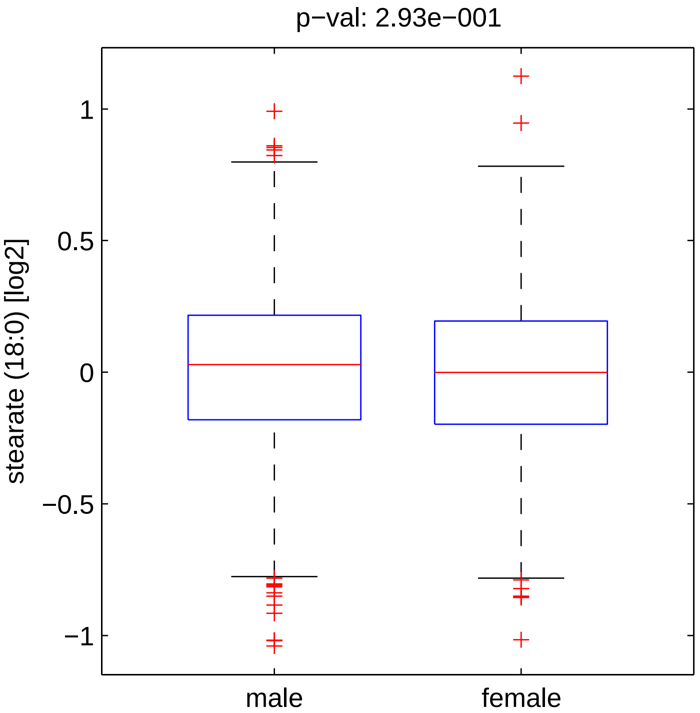

p−val: 2.93e−001
stearate (18:0) [log2]
1
0.5
0
−0.5
−1
male
female

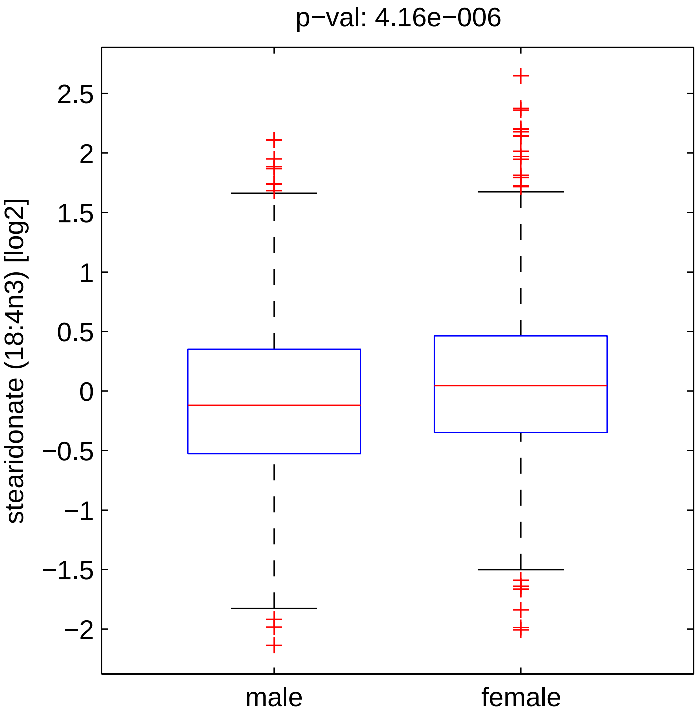
p−val: 4.16e−006
stearidonate (18:4n3) [log2]
2.5
2
1.5
1
0.5
0
−0.5
−1
−1.5
−2
male
female

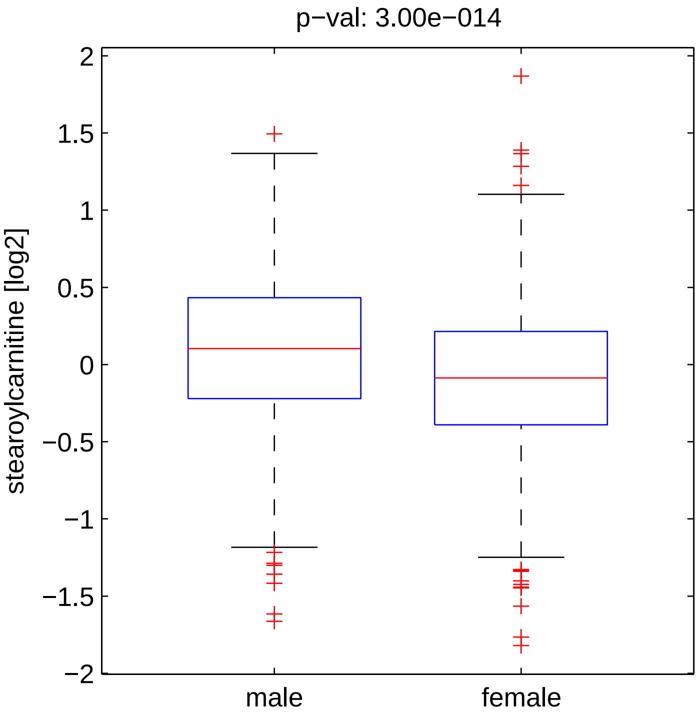

p−val: 3.00e−014
stearoylcarnitine [log2]
2
1.5
1
0.5
0
−0.5
−1
−1.5
−2
male
female

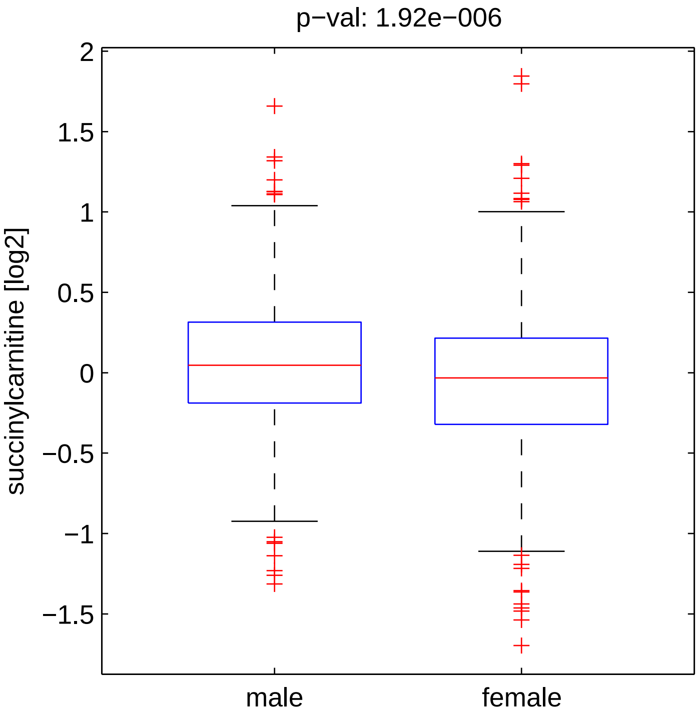
p−val: 1.92e−006
2
1.5
1
0.5
0
−0.5
−1
−1.5
succinylcarnitine [log2]
male
female

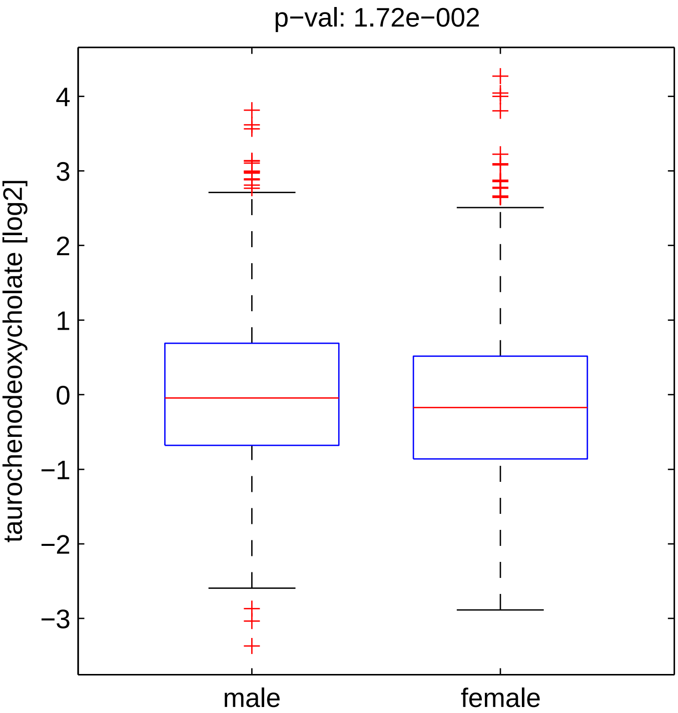

p−val: 1.72e−002
taurochenodeoxycholate [log2]
4
3
2
1
0
−1
−2
−3
male
female

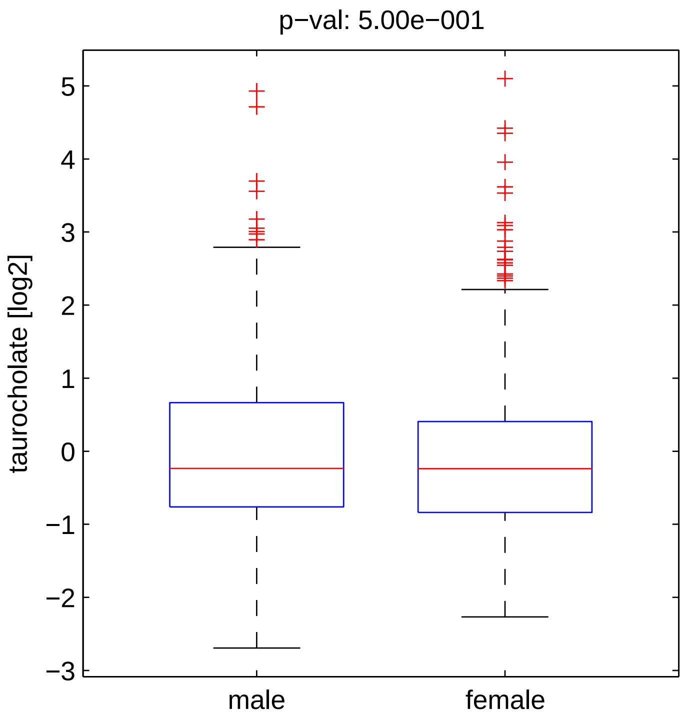
p−val: 5.00e−001
taurocholate [log2]
5
4
3
2
1
0
−1
−2
−3
male
female

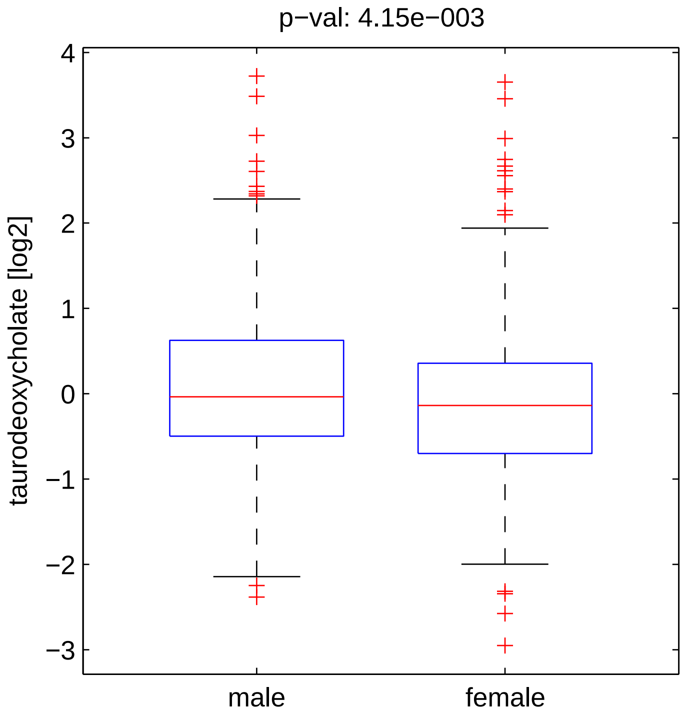

p−val: 4.15e−003
taurodeoxycholate [log2]
4
3
2
1
0
−1
−2
−3
male
female

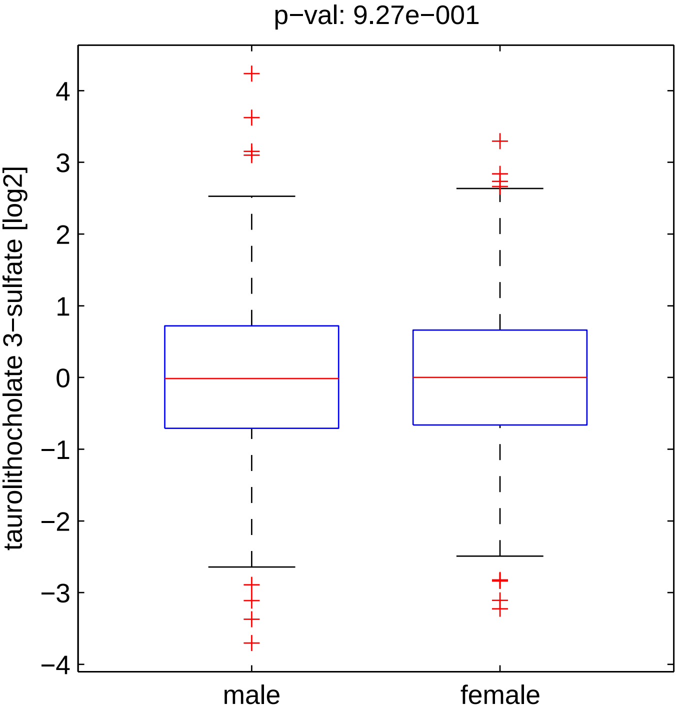
p−val: 9.27e−001
taurolithocholate 3−sulfate [log2]
4
3
2
1
0
−1
−2
−3
−4
male
female

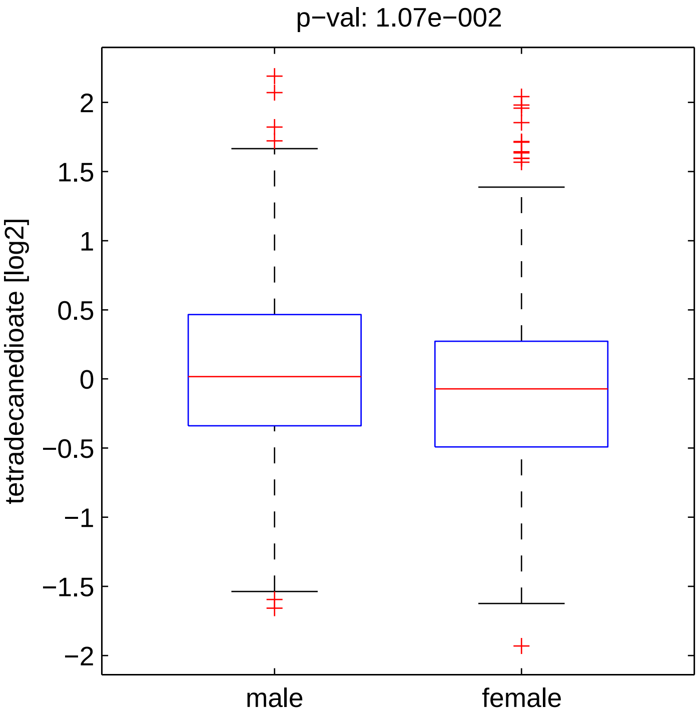
p−val: 1.07e−002
tetradecanedioate [log2]
2
1.5
1
0.5
0
−0.5
−1
−1.5
−2
male
female

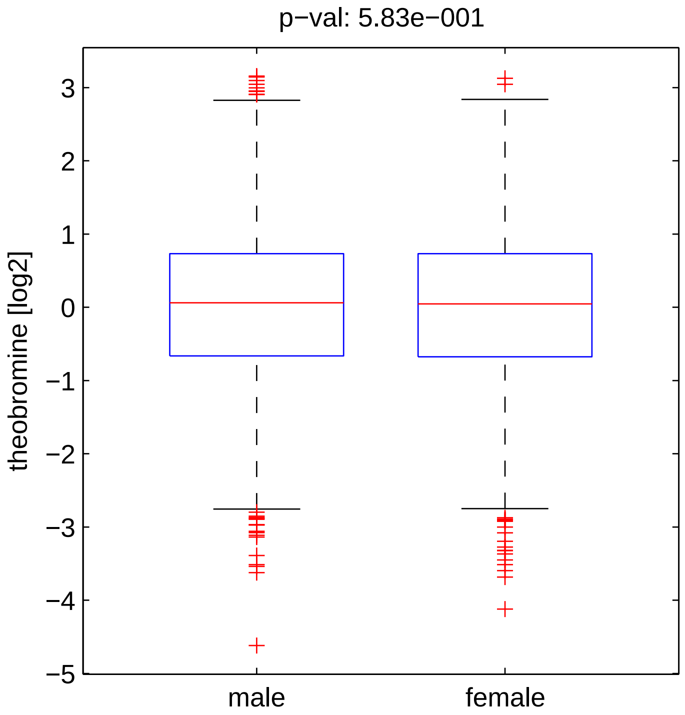
p−val: 5.83e−001
theobromine [log2]
3
2
1
0
−1
−2
−3
−4
−5
male
female

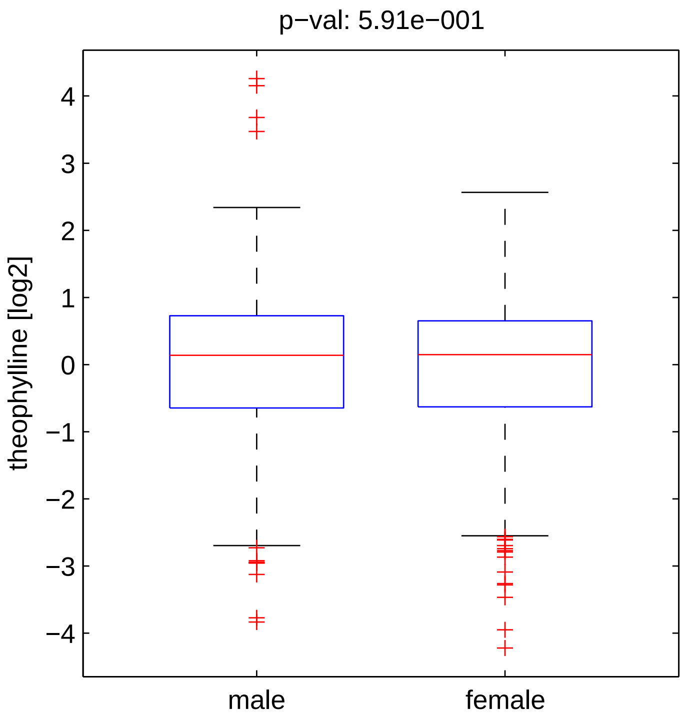

p−val: 5.91e−001
theophylline [log2]
4
3
2
1
0
−1
−2
−3
−4
male
female

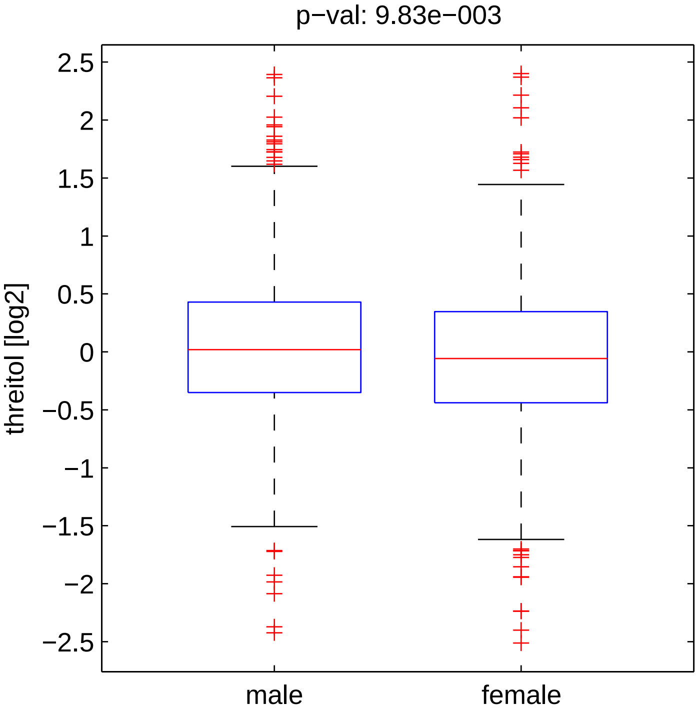
p−val: 9.83e−003
2.5
2
1.5
1
0.5
0
−0.5
−1
−1.5
−2
−2.5
threitol [log2]
male
female

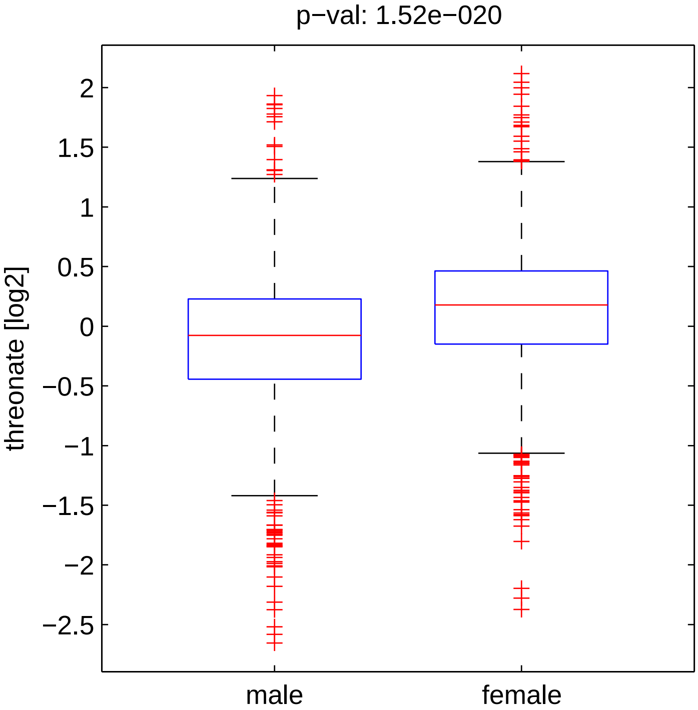
p−val: 1.52e−020
threonate [log2]
2
1.5
1
0.5
0
−0.5
−1
−1.5
−2
−2.5
male
female

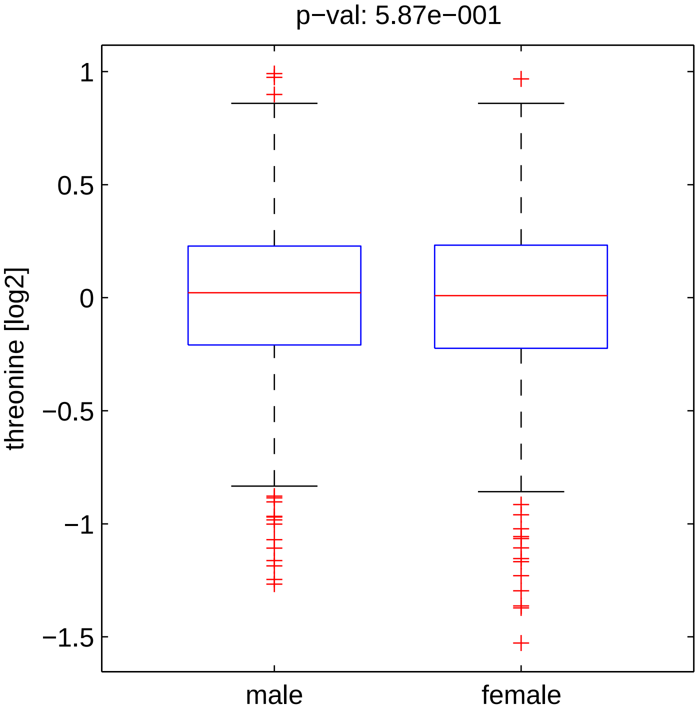

p−val: 5.87e−001
1
0.5
0
−0.5
−1
−1.5
threonine [log2]
male
female

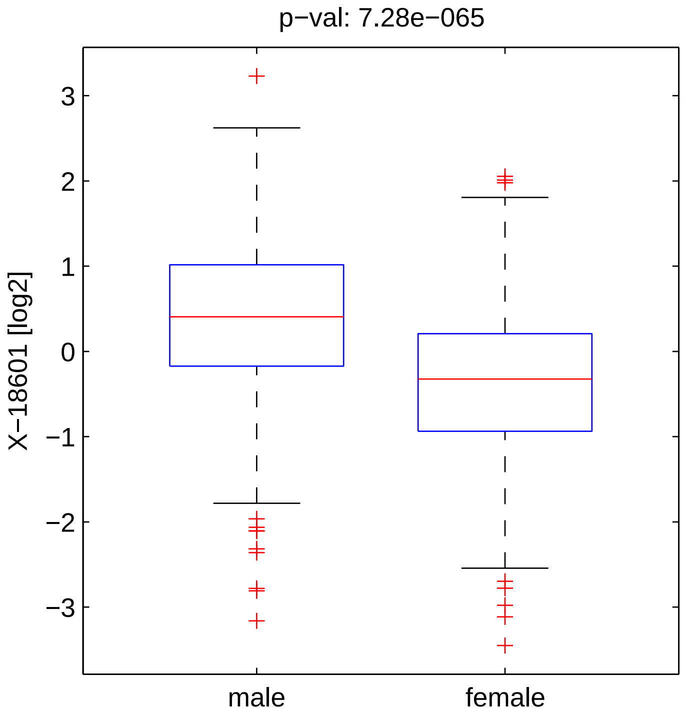
p−val: 7.28e−065
X−18601 [log2]
3
2
1
0
−1
−2
−3
male
female

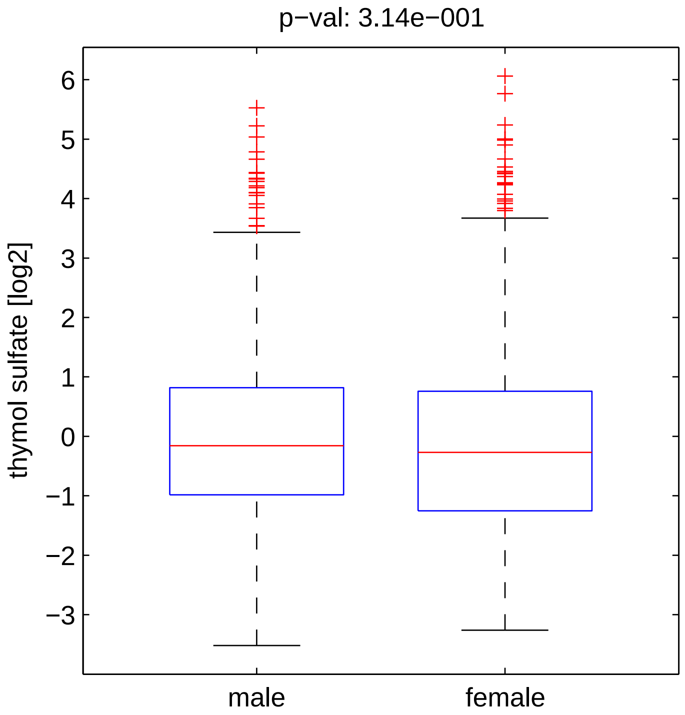

p−val: 3.14e−001
thymol sulfate [log2]
6
5
4
3
2
1
0
−1
−2
−3
male
female

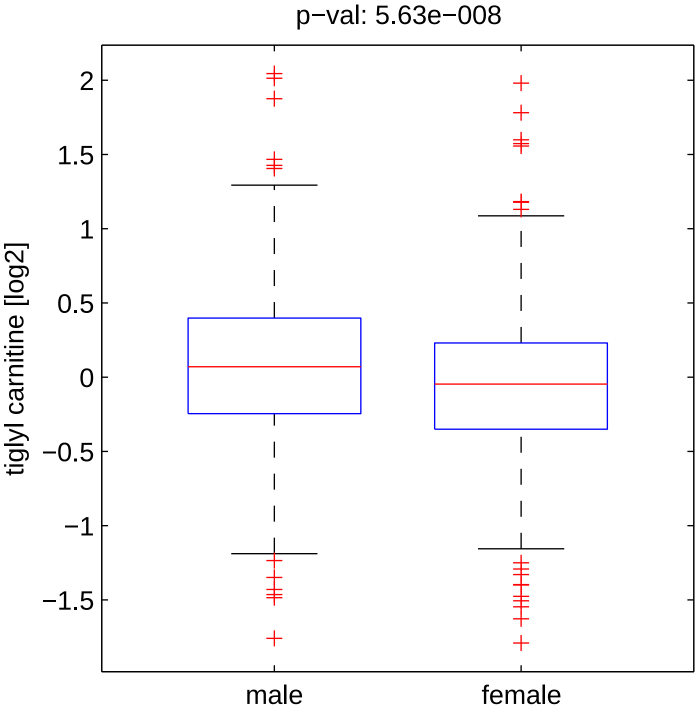
p−val: 5.63e−008
tiglyl carnitine [log2]
2
1.5
1
0.5
0
−0.5
−1
−1.5
male
female

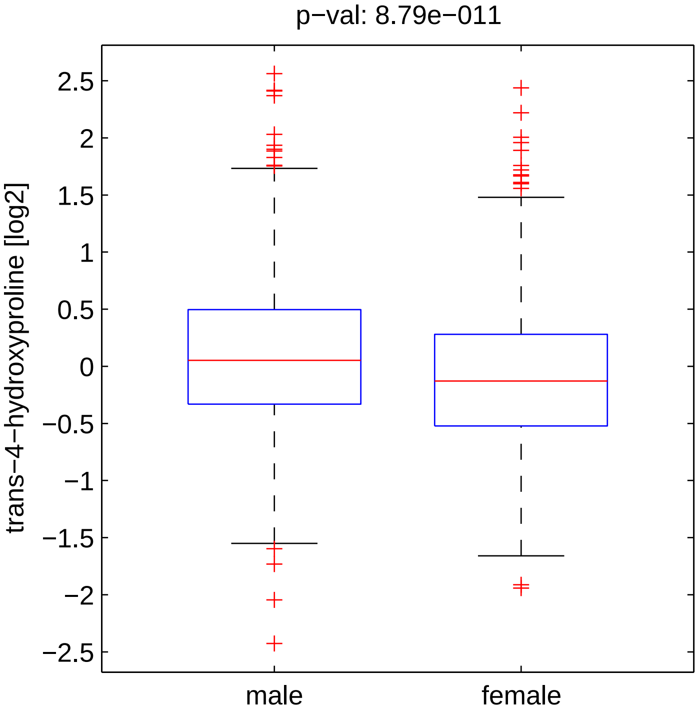
p−val: 8.79e−011
trans−4−hydroxyproline [log2]
2.5
2
1.5
1
0.5
0
−0.5
−1
−1.5
−2
−2.5
male
female

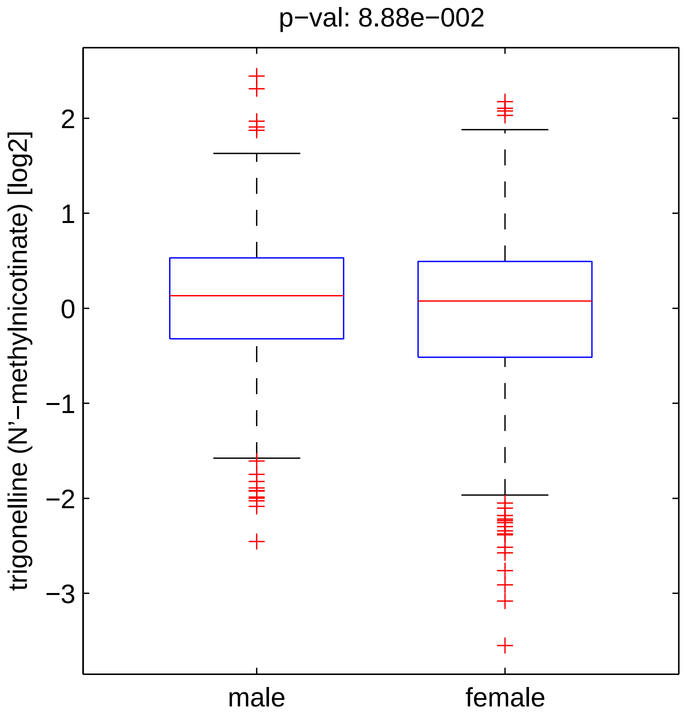

p−val: 8.88e−002
trigonelline (N'−methylnicotinate) [log2]
2
1
0
−1
−2
−3
male
female

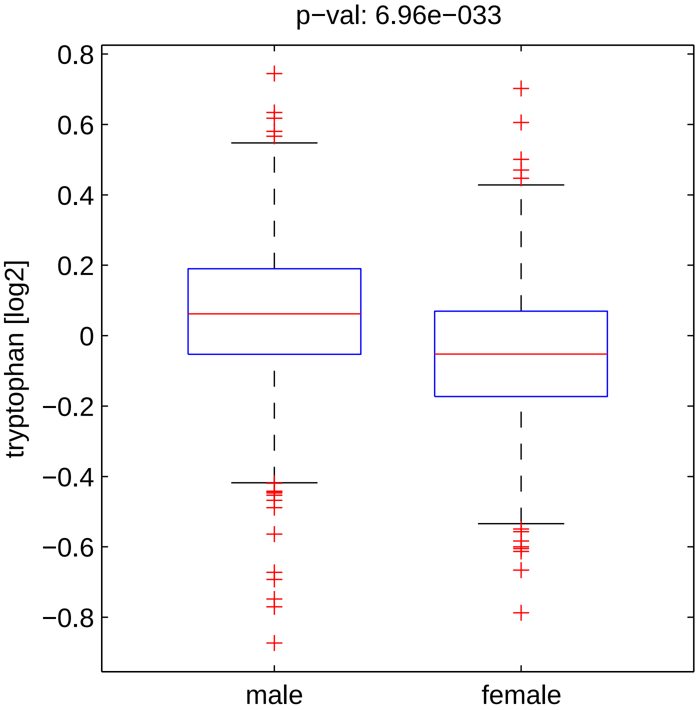
p−val: 6.96e−033
0.8
0.6
0.4
0.2
0
−0.2
−0.4
−0.6
−0.8
tryptophan [log2]
male
female

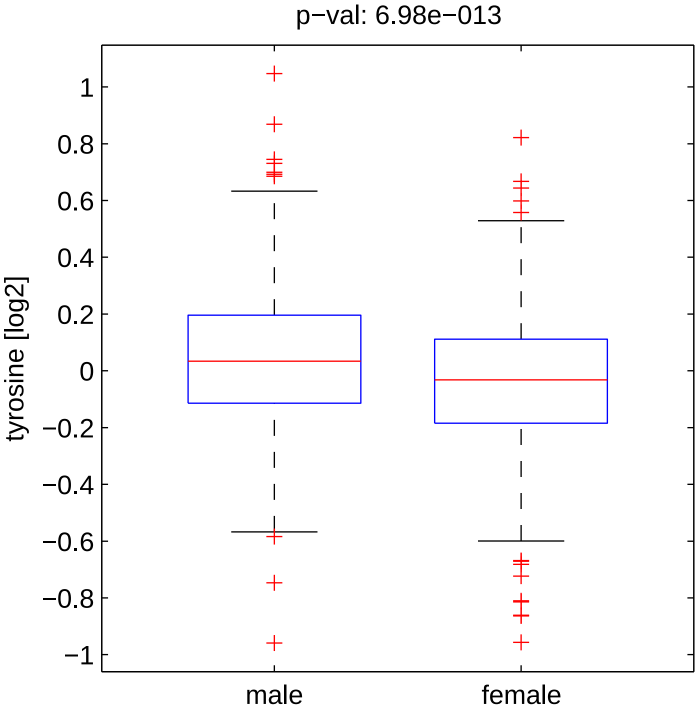
p−val: 6.98e−013
tyrosine [log2]
1
0.8
0.6
0.4
0.2
0
−0.2
−0.4
−0.6
−0.8
−1
male
female

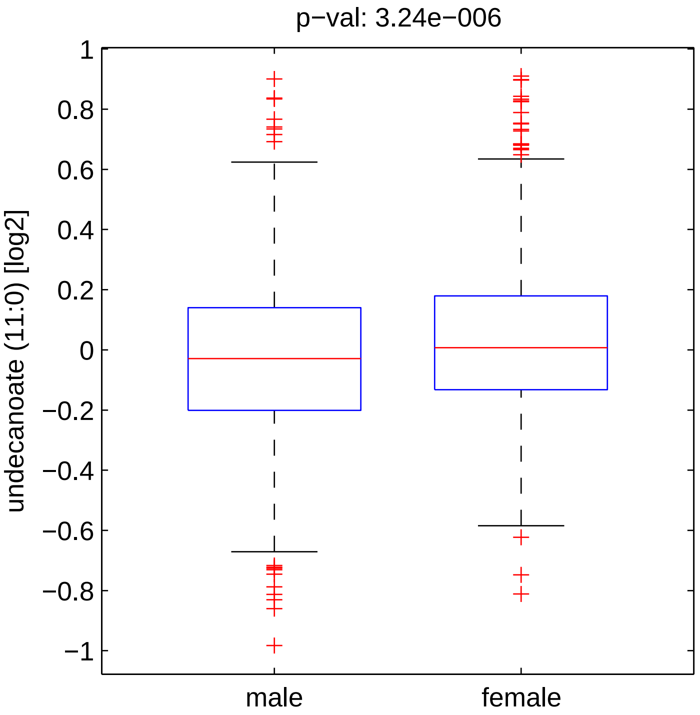

p−val: 3.24e−006
undecanoate (11:0) [log2]
1
0.8
0.6
0.4
0.2
0
−0.2
−0.4
−0.6
−0.8
−1
male
female

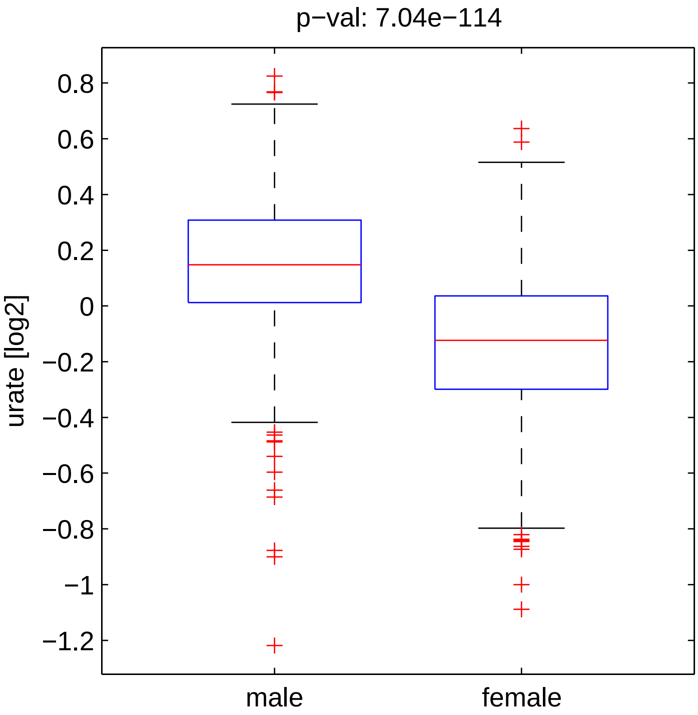

p−val: 7.04e−114
urate [log2]
0.8
0.6
0.4
0.2
0
−0.2
−0.4
−0.6
−0.8
−1
−1.2
male
female

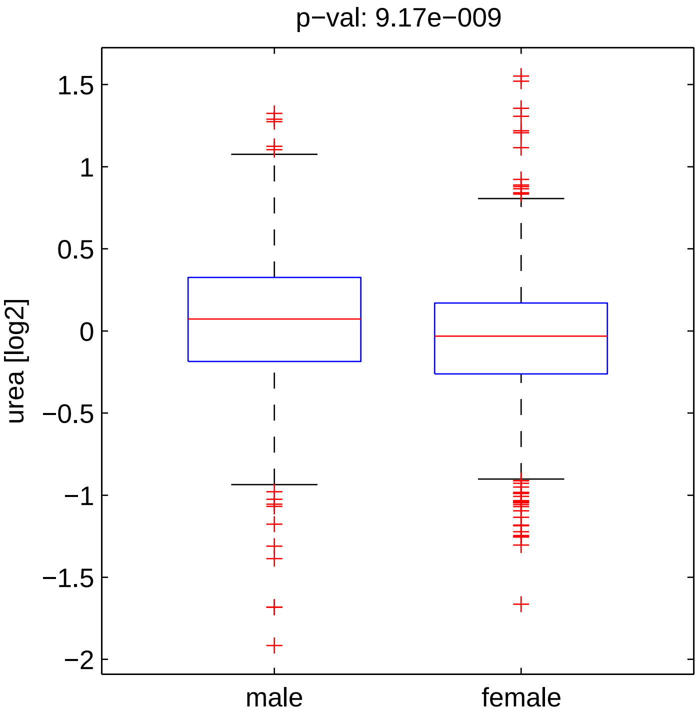
p−val: 9.17e−009
urea [log2]
1.5
1
0.5
0
−0.5
−1
−1.5
−2
male
female

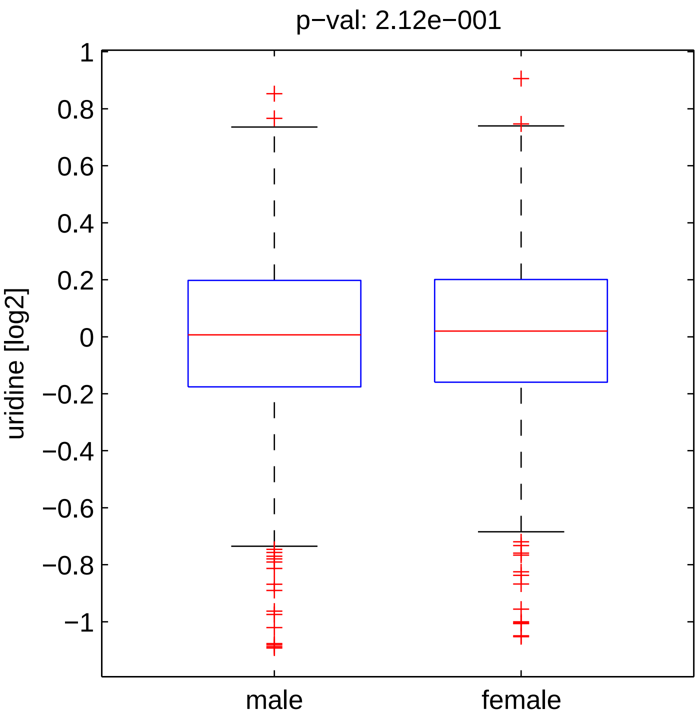

p−val: 2.12e−001
1
0.8
0.6
0.4
0.2
0
−0.2
−0.4
−0.6
−0.8
−1
uridine [log2]
male
female

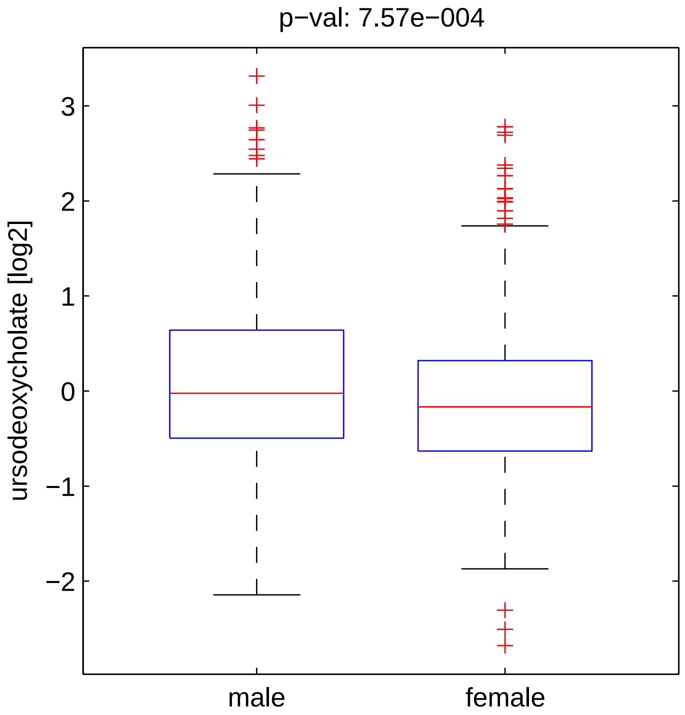

p−val: 7.57e−004
ursodeoxycholate [log2]
3
2
1
0
−1
−2
male
female

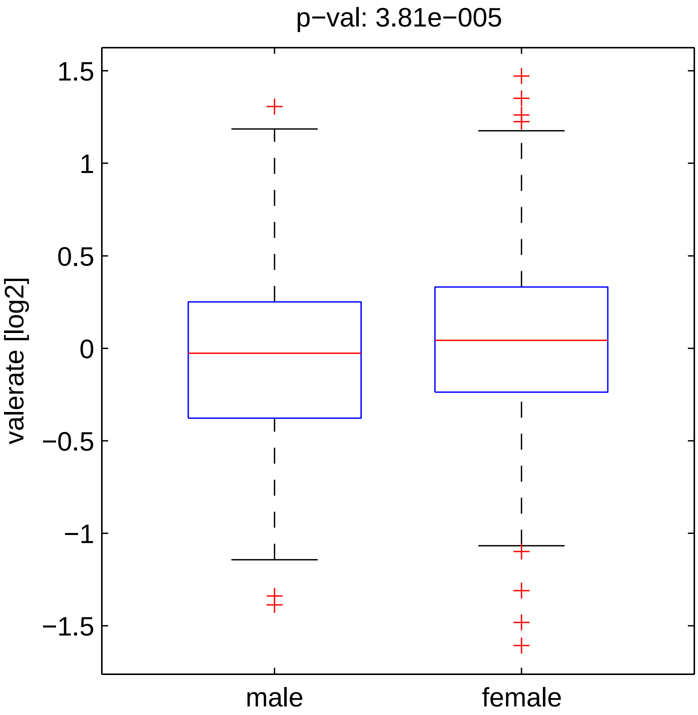
p−val: 3.81e−005
1.5
1
0.5
0
−0.5
−1
−1.5
valerate [log2]
male
female

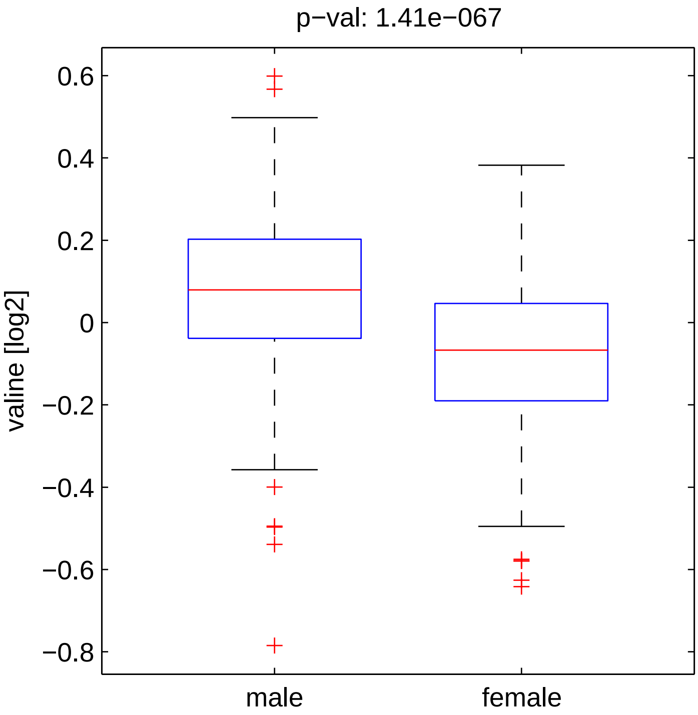
p−val: 1.41e−067
valine [log2]
0.6
0.4
0.2
0
−0.2
−0.4
−0.6
−0.8
male
female

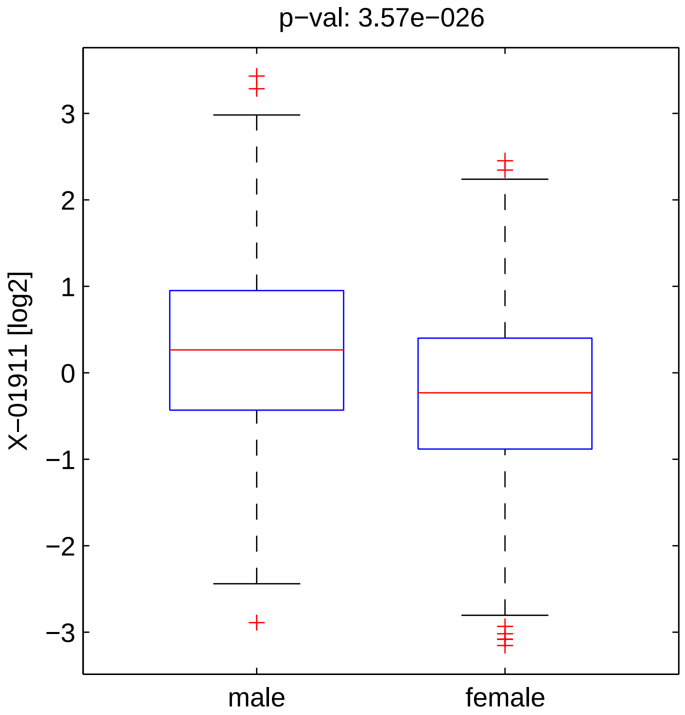
p−val: 3.57e−026
X−01911 [log2]
3
2
1
0
−1
−2
−3
male
female

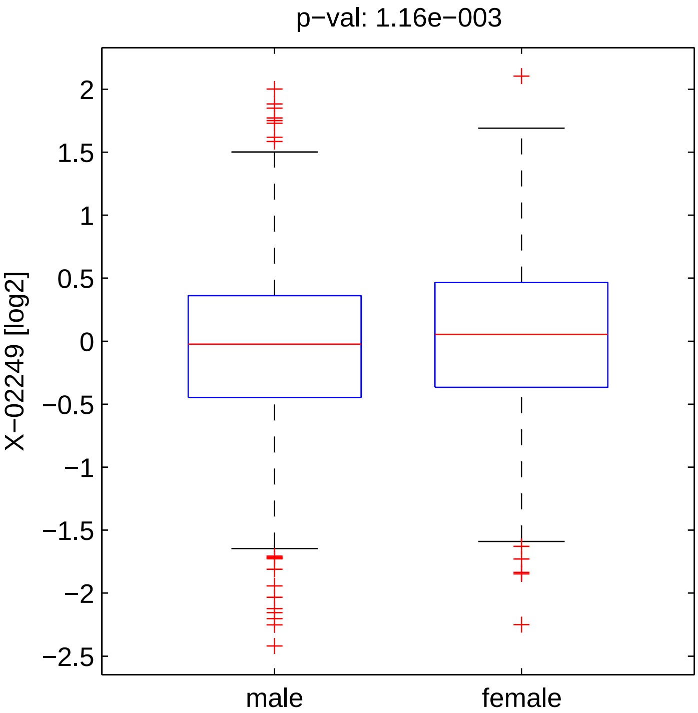
p−val: 1.16e−003
X−02249 [log2]
2
1.5
1
0.5
0
−0.5
−1
−1.5
−2
−2.5
male
female

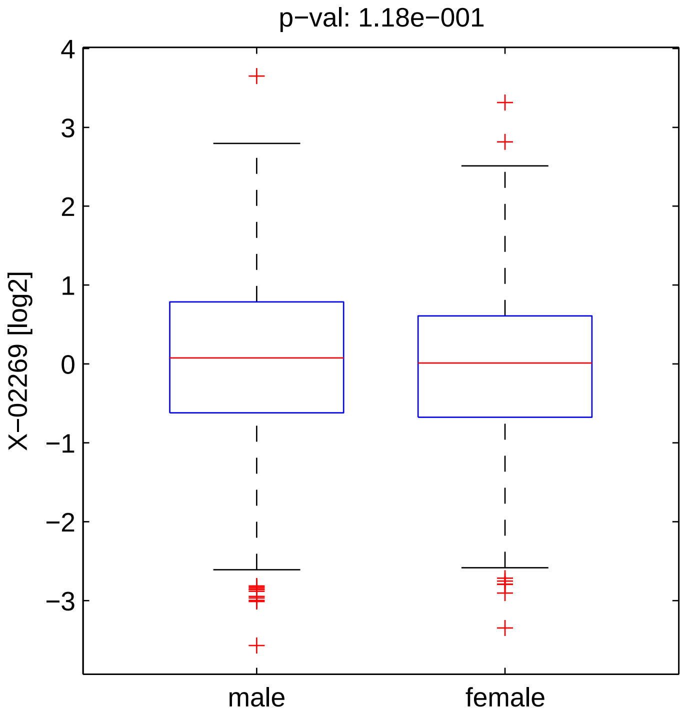

p−val: 1.18e−001
4
3
2
1
0
−1
−2
−3
X−02269 [log2]
male
female

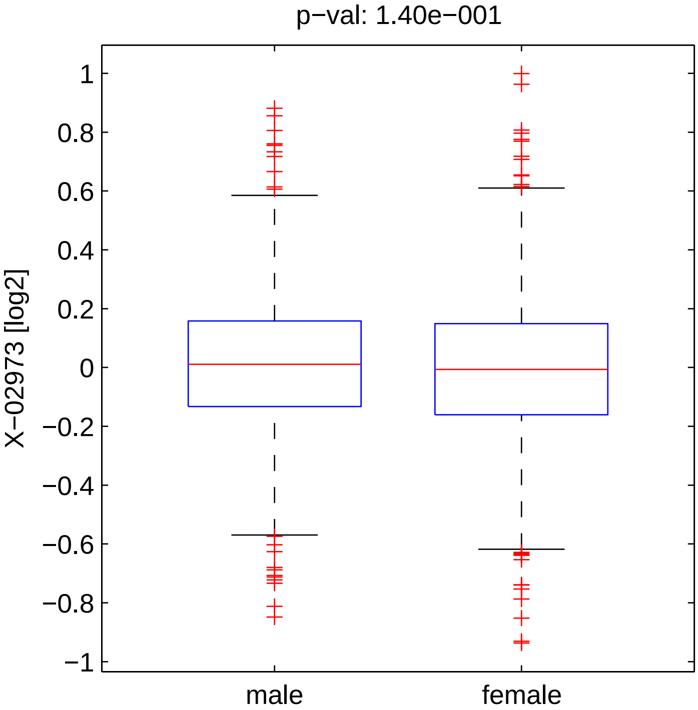
p−val: 1.40e−001
X−02973 [log2]
1
0.8
0.6
0.4
0.2
0
−0.2
−0.4
−0.6
−0.8
−1
male
female

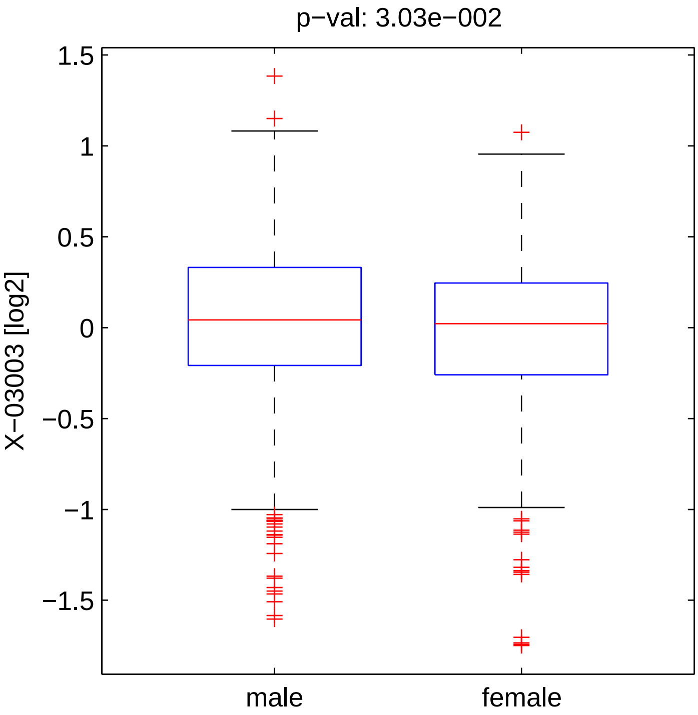
p−val: 3.03e−002
1.5
1
0.5
0
−0.5
−1
−1.5
X−03003 [log2]
male
female

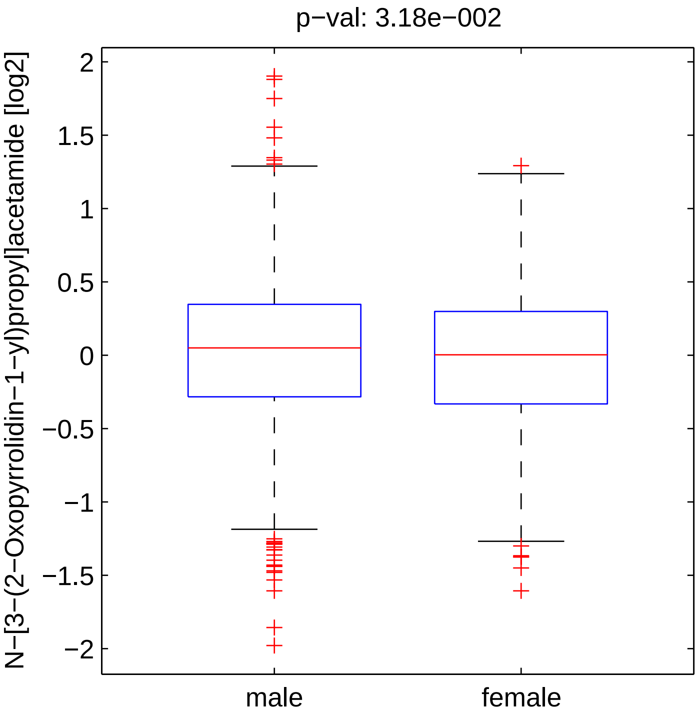

p−val: 3.18e−002
N−[3−(2−Oxopyrrolidin−1−yl)propyl]acetamide [log2]
2
1.5
1
0.5
0
−0.5
−1
−1.5
−2
male
female

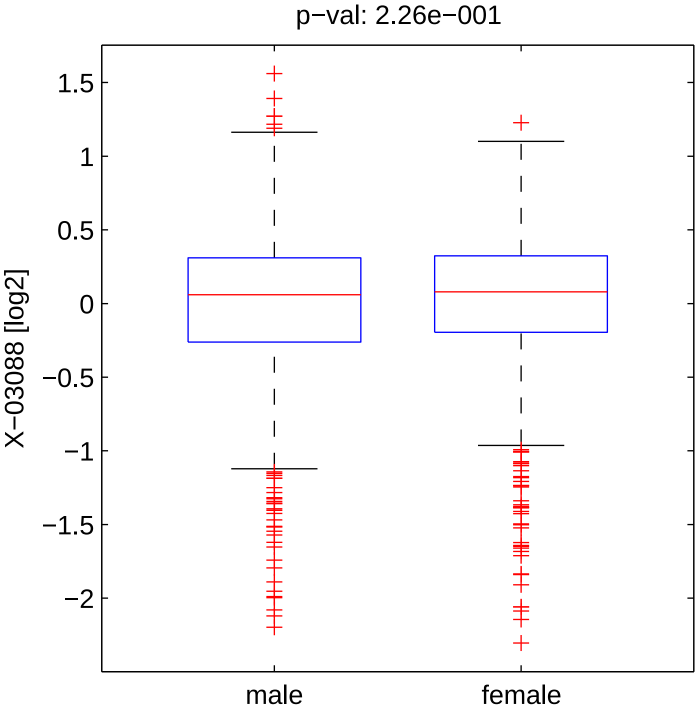
p−val: 2.26e−001
X−03088 [log2]
1.5
1
0.5
0
−0.5
−1
−1.5
−2
male
female

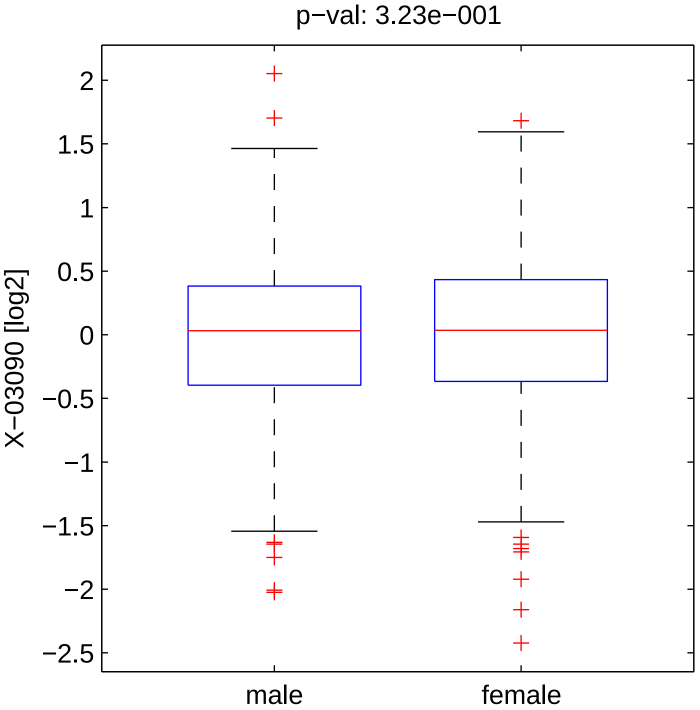
p−val: 3.23e−001
X−03090 [log2]
2
1.5
1
0.5
0
−0.5
−1
−1.5
−2
−2.5
male
female

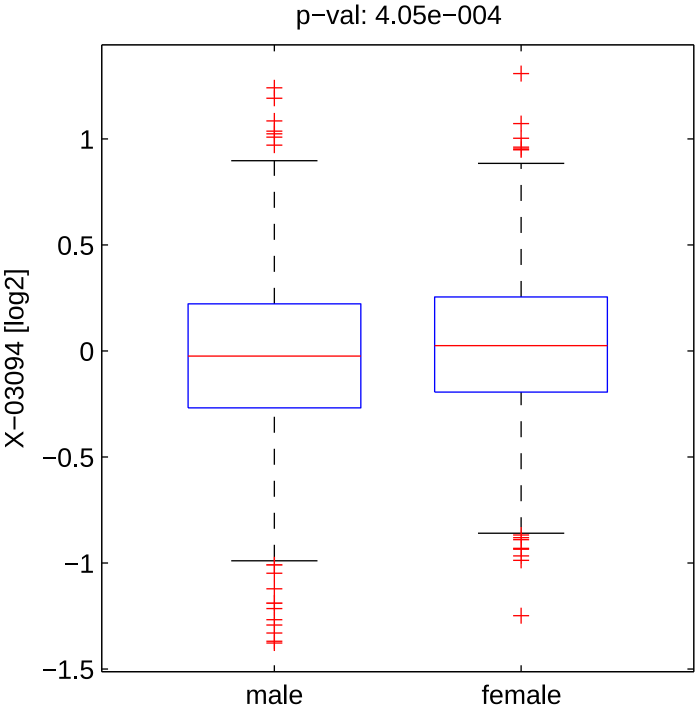

p−val: 4.05e−004
X−03094 [log2]
1
0.5
0
−0.5
−1
−1.5
male
female

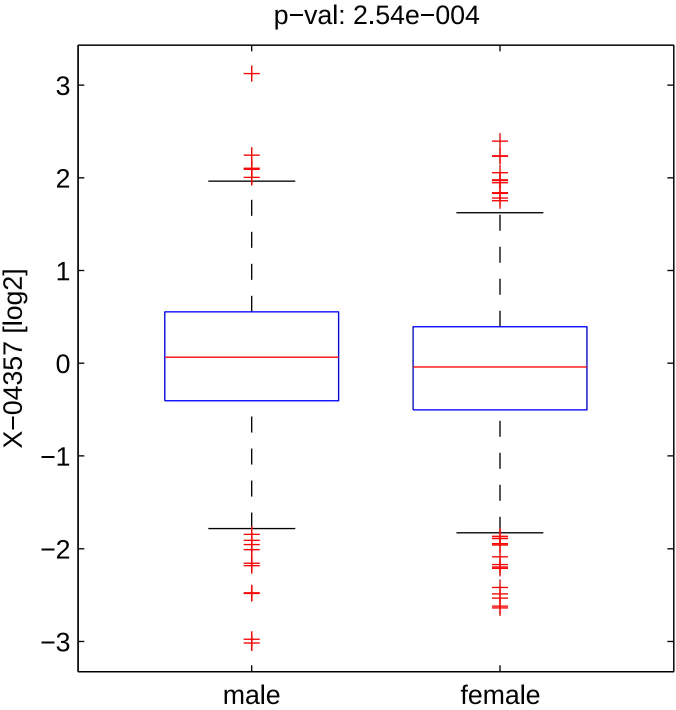

p−val: 2.54e−004
X−04357 [log2]
3
2
1
0
−1
−2
−3
male
female

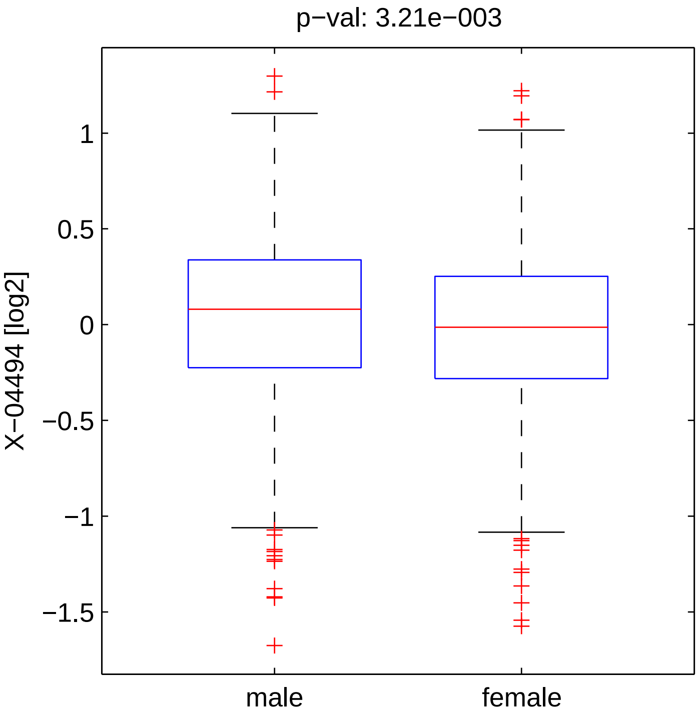

p−val: 3.21e−003
X−04494 [log2]
1
0.5
0
−0.5
−1
−1.5
male
female

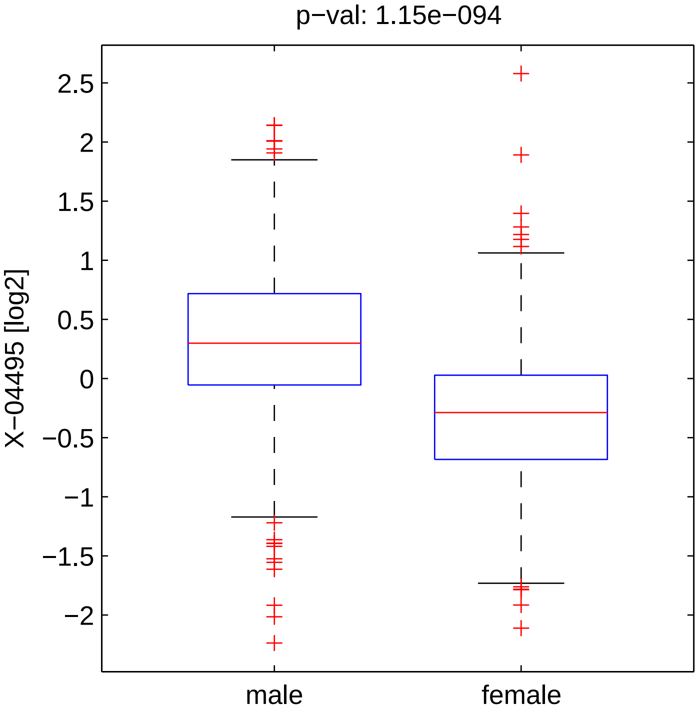
p−val: 1.15e−094
X−04495 [log2]
2.5
2
1.5
1
0.5
0
−0.5
−1
−1.5
−2
male
female

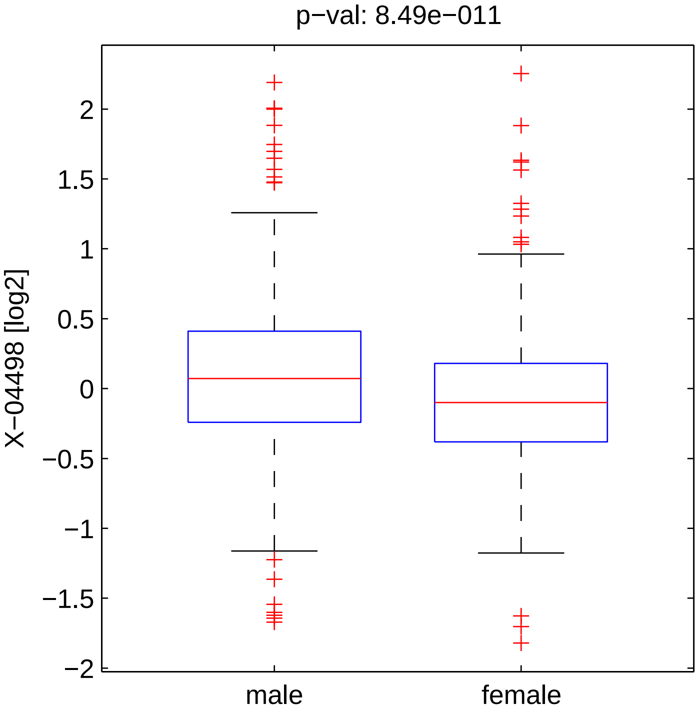

p−val: 8.49e−011
X−04498 [log2]
2
1.5
1
0.5
0
−0.5
−1
−1.5
−2
male
female

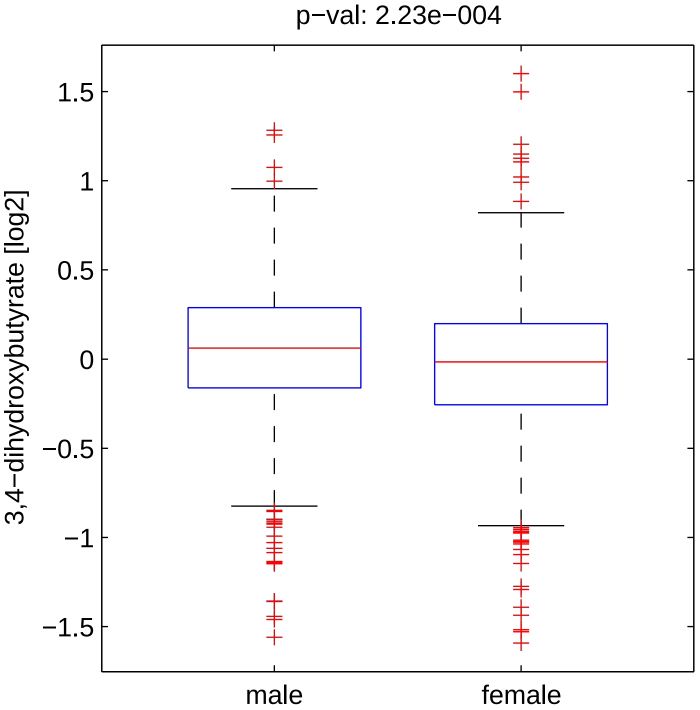
p−val: 2.23e−004
3,4−dihydroxybutyrate [log2]
1.5
1
0.5
0
−0.5
−1
−1.5
male
female

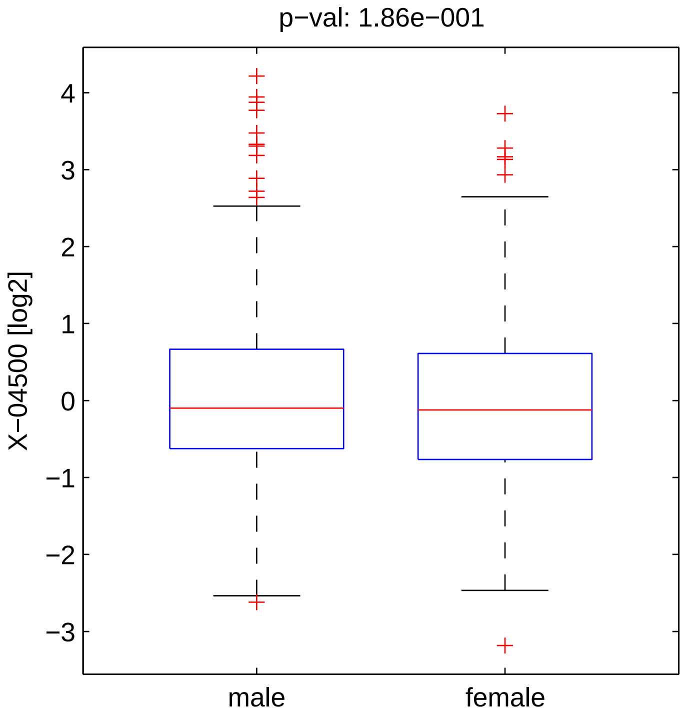
p−val: 1.86e−001
X−04500 [log2]
4
3
2
1
0
−1
−2
−3
male
female

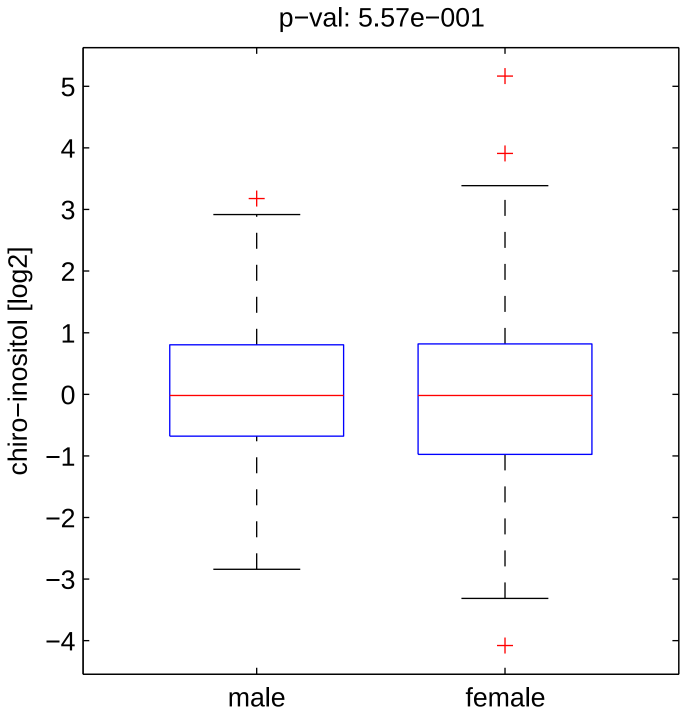
p−val: 5.57e−001
chiro−inositol [log2]
5
4
3
2
1
0
−1
−2
−3
−4
male
female

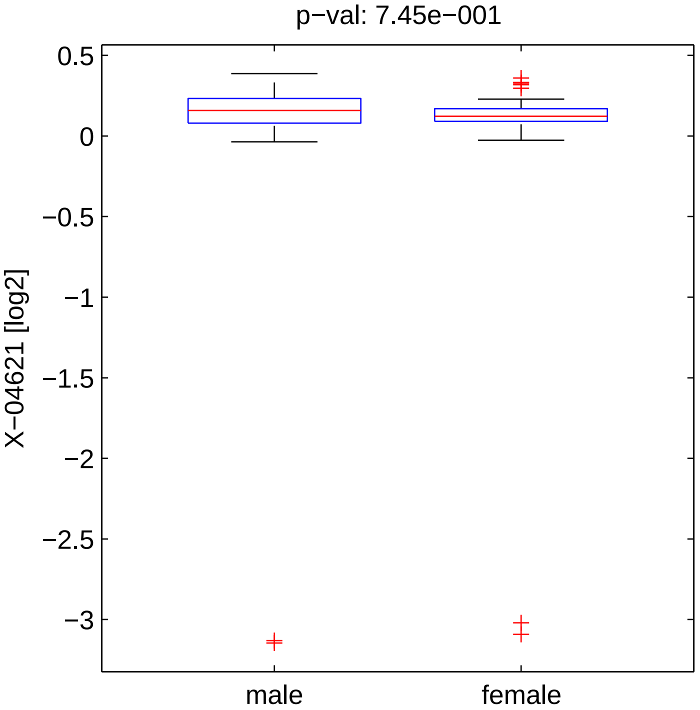

p−val: 7.45e−001
0.5
0
−0.5
−1
−1.5
−2
−2.5
−3
X−04621 [log2]
male
female

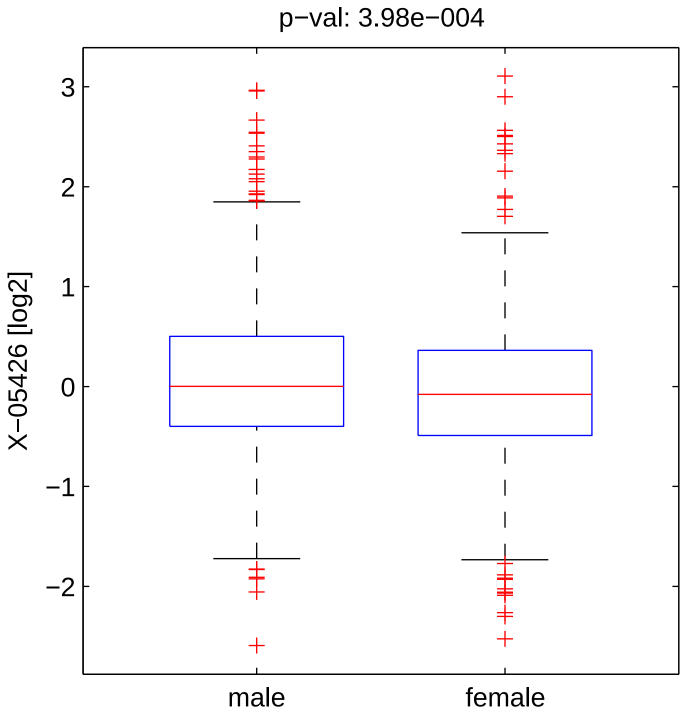
p−val: 3.98e−004
X−05426 [log2]
3
2
1
0
−1
−2
male
female

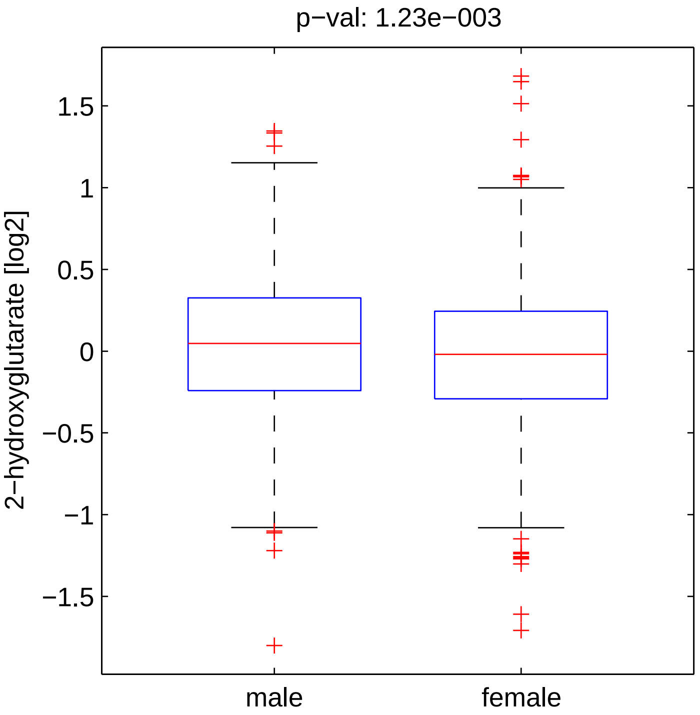
p−val: 1.23e−003
2−hydroxyglutarate [log2]
1.5
1
0.5
0
−0.5
−1
−1.5
male
female

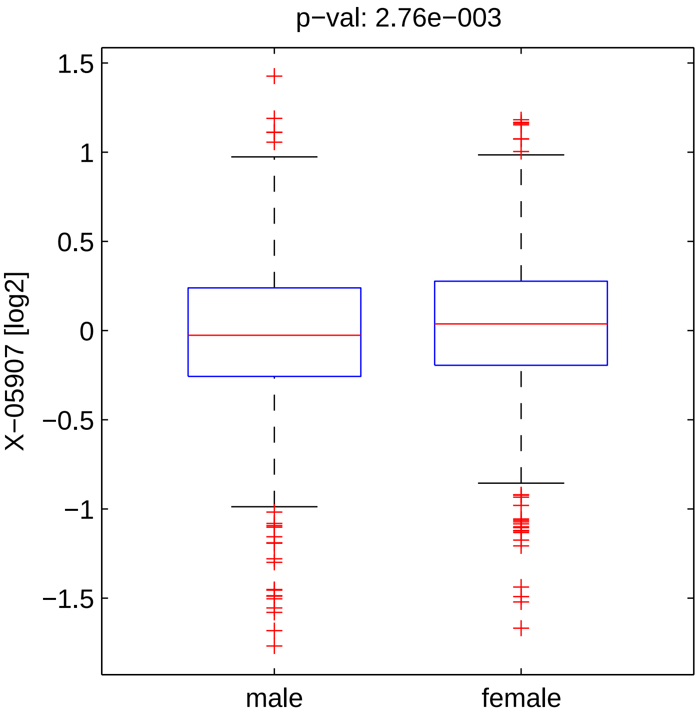
p−val: 2.76e−003
1.5
1
0.5
0
−0.5
−1
−1.5
X−05907 [log2]
male
female

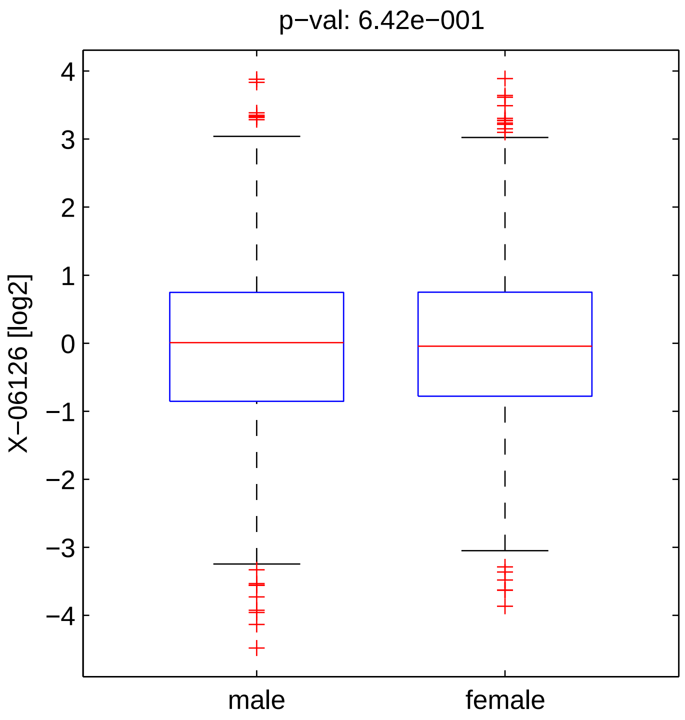

p−val: 6.42e−001
X−06126 [log2]
4
3
2
1
0
−1
−2
−3
−4
male
female

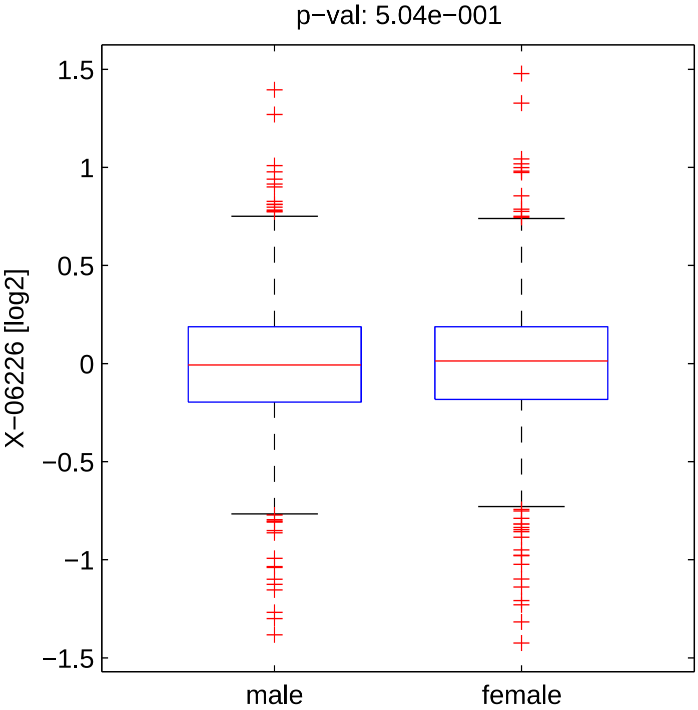

p−val: 5.04e−001
X−06226 [log2]
1.5
1
0.5
0
−0.5
−1
−1.5
male
female

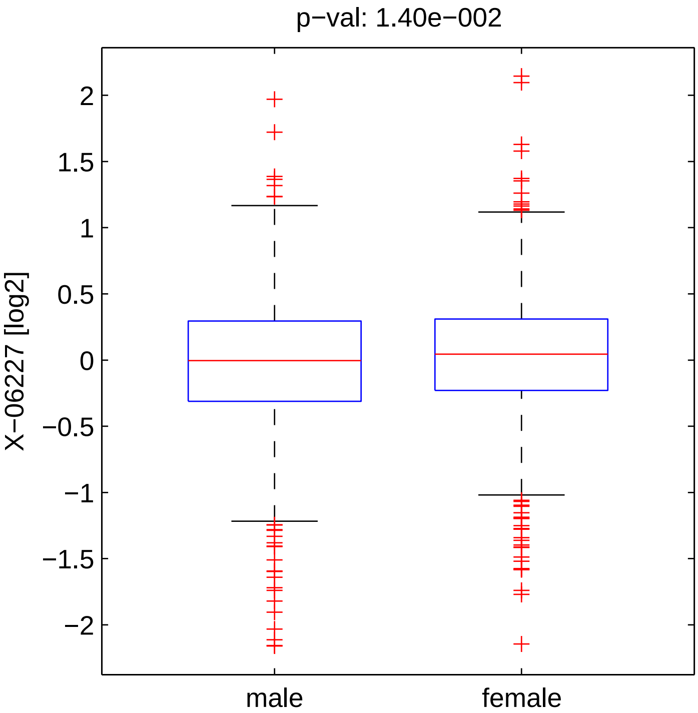
p−val: 1.40e−002
X−06227 [log2]
2
1.5
1
0.5
0
−0.5
−1
−1.5
−2
male
female

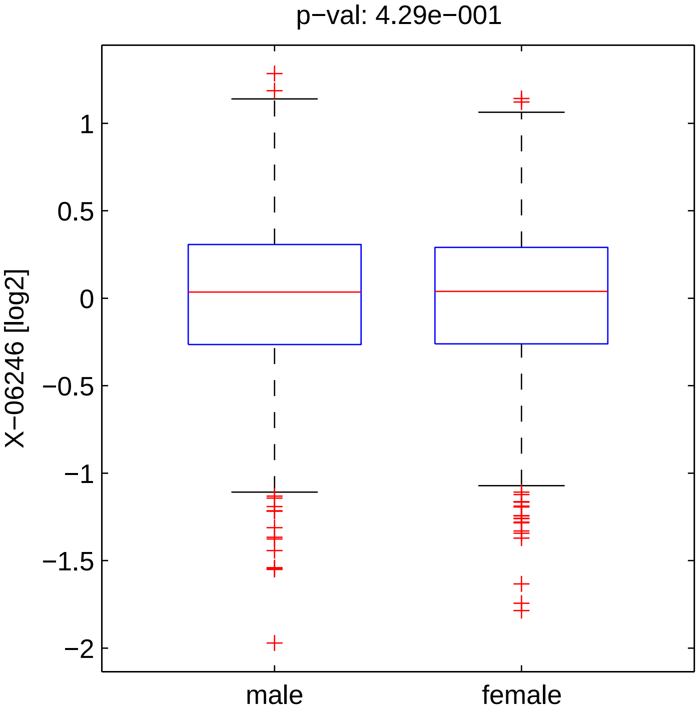
p−val: 4.29e−001
X−06246 [log2]
1
0.5
0
−0.5
−1
−1.5
−2
male
female

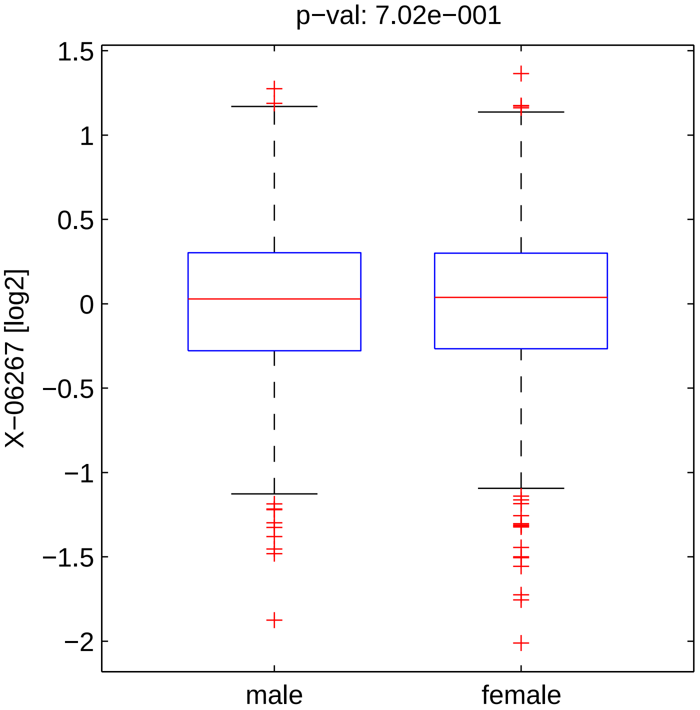

p−val: 7.02e−001
X−06267 [log2]
1.5
1
0.5
0
−0.5
−1
−1.5
−2
male
female

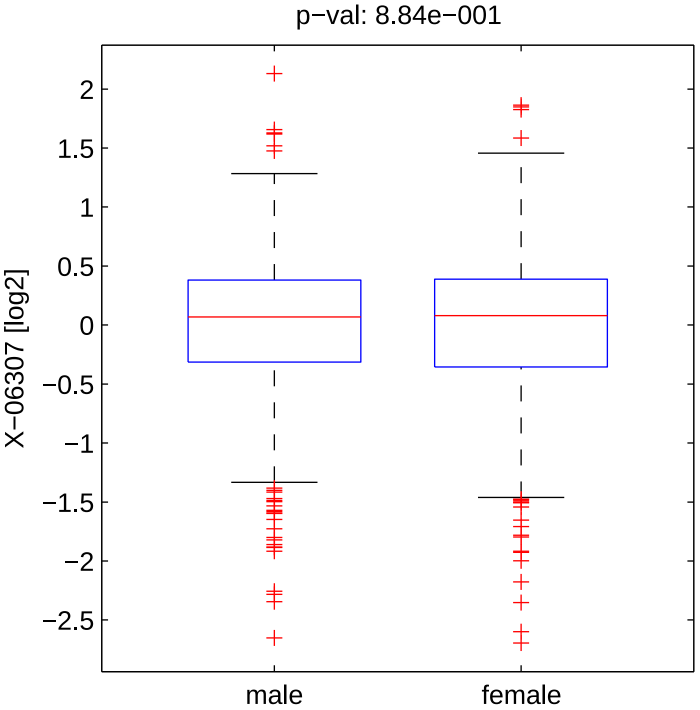
p−val: 8.84e−001
X−06307 [log2]
2
1.5
1
0.5
0
−0.5
−1
−1.5
−2
−2.5
male
female

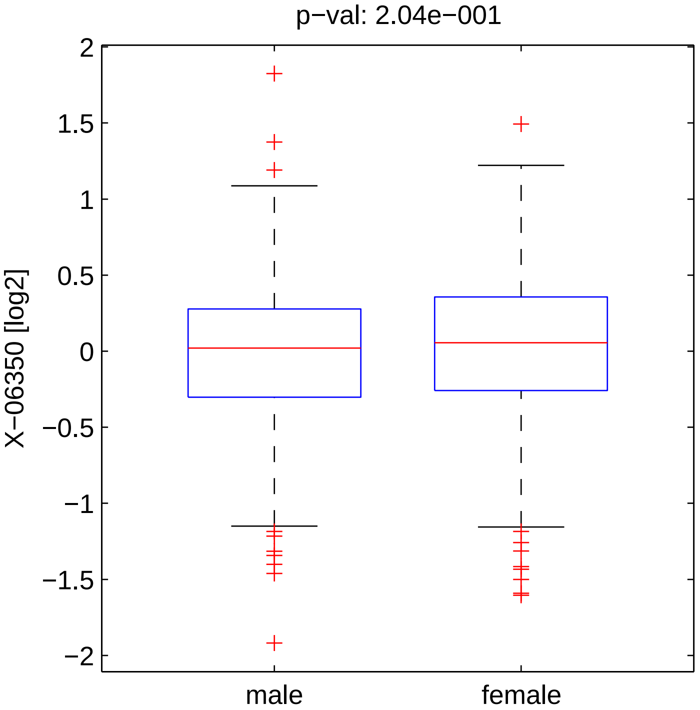

p−val: 2.04e−001
2
1.5
1
0.5
0
−0.5
−1
−1.5
−2
X−06350 [log2]
male
female

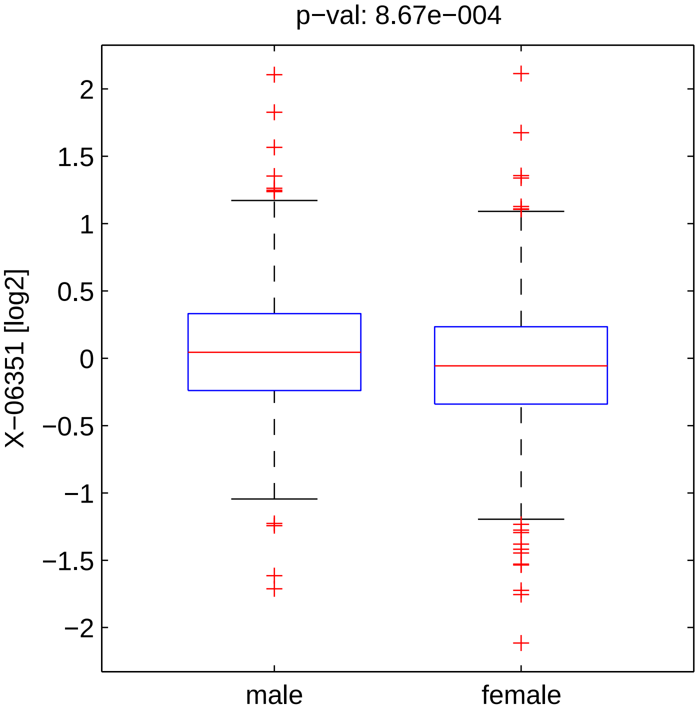

p−val: 8.67e−004
2
1.5
1
0.5
0
−0.5
−1
−1.5
−2
X−06351 [log2]
male
female

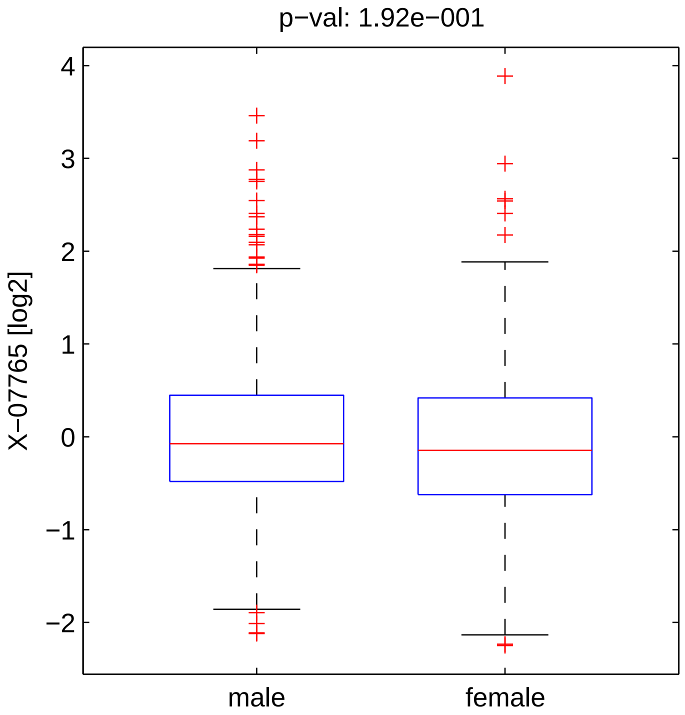

p−val: 1.92e−001
X−07765 [log2]
4
3
2
1
0
−1
−2
male
female

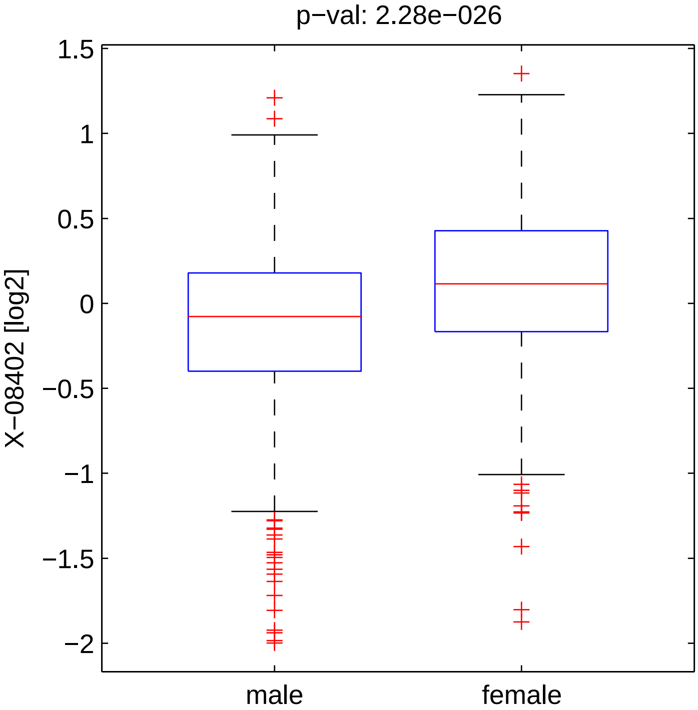

p−val: 2.28e−026
1.5
1
0.5
0
−0.5
−1
−1.5
−2
X−08402 [log2]
male
female

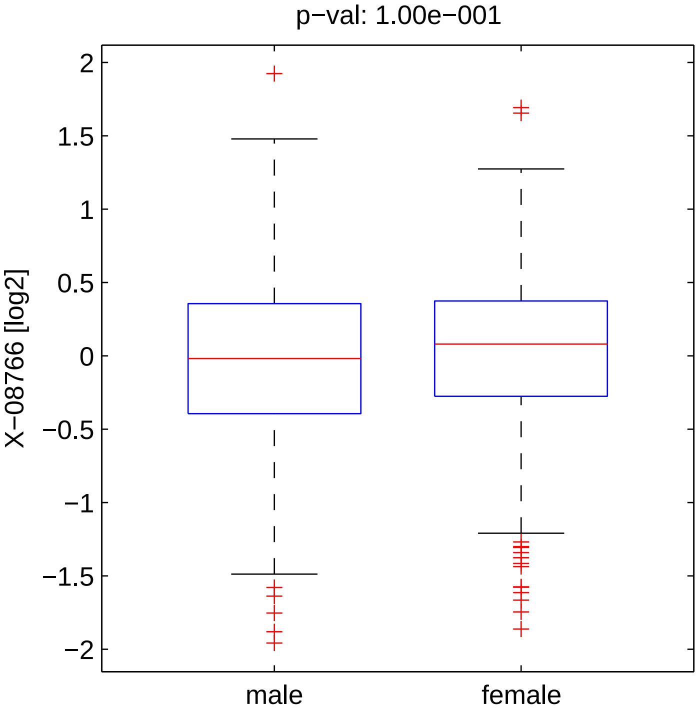

p−val: 1.00e−001
X−08766 [log2]
2
1.5
1
0.5
0
−0.5
−1
−1.5
−2
male
female

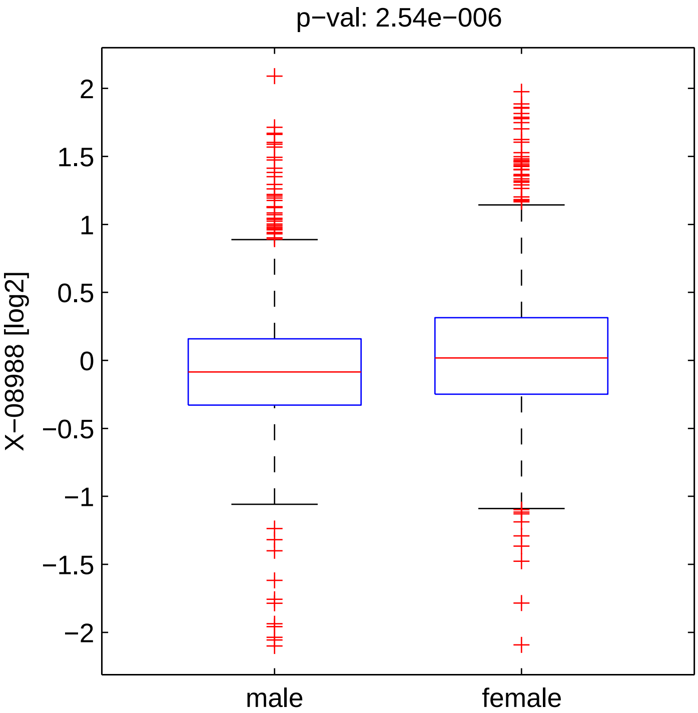
p−val: 2.54e−006
X−08988 [log2]
2
1.5
1
0.5
0
−0.5
−1
−1.5
−2
male
female

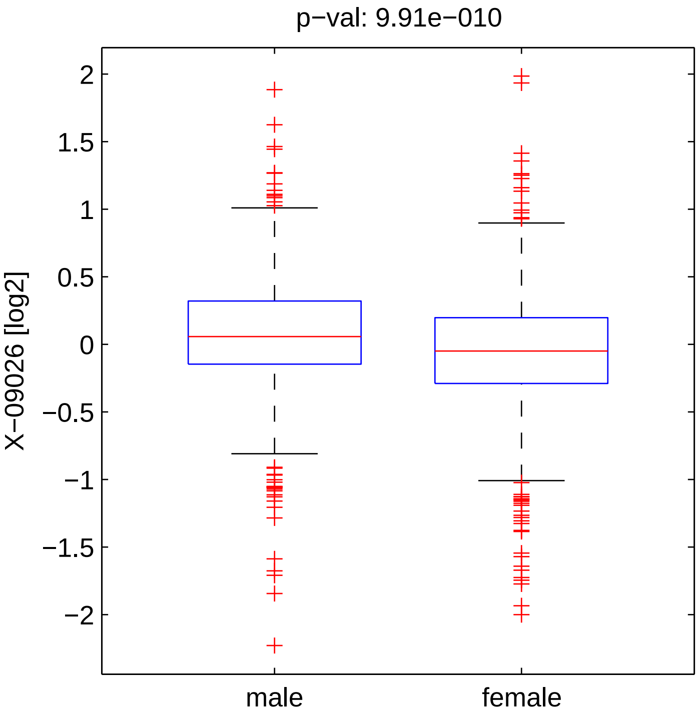
p−val: 9.91e−010
X−09026 [log2]
2
1.5
1
0.5
0
−0.5
−1
−1.5
−2
male
female

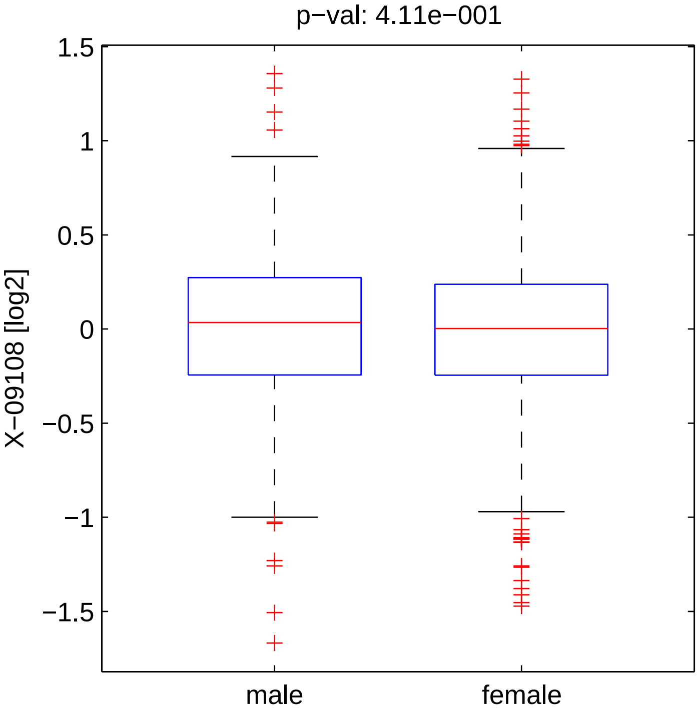

p−val: 4.11e−001
1.5
1
0.5
0
−0.5
−1
−1.5
X−09108 [log2]
male
female

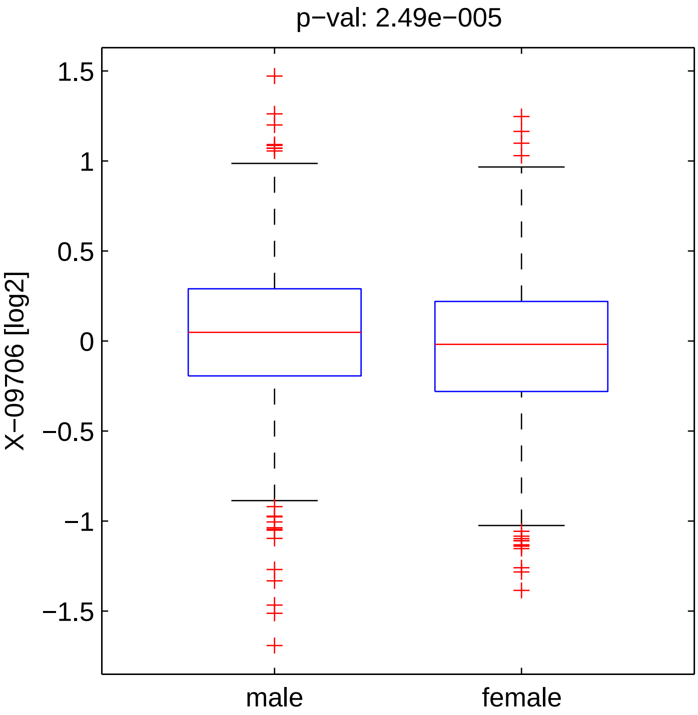
p−val: 2.49e−005
X−09706 [log2]
1.5
1
0.5
0
−0.5
−1
−1.5
male
female

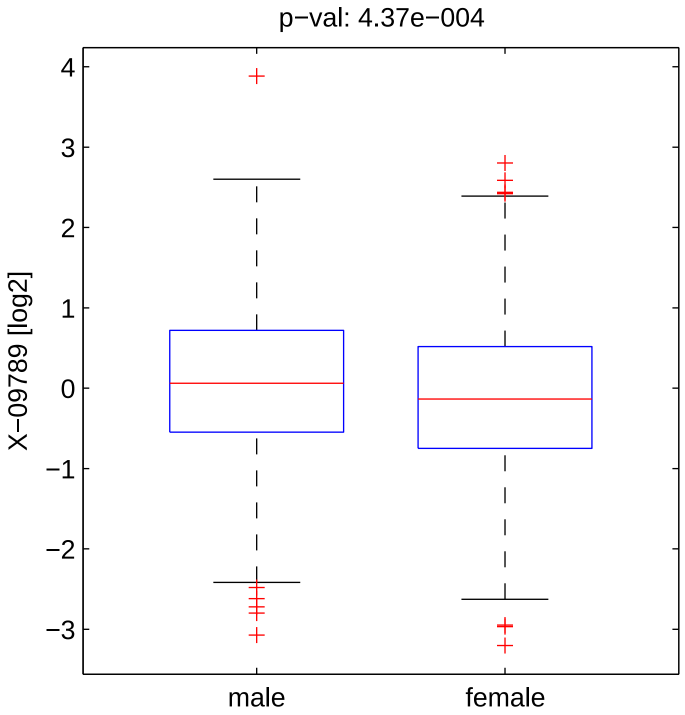

p−val: 4.37e−004
X−09789 [log2]
4
3
2
1
0
−1
−2
−3
male
female

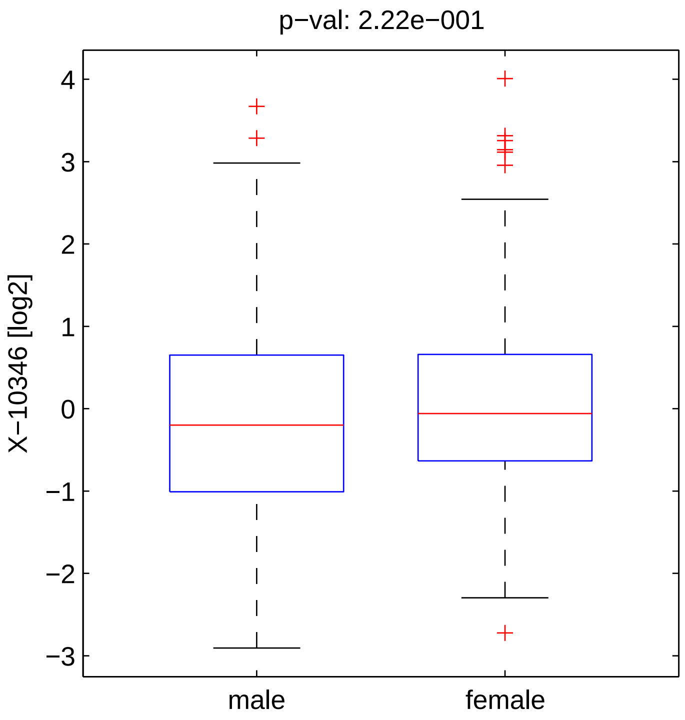

p−val: 2.22e−001
4
3
2
1
0
−1
−2
−3
X−10346 [log2]
male
female

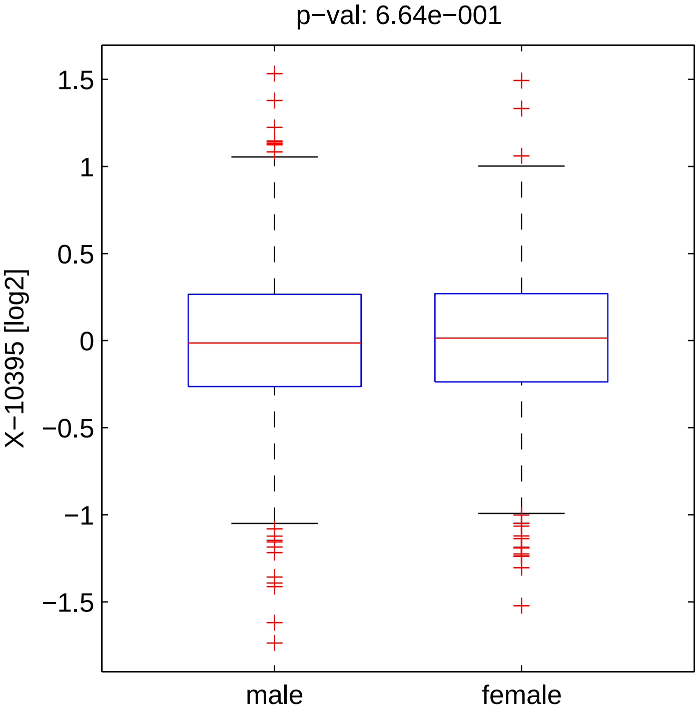
p−val: 6.64e−001
X−10395 [log2]
1.5
1
0.5
0
−0.5
−1
−1.5
male
female

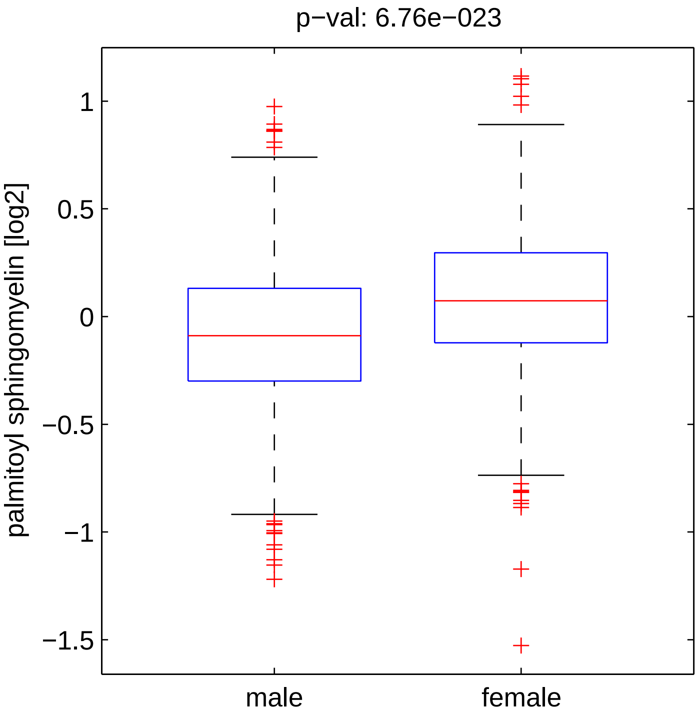
p−val: 6.76e−023
palmitoyl sphingomyelin [log2]
1
0.5
0
−0.5
−1
−1.5
male
female

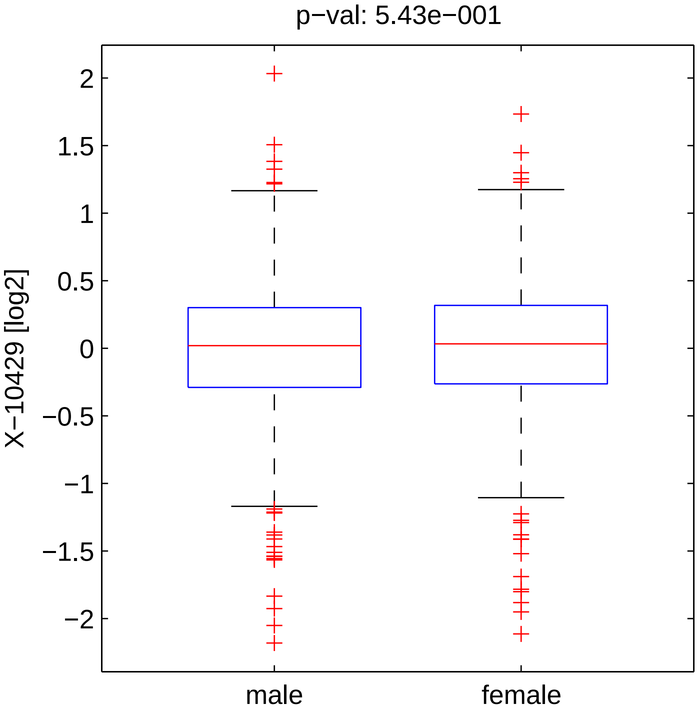

p−val: 5.43e−001
X−10429 [log2]
2
1.5
1
0.5
0
−0.5
−1
−1.5
−2
male
female

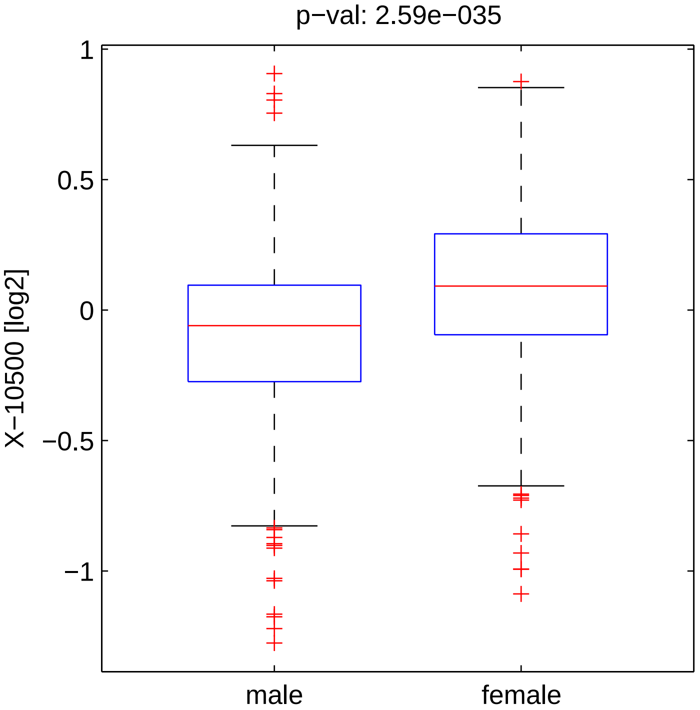
p−val: 2.59e−035
1
0.5
0
−0.5
−1
X−10500 [log2]
male
female

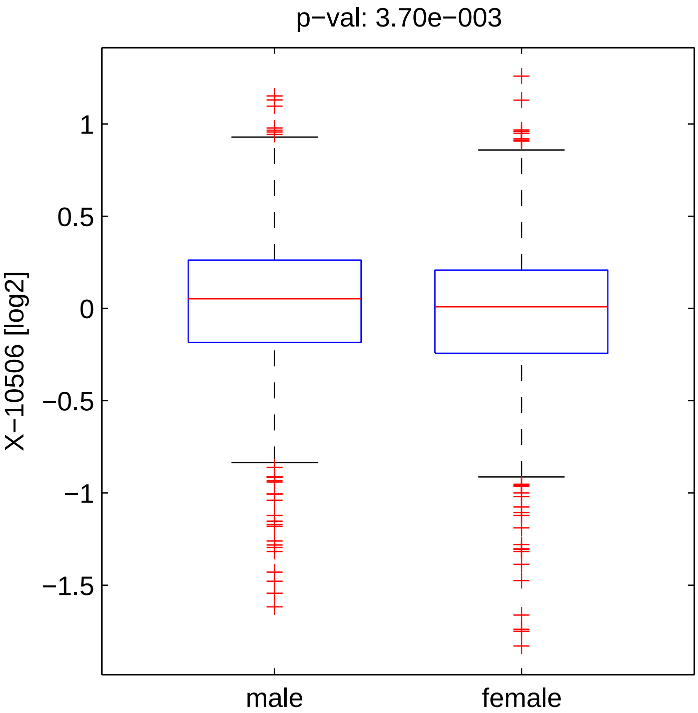
p−val: 3.70e−003
X−10506 [log2]
1
0.5
0
−0.5
−1
−1.5
male
female

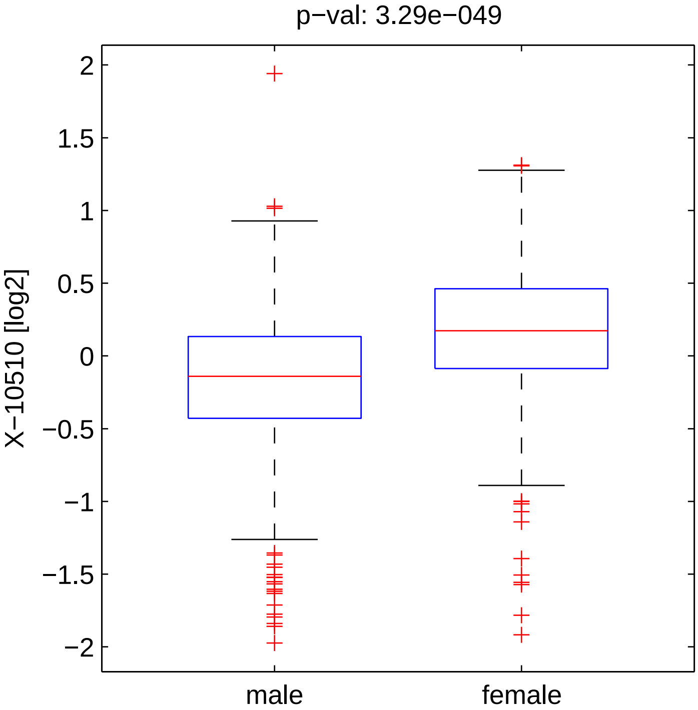
p−val: 3.29e−049
X−10510 [log2]
2
1.5
1
0.5
0
−0.5
−1
−1.5
−2
male
female

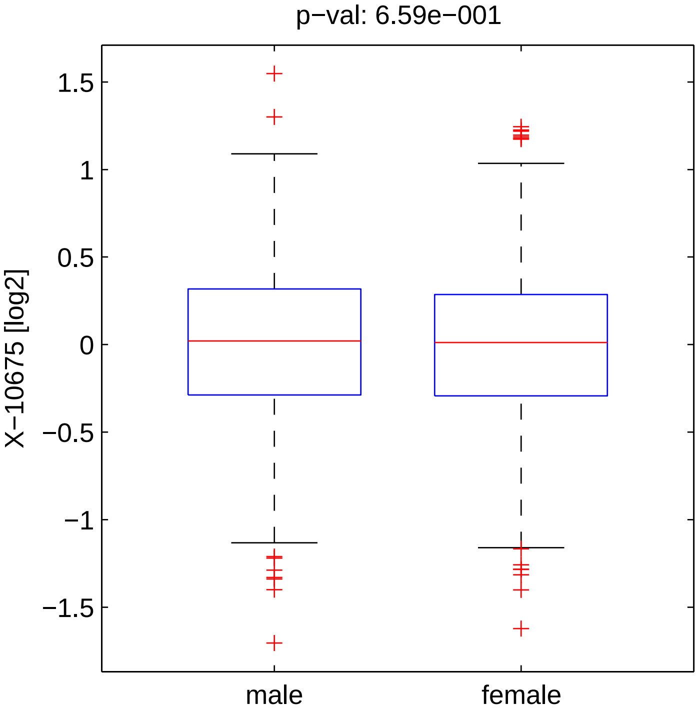

p−val: 6.59e−001
X−10675 [log2]
1.5
1
0.5
0
−0.5
−1
−1.5
male
female

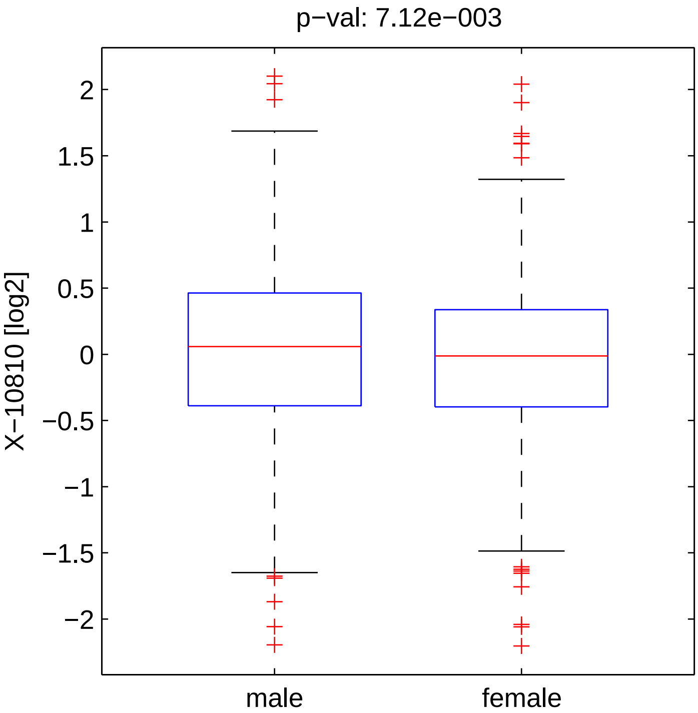
p−val: 7.12e−003
X−10810 [log2]
2
1.5
1
0.5
0
−0.5
−1
−1.5
−2
male
female

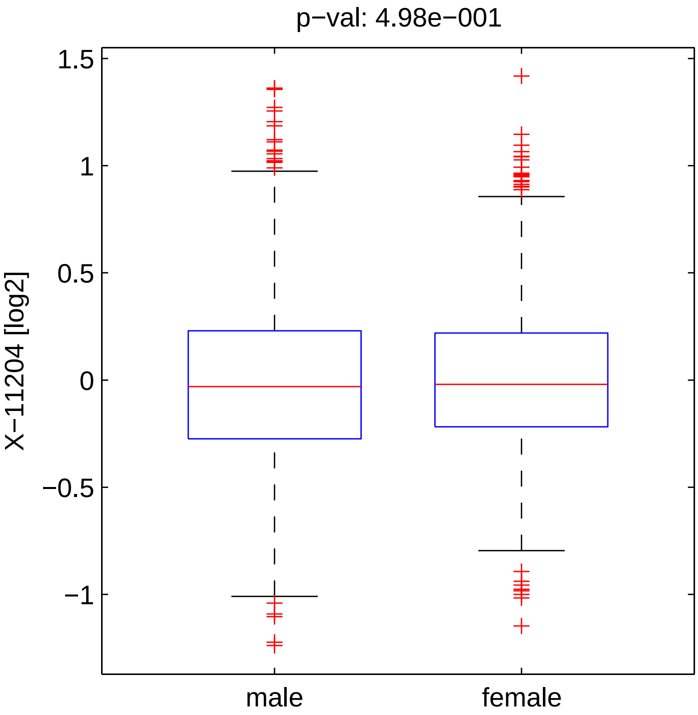

p−val: 4.98e−001
1.5
1
0.5
0
−0.5
−1
X−11204 [log2]
male
female

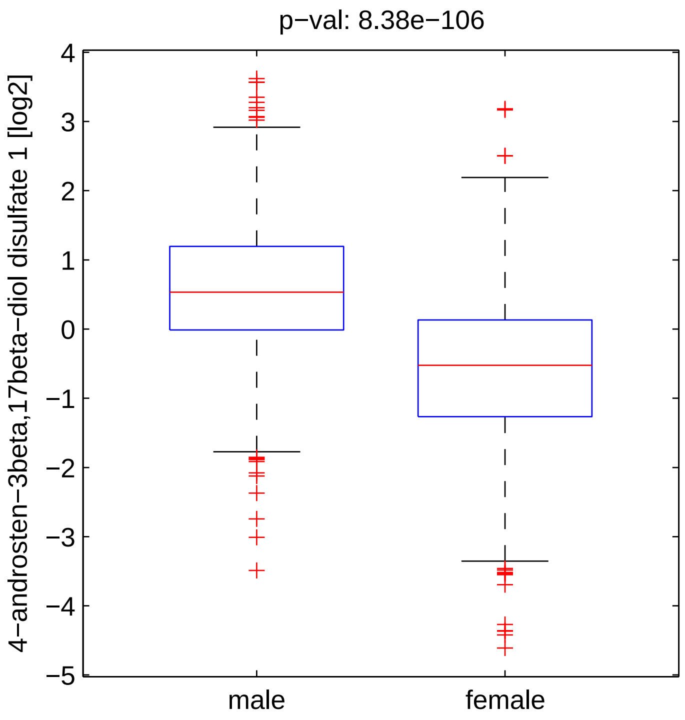

p−val: 8.38e−106
4−androsten−3beta,17beta−diol disulfate 1 [log2]
4
3
2
1
0
−1
−2
−3
−4
−5
male
female

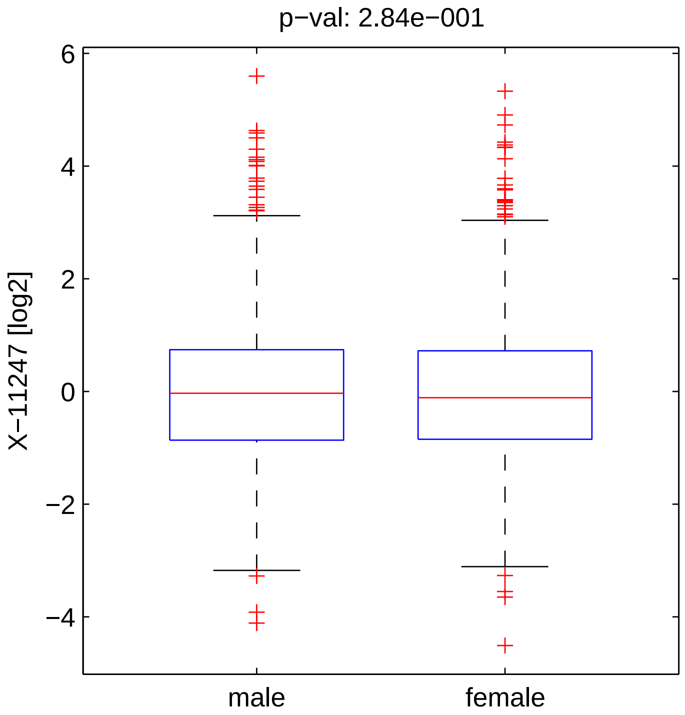
p−val: 2.84e−001
6
4
2
0
−2
−4
X−11247 [log2]
male
female

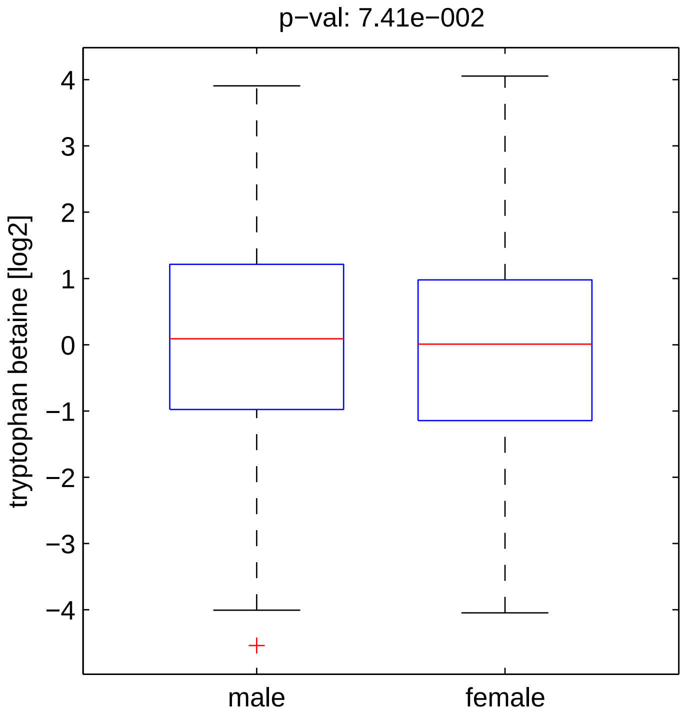

p−val: 7.41e−002
tryptophan betaine [log2]
4
3
2
1
0
−1
−2
−3
−4
male
female

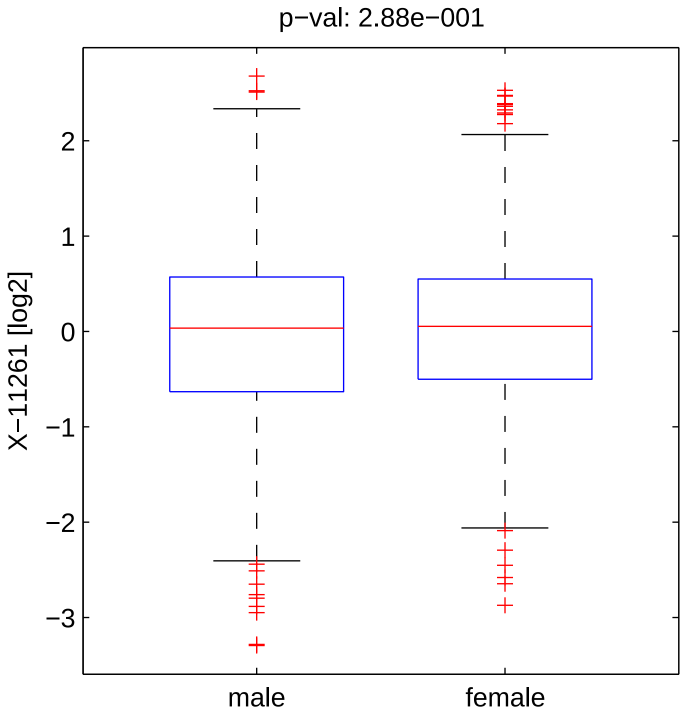
p−val: 2.88e−001
X−11261 [log2]
2
1
0
−1
−2
−3
male
female

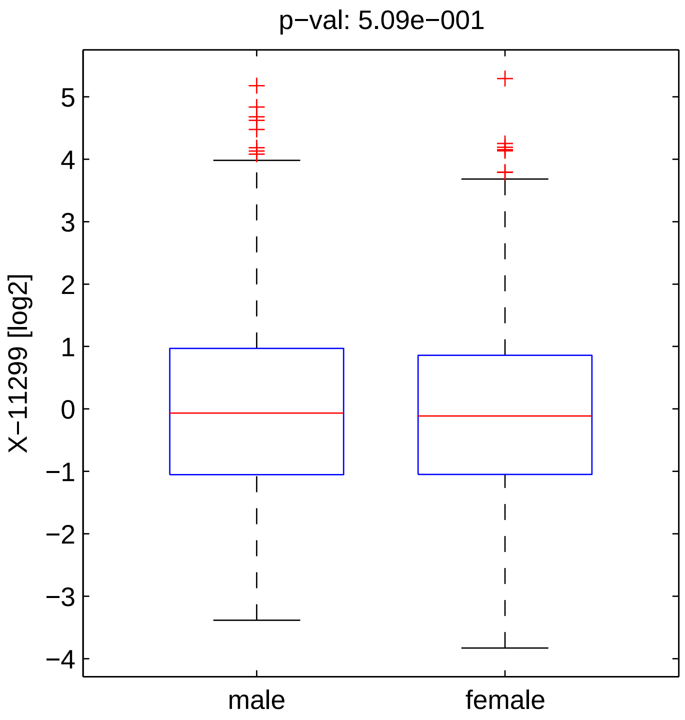

p−val: 5.09e−001
X−11299 [log2]
5
4
3
2
1
0
−1
−2
−3
−4
male
female

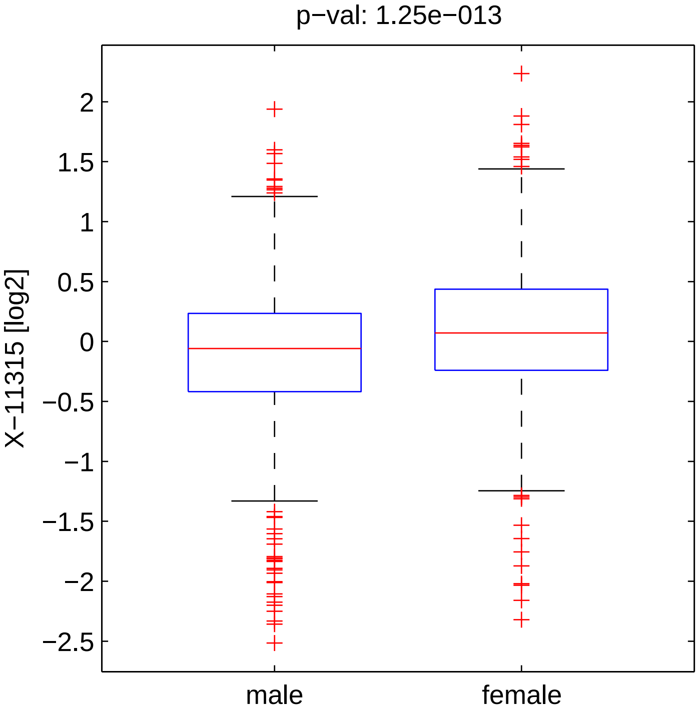
p−val: 1.25e−013
X−11315 [log2]
2
1.5
1
0.5
0
−0.5
−1
−1.5
−2
−2.5
male
female

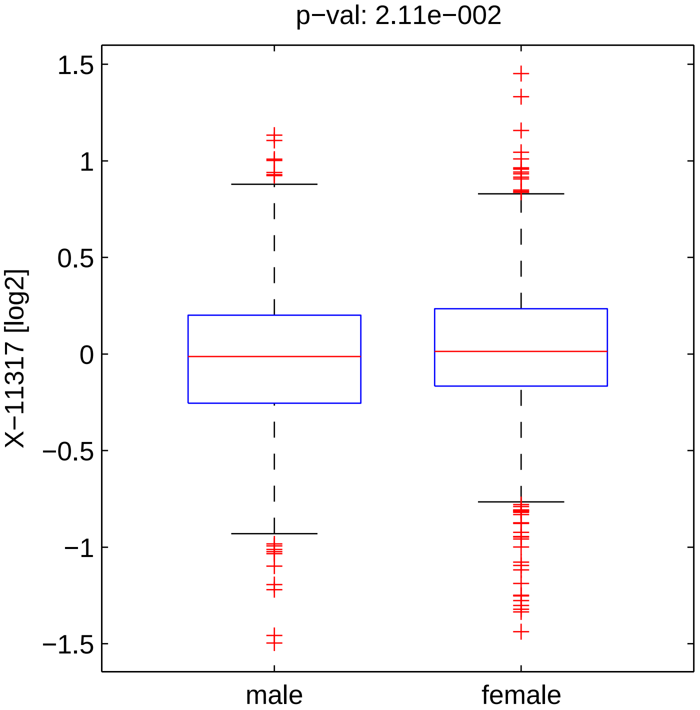
p−val: 2.11e−002
X−11317 [log2]
1.5
1
0.5
0
−0.5
−1
−1.5
male
female

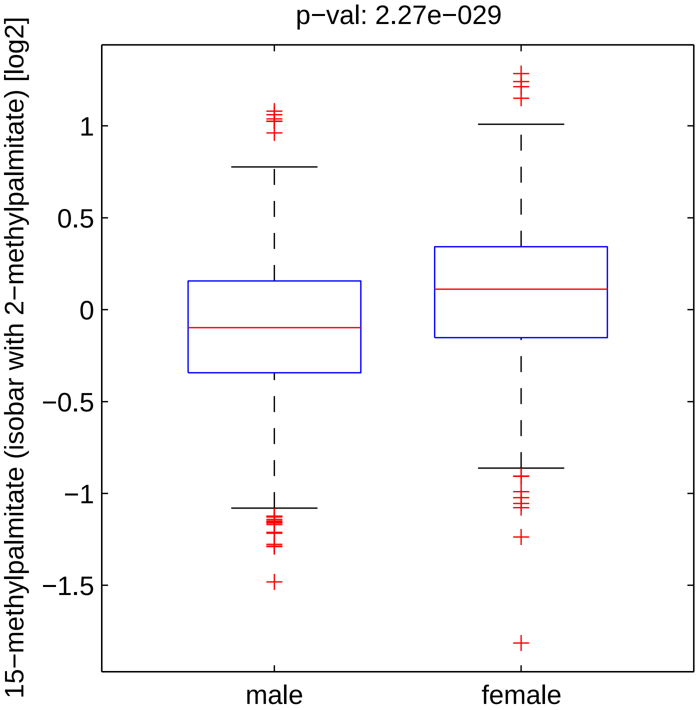

p−val: 2.27e−029
15−methylpalmitate (isobar with 2−methylpalmitate) [log2]
1
0.5
0
−0.5
−1
−1.5
male
female

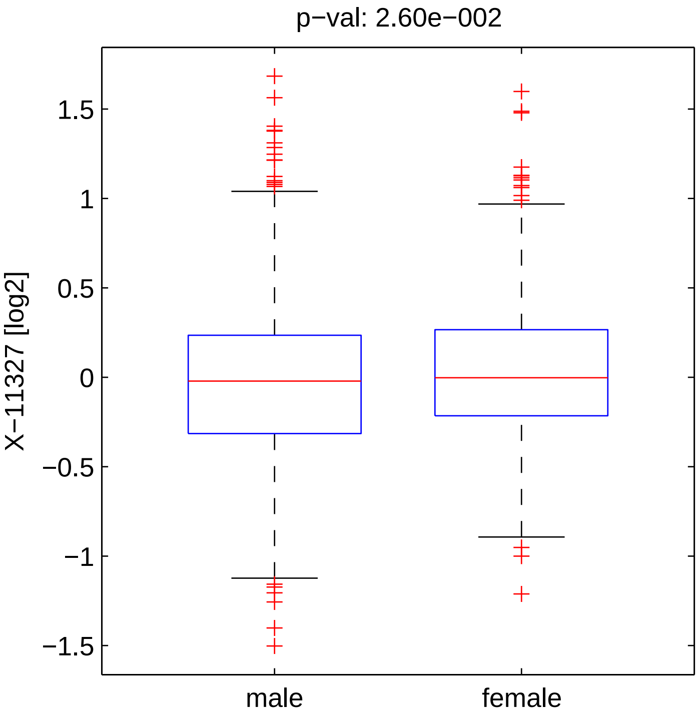

p−val: 2.60e−002
X−11327 [log2]
1.5
1
0.5
0
−0.5
−1
−1.5
male
female

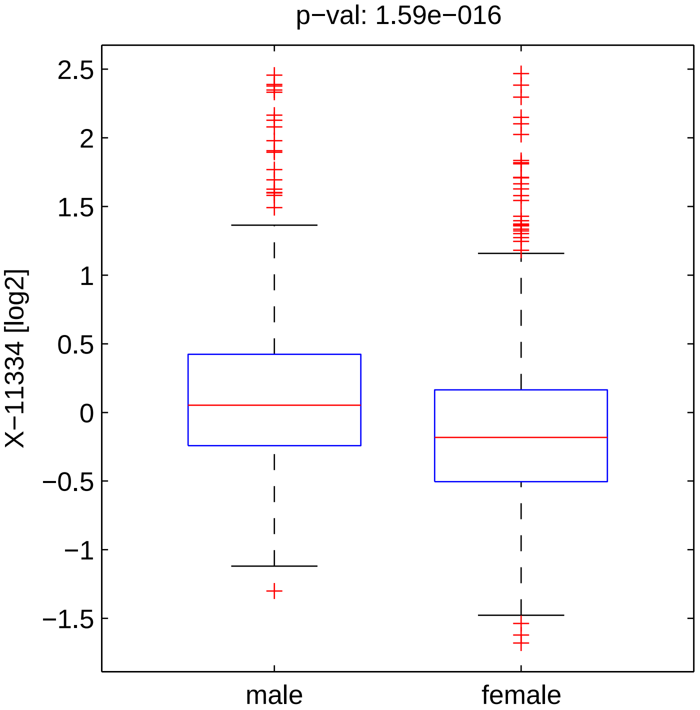

p−val: 1.59e−016
X−11334 [log2]
2.5
2
1.5
1
0.5
0
−0.5
−1
−1.5
male
female

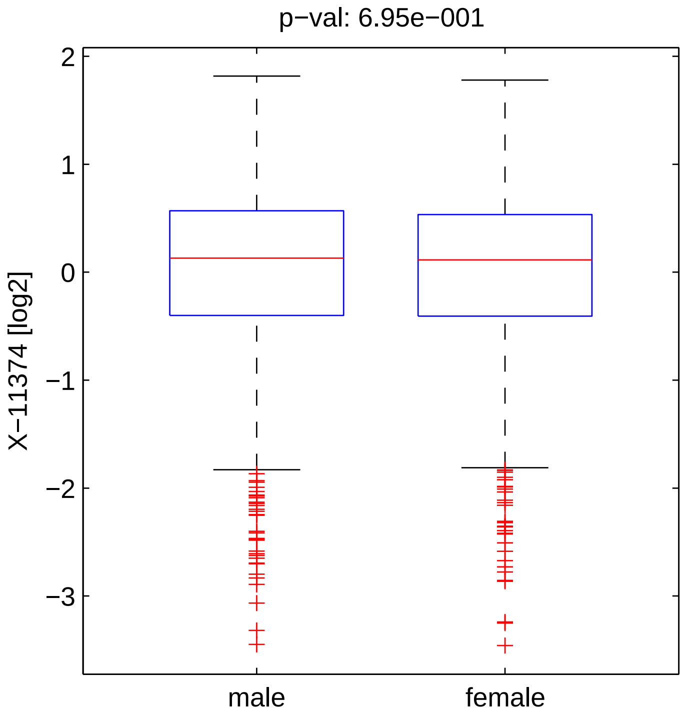
p−val: 6.95e−001
X−11374 [log2]
2
1
0
−1
−2
−3
male
female

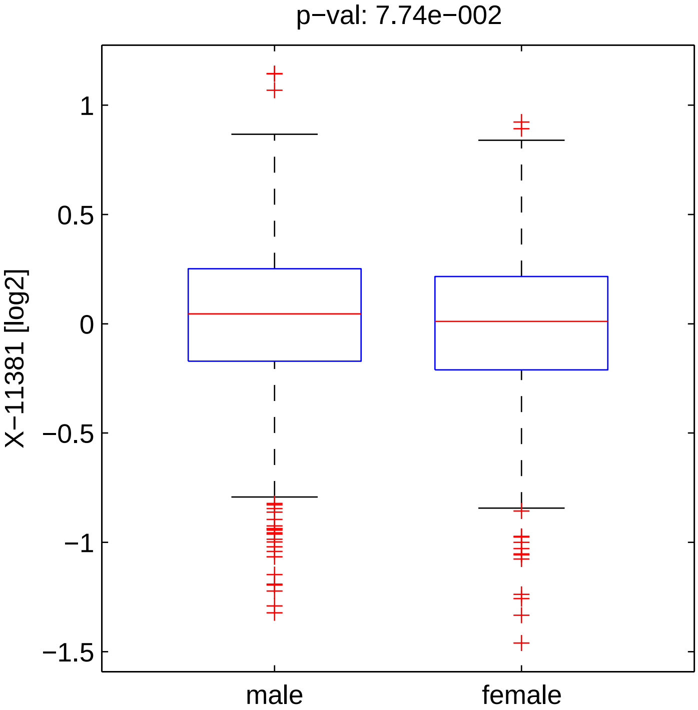

p−val: 7.74e−002
1
0.5
0
−0.5
−1
−1.5
X−11381 [log2]
male
female

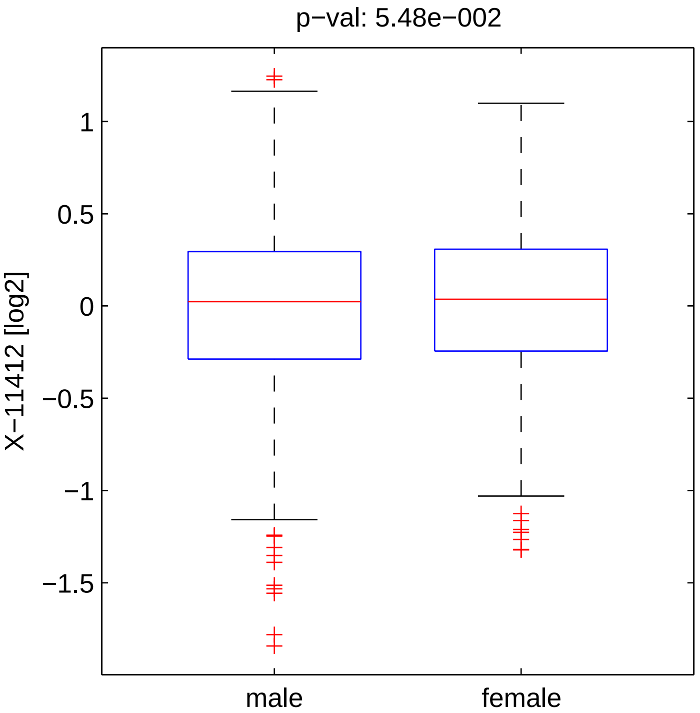
p−val: 5.48e−002
X−11412 [log2]
1
0.5
0
−0.5
−1
−1.5
male
female

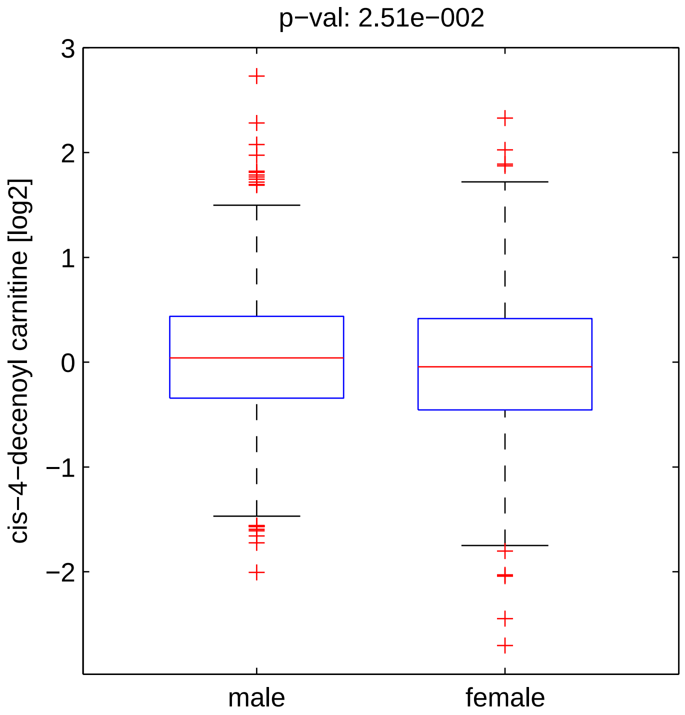
p−val: 2.51e−002
3
2
1
0
−1
−2
cis−4−decenoyl carnitine [log2]
male
female

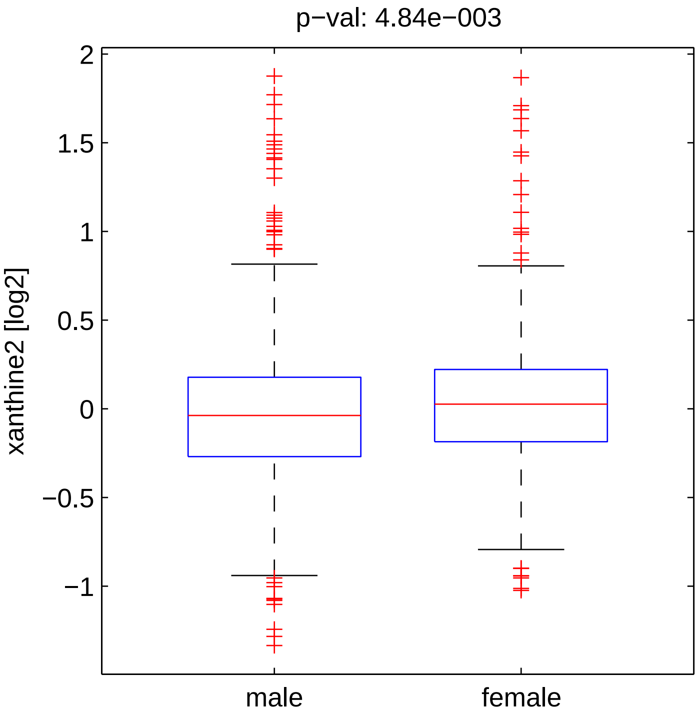
p−val: 4.84e−003
xanthine2 [log2]
2
1.5
1
0.5
0
−0.5
−1
male
female

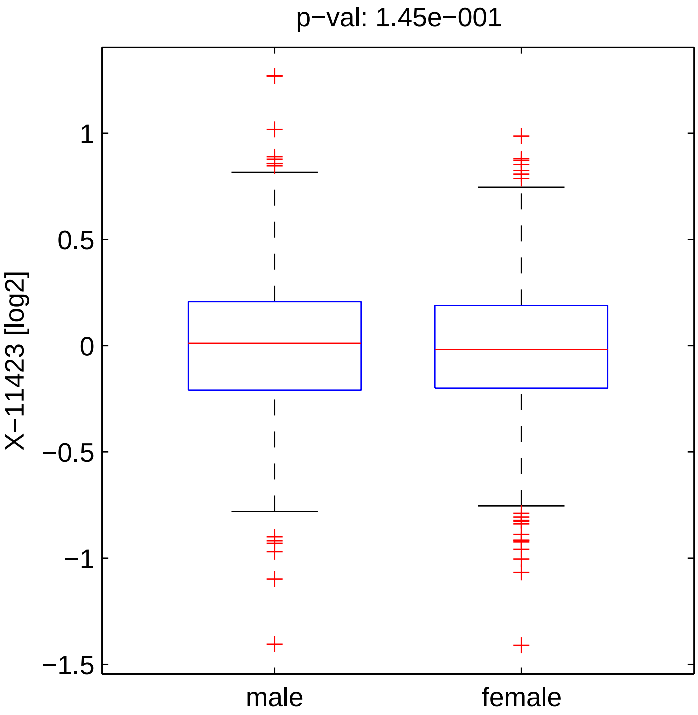

p−val: 1.45e−001
X−11423 [log2]
1
0.5
0
−0.5
−1
−1.5
male
female

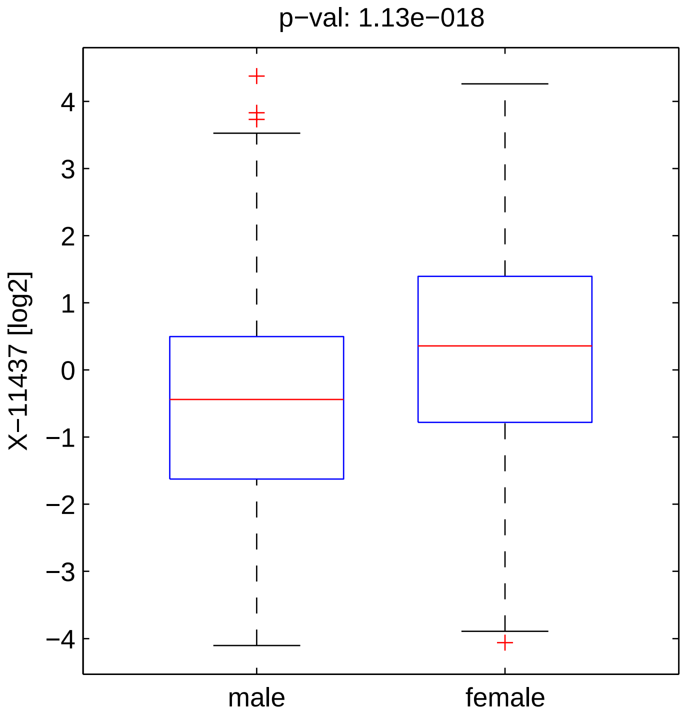

p−val: 1.13e−018
X−11437 [log2]
4
3
2
1
0
−1
−2
−3
−4
male
female

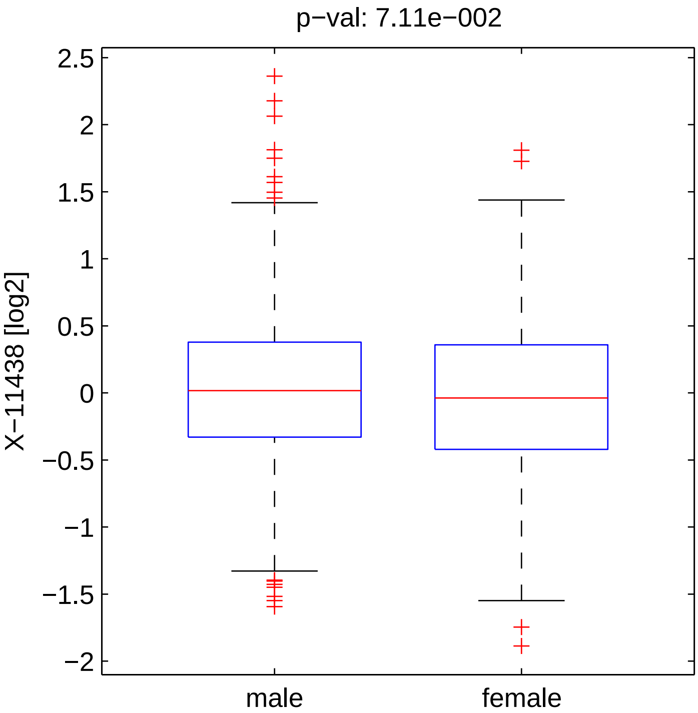

p−val: 7.11e−002
X−11438 [log2]
2.5
2
1.5
1
0.5
0
−0.5
−1
−1.5
−2
male
female

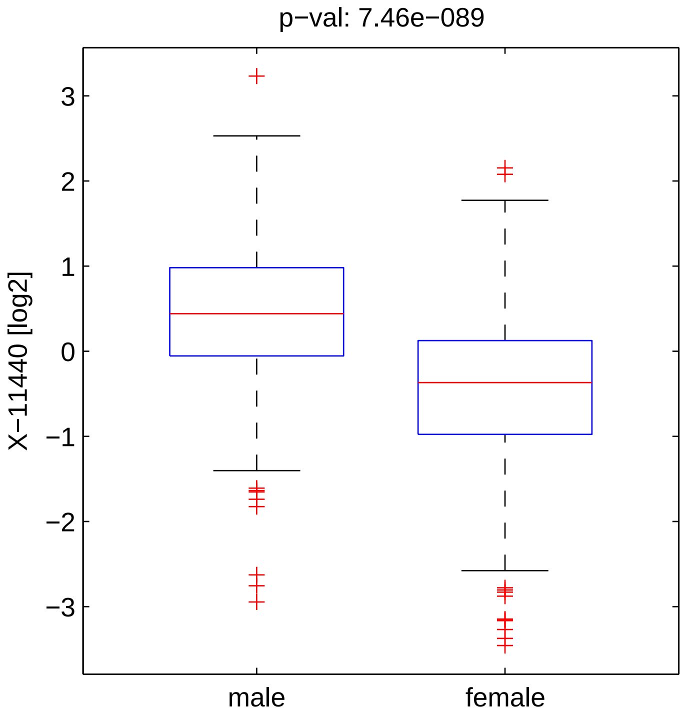

p−val: 7.46e−089
X−11440 [log2]
3
2
1
0
−1
−2
−3
male
female

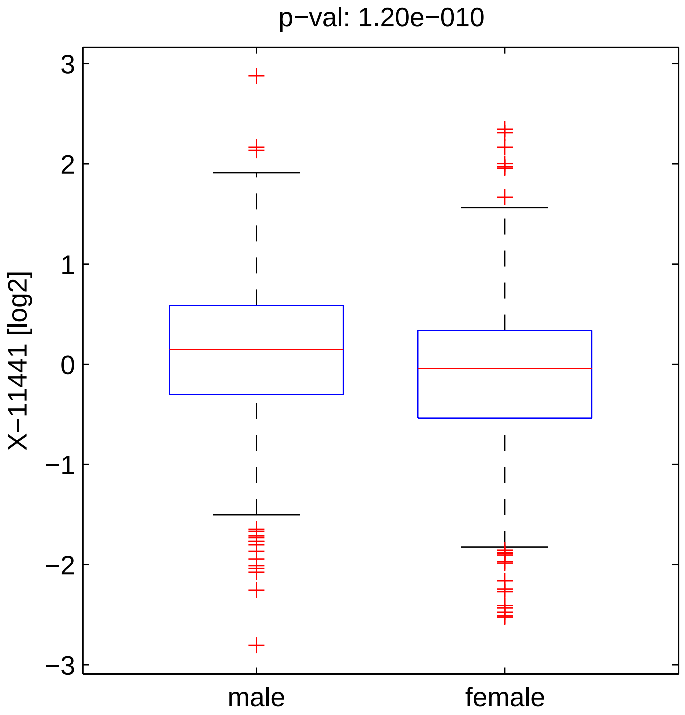
p−val: 1.20e−010
X−11441 [log2]
3
2
1
0
−1
−2
−3
male
female

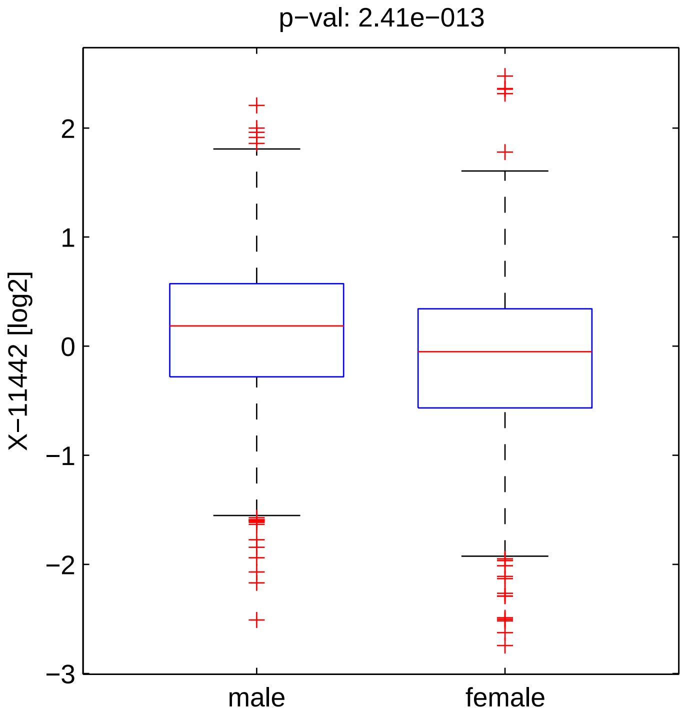
p−val: 2.41e−013
X−11442 [log2]
2
1
0
−1
−2
−3
male
female

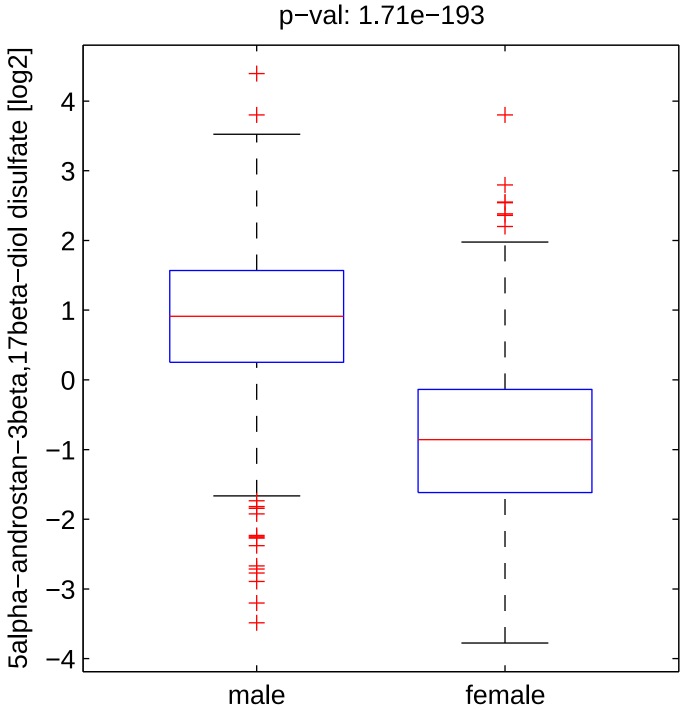
p−val: 1.71e−193
5alpha−androstan−3beta,17beta−diol disulfate [log2]
4
3
2
1
0
−1
−2
−3
−4
male
female

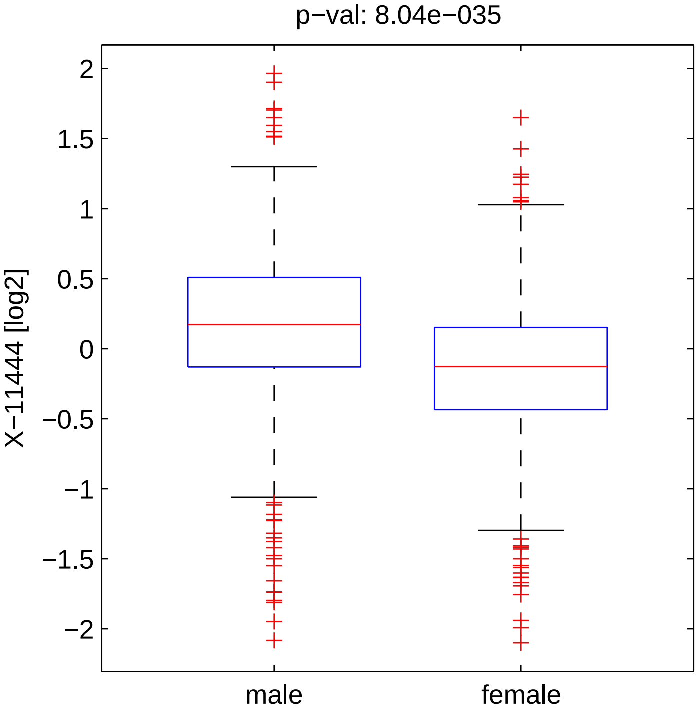

p−val: 8.04e−035
X−11444 [log2]
2
1.5
1
0.5
0
−0.5
−1
−1.5
−2
male
female

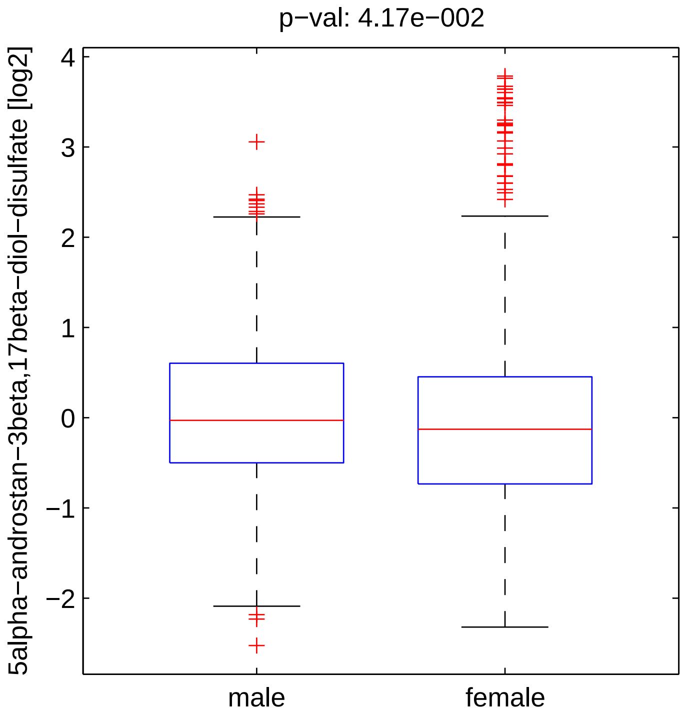

p−val: 4.17e−002
5alpha−androstan−3beta,17beta−diol−disulfate [log2]
4
3
2
1
0
−1
−2
male
female

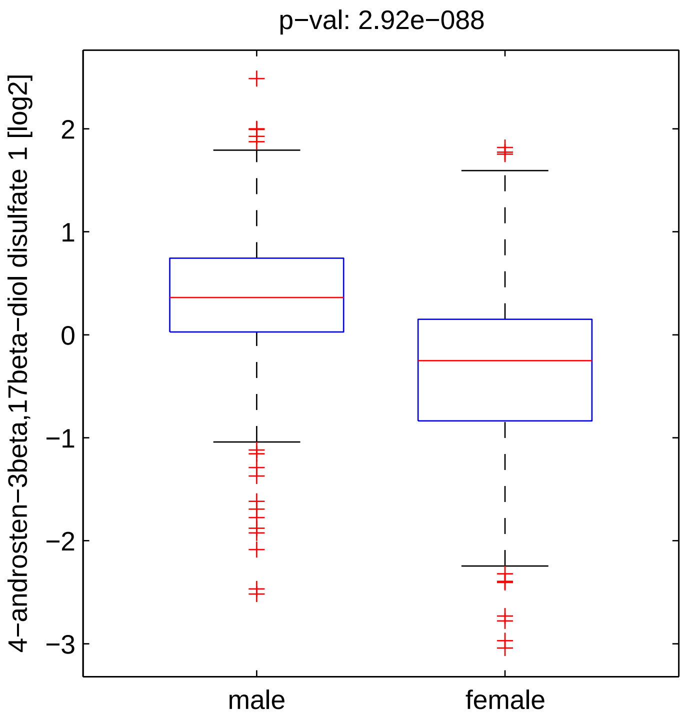
p−val: 2.92e−088
4−androsten−3beta,17beta−diol disulfate 1 [log2]
2
1
0
−1
−2
−3
male
female

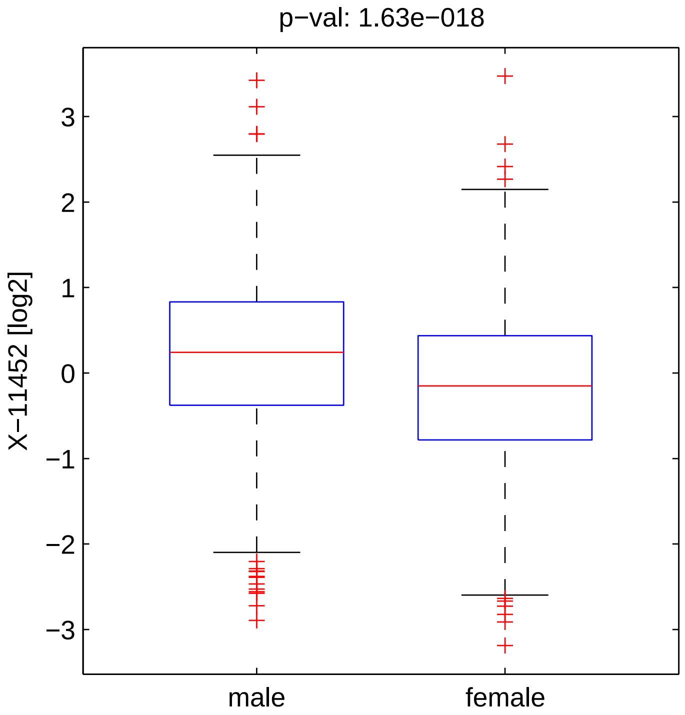

p−val: 1.63e−018
X−11452 [log2]
3
2
1
0
−1
−2
−3
male
female

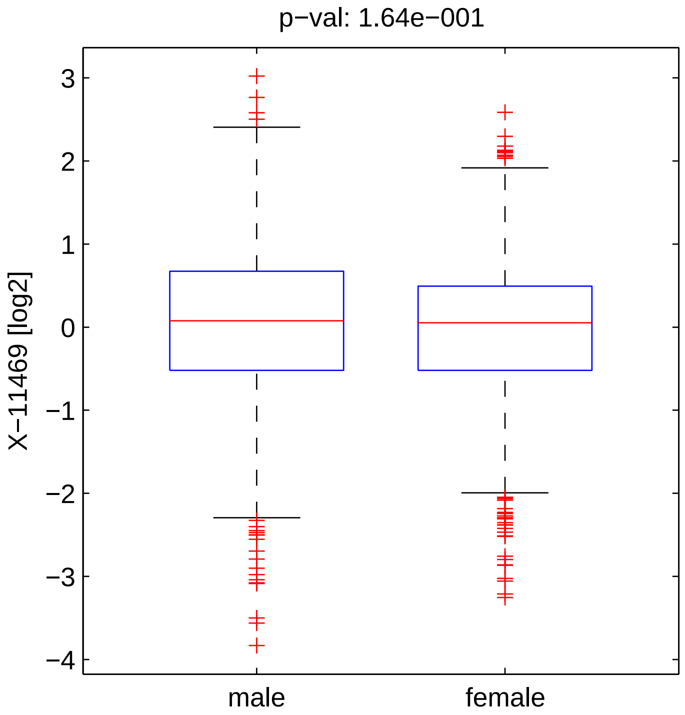
p−val: 1.64e−001
X−11469 [log2]
3
2
1
0
−1
−2
−3
−4
male
female

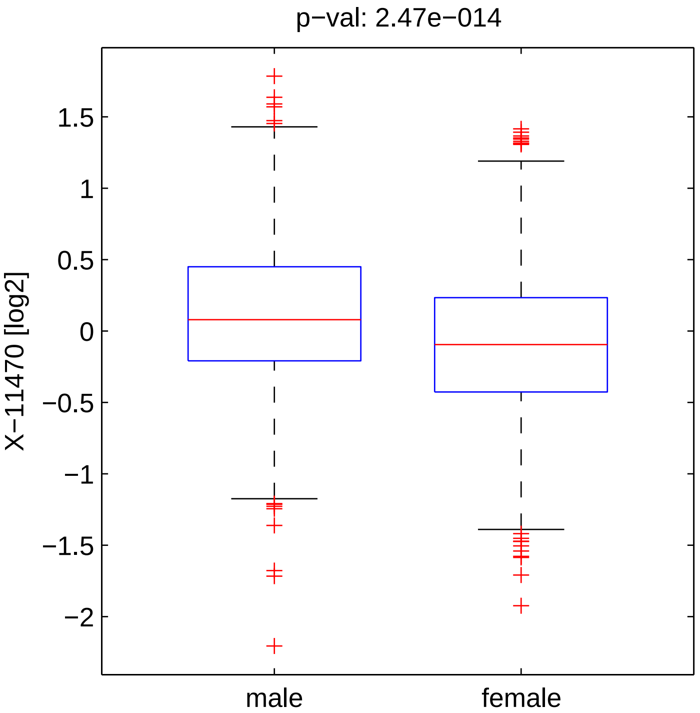
p−val: 2.47e−014
X−11470 [log2]
1.5
1
0.5
0
−0.5
−1
−1.5
−2
male
female

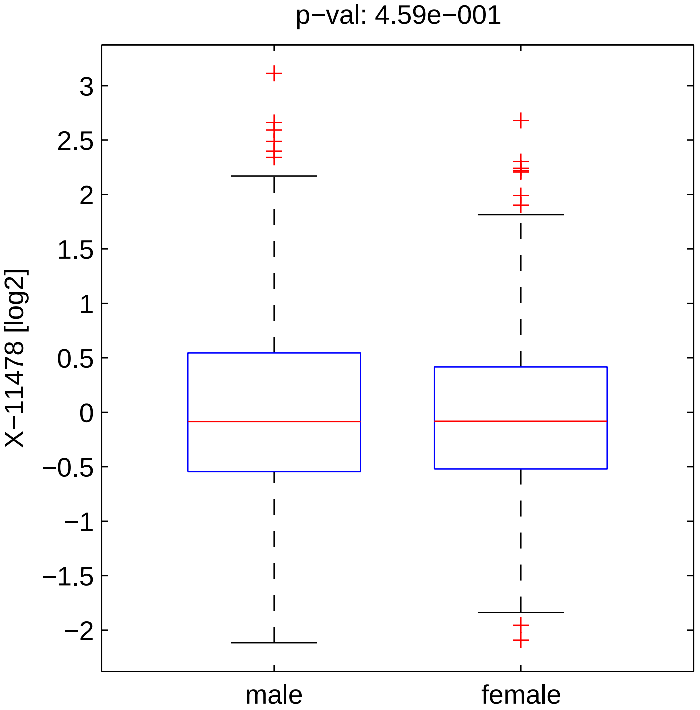

p−val: 4.59e−001
X−11478 [log2]
3
2.5
2
1.5
1
0.5
0
−0.5
−1
−1.5
−2
male
female

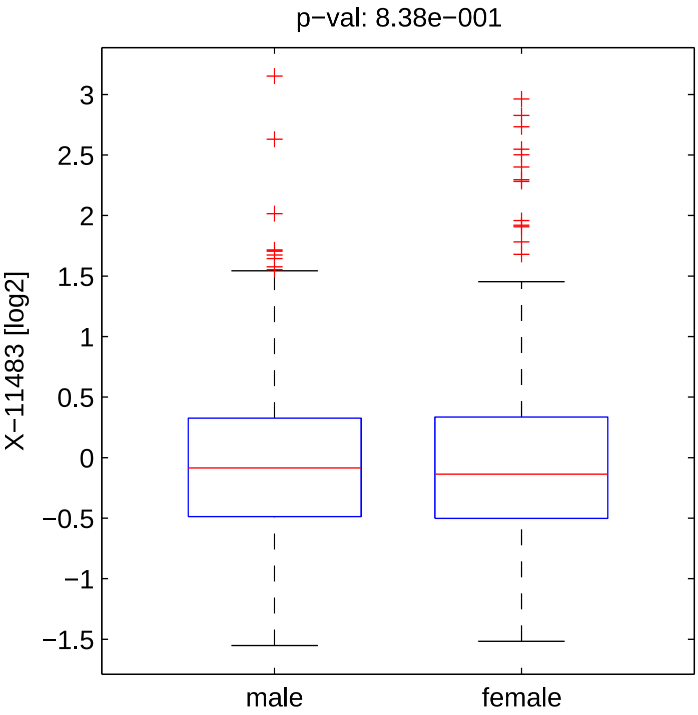
p−val: 8.38e−001
X−11483 [log2]
3
2.5
2
1.5
1
0.5
0
−0.5
−1
−1.5
male
female

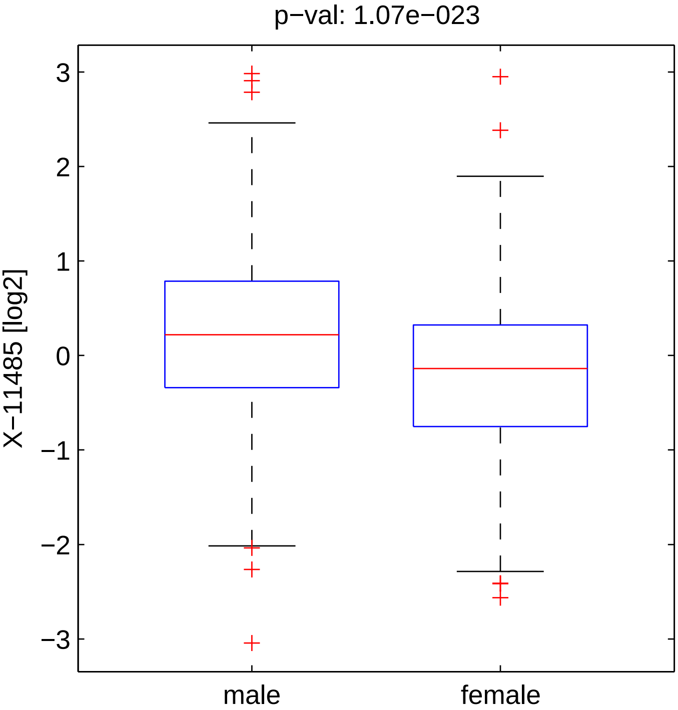
p−val: 1.07e−023
X−11485 [log2]
3
2
1
0
−1
−2
−3
male
female

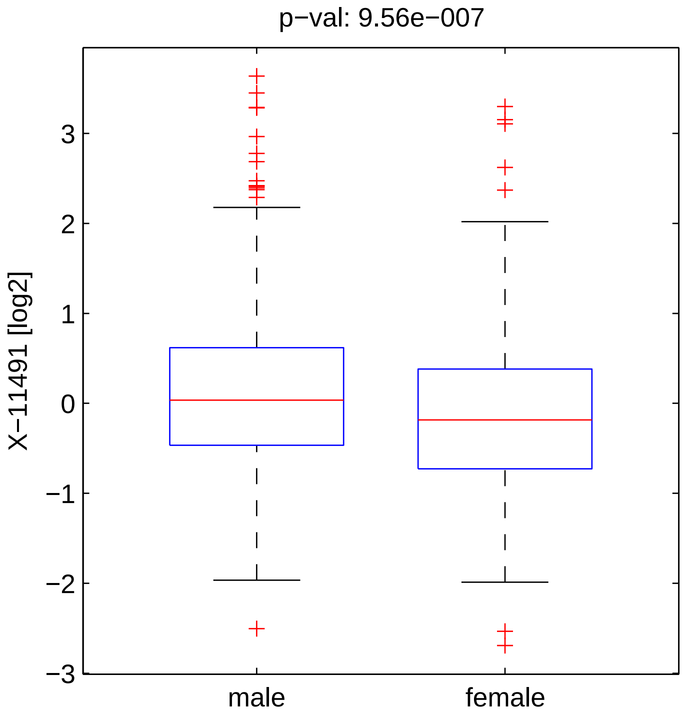
p−val: 9.56e−007
X−11491 [log2]
3
2
1
0
−1
−2
−3
male
female

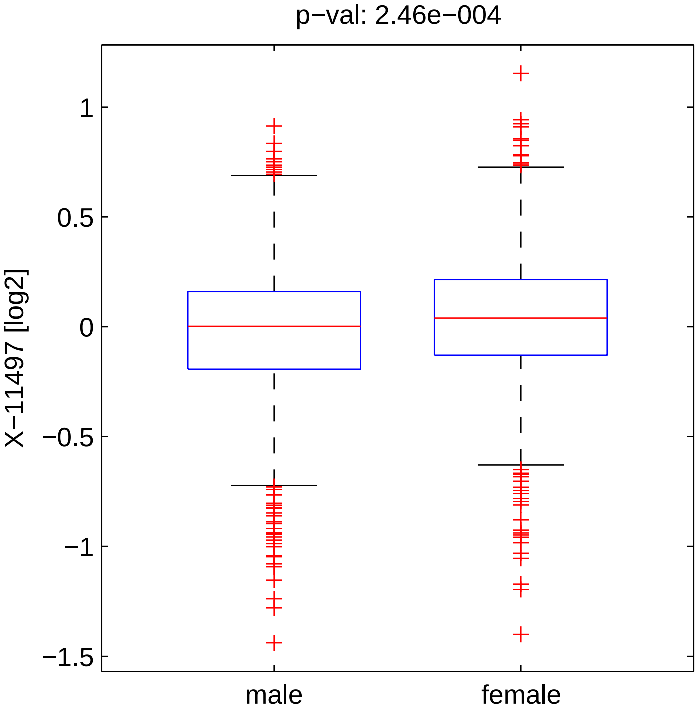
p−val: 2.46e−004
X−11497 [log2]
1
0.5
0
−0.5
−1
−1.5
male
female

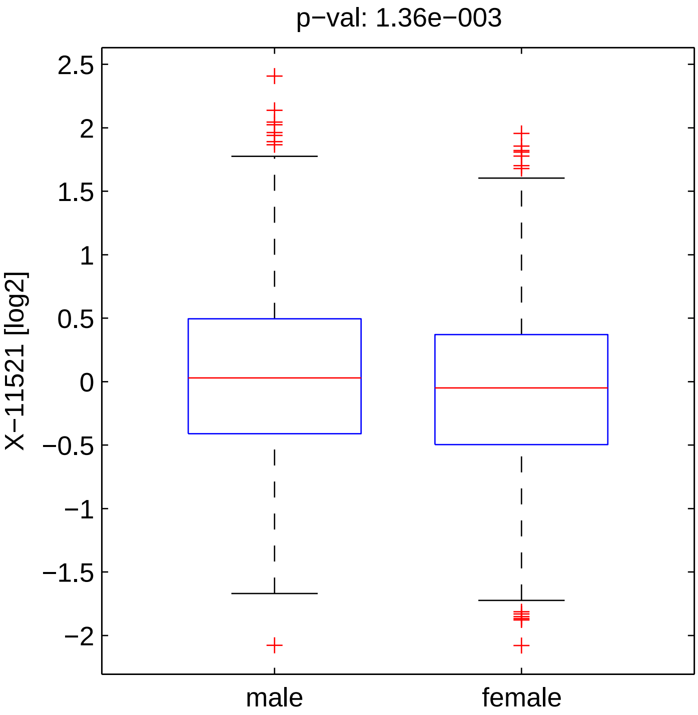
p−val: 1.36e−003
X−11521 [log2]
2.5
2
1.5
1
0.5
0
−0.5
−1
−1.5
−2
male
female

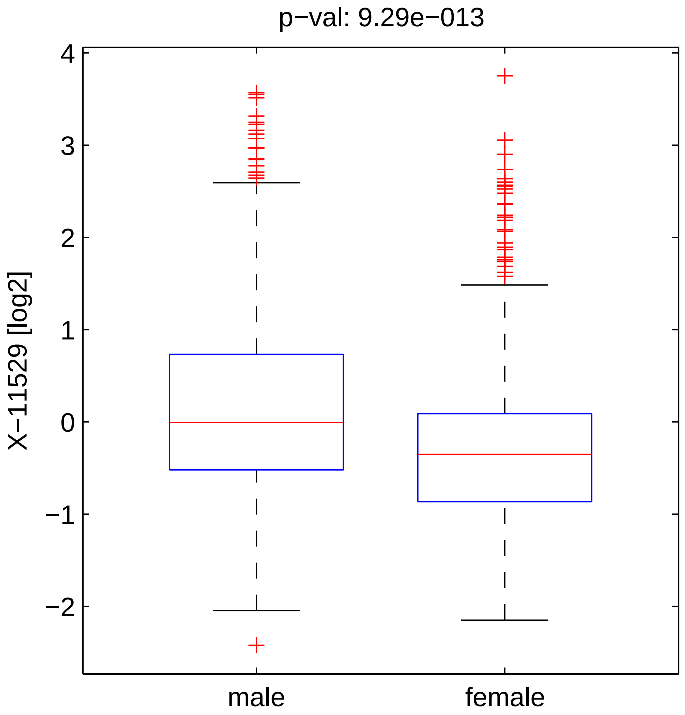
p−val: 9.29e−013
X−11529 [log2]
4
3
2
1
0
−1
−2
male
female

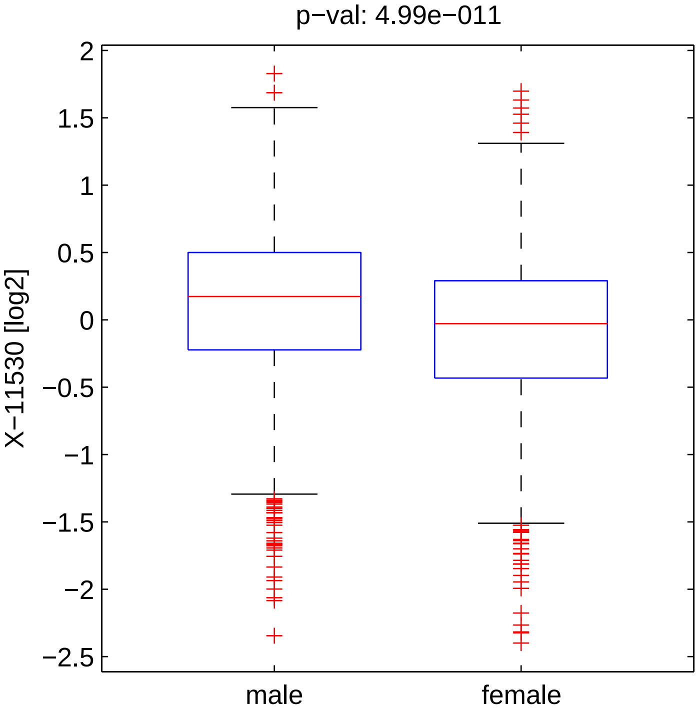

p−val: 4.99e−011
X−11530 [log2]
2
1.5
1
0.5
0
−0.5
−1
−1.5
−2
−2.5
male
female

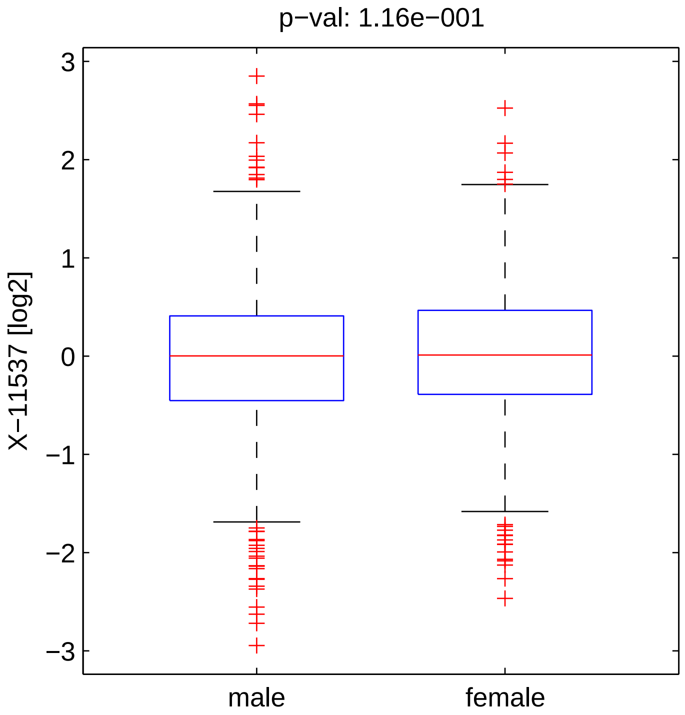
p−val: 1.16e−001
X−11537 [log2]
3
2
1
0
−1
−2
−3
male
female

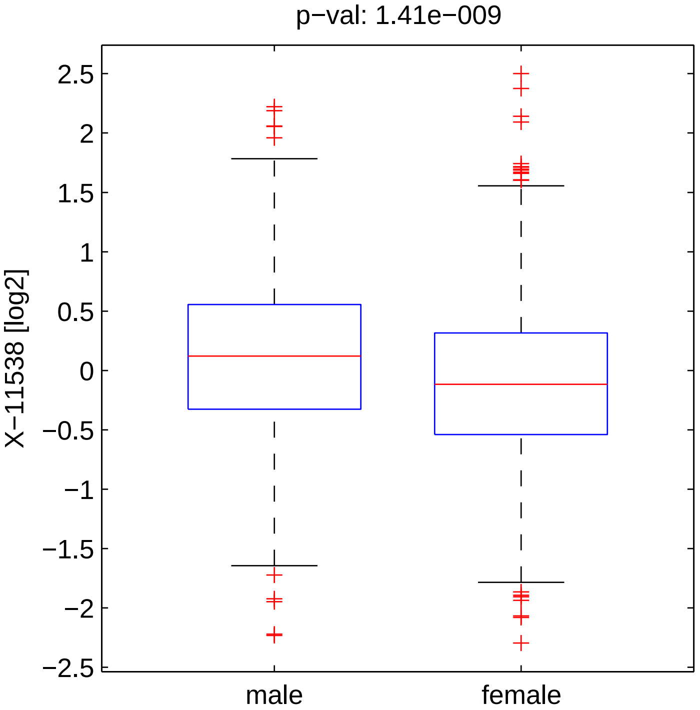
p−val: 1.41e−009
X−11538 [log2]
2.5
2
1.5
1
0.5
0
−0.5
−1
−1.5
−2
−2.5
male
female

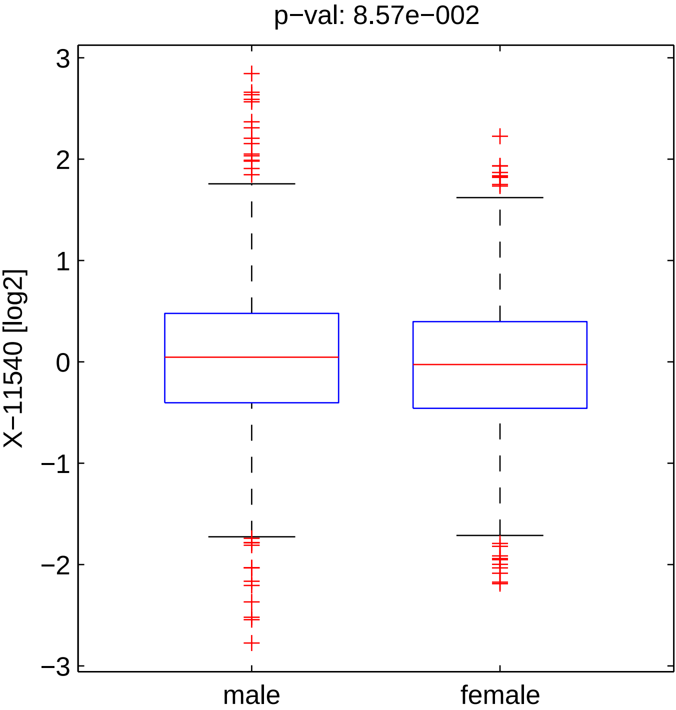
p−val: 8.57e−002
X−11540 [log2]
3
2
1
0
−1
−2
−3
male
female

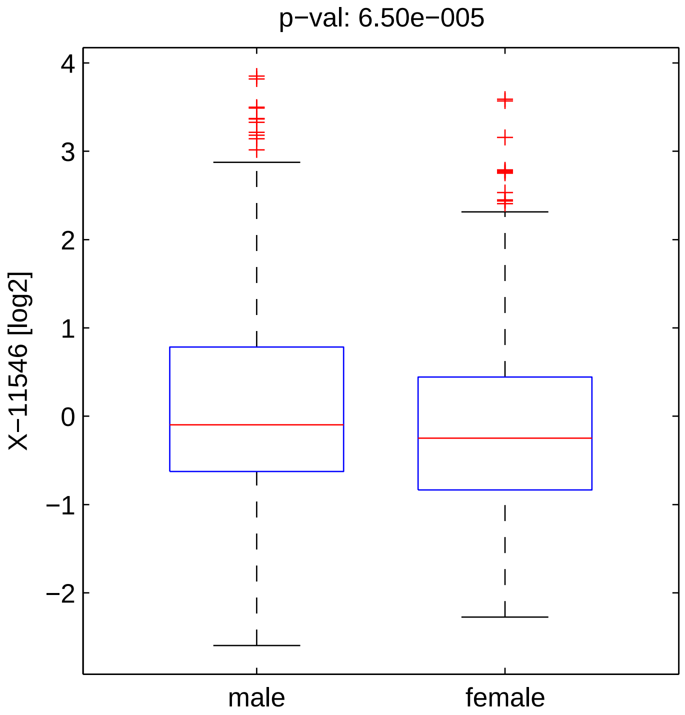
p−val: 6.50e−005
X−11546 [log2]
4
3
2
1
0
−1
−2
male
female

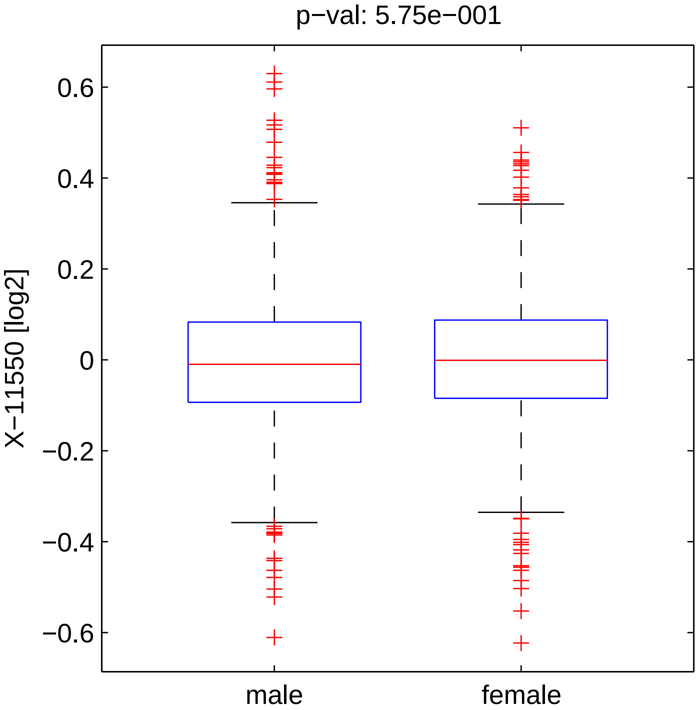

p−val: 5.75e−001
X−11550 [log2]
0.6
0.4
0.2
0
−0.2
−0.4
−0.6
male
female

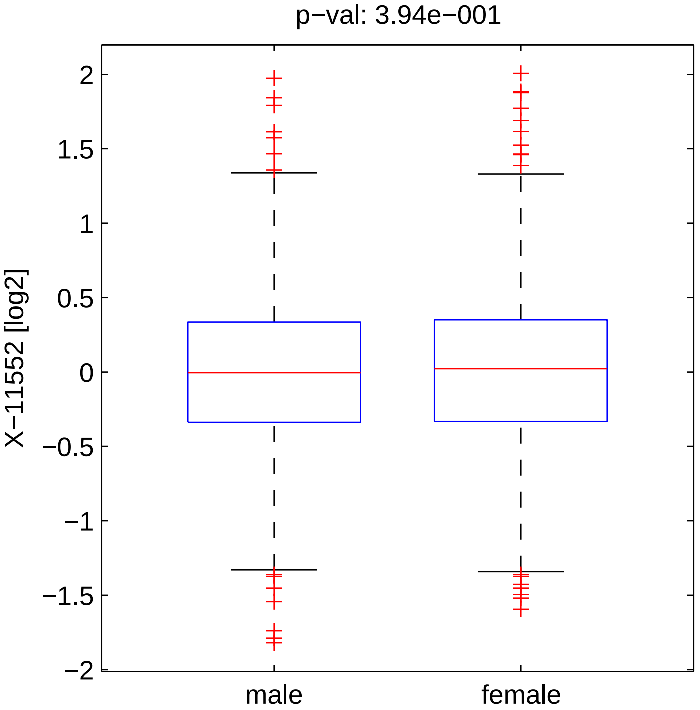
p−val: 3.94e−001
X−11552 [log2]
2
1.5
1
0.5
0
−0.5
−1
−1.5
−2
male
female

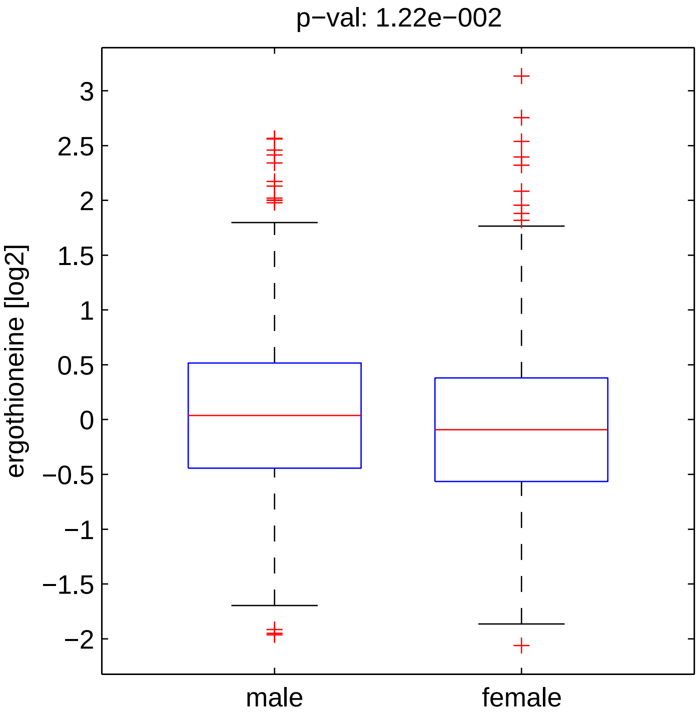

p−val: 1.22e−002
ergothioneine [log2]
3
2.5
2
1.5
1
0.5
0
−0.5
−1
−1.5
−2
male
female

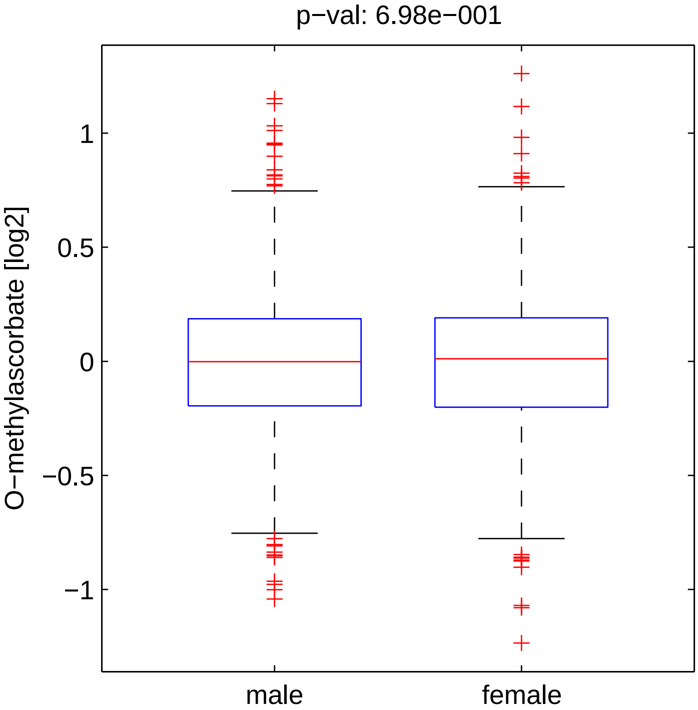

p−val: 6.98e−001
O−methylascorbate [log2]
1
0.5
0
−0.5
−1
male
female

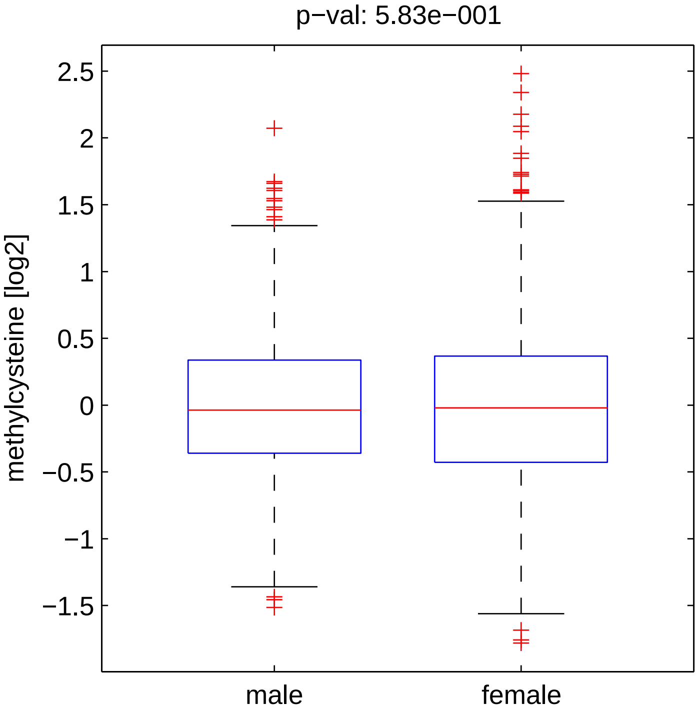
p−val: 5.83e−001
methylcysteine [log2]
2.5
2
1.5
1
0.5
0
−0.5
−1
−1.5
male
female

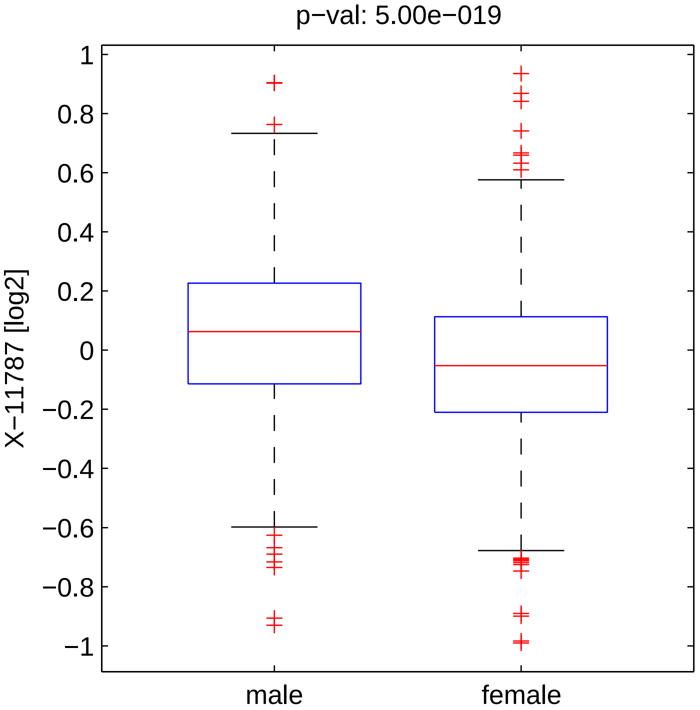
p−val: 5.00e−019
1
0.8
0.6
0.4
0.2
0
−0.2
−0.4
−0.6
−0.8
−1
X−11787 [log2]
male
female

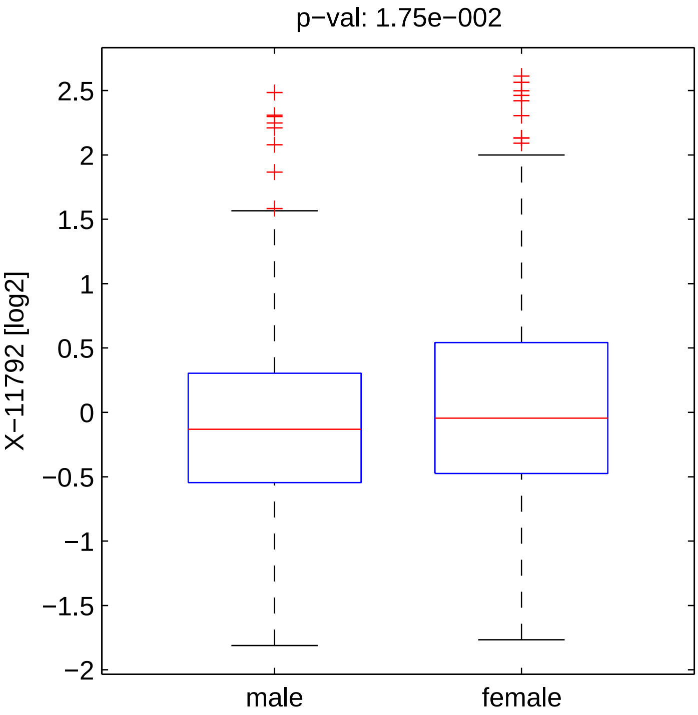

p−val: 1.75e−002
X−11792 [log2]
2.5
2
1.5
1
0.5
0
−0.5
−1
−1.5
−2
male
female

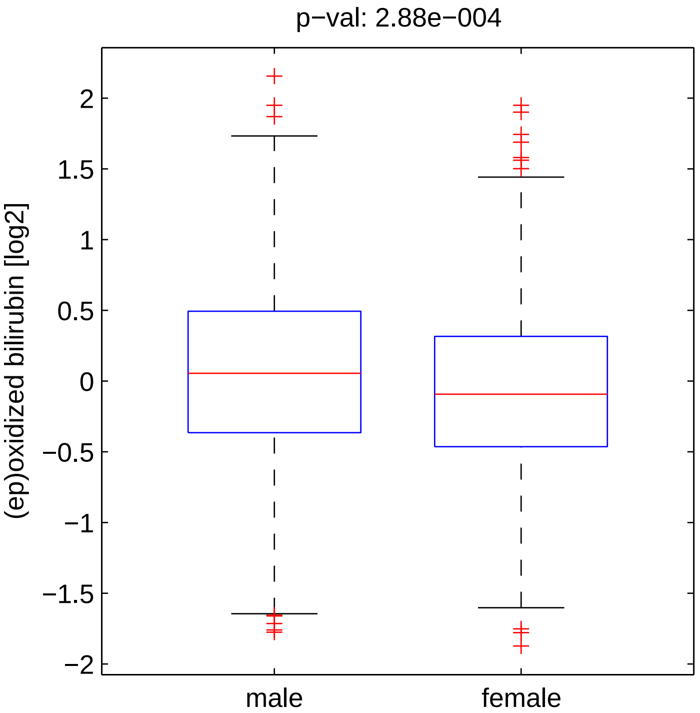

p−val: 2.88e−004
(ep)oxidized bilirubin [log2]
2
1.5
1
0.5
0
−0.5
−1
−1.5
−2
male
female

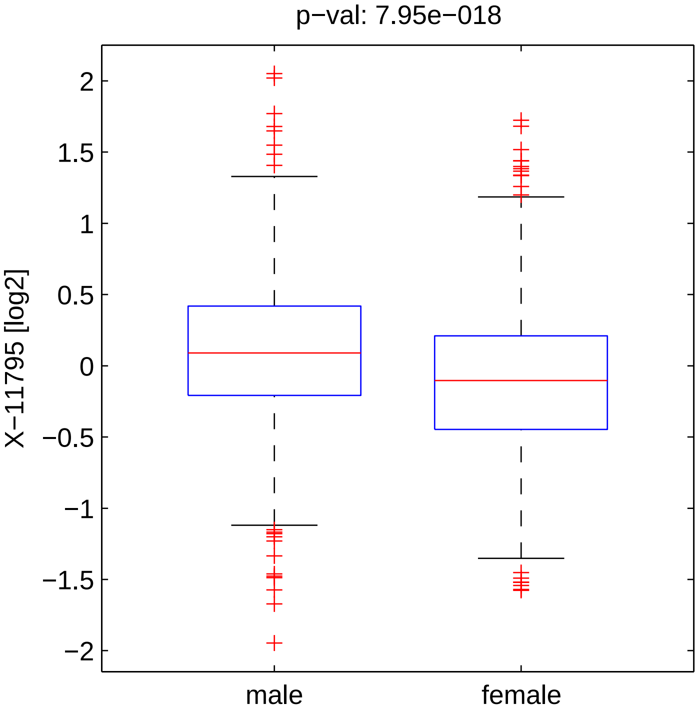
p−val: 7.95e−018
X−11795 [log2]
2
1.5
1
0.5
0
−0.5
−1
−1.5
−2
male
female

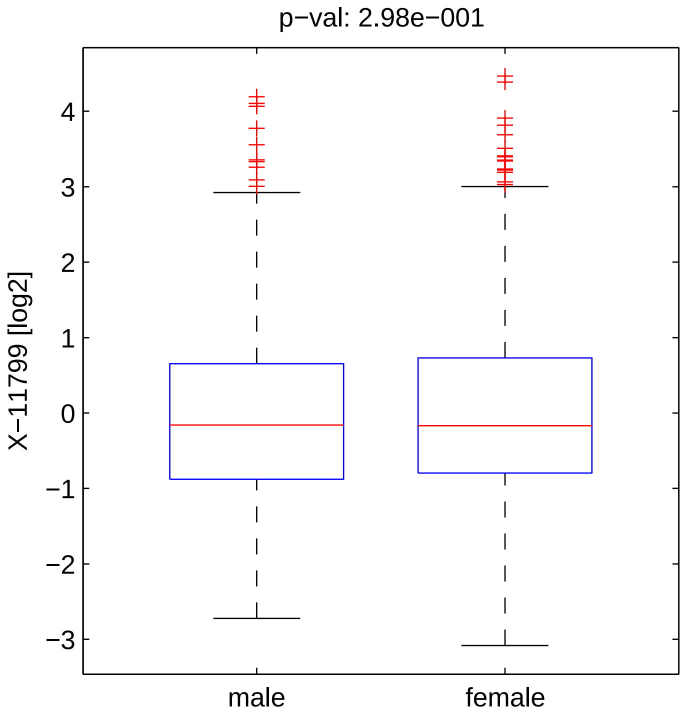

p−val: 2.98e−001
X−11799 [log2]
4
3
2
1
0
−1
−2
−3
male
female

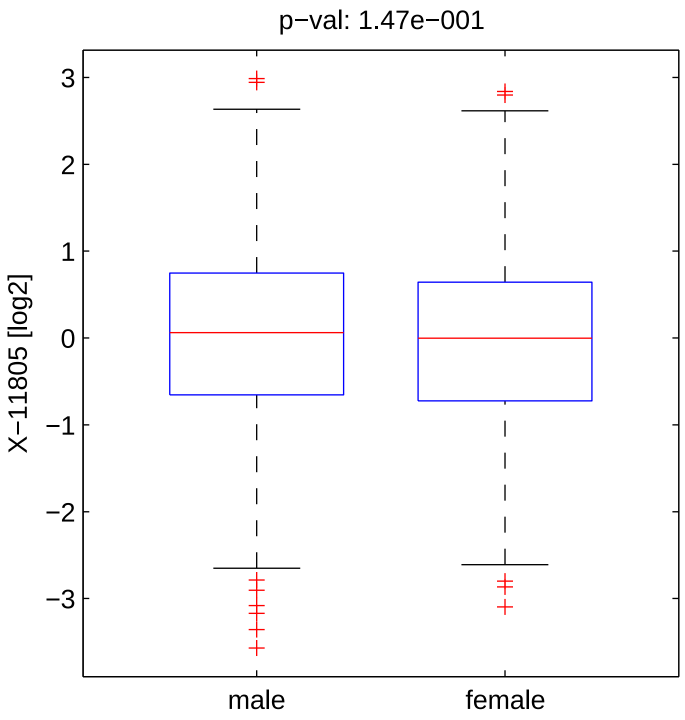
p−val: 1.47e−001
X−11805 [log2]
3
2
1
0
−1
−2
−3
male
female

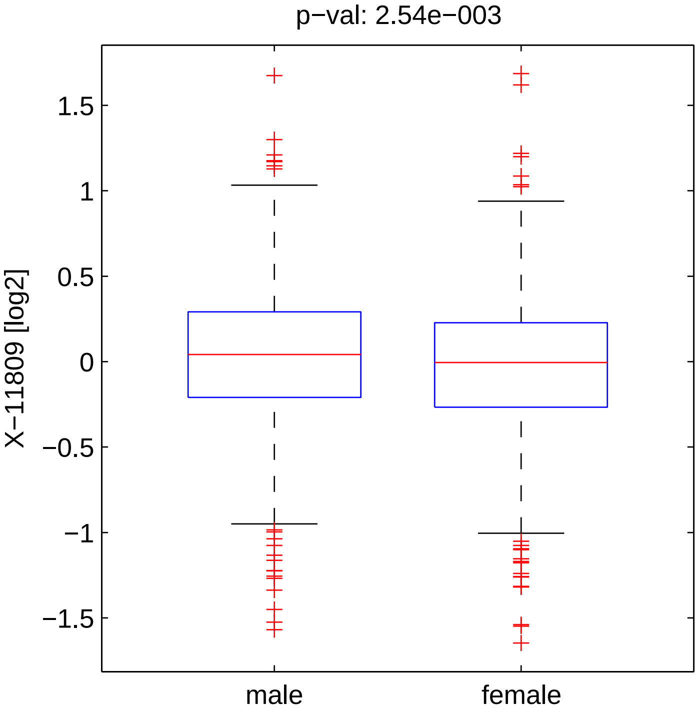
p−val: 2.54e−003
X−11809 [log2]
1.5
1
0.5
0
−0.5
−1
−1.5
male
female

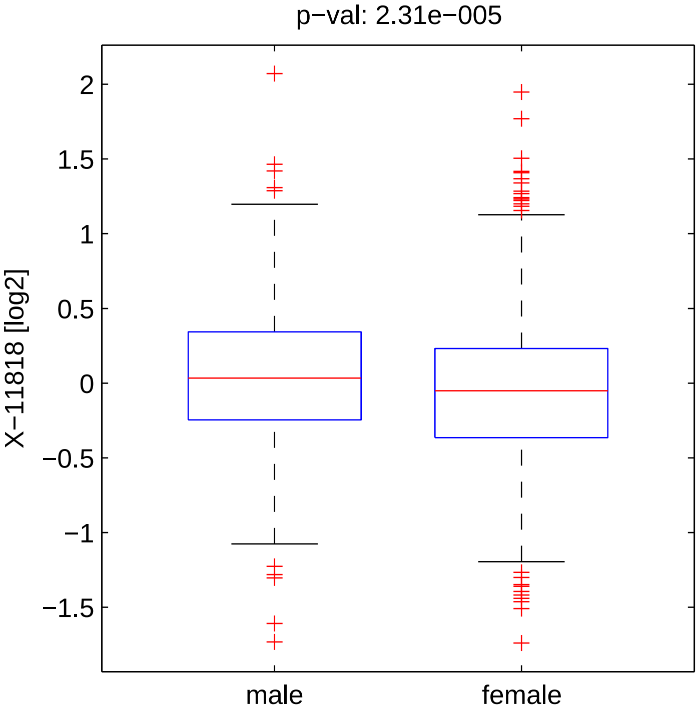

p−val: 2.31e−005
X−11818 [log2]
2
1.5
1
0.5
0
−0.5
−1
−1.5
male
female

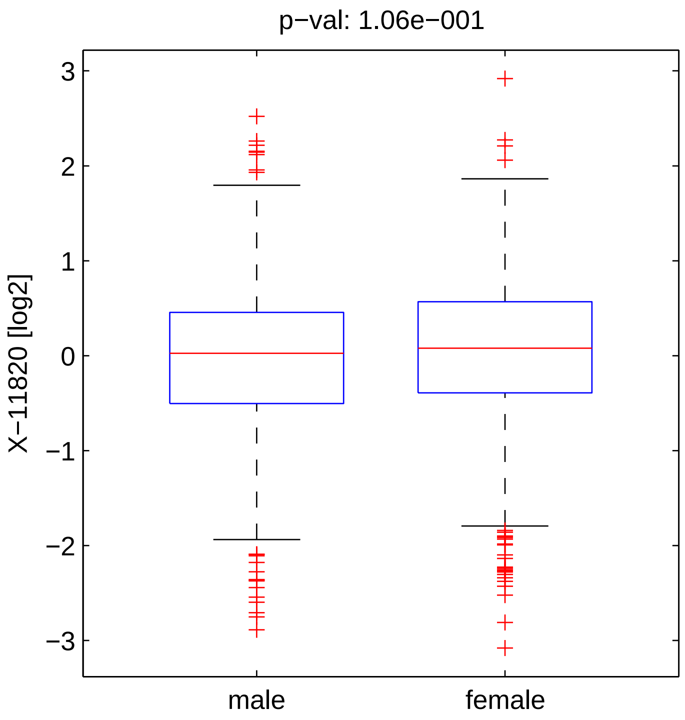

p−val: 1.06e−001
X−11820 [log2]
3
2
1
0
−1
−2
−3
male
female

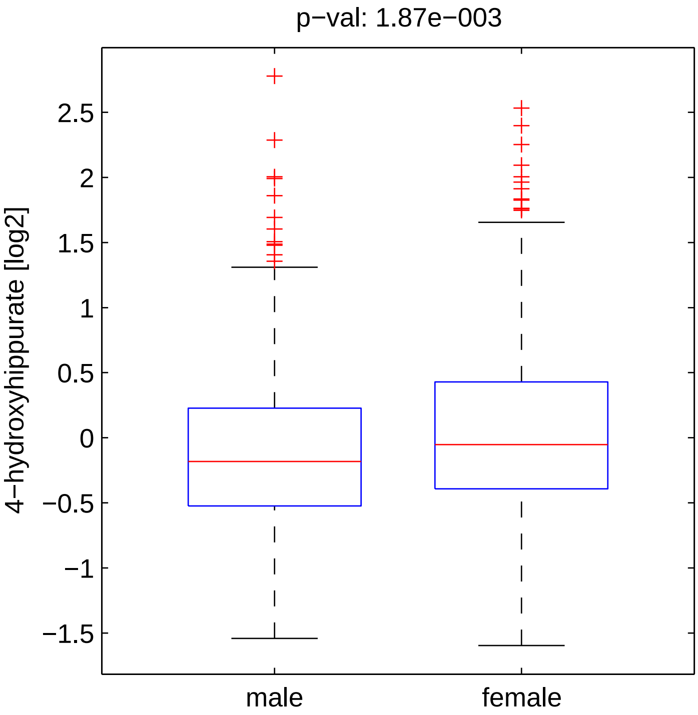
p−val: 1.87e−003
4−hydroxyhippurate [log2]
2.5
2
1.5
1
0.5
0
−0.5
−1
−1.5
male
female

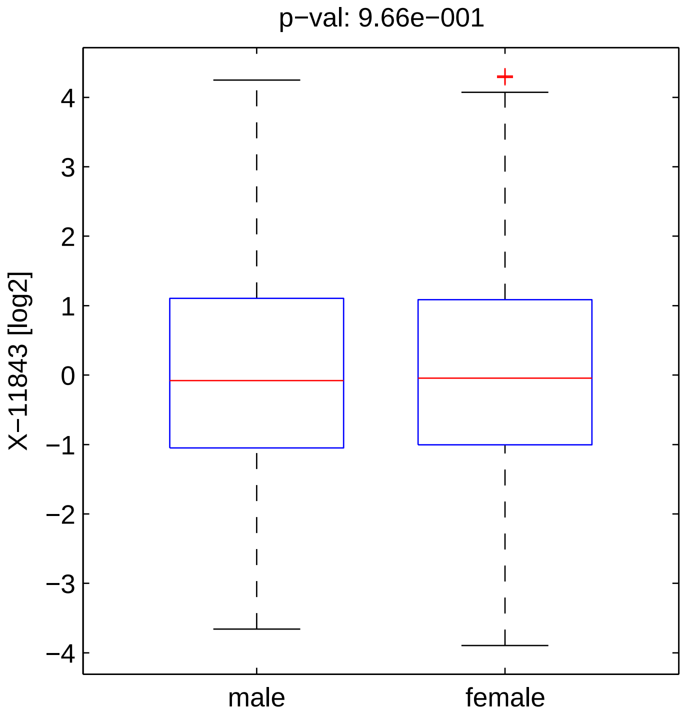
p−val: 9.66e−001
X−11843 [log2]
4
3
2
1
0
−1
−2
−3
−4
male
female

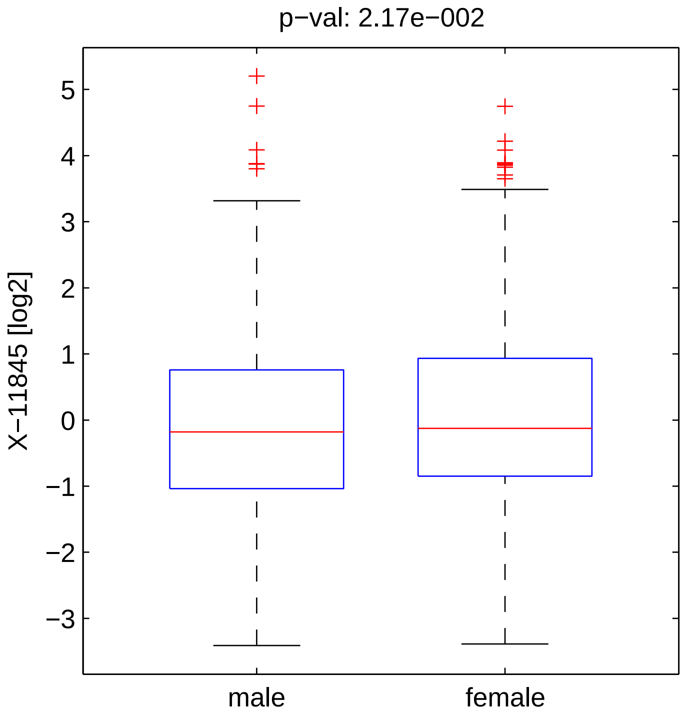
p−val: 2.17e−002
X−11845 [log2]
5
4
3
2
1
0
−1
−2
−3
male
female

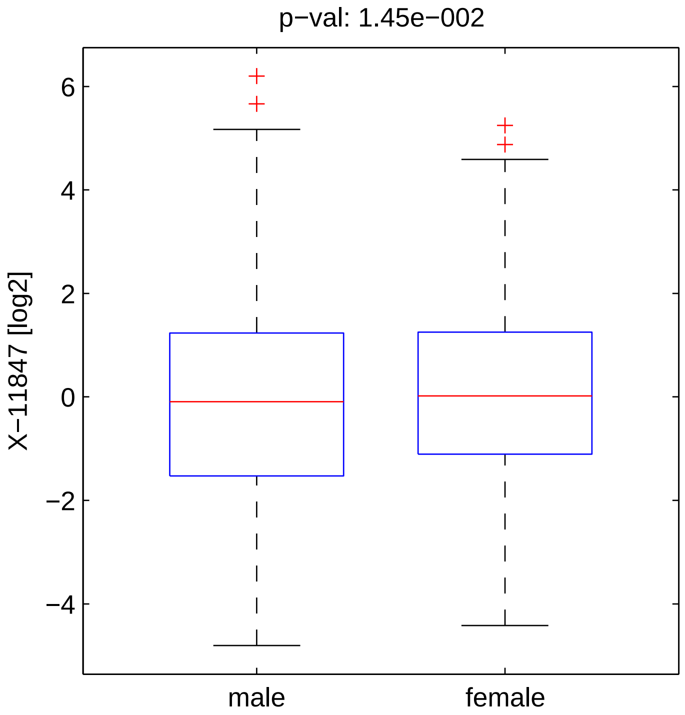
p−val: 1.45e−002
X−11847 [log2]
6
4
2
0
−2
−4
male
female

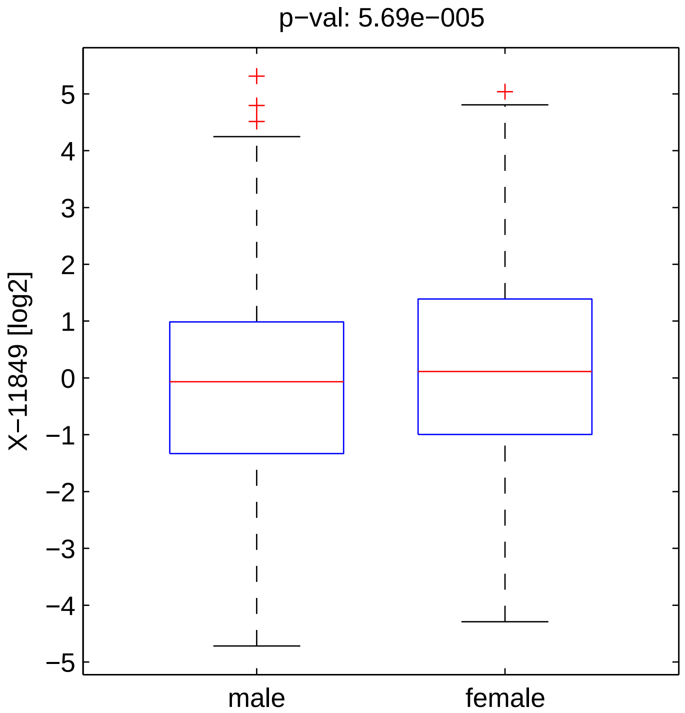
p−val: 5.69e−005
X−11849 [log2]
5
4
3
2
1
0
−1
−2
−3
−4
−5
male
female

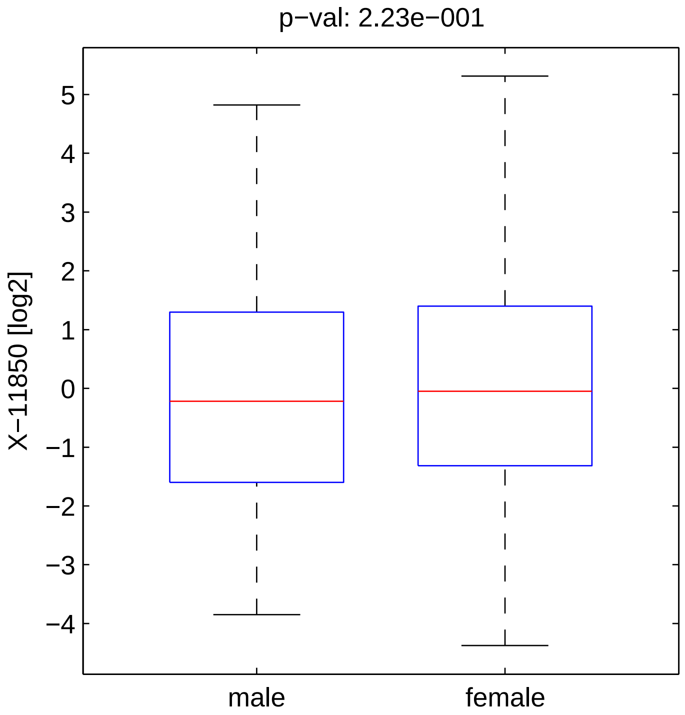
p−val: 2.23e−001
X−11850 [log2]
5
4
3
2
1
0
−1
−2
−3
−4
male
female

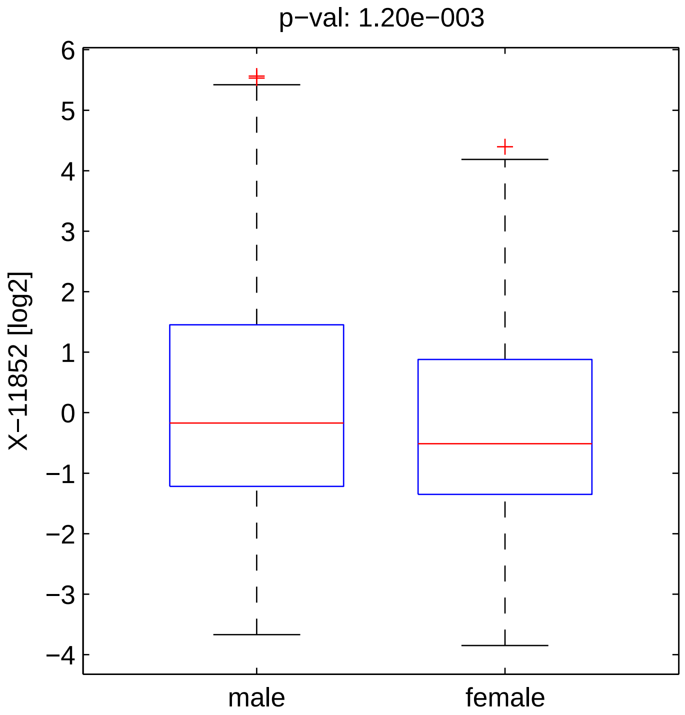

p−val: 1.20e−003
6
5
4
3
2
1
0
−1
−2
−3
−4
X−11852 [log2]
male
female

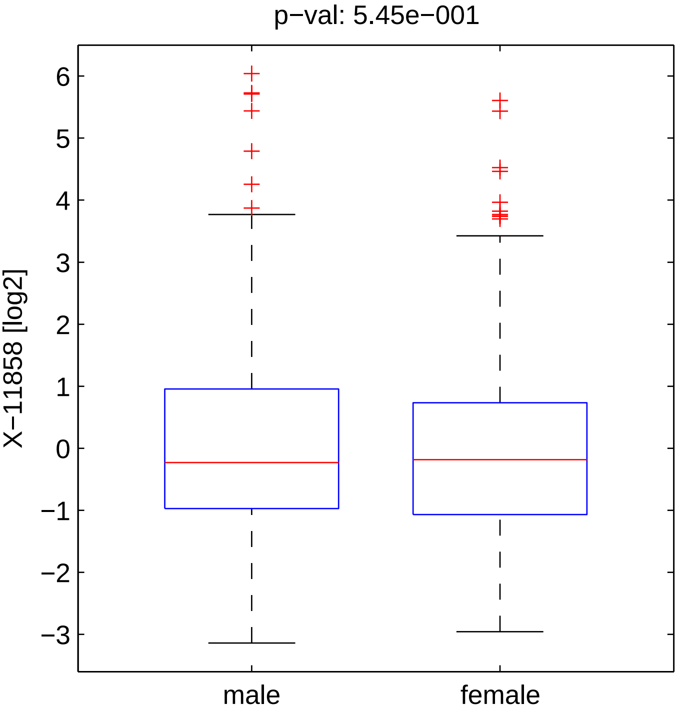
p−val: 5.45e−001
X−11858 [log2]
6
5
4
3
2
1
0
−1
−2
−3
male
female

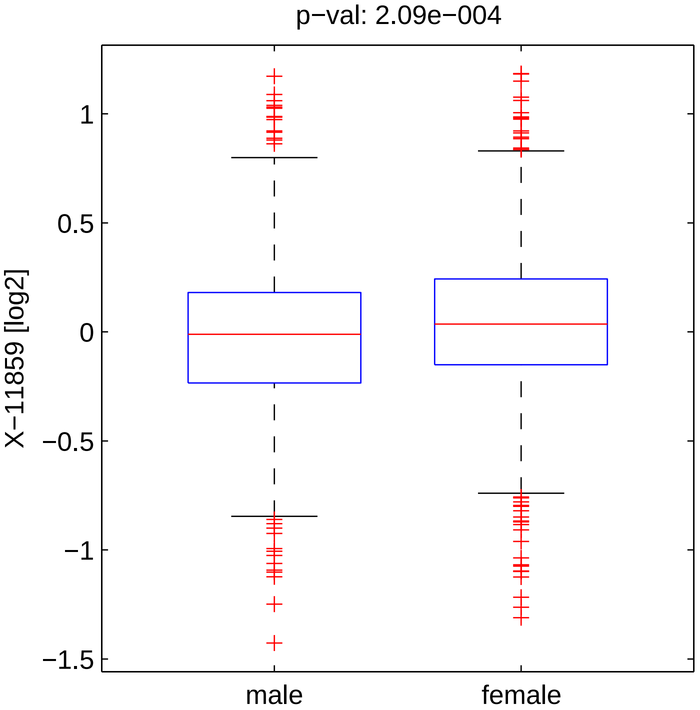

p−val: 2.09e−004
X−11859 [log2]
1
0.5
0
−0.5
−1
−1.5
male
female

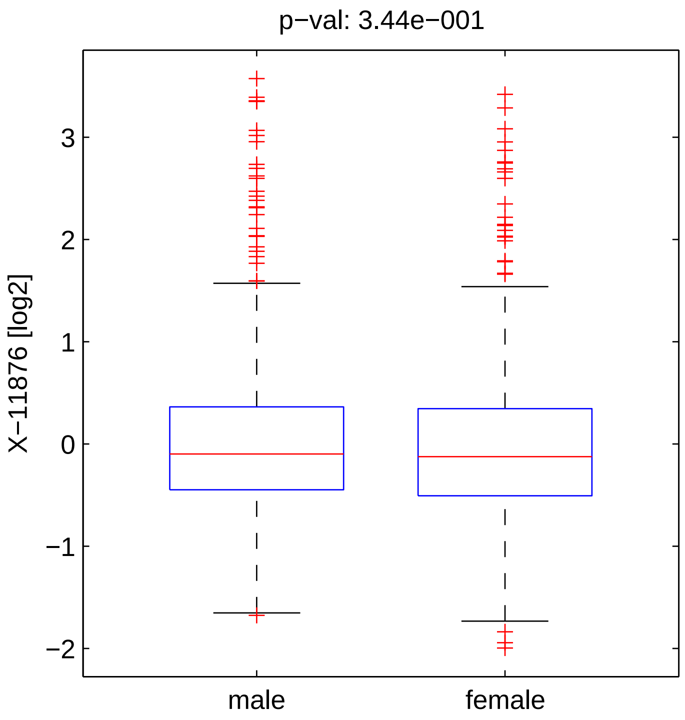
p−val: 3.44e−001
X−11876 [log2]
3
2
1
0
−1
−2
male
female

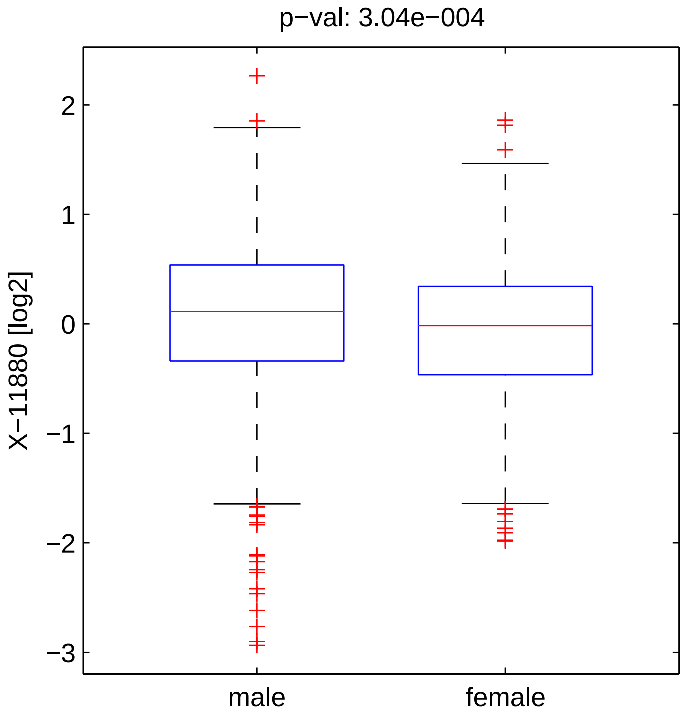

p−val: 3.04e−004
2
1
0
−1
−2
−3
X−11880 [log2]
male
female

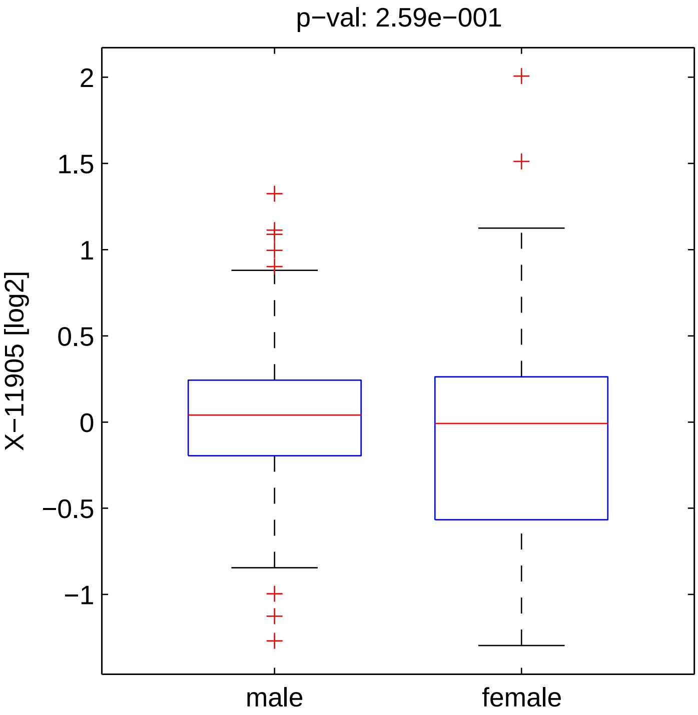
p−val: 2.59e−001
X−11905 [log2]
2
1.5
1
0.5
0
−0.5
−1
male
female

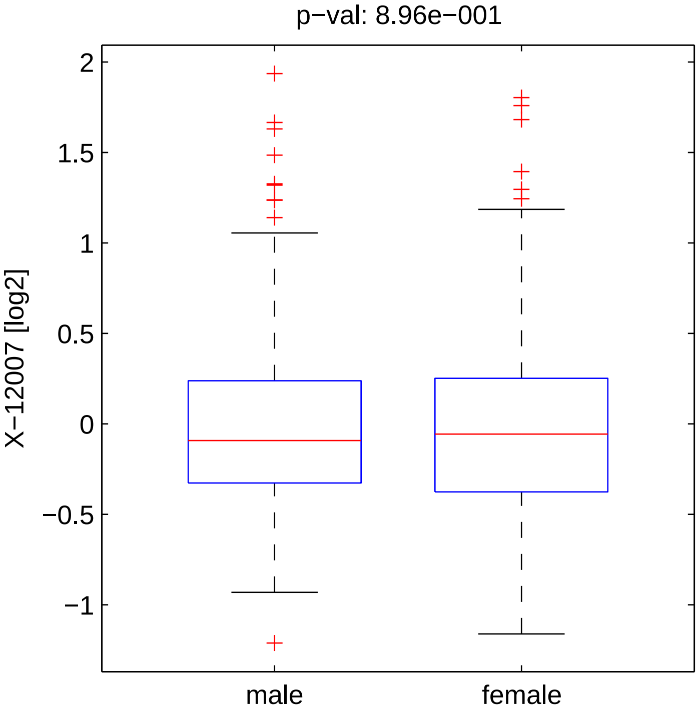
p−val: 8.96e−001
X−12007 [log2]
2
1.5
1
0.5
0
−0.5
−1
male
female

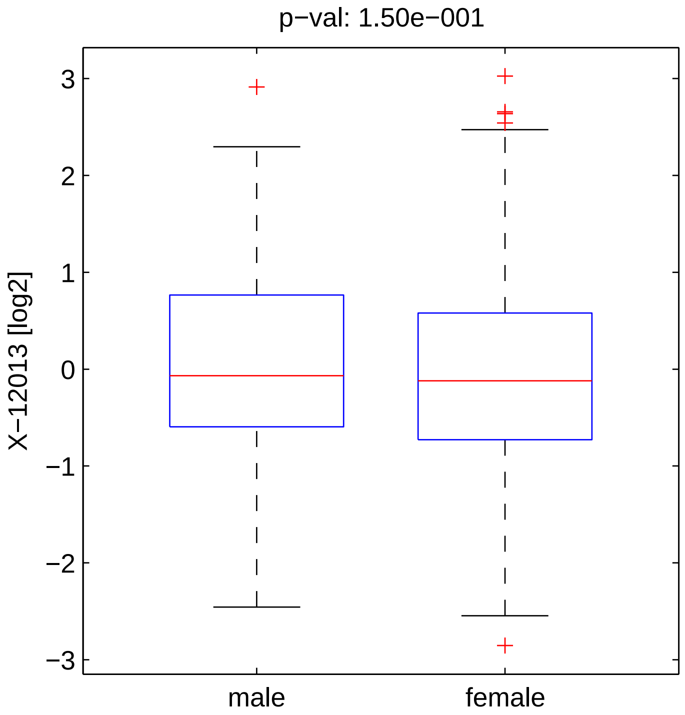
p−val: 1.50e−001
X−12013 [log2]
3
2
1
0
−1
−2
−3
male
female

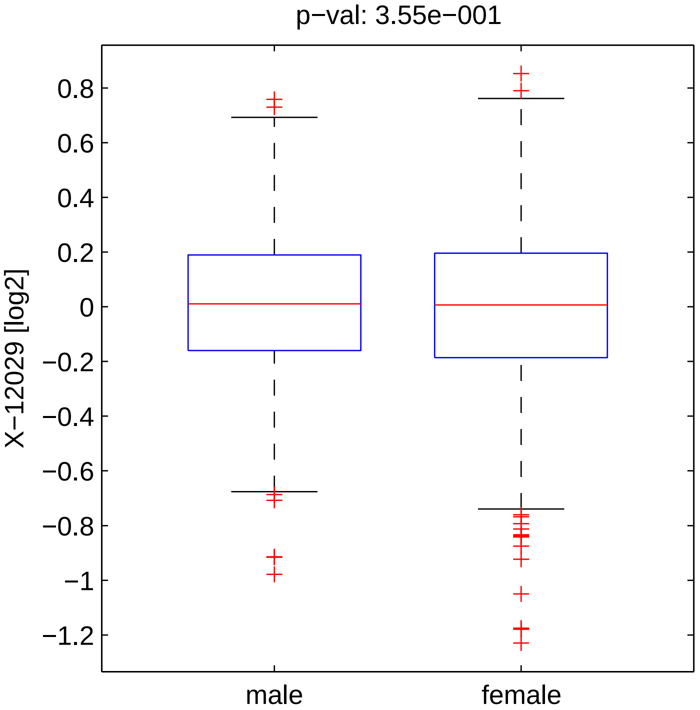
p−val: 3.55e−001
X−12029 [log2]
0.8
0.6
0.4
0.2
0
−0.2
−0.4
−0.6
−0.8
−1
−1.2
male
female

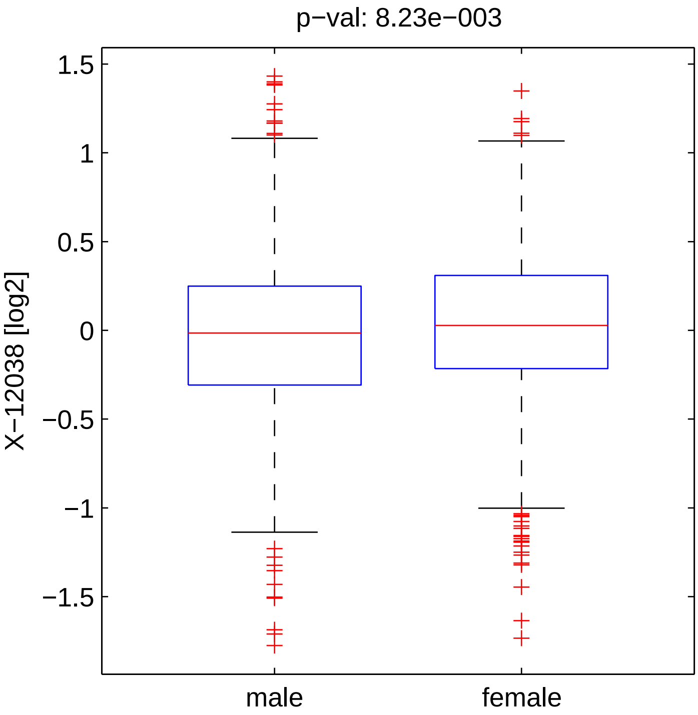
p−val: 8.23e−003
1.5
1
0.5
0
−0.5
−1
−1.5
X−12038 [log2]
male
female

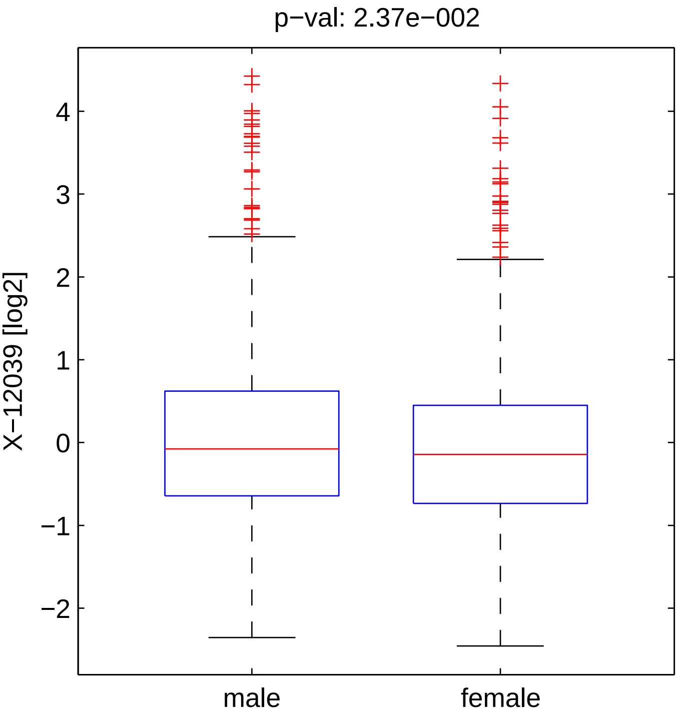
p−val: 2.37e−002
X−12039 [log2]
4
3
2
1
0
−1
−2
male
female

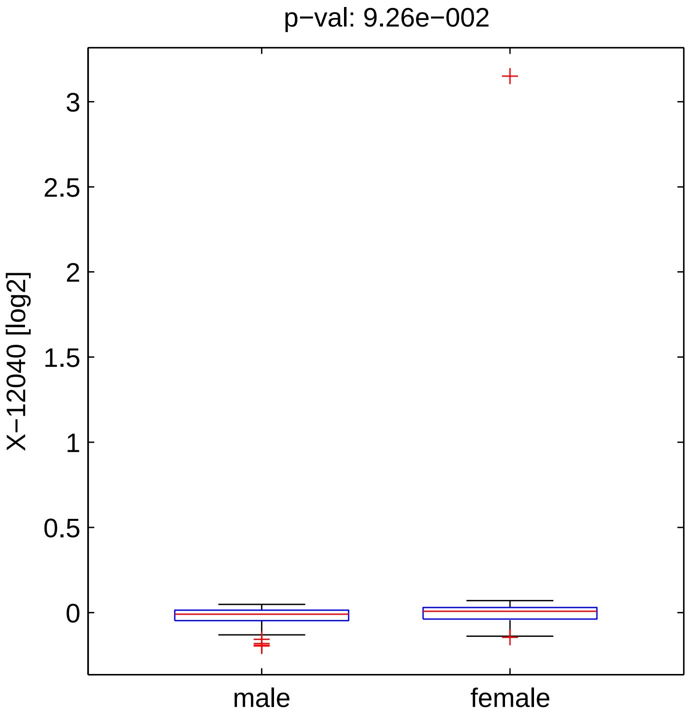

p−val: 9.26e−002
X−12040 [log2]
3
2.5
2
1.5
1
0.5
0
male
female

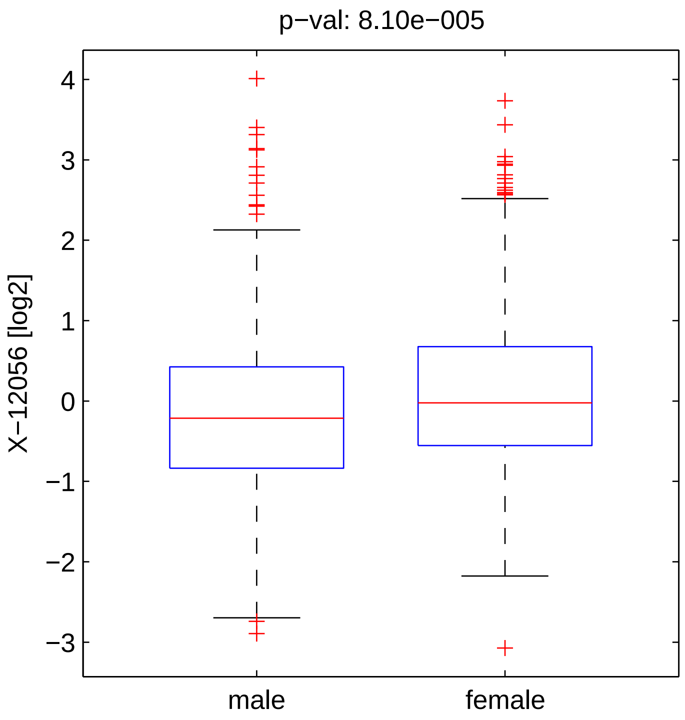
p−val: 8.10e−005
X−12056 [log2]
4
3
2
1
0
−1
−2
−3
male
female

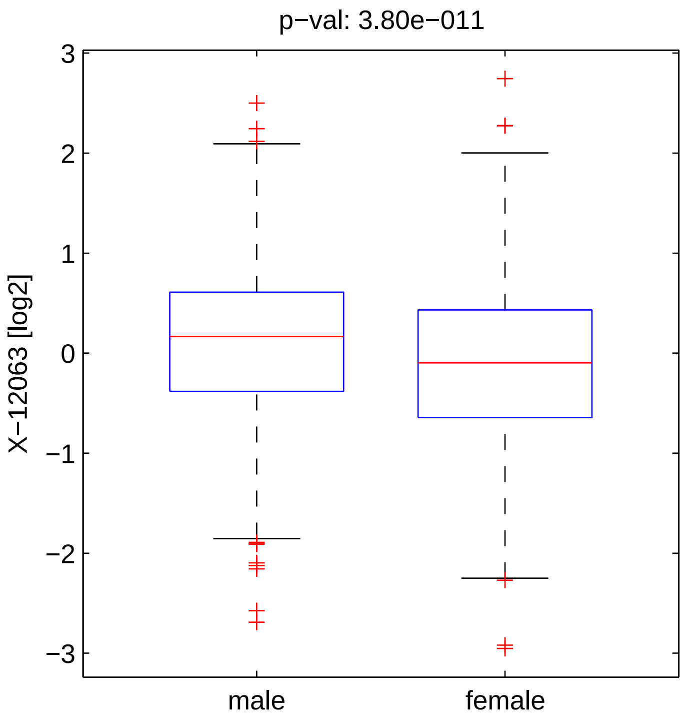
p−val: 3.80e−011
X−12063 [log2]
3
2
1
0
−1
−2
−3
male
female

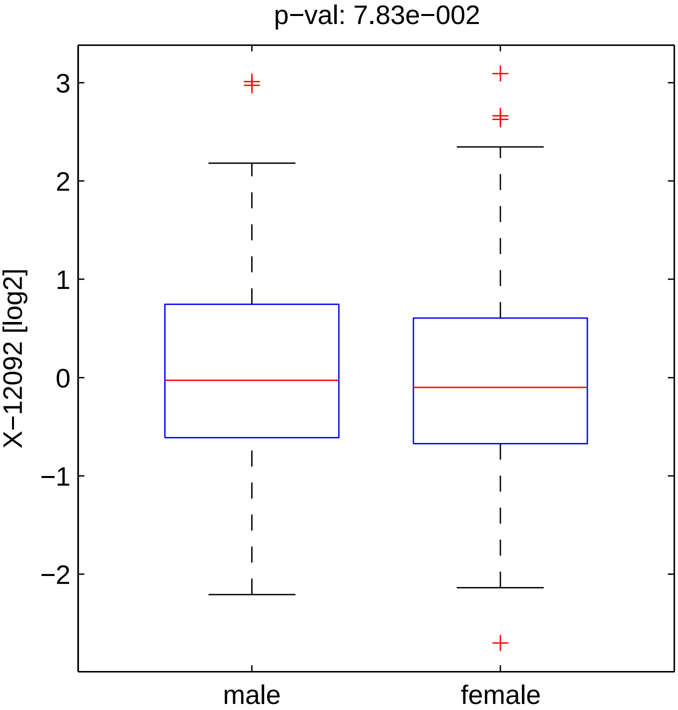
p−val: 7.83e−002
X−12092 [log2]
3
2
1
0
−1
−2
male
female

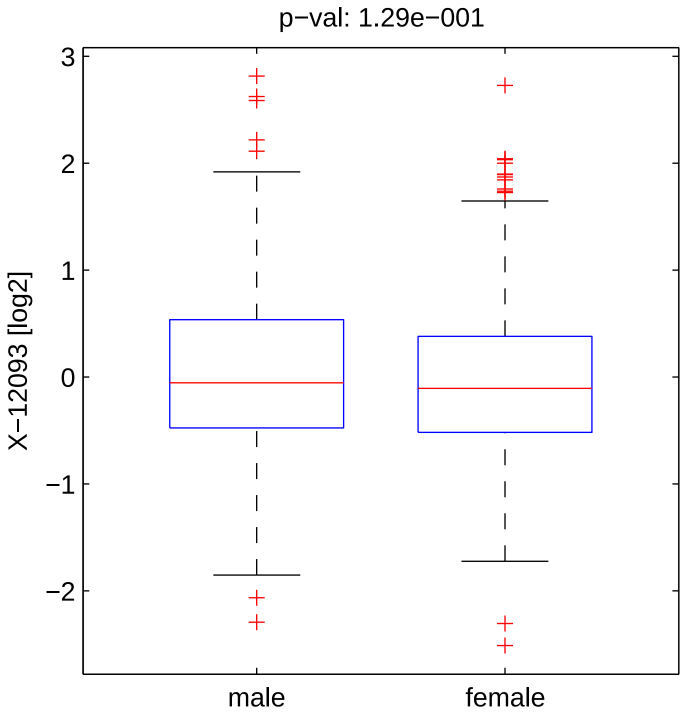

p−val: 1.29e−001
3
2
1
0
−1
−2
X−12093 [log2]
male
female

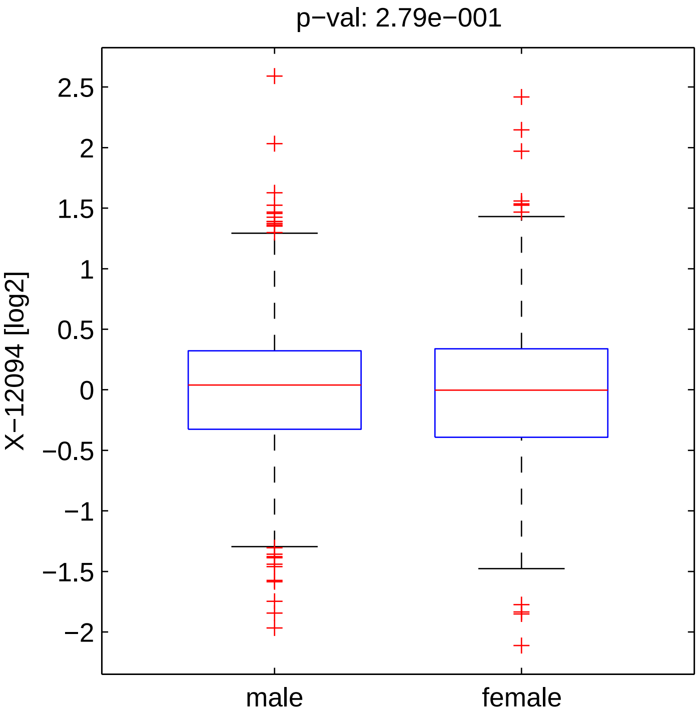

p−val: 2.79e−001
X−12094 [log2]
2.5
2
1.5
1
0.5
0
−0.5
−1
−1.5
−2
male
female

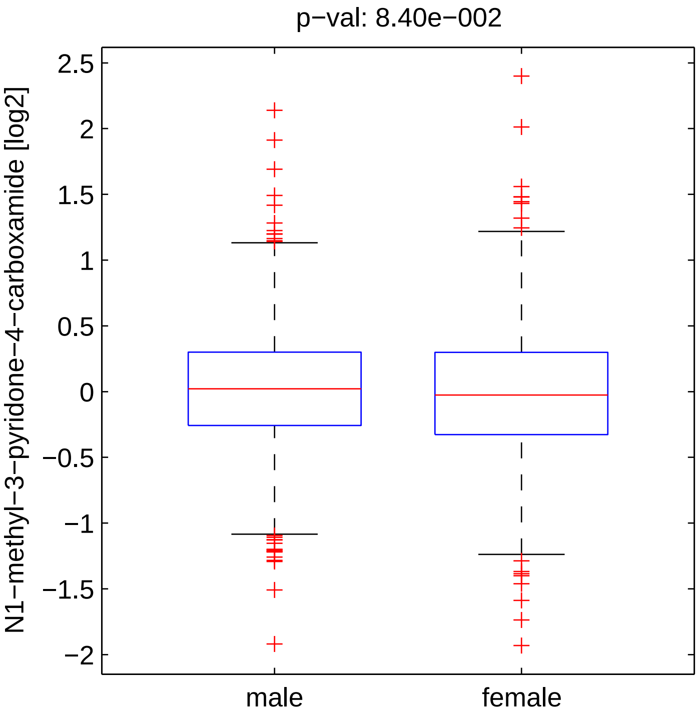
p−val: 8.40e−002
N1−methyl−3−pyridone−4−carboxamide [log2]
2.5
2
1.5
1
0.5
0
−0.5
−1
−1.5
−2
male
female

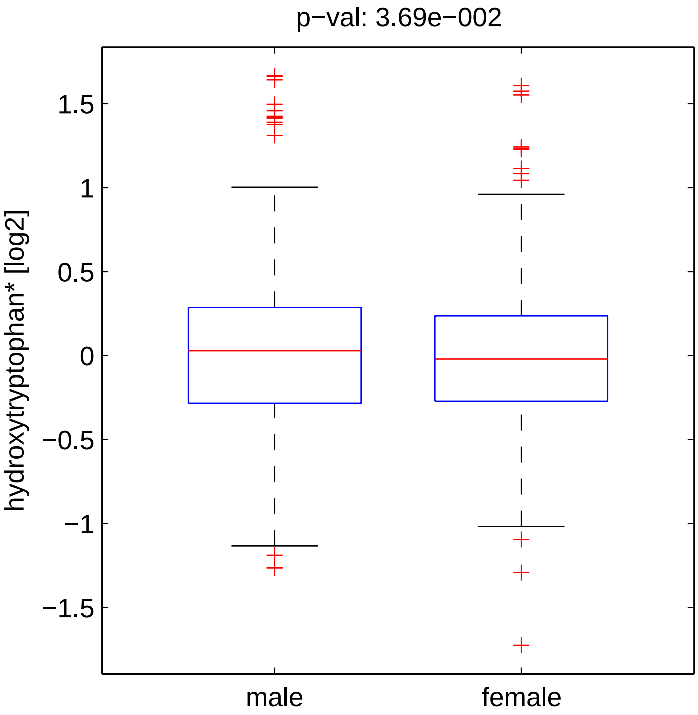

p−val: 3.69e−002
hydroxytryptophan* [log2]
1.5
1
0.5
0
−0.5
−1
−1.5
male
female

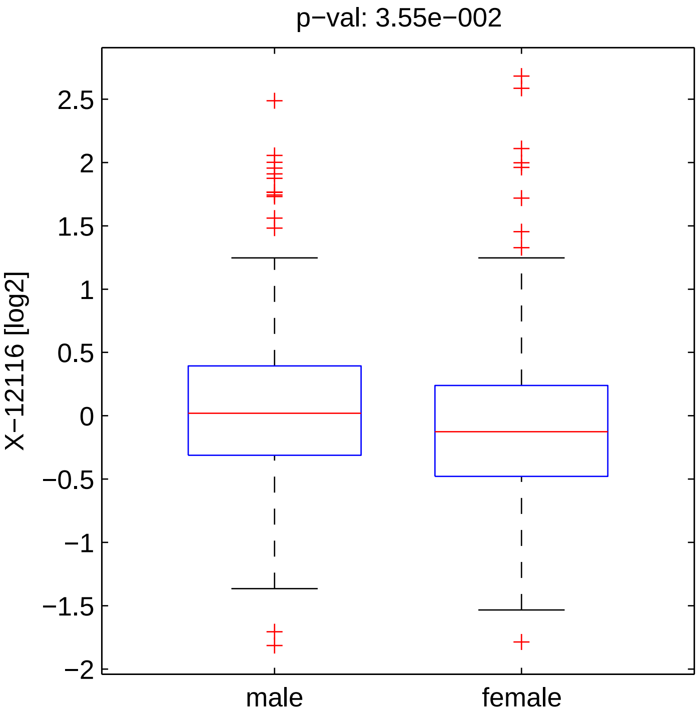
p−val: 3.55e−002
X−12116 [log2]
2.5
2
1.5
1
0.5
0
−0.5
−1
−1.5
−2
male
female

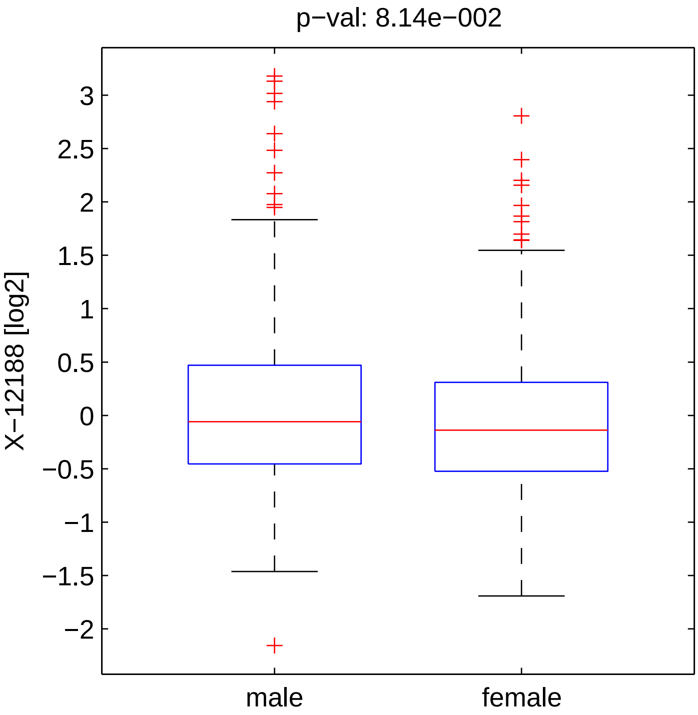

p−val: 8.14e−002
X−12188 [log2]
3
2.5
2
1.5
1
0.5
0
−0.5
−1
−1.5
−2
male
female

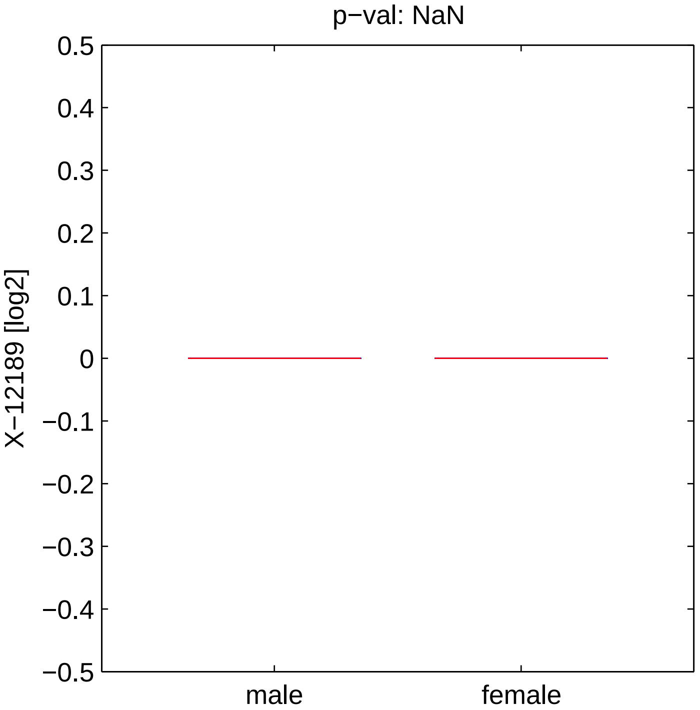

p−val: NaN
X−12189 [log2]
0.5
0.4
0.3
0.2
0.1
0
−0.1
−0.2
−0.3
−0.4
−0.5
male
female

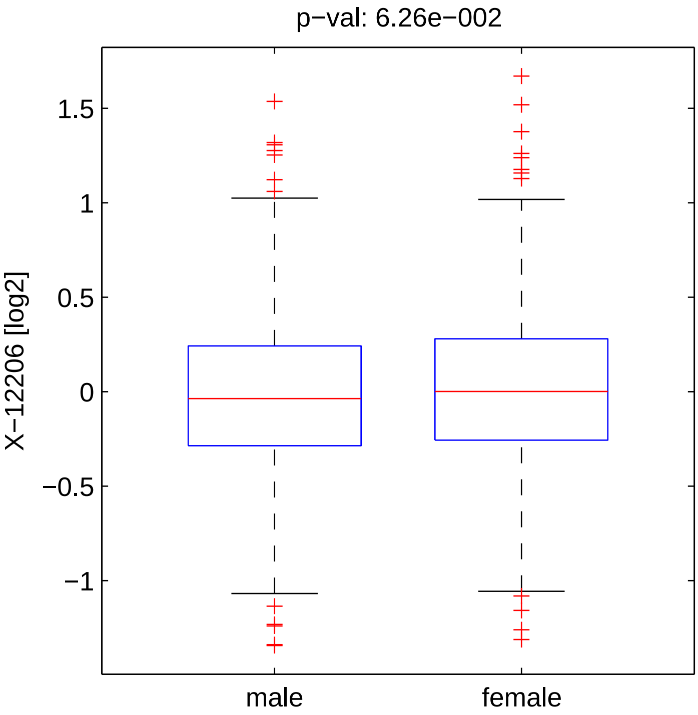

p−val: 6.26e−002
1.5
1
0.5
0
−0.5
−1
X−12206 [log2]
male
female

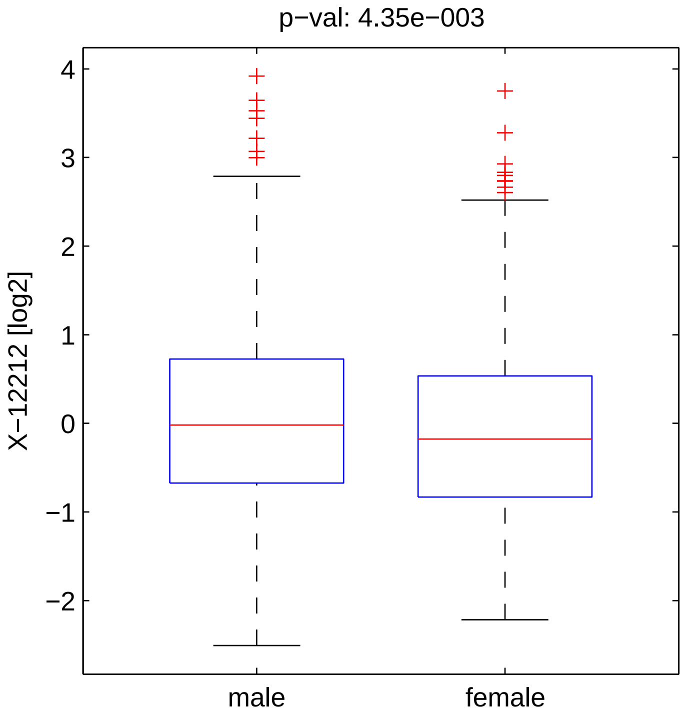

p−val: 4.35e−003
X−12212 [log2]
4
3
2
1
0
−1
−2
male
female

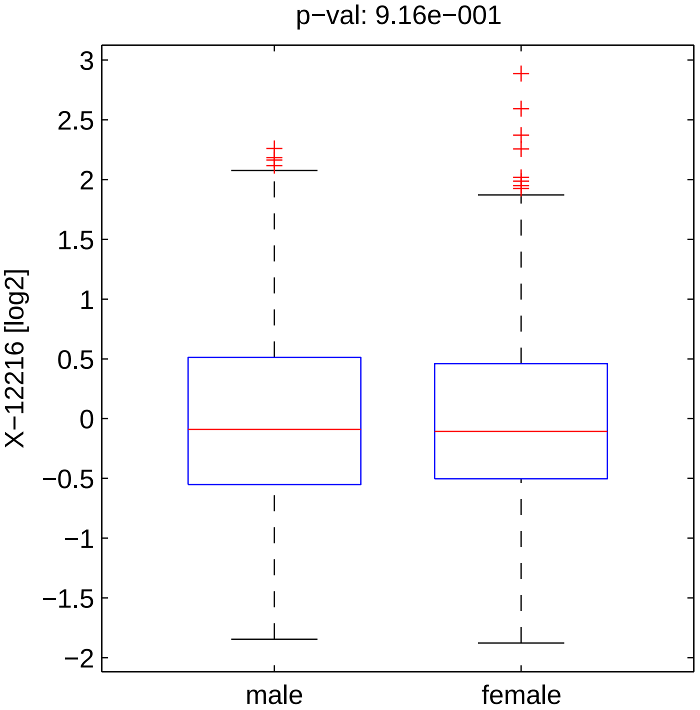
p−val: 9.16e−001
X−12216 [log2]
3
2.5
2
1.5
1
0.5
0
−0.5
−1
−1.5
−2
male
female

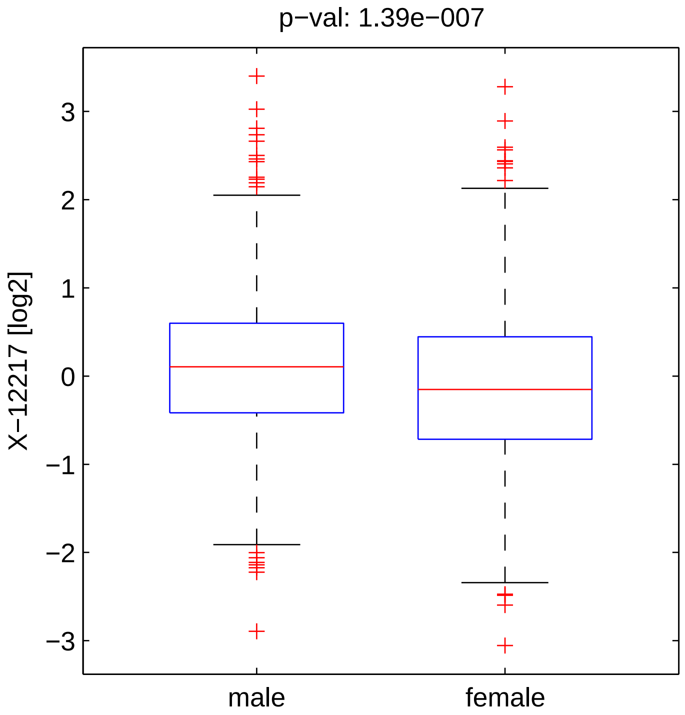
p−val: 1.39e−007
X−12217 [log2]
3
2
1
0
−1
−2
−3
male
female

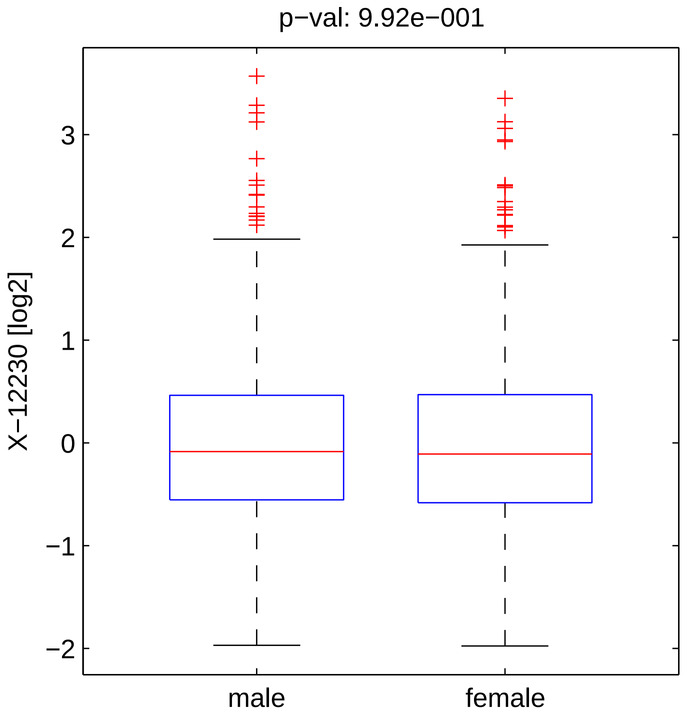
p−val: 9.92e−001
X−12230 [log2]
3
2
1
0
−1
−2
male
female

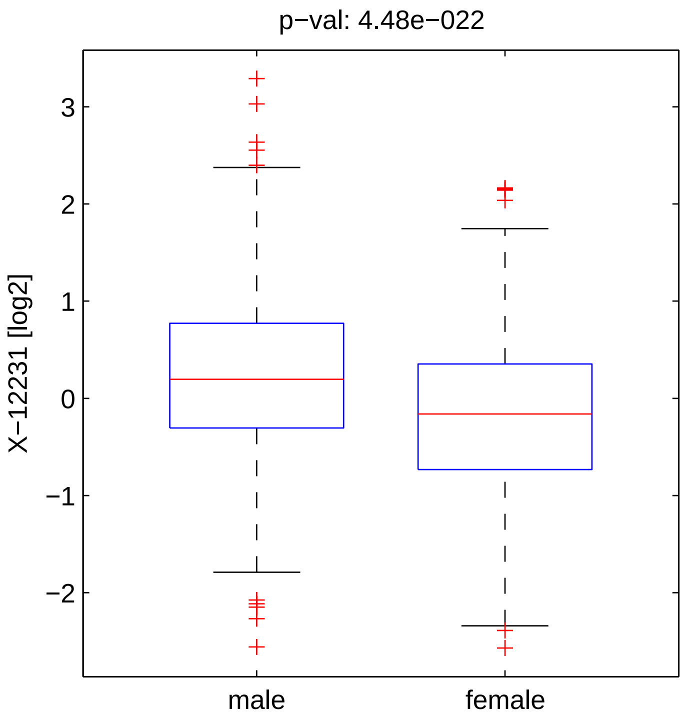
p−val: 4.48e−022
X−12231 [log2]
3
2
1
0
−1
−2
male
female

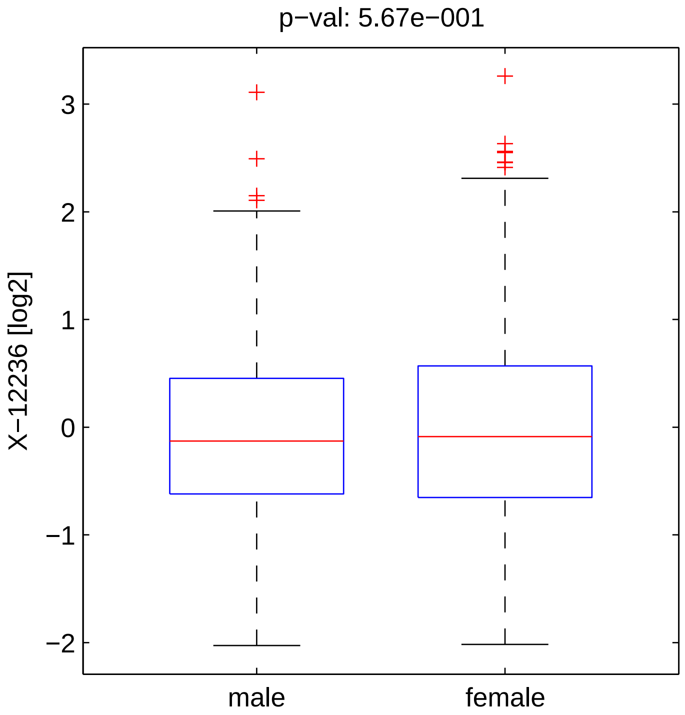

p−val: 5.67e−001
X−12236 [log2]
3
2
1
0
−1
−2
male
female

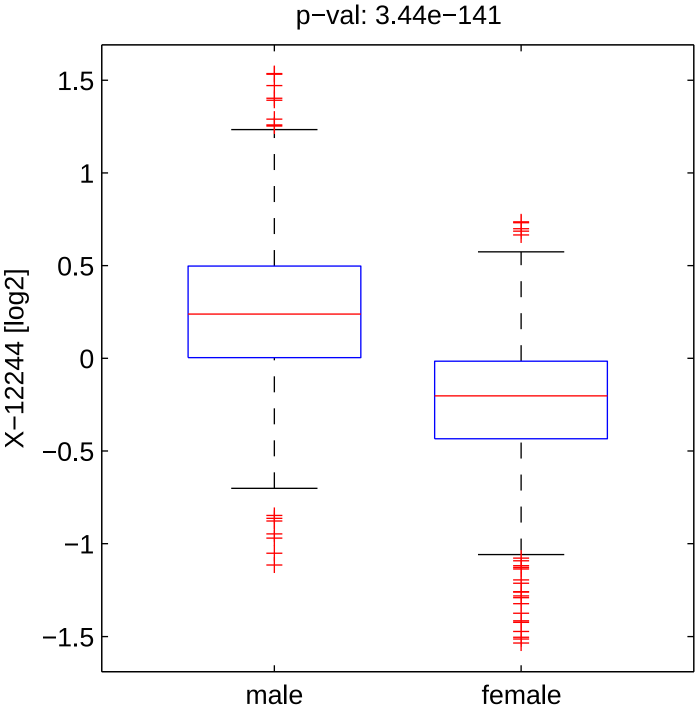

p−val: 3.44e−141
X−12244 [log2]
1.5
1
0.5
0
−0.5
−1
−1.5
male
female

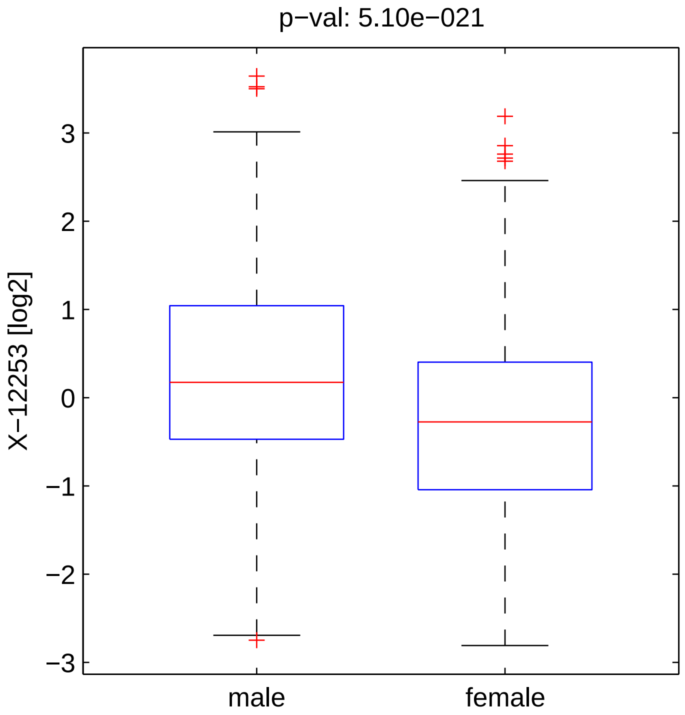

p−val: 5.10e−021
X−12253 [log2]
3
2
1
0
−1
−2
−3
male
female

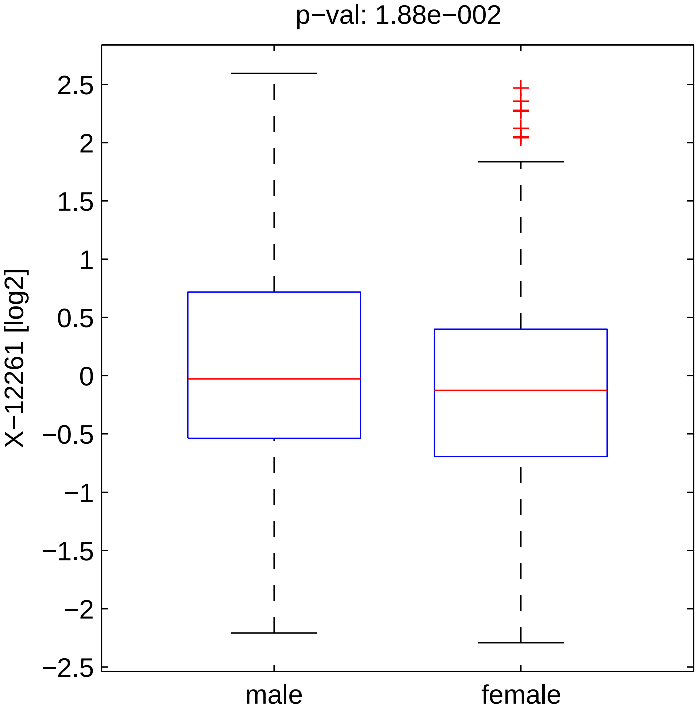
p−val: 1.88e−002
X−12261 [log2]
2.5
2
1.5
1
0.5
0
−0.5
−1
−1.5
−2
−2.5
male
female

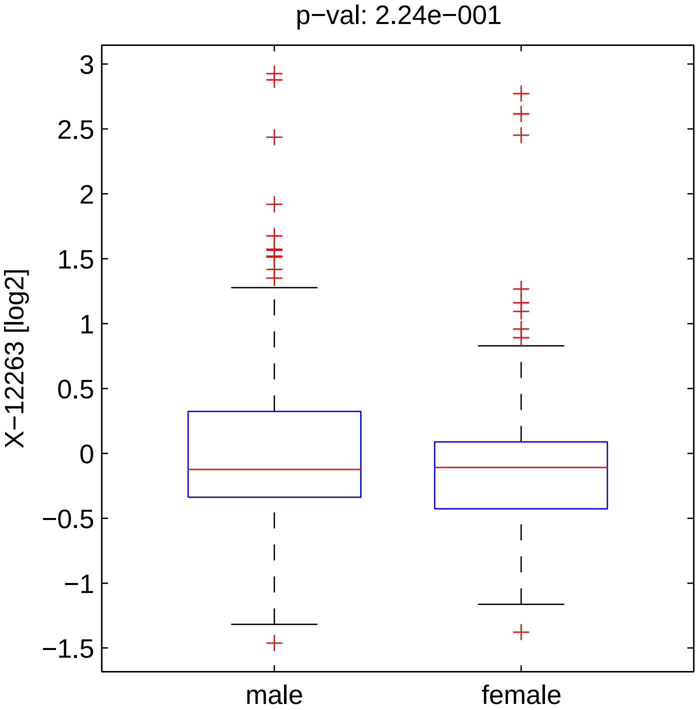
p−val: 2.24e−001
X−12263 [log2]
3
2.5
2
1.5
1
0.5
0
−0.5
−1
−1.5
male
female

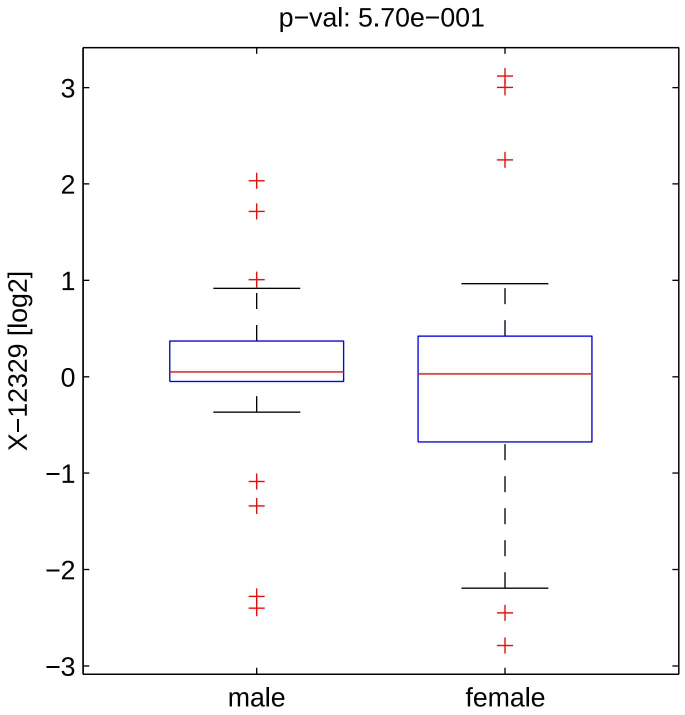
p−val: 5.70e−001
X−12329 [log2]
3
2
1
0
−1
−2
−3
male
female

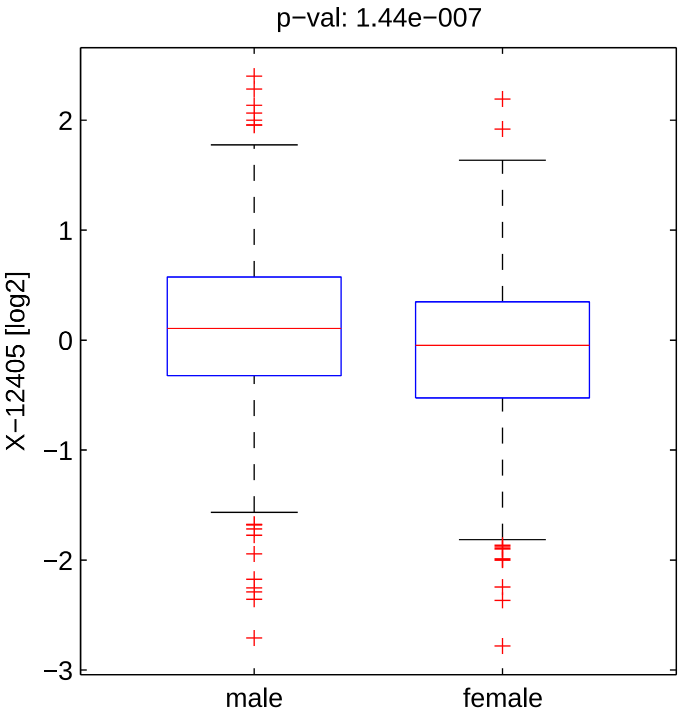
p−val: 1.44e−007
X−12405 [log2]
2
1
0
−1
−2
−3
male
female

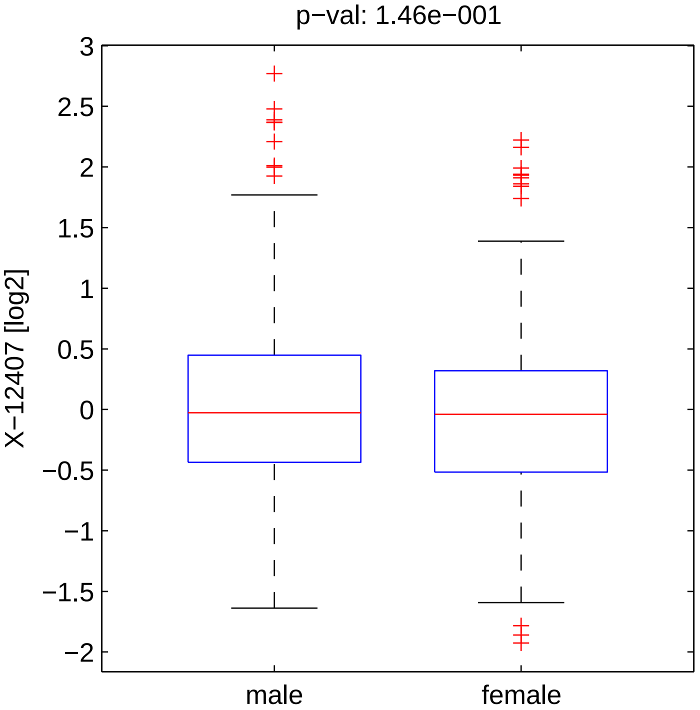

p−val: 1.46e−001
3
2.5
2
1.5
1
0.5
0
−0.5
−1
−1.5
−2
X−12407 [log2]
male
female

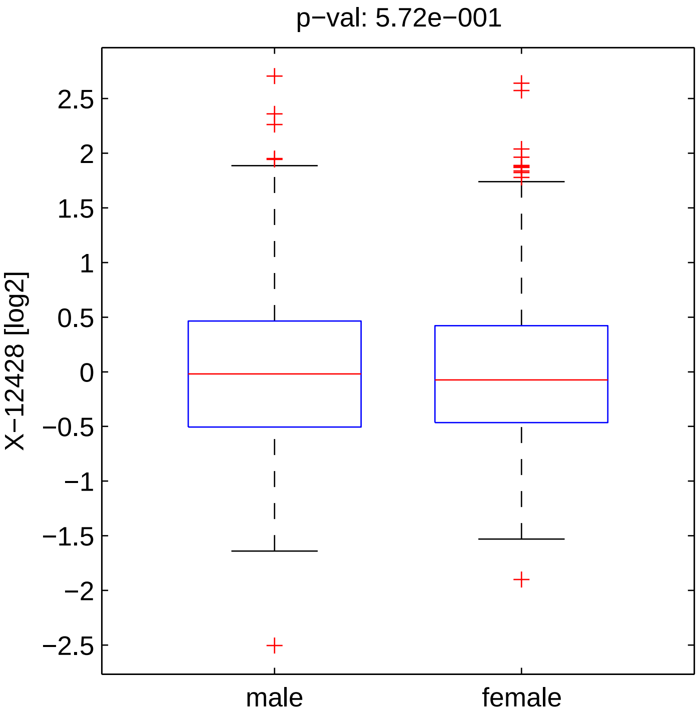
p−val: 5.72e−001
X−12428 [log2]
2.5
2
1.5
1
0.5
0
−0.5
−1
−1.5
−2
−2.5
male
female

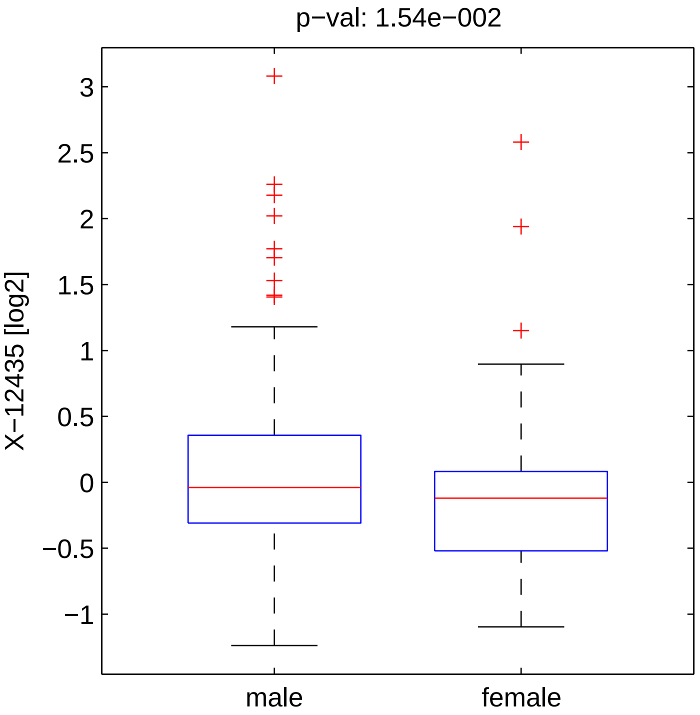
p−val: 1.54e−002
X−12435 [log2]
3
2.5
2
1.5
1
0.5
0
−0.5
−1
male
female

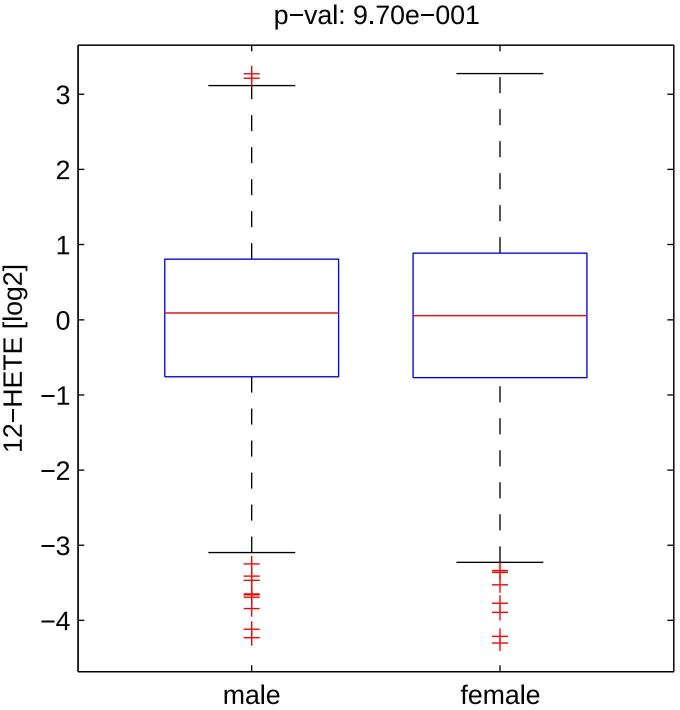

p−val: 9.70e−001
12−HETE [log2]
3
2
1
0
−1
−2
−3
−4
male
female

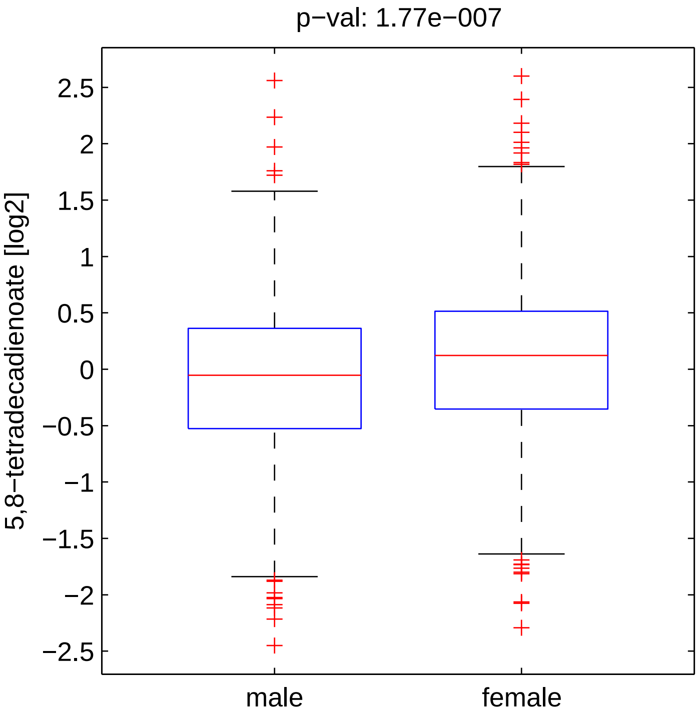

p−val: 1.77e−007
5,8−tetradecadienoate [log2]
2.5
2
1.5
1
0.5
0
−0.5
−1
−1.5
−2
−2.5
male
female

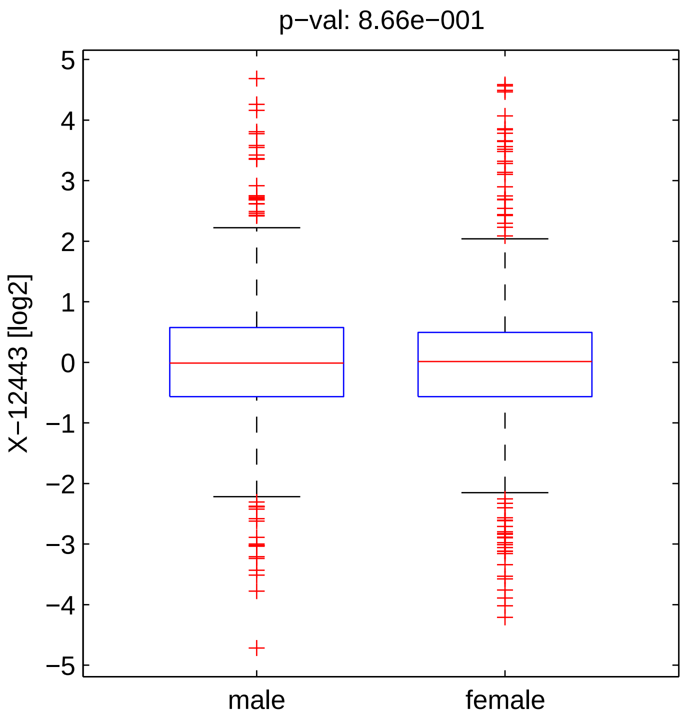
p−val: 8.66e−001
X−12443 [log2]
5
4
3
2
1
0
−1
−2
−3
−4
−5
male
female

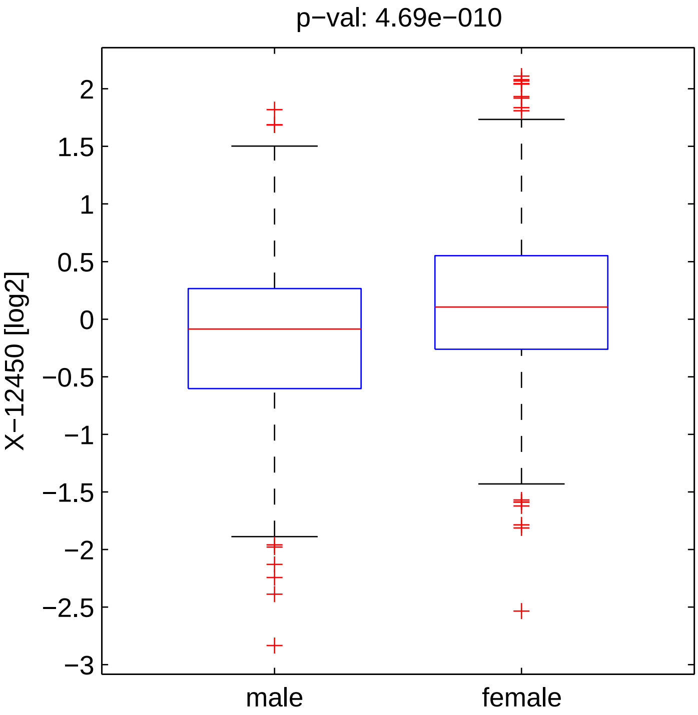
p−val: 4.69e−010
X−12450 [log2]
2
1.5
1
0.5
0
−0.5
−1
−1.5
−2
−2.5
−3
male
female

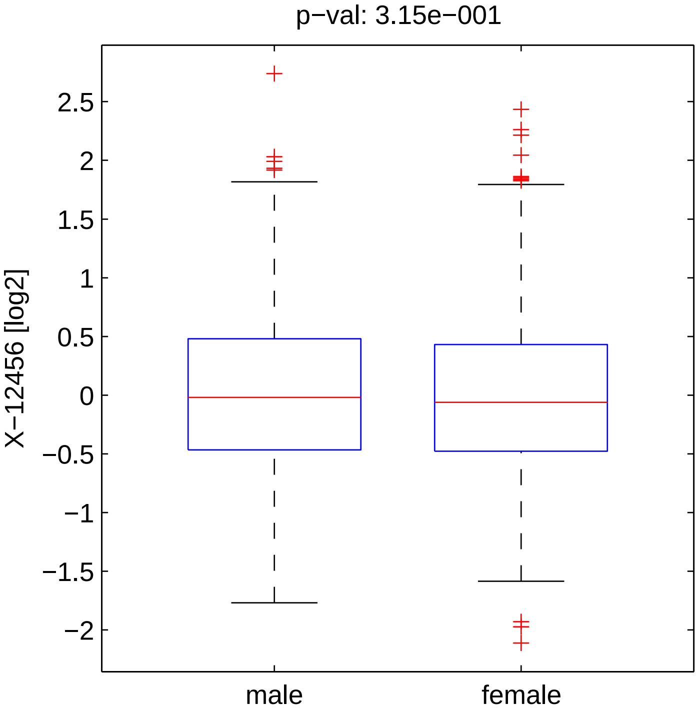

p−val: 3.15e−001
X−12456 [log2]
2.5
2
1.5
1
0.5
0
−0.5
−1
−1.5
−2
male
female

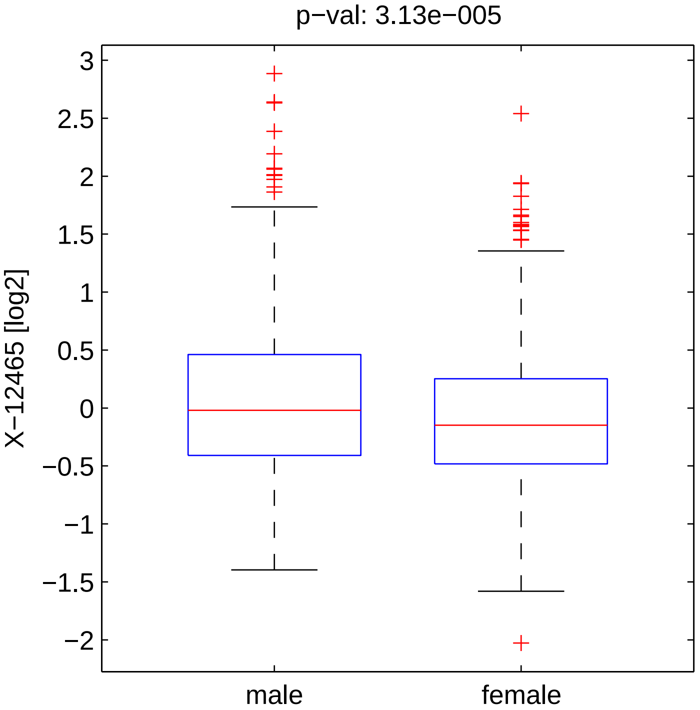
p−val: 3.13e−005
X−12465 [log2]
3
2.5
2
1.5
1
0.5
0
−0.5
−1
−1.5
−2
male
female

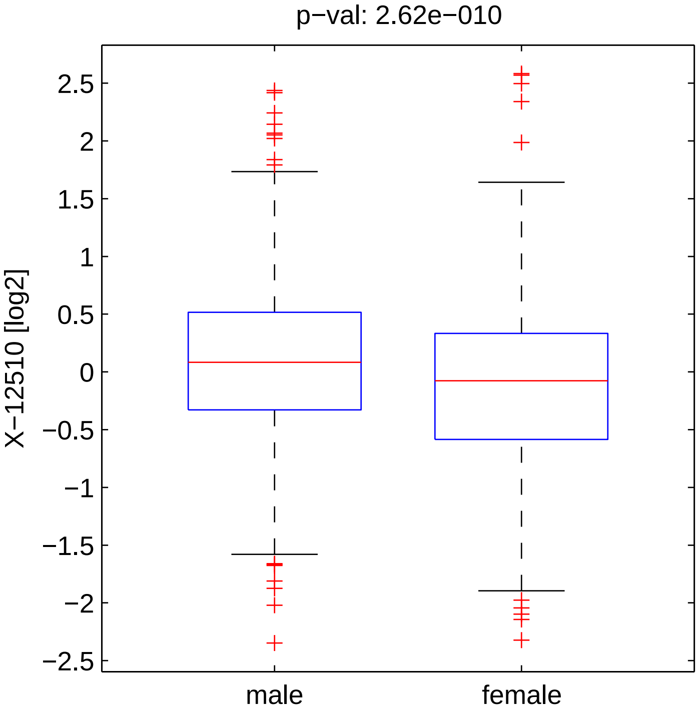
p−val: 2.62e−010
X−12510 [log2]
2.5
2
1.5
1
0.5
0
−0.5
−1
−1.5
−2
−2.5
male
female

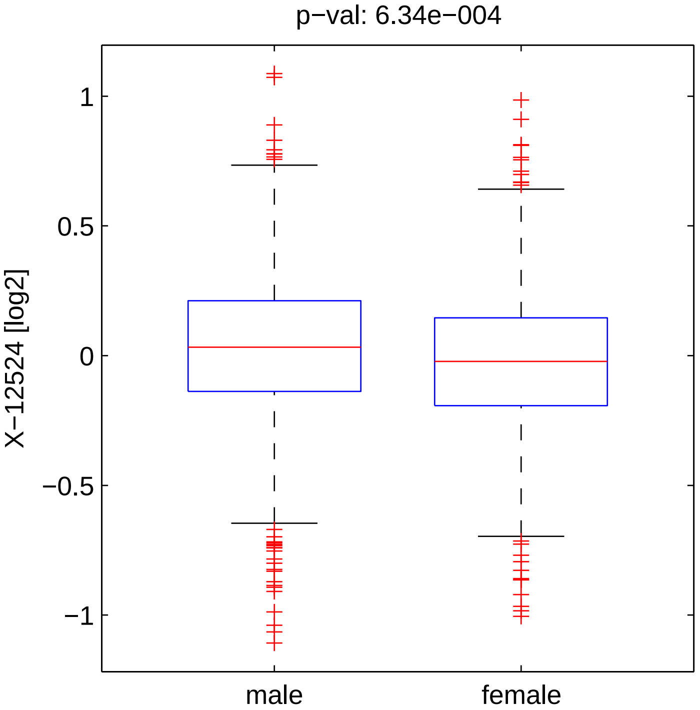
p−val: 6.34e−004
X−12524 [log2]
1
0.5
0
−0.5
−1
male
female

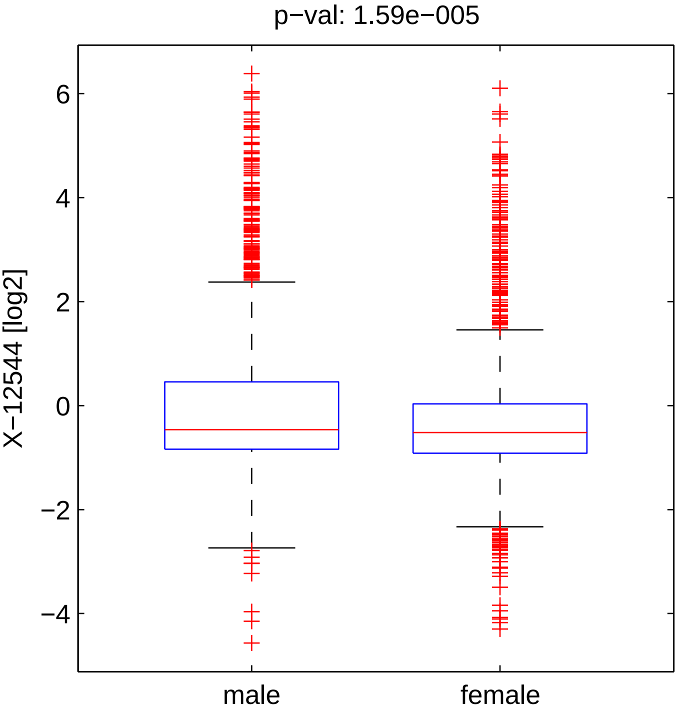

p−val: 1.59e−005
X−12544 [log2]
6
4
2
0
−2
−4
male
female

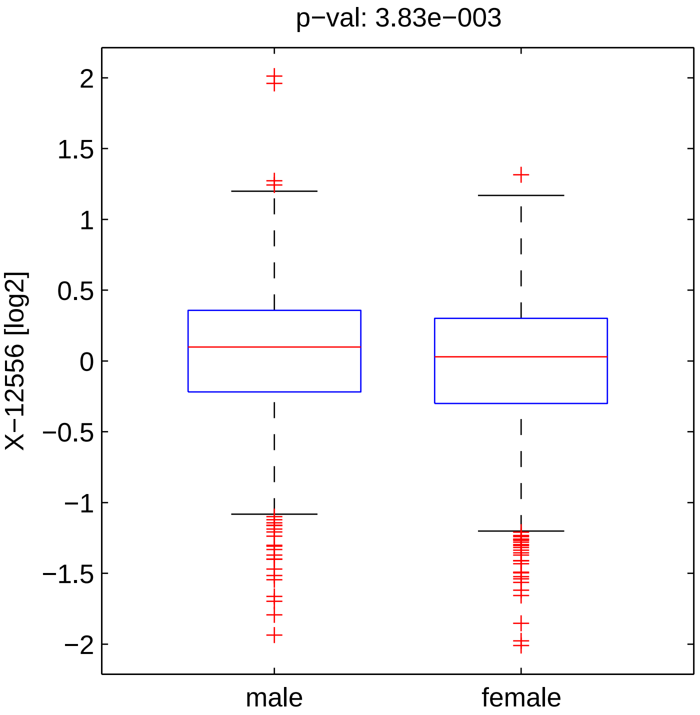
p−val: 3.83e−003
X−12556 [log2]
2
1.5
1
0.5
0
−0.5
−1
−1.5
−2
male
female

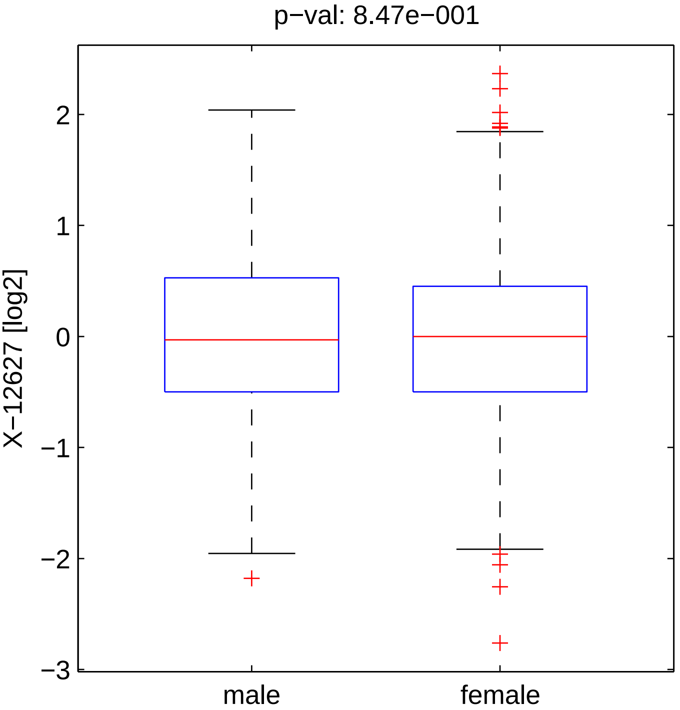
p−val: 8.47e−001
X−12627 [log2]
2
1
0
−1
−2
−3
male
female

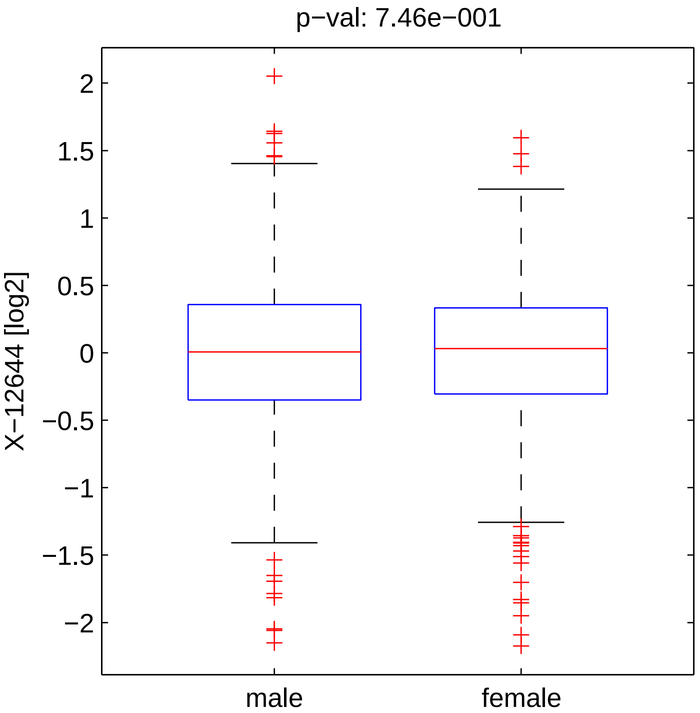

p−val: 7.46e−001
X−12644 [log2]
2
1.5
1
0.5
0
−0.5
−1
−1.5
−2
male
female

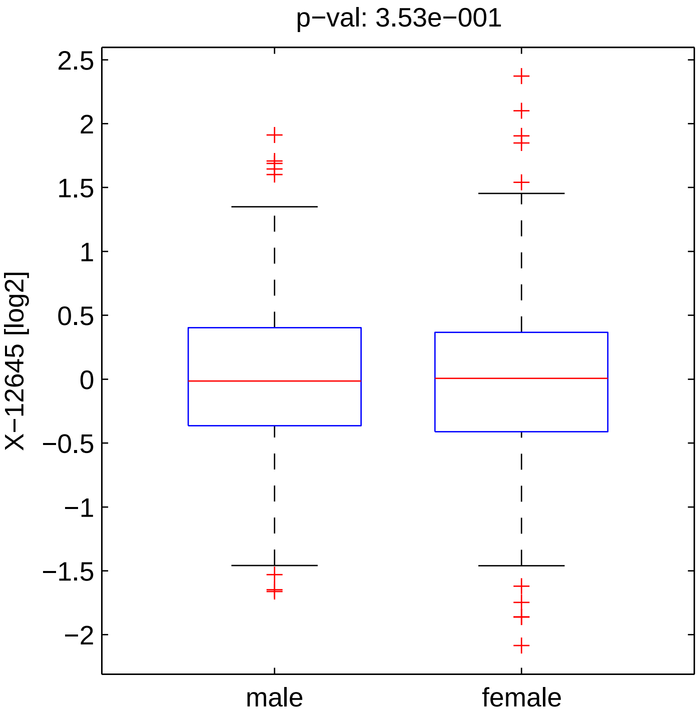
p−val: 3.53e−001
X−12645 [log2]
2.5
2
1.5
1
0.5
0
−0.5
−1
−1.5
−2
male
female

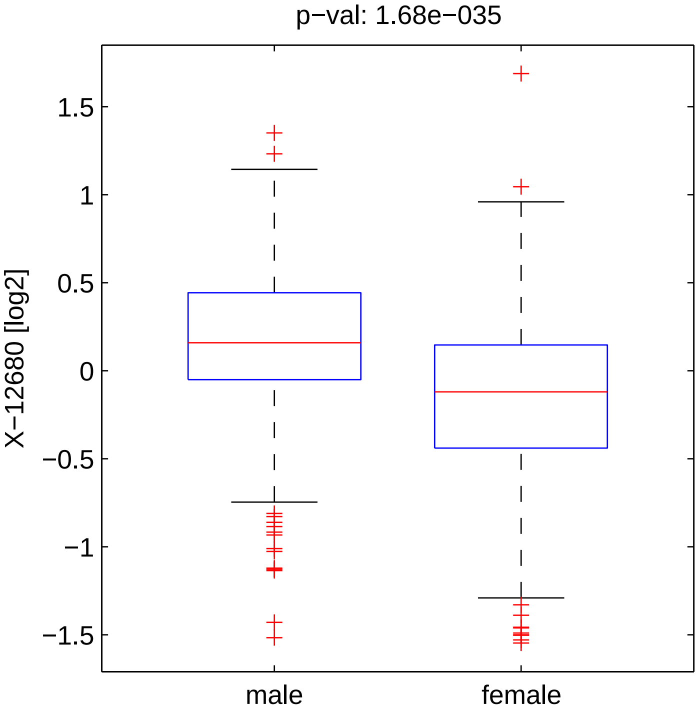
p−val: 1.68e−035
X−12680 [log2]
1.5
1
0.5
0
−0.5
−1
−1.5
male
female

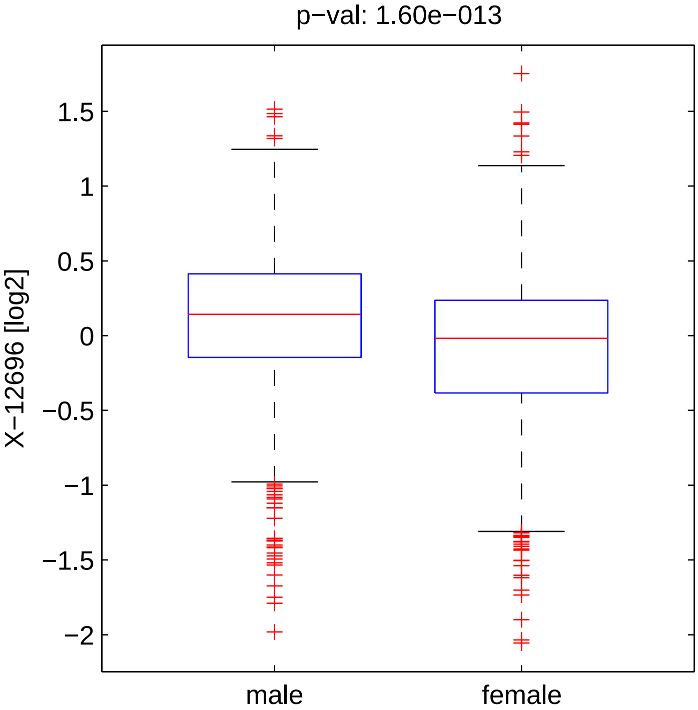
p−val: 1.60e−013
X−12696 [log2]
1.5
1
0.5
0
−0.5
−1
−1.5
−2
male
female

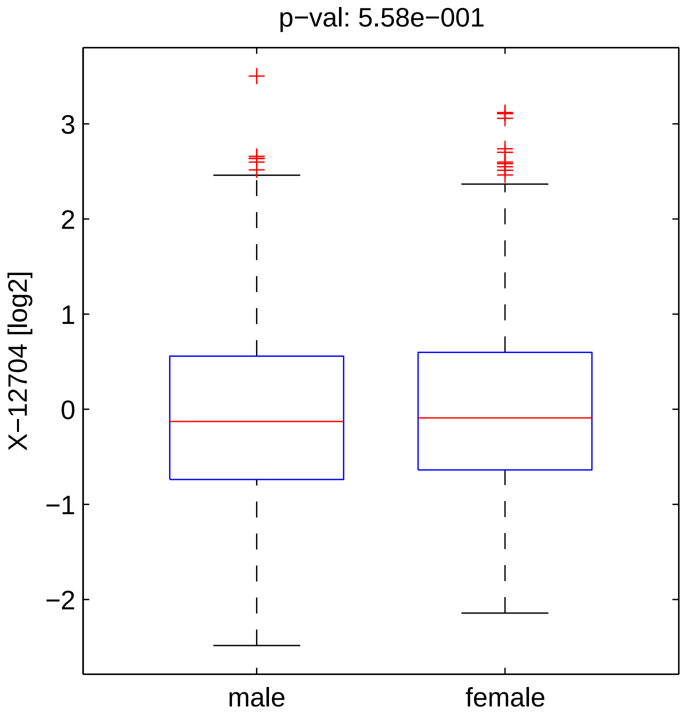

p−val: 5.58e−001
X−12704 [log2]
3
2
1
0
−1
−2
male
female

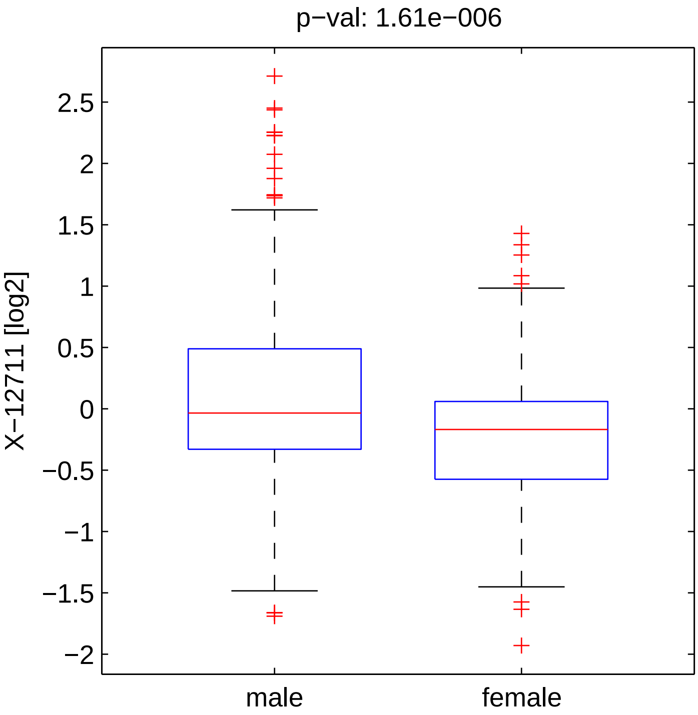

p−val: 1.61e−006
X−12711 [log2]
2.5
2
1.5
1
0.5
0
−0.5
−1
−1.5
−2
male
female

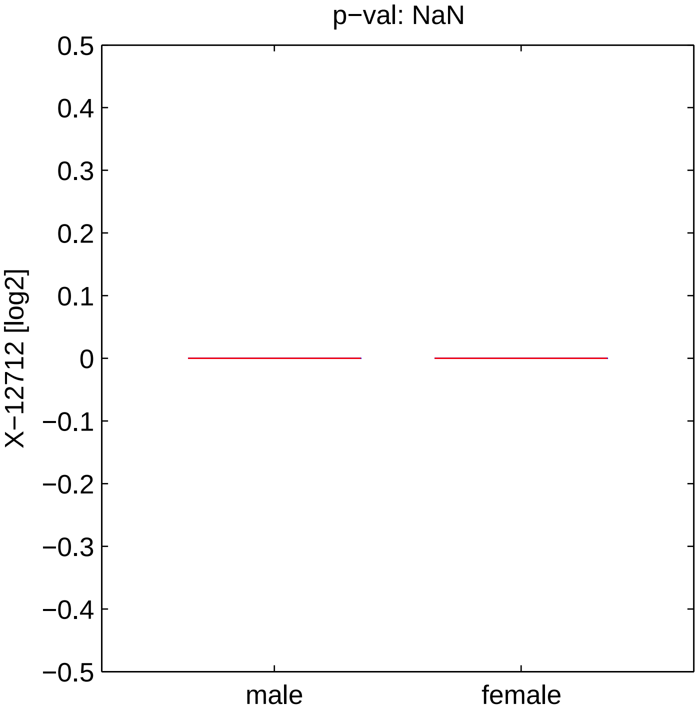
p−val: NaN
X−12712 [log2]
0.5
0.4
0.3
0.2
0.1
0
−0.1
−0.2
−0.3
−0.4
−0.5
male
female

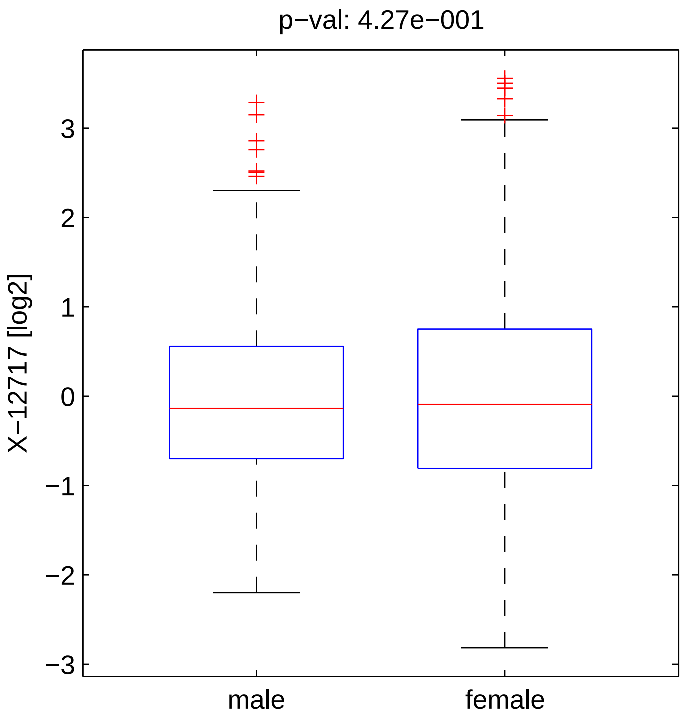
p−val: 4.27e−001
X−12717 [log2]
3
2
1
0
−1
−2
−3
male
female

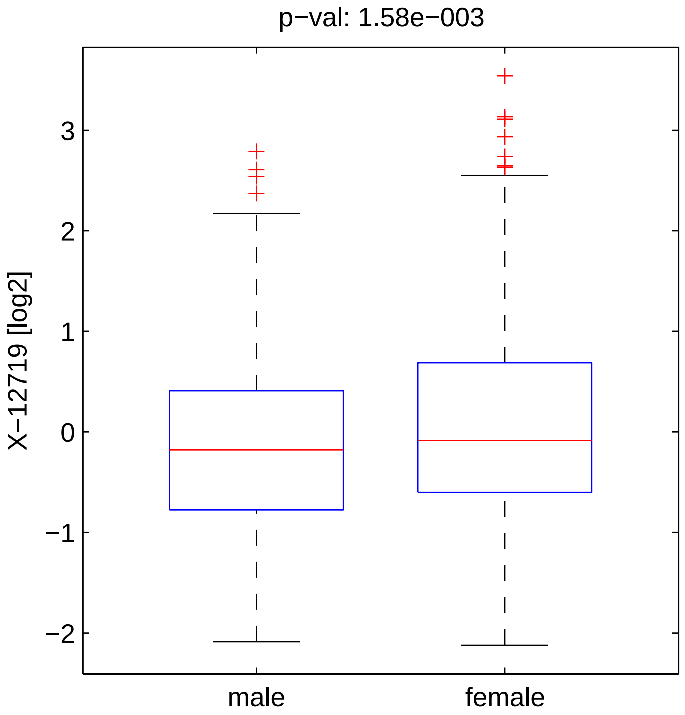

p−val: 1.58e−003
X−12719 [log2]
3
2
1
0
−1
−2
male
female

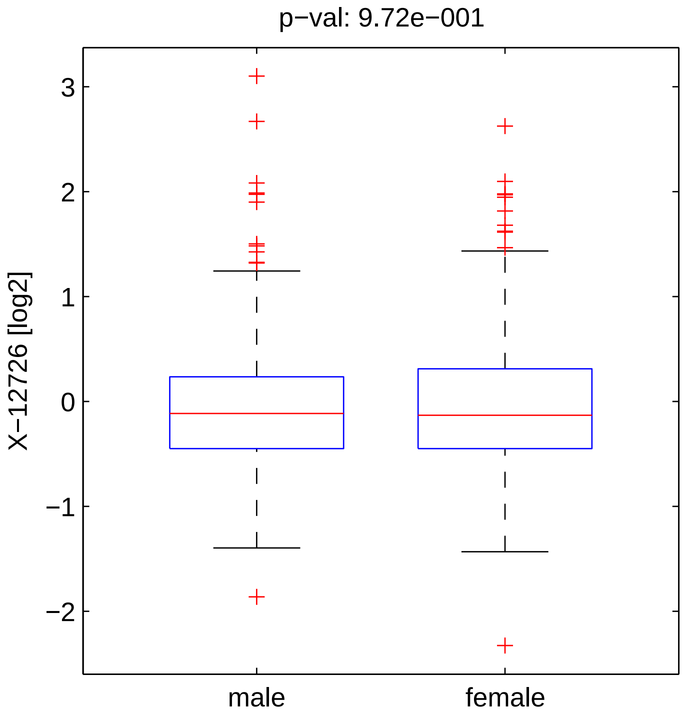

p−val: 9.72e−001
3
2
1
0
−1
−2
X−12726 [log2]
male
female

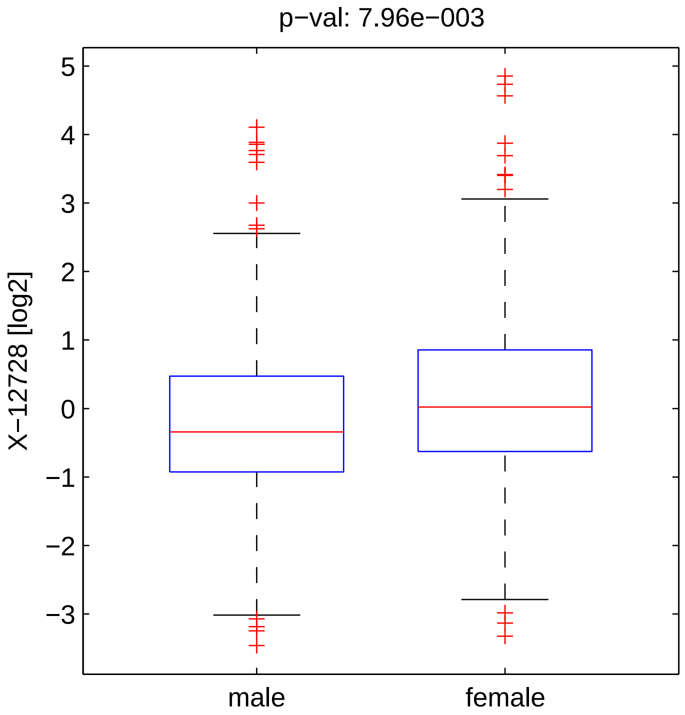
p−val: 7.96e−003
X−12728 [log2]
5
4
3
2
1
0
−1
−2
−3
male
female

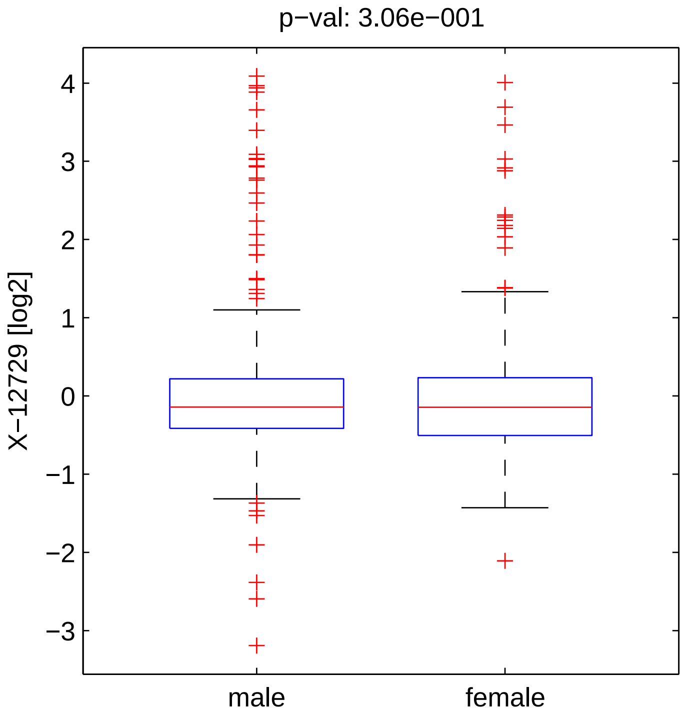
p−val: 3.06e−001
X−12729 [log2]
4
3
2
1
0
−1
−2
−3
male
female

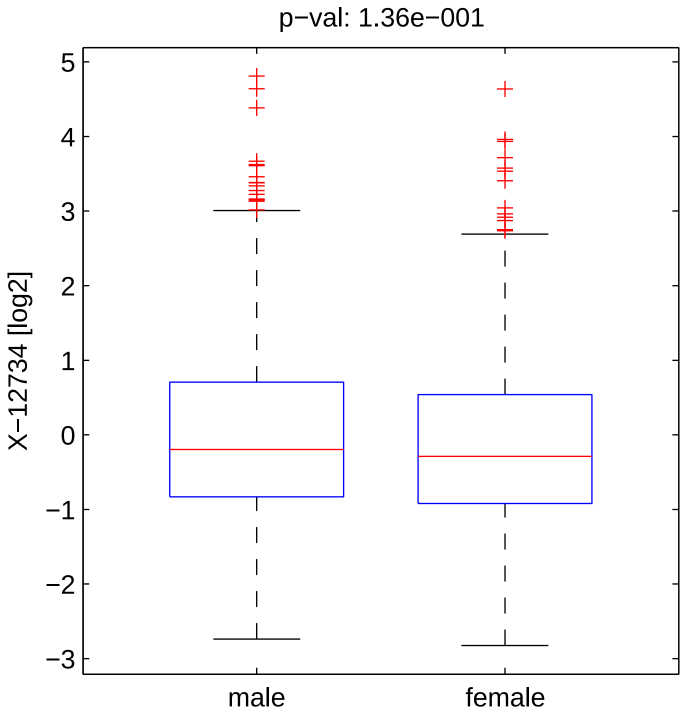
p−val: 1.36e−001
X−12734 [log2]
5
4
3
2
1
0
−1
−2
−3
male
female

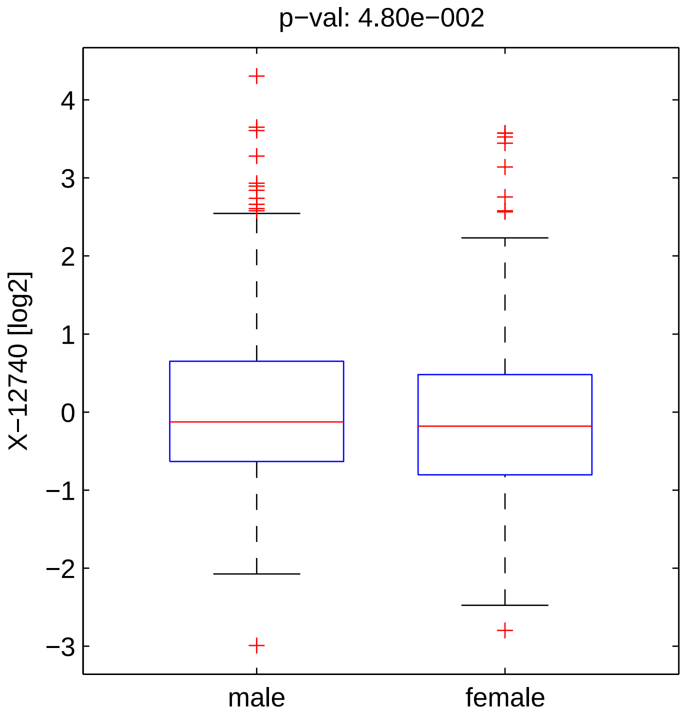
p−val: 4.80e−002
X−12740 [log2]
4
3
2
1
0
−1
−2
−3
male
female

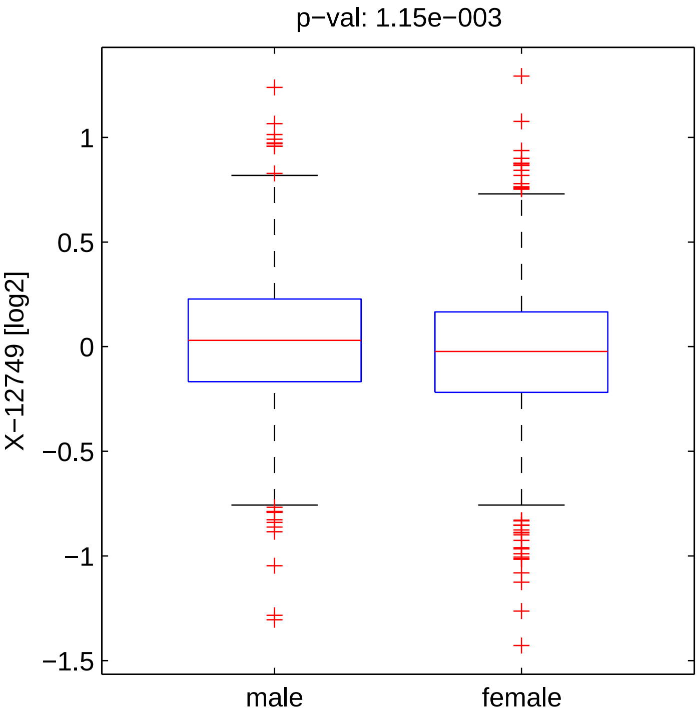

p−val: 1.15e−003
X−12749 [log2]
1
0.5
0
−0.5
−1
−1.5
male
female

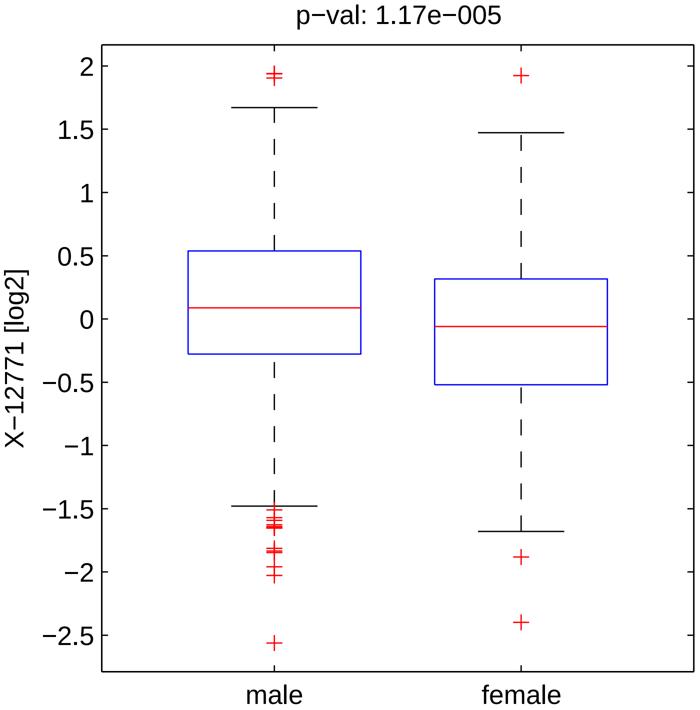
p−val: 1.17e−005
X−12771 [log2]
2
1.5
1
0.5
0
−0.5
−1
−1.5
−2
−2.5
male
female

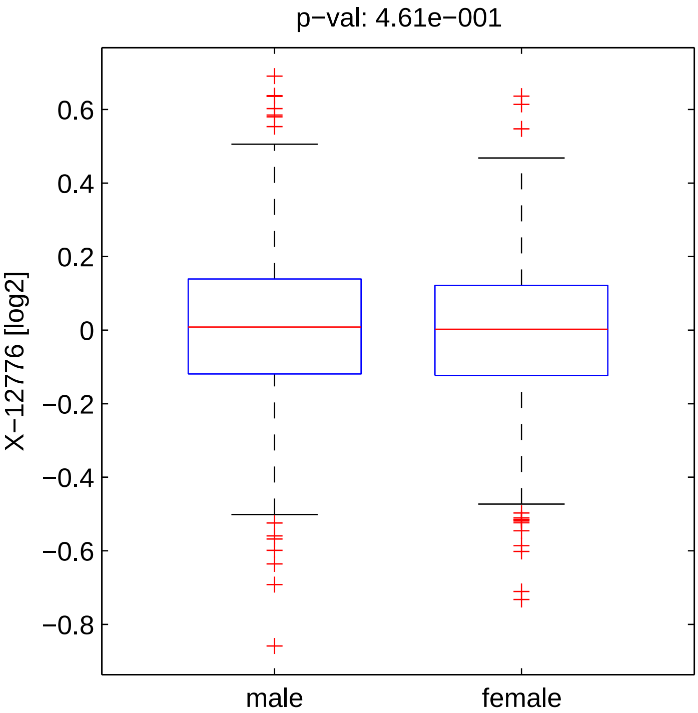
p−val: 4.61e−001
X−12776 [log2]
0.6
0.4
0.2
0
−0.2
−0.4
−0.6
−0.8
male
female

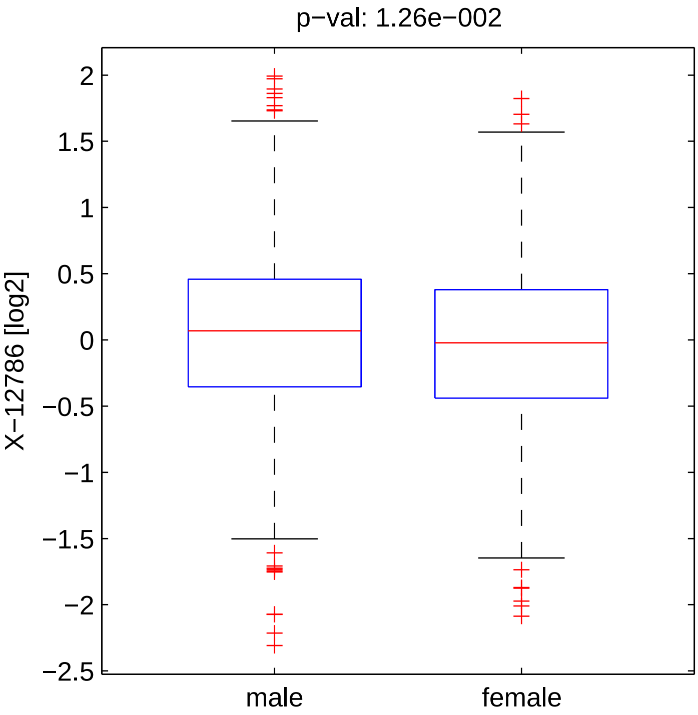
p−val: 1.26e−002
X−12786 [log2]
2
1.5
1
0.5
0
−0.5
−1
−1.5
−2
−2.5
male
female

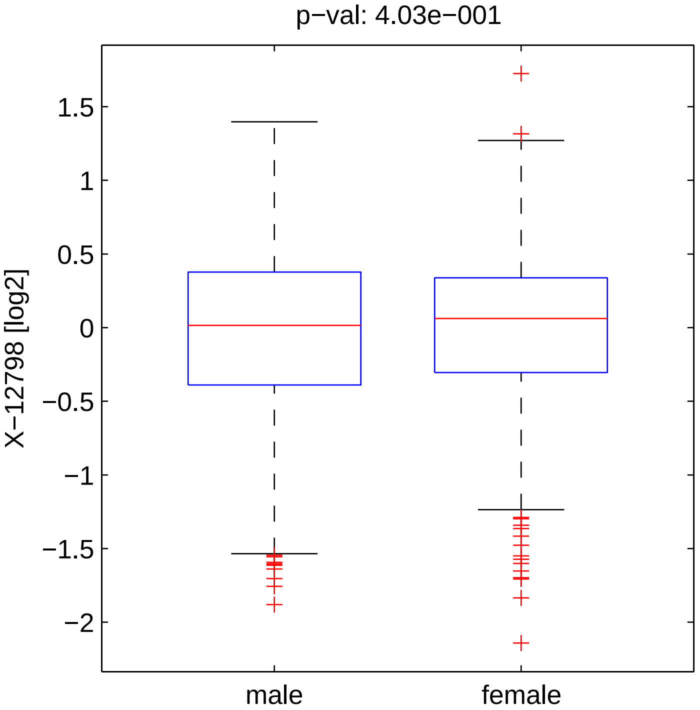

p−val: 4.03e−001
X−12798 [log2]
1.5
1
0.5
0
−0.5
−1
−1.5
−2
male
female

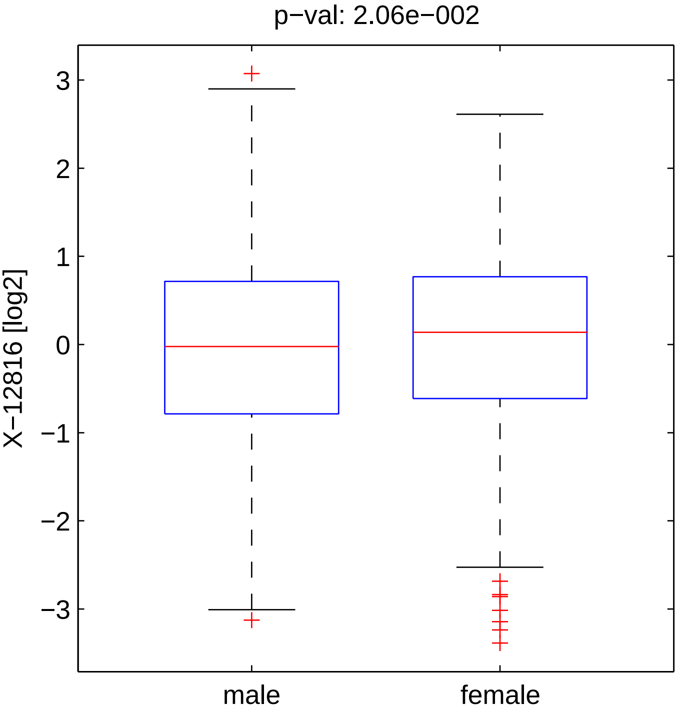

p−val: 2.06e−002
X−12816 [log2]
3
2
1
0
−1
−2
−3
male
female

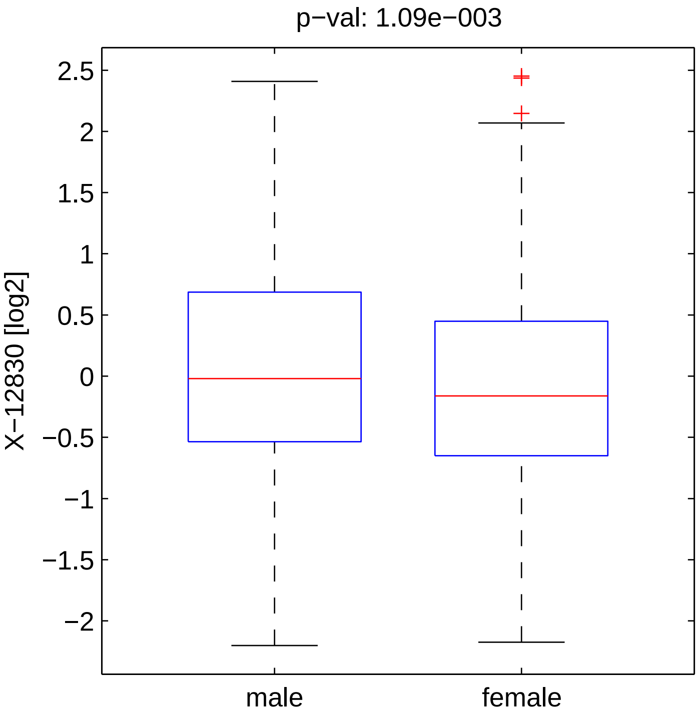
p−val: 1.09e−003
2.5
2
1.5
1
0.5
0
−0.5
−1
−1.5
−2
X−12830 [log2]
male
female

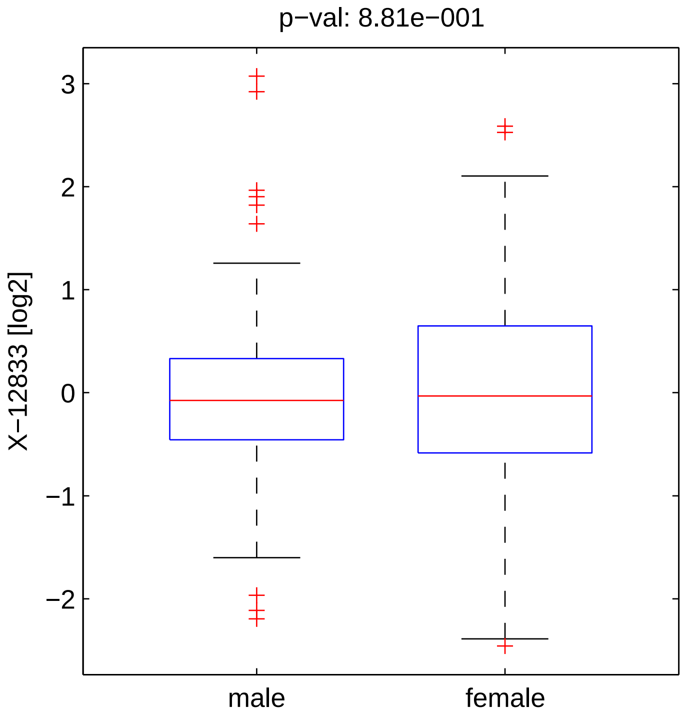
p−val: 8.81e−001
X−12833 [log2]
3
2
1
0
−1
−2
male
female

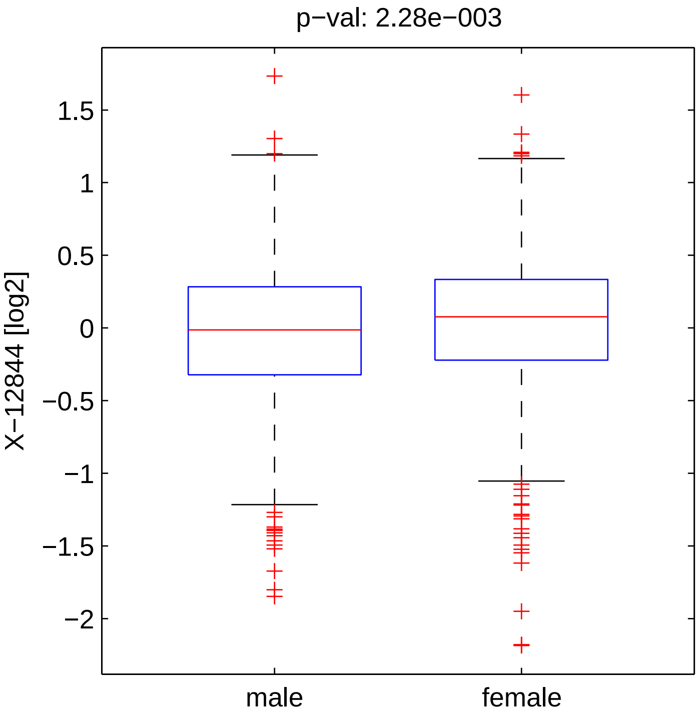
p−val: 2.28e−003
X−12844 [log2]
1.5
1
0.5
0
−0.5
−1
−1.5
−2
male
female

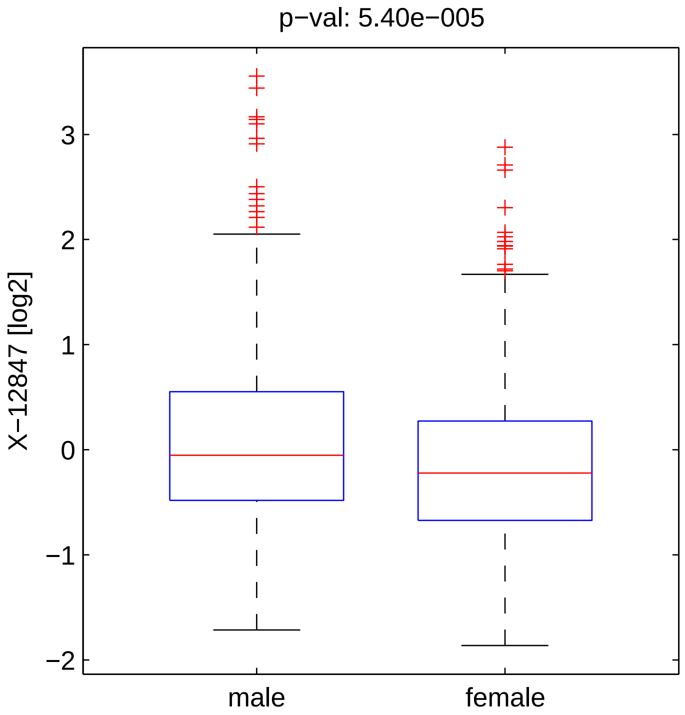
p−val: 5.40e−005
X−12847 [log2]
3
2
1
0
−1
−2
male
female

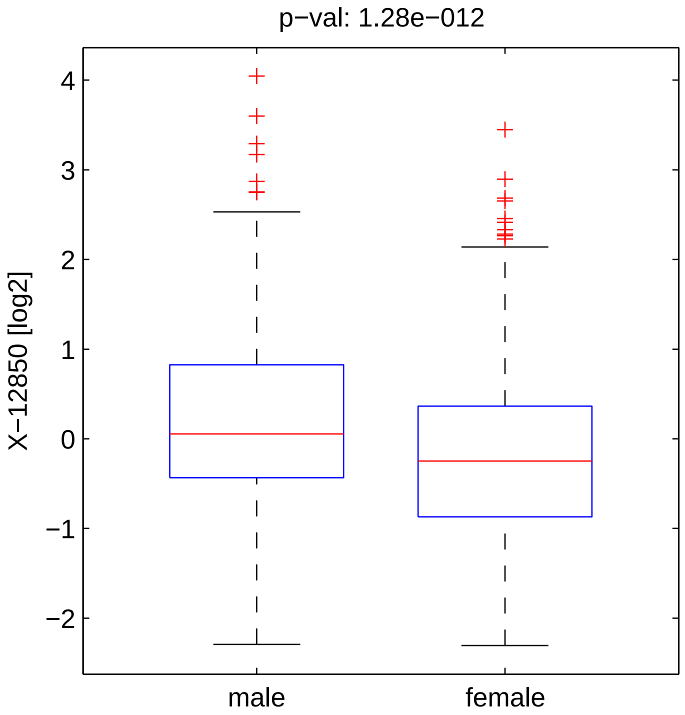
p−val: 1.28e−012
X−12850 [log2]
4
3
2
1
0
−1
−2
male
female

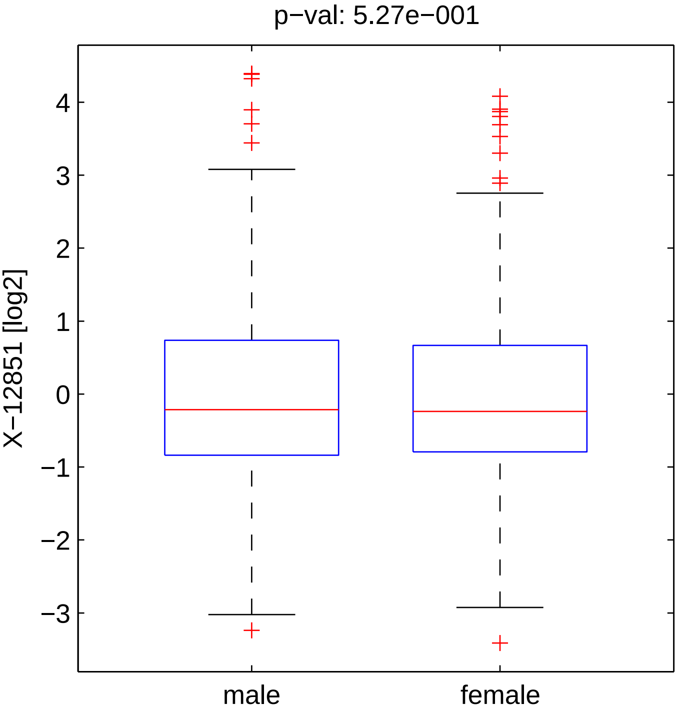
p−val: 5.27e−001
X−12851 [log2]
4
3
2
1
0
−1
−2
−3
male
female

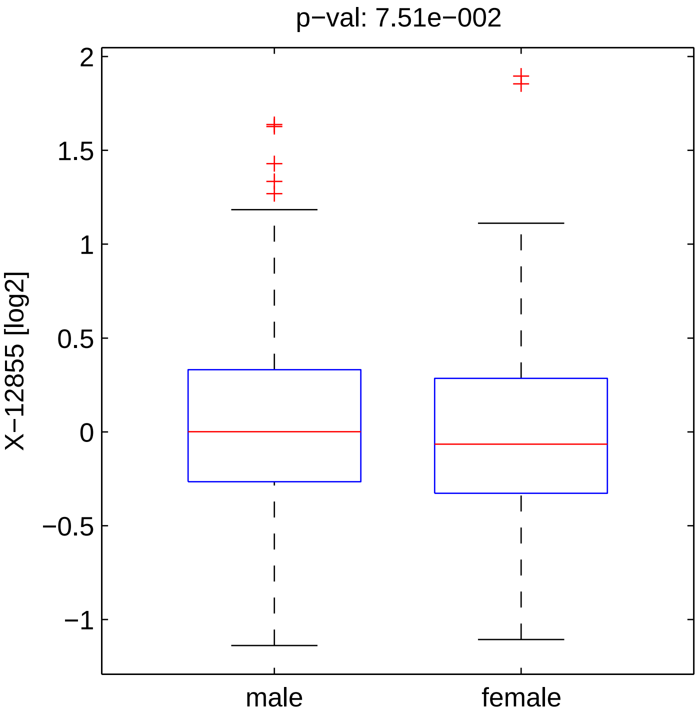
p−val: 7.51e−002
X−12855 [log2]
2
1.5
1
0.5
0
−0.5
−1
male
female

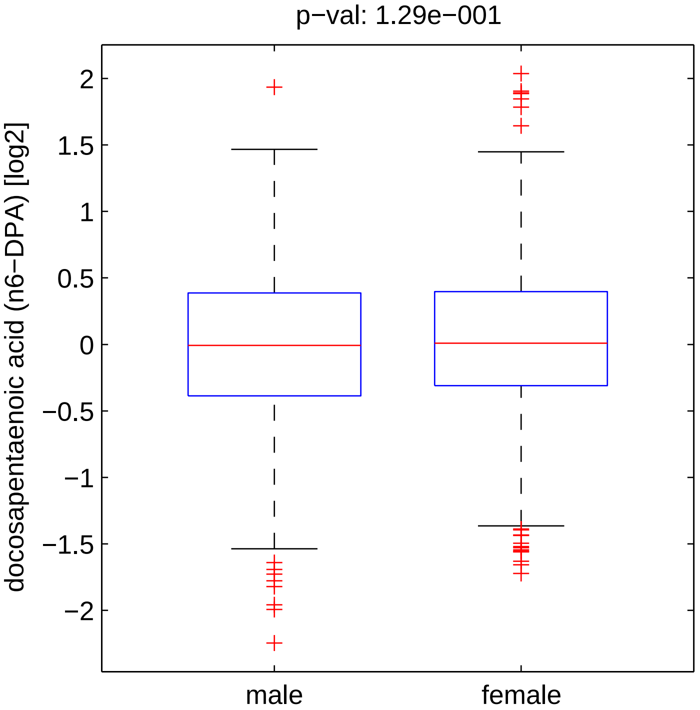

p−val: 1.29e−001
docosapentaenoic acid (n6−DPA) [log2]
2
1.5
1
0.5
0
−0.5
−1
−1.5
−2
male
female

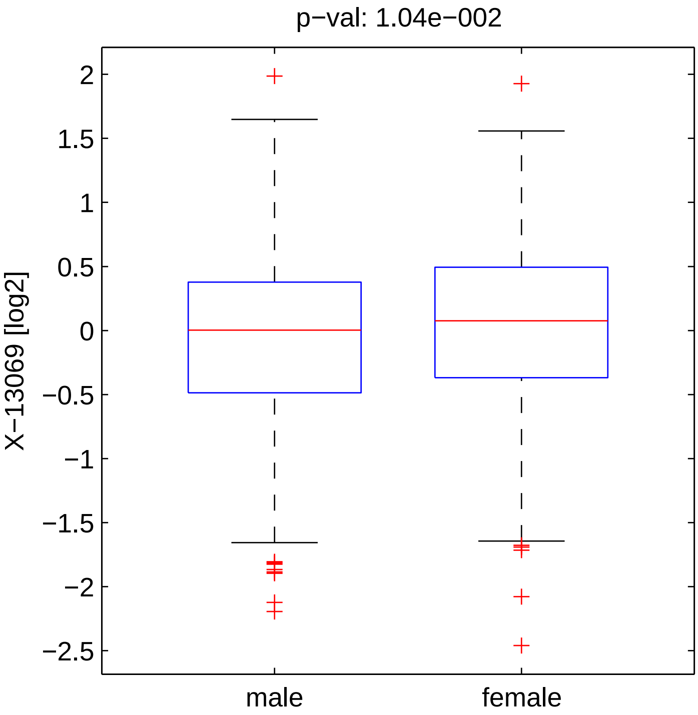

p−val: 1.04e−002
X−13069 [log2]
2
1.5
1
0.5
0
−0.5
−1
−1.5
−2
−2.5
male
female

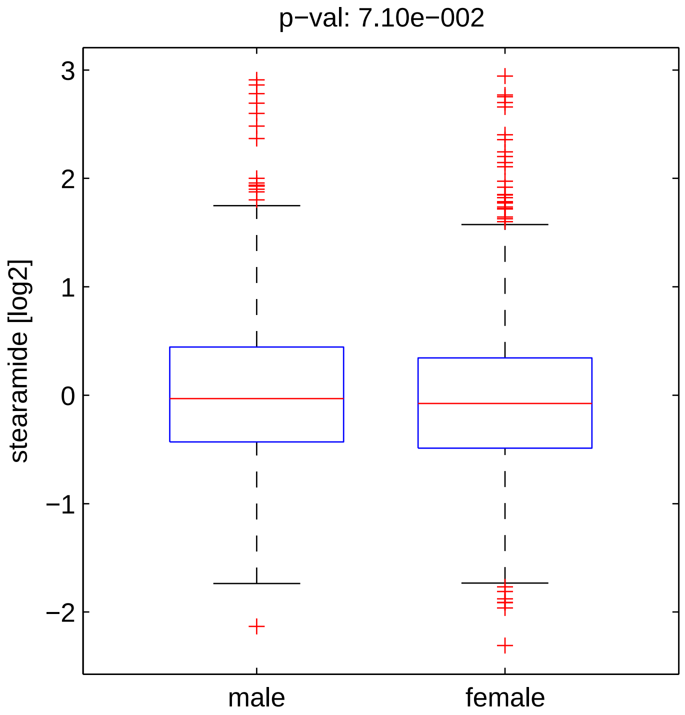
p−val: 7.10e−002
3
2
1
0
−1
−2
stearamide [log2]
male
female

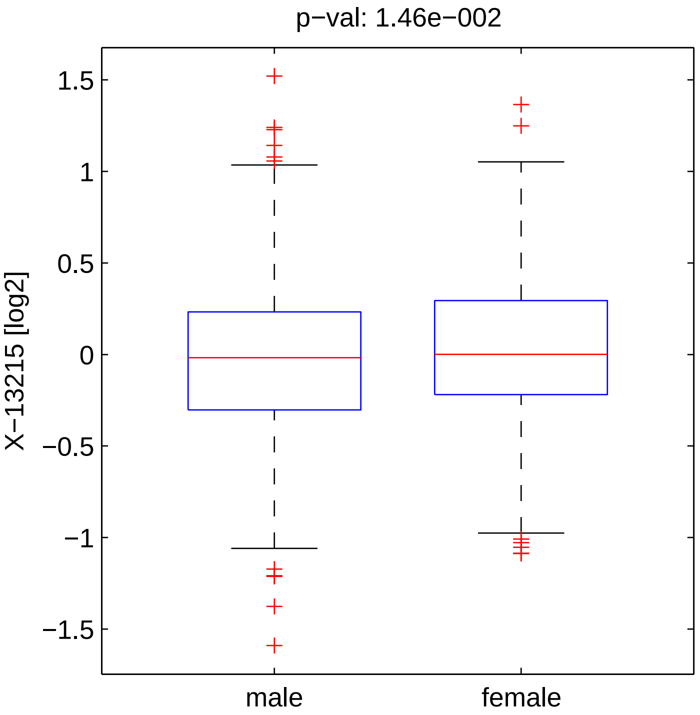
p−val: 1.46e−002
X−13215 [log2]
1.5
1
0.5
0
−0.5
−1
−1.5
male
female

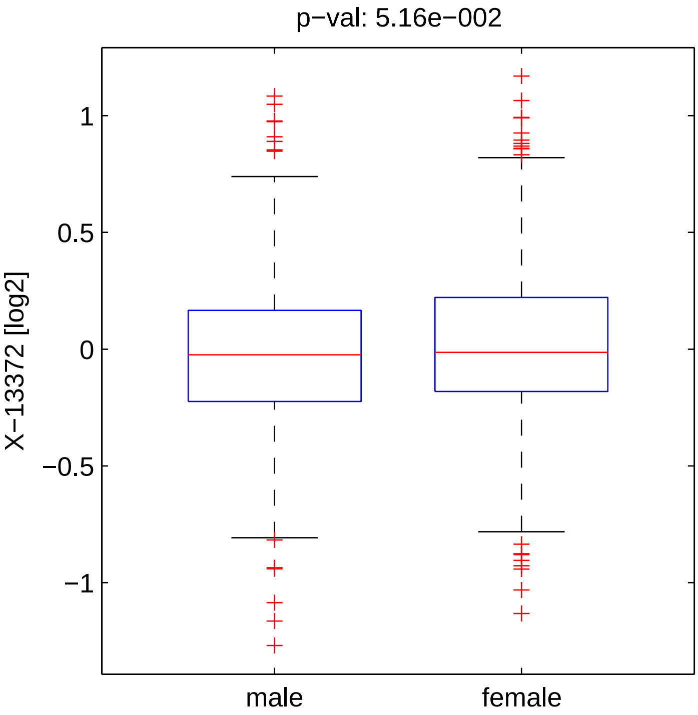
p−val: 5.16e−002
X−13372 [log2]
1
0.5
0
−0.5
−1
male
female

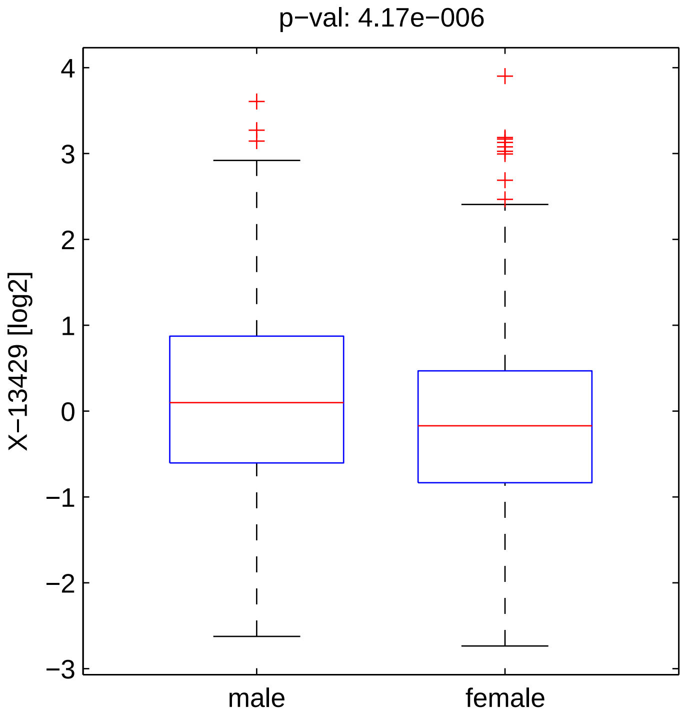
p−val: 4.17e−006
X−13429 [log2]
4
3
2
1
0
−1
−2
−3
male
female

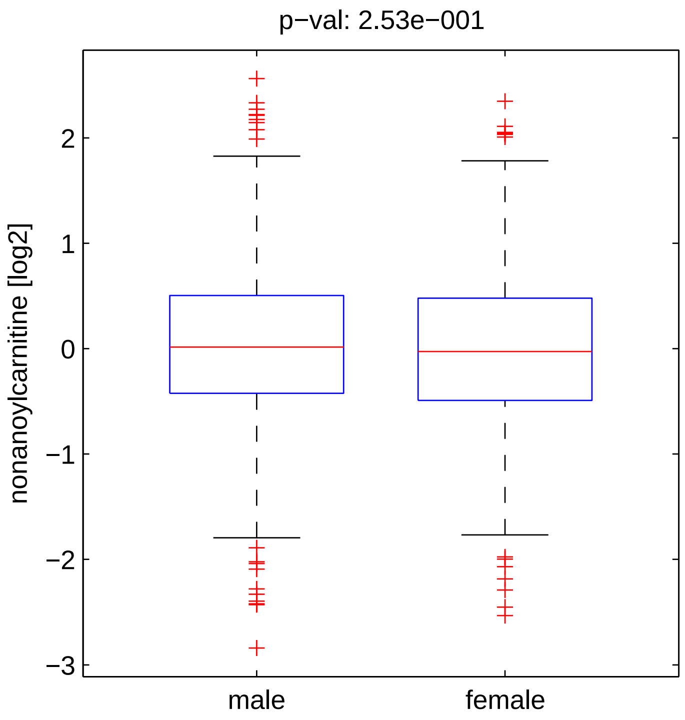
p−val: 2.53e−001
nonanoylcarnitine [log2]
2
1
0
−1
−2
−3
male
female

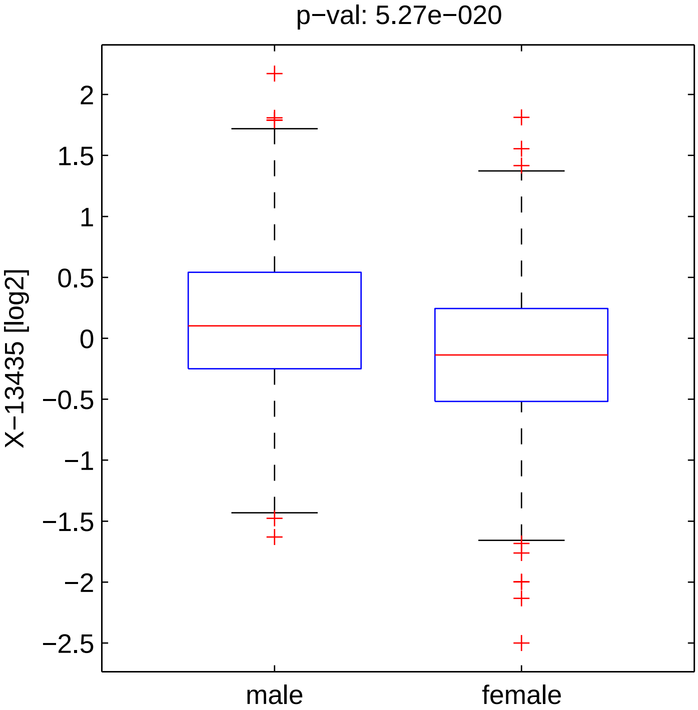
p−val: 5.27e−020
X−13435 [log2]
2
1.5
1
0.5
0
−0.5
−1
−1.5
−2
−2.5
male
female

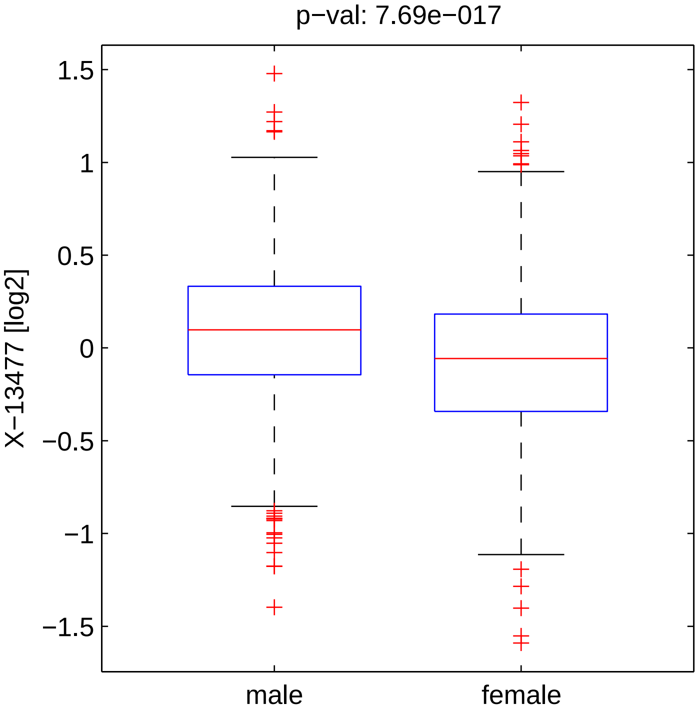
p−val: 7.69e−017
X−13477 [log2]
1.5
1
0.5
0
−0.5
−1
−1.5
male
female

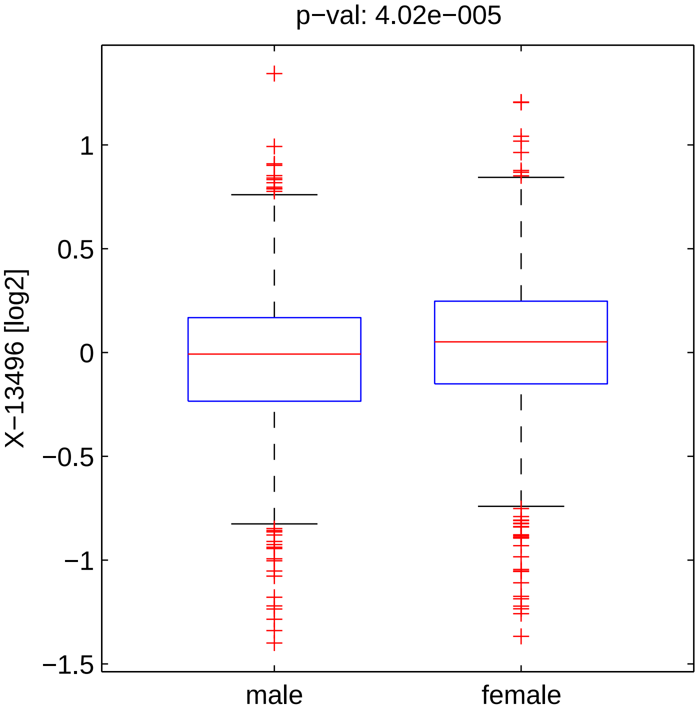
p−val: 4.02e−005
X−13496 [log2]
1
0.5
0
−0.5
−1
−1.5
male
female

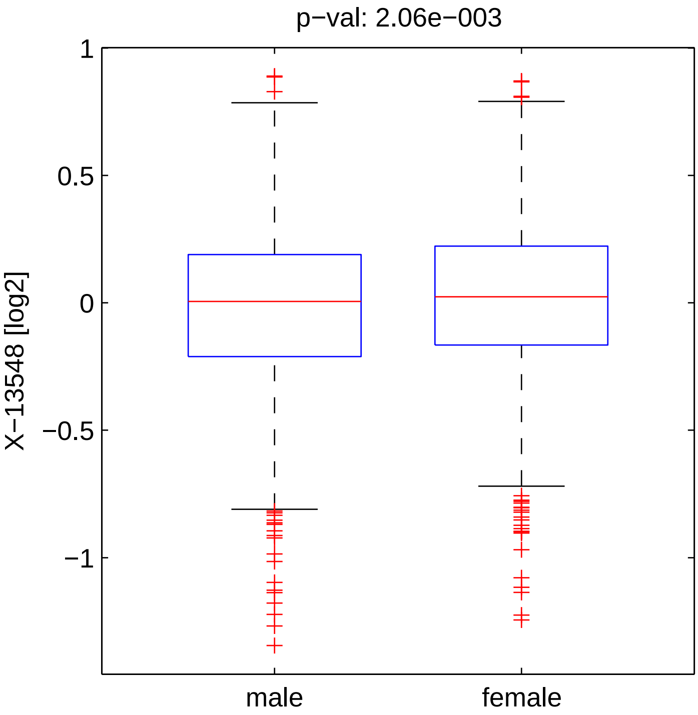
p−val: 2.06e−003
1
0.5
0
−0.5
−1
X−13548 [log2]
male
female

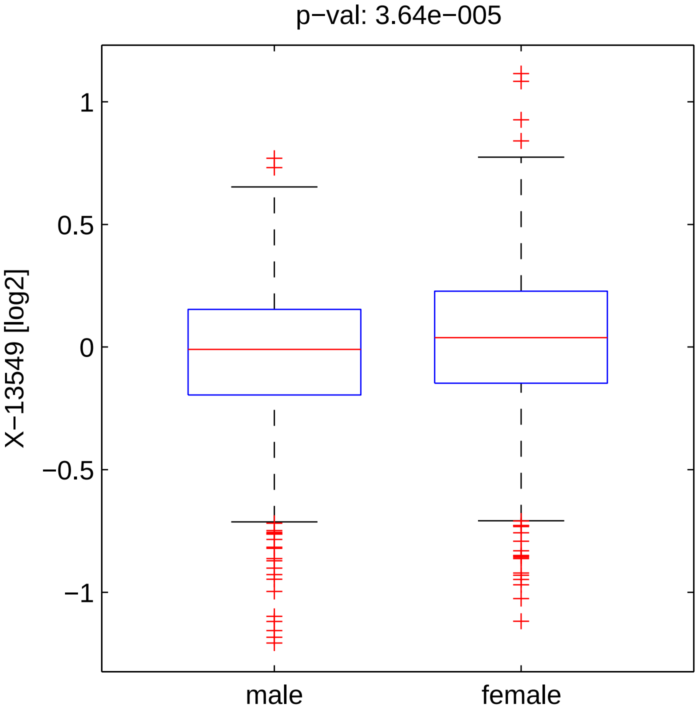

p−val: 3.64e−005
X−13549 [log2]
1
0.5
0
−0.5
−1
male
female

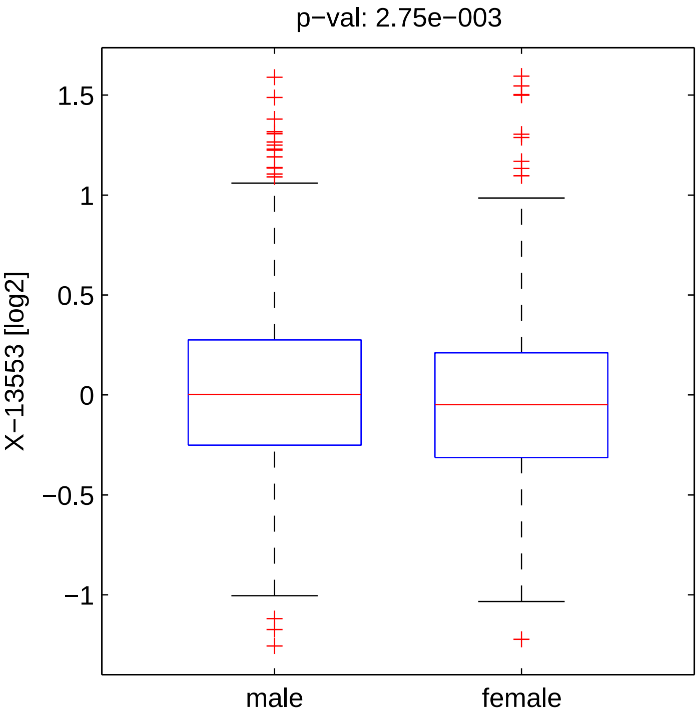
p−val: 2.75e−003
X−13553 [log2]
1.5
1
0.5
0
−0.5
−1
male
female

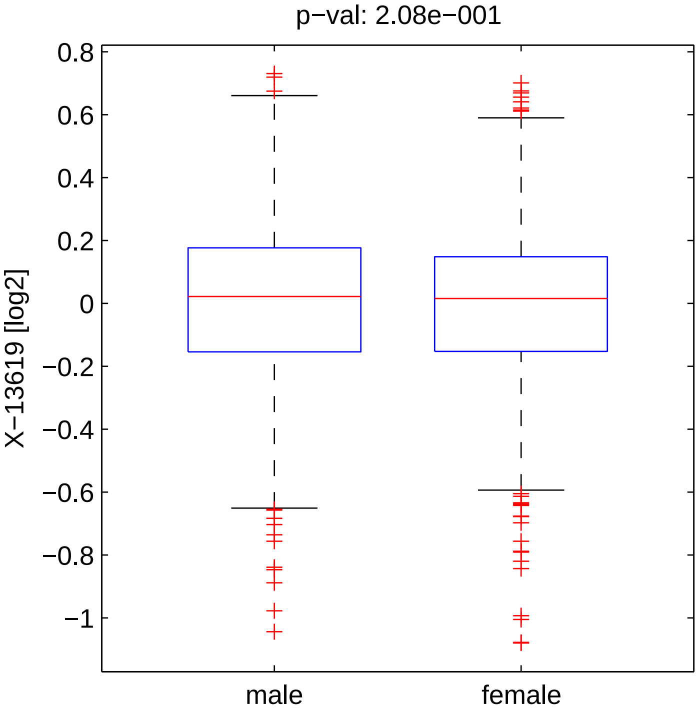
p−val: 2.08e−001
0.8
0.6
0.4
0.2
0
−0.2
−0.4
−0.6
−0.8
−1
X−13619 [log2]
male
female

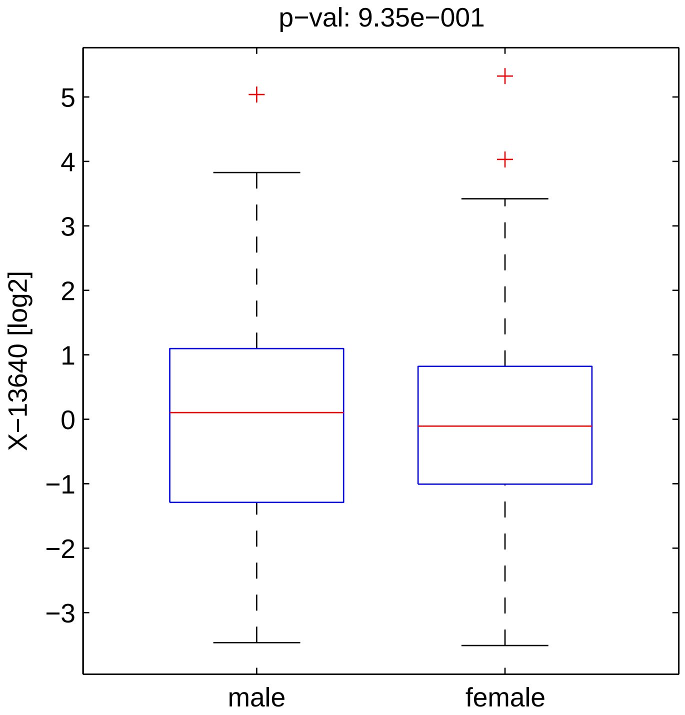

p−val: 9.35e−001
X−13640 [log2]
5
4
3
2
1
0
−1
−2
−3
male
female

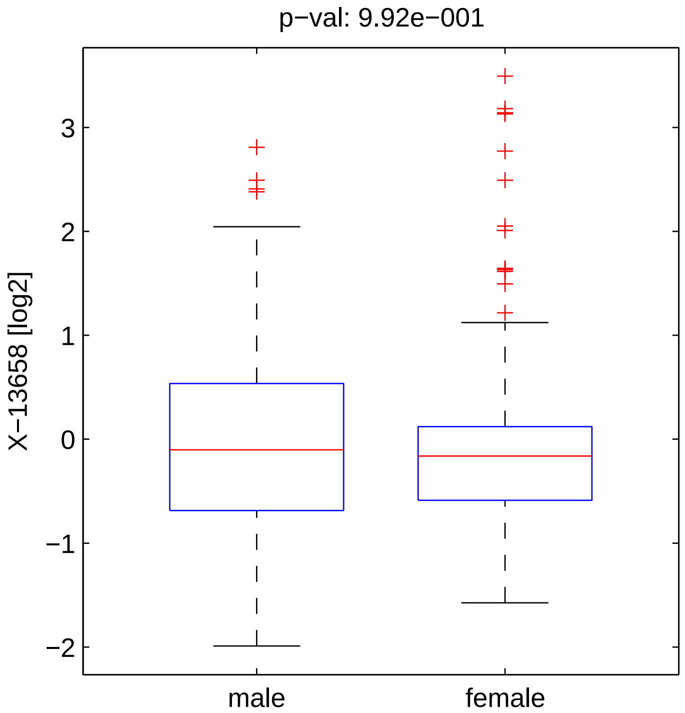

p−val: 9.92e−001
X−13658 [log2]
3
2
1
0
−1
−2
male
female

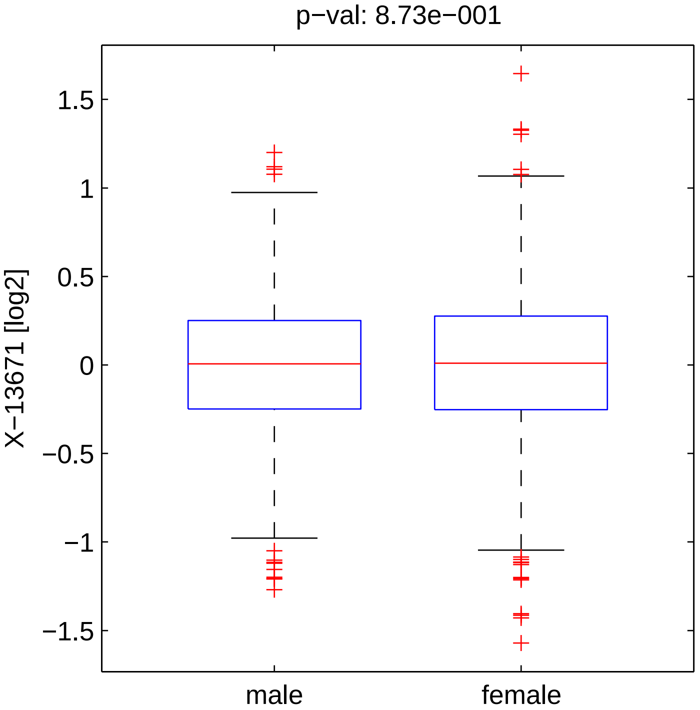
p−val: 8.73e−001
X−13671 [log2]
1.5
1
0.5
0
−0.5
−1
−1.5
male
female

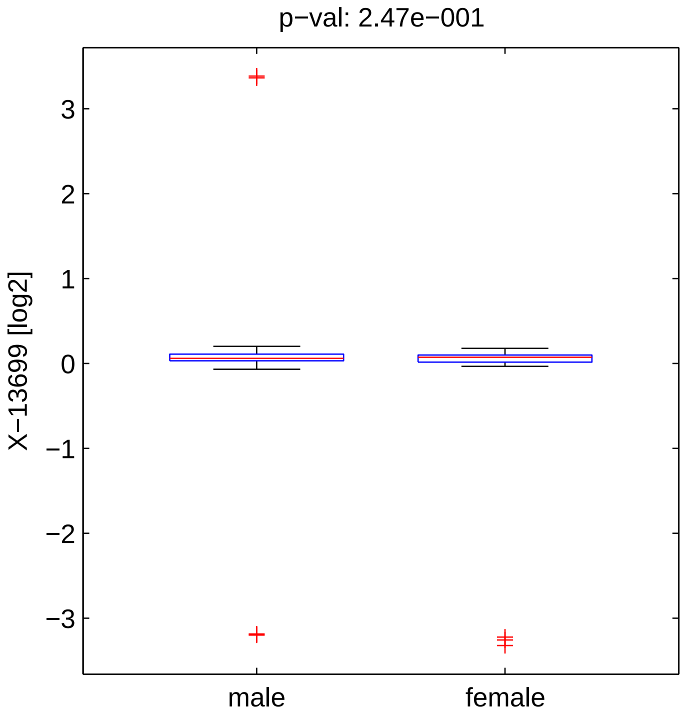
p−val: 2.47e−001
X−13699 [log2]
3
2
1
0
−1
−2
−3
male
female

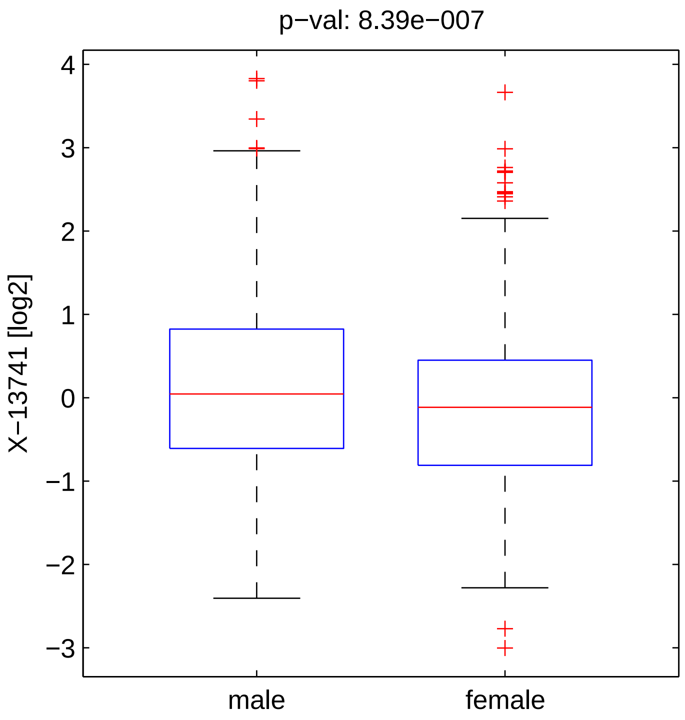

p−val: 8.39e−007
X−13741 [log2]
4
3
2
1
0
−1
−2
−3
male
female

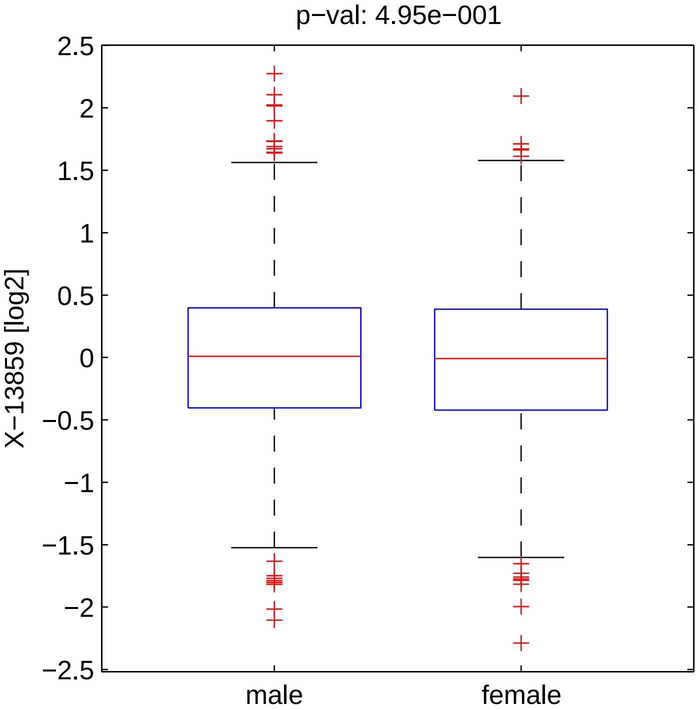
p−val: 4.95e−001
2.5
2
1.5
1
0.5
0
−0.5
−1
−1.5
−2
−2.5
X−13859 [log2]
male
female

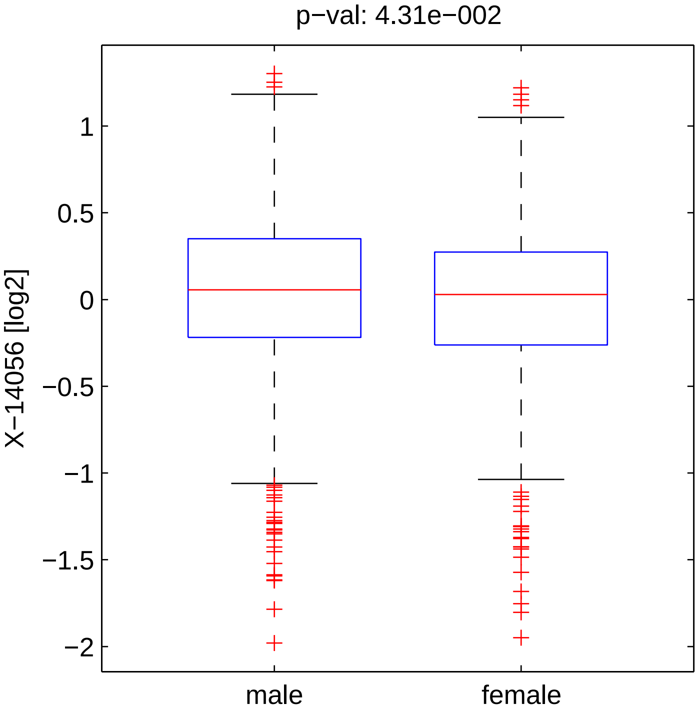

p−val: 4.31e−002
X−14056 [log2]
1
0.5
0
−0.5
−1
−1.5
−2
male
female

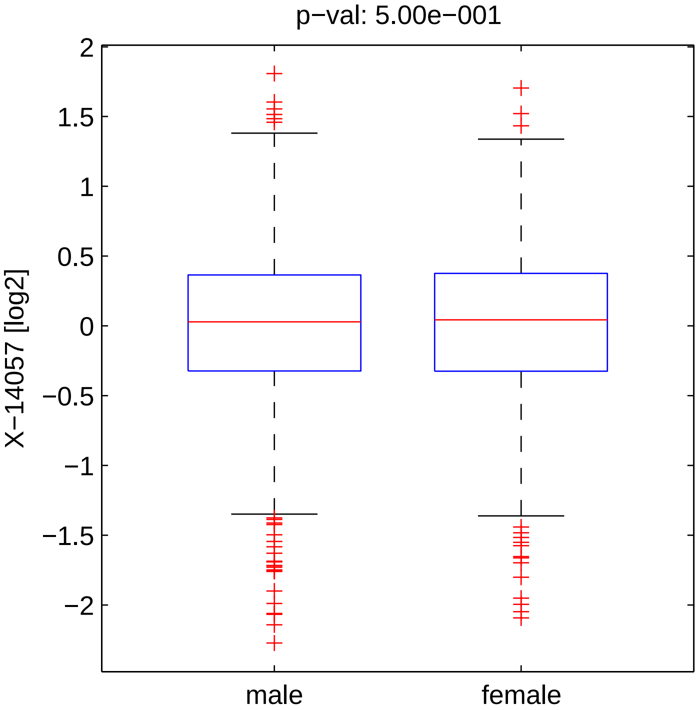
p−val: 5.00e−001
X−14057 [log2]
2
1.5
1
0.5
0
−0.5
−1
−1.5
−2
male
female

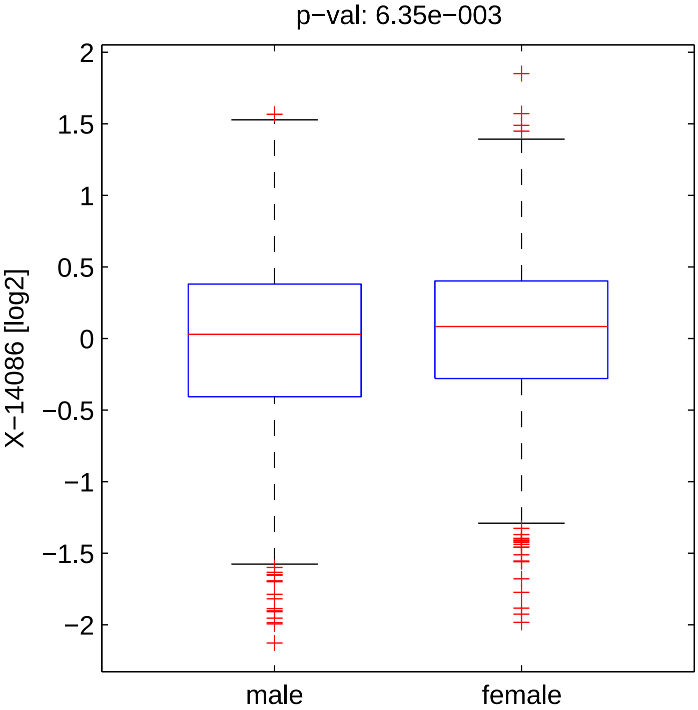

p−val: 6.35e−003
X−14086 [log2]
2
1.5
1
0.5
0
−0.5
−1
−1.5
−2
male
female

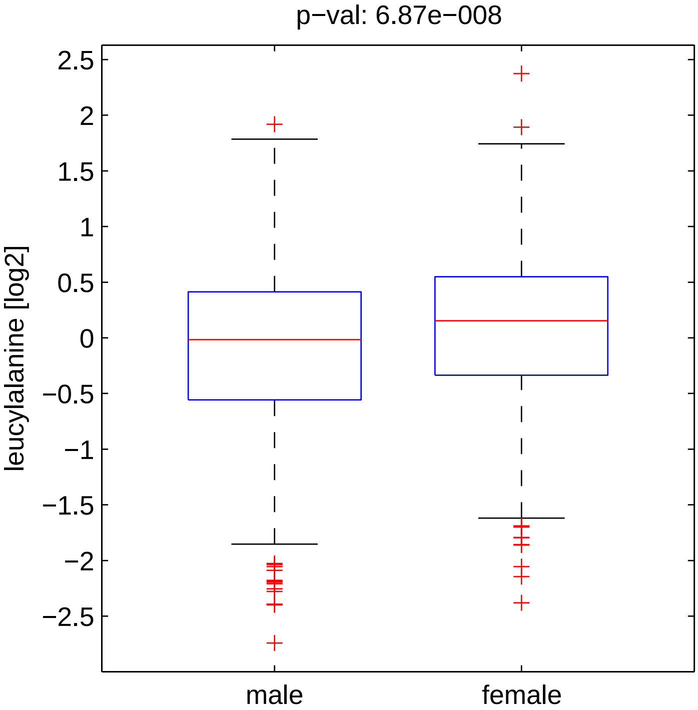
p−val: 6.87e−008
2.5
2
1.5
1
0.5
0
−0.5
−1
−1.5
−2
−2.5
leucylalanine [log2]
male
female

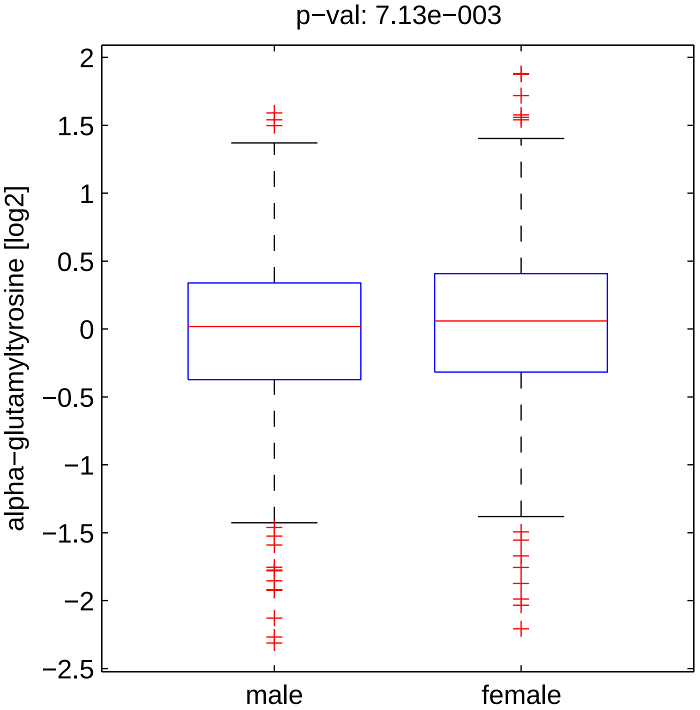

p−val: 7.13e−003
alpha−glutamyltyrosine [log2]
2
1.5
1
0.5
0
−0.5
−1
−1.5
−2
−2.5
male
female

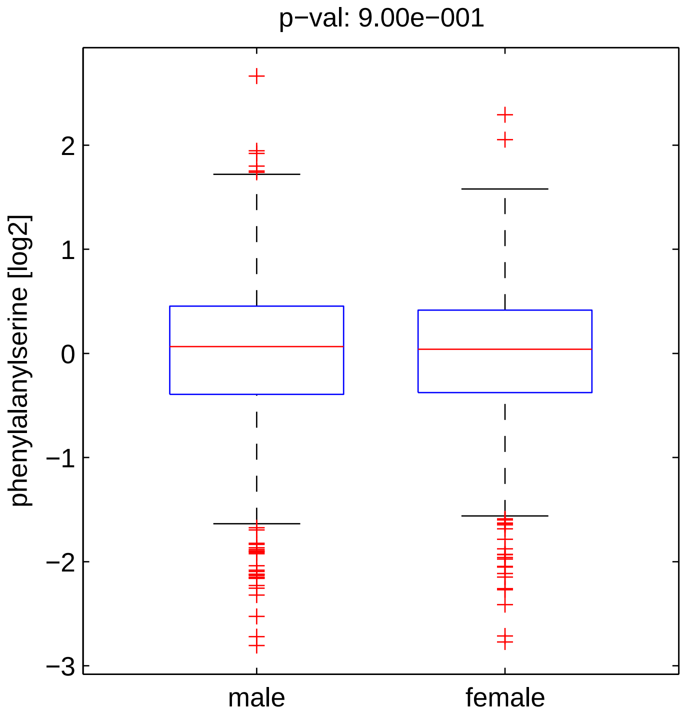
p−val: 9.00e−001
phenylalanylserine [log2]
2
1
0
−1
−2
−3
male
female

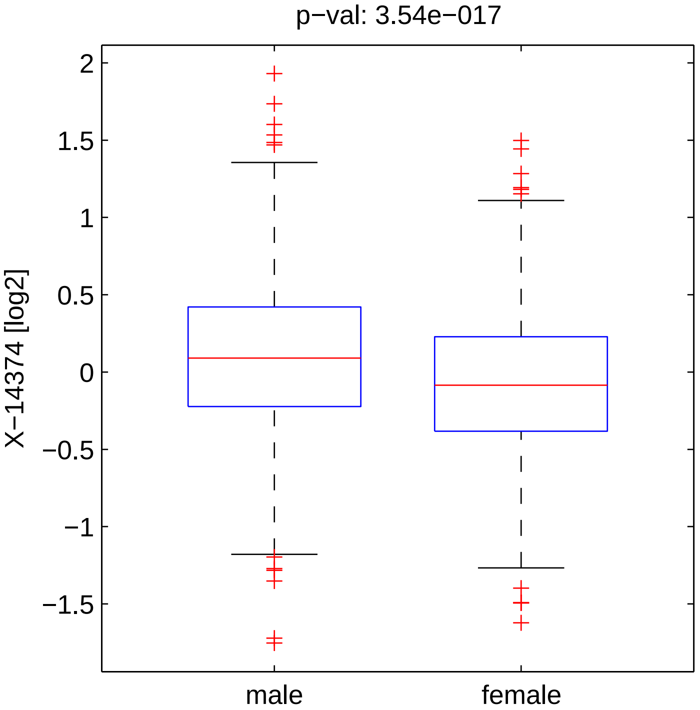

p−val: 3.54e−017
X−14374 [log2]
2
1.5
1
0.5
0
−0.5
−1
−1.5
male
female

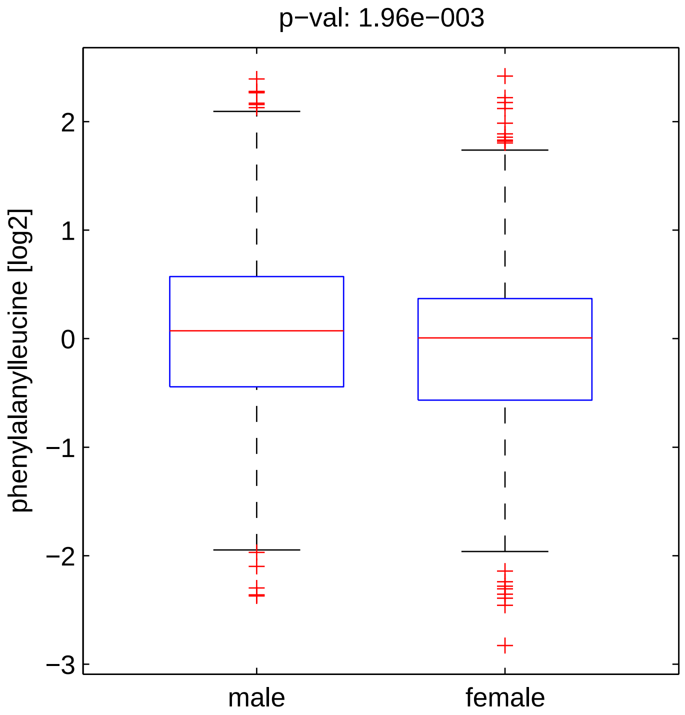
p−val: 1.96e−003
phenylalanylleucine [log2]
2
1
0
−1
−2
−3
male
female

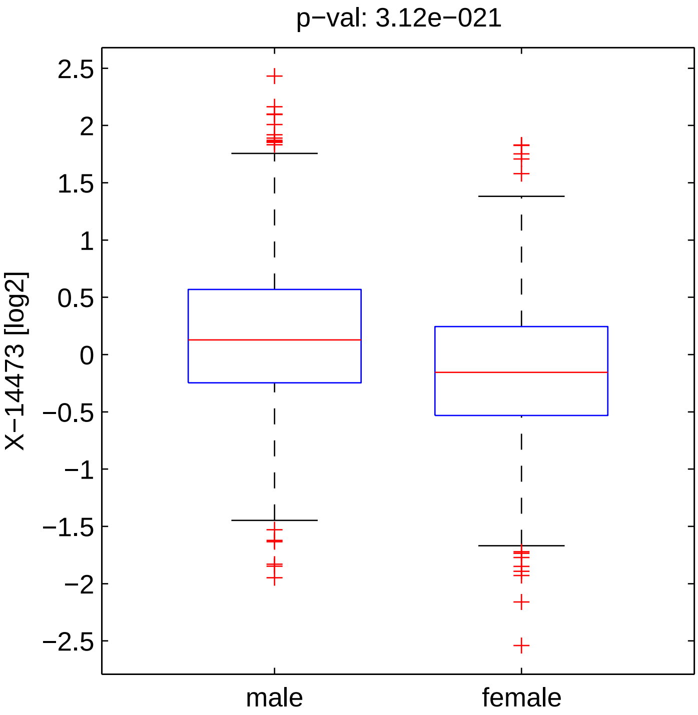

p−val: 3.12e−021
X−14473 [log2]
2.5
2
1.5
1
0.5
0
−0.5
−1
−1.5
−2
−2.5
male
female

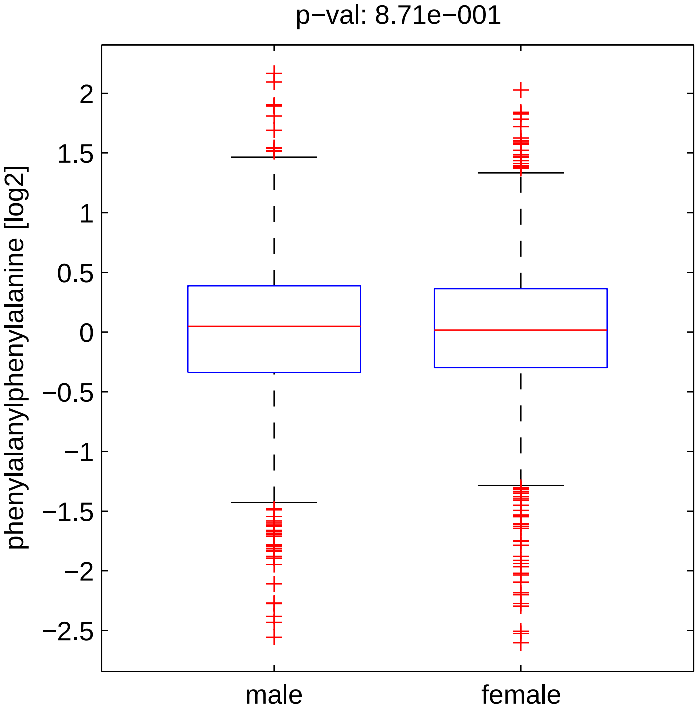

p−val: 8.71e−001
phenylalanylphenylalanine [log2]
2
1.5
1
0.5
0
−0.5
−1
−1.5
−2
−2.5
male
female

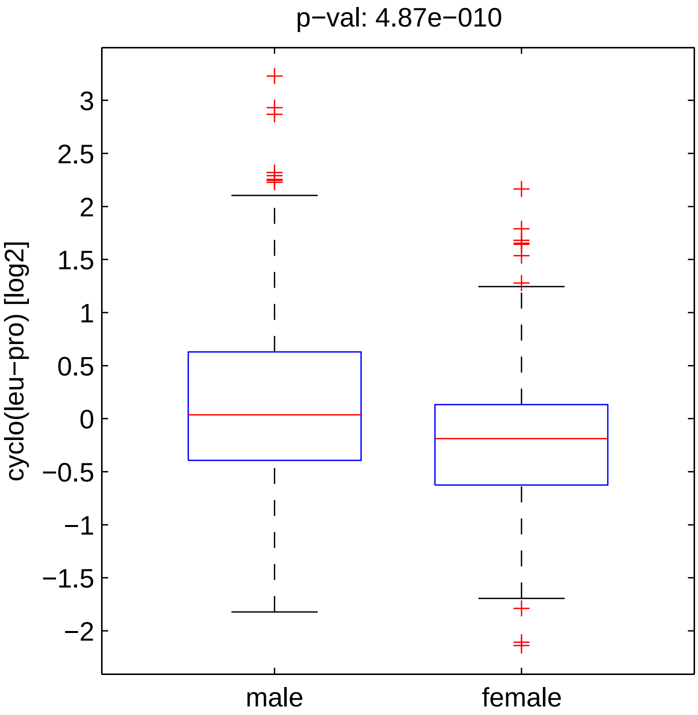
p−val: 4.87e−010
cyclo(leu−pro) [log2]
3
2.5
2
1.5
1
0.5
0
−0.5
−1
−1.5
−2
male
female

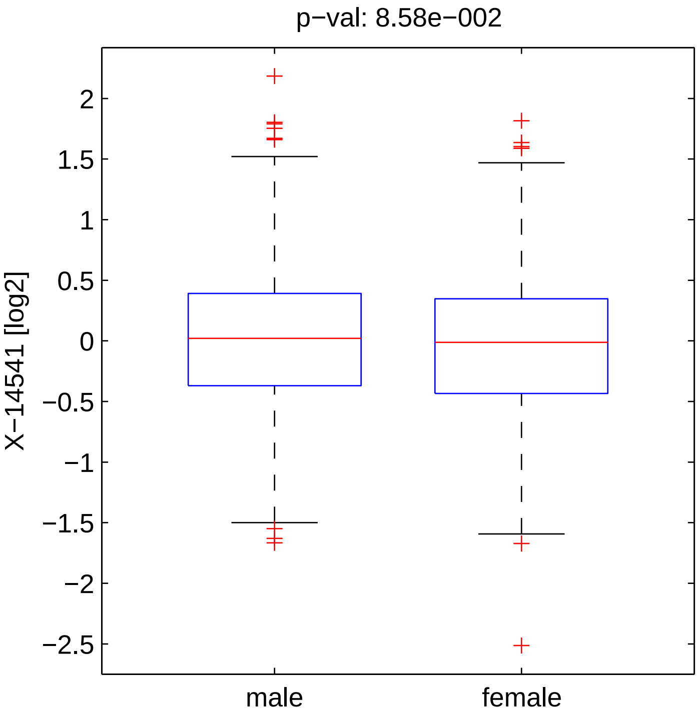
p−val: 8.58e−002
X−14541 [log2]
2
1.5
1
0.5
0
−0.5
−1
−1.5
−2
−2.5
male
female

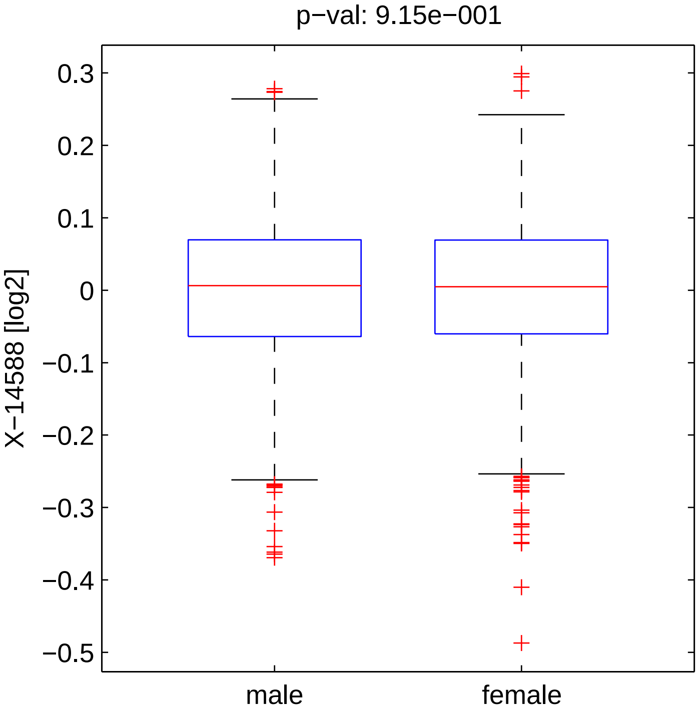

p−val: 9.15e−001
X−14588 [log2]
0.3
0.2
0.1
0
−0.1
−0.2
−0.3
−0.4
−0.5
male
female

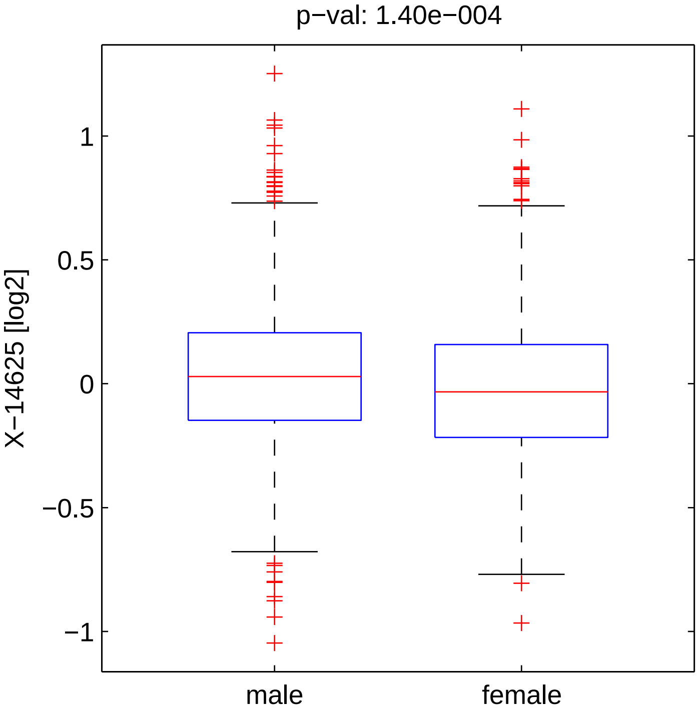
p−val: 1.40e−004
X−14625 [log2]
1
0.5
0
−0.5
−1
male
female

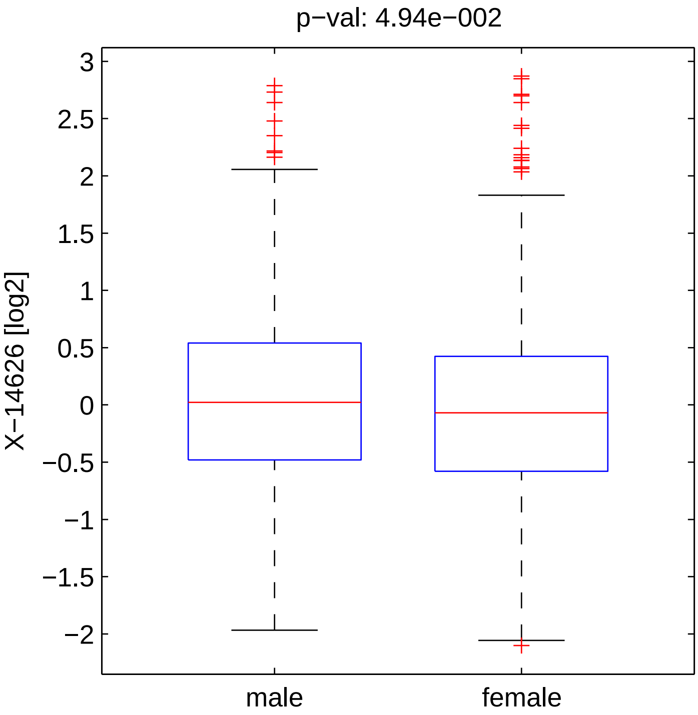

p−val: 4.94e−002
X−14626 [log2]
3
2.5
2
1.5
1
0.5
0
−0.5
−1
−1.5
−2
male
female

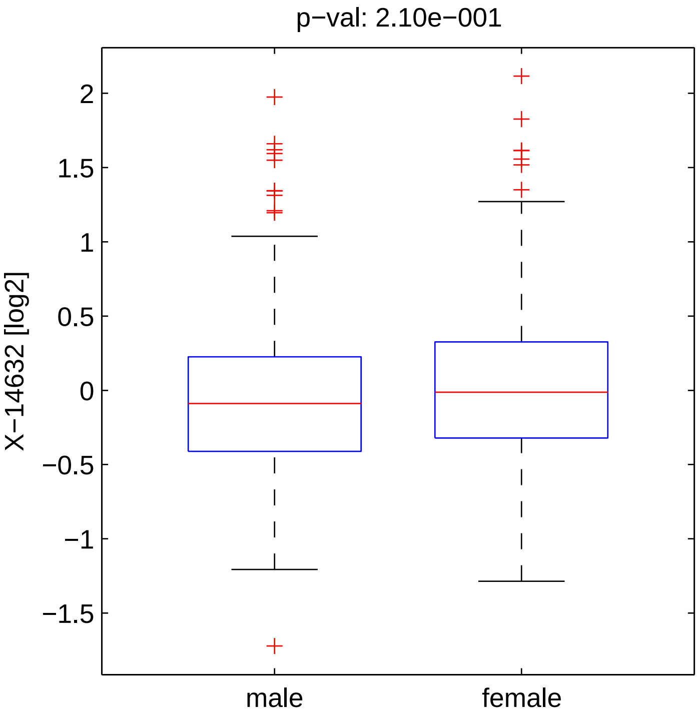
p−val: 2.10e−001
X−14632 [log2]
2
1.5
1
0.5
0
−0.5
−1
−1.5
male
female

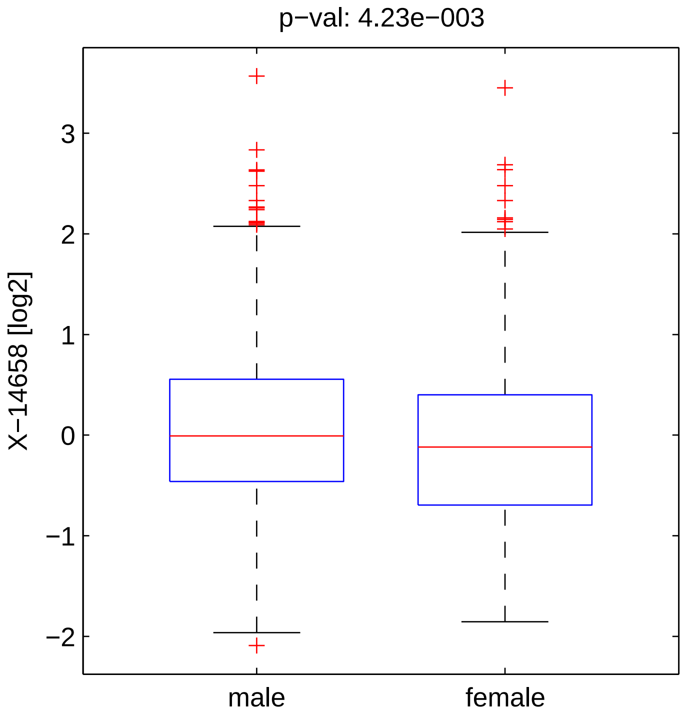
p−val: 4.23e−003
X−14658 [log2]
3
2
1
0
−1
−2
male
female

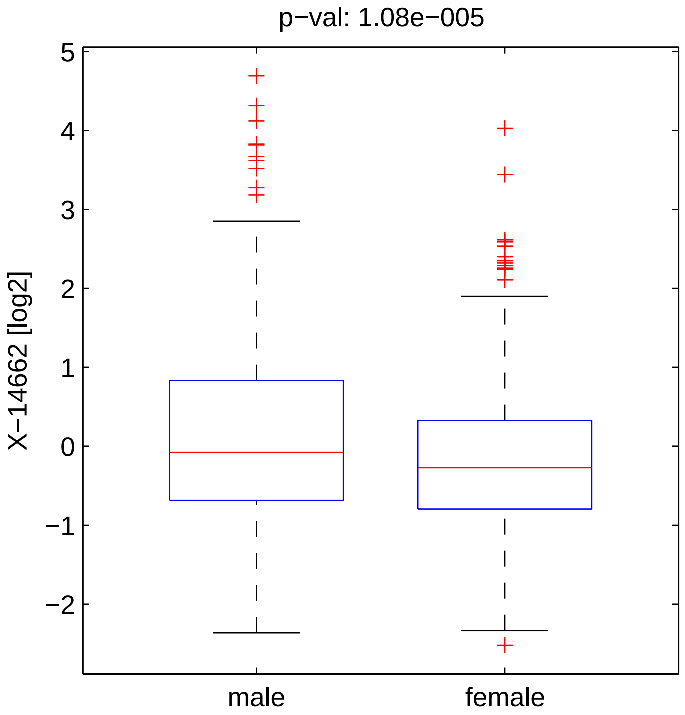
p−val: 1.08e−005
X−14662 [log2]
5
4
3
2
1
0
−1
−2
male
female

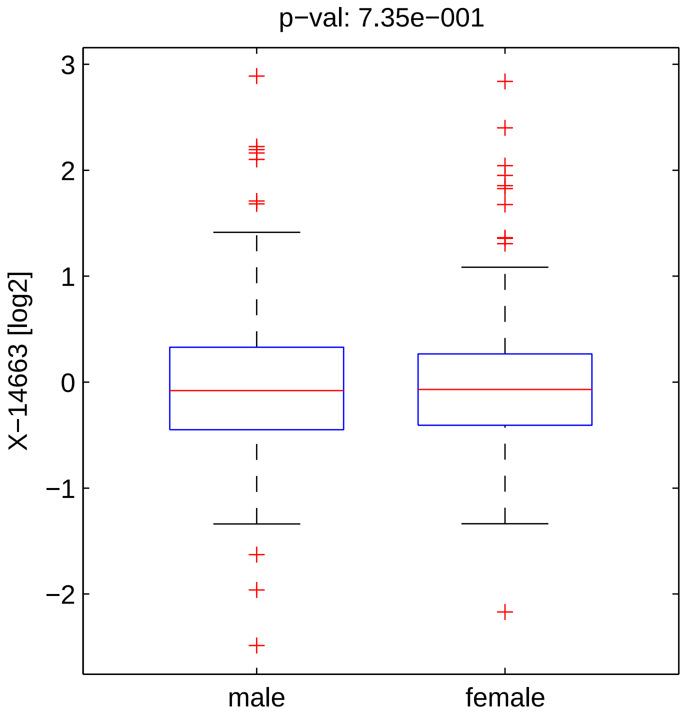
p−val: 7.35e−001
X−14663 [log2]
3
2
1
0
−1
−2
male
female

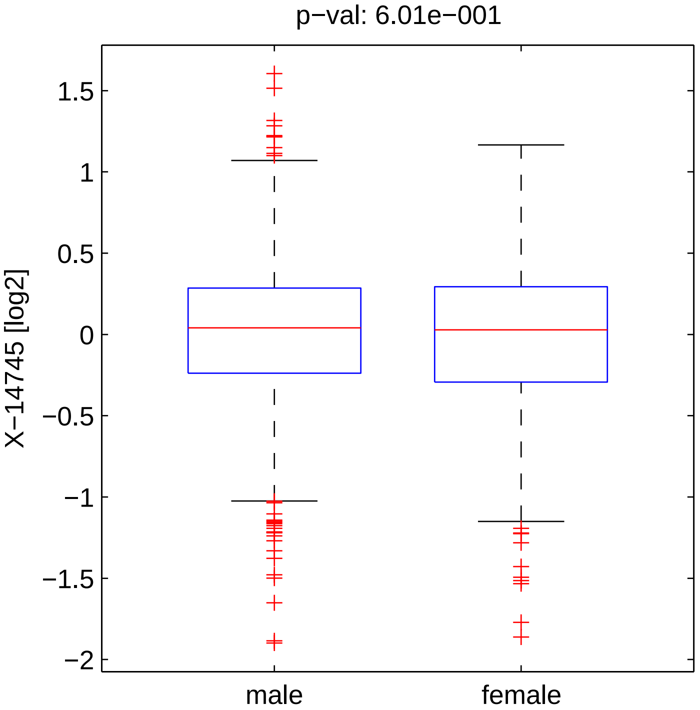
p−val: 6.01e−001
X−14745 [log2]
1.5
1
0.5
0
−0.5
−1
−1.5
−2
male
female

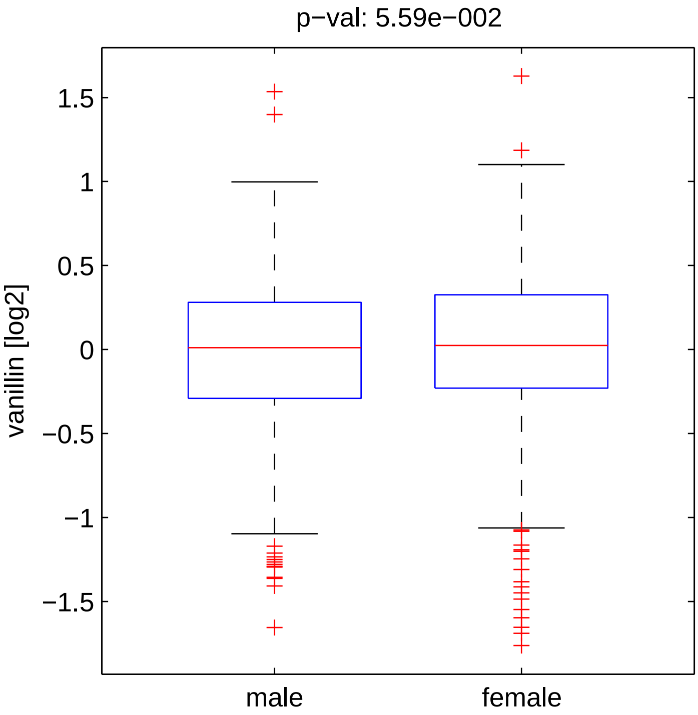
p−val: 5.59e−002
vanillin [log2]
1.5
1
0.5
0
−0.5
−1
−1.5
male
female

KORA pathway boxplots

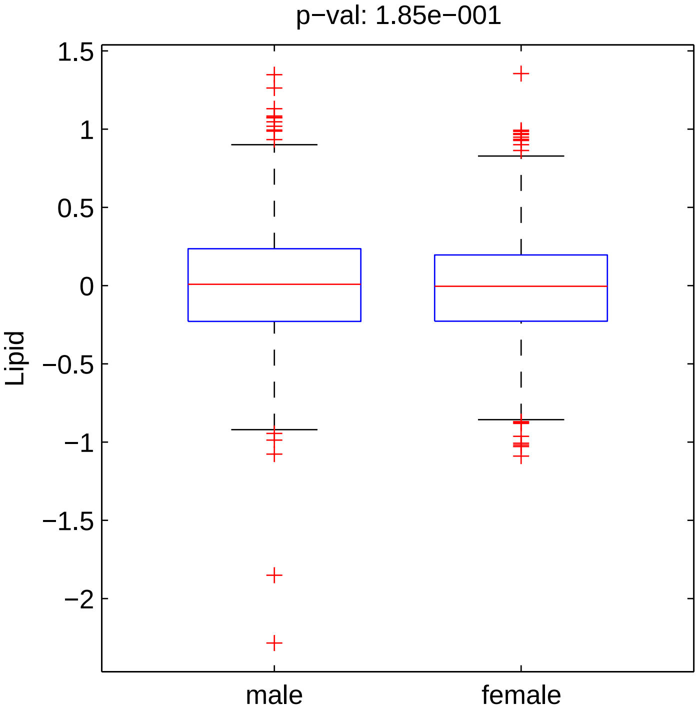
p−val: 1.85e−001
1.5
1
0.5
0
−0.5
−1
−1.5
−2
Lipid
male
female

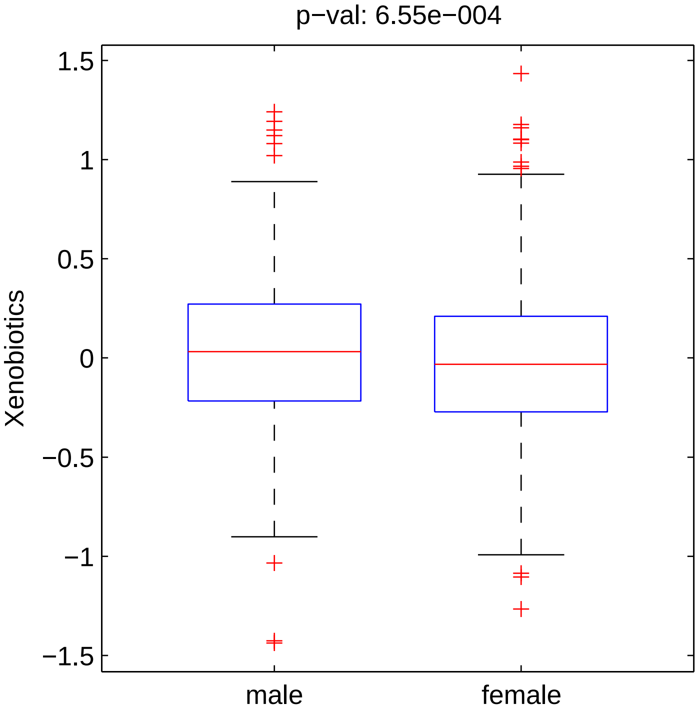
p−val: 6.55e−004
1.5
1
0.5
0
−0.5
−1
−1.5
Xenobiotics
male
female

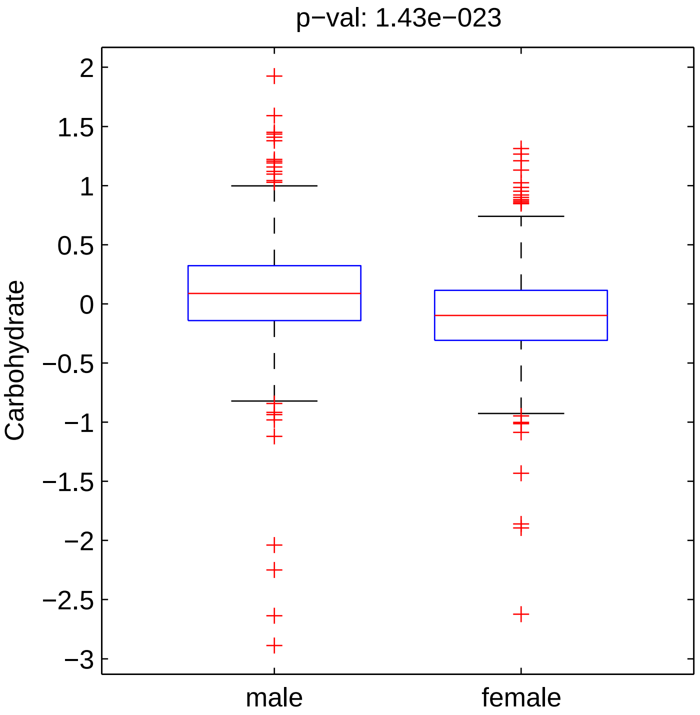

p−val: 1.43e−023
Carbohydrate
2
1.5
1
0.5
0
−0.5
−1
−1.5
−2
−2.5
−3
male
female

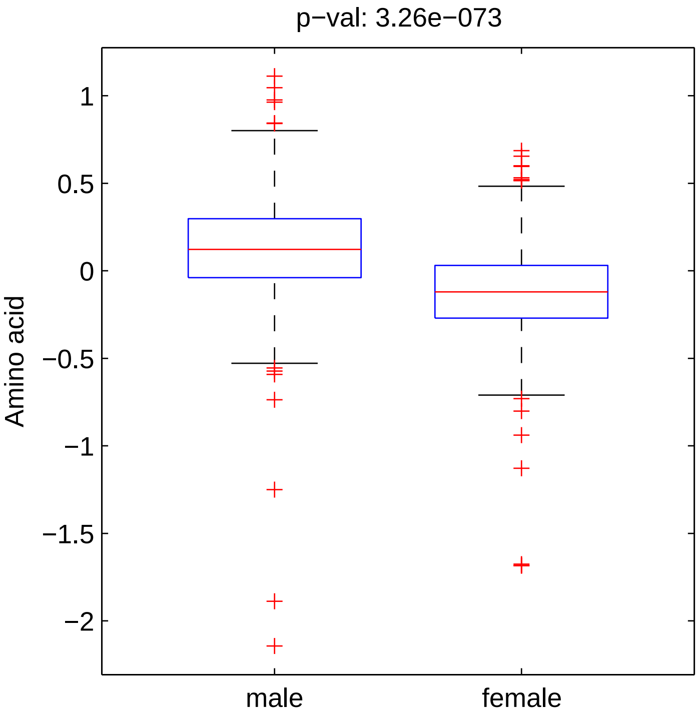

p−val: 3.26e−073
Amino acid
1
0.5
0
−0.5
−1
−1.5
−2
male
female

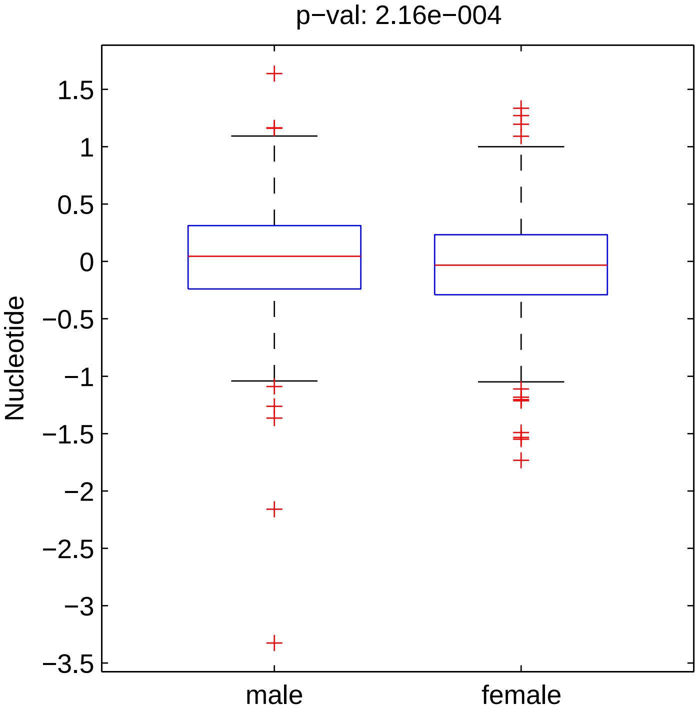
p−val: 2.16e−004
1.5
1
0.5
0
−0.5
−1
−1.5
−2
−2.5
−3
−3.5
Nucleotide
male
female

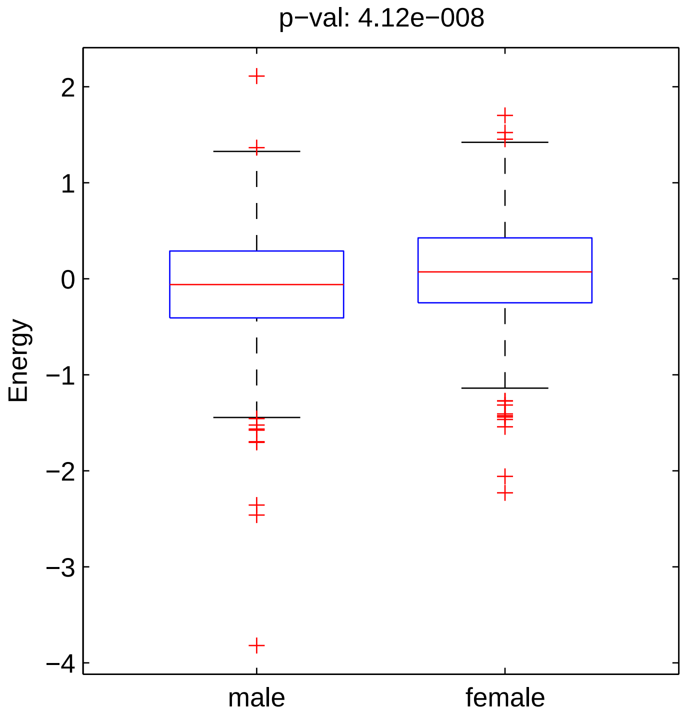
p−val: 4.12e−008
Energy
2
1
0
−1
−2
−3
−4
male
female

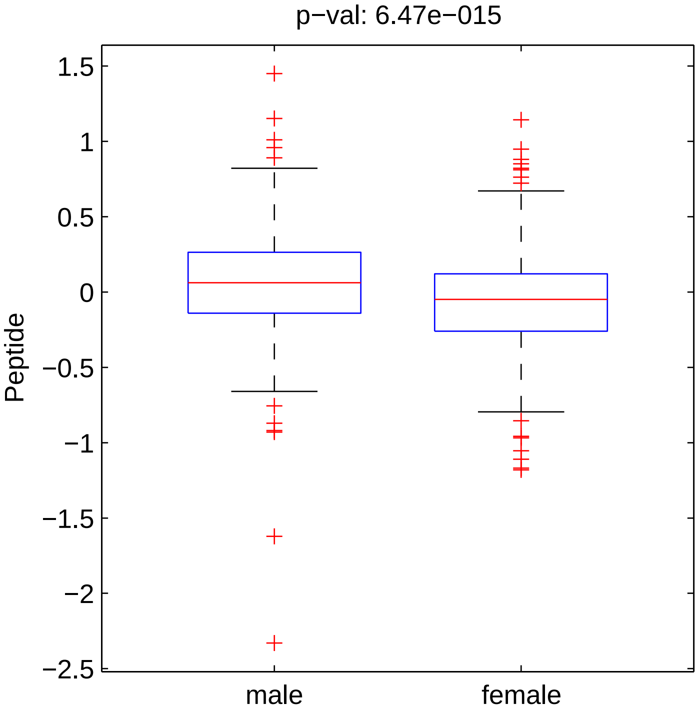
p−val: 6.47e−015
1.5
1
0.5
0
−0.5
−1
−1.5
−2
−2.5
Peptide
male
female

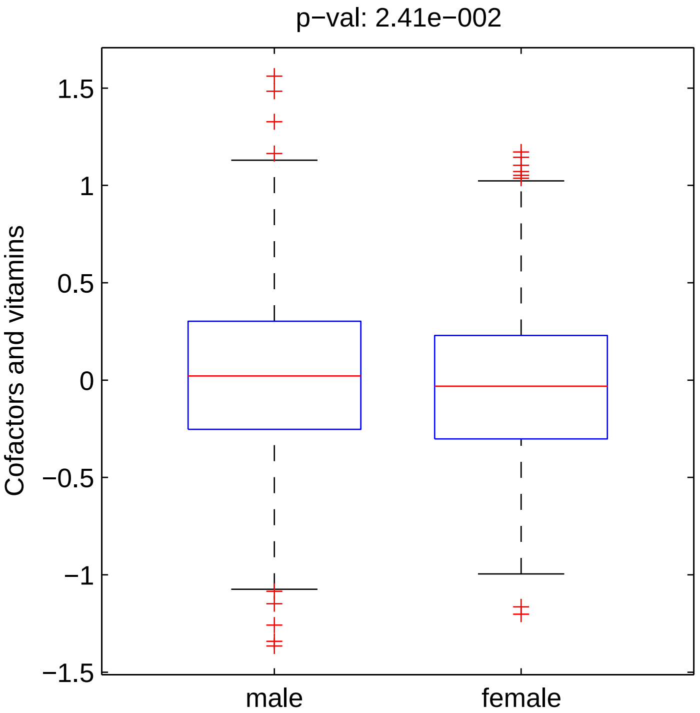
p−val: 2.41e−002
Cofactors and vitamins
1.5
1
0.5
0
−0.5
−1
−1.5
male
female

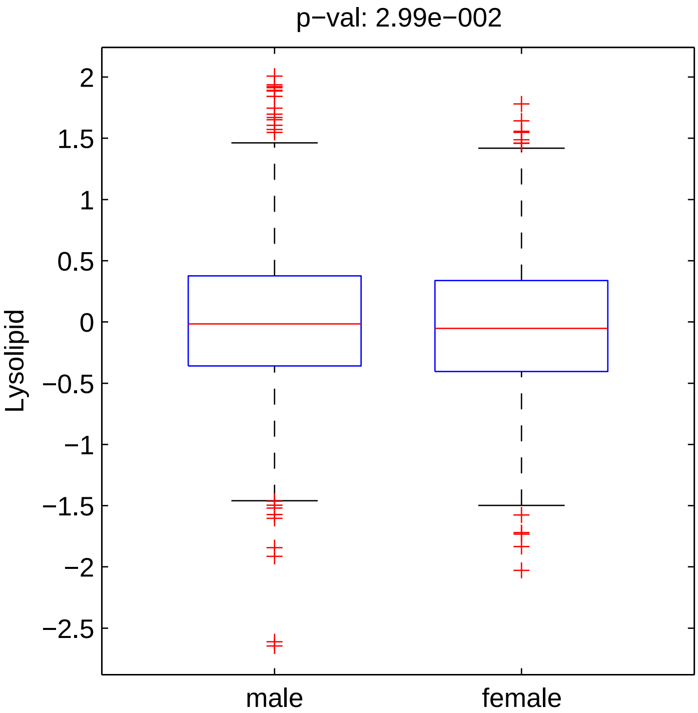

p−val: 2.99e−002
Lysolipid
2
1.5
1
0.5
0
−0.5
−1
−1.5
−2
−2.5
male
female

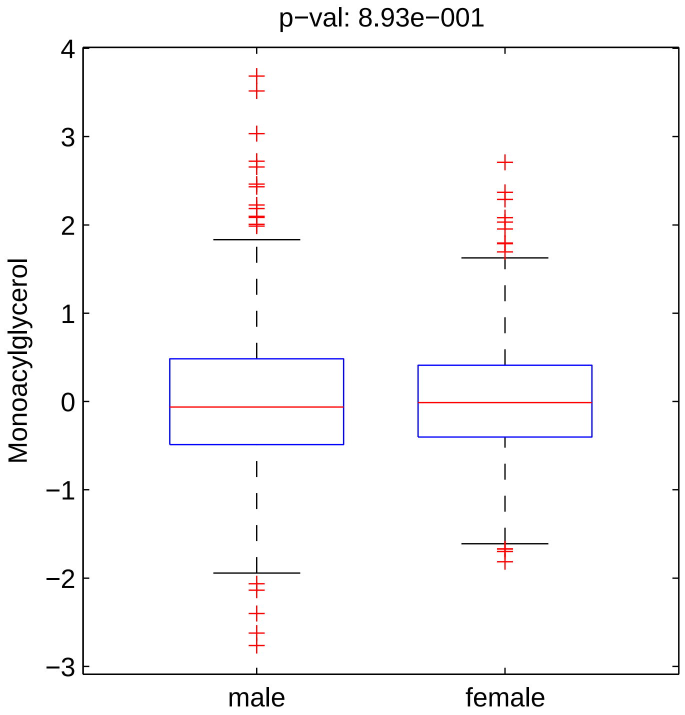
p−val: 8.93e−001
4
3
2
1
0
−1
−2
−3
Monoacylglycerol
male
female

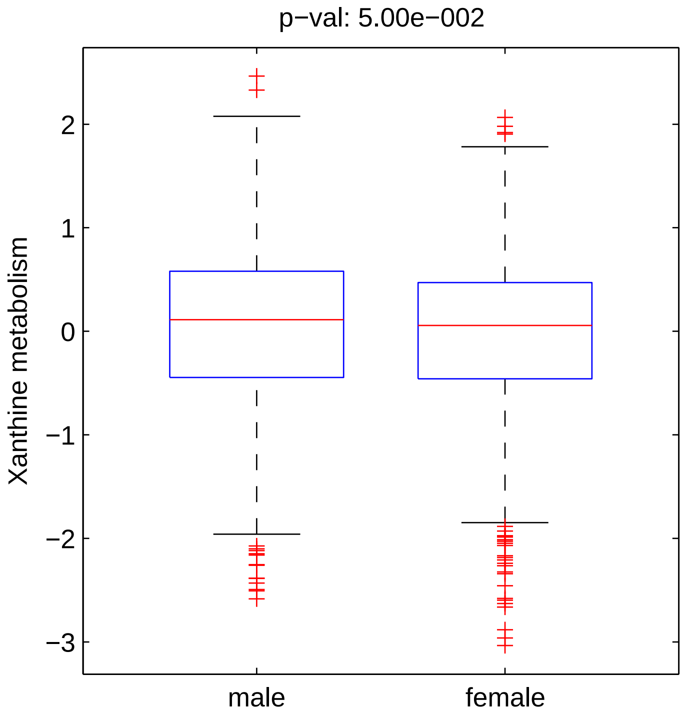
p−val: 5.00e−002
Xanthine metabolism
2
1
0
−1
−2
−3
male
female

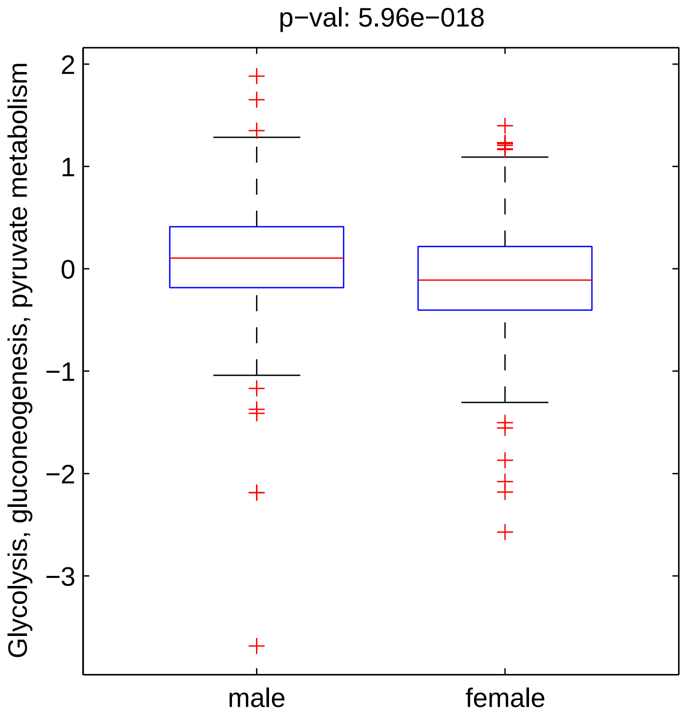
p−val: 5.96e−018
Glycolysis, gluconeogenesis, pyruvate metabolism
2
1
0
−1
−2
−3
male
female

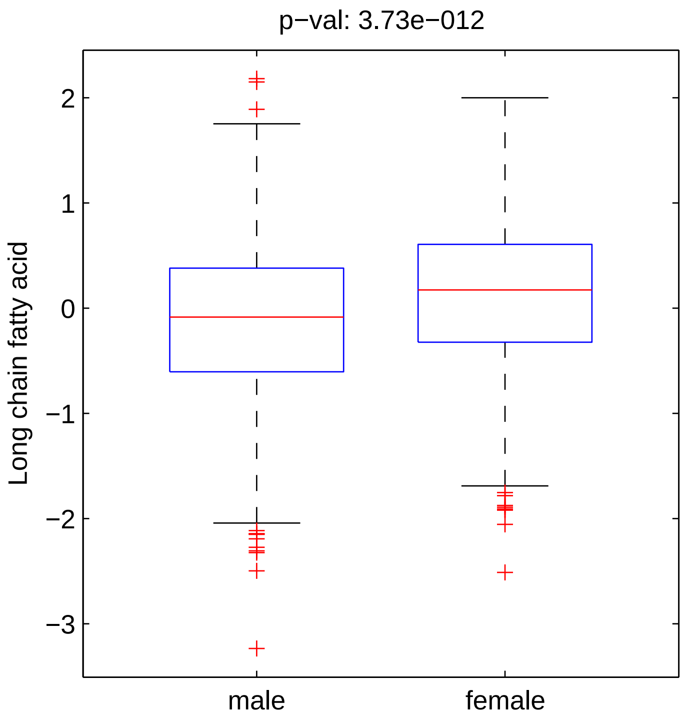

p−val: 3.73e−012
Long chain fatty acid
2
1
0
−1
−2
−3
male
female

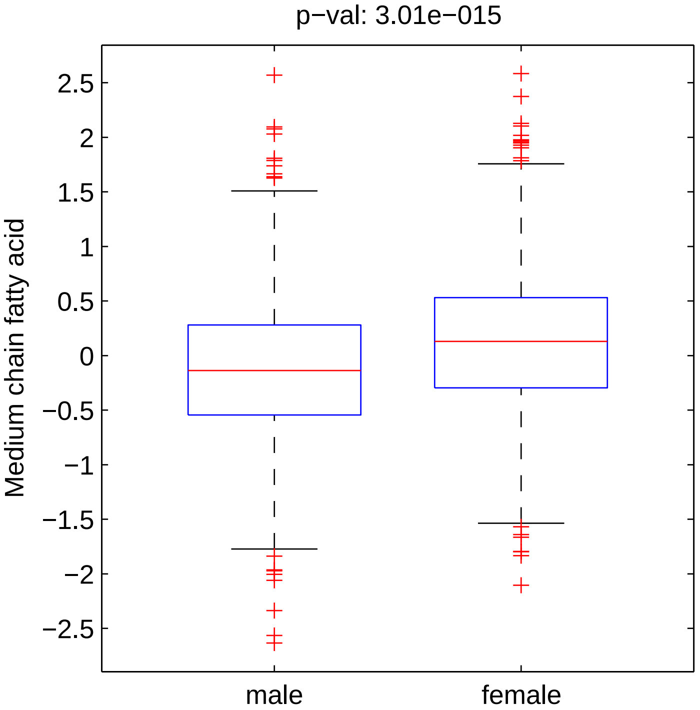
p−val: 3.01e−015
Medium chain fatty acid
2.5
2
1.5
1
0.5
0
−0.5
−1
−1.5
−2
−2.5
male
female

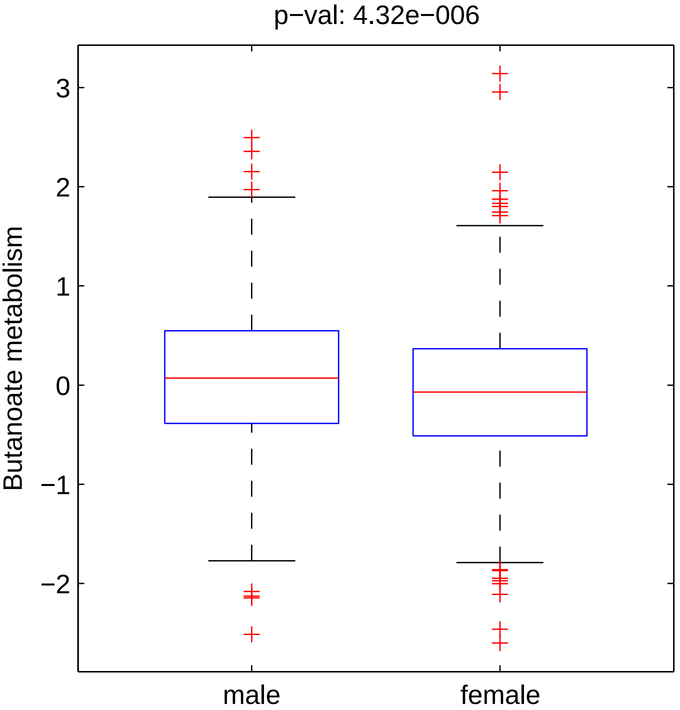

p−val: 4.32e−006
Butanoate metabolism
3
2
1
0
−1
−2
male
female

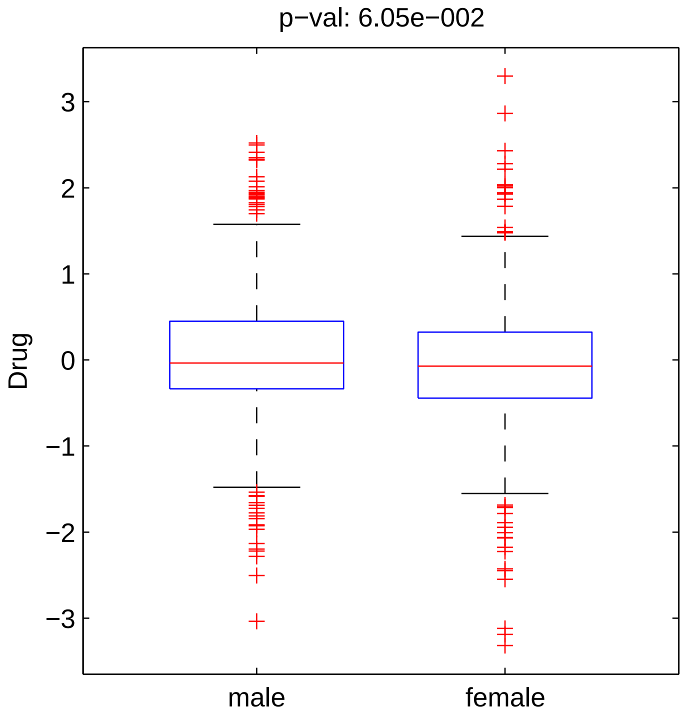
p−val: 6.05e−002
Drug
3
2
1
0
−1
−2
−3
male
female

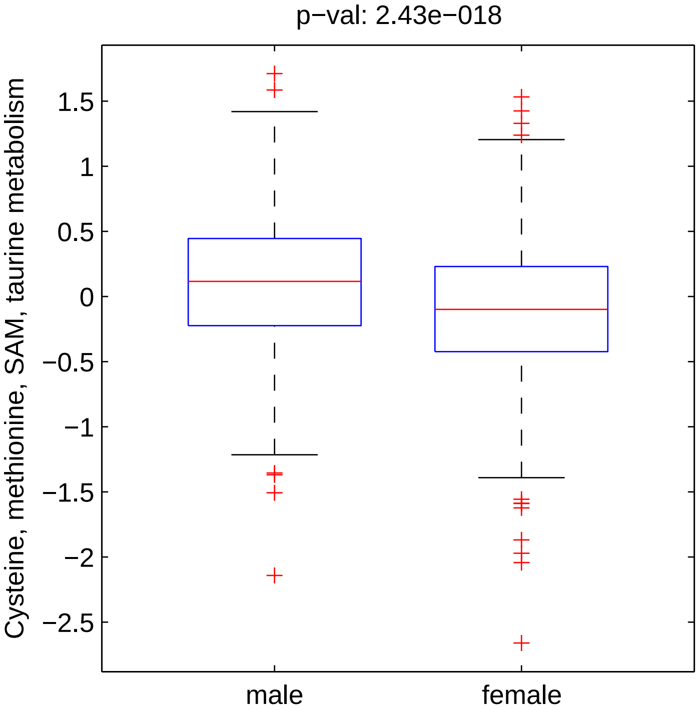
p−val: 2.43e−018
Cysteine, methionine, SAM, taurine metabolism
1.5
1
0.5
0
−0.5
−1
−1.5
−2
−2.5
male
female

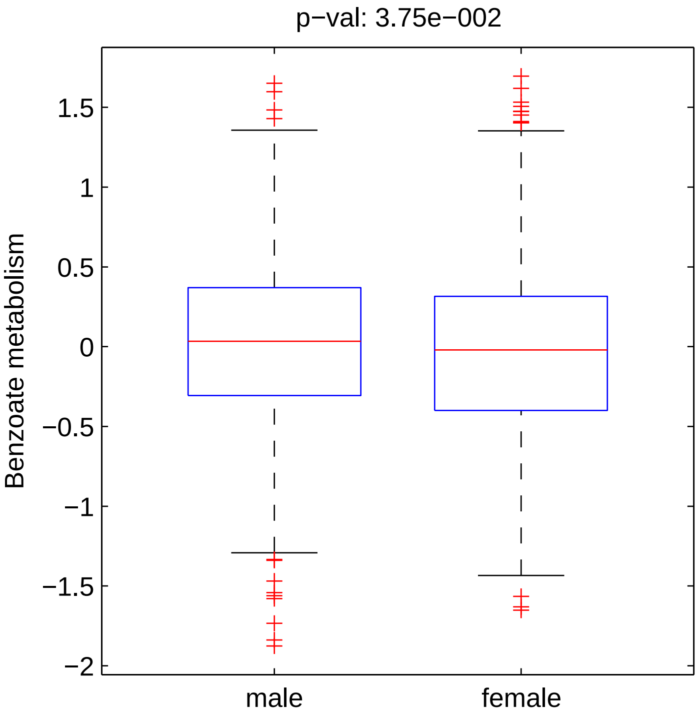

p−val: 3.75e−002
Benzoate metabolism
1.5
1
0.5
0
−0.5
−1
−1.5
−2
male
female

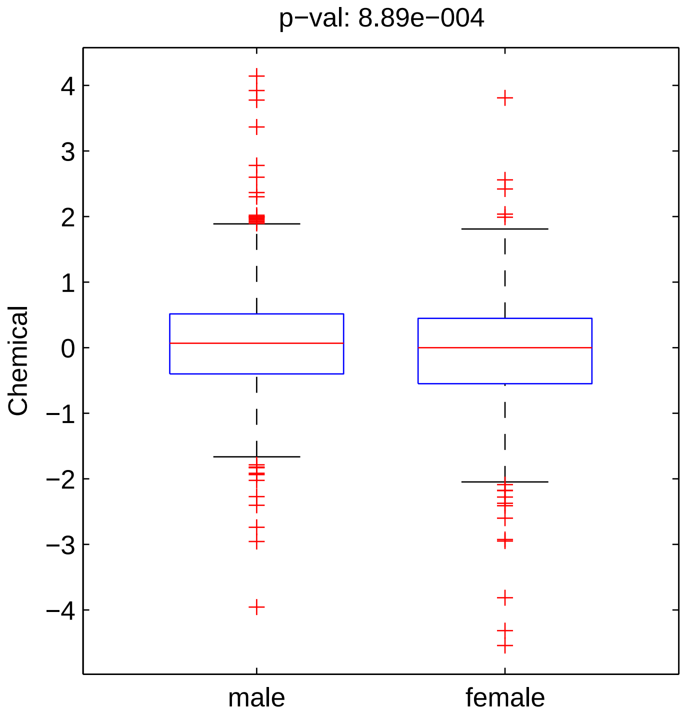
p−val: 8.89e−004
Chemical
4
3
2
1
0
−1
−2
−3
−4
male
female

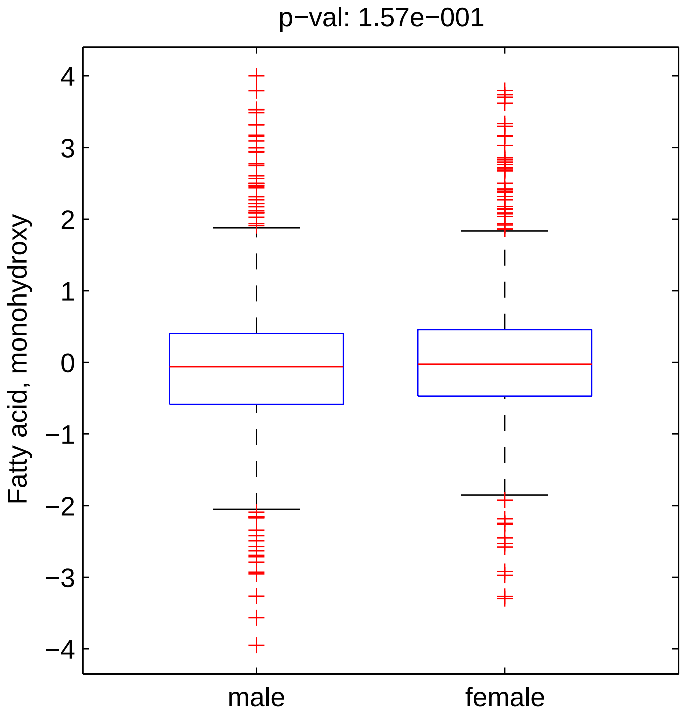
p−val: 1.57e−001
Fatty acid, monohydroxy
4
3
2
1
0
−1
−2
−3
−4
male
female

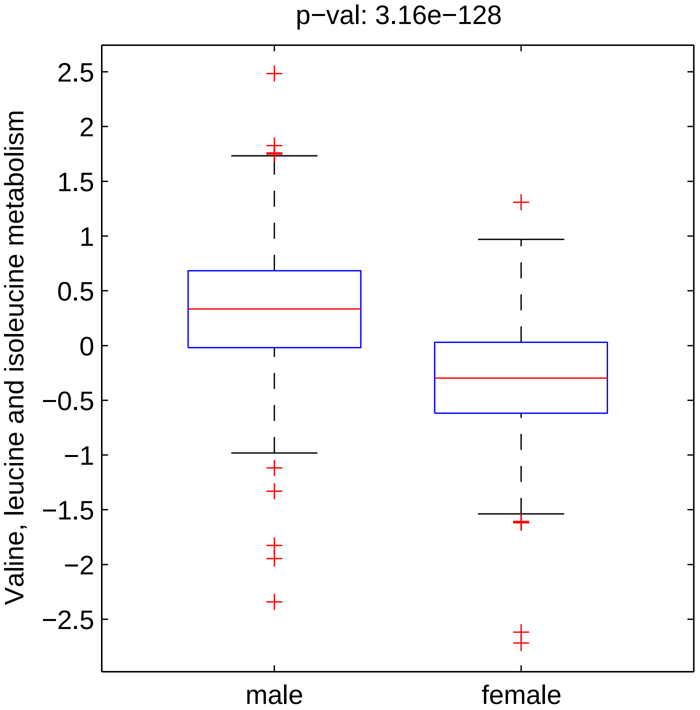
p−val: 3.16e−128
Valine, leucine and isoleucine metabolism
2.5
2
1.5
1
0.5
0
−0.5
−1
−1.5
−2
−2.5
male
female

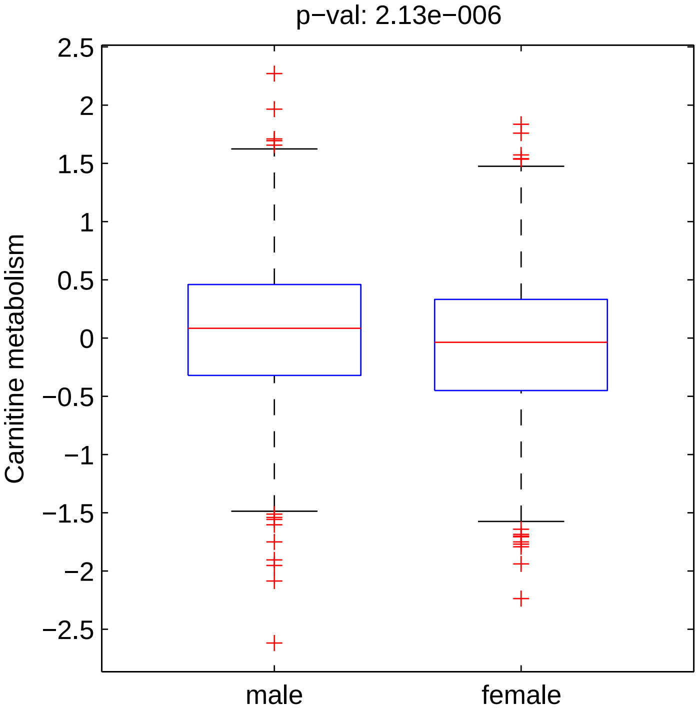
p−val: 2.13e−006
Carnitine metabolism
2.5
2
1.5
1
0.5
0
−0.5
−1
−1.5
−2
−2.5
male
female

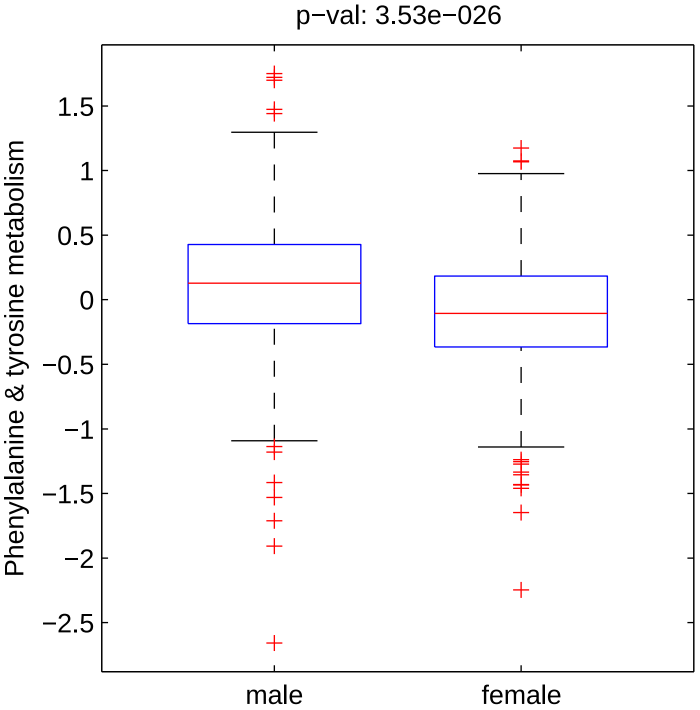
p−val: 3.53e−026
Phenylalanine & tyrosine metabolism
1.5
1
0.5
0
−0.5
−1
−1.5
−2
−2.5
male
female

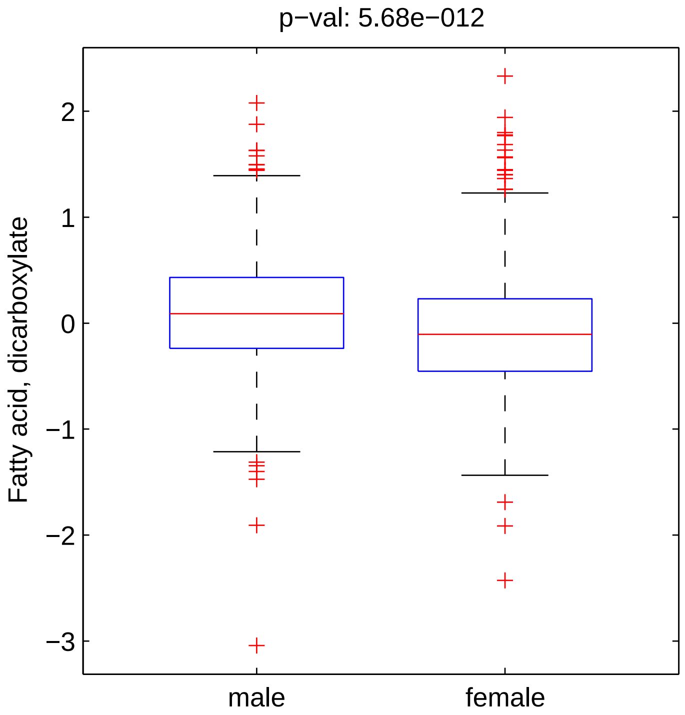

p−val: 5.68e−012
Fatty acid, dicarboxylate
2
1
0
−1
−2
−3
male
female

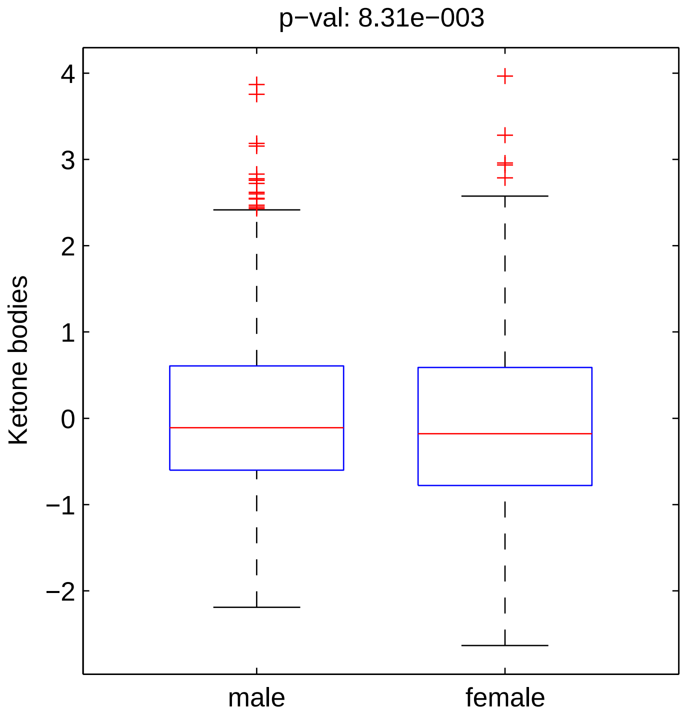
p−val: 8.31e−003
Ketone bodies
4
3
2
1
0
−1
−2
male
female

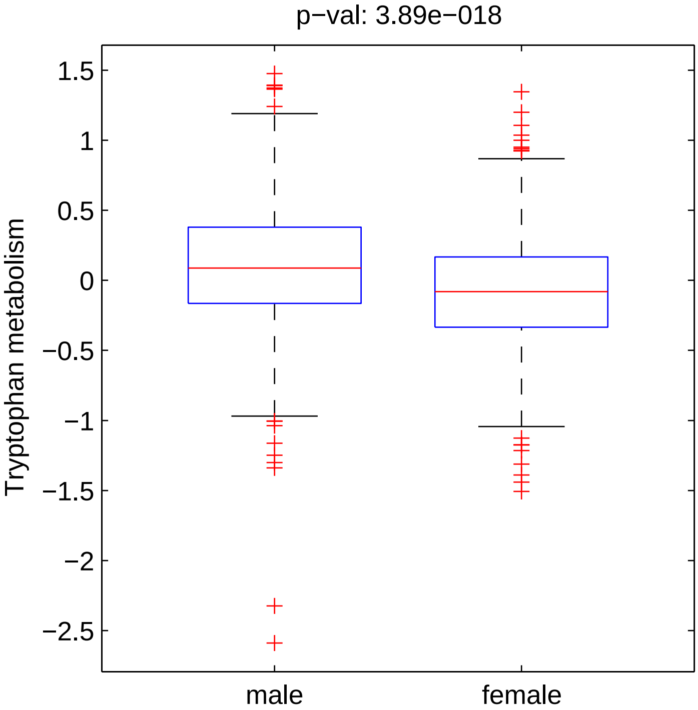
p−val: 3.89e−018
Tryptophan metabolism
1.5
1
0.5
0
−0.5
−1
−1.5
−2
−2.5
male
female

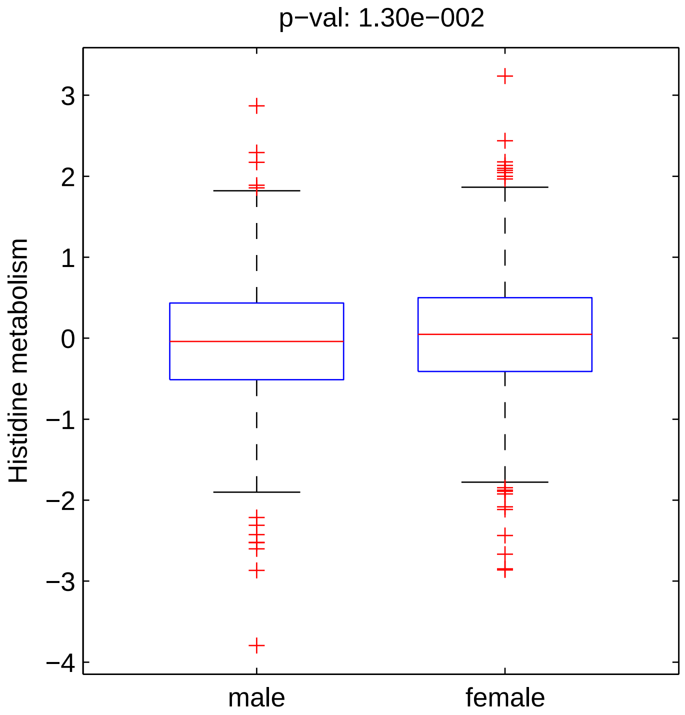
p−val: 1.30e−002
Histidine metabolism
3
2
1
0
−1
−2
−3
−4
male
female

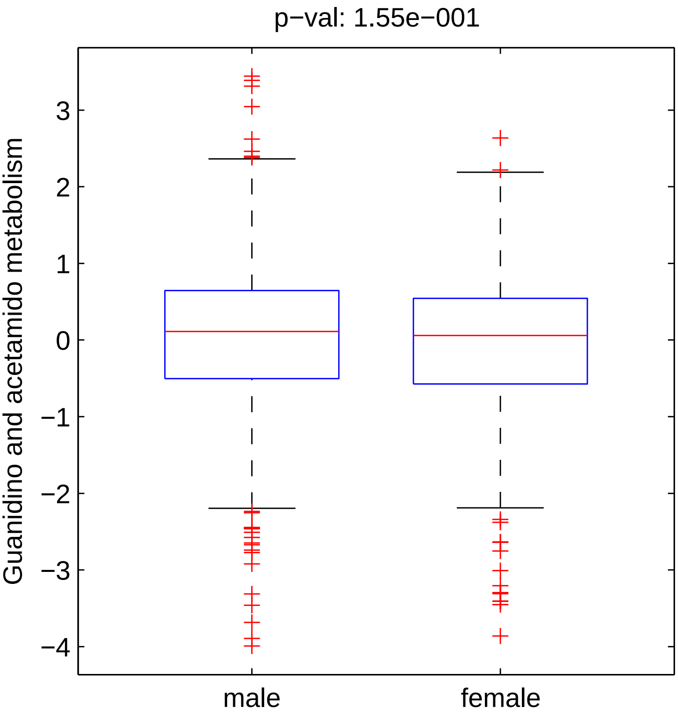

p−val: 1.55e−001
Guanidino and acetamido metabolism
3
2
1
0
−1
−2
−3
−4
male
female

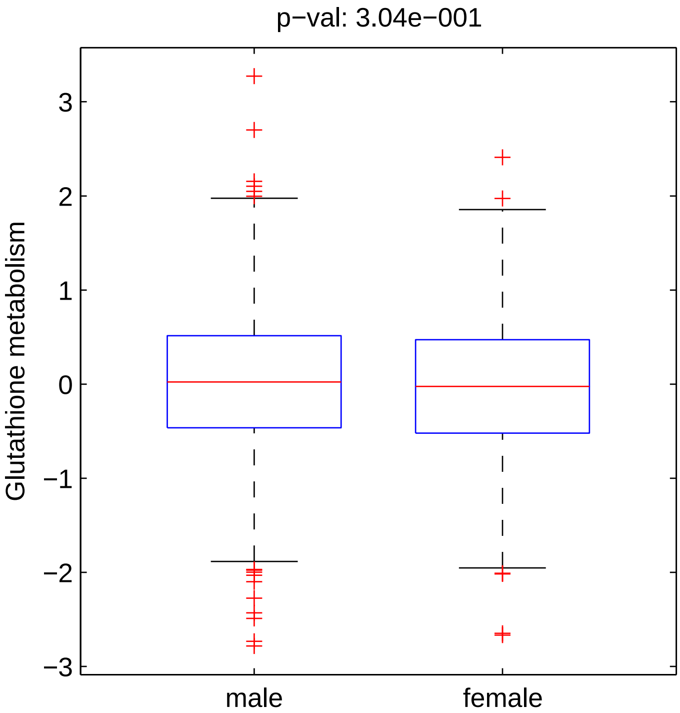
p−val: 3.04e−001
Glutathione metabolism
3
2
1
0
−1
−2
−3
male
female

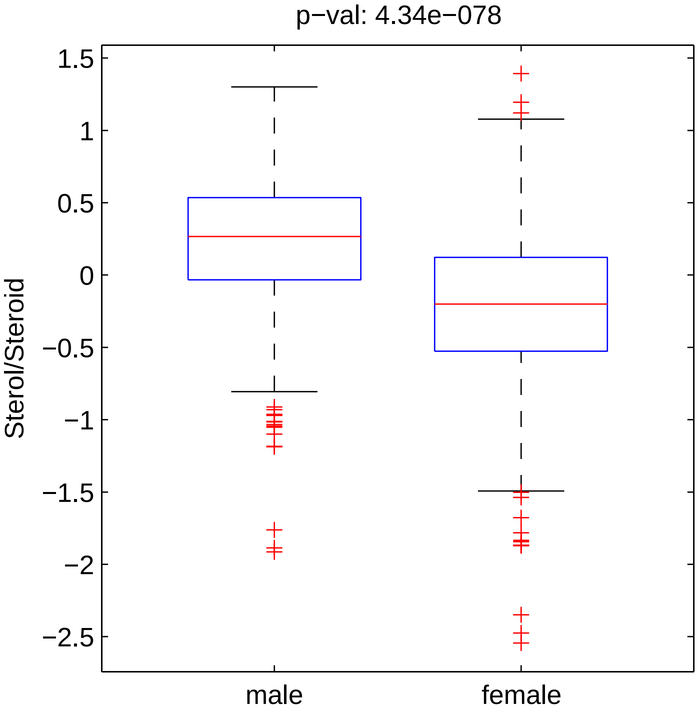
p−val: 4.34e−078
1.5
1
0.5
0
−0.5
−1
−1.5
−2
−2.5
Sterol/Steroid
male
female

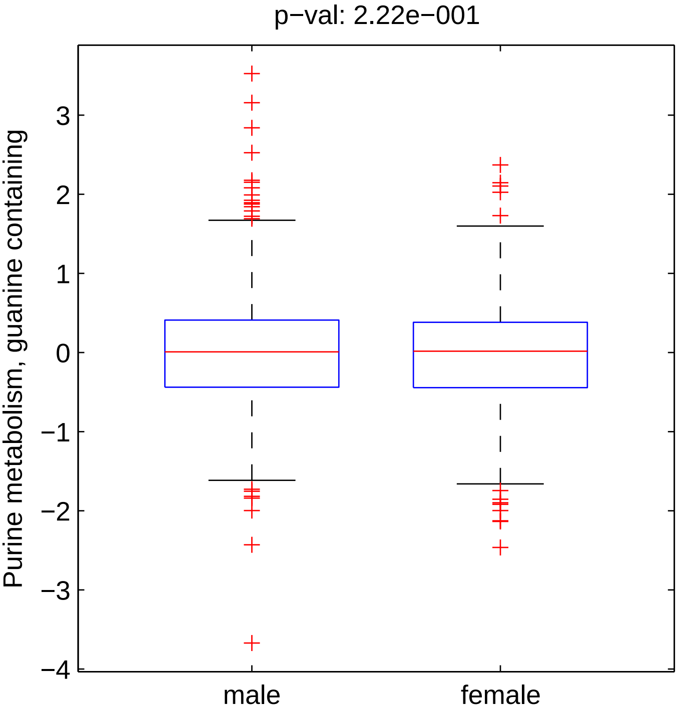
p−val: 2.22e−001
Purine metabolism, guanine containing
3
2
1
0
−1
−2
−3
−4
male
female

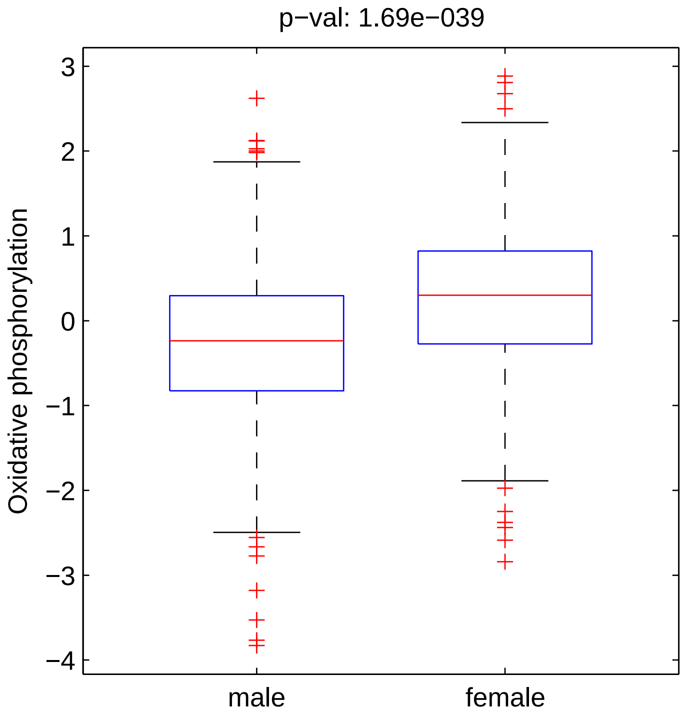

p−val: 1.69e−039
Oxidative phosphorylation
3
2
1
0
−1
−2
−3
−4
male
female

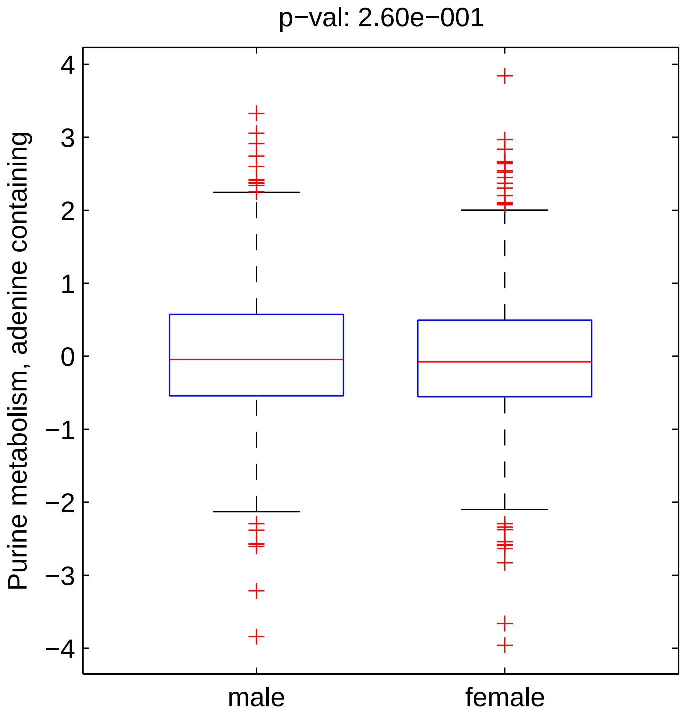
p−val: 2.60e−001
Purine metabolism, adenine containing
4
3
2
1
0
−1
−2
−3
−4
male
female

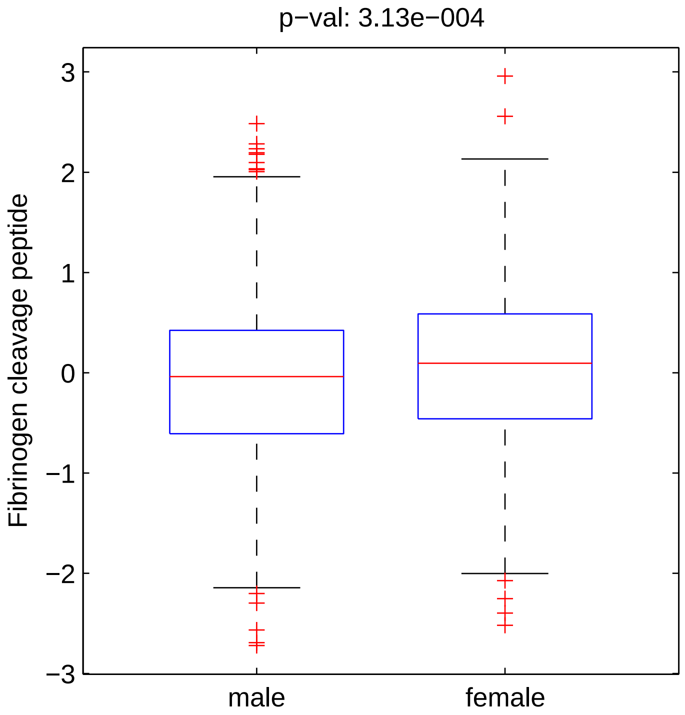

p−val: 3.13e−004
Fibrinogen cleavage peptide
3
2
1
0
−1
−2
−3
male
female

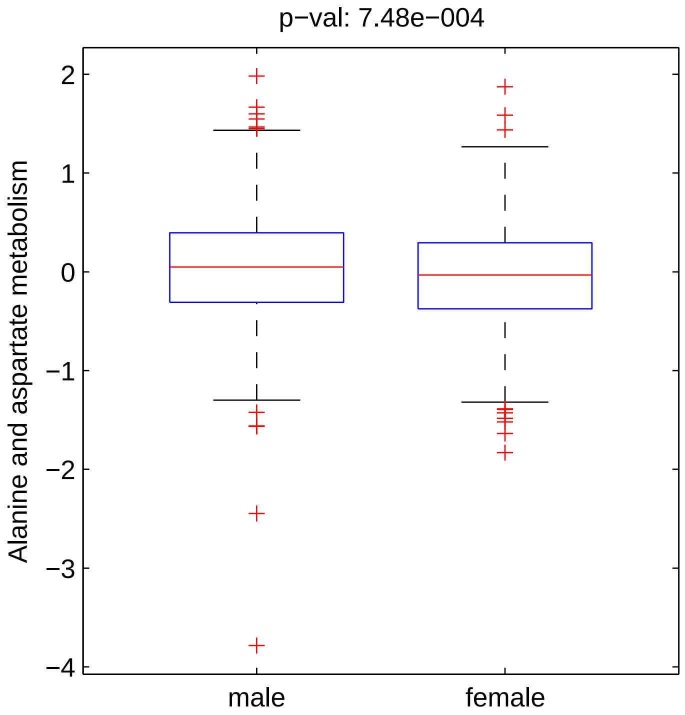

p−val: 7.48e−004
Alanine and aspartate metabolism
2
1
0
−1
−2
−3
−4
male
female

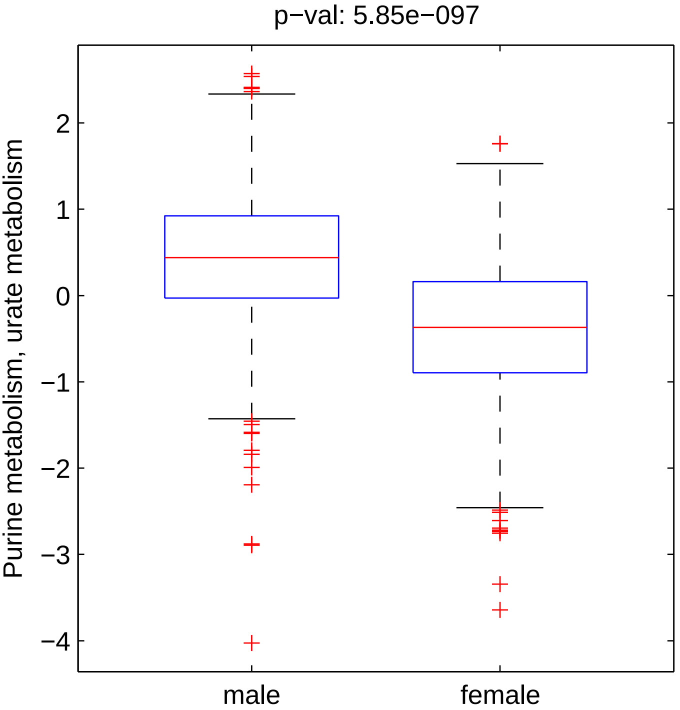

p−val: 5.85e−097
Purine metabolism, urate metabolism
2
1
0
−1
−2
−3
−4
male
female

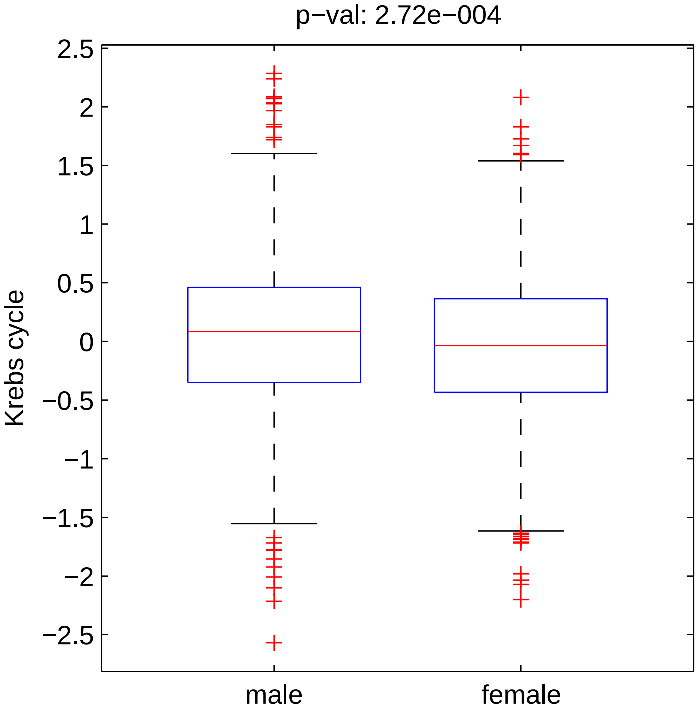

p−val: 2.72e−004
2.5
2
1.5
1
0.5
0
−0.5
−1
−1.5
−2
−2.5
Krebs cycle
male
female

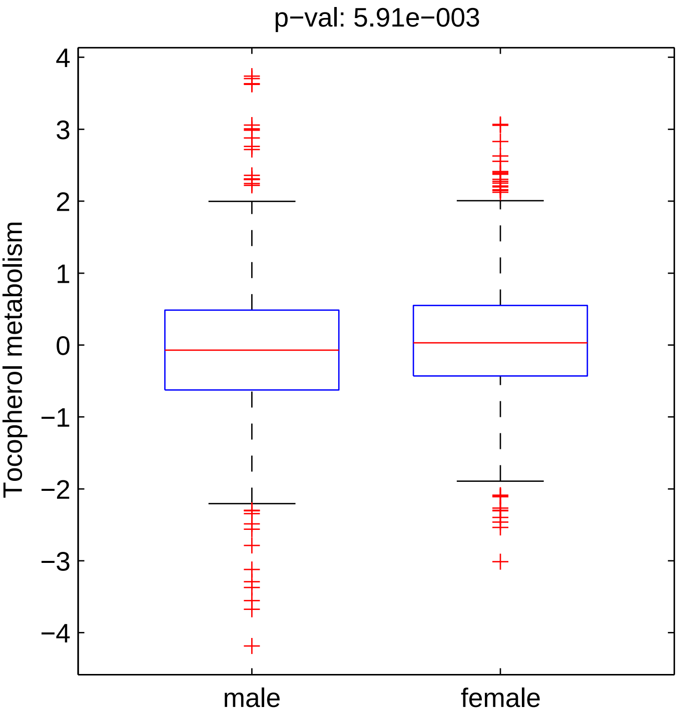
p−val: 5.91e−003
Tocopherol metabolism
4
3
2
1
0
−1
−2
−3
−4
male
female

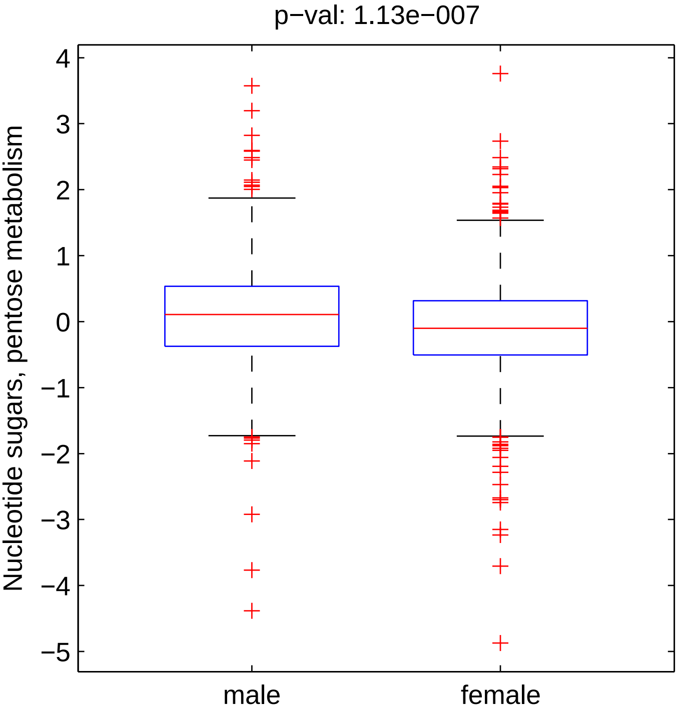
p−val: 1.13e−007
Nucleotide sugars, pentose metabolism
4
3
2
1
0
−1
−2
−3
−4
−5
male
female

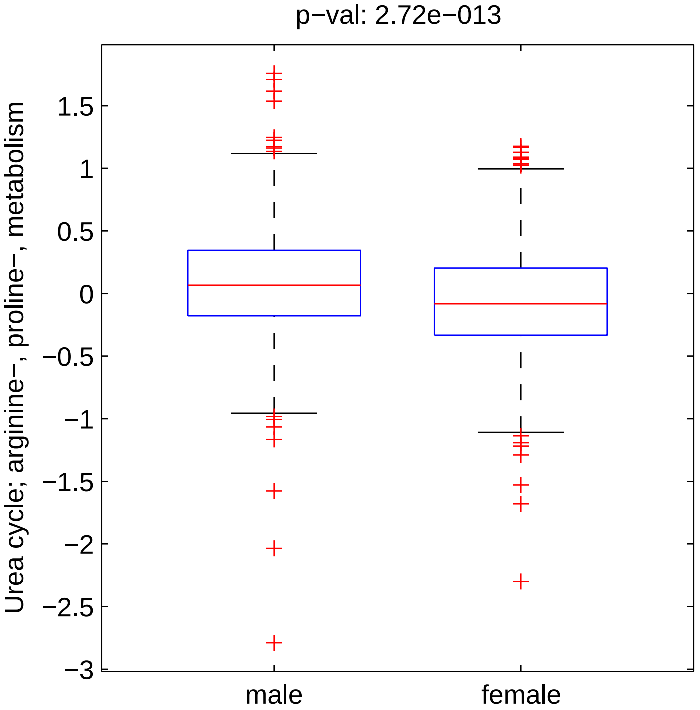
p−val: 2.72e−013
Urea cycle; arginine−, proline−, metabolism
1.5
1
0.5
0
−0.5
−1
−1.5
−2
−2.5
−3
male
female

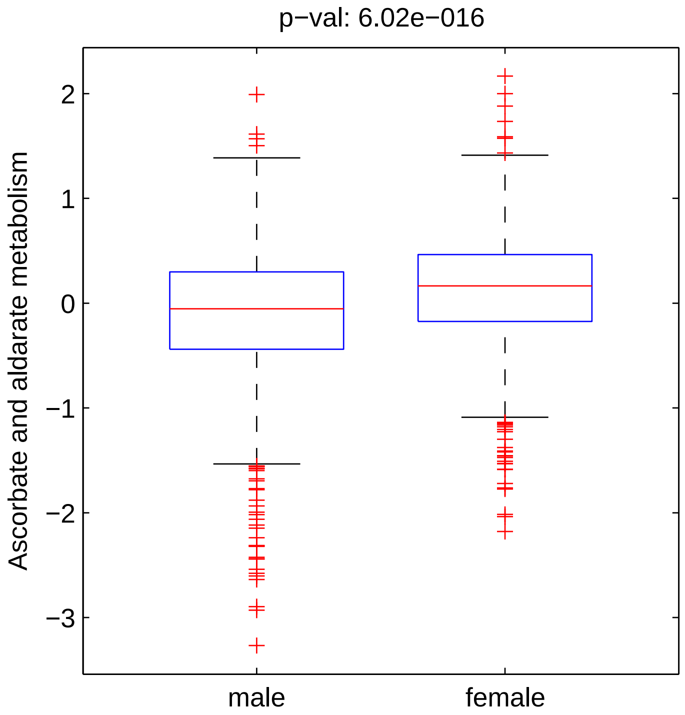

p−val: 6.02e−016
Ascorbate and aldarate metabolism
2
1
0
−1
−2
−3
male
female

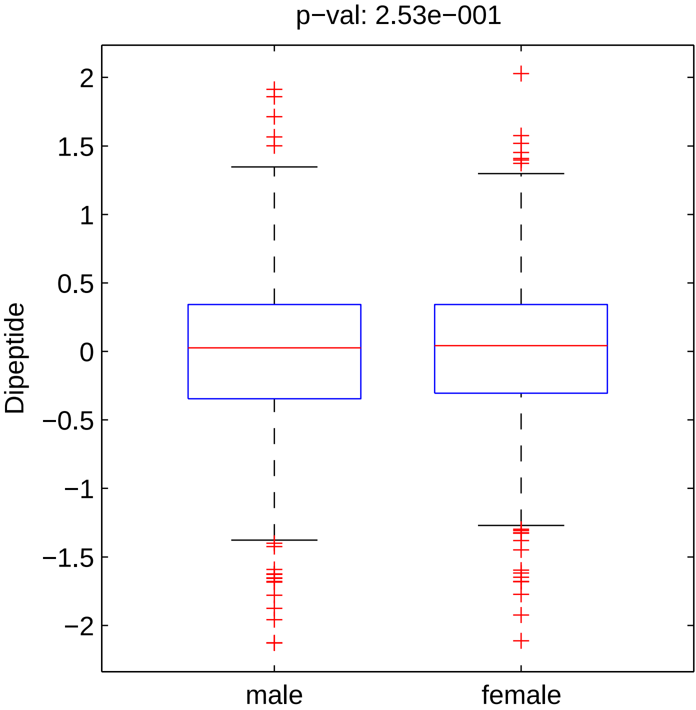
p−val: 2.53e−001
Dipeptide
2
1.5
1
0.5
0
−0.5
−1
−1.5
−2
male
female

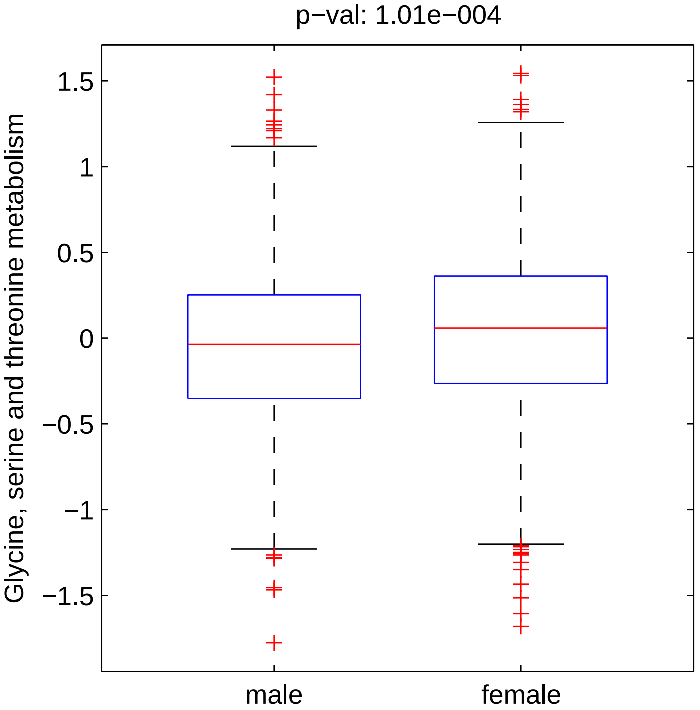

p−val: 1.01e−004
Glycine, serine and threonine metabolism
1.5
1
0.5
0
−0.5
−1
−1.5
male
female

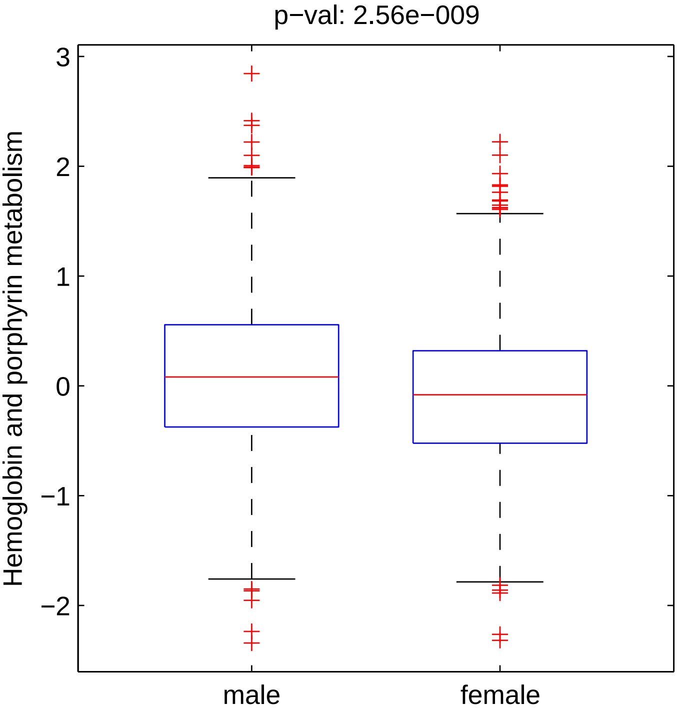

p−val: 2.56e−009
3
2
1
0
−1
−2
Hemoglobin and porphyrin metabolism
male
female

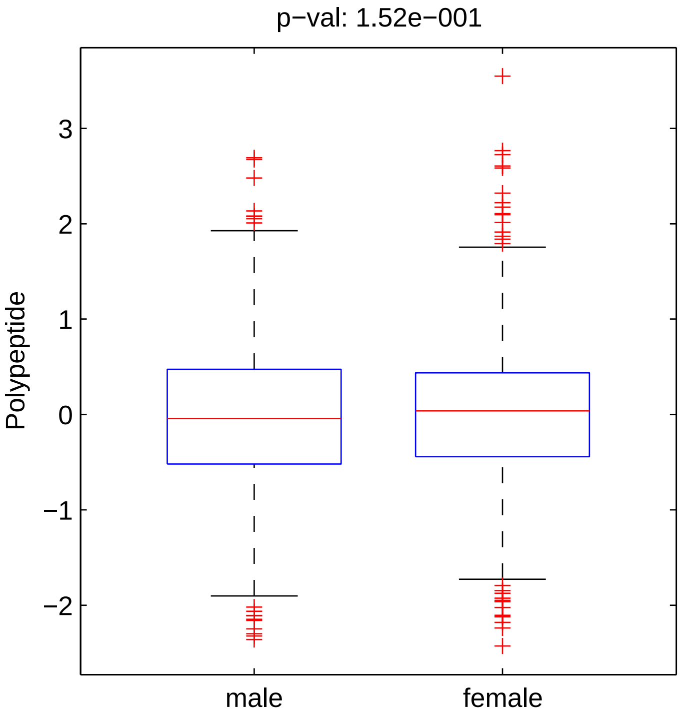

p−val: 1.52e−001
Polypeptide
3
2
1
0
−1
−2
male
female

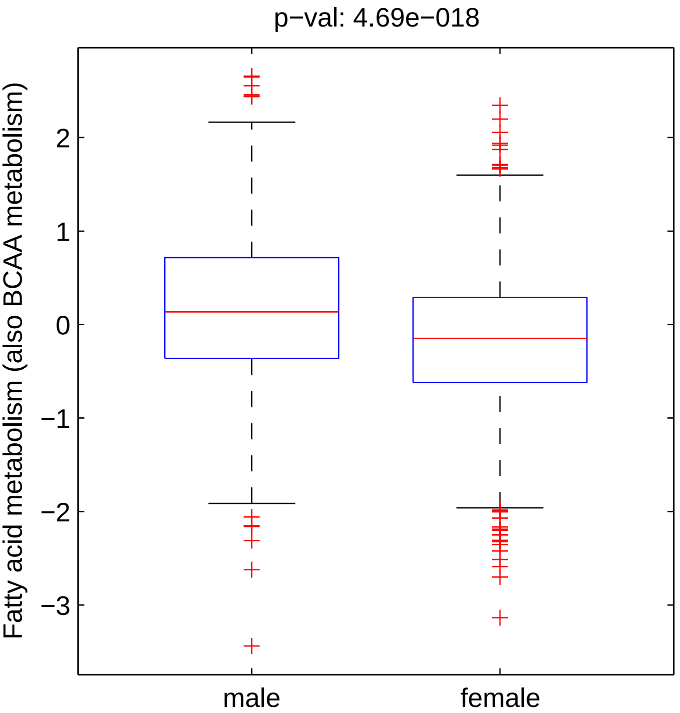

p−val: 4.69e−018
Fatty acid metabolism (also BCAA metabolism)
2
1
0
−1
−2
−3
male
female

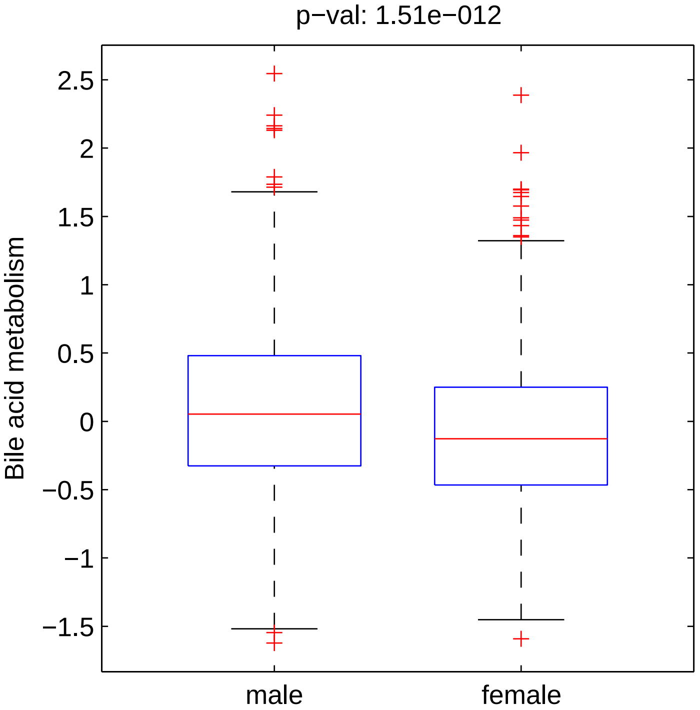

p−val: 1.51e−012
Bile acid metabolism
2.5
2
1.5
1
0.5
0
−0.5
−1
−1.5
male
female

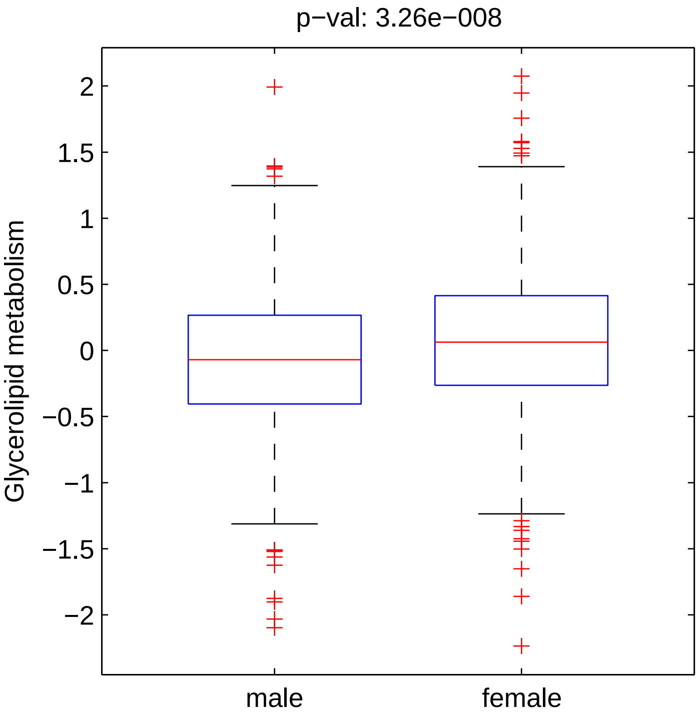
p−val: 3.26e−008
Glycerolipid metabolism
2
1.5
1
0.5
0
−0.5
−1
−1.5
−2
male
female

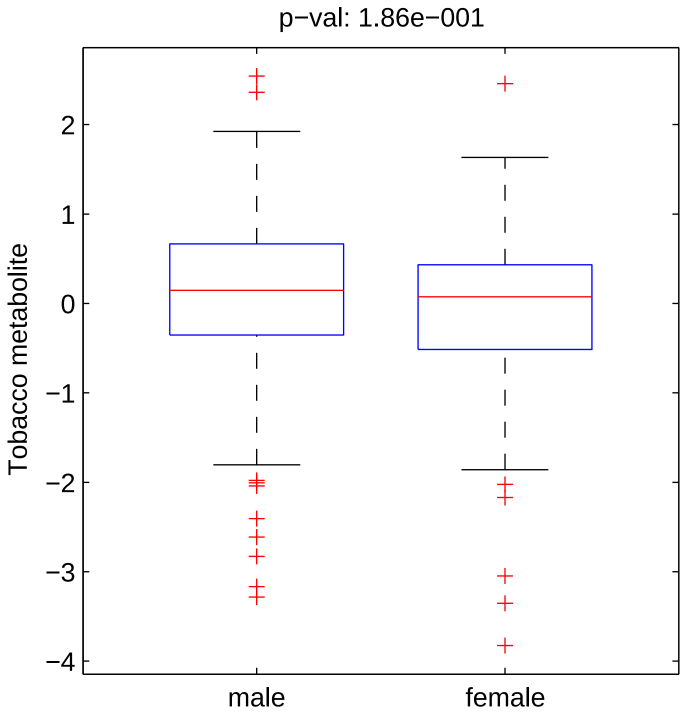
p−val: 1.86e−001
Tobacco metabolite
2
1
0
−1
−2
−3
−4
male
female

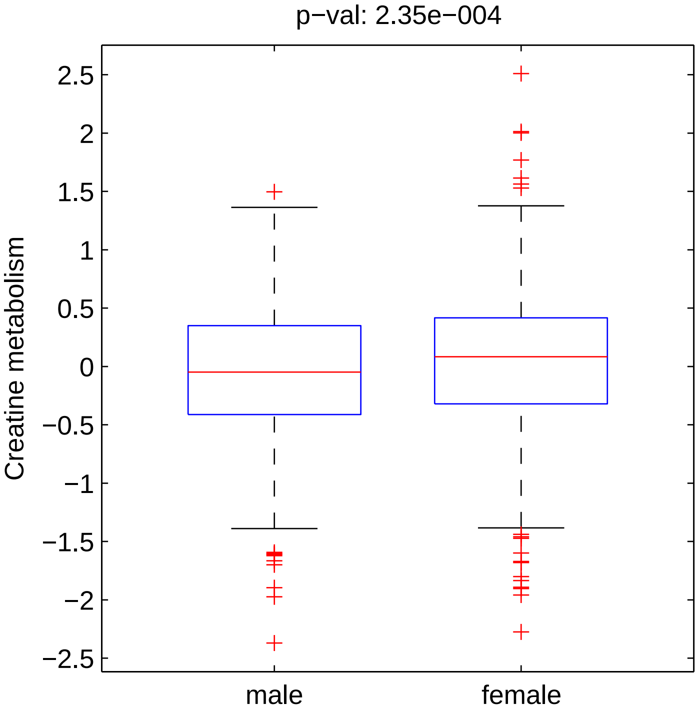

p−val: 2.35e−004
Creatine metabolism
2.5
2
1.5
1
0.5
0
−0.5
−1
−1.5
−2
−2.5
male
female

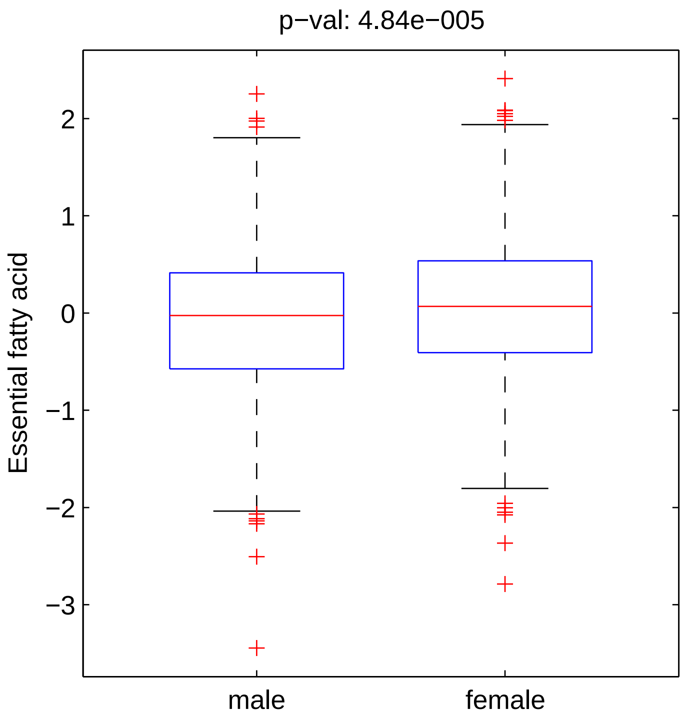
p−val: 4.84e−005
Essential fatty acid
2
1
0
−1
−2
−3
male
female

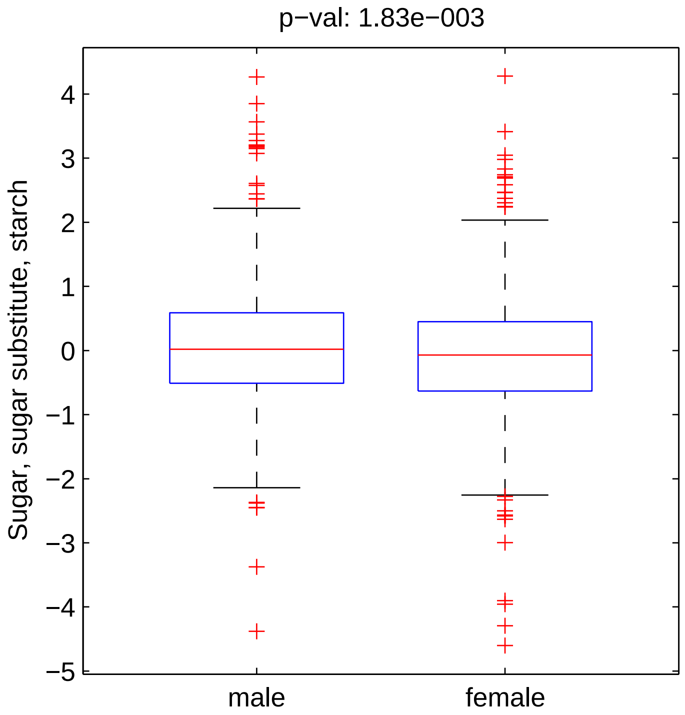
p−val: 1.83e−003
Sugar, sugar substitute, starch
4
3
2
1
0
−1
−2
−3
−4
−5
male
female

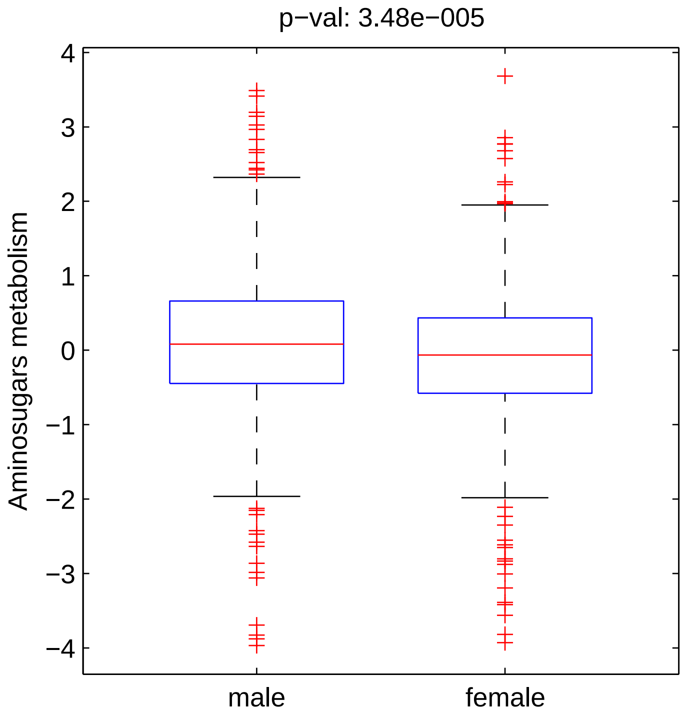

p−val: 3.48e−005
Aminosugars metabolism
4
3
2
1
0
−1
−2
−3
−4
male
female

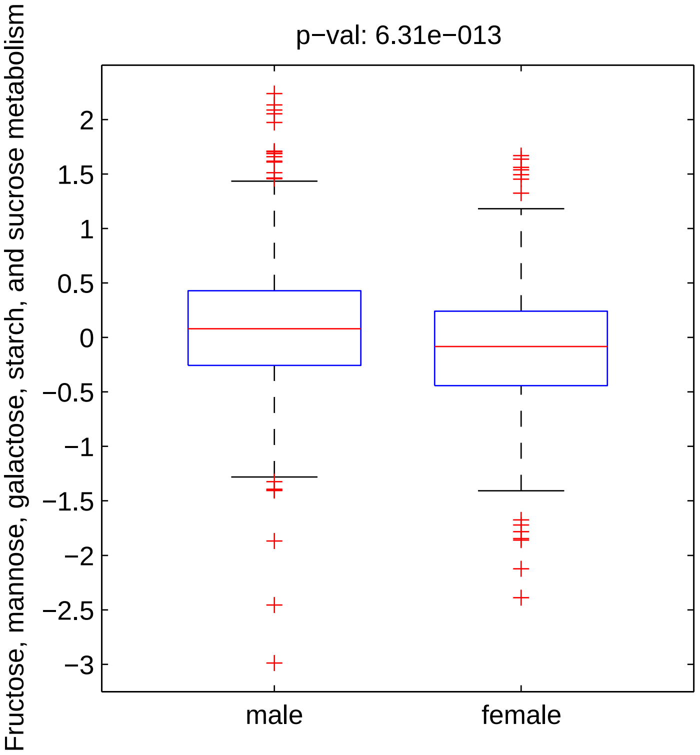
p−val: 6.31e−013
Fructose, mannose, galactose, starch, and sucrose metabolism
2
1.5
1
0.5
0
−0.5
−1
−1.5
−2
−2.5
−3
male
female

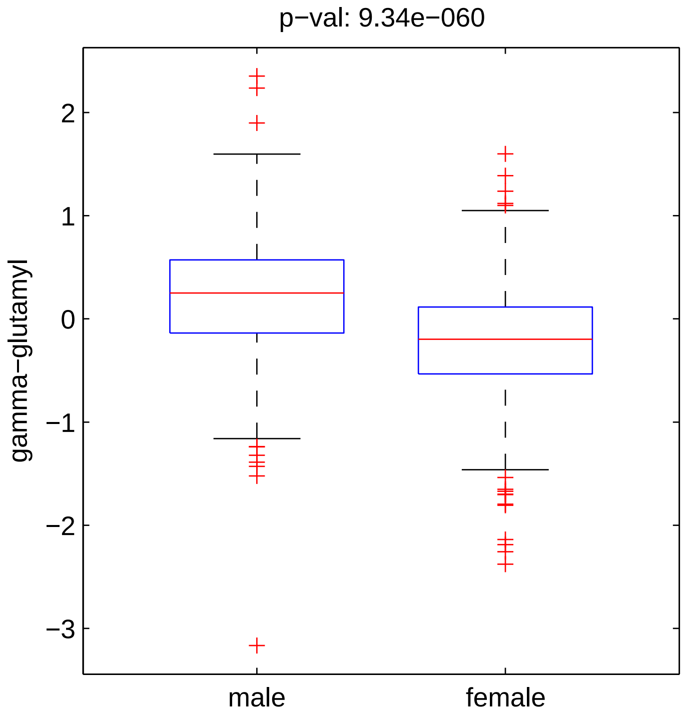
p−val: 9.34e−060
gamma-glutamyl
2
1
0
−1
−2
−3
male
female

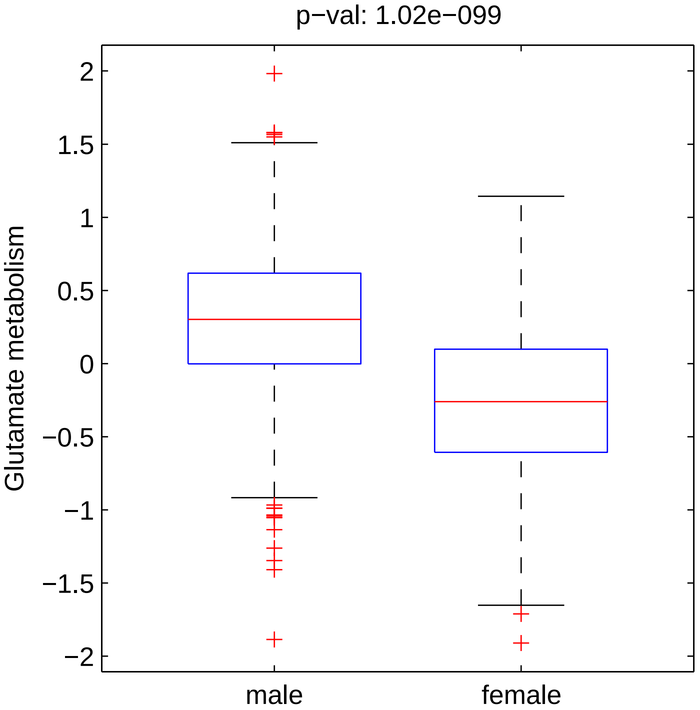

p−val: 1.02e−099
Glutamate metabolism
2
1.5
1
0.5
0
−0.5
−1
−1.5
−2
male
female

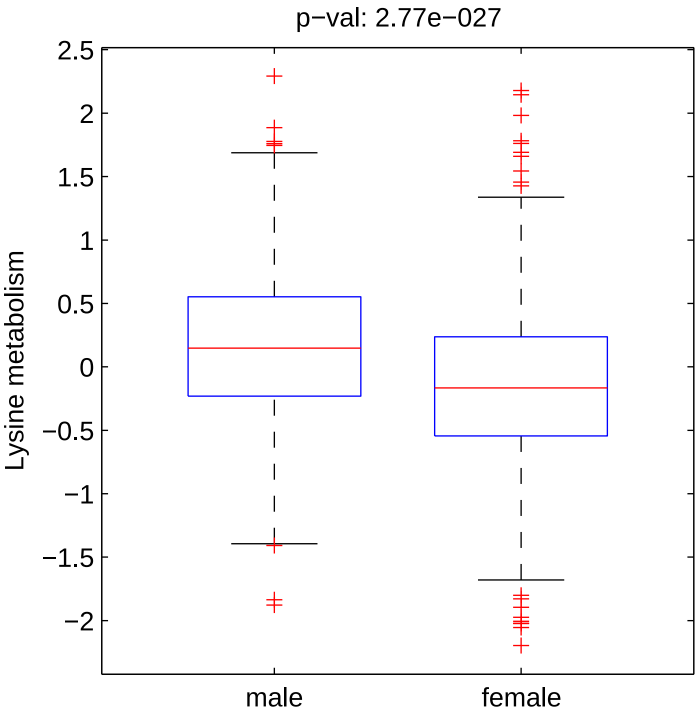

p−val: 2.77e−027
Lysine metabolism
2.5
2
1.5
1
0.5
0
−0.5
−1
−1.5
−2
male
female

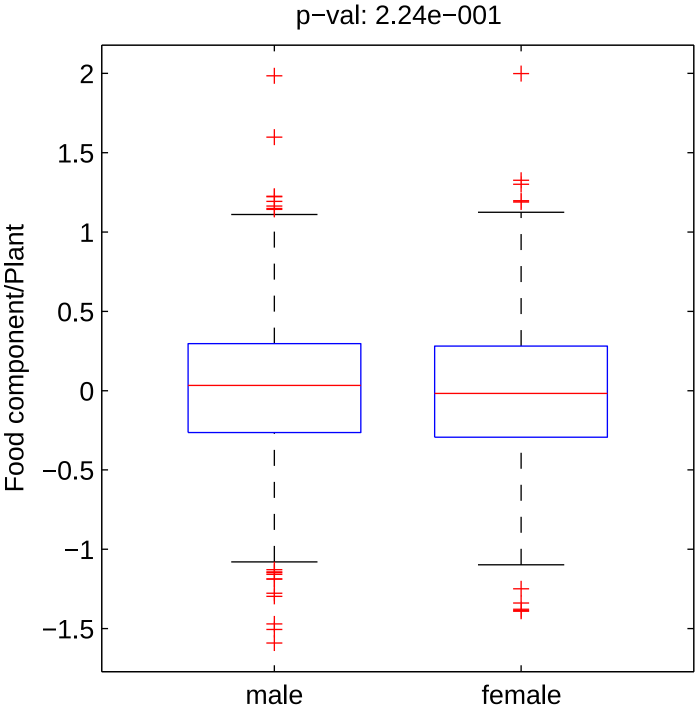
p−val: 2.24e−001
Food component/Plant
2
1.5
1
0.5
0
−0.5
−1
−1.5
male
female

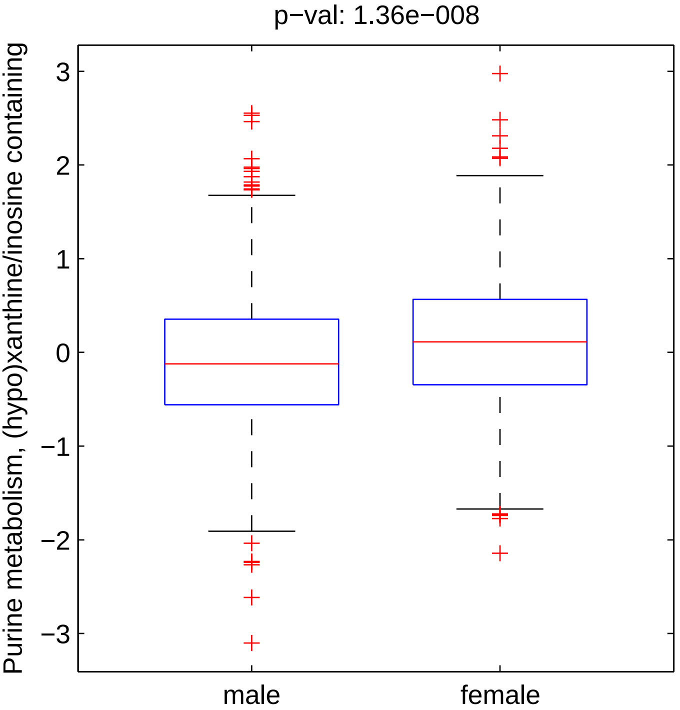
p−val: 1.36e−008
Purine metabolism, (hypo)xanthine/inosine containing
3
2
1
0
−1
−2
−3
male
female

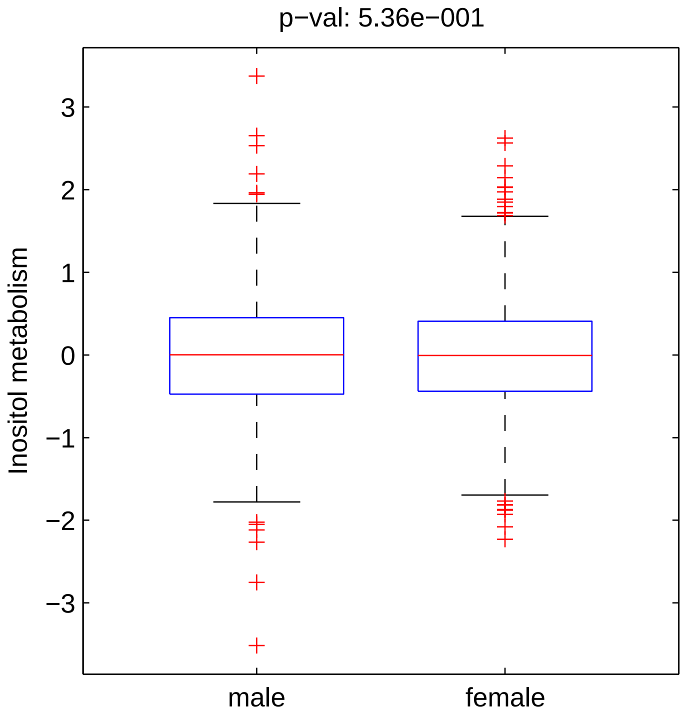
p−val: 5.36e−001
3
2
1
0
−1
−2
−3
Inositol metabolism
male
female

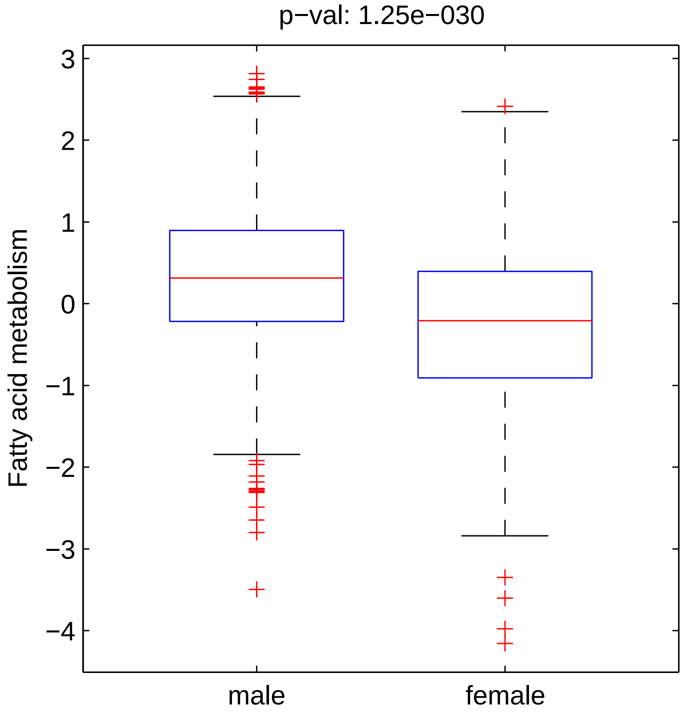

p−val: 1.25e−030
Fatty acid metabolism
3
2
1
0
−1
−2
−3
−4
male
female

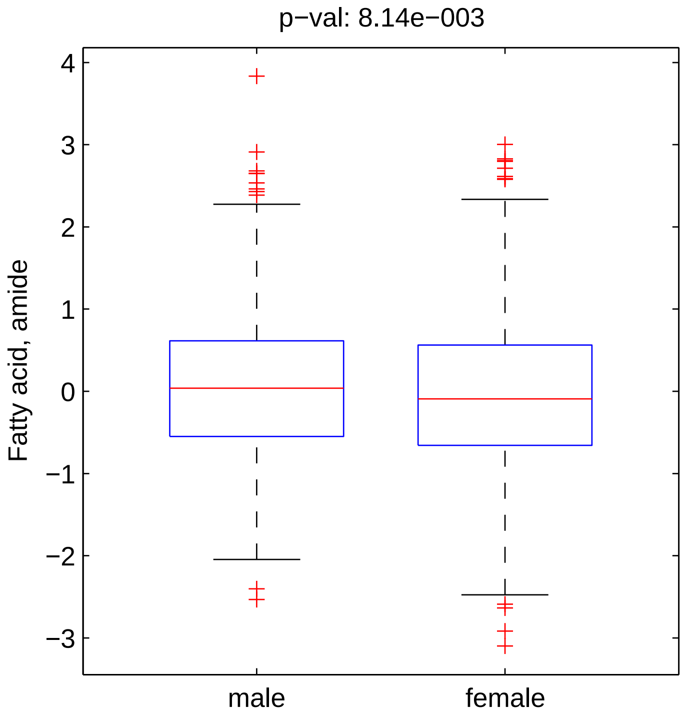
p−val: 8.14e−003
4
3
2
1
0
−1
−2
−3
Fatty acid, amide
male
female

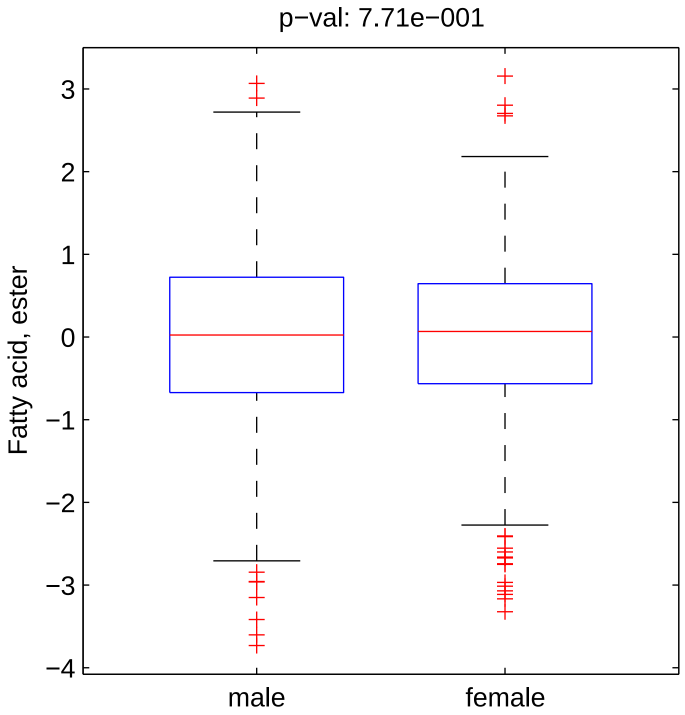
p−val: 7.71e−001
Fatty acid, ester
3
2
1
0
−1
−2
−3
−4
male
female

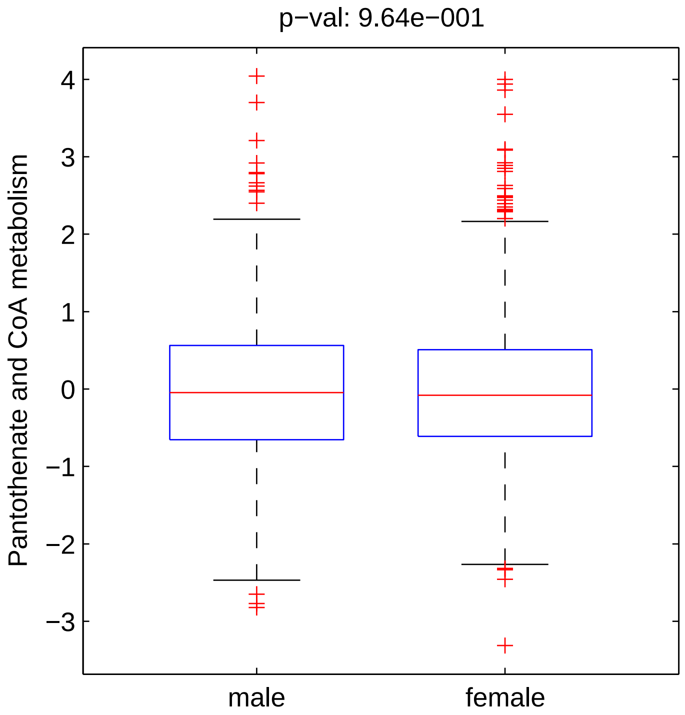

p−val: 9.64e−001
Pantothenate and CoA metabolism
4
3
2
1
0
−1
−2
−3
male
female

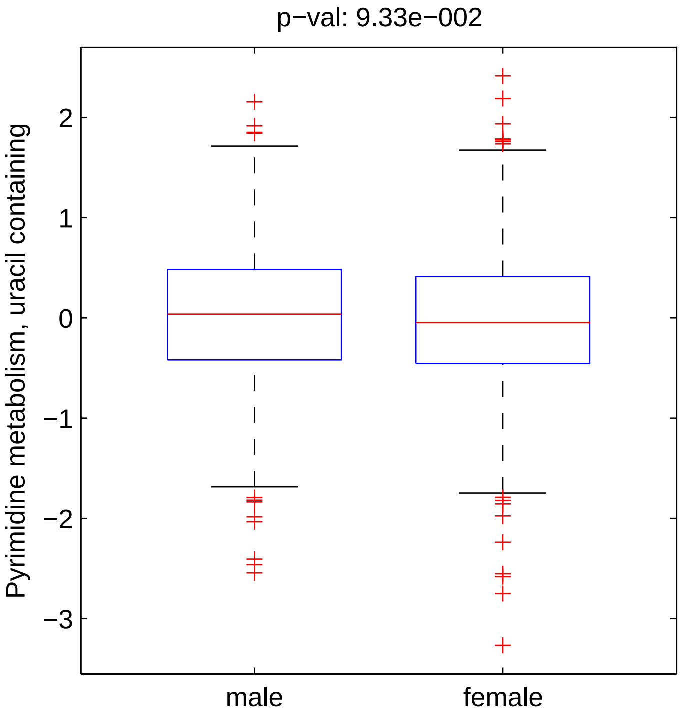

p−val: 9.33e−002
Pyrimidine metabolism, uracil containing
2
1
0
−1
−2
−3
male
female

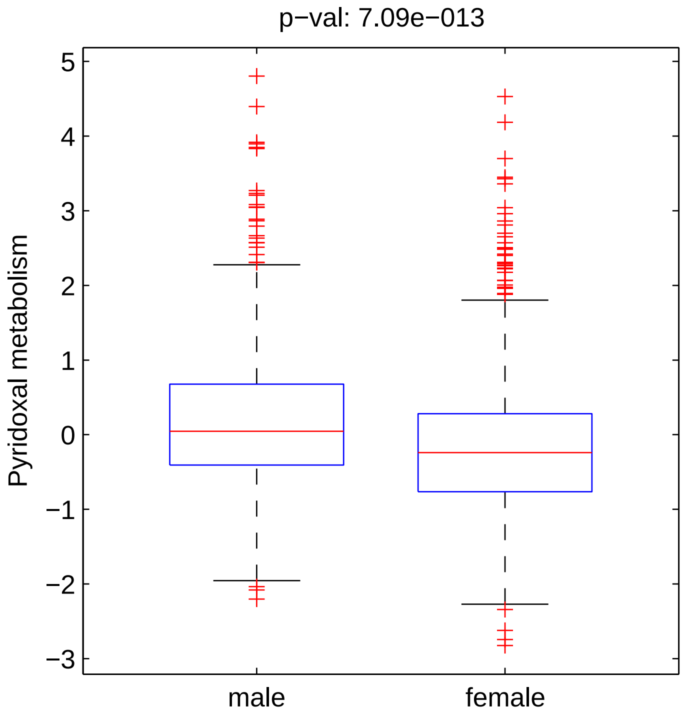

p−val: 7.09e−013
Pyridoxal metabolism
5
4
3
2
1
0
−1
−2
−3
male
female

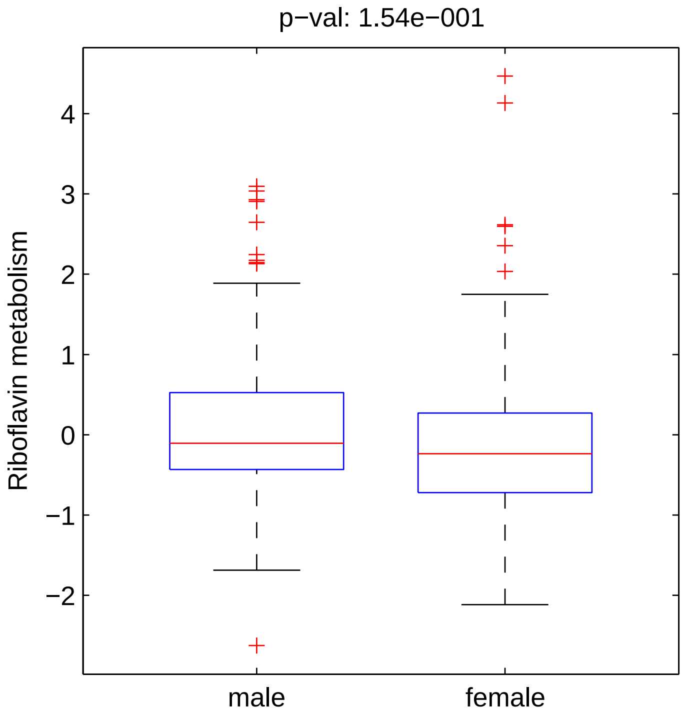
p−val: 1.54e−001
Riboflavin metabolism
4
3
2
1
0
−1
−2
male
female

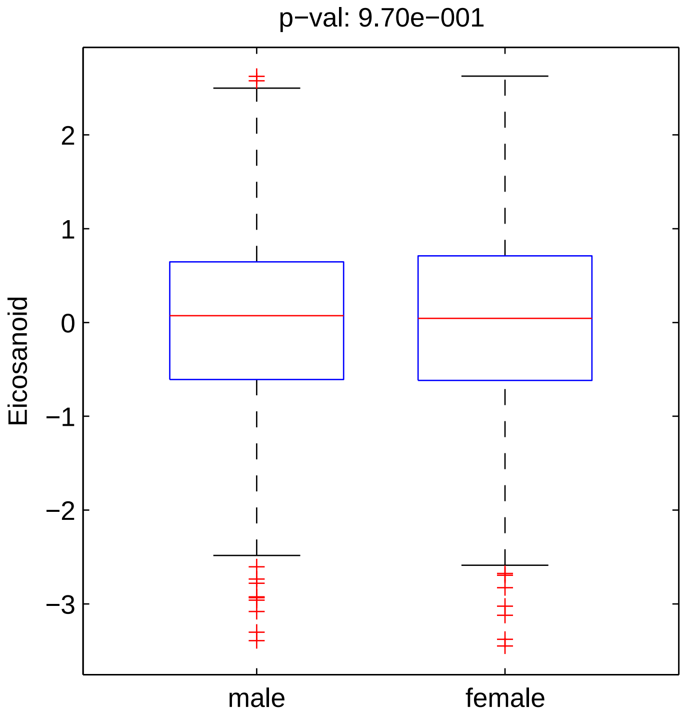
p−val: 9.70e−001
Eicosanoid
2
1
0
−1
−2
−3
male
female

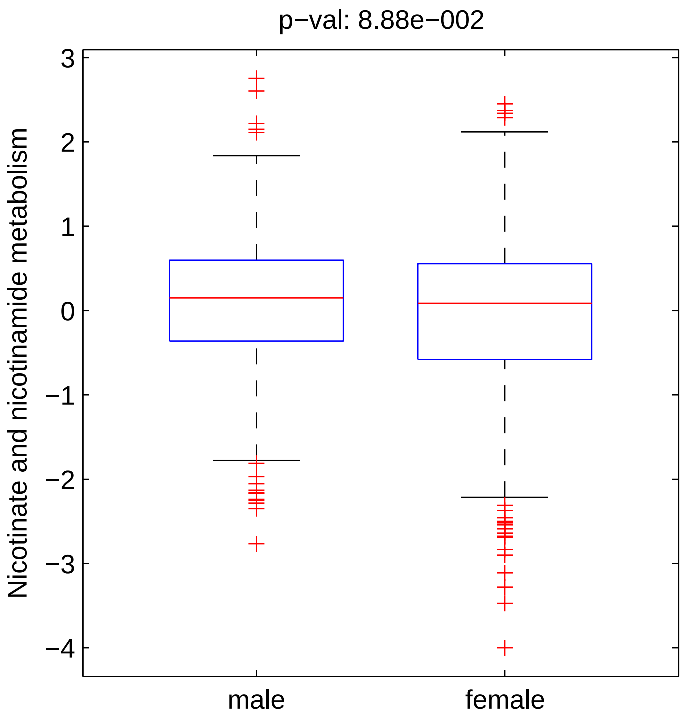

p−val: 8.88e−002
Nicotinate and nicotinamide metabolism
3
2
1
0
−1
−2
−3
−4
male
female

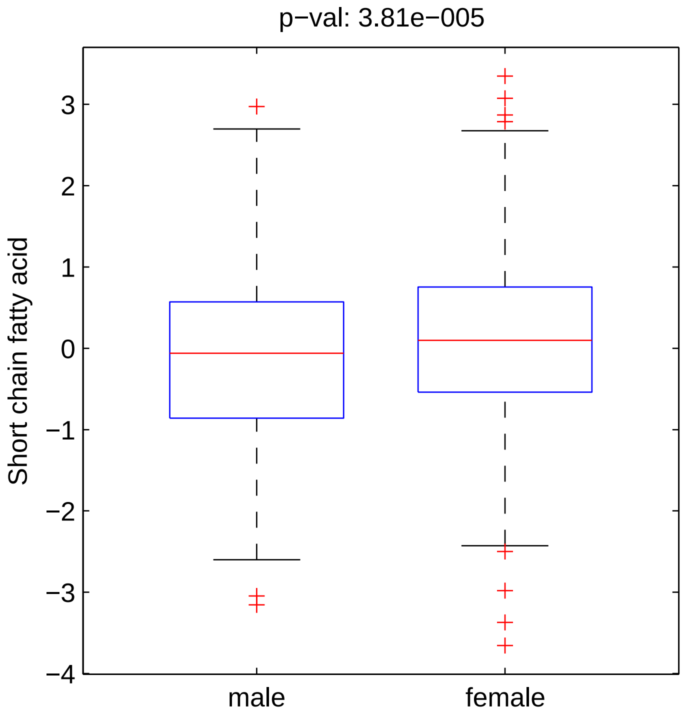
p−val: 3.81e−005
Short chain fatty acid
3
2
1
0
−1
−2
−3
−4
male
female

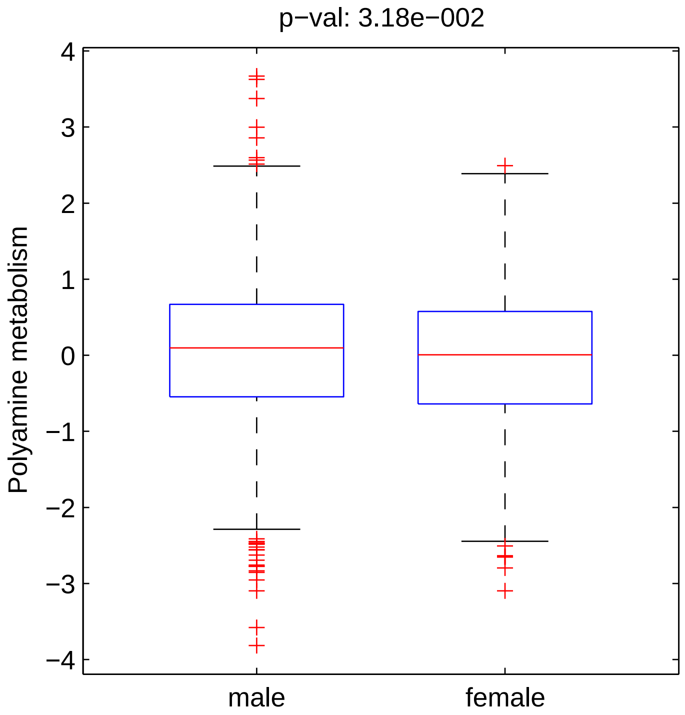
p−val: 3.18e−002
Polyamine metabolism
4
3
2
1
0
−1
−2
−3
−4
male
female

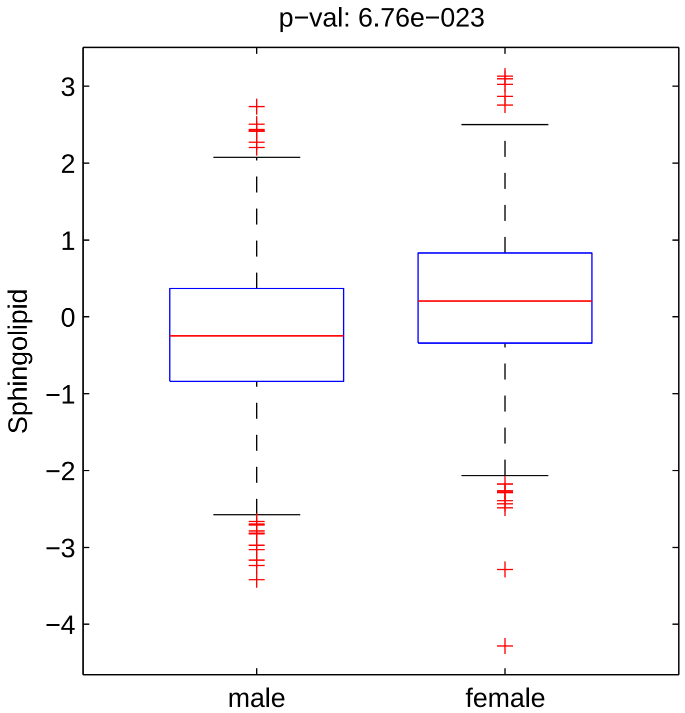
p−val: 6.76e−023
Sphingolipid
3
2
1
0
−1
−2
−3
−4
male
female

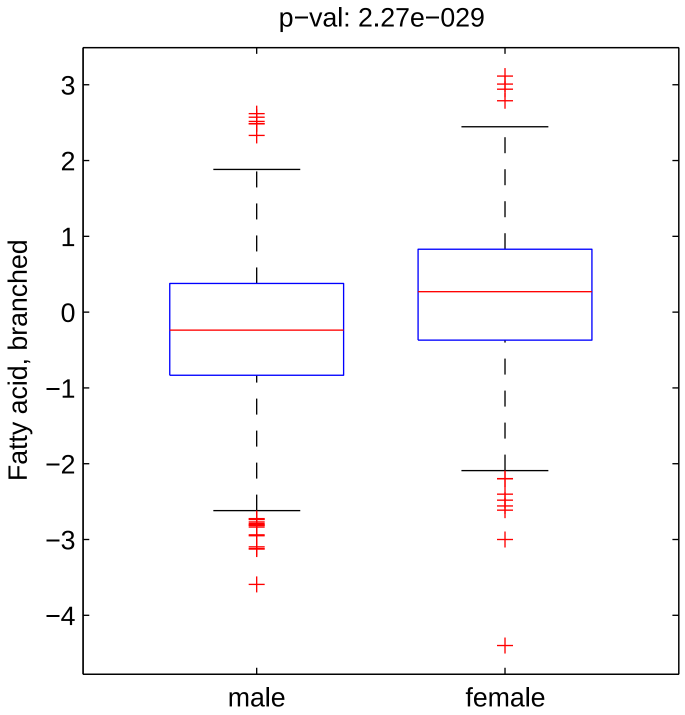
p−val: 2.27e−029
Fatty acid, branched
3
2
1
0
−1
−2
−3
−4
male
female

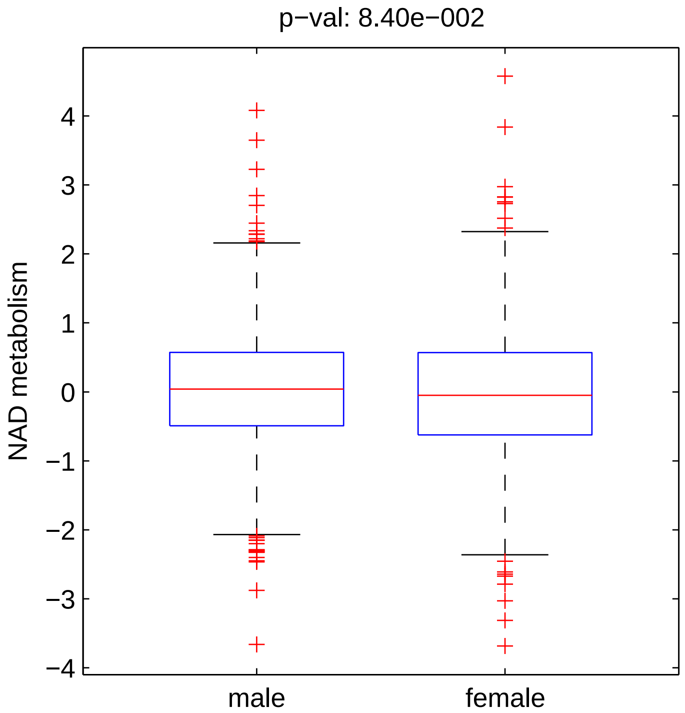
p−val: 8.40e−002
NAD metabolism
4
3
2
1
0
−1
−2
−3
−4
male
female

SHIP metabolite boxplots

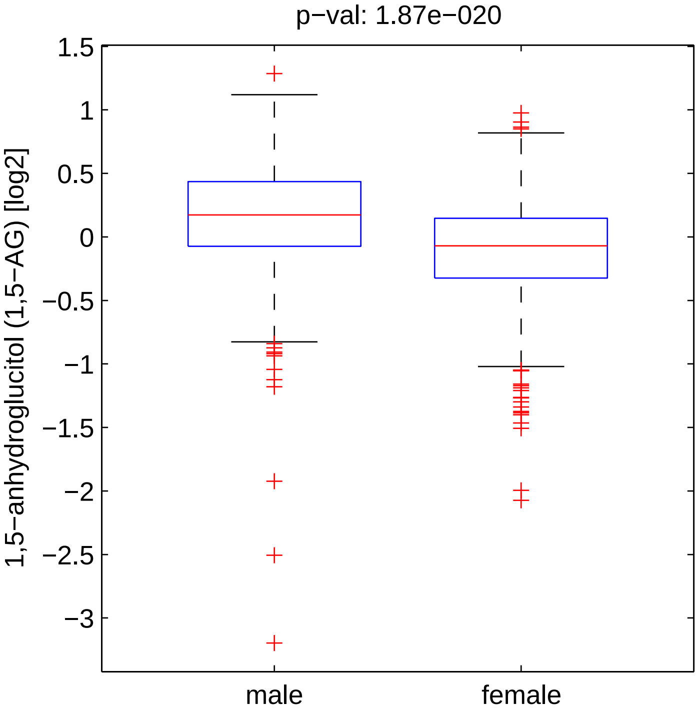
p−val: 1.87e−020
1.5
1
0.5
0
−0.5
−1
−1.5
−2
−2.5
−3
1,5−anhydroglucitol (1,5−AG) [log2]
male
female

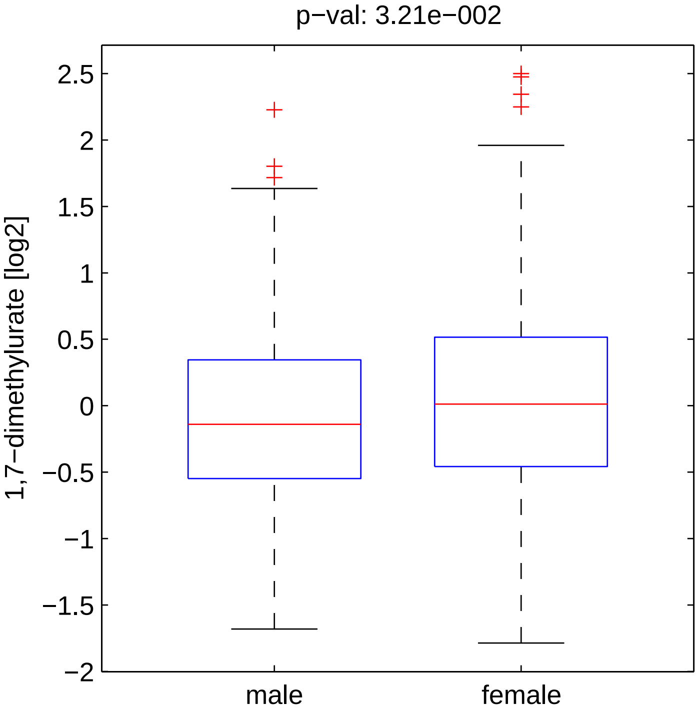

p−val: 3.21e−002
1,7−dimethylurate [log2]
2.5
2
1.5
1
0.5
0
−0.5
−1
−1.5
−2
male
female

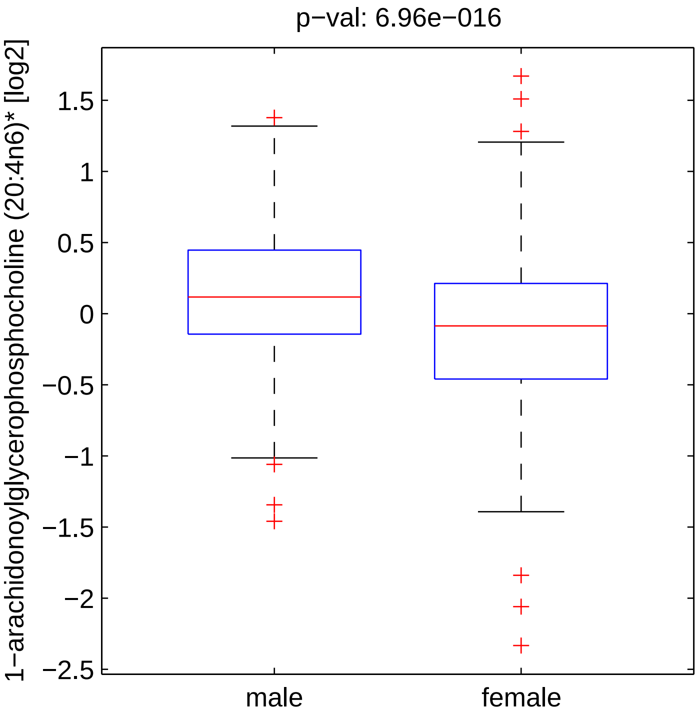
p−val: 6.96e−016
1−arachidonoylglycerophosphocholine (20:4n6)* [log2]
1.5
1
0.5
0
−0.5
−1
−1.5
−2
−2.5
male
female

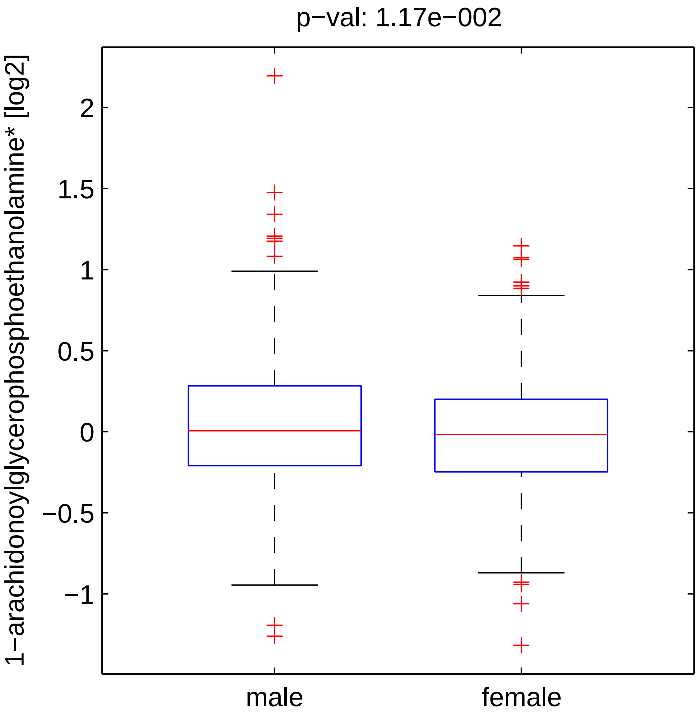
p−val: 1.17e−002
1−arachidonoylglycerophosphoethanolamine* [log2]
2
1.5
1
0.5
0
−0.5
−1
male
female

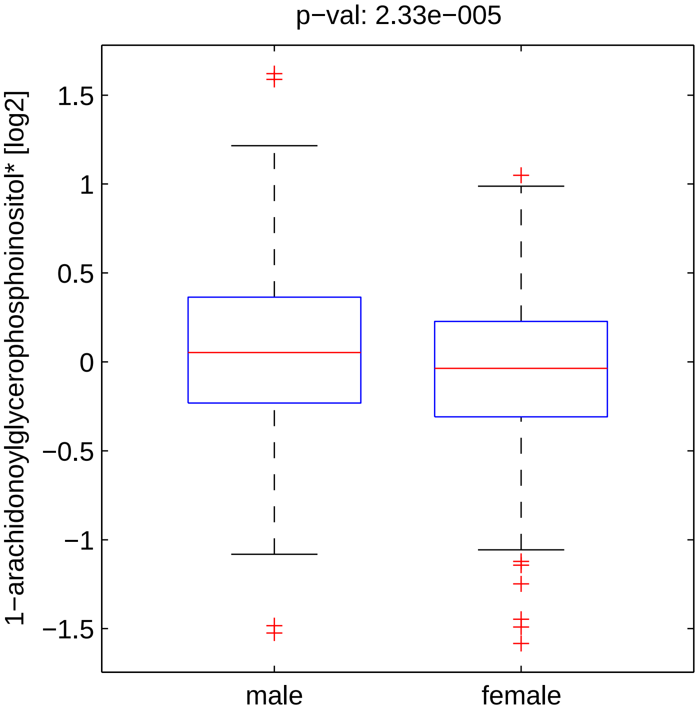
p−val: 2.33e−005
1−arachidonoylglycerophosphoinositol* [log2]
1.5
1
0.5
0
−0.5
−1
−1.5
male
female

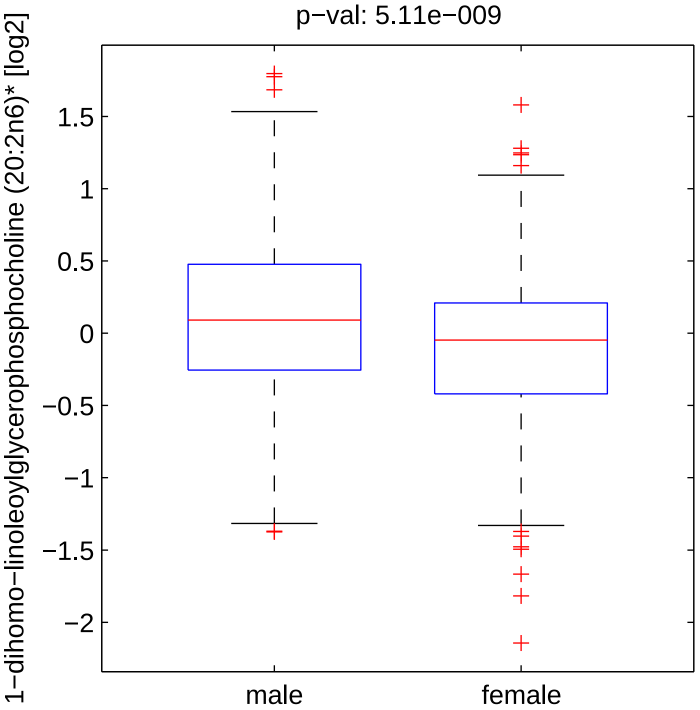

p−val: 5.11e−009
1−dihomo−linoleoylglycerophosphocholine (20:2n6)* [log2]
1.5
1
0.5
0
−0.5
−1
−1.5
−2
male
female

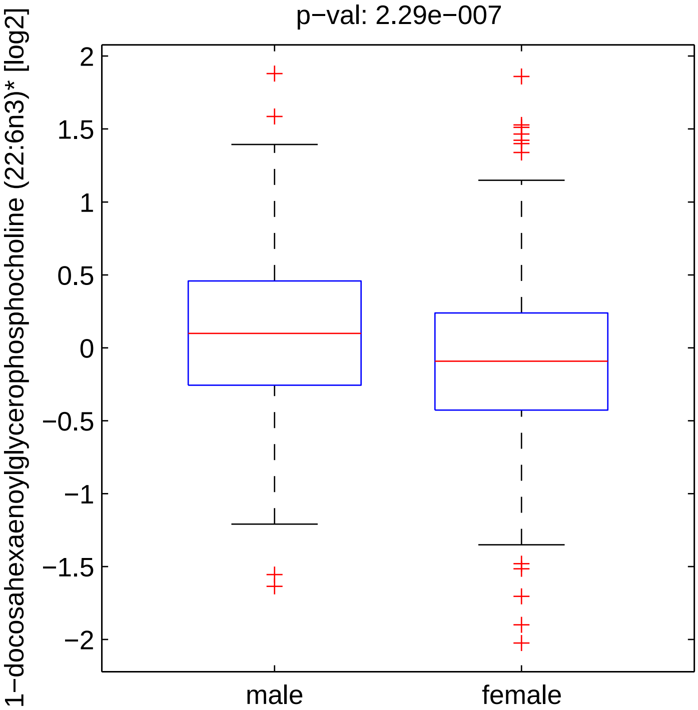

p−val: 2.29e−007
1−docosahexaenoylglycerophosphocholine (22:6n3)* [log2]
2
1.5
1
0.5
0
−0.5
−1
−1.5
−2
male
female

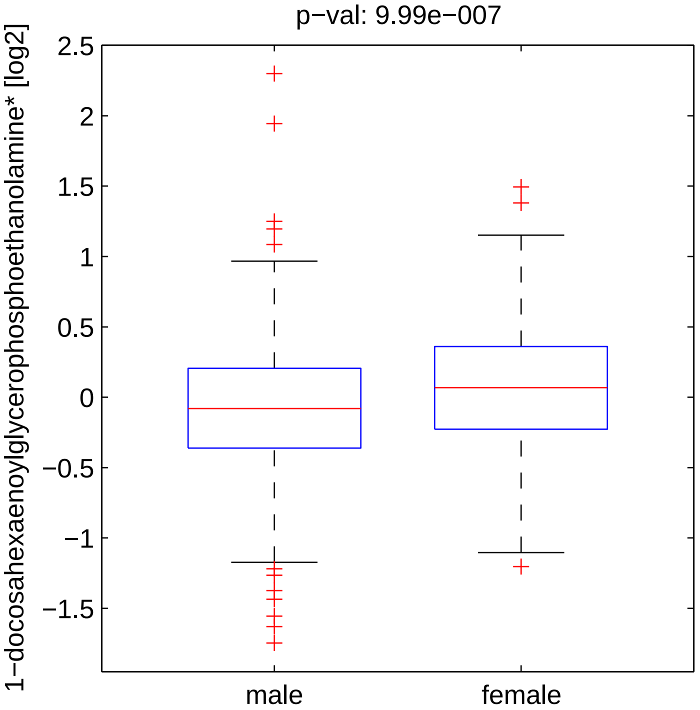

p−val: 9.99e−007
1−docosahexaenoylglycerophosphoethanolamine* [log2]
2.5
2
1.5
1
0.5
0
−0.5
−1
−1.5
male
female

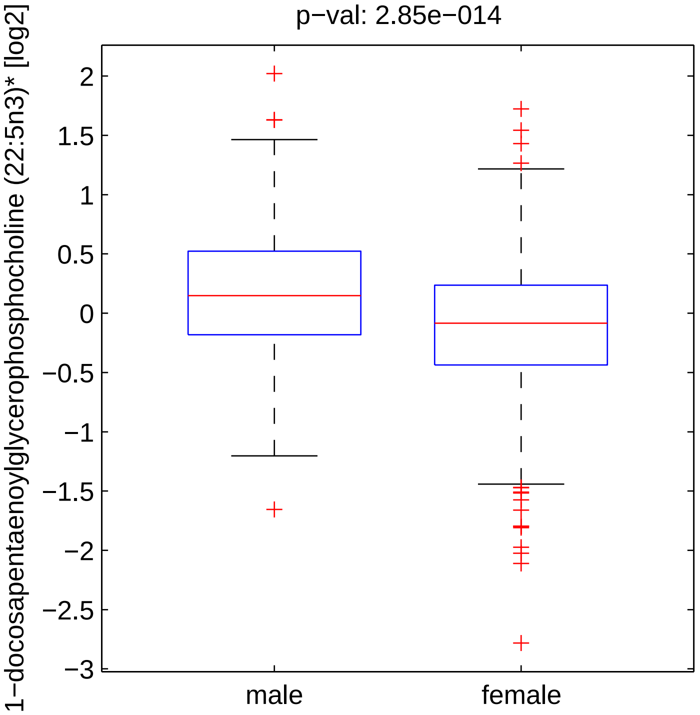

p−val: 2.85e−014
1−docosapentaenoylglycerophosphocholine (22:5n3)* [log2]
2
1.5
1
0.5
0
−0.5
−1
−1.5
−2
−2.5
−3
male
female

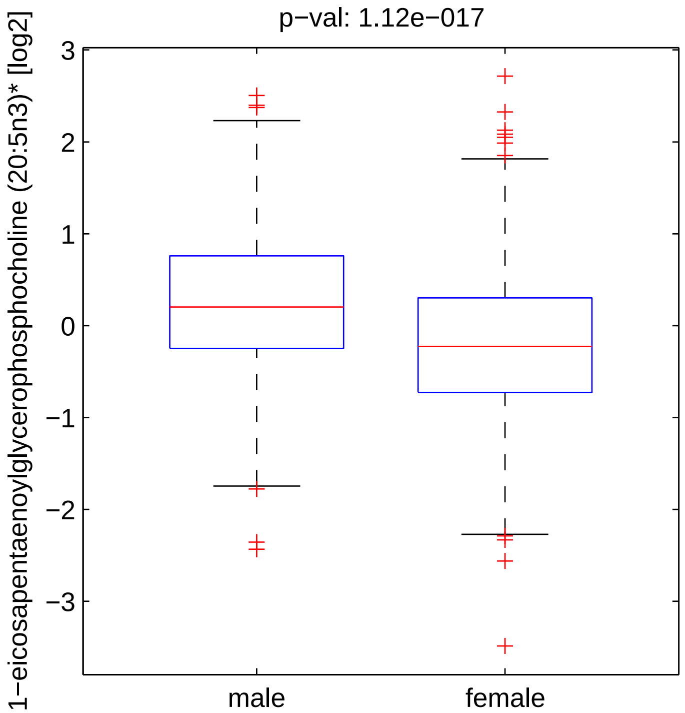

p−val: 1.12e−017
1−eicosapentaenoylglycerophosphocholine (20:5n3)* [log2]
3
2
1
0
−1
−2
−3
male
female

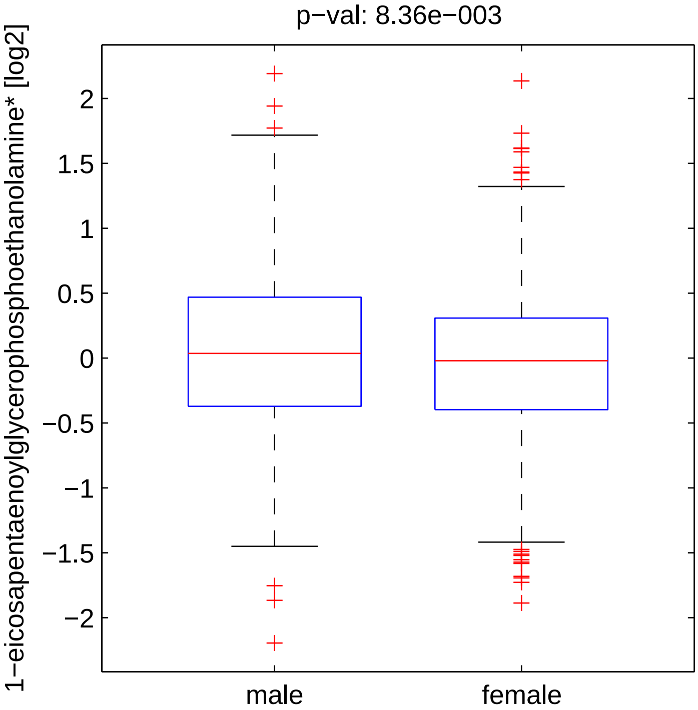
p−val: 8.36e−003
1−eicosapentaenoylglycerophosphoethanolamine* [log2]
2
1.5
1
0.5
0
−0.5
−1
−1.5
−2
male
female

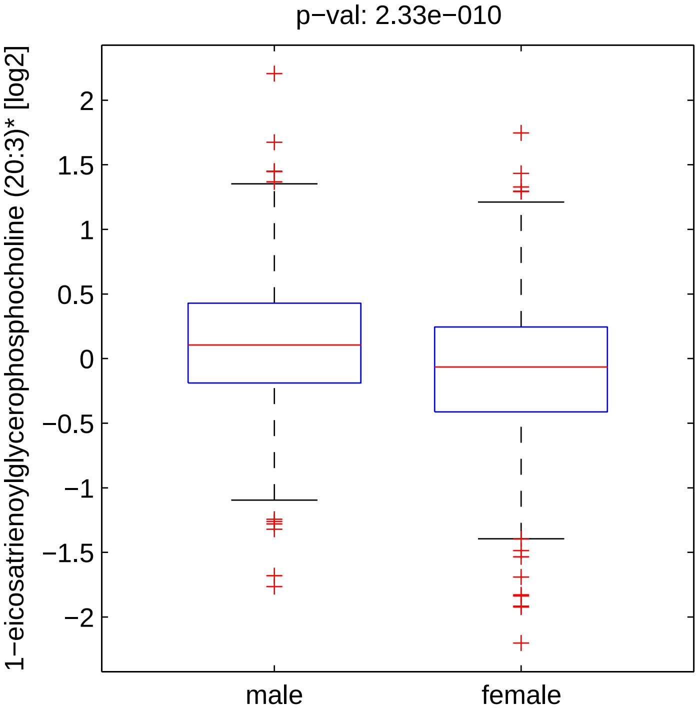

p−val: 2.33e−010
1−eicosatrienoylglycerophosphocholine (20:3)* [log2]
2
1.5
1
0.5
0
−0.5
−1
−1.5
−2
male
female

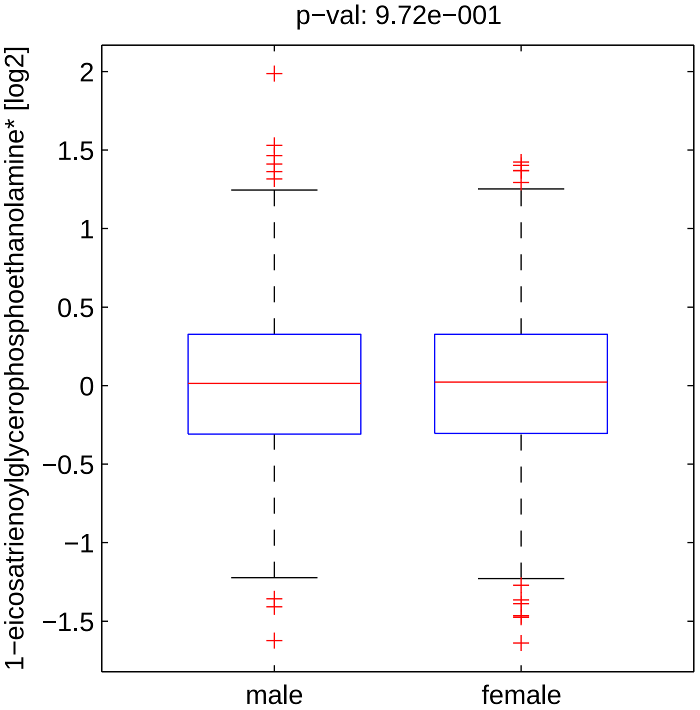
p−val: 9.72e−001
1−eicosatrienoylglycerophosphoethanolamine* [log2]
2
1.5
1
0.5
0
−0.5
−1
−1.5
male
female

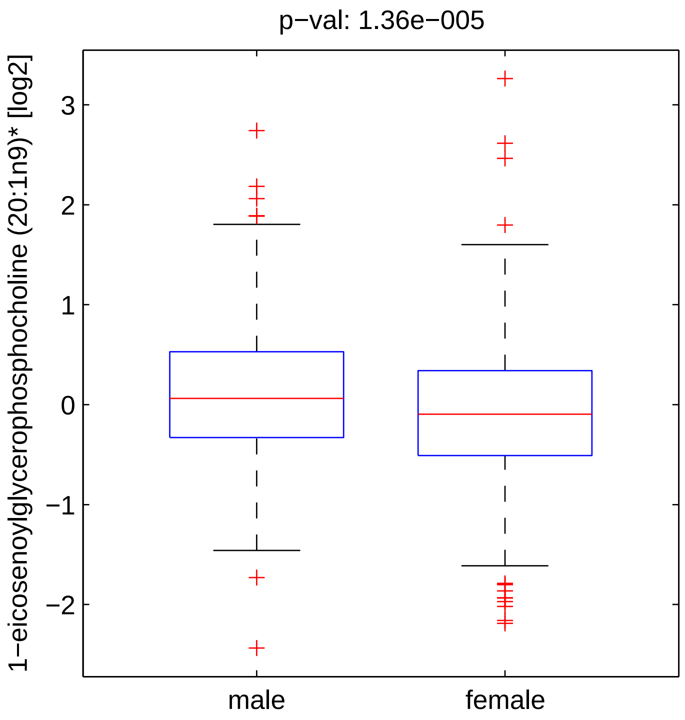

p−val: 1.36e−005
1−eicosenoylglycerophosphocholine (20:1n9)* [log2]
3
2
1
0
−1
−2
male
female

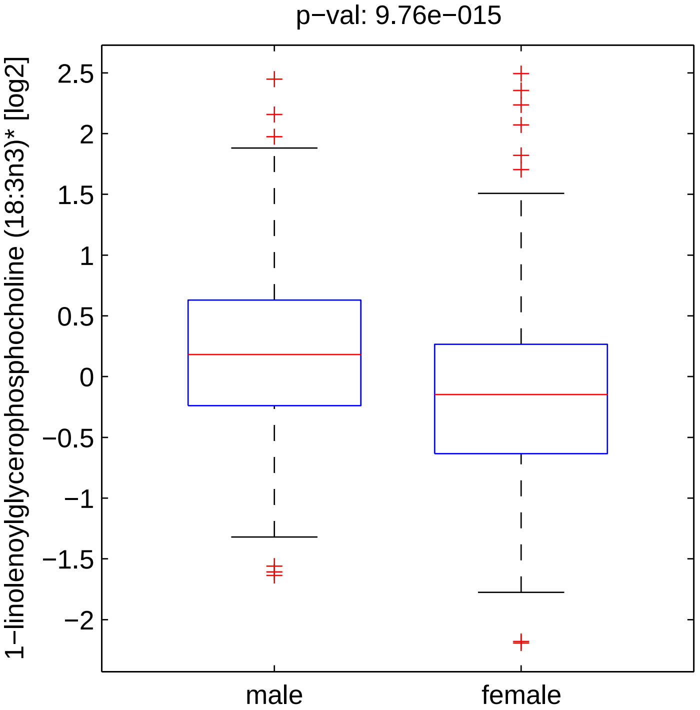
p−val: 9.76e−015
1−linolenoylglycerophosphocholine (18:3n3)* [log2]
2.5
2
1.5
1
0.5
0
−0.5
−1
−1.5
−2
male
female

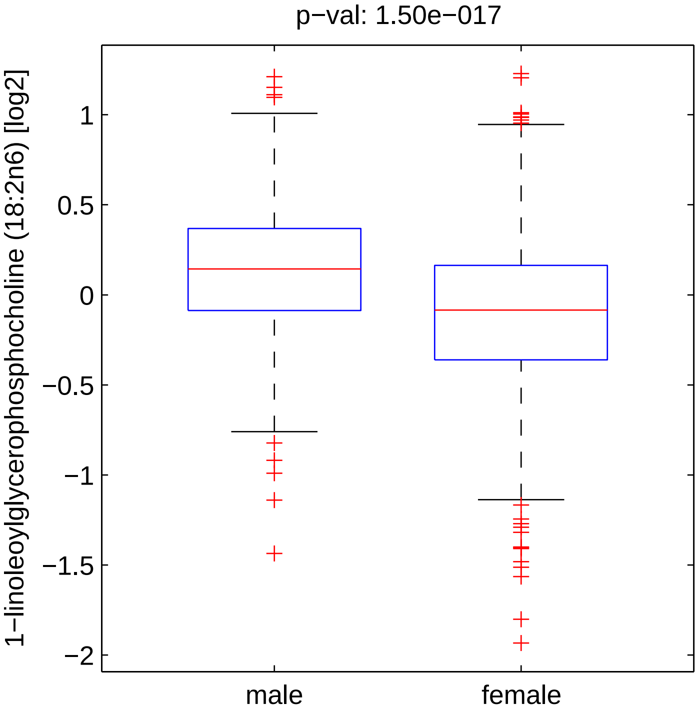
p−val: 1.50e−017
1−linoleoylglycerophosphocholine (18:2n6) [log2]
1
0.5
0
−0.5
−1
−1.5
−2
male
female

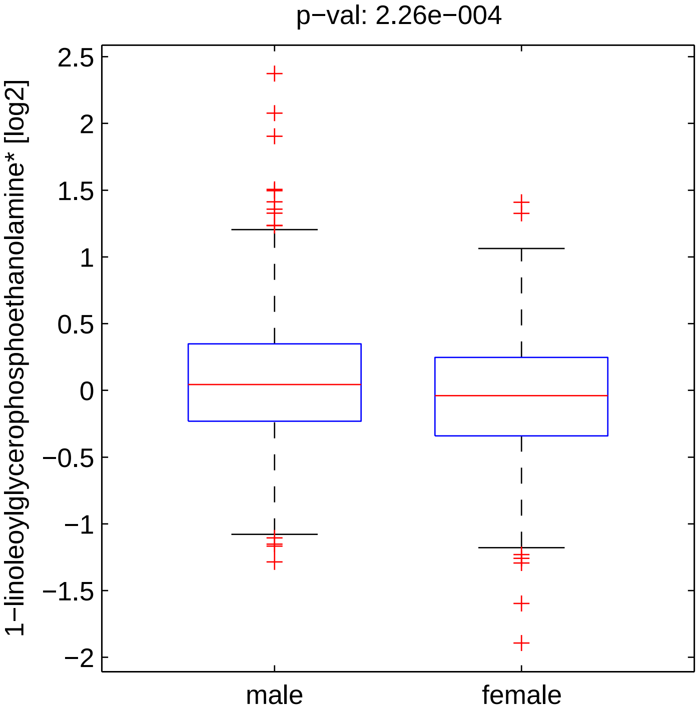
p−val: 2.26e−004
1−linoleoylglycerophosphoethanolamine* [log2]
2.5
2
1.5
1
0.5
0
−0.5
−1
−1.5
−2
male
female

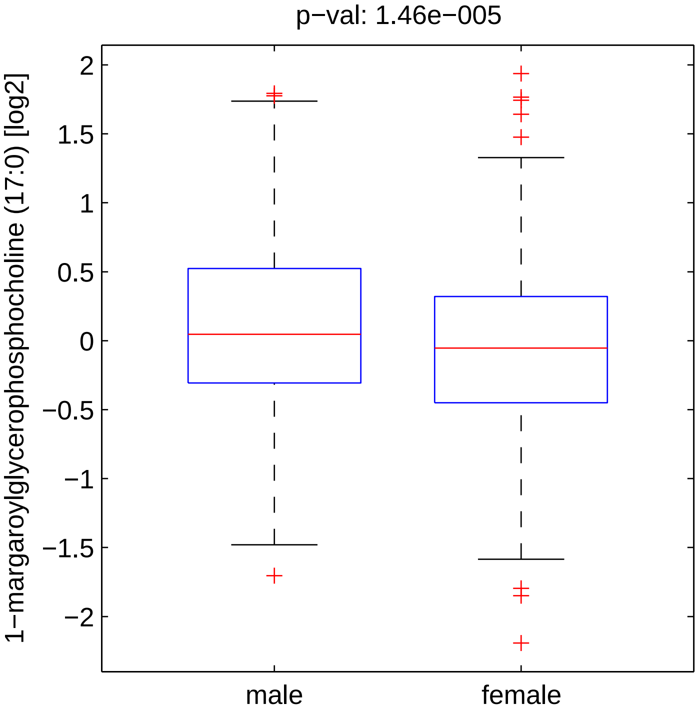

p−val: 1.46e−005
1−margaroylglycerophosphocholine (17:0) [log2]
2
1.5
1
0.5
0
−0.5
−1
−1.5
−2
male
female

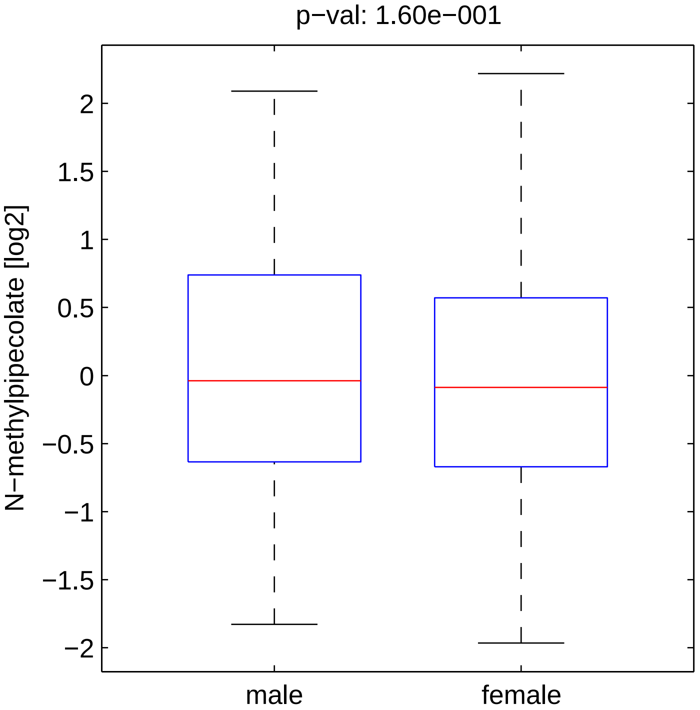
p−val: 1.60e−001
N−methylpipecolate [log2]
2
1.5
1
0.5
0
−0.5
−1
−1.5
−2
male
female

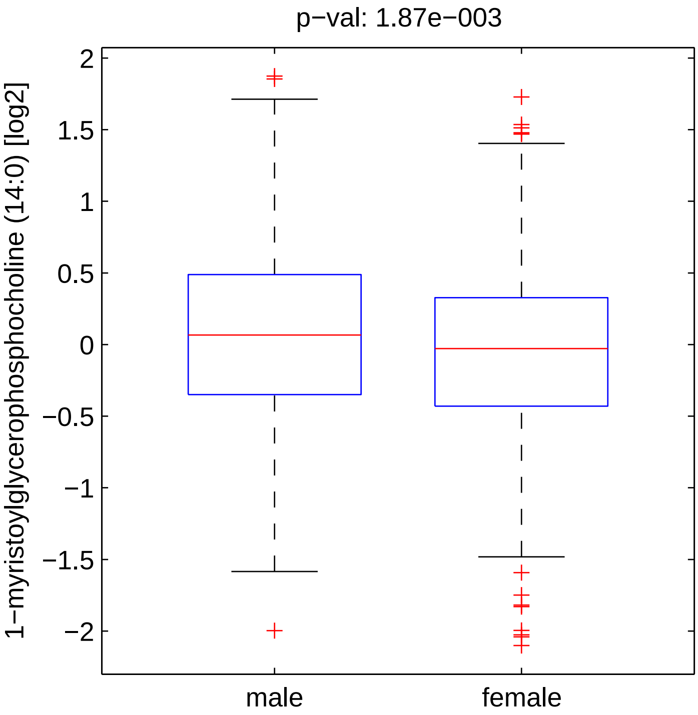
p−val: 1.87e−003
1−myristoylglycerophosphocholine (14:0) [log2]
2
1.5
1
0.5
0
−0.5
−1
−1.5
−2
male
female

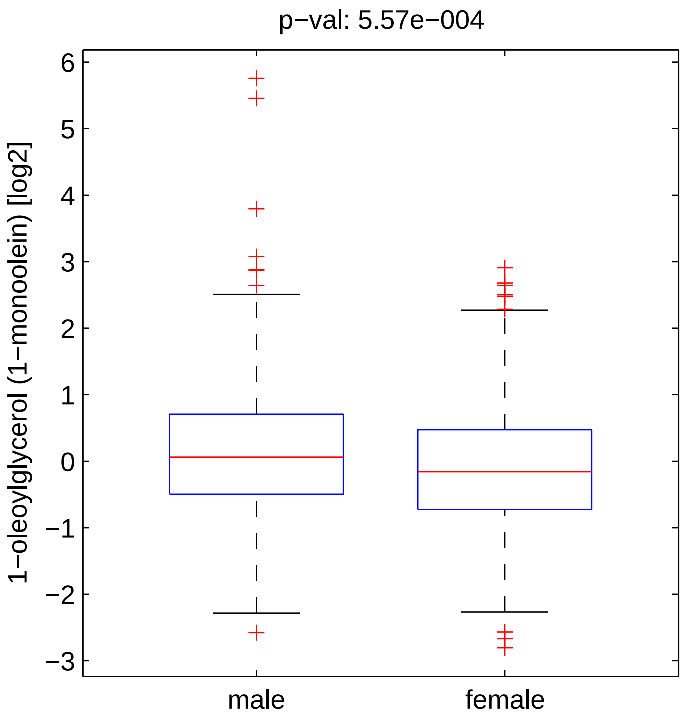
p−val: 5.57e−004
1−oleoylglycerol (1−monoolein) [log2]
6
5
4
3
2
1
0
−1
−2
−3
male
female

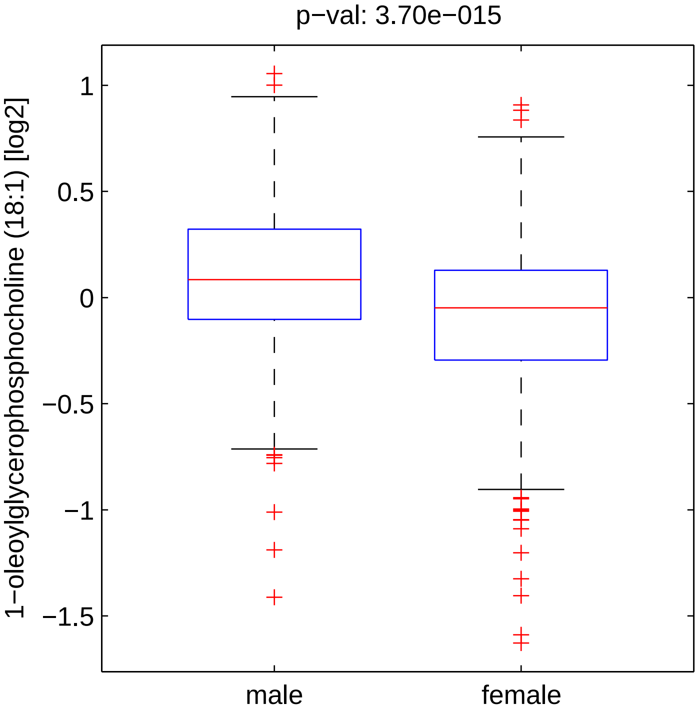
p−val: 3.70e−015
1−oleoylglycerophosphocholine (18:1) [log2]
1
0.5
0
−0.5
−1
−1.5
male
female

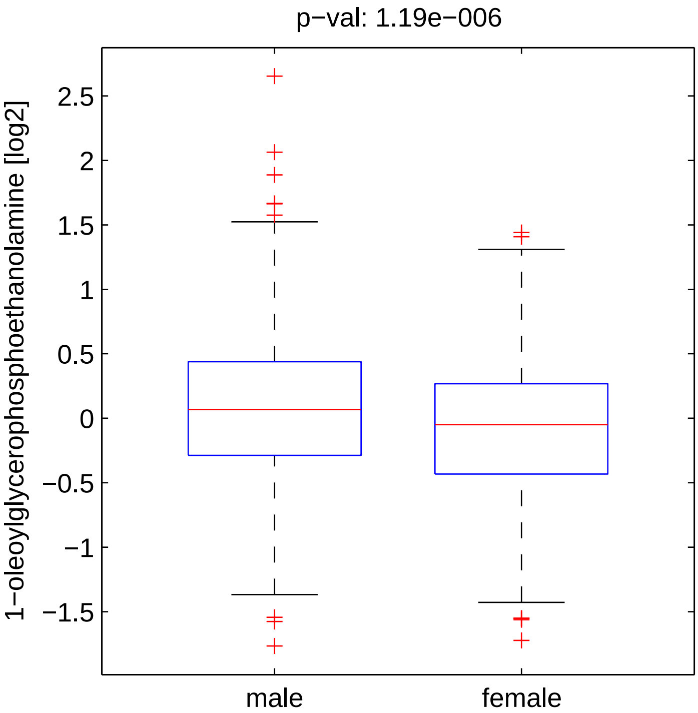
p−val: 1.19e−006
1−oleoylglycerophosphoethanolamine [log2]
2.5
2
1.5
1
0.5
0
−0.5
−1
−1.5
male
female

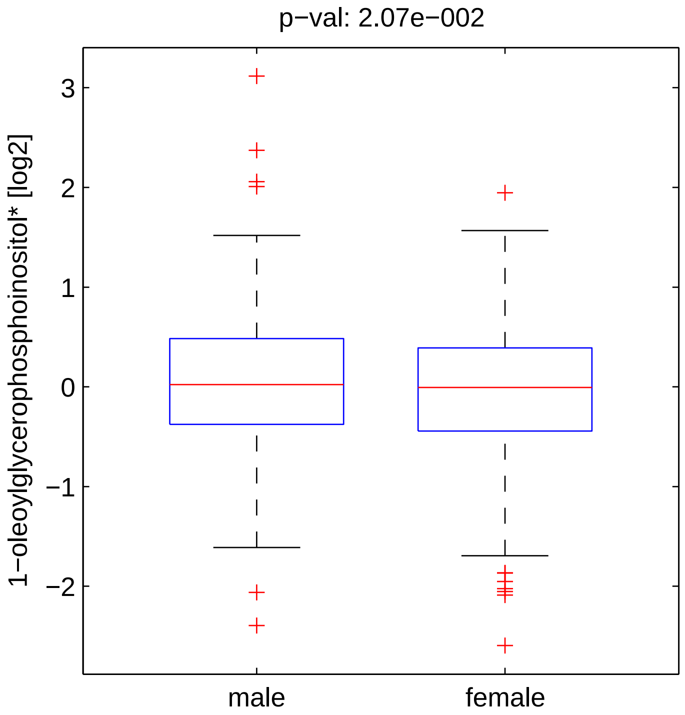
p−val: 2.07e−002
1−oleoylglycerophosphoinositol* [log2]
3
2
1
0
−1
−2
male
female

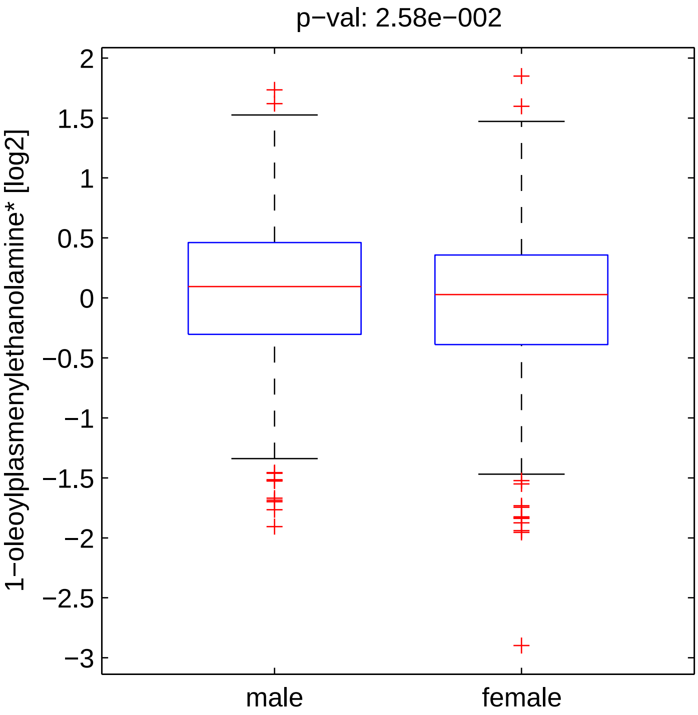

p−val: 2.58e−002
1−oleoylplasmenylethanolamine* [log2]
2
1.5
1
0.5
0
−0.5
−1
−1.5
−2
−2.5
−3
male
female

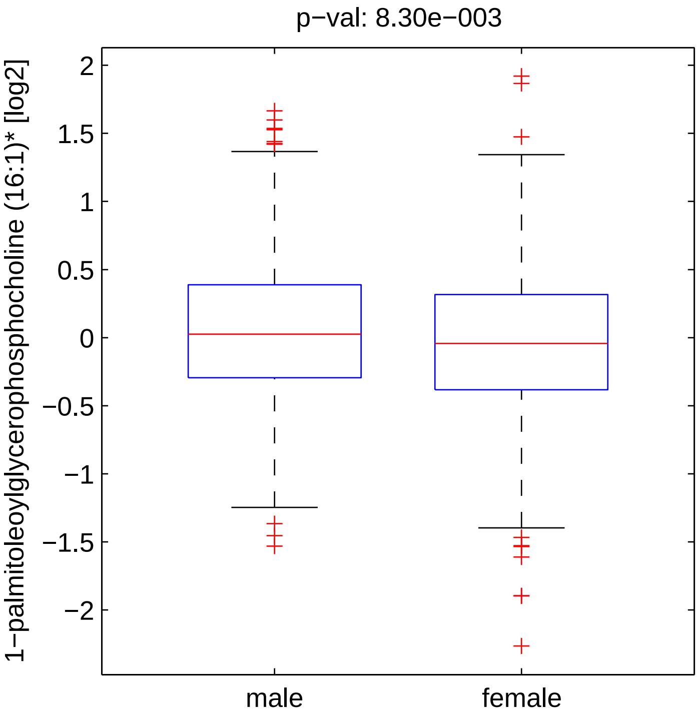
p−val: 8.30e−003
1−palmitoleoylglycerophosphocholine (16:1)* [log2]
2
1.5
1
0.5
0
−0.5
−1
−1.5
−2
male
female

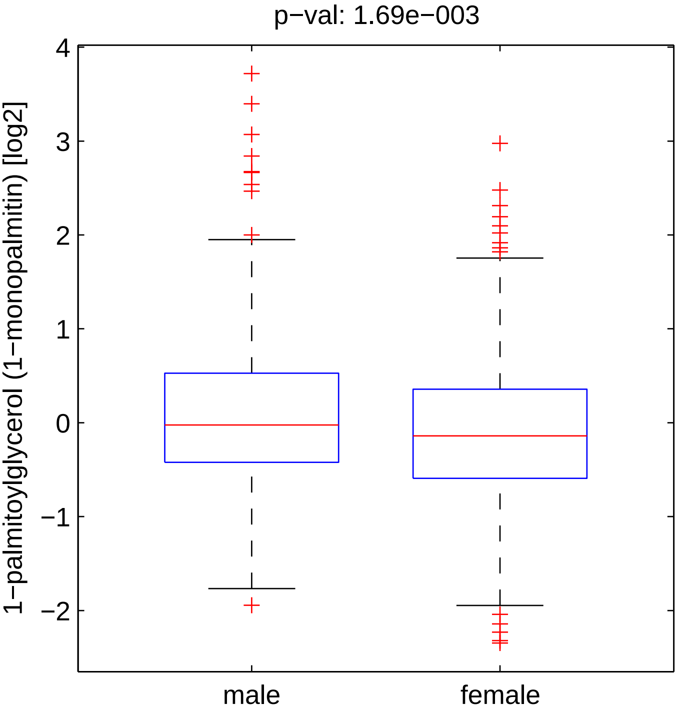
p−val: 1.69e−003
1−palmitoylglycerol (1−monopalmitin) [log2]
4
3
2
1
0
−1
−2
male
female

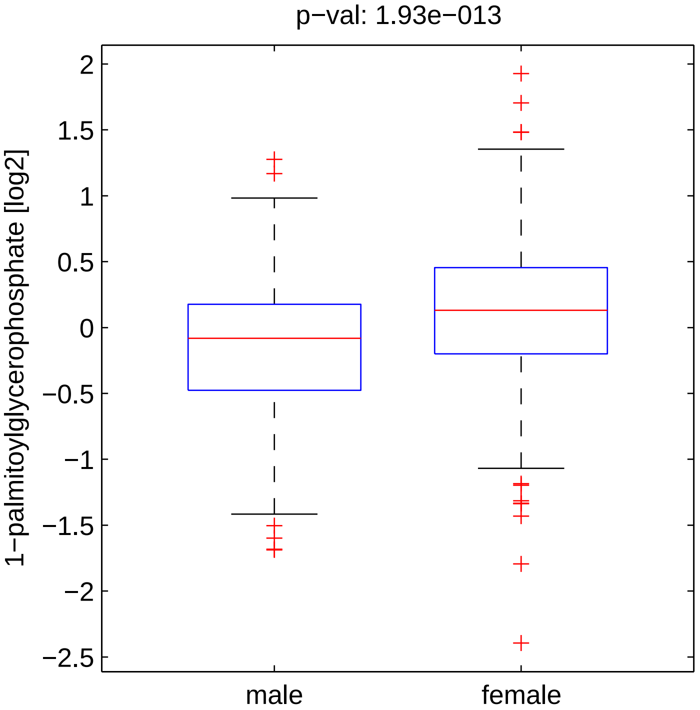
p−val: 1.93e−013
1−palmitoylglycerophosphate [log2]
2
1.5
1
0.5
0
−0.5
−1
−1.5
−2
−2.5
male
female

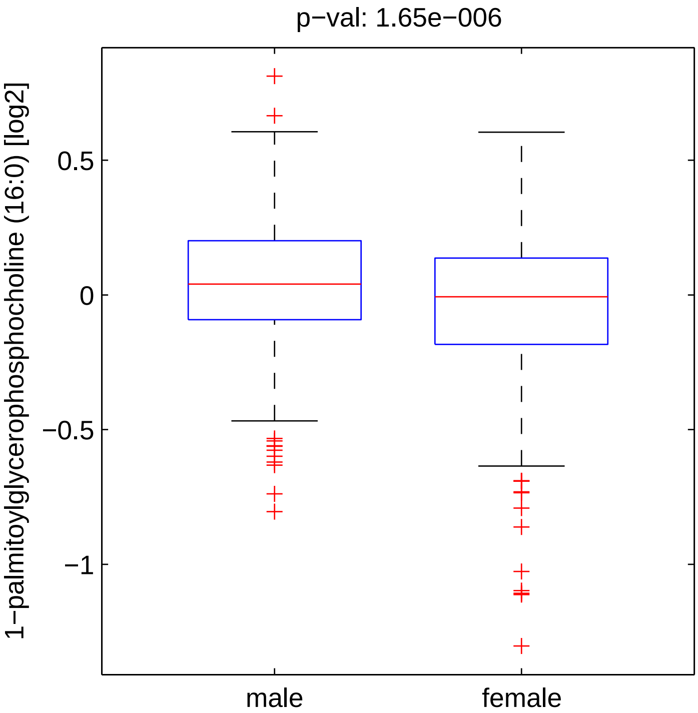

p−val: 1.65e−006
1−palmitoylglycerophosphocholine (16:0) [log2]
0.5
0
−0.5
−1
male
female

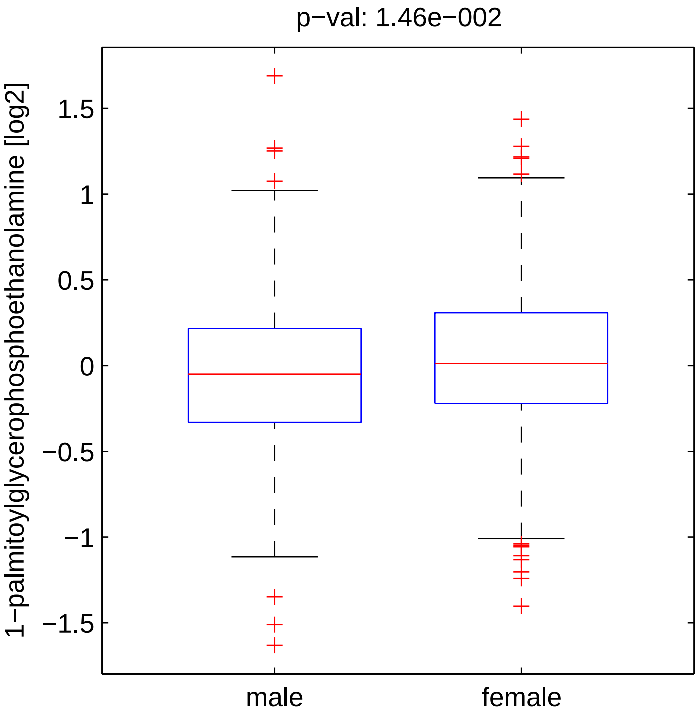
p−val: 1.46e−002
1−palmitoylglycerophosphoethanolamine [log2]
1.5
1
0.5
0
−0.5
−1
−1.5
male
female

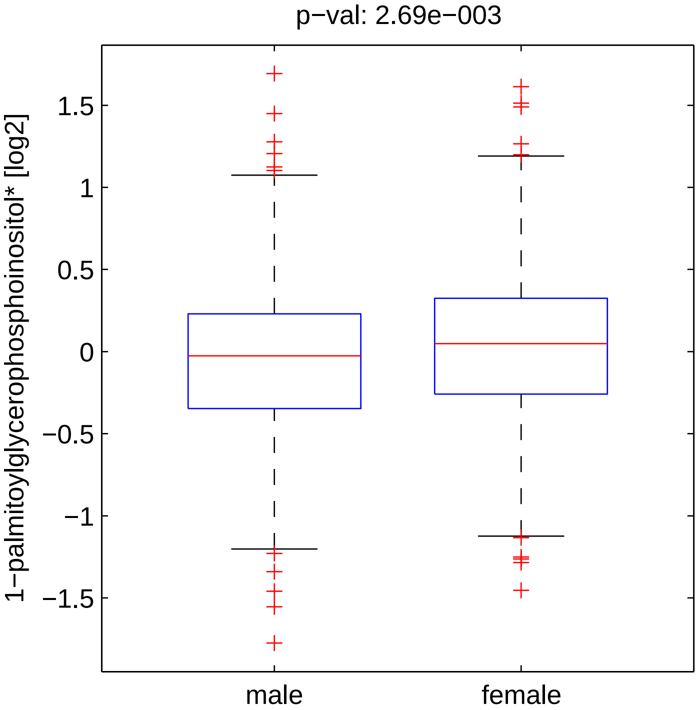

p−val: 2.69e−003
1−palmitoylglycerophosphoinositol* [log2]
1.5
1
0.5
0
−0.5
−1
−1.5
male
female

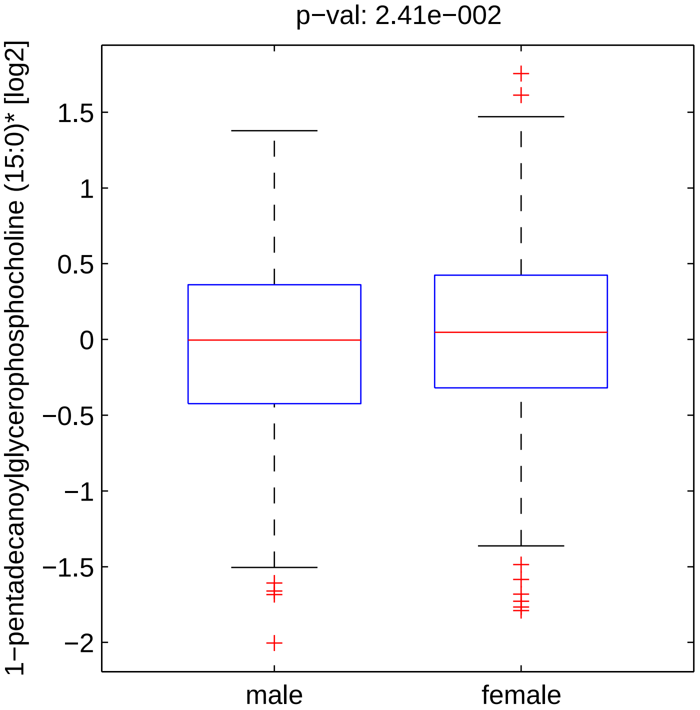

p−val: 2.41e−002
1−pentadecanoylglycerophosphocholine (15:0)* [log2]
1.5
1
0.5
0
−0.5
−1
−1.5
−2
male
female

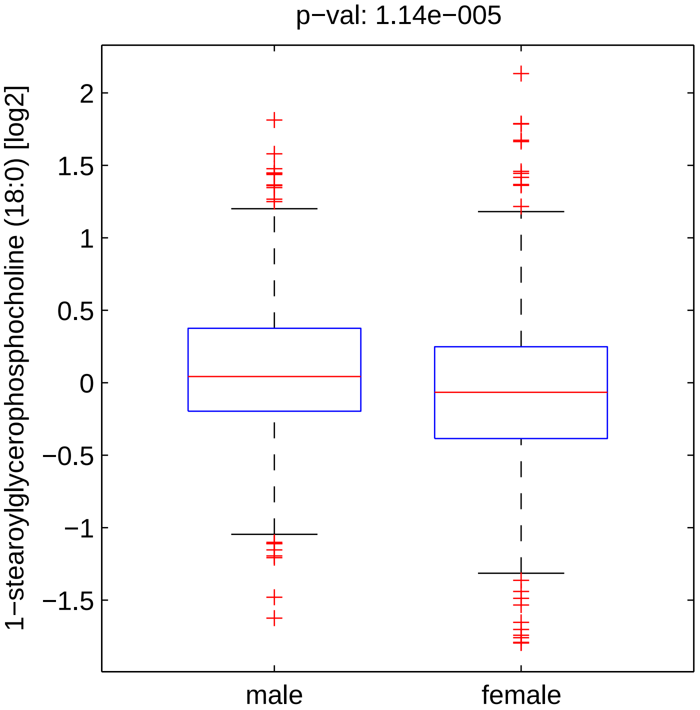

p−val: 1.14e−005
1−stearoylglycerophosphocholine (18:0) [log2]
2
1.5
1
0.5
0
−0.5
−1
−1.5
male
female

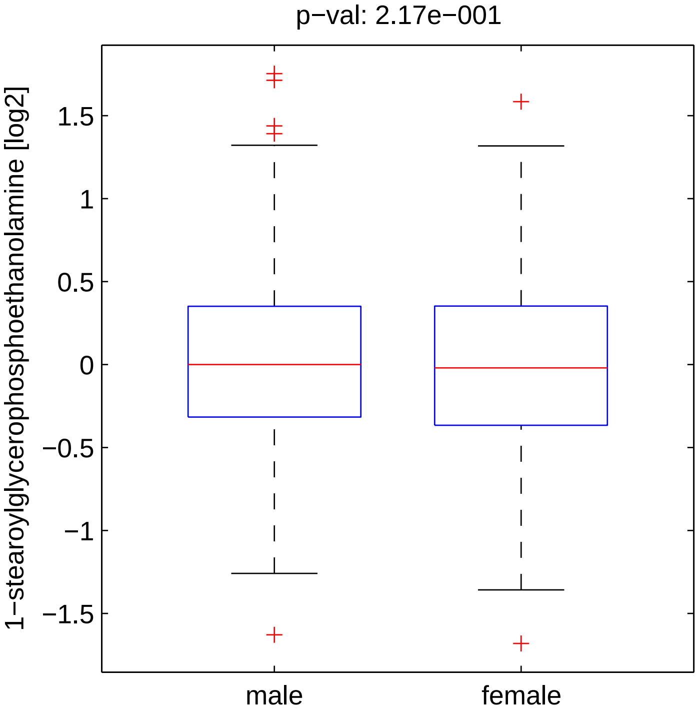
p−val: 2.17e−001
1−stearoylglycerophosphoethanolamine [log2]
1.5
1
0.5
0
−0.5
−1
−1.5
male
female

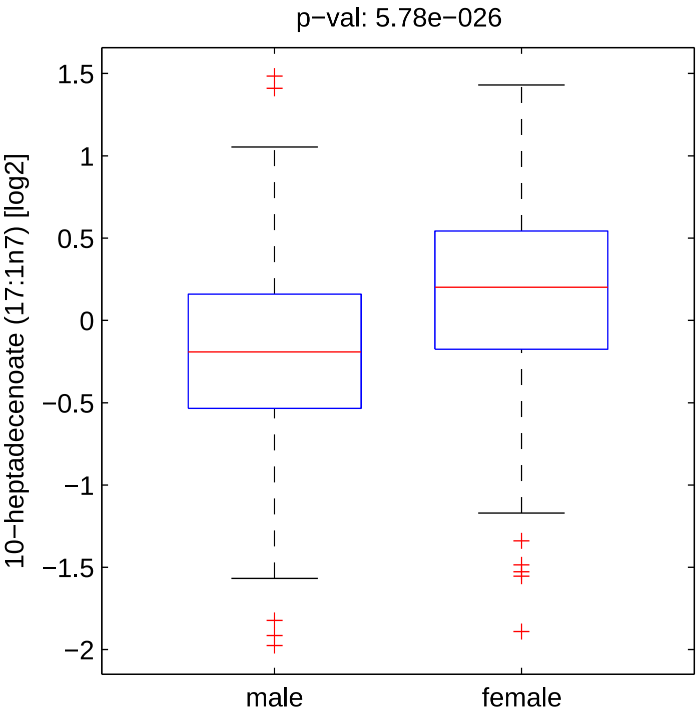

p−val: 5.78e−026
10−heptadecenoate (17:1n7) [log2]
1.5
1
0.5
0
−0.5
−1
−1.5
−2
male
female

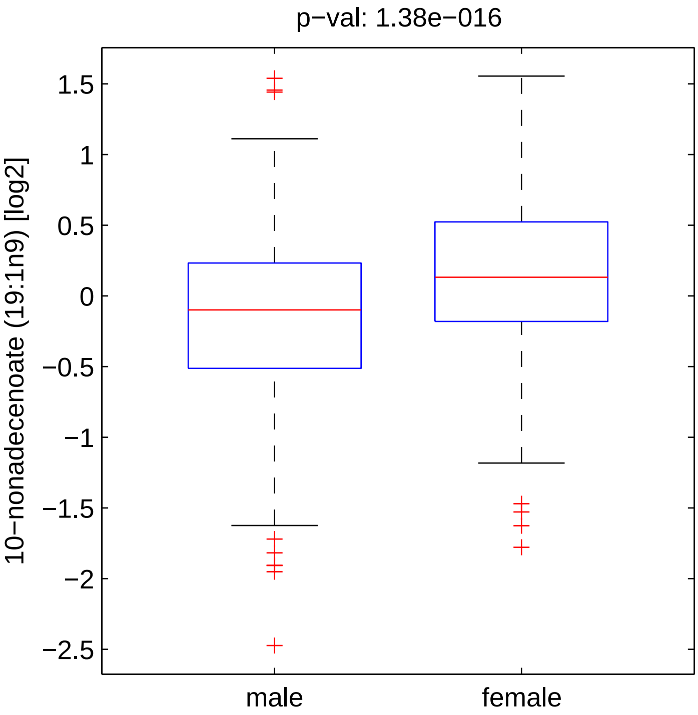
p−val: 1.38e−016
10−nonadecenoate (19:1n9) [log2]
1.5
1
0.5
0
−0.5
−1
−1.5
−2
−2.5
male
female

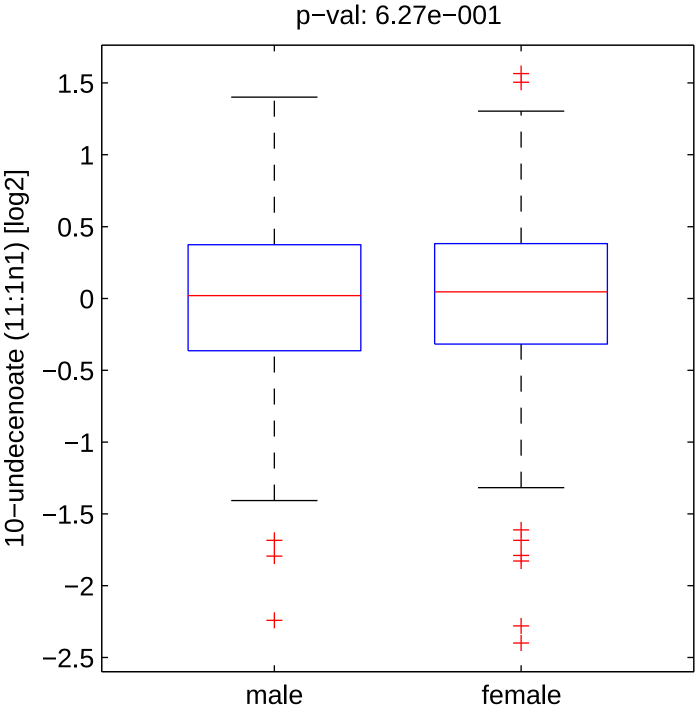
p−val: 6.27e−001
10−undecenoate (11:1n1) [log2]
1.5
1
0.5
0
−0.5
−1
−1.5
−2
−2.5
male
female

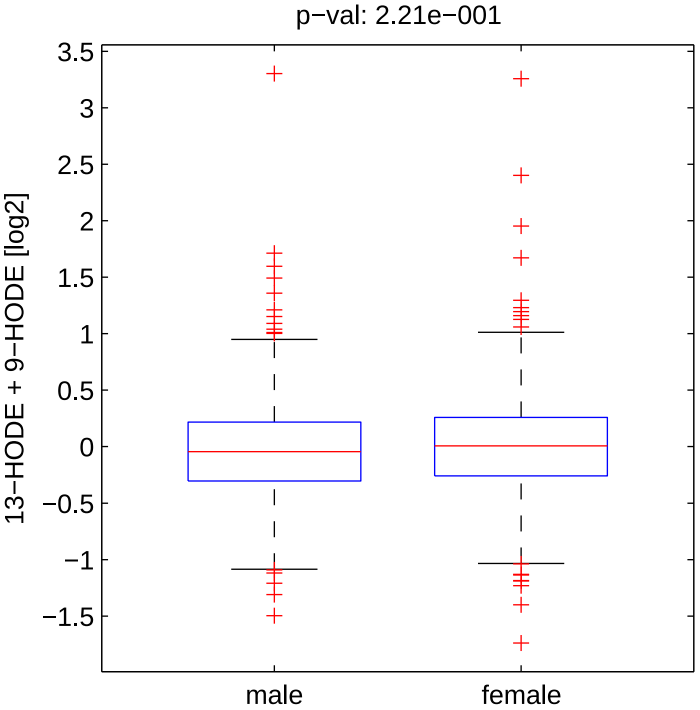
p−val: 2.21e−001
13−HODE + 9−HODE [log2]
3.5
3
2.5
2
1.5
1
0.5
0
−0.5
−1
−1.5
male
female

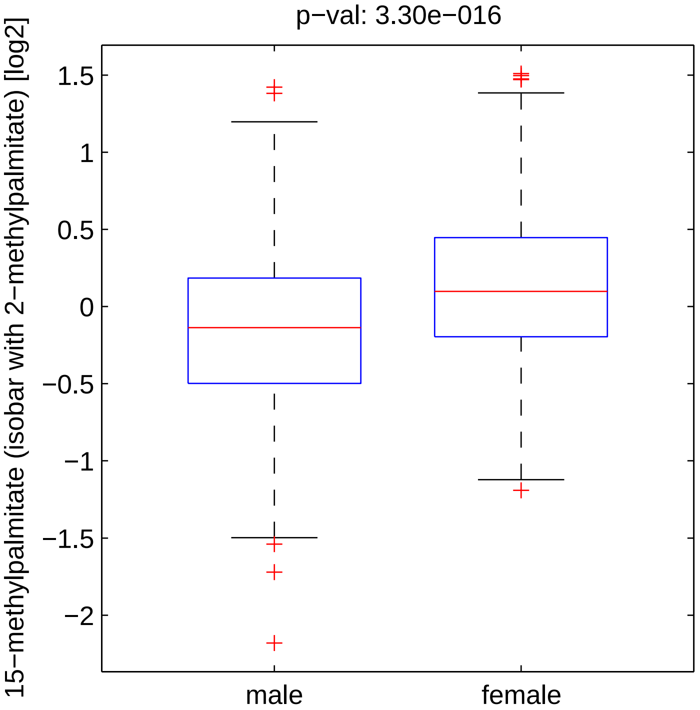
p−val: 3.30e−016
15−methylpalmitate (isobar with 2−methylpalmitate) [log2]
1.5
1
0.5
0
−0.5
−1
−1.5
−2
male
female

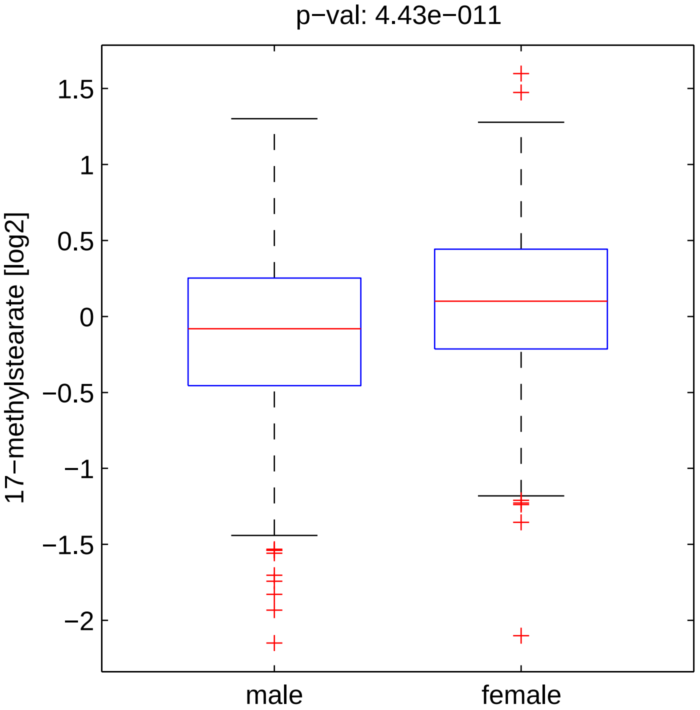
p−val: 4.43e−011
17−methylstearate [log2]
1.5
1
0.5
0
−0.5
−1
−1.5
−2
male
female

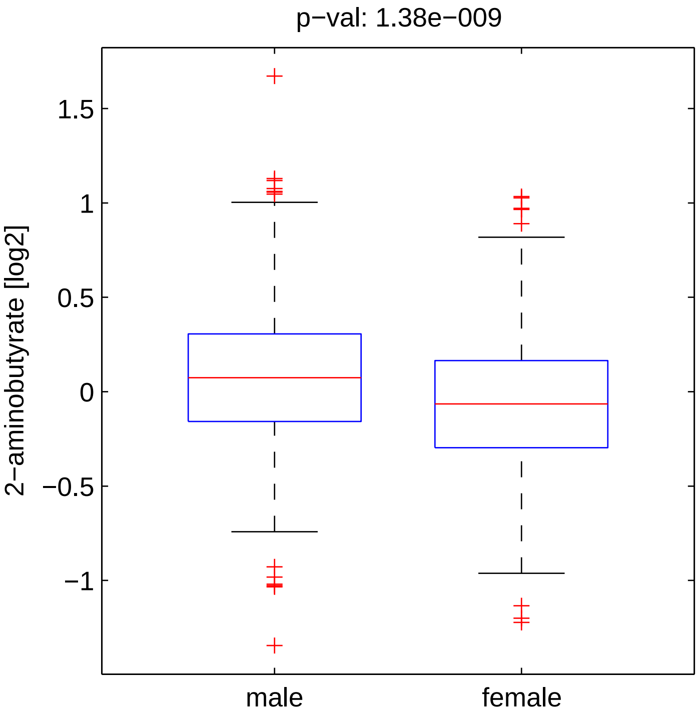
p−val: 1.38e−009
2−aminobutyrate [log2]
1.5
1
0.5
0
−0.5
−1
male
female

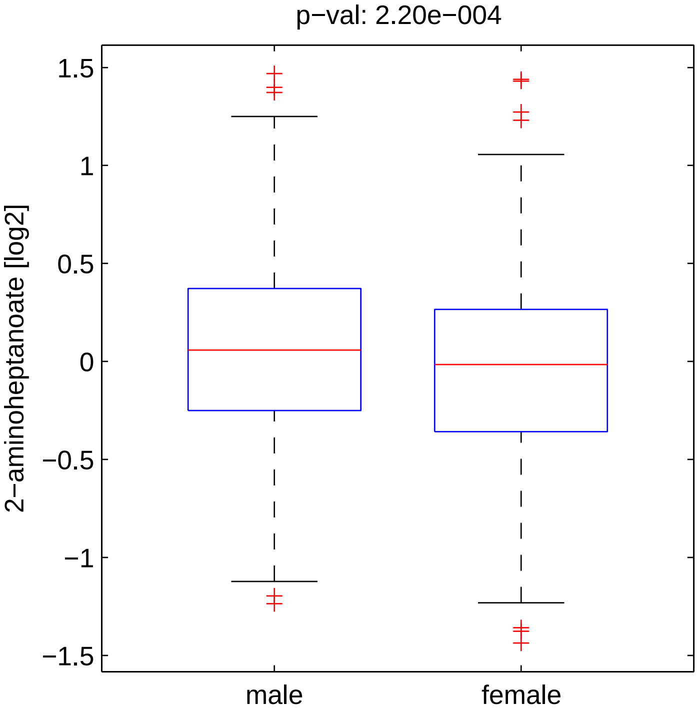
p−val: 2.20e−004
2−aminoheptanoate [log2]
1.5
1
0.5
0
−0.5
−1
−1.5
male
female

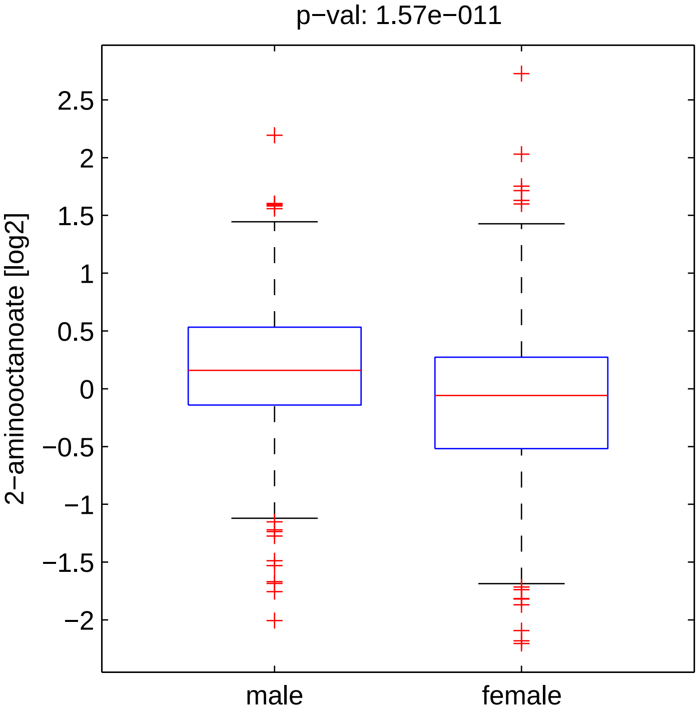
p−val: 1.57e−011
2−aminooctanoate [log2]
2.5
2
1.5
1
0.5
0
−0.5
−1
−1.5
−2
male
female

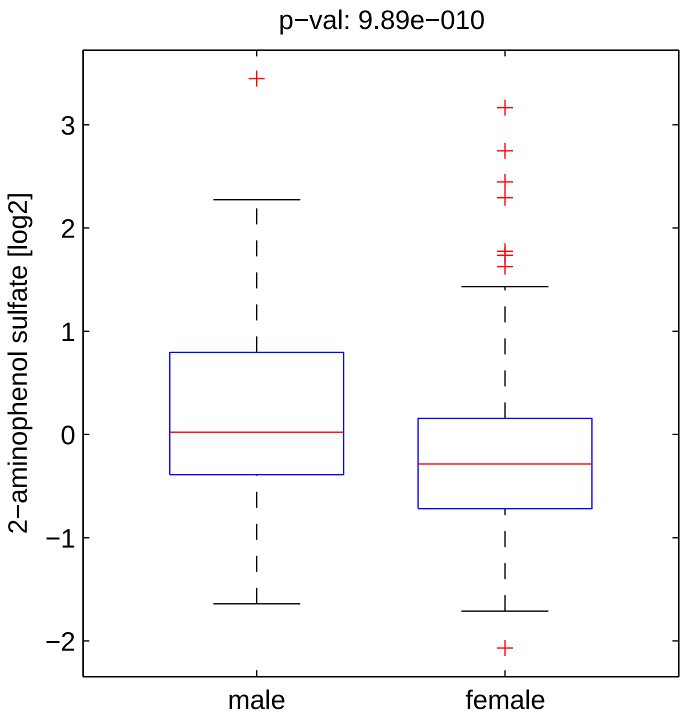
p−val: 9.89e−010
2−aminophenol sulfate [log2]
3
2
1
0
−1
−2
male
female

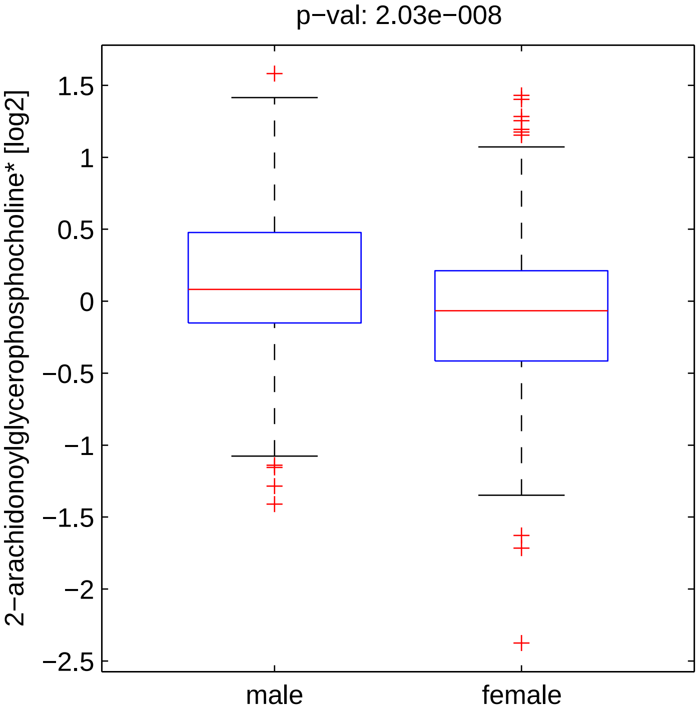
p−val: 2.03e−008
2−arachidonoylglycerophosphocholine* [log2]
1.5
1
0.5
0
−0.5
−1
−1.5
−2
−2.5
male
female

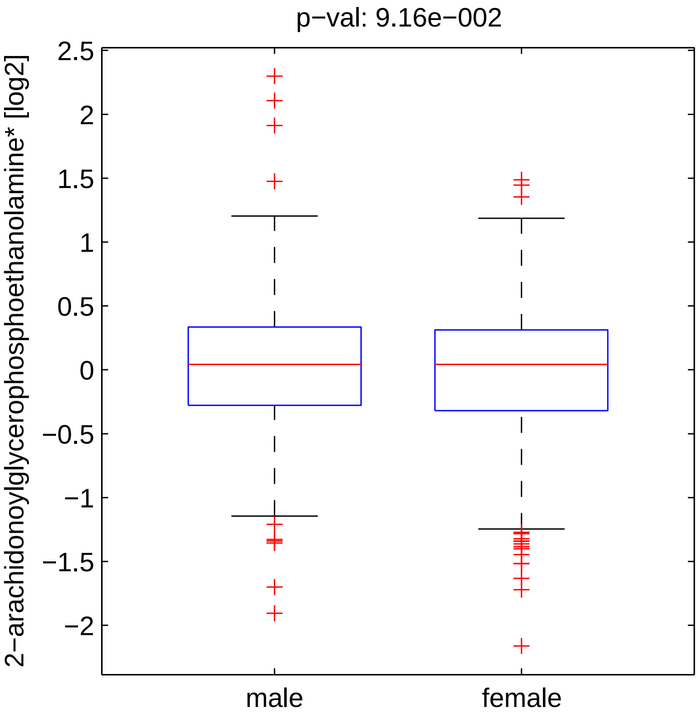

p−val: 9.16e−002
2−arachidonoylglycerophosphoethanolamine* [log2]
2.5
2
1.5
1
0.5
0
−0.5
−1
−1.5
−2
male
female

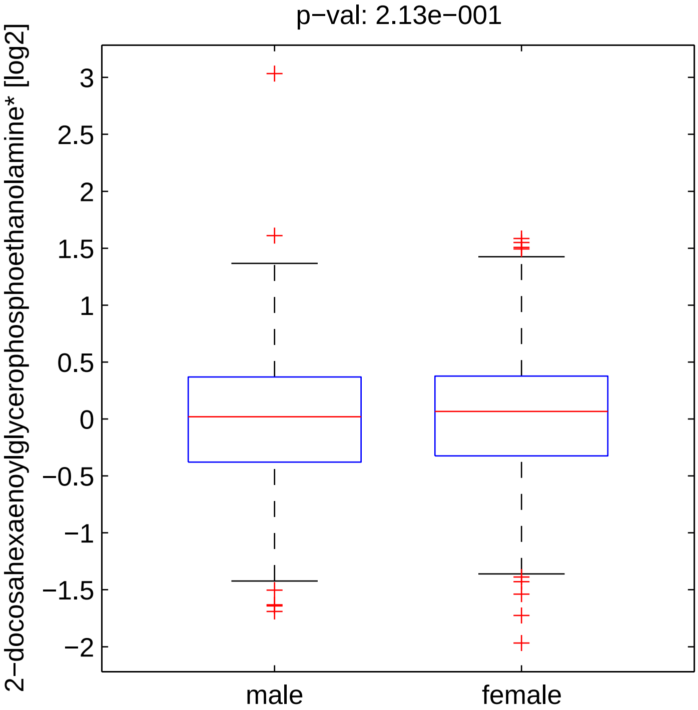

p−val: 2.13e−001
2−docosahexaenoylglycerophosphoethanolamine* [log2]
3
2.5
2
1.5
1
0.5
0
−0.5
−1
−1.5
−2
male
female

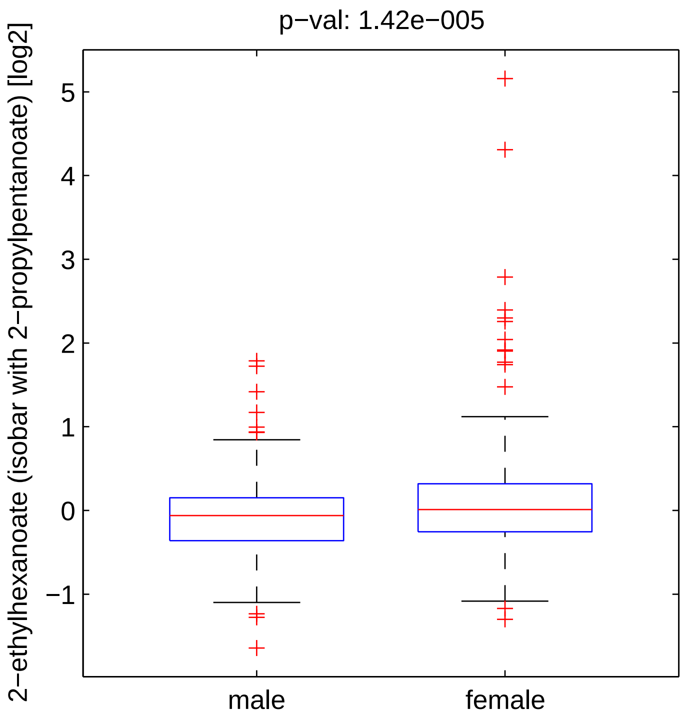
p−val: 1.42e−005
2−ethylhexanoate (isobar with 2−propylpentanoate) [log2]
5
4
3
2
1
0
−1
male
female

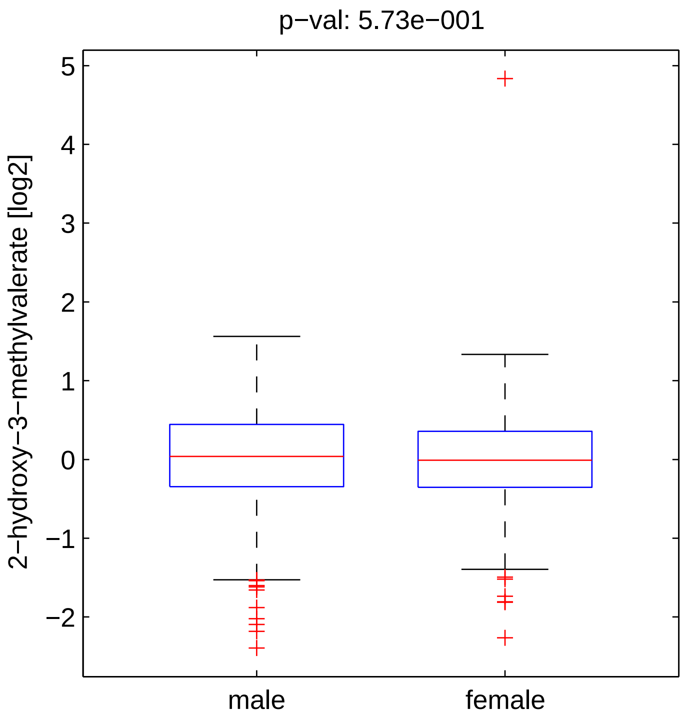

p−val: 5.73e−001
2−hydroxy−3−methylvalerate [log2]
5
4
3
2
1
0
−1
−2
male
female

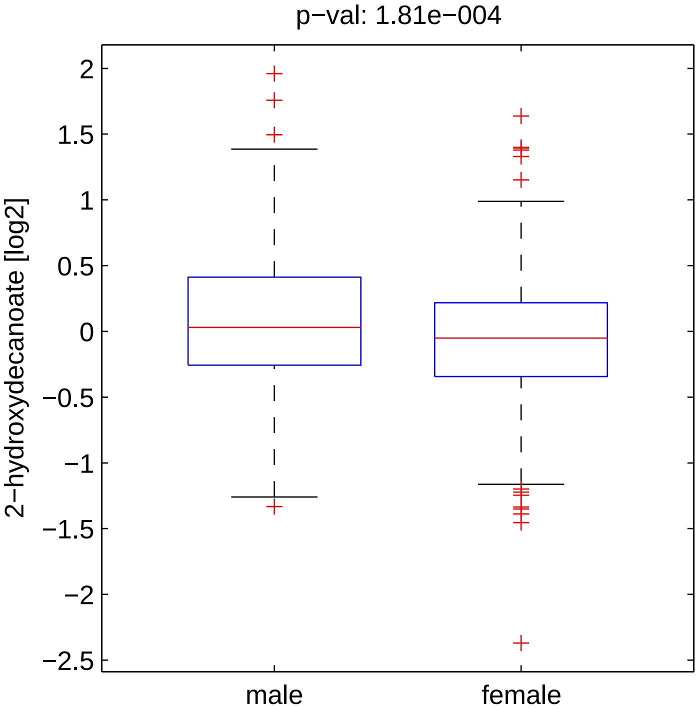

p−val: 1.81e−004
2−hydroxydecanoate [log2]
2
1.5
1
0.5
0
−0.5
−1
−1.5
−2
−2.5
male
female

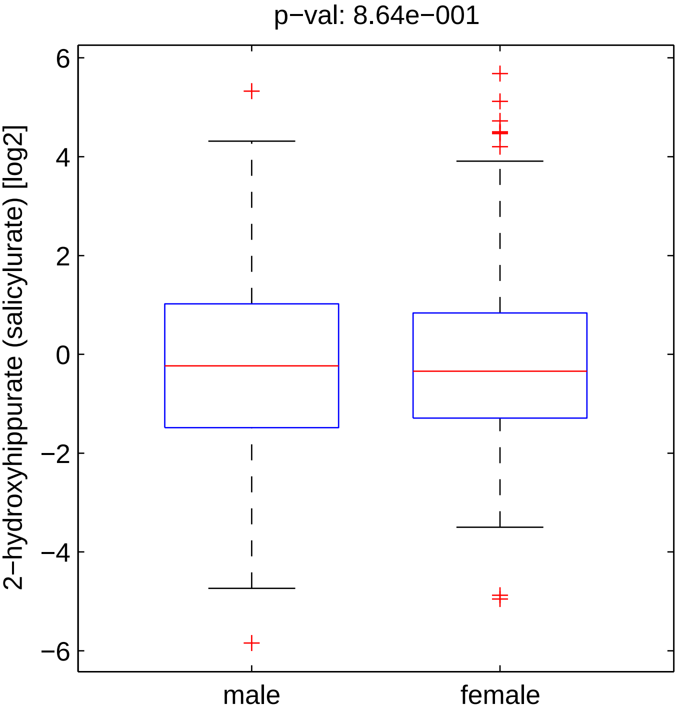

p−val: 8.64e−001
2−hydroxyhippurate (salicylurate) [log2]
6
4
2
0
−2
−4
−6
male
female

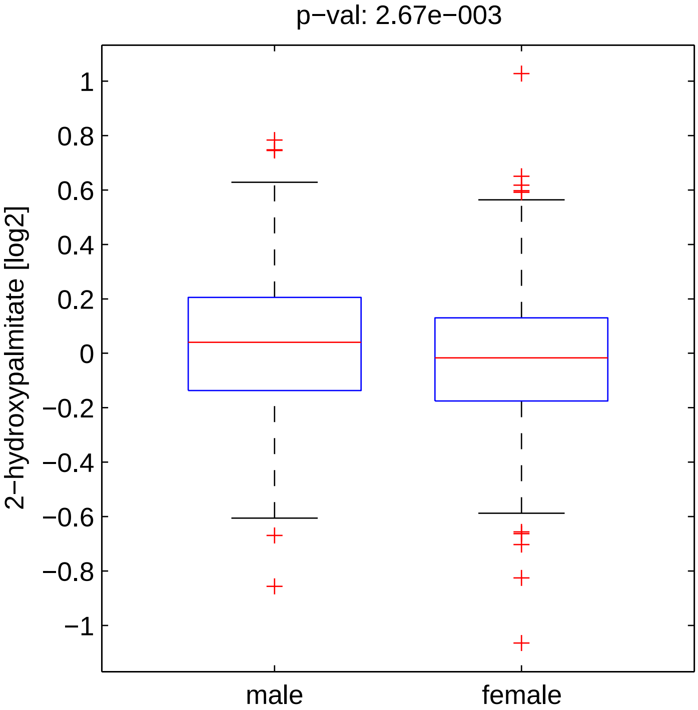

p−val: 2.67e−003
2−hydroxypalmitate [log2]
1
0.8
0.6
0.4
0.2
0
−0.2
−0.4
−0.6
−0.8
−1
male
female

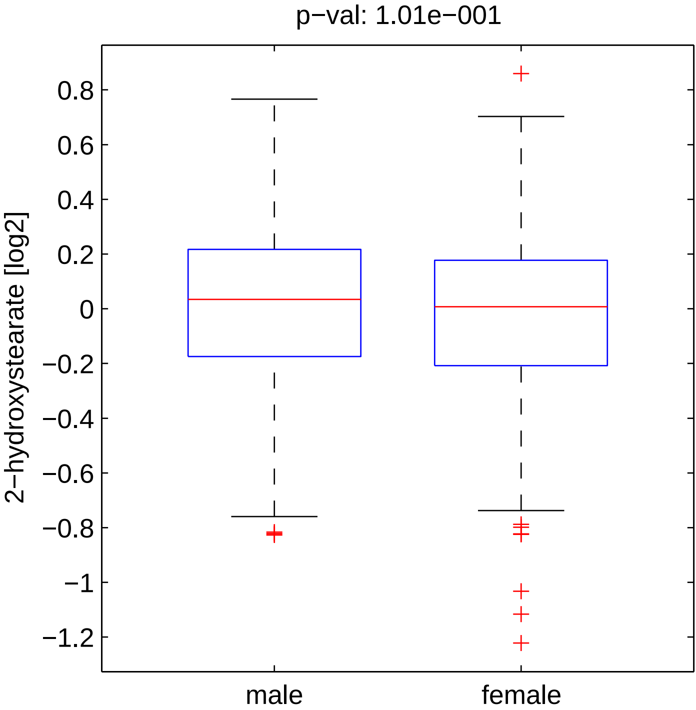

p−val: 1.01e−001
0.8
0.6
0.4
0.2
0
−0.2
−0.4
−0.6
−0.8
−1
−1.2
2−hydroxystearate [log2]
male
female

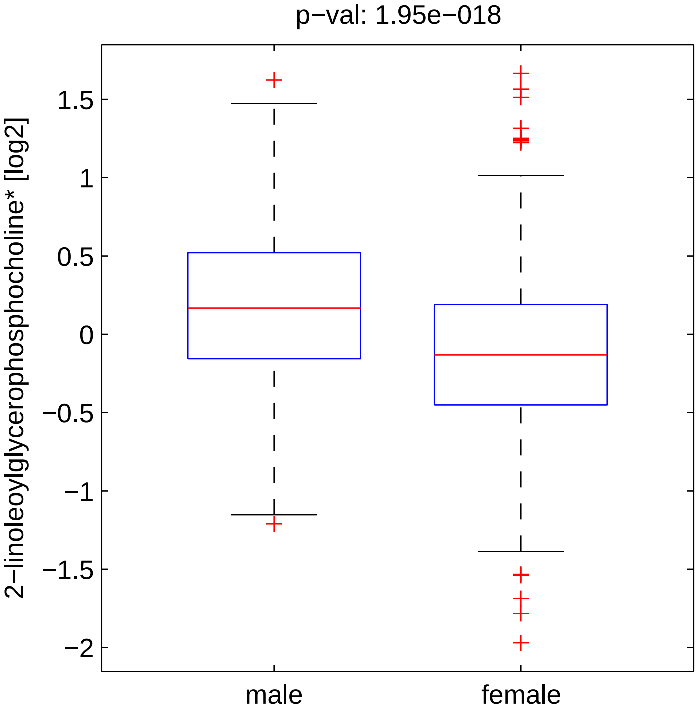
p−val: 1.95e−018
2−linoleoylglycerophosphocholine* [log2]
1.5
1
0.5
0
−0.5
−1
−1.5
−2
male
female

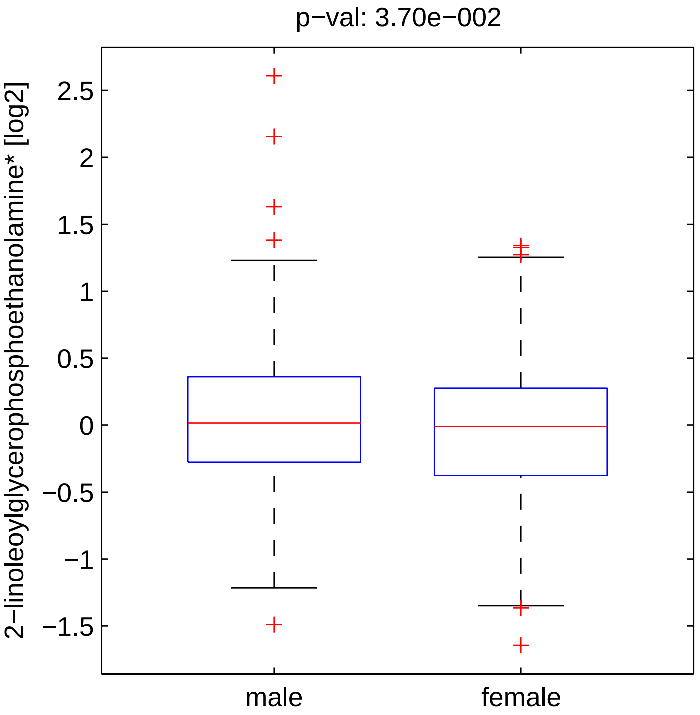

p−val: 3.70e−002
2−linoleoylglycerophosphoethanolamine* [log2]
2.5
2
1.5
1
0.5
0
−0.5
−1
−1.5
male
female

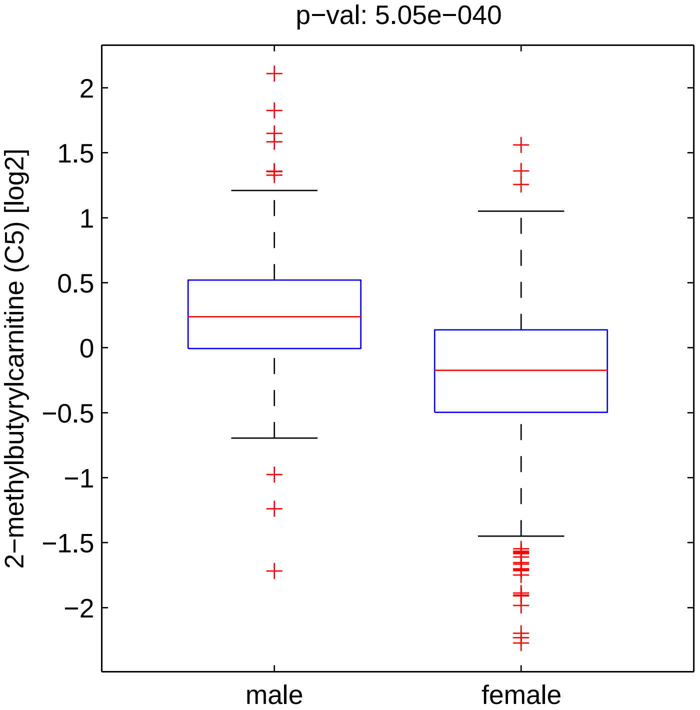
p−val: 5.05e−040
2−methylbutyrylcarnitine (C5) [log2]
2
1.5
1
0.5
0
−0.5
−1
−1.5
−2
male
female

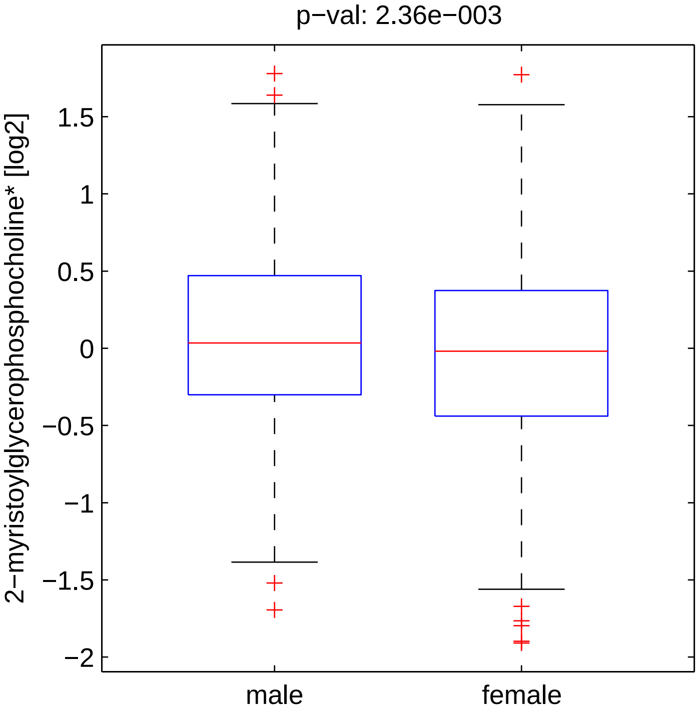
p−val: 2.36e−003
2−myristoylglycerophosphocholine* [log2]
1.5
1
0.5
0
−0.5
−1
−1.5
−2
male
female

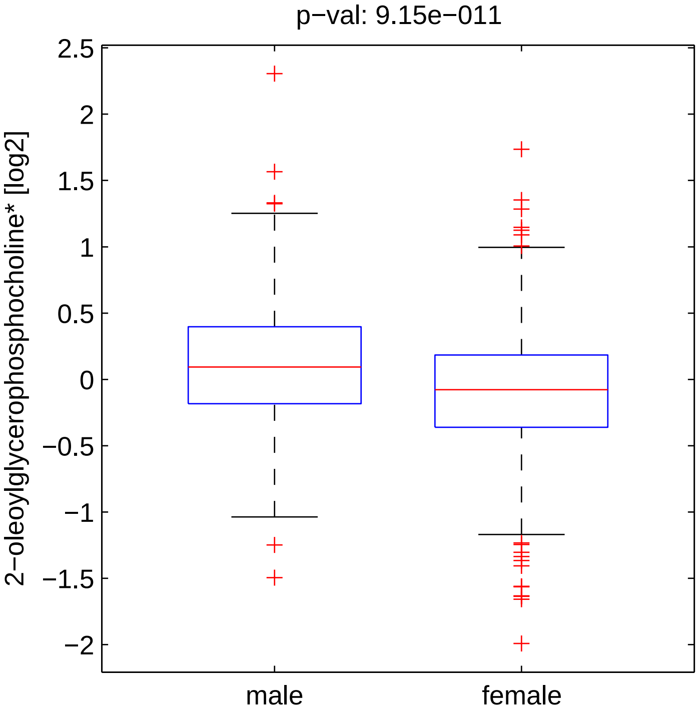

p−val: 9.15e−011
2−oleoylglycerophosphocholine* [log2]
2.5
2
1.5
1
0.5
0
−0.5
−1
−1.5
−2
male
female

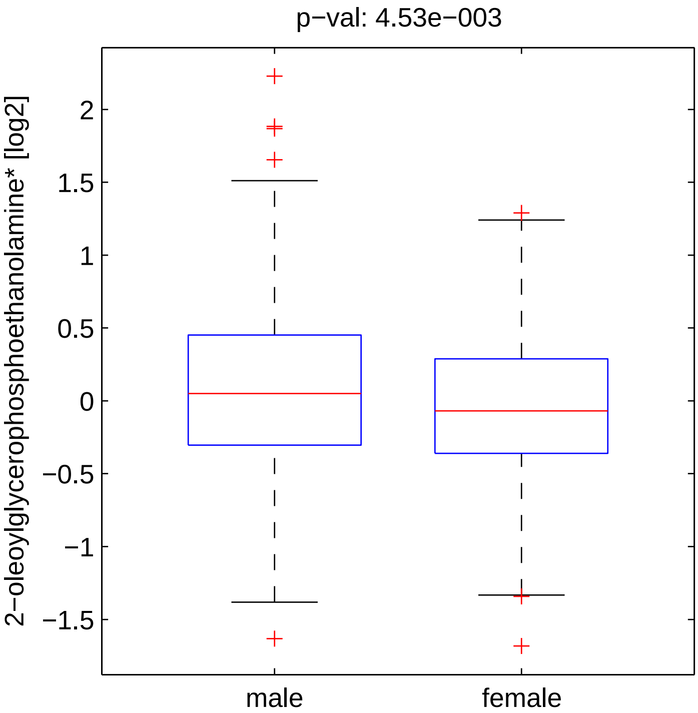
p−val: 4.53e−003
2−oleoylglycerophosphoethanolamine* [log2]
2
1.5
1
0.5
0
−0.5
−1
−1.5
male
female

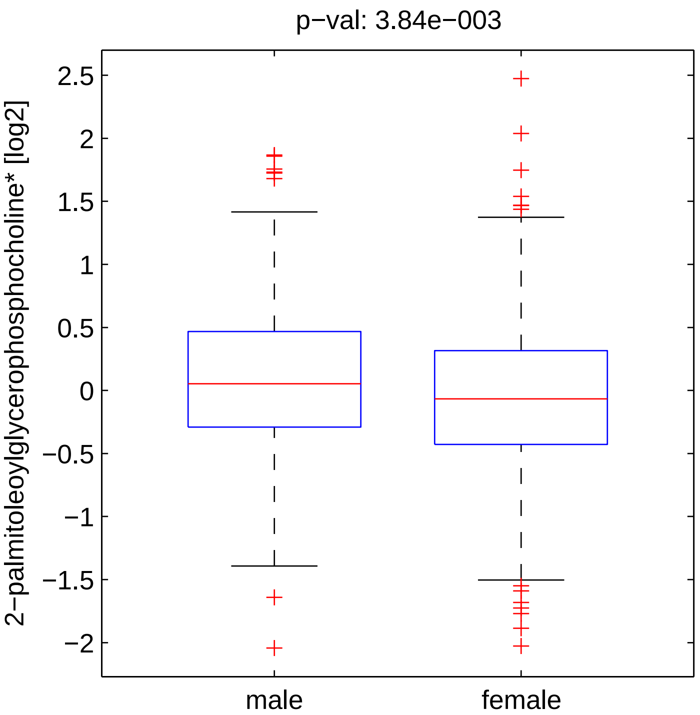
p−val: 3.84e−003
2−palmitoleoylglycerophosphocholine* [log2]
2.5
2
1.5
1
0.5
0
−0.5
−1
−1.5
−2
male
female

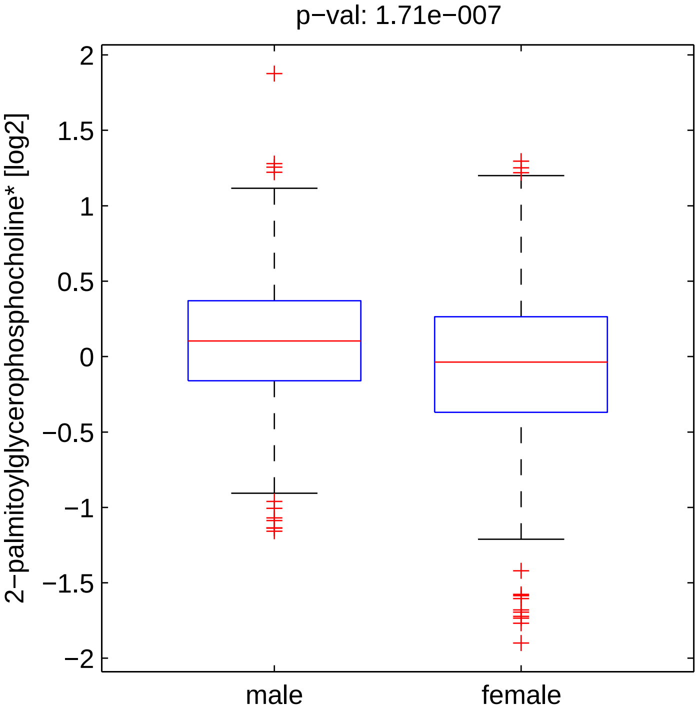

p−val: 1.71e−007
2−palmitoylglycerophosphocholine* [log2]
2
1.5
1
0.5
0
−0.5
−1
−1.5
−2
male
female

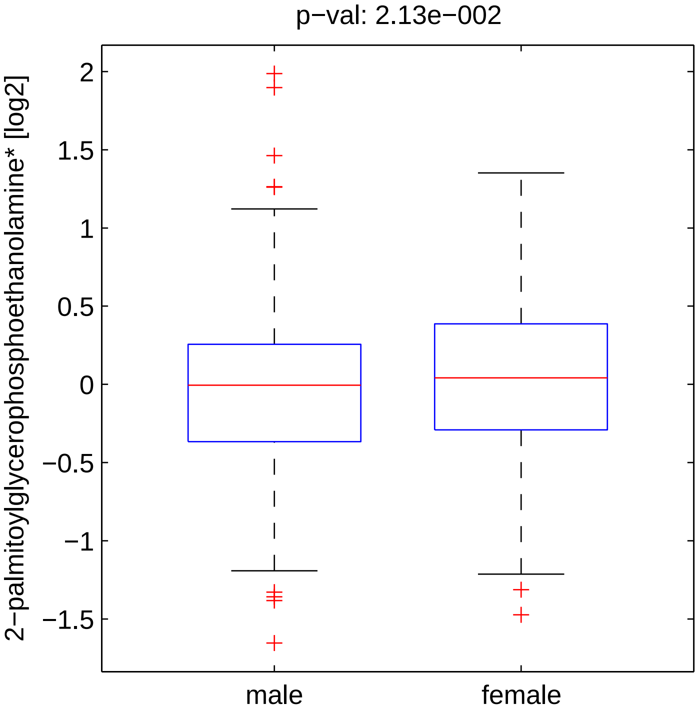
p−val: 2.13e−002
2−palmitoylglycerophosphoethanolamine* [log2]
2
1.5
1
0.5
0
−0.5
−1
−1.5
male
female

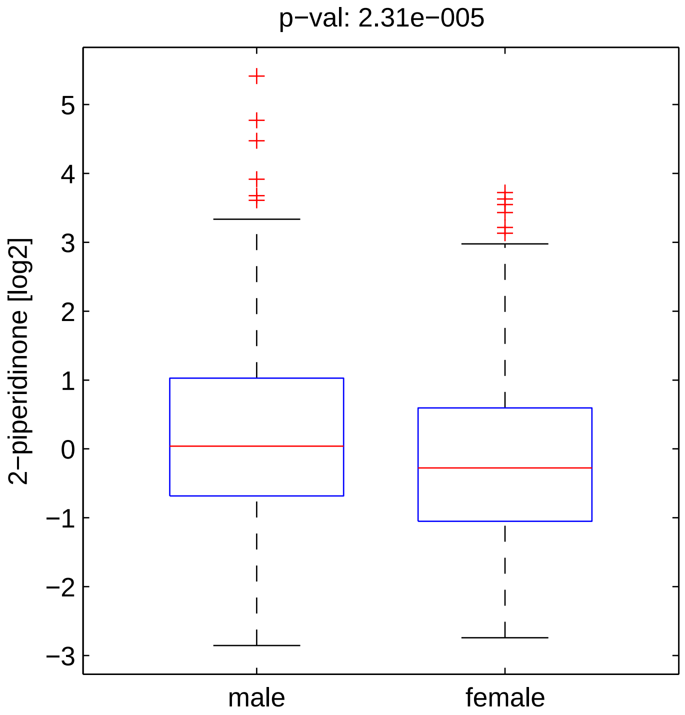

p−val: 2.31e−005
2−piperidinone [log2]
5
4
3
2
1
0
−1
−2
−3
male
female

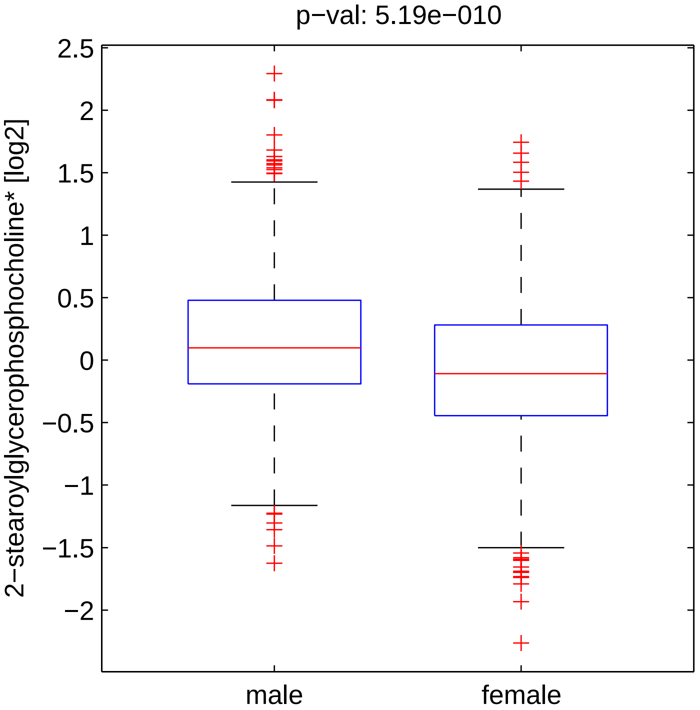
p−val: 5.19e−010
2−stearoylglycerophosphocholine* [log2]
2.5
2
1.5
1
0.5
0
−0.5
−1
−1.5
−2
male
female

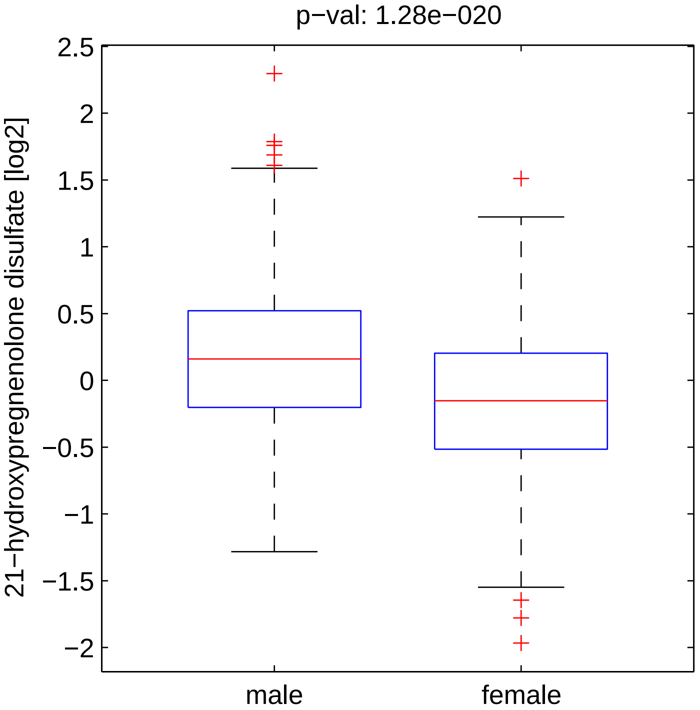
p−val: 1.28e−020
2.5
2
1.5
1
0.5
0
−0.5
−1
−1.5
−2
21−hydroxypregnenolone disulfate [log2]
male
female

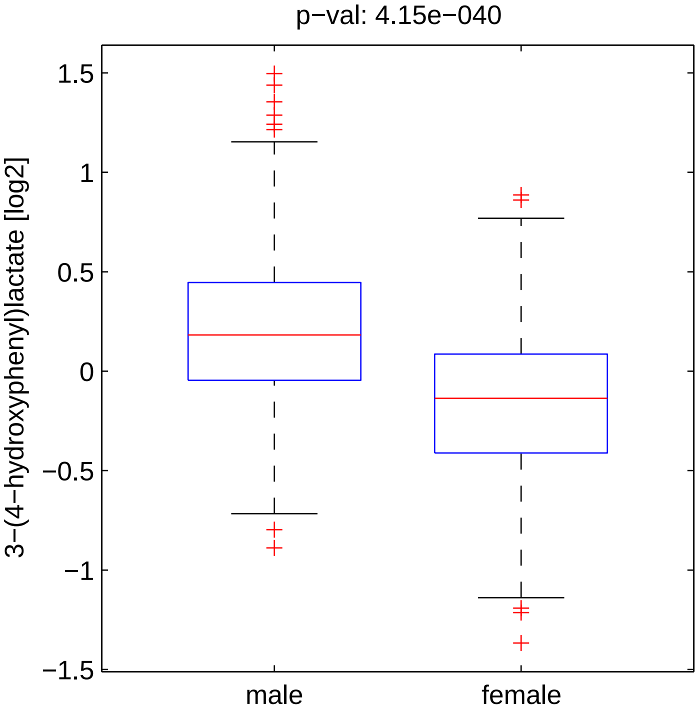

p−val: 4.15e−040
3−(4−hydroxyphenyl)lactate [log2]
1.5
1
0.5
0
−0.5
−1
−1.5
male
female

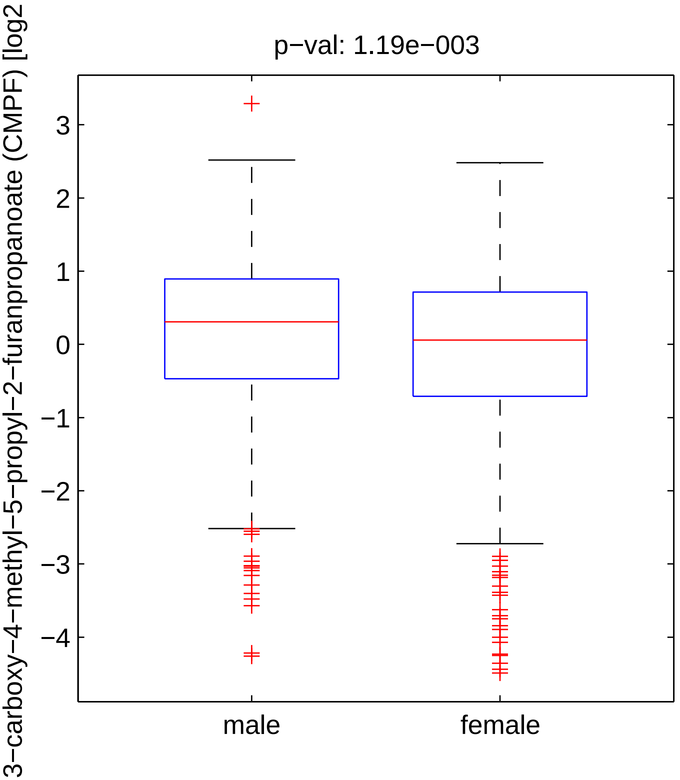

p−val: 1.19e−003
3−carboxy−4−methyl−5−propyl−2−furanpropanoate (CMPF) [log2
3
2
1
0
−1
−2
−3
−4
male
female

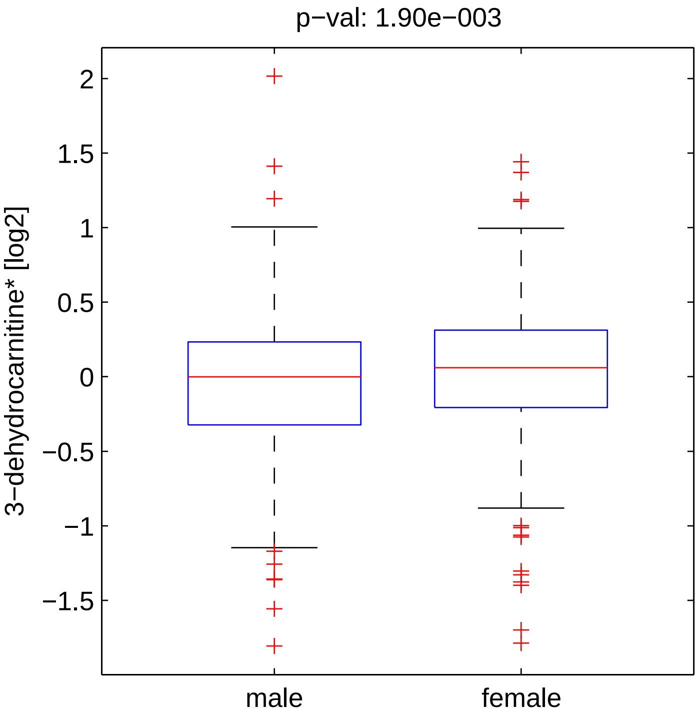

p−val: 1.90e−003
2
1.5
1
0.5
0
−0.5
−1
−1.5
3−dehydrocarnitine* [log2]
male
female

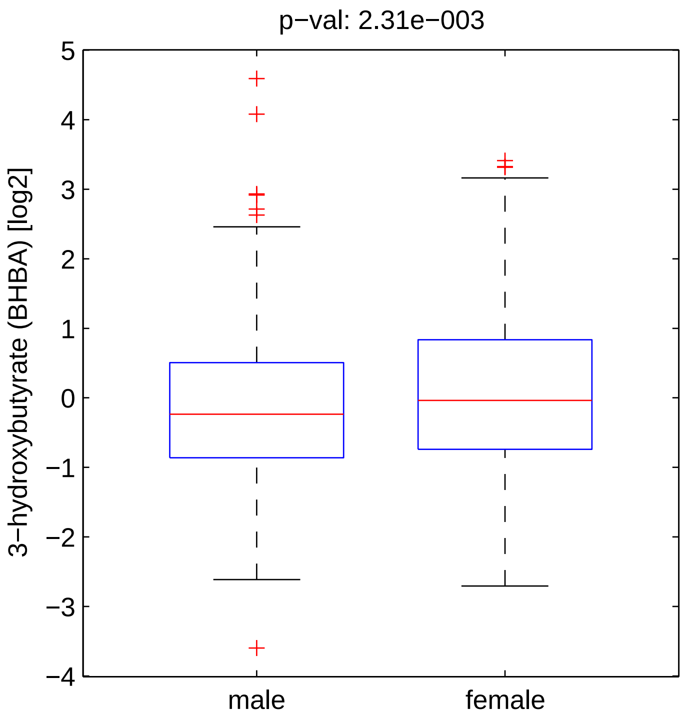
p−val: 2.31e−003
5
4
3
2
1
0
−1
−2
−3
−4
3−hydroxybutyrate (BHBA) [log2]
male
female

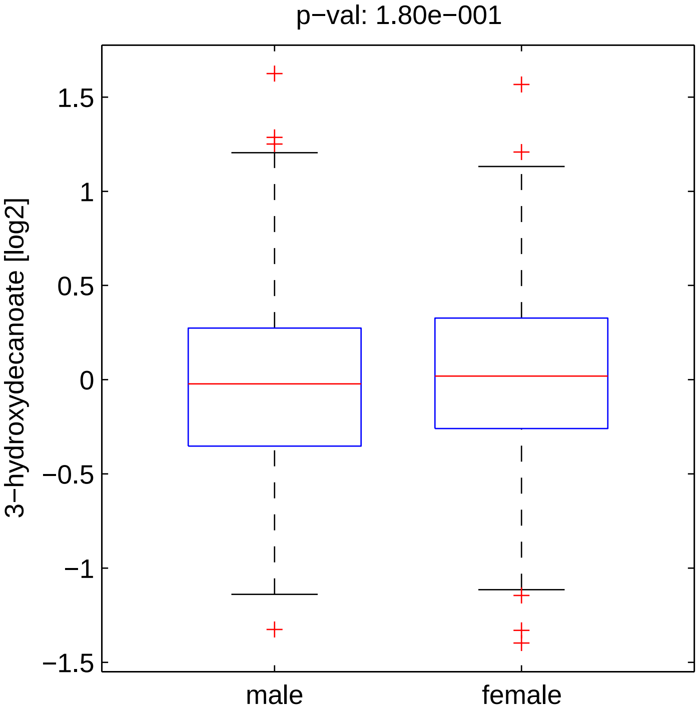
p−val: 1.80e−001
3−hydroxydecanoate [log2]
1.5
1
0.5
0
−0.5
−1
−1.5
male
female

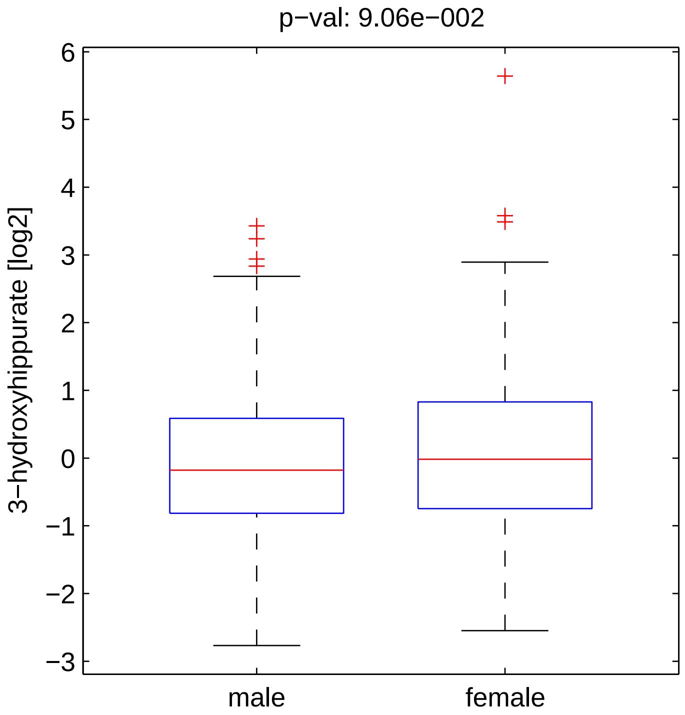
p−val: 9.06e−002
6
5
4
3
2
1
0
−1
−2
−3
3−hydroxyhippurate [log2]
male
female

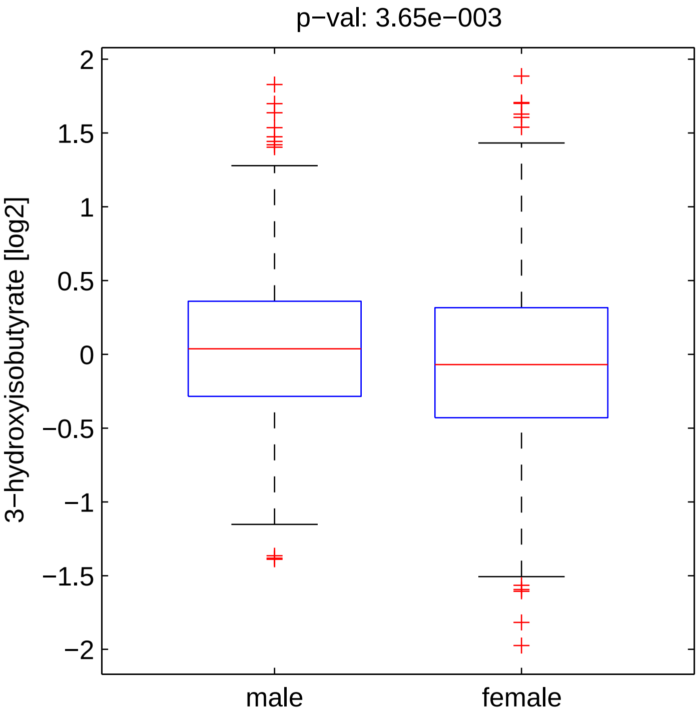
p−val: 3.65e−003
3−hydroxyisobutyrate [log2]
2
1.5
1
0.5
0
−0.5
−1
−1.5
−2
male
female

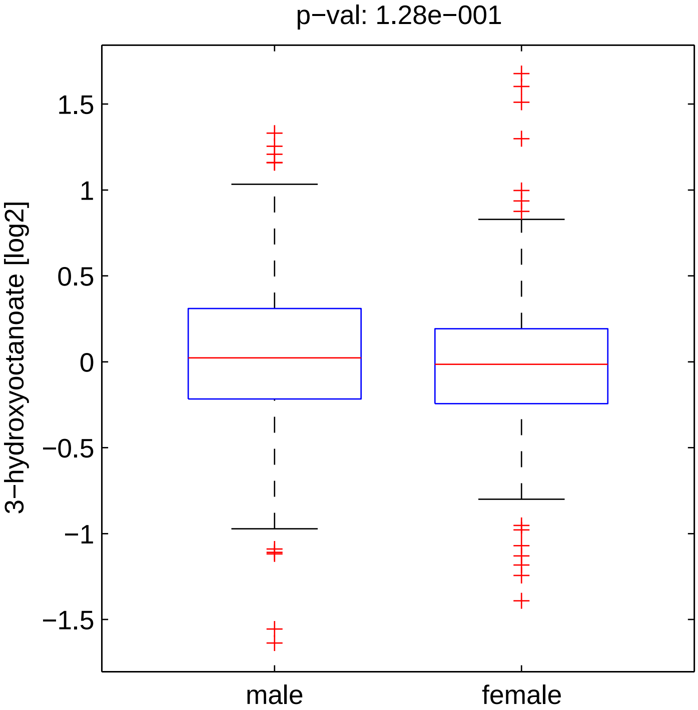
p−val: 1.28e−001
3−hydroxyoctanoate [log2]
1.5
1
0.5
0
−0.5
−1
−1.5
male
female

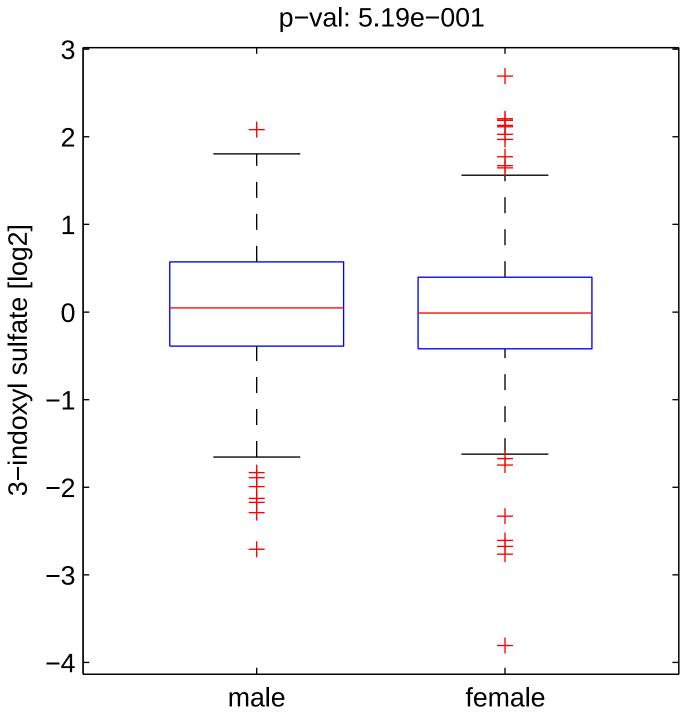

p−val: 5.19e−001
3−indoxyl sulfate [log2]
3
2
1
0
−1
−2
−3
−4
male
female

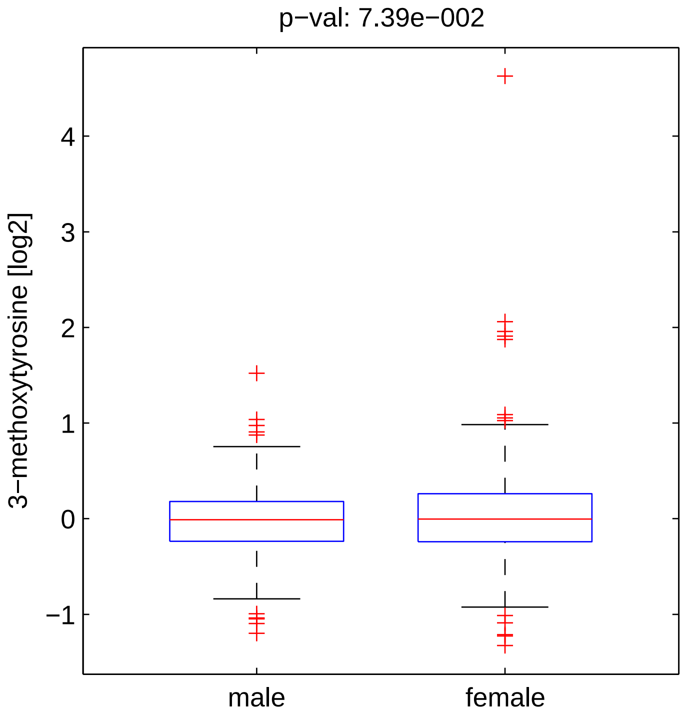
p−val: 7.39e−002
3−methoxytyrosine [log2]
4
3
2
1
0
−1
male
female

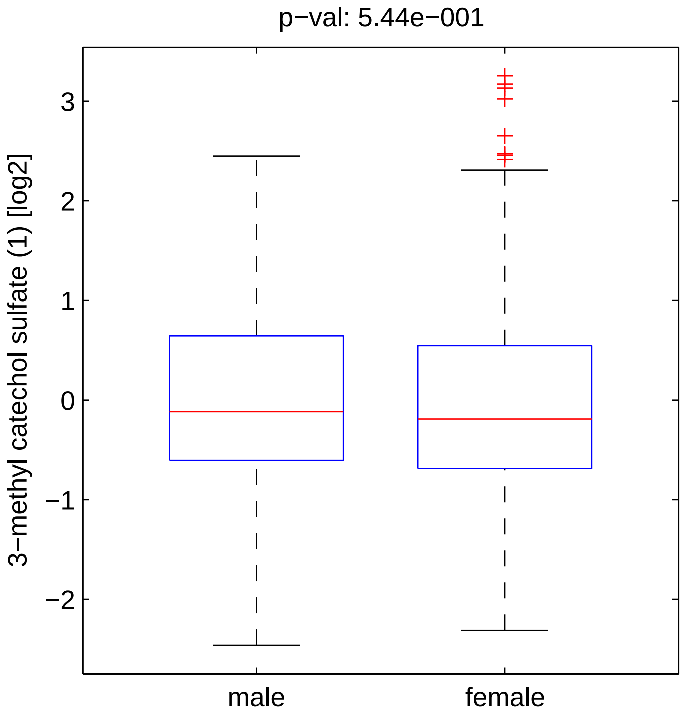

p−val: 5.44e−001
3−methyl catechol sulfate (1) [log2]
3
2
1
0
−1
−2
male
female

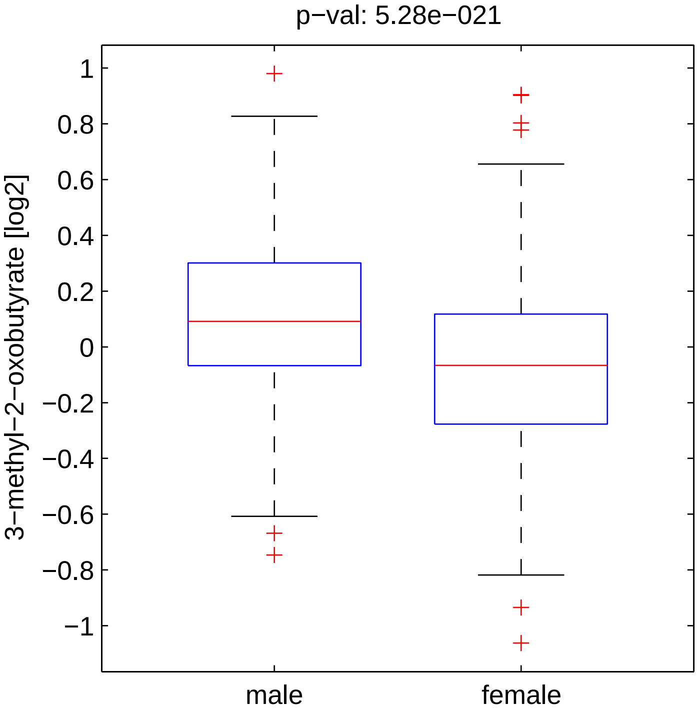
p−val: 5.28e−021
3−methyl−2−oxobutyrate [log2]
1
0.8
0.6
0.4
0.2
0
−0.2
−0.4
−0.6
−0.8
−1
male
female

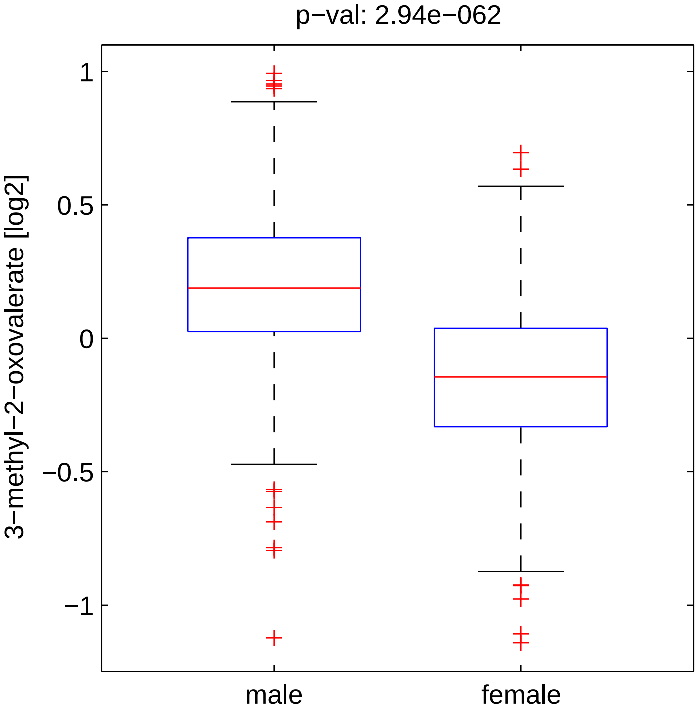
p−val: 2.94e−062
3−methyl−2−oxovalerate [log2]
1
0.5
0
−0.5
−1
male
female

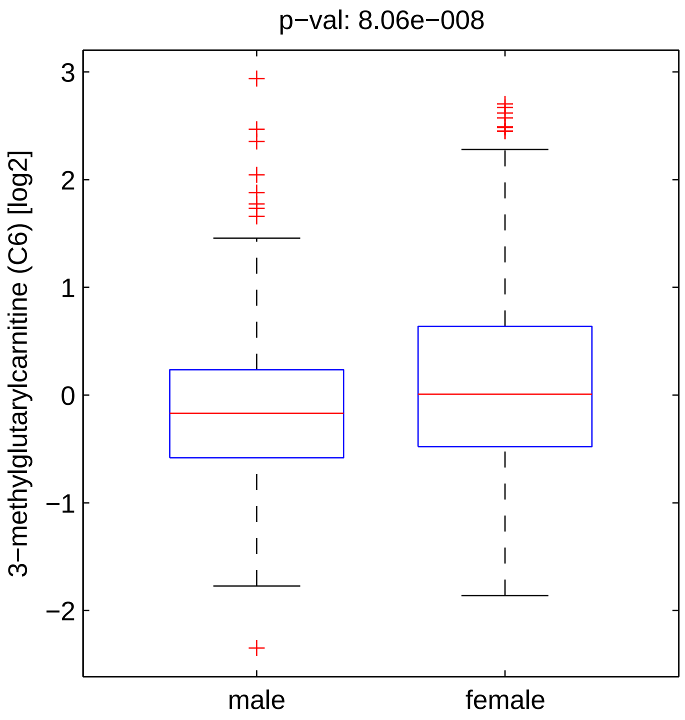
p−val: 8.06e−008
3−methylglutarylcarnitine (C6) [log2]
3
2
1
0
−1
−2
male
female

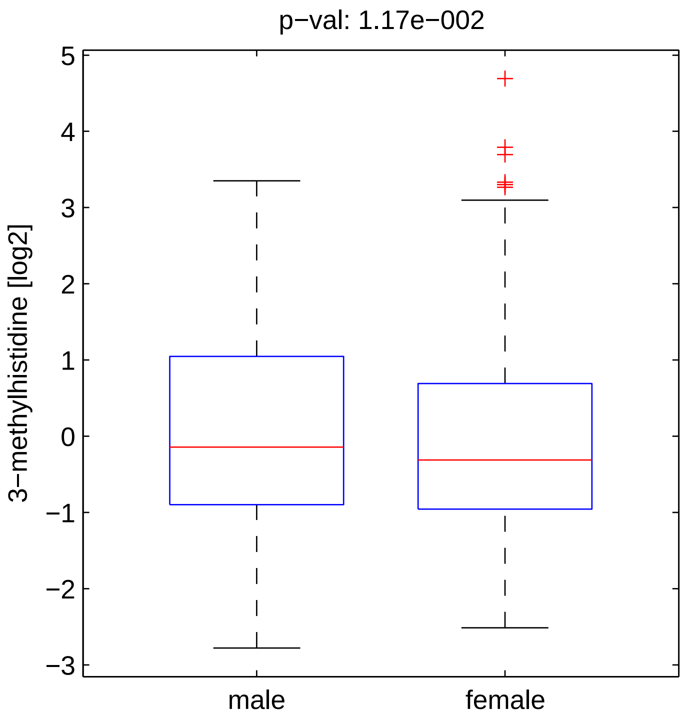
p−val: 1.17e−002
3−methylhistidine [log2]
5
4
3
2
1
0
−1
−2
−3
male
female

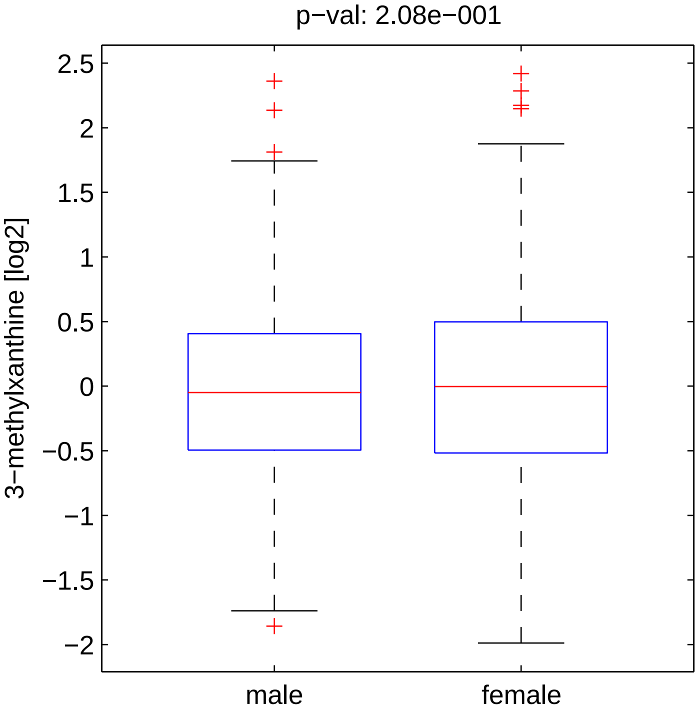
p−val: 2.08e−001
3−methylxanthine [log2]
2.5
2
1.5
1
0.5
0
−0.5
−1
−1.5
−2
male
female

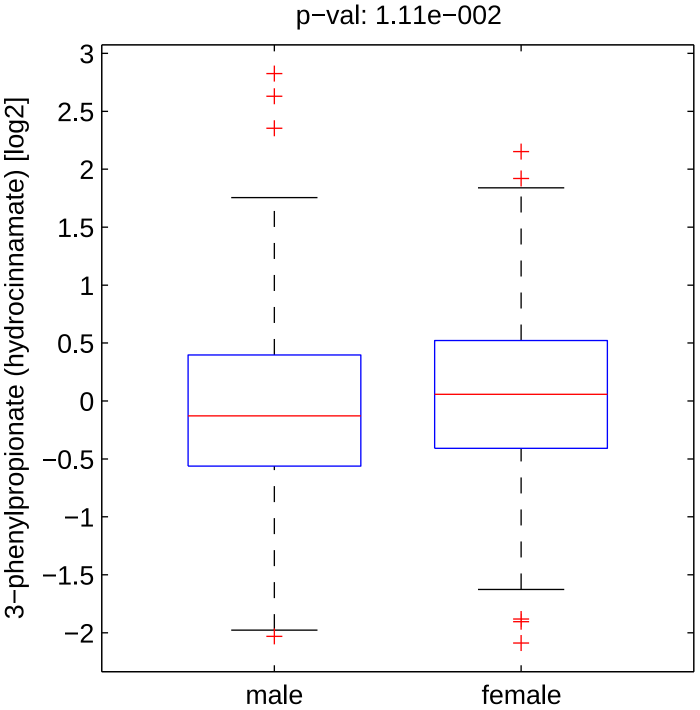

p−val: 1.11e−002
3−phenylpropionate (hydrocinnamate) [log2]
3
2.5
2
1.5
1
0.5
0
−0.5
−1
−1.5
−2
male
female

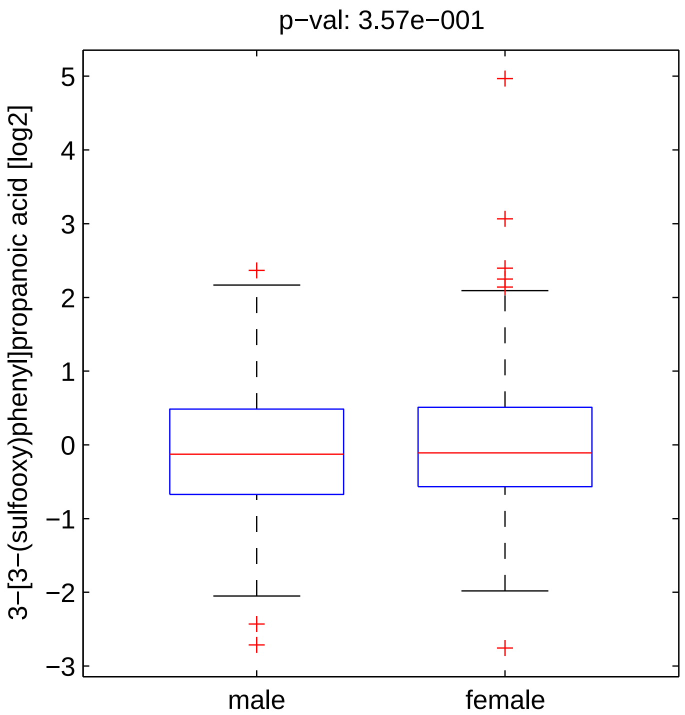

p−val: 3.57e−001
3−[3−(sulfooxy)phenyl]propanoic acid [log2]
5
4
3
2
1
0
−1
−2
−3
male
female

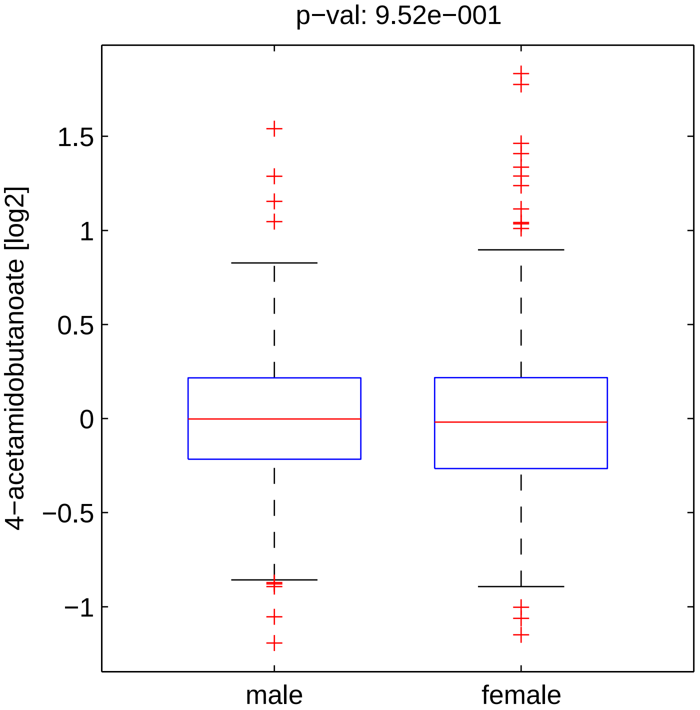
p−val: 9.52e−001
4−acetamidobutanoate [log2]
1.5
1
0.5
0
−0.5
−1
male
female

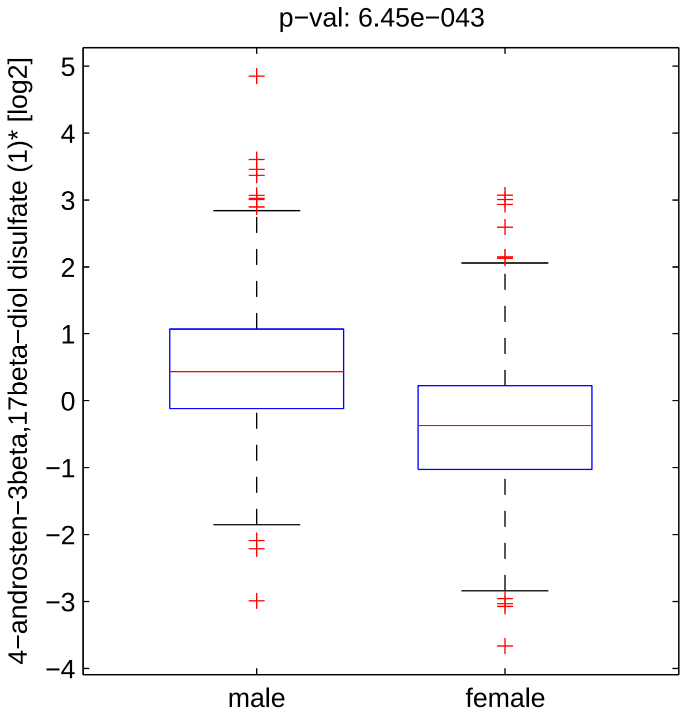

p−val: 6.45e−043
4−androsten−3beta,17beta−diol disulfate (1)* [log2]
5
4
3
2
1
0
−1
−2
−3
−4
male
female

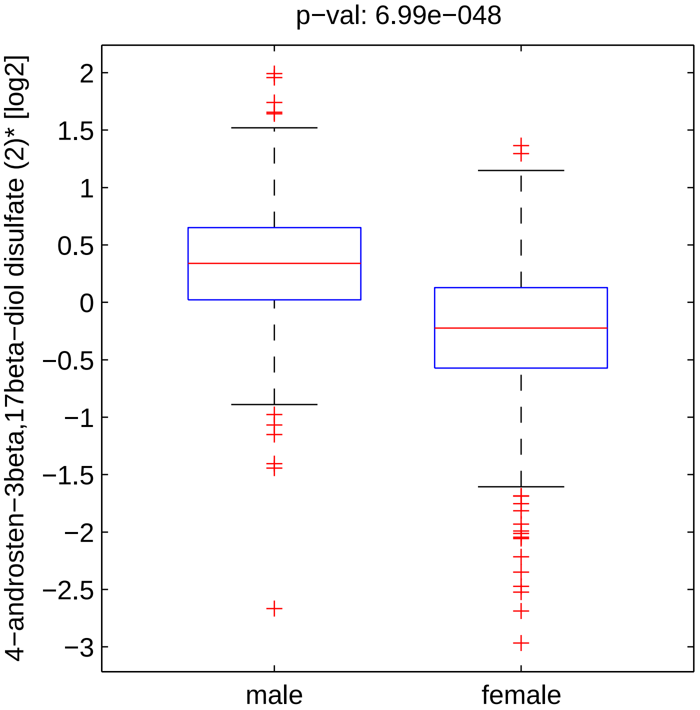

p−val: 6.99e−048
4−androsten−3beta,17beta−diol disulfate (2)* [log2]
2
1.5
1
0.5
0
−0.5
−1
−1.5
−2
−2.5
−3
male
female

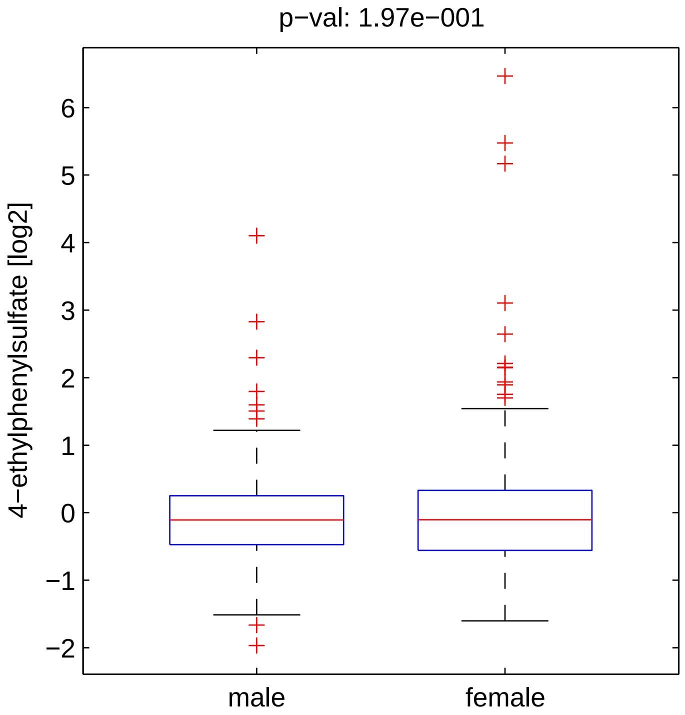

p−val: 1.97e−001
4−ethylphenylsulfate [log2]
6
5
4
3
2
1
0
−1
−2
male
female

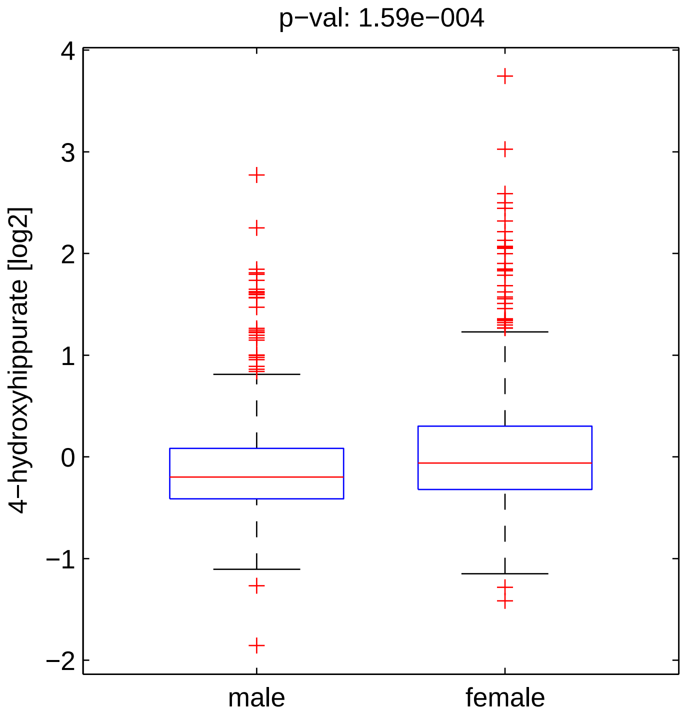
p−val: 1.59e−004
4−hydroxyhippurate [log2]
4
3
2
1
0
−1
−2
male
female

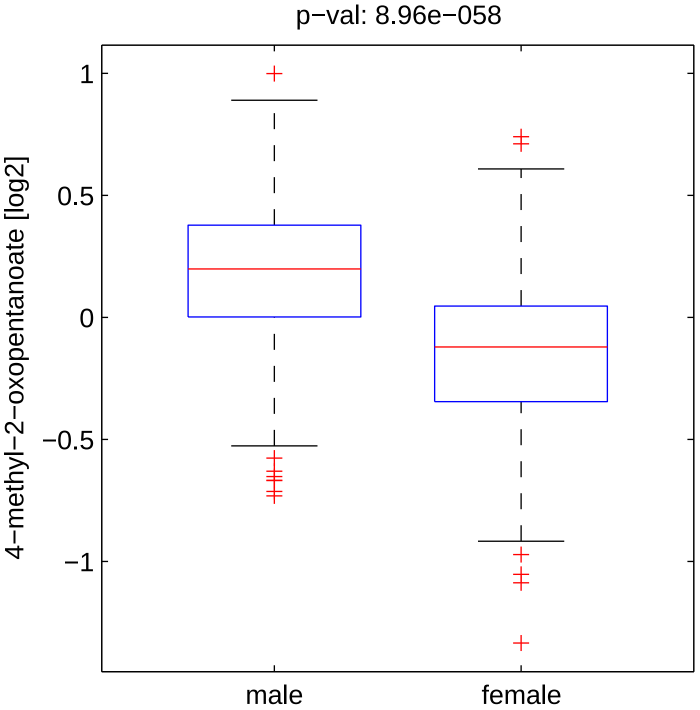
p−val: 8.96e−058
4−methyl−2−oxopentanoate [log2]
1
0.5
0
−0.5
−1
male
female

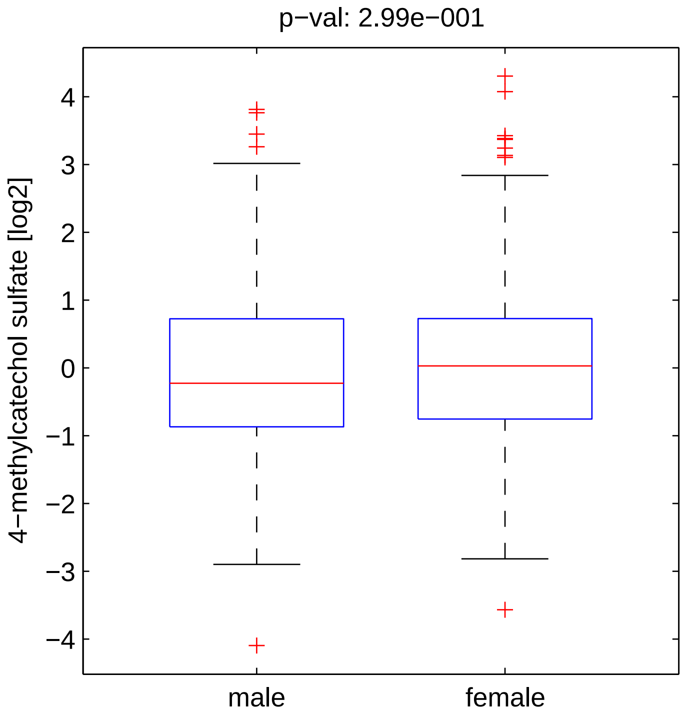
p−val: 2.99e−001
4−methylcatechol sulfate [log2]
4
3
2
1
0
−1
−2
−3
−4
male
female

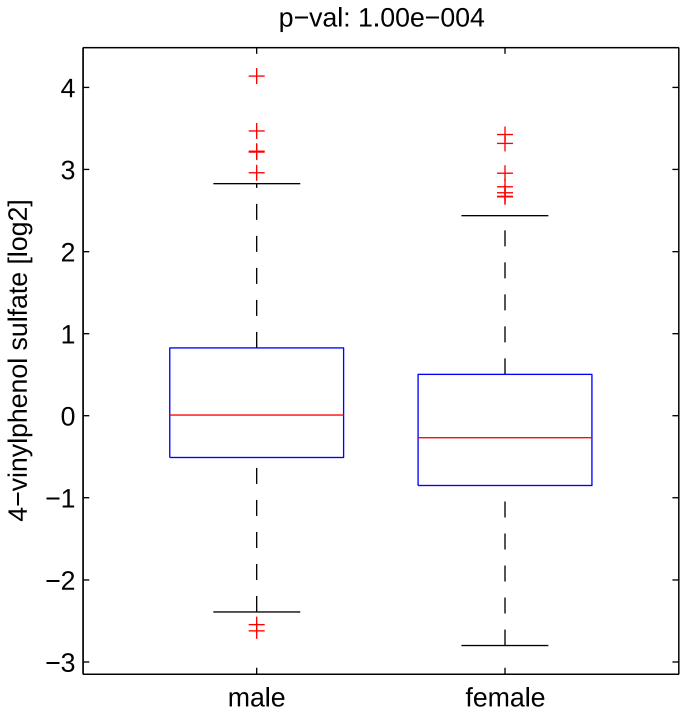
p−val: 1.00e−004
4−vinylphenol sulfate [log2]
4
3
2
1
0
−1
−2
−3
male
female

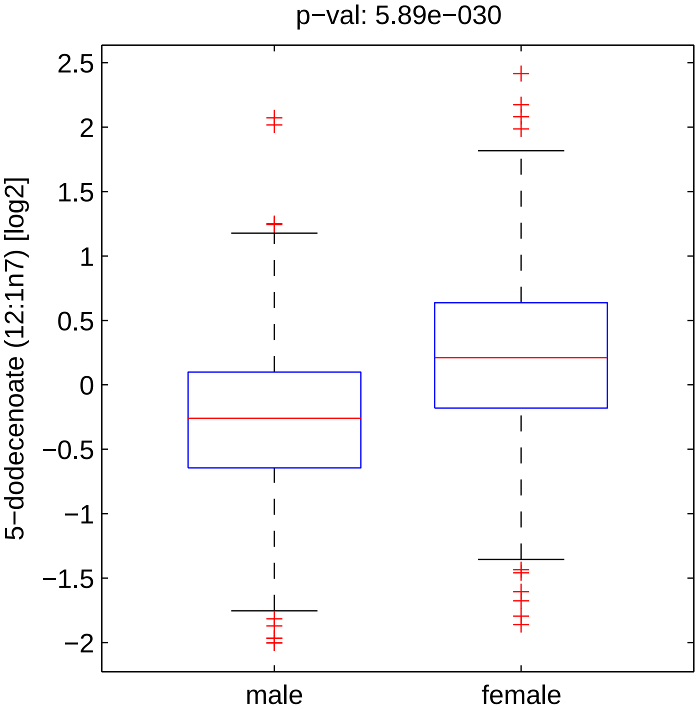
p−val: 5.89e−030
2.5
2
1.5
1
0.5
0
−0.5
−1
−1.5
−2
5−dodecenoate (12:1n7) [log2]
male
female

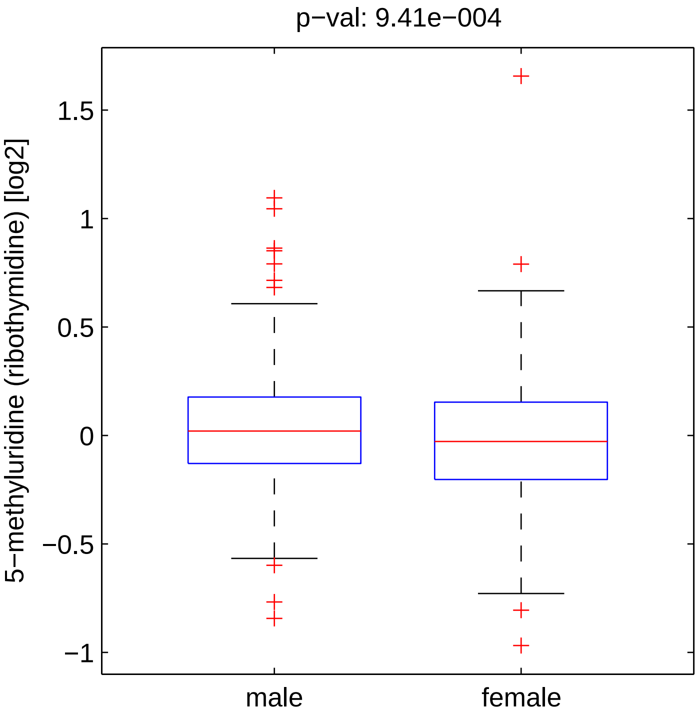
p−val: 9.41e−004
5−methyluridine (ribothymidine) [log2]
1.5
1
0.5
0
−0.5
−1
male
female

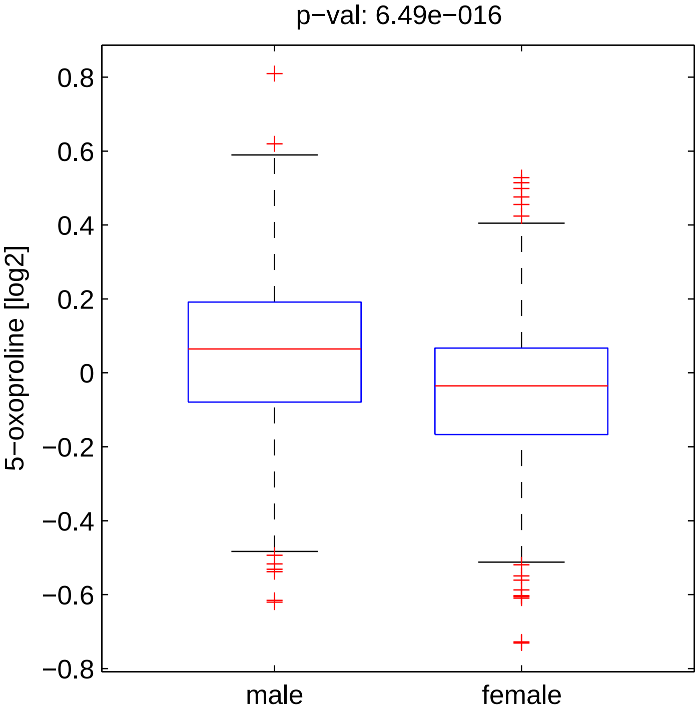
p−val: 6.49e−016
5−oxoproline [log2]
0.8
0.6
0.4
0.2
0
−0.2
−0.4
−0.6
−0.8
male
female

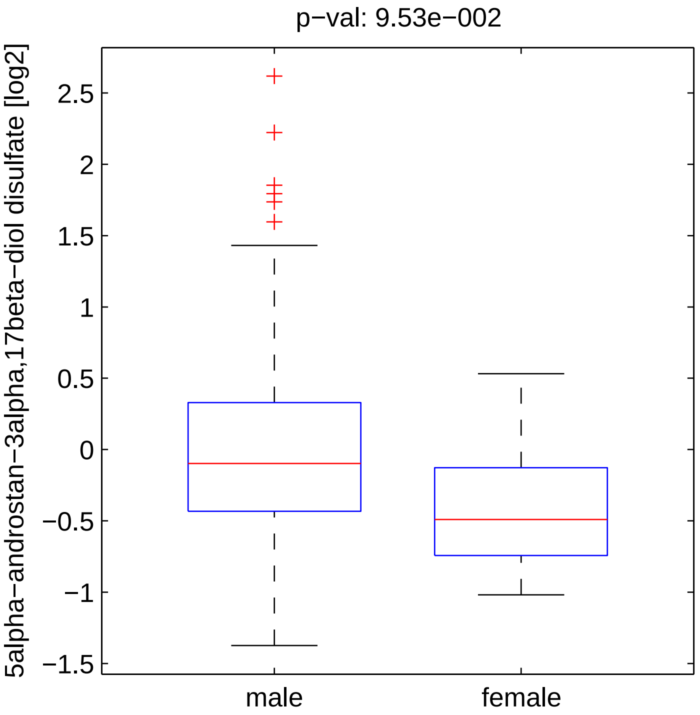
p−val: 9.53e−002
5alpha−androstan−3alpha,17beta−diol disulfate [log2]
2.5
2
1.5
1
0.5
0
−0.5
−1
−1.5
male
female

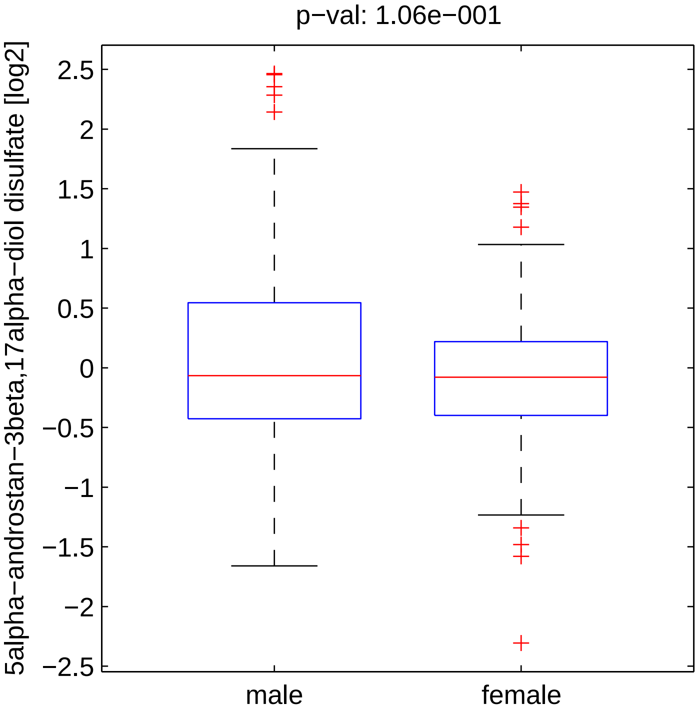

p−val: 1.06e−001
5alpha−androstan−3beta,17alpha−diol disulfate [log2]
2.5
2
1.5
1
0.5
0
−0.5
−1
−1.5
−2
−2.5
male
female

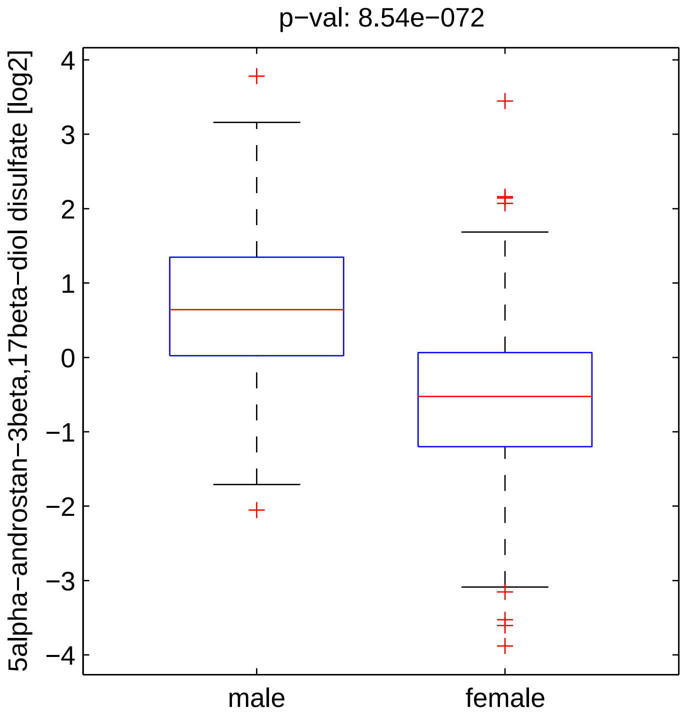
p−val: 8.54e−072
5alpha−androstan−3beta,17beta−diol disulfate [log2]
4
3
2
1
0
−1
−2
−3
−4
male
female

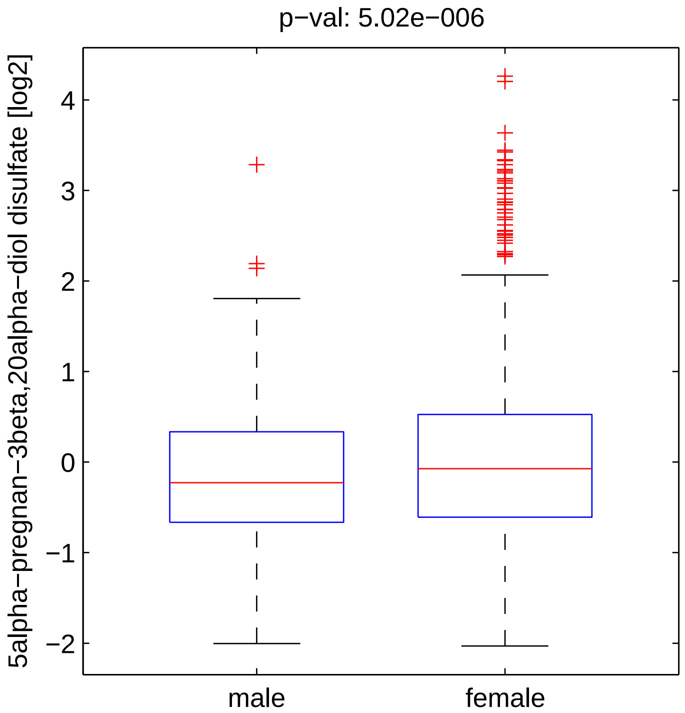
p−val: 5.02e−006
5alpha−pregnan−3beta,20alpha−diol disulfate [log2]
4
3
2
1
0
−1
−2
male
female

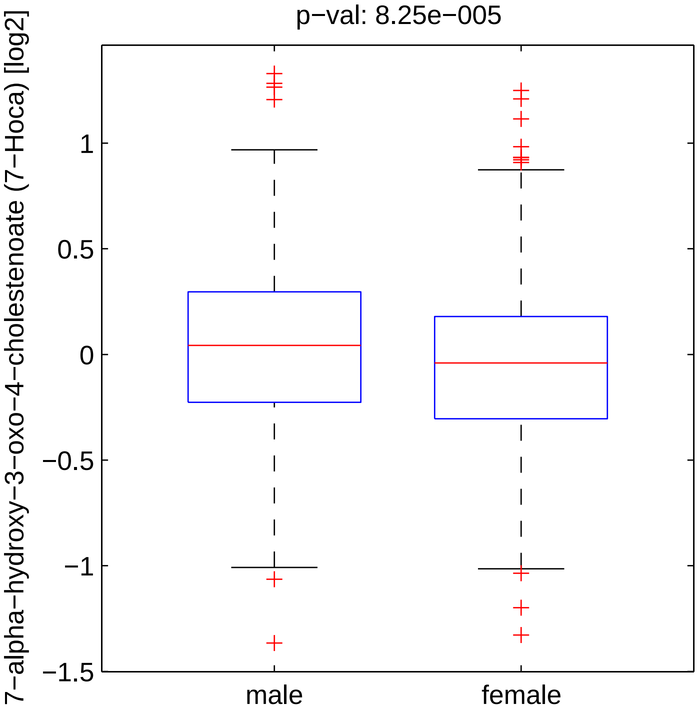

p−val: 8.25e−005
7−alpha−hydroxy−3−oxo−4−cholestenoate (7−Hoca) [log2]
1
0.5
0
−0.5
−1
−1.5
male
female

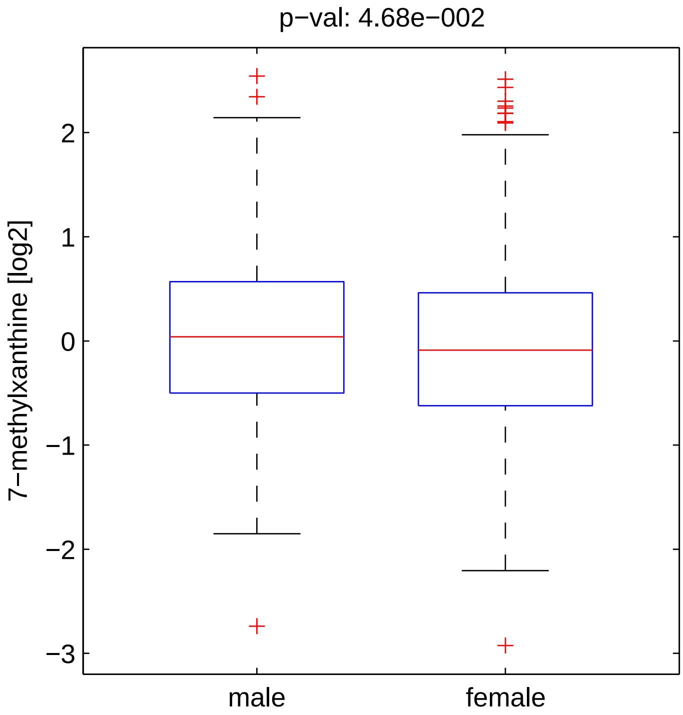
p−val: 4.68e−002
7−methylxanthine [log2]
2
1
0
−1
−2
−3
male
female

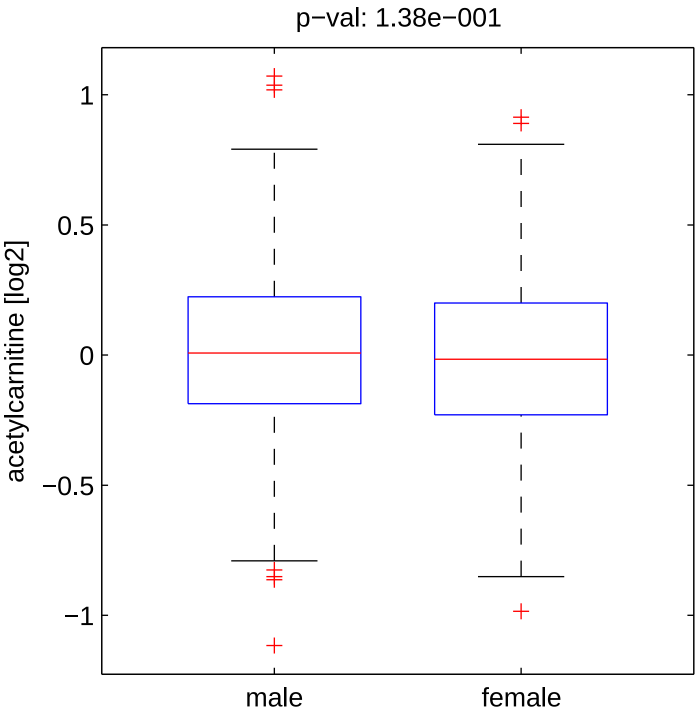
p−val: 1.38e−001
acetylcarnitine [log2]
1
0.5
0
−0.5
−1
male
female

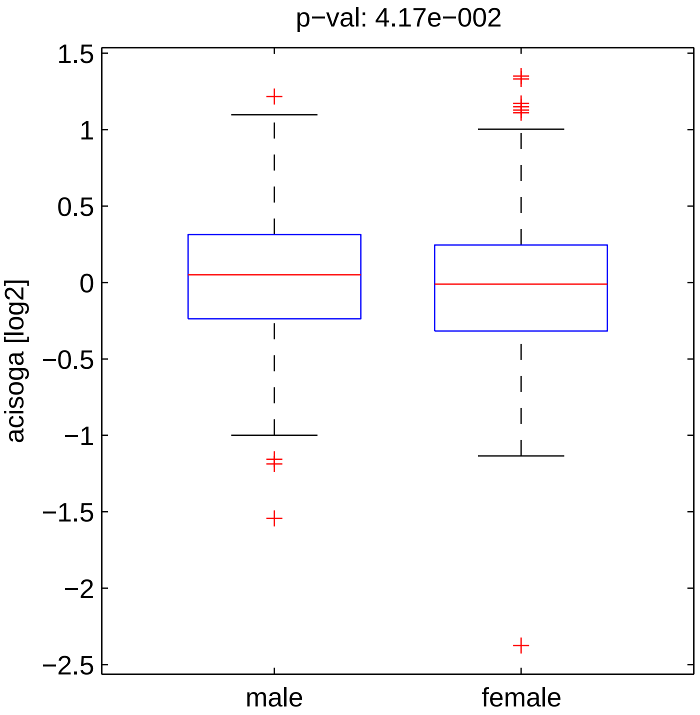
p−val: 4.17e−002
1.5
1
0.5
0
−0.5
−1
−1.5
−2
−2.5
acisoga [log2]
male
female

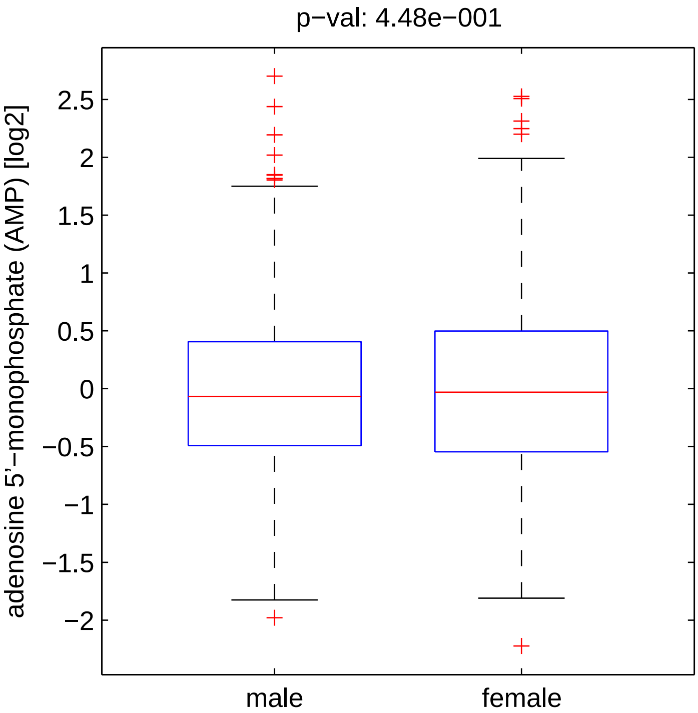
p−val: 4.48e−001
adenosine 5'−monophosphate (AMP) [log2]
2.5
2
1.5
1
0.5
0
−0.5
−1
−1.5
−2
male
female

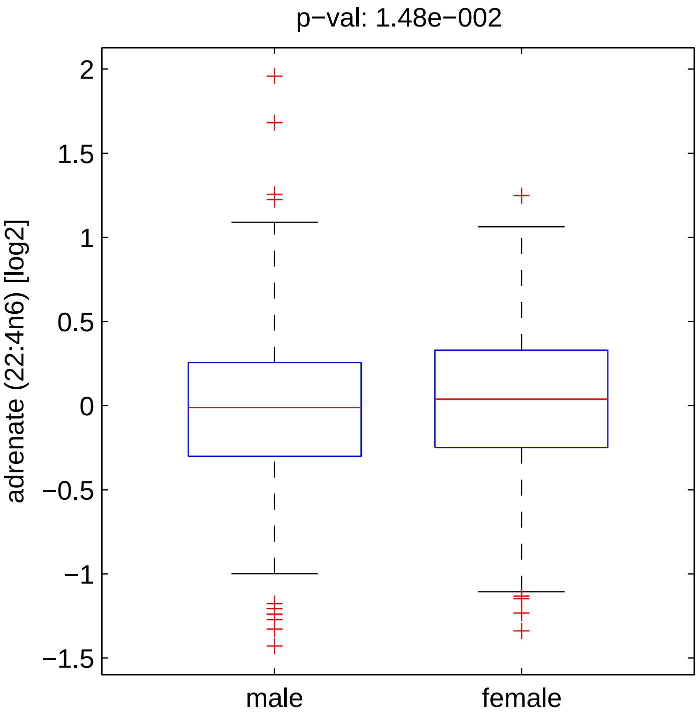
p−val: 1.48e−002
adrenate (22:4n6) [log2]
2
1.5
1
0.5
0
−0.5
−1
−1.5
male
female

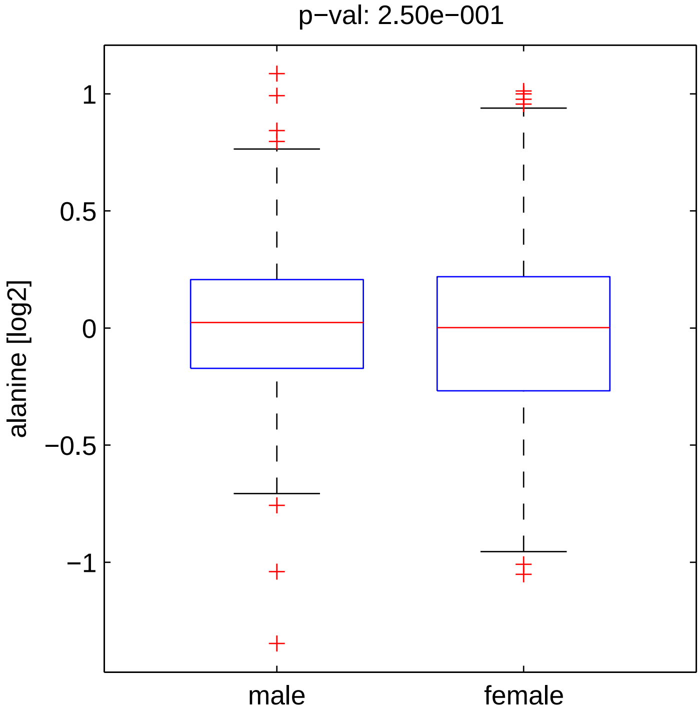

p−val: 2.50e−001
1
0.5
0
−0.5
−1
alanine [log2]
male
female

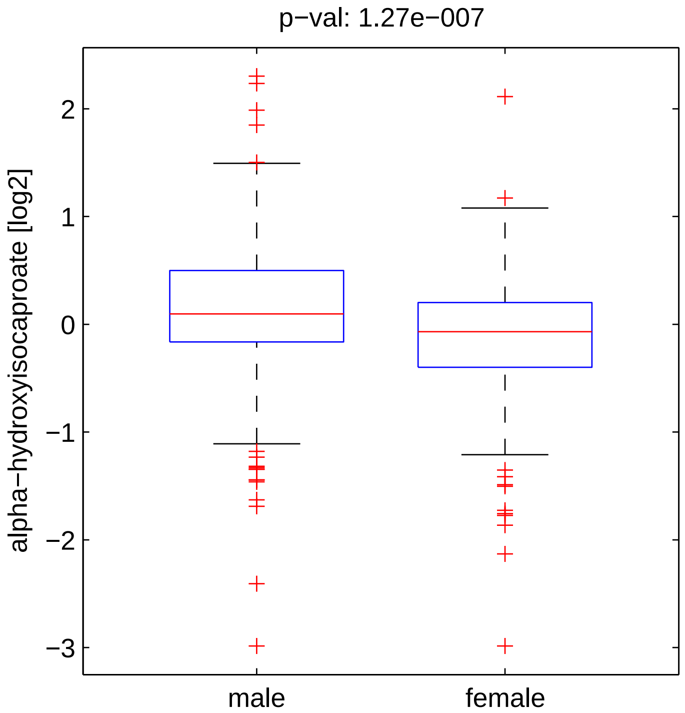

p−val: 1.27e−007
alpha−hydroxyisocaproate [log2]
2
1
0
−1
−2
−3
male
female

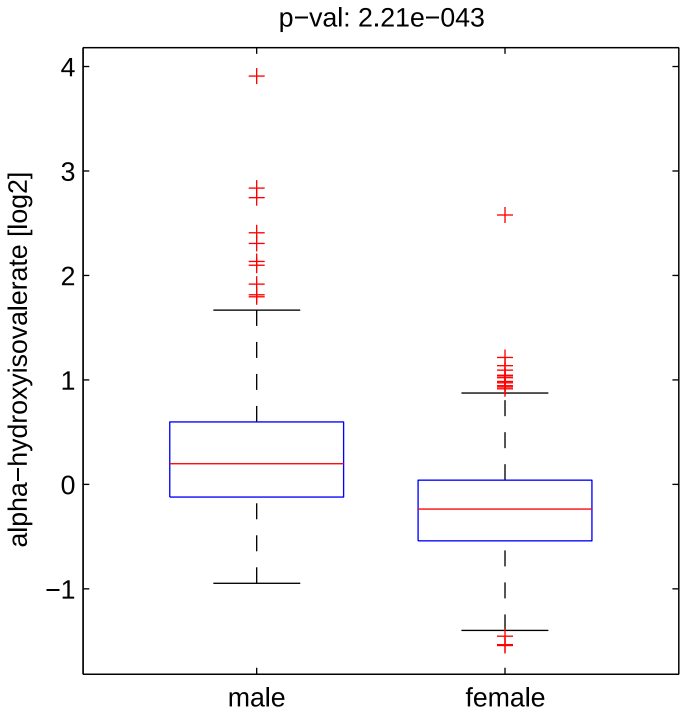

p−val: 2.21e−043
alpha−hydroxyisovalerate [log2]
4
3
2
1
0
−1
male
female

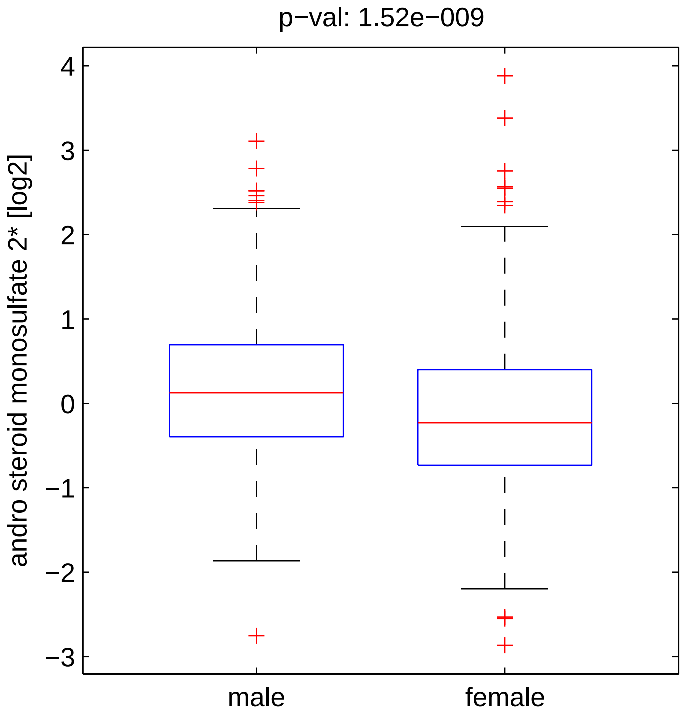
p−val: 1.52e−009
andro steroid monosulfate 2* [log2]
4
3
2
1
0
−1
−2
−3
male
female

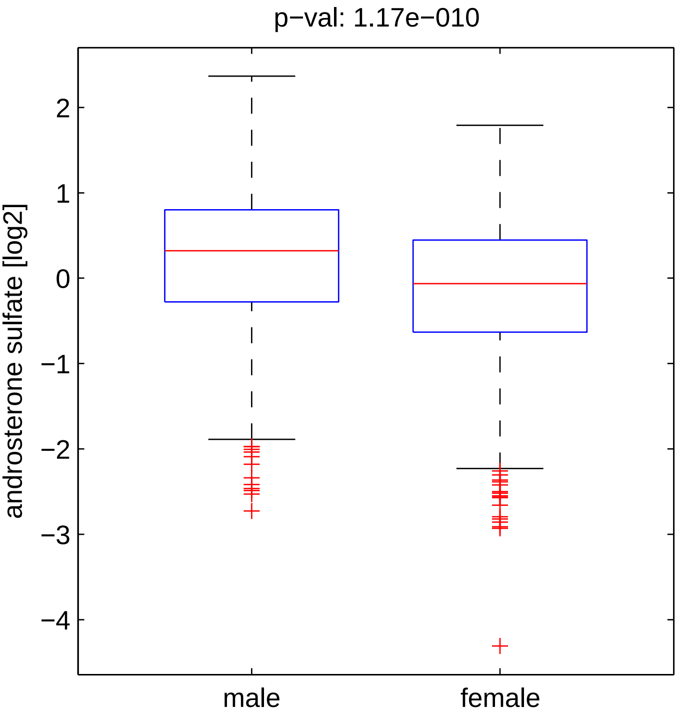

p−val: 1.17e−010
androsterone sulfate [log2]
2
1
0
−1
−2
−3
−4
male
female

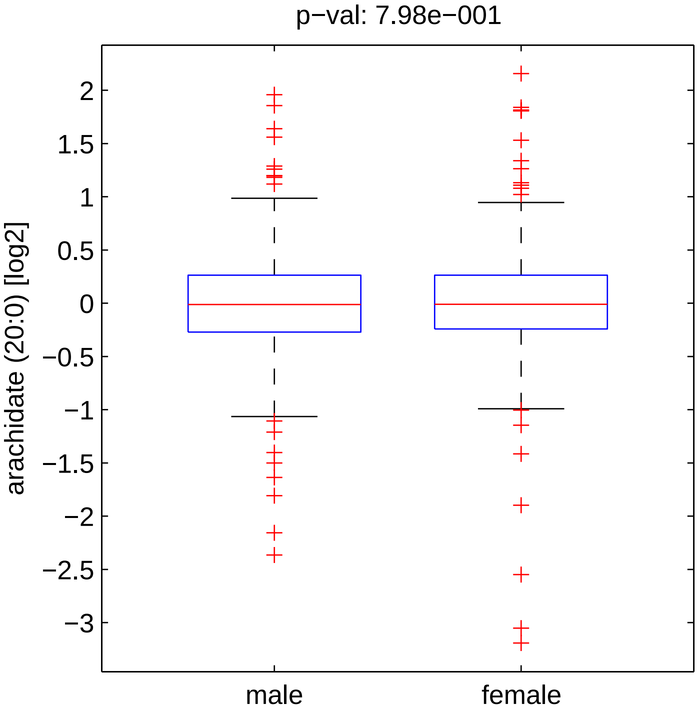

p−val: 7.98e−001
arachidate (20:0) [log2]
2
1.5
1
0.5
0
−0.5
−1
−1.5
−2
−2.5
−3
male
female

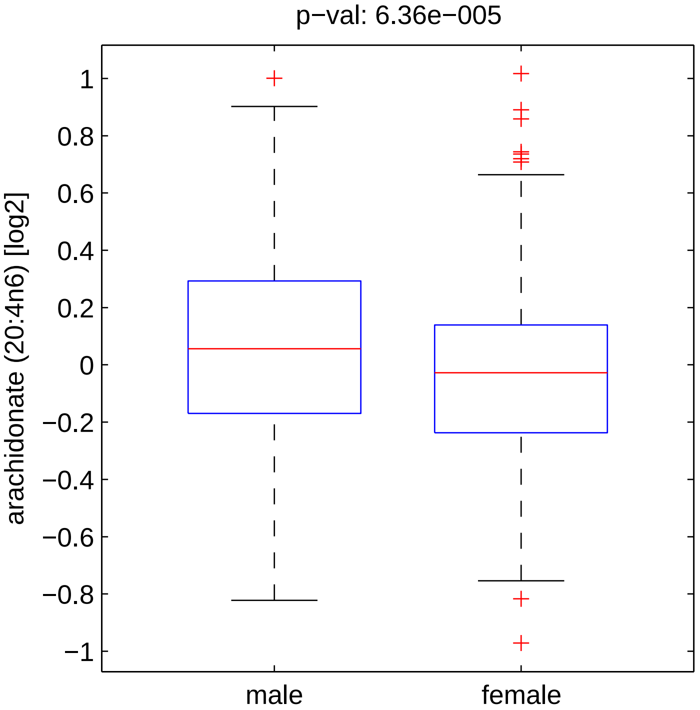
p−val: 6.36e−005
arachidonate (20:4n6) [log2]
1
0.8
0.6
0.4
0.2
0
−0.2
−0.4
−0.6
−0.8
−1
male
female

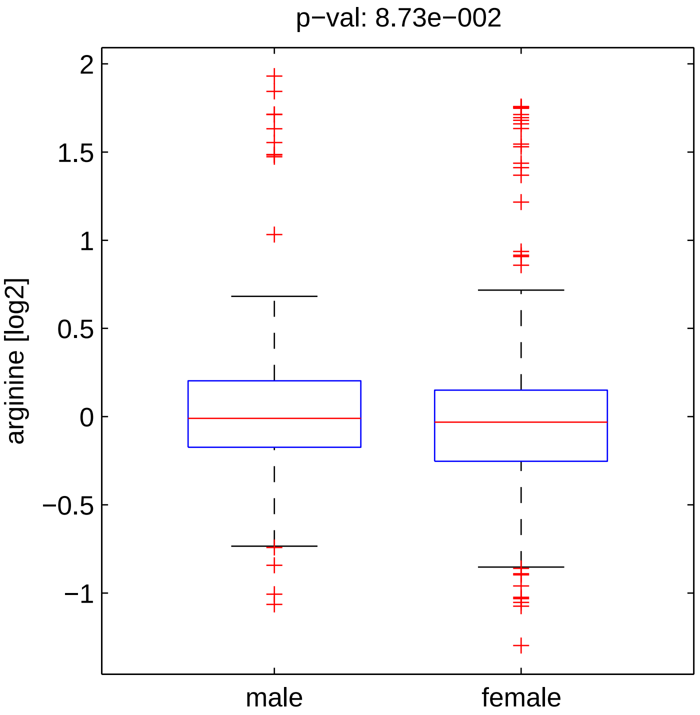
p−val: 8.73e−002
arginine [log2]
2
1.5
1
0.5
0
−0.5
−1
male
female

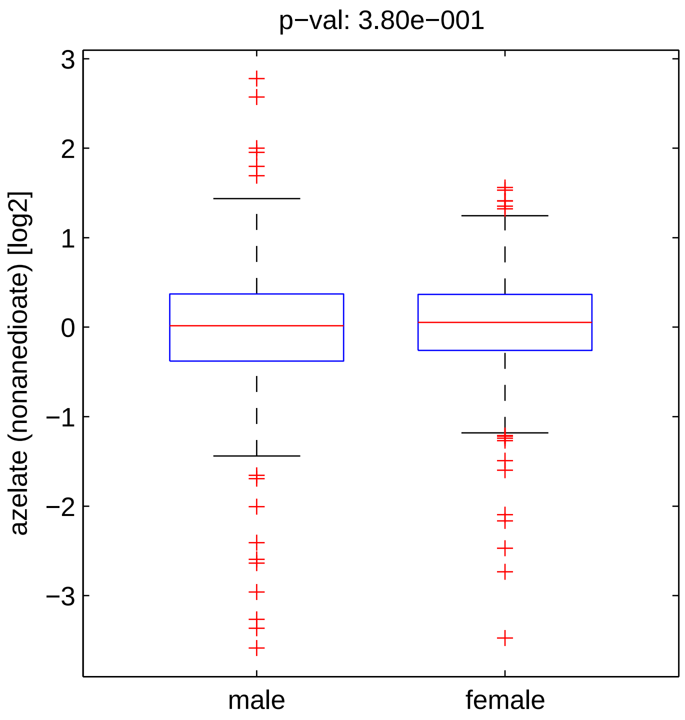

p−val: 3.80e−001
azelate (nonanedioate) [log2]
3
2
1
0
−1
−2
−3
male
female

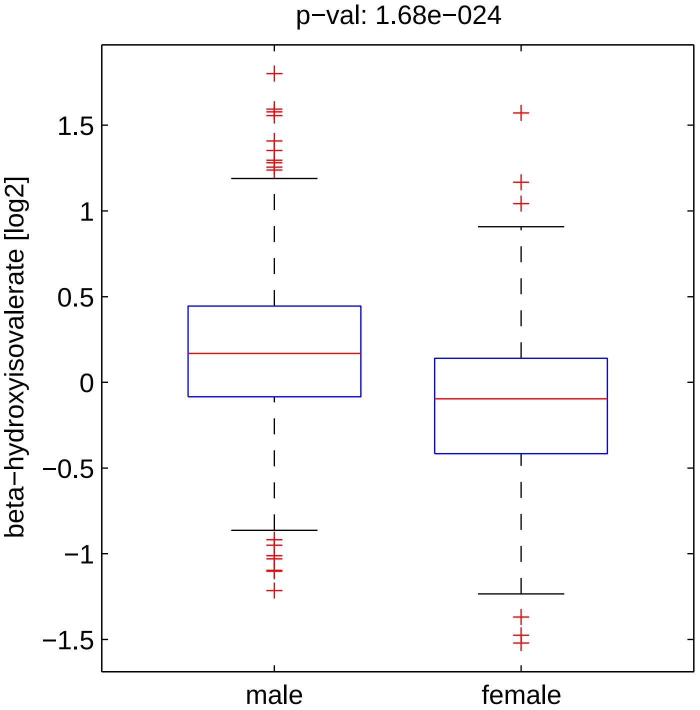
p−val: 1.68e−024
beta−hydroxyisovalerate [log2]
1.5
1
0.5
0
−0.5
−1
−1.5
male
female

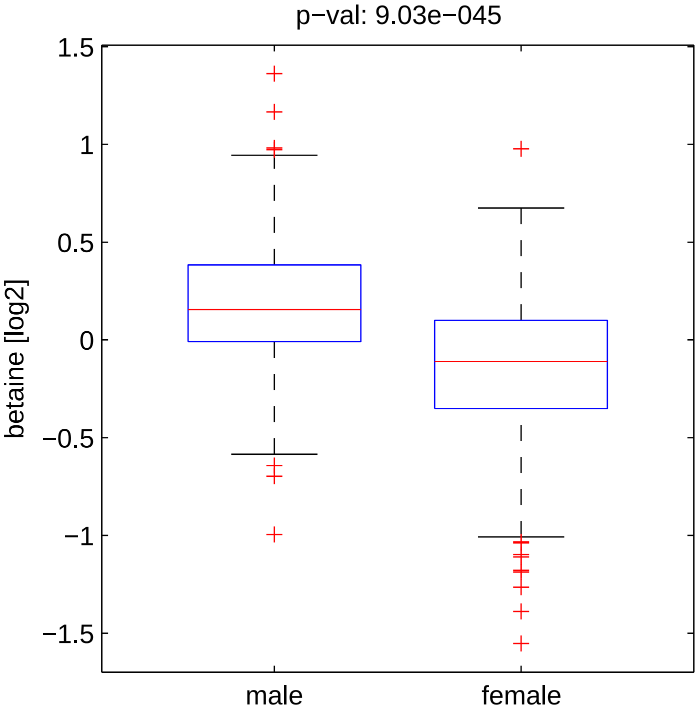
p−val: 9.03e−045
1.5
1
0.5
0
−0.5
−1
−1.5
betaine [log2]
male
female

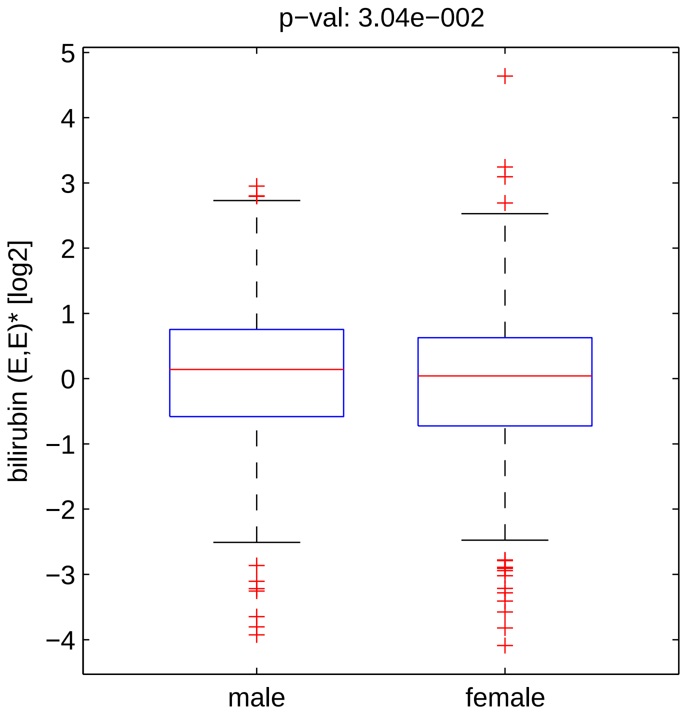
p−val: 3.04e−002
bilirubin (E,E)* [log2]
5
4
3
2
1
0
−1
−2
−3
−4
male
female

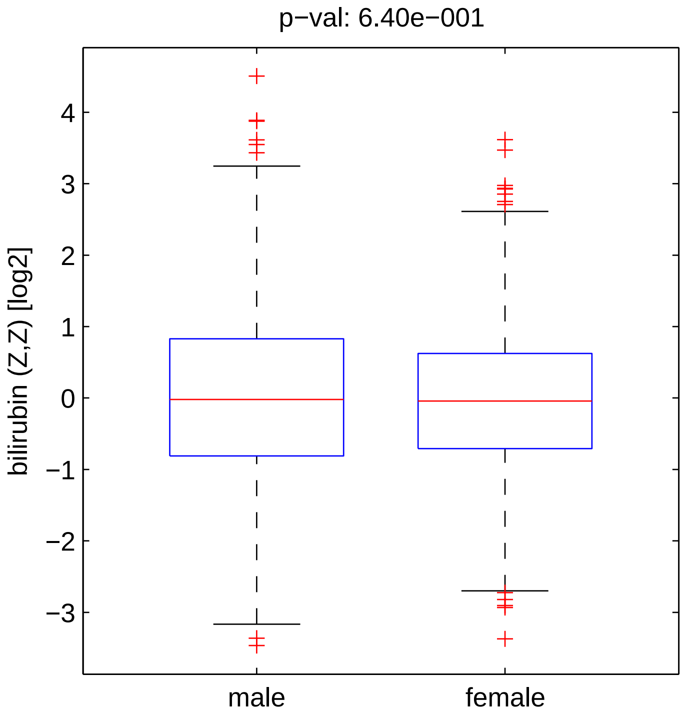
p−val: 6.40e−001
bilirubin (Z,Z) [log2]
4
3
2
1
0
−1
−2
−3
male
female

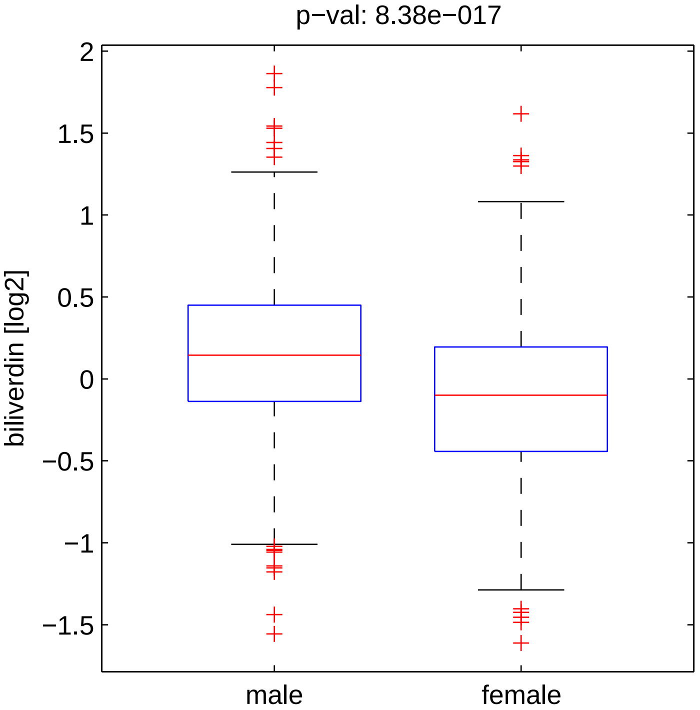

p−val: 8.38e−017
biliverdin [log2]
2
1.5
1
0.5
0
−0.5
−1
−1.5
male
female

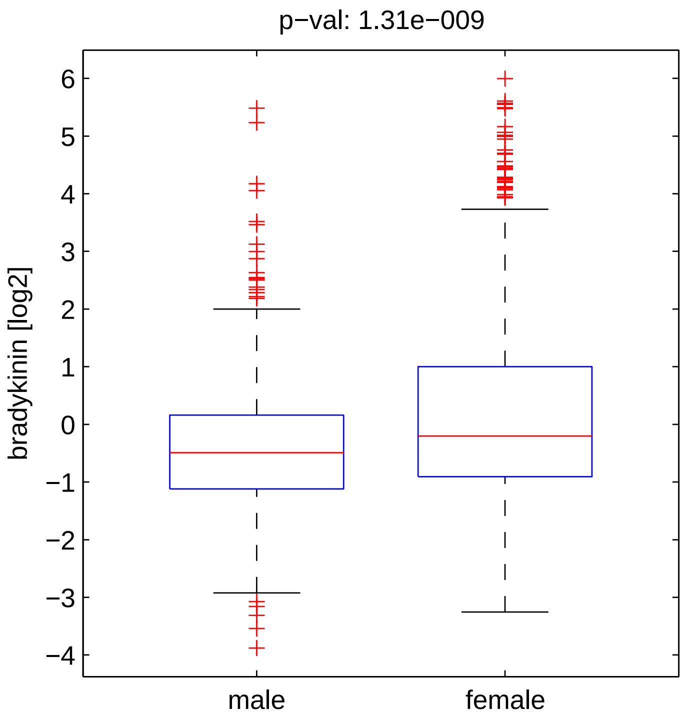
p−val: 1.31e−009
bradykinin [log2]
6
5
4
3
2
1
0
−1
−2
−3
−4
male
female

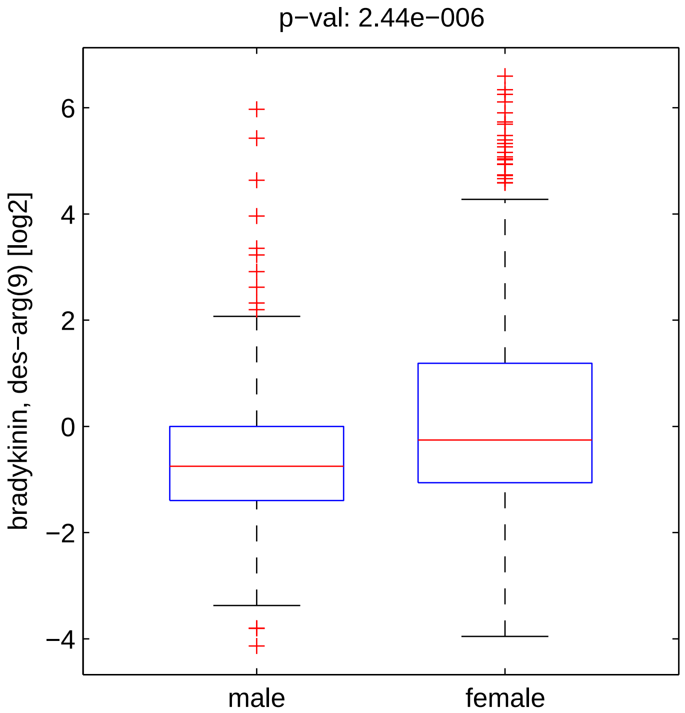

p−val: 2.44e−006
bradykinin, des−arg(9) [log2]
6
4
2
0
−2
−4
male
female

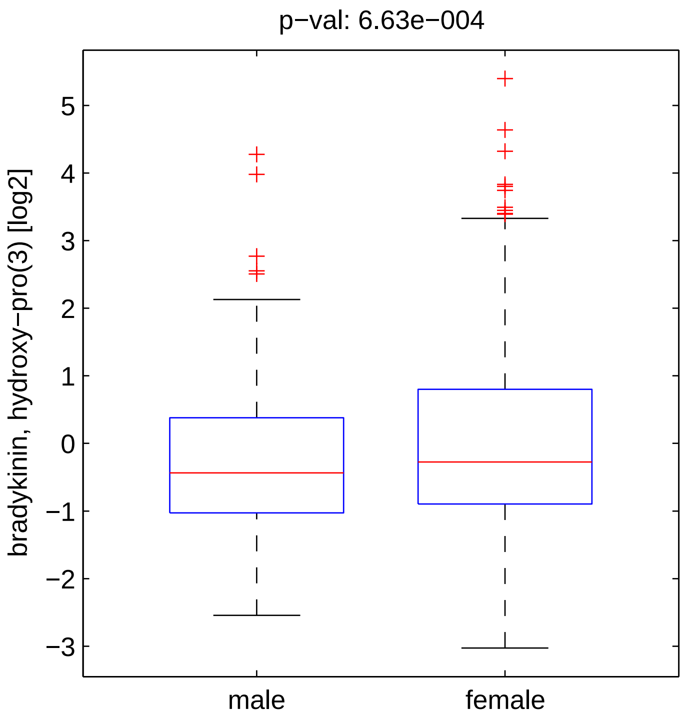

p−val: 6.63e−004
bradykinin, hydroxy−pro(3) [log2]
5
4
3
2
1
0
−1
−2
−3
male
female

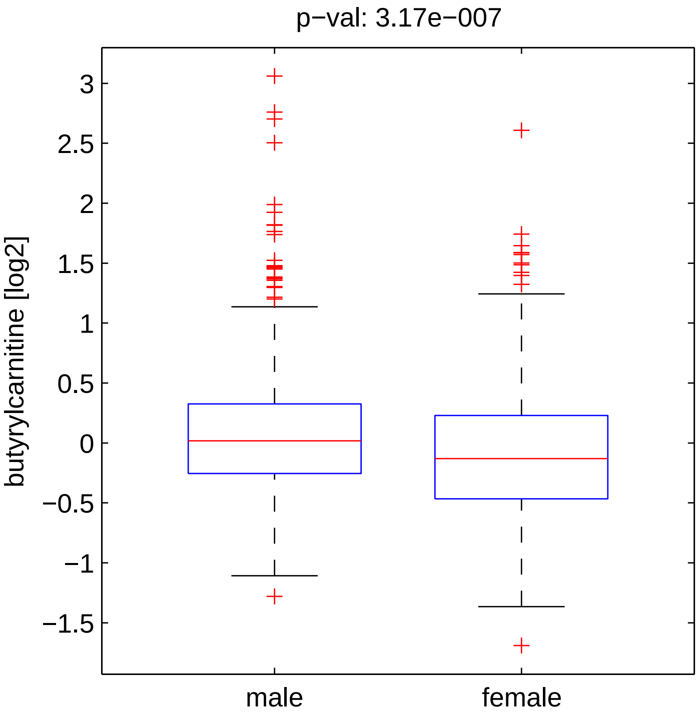
p−val: 3.17e−007
butyrylcarnitine [log2]
3
2.5
2
1.5
1
0.5
0
−0.5
−1
−1.5
male
female

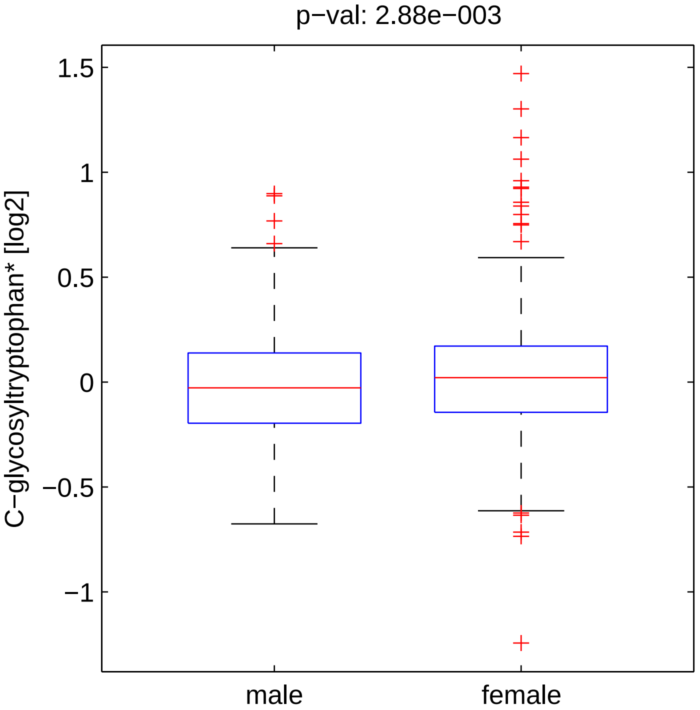

p−val: 2.88e−003
1.5
1
0.5
0
−0.5
−1
C−glycosyltryptophan* [log2]
male
female

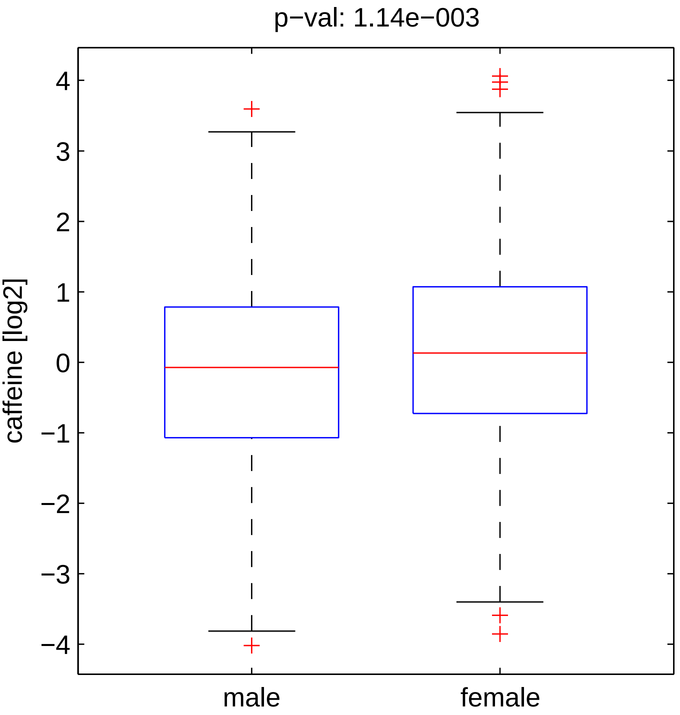
p−val: 1.14e−003
caffeine [log2]
4
3
2
1
0
−1
−2
−3
−4
male
female

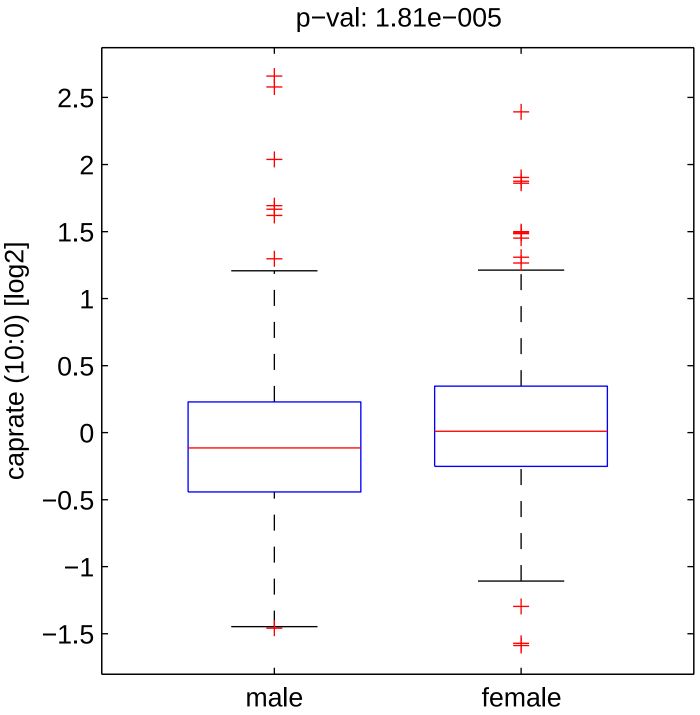
p−val: 1.81e−005
caprate (10:0) [log2]
2.5
2
1.5
1
0.5
0
−0.5
−1
−1.5
male
female

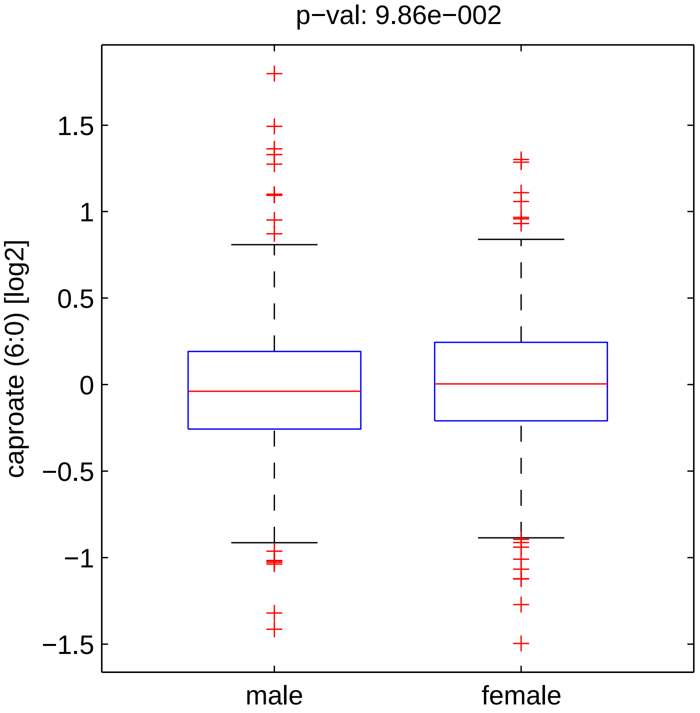
p−val: 9.86e−002
caproate (6:0) [log2]
1.5
1
0.5
0
−0.5
−1
−1.5
male
female

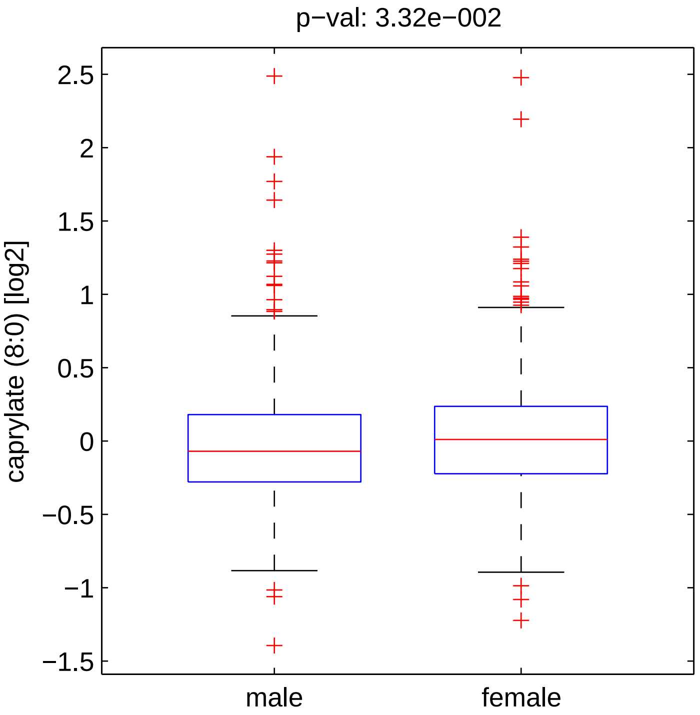

p−val: 3.32e−002
caprylate (8:0) [log2]
2.5
2
1.5
1
0.5
0
−0.5
−1
−1.5
male
female

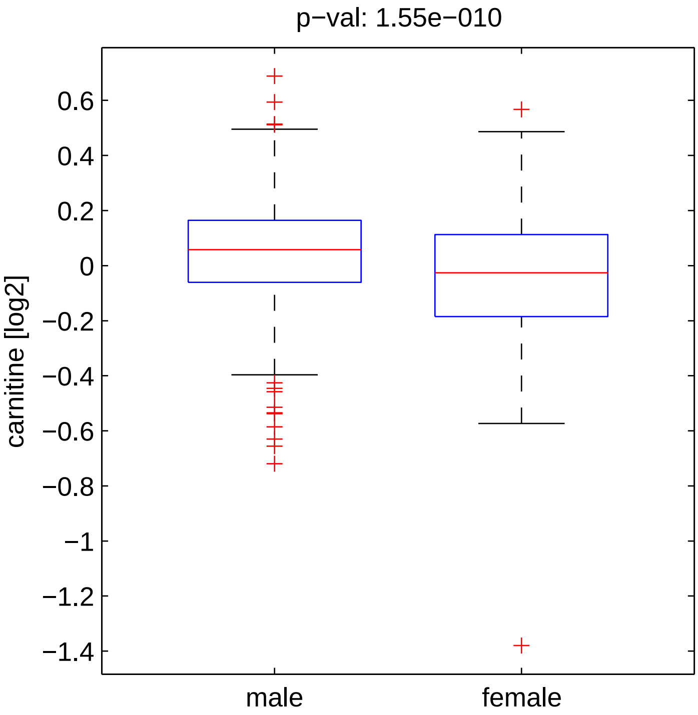
p−val: 1.55e−010
carnitine [log2]
0.6
0.4
0.2
0
−0.2
−0.4
−0.6
−0.8
−1
−1.2
−1.4
male
female

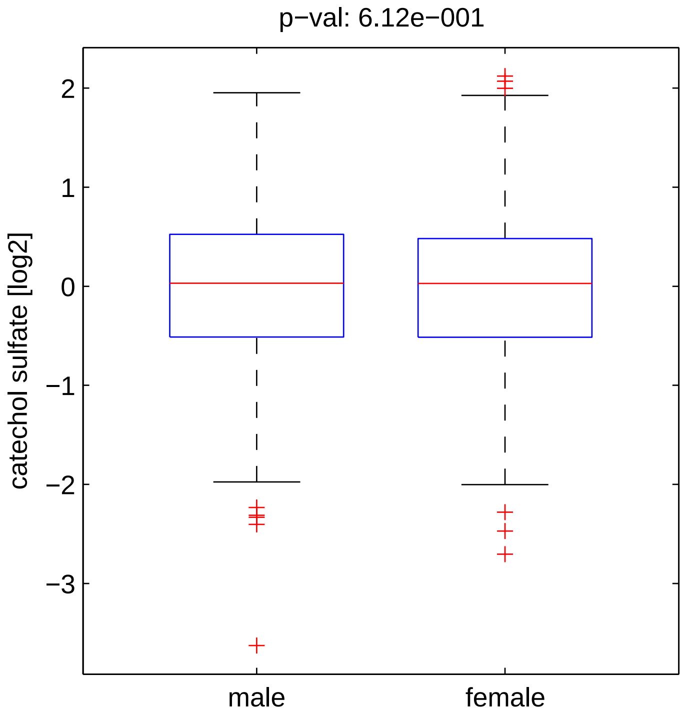

p−val: 6.12e−001
catechol sulfate [log2]
2
1
0
−1
−2
−3
male
female

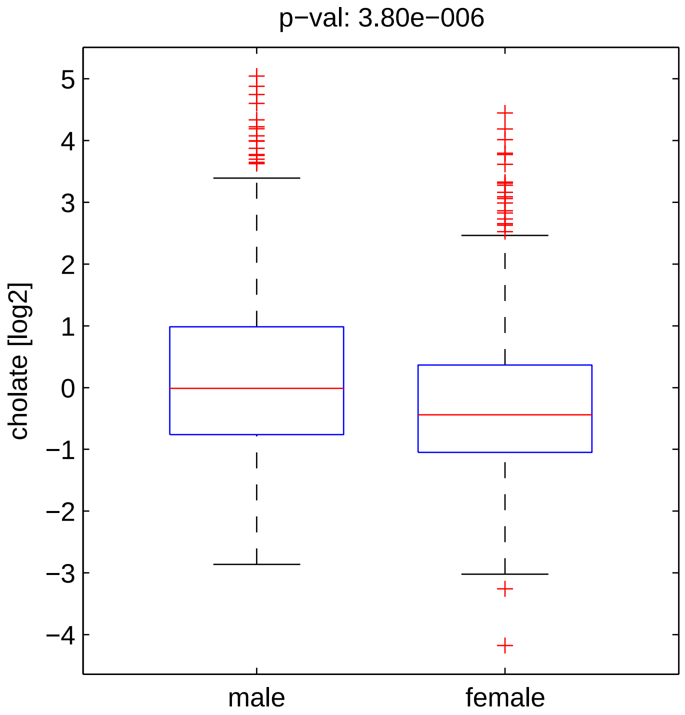

p−val: 3.80e−006
cholate [log2]
5
4
3
2
1
0
−1
−2
−3
−4
male
female

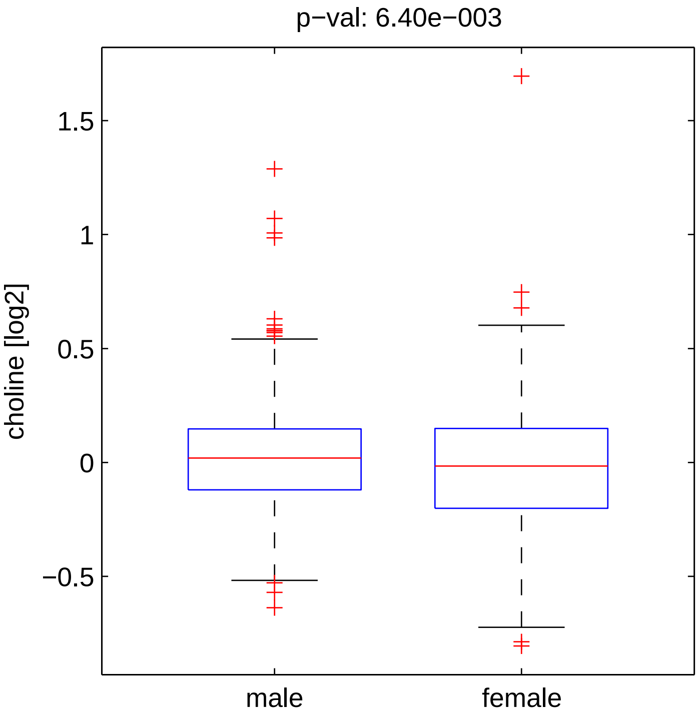
p−val: 6.40e−003
choline [log2]
1.5
1
0.5
0
−0.5
male
female

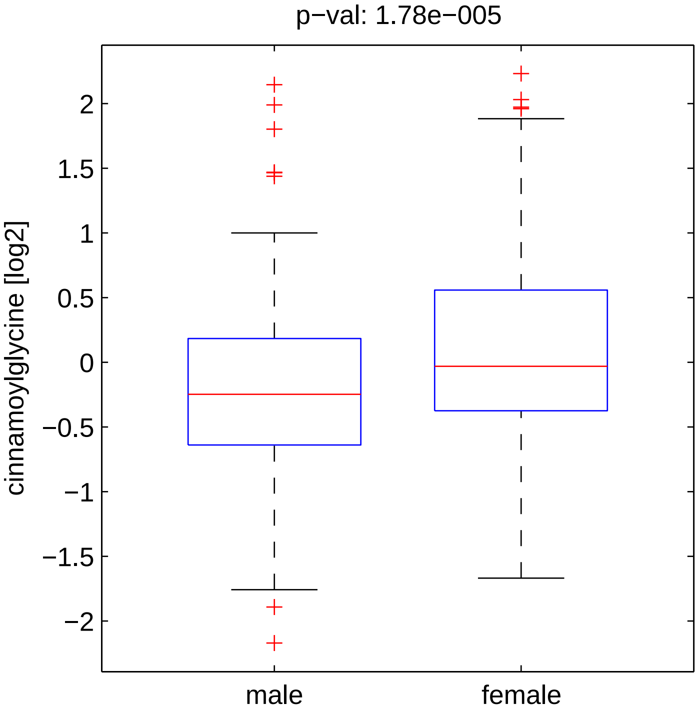

p−val: 1.78e−005
cinnamoylglycine [log2]
2
1.5
1
0.5
0
−0.5
−1
−1.5
−2
male
female

p−val: 3.11e−003

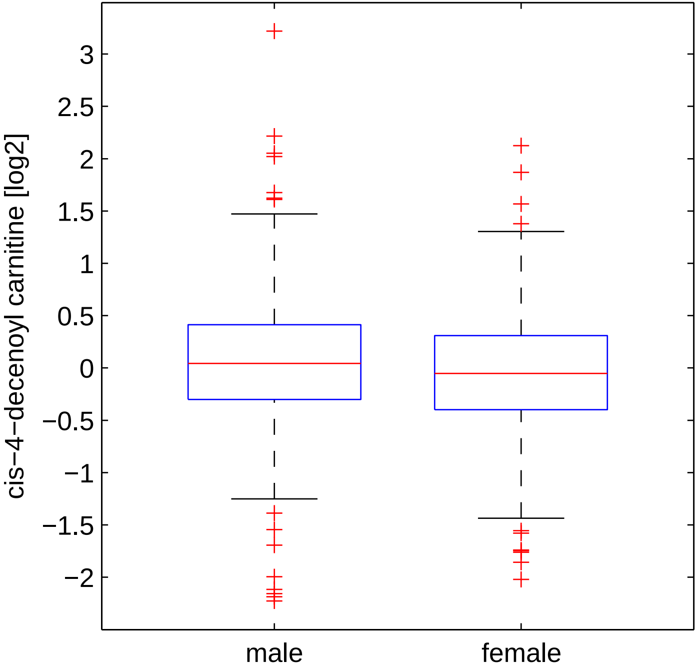

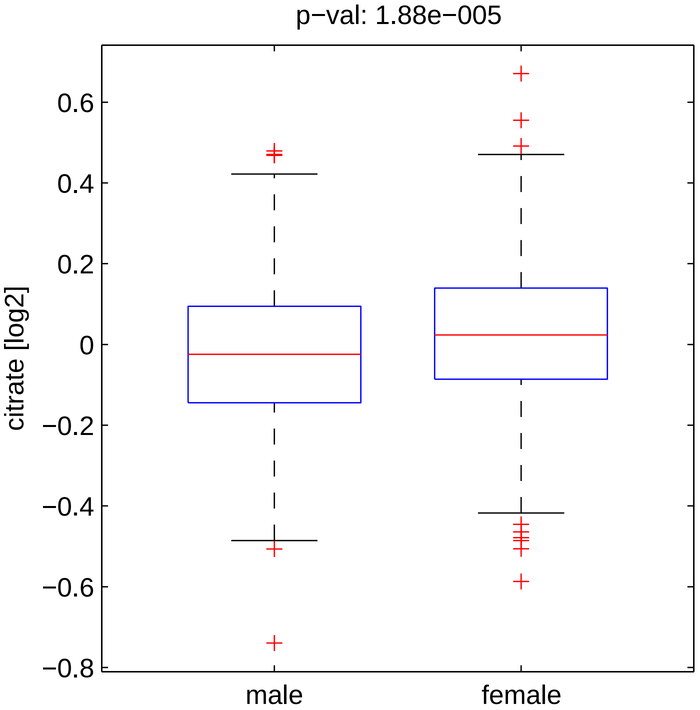
p−val: 1.88e−005
citrate [log2]
0.6
0.4
0.2
0
−0.2
−0.4
−0.6
−0.8
male
female

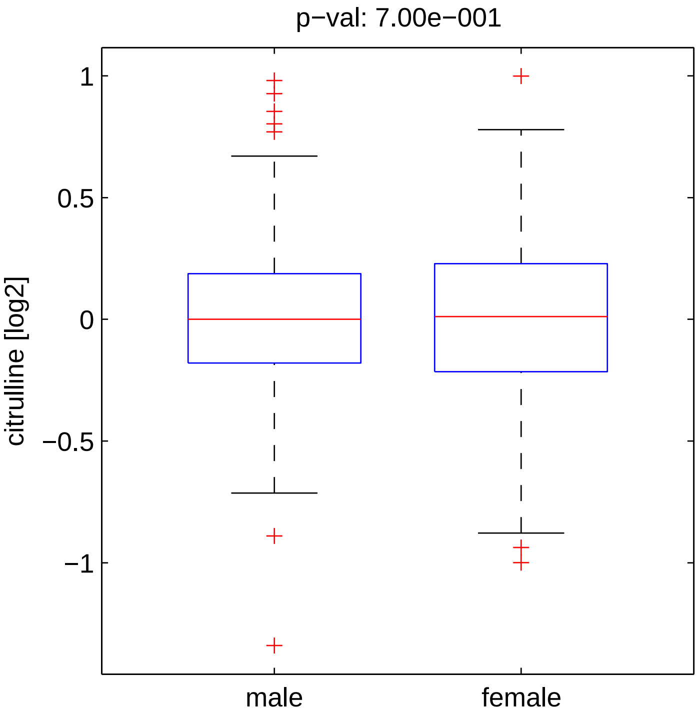

p−val: 7.00e−001
1
0.5
0
−0.5
−1
citrulline [log2]
male
female

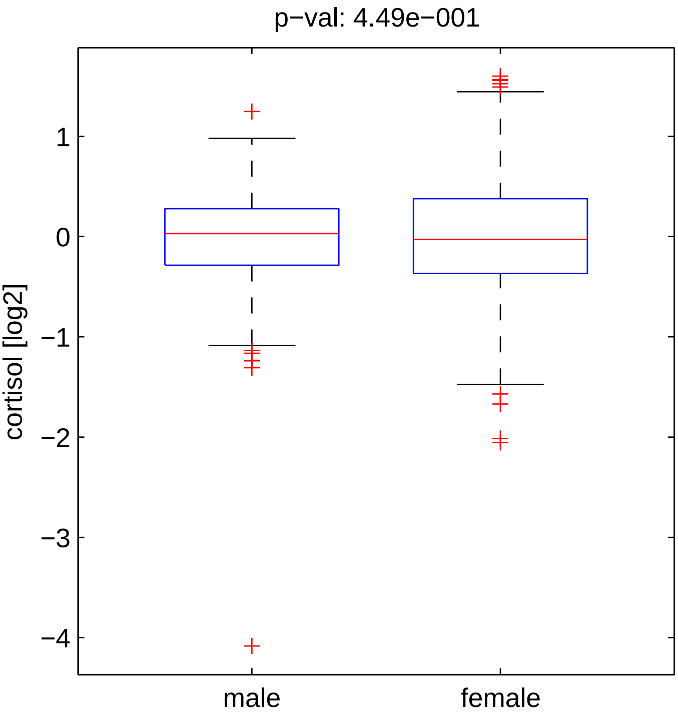
p−val: 4.49e−001
1
0
−1
−2
−3
−4
cortisol [log2]
male
female

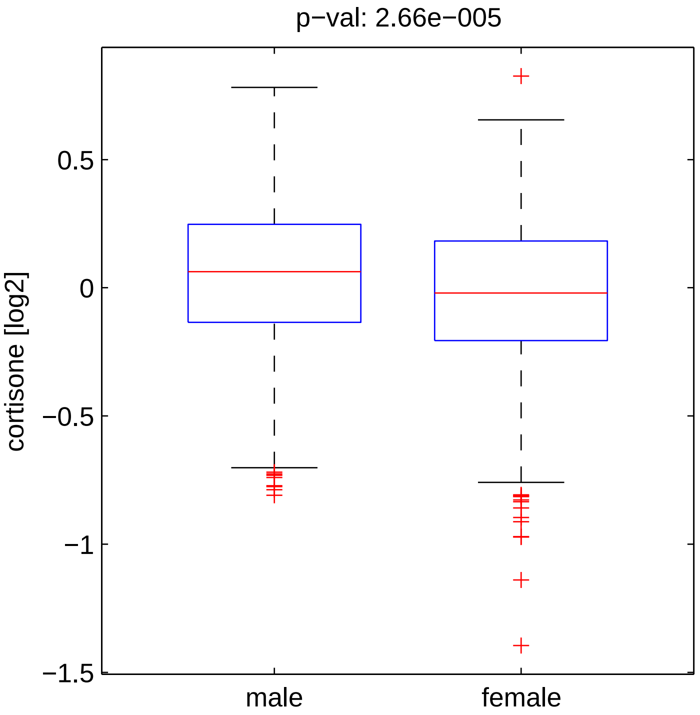

p−val: 2.66e−005
cortisone [log2]
0.5
0
−0.5
−1
−1.5
male
female

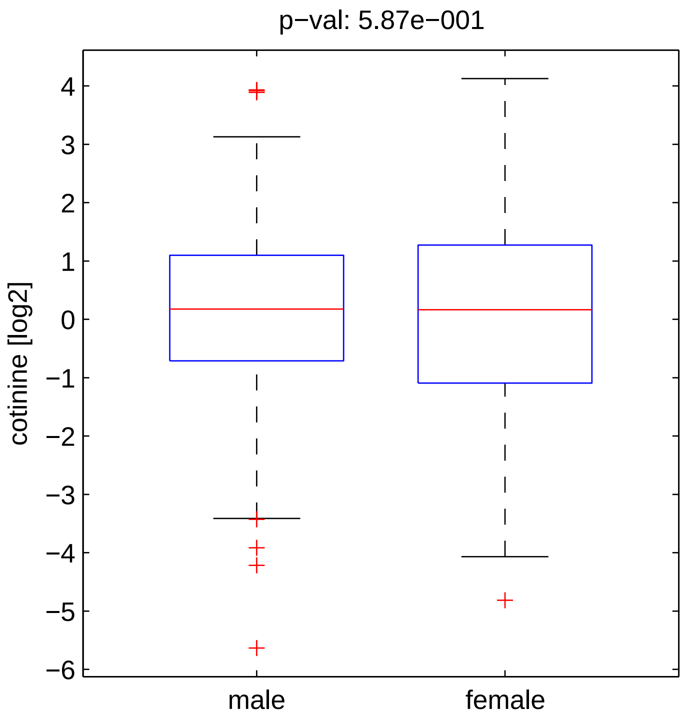

p−val: 5.87e−001
cotinine [log2]
4
3
2
1
0
−1
−2
−3
−4
−5
−6
male
female

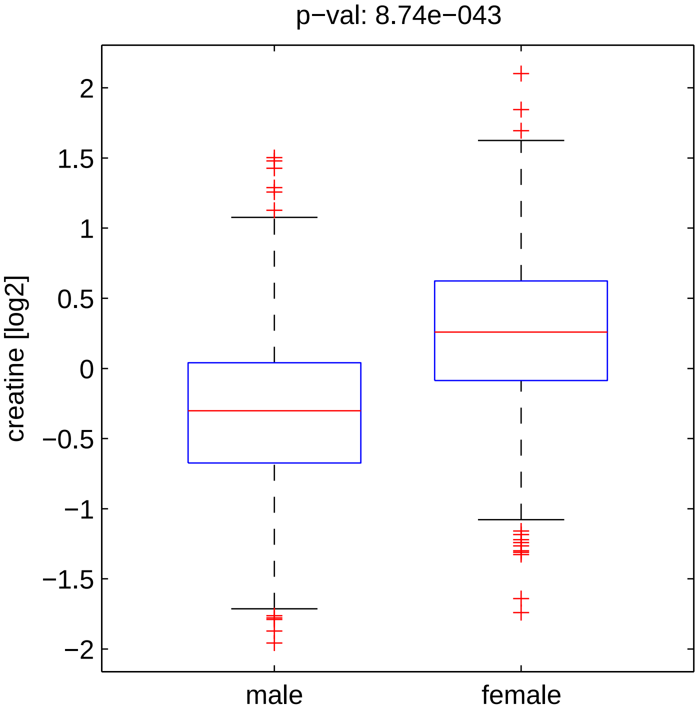

p−val: 8.74e−043
creatine [log2]
2
1.5
1
0.5
0
−0.5
−1
−1.5
−2
male
female

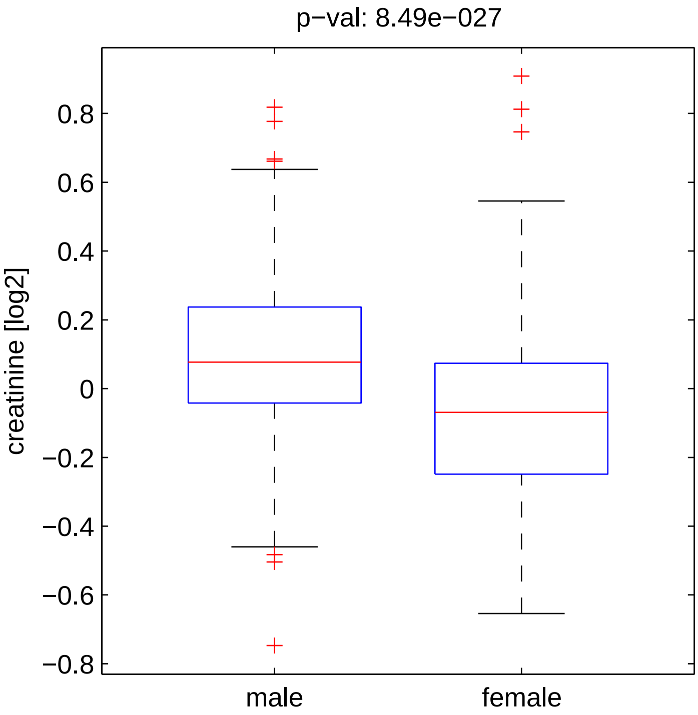
p−val: 8.49e−027
creatinine [log2]
0.8
0.6
0.4
0.2
0
−0.2
−0.4
−0.6
−0.8
male
female

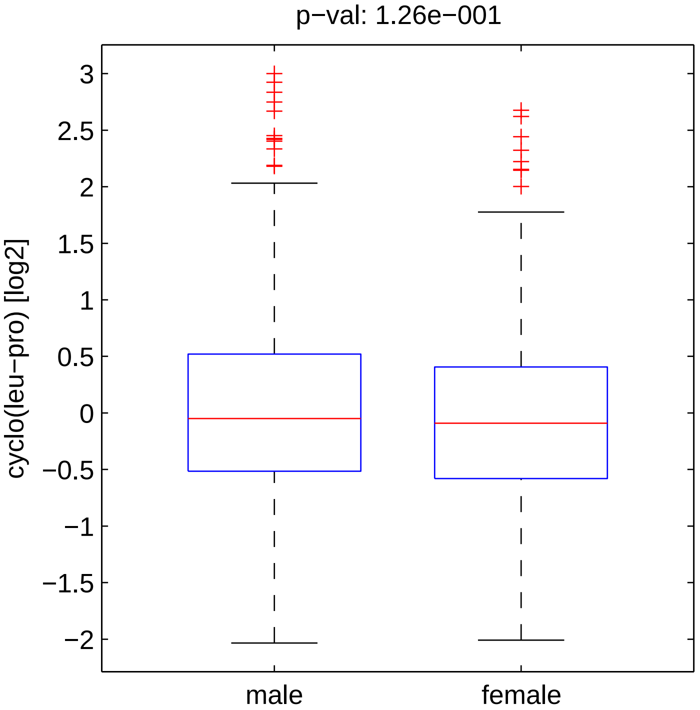
p−val: 1.26e−001
cyclo(leu−pro) [log2]
3
2.5
2
1.5
1
0.5
0
−0.5
−1
−1.5
−2
male
female

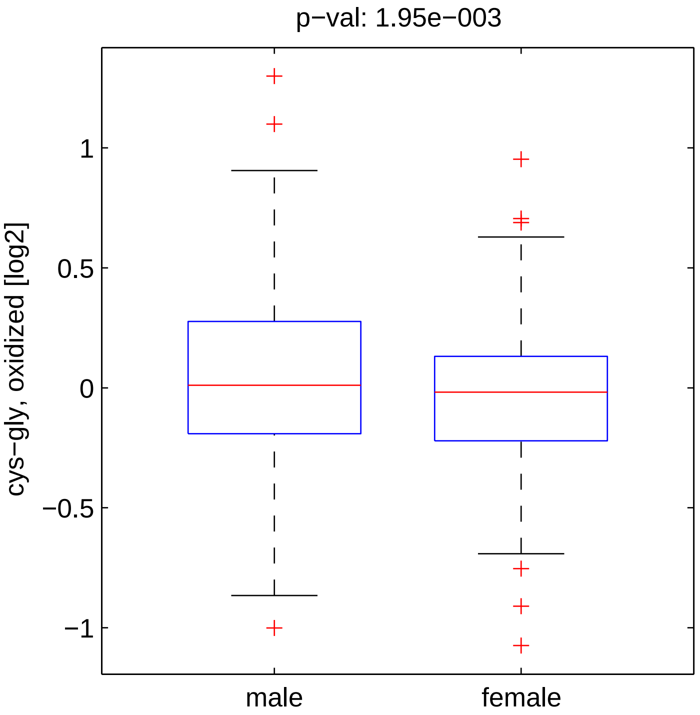

p−val: 1.95e−003
cys−gly, oxidized [log2]
1
0.5
0
−0.5
−1
male
female

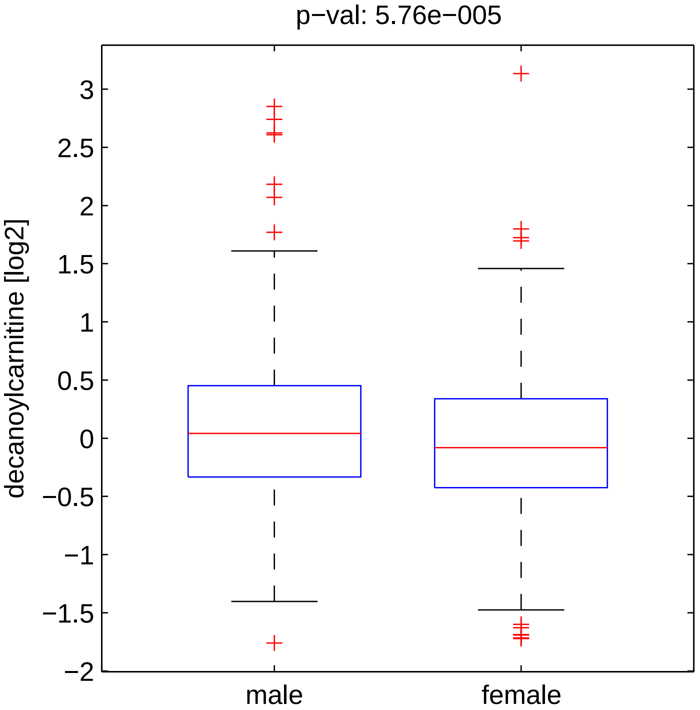

p−val: 5.76e−005
decanoylcarnitine [log2]
3
2.5
2
1.5
1
0.5
0
−0.5
−1
−1.5
−2
male
female

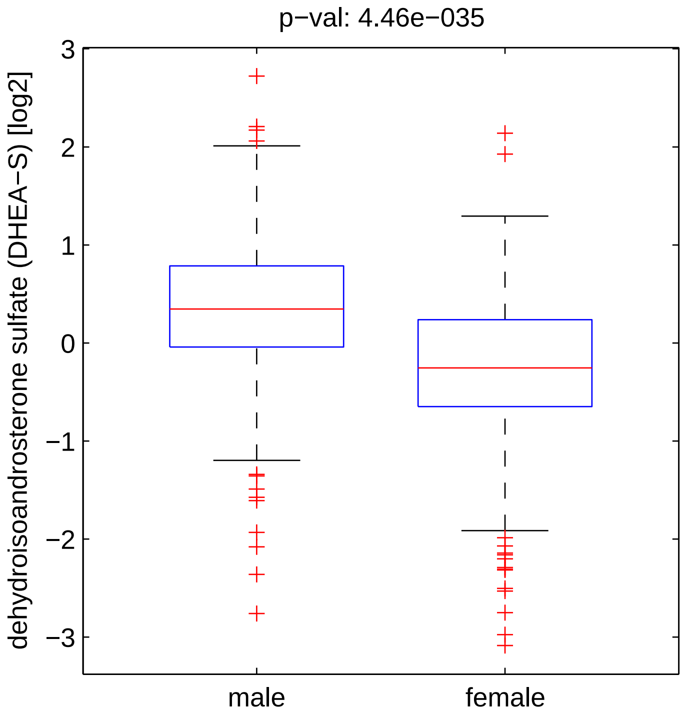
p−val: 4.46e−035
dehydroisoandrosterone sulfate (DHEA−S) [log2]
3
2
1
0
−1
−2
−3
male
female

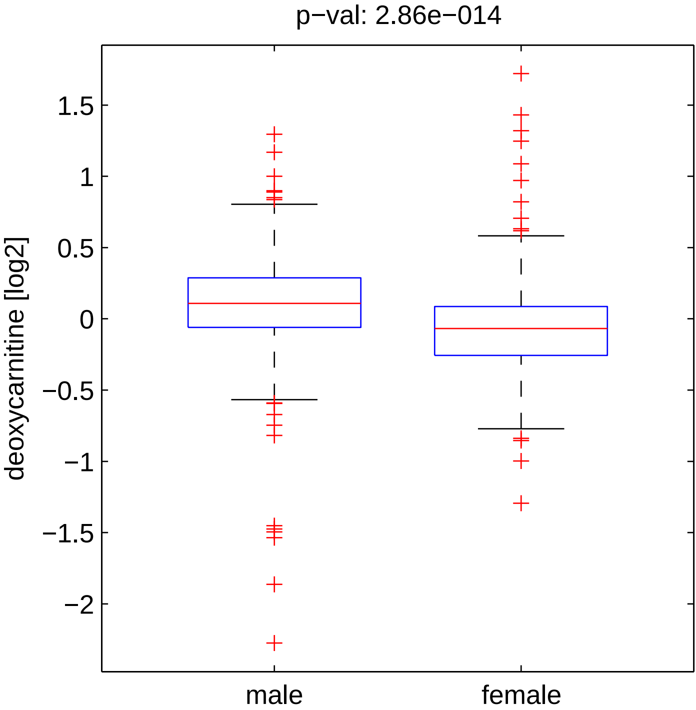
p−val: 2.86e−014
deoxycarnitine [log2]
1.5
1
0.5
0
−0.5
−1
−1.5
−2
male
female

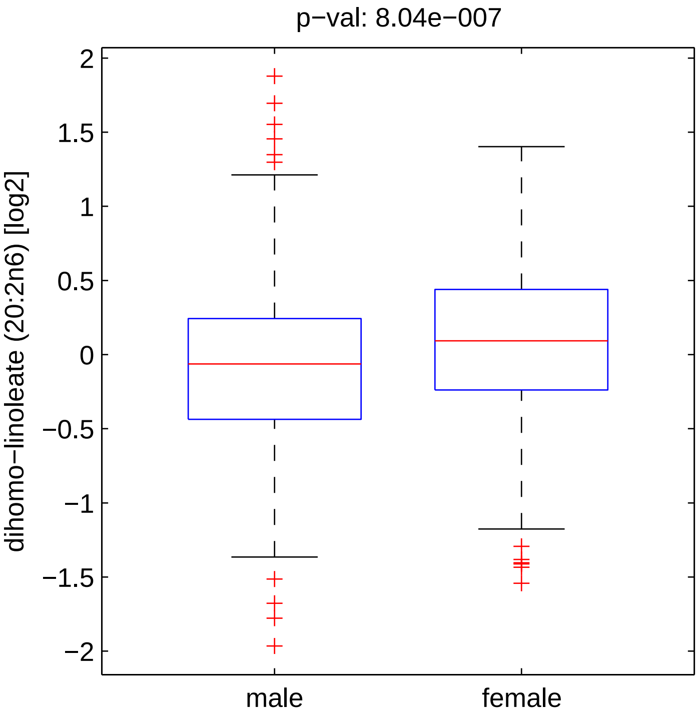

p−val: 8.04e−007
dihomo−linoleate (20:2n6) [log2]
2
1.5
1
0.5
0
−0.5
−1
−1.5
−2
male
female

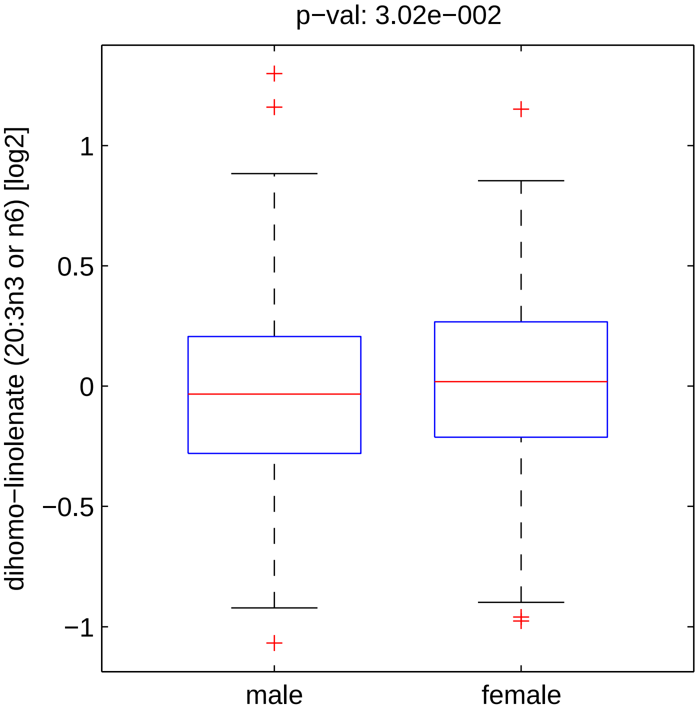
p−val: 3.02e−002
dihomo−linolenate (20:3n3 or n6) [log2]
1
0.5
0
−0.5
−1
male
female

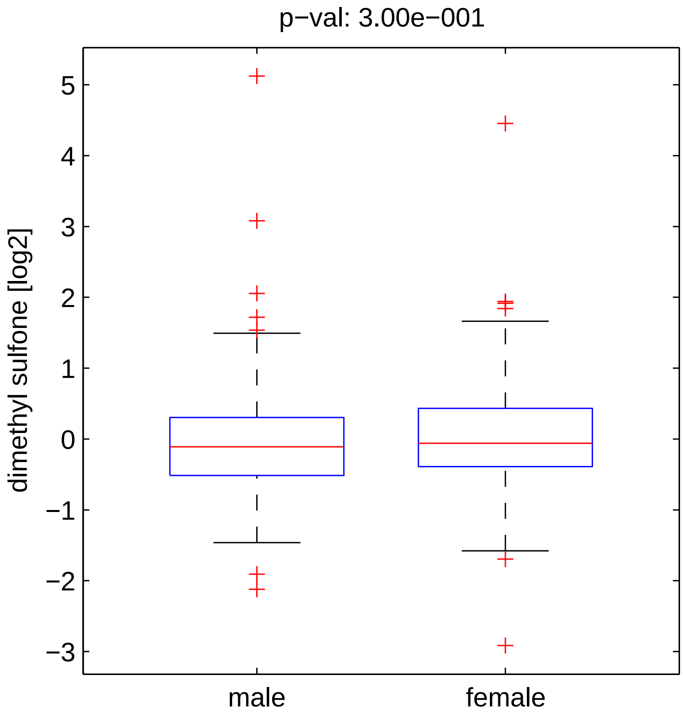
p−val: 3.00e−001
dimethyl sulfone [log2]
5
4
3
2
1
0
−1
−2
−3
male
female

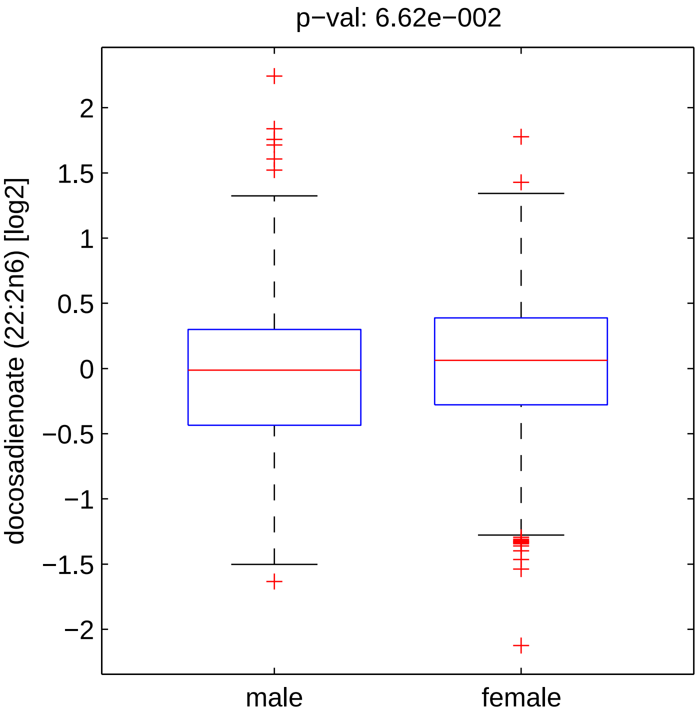

p−val: 6.62e−002
docosadienoate (22:2n6) [log2]
2
1.5
1
0.5
0
−0.5
−1
−1.5
−2
male
female

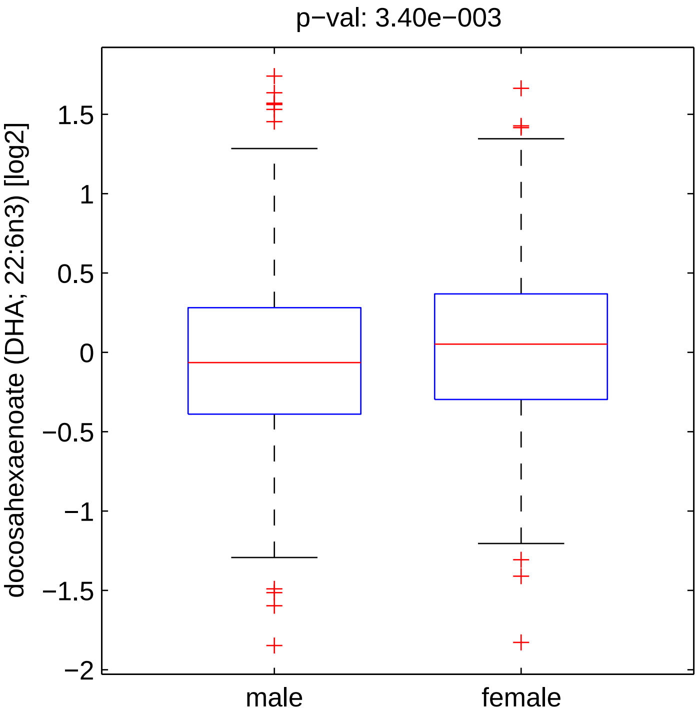
p−val: 3.40e−003
docosahexaenoate (DHA; 22:6n3) [log2]
1.5
1
0.5
0
−0.5
−1
−1.5
−2
male
female

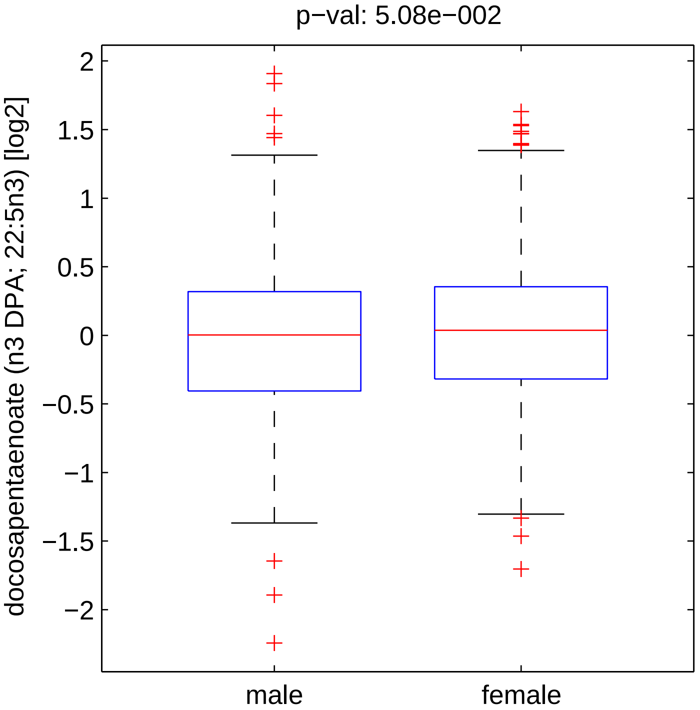

p−val: 5.08e−002
docosapentaenoate (n3 DPA; 22:5n3) [log2]
2
1.5
1
0.5
0
−0.5
−1
−1.5
−2
male
female

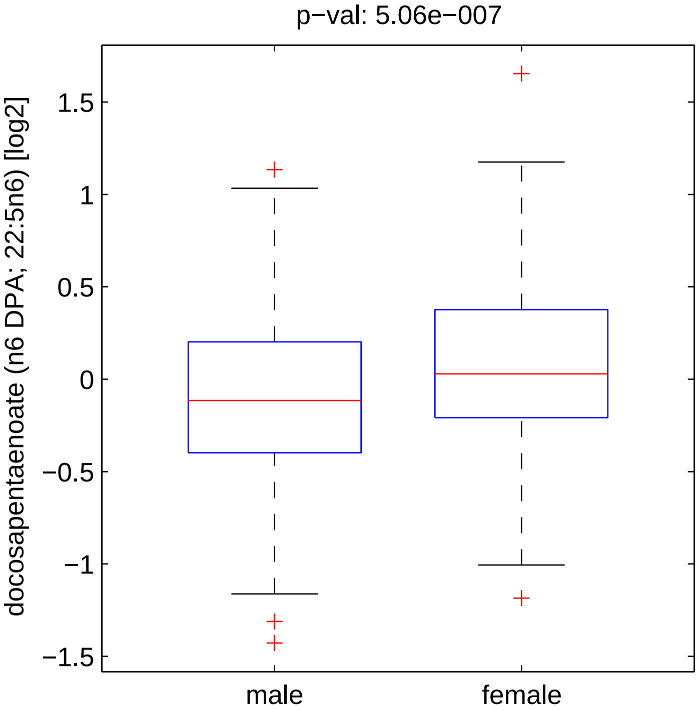

p−val: 5.06e−007
docosapentaenoate (n6 DPA; 22:5n6) [log2]
1.5
1
0.5
0
−0.5
−1
−1.5
male
female

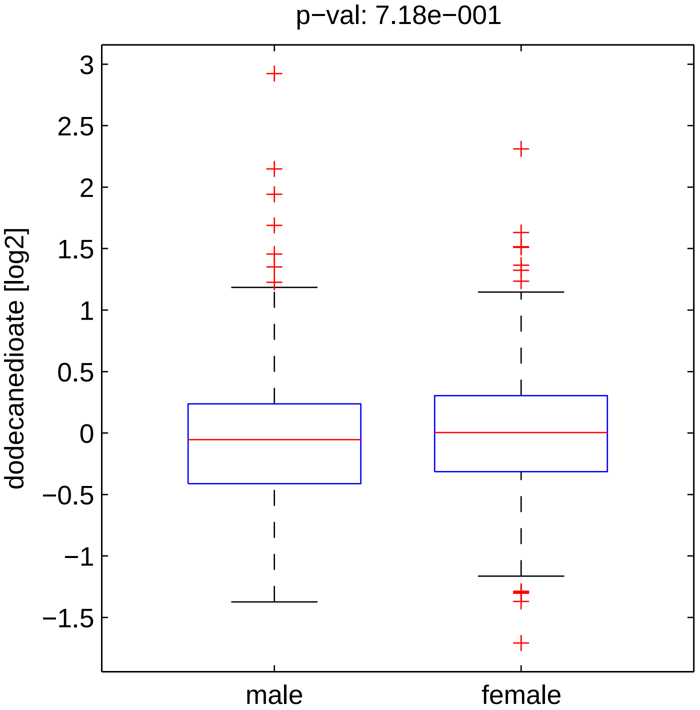
p−val: 7.18e−001
dodecanedioate [log2]
3
2.5
2
1.5
1
0.5
0
−0.5
−1
−1.5
male
female

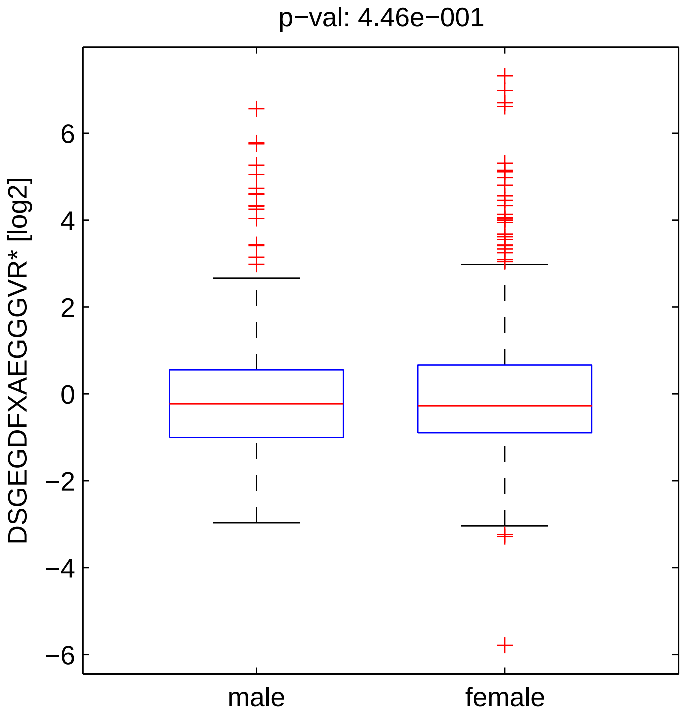

p−val: 4.46e−001
DSGEGDFXAEGGGVR* [log2]
6
4
2
0
−2
−4
−6
male
female

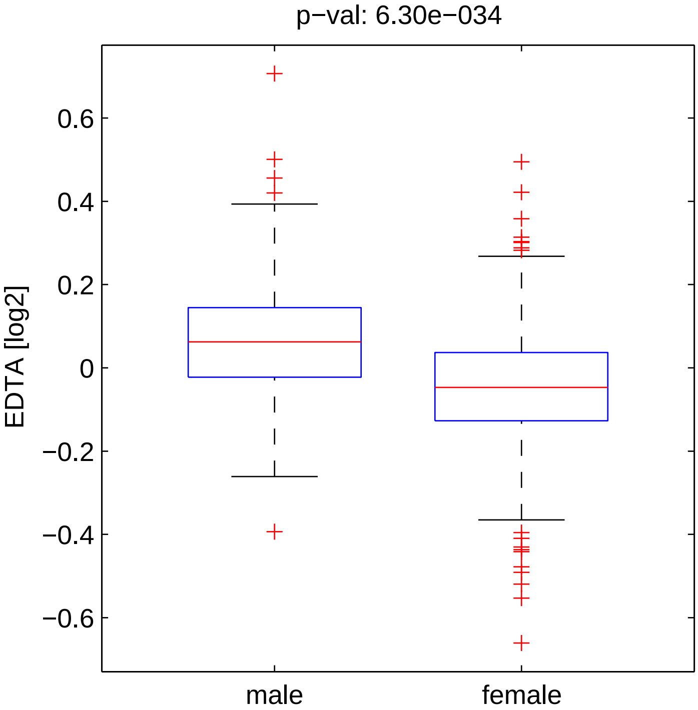
p−val: 6.30e−034
EDTA [log2]
0.6
0.4
0.2
0
−0.2
−0.4
−0.6
male
female

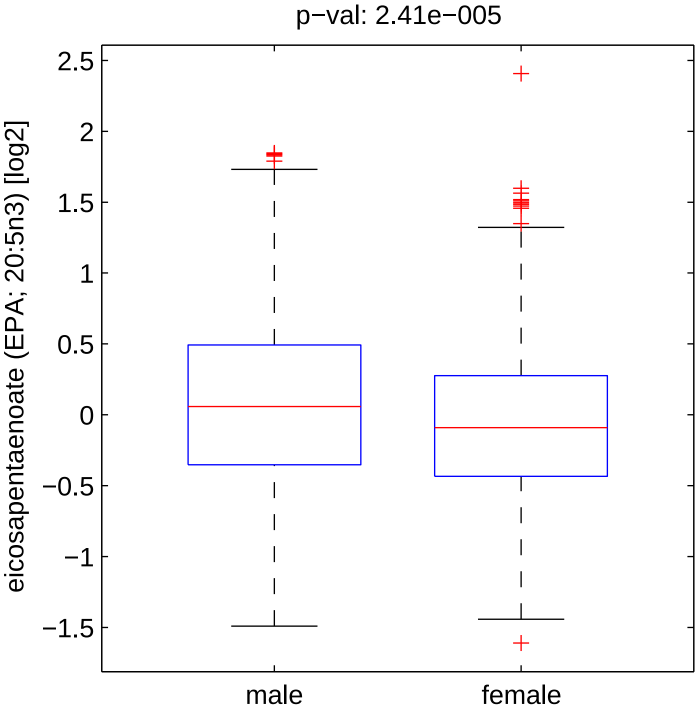
p−val: 2.41e−005
eicosapentaenoate (EPA; 20:5n3) [log2]
2.5
2
1.5
1
0.5
0
−0.5
−1
−1.5
male
female

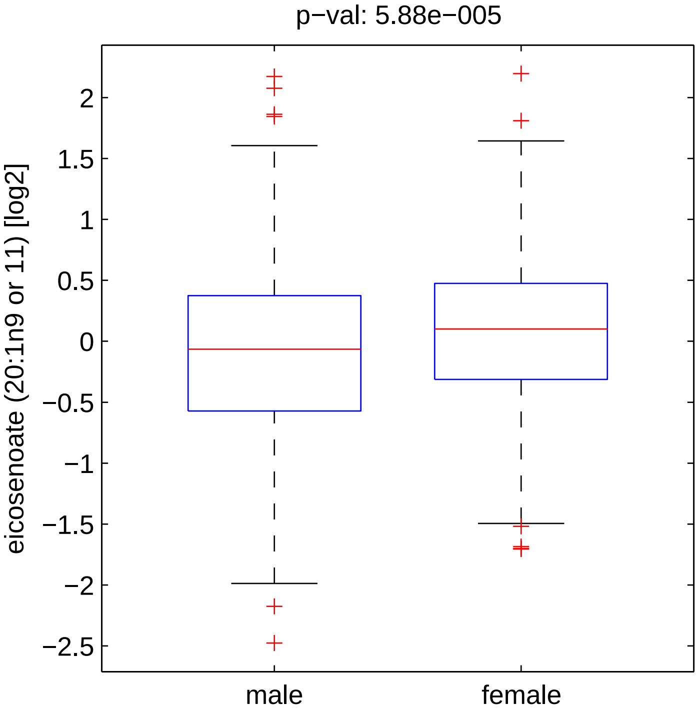

p−val: 5.88e−005
eicosenoate (20:1n9 or 11) [log2]
2
1.5
1
0.5
0
−0.5
−1
−1.5
−2
−2.5
male
female

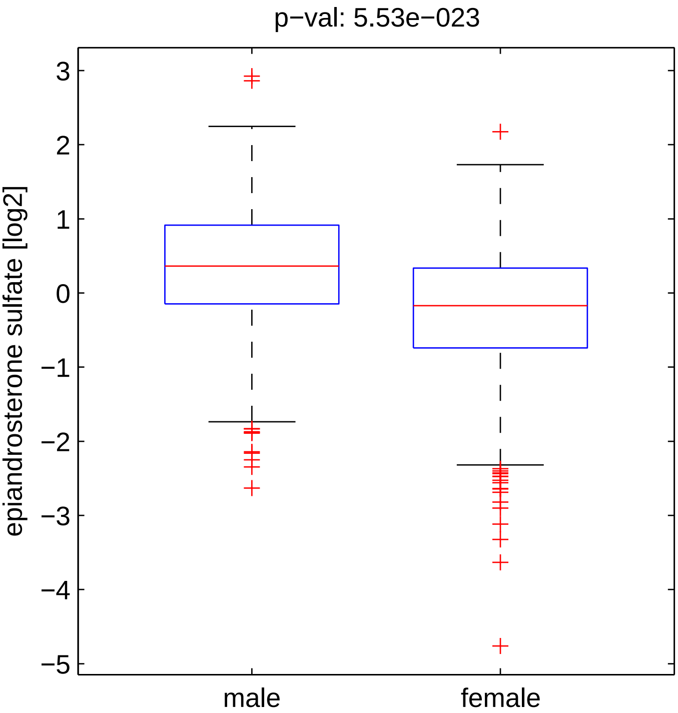
p−val: 5.53e−023
epiandrosterone sulfate [log2]
3
2
1
0
−1
−2
−3
−4
−5
male
female

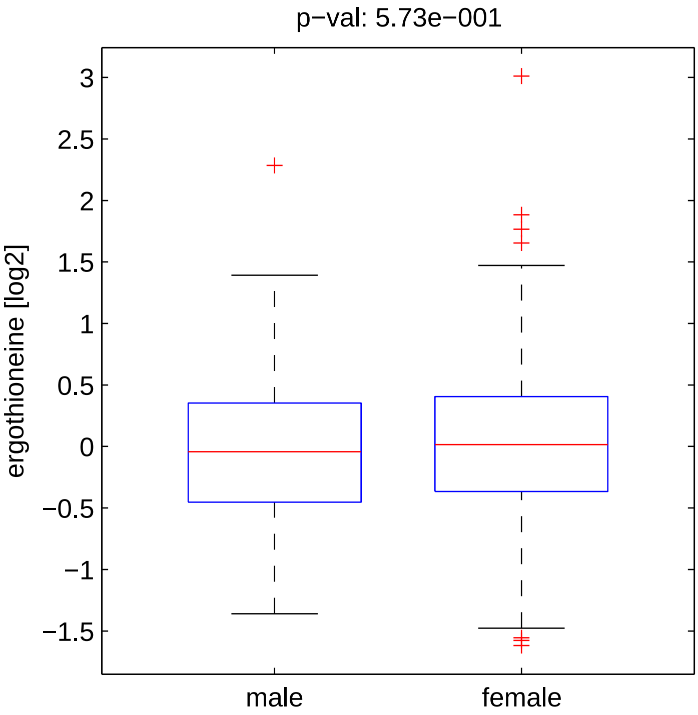

p−val: 5.73e−001
ergothioneine [log2]
3
2.5
2
1.5
1
0.5
0
−0.5
−1
−1.5
male
female

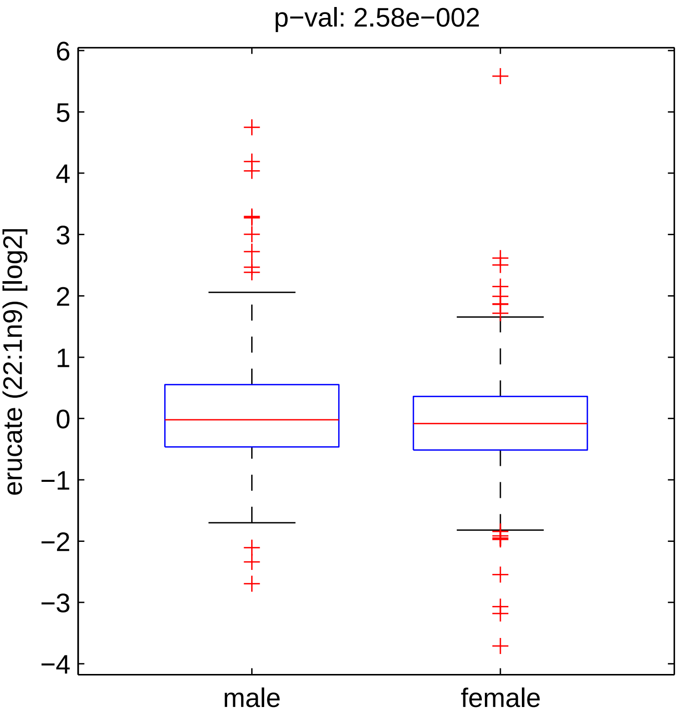
p−val: 2.58e−002
erucate (22:1n9) [log2]
6
5
4
3
2
1
0
−1
−2
−3
−4
male
female

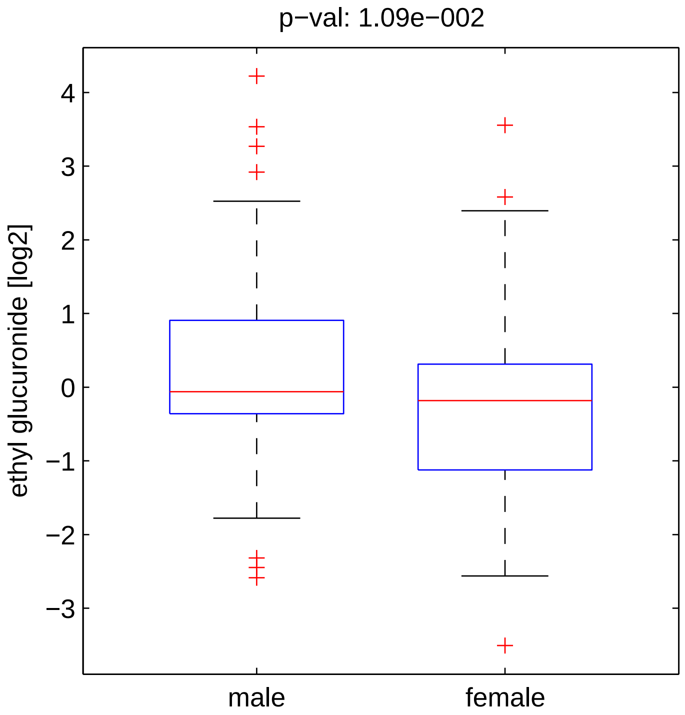
p−val: 1.09e−002
ethyl glucuronide [log2]
4
3
2
1
0
−1
−2
−3
male
female

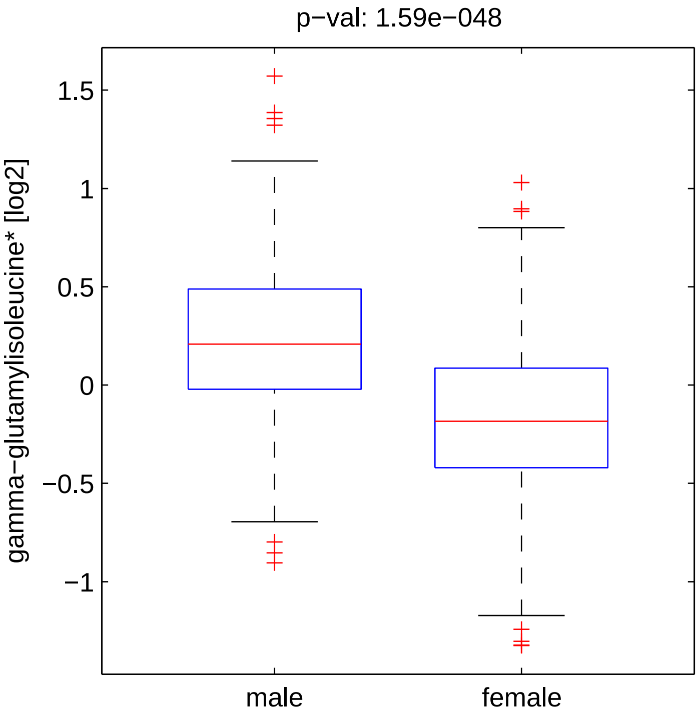
p−val: 1.59e−048
gamma−glutamylisoleucine* [log2]
1.5
1
0.5
0
−0.5
−1
male
female

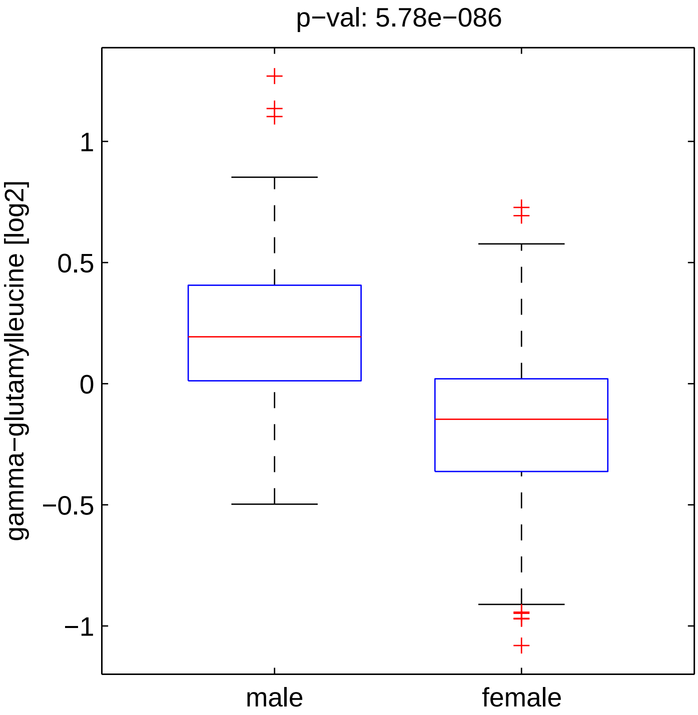
p−val: 5.78e−086
gamma−glutamylleucine [log2]
1
0.5
0
−0.5
−1
male
female

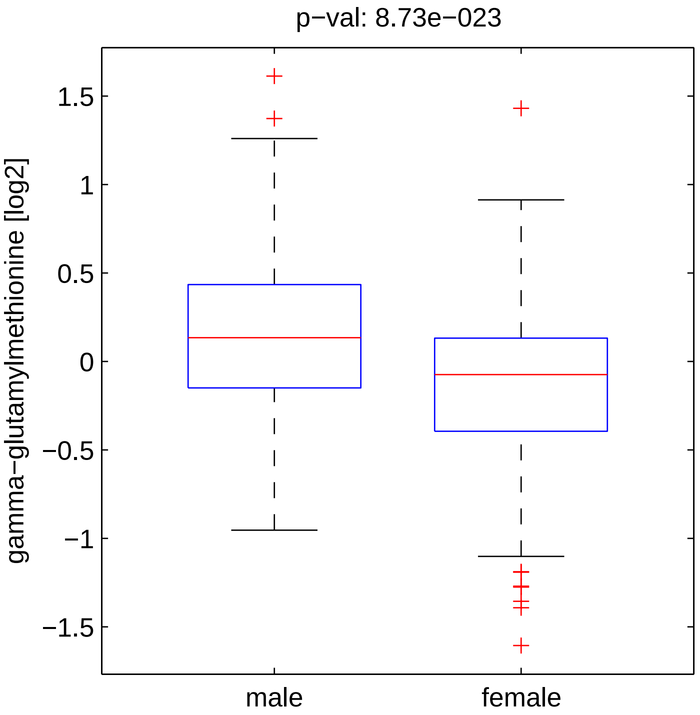

p−val: 8.73e−023
gamma−glutamylmethionine [log2]
1.5
1
0.5
0
−0.5
−1
−1.5
male
female

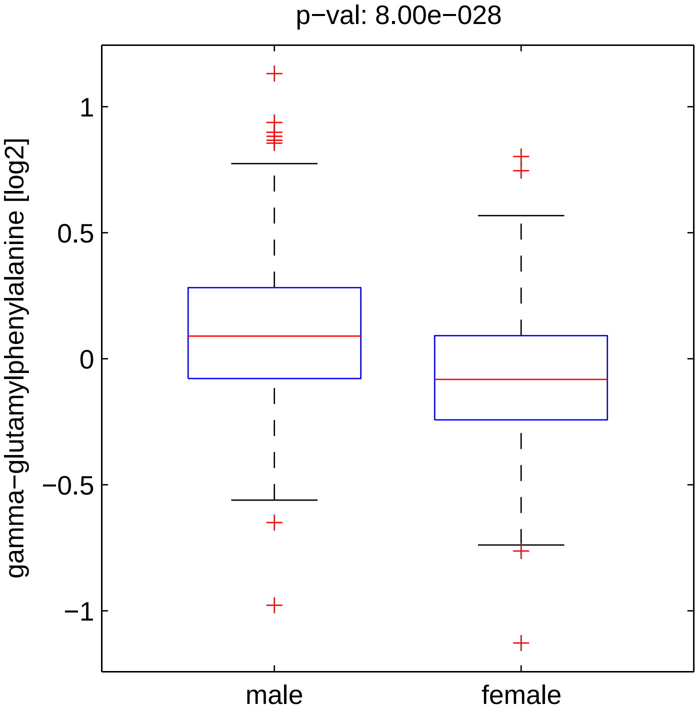
p−val: 8.00e−028
gamma−glutamylphenylalanine [log2]
1
0.5
0
−0.5
−1
male
female

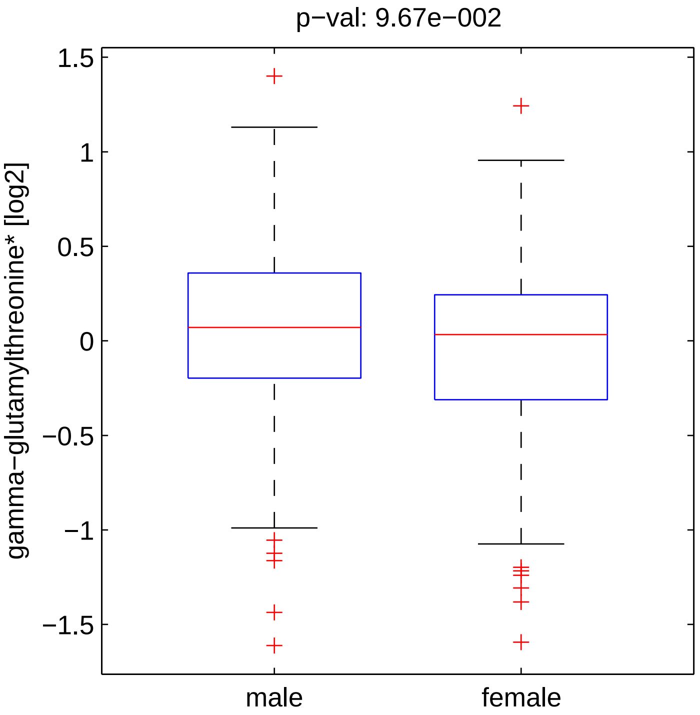
p−val: 9.67e−002
gamma−glutamylthreonine* [log2]
1.5
1
0.5
0
−0.5
−1
−1.5
male
female

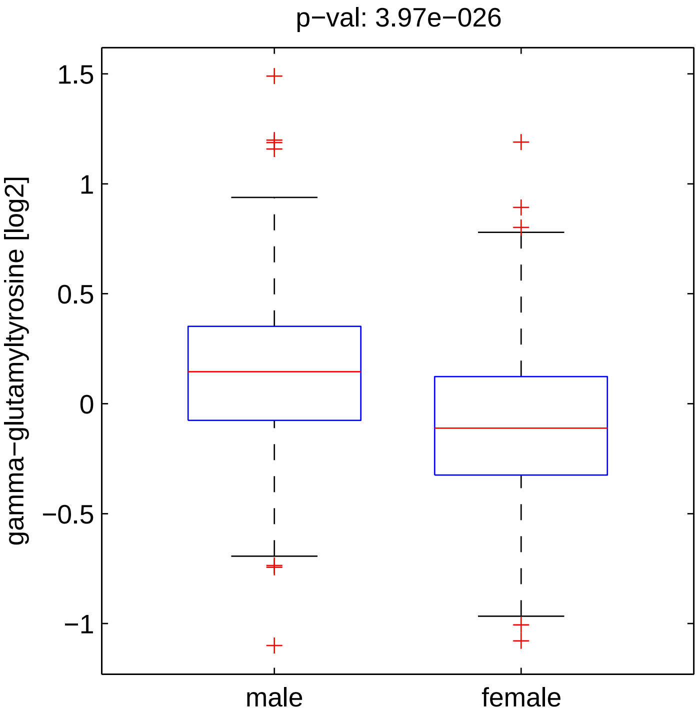

p−val: 3.97e−026
gamma−glutamyltyrosine [log2]
1.5
1
0.5
0
−0.5
−1
male
female

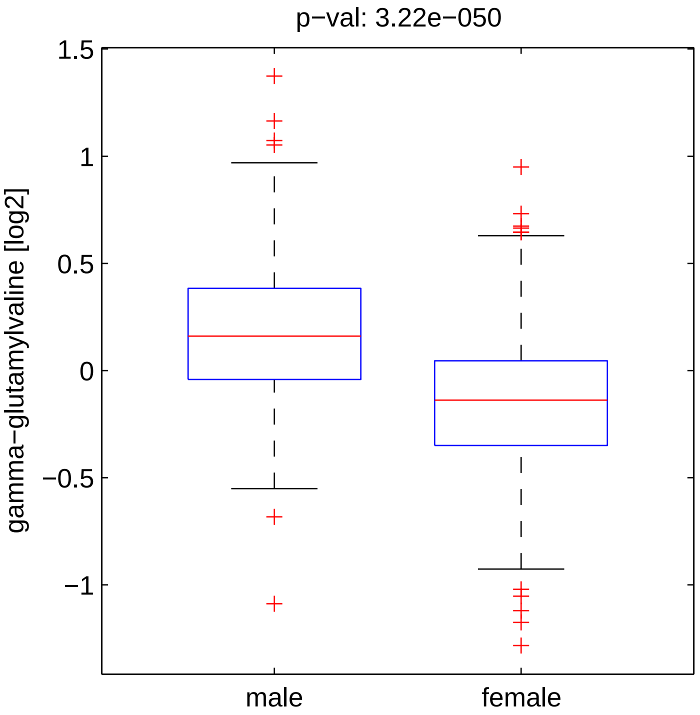
p−val: 3.22e−050
1.5
1
0.5
0
−0.5
−1
gamma−glutamylvaline [log2]
male
female

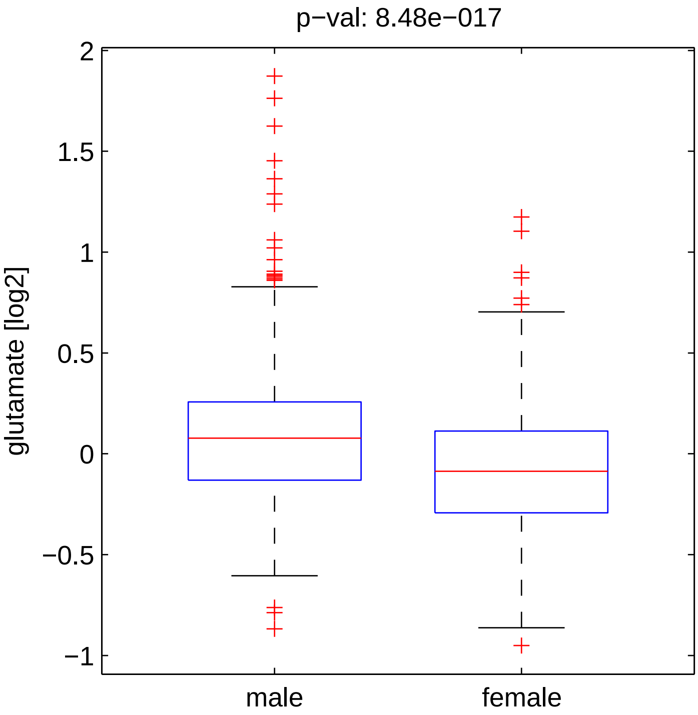
p−val: 8.48e−017
2
1.5
1
0.5
0
−0.5
−1
glutamate [log2]
male
female

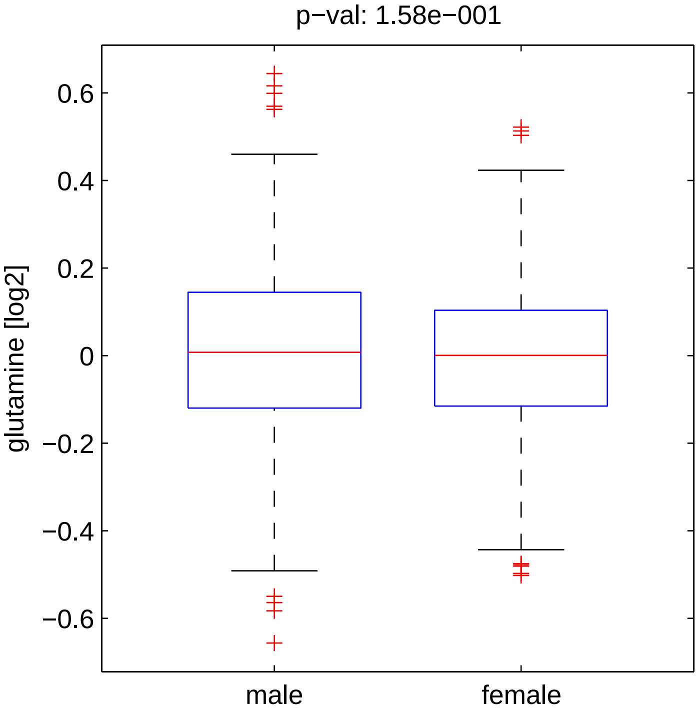

p−val: 1.58e−001
0.6
0.4
0.2
0
−0.2
−0.4
−0.6
glutamine [log2]
male
female

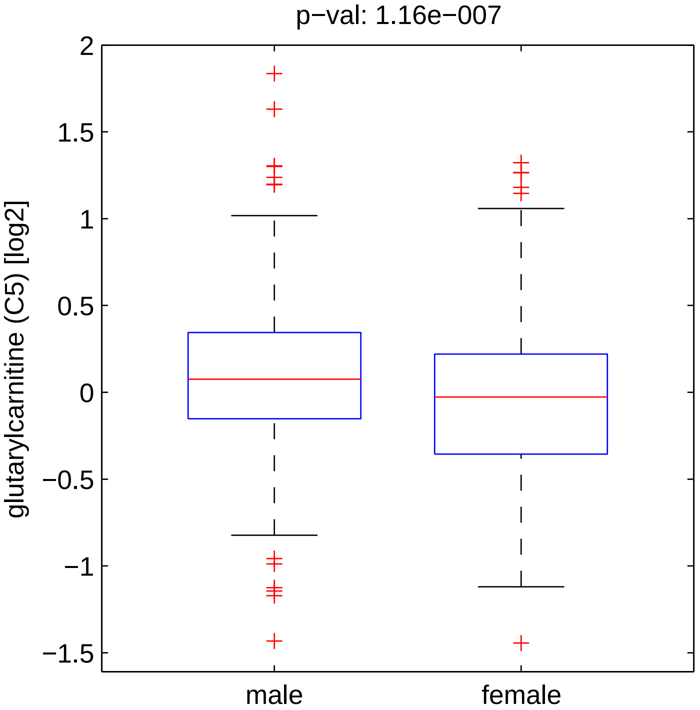
p−val: 1.16e−007
glutarylcarnitine (C5) [log2]
2
1.5
1
0.5
0
−0.5
−1
−1.5
male
female

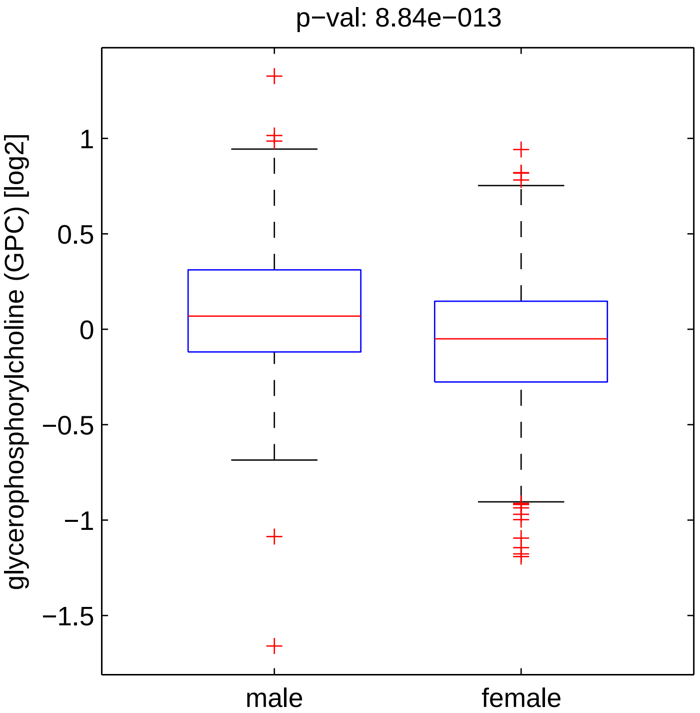

p−val: 8.84e−013
glycerophosphorylcholine (GPC) [log2]
1
0.5
0
−0.5
−1
−1.5
male
female

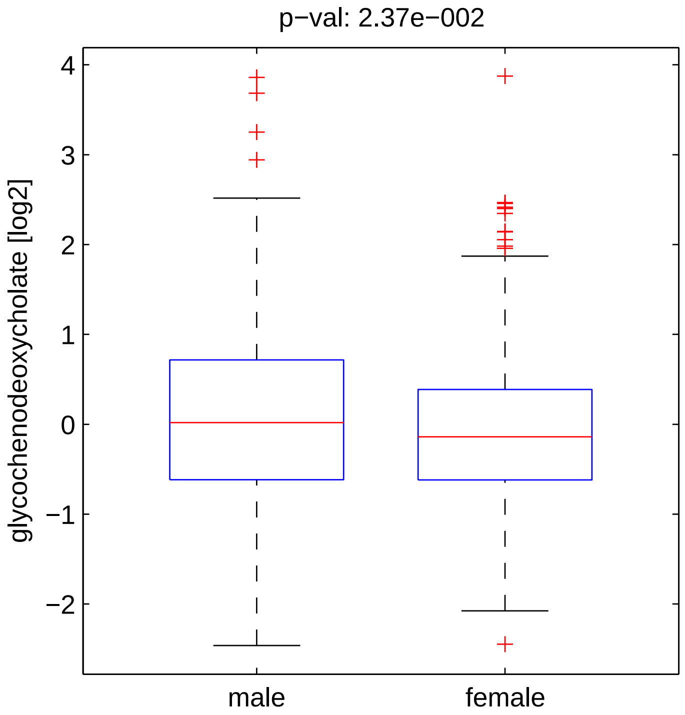
p−val: 2.37e−002
glycochenodeoxycholate [log2]
4
3
2
1
0
−1
−2
male
female

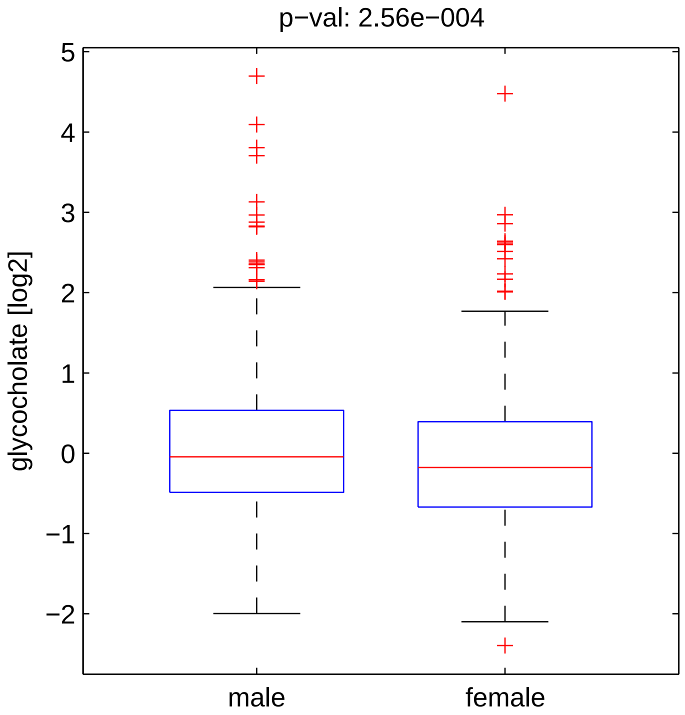

p−val: 2.56e−004
glycocholate [log2]
5
4
3
2
1
0
−1
−2
male
female

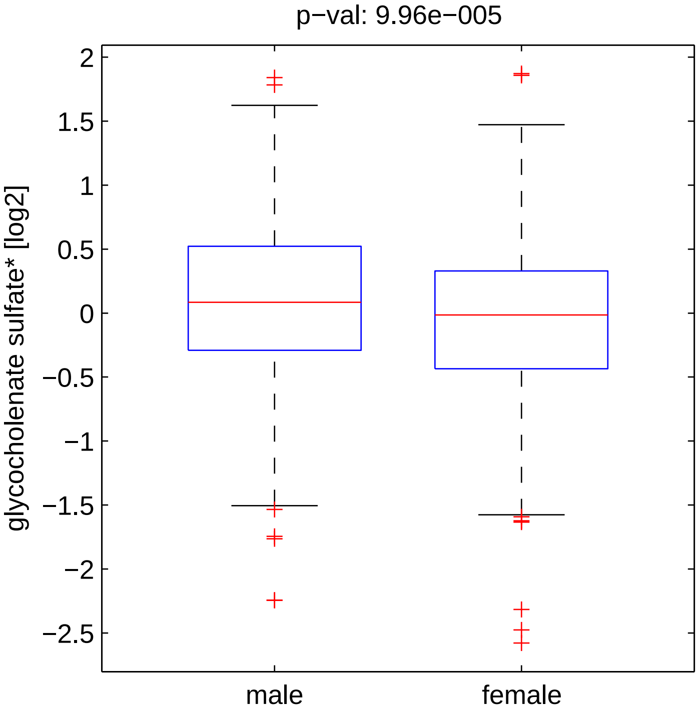

p−val: 9.96e−005
glycocholenate sulfate* [log2]
2
1.5
1
0.5
0
−0.5
−1
−1.5
−2
−2.5
male
female

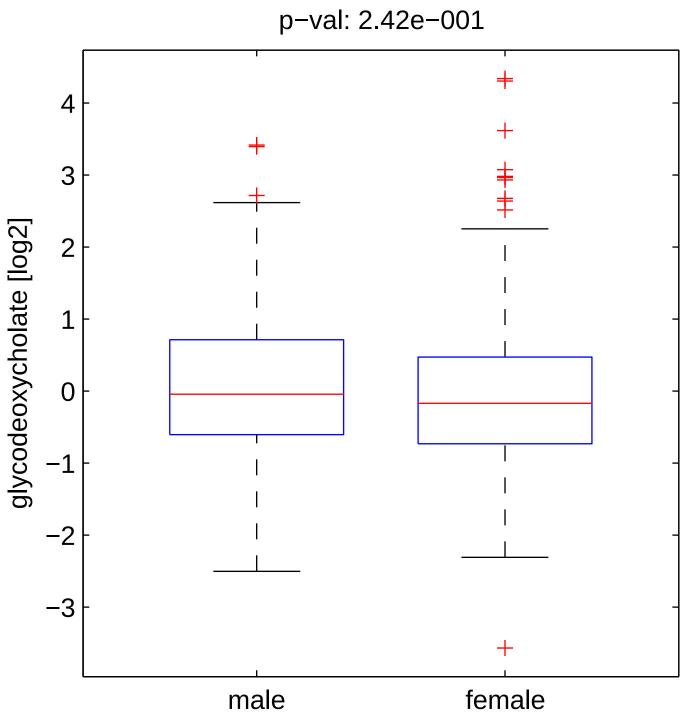
p−val: 2.42e−001
glycodeoxycholate [log2]
4
3
2
1
0
−1
−2
−3
male
female

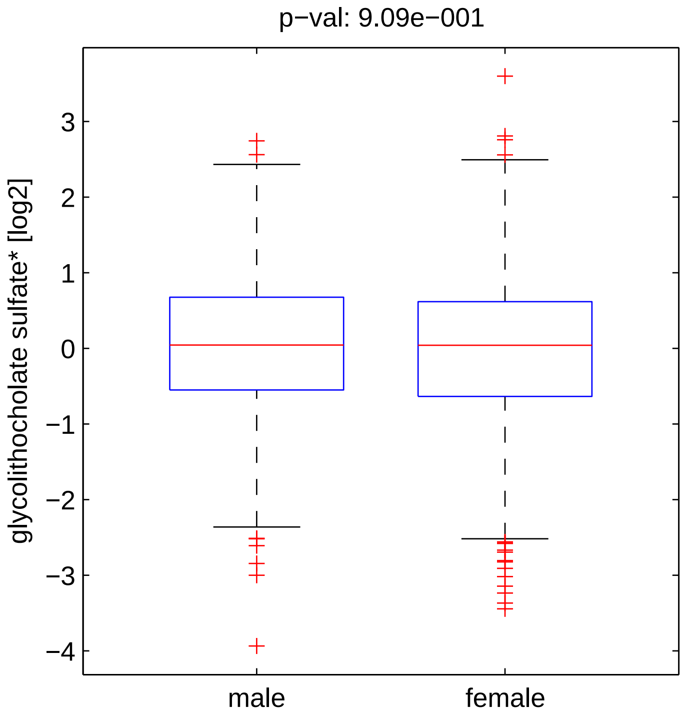
p−val: 9.09e−001
glycolithocholate sulfate* [log2]
3
2
1
0
−1
−2
−3
−4
male
female

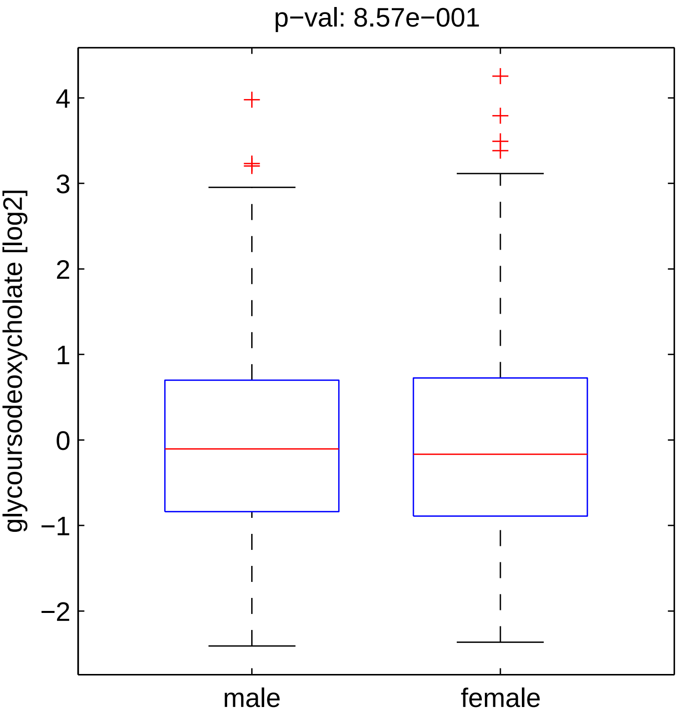
p−val: 8.57e−001
glycoursodeoxycholate [log2]
4
3
2
1
0
−1
−2
male
female

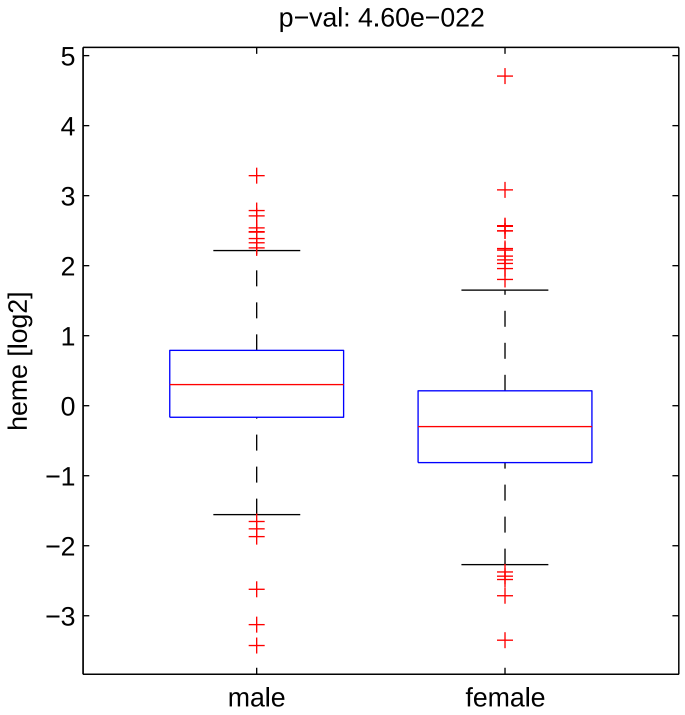
p−val: 4.60e−022
5
4
3
2
1
0
−1
−2
−3
heme [log2]
male
female

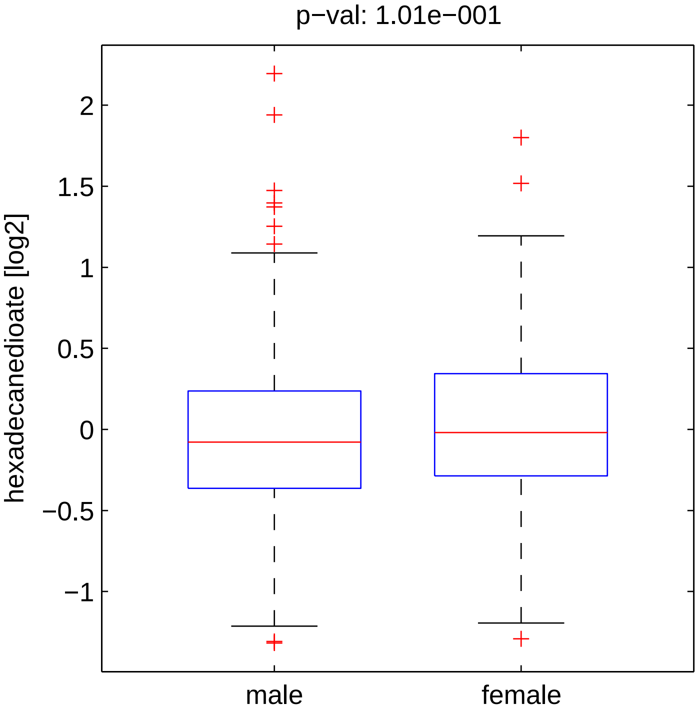

p−val: 1.01e−001
hexadecanediodate [log2]
2
1.5
1
0.5
0
−0.5
−1
male
female

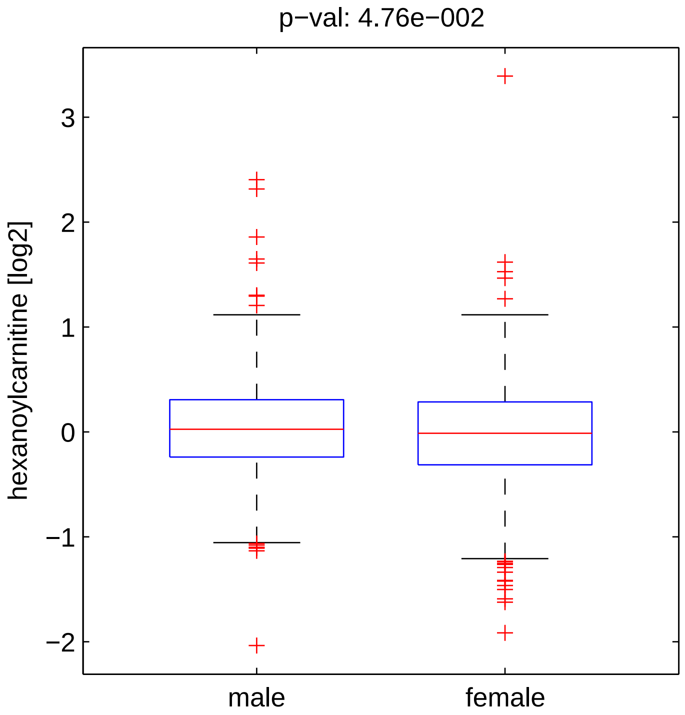
p−val: 4.76e−002
hexanoylcarnitine [log2]
3
2
1
0
−1
−2
male
female

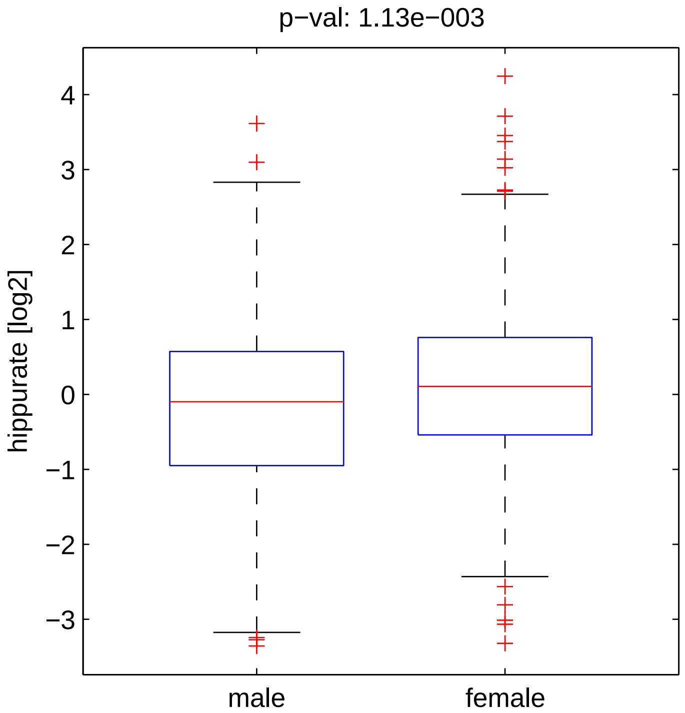

p−val: 1.13e−003
hippurate [log2]
4
3
2
1
0
−1
−2
−3
male
female

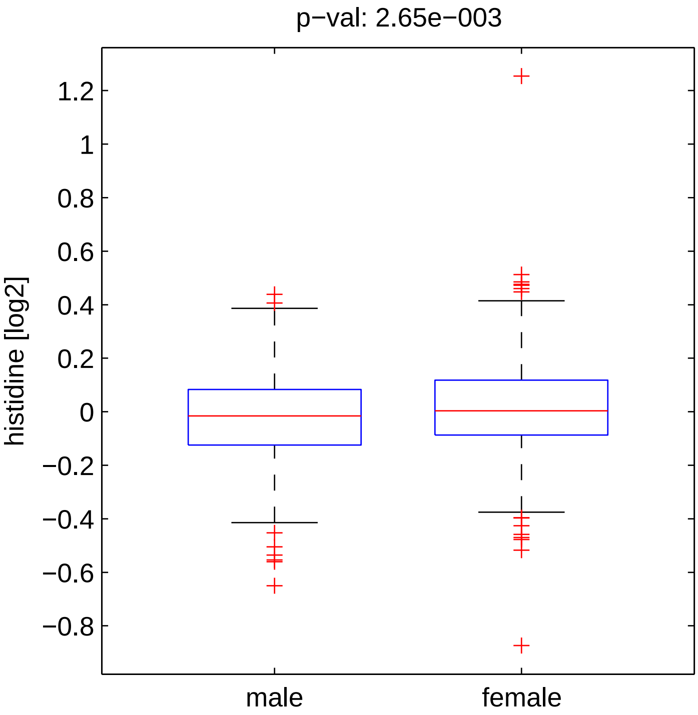

p−val: 2.65e−003
histidine [log2]
1.2
1
0.8
0.6
0.4
0.2
0
−0.2
−0.4
−0.6
−0.8
male
female

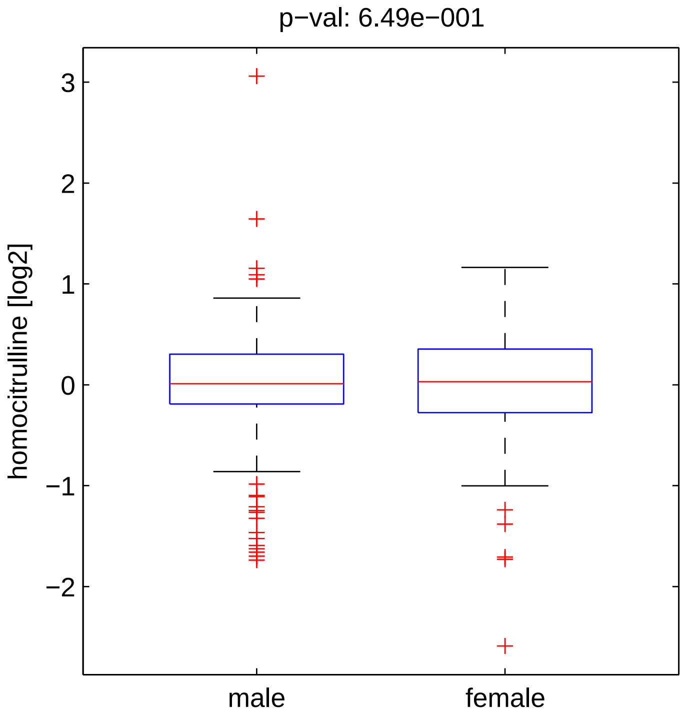
p−val: 6.49e−001
homocitrulline [log2]
3
2
1
0
−1
−2
male
female

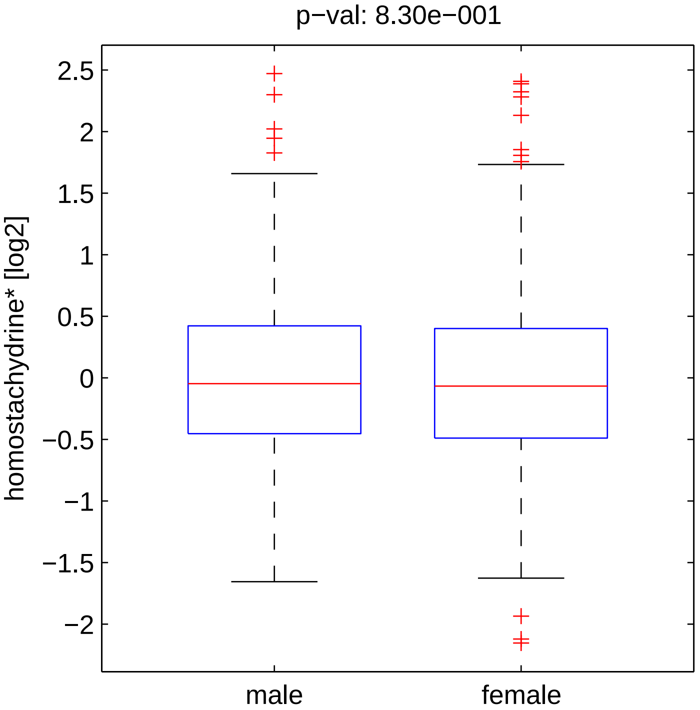

p−val: 8.30e−001
homostachydrine* [log2]
2.5
2
1.5
1
0.5
0
−0.5
−1
−1.5
−2
male
female

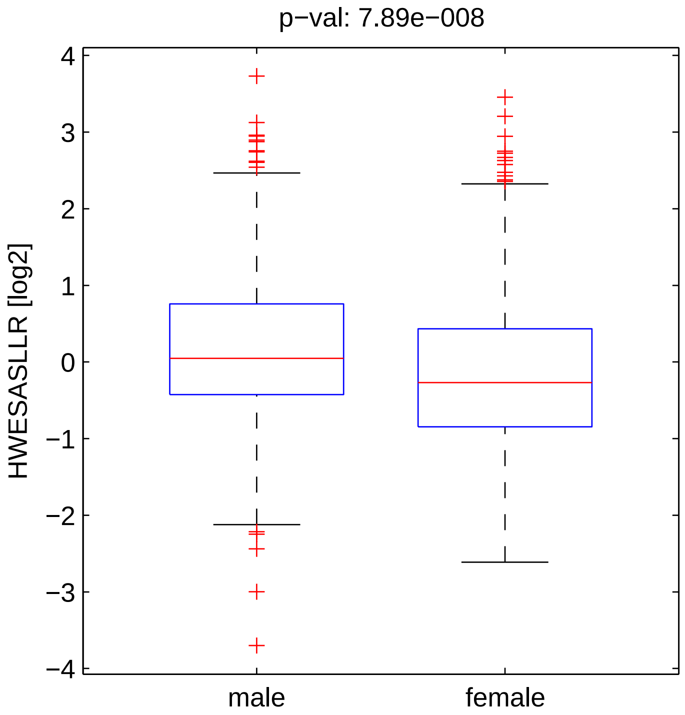
p−val: 7.89e−008
HWESASLLR [log2]
4
3
2
1
0
−1
−2
−3
−4
male
female

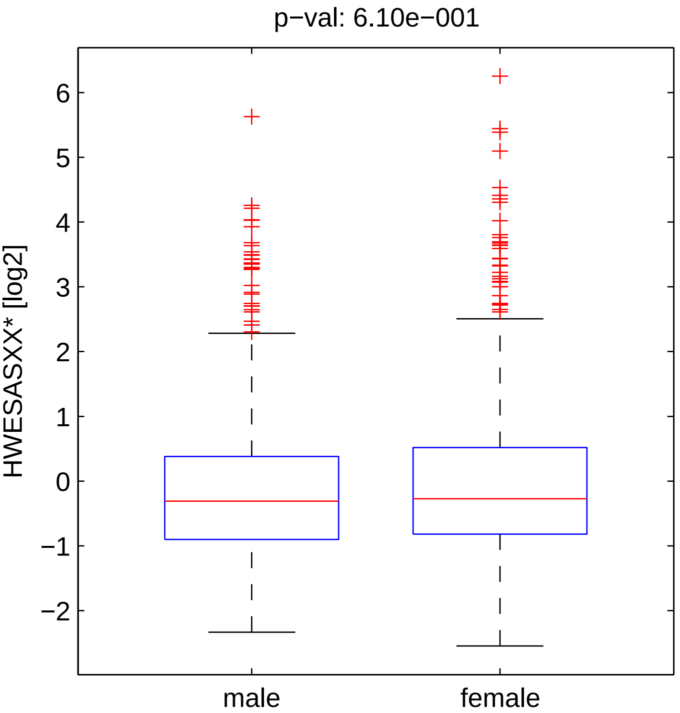
p−val: 6.10e−001
HWESASXX* [log2]
6
5
4
3
2
1
0
−1
−2
male
female

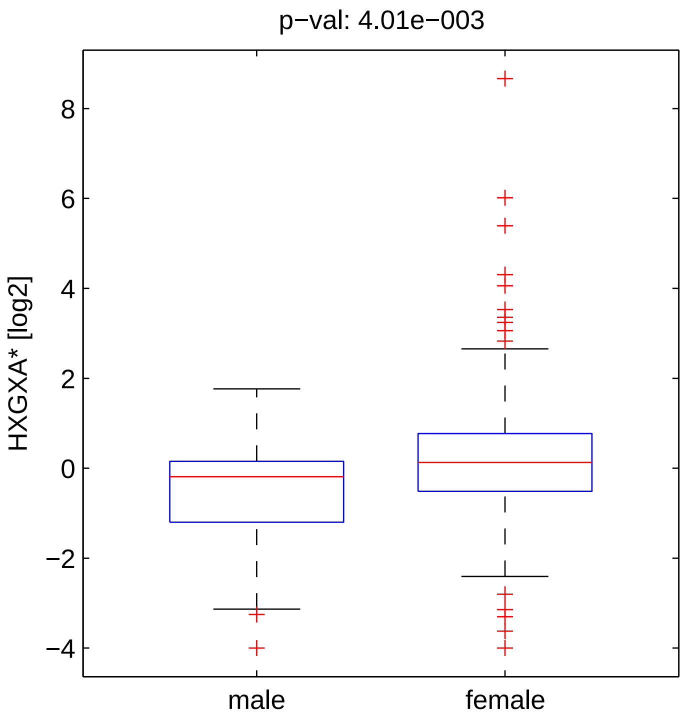
p−val: 4.01e−003
HXGXA* [log2]
8
6
4
2
0
−2
−4
male
female

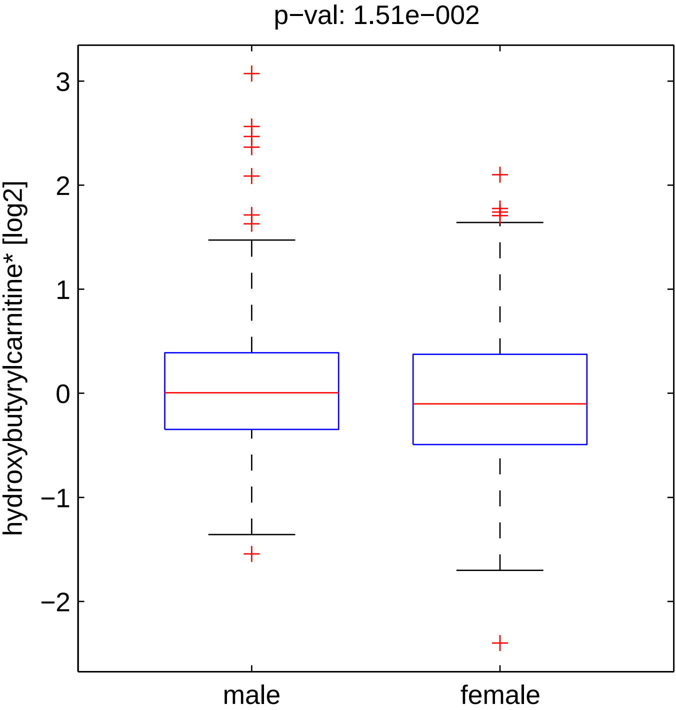
p−val: 1.51e−002
hydroxybutyrylcarnitine* [log2]
3
2
1
0
−1
−2
male
female

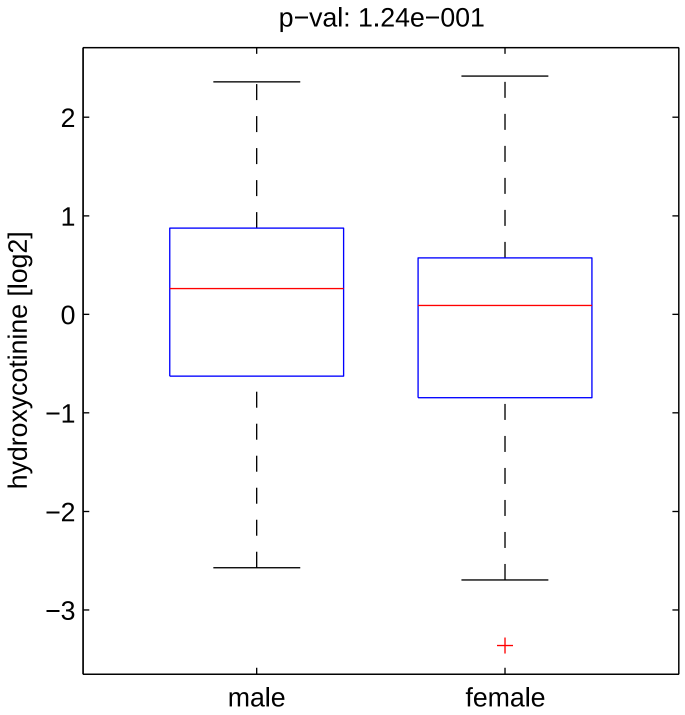

p−val: 1.24e−001
hydroxycotinine [log2]
2
1
0
−1
−2
−3
male
female

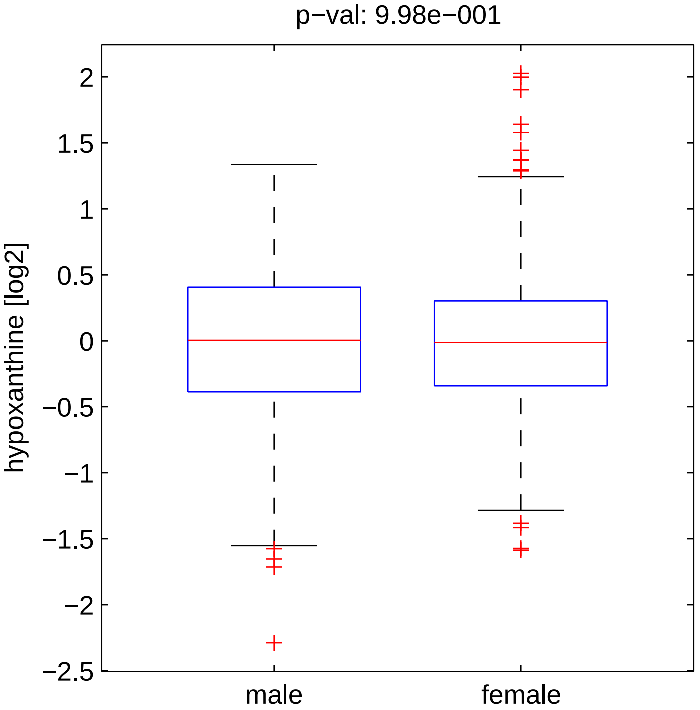
p−val: 9.98e−001
hypoxanthine [log2]
2
1.5
1
0.5
0
−0.5
−1
−1.5
−2
−2.5
male
female

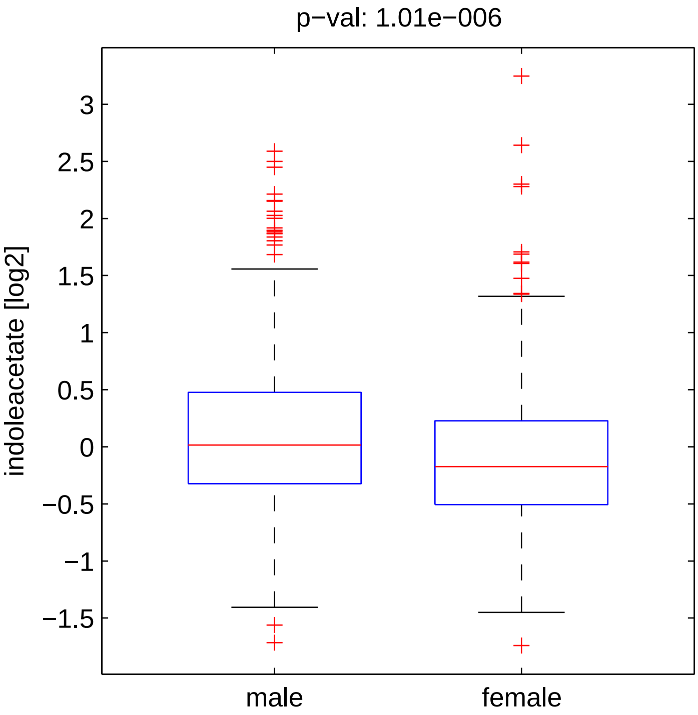
p−val: 1.01e−006
indoleacetate [log2]
3
2.5
2
1.5
1
0.5
0
−0.5
−1
−1.5
male
female

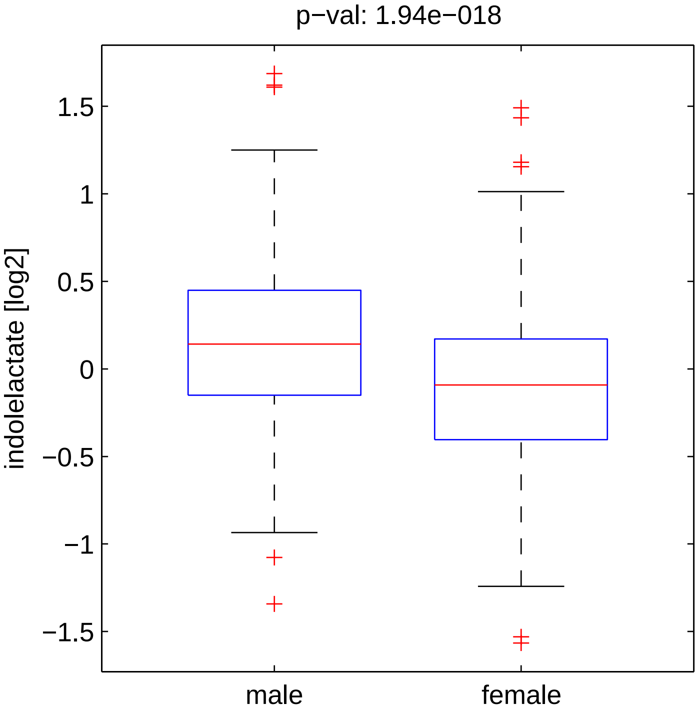

p−val: 1.94e−018
indolelactate [log2]
1.5
1
0.5
0
−0.5
−1
−1.5
male
female

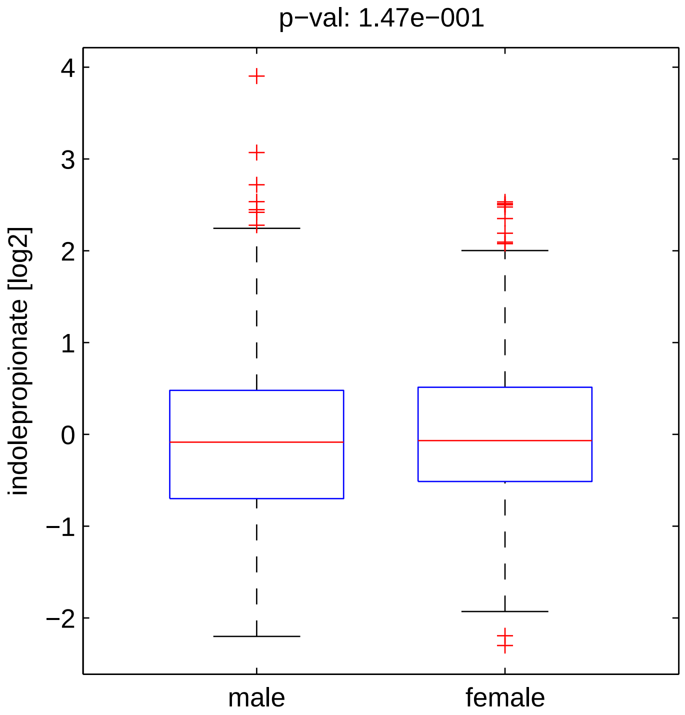
p−val: 1.47e−001
indolepropionate [log2]
4
3
2
1
0
−1
−2
male
female

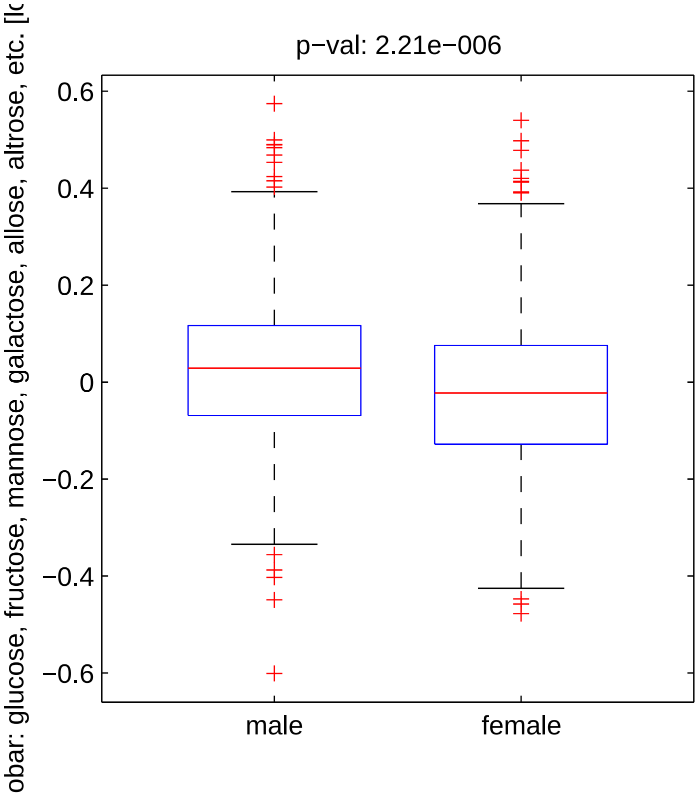
p−val: 2.21e−006
0.6
0.4
0.2
0
−0.2
−0.4
−0.6
male
female
obar: glucose, fructose, mannose, galactose, allose, altrose, etc. [lo

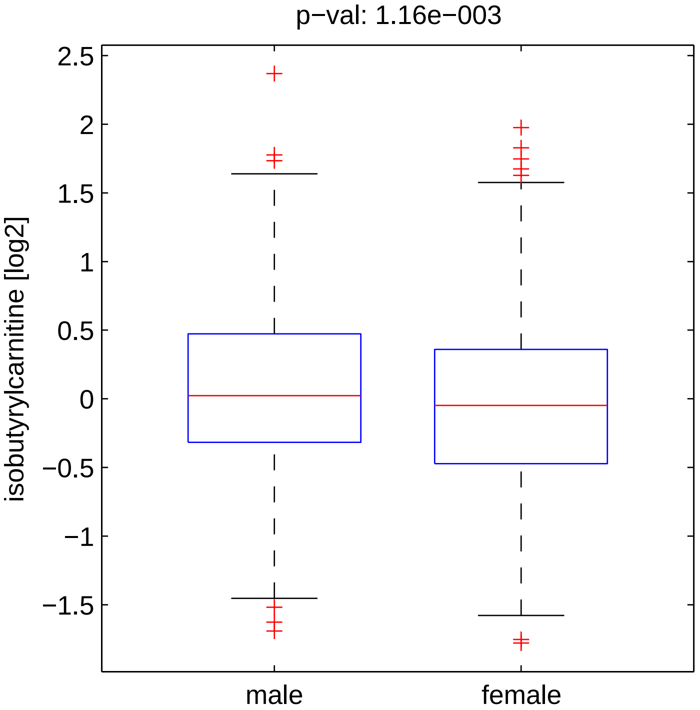

p−val: 1.16e−003
isobutyrylcarnitine [log2]
2.5
2
1.5
1
0.5
0
−0.5
−1
−1.5
male
female

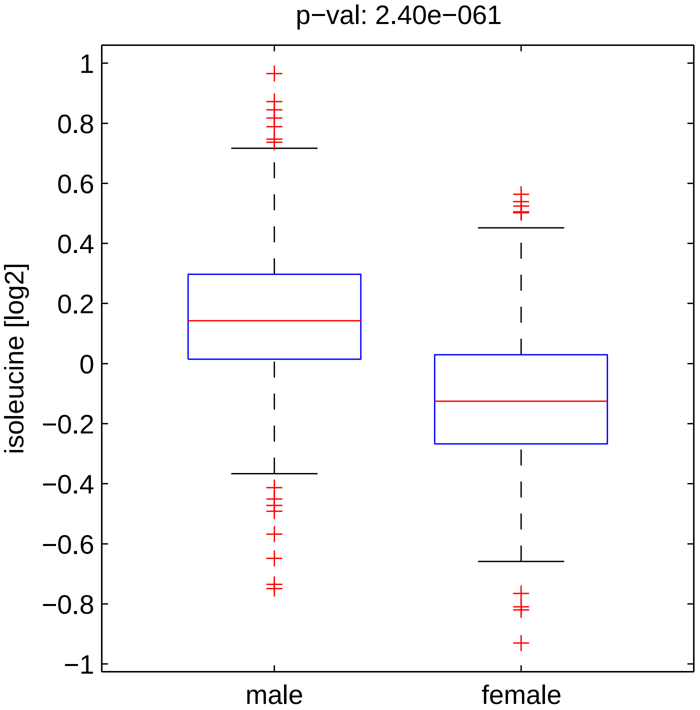

p−val: 2.40e−061
1
0.8
0.6
0.4
0.2
0
−0.2
−0.4
−0.6
−0.8
−1
isoleucine [log2]
male
female

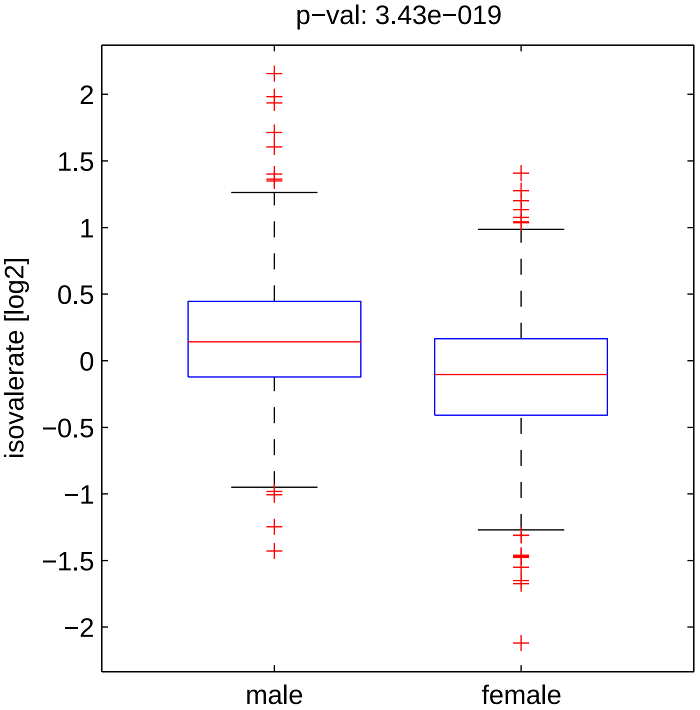
p−val: 3.43e−019
isovalerate [log2]
2
1.5
1
0.5
0
−0.5
−1
−1.5
−2
male
female

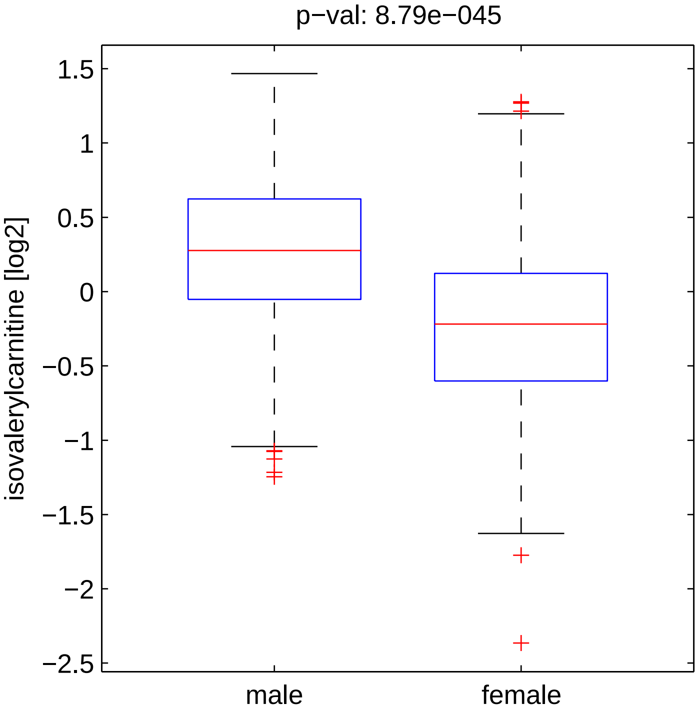

p−val: 8.79e−045
isovalerylcarnitine [log2]
1.5
1
0.5
0
−0.5
−1
−1.5
−2
−2.5
male
female

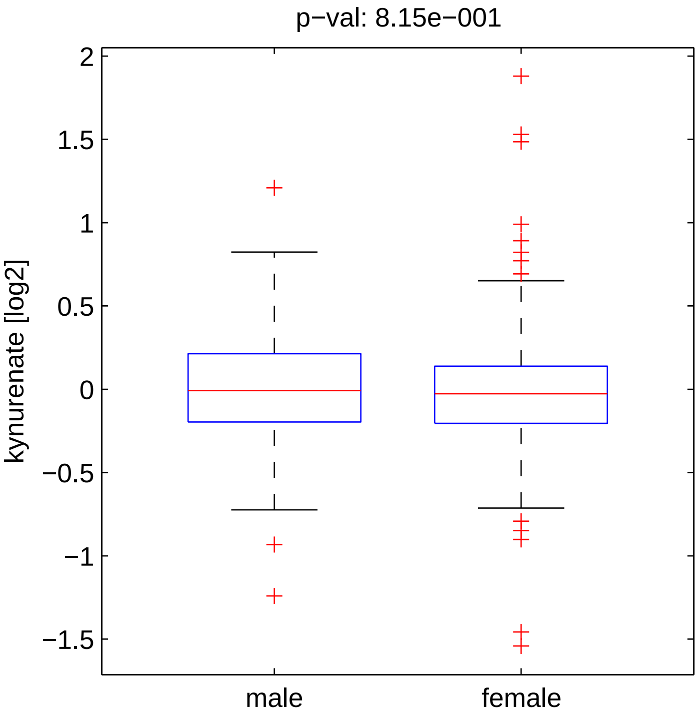

p−val: 8.15e−001
kynurenate [log2]
2
1.5
1
0.5
0
−0.5
−1
−1.5
male
female

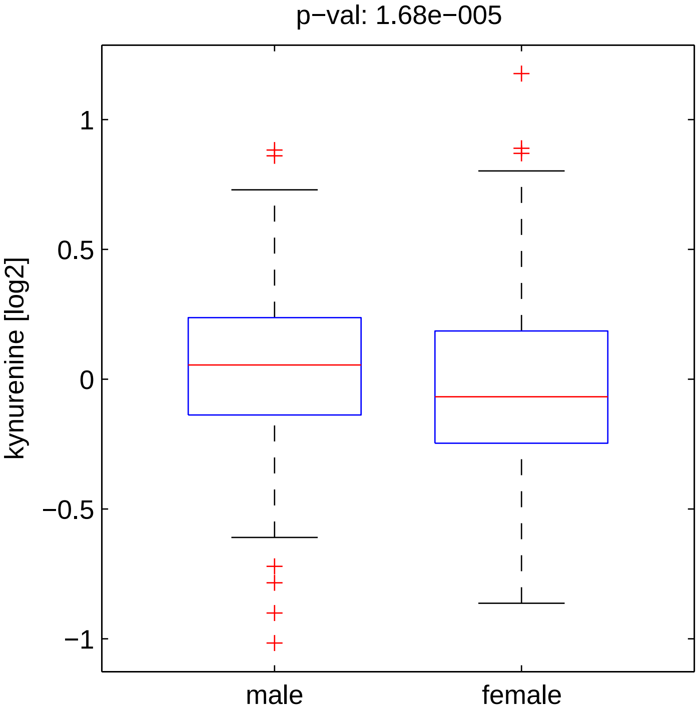
p−val: 1.68e−005
kynurenine [log2]
1
0.5
0
−0.5
−1
male
female

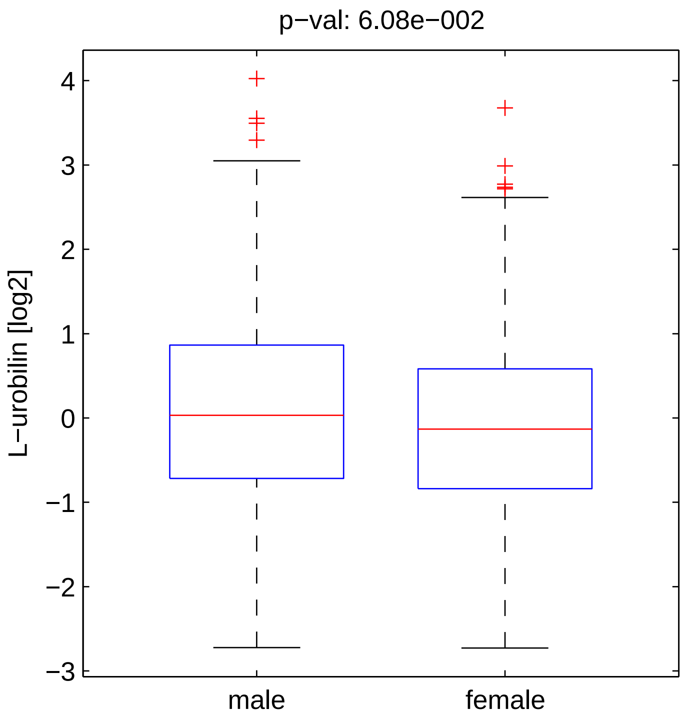
p−val: 6.08e−002
L−urobilin [log2]
4
3
2
1
0
−1
−2
−3
male
female

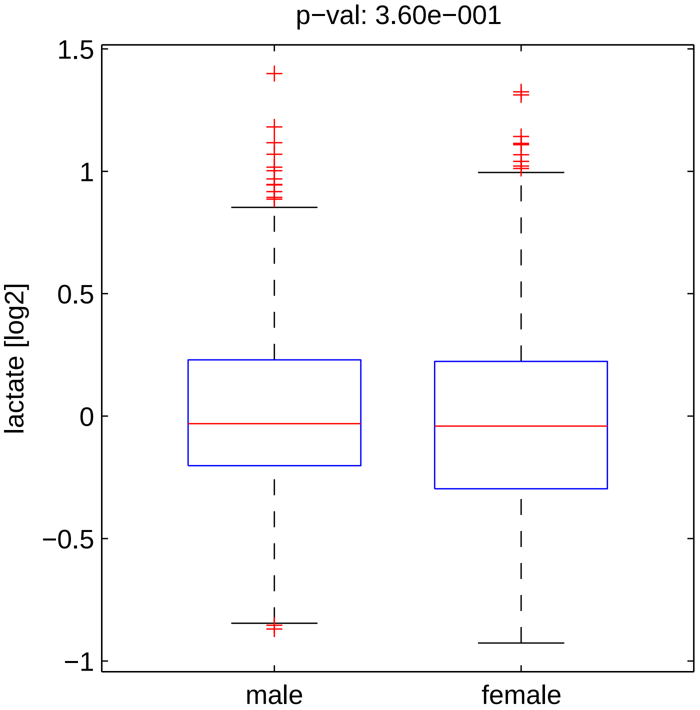

p−val: 3.60e−001
1.5
1
0.5
0
−0.5
−1
lactate [log2]
male
female

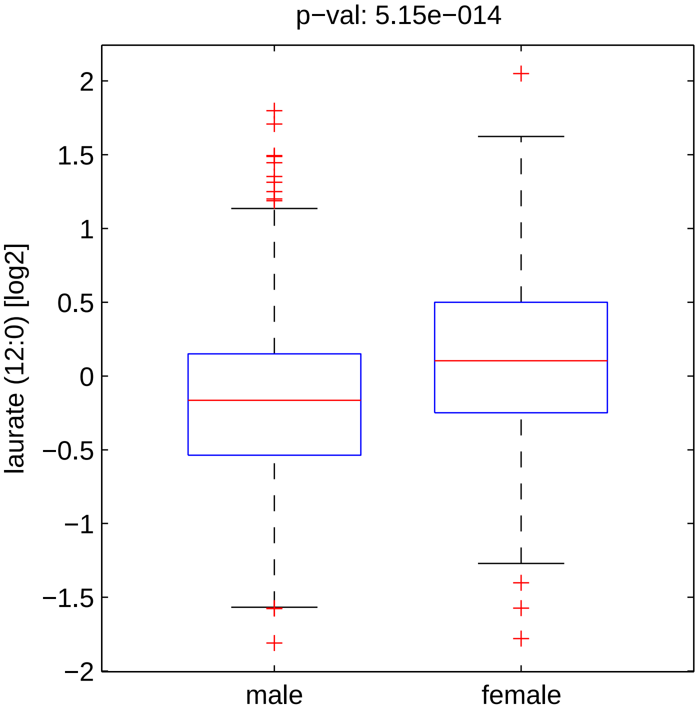
p−val: 5.15e−014
laurate (12:0) [log2]
2
1.5
1
0.5
0
−0.5
−1
−1.5
−2
male
female

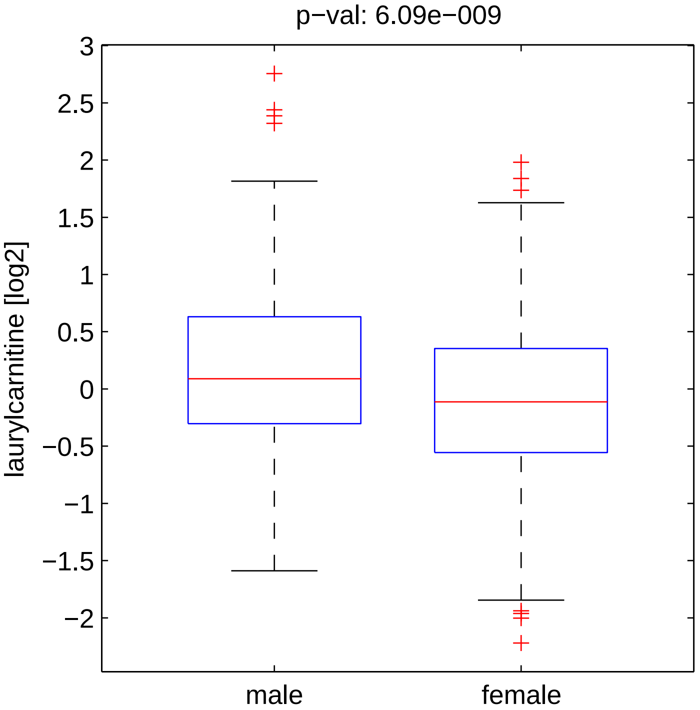
p−val: 6.09e−009
3
2.5
2
1.5
1
0.5
0
−0.5
−1
−1.5
−2
laurylcarnitine [log2]
male
female

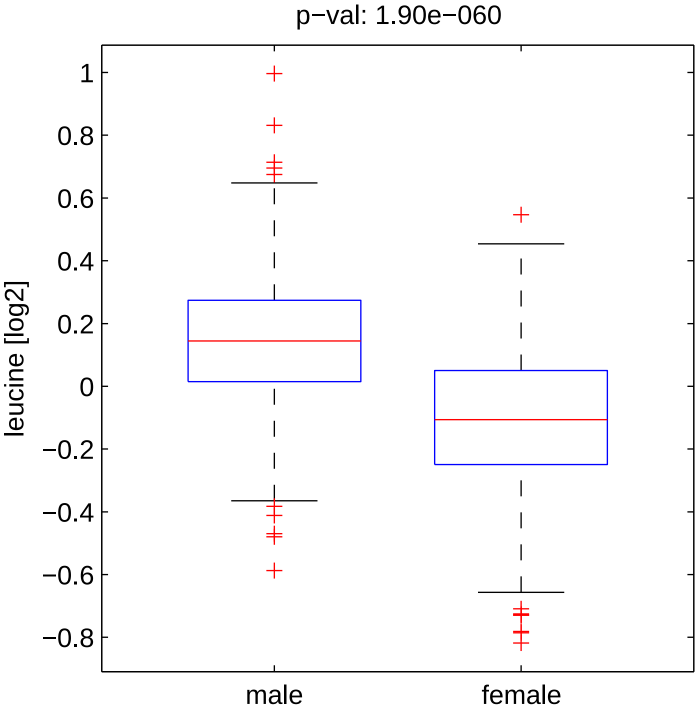

p−val: 1.90e−060
1
0.8
0.6
0.4
0.2
0
−0.2
−0.4
−0.6
−0.8
leucine [log2]
male
female

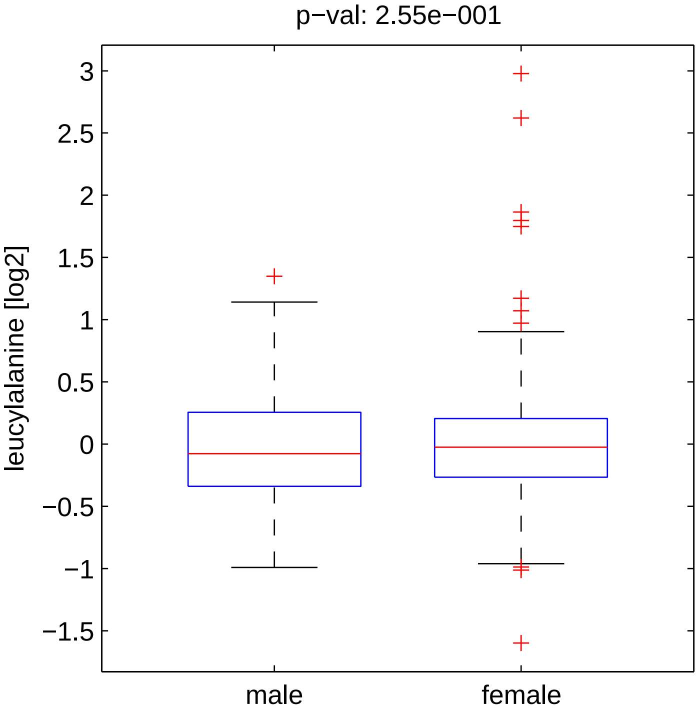
p−val: 2.55e−001
leucylalanine [log2]
3
2.5
2
1.5
1
0.5
0
−0.5
−1
−1.5
male
female

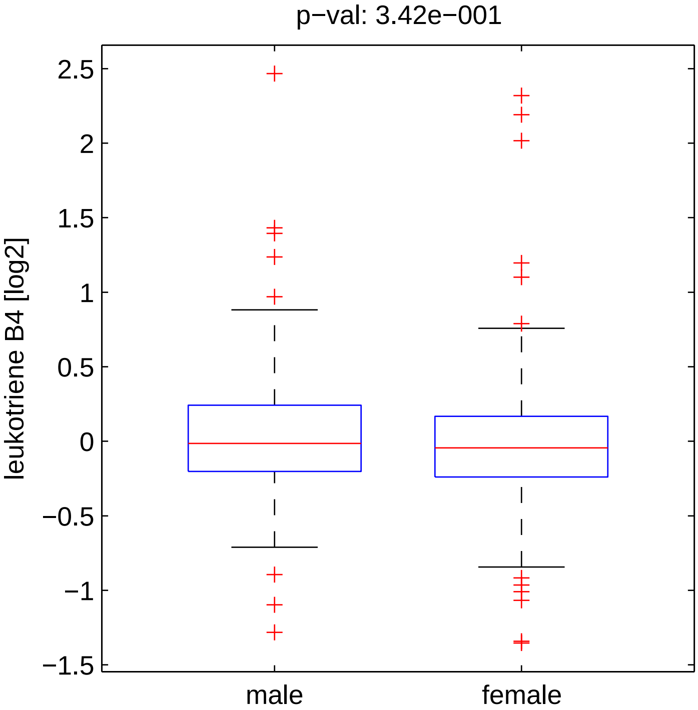
p−val: 3.42e−001
leukotriene B4 [log2]
2.5
2
1.5
1
0.5
0
−0.5
−1
−1.5
male
female

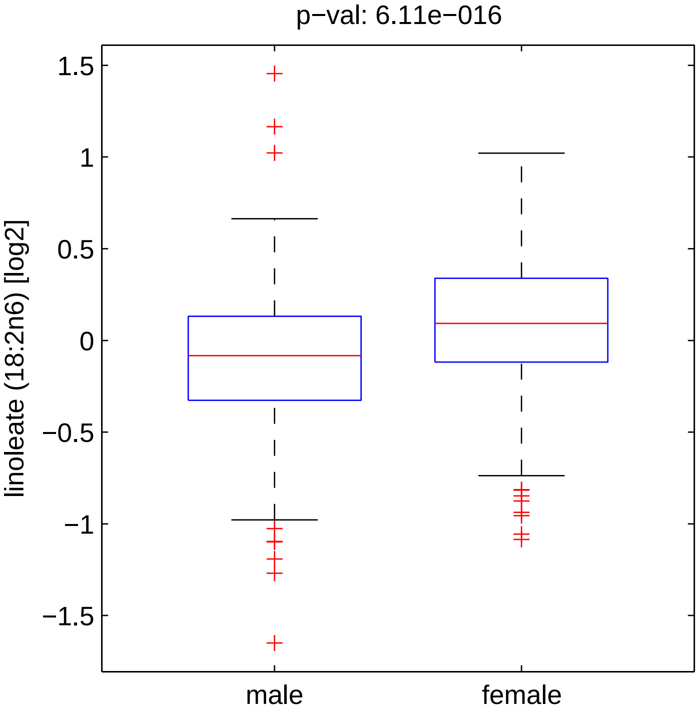
p−val: 6.11e−016
linoleate (18:2n6) [log2]
1.5
1
0.5
0
−0.5
−1
−1.5
male
female

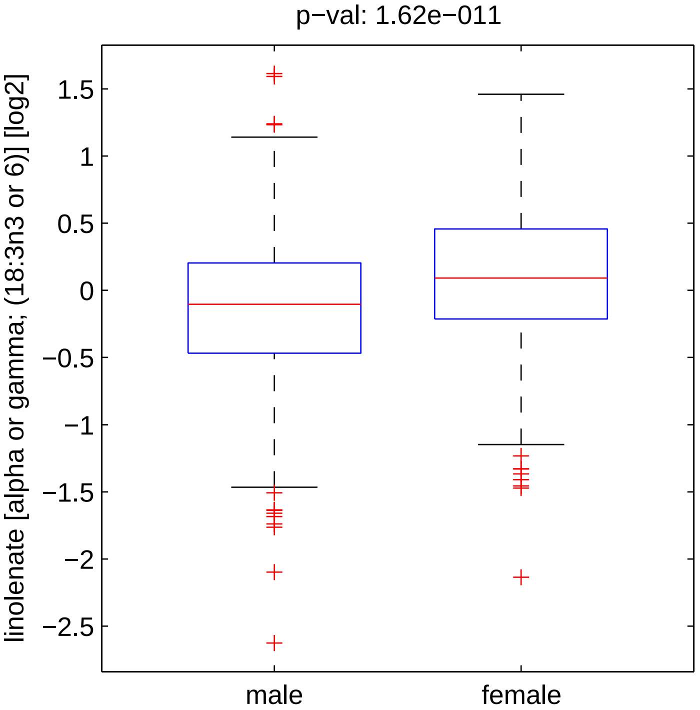

p−val: 1.62e−011
linolenate [alpha or gamma; (18:3n3 or 6)] [log2]
1.5
1
0.5
0
−0.5
−1
−1.5
−2
−2.5
male
female

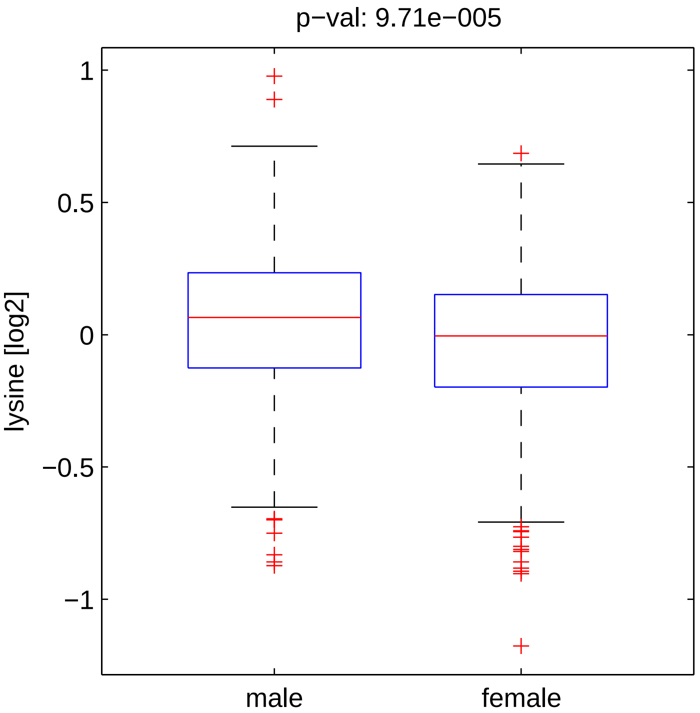

p−val: 9.71e−005
1
0.5
0
−0.5
−1
lysine [log2]
male
female

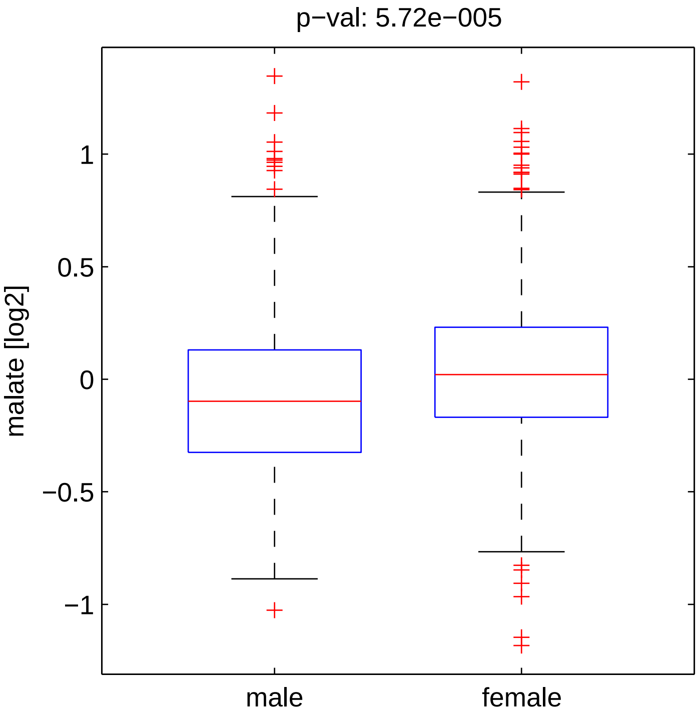

p−val: 5.72e−005
malate [log2]
1
0.5
0
−0.5
−1
male
female

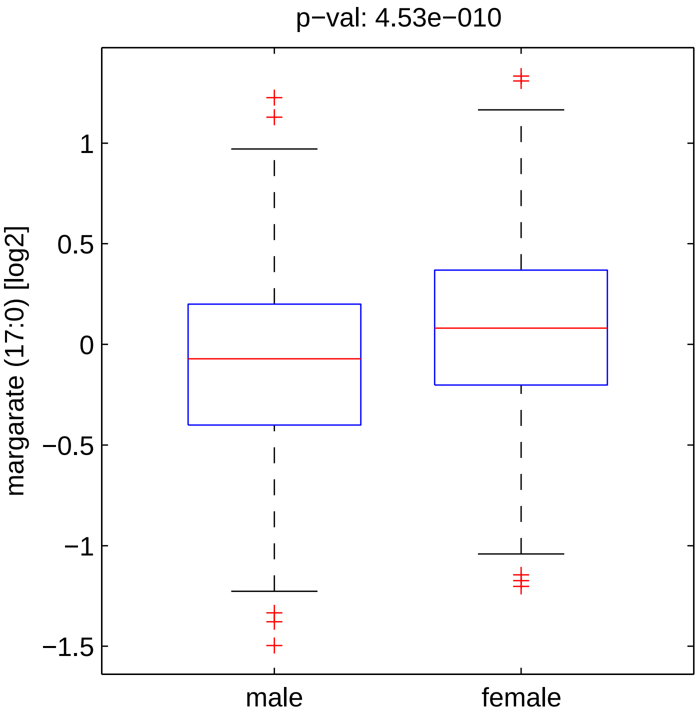
p−val: 4.53e−010
margarate (17:0) [log2]
1
0.5
0
−0.5
−1
−1.5
male
female

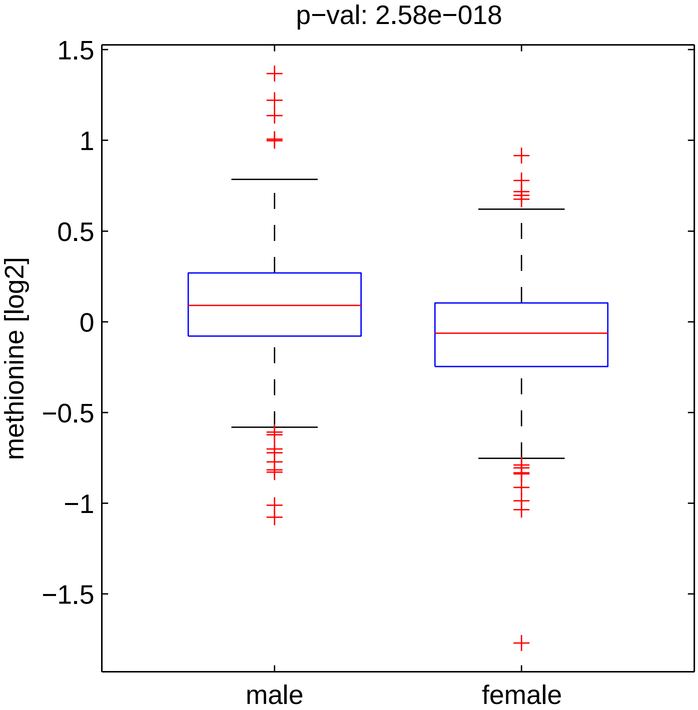
p−val: 2.58e−018
1.5
1
0.5
0
−0.5
−1
−1.5
methionine [log2]
male
female

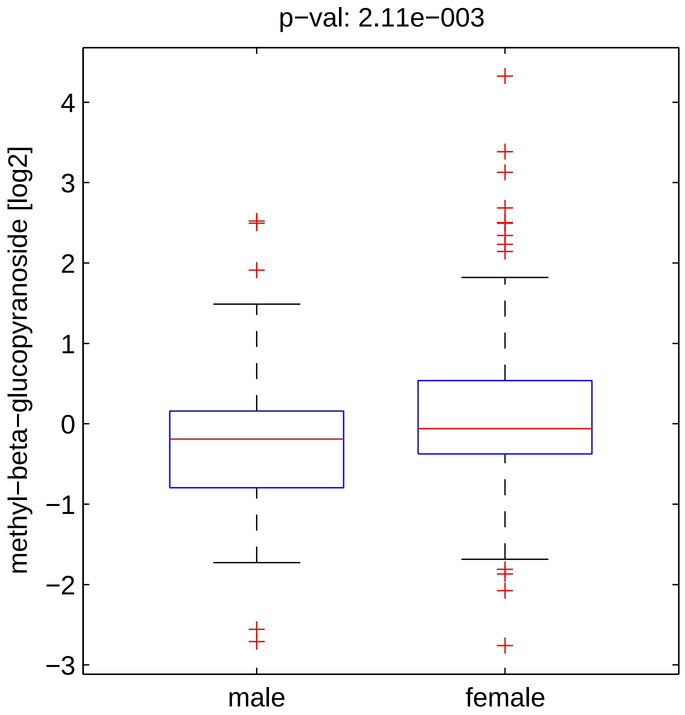

p−val: 2.11e−003
methyl−beta−glucopyranoside [log2]
4
3
2
1
0
−1
−2
−3
male
female

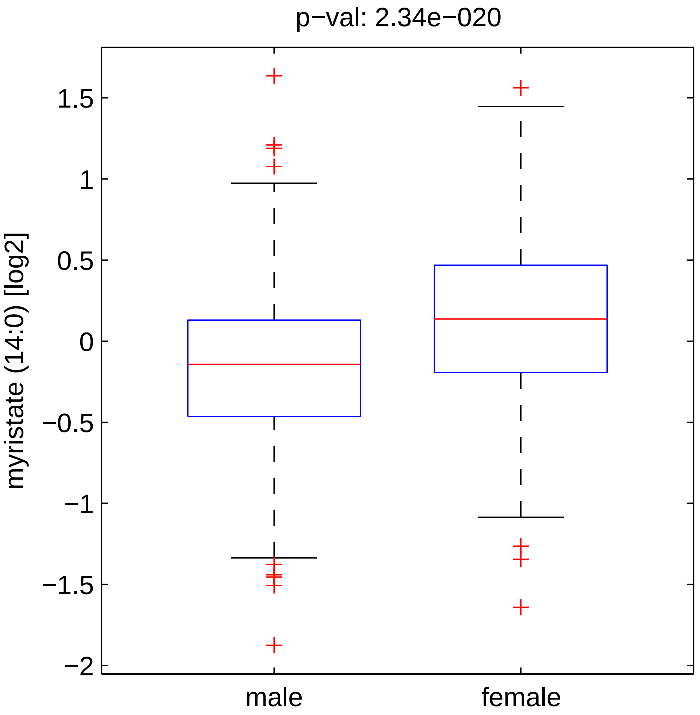
p−val: 2.34e−020
myristate (14:0) [log2]
1.5
1
0.5
0
−0.5
−1
−1.5
−2
male
female

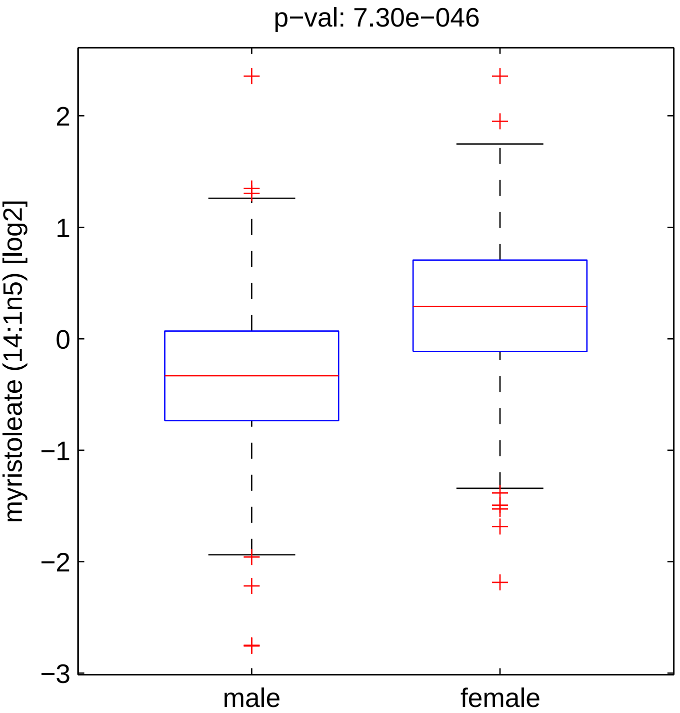
p−val: 7.30e−046
myristoleate (14:1n5) [log2]
2
1
0
−1
−2
−3
male
female

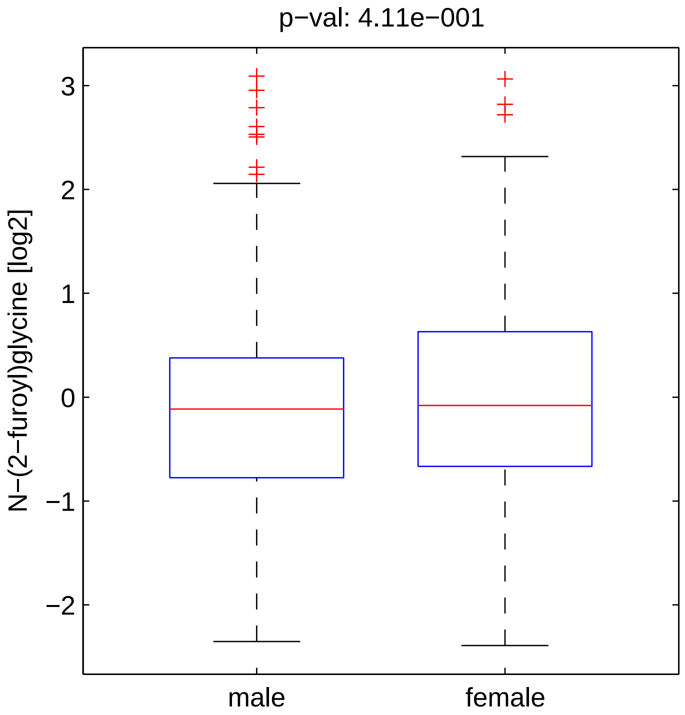
p−val: 4.11e−001
N−(2−furoyl)glycine [log2]
3
2
1
0
−1
−2
male
female

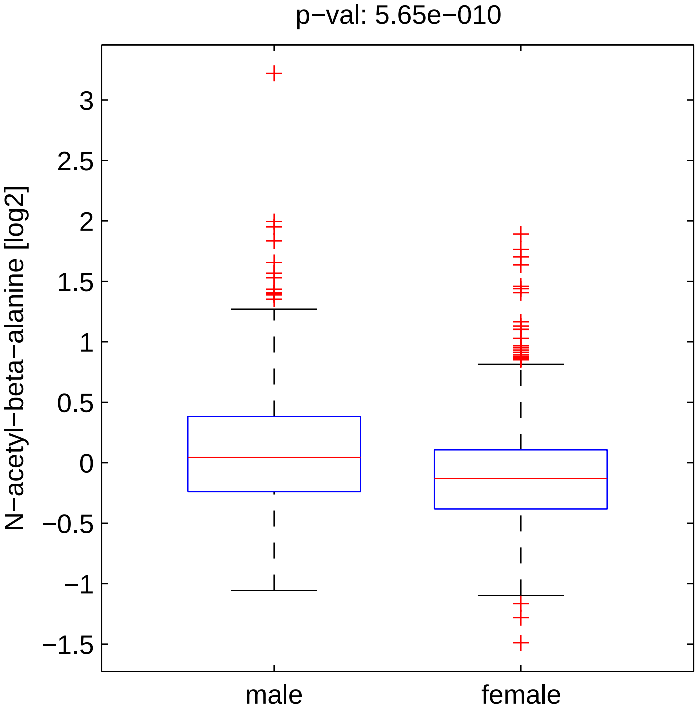
p−val: 5.65e−010
N−acetyl−beta−alanine [log2]
3
2.5
2
1.5
1
0.5
0
−0.5
−1
−1.5
male
female

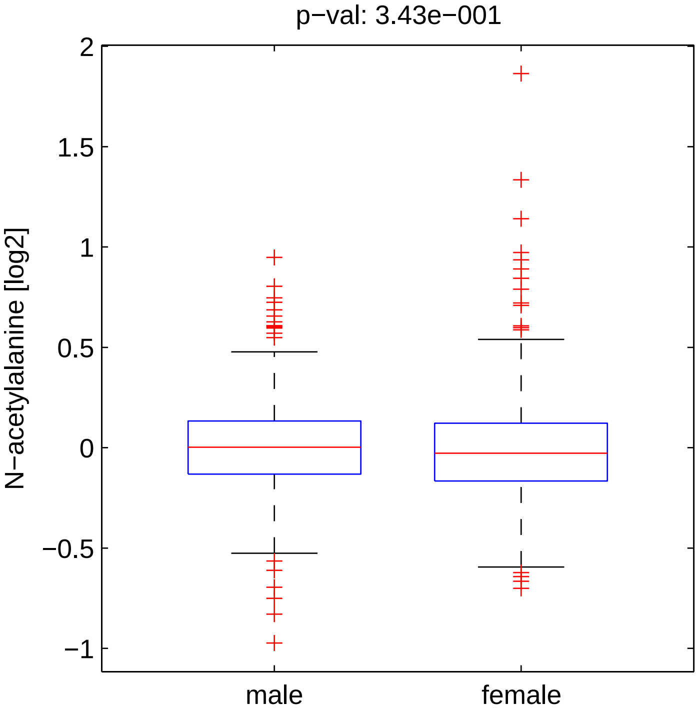
p−val: 3.43e−001
N−acetylalanine [log2]
2
1.5
1
0.5
0
−0.5
−1
male
female

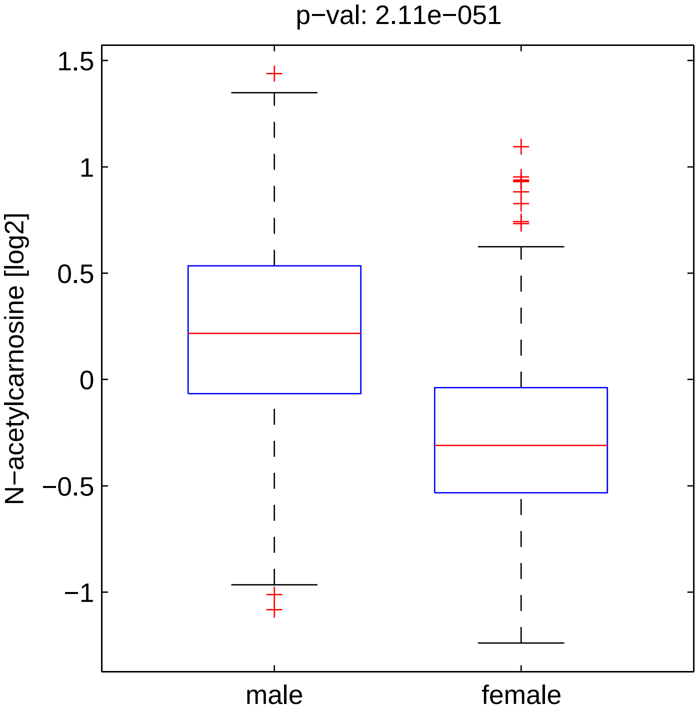

p−val: 2.11e−051
1.5
1
0.5
0
−0.5
−1
N−acetylcarnosine [log2]
male
female

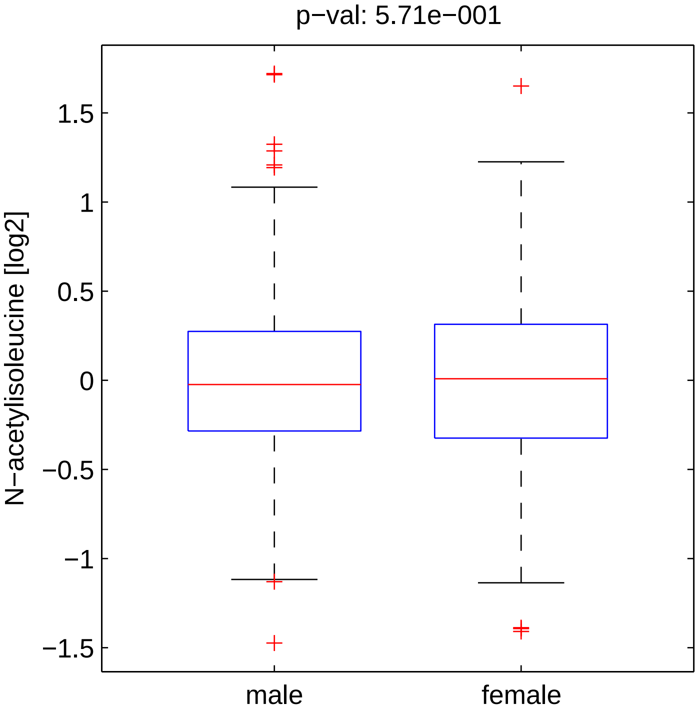
p−val: 5.71e−001
N−acetylisoleucine [log2]
1.5
1
0.5
0
−0.5
−1
−1.5
male
female

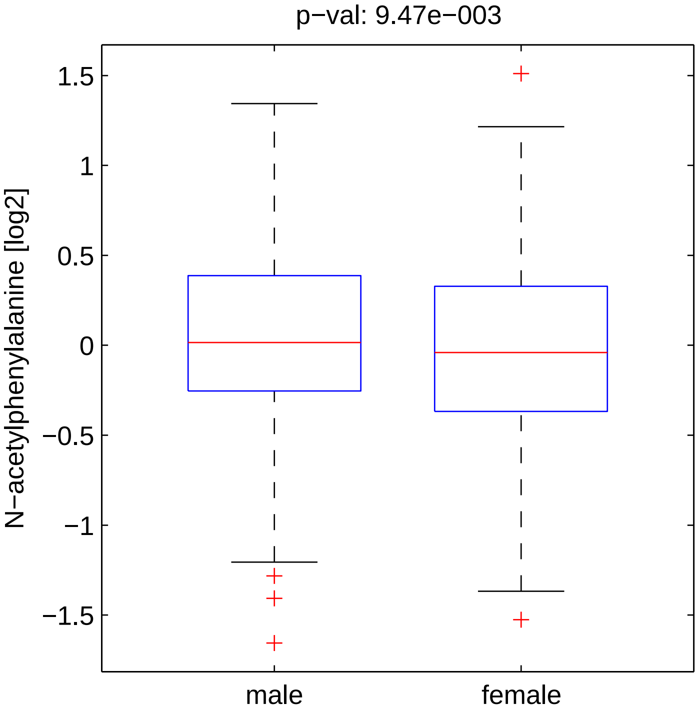
p−val: 9.47e−003
N−acetylphenylalanine [log2]
1.5
1
0.5
0
−0.5
−1
−1.5
male
female

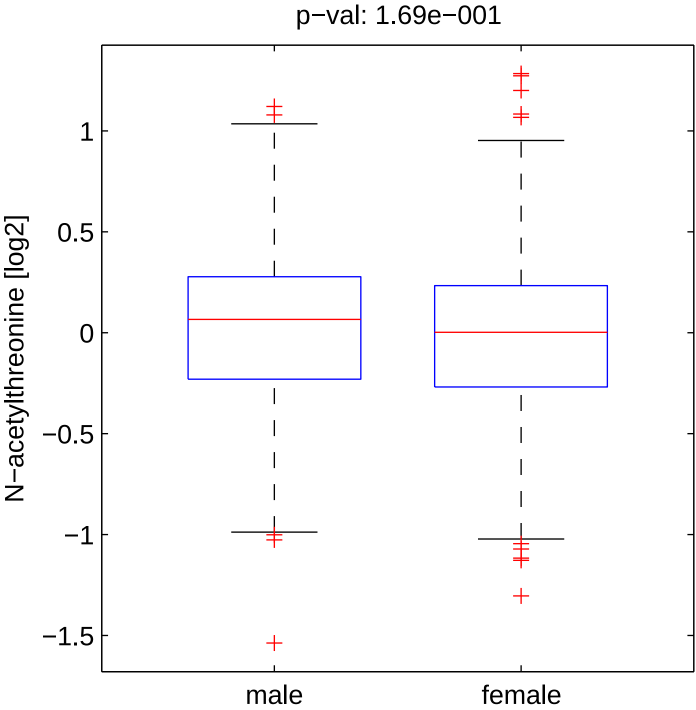

p−val: 1.69e−001
N−acetylthreonine [log2]
1
0.5
0
−0.5
−1
−1.5
male
female

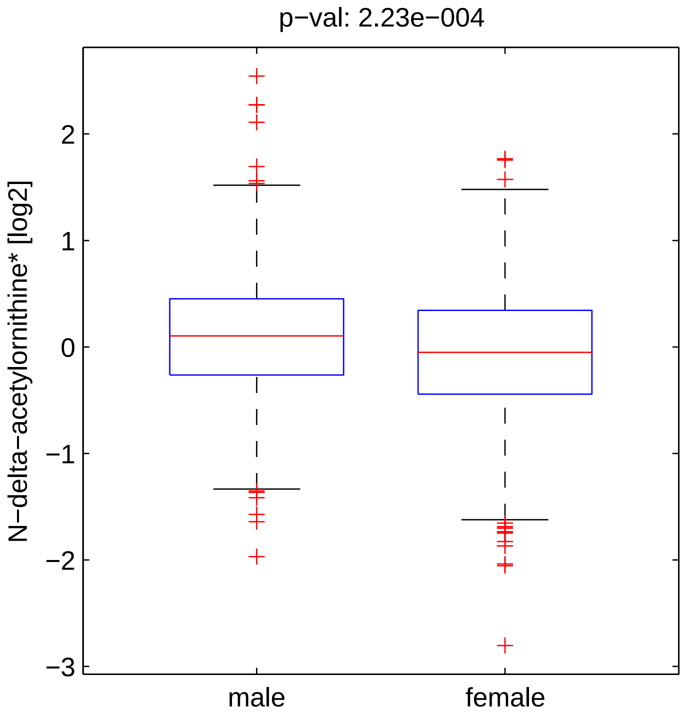

p−val: 2.23e−004
N−delta−acetylornithine* [log2]
2
1
0
−1
−2
−3
male
female

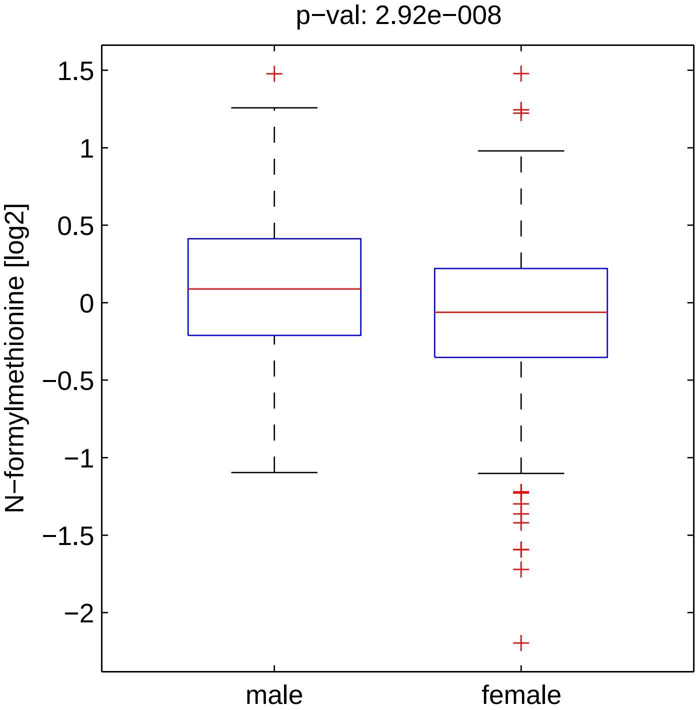

p−val: 2.92e−008
N−formylmethionine [log2]
1.5
1
0.5
0
−0.5
−1
−1.5
−2
male
female

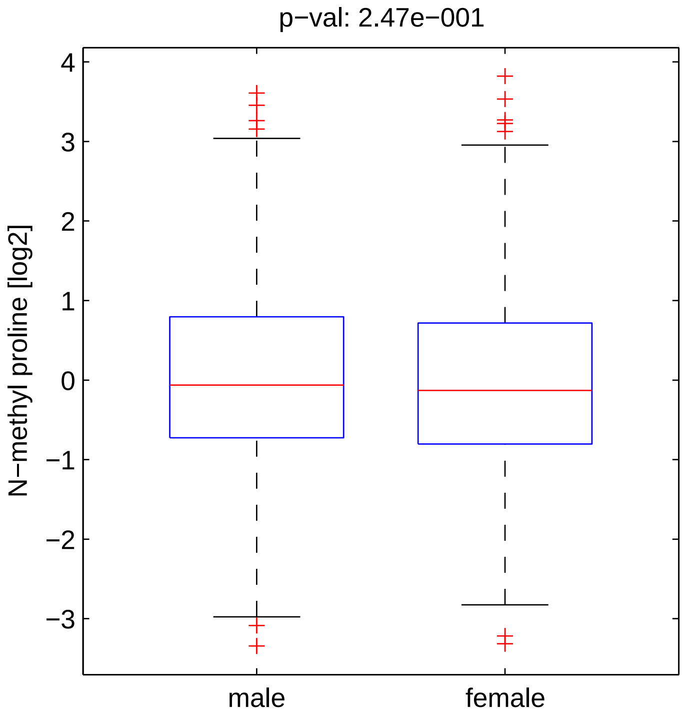
p−val: 2.47e−001
N−methyl proline [log2]
4
3
2
1
0
−1
−2
−3
male
female

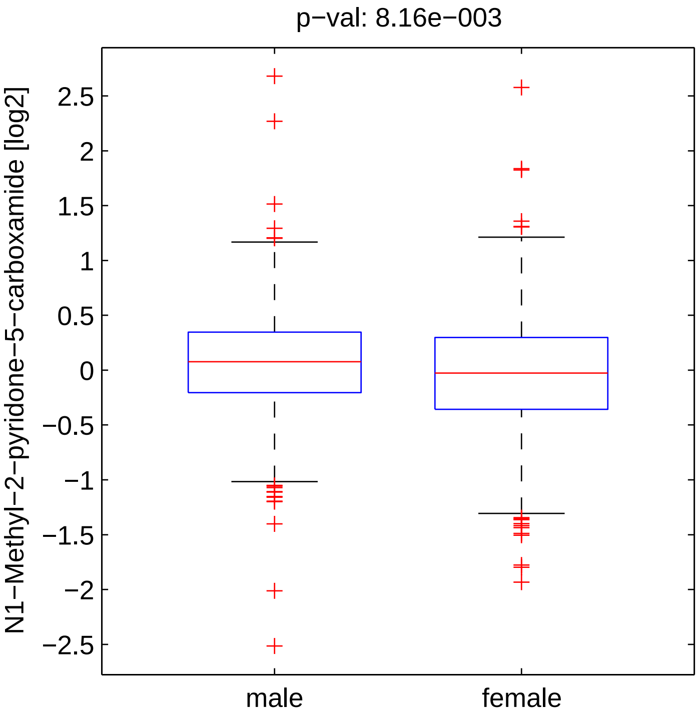
p−val: 8.16e−003
N1−Methyl−2−pyridone−5−carboxamide [log2]
2.5
2
1.5
1
0.5
0
−0.5
−1
−1.5
−2
−2.5
male
female

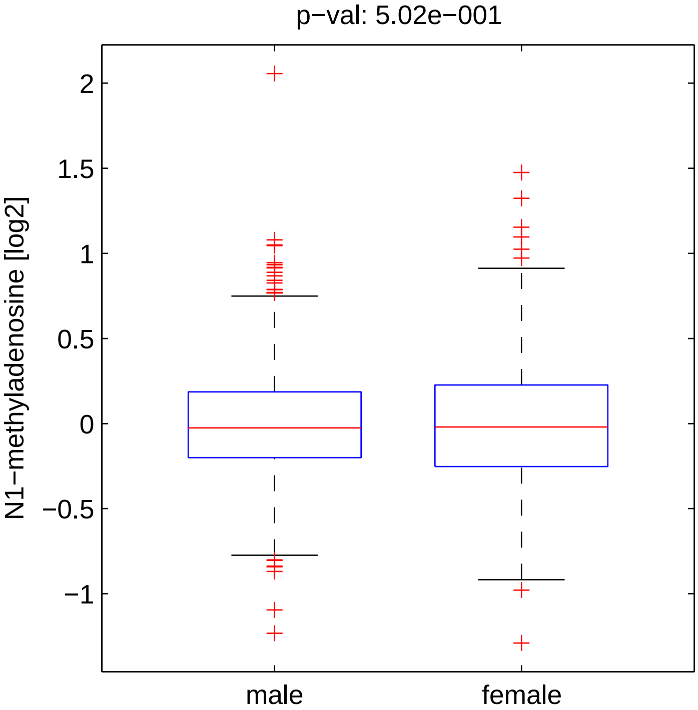
p−val: 5.02e−001
N1−methyladenosine [log2]
2
1.5
1
0.5
0
−0.5
−1
male
female

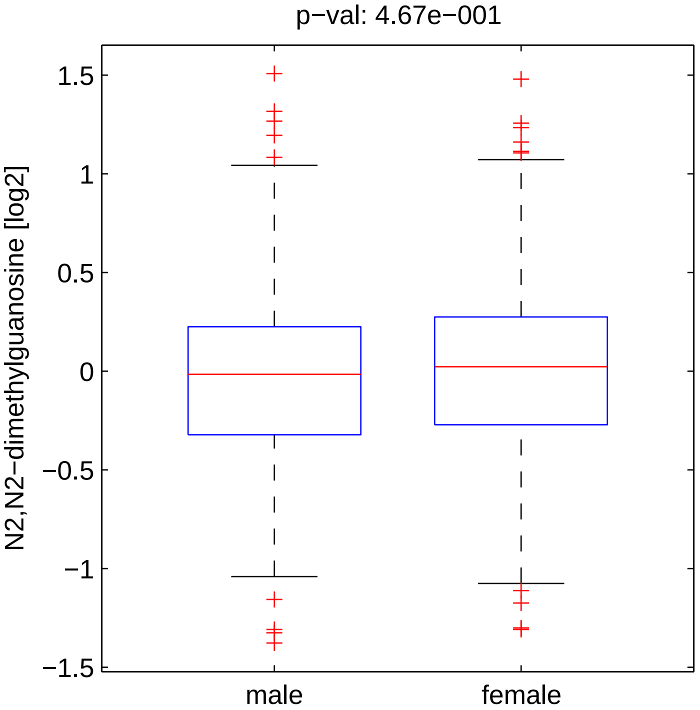
p−val: 4.67e−001
N2,N2−dimethylguanosine [log2]
1.5
1
0.5
0
−0.5
−1
−1.5
male
female

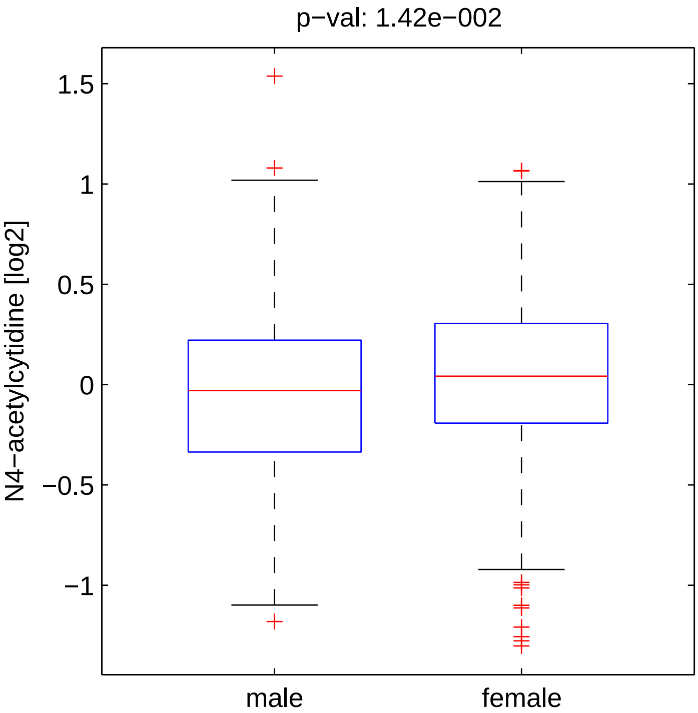
p−val: 1.42e−002
N4−acetylcytidine [log2]
1.5
1
0.5
0
−0.5
−1
male
female

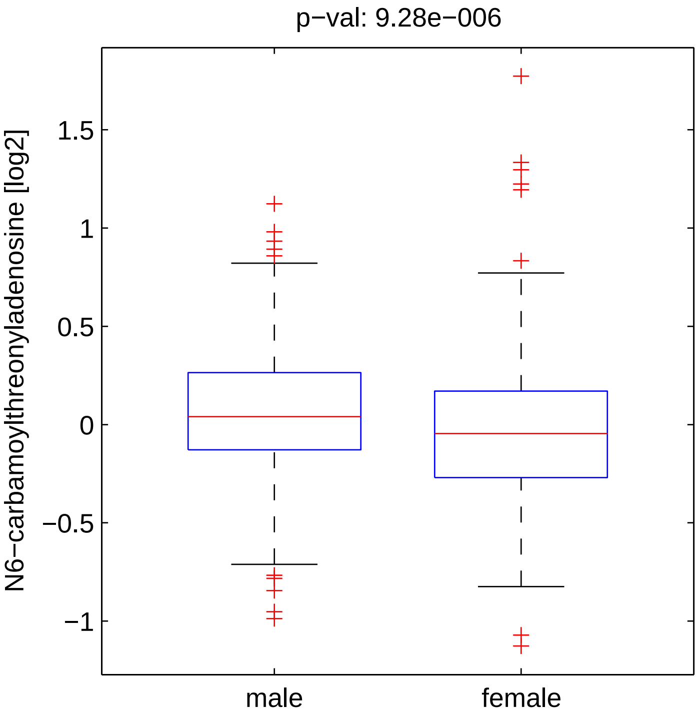

p−val: 9.28e−006
N6−carbamoylthreonyladenosine [log2]
1.5
1
0.5
0
−0.5
−1
male
female

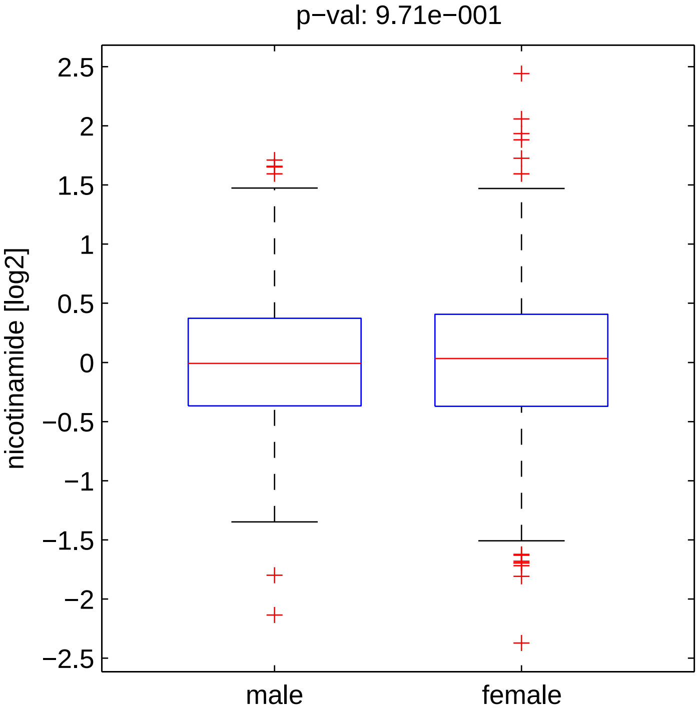
p−val: 9.71e−001
nicotinamide [log2]
2.5
2
1.5
1
0.5
0
−0.5
−1
−1.5
−2
−2.5
male
female

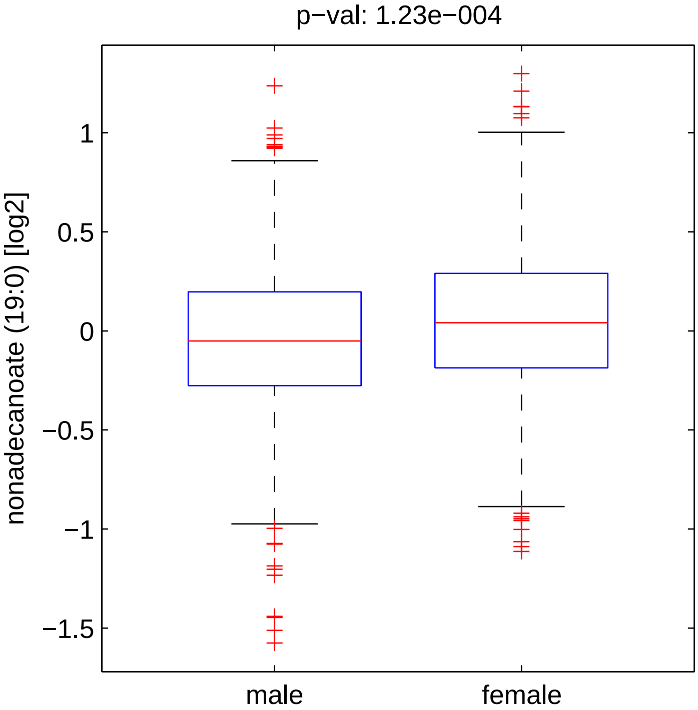
p−val: 1.23e−004
nonadecanoate (19:0) [log2]
1
0.5
0
−0.5
−1
−1.5
male
female

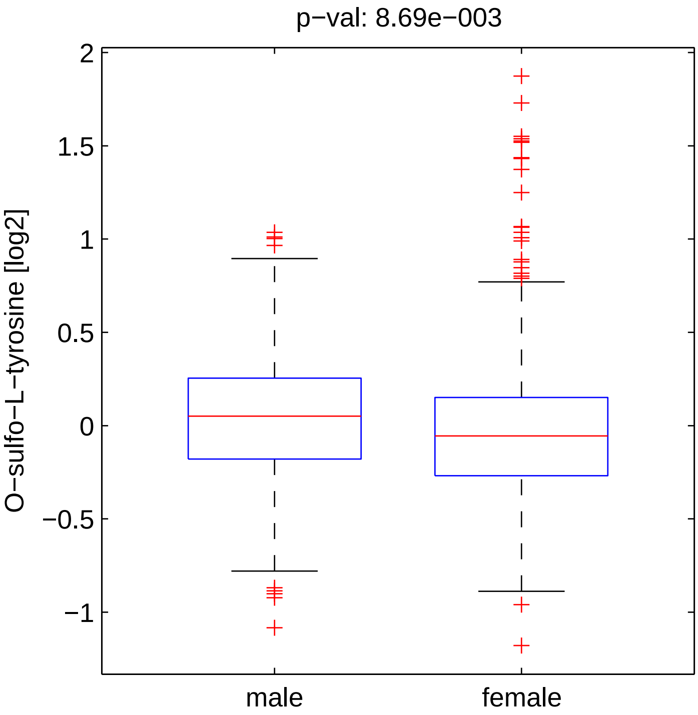
p−val: 8.69e−003
O−sulfo−L−tyrosine [log2]
2
1.5
1
0.5
0
−0.5
−1
male
female

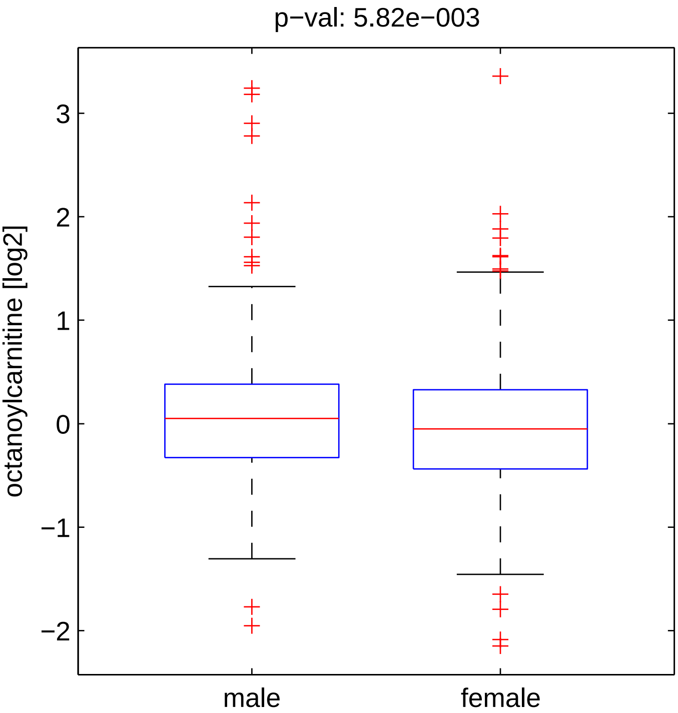
p−val: 5.82e−003
octanoylcarnitine [log2]
3
2
1
0
−1
−2
male
female

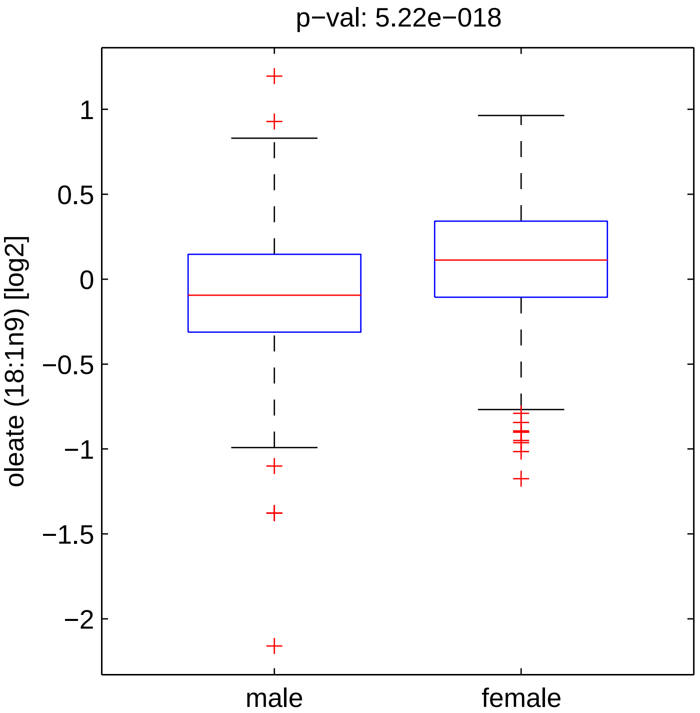

p−val: 5.22e−018
oleate (18:1n9) [log2]
1
0.5
0
−0.5
−1
−1.5
−2
male
female

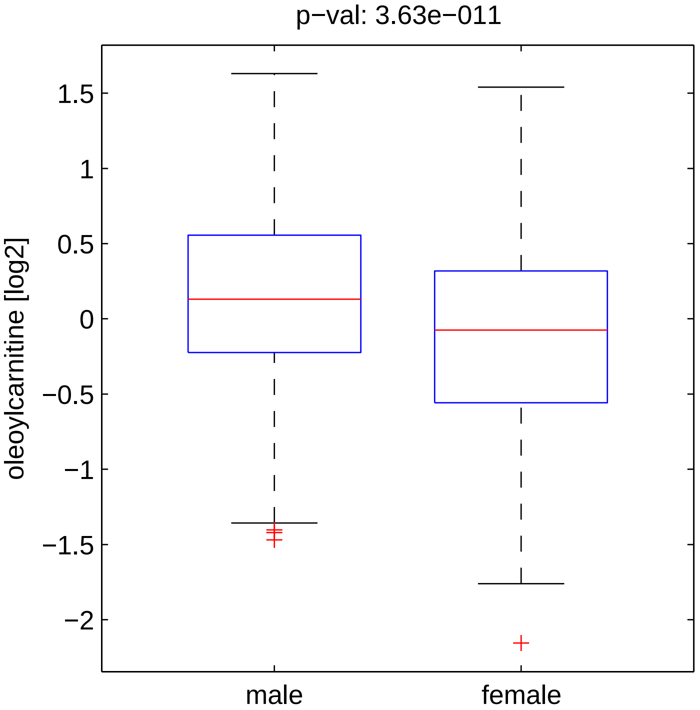
p−val: 3.63e−011
oleoylcarnitine [log2]
1.5
1
0.5
0
−0.5
−1
−1.5
−2
male
female

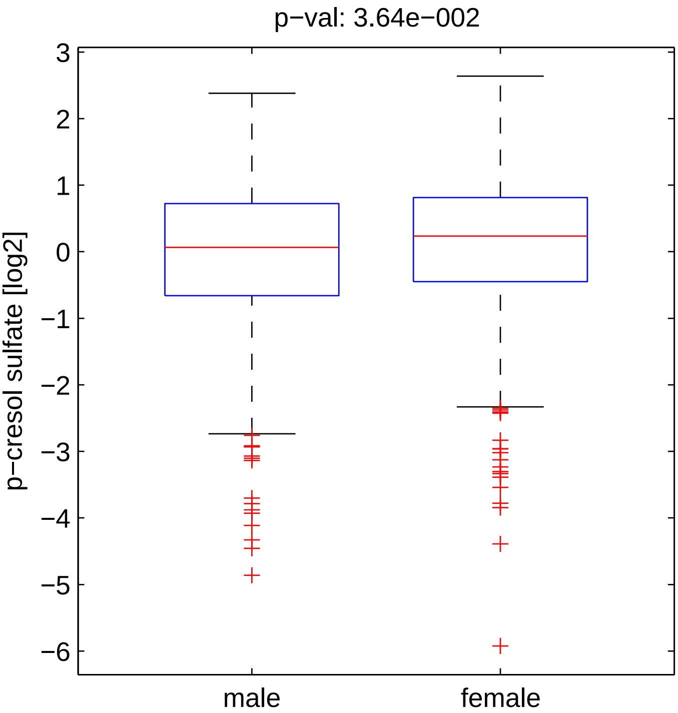

p−val: 3.64e−002
p−cresol sulfate [log2]
3
2
1
0
−1
−2
−3
−4
−5
−6
male
female

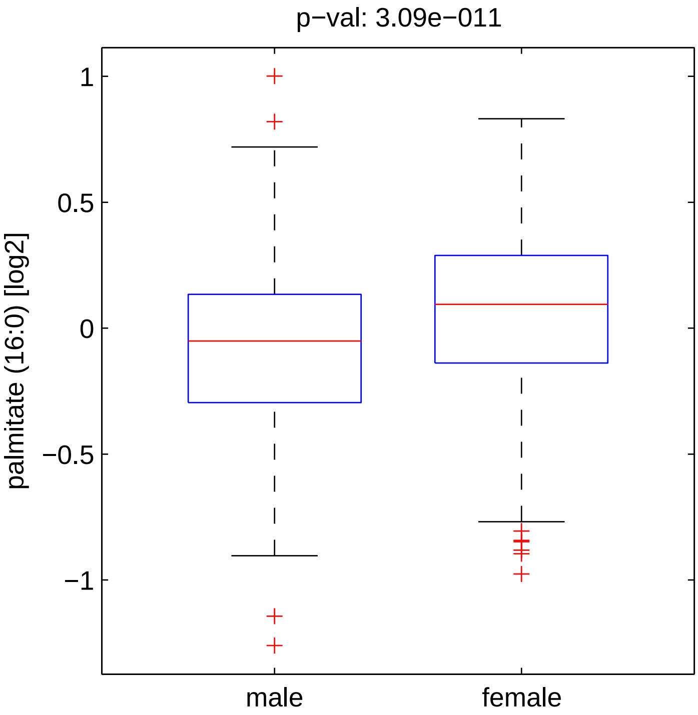

p−val: 3.09e−011
1
0.5
0
−0.5
−1
palmitate (16:0) [log2]
male
female

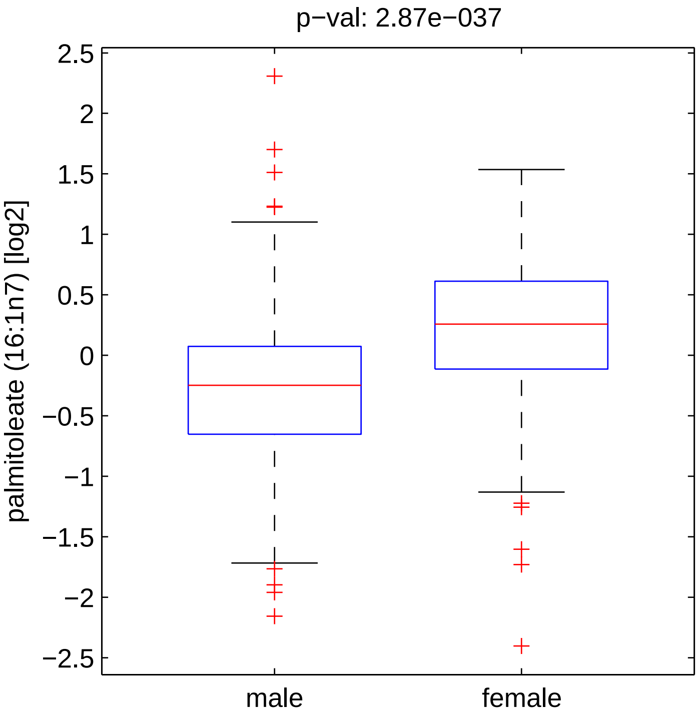
p−val: 2.87e−037
palmitoleate (16:1n7) [log2]
2.5
2
1.5
1
0.5
0
−0.5
−1
−1.5
−2
−2.5
male
female

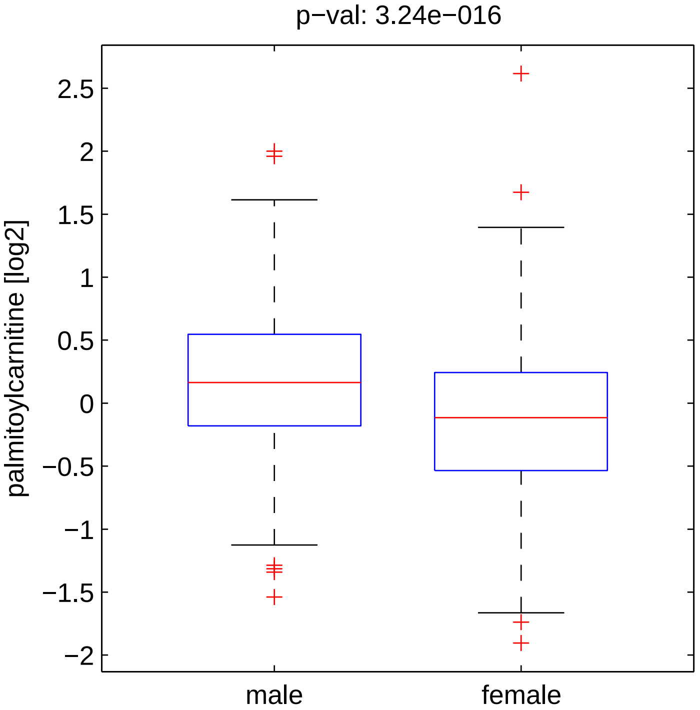
p−val: 3.24e−016
palmitoylcarnitine [log2]
2.5
2
1.5
1
0.5
0
−0.5
−1
−1.5
−2
male
female

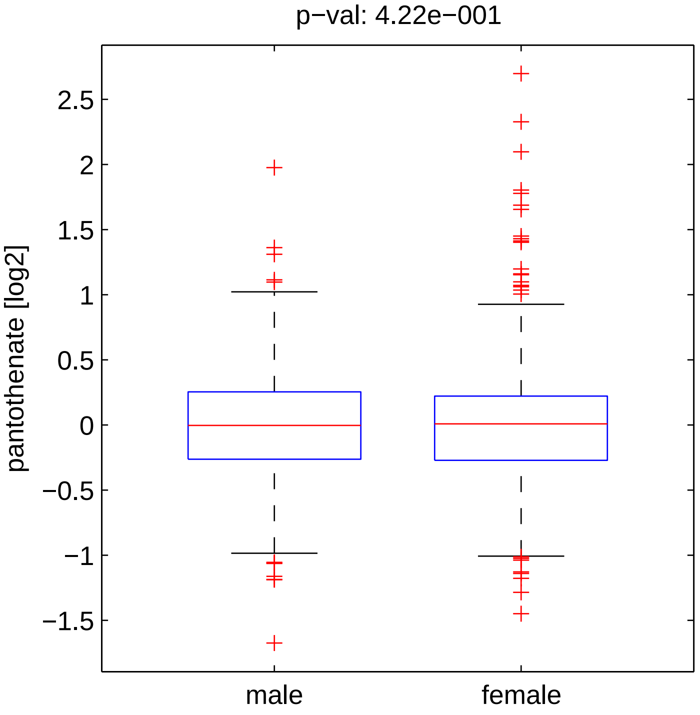

p−val: 4.22e−001
pantothenate [log2]
2.5
2
1.5
1
0.5
0
−0.5
−1
−1.5
male
female

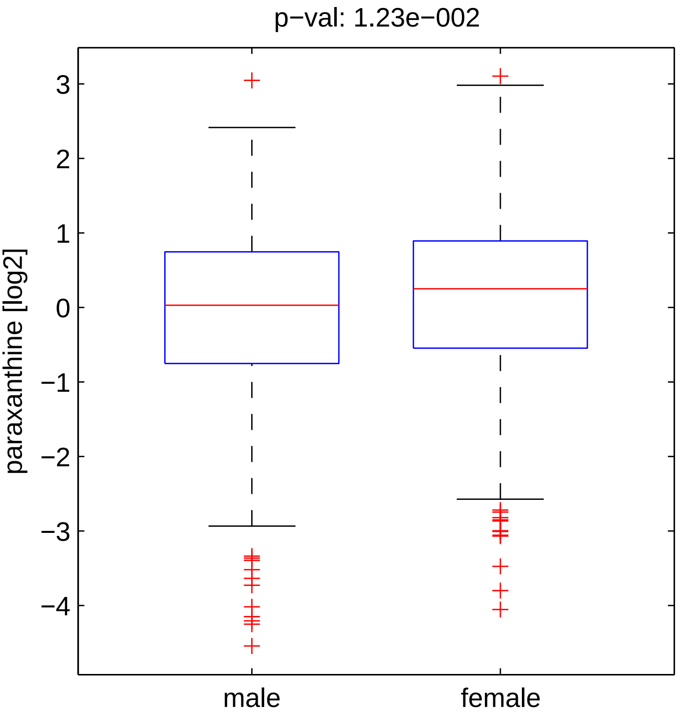
p−val: 1.23e−002
paraxanthine [log2]
3
2
1
0
−1
−2
−3
−4
male
female

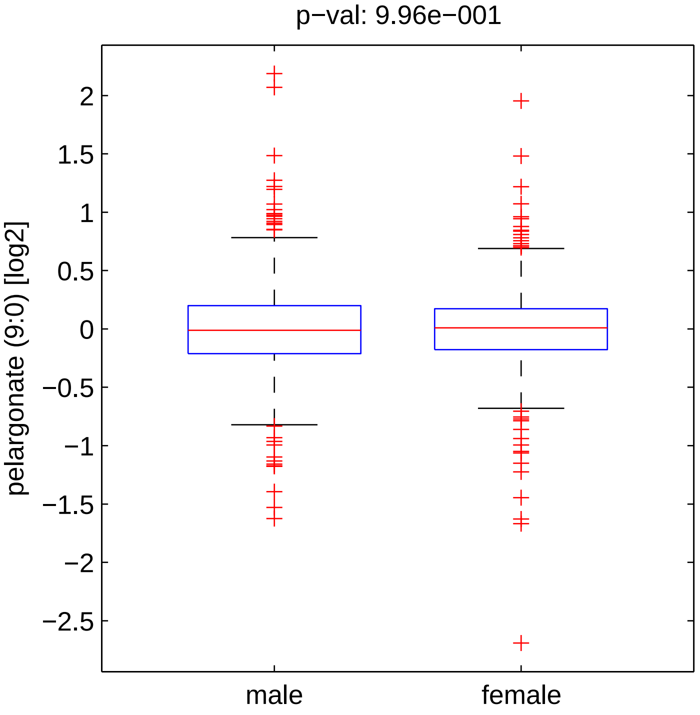

p−val: 9.96e−001
2
1.5
1
0.5
0
−0.5
−1
−1.5
−2
−2.5
pelargonate (9:0) [log2]
male
female

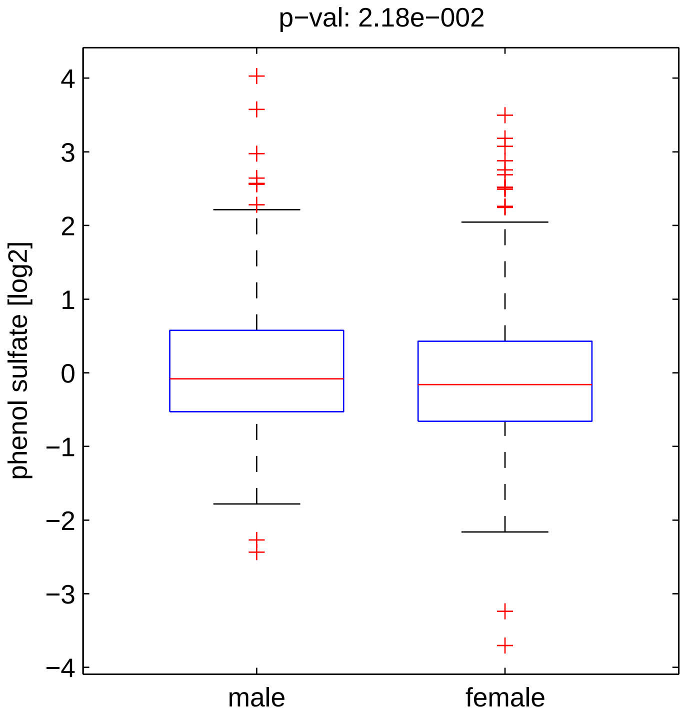
p−val: 2.18e−002
phenol sulfate [log2]
4
3
2
1
0
−1
−2
−3
−4
male
female

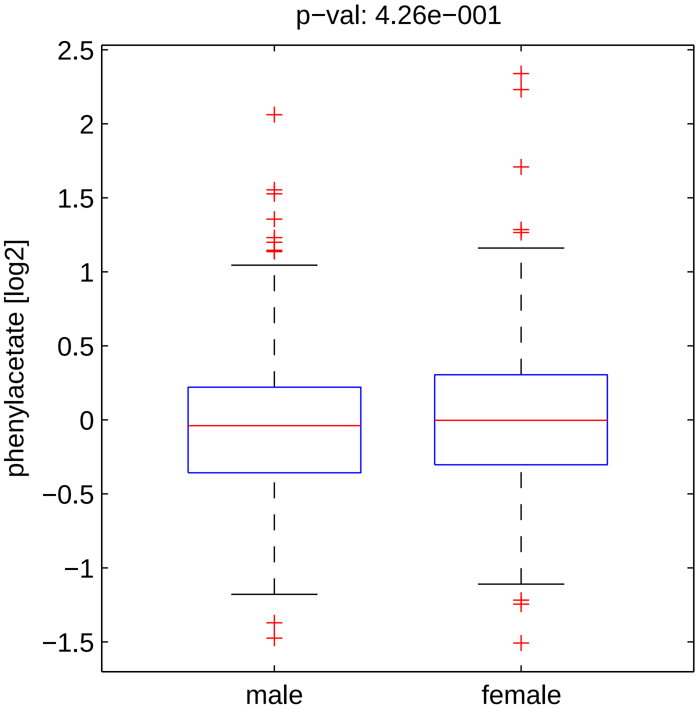
p−val: 4.26e−001
phenylacetate [log2]
2.5
2
1.5
1
0.5
0
−0.5
−1
−1.5
male
female

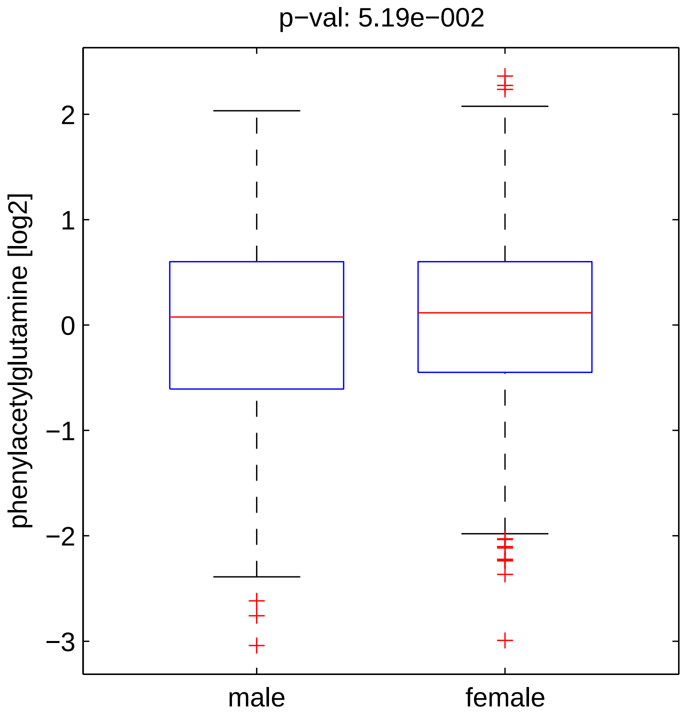

p−val: 5.19e−002
phenylacetylglutamine [log2]
2
1
0
−1
−2
−3
male
female

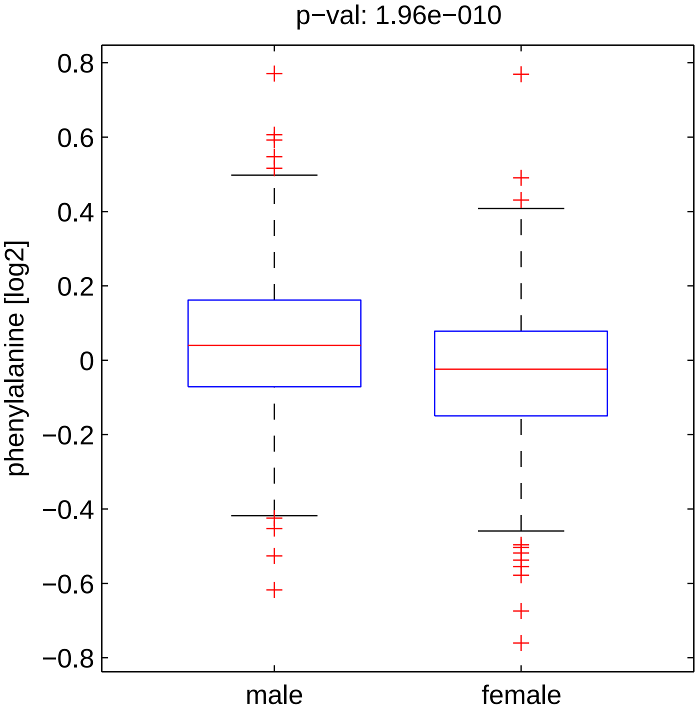
p−val: 1.96e−010
0.8
0.6
0.4
0.2
0
−0.2
−0.4
−0.6
−0.8
phenylalanine [log2]
male
female

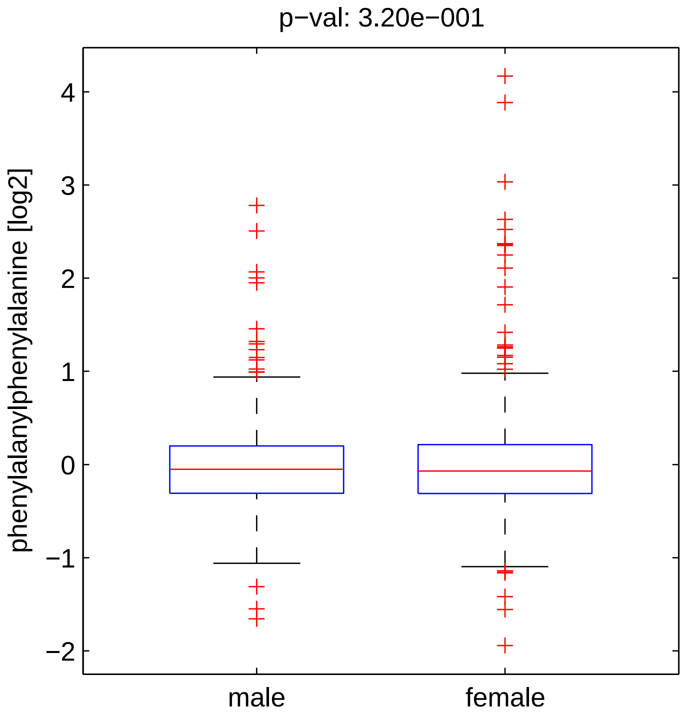
p−val: 3.20e−001
phenylalanylphenylalanine [log2]
4
3
2
1
0
−1
−2
male
female

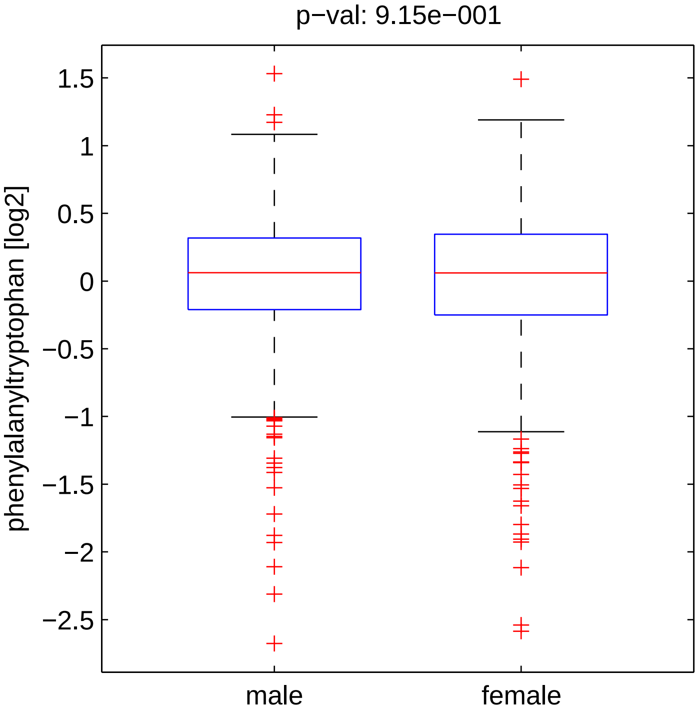

p−val: 9.15e−001
phenylalanyltryptophan [log2]
1.5
1
0.5
0
−0.5
−1
−1.5
−2
−2.5
male
female

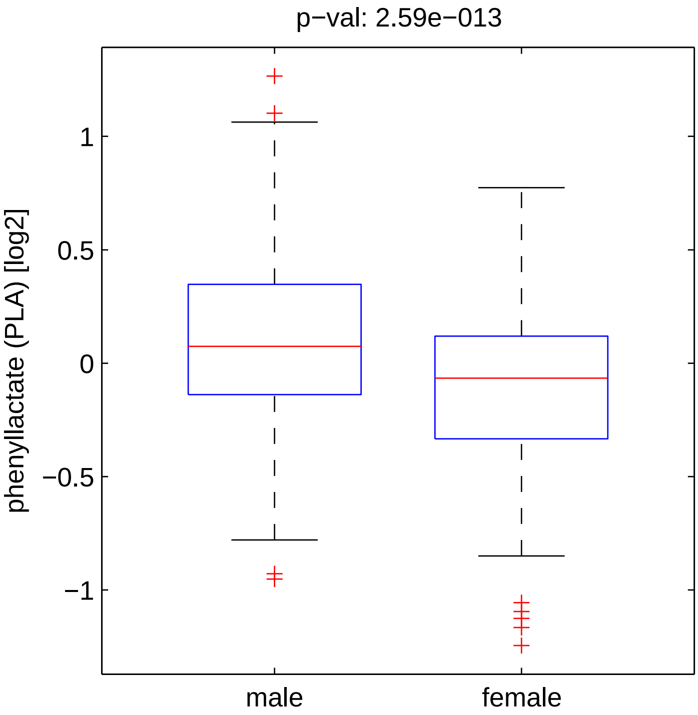

p−val: 2.59e−013
phenyllactate (PLA) [log2]
1
0.5
0
−0.5
−1
male
female

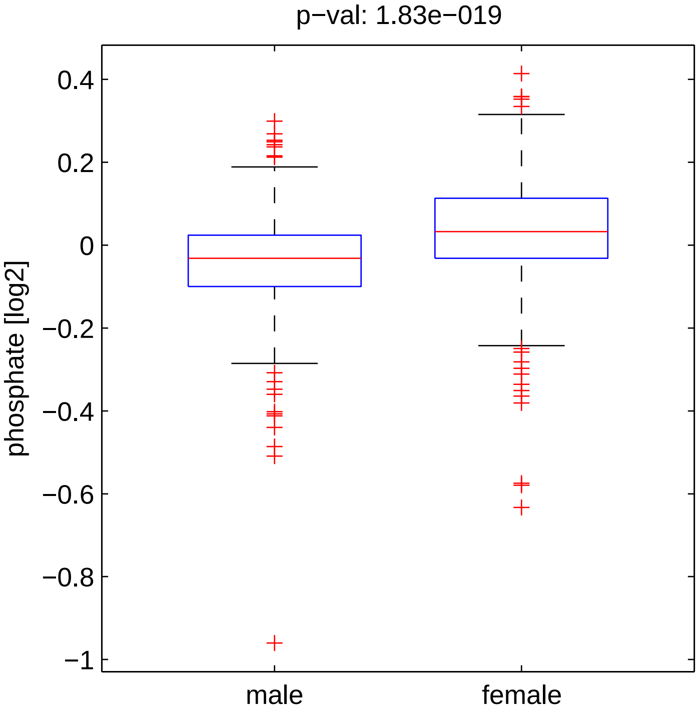

p−val: 1.83e−019
0.4
0.2
0
−0.2
−0.4
−0.6
−0.8
−1
phosphate [log2]
male
female

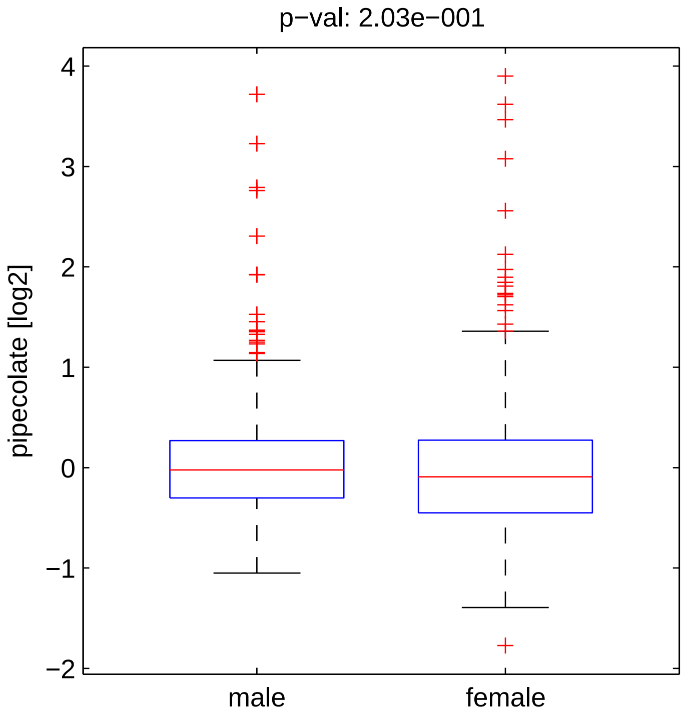
p−val: 2.03e−001
pipecolate [log2]
4
3
2
1
0
−1
−2
male
female

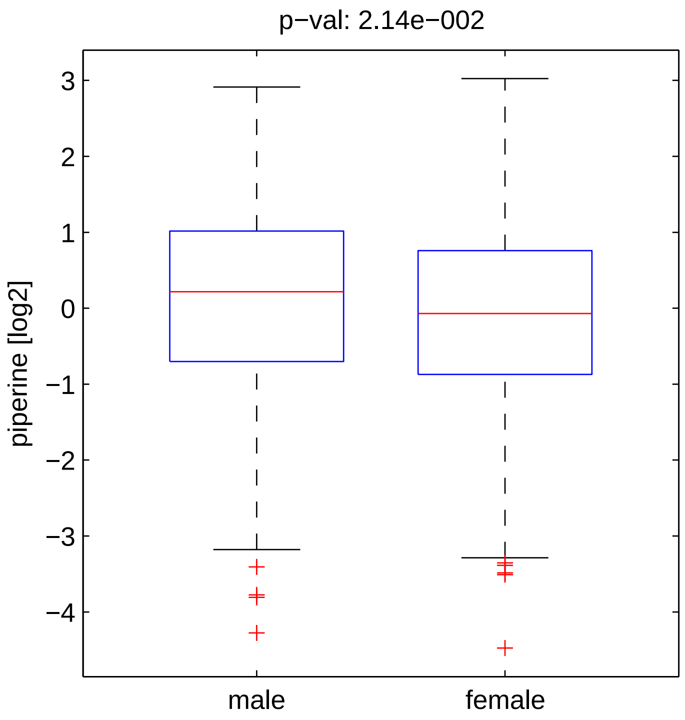

p−val: 2.14e−002
3
2
1
0
−1
−2
−3
−4
piperine [log2]
male
female

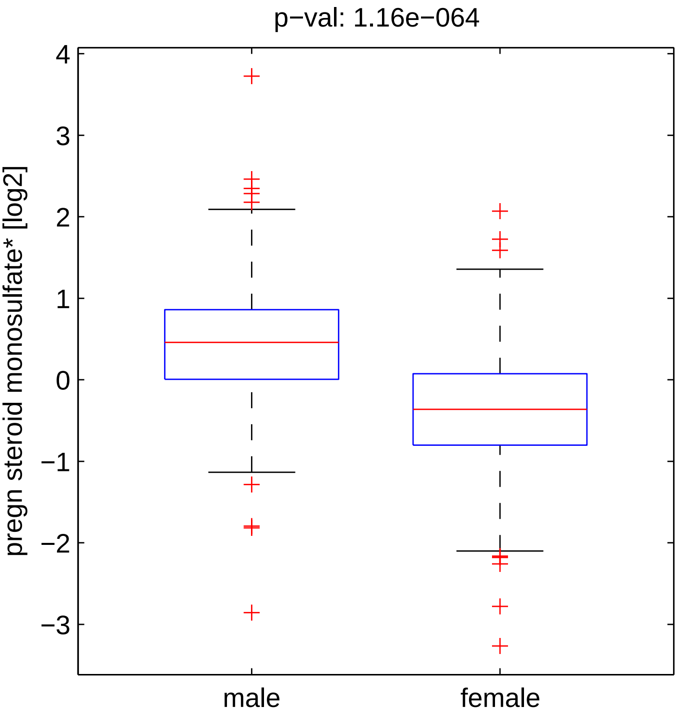
p−val: 1.16e−064
pregn steroid monosulfate* [log2]
4
3
2
1
0
−1
−2
−3
male
female

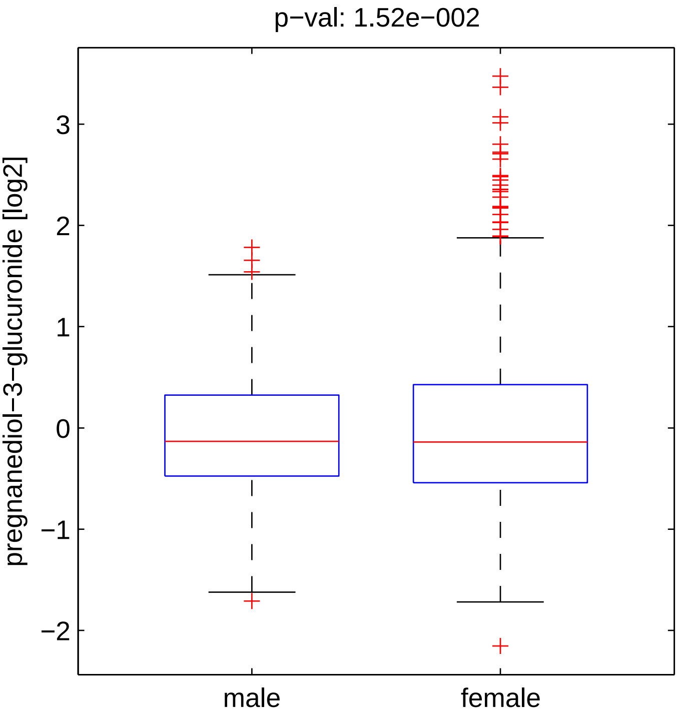
p−val: 1.52e−002
pregnanediol−3−glucuronide [log2]
3
2
1
0
−1
−2
male
female

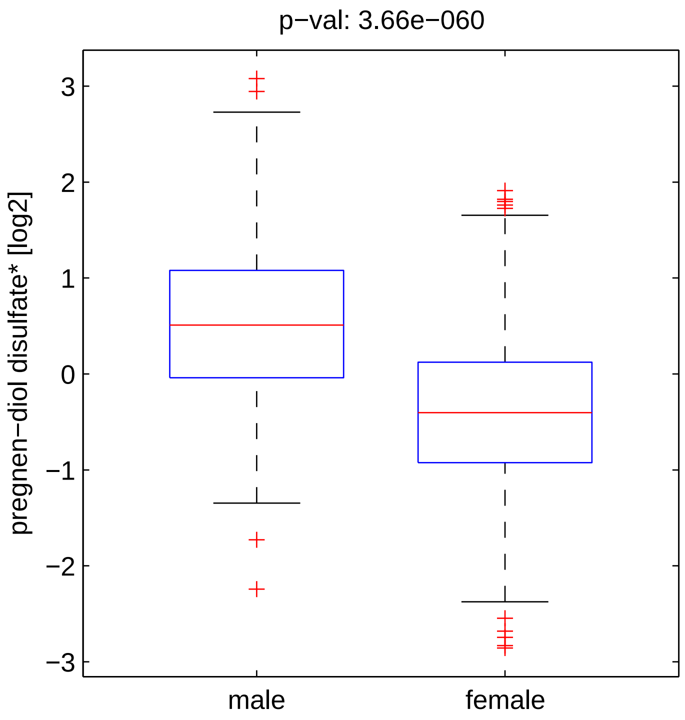
p−val: 3.66e−060
pregnen−diol disulfate* [log2]
3
2
1
0
−1
−2
−3
male
female

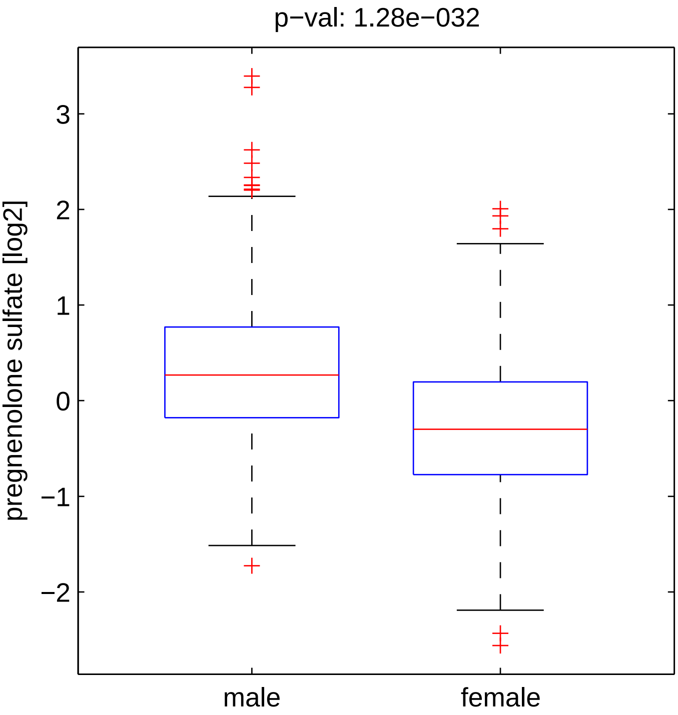

p−val: 1.28e−032
pregnenolone sulfate [log2]
3
2
1
0
−1
−2
male
female

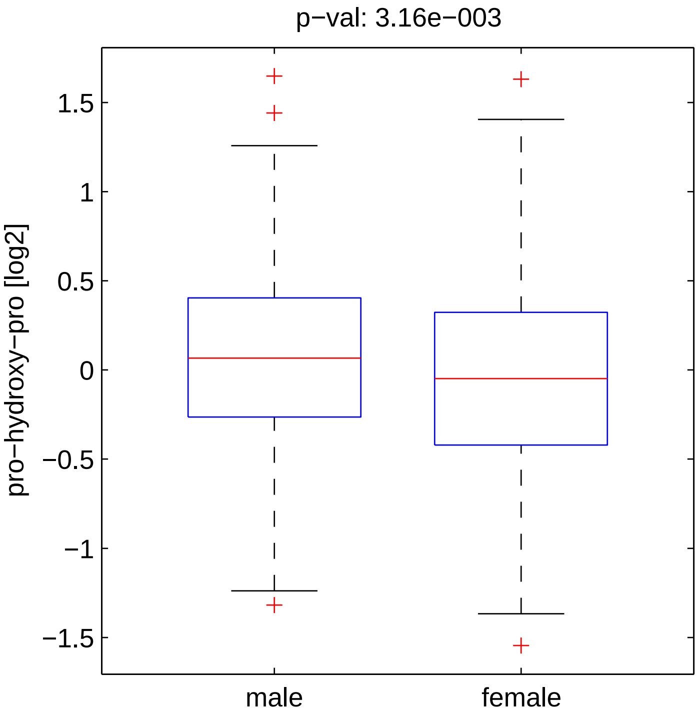

p−val: 3.16e−003
pro−hydroxy−pro [log2]
1.5
1
0.5
0
−0.5
−1
−1.5
male
female

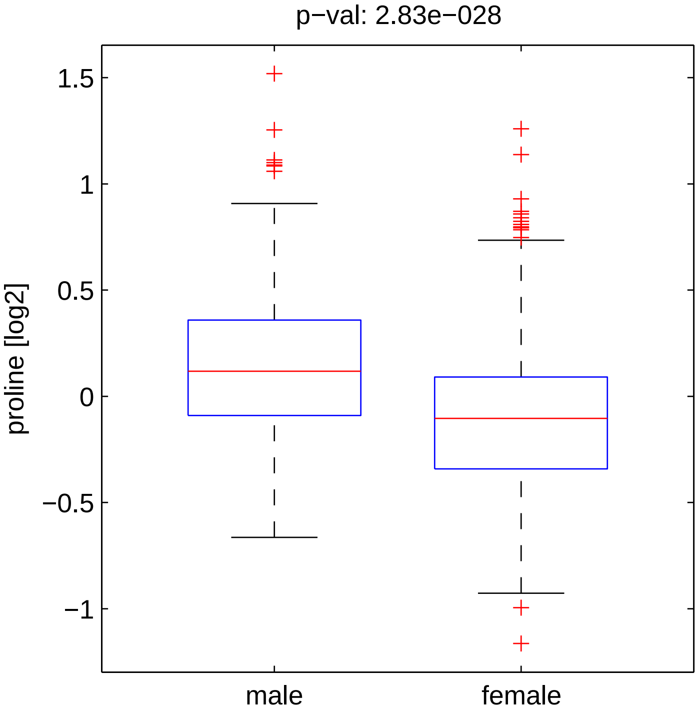
p−val: 2.83e−028
proline [log2]
1.5
1
0.5
0
−0.5
−1
male
female

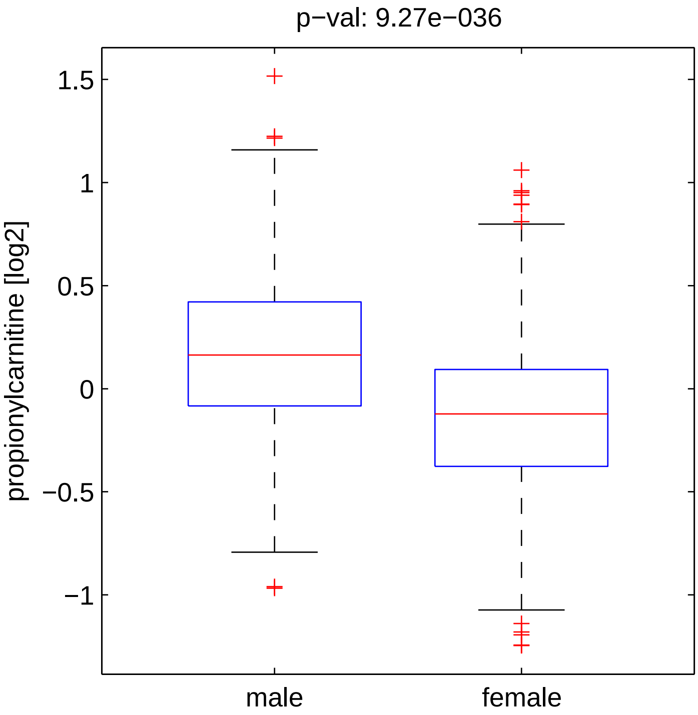
p−val: 9.27e−036
1.5
1
0.5
0
−0.5
−1
propionylcarnitine [log2]
male
female

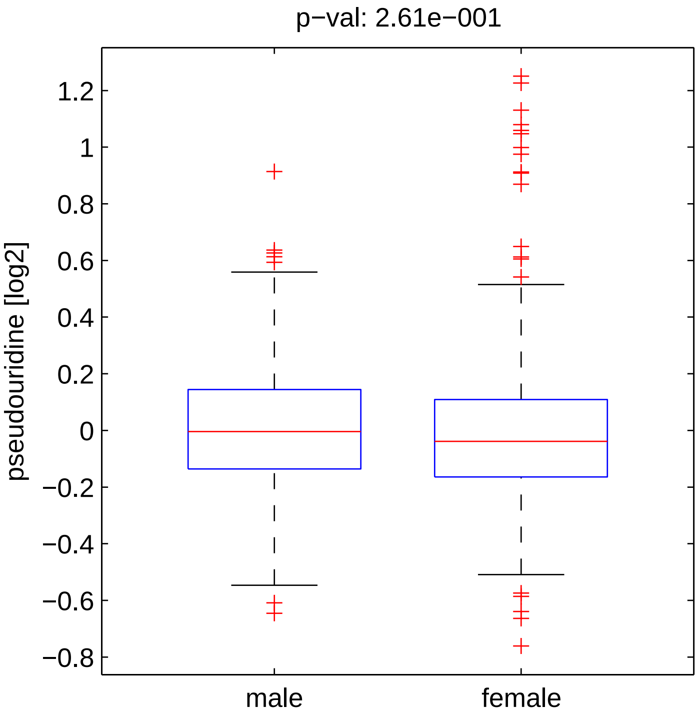
p−val: 2.61e−001
pseudouridine [log2]
1.2
1
0.8
0.6
0.4
0.2
0
−0.2
−0.4
−0.6
−0.8
male
female

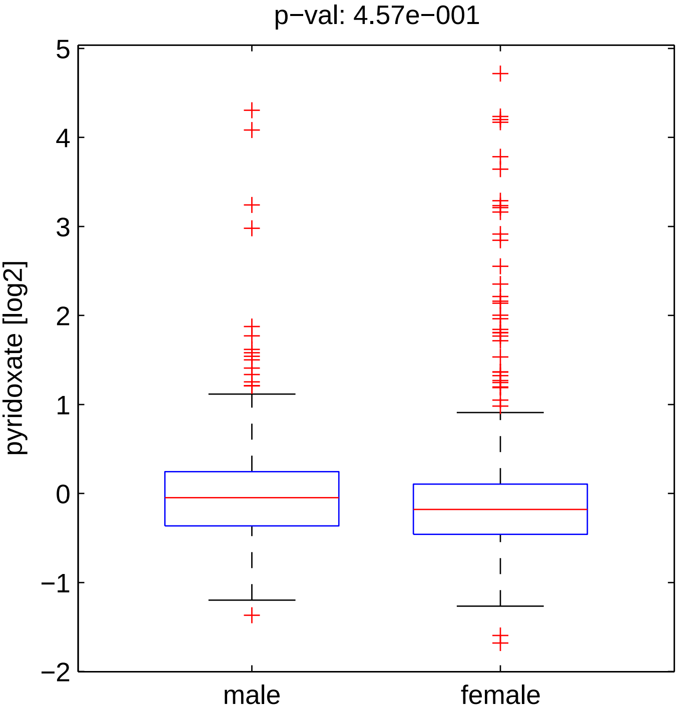

p−val: 4.57e−001
pyridoxate [log2]
5
4
3
2
1
0
−1
−2
male
female

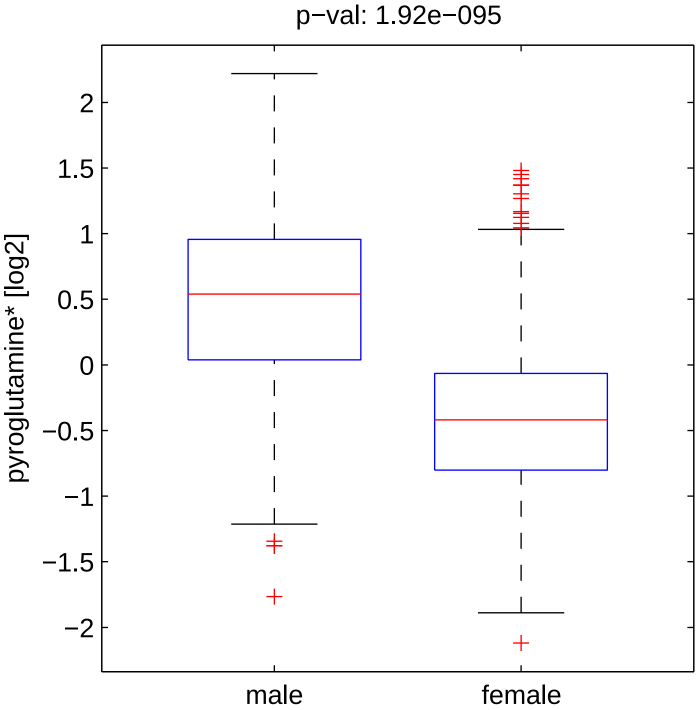
p−val: 1.92e−095
pyroglutamine* [log2]
2
1.5
1
0.5
0
−0.5
−1
−1.5
−2
male
female

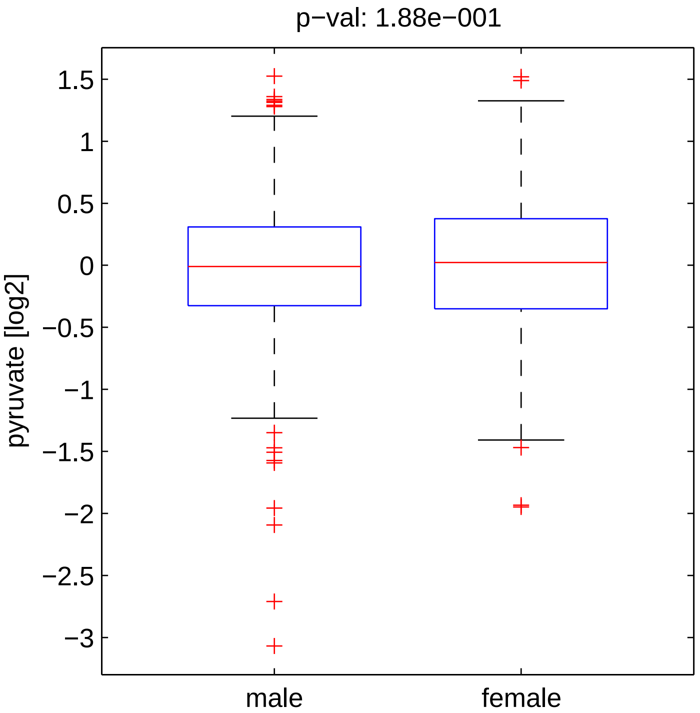

p−val: 1.88e−001
pyruvate [log2]
1.5
1
0.5
0
−0.5
−1
−1.5
−2
−2.5
−3
male
female

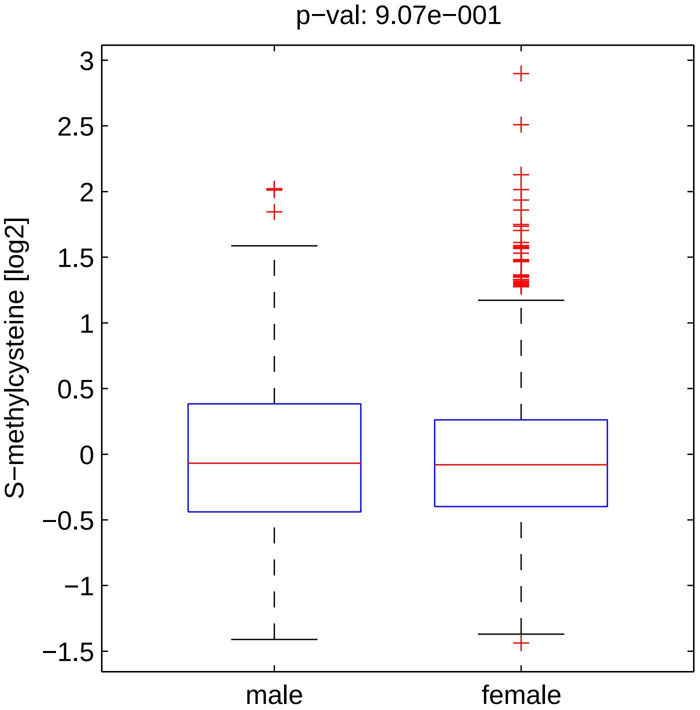
p−val: 9.07e−001
S−methylcysteine [log2]
3
2.5
2
1.5
1
0.5
0
−0.5
−1
−1.5
male
female

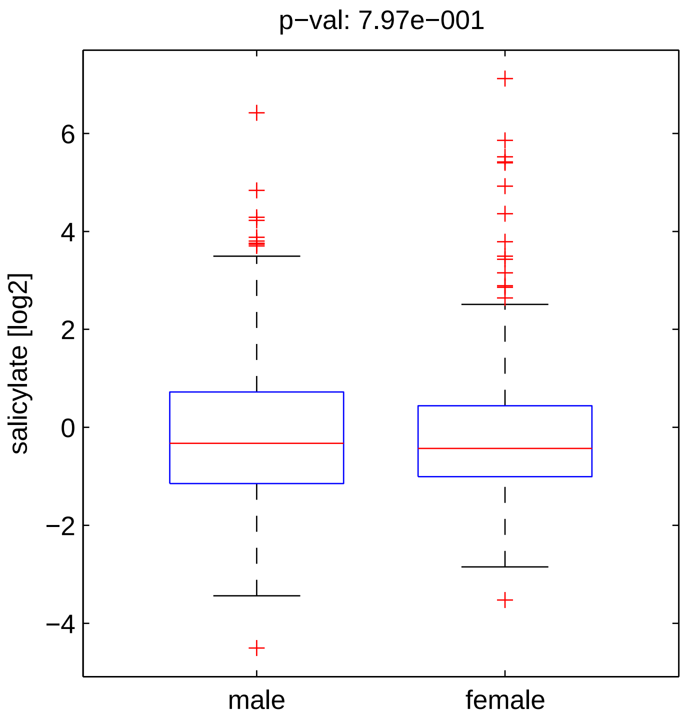
p−val: 7.97e−001
salicylate [log2]
6
4
2
0
−2
−4
male
female

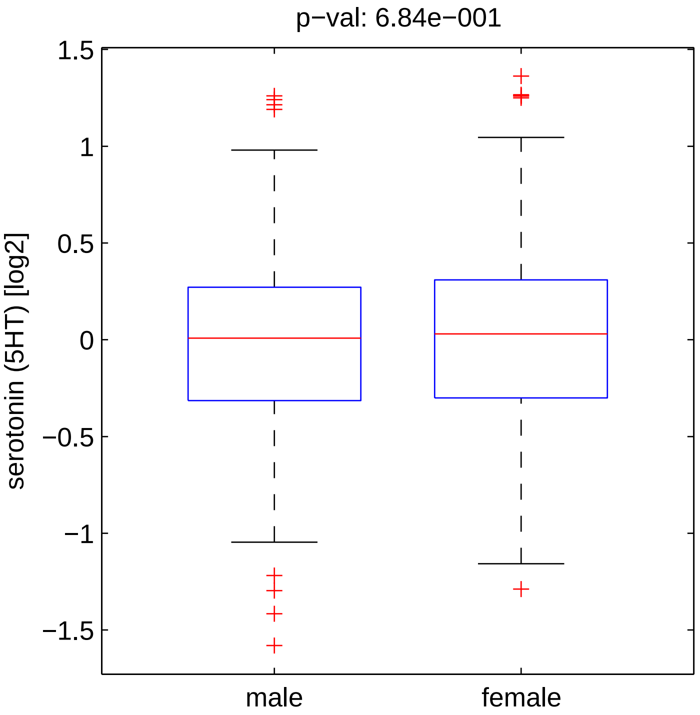
p−val: 6.84e−001
1.5
1
0.5
0
−0.5
−1
−1.5
serotonin (5HT) [log2]
male
female

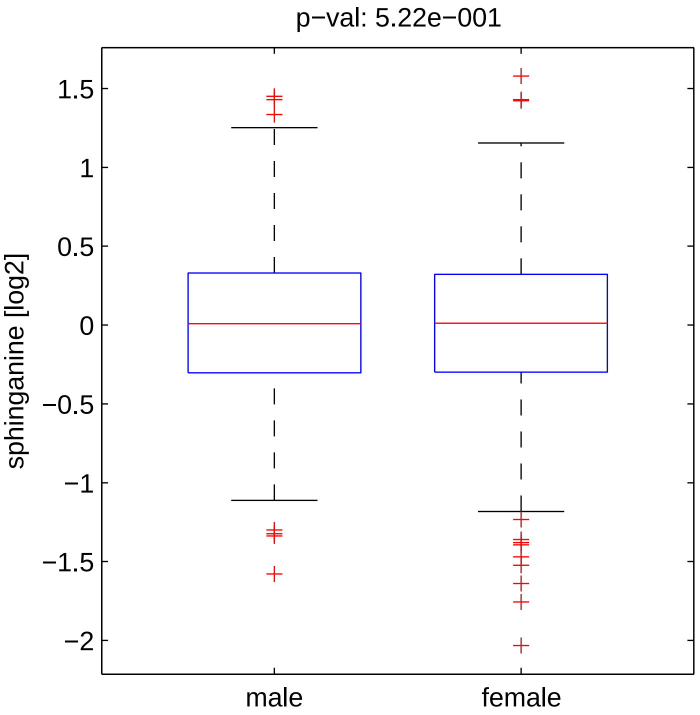
p−val: 5.22e−001
sphinganine [log2]
1.5
1
0.5
0
−0.5
−1
−1.5
−2
male
female

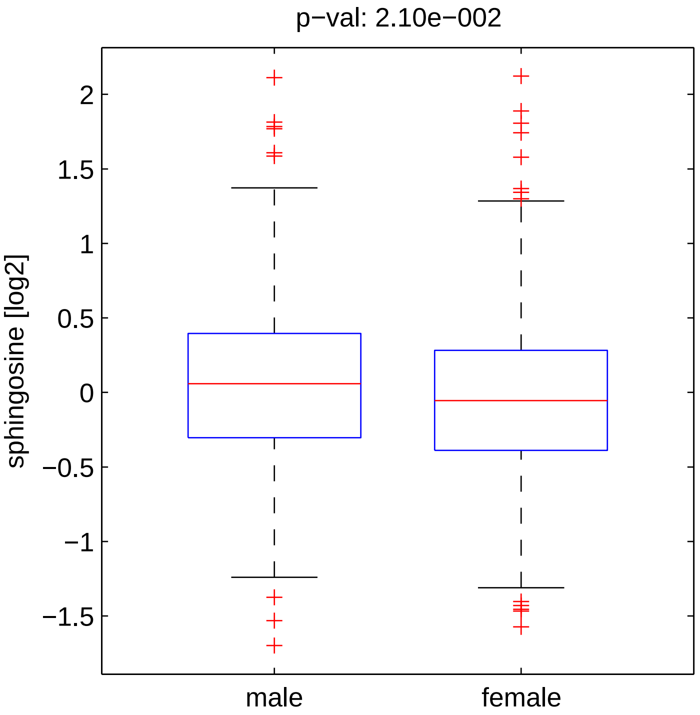
p−val: 2.10e−002
sphingosine [log2]
2
1.5
1
0.5
0
−0.5
−1
−1.5
male
female

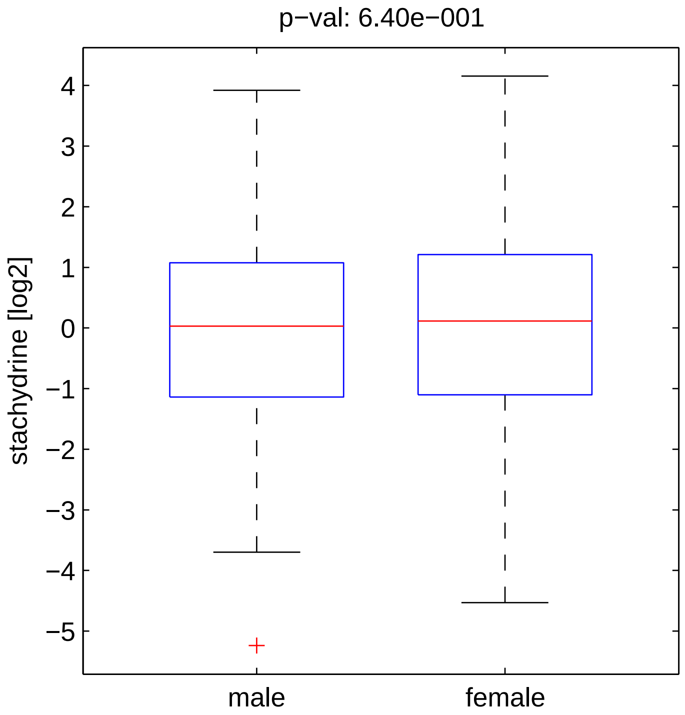
p−val: 6.40e−001
stachydrine [log2]
4
3
2
1
0
−1
−2
−3
−4
−5
male
female

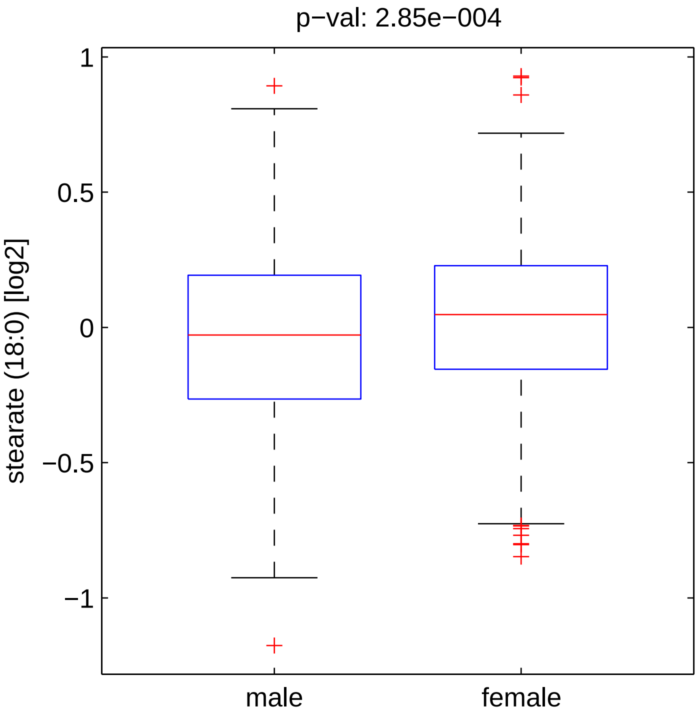

p−val: 2.85e−004
1
0.5
0
−0.5
−1
stearate (18:0) [log2]
male
female

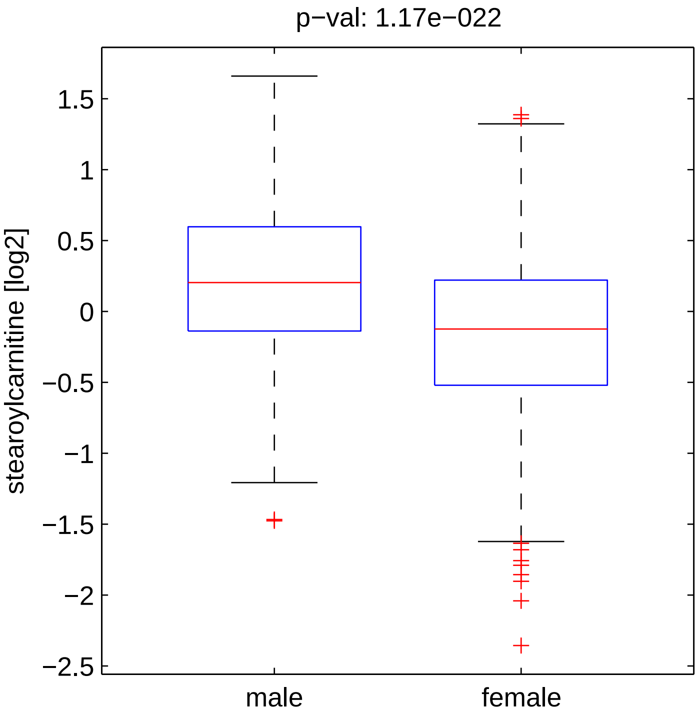
p−val: 1.17e−022
stearoylcarnitine [log2]
1.5
1
0.5
0
−0.5
−1
−1.5
−2
−2.5
male
female

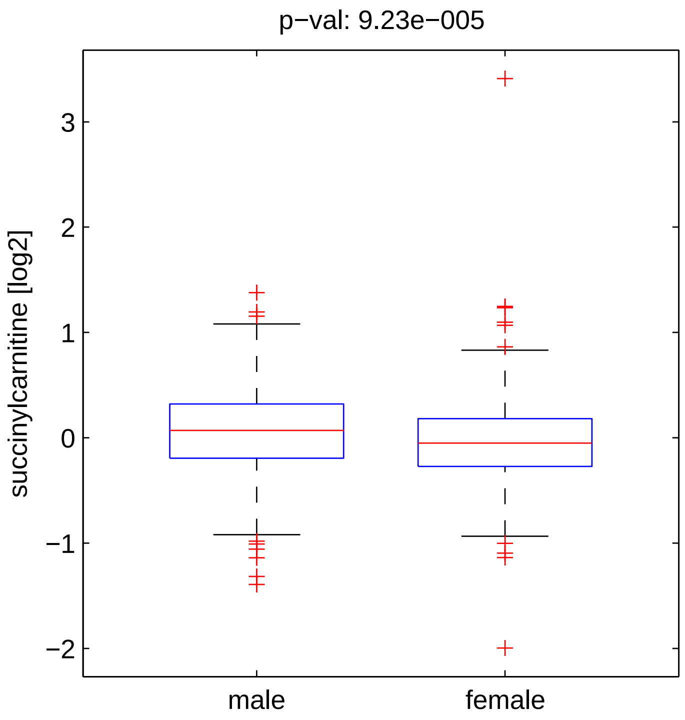
p−val: 9.23e−005
succinylcarnitine [log2]
3
2
1
0
−1
−2
male
female

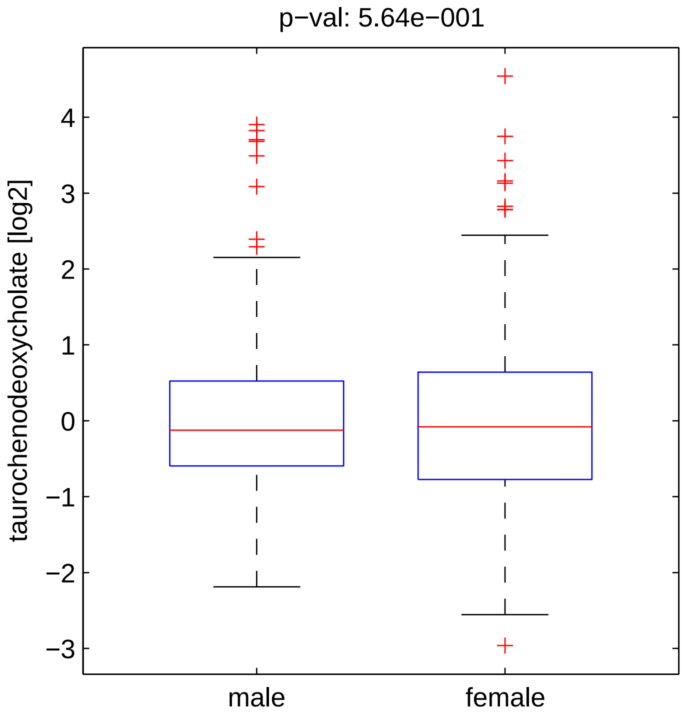
p−val: 5.64e−001
taurochenodeoxycholate [log2]
4
3
2
1
0
−1
−2
−3
male
female

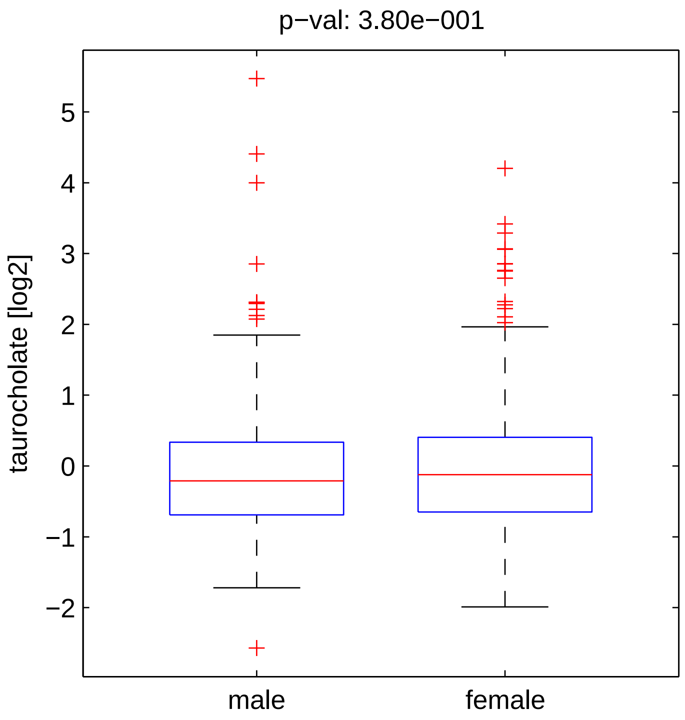

p−val: 3.80e−001
taurocholate [log2]
5
4
3
2
1
0
−1
−2
male
female

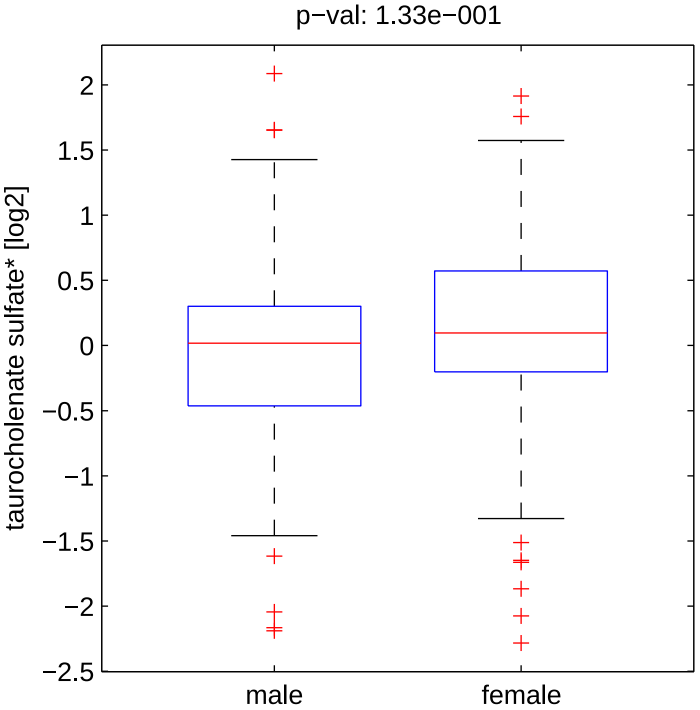
p−val: 1.33e−001
taurocholenate sulfate* [log2]
2
1.5
1
0.5
0
−0.5
−1
−1.5
−2
−2.5
male
female

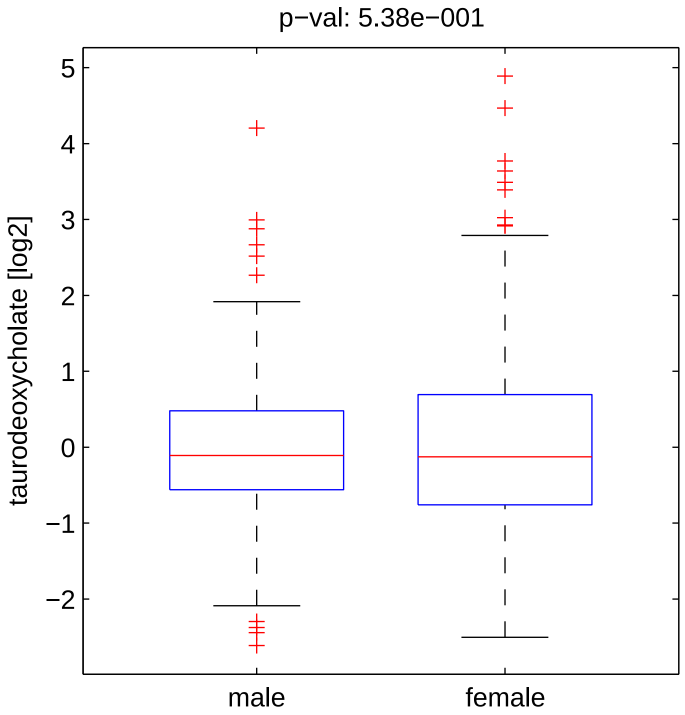
p−val: 5.38e−001
taurodeoxycholate [log2]
5
4
3
2
1
0
−1
−2
male
female

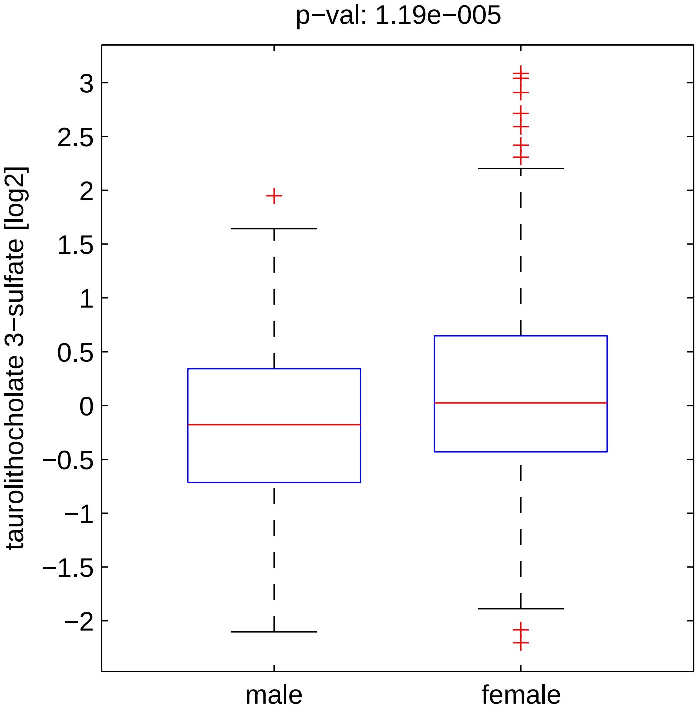
p−val: 1.19e−005
taurolithocholate 3−sulfate [log2]
3
2.5
2
1.5
1
0.5
0
−0.5
−1
−1.5
−2
male
female

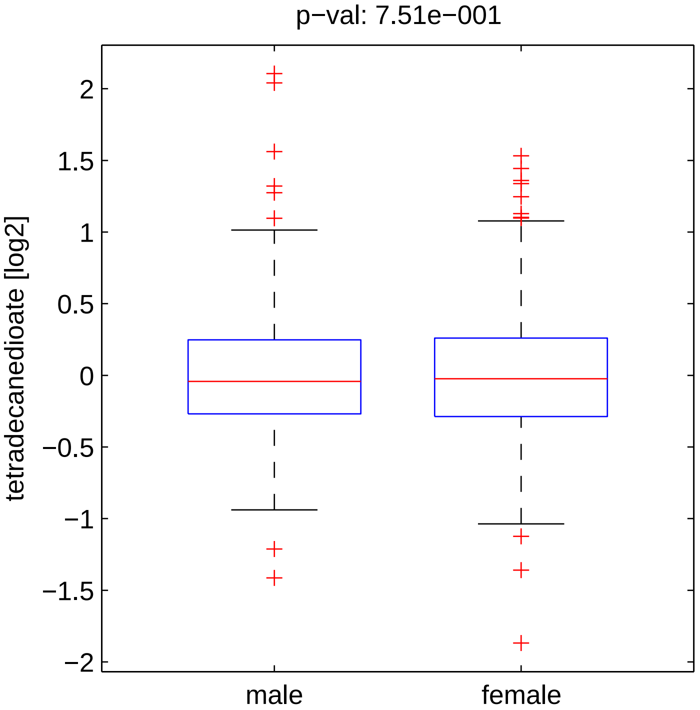
p−val: 7.51e−001
tetradecanedioate [log2]
2
1.5
1
0.5
0
−0.5
−1
−1.5
−2
male
female

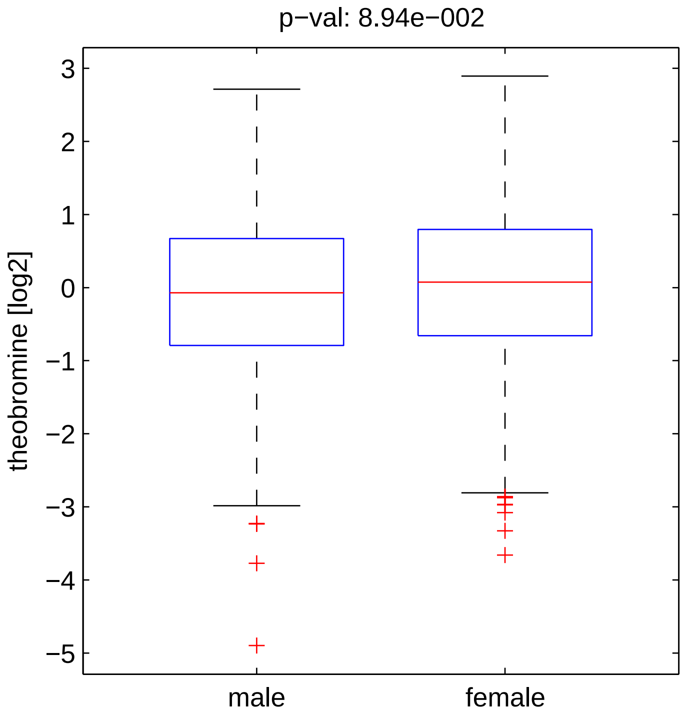

p−val: 8.94e−002
theobromine [log2]
3
2
1
0
−1
−2
−3
−4
−5
male
female

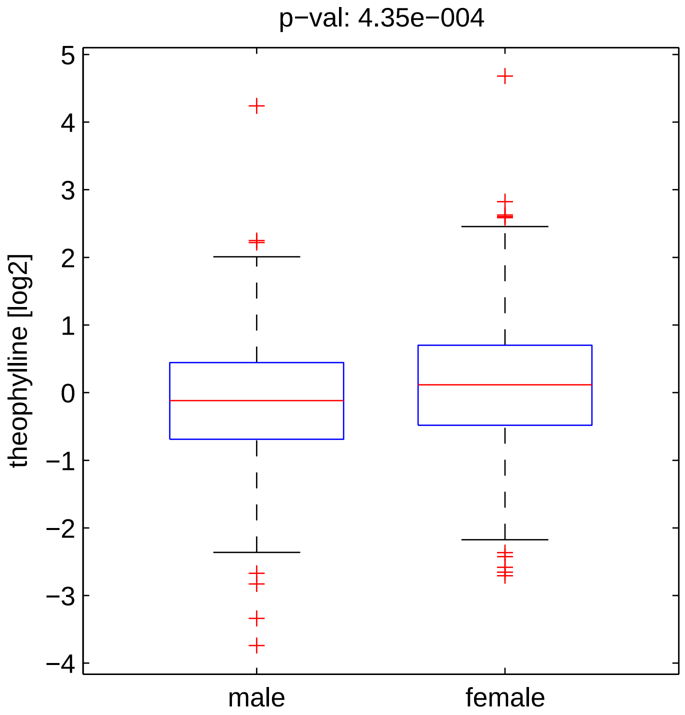

p−val: 4.35e−004
theophylline [log2]
5
4
3
2
1
0
−1
−2
−3
−4
male
female

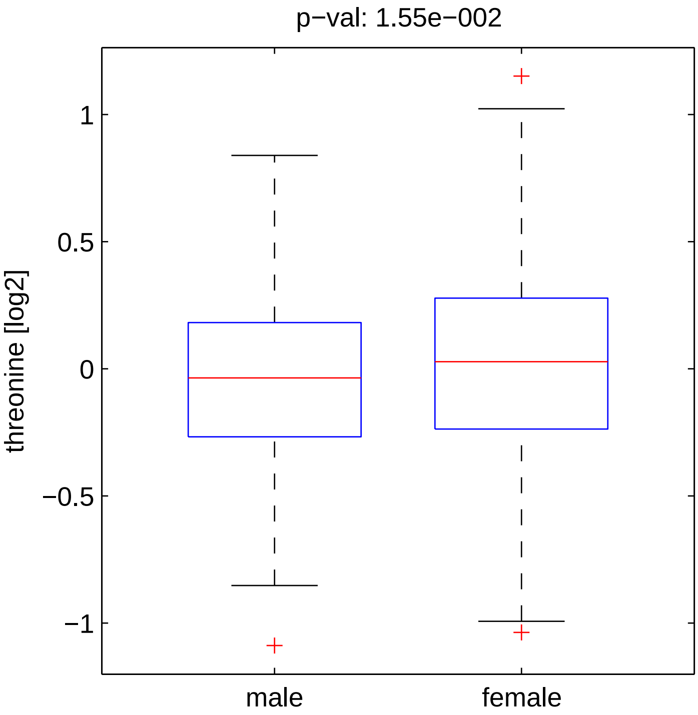
p−val: 1.55e−002
threonine [log2]
1
0.5
0
−0.5
−1
male
female

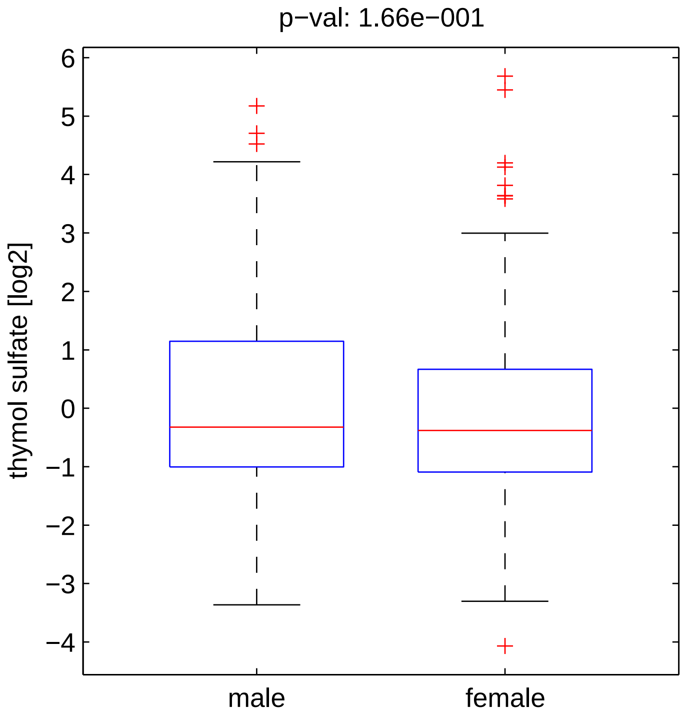
p−val: 1.66e−001
6
5
4
3
2
1
0
−1
−2
−3
−4
thymol sulfate [log2]
male
female

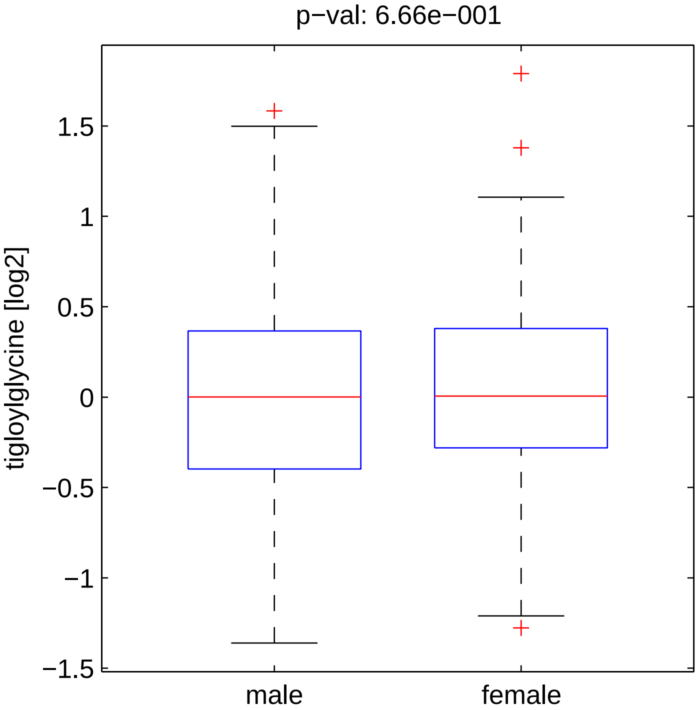
p−val: 6.66e−001
tigloylglycine [log2]
1.5
1
0.5
0
−0.5
−1
−1.5
male
female

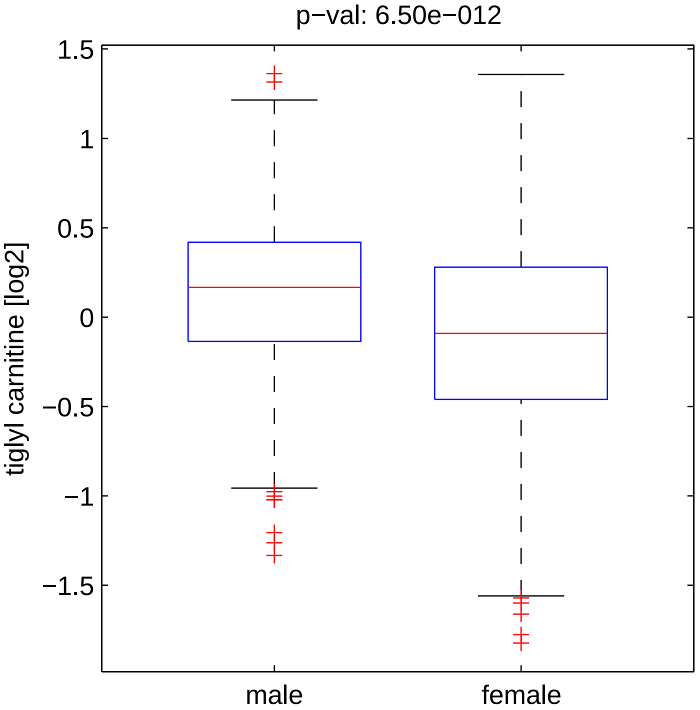
p−val: 6.50e−012
1.5
1
0.5
0
−0.5
−1
−1.5
tiglyl carnitine [log2]
male
female

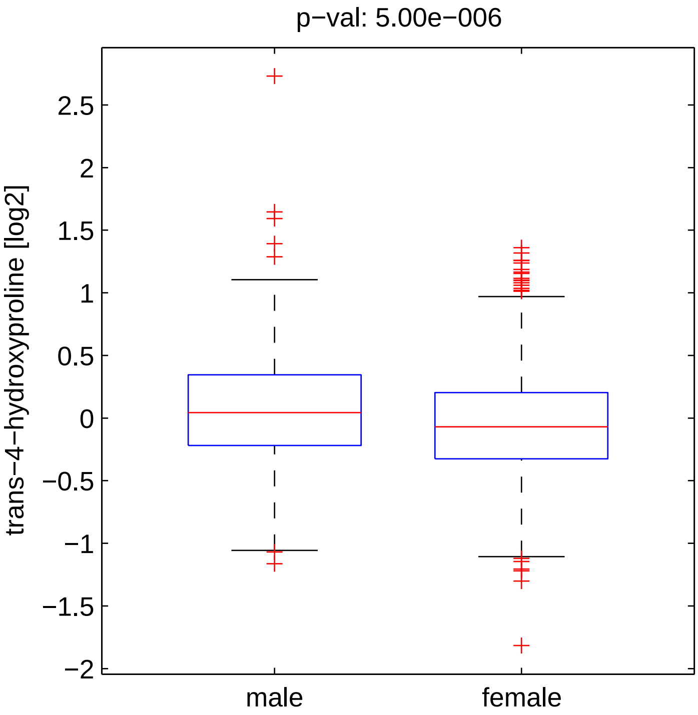
p−val: 5.00e−006
trans−4−hydroxyproline [log2]
2.5
2
1.5
1
0.5
0
−0.5
−1
−1.5
−2
male
female

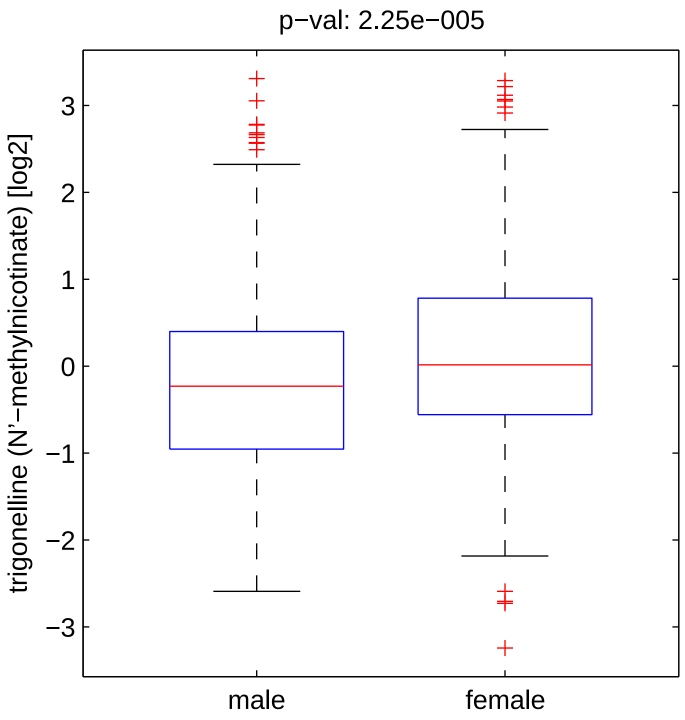
p−val: 2.25e−005
trigonelline (N’−methylnicotinate) [log2]
3
2
1
0
−1
−2
−3
male
female

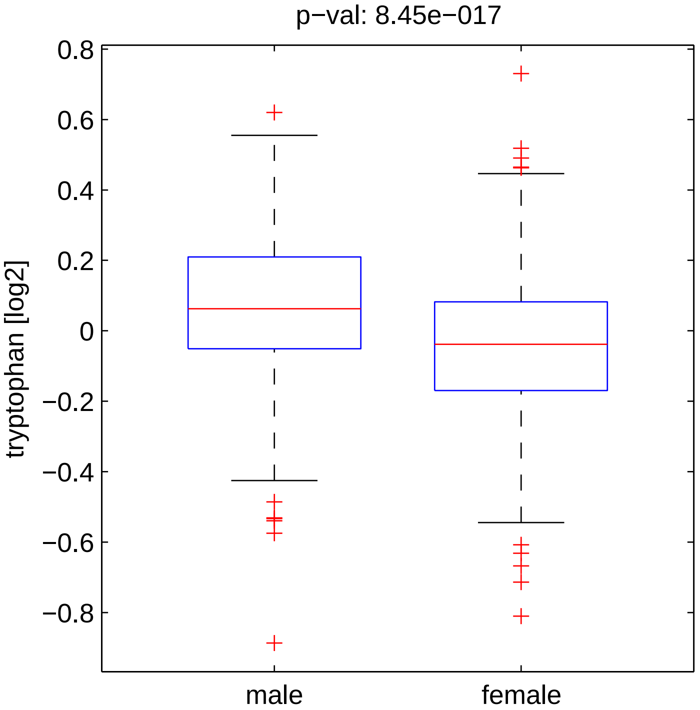
p−val: 8.45e−017
0.8
0.6
0.4
0.2
0
−0.2
−0.4
−0.6
−0.8
tryptophan [log2]
male
female

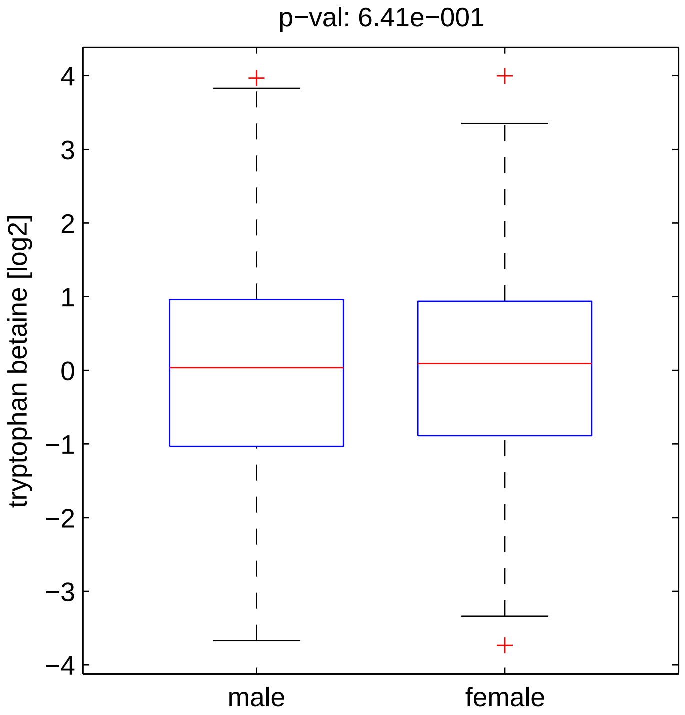

p−val: 6.41e−001
tryptophan betaine [log2]
4
3
2
1
0
−1
−2
−3
−4
male
female

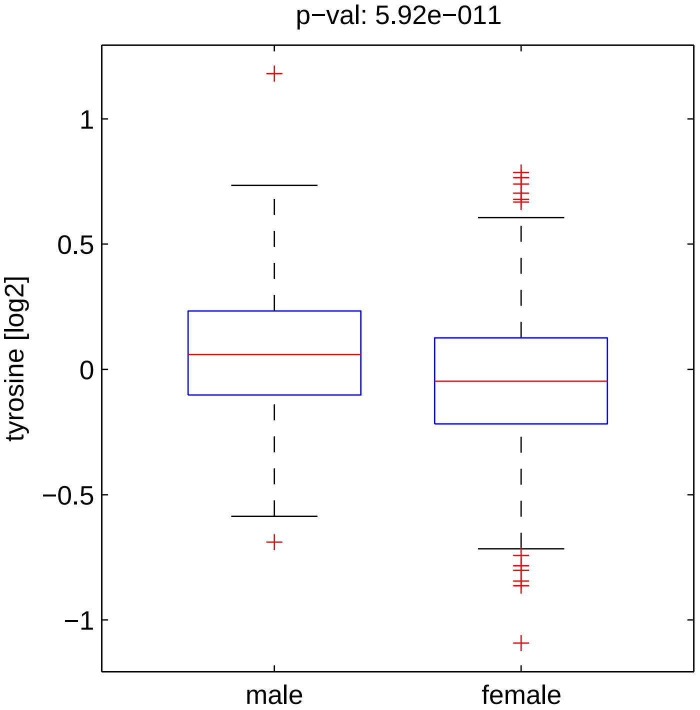
p−val: 5.92e−011
tyrosine [log2]
1
0.5
0
−0.5
−1
male
female

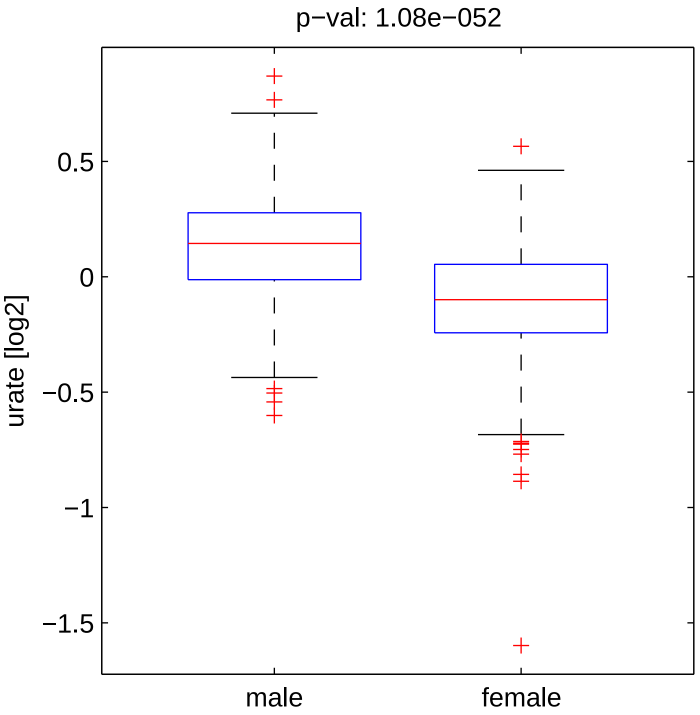
p−val: 1.08e−052
urate [log2]
0.5
0
−0.5
−1
−1.5
male
female

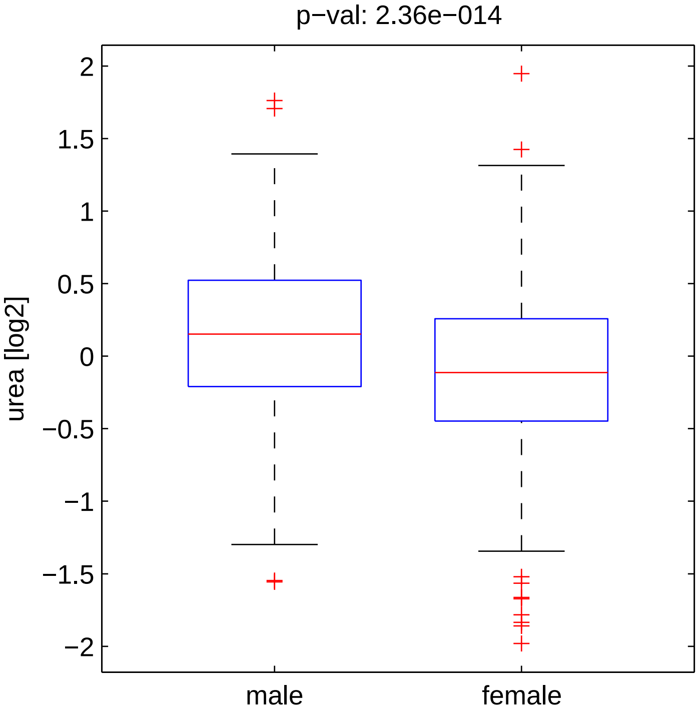
p−val: 2.36e−014
urea [log2]
2
1.5
1
0.5
0
−0.5
−1
−1.5
−2
male
female

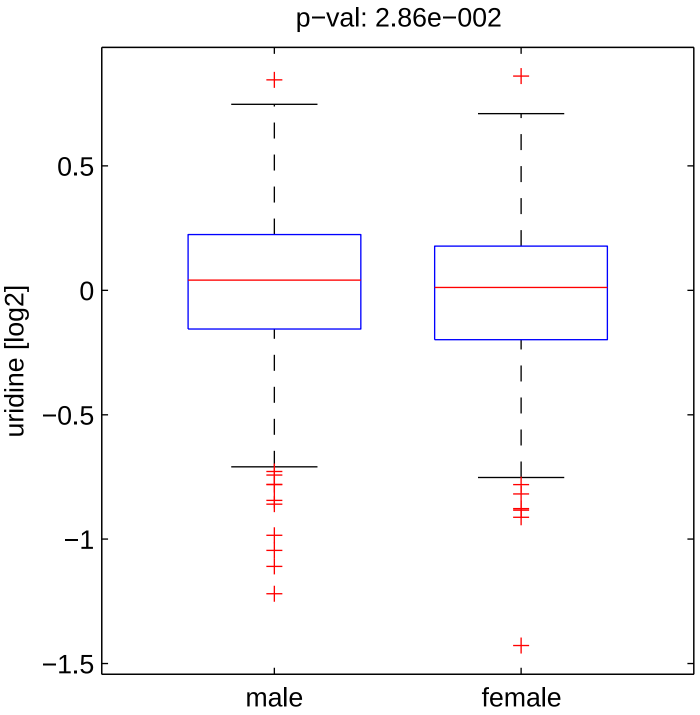

p−val: 2.86e−002
uridine [log2]
0.5
0
−0.5
−1
−1.5
male
female

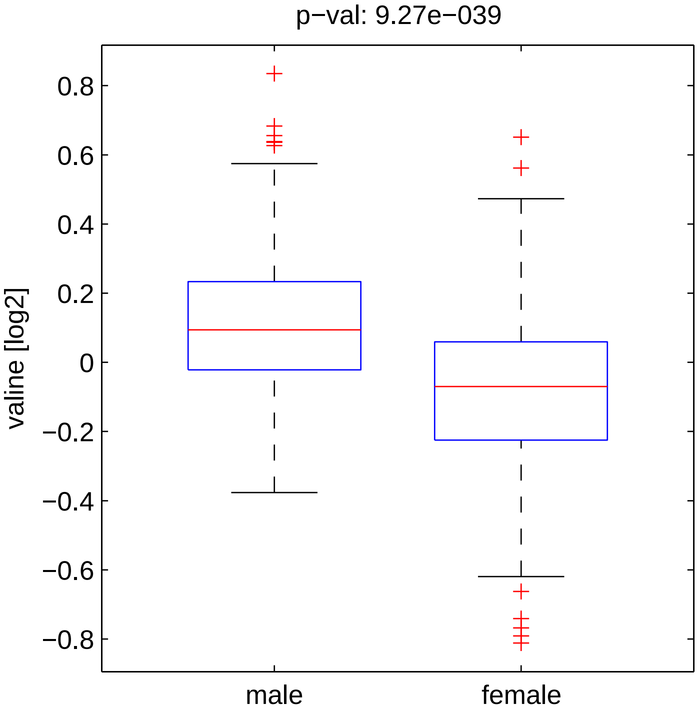

p−val: 9.27e−039
valine [log2]
0.8
0.6
0.4
0.2
0
−0.2
−0.4
−0.6
−0.8
male
female

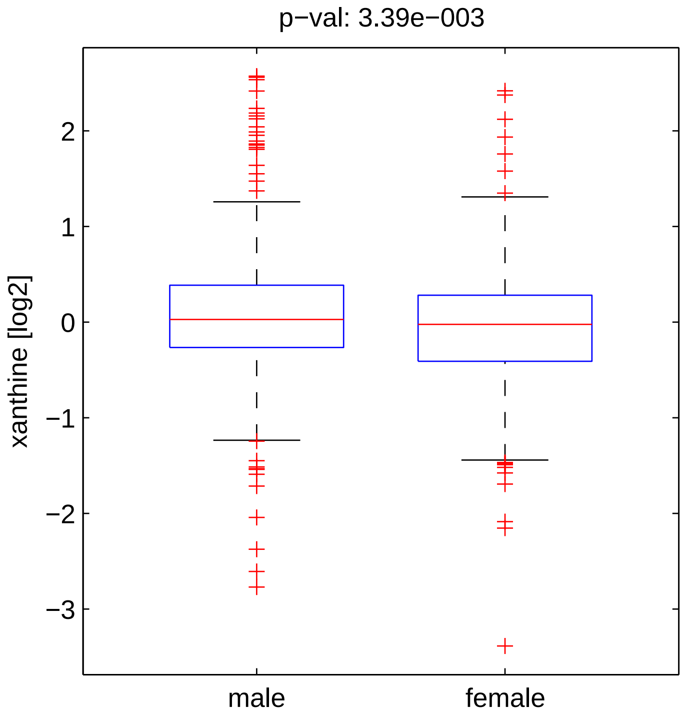
p−val: 3.39e−003
xanthine [log2]
2
1
0
−1
−2
−3
male
female

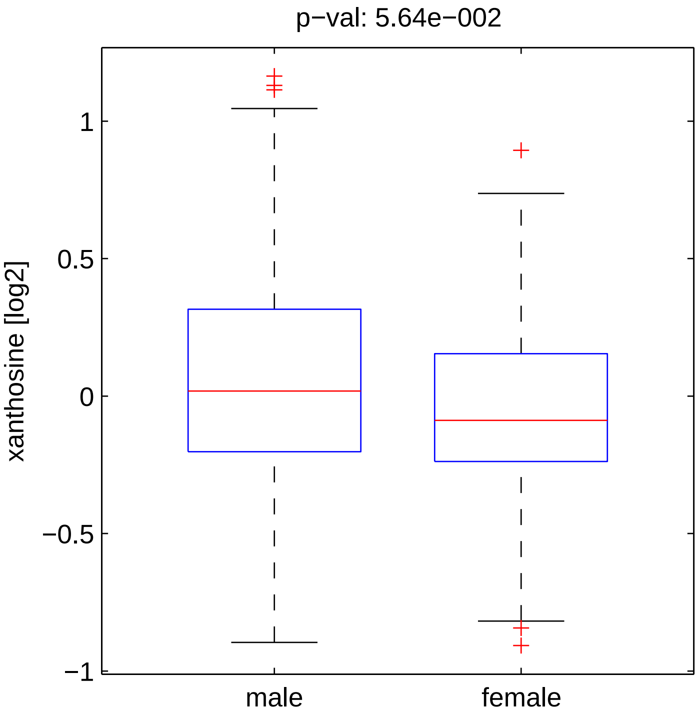
p−val: 5.64e−002
xanthosine [log2]
1
0.5
0
−0.5
−1
male
female

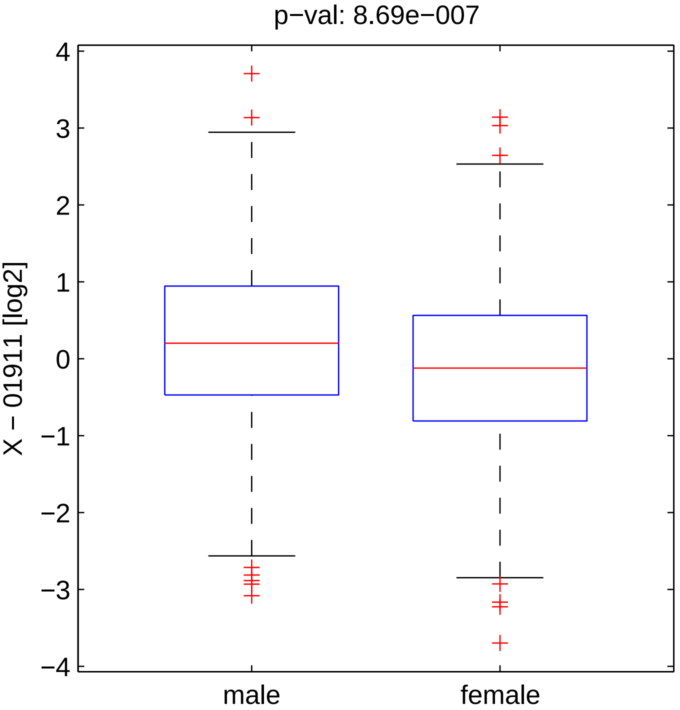

p−val: 8.69e−007
X − 01911 [log2]
4
3
2
1
0
−1
−2
−3
−4
male
female

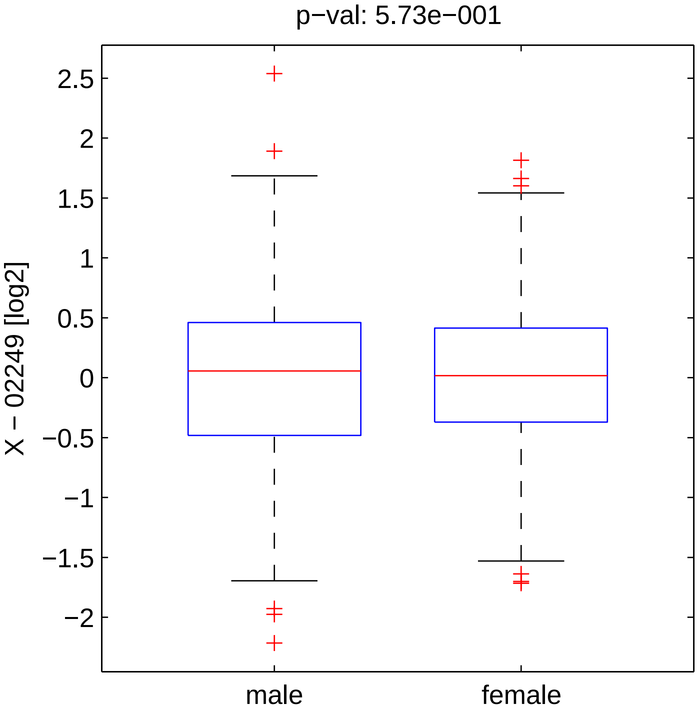
p−val: 5.73e−001
X − 02249 [log2]
2.5
2
1.5
1
0.5
0
−0.5
−1
−1.5
−2
male
female

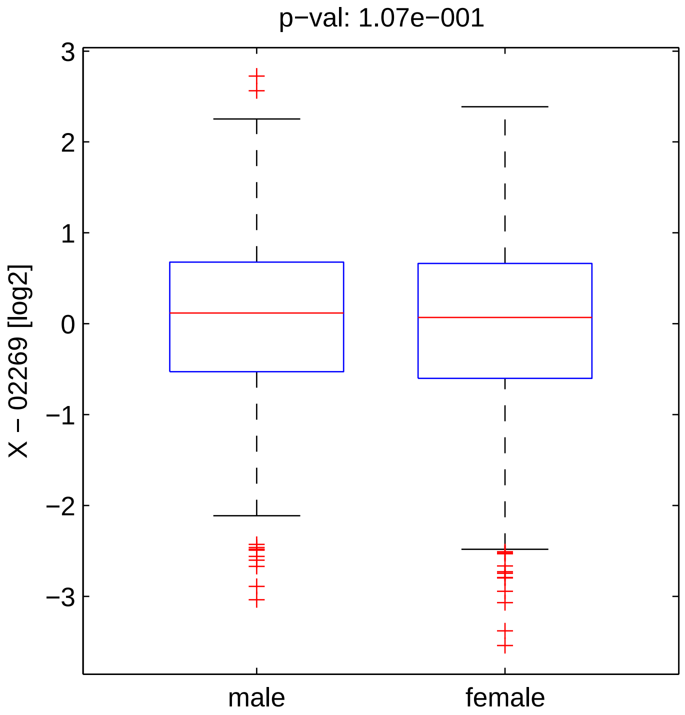
p−val: 1.07e−001
X − 02269 [log2]
3
2
1
0
−1
−2
−3
male
female

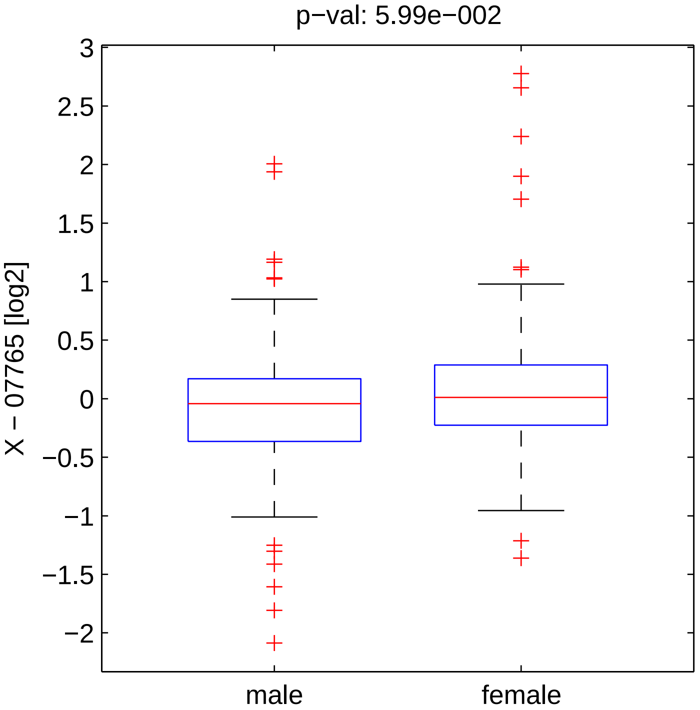
p−val: 5.99e−002
X − 07765 [log2]
3
2.5
2
1.5
1
0.5
0
−0.5
−1
−1.5
−2
male
female

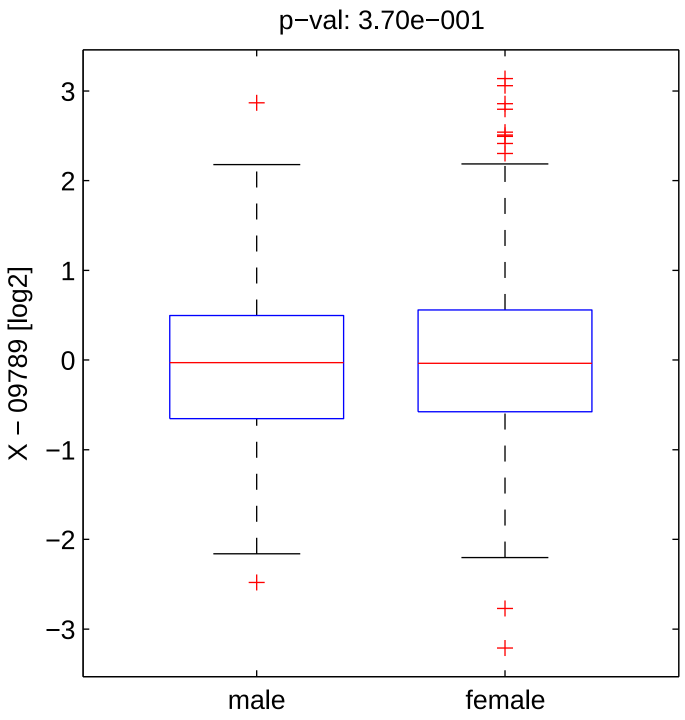
p−val: 3.70e−001
3
2
1
0
−1
−2
−3
X − 09789 [log2]
male
female

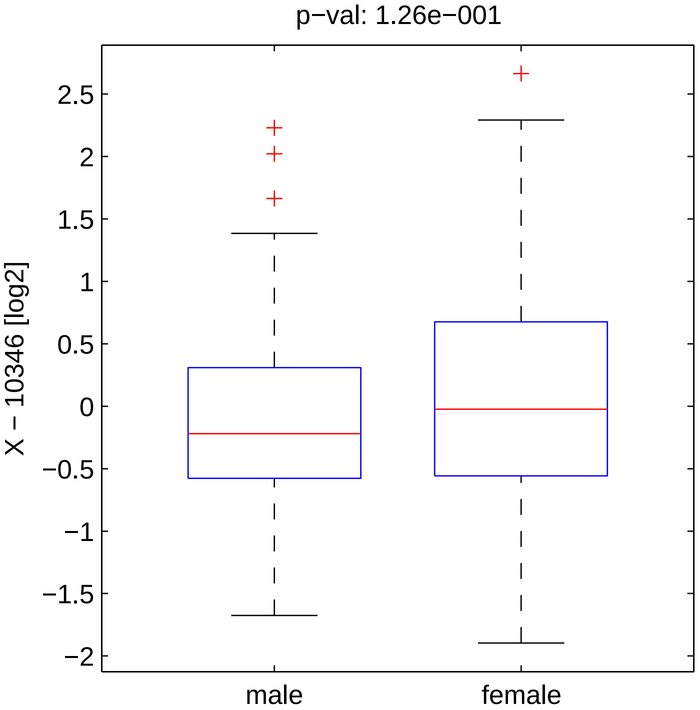
p−val: 1.26e−001
X − 10346 [log2]
2.5
2
1.5
1
0.5
0
−0.5
−1
−1.5
−2
male
female

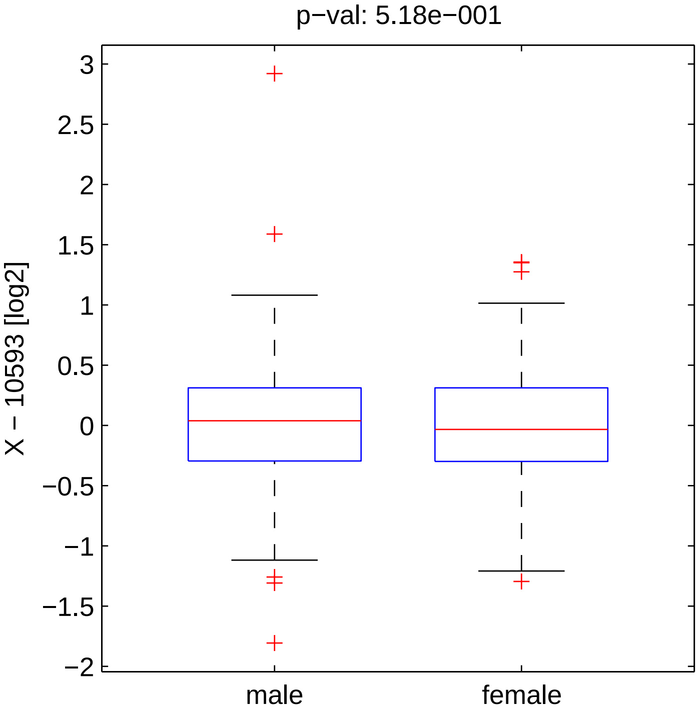
p−val: 5.18e−001
3
2.5
2
1.5
1
0.5
0
−0.5
−1
−1.5
−2
X − 10593 [log2]
male
female

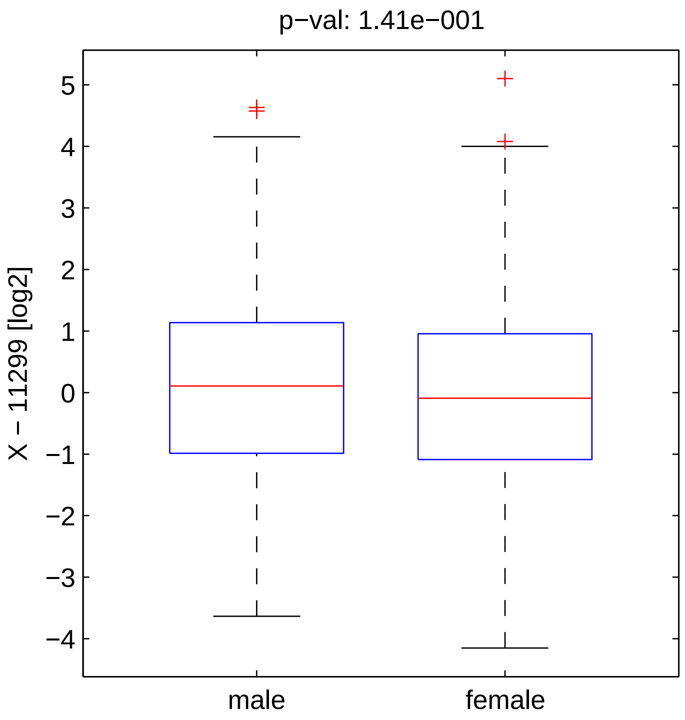

p−val: 1.41e−001
X − 11299 [log2]
5
4
3
2
1
0
−1
−2
−3
−4
male
female

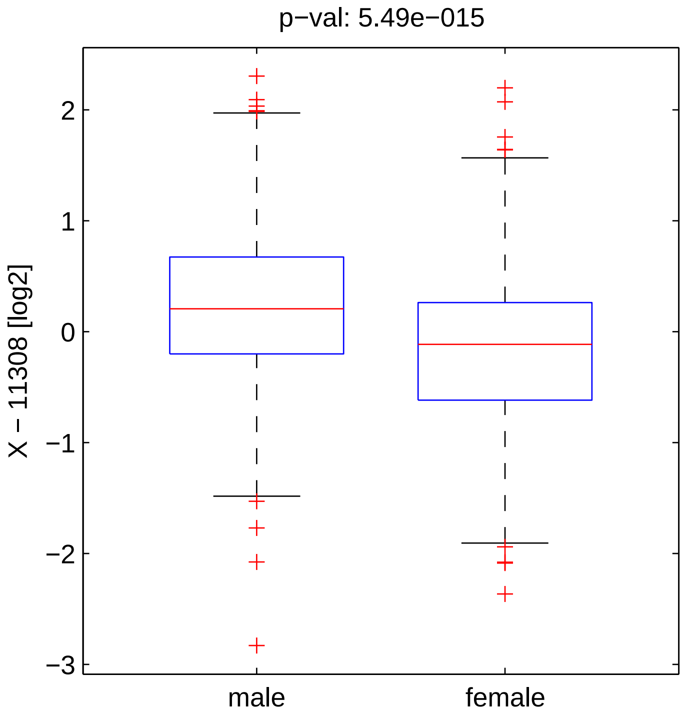

p−val: 5.49e−015
X − 11308 [log2]
2
1
0
−1
−2
−3
male
female

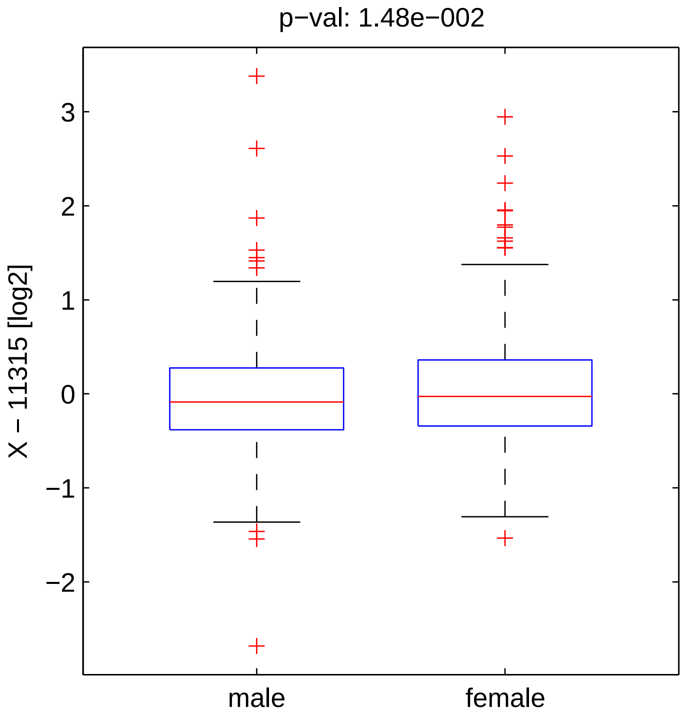

p−val: 1.48e−002
3
2
1
0
−1
−2
X − 11315 [log2]
male
female

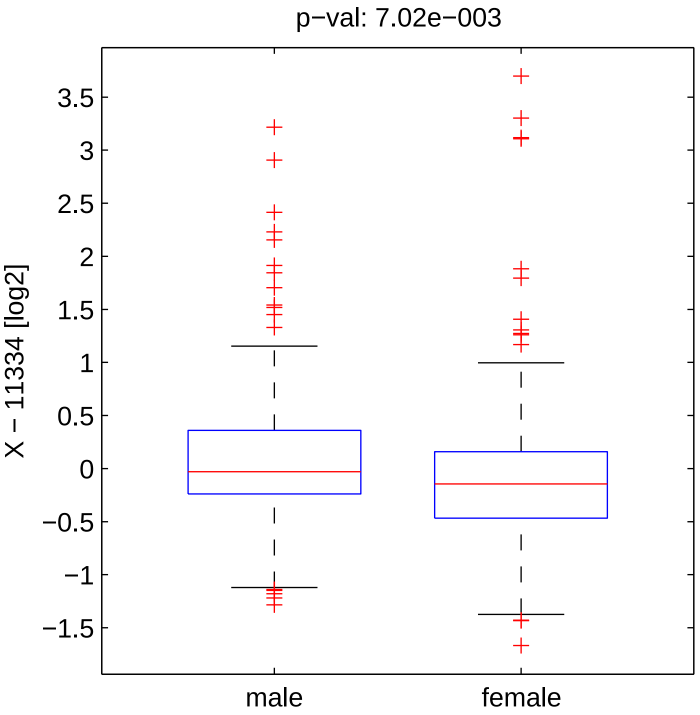

p−val: 7.02e−003
3.5
3
2.5
2
1.5
1
0.5
0
−0.5
−1
−1.5
X − 11334 [log2]
male
female

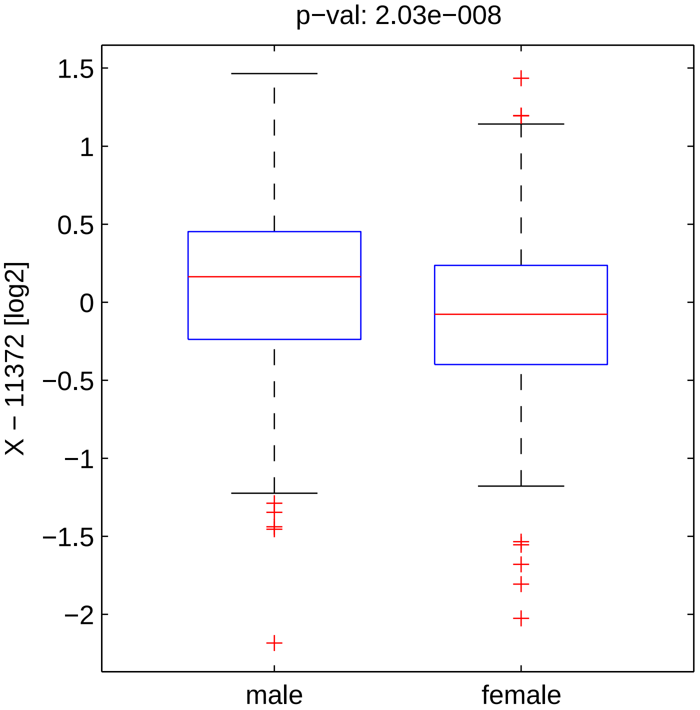
p−val: 2.03e−008
X − 11372 [log2]
1.5
1
0.5
0
−0.5
−1
−1.5
−2
male
female

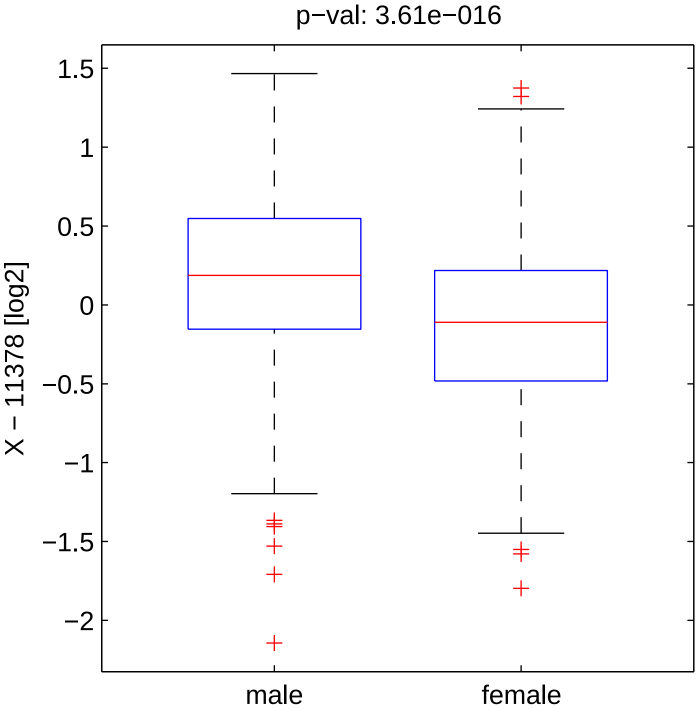

p−val: 3.61e−016
1.5
1
0.5
0
−0.5
−1
−1.5
−2
X − 11378 [log2]
male
female

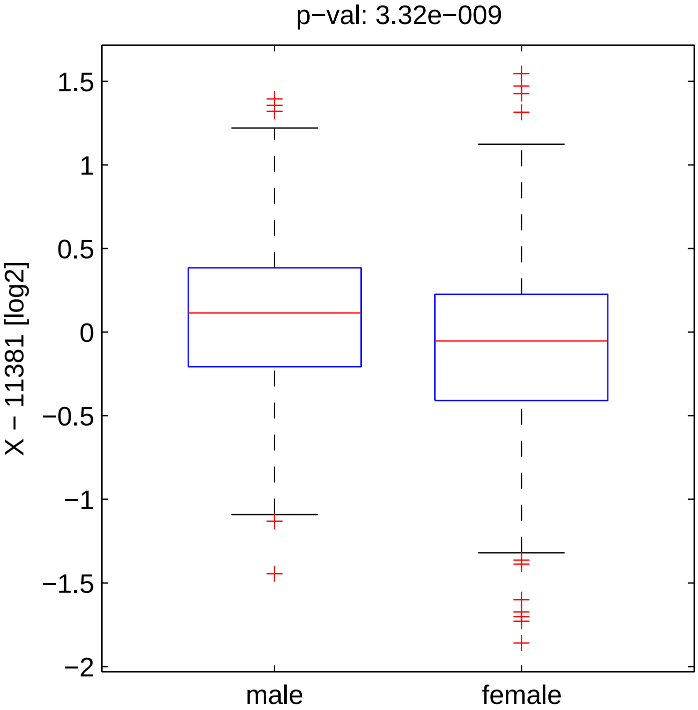
p−val: 3.32e−009
1.5
1
0.5
0
−0.5
−1
−1.5
−2
X − 11381 [log2]
male
female

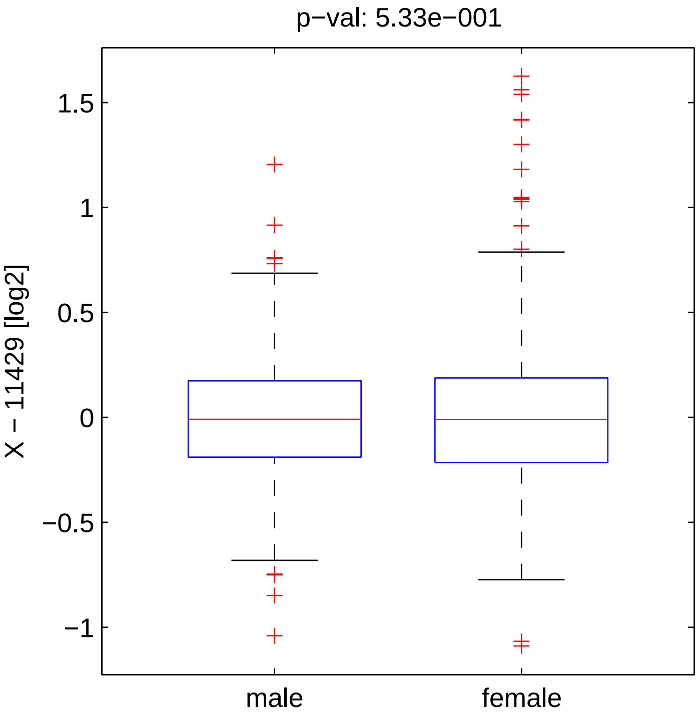

p−val: 5.33e−001
1.5
1
0.5
0
−0.5
−1
X − 11429 [log2]
male
female

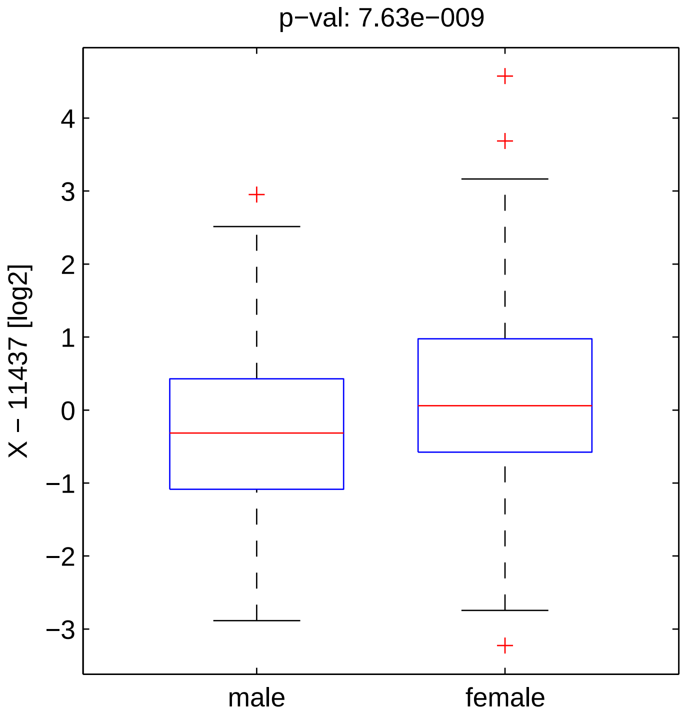

p−val: 7.63e−009
X − 11437 [log2]
4
3
2
1
0
−1
−2
−3
male
female

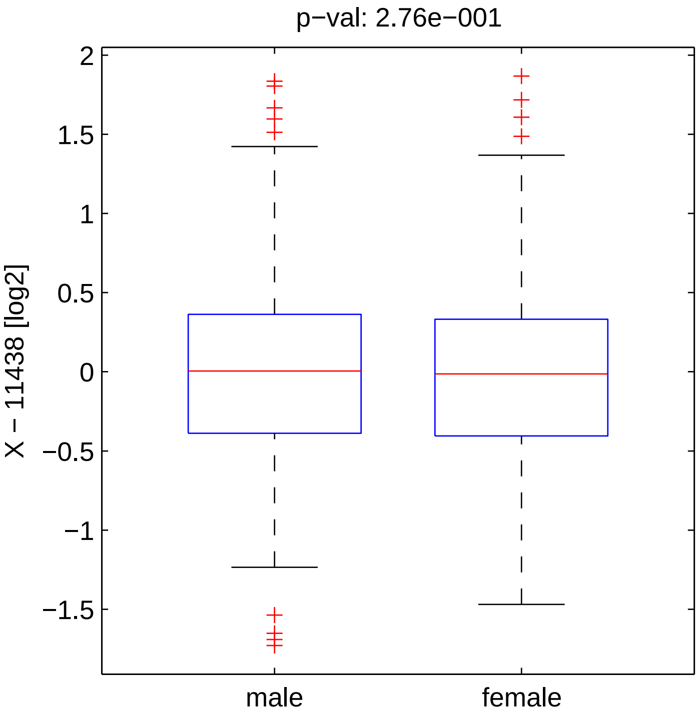
p−val: 2.76e−001
X − 11438 [log2]
2
1.5
1
0.5
0
−0.5
−1
−1.5
male
female

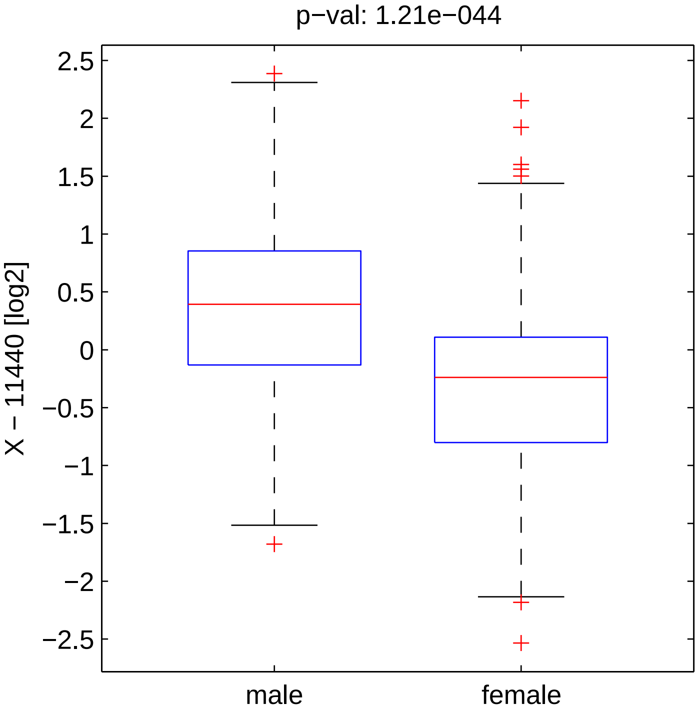

p−val: 1.21e−044
2.5
2
1.5
1
0.5
0
−0.5
−1
−1.5
−2
−2.5
X − 11440 [log2]
male
female

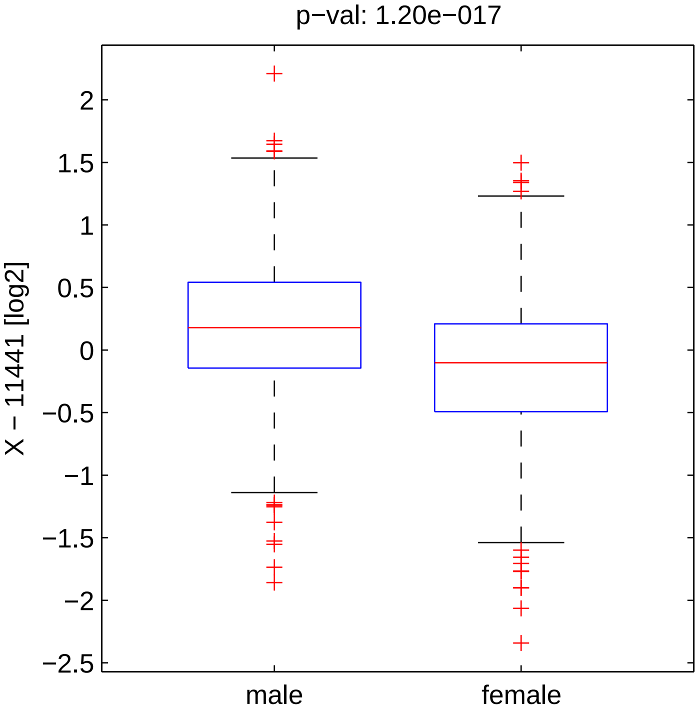

p−val: 1.20e−017
X − 11441 [log2]
2
1.5
1
0.5
0
−0.5
−1
−1.5
−2
−2.5
male
female

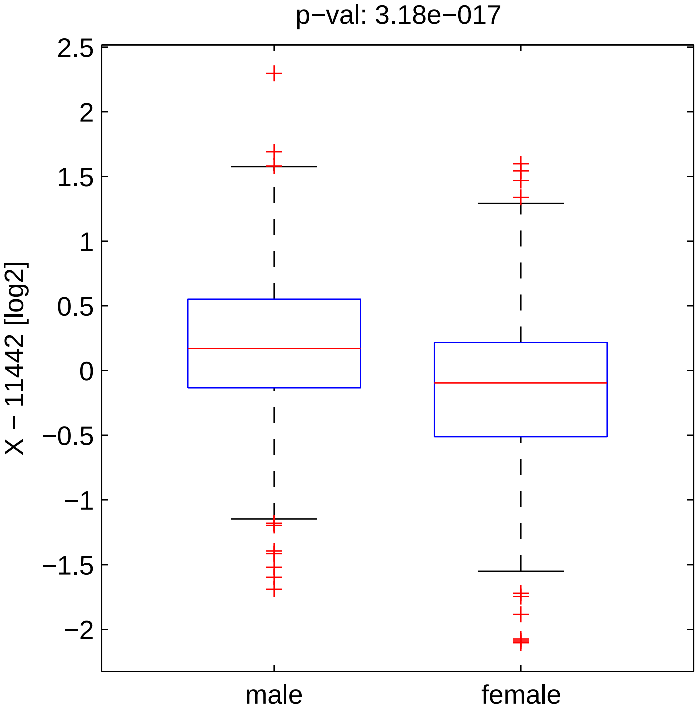

p−val: 3.18e−017
2.5
2
1.5
1
0.5
0
−0.5
−1
−1.5
−2
X − 11442 [log2]
male
female

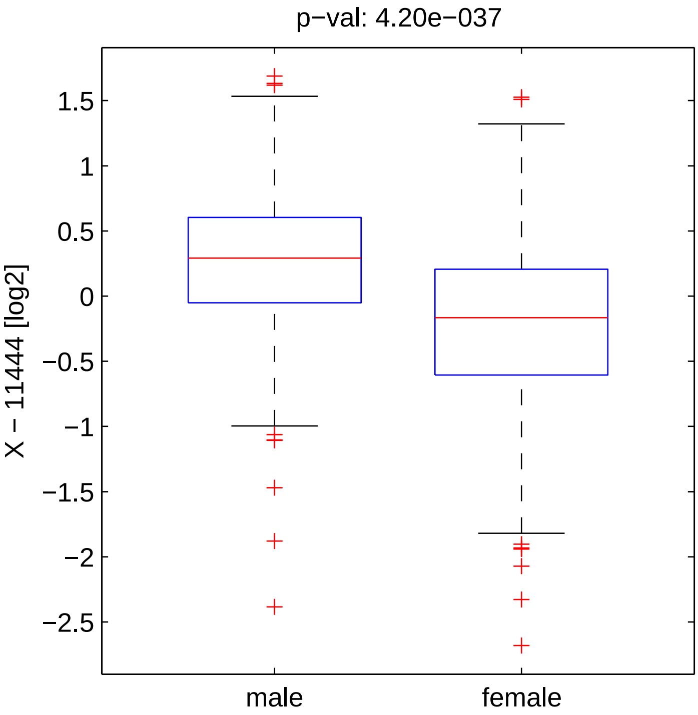

p−val: 4.20e−037
1.5
1
0.5
0
−0.5
−1
−1.5
−2
−2.5
X − 11444 [log2]
male
female

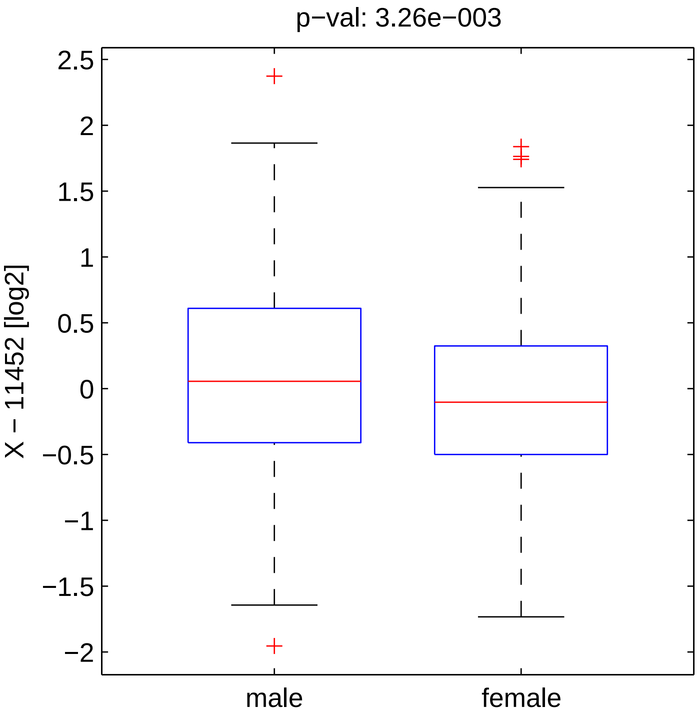
p−val: 3.26e−003
X − 11452 [log2]
2.5
2
1.5
1
0.5
0
−0.5
−1
−1.5
−2
male
female

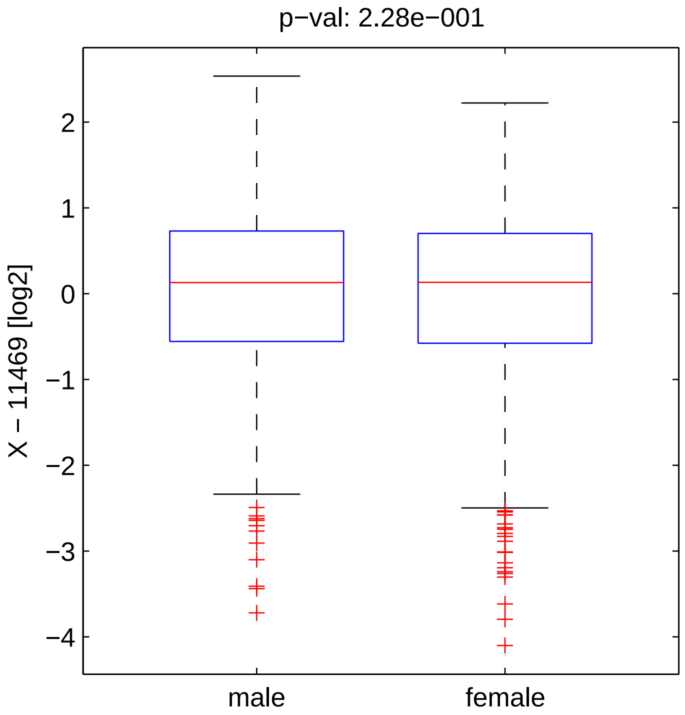

p−val: 2.28e−001
X − 11469 [log2]
2
1
0
−1
−2
−3
−4
male
female

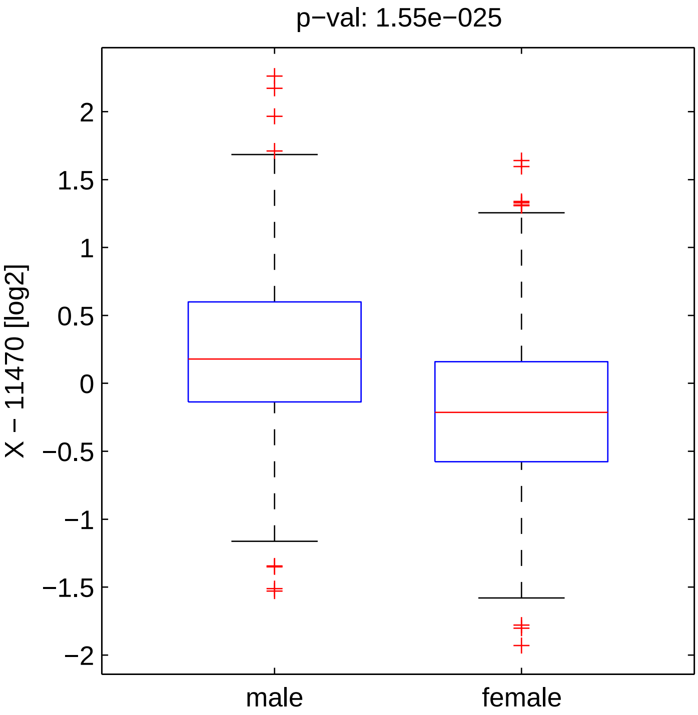

p−val: 1.55e−025
X − 11470 [log2]
2
1.5
1
0.5
0
−0.5
−1
−1.5
−2
male
female

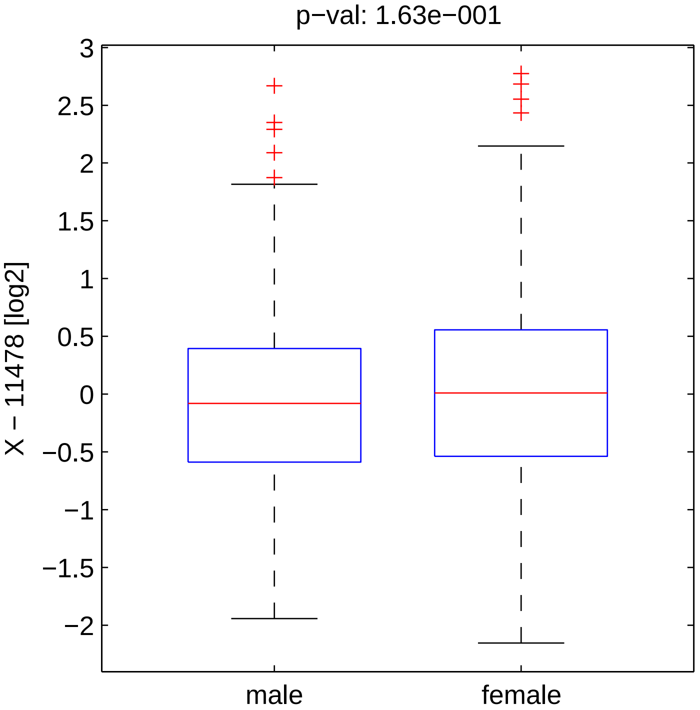
p−val: 1.63e−001
X − 11478 [log2]
3
2.5
2
1.5
1
0.5
0
−0.5
−1
−1.5
−2
male
female

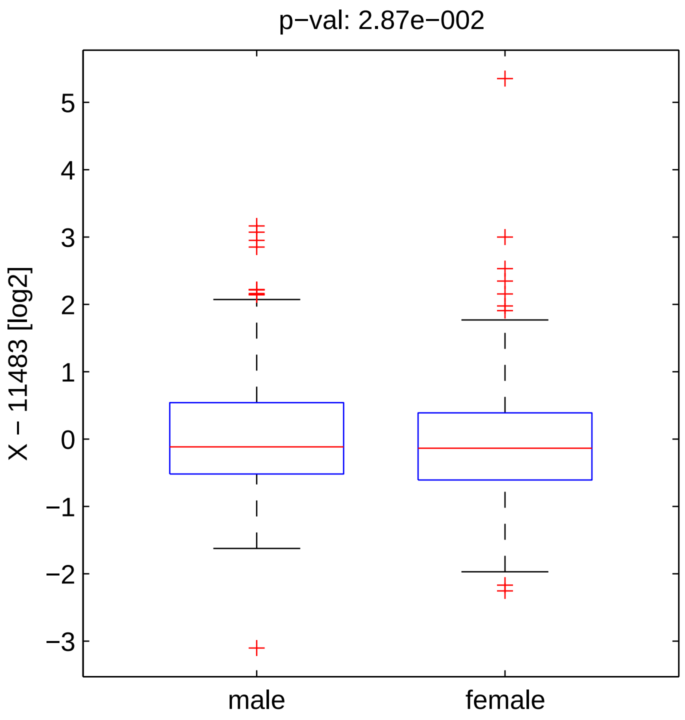
p−val: 2.87e−002
X − 11483 [log2]
5
4
3
2
1
0
−1
−2
−3
male
female

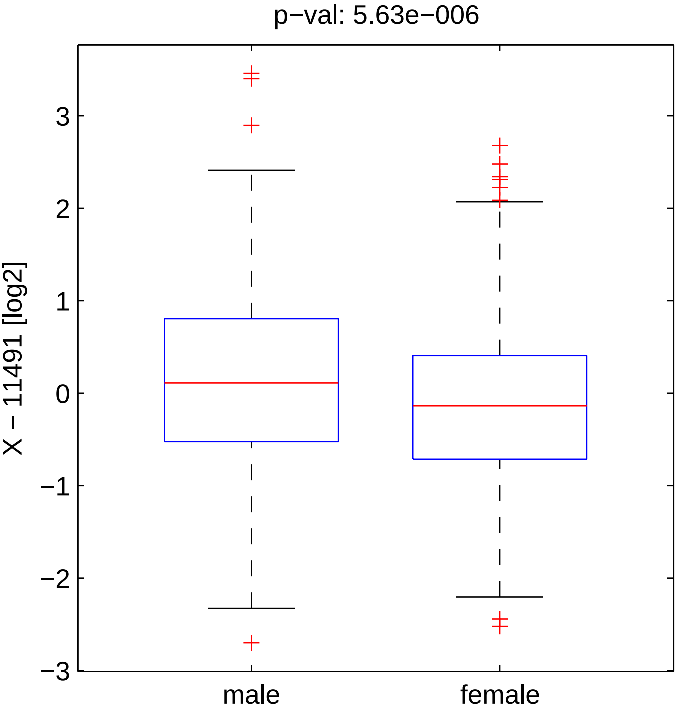

p−val: 5.63e−006
X − 11491 [log2]
3
2
1
0
−1
−2
−3
male
female

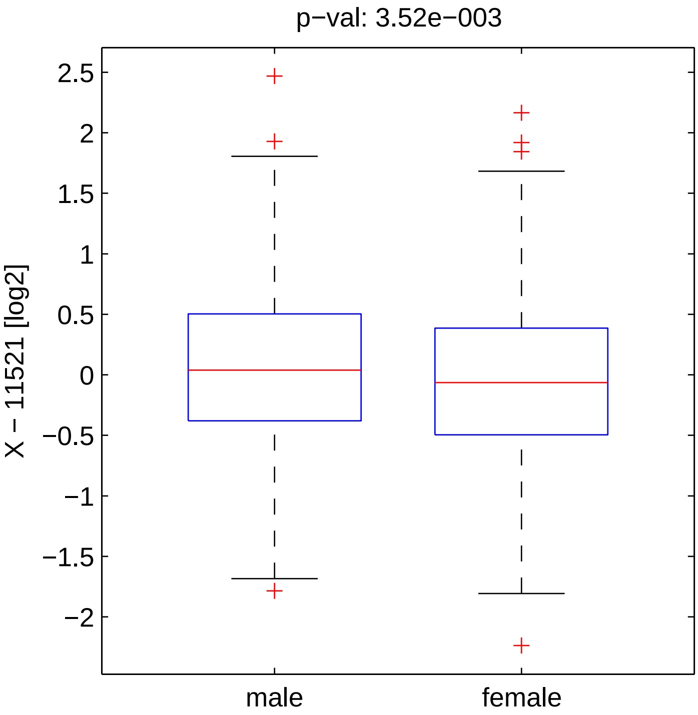
p−val: 3.52e−003
2.5
2
1.5
1
0.5
0
−0.5
−1
−1.5
−2
X − 11521 [log2]
male
female

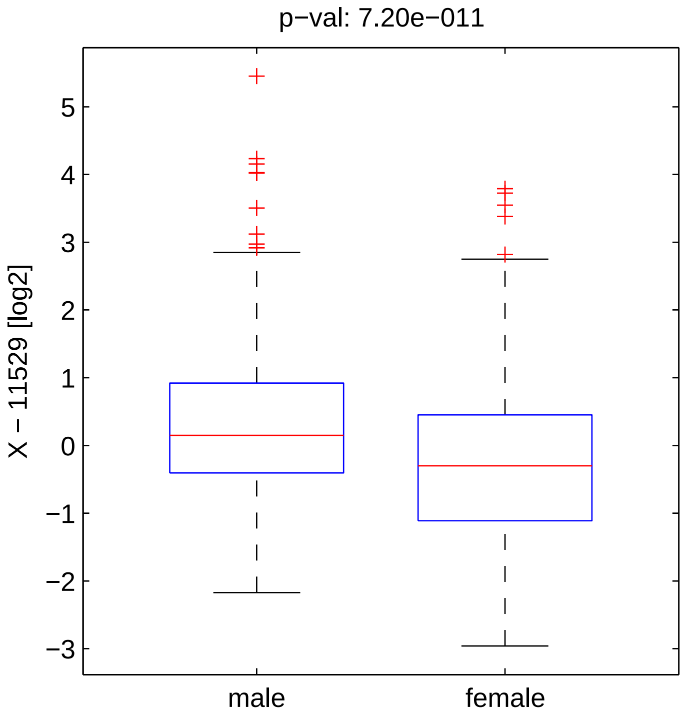
p−val: 7.20e−011
X − 11529 [log2]
5
4
3
2
1
0
−1
−2
−3
male
female

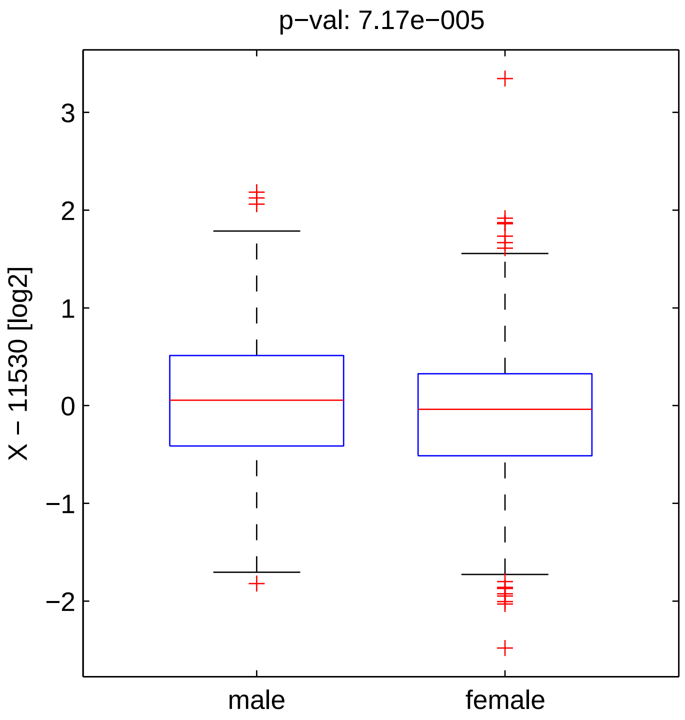
p−val: 7.17e−005
X − 11530 [log2]
3
2
1
0
−1
−2
male
female

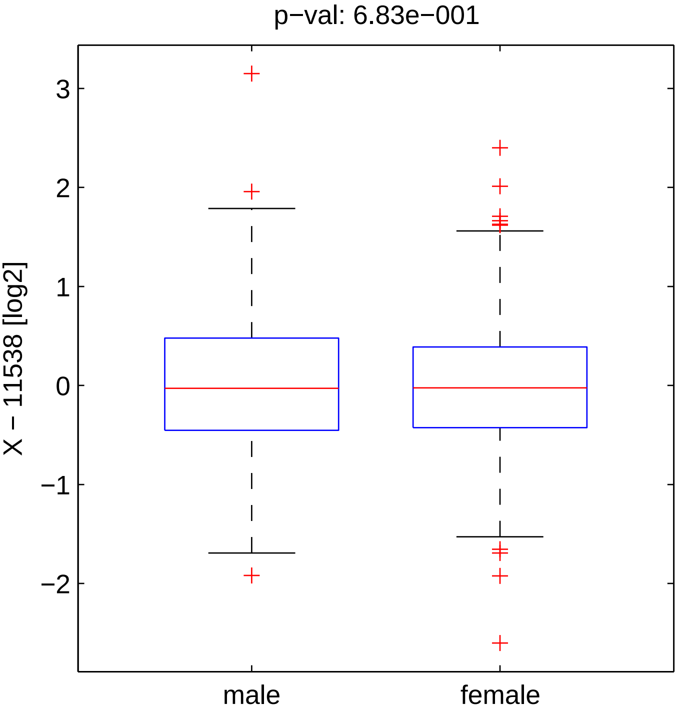
p−val: 6.83e−001
X − 11538 [log2]
3
2
1
0
−1
−2
male
female

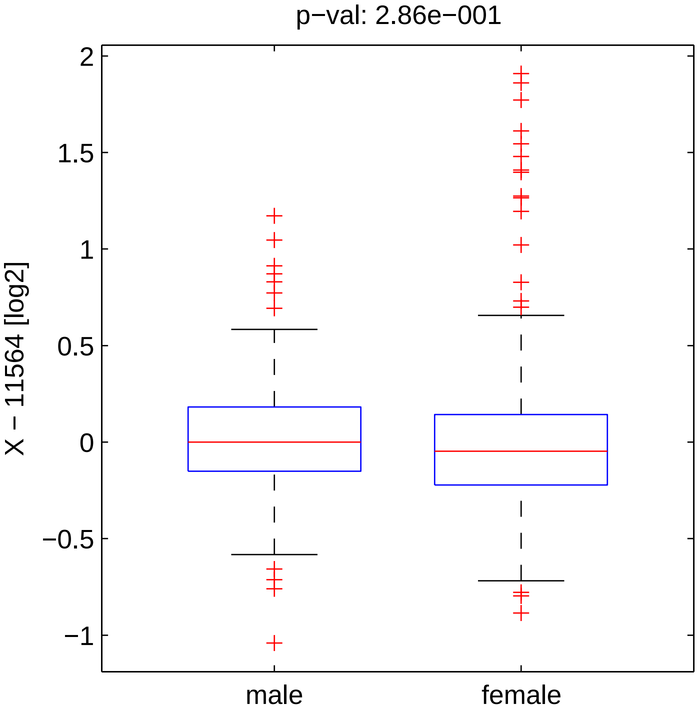

p−val: 2.86e−001
X − 11564 [log2]
2
1.5
1
0.5
0
−0.5
−1
male
female

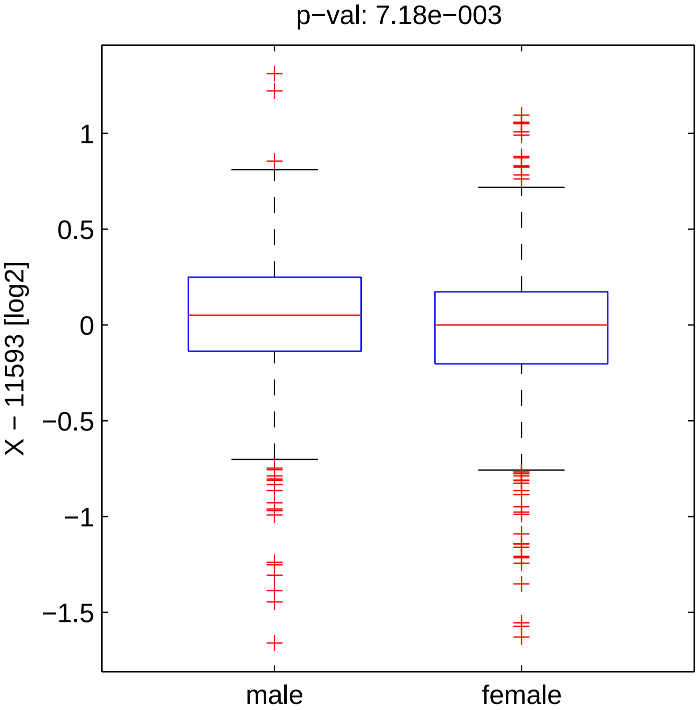

p−val: 7.18e−003
X − 11593 [log2]
1
0.5
0
−0.5
−1
−1.5
male
female

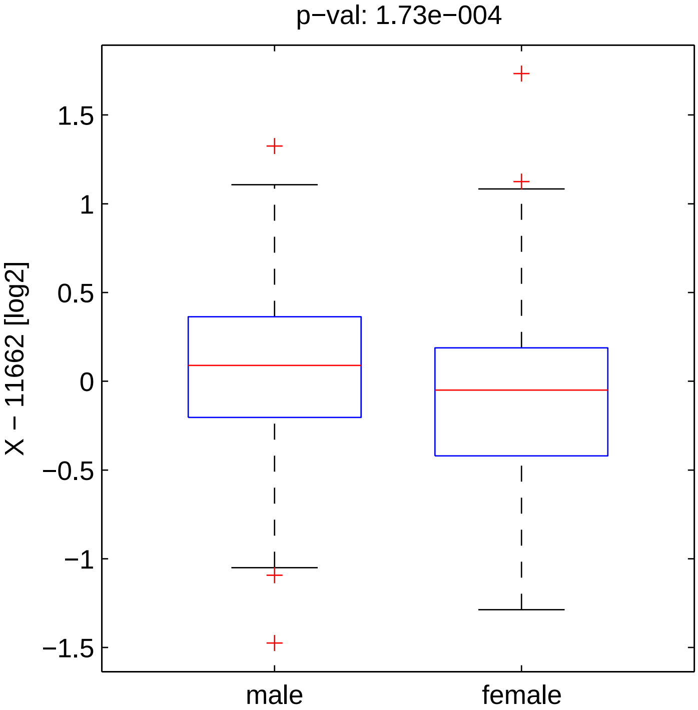
p−val: 1.73e−004
1.5
1
0.5
0
−0.5
−1
−1.5
X − 11662 [log2]
male
female

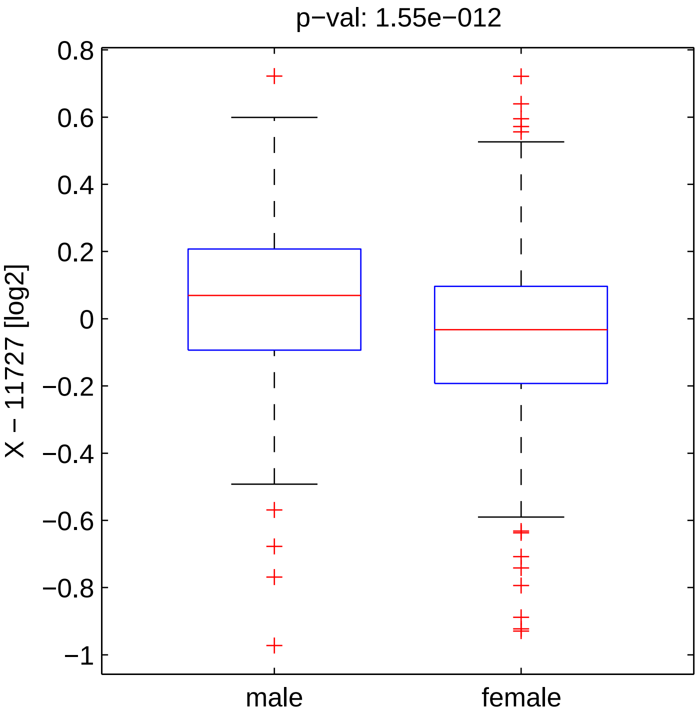

p−val: 1.55e−012
0.8
0.6
0.4
0.2
0
−0.2
−0.4
−0.6
−0.8
−1
X − 11727 [log2]
male
female

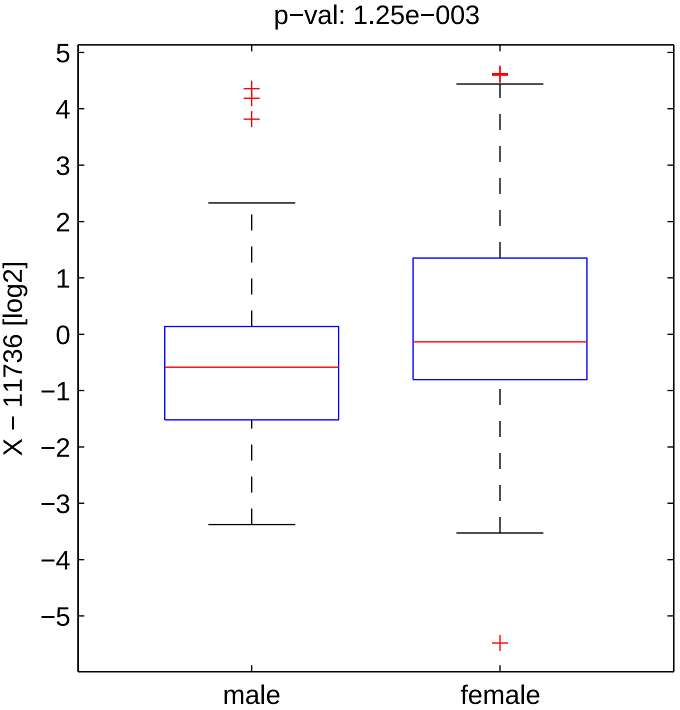
p−val: 1.25e−003
X − 11736 [log2]
5
4
3
2
1
0
−1
−2
−3
−4
−5
male
female

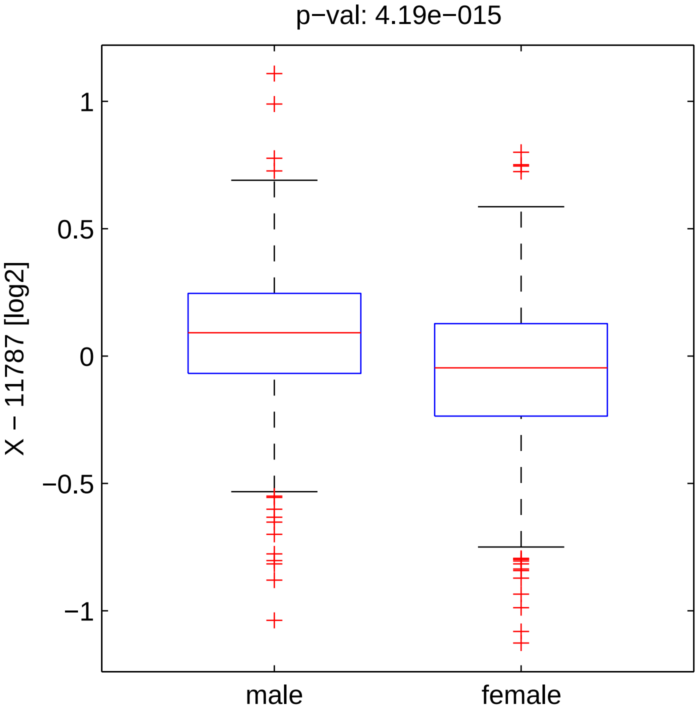

p−val: 4.19e−015
X − 11787 [log2]
1
0.5
0
−0.5
−1
male
female

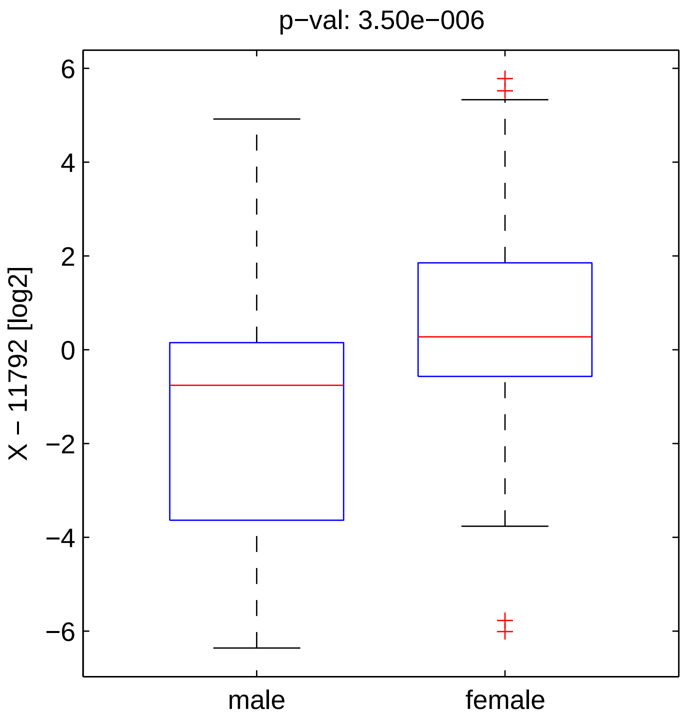

p−val: 3.50e−006
6
4
2
0
−2
−4
−6
X − 11792 [log2]
male
female

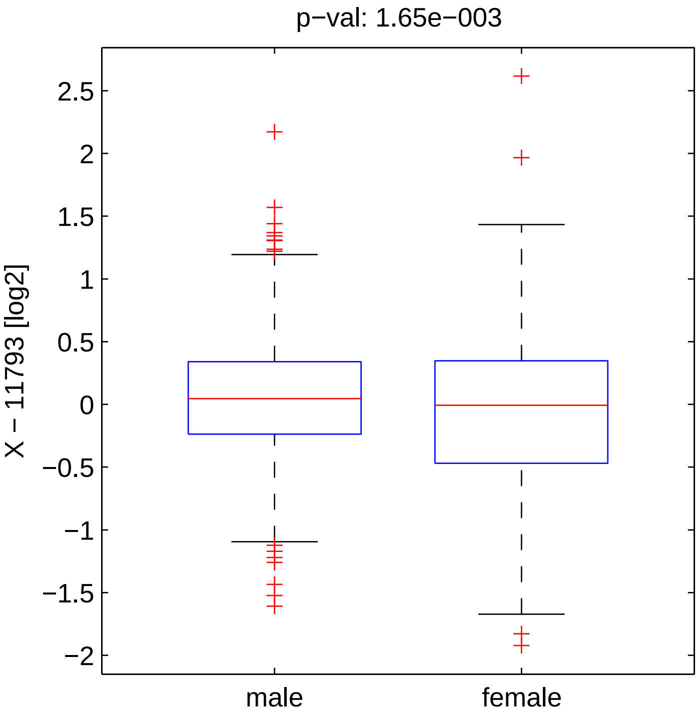
p−val: 1.65e−003
X − 11793 [log2]
2.5
2
1.5
1
0.5
0
−0.5
−1
−1.5
−2
male
female

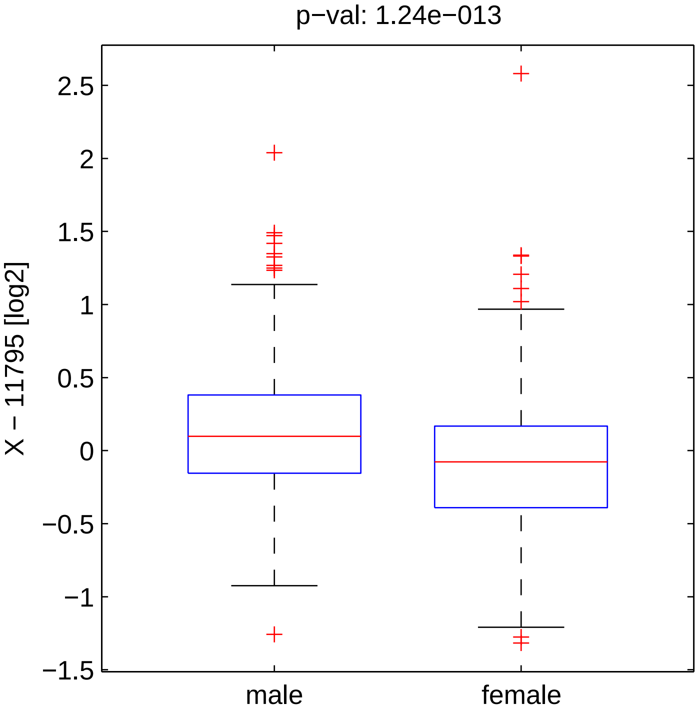
p−val: 1.24e−013
X − 11795 [log2]
2.5
2
1.5
1
0.5
0
−0.5
−1
−1.5
male
female

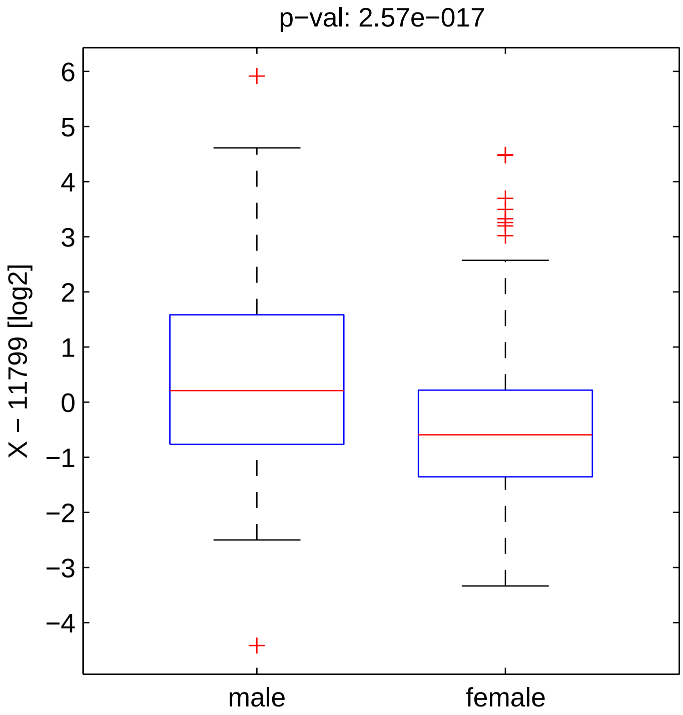
p−val: 2.57e−017
X − 11799 [log2]
6
5
4
3
2
1
0
−1
−2
−3
−4
male
female

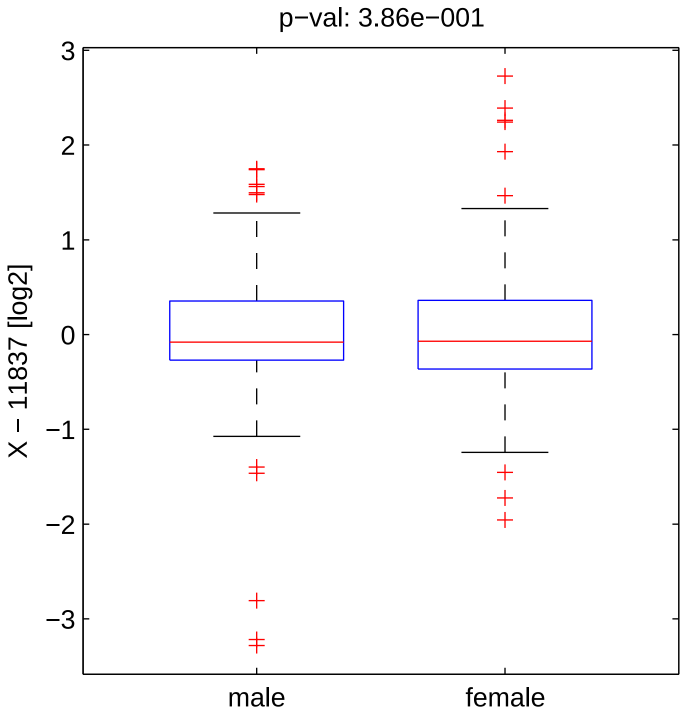

p−val: 3.86e−001
X − 11837 [log2]
3
2
1
0
−1
−2
−3
male
female

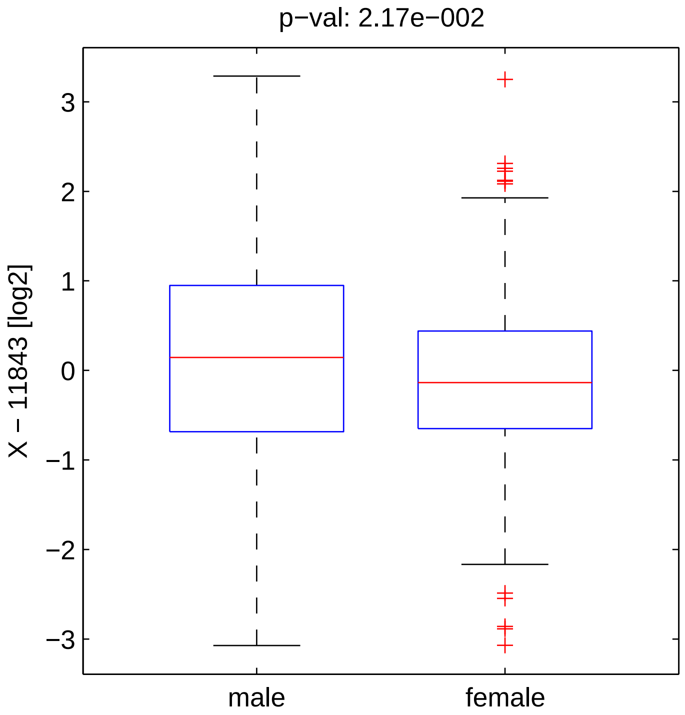

p−val: 2.17e−002
X − 11843 [log2]
3
2
1
0
−1
−2
−3
male
female

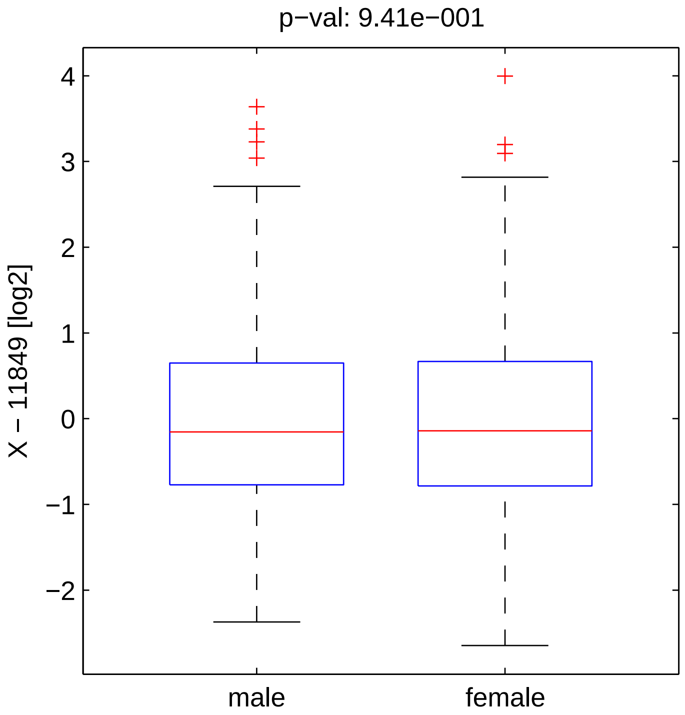

p−val: 9.41e−001
X − 11849 [log2]
4
3
2
1
0
−1
−2
male
female

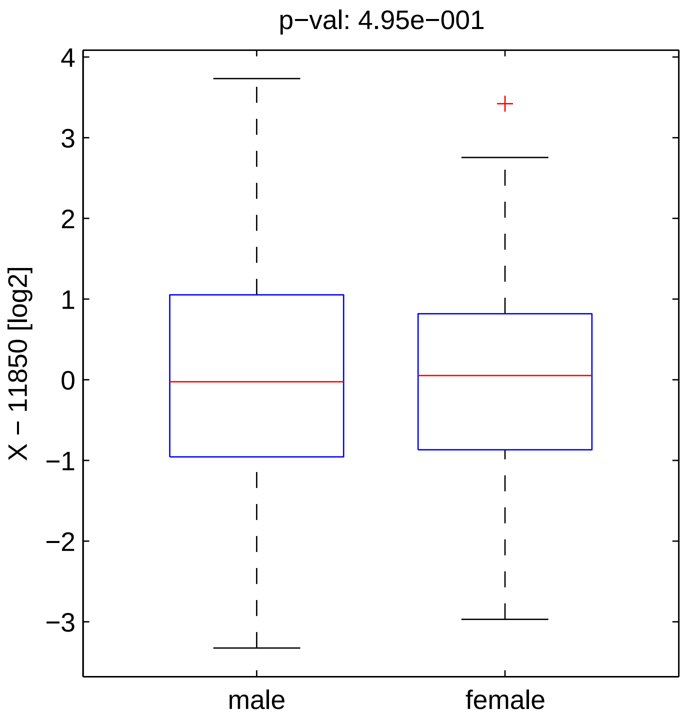

p−val: 4.95e−001
4
3
2
1
0
−1
−2
−3
X − 11850 [log2]
male
female

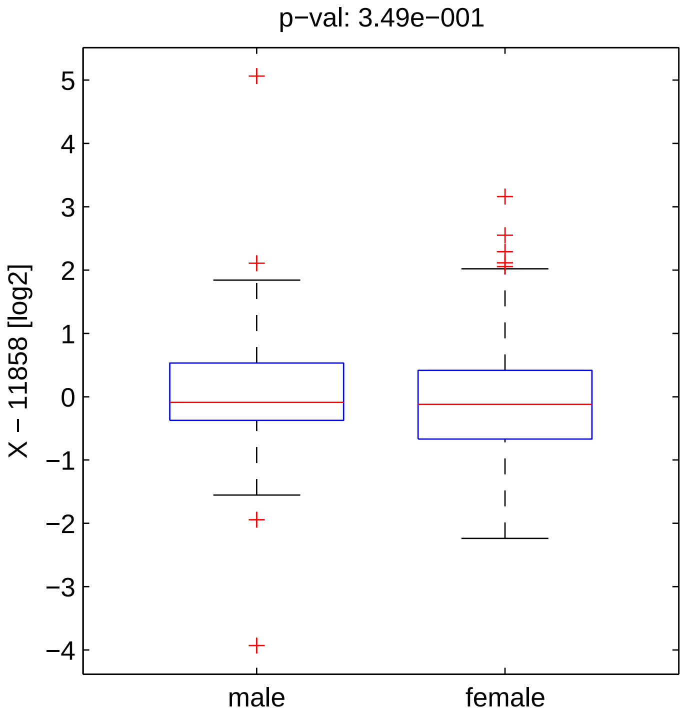

p−val: 3.49e−001
X − 11858 [log2]
5
4
3
2
1
0
−1
−2
−3
−4
male
female

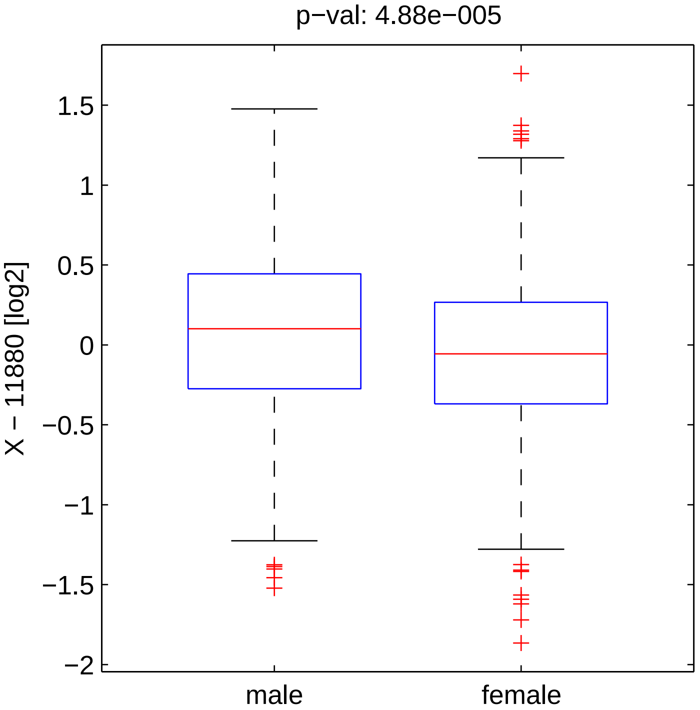
p−val: 4.88e−005
1.5
1
0.5
0
−0.5
−1
−1.5
−2
X − 11880 [log2]
male
female

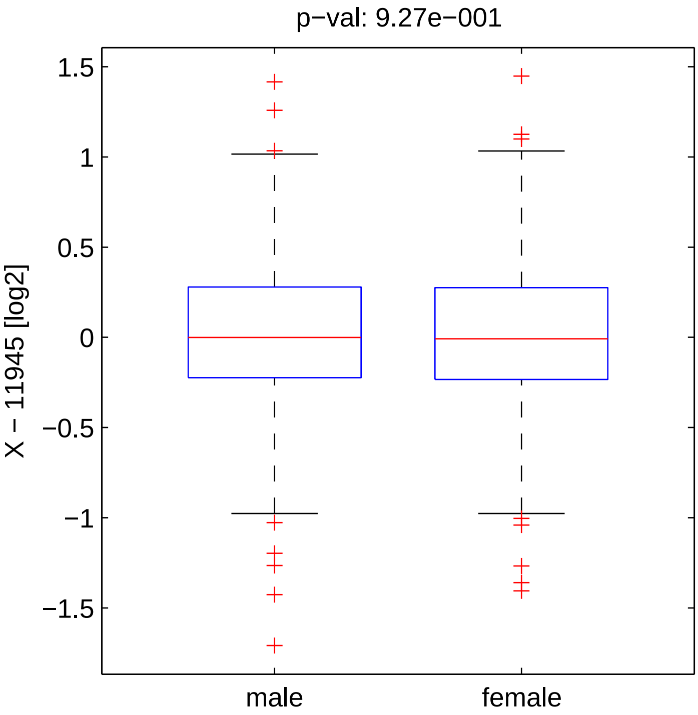

p−val: 9.27e−001
1.5
1
0.5
0
−0.5
−1
−1.5
X − 11945 [log2]
male
female

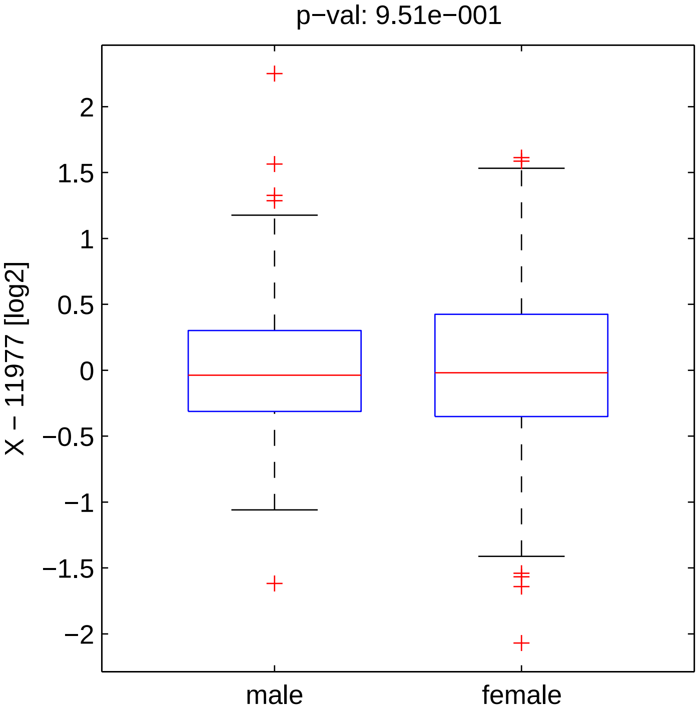

p−val: 9.51e−001
X − 11977 [log2]
2
1.5
1
0.5
0
−0.5
−1
−1.5
−2
male
female

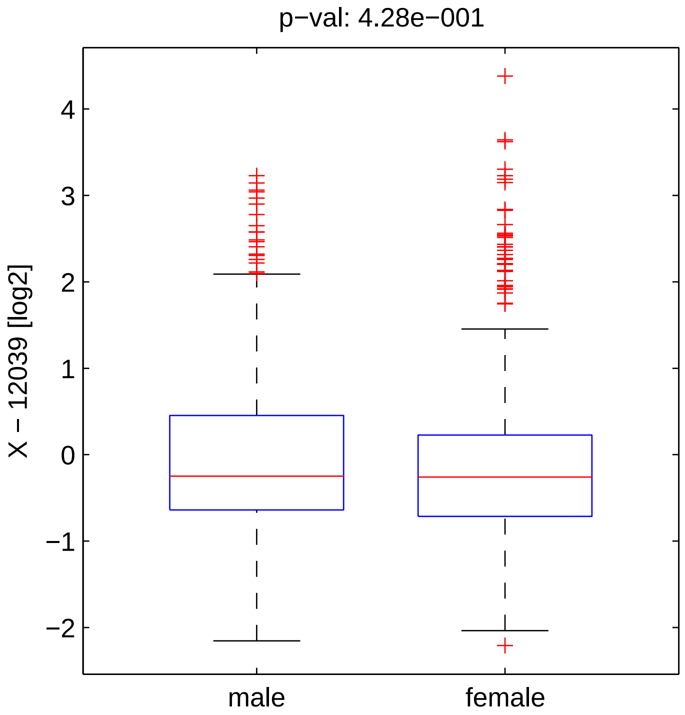
p−val: 4.28e−001
X − 12039 [log2]
4
3
2
1
0
−1
−2
male
female

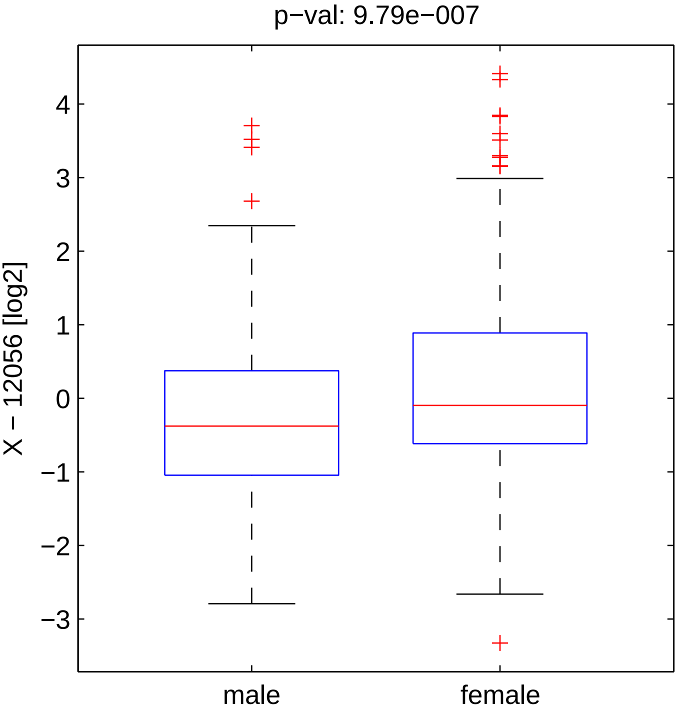

p−val: 9.79e−007
X − 12056 [log2]
4
3
2
1
0
−1
−2
−3
male
female

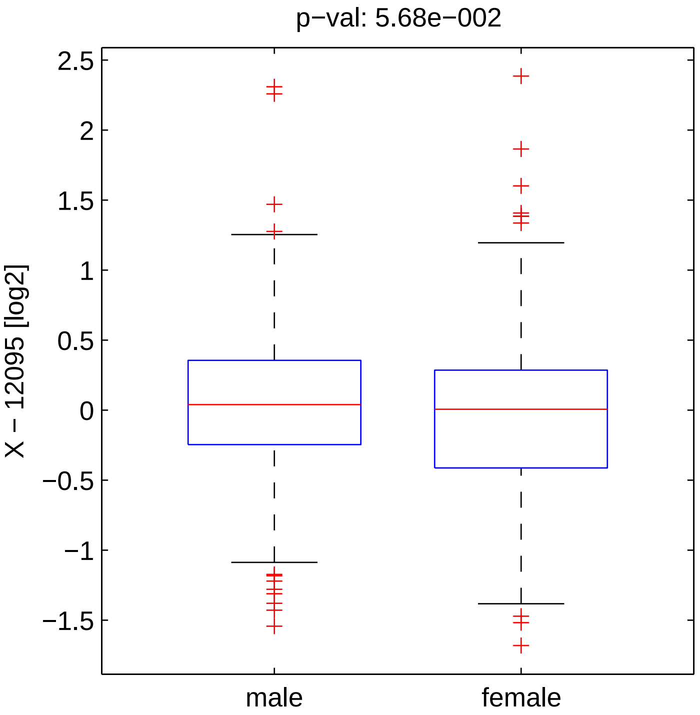
p−val: 5.68e−002
X − 12095 [log2]
2.5
2
1.5
1
0.5
0
−0.5
−1
−1.5
male
female

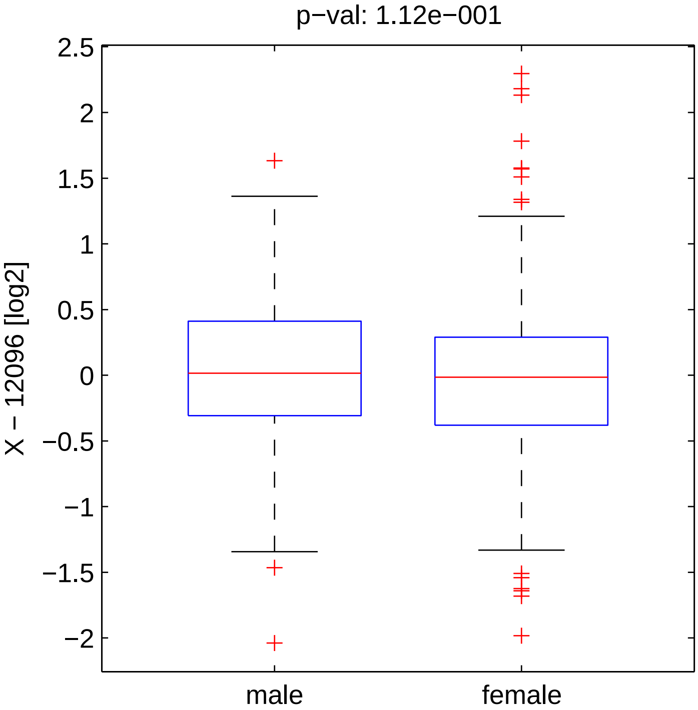
p−val: 1.12e−001
X − 12096 [log2]
2.5
2
1.5
1
0.5
0
−0.5
−1
−1.5
−2
male
female

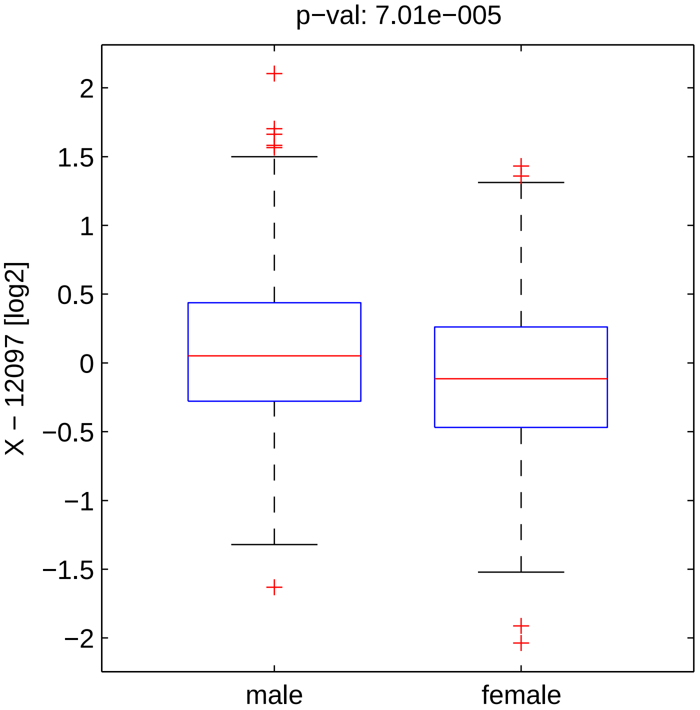
p−val: 7.01e−005
X − 12097 [log2]
2
1.5
1
0.5
0
−0.5
−1
−1.5
−2
male
female

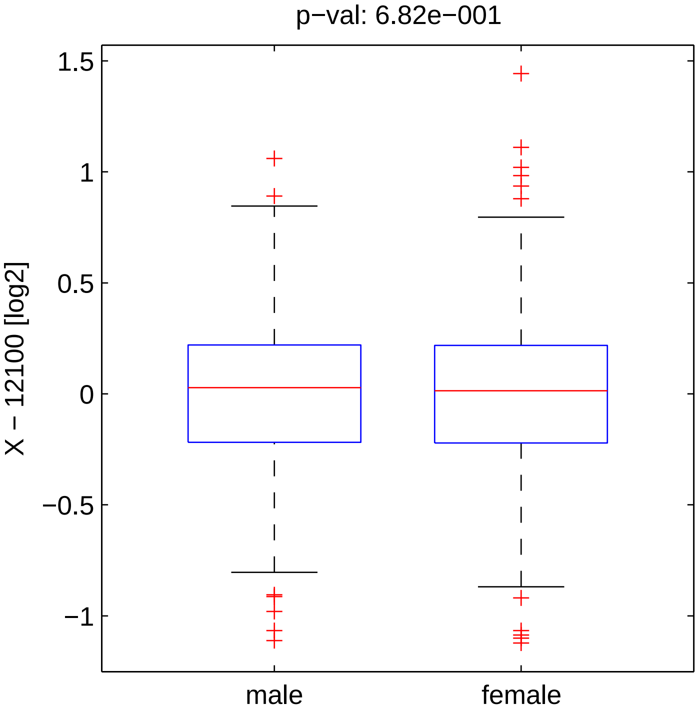
p−val: 6.82e−001
1.5
1
0.5
0
−0.5
−1
X − 12100 [log2]
male
female

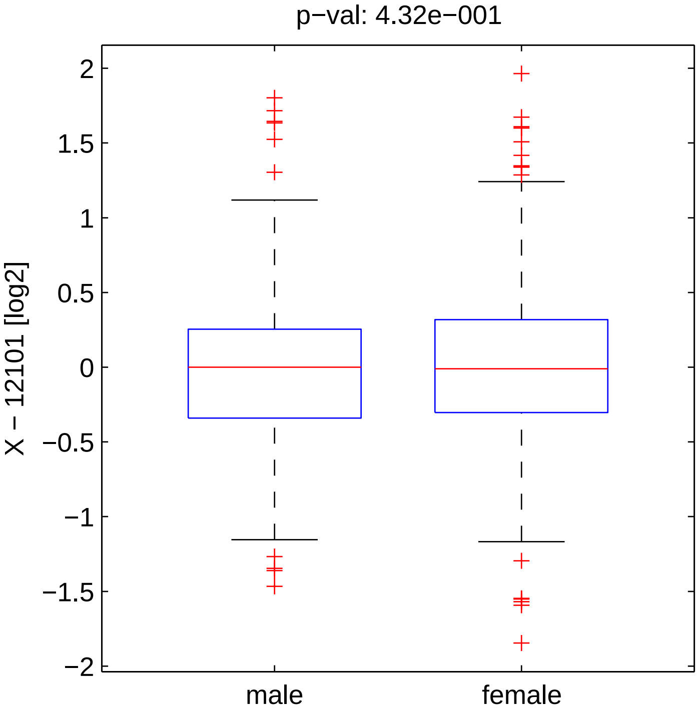

p−val: 4.32e−001
X − 12101 [log2]
2
1.5
1
0.5
0
−0.5
−1
−1.5
−2
male
female

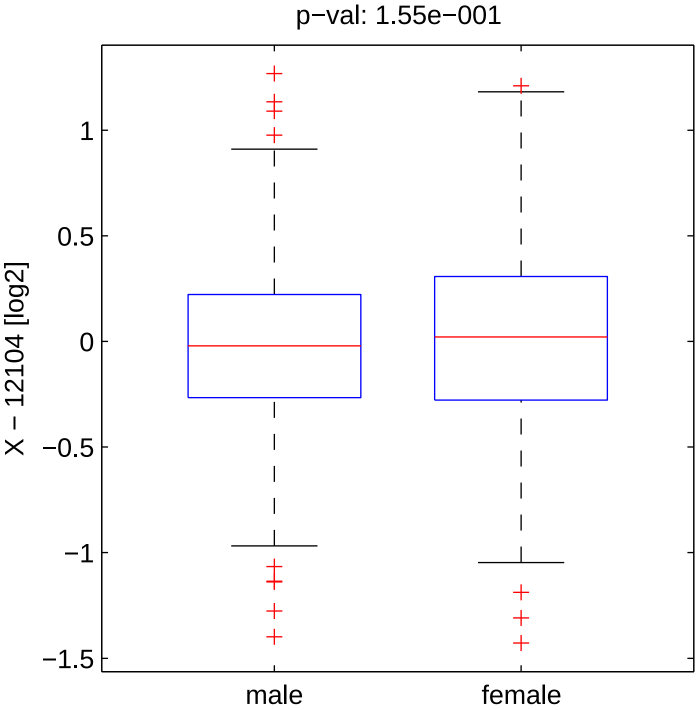
p−val: 1.55e−001
X − 12104 [log2]
1
0.5
0
−0.5
−1
−1.5
male
female

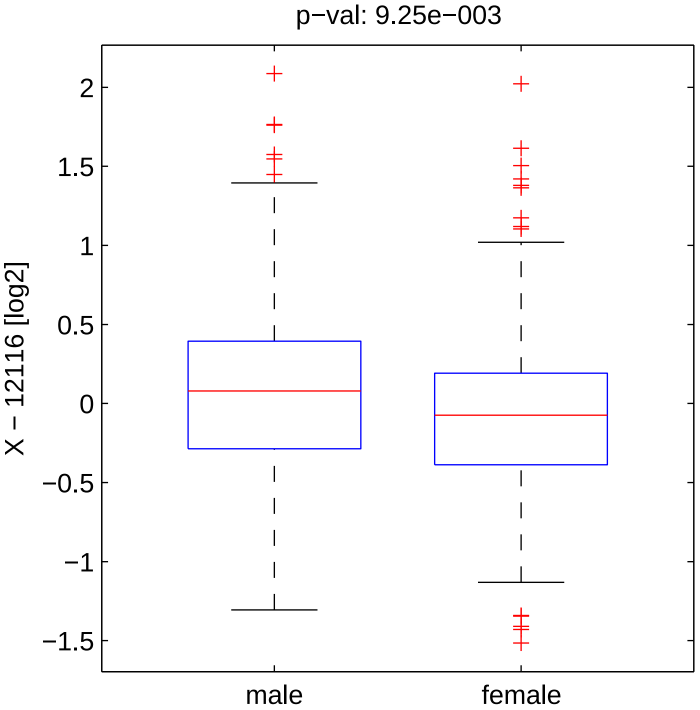
p−val: 9.25e−003
X − 12116 [log2]
2
1.5
1
0.5
0
−0.5
−1
−1.5
male
female

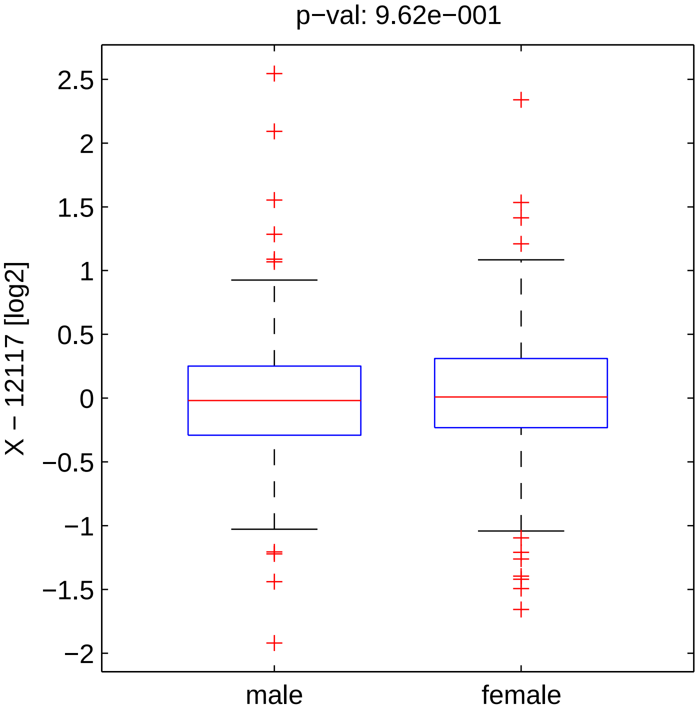

p−val: 9.62e−001
X − 12117 [log2]
2.5
2
1.5
1
0.5
0
−0.5
−1
−1.5
−2
male
female

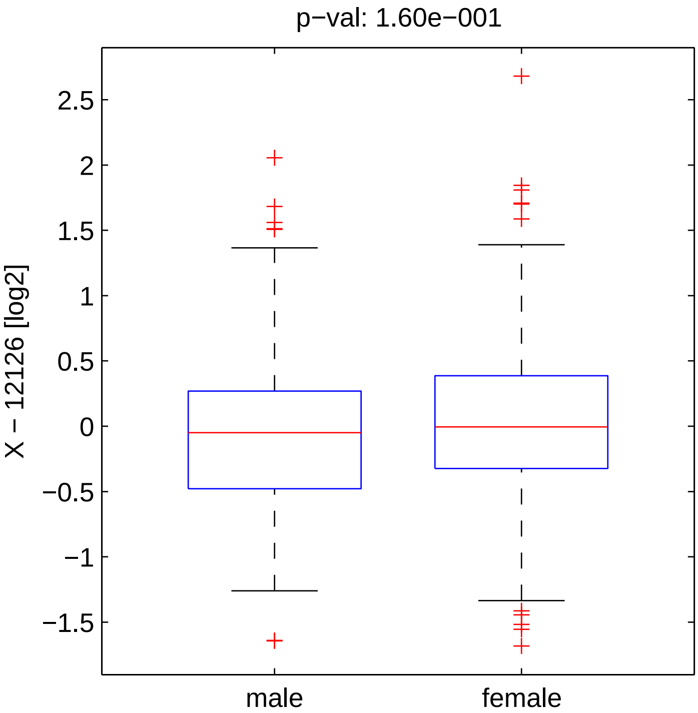

p−val: 1.60e−001
2.5
2
1.5
1
0.5
0
−0.5
−1
−1.5
X − 12126 [log2]
male
female

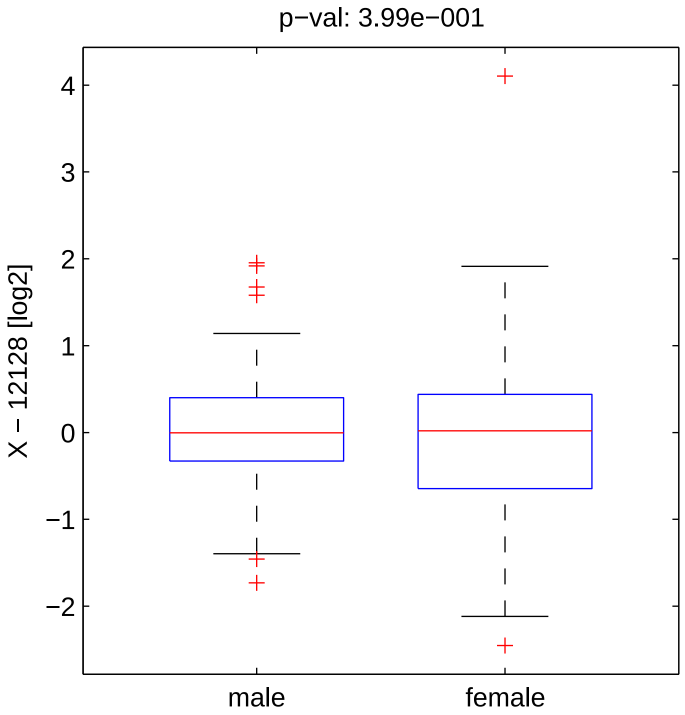

p−val: 3.99e−001
X − 12128 [log2]
4
3
2
1
0
−1
−2
male
female

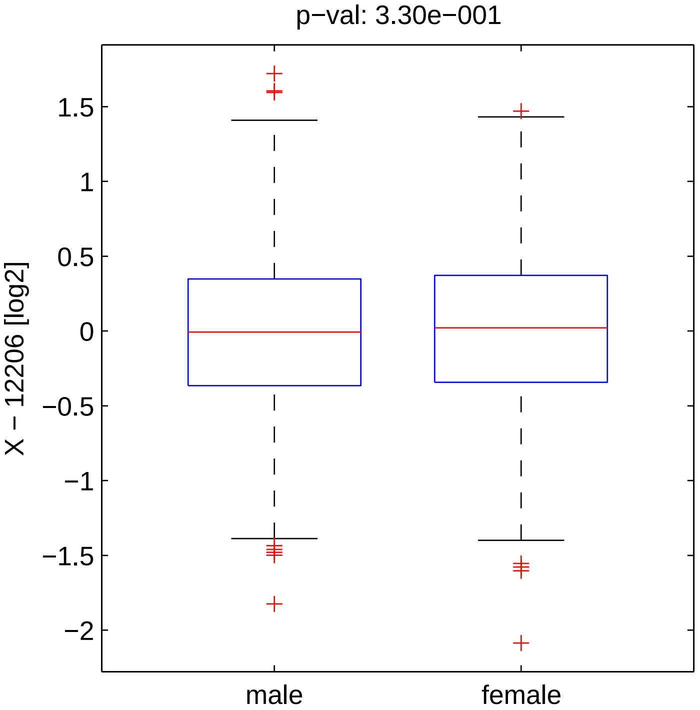
p−val: 3.30e−001
X − 12206 [log2]
1.5
1
0.5
0
−0.5
−1
−1.5
−2
male
female

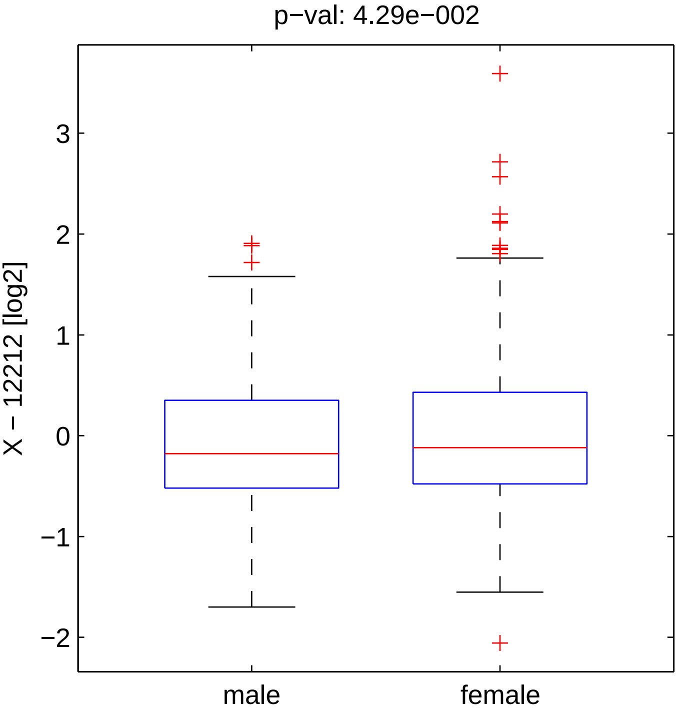
p−val: 4.29e−002
X − 12212 [log2]
3
2
1
0
−1
−2
male
female

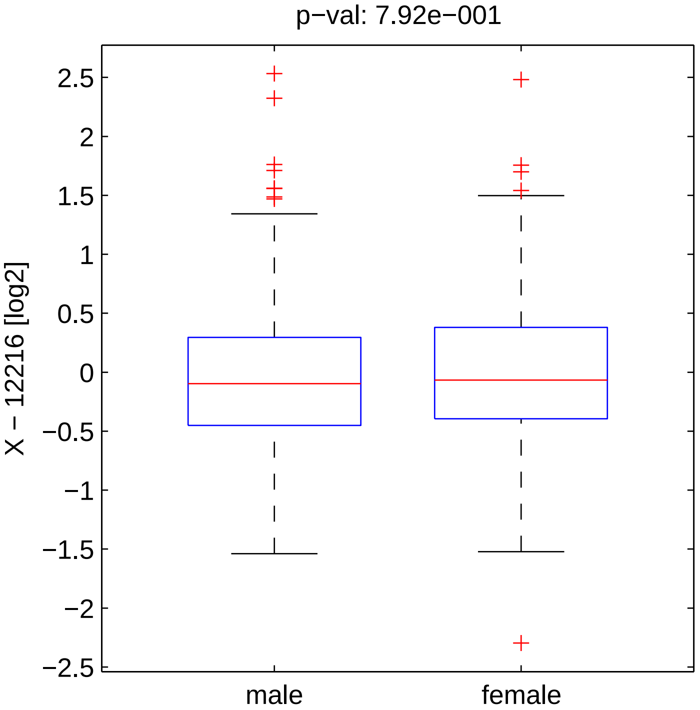
p−val: 7.92e−001
2.5
2
1.5
1
0.5
0
−0.5
−1
−1.5
−2
−2.5
X − 12216 [log2]
male
female

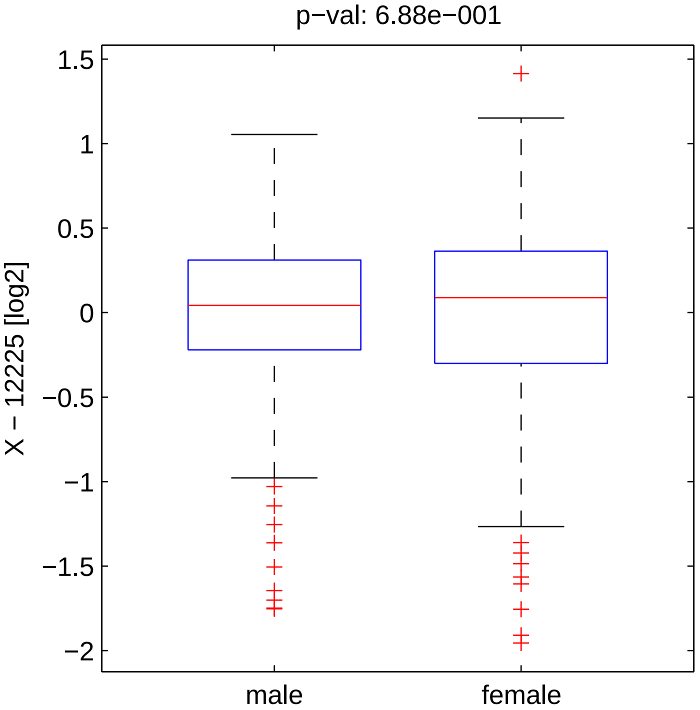
p−val: 6.88e−001
1.5
1
0.5
0
−0.5
−1
−1.5
−2
X − 12225 [log2]
male
female

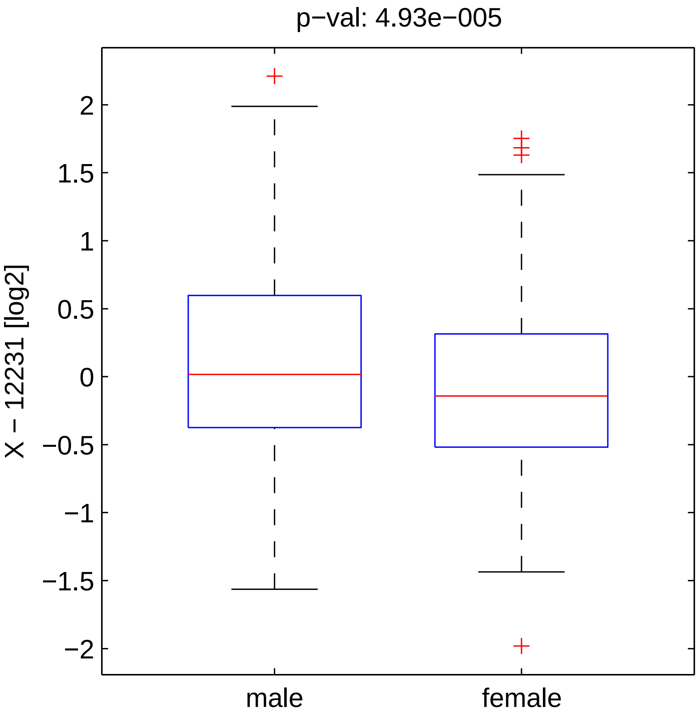
p−val: 4.93e−005
X − 12231 [log2]
2
1.5
1
0.5
0
−0.5
−1
−1.5
−2
male
female

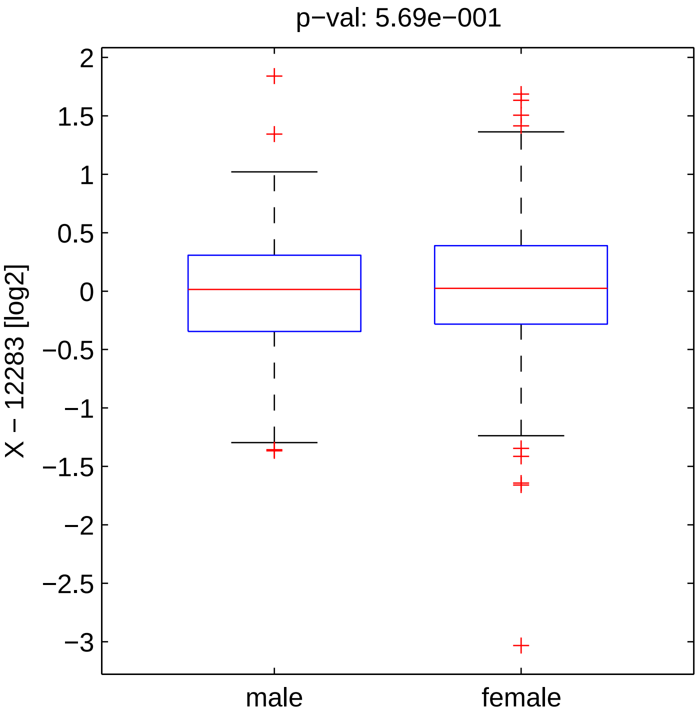
p−val: 5.69e−001
X − 12283 [log2]
2
1.5
1
0.5
0
−0.5
−1
−1.5
−2
−2.5
−3
male
female

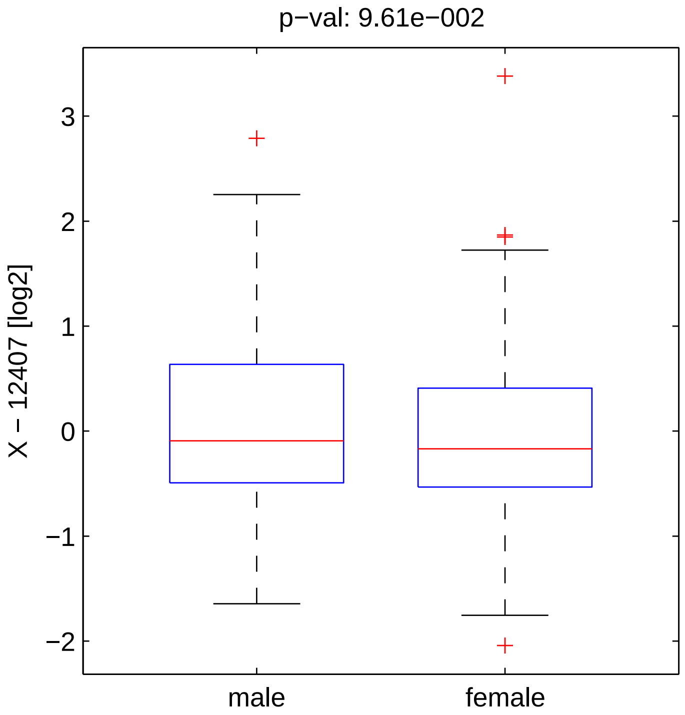
p−val: 9.61e−002
X − 12407 [log2]
3
2
1
0
−1
−2
male
female

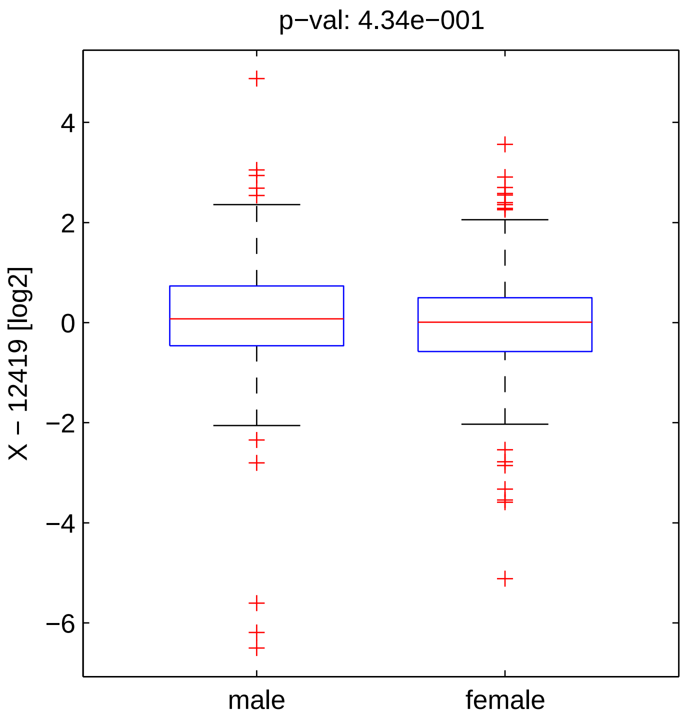
p−val: 4.34e−001
X − 12419 [log2]
4
2
0
−2
−4
−6
male
female

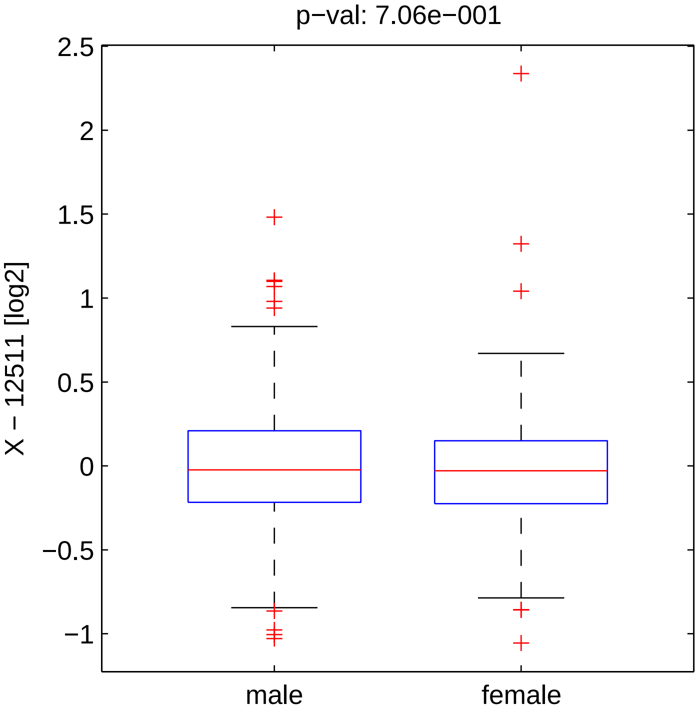

p−val: 7.06e−001
2.5
2
1.5
1
0.5
0
−0.5
−1
X − 12511 [log2]
male
female

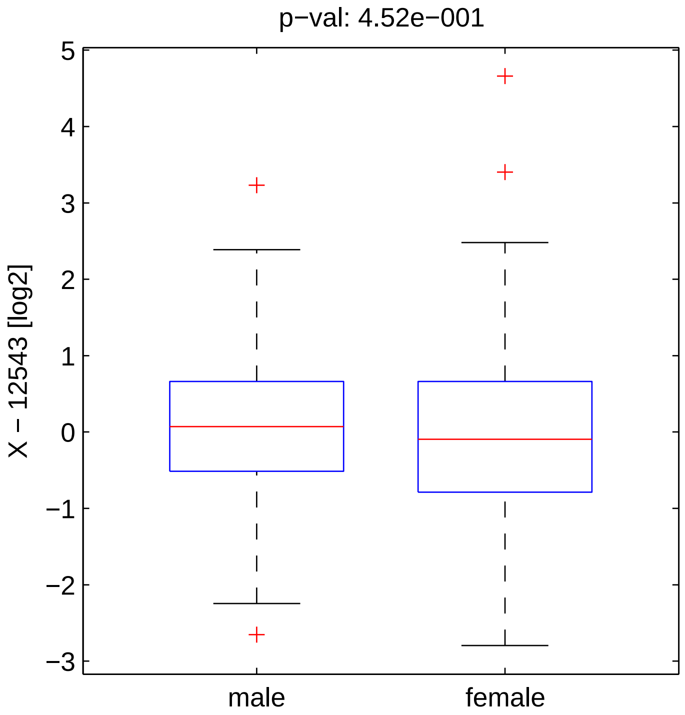

p−val: 4.52e−001
X − 12543 [log2]
5
4
3
2
1
0
−1
−2
−3
male
female

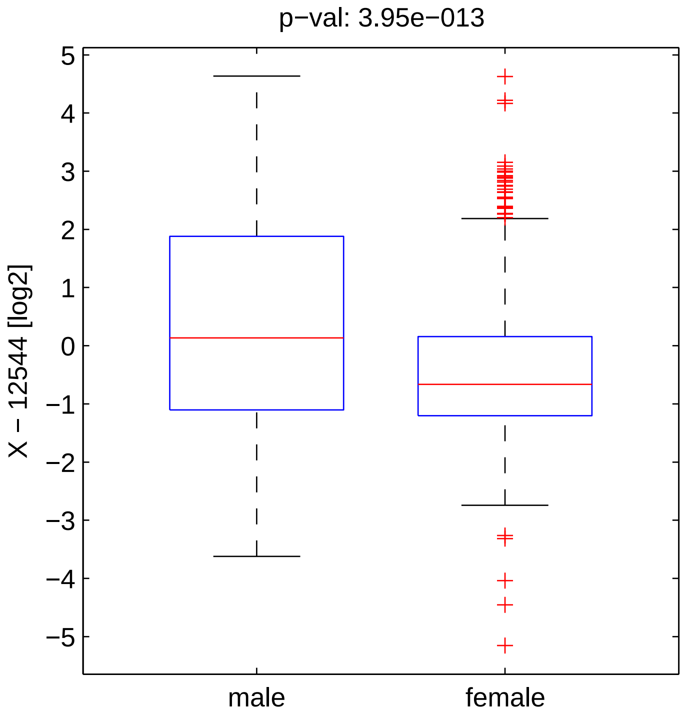
p−val: 3.95e−013
X − 12544 [log2]
5
4
3
2
1
0
−1
−2
−3
−4
−5
male
female

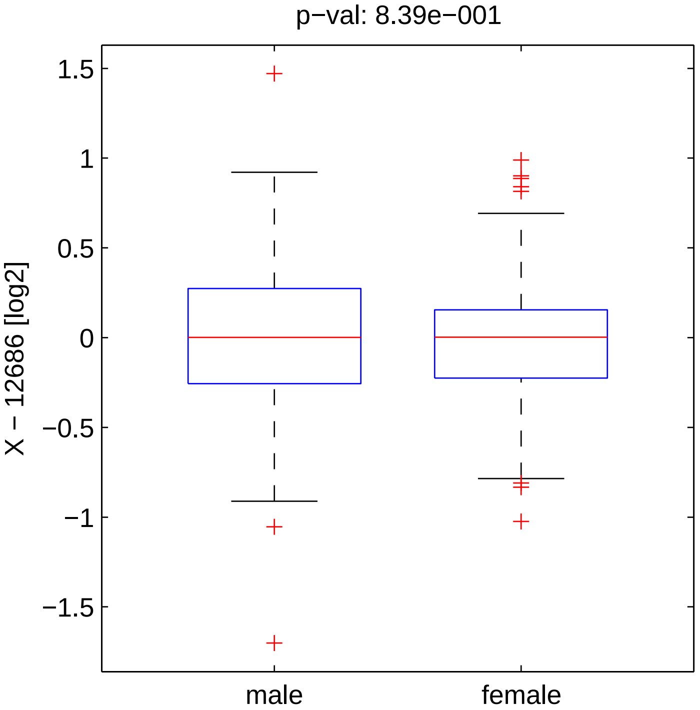

p−val: 8.39e−001
1.5
1
0.5
0
−0.5
−1
−1.5
X − 12686 [log2]
male
female

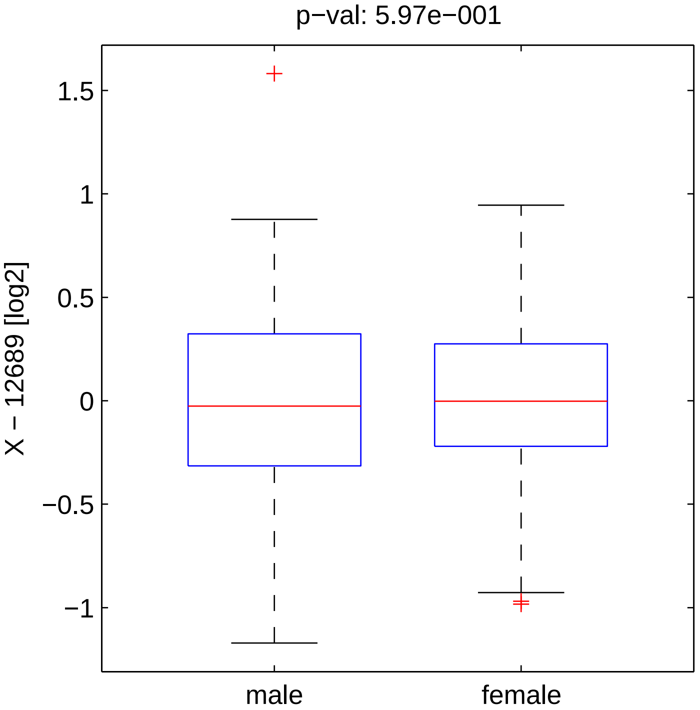
p−val: 5.97e−001
X − 12689 [log2]
1.5
1
0.5
0
−0.5
−1
male
female

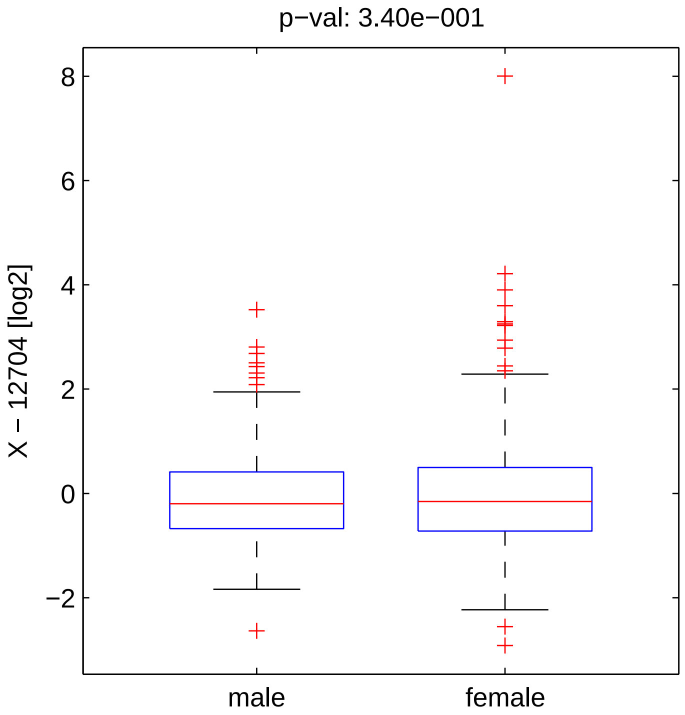

p−val: 3.40e−001
X − 12704 [log2]
8
6
4
2
0
−2
male
female

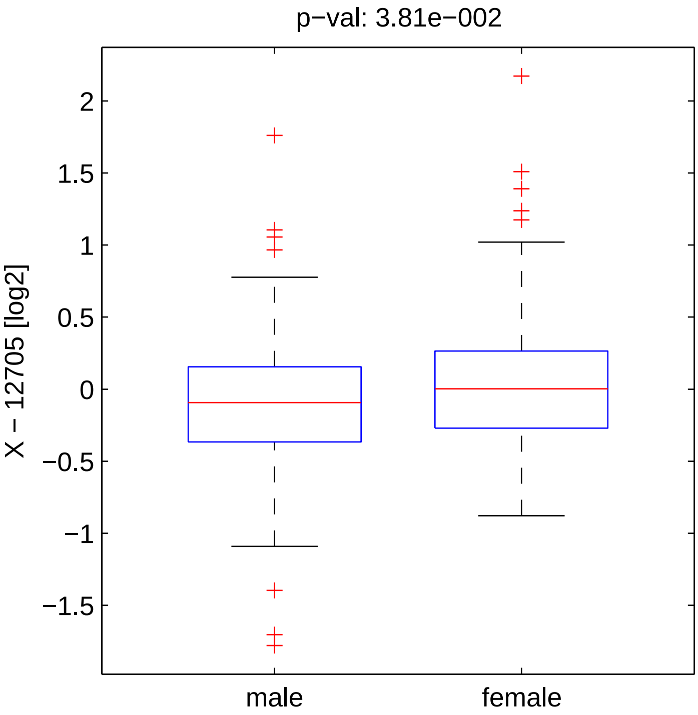
p−val: 3.81e−002
X − 12705 [log2]
2
1.5
1
0.5
0
−0.5
−1
−1.5
male
female

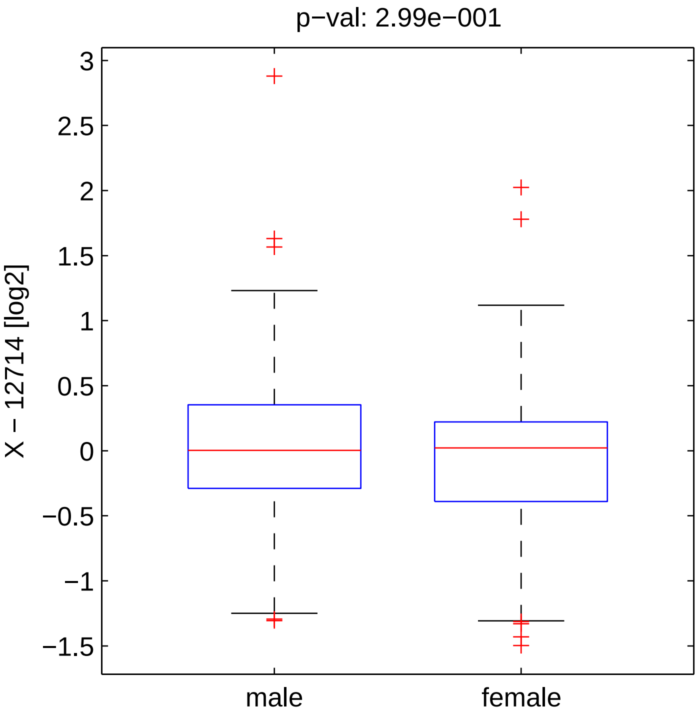

p−val: 2.99e−001
X − 12714 [log2]
3
2.5
2
1.5
1
0.5
0
−0.5
−1
−1.5
male
female

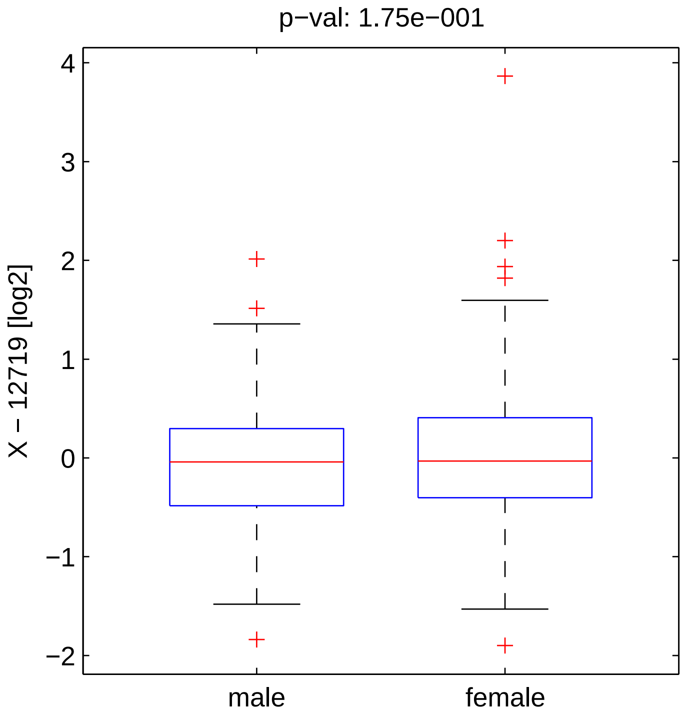
p−val: 1.75e−001
X − 12719 [log2]
4
3
2
1
0
−1
−2
male
female

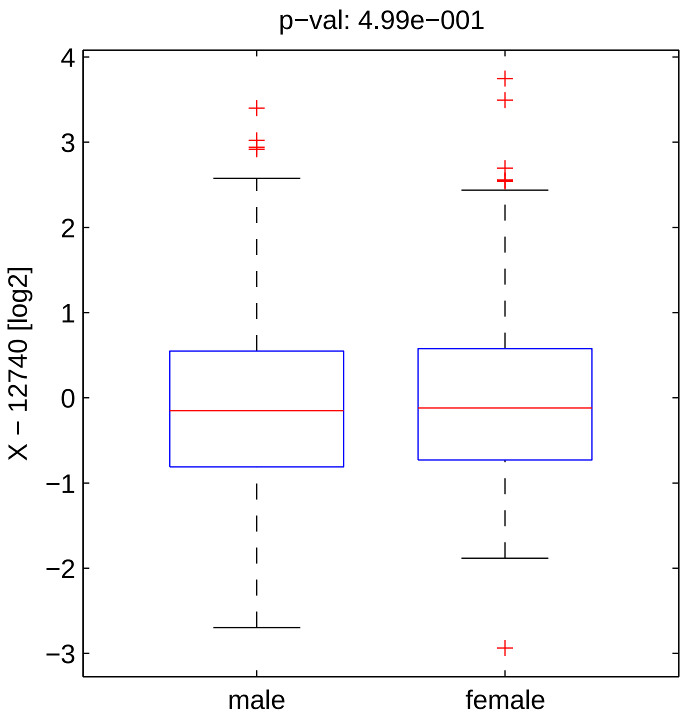
p−val: 4.99e−001
X − 12740 [log2]
4
3
2
1
0
−1
−2
−3
male
female

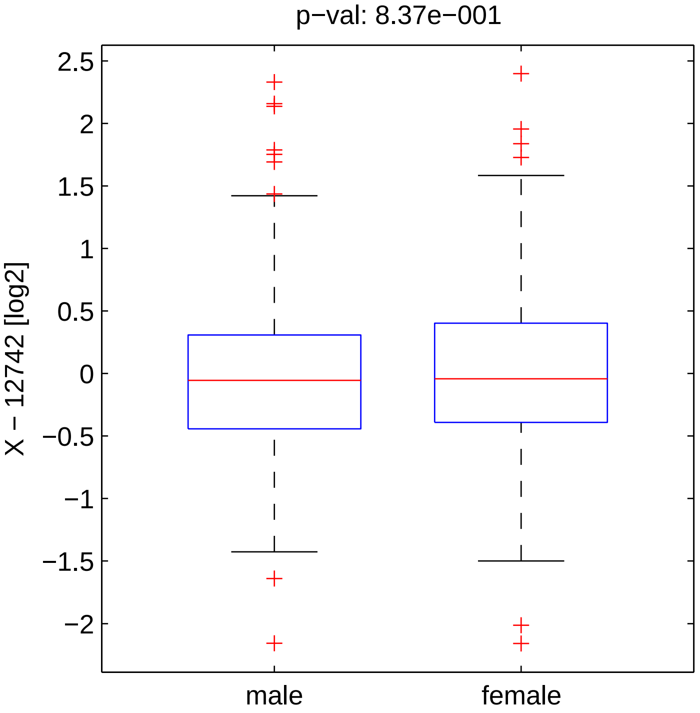
p−val: 8.37e−001
2.5
2
1.5
1
0.5
0
−0.5
−1
−1.5
−2
X − 12742 [log2]
male
female

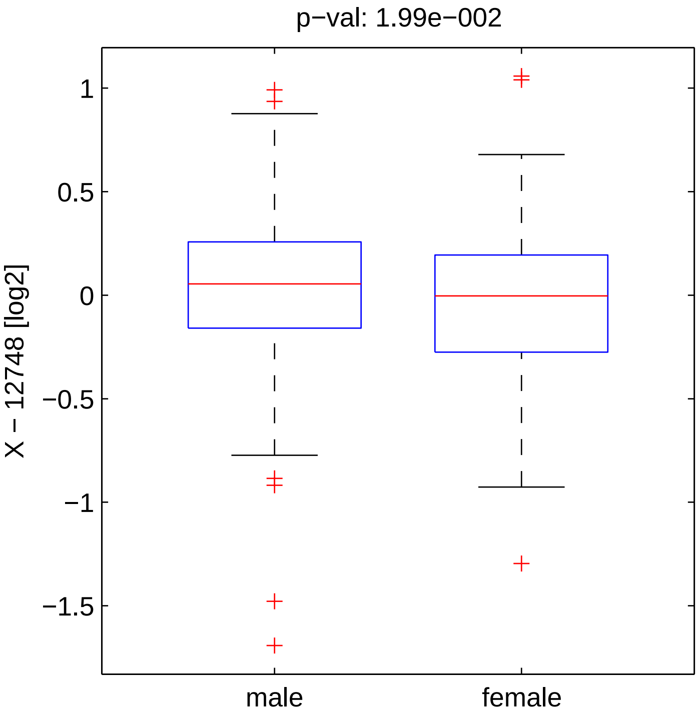
p−val: 1.99e−002
X − 12748 [log2]
1
0.5
0
−0.5
−1
−1.5
male
female

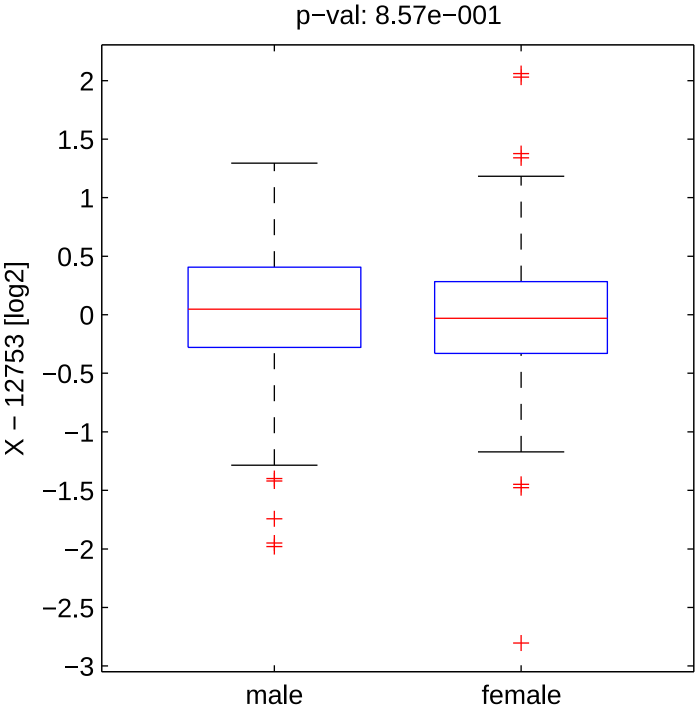

p−val: 8.57e−001
X − 12753 [log2]
2
1.5
1
0.5
0
−0.5
−1
−1.5
−2
−2.5
−3
male
female

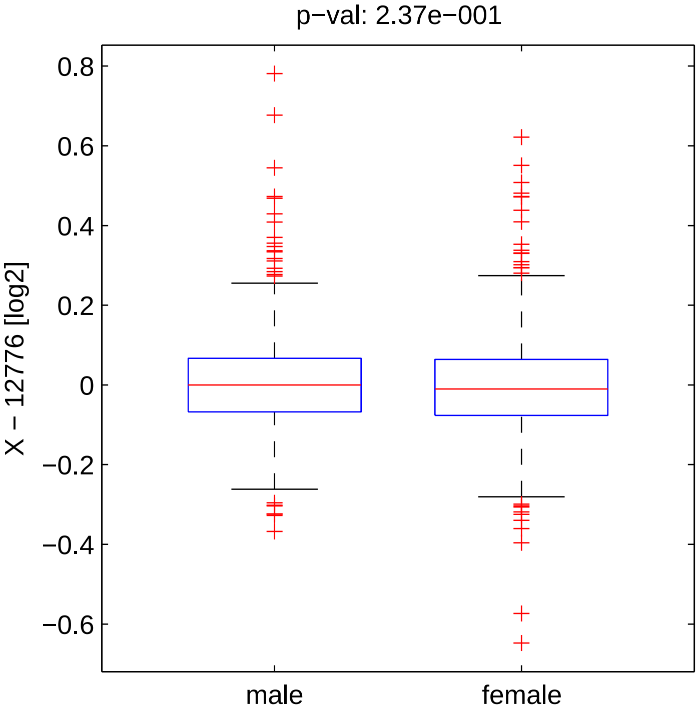
p−val: 2.37e−001
0.8
0.6
0.4
0.2
0
−0.2
−0.4
−0.6
X − 12776 [log2]
male
female

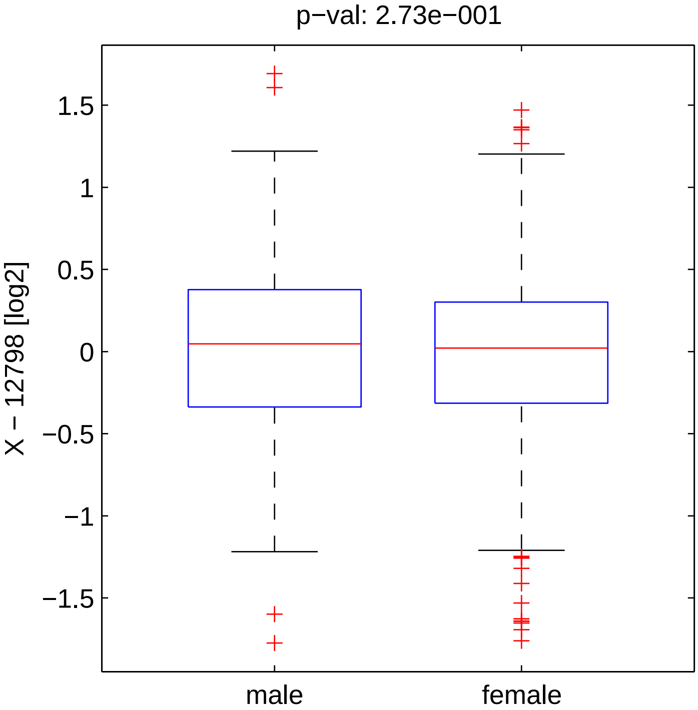

p−val: 2.73e−001
1.5
1
0.5
0
−0.5
−1
−1.5
X − 12798 [log2]
male
female

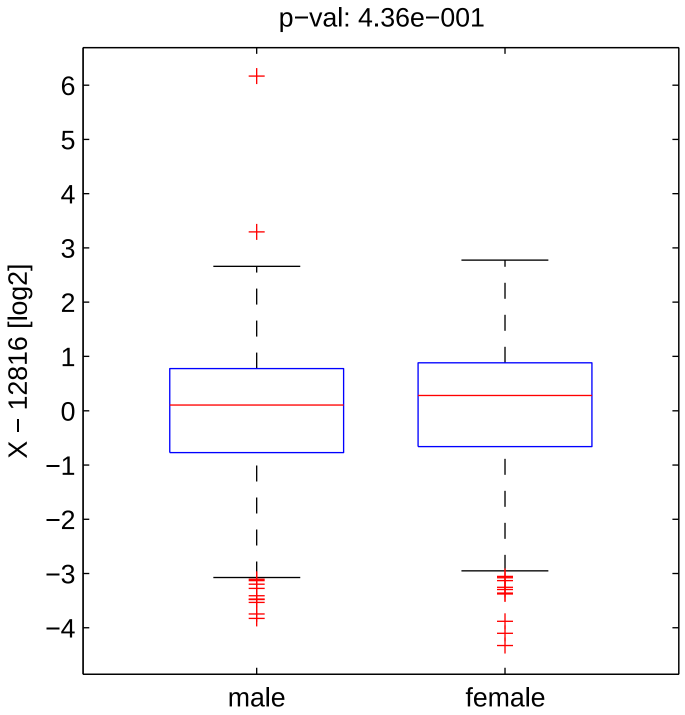
p−val: 4.36e−001
X − 12816 [log2]
6
5
4
3
2
1
0
−1
−2
−3
−4
male
female

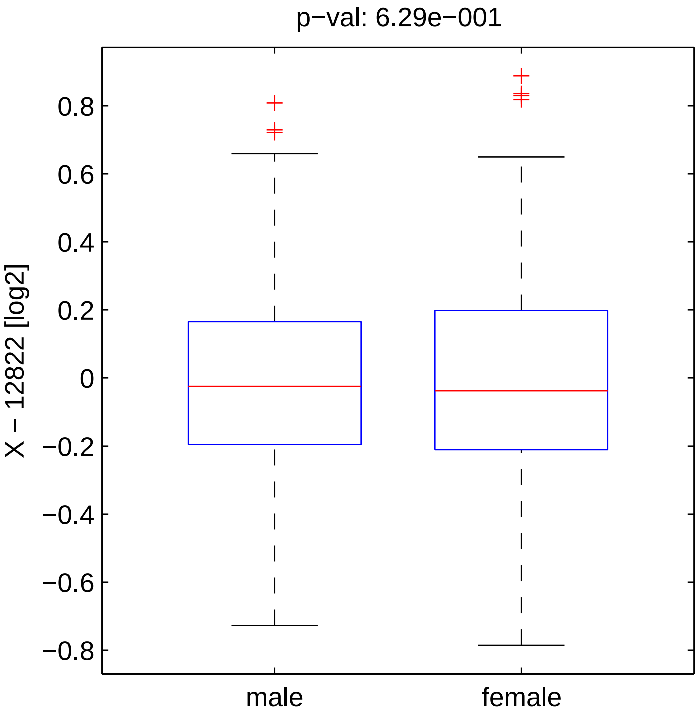
p−val: 6.29e−001
X − 12822 [log2]
0.8
0.6
0.4
0.2
0
−0.2
−0.4
−0.6
−0.8
male
female

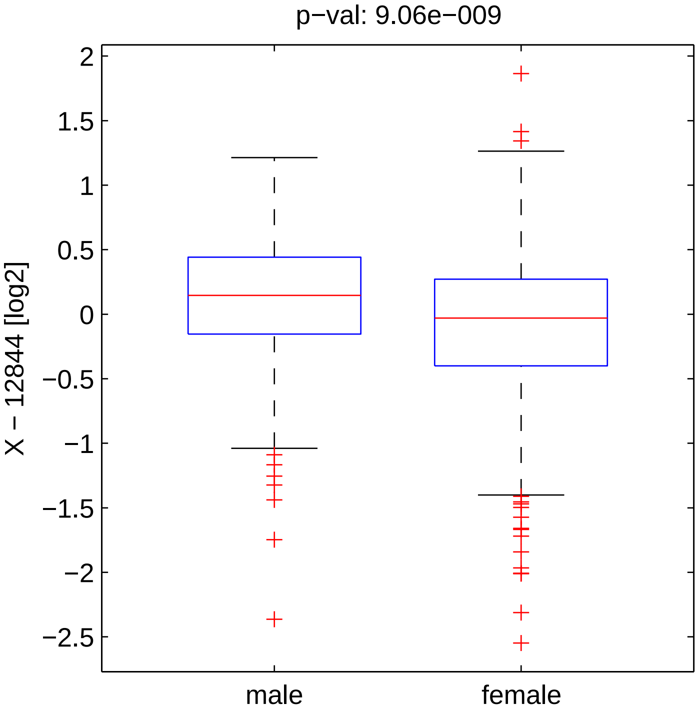
p−val: 9.06e−009
2
1.5
1
0.5
0
−0.5
−1
−1.5
−2
−2.5
X − 12844 [log2]
male
female

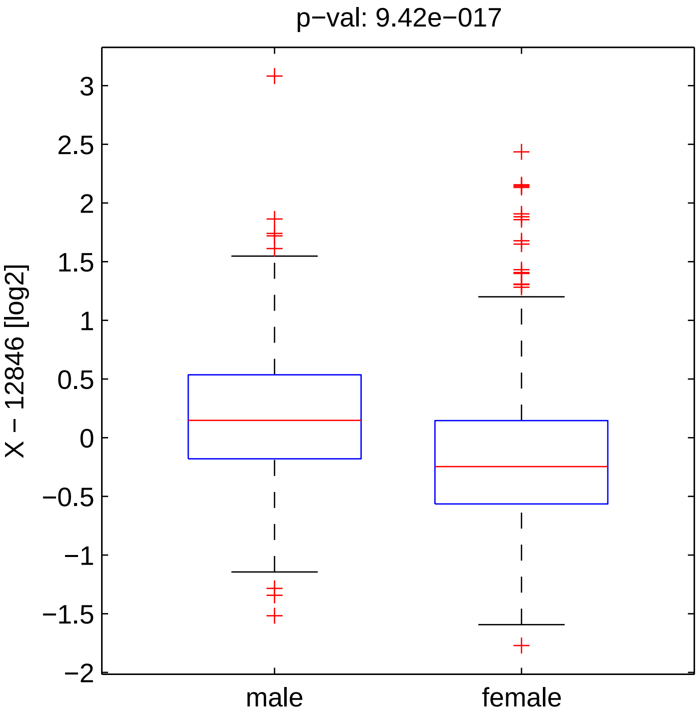
p−val: 9.42e−017
X − 12846 [log2]
3
2.5
2
1.5
1
0.5
0
−0.5
−1
−1.5
−2
male
female

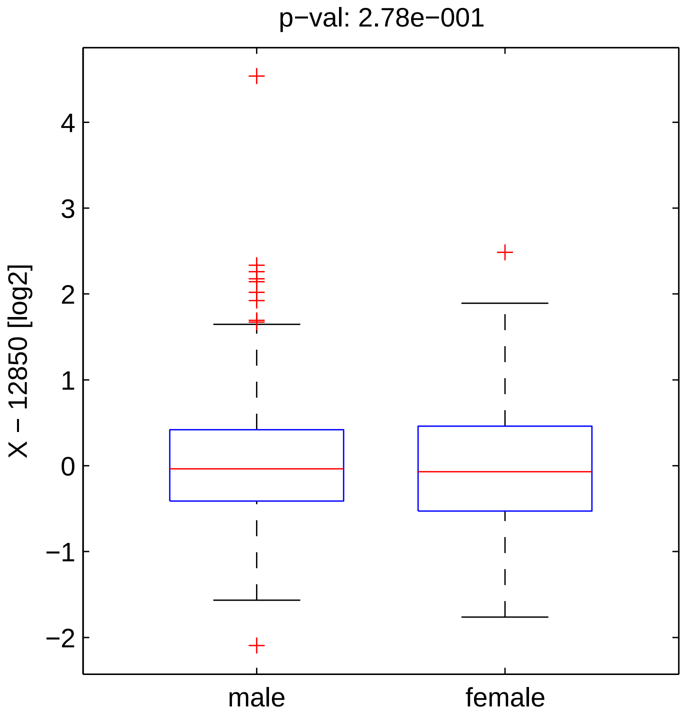

p−val: 2.78e−001
X − 12850 [log2]
4
3
2
1
0
−1
−2
male
female

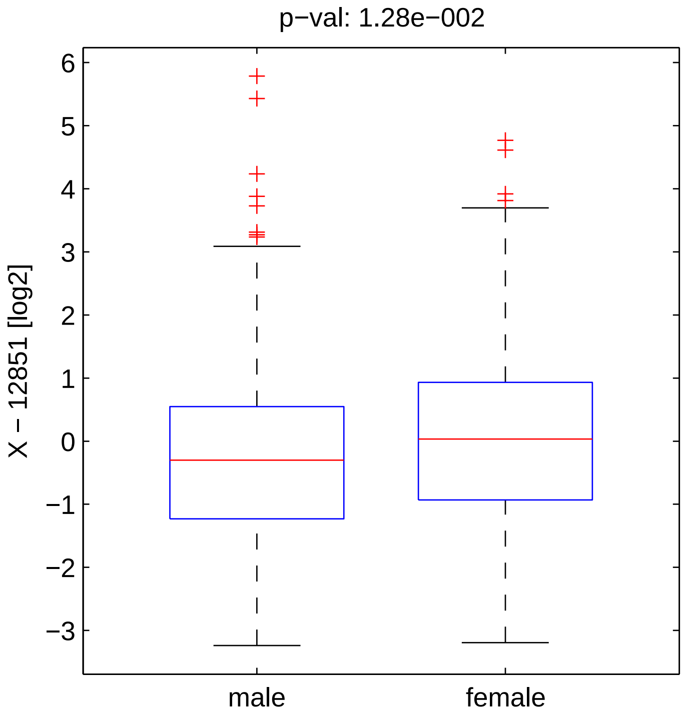

p−val: 1.28e−002
X − 12851 [log2]
6
5
4
3
2
1
0
−1
−2
−3
male
female

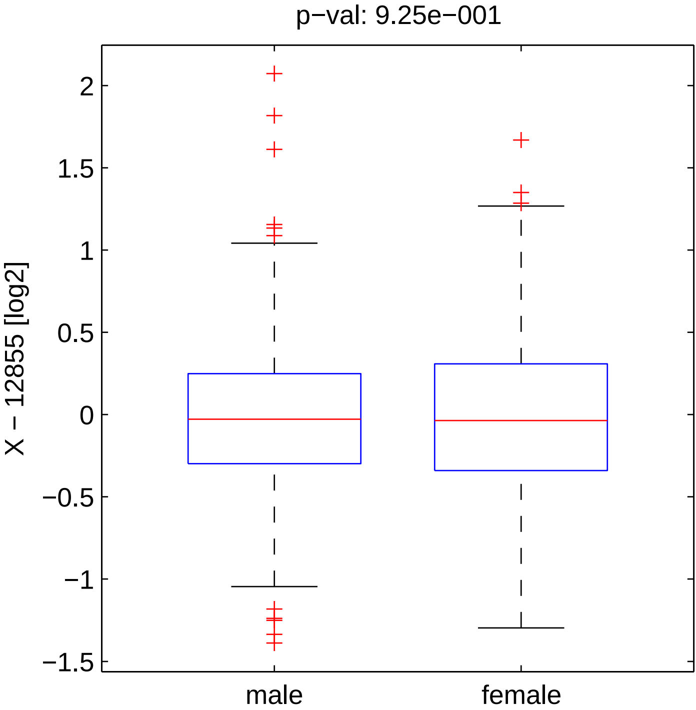

p−val: 9.25e−001
X − 12855 [log2]
2
1.5
1
0.5
0
−0.5
−1
−1.5
male
female

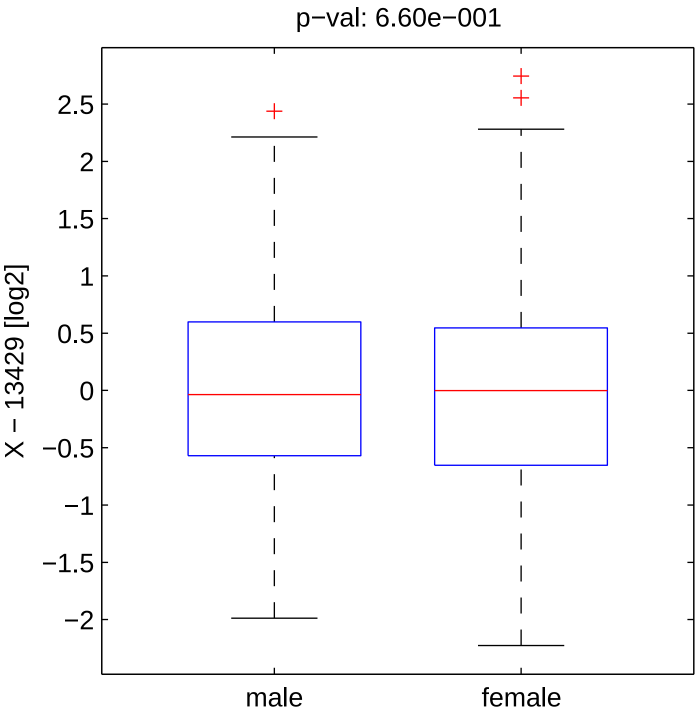
p−val: 6.60e−001
X − 13429 [log2]
2.5
2
1.5
1
0.5
0
−0.5
−1
−1.5
−2
male
female

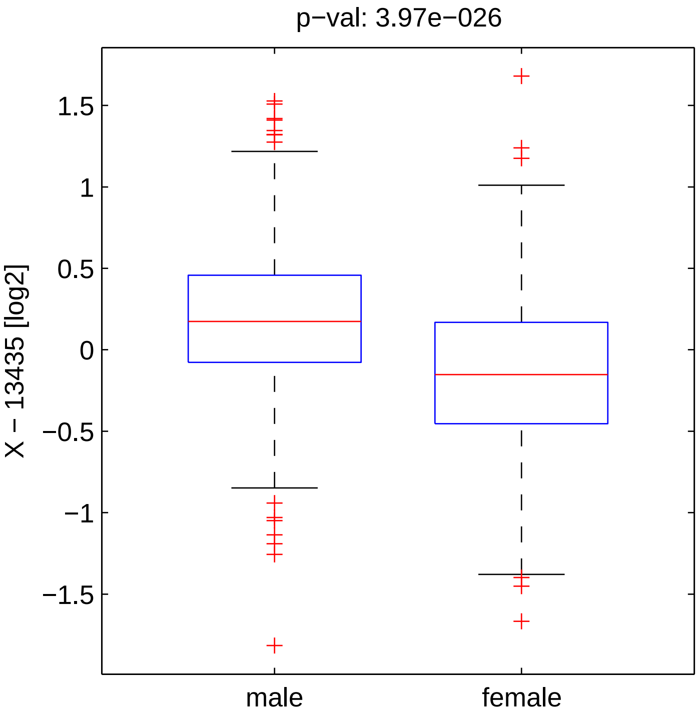
p−val: 3.97e−026
X − 13435 [log2]
1.5
1
0.5
0
−0.5
−1
−1.5
male
female

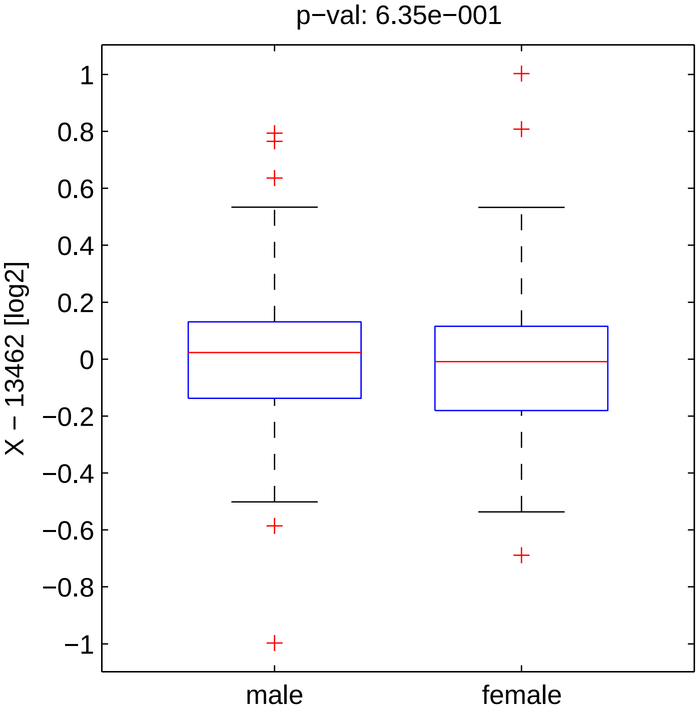
p−val: 6.35e−001
X − 13462 [log2]
1
0.8
0.6
0.4
0.2
0
−0.2
−0.4
−0.6
−0.8
−1
male
female

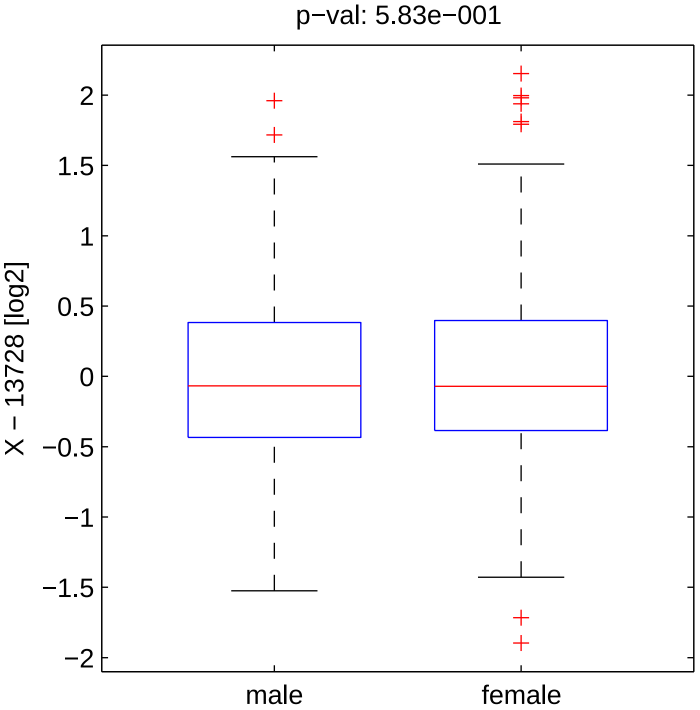
p−val: 5.83e−001
X − 13728 [log2]
2
1.5
1
0.5
0
−0.5
−1
−1.5
−2
male
female

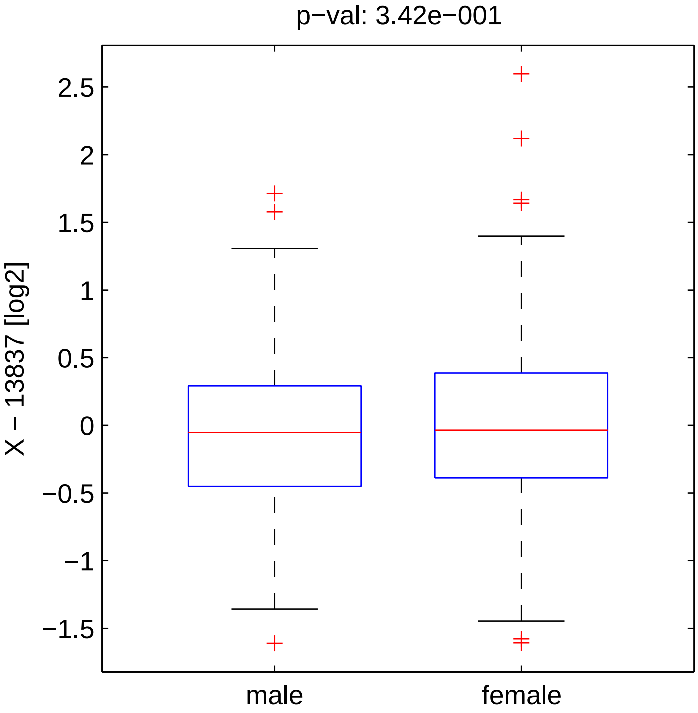
p−val: 3.42e−001
X − 13837 [log2]
2.5
2
1.5
1
0.5
0
−0.5
−1
−1.5
male
female

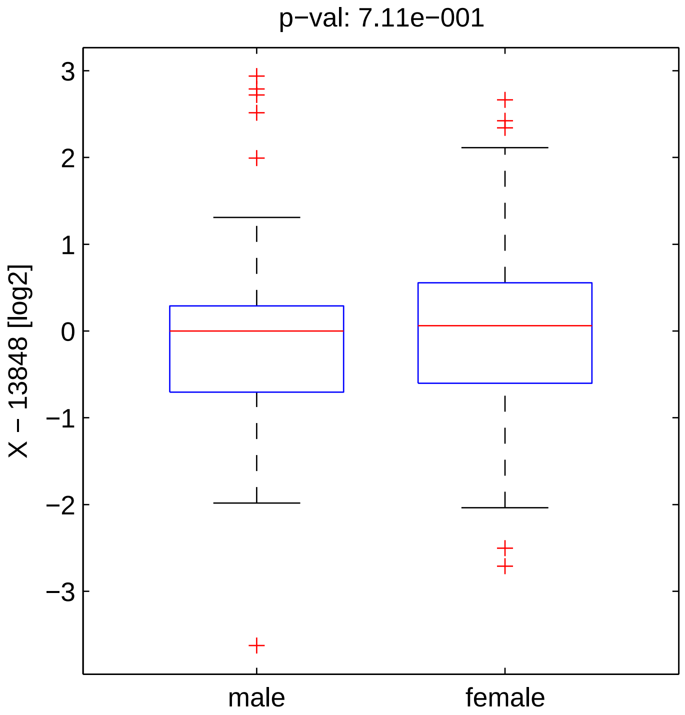

p−val: 7.11e−001
3
2
1
0
−1
−2
−3
X − 13848 [log2]
male
female

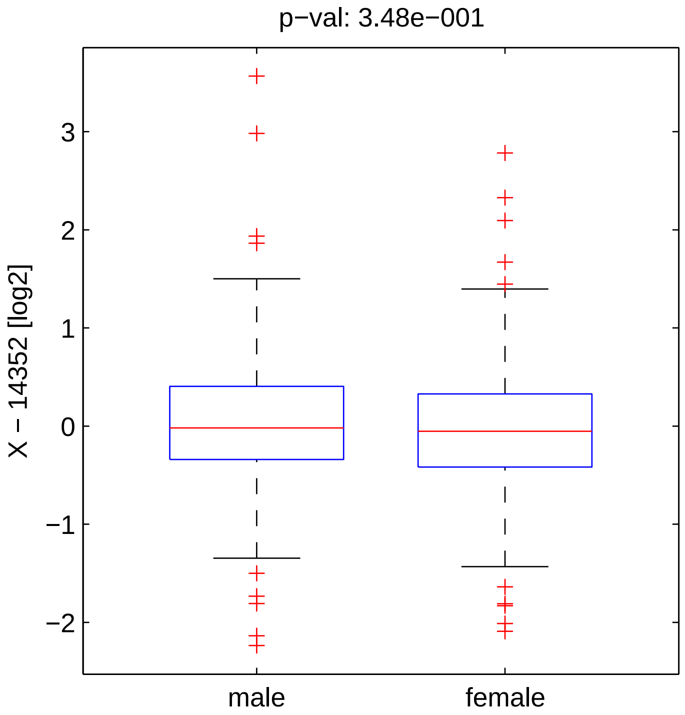

p−val: 3.48e−001
X − 14352 [log2]
3
2
1
0
−1
−2
male
female

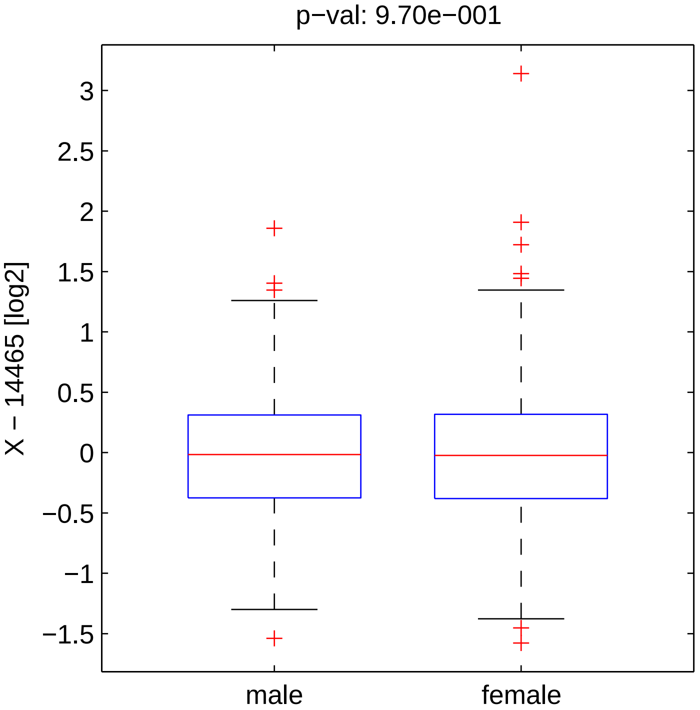
p−val: 9.70e−001
X − 14465 [log2]
3
2.5
2
1.5
1
0.5
0
−0.5
−1
−1.5
male
female

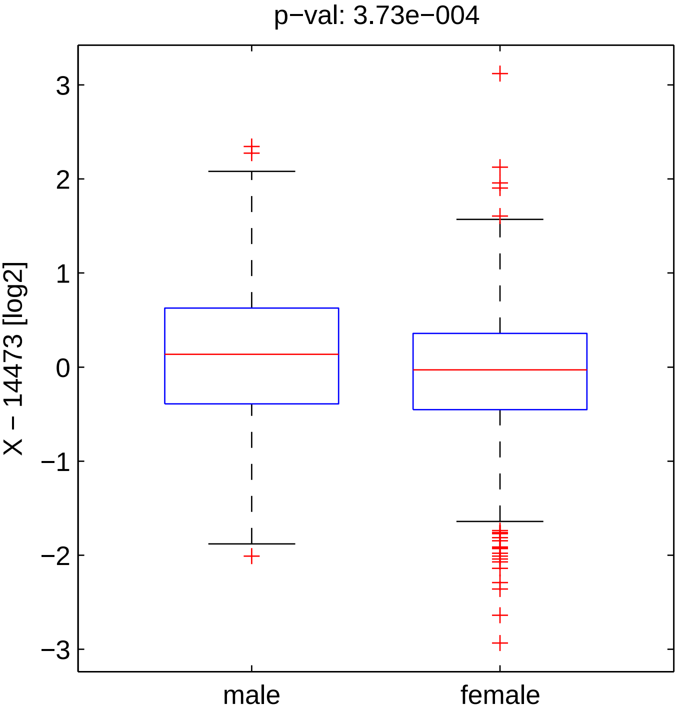
p−val: 3.73e−004
X − 14473 [log2]
3
2
1
0
−1
−2
−3
male
female

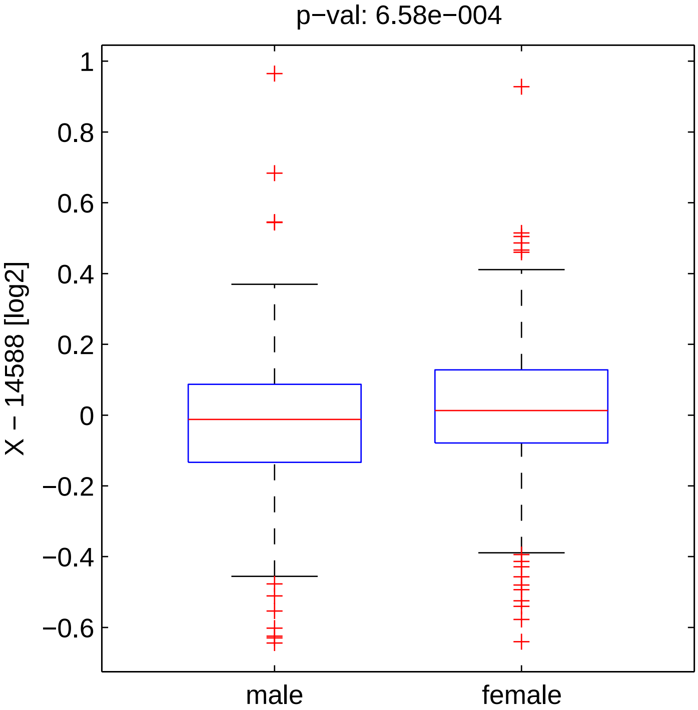
p−val: 6.58e−004
X − 14588 [log2]
1
0.8
0.6
0.4
0.2
0
−0.2
−0.4
−0.6
male
female

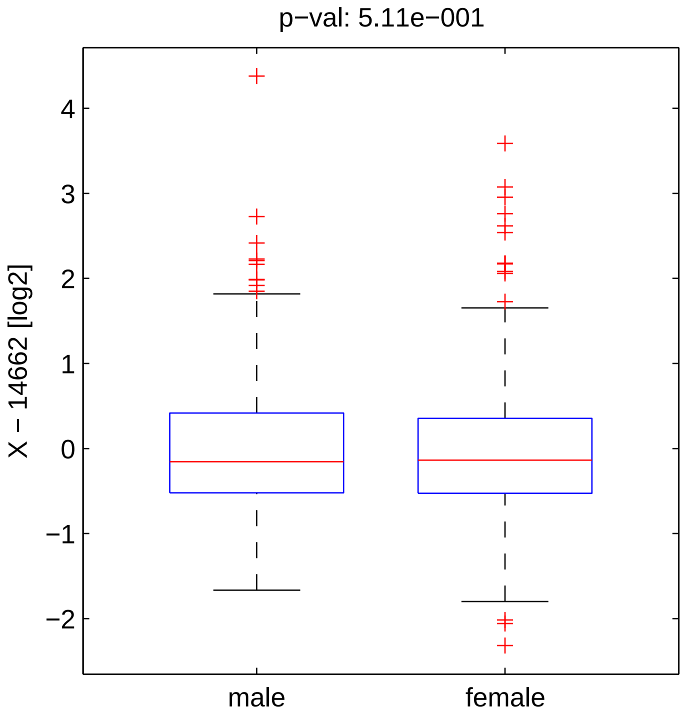

p−val: 5.11e−001
X − 14662 [log2]
4
3
2
1
0
−1
−2
male
female

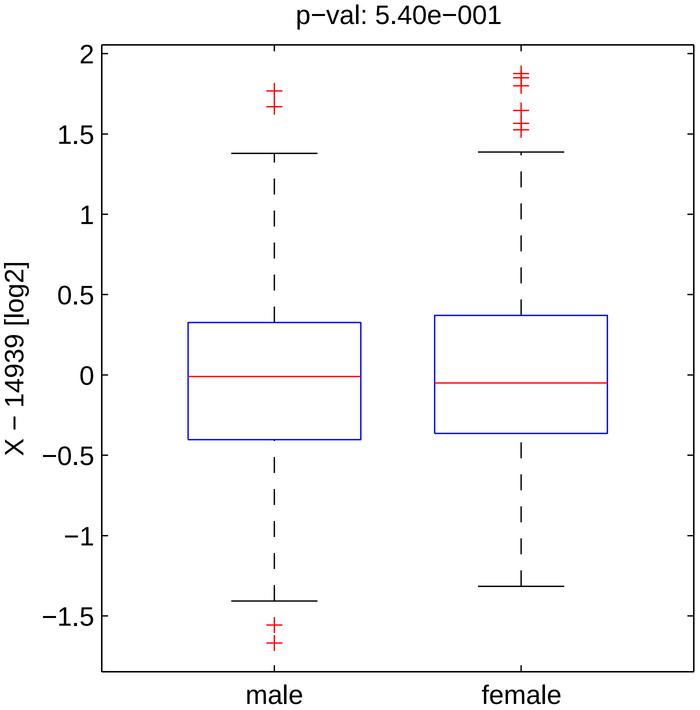

p−val: 5.40e−001
2
1.5
1
0.5
0
−0.5
−1
−1.5
X − 14939 [log2]
male
female

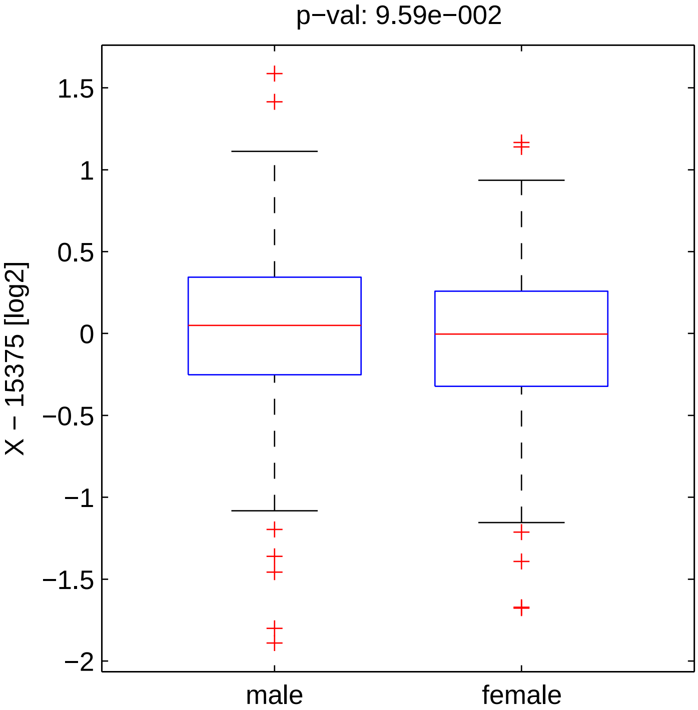
p−val: 9.59e−002
1.5
1
0.5
0
−0.5
−1
−1.5
−2
X − 15375 [log2]
male
female

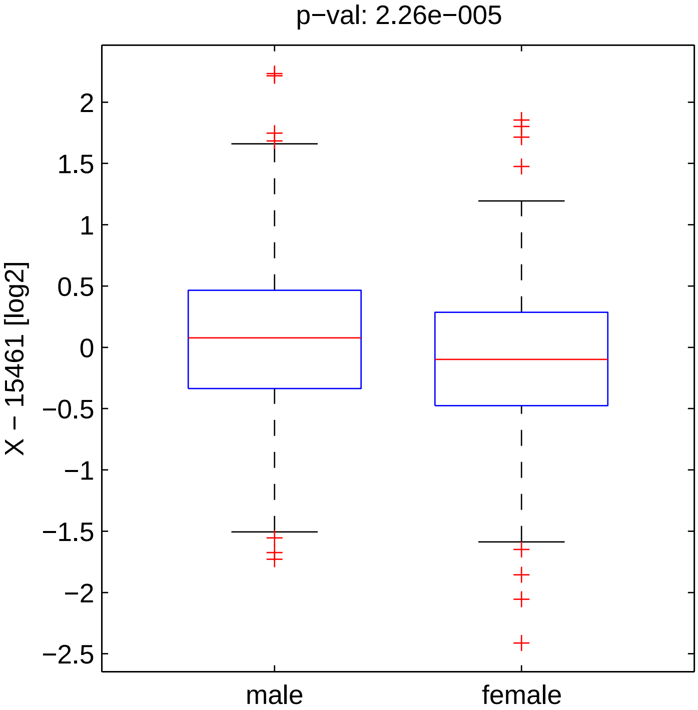

p−val: 2.26e−005
X − 15461 [log2]
2
1.5
1
0.5
0
−0.5
−1
−1.5
−2
−2.5
male
female

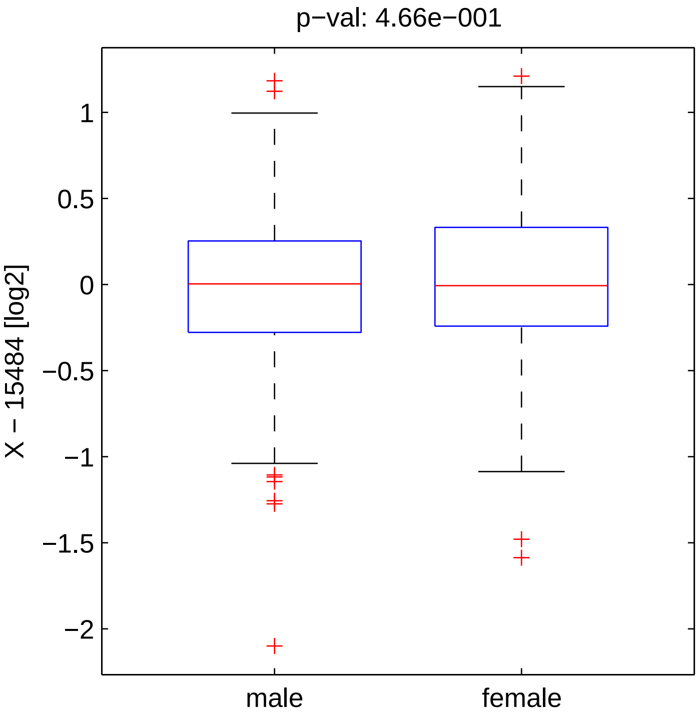

p−val: 4.66e−001
X − 15484 [log2]
1
0.5
0
−0.5
−1
−1.5
−2
male
female

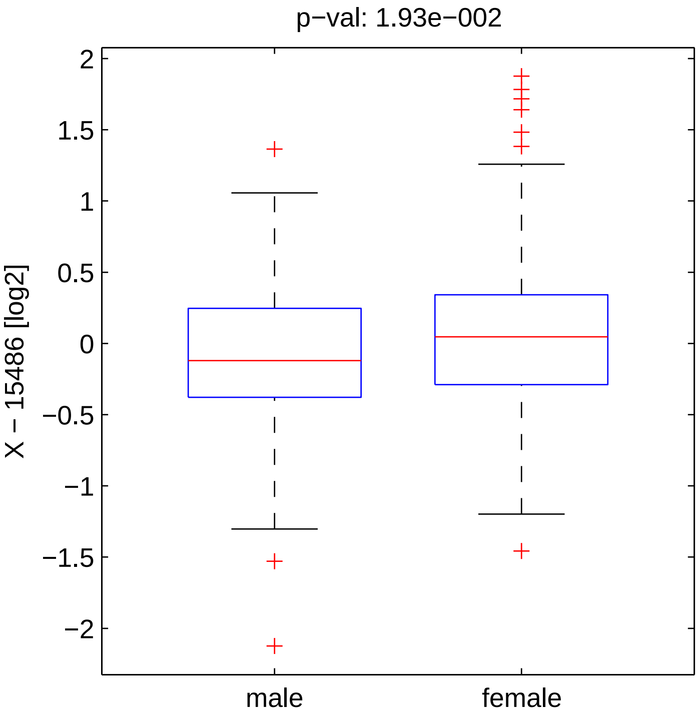

p−val: 1.93e−002
X − 15486 [log2]
2
1.5
1
0.5
0
−0.5
−1
−1.5
−2
male
female

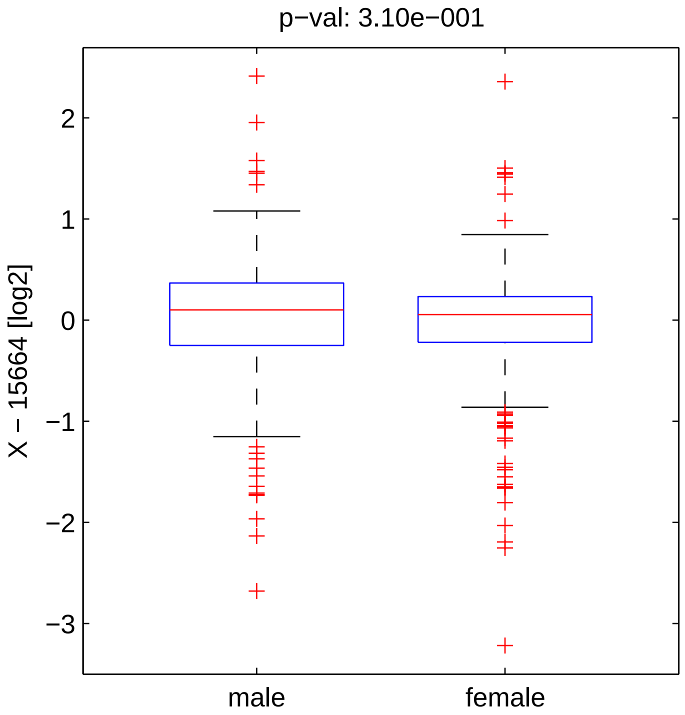
p−val: 3.10e−001
X − 15664 [log2]
2
1
0
−1
−2
−3
male
female

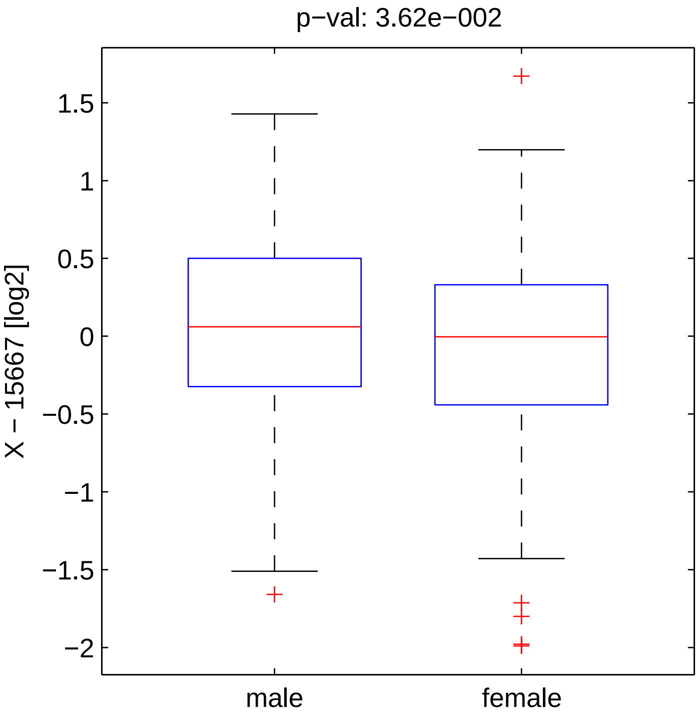

p−val: 3.62e−002
1.5
1
0.5
0
−0.5
−1
−1.5
−2
X − 15667 [log2]
male
female

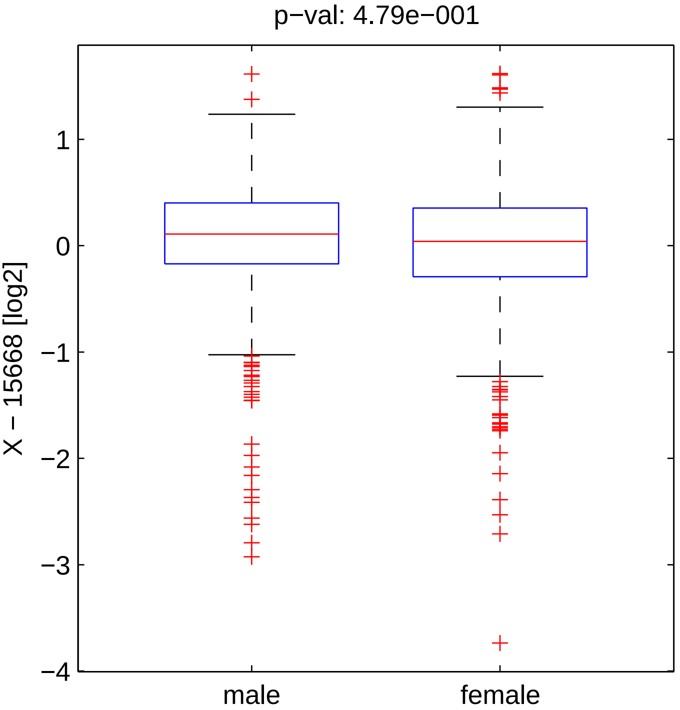
p−val: 4.79e−001
X − 15668 [log2]
1
0
−1
−2
−3
−4
male
female

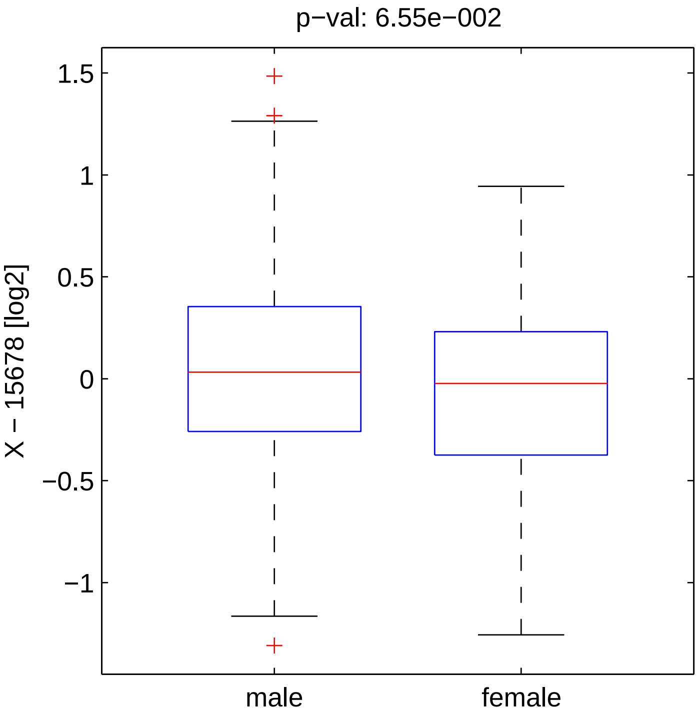
p−val: 6.55e−002
X − 15678 [log2]
1.5
1
0.5
0
−0.5
−1
male
female

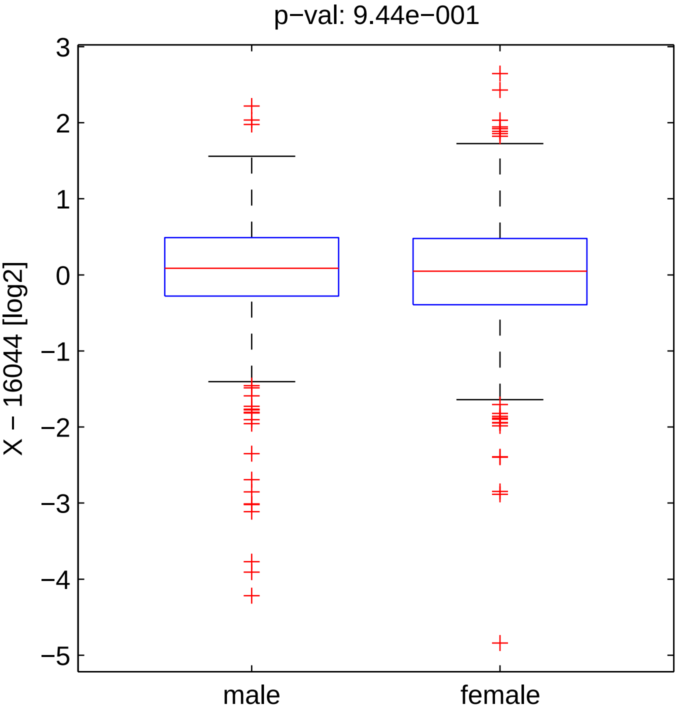
p−val: 9.44e−001
X − 16044 [log2]
3
2
1
0
−1
−2
−3
−4
−5
male
female

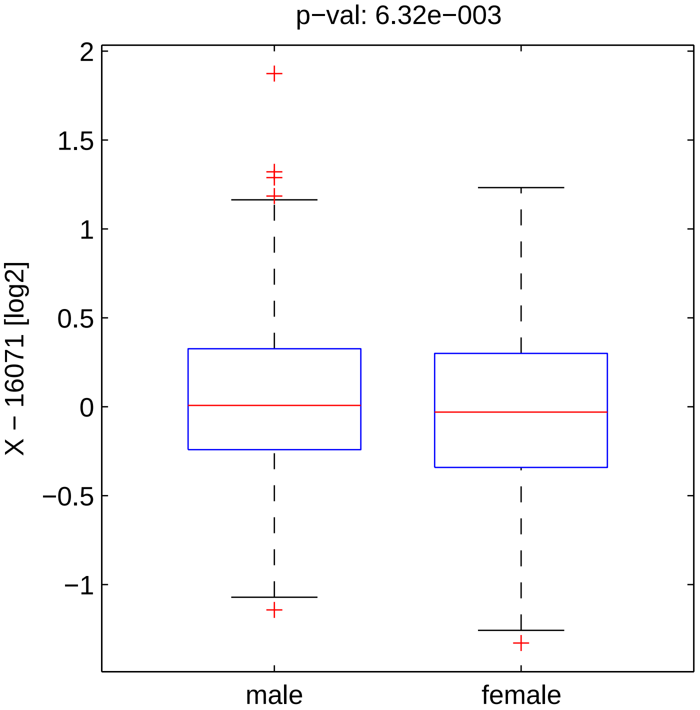
p−val: 6.32e−003
X − 16071 [log2]
2
1.5
1
0.5
0
−0.5
−1
male
female

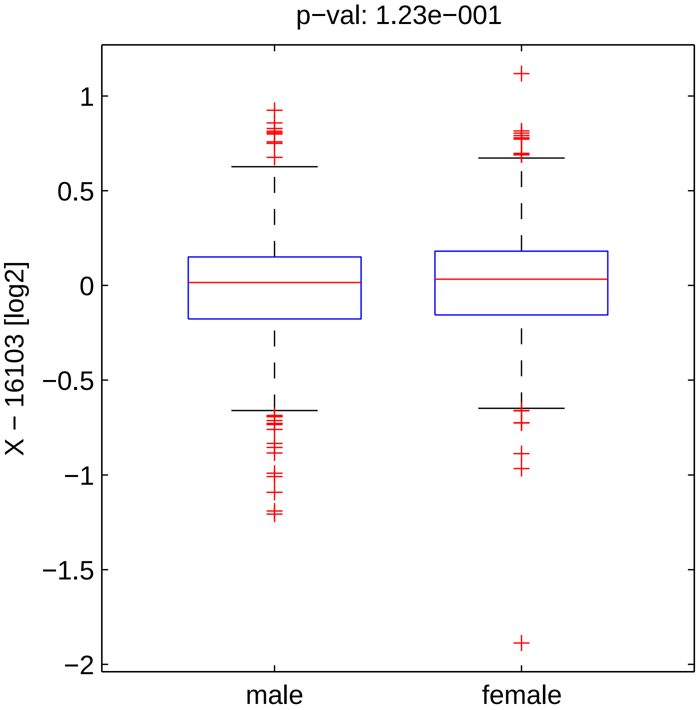
p−val: 1.23e−001
X − 16103 [log2]
1
0.5
0
−0.5
−1
−1.5
−2
male
female

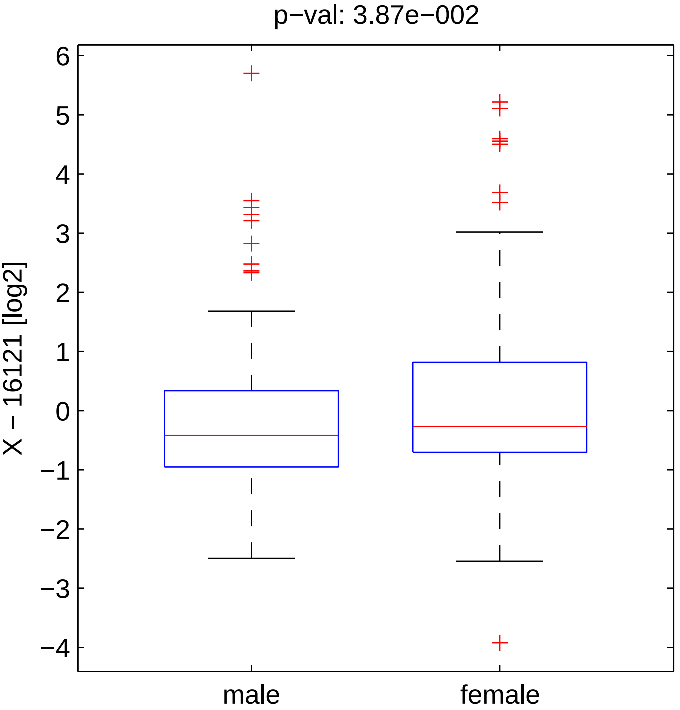

p−val: 3.87e−002
X − 16121 [log2]
6
5
4
3
2
1
0
−1
−2
−3
−4
male
female

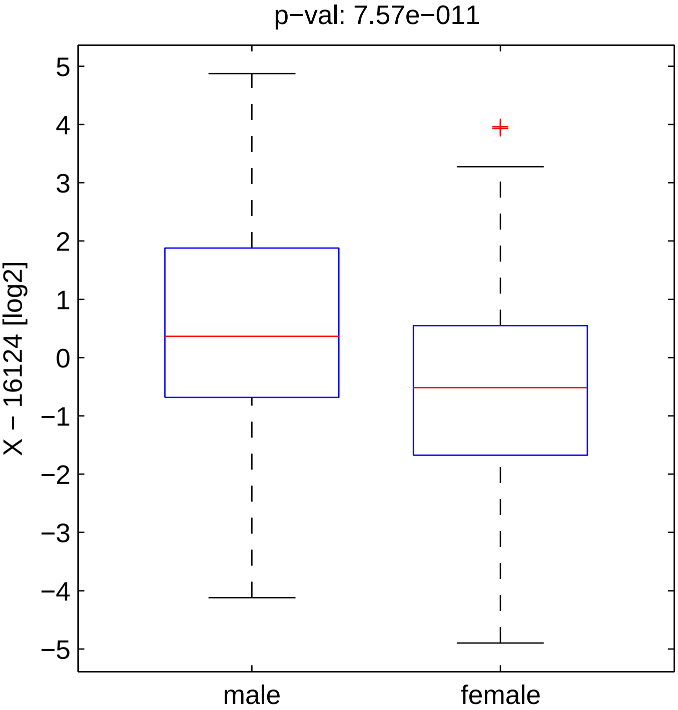
p−val: 7.57e−011
X − 16124 [log2]
5
4
3
2
1
0
−1
−2
−3
−4
−5
male
female

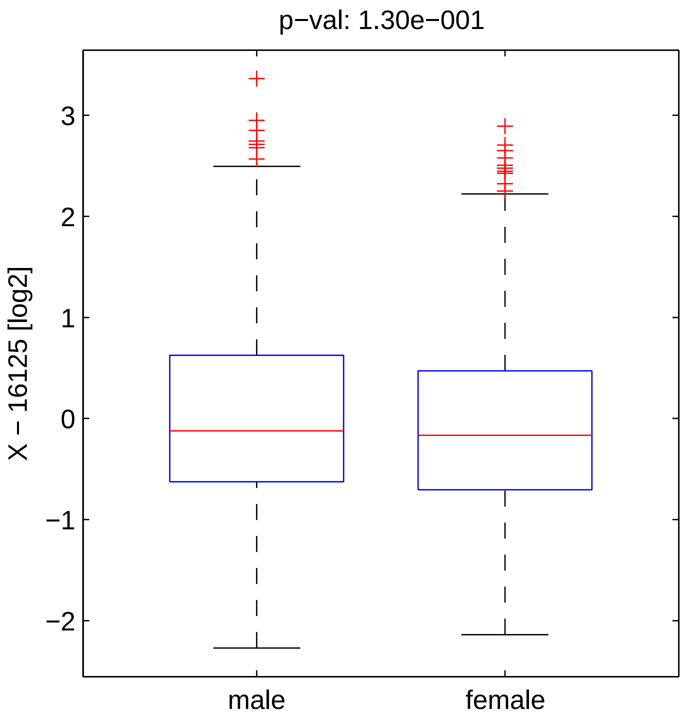

p−val: 1.30e−001
X − 16125 [log2]
3
2
1
0
−1
−2
male
female

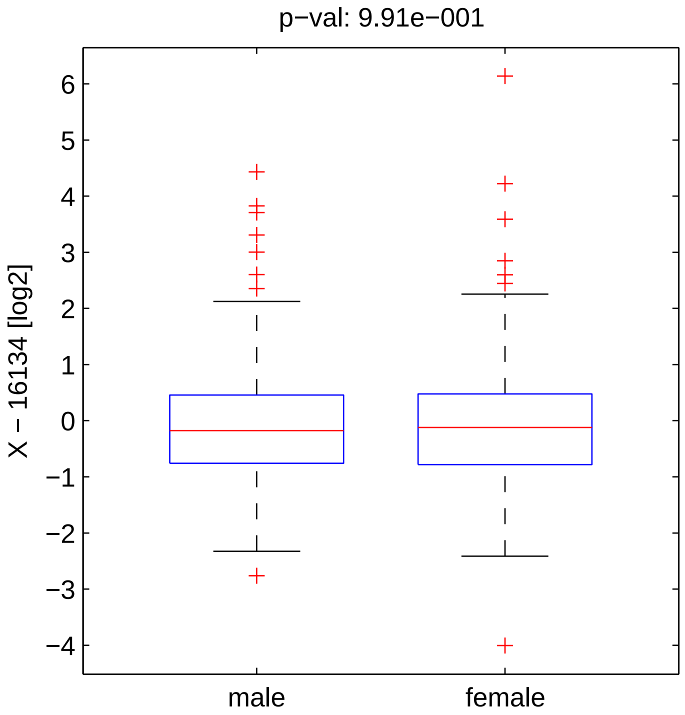
p−val: 9.91e−001
X − 16134 [log2]
6
5
4
3
2
1
0
−1
−2
−3
−4
male
female

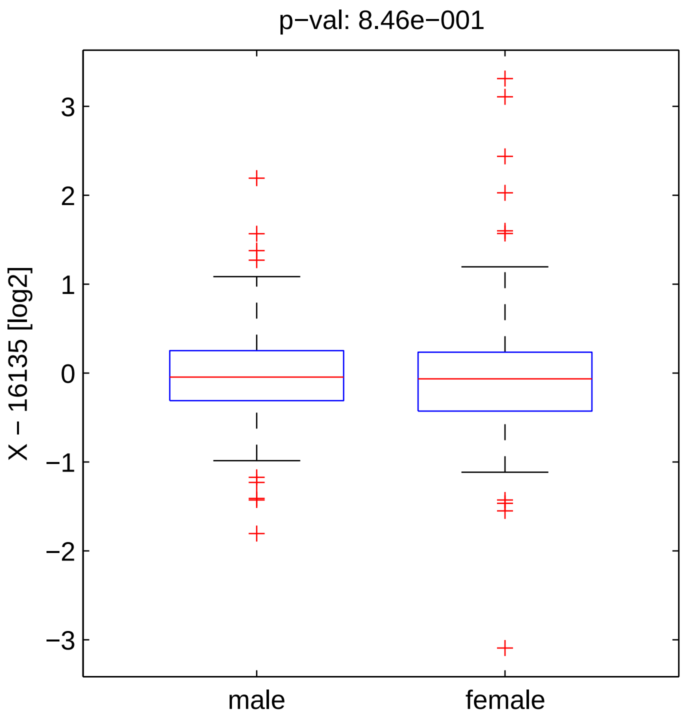
p−val: 8.46e−001
X − 16135 [log2]
3
2
1
0
−1
−2
−3
male
female

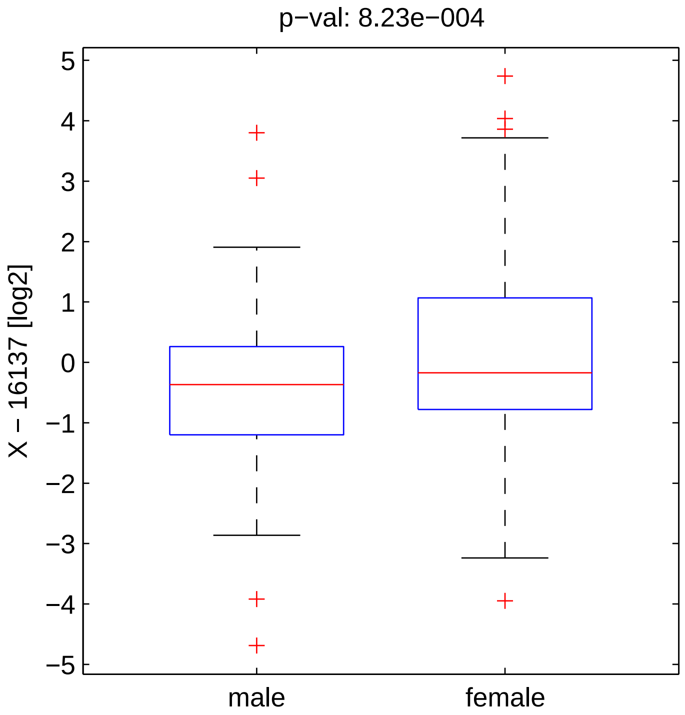

p−val: 8.23e−004
X − 16137 [log2]
5
4
3
2
1
0
−1
−2
−3
−4
−5
male
female

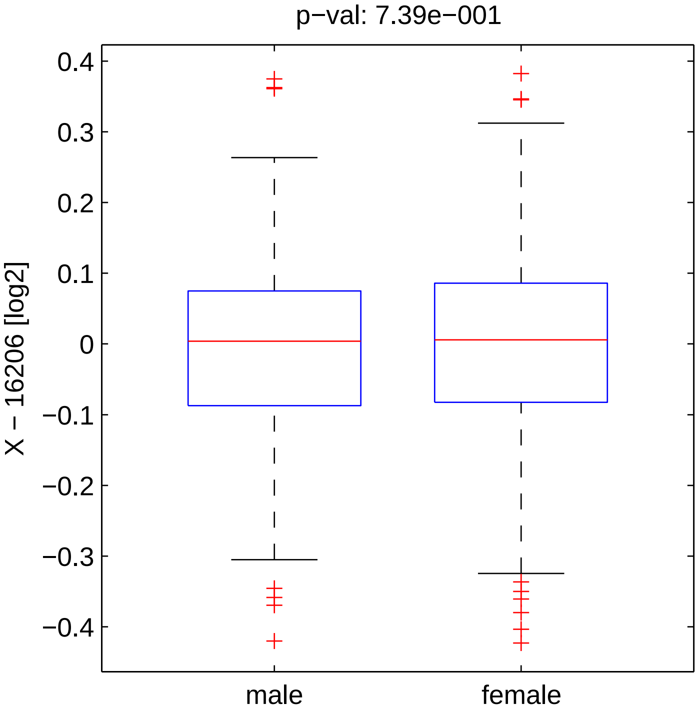

p−val: 7.39e−001
X − 16206 [log2]
0.4
0.3
0.2
0.1
0
−0.1
−0.2
−0.3
−0.4
male
female

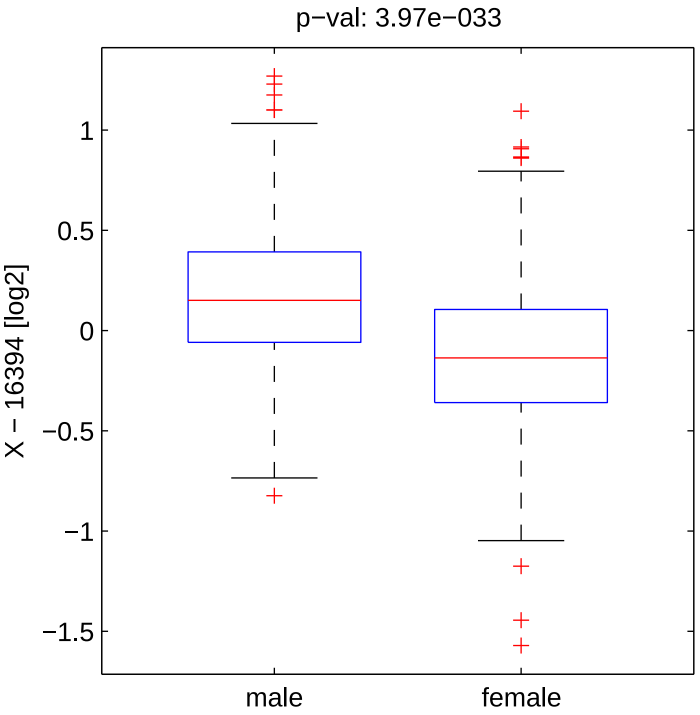

p−val: 3.97e−033
X − 16394 [log2]
1
0.5
0
−0.5
−1
−1.5
male
female

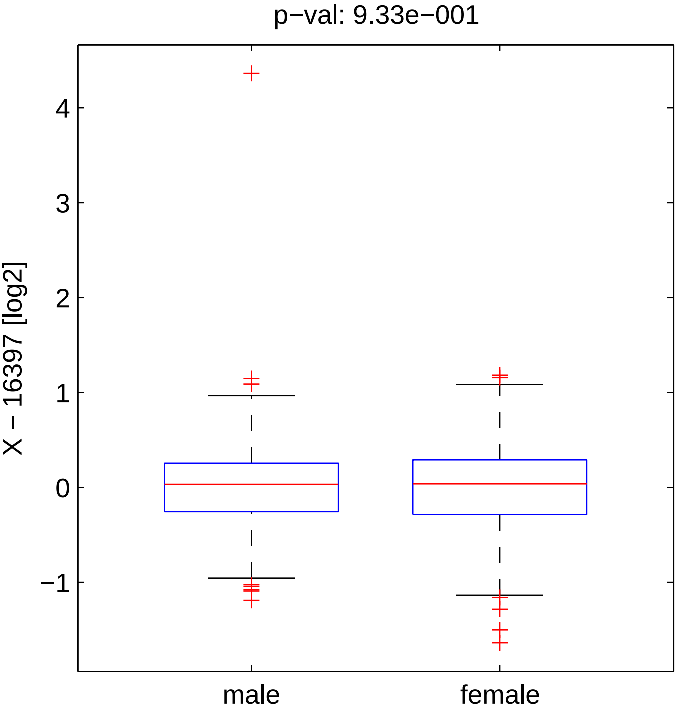
p−val: 9.33e−001
X − 16397 [log2]
4
3
2
1
0
−1
male
female

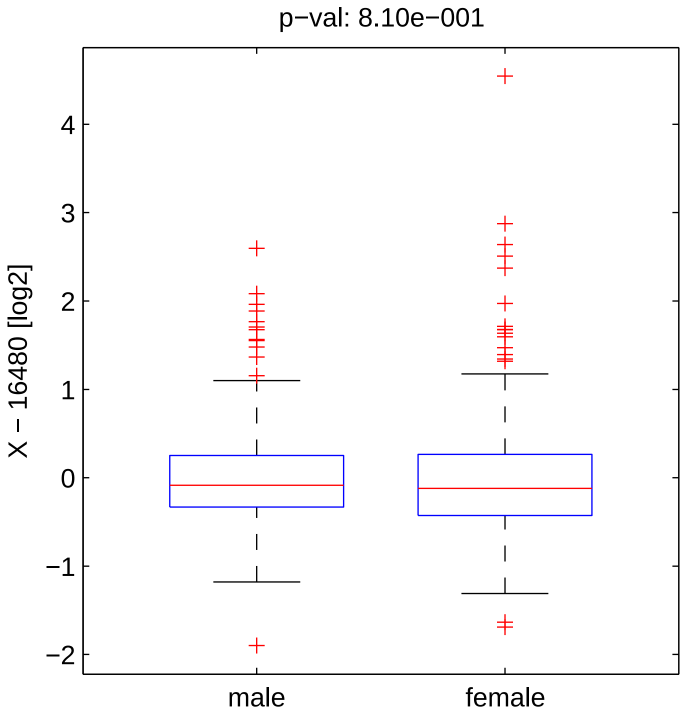
p−val: 8.10e−001
X − 16480 [log2]
4
3
2
1
0
−1
−2
male
female

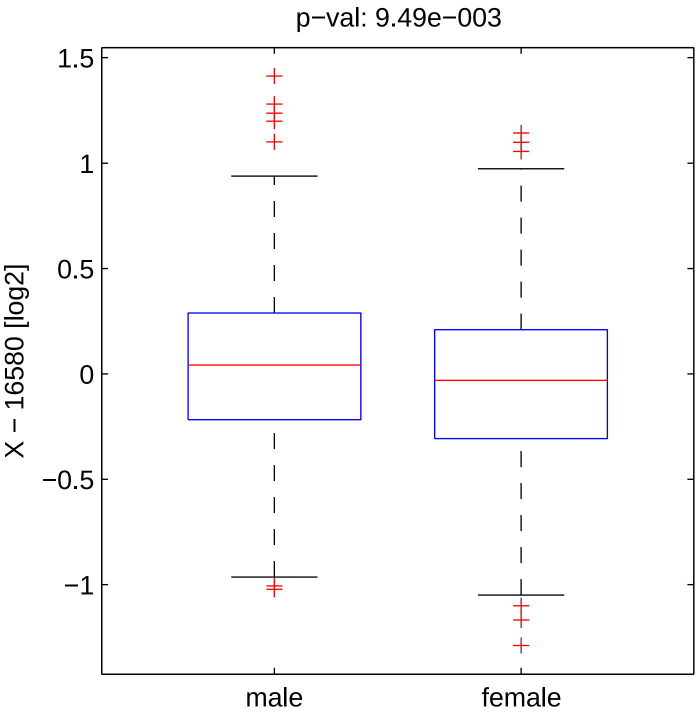

p−val: 9.49e−003
1.5
1
0.5
0
−0.5
−1
X − 16580 [log2]
male
female

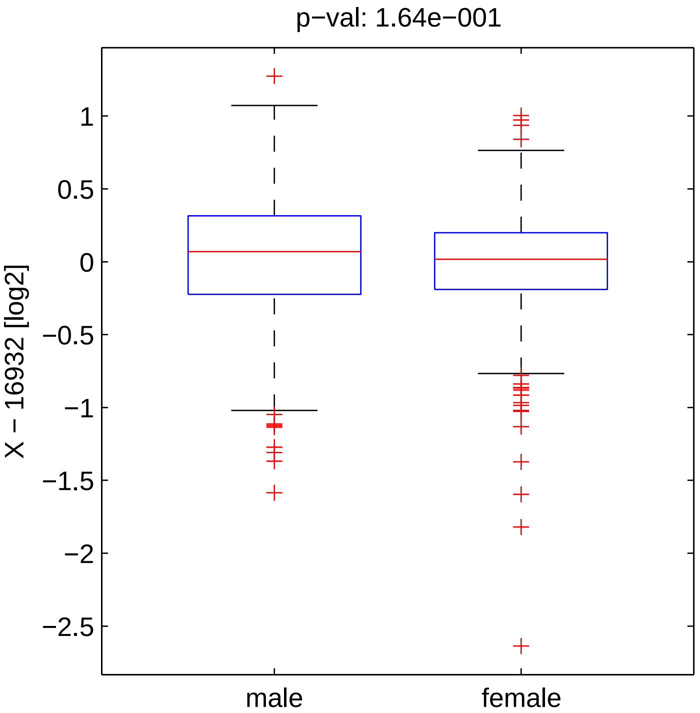

p−val: 1.64e−001
1
0.5
0
−0.5
−1
−1.5
−2
−2.5
X − 16932 [log2]
male
female

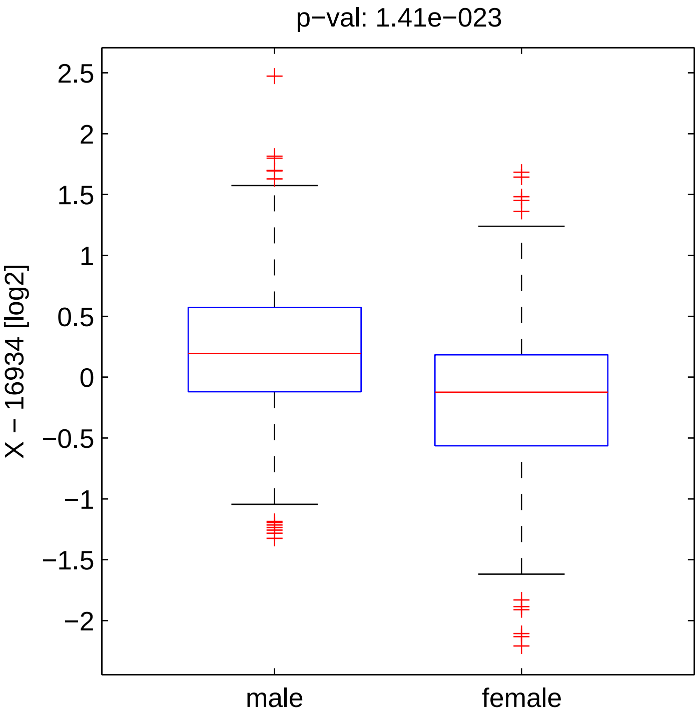
p−val: 1.41e−023
X − 16934 [log2]
2.5
2
1.5
1
0.5
0
−0.5
−1
−1.5
−2
male
female

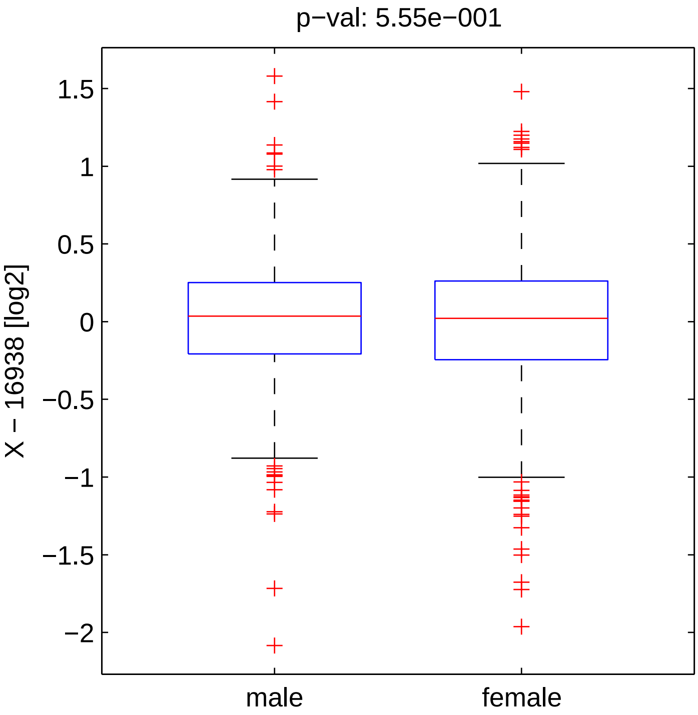
p−val: 5.55e−001
X − 16938 [log2]
1.5
1
0.5
0
−0.5
−1
−1.5
−2
male
female

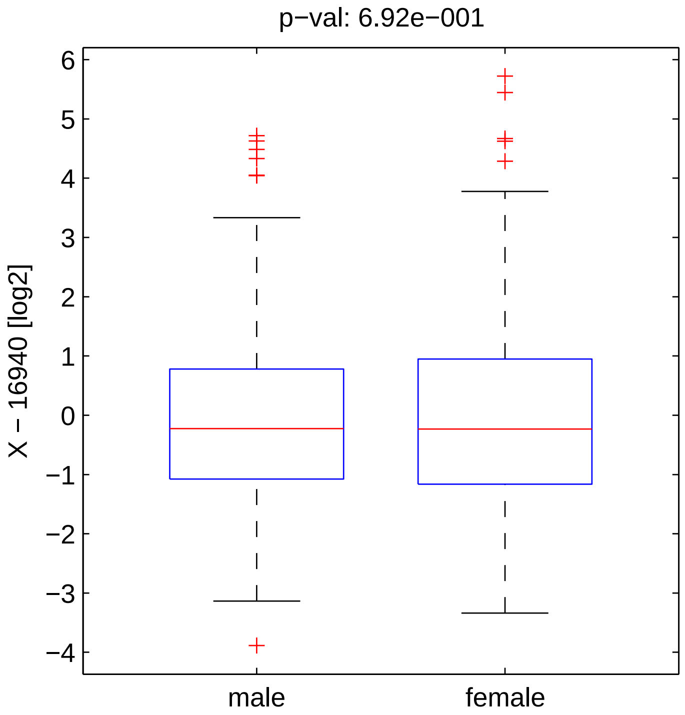

p−val: 6.92e−001
X − 16940 [log2]
6
5
4
3
2
1
0
−1
−2
−3
−4
male
female

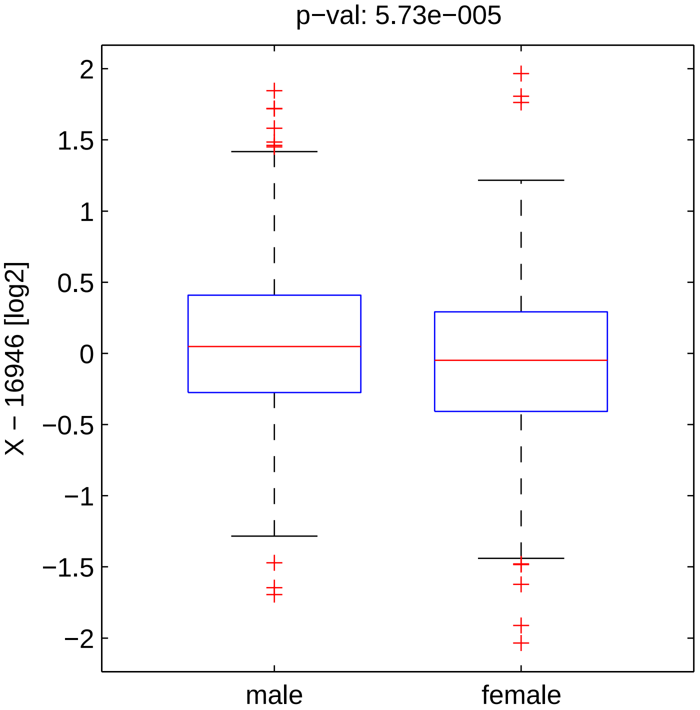

p−val: 5.73e−005
X − 16946 [log2]
2
1.5
1
0.5
0
−0.5
−1
−1.5
−2
male
female

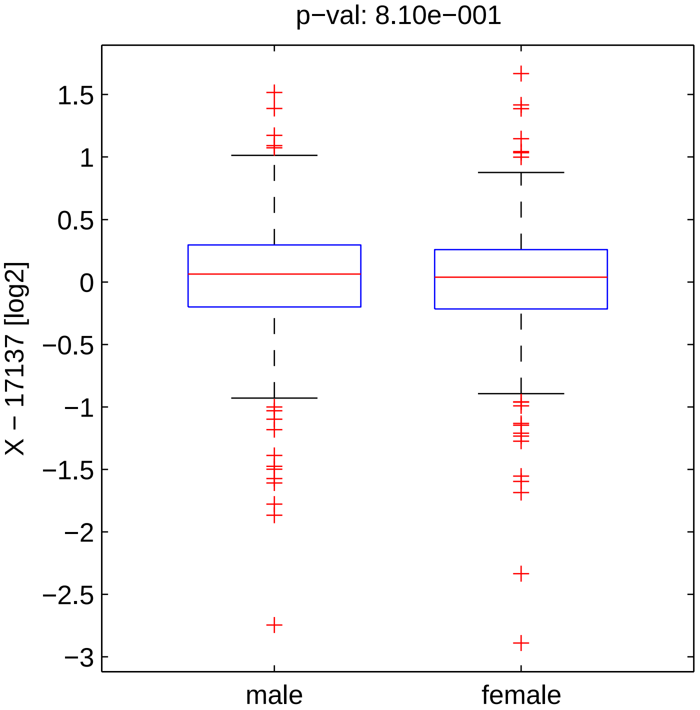

p−val: 8.10e−001
1.5
1
0.5
0
−0.5
−1
−1.5
−2
−2.5
−3
X − 17137 [log2]
male
female

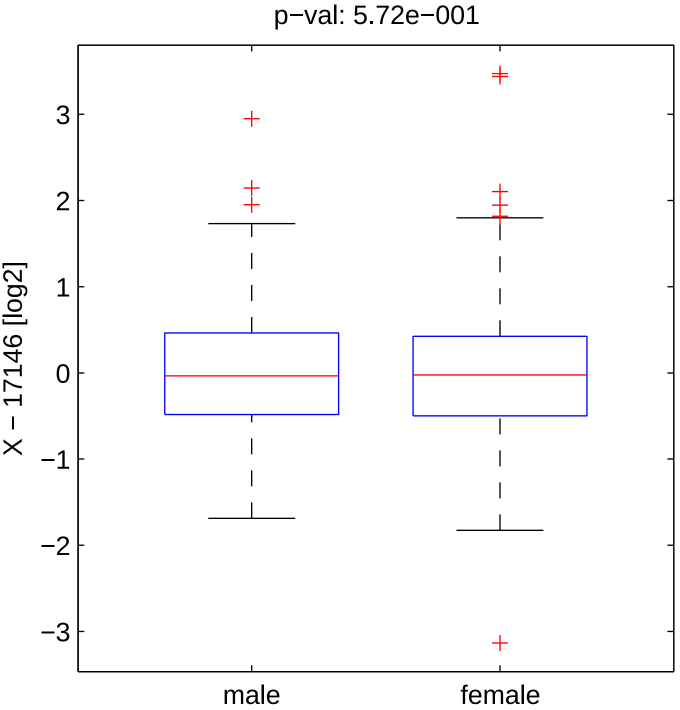

p−val: 5.72e−001
X − 17146 [log2]
3
2
1
0
−1
−2
−3
male
female

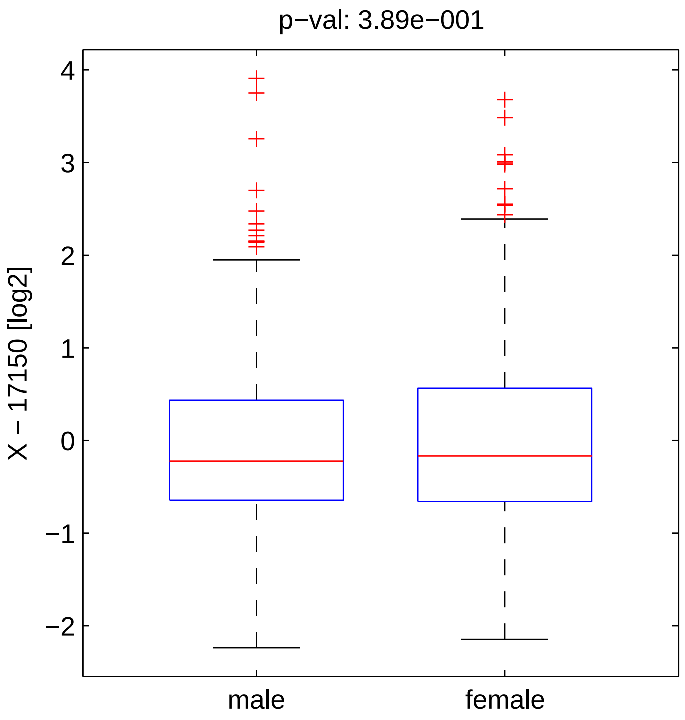

p−val: 3.89e−001
X − 17150 [log2]
4
3
2
1
0
−1
−2
male
female

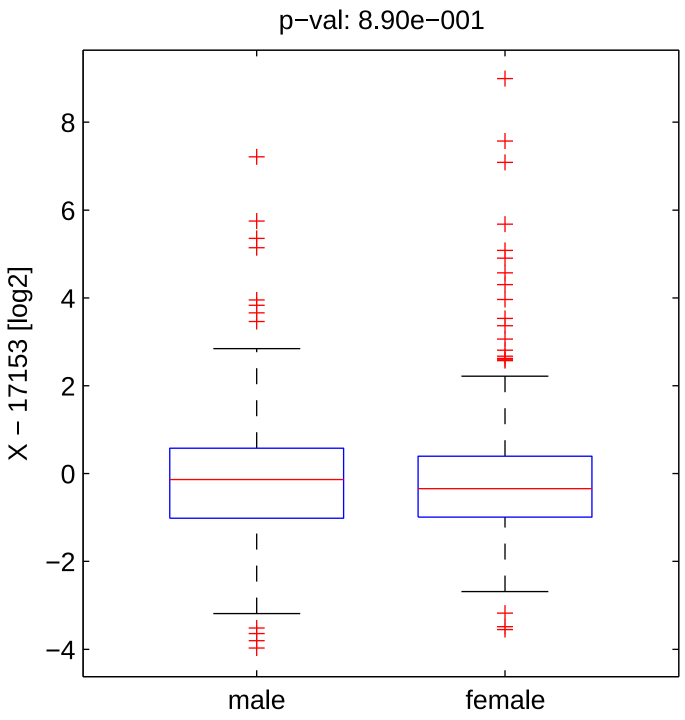

p−val: 8.90e−001
X − 17153 [log2]
8
6
4
2
0
−2
−4
male
female

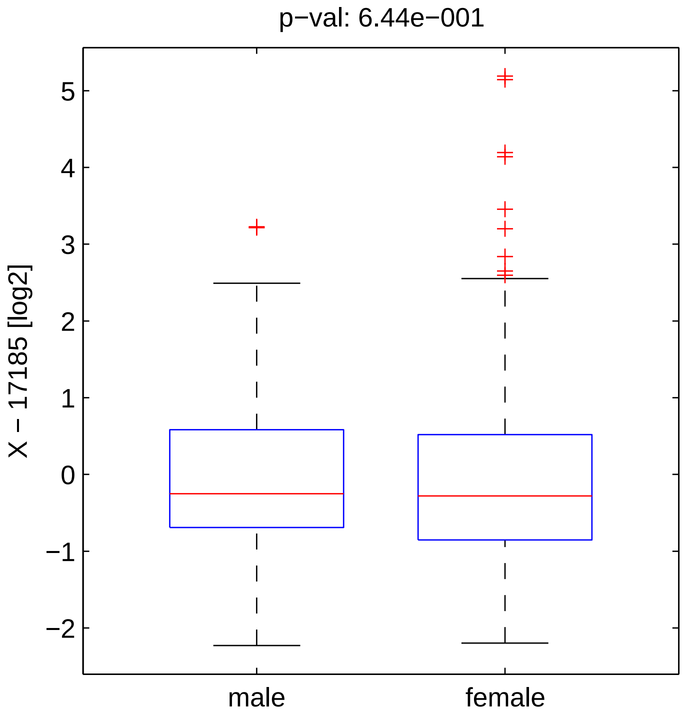

p−val: 6.44e−001
X − 17185 [log2]
5
4
3
2
1
0
−1
−2
male
female

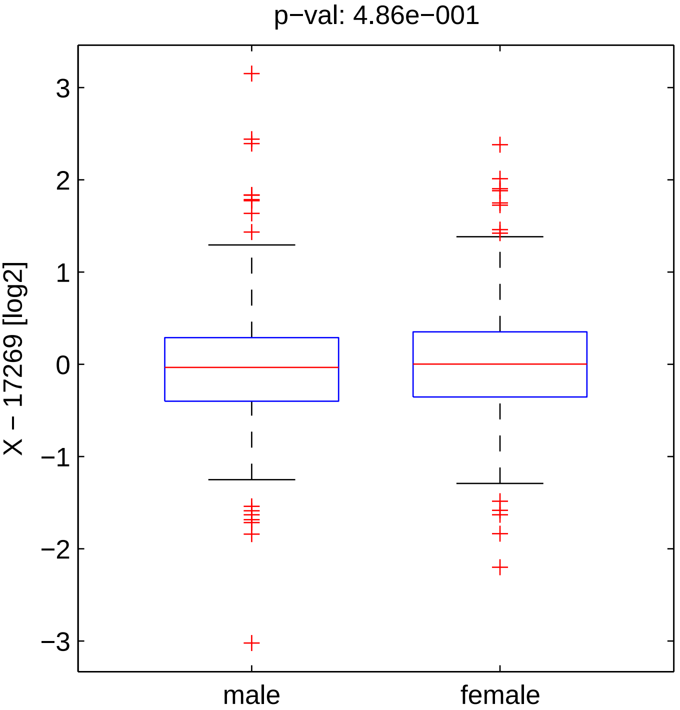
p−val: 4.86e−001
X − 17269 [log2]
3
2
1
0
−1
−2
−3
male
female

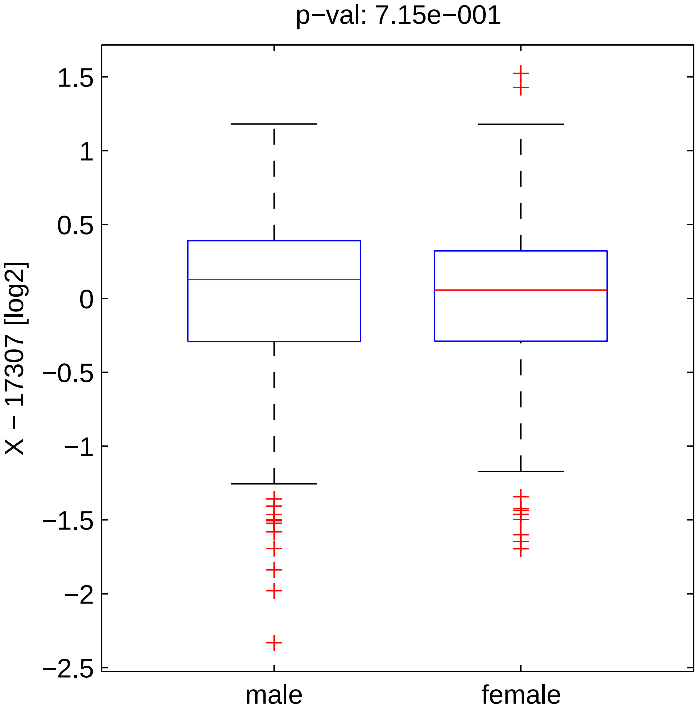
p−val: 7.15e−001
X − 17307 [log2]
1.5
1
0.5
0
−0.5
−1
−1.5
−2
−2.5
male
female

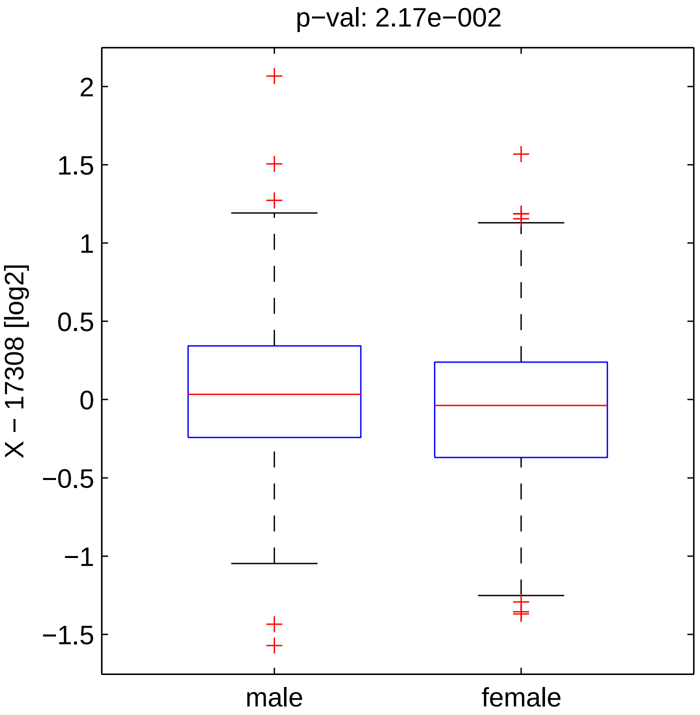
p−val: 2.17e−002
X − 17308 [log2]
2
1.5
1
0.5
0
−0.5
−1
−1.5
male
female

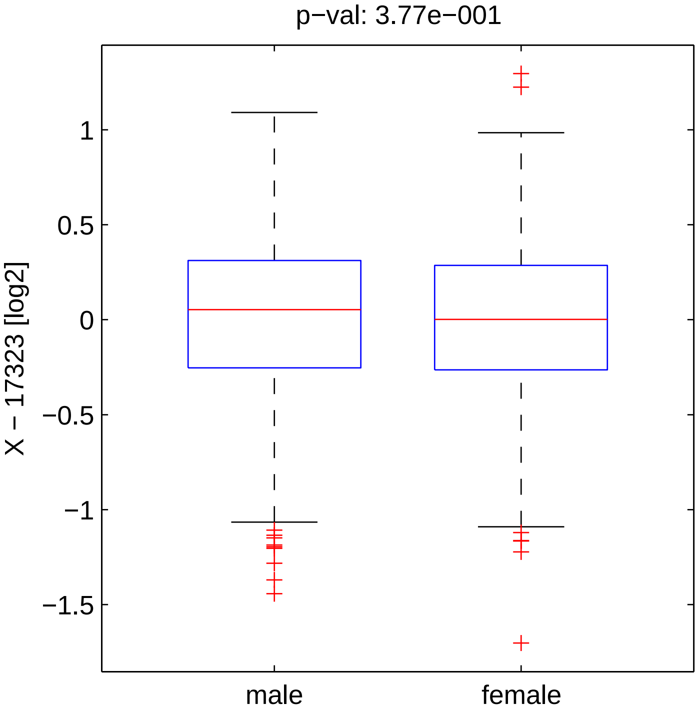

p−val: 3.77e−001
X − 17323 [log2]
1
0.5
0
−0.5
−1
−1.5
male
female

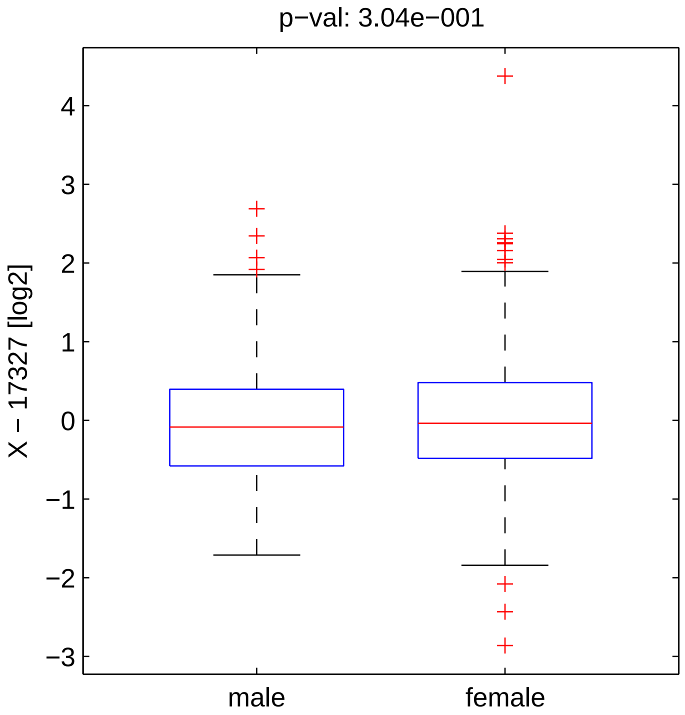
p−val: 3.04e−001
X − 17327 [log2]
4
3
2
1
0
−1
−2
−3
male
female

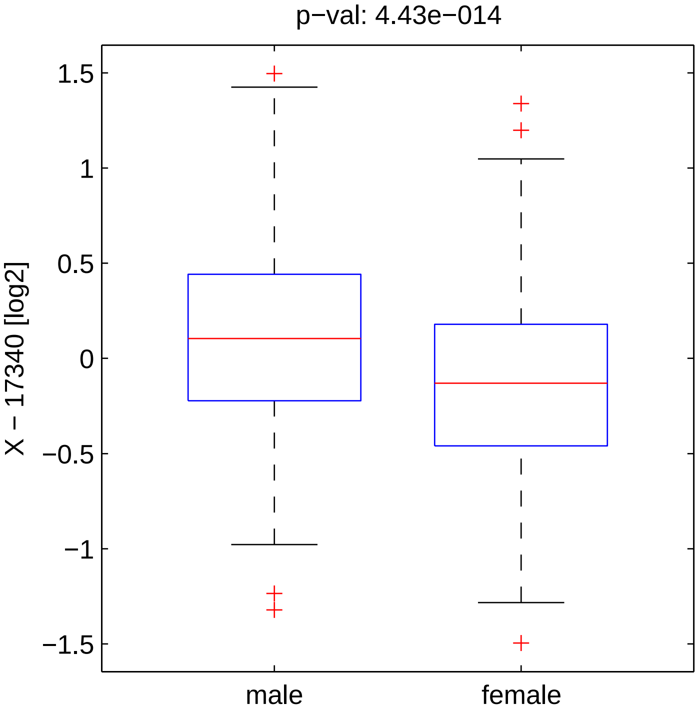

p−val: 4.43e−014
X − 17340 [log2]
1.5
1
0.5
0
−0.5
−1
−1.5
male
female

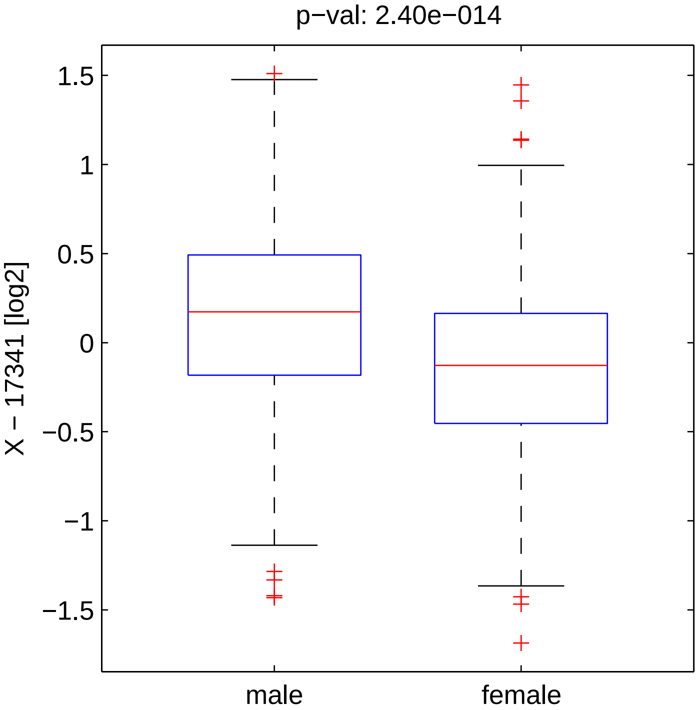
p−val: 2.40e−014
X − 17341 [log2]
1.5
1
0.5
0
−0.5
−1
−1.5
male
female

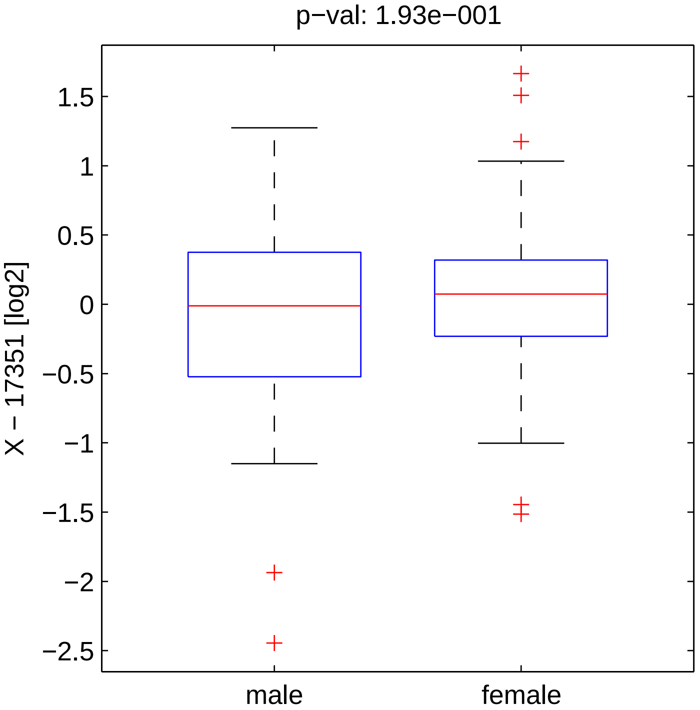
p−val: 1.93e−001
1.5
1
0.5
0
−0.5
−1
−1.5
−2
−2.5
X − 17351 [log2]
male
female

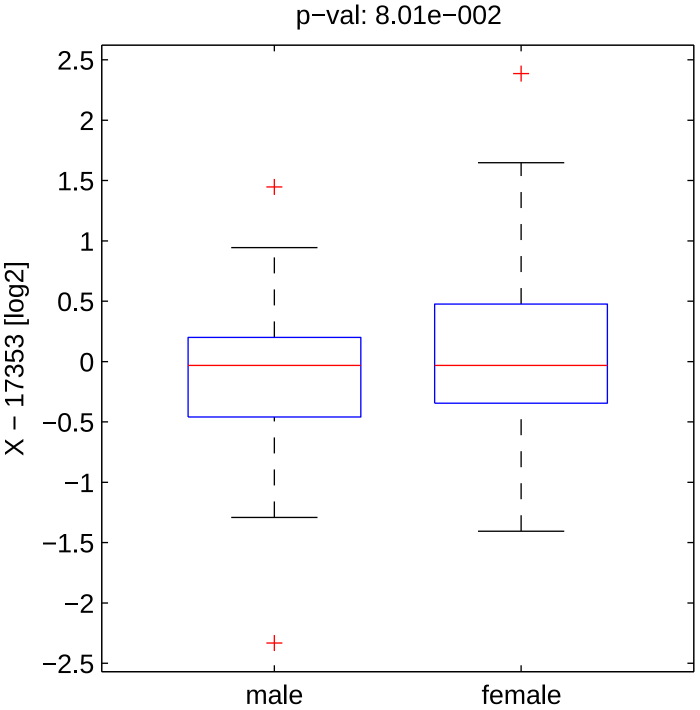
p−val: 8.01e−002
X − 17353 [log2]
2.5
2
1.5
1
0.5
0
−0.5
−1
−1.5
−2
−2.5
male
female

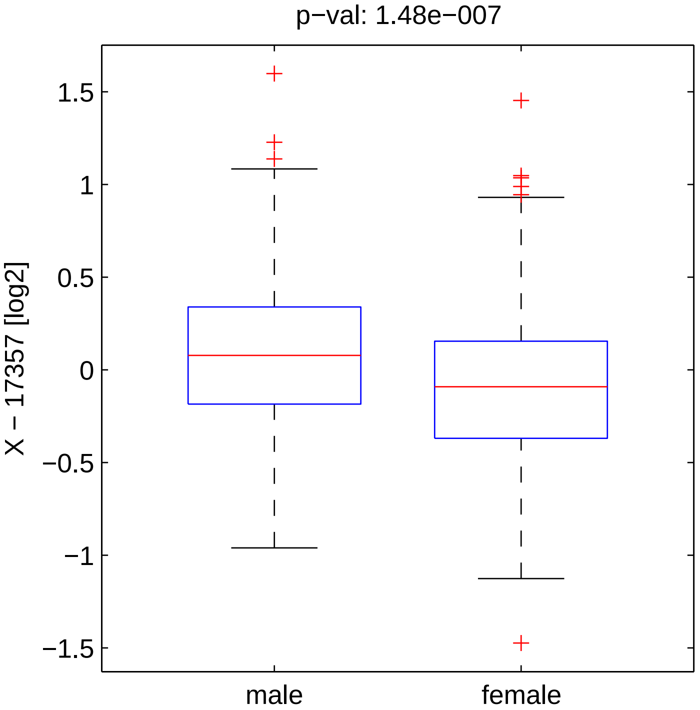

p−val: 1.48e−007
X − 17357 [log2]
1.5
1
0.5
0
−0.5
−1
−1.5
male
female

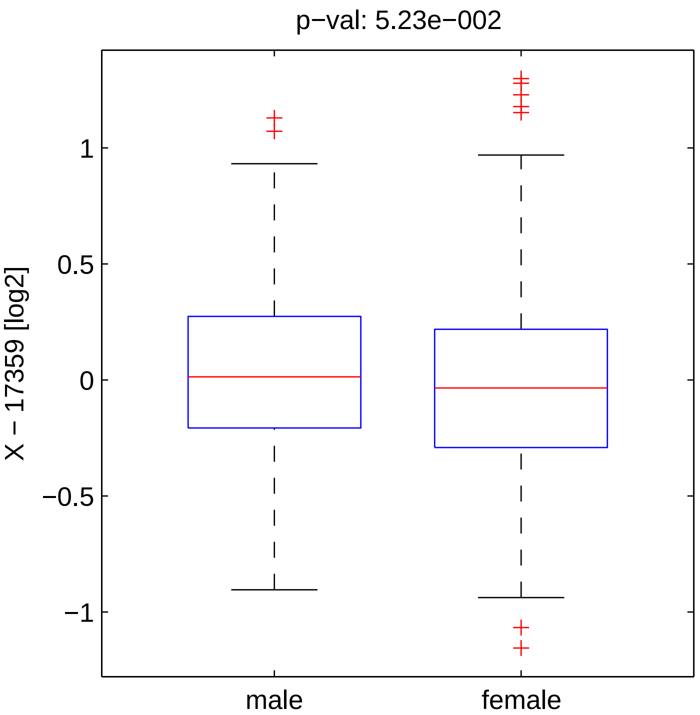
p−val: 5.23e−002
X − 17359 [log2]
1
0.5
0
−0.5
−1
male
female

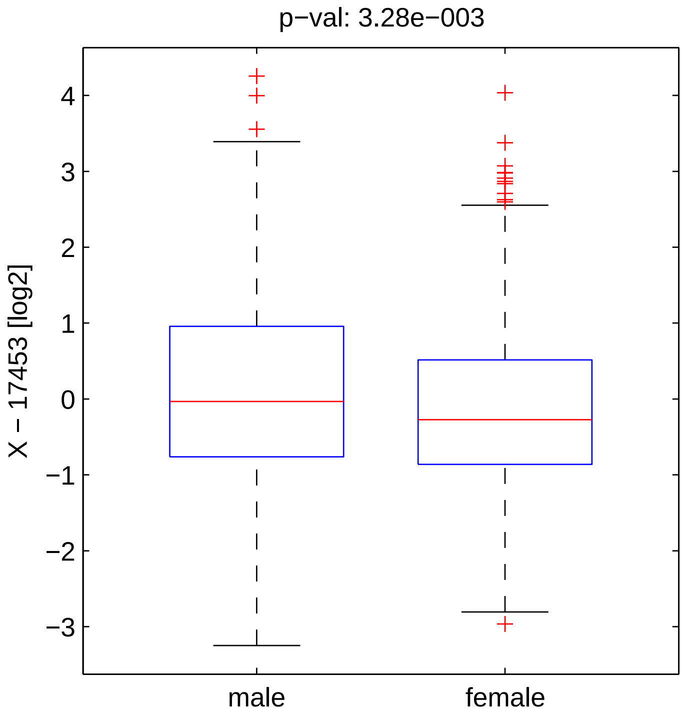

p−val: 3.28e−003
X − 17453 [log2]
4
3
2
1
0
−1
−2
−3
male
female

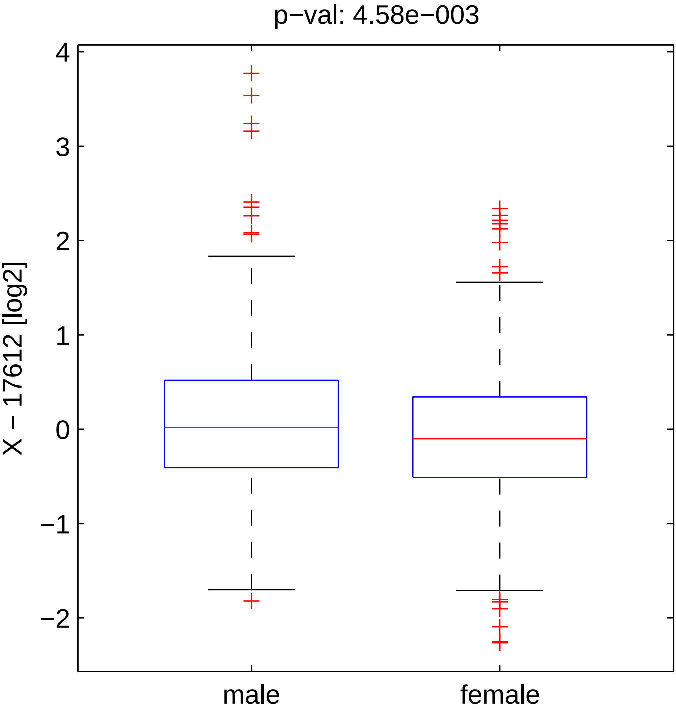

p−val: 4.58e−003
X − 17612 [log2]
4
3
2
1
0
−1
−2
male
female

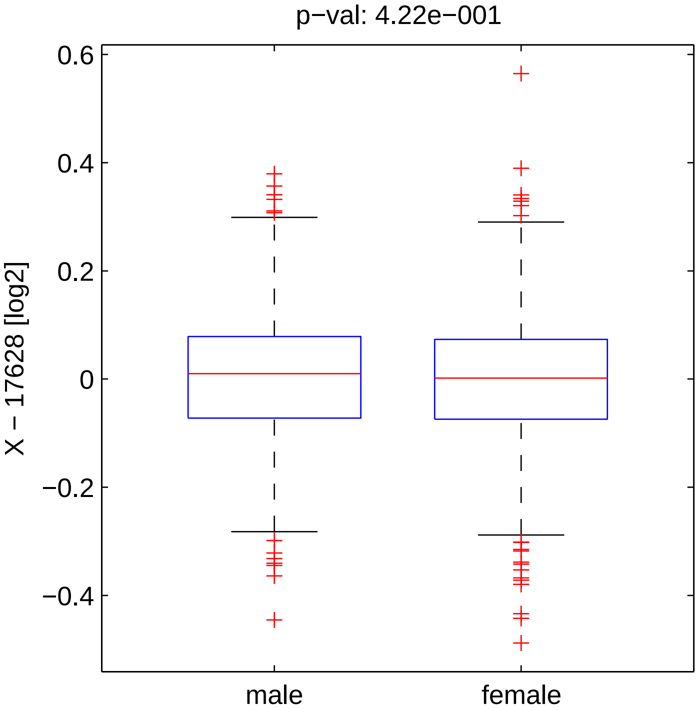

p−val: 4.22e−001
0.6
0.4
0.2
0
−0.2
−0.4
X − 17628 [log2]
male
female

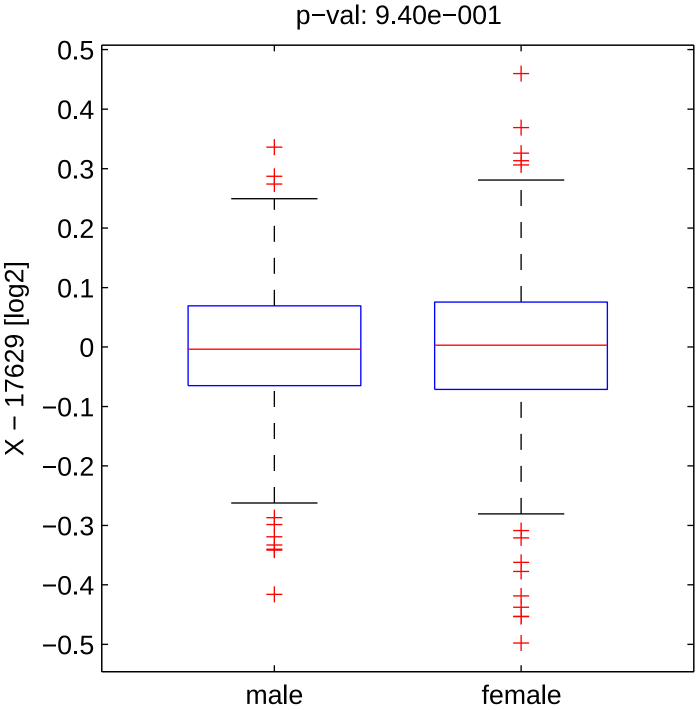

p−val: 9.40e−001
X − 17629 [log2]
0.5
0.4
0.3
0.2
0.1
0
−0.1
−0.2
−0.3
−0.4
−0.5
male
female

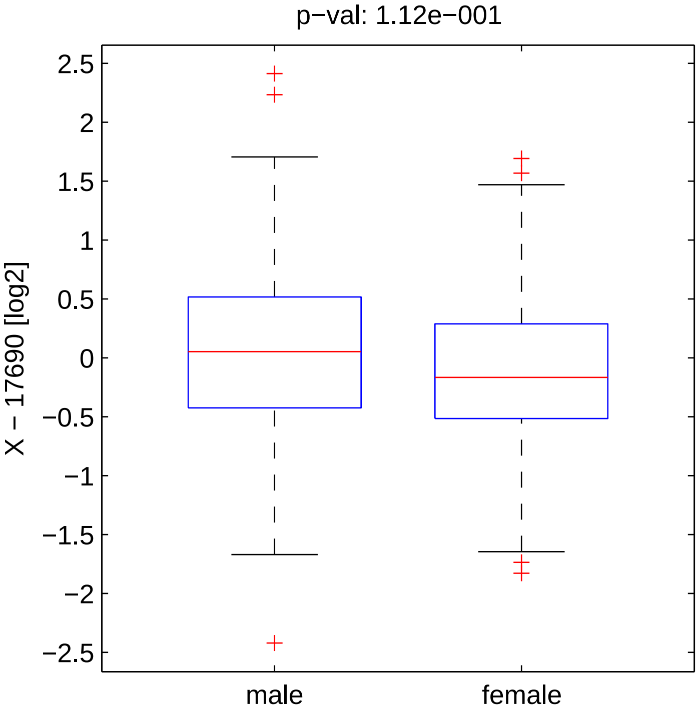

p−val: 1.12e−001
X − 17690 [log2]
2.5
2
1.5
1
0.5
0
−0.5
−1
−1.5
−2
−2.5
male
female

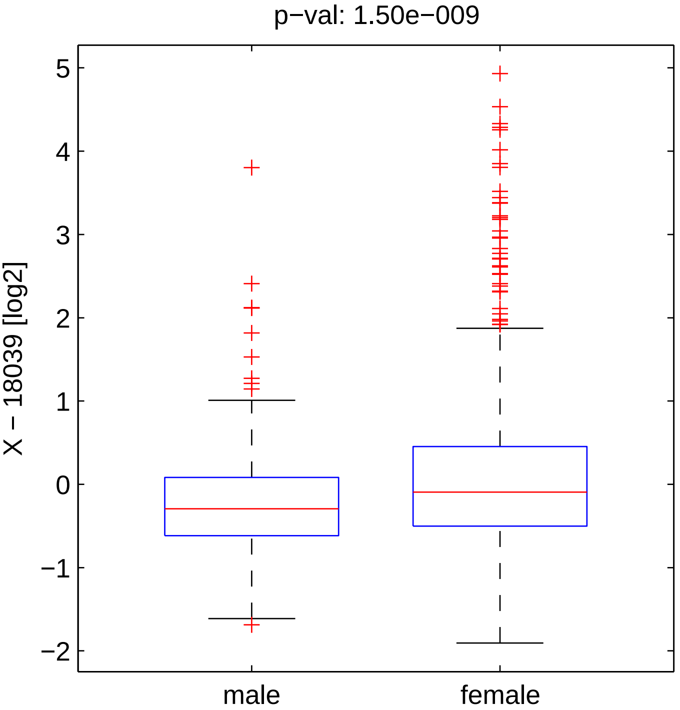

p−val: 1.50e−009
X − 18039 [log2]
5
4
3
2
1
0
−1
−2
male
female

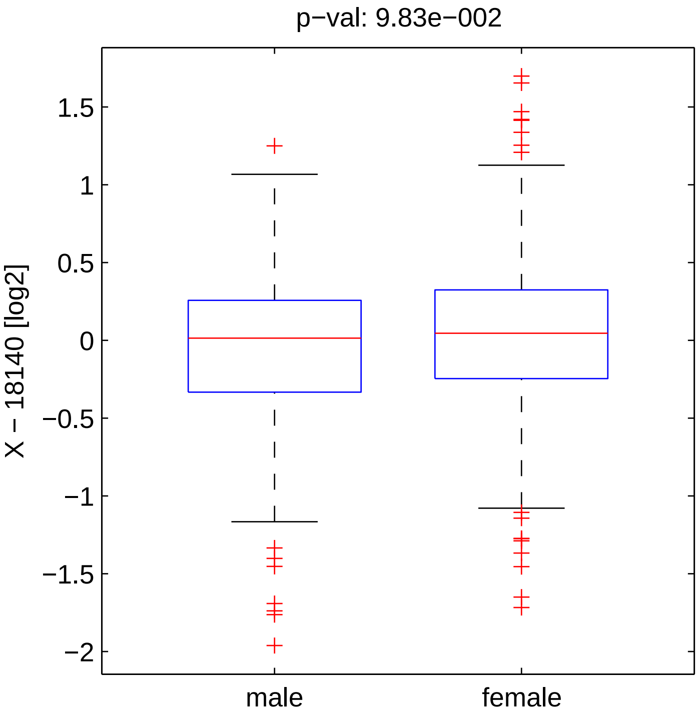
p−val: 9.83e−002
X − 18140 [log2]
1.5
1
0.5
0
−0.5
−1
−1.5
−2
male
female

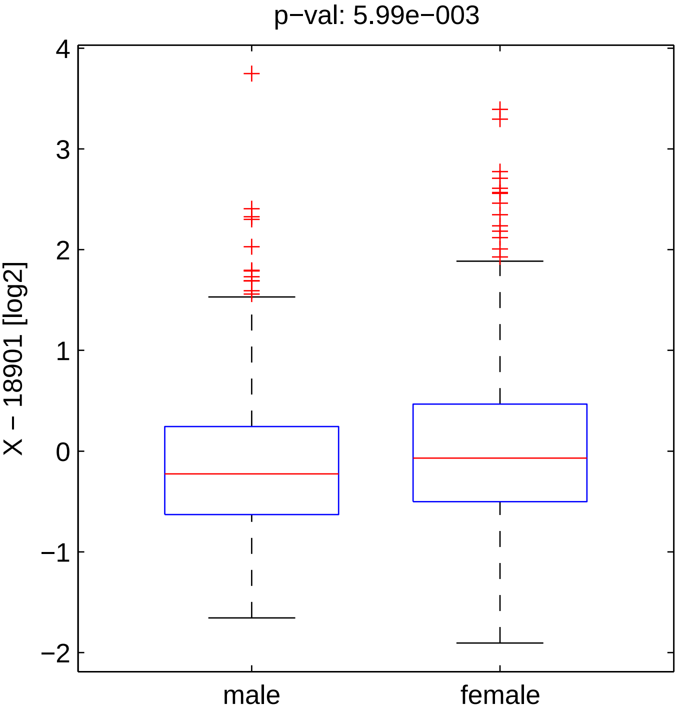

p−val: 5.99e−003
X − 18901 [log2]
4
3
2
1
0
−1
−2
male
female

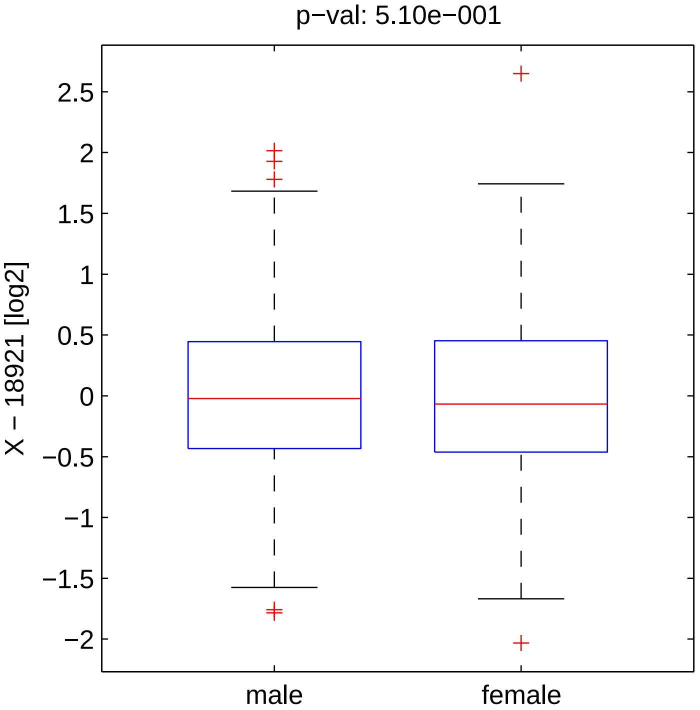
p−val: 5.10e−001
X − 18921 [log2]
2.5
2
1.5
1
0.5
0
−0.5
−1
−1.5
−2
male
female

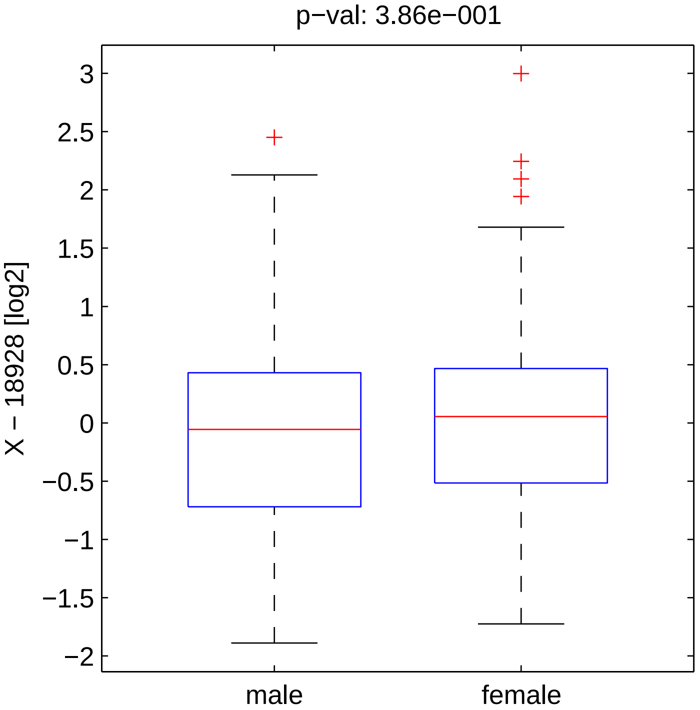

p−val: 3.86e−001
X − 18928 [log2]
3
2.5
2
1.5
1
0.5
0
−0.5
−1
−1.5
−2
male
female

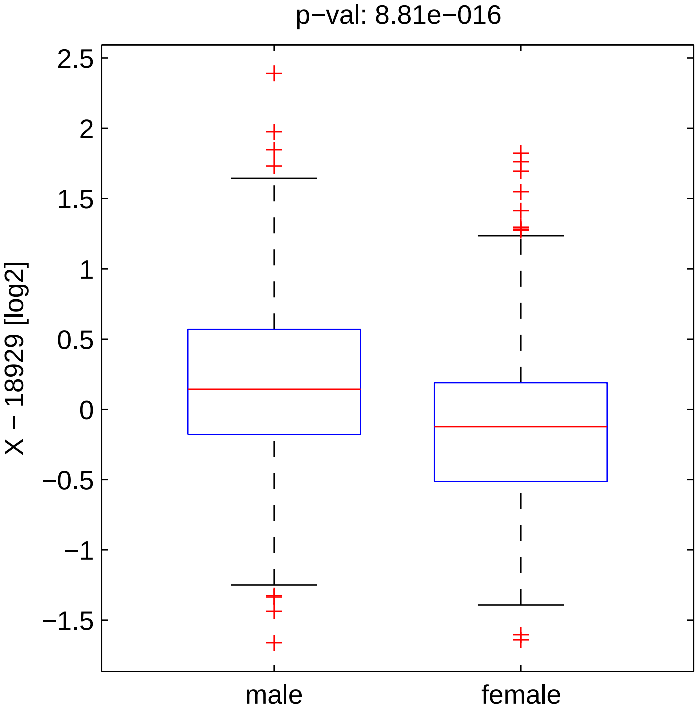
p−val: 8.81e−016
X − 18929 [log2]
2.5
2
1.5
1
0.5
0
−0.5
−1
−1.5
male
female

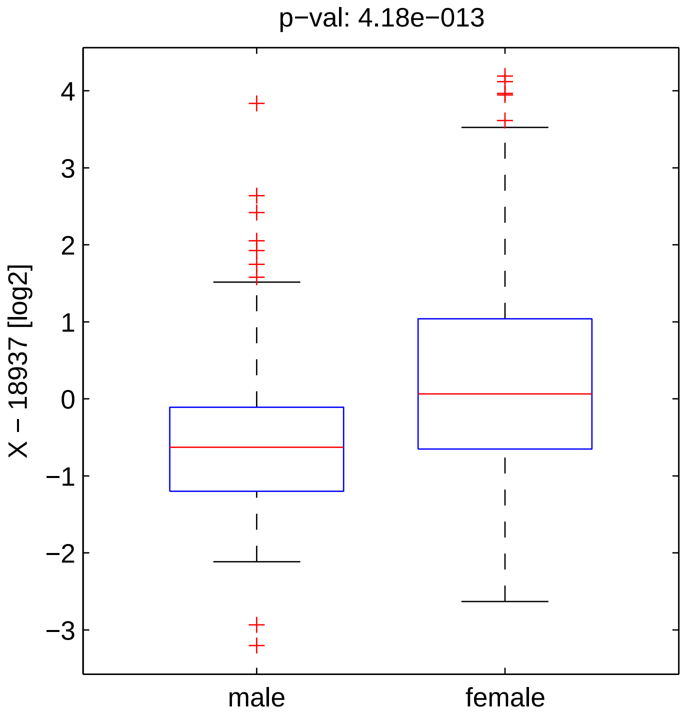

p−val: 4.18e−013
X − 18937 [log2]
4
3
2
1
0
−1
−2
−3
male
female

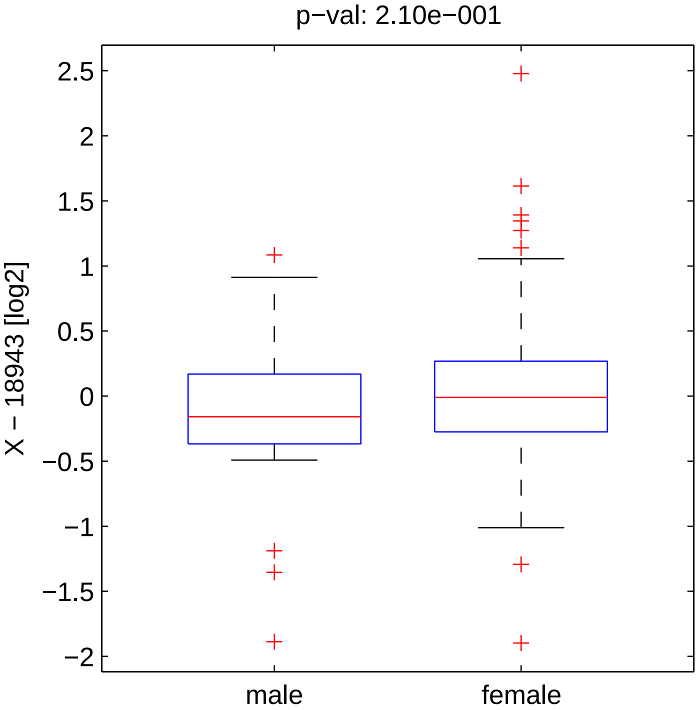
p−val: 2.10e−001
X − 18943 [log2]
2.5
2
1.5
1
0.5
0
−0.5
−1
−1.5
−2
male
female

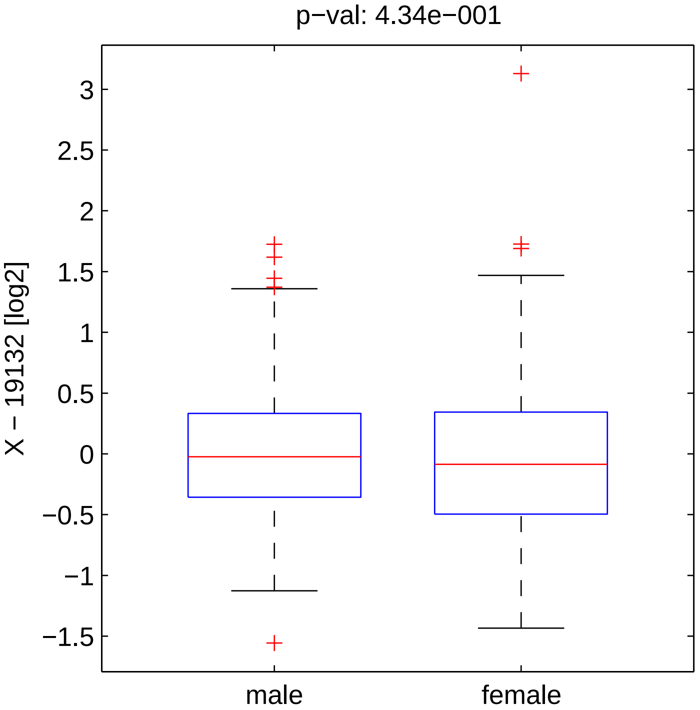
p−val: 4.34e−001
X − 19132 [log2]
3
2.5
2
1.5
1
0.5
0
−0.5
−1
−1.5
male
female

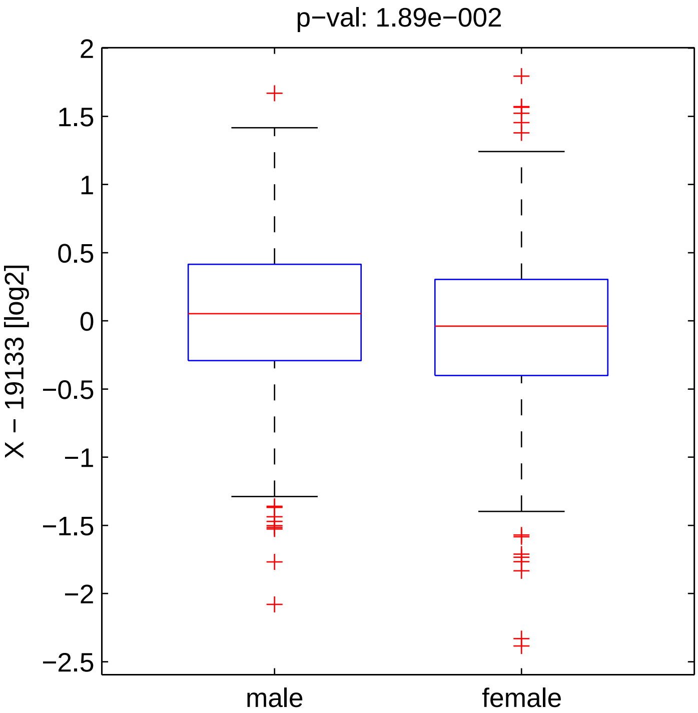
p−val: 1.89e−002
X − 19133 [log2]
2
1.5
1
0.5
0
−0.5
−1
−1.5
−2
−2.5
male
female

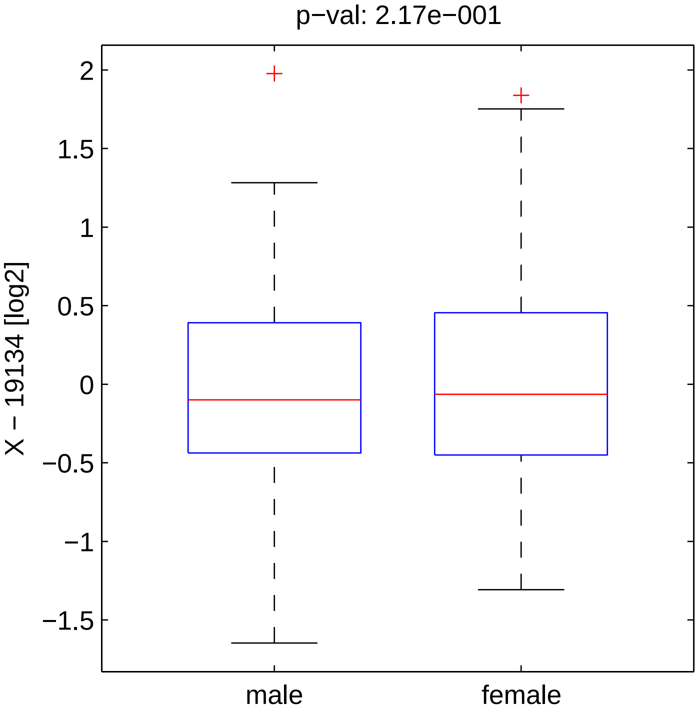

p−val: 2.17e−001
X − 19134 [log2]
2
1.5
1
0.5
0
−0.5
−1
−1.5
male
female

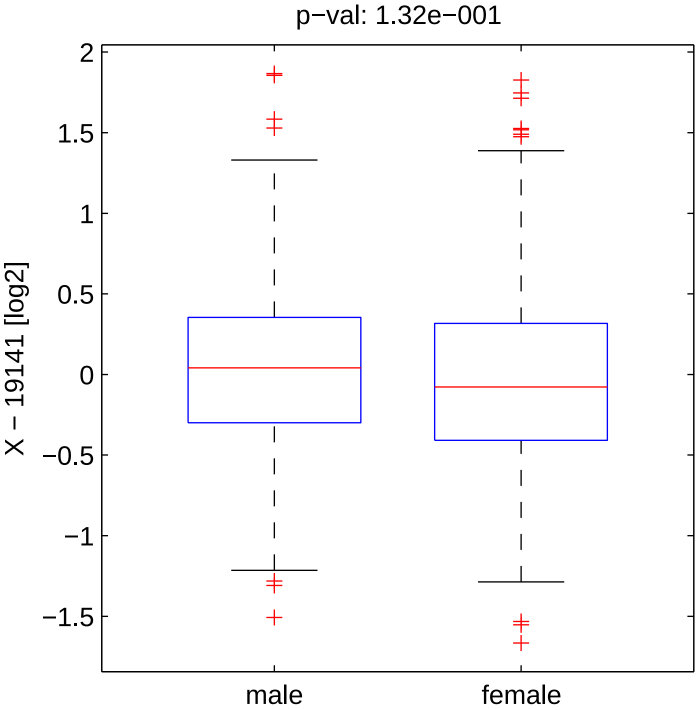

p−val: 1.32e−001
X − 19141 [log2]
2
1.5
1
0.5
0
−0.5
−1
−1.5
male
female

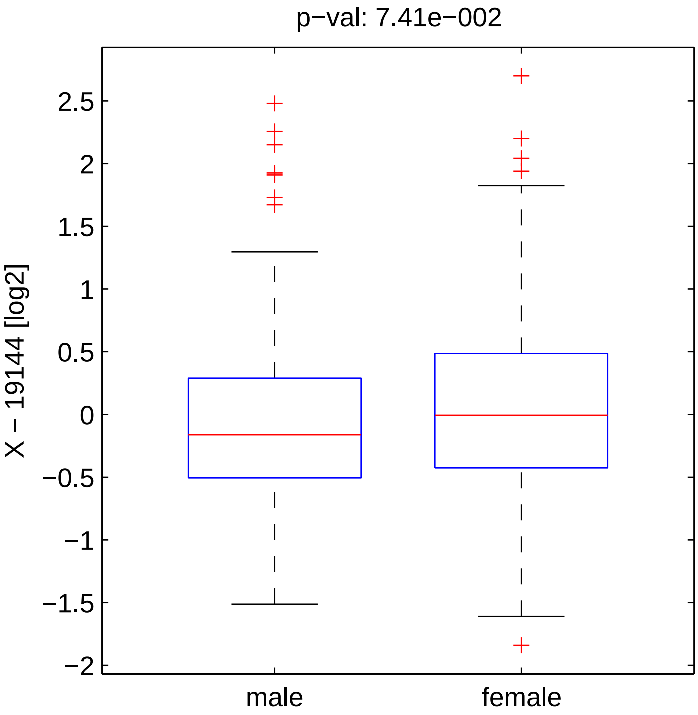

p−val: 7.41e−002
X − 19144 [log2]
2.5
2
1.5
1
0.5
0
−0.5
−1
−1.5
−2
male
female

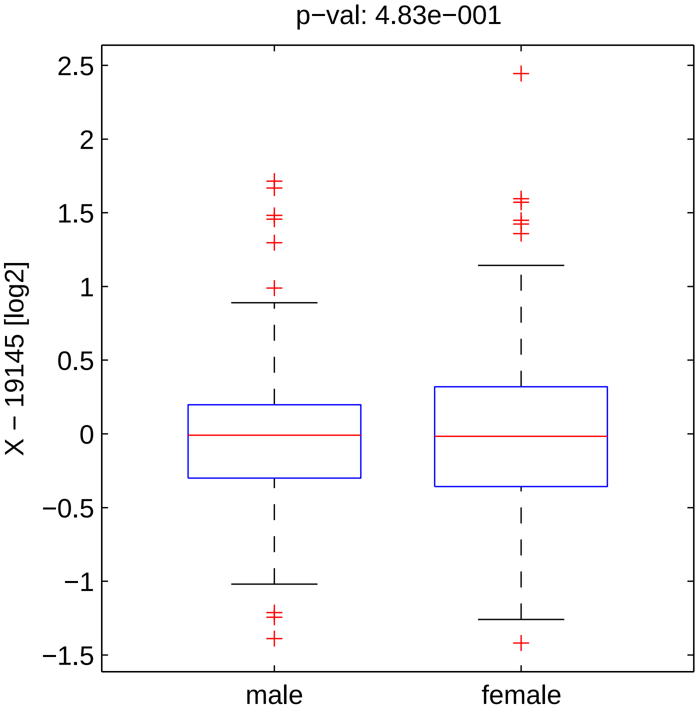
p−val: 4.83e−001
X − 19145 [log2]
2.5
2
1.5
1
0.5
0
−0.5
−1
−1.5
male
female

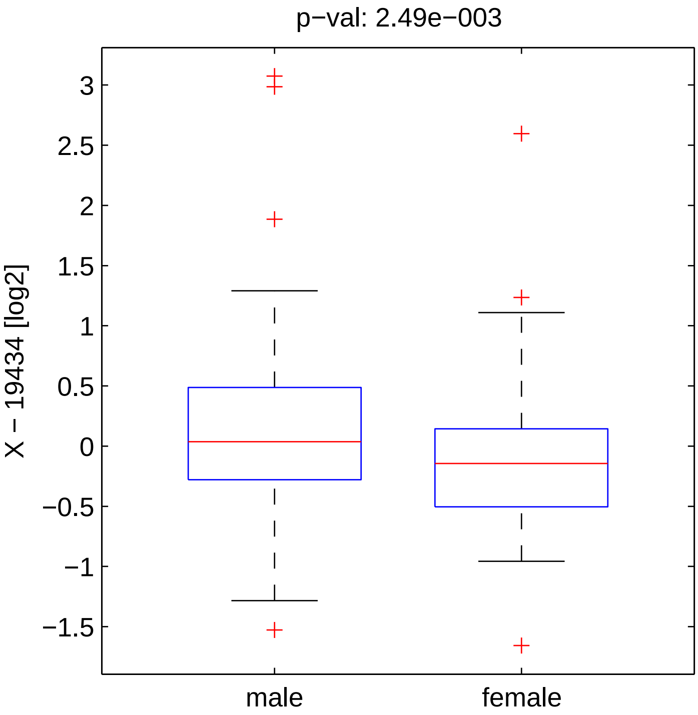

p−val: 2.49e−003
X − 19434 [log2]
3
2.5
2
1.5
1
0.5
0
−0.5
−1
−1.5
male
female

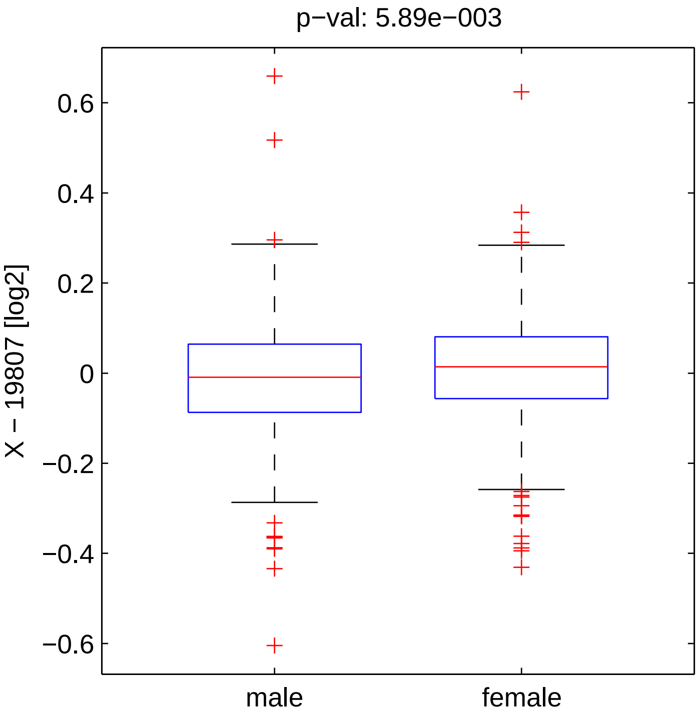

p−val: 5.89e−003
X − 19807 [log2]
0.6
0.4
0.2
0
−0.2
−0.4
−0.6
male
female

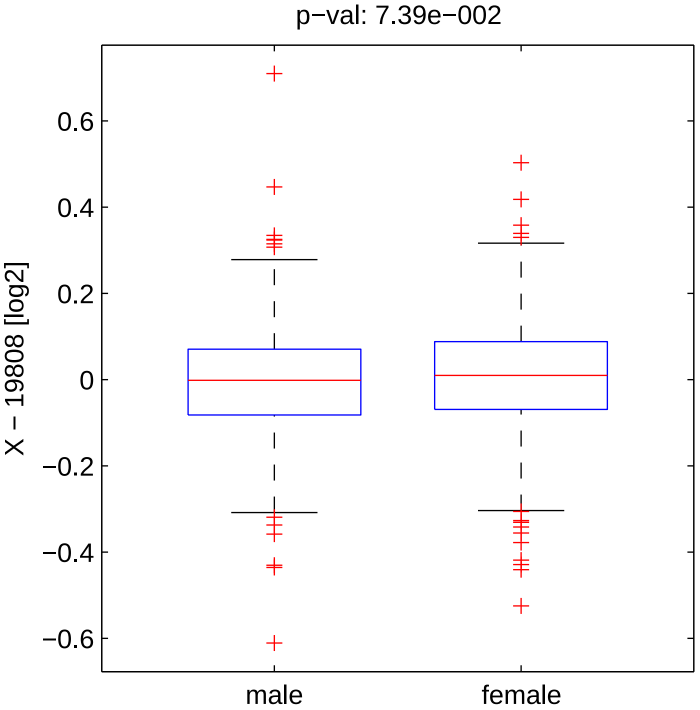

p−val: 7.39e−002
X − 19808 [log2]
0.6
0.4
0.2
0
−0.2
−0.4
−0.6
male
female

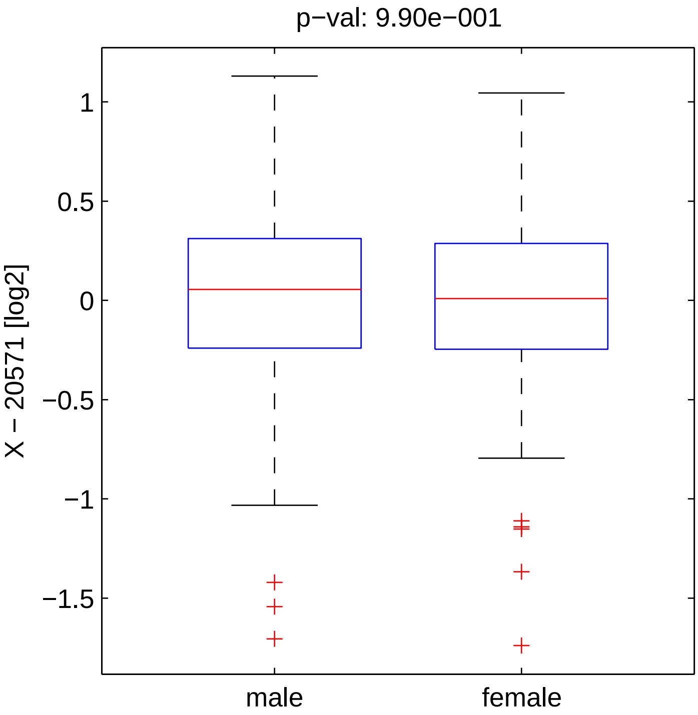
p−val: 9.90e−001
X − 20571 [log2]
1
0.5
0
−0.5
−1
−1.5
male
female

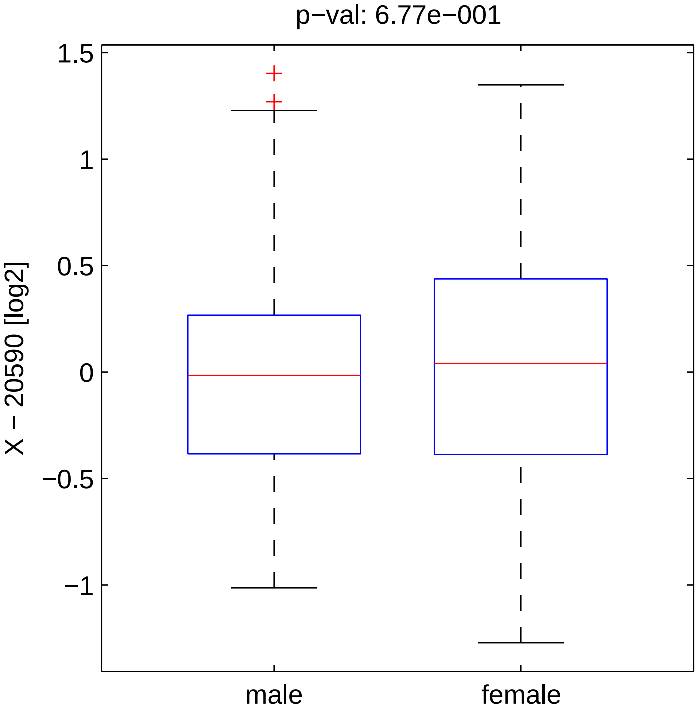

p−val: 6.77e−001
1.5
1
0.5
0
−0.5
−1
X − 20590 [log2]
male
female

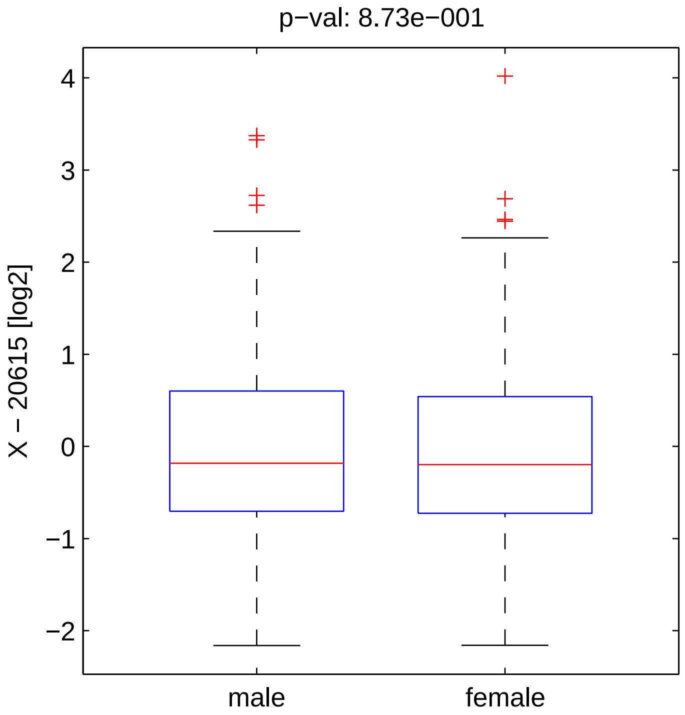
p−val: 8.73e−001
X − 20615 [log2]
4
3
2
1
0
−1
−2
male
female

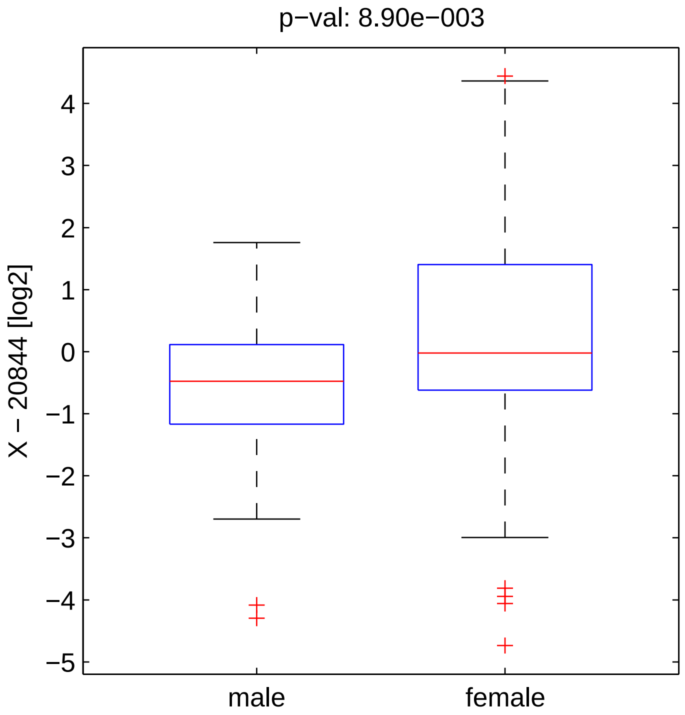
p−val: 8.90e−003
X − 20844 [log2]
4
3
2
1
0
−1
−2
−3
−4
−5
male
female

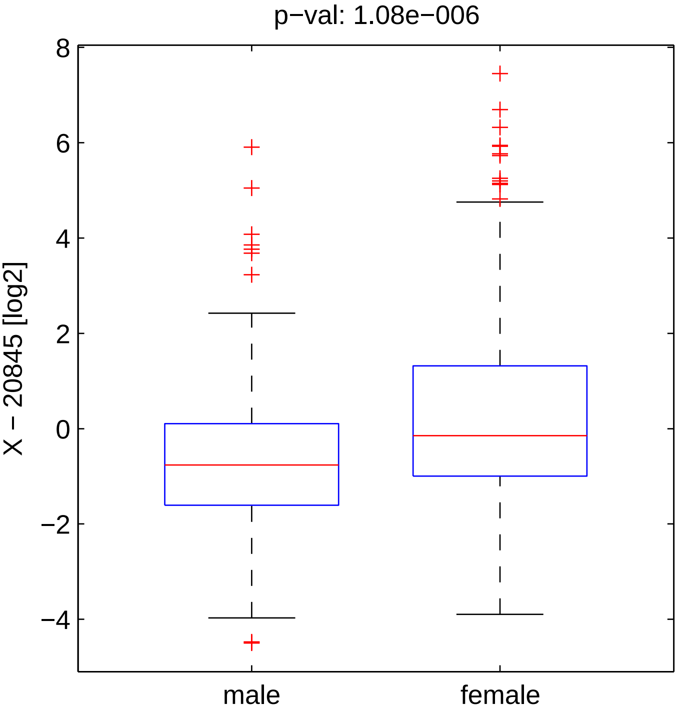

p−val: 1.08e−006
X − 20845 [log2]
8
6
4
2
0
−2
−4
male
female

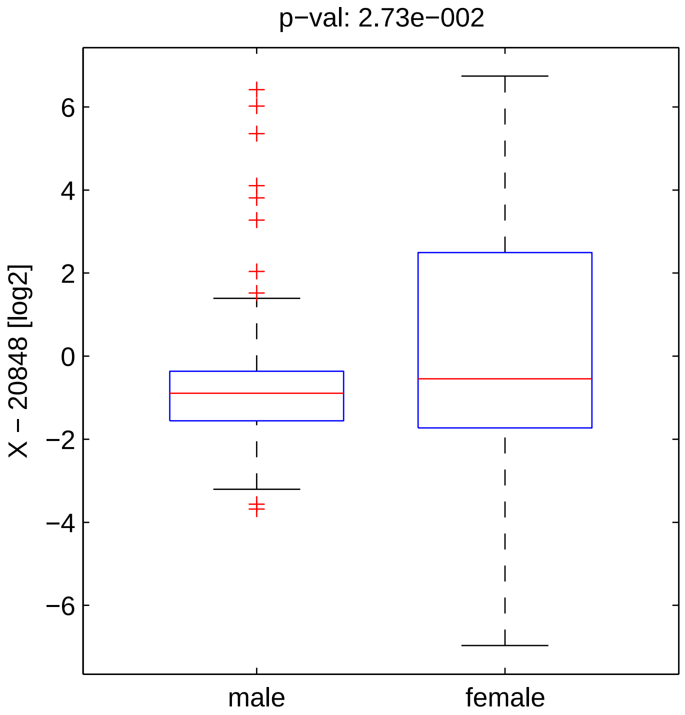

p−val: 2.73e−002
X − 20848 [log2]
6
4
2
0
−2
−4
−6
male
female

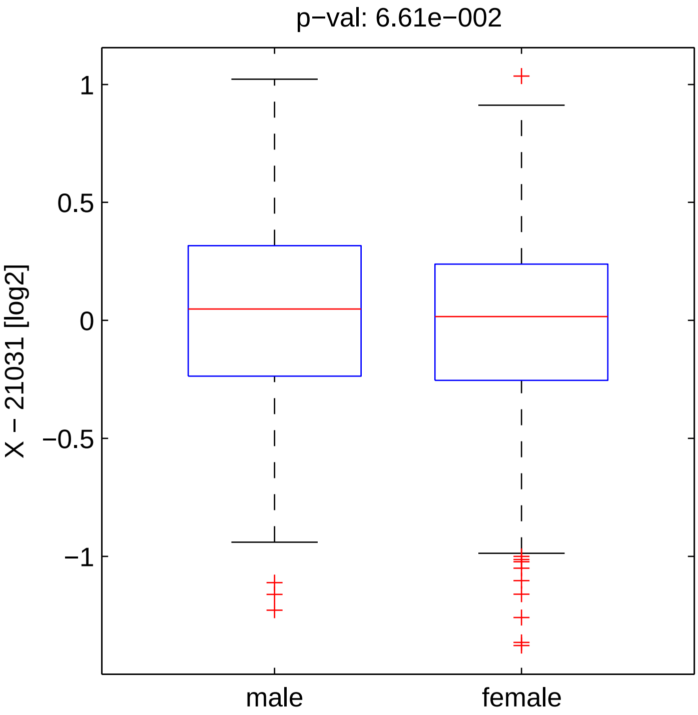

p−val: 6.61e−002
X − 21031 [log2]
1
0.5
0
−0.5
−1
male
female

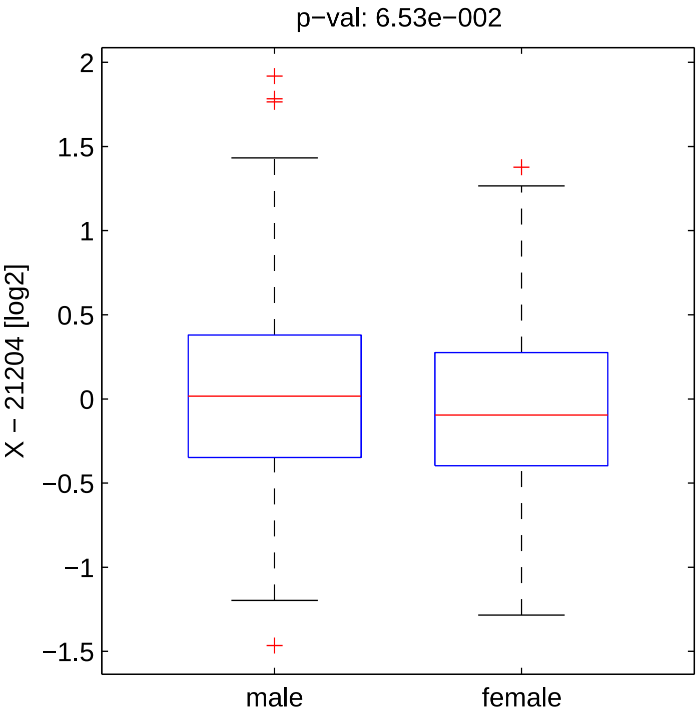
p−val: 6.53e−002
X − 21204 [log2]
2
1.5
1
0.5
0
−0.5
−1
−1.5
male
female

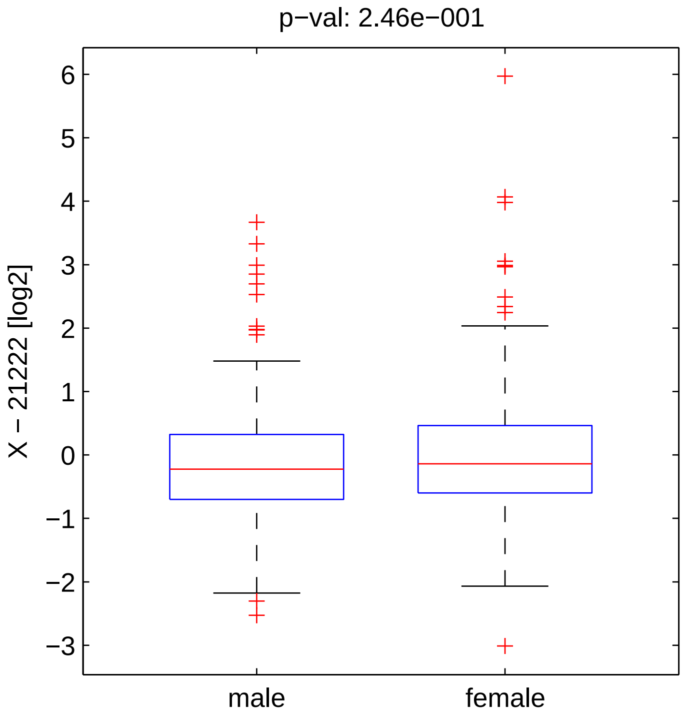

p−val: 2.46e−001
X − 21222 [log2]
6
5
4
3
2
1
0
−1
−2
−3
male
female

SHIP pathway boxplots

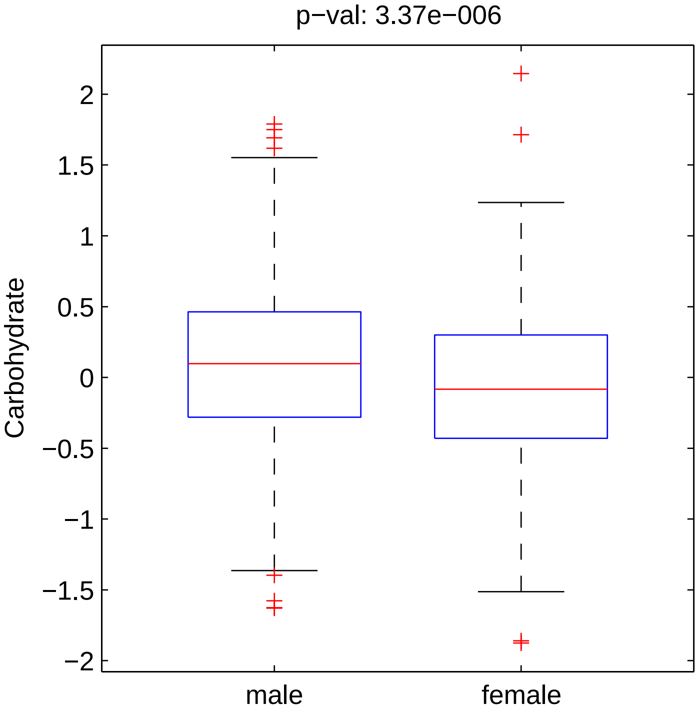
p−val: 3.37e−006
Carbohydrate
2
1.5
1
0.5
0
−0.5
−1
−1.5
−2
male
female

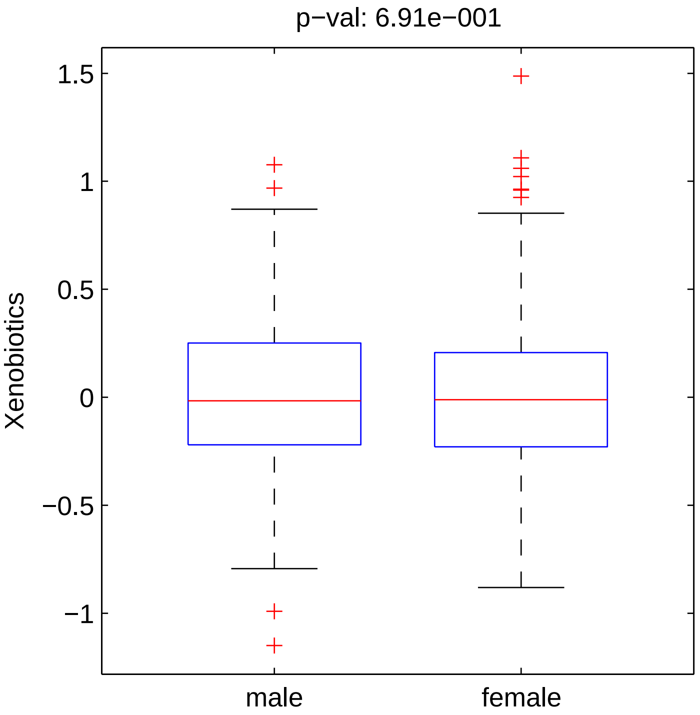
p−val: 6.91e−001
1.5
1
0.5
0
−0.5
−1
Xenobiotics
male
female

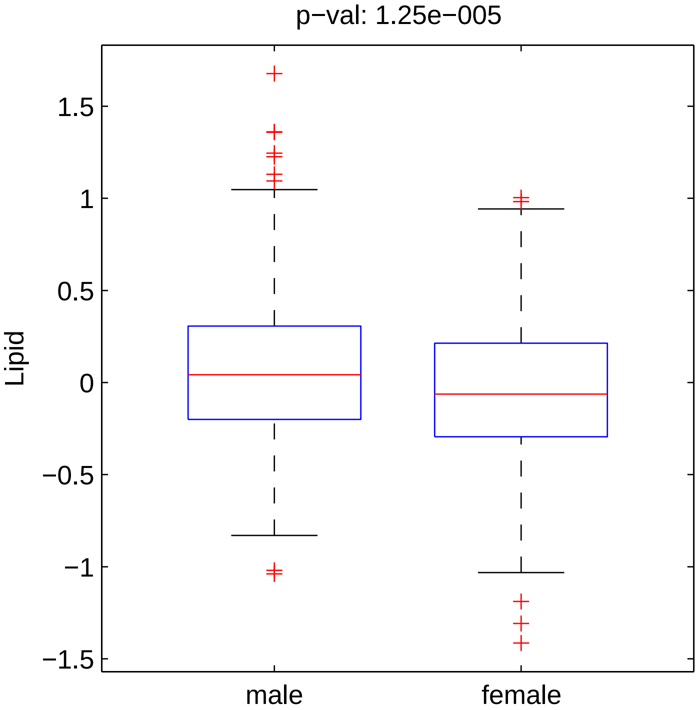

p−val: 1.25e−005
Lipid
1.5
1
0.5
0
−0.5
−1
−1.5
male
female

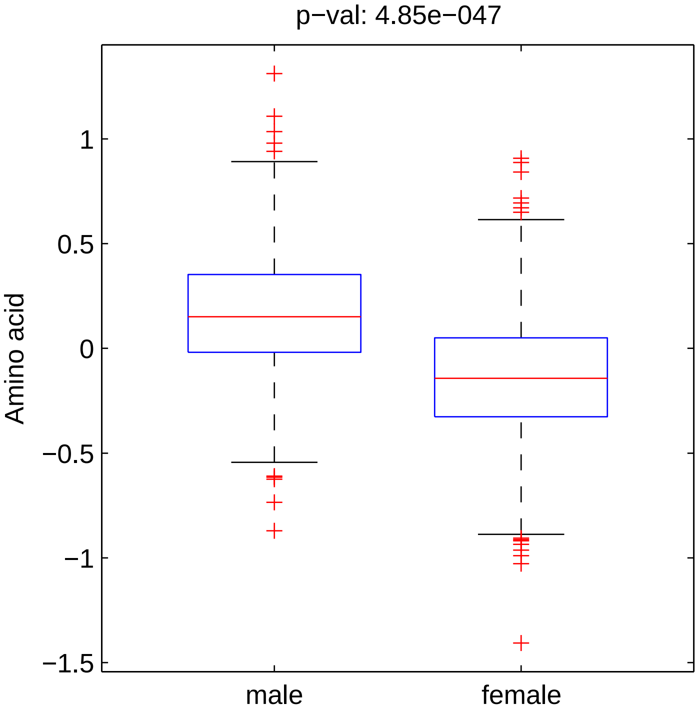
p−val: 4.85e−047
Amino acid
1
0.5
0
−0.5
−1
−1.5
male
female

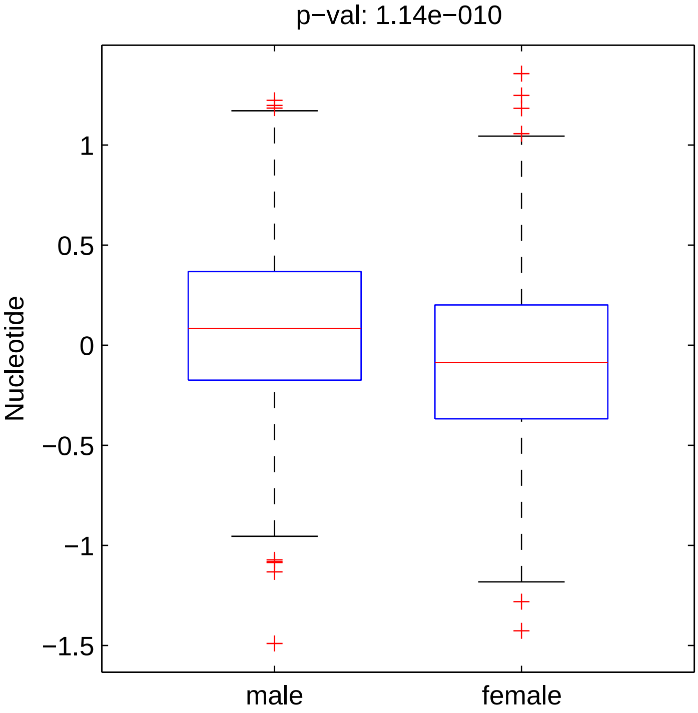
p−val: 1.14e−010
Nucleotide
1
0.5
0
−0.5
−1
−1.5
male
female

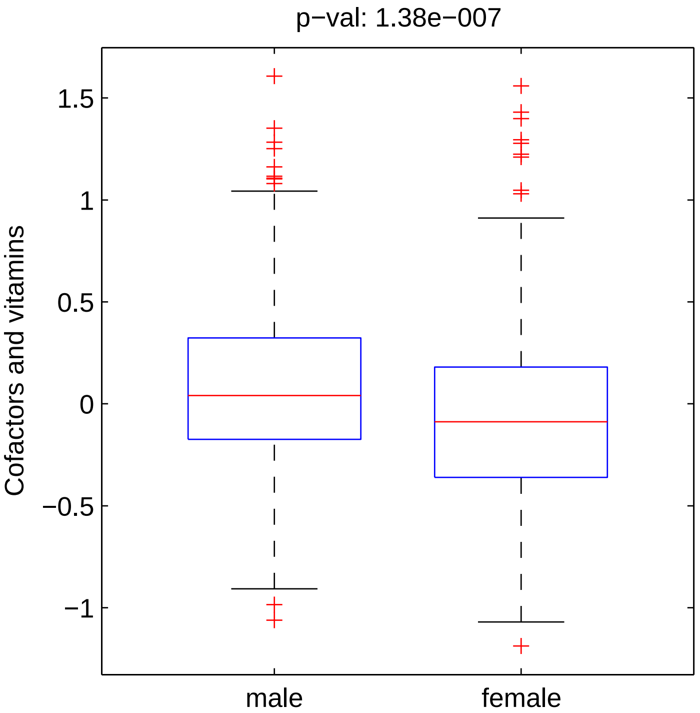

p−val: 1.38e−007
Cofactors and vitamins
1.5
1
0.5
0
−0.5
−1
male
female

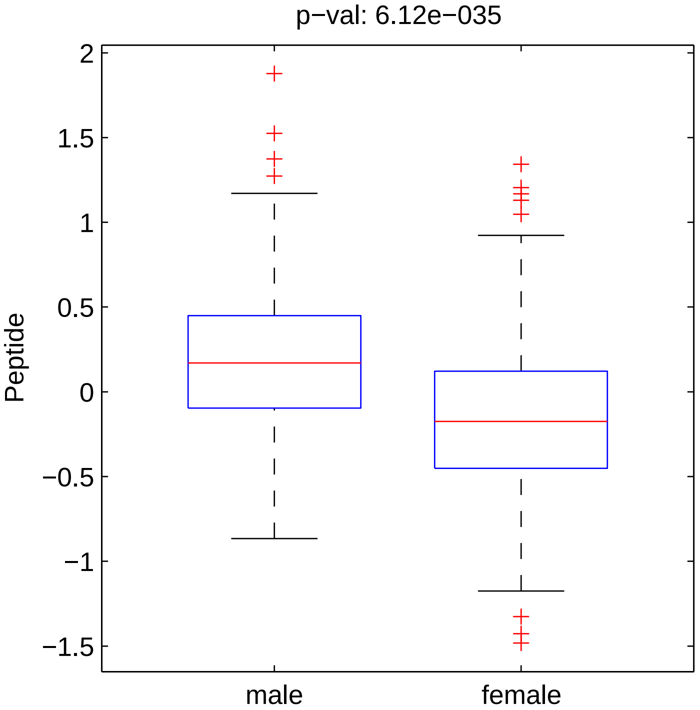
p−val: 6.12e−035
2
1.5
1
0.5
0
−0.5
−1
−1.5
Peptide
male
female

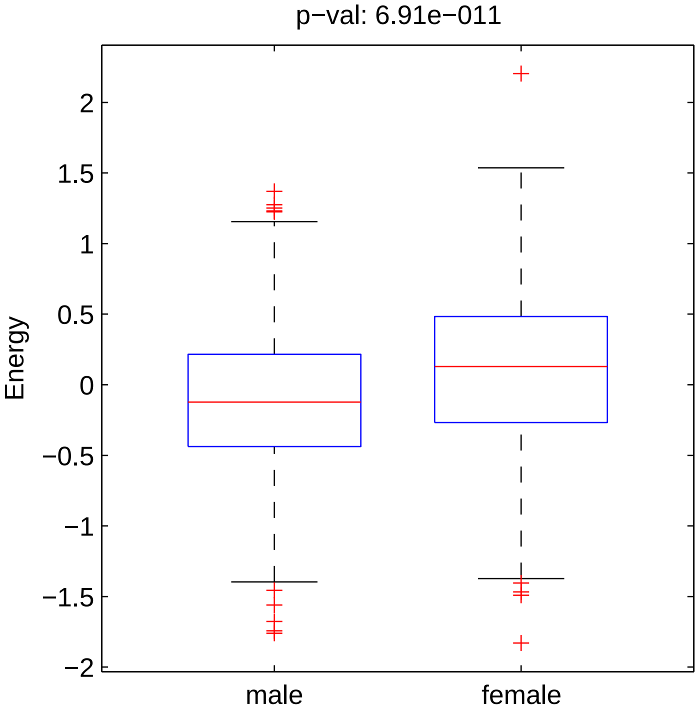
p−val: 6.91e−011
Energy
2
1.5
1
0.5
0
−0.5
−1
−1.5
−2
male
female

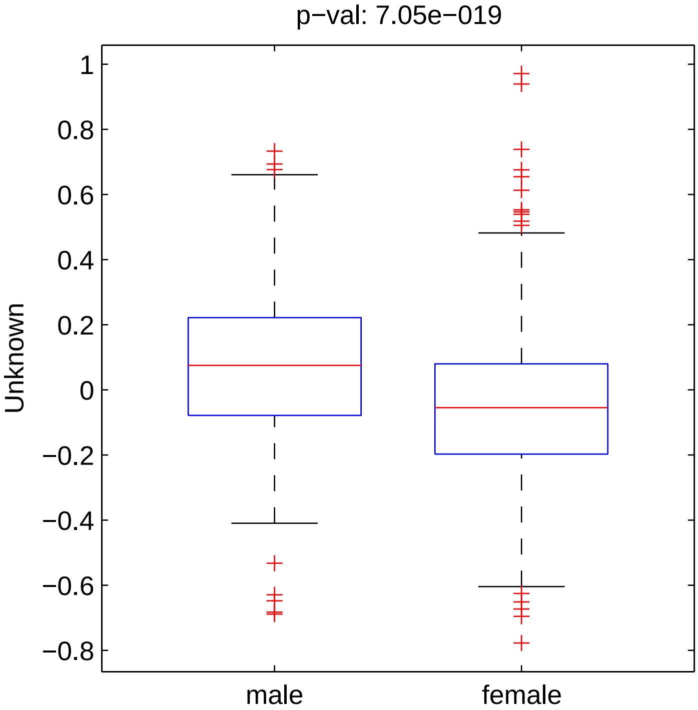
p−val: 7.05e−019
Unknown
1
0.8
0.6
0.4
0.2
0
−0.2
−0.4
−0.6
−0.8
male
female

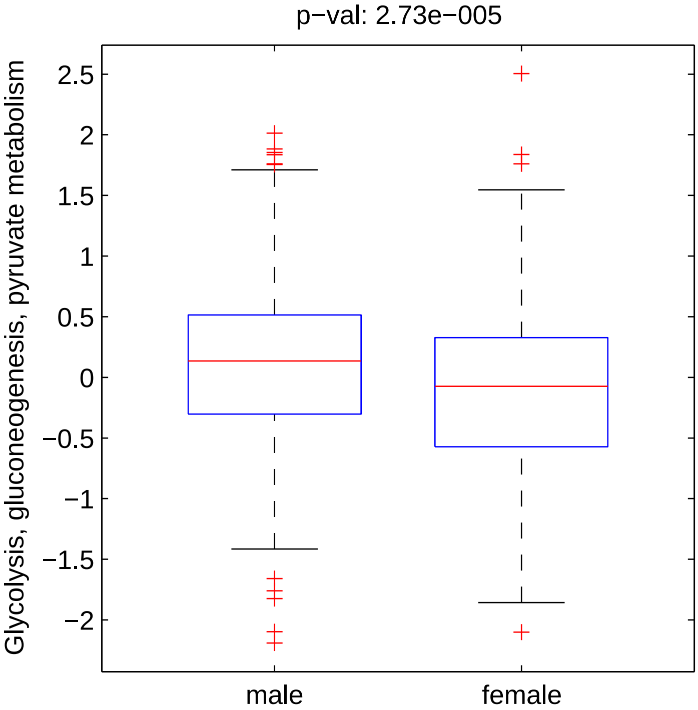
p−val: 2.73e−005
Glycolysis, gluconeogenesis, pyruvate metabolism
2.5
2
1.5
1
0.5
0
−0.5
−1
−1.5
−2
male
female

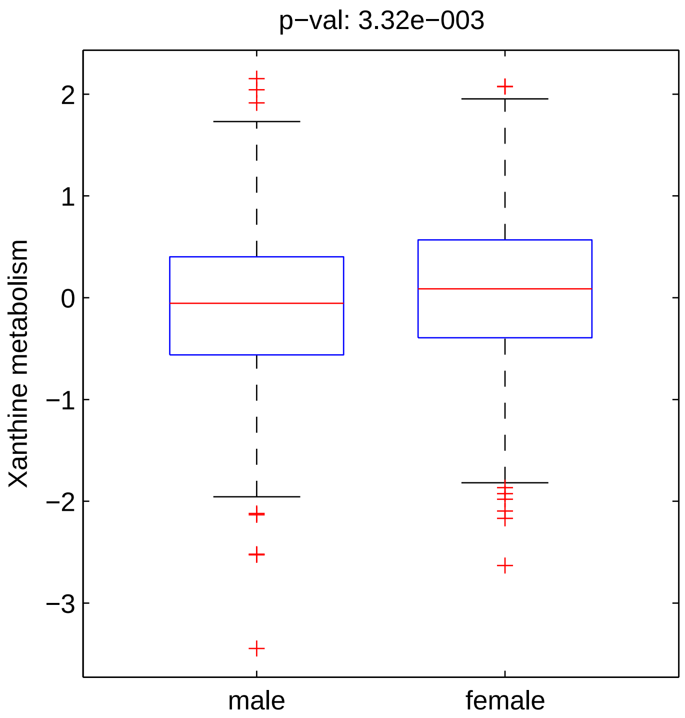

p−val: 3.32e−003
Xanthine metabolism
2
1
0
−1
−2
−3
male
female

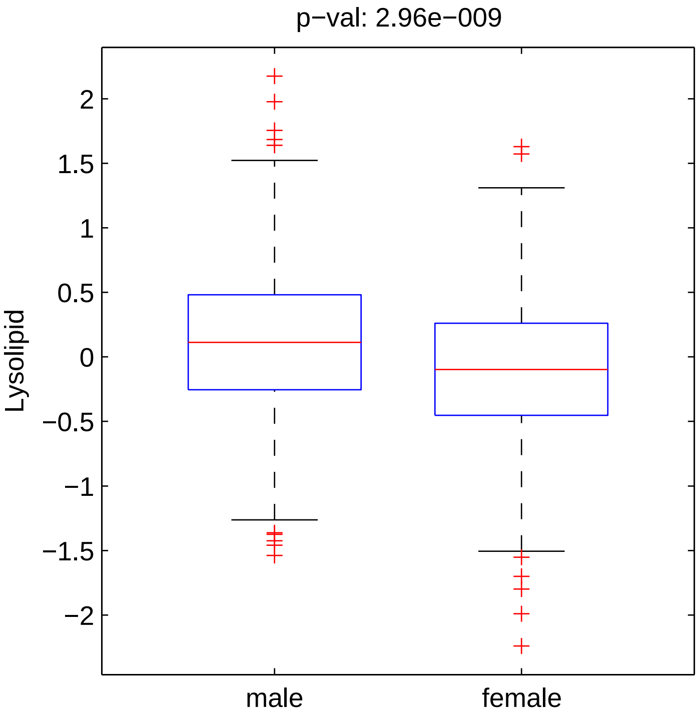
p−val: 2.96e−009
Lysolipid
2
1.5
1
0.5
0
−0.5
−1
−1.5
−2
male
female

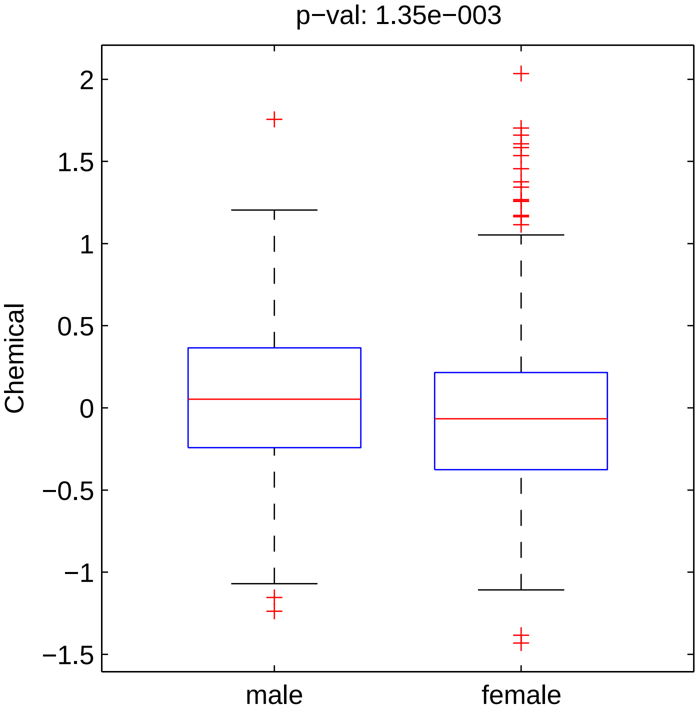

p−val: 1.35e−003
Chemical
2
1.5
1
0.5
0
−0.5
−1
−1.5
male
female

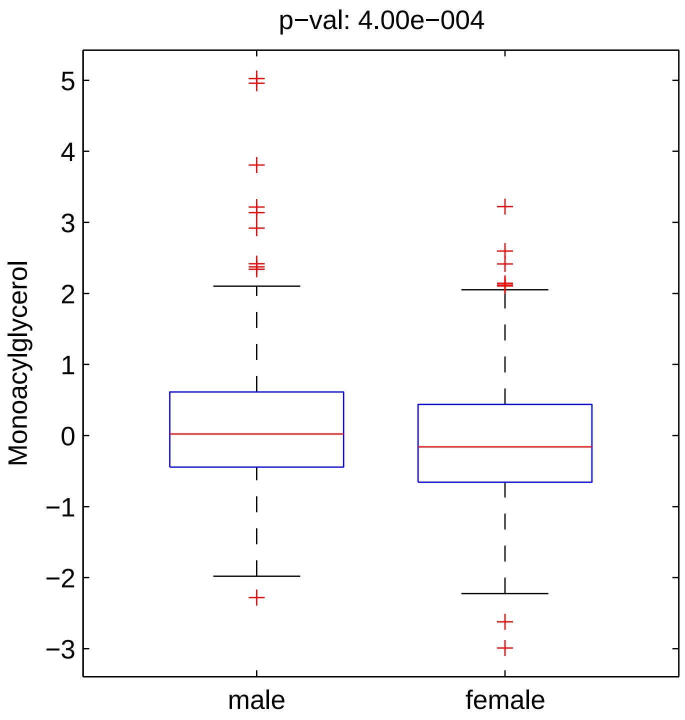

p−val: 4.00e−004
Monoacylglycerol
5
4
3
2
1
0
−1
−2
−3
male
female

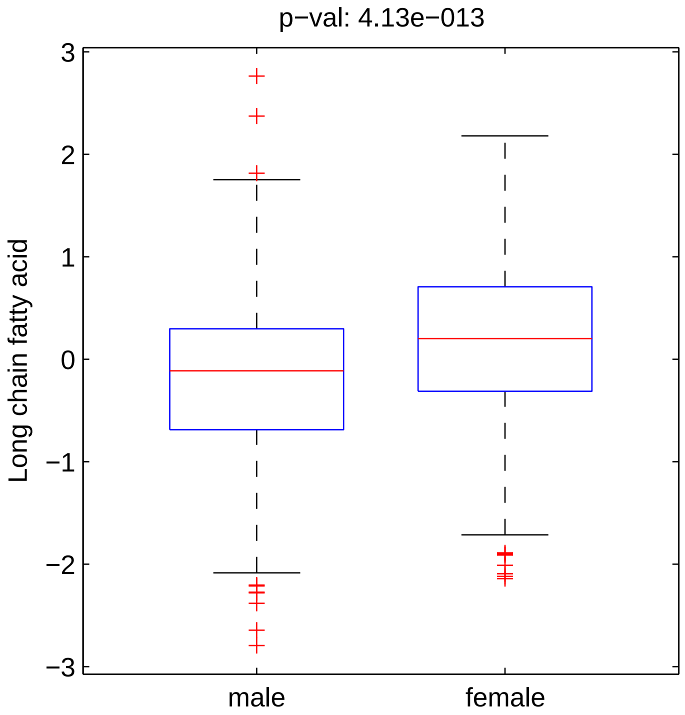

p−val: 4.13e−013
Long chain fatty acid
3
2
1
0
−1
−2
−3
male
female

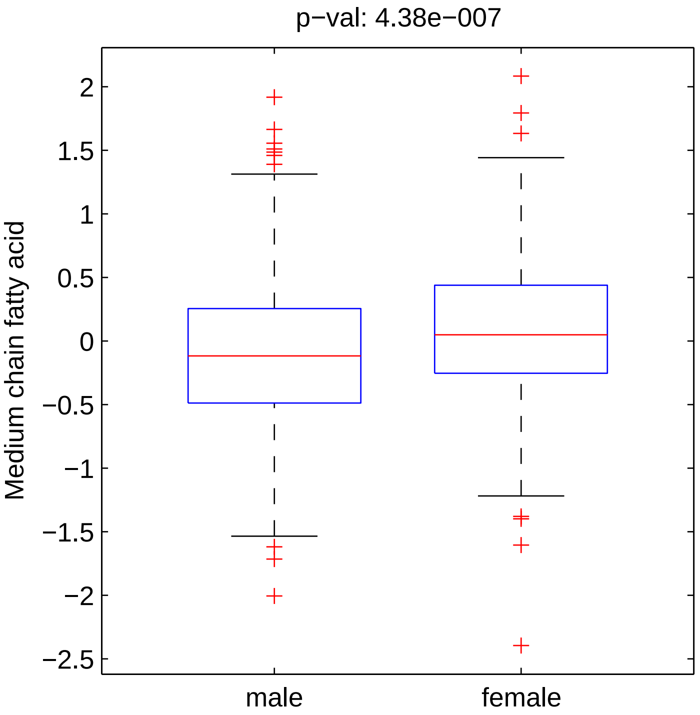
p−val: 4.38e−007
Medium chain fatty acid
2
1.5
1
0.5
0
−0.5
−1
−1.5
−2
−2.5
male
female

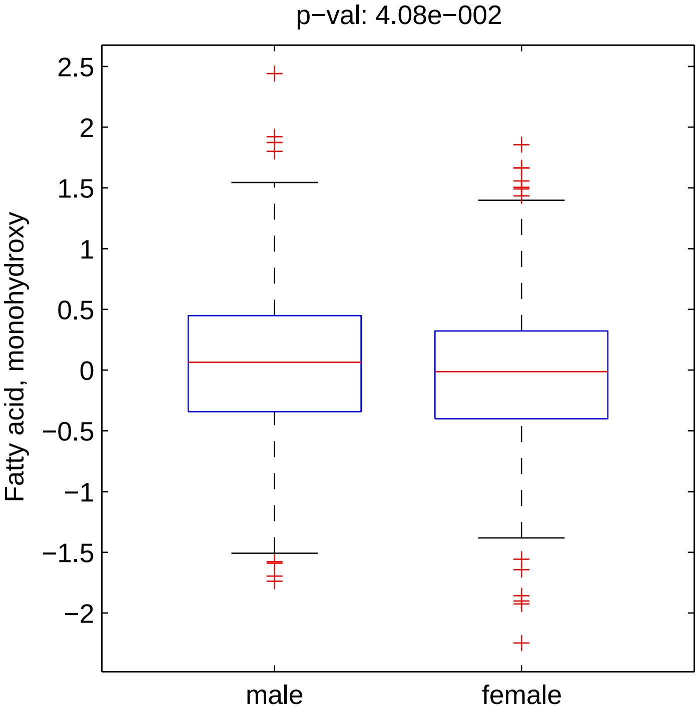

p−val: 4.08e−002
Fatty acid, monohydroxy
2.5
2
1.5
1
0.5
0
−0.5
−1
−1.5
−2
male
female

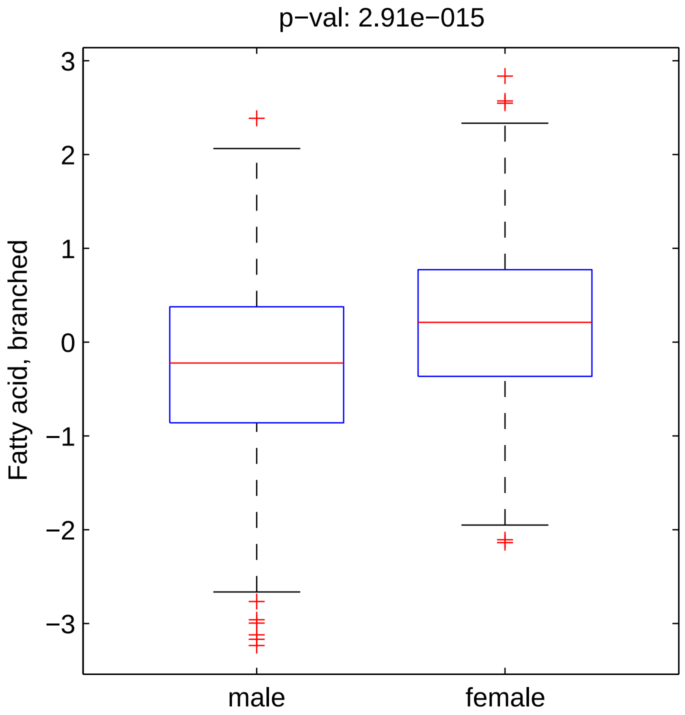
p−val: 2.91e−015
3
2
1
0
−1
−2
−3
Fatty acid, branched
male
female

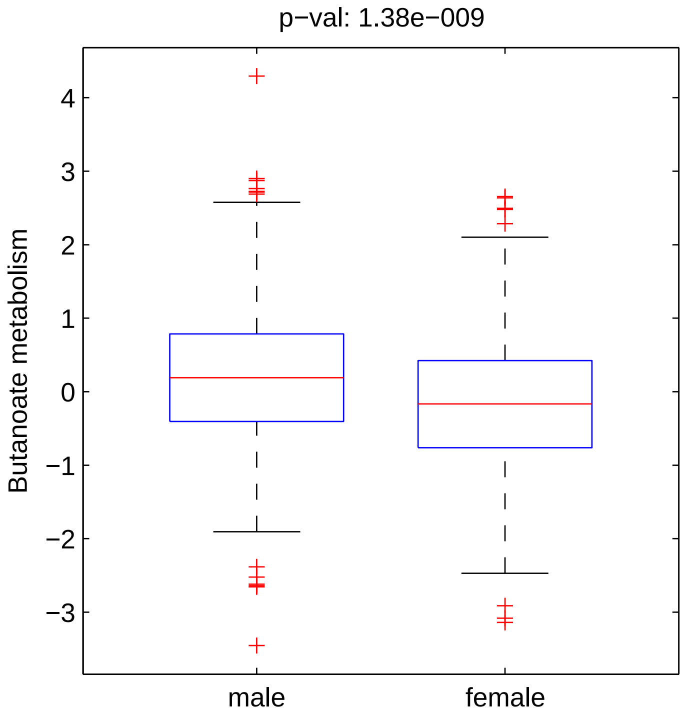
p−val: 1.38e−009
Butanoate metabolism
4
3
2
1
0
−1
−2
−3
male
female

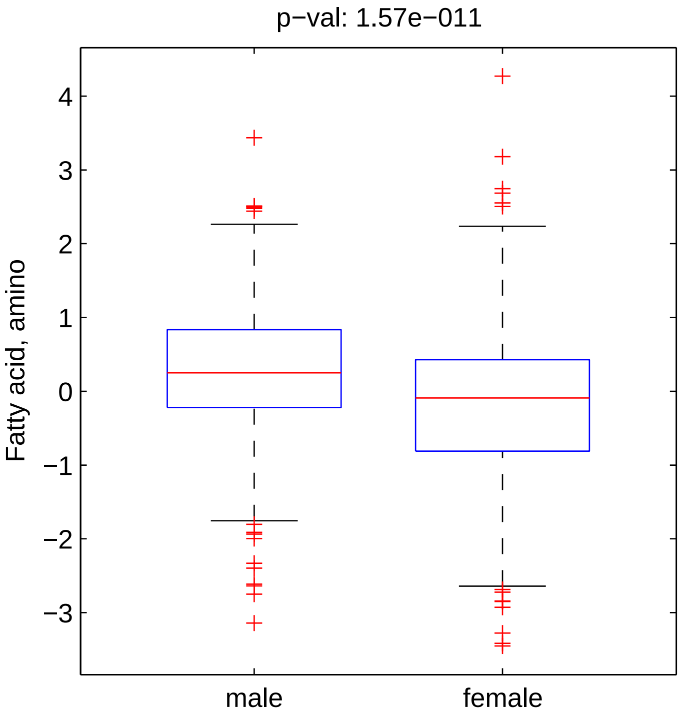
p−val: 1.57e−011
Fatty acid, amino
4
3
2
1
0
−1
−2
−3
male
female

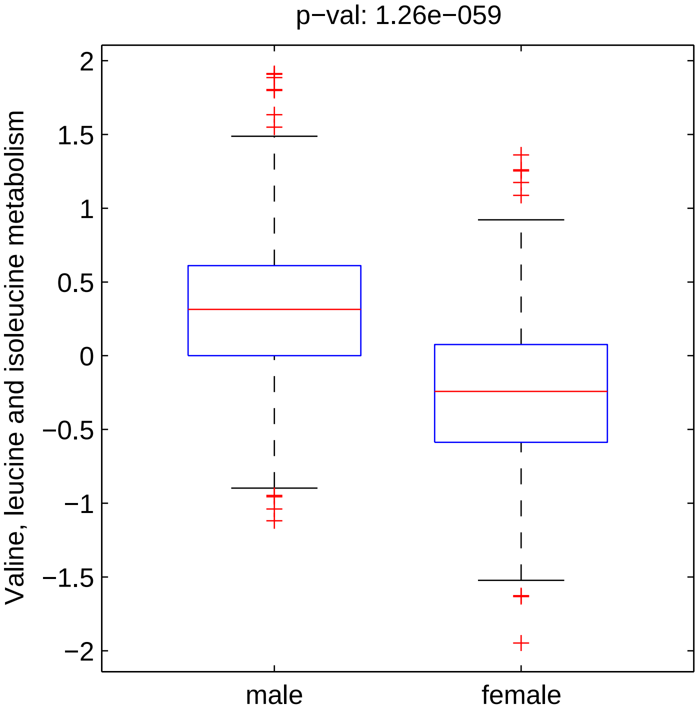

p−val: 1.26e−059
Valine, leucine and isoleucine metabolism
2
1.5
1
0.5
0
−0.5
−1
−1.5
−2
male
female

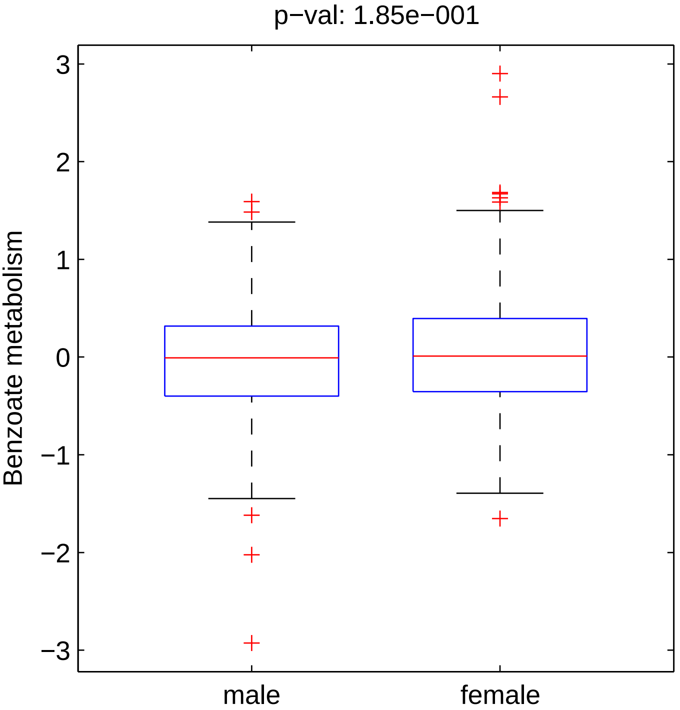
p−val: 1.85e−001
Benzoate metabolism
3
2
1
0
−1
−2
−3
male
female

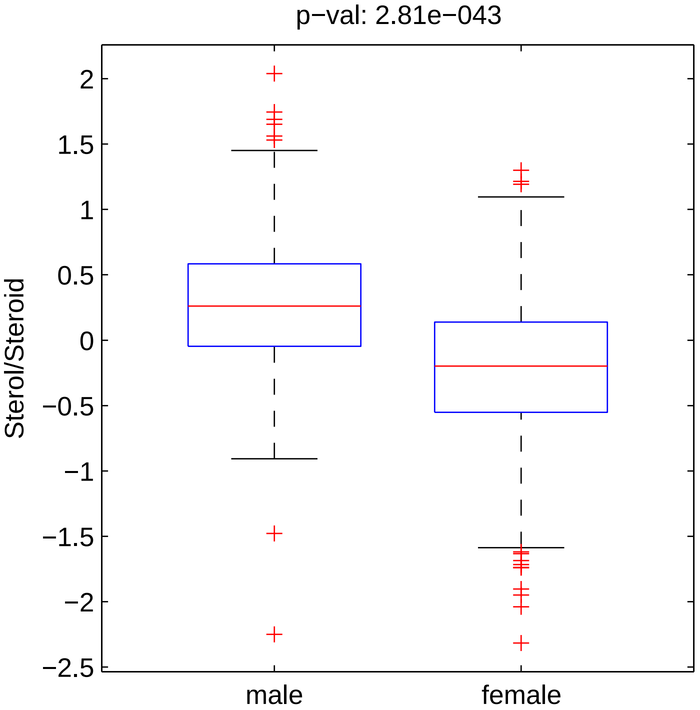

p−val: 2.81e−043
Sterol/Steroid
2
1.5
1
0.5
0
−0.5
−1
−1.5
−2
−2.5
male
female

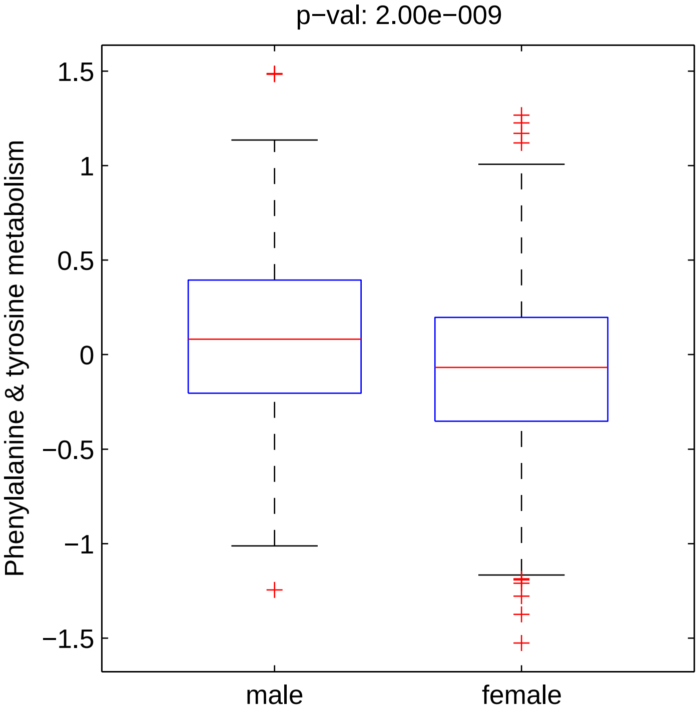

p−val: 2.00e−009
1.5
1
0.5
0
−0.5
−1
−1.5
Phenylalanine & tyrosine metabolism
male
female

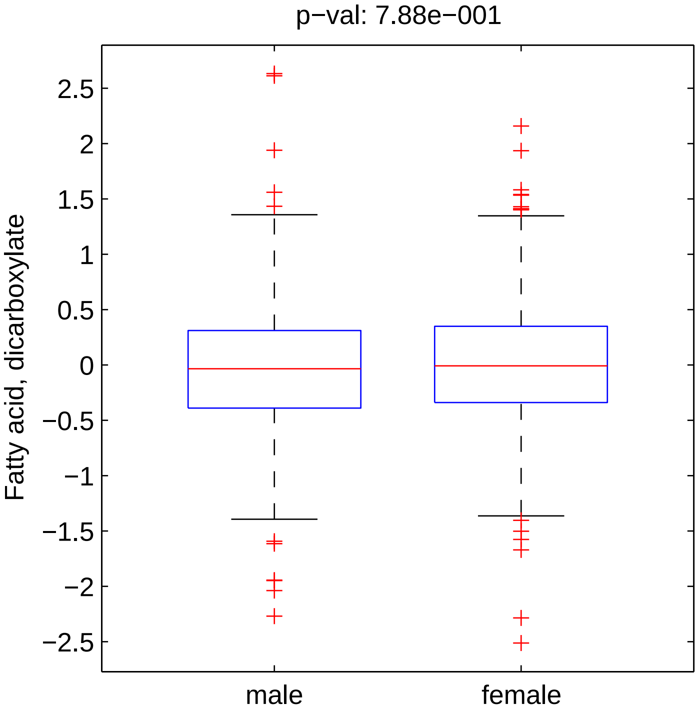
p−val: 7.88e−001
Fatty acid, dicarboxylate
2.5
2
1.5
1
0.5
0
−0.5
−1
−1.5
−2
−2.5
male
female

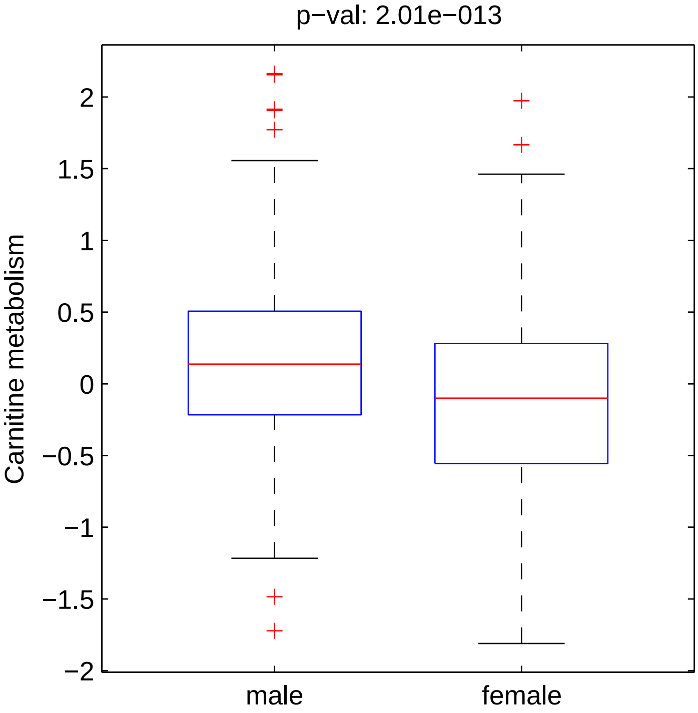
p−val: 2.01e−013
Carnitine metabolism
2
1.5
1
0.5
0
−0.5
−1
−1.5
−2
male
female

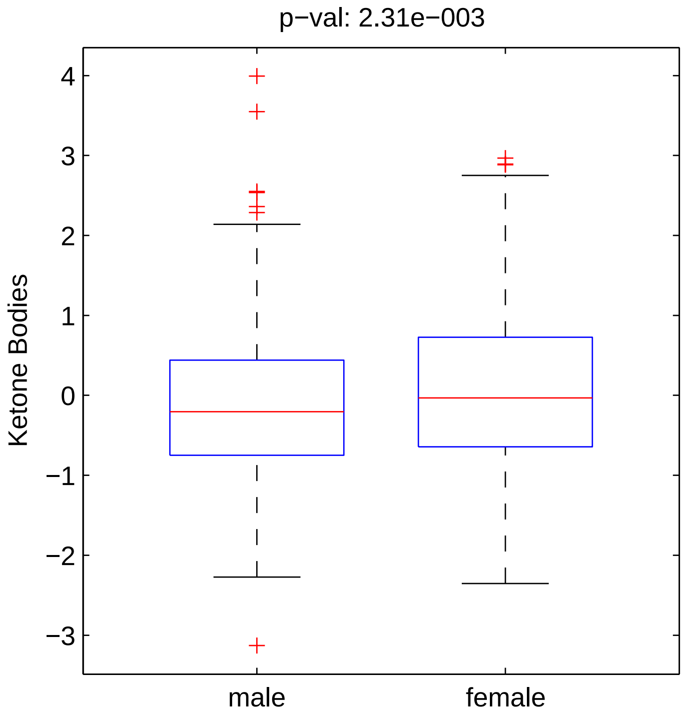

p−val: 2.31e−003
Ketone Bodies
4
3
2
1
0
−1
−2
−3
male
female

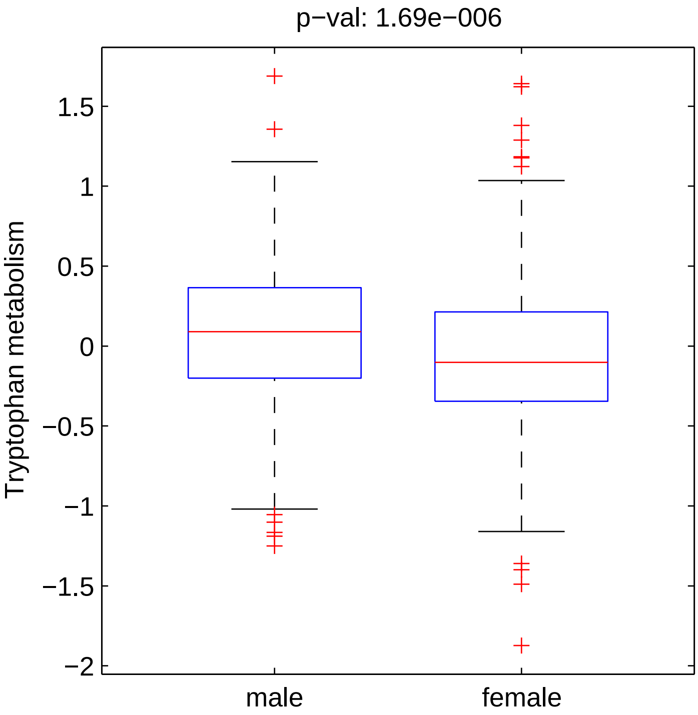
p−val: 1.69e−006
Tryptophan metabolism
1.5
1
0.5
0
−0.5
−1
−1.5
−2
male
female

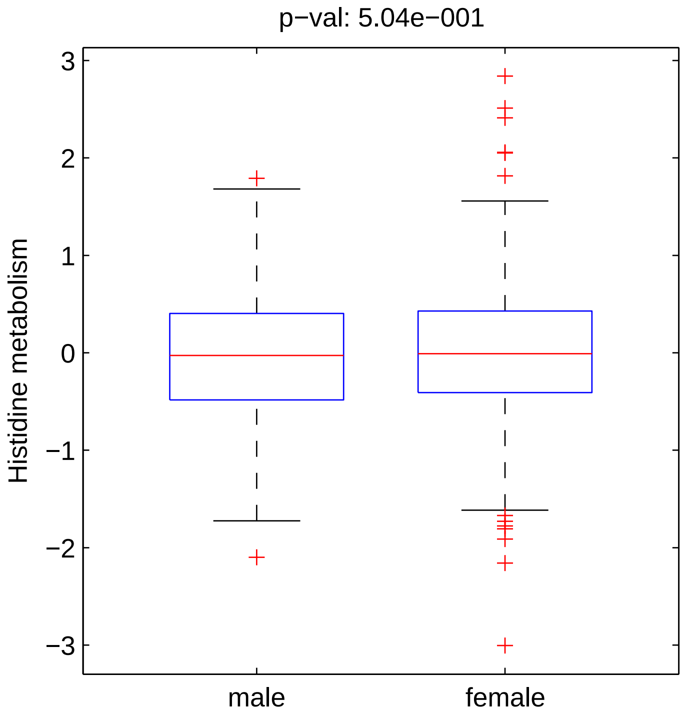
p−val: 5.04e−001
Histidine metabolism
3
2
1
0
−1
−2
−3
male
female

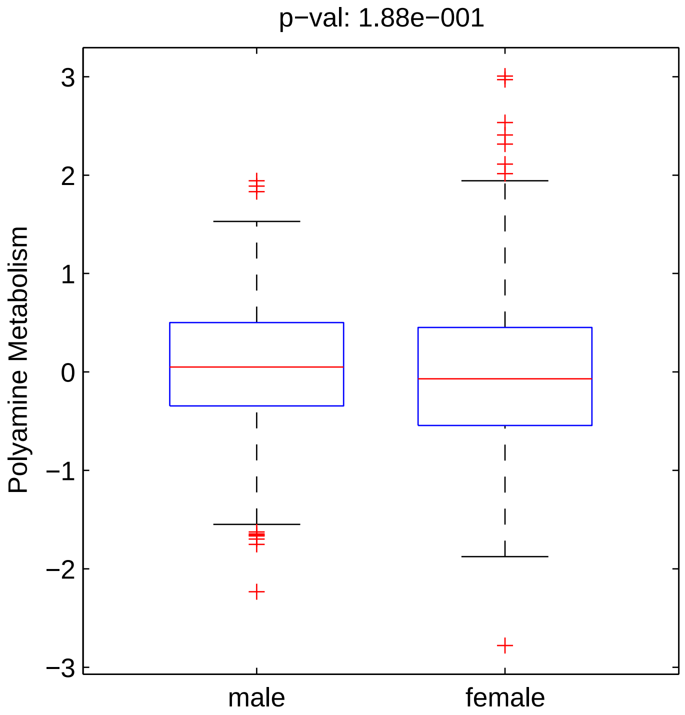
p−val: 1.88e−001
Polyamine Metabolism
3
2
1
0
−1
−2
−3
male
female

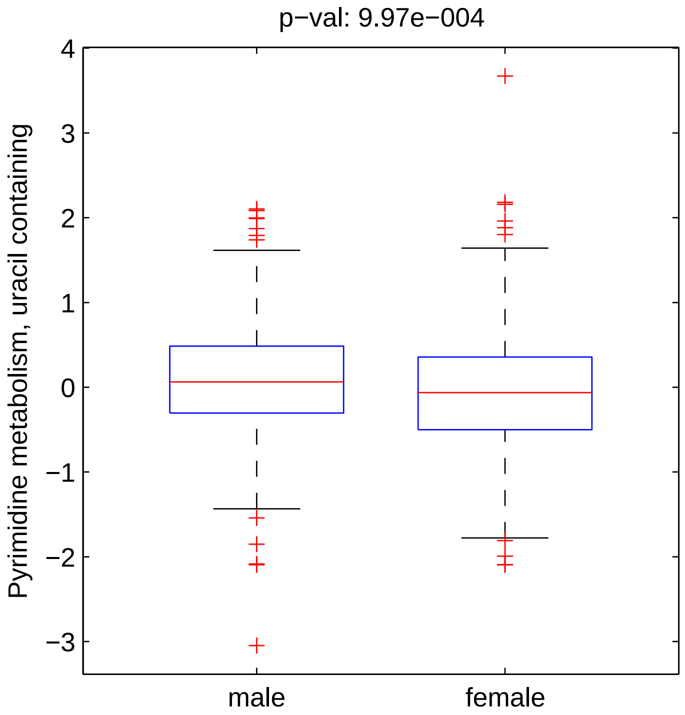

p−val: 9.97e−004
Pyrimidine metabolism, uracil containing
4
3
2
1
0
−1
−2
−3
male
female

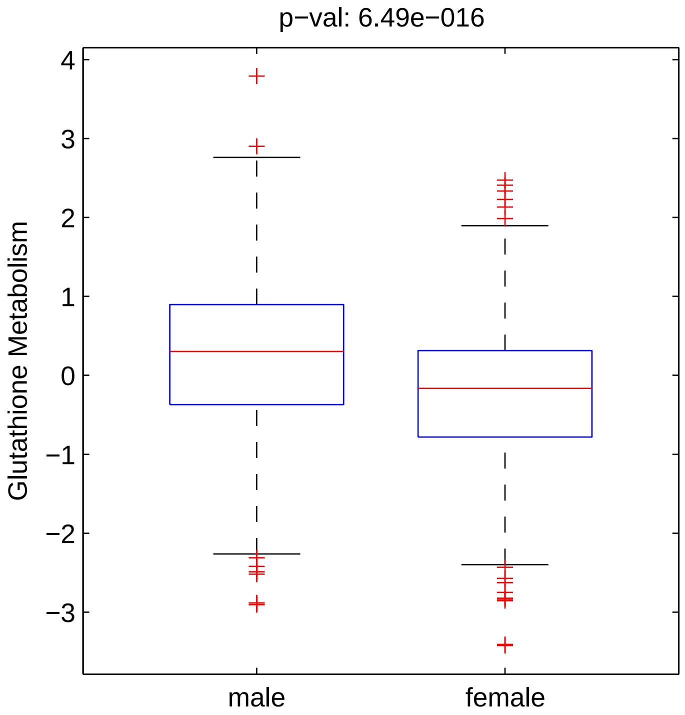
p−val: 6.49e−016
Glutathione Metabolism
4
3
2
1
0
−1
−2
−3
male
female

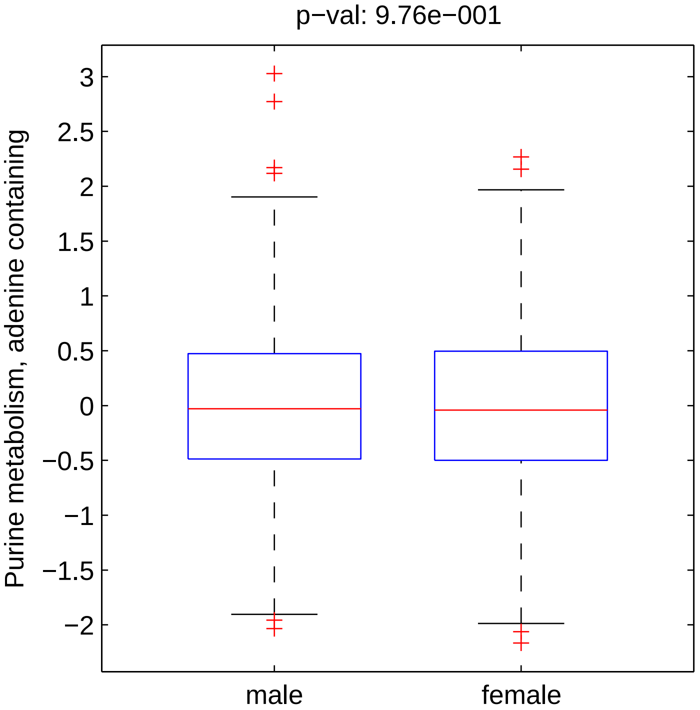
p−val: 9.76e−001
Purine metabolism, adenine containing
3
2.5
2
1.5
1
0.5
0
−0.5
−1
−1.5
−2
male
female

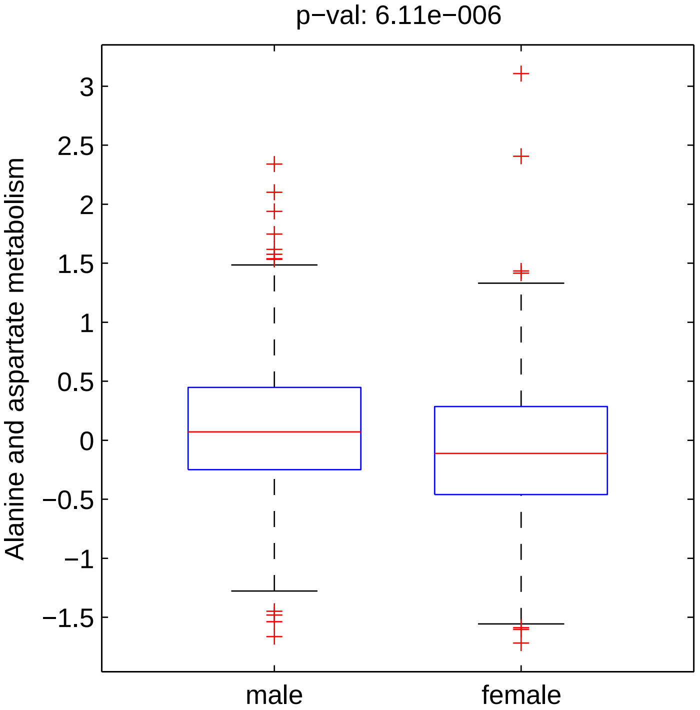
p−val: 6.11e−006
Alanine and aspartate metabolism
3
2.5
2
1.5
1
0.5
0
−0.5
−1
−1.5
male
female

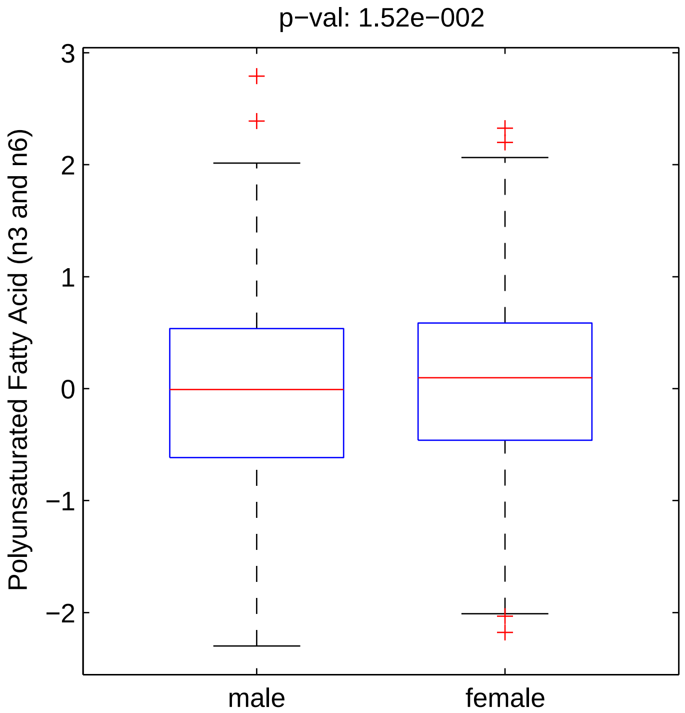

p−val: 1.52e−002
Polyunsaturated Fatty Acid (n3 and n6)
3
2
1
0
−1
−2
male
female

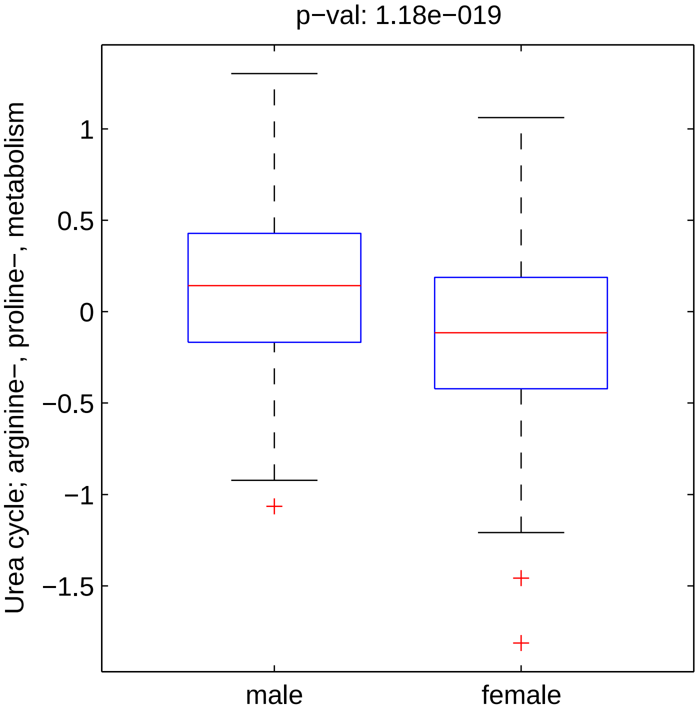

p−val: 1.18e−019
Urea cycle; arginine−, proline−, metabolism
1
0.5
0
−0.5
−1
−1.5
male
female

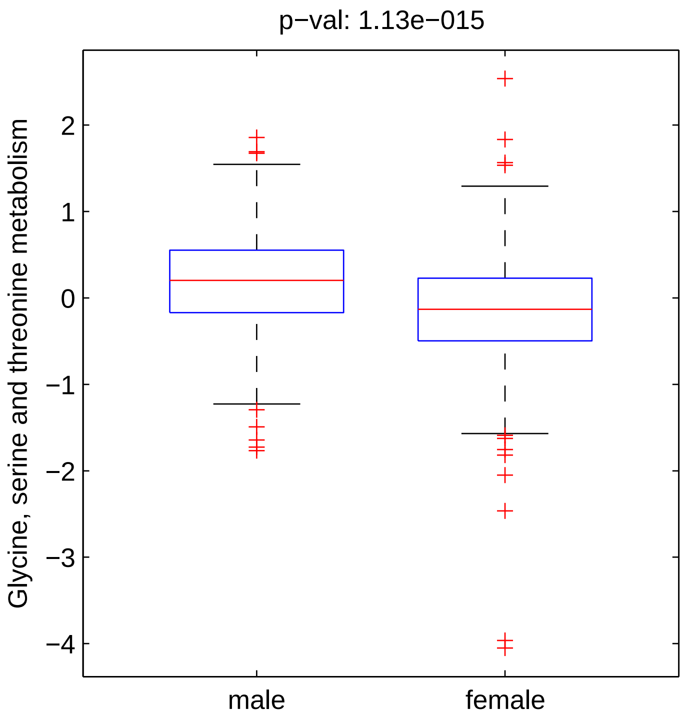
p−val: 1.13e−015
Glycine, serine and threonine metabolism
2
1
0
−1
−2
−3
−4
male
female

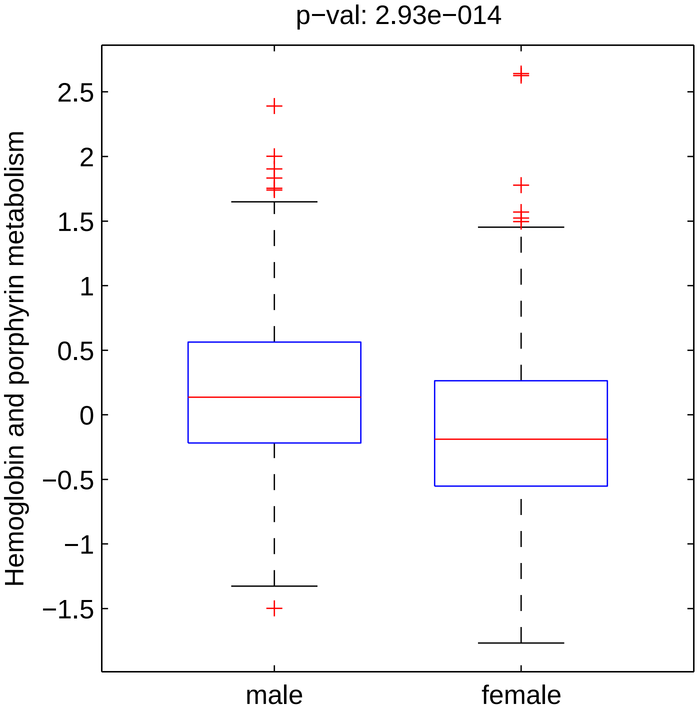
p−val: 2.93e−014
Hemoglobin and porphyrin metabolism
2.5
2
1.5
1
0.5
0
−0.5
−1
−1.5
male
female

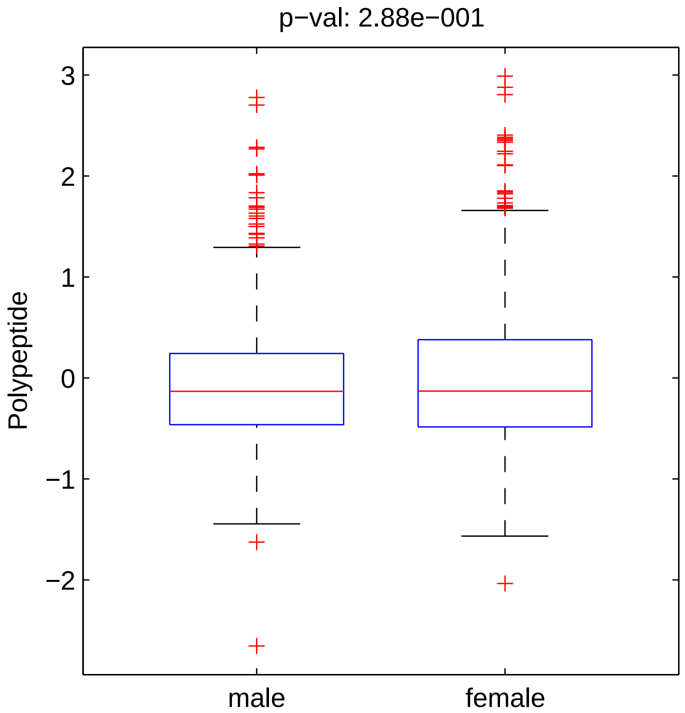
p−val: 2.88e−001
Polypeptide
3
2
1
0
−1
−2
male
female

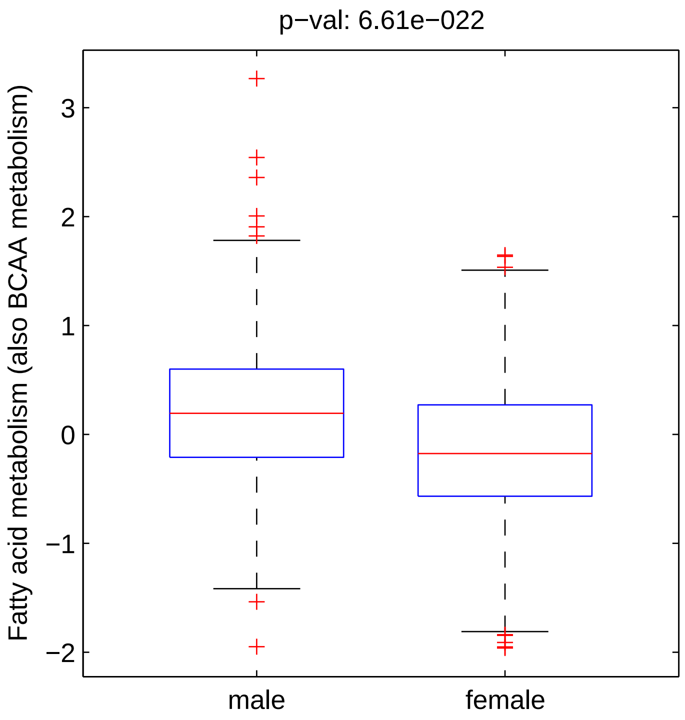
p−val: 6.61e−022
Fatty acid metabolism (also BCAA metabolism)
3
2
1
0
−1
−2
male
female

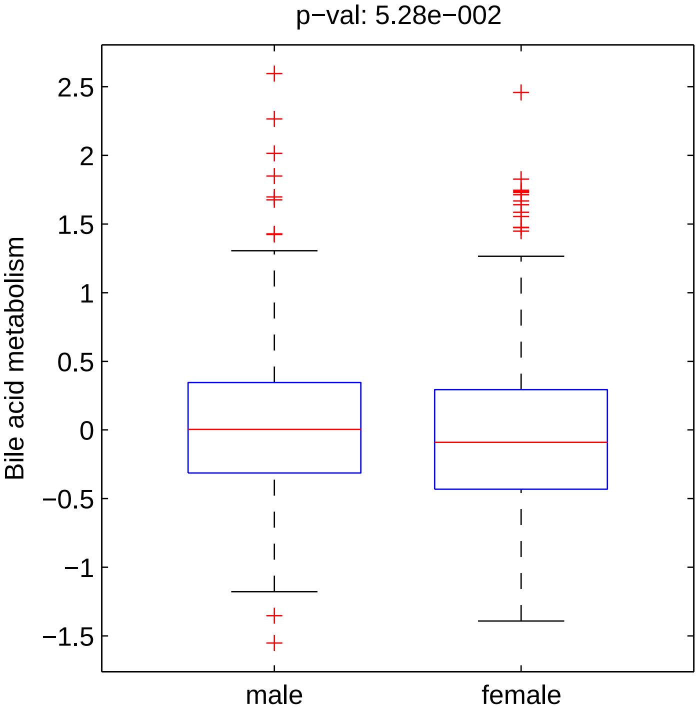

p−val: 5.28e−002
Bile acid metabolism
2.5
2
1.5
1
0.5
0
−0.5
−1
−1.5
male
female

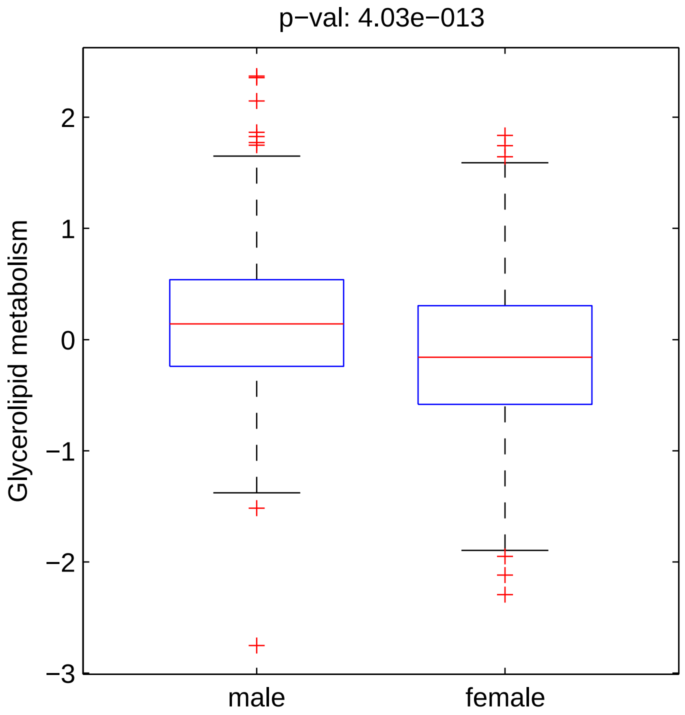

p−val: 4.03e−013
Glycerolipid metabolism
2
1
0
−1
−2
−3
male
female

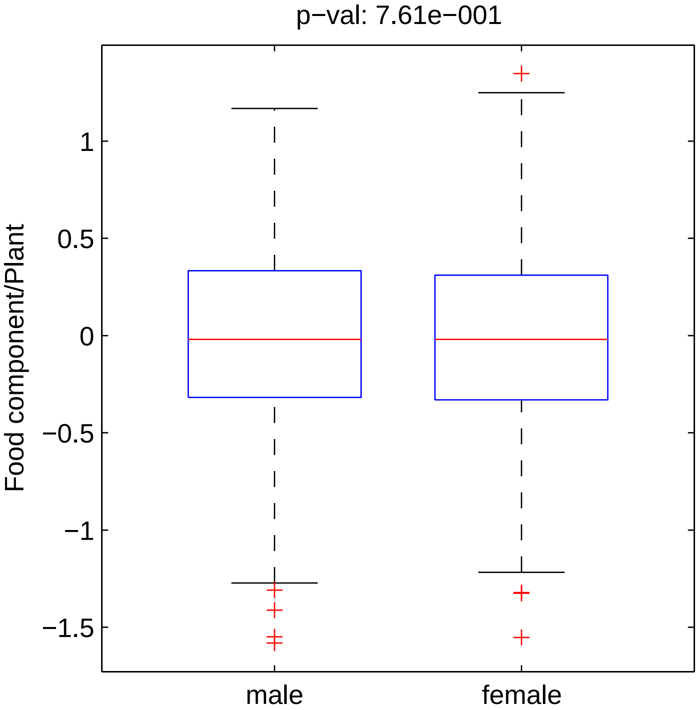
p−val: 7.61e−001
Food component/Plant
1
0.5
0
−0.5
−1
−1.5
male
female

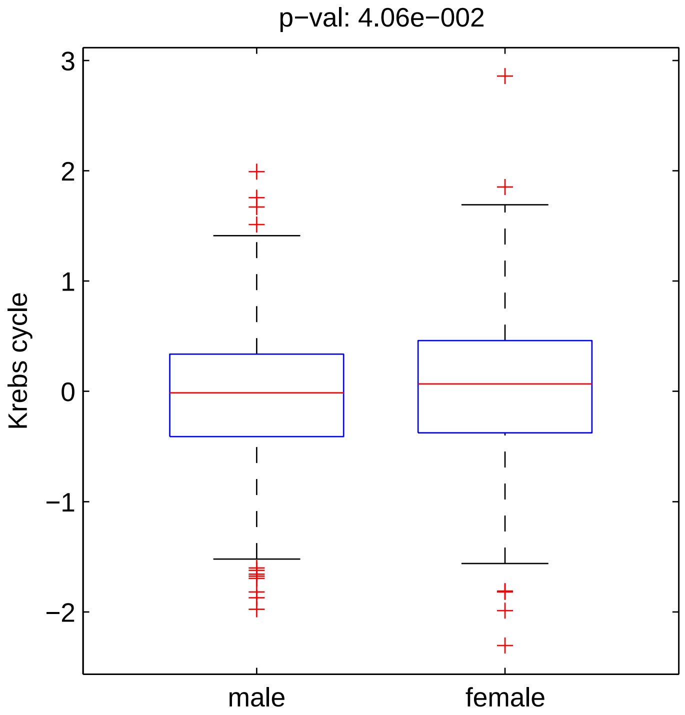

p−val: 4.06e−002
Krebs cycle
3
2
1
0
−1
−2
male
female

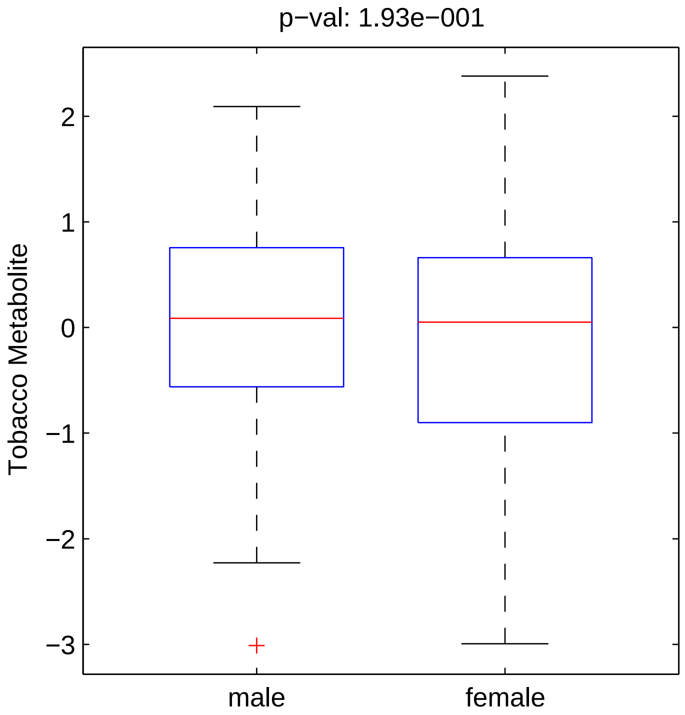
p−val: 1.93e−001
Tobacco Metabolite
2
1
0
−1
−2
−3
male
female

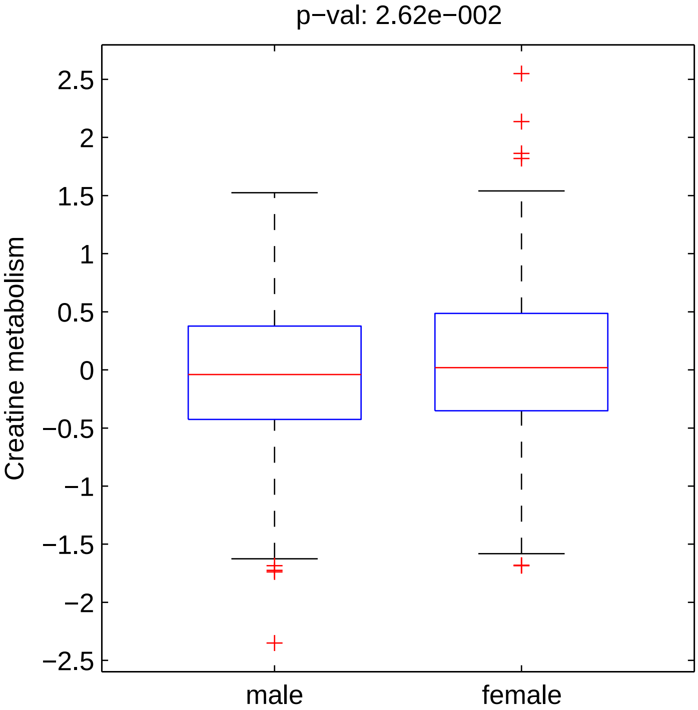
p−val: 2.62e−002
Creatine metabolism
2.5
2
1.5
1
0.5
0
−0.5
−1
−1.5
−2
−2.5
male
female

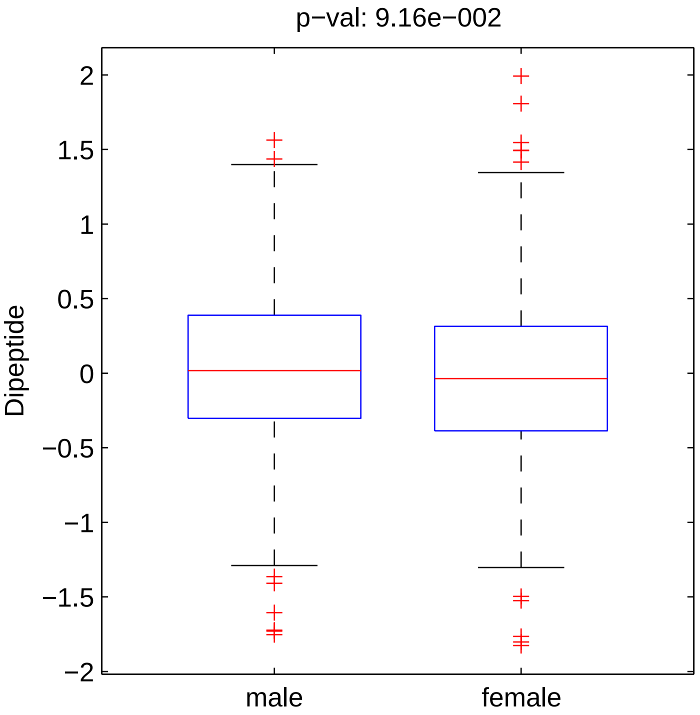

p−val: 9.16e−002
Dipeptide
2
1.5
1
0.5
0
−0.5
−1
−1.5
−2
male
female

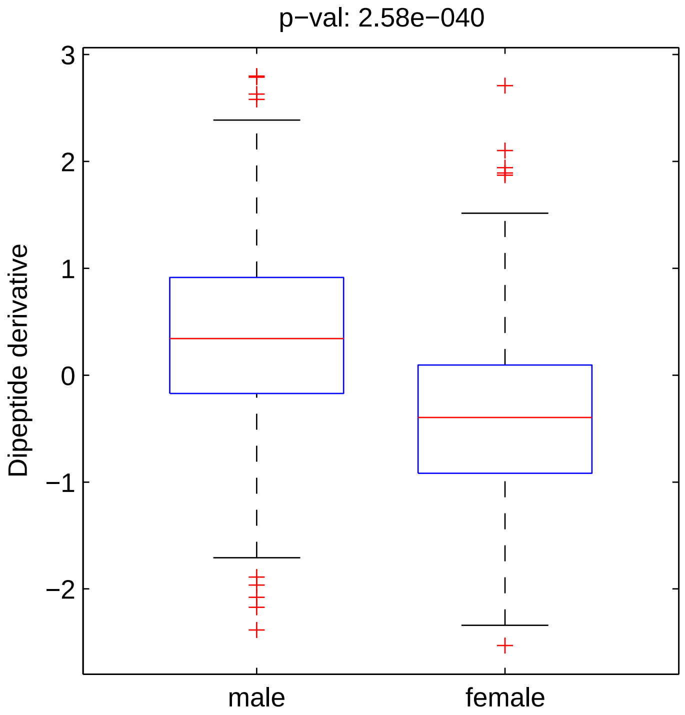

p−val: 2.58e−040
Dipeptide derivative
3
2
1
0
−1
−2
male
female

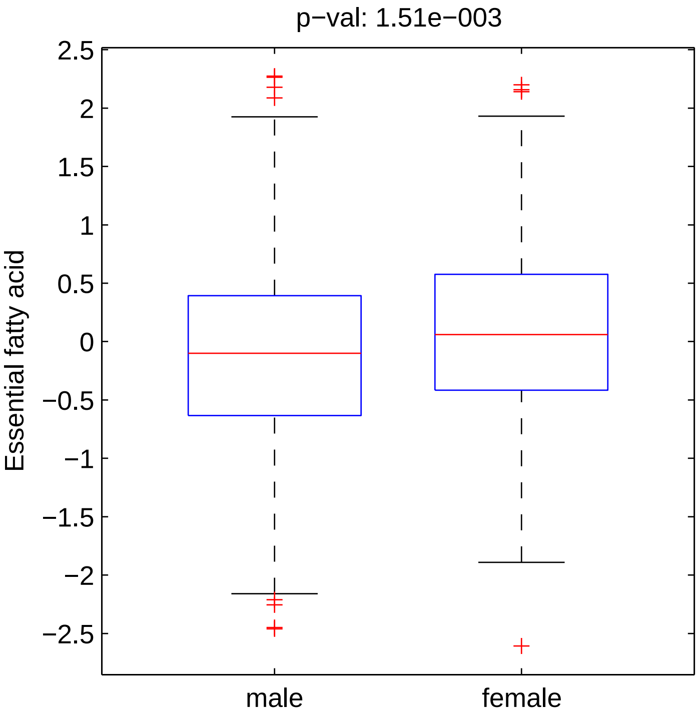

p−val: 1.51e−003
Essential fatty acid
2.5
2
1.5
1
0.5
0
−0.5
−1
−1.5
−2
−2.5
male
female

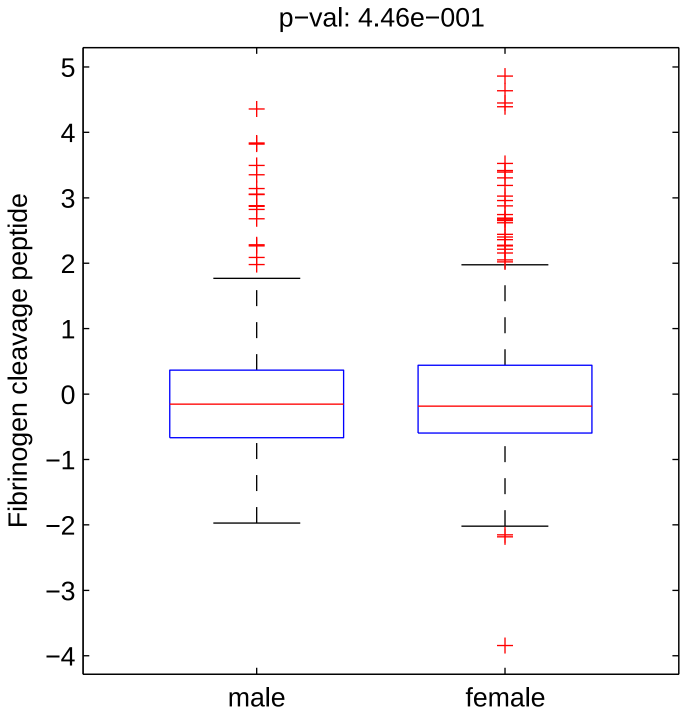

p−val: 4.46e−001
Fibrinogen cleavage peptide
5
4
3
2
1
0
−1
−2
−3
−4
male
female

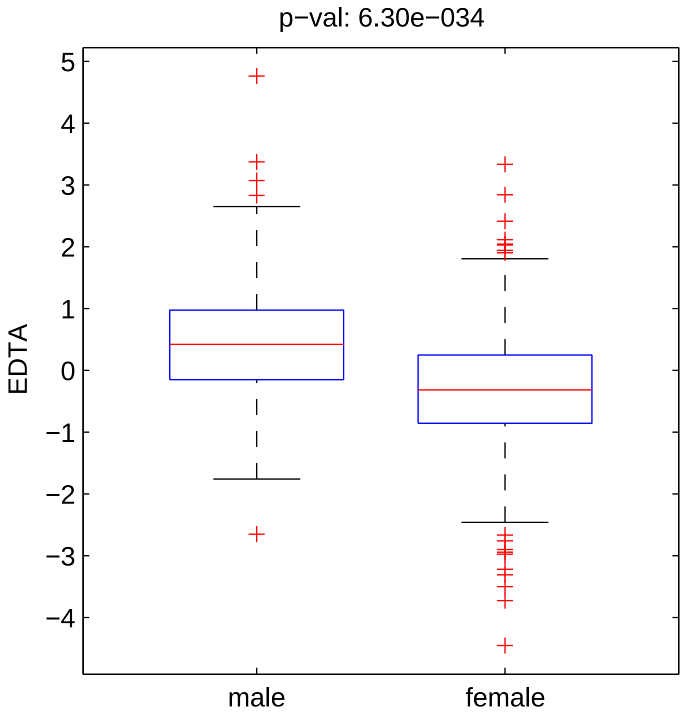

p−val: 6.30e−034
EDTA
5
4
3
2
1
0
−1
−2
−3
−4
male
female

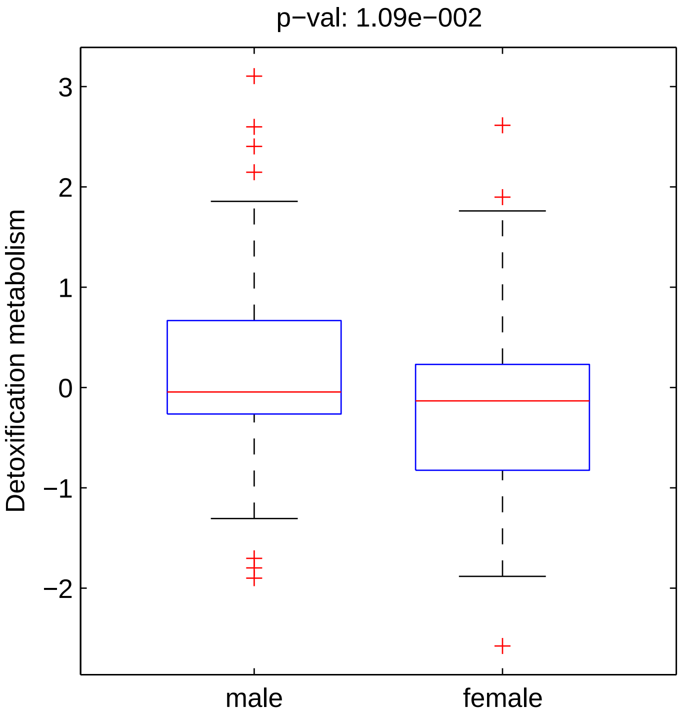
p−val: 1.09e−002
3
2
1
0
−1
−2
Detoxification metabolism
male
female

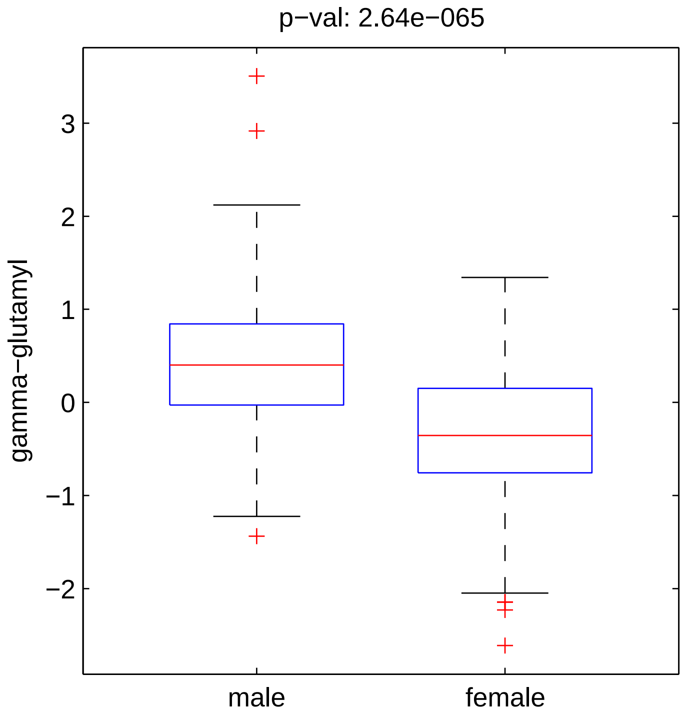
p−val: 2.64e−065
gamma−glutamyl
3
2
1
0
−1
−2
male
female

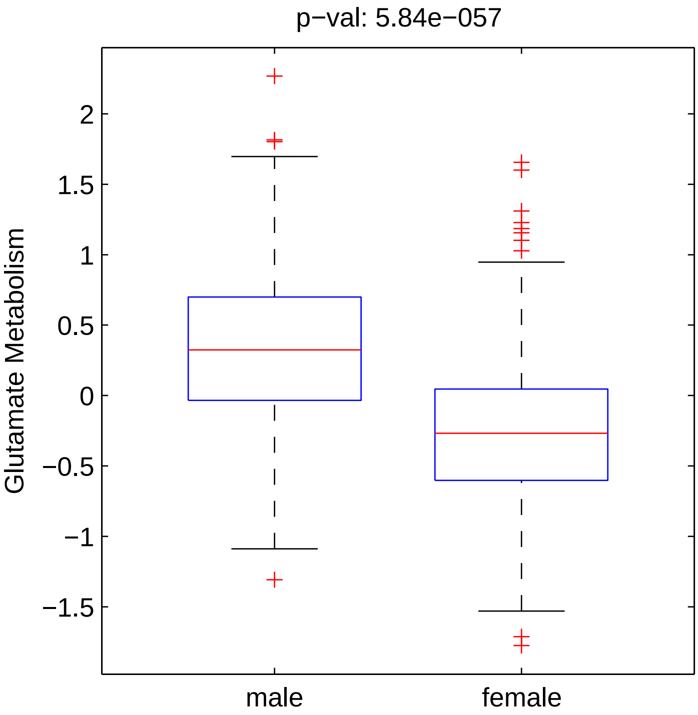

p−val: 5.84e−057
Glutamate Metabolism
2
1.5
1
0.5
0
−0.5
−1
−1.5
male
female

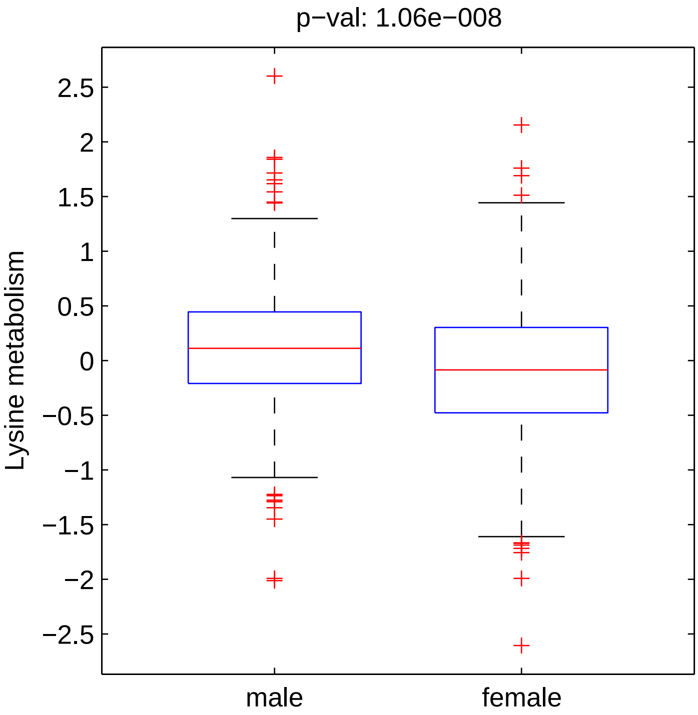

p−val: 1.06e−008
Lysine metabolism
2.5
2
1.5
1
0.5
0
−0.5
−1
−1.5
−2
−2.5
male
female

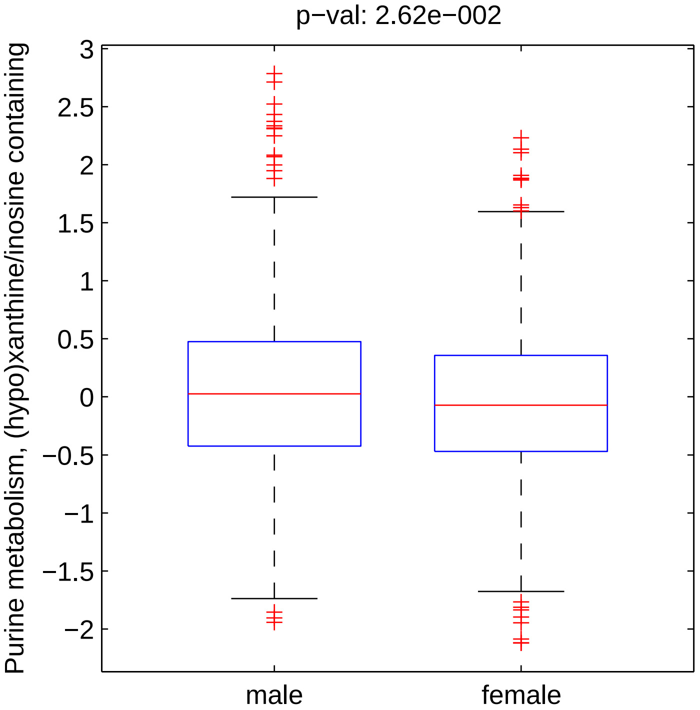

p−val: 2.62e−002
Purine metabolism, (hypo)xanthine/inosine containing
3
2.5
2
1.5
1
0.5
0
−0.5
−1
−1.5
−2
male
female

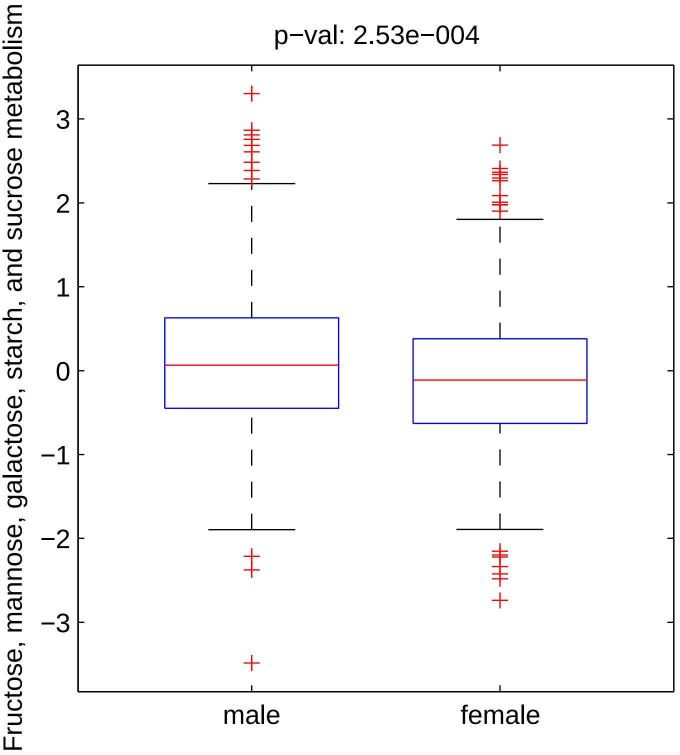

p−val: 2.53e−004
Fructose, mannose, galactose, starch, and sucrose metabolism
3
2
1
0
−1
−2
−3
male
female

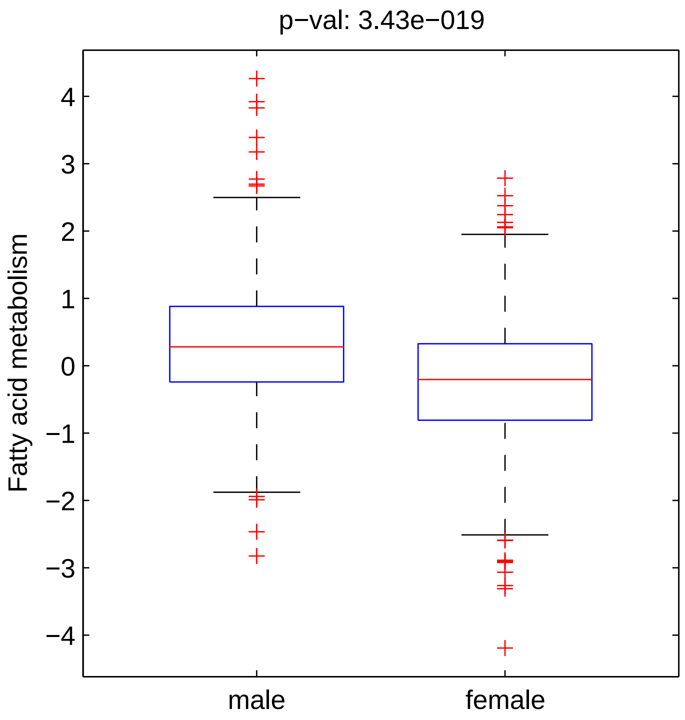
p−val: 3.43e−019
Fatty acid metabolism
4
3
2
1
0
−1
−2
−3
−4
male
female

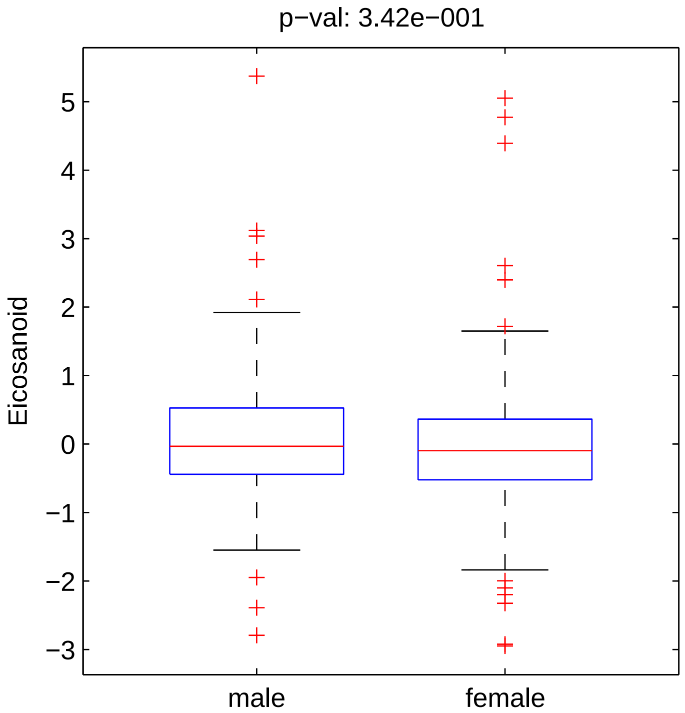

p−val: 3.42e−001
Eicosanoid
5
4
3
2
1
0
−1
−2
−3
male
female

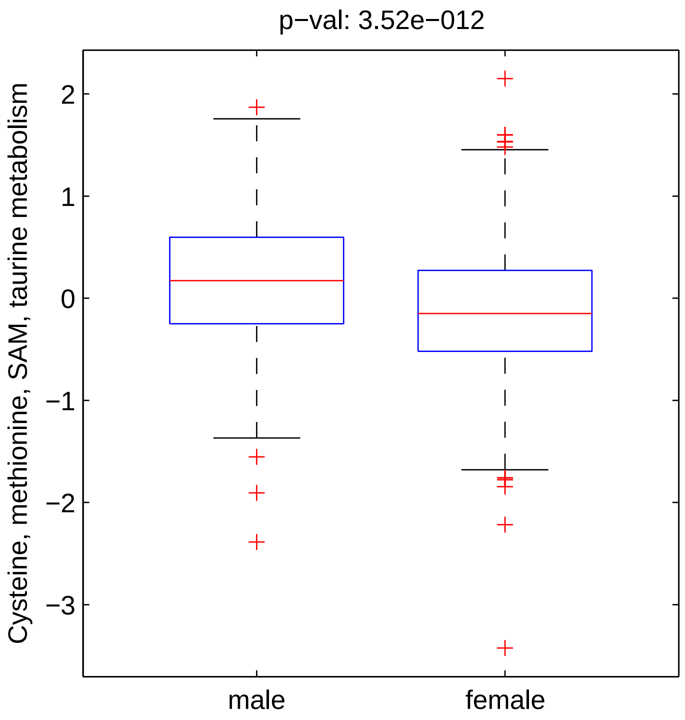

p−val: 3.52e−012
Cysteine, methionine, SAM, taurine metabolism
2
1
0
−1
−2
−3
male
female

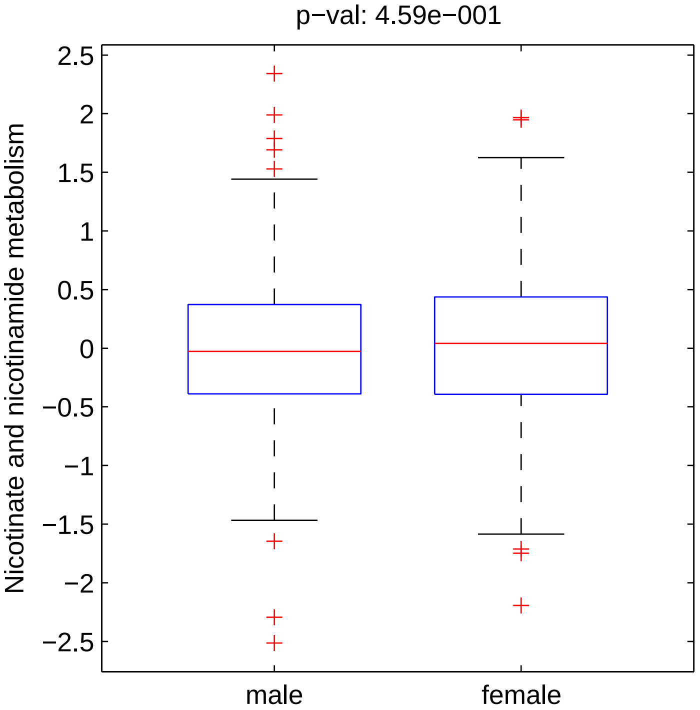
p−val: 4.59e−001
Nicotinate and nicotinamide metabolism
2.5
2
1.5
1
0.5
0
−0.5
−1
−1.5
−2
−2.5
male
female

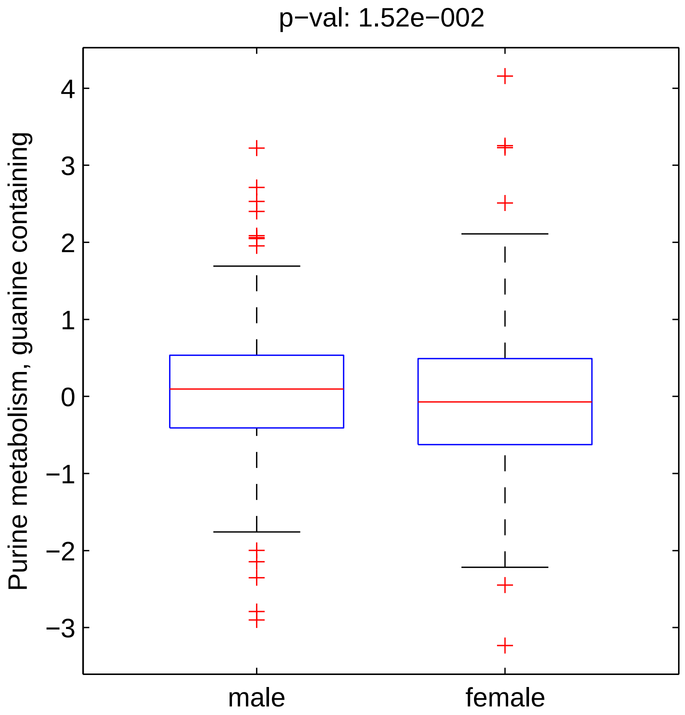

p−val: 1.52e−002
Purine metabolism, guanine containing
4
3
2
1
0
−1
−2
−3
male
female

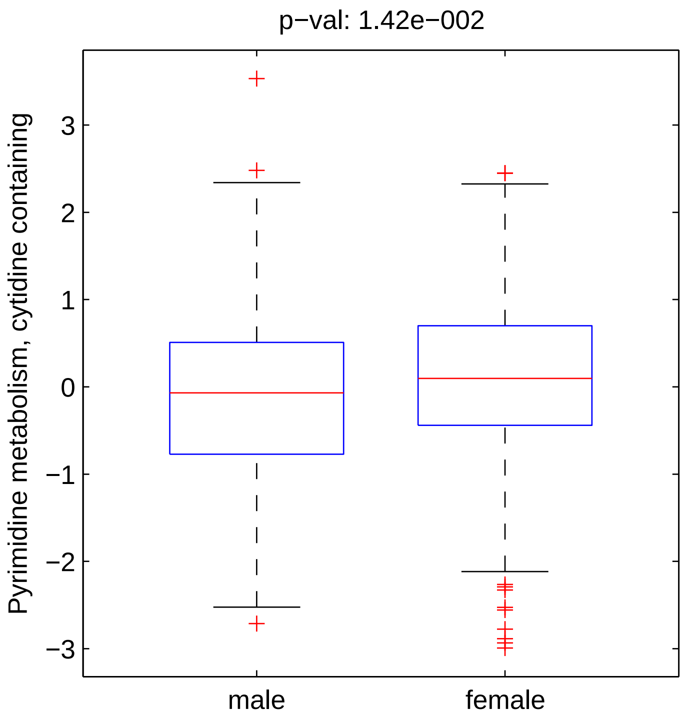
p−val: 1.42e−002
Pyrimidine metabolism, cytidine containing
3
2
1
0
−1
−2
−3
male
female

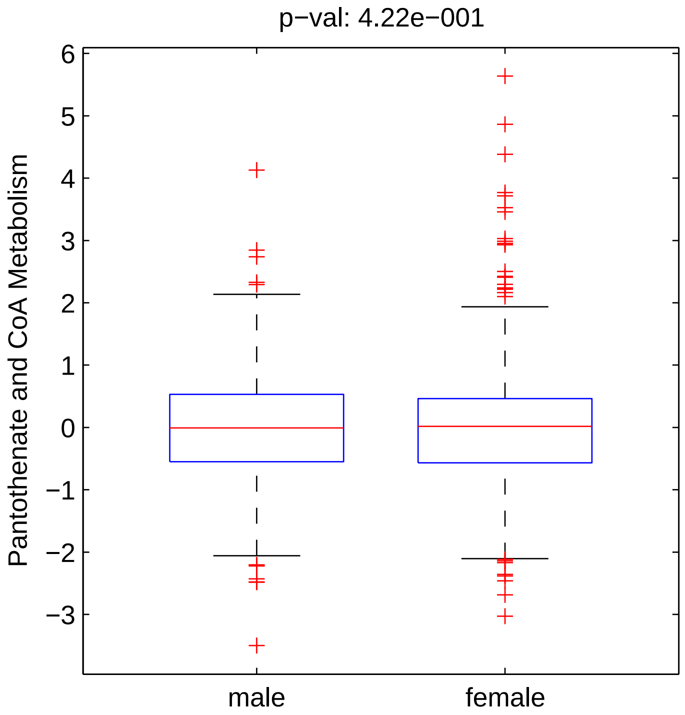

p−val: 4.22e−001
Pantothenate and CoA Metabolism
6
5
4
3
2
1
0
−1
−2
−3
male
female

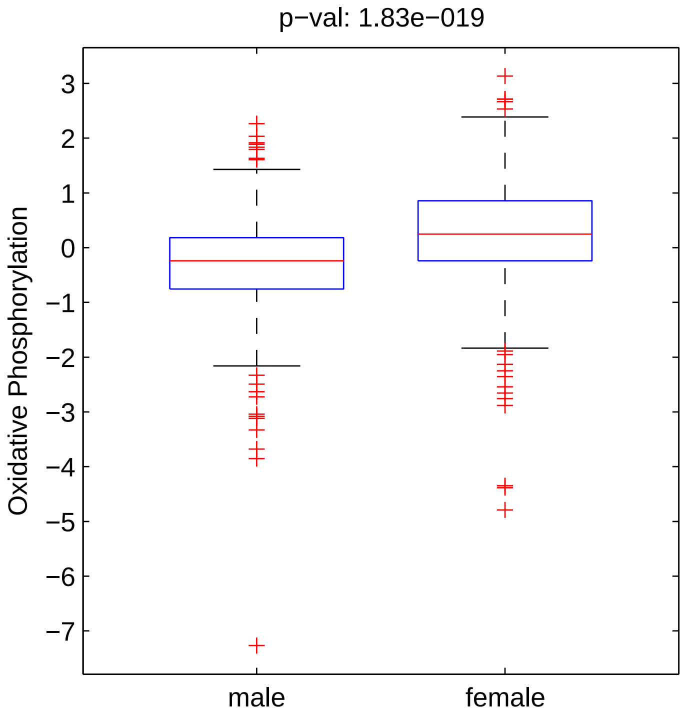
p−val: 1.83e−019
Oxidative Phosphorylation
3
2
1
0
−1
−2
−3
−4
−5
−6
−7
male
female

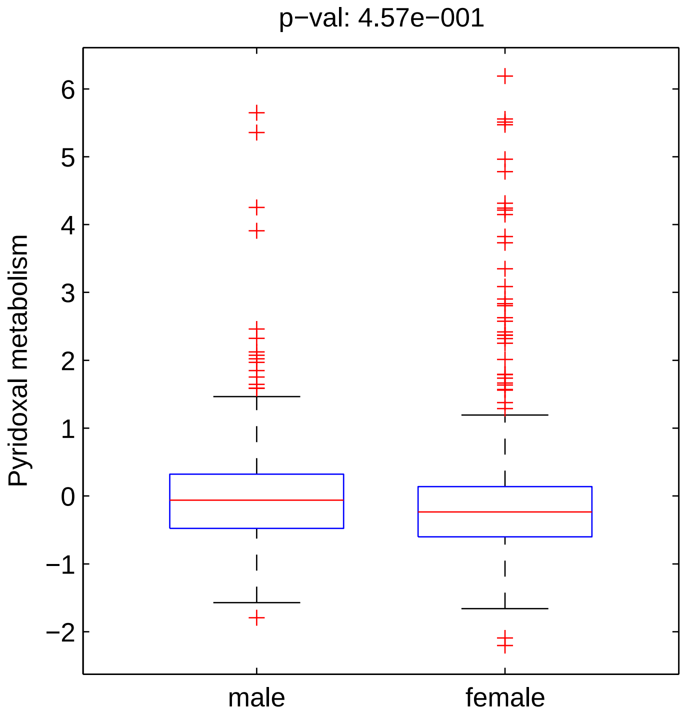
p−val: 4.57e−001
Pyridoxal metabolism
6
5
4
3
2
1
0
−1
−2
male
female

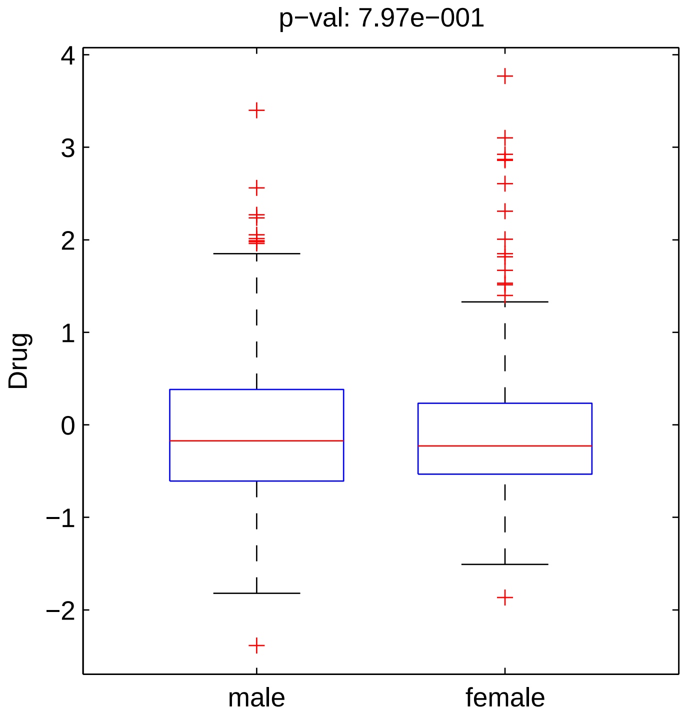

p−val: 7.97e−001
Drug
4
3
2
1
0
−1
−2
male
female

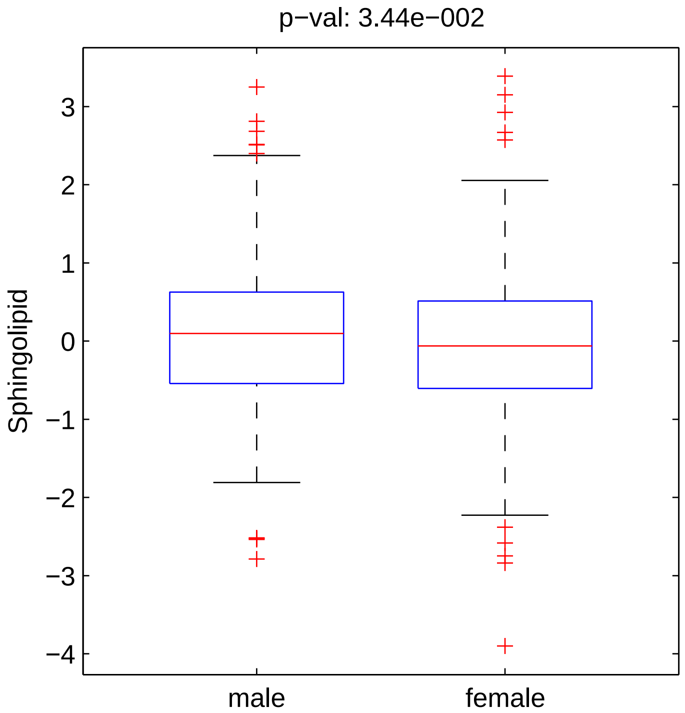

p−val: 3.44e−002
Sphingolipid
3
2
1
0
−1
−2
−3
−4
male
female

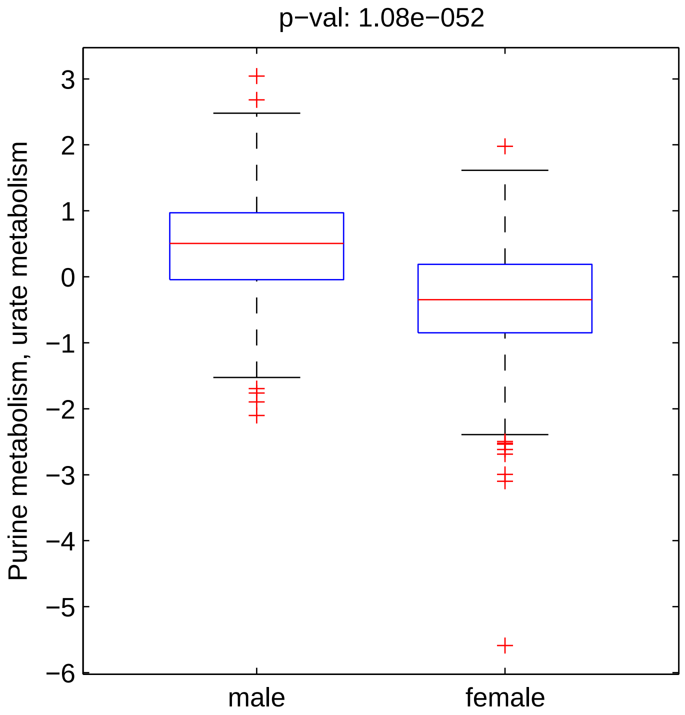

p−val: 1.08e−052
Purine metabolism, urate metabolism
3
2
1
0
−1
−2
−3
−4
−5
−6
male
female

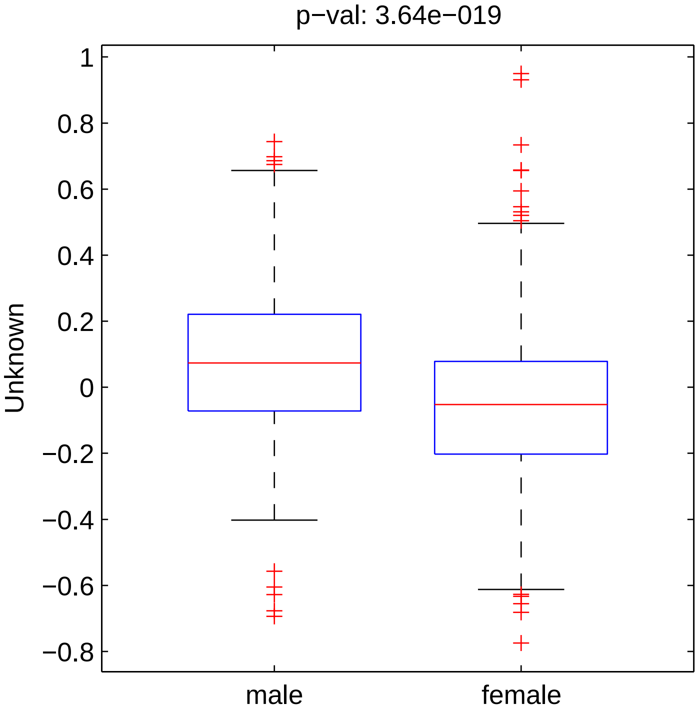
p−val: 3.64e−019
Unknown
1
0.8
0.6
0.4
0.2
0
−0.2
−0.4
−0.6
−0.8
male
female

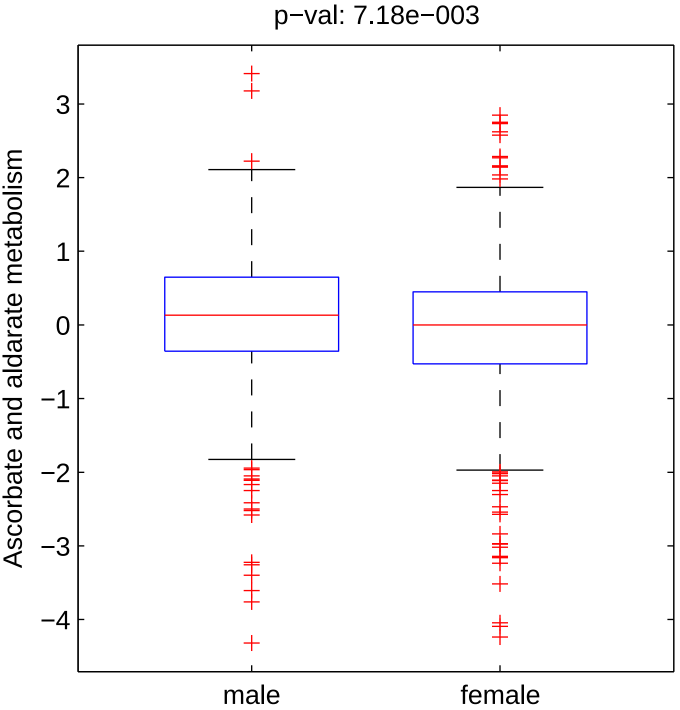

p−val: 7.18e−003
Ascorbate and aldarate metabolism
3
2
1
0
−1
−2
−3
−4
male
female

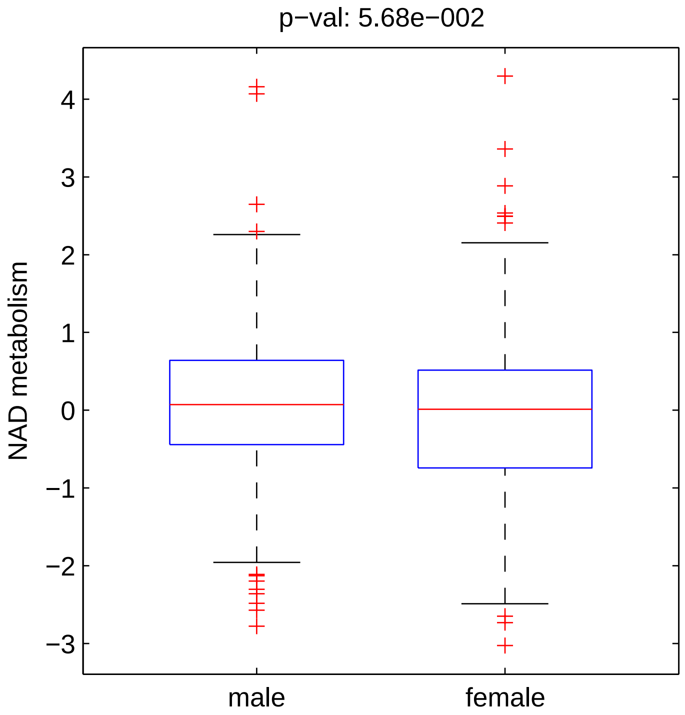

p−val: 5.68e−002
NAD metabolism
4
3
2
1
0
−1
−2
−3
male
female
