# Supplementary material for: Gender-specific pathway differences in the human serum metabolome
Source: Metabolomics. 2015 Aug 4;11(6):1815–33. doi: 10.1007/s11306-015-0829-0 (PMC4605991; doi:10.1007/s11306-015-0829-0)
Supplement: Supplementary file 5 — Supplementary material 5 (PDF 401 kb) [file 11306_2015_829_MOESM5_ESM.pdf]

Supplementary material 5: Network clustering

Clustering approach

This section describes the clustering approach for partial correlation matrices used in the manuscript. Due to their sparsity properties, partial correlations are not suitable as a distance measure for clustering. Intuitively, partial correlations cannot distinguish pairs of nodes that are only two steps apart in the network from nodes which are far apart. The partial correlation will always be close to zero. We therefore developed a transformation of the partial correlation matrix that emphasizes network neighborhood by taking the multiplicative strongest path scoring. This approach assumes edge weights to be between 0 and 1, so negative partial correlations either have to be set to zero or their respective absolute value before the analysis. Note that standard graph clustering methods on partial correlation matrices from this dataset resulted in poor results, usually with one large connected component and several scattered small clusters (data not shown).

Computationally, the multiplicative strongest path can be calculated by applying regular shortest path calculation on log-transformed weights. Let G be the original matrix of all edge weights to be clustered and $e_1, e_2, \dots, e_n$ be the edge weights of a path of length n. Then the multiplicative weight along these edges can be expressed additively as

$$\prod_{i=1}^{n} e_i = \exp\left(\log\left(\prod_{i=1}^{n} e_i\right)\right) = \exp\left(\sum_{i=1}^{n} \log(e_i)\right)$$

Now let $\Psi: [0,1]^p \to [0,1]^p$ be a shortest path matrix transformation function, e.g. Johnson's algorithm [1], for a graph of p nodes. We can derive the multiplicative strongest path matrix M from G as

$$M \coloneqq \exp(-\Psi(-\log(G)))$$

Note taking the negative of the matrix before and after shortest path calculation, which turns the *shortest* path calculation into a *strongest* path calculation.

In our study two preprocessing steps were performed on the partial correlation matrix before subjecting it to this procedure. First, negative partial correlations where set to zero, since those values can be difficult to interpret and might represent statistical artifacts [2]. Second, to put stronger emphasis also on smaller values, we used the square root of each partial correlation value.

The concept of multiplicative scoring is visualized in the following diagram.

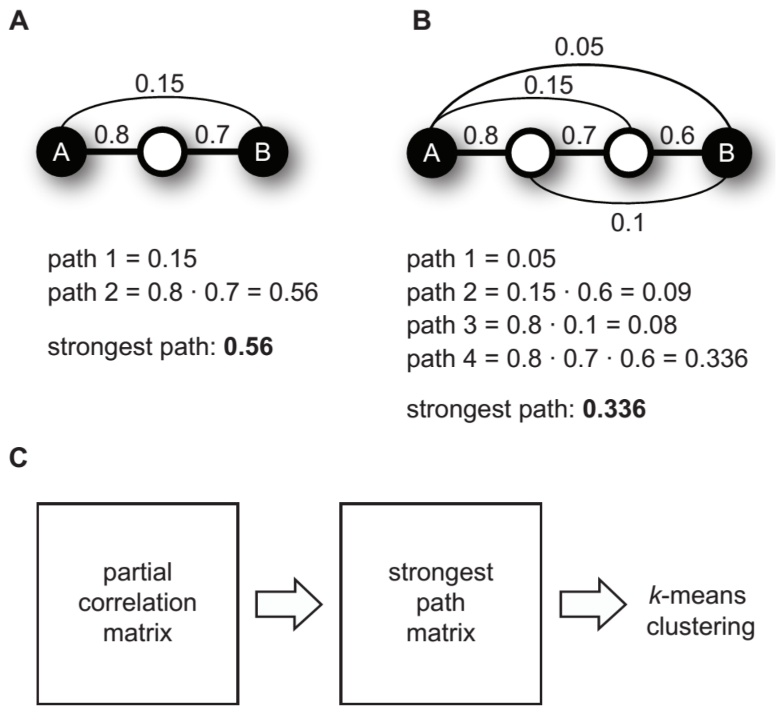

A: Simple example of distance calculation in a 3 node network. The distance between the two nodes A and B is calculated. The direct path from A to B has a weight of only 0.15, whereas the multiplicative path strength through the intermediate node is $0.8 \cdot 0.7 = 0.56$. Thus the new similarity value between A and B for clustering is 0.56.
B: Example with 4 nodes. The path between A and B that passes through both intermediate nodes is stronger than any other possible path between A and B.
C: Clustering process. We first transform the partial correlation matrix to a strongest path matrix and then perform regular *k*-means clustering on that matrix.

Note that the approach is not restricted to partial correlation matrices and could be applied to any weighted network with edge weights between 0 and 1.

Cluster quality

The quality of a network cluster is assessed as the mean edge weight of its maximal spanning tree. The maximal spanning tree represents the strongest set of edges such that all nodes in the network are connected. In the following diagram, the calculation is shown for an example subnetwork:

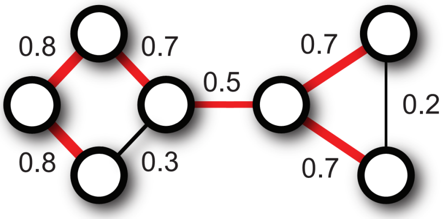

quality: (0.8+0.8+0.7+0.5+0.7+0.7)/6 = 0.7

The maximal spanning tree is represented by the red edges. Note that here in this diagram, several weaker links are shown for visualization purposes. Of course, all possible node pairs have a value in the partial correlation matrix. In case of a weakly connected cluster, one or more weak links have to be used in the spanning tree, causing the cluster quality score to drop.

References

1. Johnson DB (1977) Efficient Algorithms for Shortest Paths in Sparse Networks. J ACM 24: 1–13. doi:10.1145/321992.321993.

2. Krumsiek J, Suhre K, Illig T, Adamski J, Theis FJ (2011) Gaussian graphical modeling reconstructs pathway reactions from high-throughput metabolomics data. BMC Syst Biol 5: 21.
