# Supplementary material for: Gender-specific pathway differences in the human serum metabolome
Source: Metabolomics. 2015 Aug 4;11(6):1815–33. doi: 10.1007/s11306-015-0829-0 (PMC4605991; doi:10.1007/s11306-015-0829-0)
Supplement: Supplementary file 6 — Supplementary material 6 (PDF 1147 kb) [file 11306_2015_829_MOESM6_ESM.pdf]

Supplementary material 6: Cluster stability analysis

For network clustering in this study, we chose an ad-hoc number of k=75 clusters. This supporting information text provides an evaluation of further choices of k as well as an assessment of the stability of clustering.

Choice of the number of clusters *k*

We calculated the mean silhouette coefficient [1] as an objective measure for the number of clusters. The higher this coefficient, the closer the distances of metabolites within a cluster compared to between clusters. Importantly, this measure is independent of the choice of k, unlike e.g. the within-cluster sum of squares used in k-means clustering. Silhouettes are commonly used to determine the optimal choice of k. The following figure shows boxplots of the mean silhouette coefficients in each clustering for k ranging from 10 to 100, with 100 repetitions of the clustering process to account for non-deterministic effects in the k-means algorithm. We chose 10-100 given our set of 494 metabolites in the networks as a reasonable range of clusters, avoiding very fine-grained clusters.

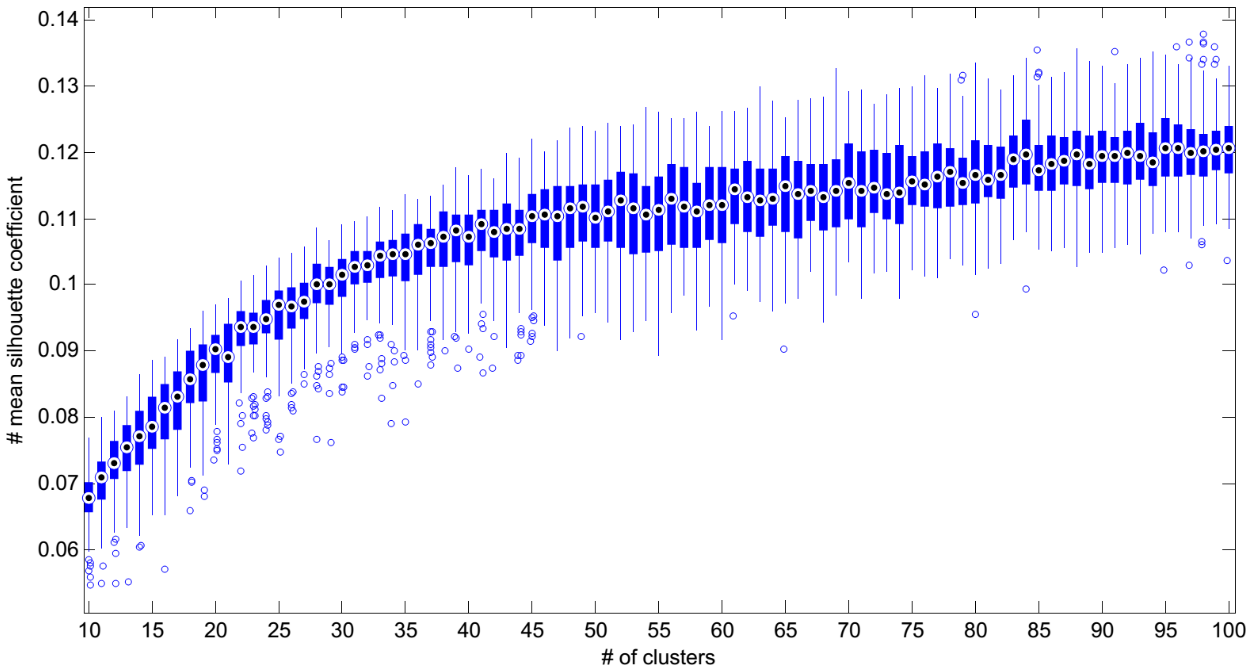

We observe poor performance if the number of clusters is below 50. Between 50 and 100 clusters the curve flattens out, with a slight trend towards higher quality for a higher number of clusters. However, given the variation of mean silhouette coefficients over multiple runs, this effect can be considered as neglectable. Importantly, while silhouette coefficients range between -1 and 1, the mean values over all clusters shown here should not be used as a general measure of clustering quality. Any clustering will contain both high-quality and low-quality clusters, especially in a high-dimensional data space, and thus generally high values of the coefficient cannot be expected. It should thus not be used as an overall indicator of cluster quality, but rather as a comparative measure.

The analysis demonstrates that, from a data-driven point-of-view, there is no objective choice of the number of clusters the range between 10 and 100. We will use an ad-hoc choice of 75 clusters for the analysis. The following sections of this supplementary information further investigate the clustering and stability for the choice of 75 clusters.

Stability analysis via bootstrapping for k=75

We next assessed the stability of clusters for the choice of k=75 by bootstrapping the original dataset, i.e. randomly generating new datasets by drawing from the original set of samples with replacement [2]. For this analysis, we generated 100 bootstrap samples. As a measure of stability we calculated the maximal Jaccard index [3] with any cluster from the bootstrap sample. The Jaccard index is a measure of normalized overlap between two sets, with values ranging between 0 (no overlap) and 1 (perfect overlap). The following figure shows boxplots of the maximal Jaccard index with a bootstrap cluster for each original cluster.

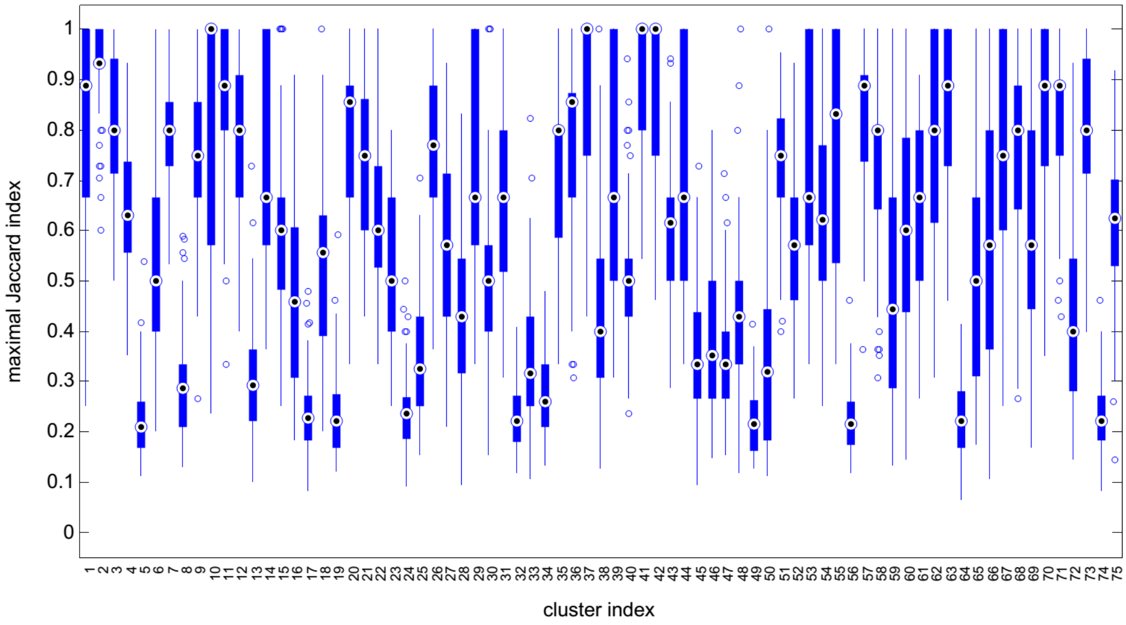

We observe many clusters with high stability (i.e. high maximal Jaccard index), indicating that the same or a very similar cluster also appeared in many of the bootstrap sample clusterings. A substantial amount of clusters, however, also shows poor stability. It is to be expected that instable clusters also show a poor cluster quality in the original run (see Text S2 for the definition of cluster quality). We therefore assessed the relationship between cluster stability in the data and the quality of the cluster determined by its inner weight:

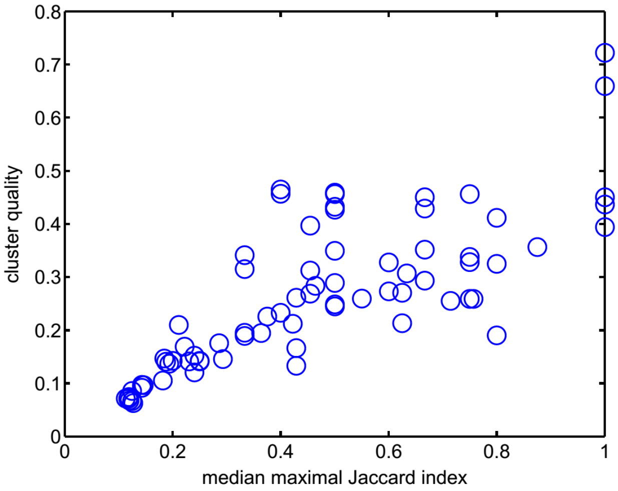

Clusters with a high quality, which are the clusters that we focused our analysis on the study, tend to show high stability. Low quality clusters are, as expected, also rather unstable and should be excluded from the analysis.

Cluster stability for different choices of *k*

In the final step, we evaluated whether the above-mentioned relationship between cluster quality and cluster stability also holds for different values of the number of clusters *k*. That is, we aimed to verify whether high quality clusters would appear independent of the choice of *k*.

The following plots show the relationship for *k* ranging from 70 to 80, i.e. symmetrically around our ad-hoc choice of 75 clusters.

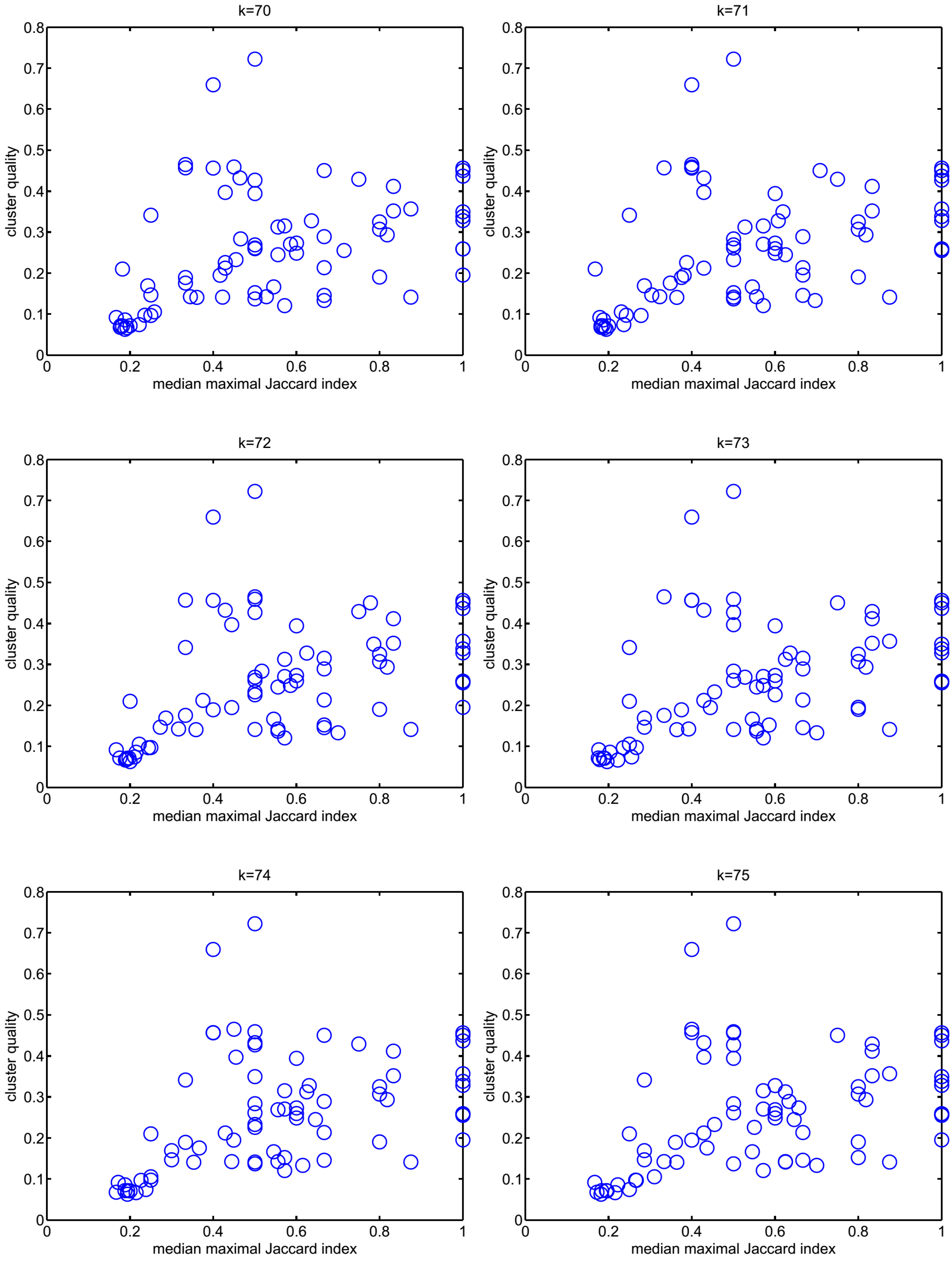

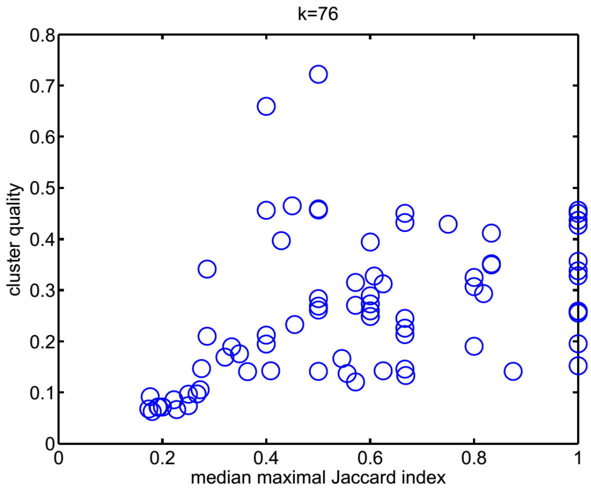

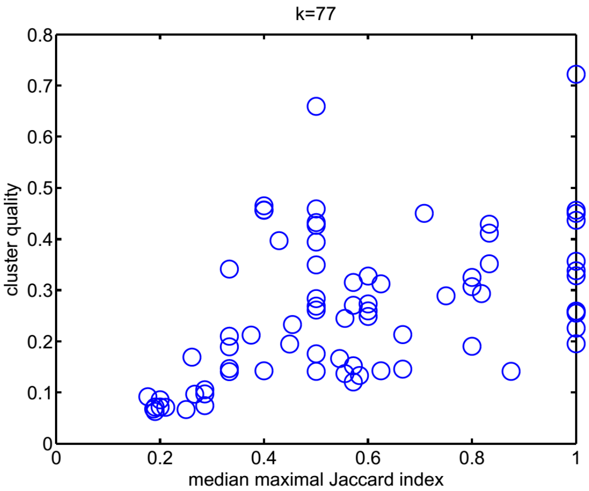

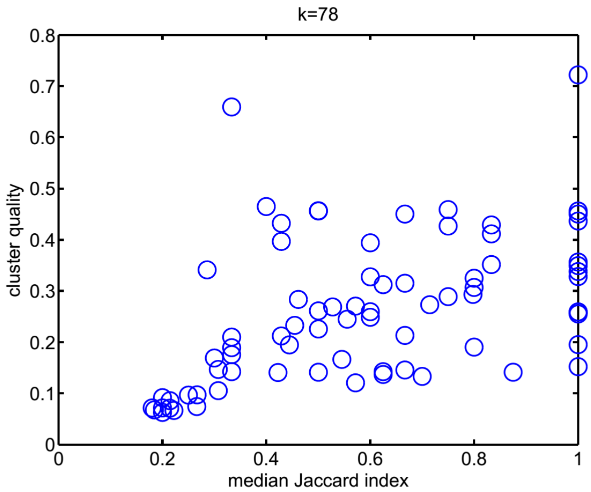

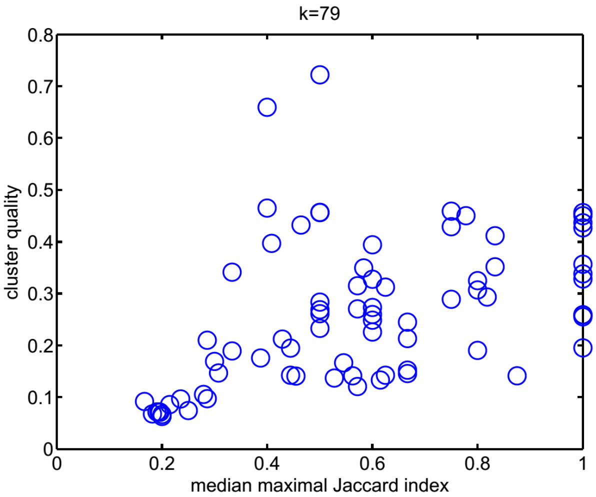

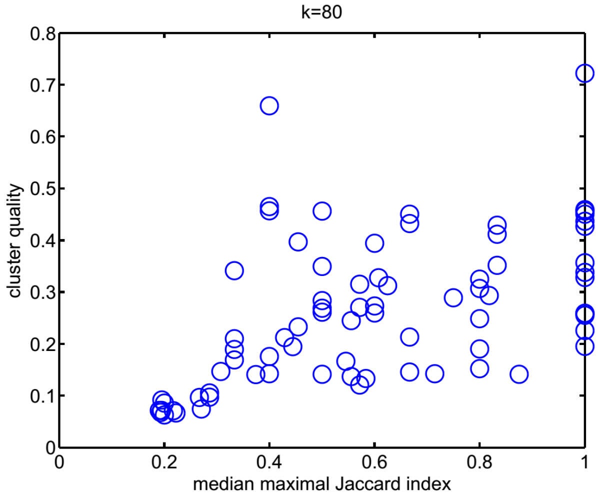

The results demonstrate that high-quality clusters are preserved even for varying valus of the number of clusters parameter *k*. Note the the result for k=75 is not identical to the plot in the section above, since in this analysis we did not perform bootstrapping (just multiple runs of the clustering process).

References

1. Rousseeuw PJ. Silhouettes: A graphical aid to the interpretation and validation of cluster analysis. J Comput Appl Math. 1987;20: 53–65. doi:10.1016/0377-0427(87)90125-7

2. Efron B. Bootstrap Methods: Another Look at the Jackknife. Ann Stat. 1979;7: 1–26. doi:10.1214/aos/1176344552

3. Jaccard P. Étude comparative de la distribution florale dans une portion des Alpes et des Jura. Bull Société Vaudoise Sci Nat. 1901;37: 547–579.
