# Supplementary material for: Gender-specific pathway differences in the human serum metabolome
Source: Metabolomics. 2015 Aug 4;11(6):1815–33. doi: 10.1007/s11306-015-0829-0 (PMC4605991; doi:10.1007/s11306-015-0829-0)
Supplement: Supplementary file 8 — Supplementary material 8 (PDF 201 kb) [file 11306_2015_829_MOESM8_ESM.pdf]

Supplementary material 8: Sex-specific genome-wide association study

This supplementary text contains detailed statistics and plots for the genome-wide significant hit.

Genome-wide significant differences

						females			males				
Metabolite	SNP	chr	pos	info	MAF (f/m)	β	SE	p-val	β	SE	p-val	p-val β diff	count (f/m)
glycine	rs1047891	2	211540507	0.95	0.32/0.32	0.09	0.006	1.8E-45	0.03	0.005	1.3E-06	7.2E-14	872/823
glycine	rs715	2	211543055	1.00	0.31/0.31	0.08	0.006	1.6E-43	0.02	0.005	3.7E-06	1.5E-13	872/823

Explanations: chr = chromosome, pos = chromosomal position, info = imputation quality, MAF = major allele frequency, β = effect size from additive genetic model, SE = standard error

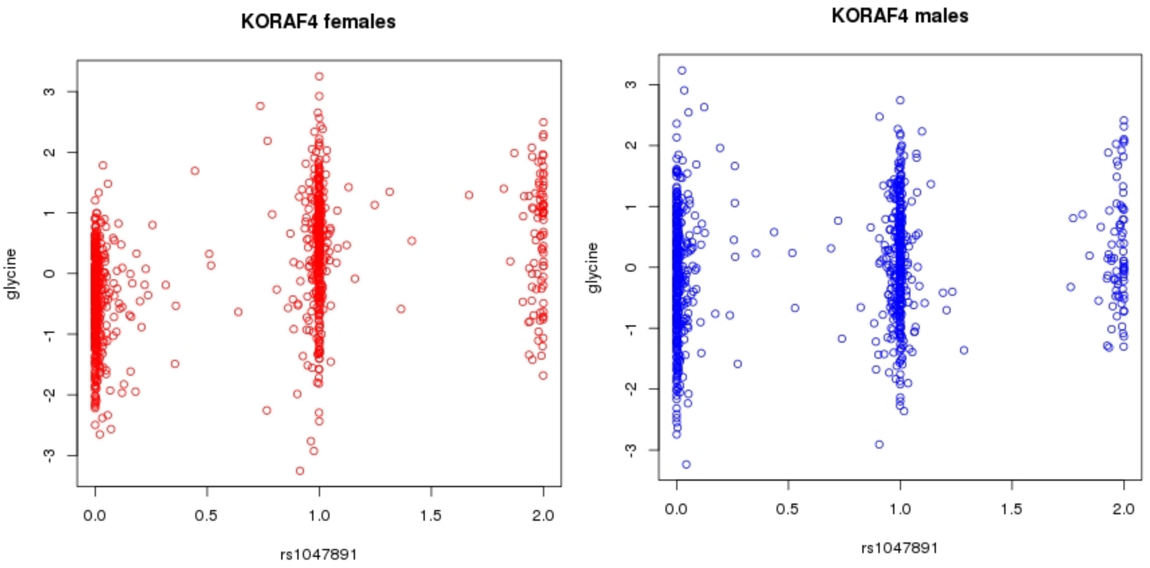

Figure: Associations between glycine and rs1047891 stratified by females and males. Note that genotypes on the x-axis can have non-integer values due to the imputation process.
