# Supplementary material for: Gender-specific pathway differences in the human serum metabolome
Source: Metabolomics. 2015 Aug 4;11(6):1815–33. doi: 10.1007/s11306-015-0829-0 (PMC4605991; doi:10.1007/s11306-015-0829-0)
Supplement: Supplementary file 9 — Supplementary material 9 (PDF 69 kb) [file 11306_2015_829_MOESM9_ESM.pdf]

Supplementary material 9: Experimental procedures

Relative abundances of metabolites in blood serum samples were determined at Metabolon Inc. (Durham, NC, USA) using a non-targeted mass spectrometry-based (MS) metabolomics approach. To this end, 100 µl of each thawed serum sample were pipetted in a 96 deep well plate format. After adding 400 µl of extraction solvent (methanol containing the recovery standards: tridecanoic acid, fluorophenylglycine, chlorophenylalanine, and d6-cholesterol), the mixture was shaken for two minutes using a Geno/Grinder 2000 (Glen Mills Inc., Clifton, NJ, USA). After centrifugation, the supernatant was split into four 25 µl aliquots. The solvent was removed from the aliquots on a TurboVap (Zymark) and dried under vacuum overnight. All steps of the extraction process were performed while keeping samples at 4°C. For MS analysis in positive ion mode coupled to liquid chromatography (LC/MS pos), the first aliquot was reconstituted with 0.1% formic acid containing internal standards for quality control. For MS analysis in negative ion mode coupled to liquid chromatography (LC/MS neg), the second aliquot was reconstituted with 6.5 mM ammonium bicarbonate (pH 8.0) also containing internal standards. The third aliquot was mixed with 1:1 mixture N,O-bistrimethylsilyl-trifluoroacetamide and solvent (acetonitrile:dichloromethane: cyclohexane (5:4:1), containing 5% triethylamine and a series of alkylbenzenes used as retention time markers) and kept at 60°C for 1h to derivatize for MS analysis coupled to gas chromatography (GC/MS). Sample preparation was performed on a Hamilton MLStar (Hamilton Company, Salt Lake City, UT, USA) robotics system.

LC/MS analysis was performed on an LTQ mass spectrometer (Thermo Fisher Scientific Inc., Waltham, MA, USA) coupled to a Waters Acquity UPLC system (Waters Corporation, Milford, MA, USA). The UPLC was directly connected to the electrospray ionization (ESI) source of the spectrometer. Two separate columns (2.1 x 100 mm Waters BEH C18 1.7 µm particle) were used for acidic (solvent A: 0.1% formic acid in H2O, solvent B: 0.1% formic acid in methanol) and basic (A: 6.5 mM ammonium bicarbonate pH 8.0, B: 6.5 mM ammonium bicarbonate in 98% methanol) mobile phase conditions, optimized for positive and negative electrospray ionization, respectively. After injection of the sample extracts, a gradient was applied from 100% A to 98% B in 11 min runtime at 350 µl/min flow rate. Full scan mass spectra (99–1000 m/z) and data dependent fragmentation spectra (dynamic exclusion of already fragmented ions) were recorded in turns. GC/MS analysis was performed on a Thermo-Finnigan Trace DSQ fast-scanning single-quadrupole mass spectrometer, equipped with a 20 m x 0.18 mm GC column with 0.18 µm film phase consisting of 5% phenyldimethyl silicone. The column temperature was ramped between 60°C and 340°C with helium as carrier gas. Using electron impact ionization at 70 eV, mass spectra in a range from 50-750 m/z, were recorded. Detailed descriptions of the analytical method can be found in [1].

Metabolites were identified from the experimental LC/MS and GC/MS spectra by semi-automated comparison to Metabolon's proprietary reference library entries following the Metabolomics Standardization Initiatives guidelines [2]. Library entries of known metabolites were created based on spectra recorded from commercially available authentic compounds. All compounds were analyzed at multiple concentrations and under the same conditions as the experimental samples. Some of these spectra have been made publicly available (MassBank). Additionally, the library contains reference spectra for known metabolites (as derived from one of the three platforms) for which the respective authentic compound was unavailable. In the same way, the library also stores the retention times, characteristic m/z values and fragmentation spectra of unknown metabolites that were repeatedly observed in experimental samples. For identification, experimental peaks are compared to the reference entries based on three criteria: (i) experimental retention time within 40 retention index units of the reference? (ii) experimental precursor m/z value(s) within 0.4 m/z of the reference? (iii) experimental and reference fragmentation spectra match (forward and reverse comparison). The resulting identifications from the automated matching process are manually confirmed by experts. For detailed description of the metabolite identification software are given in [3].

References

1. Evans AM, DeHaven CD, Barrett T, Mitchell M, Milgram E. Integrated, nontargeted ultrahigh performance liquid chromatography/electrospray ionization tandem mass spectrometry platform for the identification and relative quantification of the small-molecule complement of biological systems. Anal Chem. 2009;81: 6656–6667. doi:10.1021/ac901536h

2. Sansone S-A, Fan T, Goodacre R, Griffin JL, Hardy NW, Kaddurah-Daouk R, et al. The Metabolomics Standards Initiative. Nat Biotechnol. 2007;25: 846–848. doi:10.1038/nbt0807-846b

3. Dehaven CD, Evans AM, Dai H, Lawton KA. Organization of GC/MS and LC/MS metabolomics data into chemical libraries. J Cheminformatics. 2010;2: 9. doi:10.1186/1758-2946-2-9
