# Supplementary material for: Gender-specific pathway differences in the human serum metabolome
Source: Metabolomics. 2015 Aug 4;11(6):1815–33. doi: 10.1007/s11306-015-0829-0 (PMC4605991; doi:10.1007/s11306-015-0829-0)
Supplement: Supplementary file 10 — Supplementary material 10 (PDF 123 kb) [file 11306_2015_829_MOESM10_ESM.pdf]

## Supplementary material 10: Tests for non-normality

For each metabolite in the KORA discovery cohort, the non-normality of both log10-transformed and non-transformed concentrations was assessed using Kolmogorov-Smirnov tests [1]. The following plot shows the log10 p-values for each metabolite:

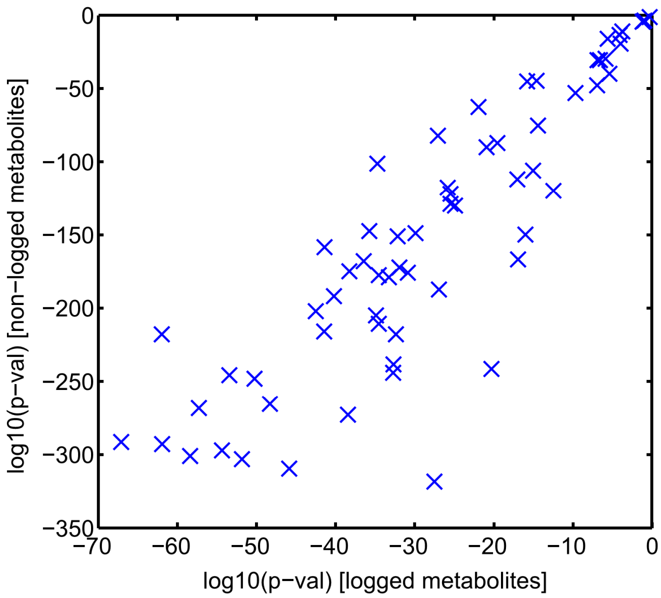


The non-logarithmized metabolite concentration distributions are substantially less normal, i.e. have lower p-values from the Kolmogorov-Smirnov tests. It is to be noted that also the p-values for logarithmized concentrations are very low, which can be attributed to the high sample number (n=1,756) in the dataset. There are ongoing discussions in the community that for sufficiently large sample sizes, the normality assumption of a statistical test will not be a major issue [2]. Nevertheless, logarithmizing concentrations appears to ensure "more normal" distributions than non-transformed data.

## References


1. Massey FJ Jr. The Kolmogorov-Smirnov Test for Goodness of Fit. J Am Stat Assoc. 1951;46: 68–78. doi:10.2307/2280095

2. Ghasemi A, Zahediasl S. Normality Tests for Statistical Analysis: A Guide for Non-Statisticians. Int J Endocrinol Metab. 2012;10: 486–489. doi:10.5812/ijem.3505
